# Unidentifiable by morphology: DNA barcoding of plant material in local markets in Iran

**DOI:** 10.1371/journal.pone.0175722

**Published:** 2017-04-18

**Authors:** Abdolbaset Ghorbani, Yousef Saeedi, Hugo J. de Boer

**Affiliations:** 1 Department of Organismal Biology, Evolutionary Biology Centre, Uppsala University, Uppsala, Sweden; 2 Traditional Medicine and Materia Medica Research Center, Shahid Beheshti University of Medical Sciences, Tehran, Iran; 3 Naturalis Biodiversity Center, Leiden, The Netherlands; 4 The Natural History Museum, University of Oslo, Oslo, Norway; Missouri Botanical Garden, UNITED STATES

## Abstract

Local markets provide a rapid insight into the medicinal plants growing in a region as well as local traditional health concerns. Identification of market plant material can be challenging as plants are often sold in dried or processed forms. In this study, three approaches of DNA barcoding-based molecular identification of market samples are evaluated, two objective sequence matching approaches and an integrative approach that coalesces sequence matching with *a priori* and *a posteriori* data from other markers, morphology, ethnoclassification and species distribution. Plant samples from markets and herbal shops were identified using morphology, descriptions of local use, and vernacular names with relevant floras and pharmacopoeias. DNA barcoding was used for identification of samples that could not be identified to species level using morphology. Two methods based on BLAST similarity-based identification, were compared with an integrative identification approach. Integrative identification combining the optimized similarity-based approach with *a priori* and *a posteriori* information resulted in a 1.67, 1.95 and 2.00 fold increase for ITS, *trn*L-F spacer, and both combined, respectively. DNA barcoding of traded plant material requires objective strategies to include data from multiple markers, morphology, and traditional knowledge to optimize species level identification success.

## Introduction

In places where traditional medicine plays a major role in delivering health care, local markets can provide a rapid insight into medicinal plants harvested and traded in a region [1]. Marketplaces do not only reflect the diversity and prevalence of medicinal plants growing in the region, but also yield an understanding of the plants’ utilization, seasonal availability, and chain of commercialization, as well as local health and disease concerns and the importance of traditional medicine among local people [1,2].

Medicinal plants in markets and traditional herbal shops are normally traded as dried leaves, roots and barks, or in processed forms such as powdered parts, mixtures, or extracts. Such plant material lacks many of the morphological characteristics necessary for accurate identification by retailers and customers. Aerial parts may lose important diagnostic characters necessary for taxonomic identification, and the identification of roots is always challenging due to a lack of distinctive morphology [3,4]. Moreover, taxonomic identification using macro- and micro-morphological and organoleptic methods can be time-consuming, error-prone and requires expertise and reliable references [5,6].

Many studies of market ethnobotany include vernacular names as part of the identification diagnostics, combined with morphological or molecular identification [3,7–10]. In ethnopharmacology, pharmacognosy, and pharmacovigilance there is also strong reliance on vernacular names from products, interviews, pharmacopoeias, and literature for identification of plant material [11,12]. However, vernacular names are prone to ambiguity as one species may have multiple vernacular names or conversely one vernacular name may be applied to many, and often widely divergent, species [13,14]. Vernacular names also vary across geographical regions and languages, resulting in many orthographic variants of the same name [13,15–17]. An example of under-differentiation is the common name “Avishan,” which is used for any of 14 species of *Thymus* L. in different parts of Iran [18]. Likewise, an example of homonymy is the vernacular name “Goosh Fiel,” which is given to the unrelated species *Arctium lappa* L., *Colocasia* spp. and *Caladium* spp. [19]. Vernacular names are important in the preservation of traditional knowledge and recording ethnotaxa, but relying on them as a basis for scientific identification can result in erroneous identification that may invalidate research findings [17,20].

A further potential complication results from the substitution and adulteration of herbal medicinal products. The retailers of herbal products are often not the producers of the products or the harvesters of the raw materials, and this chain of commercialization often involves many middlemen between the harvest of the plant and its final purchase by the consumer. For example, vendors in *Attari* (traditional herbal shops in Iranian bazaars) typically buy dried plant material from medicinal plant collectors, middlemen or wholesalers [14,21]. Vendors are often able to identify herbal products from their morphological characteristics, but lack the ability to identify the living plants [9]. Plant collectors in turn might have a broad knowledge of plant diversity, but might not be able to tell the medicinal species from other similar species in the same genus [22]. Moreover, morphological similarities among some plant species and dried plant parts, scarcity of medicinal species in nature, careless collection practices and a lack of standard identification and control system are all factors contributing to both accidental and intentional substitution [22,23]. The substitution and adulteration of plant ingredients in herbal products can cause health and safety concerns and has public health implications [24,25]. Ize-Ludlow et al. [26] report that the Chinese star anise (*Illicium verum* Hook f.) in herbal teas is substituted with the morphologically similar but toxic Japanese star anise (*I*. *anisatum* L.), and can result in neurotoxic effects in infants. In Thailand, *Thunbergia laurifolia* Lindl., a common Thai herbal medicine, and *Crotalaria spectabilis* Roth share the same common name [27], but pyrrolizidine alkaloids in *C*. *spectabilis* cause pulmonary arteritis [28] and acute hepatotoxicity [29]. A method to accurately identify plant materials in traditional herbal medicines is essential in order to guarantee consumer safety, and it is also important for quality control and product authentication [12,25]. Moreover, taxonomic identification of plant samples is crucial in ethnobotanical and ethnopharmacological studies, as species identity connects local classifications to scientific names [13,15,16].

DNA barcoding can provide an accurate and reliable alternative to morphological identification for biological material and is often used when identification using macroscopic or microscopic methods is challenging [30]. It can be used to identify and discriminate species in any developmental or processed stage from which DNA can be extracted [30,31], and from minute amounts of material such as those found in dung [32], pollen [33], degraded herbarium vouchers [34], permafrost preserved subfossils [35], and ancient sediment cores [36,37]. Plant DNA barcoding has been applied in molecular systematics [38,39], biodiversity inventories [40], wildlife forensics and bio-piracy [41,42], and authentication of herbal products [3,25,43]. Degraded material can include modern material that is no longer fresh or old samples, faeces samples, samples exposed to contamination from DNA of other organisms, samples that have been heated or dried at high temperatures, have been wet, damp or dried improperly, have been processed intensively, or exposed to insect infestations or fungal infection, but also ancient samples such as those listed above.

In DNA barcoding, a short standardized region of genomic DNA from an unidentified sample is queried against a reference sequence database and the identity of the matching sequence is assigned to the sample [30,31,44]. Several genetic regions have been proposed as standard barcodes for land plants, and the ideal barcode needs to be both easily amplifiable and efficiently retrieved from any of the 300,000+ species of plants [44,45]. A single barcoding locus combining these two traits has not been found for plants, and the focus has shifted to a combination of two or more loci to approach a satisfactory level of species discrimination and universality [46]. Most studies now employ a tiered approach, which is based on the use of a common, easily amplified and aligned region such as *rbc*L, *rpo*C1, or *trn*L-F spacer that can act as a scaffold on which to place data from a more variable noncoding region such as *mat*K, *trn*H*-psb*A, nrITS1, nrITS2 or the full ITS1-5.8S-ITS2 (nrITS). The CBOL Plant Working Group recommends the use of *rbc*L and *mat*K [47], and together with nrITS these are plant barcoding markers curated by BOLD [48]. Using a tiered approach including at least one of these markers, most species (approx. 75–85%) can be identified, and the subsequent addition of surrogate regions can increase barcoding success to over 90% in some floras [49–52]. The barcoding marker *mat*K can be difficult to amplify especially from degraded material [3,9,53,54], but by using target group specific primers amplification success can be greatly increased [55–58].

Sequence similarity [59], tree-based criteria [60], or character-based methods [61,62] can be used for matching unknown query sequences with a reference sequence database for barcode identification. Recent studies show that similarity-based and diagnostic methods that include taking into account non-sequence information significantly outperform tree-based methods [39]. However, the success rate of any method used to assign sequences to a certain taxon is ultimately dependent on the taxonomic coverage of the reference database [3,9].

This study tests the hypothesis that DNA barcode identification by sequence matching can be enhanced by including *a priori* and *a posteriori* data. It employs a two-tiered identification approach using two markers suitable for degraded medicinal plant material, nrITS and *trn*L-F spacer [3,6,9,63,64]. Two methods based on BLAST similarity-based identification, a simple method taking the top hit and an optimized method putting extra weight on the identity value of the query-reference comparison and the deviation of other hits from the top hit, were compared with an integrative identification approach that coalesces sequence matching with *a priori* and *a posteriori* data from other molecular markers, morphology-based identification, pharmacopoeias, scientific literature, vernacular names, and informant identifications. The performances of these respective approaches are evaluated in terms of species level identifications. The hypothesis is tested using a large dataset compiled from medicinal plant samples purchased from herbal vendors in markets throughout northern Iran.

## Methods

### Market samples

Medicinal plant samples were purchased from 17 traditional herbal shops (*Attari*) in six cities in Northern Khorasan province, Iran, as part of an ethnobotany project on medicinal plants from local markets (S1 Table). In total 229 medicinal plant samples were collected (S2 Table). Information regarding vernacular names, plant parts, their uses, and the processing and preparation methods were recorded (S3 Table). Samples were sold as leaves, fruit, seeds, flower petals, roots, stems or gums, and were either sold whole, crushed or powdered (S3 Table). All plant samples were purchased as single ingredient products. The plant samples were deposited in the Herbarium of Traditional Medicine and Materia Medica Research Center (HTMRC), Shahid Beheshti University of Medical Sciences, Tehran, Iran and the Herbarium of Uppsala University (UPS), Uppsala, Sweden.

### Sample identification

All samples were assigned when possible to family, genus and species based on existing morphological characteristics by professional botanists and pharmacognocists trained in morphological and micro-morphological identification of medicinal plants identification using available scientific literature [65–68] (S2 Table). Each sample was tentatively identified to species based on matching vernacular names from herbal pharmacopoeia and reference literature [65–68] (S3 Table). Sixty-eight out of 229 samples were not identifiable beyond genus level, and were subsequently selected for identification through DNA barcoding. Nomenclature of plant names follows The Plant List [69].

### DNA extraction, amplification and sequencing

Total genomic DNA of 68 market samples was extracted using a CTAB protocol [70]. The extracted DNA was purified using the GE Illustra GFX^TM^ PCR DNA and Gel Band Purification kit following the manufacturer’s protocol (GE Healthcare, Little Chalfont, United Kingdom). Two markers were amplified and sequenced for DNA barcoding, the nuclear ribosomal internal transcribed spacer (nrITS), and a plastid marker (*trn*L-F spacer). nrITS was amplified using primers ITS5 (5’- GGAAGTAAAAGTCGTAACAAGG -3’) and ITS4 (5’- TCCTCCGCTTATTGATATGC- 3’) [71] and *trn*L-F spacer using primers trnL_c2 (5’- GGATAGGTGCAGAGACTCAAT -3’) [72] and trnL_f (5’- ATTTGAACTGGTGACACGAG -3’) [73]. PCR amplification was performed in 50 μl reactions containing 5 μl reaction buffer IV (Qiagen NV, Venlo, Netherlands) (10x), 5 μl MgCl_2_ (25mM), 1 μl dNTP (10 μM), 0.25 μl Taq-polymerase (Qiagen NV, Venlo, Netherlands) (5 U/μl), 0.5 μl BSA, 1 μl of each primer (10 mM) and 1 μl of template DNA. The PCR protocol for nrITS was an initial 3 min of denaturation at 95°C, followed by 35 cycles of 20 sec of denaturation at 95°C, 1 min of annealing at 55°C and 2 min of elongation at 72°C, and a final elongation of 10 min at 72°C. For *trn*L-F, the PCR protocol started with an initial 3 min denaturation at 95°C, followed by 35 cycles of 15 sec denaturation at 95°C, 50 sec of annealing at 55°C, and 4 min of elongation at 72°C, and a final elongation of 8 min at 72°C. Sequencing was performed by Macrogen Europe Inc. (Amsterdam, the Netherlands) on an ABI3730XL automated sequencer (Applied Biosystems, Waltham, Massachusetts, USA). Primers used for PCR amplification were also used for sequencing reactions. Sequence trace files were assembled using Pregap4 and Gap4 in the Staden Package [74]. All plant sequences were submitted to BOLD and linked to NCBI GenBank (S3 Table).

### DNA barcode identification

Three approaches were used for DNA barcode identification, two sequence similarity-based methods using BLAST [59] and an integrative method that coalesces similarity-based results with *a priori* and *a posteriori* data. The two methods based on BLAST similarity-based identification were a simple method taking the top hit and an optimized method putting extra weight on the identity value of the query-reference comparison. For both methods sequences were sequentially queried using megablast [59] online at NCBI nucleotide BLAST against the nucleotide database. For the simple method all top hits within 10 points deviation down of the max score were considered: if the max score (-10 points) included only a single species then a species level identification was assigned; if the max score (-10 points) included multiple species in the same genus then a genus level identification was assigned; and if the max score (-10 points) included multiple species in different genera in the same family then a family level identification was assigned. However, the length of the query coverage and the identity between the query and the reference sequence influences the max score in BLAST, and hits with low identity but high query coverage can have higher max scores than hits with high identity but low coverage. For the optimized method a similarity score was calculated for up to 100 BLAST hits if the query cover was 70% or higher: max score*(query cover/identity). Subsequently all hits were ordered by this score, and the deviation for each similarity score value from the highest similarity score was calculated (S4 Table). Identifications were assigned based on a combination of the identity score (High identity: i ≥ 95%; Medium identity: 90% ≤ i < 95%; Low identity: i < 90%) and the number of species within 1% deviation of the calculated similarity score. High identity and one species within 1% deviation was assigned species-level confidence; high identity and more than one species was assigned genus-level confidence; medium identity and one or more species within the same genus was assigned genus-level confidence; medium identity and species from more than one genus was assigned family-level confidence; and low identity was assigned family-level confidence (S5 Table).

### Integrative approach for identification

The integrative approach coalesced the optimized BLAST-based sequence matching results with *a priori* data from morphological characteristics of the material, interview data on vernacular names and studies of literature and pharmacopeias for these names, along with *a posteriori* data from multiple molecular markers and data on traditional use, occurrence, and distribution of putative species in the study area. For example, *a priori* data suggests that the inflorescences Kh111 are *Amaranthus caudatus* L. based on literature, *Amaranthus* sp. based on ethnobotanical interview data and *Amaranthus* sp. based on morphology (lacking spiny hairs). The BLAST hits suggest that the sequence matches with either *Amaranthus hybridus* L. (based on ITS) or *Amaranthus spinosus* L. (based on *trn*L-F spacer). *A posteriori* data gives two additional clues that aid the identification process: 1) Consulting the Flora of Iran and other literature shows us that *Amaranthus spinosus* L. does not occur in Iran; and 2) *Amaranthus spinosus* L. has spiny hairs on the inflorescence which are absent in this sample. As a result, using an integrative approach the identification of this sample would be *Amaranthus hybridus* L. For a full example of this process see S1 Text.

## Results

### Sequencing success and BLAST matching

The amplification success of market samples for nrITS was 96% (65 samples). However, 17 samples (25%) yielded sequences of fungal DNA due to contamination of the original market samples. After exclusion of these contaminated samples the sequencing success rate for nrITS was 71% (48). The amplification success of market samples for the *trn*L-F spacer was also 96% (65 samples). For the *trn*L-F spacer five products (7%) failed to yield usable sequences, and thus the sequencing success rate for the *trn*L-F spacer was 88% (60 samples). Out of the 68 samples, there were 40 with both nrITS and *trn*L-F spacer sequences, 20 with only the *trn*L-F spacer and 8 with only nrITS.

The simple and optimized BLAST results based on sequence matching as well as the putative species identification for each of the 68 tested samples are included in S3 Table. The identification success was dependent on the marker and availability of reference sequences in GenBank. For some putative species, reference sequences in GenBank were available for only one of the two markers. The BLAST sequence matching method included 60 *trn*L-F spacer and 48 nrITS query sequences. The simple *trn*L-F spacer BLAST search results identified 18% (11 samples) to species level, 53% (32) to genus level, and 28% (17) to family level. The optimized *trn*L-F spacer BLAST search results identified 33% (20 samples) to species level, 45% (27) to genus level, and 16% (10) to family level and 5% (3) could not be identified. The simple nrITS spacer BLAST search results identified 35% (17 samples) to species level, 58% (28) to genus level, and 6% (3) to family level. The optimized nrITS spacer BLAST search results identified 37% (18 samples) to species level, 50% (24) to genus level, and 6% (3) to family level and 6% (3) could not be identified. Combined data from both markers using the simple BLAST sequence matching method identified 32% (22 samples) to species level, 47% (32) to genus level, and 21% (14) to family level. Combined data from both markers using the optimized methods identified 38% (26 samples) to species level, 40% (27) to genus level, and 19% (13) to family level and 3% (2) could not be identified.

**Fig 1:**
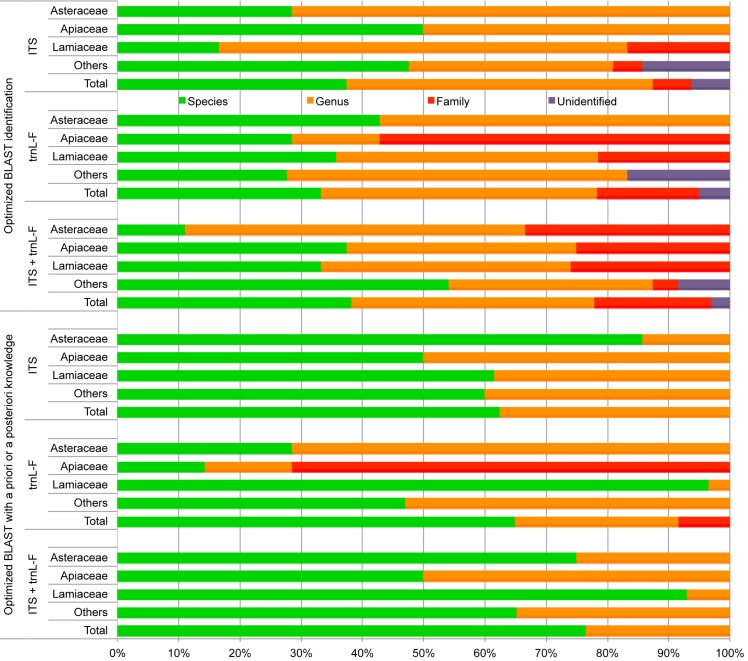
Comparison of identification success rates among different plant families 1) when relying on sequence matching alone, and 2) when *a priori* and *a posteriori* information is incorporated in the identification process for each of the two markers separately and when they are combined.

The integrative approach coalesces sequence matching results with *a priori* and *a posteriori* data. This approach resulted in the identification of 65% (39 samples) to species level, 27% (16) to genus level, and 8% (5) to family level for the *trn*L-F spacer. For the second marker, nrITS, the integrative approach resulted in the identification of all samples to either species or genus level, with 62% (30 samples) identified to species level, and 38% (18) identified to genus level. Combining data from both markers resulted in 77% (52 samples) species level identification and 23% (16) genus level identification. Fig 1 shows the results for the two sequence matching approaches: simple and optimized sequence matching; and the integrative approach where *a priori* and *a posteriori* information is incorporated in the identification process. Evaluating both methods, the integrative approach gives a 1.67, 1.95 and 2.00 fold increase in species level identification rates for nrITS, the *trn*L-F spacer, and combined markers respectively (Table 1).

**Table 1:** Comparison of species level identification rates for optimized BLAST similarity-based and those using the outlined integrative approach.

Sequencing success (out of 68)		Species level identification						
		Simple BLAST		Optimized BLAST			Integrative approach	
Marker	No.	Absolute	Relative	Absolute	Relative		Absolute	Relative
nrITS	48	17	35%	18	38%		30	63%
*trn*L-F	60	11	18%	20	33%		39	65%
*trn*L-F + ITS	68	22	32%	26	38%		52	76%
Sequencing success (out of 68)		Comparing approaches						
		Integrative approach improvement from simple BLAST			Integrative approach improvement from optimized BLAST			
Marker	No.	Absolute	Relative	Increase	Absolute	Relative		Increase
nrITS	48	13	27%	1.76	12	25%		1.67
*trn*L-F	60	28	47%	3.55	19	32%		1.95
*trn*L-F + ITS	68	30	44%	2.36	26	38%		2.00

## Discussion

### DNA barcode identification, morphology and herbal pharmacopeia

Fifty-eight of the 68 samples that could not be identified to species-level based on morphology, were identified to genus and another ten to family only. Applying the integrative approach outlined here, 52 (76%) of these samples could be assigned a species-level identification (Table 1, Fig 1 and S3 Table). Combining this integrative approach with the CBOL PWG recommended barcoding markers *rbc*L and *mat*K [47] in addition to nrITS could have further increased the identification rate. In this study we choose not to include these as *rbc*L has low sequence variation [6,63] and *mat*K has low primer universality [3,53,54]. Primer universality is important when identifying unknown species such as biodiversity samples or market products, but could be mitigated using a tiered amplification approach with multiple primers sets. Different studies have reported different species identification rates for the combination of *rbc*L and *mat*K, but the most comprehensive study to date reports an identification rate of as low as 49.7% [6]. Two approaches that are relevant for the type of degraded and contaminated material included in this study that could have improved amplification success and limited amplification of fungal contaminants would have been targeting the shorter nrITS2 fragment of nrITS and the use of novel plant specific primers such as those published by Cheng et al. [64].

**Fig 2:**
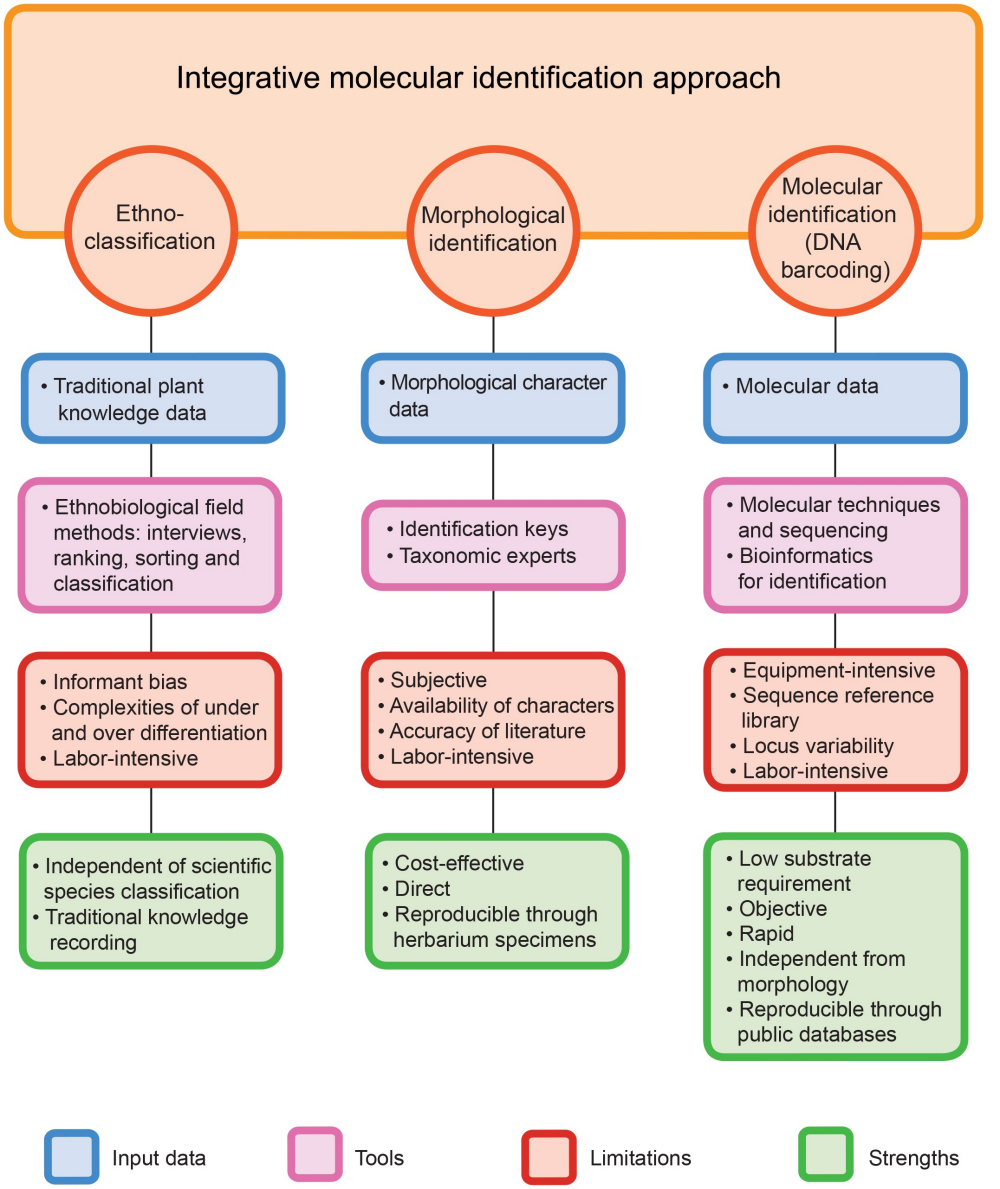
The strengths of DNA barcoding outweigh its weaknesses, but an integrative approach is necessary to optimize identification of unknown plant material.

The identification results from DNA barcoding compared with putative species names derived from herbal pharmacopeia showed inconsistency. Agreement in identification at the species level between the herbal pharmacopoeia and the integrative barcoding approach was found in 71% of cases (48 samples), whereas in 24% (18 samples) identification using herbal pharmacopoeia resulted in erroneous identifications (S6 Table). It should be noted however, that matches were higher at genus and family level, respectively 72% and 89%. It is unlikely that these mismatches are due to incomplete sequence reference libraries as accessions of most species and all genera from the herbal pharmacopoeia assignments were present in NCBI GenBank. These findings imply that ethnopharmacological studies should be careful about relying on herbal pharmacopoeia as this may result in incorrect identifications. Accurate species identification of samples helps ethnopharmacologists and ethnobotanists to relate ethnobiological information about species to scientific literature for further research. Most studies on molecular identification of herbal products focus on specific families and genera because building a comprehensive sequence reference database is more feasible for defined groups than for entire families or all plants [12,75,76]. However, when dealing with completely unknown samples from a broad range of taxa, incomplete reference database coverage can be problematic. In such cases the best way to overcome this problem is an integrative approach in which samples are identified using a total evidence approach that includes multi-marker DNA barcoding, morphology, distribution, pharmacopoeias, and literature. The results from molecular identification of samples have a low identification rate if only BLAST is used and no other evidence is incorporated. However, this can be augmented using an integrative approach (Table 1 and Fig 1). Fig 2 outlines the integrative approach, as used in this study, such that it can be adopted for similar investigations that aim to identify samples of unknown taxonomic identity or authentication of herbal products by pharmaceutical manufacturers or pharmaceutical quality control agencies (see S1 Text for an example). Combining the three identification methods used in this study (sequence similarity matching, morphological classification, and ethnoclassification matching) in an integrative way enables single species assignments for 52 out of 68 samples, whereas unambiguous species assignments are only possible in 22–26 samples using similarity matching (Table 1). It must be noted that these identifications cannot be verified independently. However the objective here was to see whether a combination of similarity matching and *a priori* and *a posteriori* data could reduce ambiguity and enable limiting the number of putative species to one and thus a species level identification.

### Substitution and adulteration

The total evidence results revealed that 26% (18 samples) of the medicinal plants sampled from the markets did not match the intended species recorded in the herbal pharmacopeia [68] (S6 Table). These results could suggest that the species intended for medicinal use, as identified by the herbal pharmacopeia, are substituted locally for other species within the same genus or for different species altogether. Alternatively, the traditional products may consist of different species than those mentioned in the pharmacopoeia. Similar research in Morocco also showed a high level of substitution and suggested that, in addition to the options above, gradual substitution over time of one species for another could explain the discrepancy [9,22]. Some examples of cases where DNA barcoding showed samples to be completely different from the putative species in this study are “Marzeh” and “Maryam goli.” The vernacular name “Marzeh” refers to either *Satureja laxiflora* C.Koch or *S*. *hortensis* L. (Lamiaceae) according to the literature, but molecular identification showed samples with this name to be *Urtica dioica* L. (Urticaceae), an unrelated species from a different family. “Maryam goli” refers to either *Salvia sclarea* L. or *S*. *officinalis* L. (Lamiaceae) based on herbal pharmacopoeias [67,68], but molecular identification showed the sample analyzed under this name to be *Althaea cannabina* L. (Malvaceae).

In this study, we applied DNA barcoding only to samples lacking morphological characteristics for identification. One can assume that identification of these plant products is similarly challenging for traders and consumers. Quite a few samples (6%) belonged to families other than those expected based on the putative identifications, and many more (27%) belonged to different genera than those expected, within the same family. S6 Table lists samples where family and genus identifications using the integrative molecular identification approach did not match putative species identifications from the official herbal pharmacopeia. A list of all samples and identifications are given in S3 Table.

## Conclusions

The present study shows that molecular identification through DNA barcoding is a very useful tool for the identification of traded medicinal plant products originating from a wide range of taxonomic groups. The identification rates of samples that were unidentifiable by morphology alone show the importance of having a complete sequence reference databases for DNA barcoding. Species assignments using DNA barcoding are limited by the comprehensiveness of the sequence reference database, and at the moment no such complete database exists. It is essential to support efforts by iBOL [International Barcode of Life (http://www.ibol.org)], CBOL [Consortium for the Barcode of Life (http://www.barcodeoflife.org)], GBIF [Global Biodiversity Information Facility (http://www.gbif.org)] and NCBI GenBank [National Center for Biotechnology Information (http://www.ncbi.nlm.nih.gov)] to expand barcode reference databases. Identification of plant material does not necessarily need to rely on sequence matching alone, but can also take into account *a priori* data from morphological characteristics of the material, interview data on vernacular names and traditional knowledge, studies of old and current literature, and pharmacopeias for these names, and *a posteriori data* from multiple molecular markers and data on traditional use, flowering time, occurrence and distribution of putative species. The evaluation of the sequence matching approaches shows that DNA barcode identification rates can be enhanced by relying on an integrative approach that combines *a priori* and *a posteriori* data. Although these identifications cannot be verified independently, the method shows that a combination of similarity matching and *a priori* and *a posteriori* data reduces ambiguity and make it possible to assign a single species identification to an increased number of samples. These results do not advocate a novel method of identification, but rather highlight the risk of using automated identification based on sequence similarity for identification of unknown material. As DNA barcoding becomes more and more mainstream, researchers and non-academic professionals without a strong background in the studied organism group, rely on automated identifications without taking other evidence into account. Other resources and data for proper identification require expertise in taxonomy and manual intervention in the identification process. This means that a person should have a good overview of the plant group that they are working on for samples under investigation.

## Supporting information

S1 Table List of herbal shops and their locations visited for this study in Iran. (PDF)

S2 Table Medicinal plant samples from Northern Khorasan province, Iran. (PDF)

S3 Table List of samples, vernacular names, putative species identification, Genbank accession numbers, simple and optimized BLAST similarity identifications and final identifications based on the integrative approach. (PDF)

S4 Table Optimized BLAST similarity identifications. BLAST hits sorted by max score*(identity/cover). Colored by deviation (d) from highest hit: d = < 1%: green, 1% < d = < 2%: orange, 2% < d = < 3%: red, 3% < d: no color.
(PDF)

S5 Table Optimized BLAST similarity identifications per sample. Identifications are assigned based on a combination of the identity score (High identity: i ≥ 95%; Medium identity: 90% ≤ i < 95%; Low identity: i < 90%) and the number of species within 1% deviation of the calculated similarity score.
(PDF)

S6 Table Samples for which family and genus identifications based on DNA barcoding results did not match putative species identifications from the official herbal pharmacopeia. (PDF)

S1 Text An example illustrating the identification process using the integrative approach coalescing *a priori* and *a posteriori* data. (PDF)
