# Supplementary material for: Unidentifiable by morphology: DNA barcoding of plant material in local markets in Iran
Source: PLoS One. 2017 Apr 18;12(4):e0175722. doi: 10.1371/journal.pone.0175722 (PMC5395179; doi:10.1371/journal.pone.0175722)
Supplement: S1 Table — (PDF) [file pone.0175722.s001.pdf]

S1 Table. List of herbal shops and their locations visited for this study in Iran.

ID	Herbal Shop Name	Location	Gender of informant	Number of samples purchased
1	Bakhshi	Esfarayen	Male	28
2	Balut	Esfarayen	Male	8
3	Kimiya	Ashkhaneh	Female	29
4	Giyahestan Mohamad	Ashkhaneh	Male	13
5	Yadegar Pedar	Ashkhaneh	Male	8
6	Iran	Bojnurd	Male	11
7	Saadat	Bojnurd	Female	16
8	Kamali	Bojnurd	Male	8
9	Par Siyavash	Bojnurd	Male	7
10	Ale-Taha	Bojnurd	Male	9
11	Sina	Jajarm	Male	14
12	Asghari	Shirvan	Male	7
13	Hakim	Shirvan	Male	23
14	Ghuchani	Shirvan	Male	14
15	Avishan	Faruj	Male	11
16	Armaghan Tabiaat	Faruj	Male	7
17	Jalali	Garmeh	Male	16
