# Supplementary material for: Unidentifiable by morphology: DNA barcoding of plant material in local markets in Iran
Source: PLoS One. 2017 Apr 18;12(4):e0175722. doi: 10.1371/journal.pone.0175722 (PMC5395179; doi:10.1371/journal.pone.0175722)
Supplement: S2 Table — (PDF) [file pone.0175722.s002.pdf]

S2 Table. Medicinal plant samples from Northern Khorasan province, Iran.

Collection No.	Family	Species	Identification	
			Morphology	Integrative approach
kh001	Apiaceae	*Cuminum cyminum* L.		✓
kh002	Plantaginaceae	*Plantago major* L.	✓	
kh003	Lamiaceae	*Nepeta racemosa* Lam.		✓
kh004	Lamiaceae	*Satureja* sp.		✓
kh005	Lamiaceae	*Ziziphora clinopodioides* Lam.	✓	
kh006	Rosaceae	*Rosa* sp.	✓	
kh007	Boraginaceae	*Echium* sp.		✓
kh008	Lamiaceae	*Thymus serpyllum* L.		✓
kh009	Malvaceae	*Alcea* sp.		✓
kh010	Lamiaceae	*Hymenocrater bituminosus* Fisch. & C.A.Mey.		✓
kh011	Asteraceae	*Anthemis cotula* L.		✓
kh012	Malvaceae	*Althaea cannabina* L.		✓
kh013	Fabaceae	*Glycyrrhiza glabra* L.	✓	
kh014	Poaceae	*Zea mays* L.	✓	
kh015	Lamiaceae	*Ferula* cf. *gummosa* Boiss.	✓	
kh016	Apiaceae	*Thymus serpyllum* L.		✓
kh017	Asteraceae	*Achillea millefolium* L.		✓
kh018	Lamiaceae	*Stachys* sp.		✓
kh019	Lamiaceae	*Teucrium polium* L.		✓
kh020	Lamiaceae	*Ziziphora tenuior* L.		✓
kh021	Malvaceae	*Althaea cannabina* L.		✓
kh022	Fabaceae	*Glycyrrhiza glabra* L.	✓	
Kh023	Lamiaceae	*Teucrium polium* L.		✓
Kh024	Lamiaceae	*Thymus serpyllum* L.		✓
Kh025	Asteraceae	*Carthamus tinctorius* L.		✓
Kh026	Apiaceae	*Foeniculum vulgare* Mill.	✓	
Kh027	Rosaceae	*Rosa* x *damascena* Herrm.	✓	
Kh028	Hypericaceae	*Hypericum scabrum* L.		✓
Kh029	Moraceae	*Morus nigra* L.	✓	
Kh030	Ericaceae	*Vaccinium* sp.	✓	
Kh031	Solanaceae	*Physalis alkekengi* L.		✓
Kh032	Ephedraceae	*Ephedra intermedia* Schrenk & C.A.Mey.		✓
Kh033	Lamiaceae	*Vitex agnus-castus* L.		✓
kh034	Oleaceae	*Fraxinus excelsior* L.		✓
kh035	Pedaliaceae	*Sesamum indicum* L.	✓	
kh036	Violaceae	*Viola alba* Besser		✓
kh037	Elaeagnaceae	*Elaeagnus angustifolia* L.	✓	
kh038	Apiaceae	*Cuminum cyminum* L.		✓
kh039	Plantaginaceae	*Plantago major* L.		✓
kh040	Nitrariaceae	*Peganum harmala* L.	✓	
kh041	Lamiaceae	*Teucrium polium* L.	✓	
kh042	Rhamnaceae	*Ziziphus jujuba* Mill.	✓	
kh043	Asteraceae	*Carthamus tinctorius* L.	✓	
kh044	Berberidaceae	*Berberis integerrima* Bunge		✓
kh045	Apiaceae	*Bunium* sp.		✓
kh046	Lamiaceae	*Stachys lavandulifolia* Vahl	✓	
kh047	Rosaceae	*Rosa* x *damascena* Herrm.	✓	
kh048	Apiaceae	*Zosima absinthifolia* Link		✓
kh049	Rosaceae	*Prunus dulcis* (Mill.) D.A.Webb	✓	
kh050	Leguminosae	*Alhagi maurorum* Medik.	✓	
kh051	Lamiaceae	*Hymenocrater bituminosus* Fisch. & C.A.Mey.		✓
kh052	Rosaceae	*Cydonia oblonga* Mill.	✓	
kh053	Lamiaceae	*Mentha arvensis* L.		✓
kh054	Lamiaceae	*Ziziphora clinopodioides* Lam.	✓	
kh055	Lamiaceae	*Nepeta menthoides* Boiss. & Buhse		✓
kh056	Amarylidaceae	*Allium atroviolaceum* Boiss.		✓
kh057	Lamiaceae	*Thymus daenensis* Celak.		✓
kh058	Asteraceae	*Achillea biebersteinii* Hub.-Mor.		✓

kh059	Malvaceae	*Alcea* sp.		✓
kh060	Leguminosae	*Glycyrrhiza* glabra L.	✓	
kh061	Nitrariaceae	*Peganum harmala* L.	✓	
kh062	Lamiaceae	*Mentha spicata* L.		✓
kh063	Asteraceae	*Tripleurospermum* sp.		✓
kh064	Lamiaceae	*Ziziphora clinopodioides* Lam.	✓	
kh065	Lamiaceae	*Thymus serpyllum* L.		✓
kh066	Lamiaceae	*Teucrium polium* L.		✓
kh067	Lamiaceae	*Hymenocrater bituminosus* Fisch. & C.A.Mey.		✓
kh068	Malvaceae	*Alcea* sp.		✓
kh069	Lamiaceae	*Thymus serpyllum* L.		✓
kh070	Lamiaceae	*Satureja hortensis* L.		✓
kh071	Apiaceae	*Bunium* sp.		✓
kh072	Leguminosae	*Glycyrrhiza glabra* L.	✓	
kh073	Lamiaceae	*Teucrium polium* L.		✓
kh074	Lamiaceae	*Perovskia atriplicifolia* Benth.		✓
kh075	Lamiaceae	*Ziziphora tenuior* L.		✓
kh076	Lamiaceae	*Tanacetum parthenium* (L.) Sch.Bip.		✓
kh077	Scrophulariaceae	*Verbascum* sp.	✓	
kh078	Rosaceae	*Prunus avium* (L.) L.	✓	
kh079	Lamiaceae	*Stachys lavandulifolia* Vahl	✓	
kh080	Fumariaceae	*Fumaria parviflora* Lam.	✓	
kh081	Nitrariaceae	*Peganum harmala* L.	✓	
kh082	Lamiaceae	*Hymenocrater bituminosus* Fisch. & C.A.Mey.		✓
kh083	Pteridaceae	*Adiantum capillus-veneris* L.	✓	
kh084	Zygophyllaceae	*Tribulus terrestris* L.	✓	
kh085	Berberidaceae	*Berberis integerrima* Bunge	✓	
kh086	Lamiaceae	*Nepeta menthoides* Boiss. & Buhse	✓	
kh087	Apiaceae	*Heracleum persicum* Desf. ex Fisch., C.A.Mey. & Avé-Lall.	✓	
kh088	Poaceae	*Zea mays* L.	✓	
kh089	Asteraceae	*Achillea beibersteinii* Afan.	✓	
kh090	Lamiaceae	*Satureja mutica* Fisch. & C.A.Mey.		✓
kh091	Asteraceae	*Cichorium intybus* L.	✓	
kh092	Asteraceae	*Carthamus tinctorius* L.	✓	
kh093	Urticaceae	*Urtica dioica* L.	✓	
kh094	Leguminosae	*Astragalus hamosus* L.	✓	
kh095	Asteraceae	*Tanacetum* sp.		✓
kh096	Juglandaceae	*Juglans regia* L.	✓	
kh097	Malvaceae	*Malva sylvestris* L.	✓	
kh098	Brassicaceae	*Descurainia sophia* (L.) Webb ex Prantl	✓	
kh099	Oleaceae	*Olea europaea* L.	✓	
kh100	Hypericaceae	*Hypericum scabrum* L.	✓	
kh101	Apiaceae	*Ferula* sp.	✓	
kh102	Papaveraceae	*Papaver bracteatum* Lindl.		✓
kh103	Rosaceae	*Rosa × damascena* Herrm.	✓	
kh104	Leguminosae	*Glycyrrhiza glabra* L.	✓	
kh105	Malvaceae	*Alcea* sp.	✓	
kh106	Nitrariaceae	*Peganum harmala* L.	✓	
kh107	Brassicaceae	*Descurainia sophia* (L.) Webb ex Prantl	✓	
kh108	Lamiaceae	*Teucrium polium* L.		✓
kh109	Fabaceae	*Glycyrrhiza glabra* L.	✓	
kh110	Asteraceae	*Achillea vermicularis* Trin.		✓
kh111	Amaranthaceae	*Amaranthus hybridus* L.		✓
kh112	Lamiaceae	*Ziziphora tenuior* L.		✓
kh113	Boraginaceae	*Echium* sp.		✓
kh114	Malvaceae	*Alcea* sp.		✓
kh115	Apiaceae	*Bunium* sp.		✓
kh116	Berberidaceae	*Berberis integerrima* Bunge		✓
kh117	Urticaceae	*Urtica dioica* L.		✓
kh118	Fabaceae	*Astragalus* sp.		✓
kh119	Apiaceae	*Ferula* sp.	✓	
Kh120	Rhamnaceae	*Ziziphus jujuba* Mill.	✓	

Kh121	Lamiaceae	*Stachys lavandulifolia* Vahl	✓	
Kh122	Poaceae	*Zea mays* L.	✓	
Kh123	Rosaceae	*Prunus avium* (L.) L.	✓	
Kh124	Rosaceae	*Cydonia oblonga* Mill.	✓	
Kh125	Elaeagnaceae	*Elaeagnus angustifolia* L.	✓	
Kh126	Rosaceae	*Prunus armeniaca* L.	✓	
kh127	Brassicaceae	*Descurainia sophia* (L.) Webb ex Prantl	✓	
kh128	Brassicaceae	*Alyssum homalocarpum* (Fisch. & C.A.Mey.) Boiss.	✓	
kh129	Lamiaceae	*Ziziphora clinopodioides* Lam.	✓	
kh130	Rhamnaceae	*Ziziphus jujuba* Mill.	✓	
kh131	Rosaceae	*Cydonia oblonga* Mill.	✓	
kh132	Leguminosae	*Astragalus* sp.		✓
kh133	Lamiaceae	*Zataria multiflora* Boiss.		✓
kh134	Rosaceae	*Rosa × damascena* Herrm.	✓	
kh135	Malvaceae	*Malva nicaeensis* All.		✓
kh136	Rosaceae	*Prunus avium* (L.) L.	✓	
kh137	Lamiaceae	*Stachys lavandulifolia* Vahl	✓	
kh138	Apiaceae	*Bunium* sp.		✓
kh139	Apiaceae	*Foeniculum vulgare* Mill.	✓	
kh140	Apiaceae	*Cuminum cyminum* L.	✓	
kh141	Apiaceae	*Pimpinella anisum* L.		✓
kh142	Apiaceae	*Coriandrum sativum* L.	✓	
kh143	Apiaceae	*Cuminum cyminum* L.	✓	
kh144	Lamiaceae	*Satureja mutica* Fisch. & C.A.Mey.	✓	
kh145	Apiaceae	*Trachyspermum ammi* (L.) Sprague	✓	
kh146	Lamiaceae	*Ziziphora clinopodioides* Lam.	✓	
kh147	Apiaceae	*Anethum graveolens* L.	✓	
kh148	Ranunculaceae	*Nigella sativa* L.	✓	
kh149	Apiaceae	*Foeniculum vulgare* Mill.	✓	
kh150	Asteraceae	*Achillea beibersteinii* Afan.	✓	
kh151	Boraginaceae	*Onosma* sp.	✓	
kh152	Lamiaceae	*Draeocephalum* sp.	✓	
kh153	Fabaceae	*Senna italica* Mill.	✓	
kh154	Lamiaceae	*Teucrium polium* L.	✓	
kh155	Lamiaceae	*Stachys lavandulifolia* Vahl	✓	
kh156	Papaveraceae	*Fumaria parviflora* Lam.	✓	
kh157	Boraginaceae	*Echium amoenum* Fisch. & C. A. Mey	✓	
kh158	Apiaceae	*Ferula cf. gummosa* Boiss.	✓	
kh159	Nitrariaceae	*Peganum harmala* L.	✓	
kh160	Apiaceae	*Heracleum persicum* Desf. ex Fisch., C.A.Mey. & Avé-Lall.	✓	
kh161	Urticaceae	*Urtica dioica* L.	✓	
kh162	Lamiaceae	*Nepeta menthoides* Boiss. & Buhse	✓	
kh163	Malvaceae	*Malva sylvestris* L.	✓	
kh164	Asteraceae	*Achillea arabica* Kotschy	✓	
kh165	Lamiaceae	*Ziziphora clinopodioides* Lam.	✓	
kh166	Fabaceae	*Glycyrrhiza glabra* L.	✓	
kh167	Lamiaceae	*Nepeta menthoides* Boiss. & Buhse	✓	
kh168	Lamiaceae	*Zataria multiflora* Boiss.	✓	
kh169	Apiaceae	*Bunium persicum* (Boiss.) B.Fedtsch.	✓	
kh170	Nitrariaceae	*Peganum harmala* L.	✓	
kh171	Berberidaceae	*Berberis integerrima* Bunge	✓	
kh172	Lamiaceae	*Ziziphora clinopodioides* Lam.	✓	
kh173	Malvaceae	*Malva sylvestris* L.	✓	
kh174	Malvaceae	*Alcea kurdica* (Schlescht.) Alef.	✓	
kh175	Asteraceae	*Cichorium intybus* L.	✓	
kh176	Zygophyllaceae	*Tribulus terrestris* L.	✓	
kh177	Lamiaceae	*Ziziphora* sp.	✓	
kh178	Malvaceae	*Malva sylvestris* L.	✓	
kh179	Papaveraceae	*Fumaria parviflora* Lam.	✓	
kh180	Asteraceae	*Anthemis cotula* L.	✓	
kh181	Fabaceae	*Astragalus* sp.	✓	
kh182	Apiaceae	*Bunium persicum* (Boiss.) B.Fedtsch.	✓	

kh183	Apiaceae	*Cuminum cyminum* L.	✓	
kh184	Lamiaceae	*Mentha longifolia* (L.) L.	✓	
kh185	Plantaginaceae	*Plantago ovata* Forssk.	✓	
kh186	Lamiaceae	*Teucrium polium* L.	✓	
kh187	Urticaceae	*Urtica dioica* L.	✓	
kh188	Alliaceae	*Allium stipitatum* Regel	✓	
kh189	Malvaceae	*Alcea kurdica* (Schlescht.) Alef.	✓	
kh190	Lamiaceae	*Stachys lavandulifolia* Vahl	✓	
kh191	Poaceae	*Zea mays* L.	✓	
kh192	Lamiaceae	*Ziziphora clinopodioides* Lam.	✓	
kh193	Lamiaceae	*Perovskia abrotanoides* Kar.	✓	
kh194	Lamiaceae	*Hymenocrater bituminosus* Fisch. & C.A.Mey.	✓	
kh195	Oleaceae	*Fraxinus excelsior* L.	✓	
kh196	Malvaceae	*Alcea* sp.	✓	
kh197	Lamiaceae	*Nepeta* sp.	✓	
kh198	Apiaceae	*Cuminum cyminum* L.	✓	
kh199	Malvaceae	*Alcea* sp.	✓	
kh200	Brassicaceae	*Descurainia sophia* (L.) Webb ex Prantl	✓	
kh201	Portulacaceae	*Portulaca oleracea* L.	✓	
kh202	Papaveraceae	*Fumaria parviflora* Lam.	✓	
kh203	Asteraceae	*Achillea millefolium* L.	✓	
kh204	Elaeagnaceae	*Elaeagnus angustifolia* L.	✓	
kh205	Rosaceae	*Rosa × damascena* Herrm.	✓	
kh206	Amaranthaceae	*Amaranthus hybridus* L.	✓	
kh207	Fabaceae	*Astragalus* sp.	✓	
kh208	Fabaceae	*Glycyrrhiza glabra* L.	✓	
kh209	Poaceae	*Zea mays* L.	✓	
kh210	Berberidaceae	*Berberis integerrima* Bunge	✓	
kh211	Malvaceae	*Malva sylvestris* L.	✓	
kh212	Fabaceae	*Glycyrrhiza glabra* L.	✓	
kh213	Asteraceae	*Achillea arabica* Kotschy	✓	
kh214	Apiaceae	*Cuminum cyminum* L.	✓	
kh215	Lamiaceae	*Teucrium polium* L.	✓	
kh216	Lamiaceae	*Stachys lavandulifolia* Vahl	✓	
kh217	Asteraceae	*Anthemis cotula* L.	✓	
kh218	Rosaceae	*Rosa × damascena* Herrm.	✓	
kh219	Fabaceae	*Astragalus* sp.	✓	
kh220	Lamiaceae	*Hymenocrater bituminosus* Fisch. & C.A.Mey.	✓	
kh221	Pteridaceae	*Adiantum capillus-veneris* L.	✓	
kh222	Apiaceae	*Ferula* sp.	✓	
kh223	Poaceae	*Zea mays* L.	✓	
kh224	Boraginaceae	*Onosma* sp.	✓	
kh225	Plantaginaceae	*Plantago ovata* Forssk.	✓	
kh226	Rosaceae	*Rosa × damascena* Herrm.	✓	
kh227	Amaranthaceae	*Amaranthus hybridus* L.	✓	
kh228	Apiaceae	*Cuminum cyminum* L.	✓	
kh229	Poaceae	*Zea mays* L.	✓	
		Total	**161**	**68**
