# Supplementary material for: Unidentifiable by morphology: DNA barcoding of plant material in local markets in Iran
Source: PLoS One. 2017 Apr 18;12(4):e0175722. doi: 10.1371/journal.pone.0175722 (PMC5395179; doi:10.1371/journal.pone.0175722)
Supplement: S3 Table — (PDF) [file pone.0175722.s003.pdf]

S3 Table. List of samples, vernacular names, Genbank accession numbers, putative species identification, simple and optimized BLAST results and final identifications based on the integrative approach.

Voucher	Plant part	Vernacular name	Family	Scientific name based on morphology	Putative scientific name based on matching vernacular names with scientific literature	Sequence in GenBank for ITS	Sequence in GenBank for *trn*L-F	Deposited ITS GenBank accessions	Deposited trn*L-F* GenBank accessions	Simple BLAST approach			Optimized BLAST approach			Integrative approach				
										ITS	*trn*L-F	ITS and *trn*L-F combined	ITS	*trn*L-F	ITS and *trn*L-F combined	BLAST ITS with *a priori* & *a posteriori* data	GB matches V=vouchered U=unvouchered	BLAST *trn*L-F with *a priori* & *a posteriori* data	GB matches V=vouchered U=unvouchered	ITS + *trn*L-F + Morphology
Kh001	Seed	Zireh sabz	Apiaceae	*Cuminum* sp.	*Cuminum cyminum* L.	Species	Family	KR150163	KR150213	*Cuminum cyminum* L.	Apiaceae	*Cuminum cyminum* L.	*Cuminum cyminum* L.	*Osmorhiza bipatriata* Constance & Shan	Apiaceae	*Cuminum cyminum* L.	KF160677 (V)	*Anthriscus cerefolium* (L.) Hoffm.	GU456628 (U)	*Cuminum cyminum* L.
Kh003	Crushed aerial parts	Ostokhodoos	Lamiaceae	*Nepeta* sp.	*Stachys* ssp., *Nepeta menthoides* Boiss. & Buhse, *Lavandula* ssp.	G, S, G	G, S, G	KR150166	KR150216	*Nepeta* sp.	*Nepeta* sp.	*Nepeta* sp.	*Nepeta* sp.	*Nepeta racemosa* Lam.	*Nepeta racemosa* Lam.	*Nepeta glomerulosa* Boiss.	AJ515317 (U)	*Nepeta racemosa* Lam.	AJ505432 (V)	*Nepeta racemosa* Lam.
Kh004	Leaves	Marzeh	Lamiaceae	*Satureja* sp.	*Satureja laxiflora* C.Koch	Genus	Genus	KR150164	KR150214	*Satureja montana* L.	*Satureja* sp.	*Satureja montana* L.	*Satureja* sp.	*Satureja* sp.	*Satureja* sp.	*Satureja hortensis* L.	AY227143 (U)	*Satureja mutica* Fisch. & C.A.Mey.	GU381619 (V)	*Satureja* sp.
Kh007	Flowers (petals)	Gole gavzaban	Boraginaceae	*Echium* sp.	*Echium amoenum* Fisch. & C. A. Mey., *Borago officinalis* L.	Genus, Species	Genus, Species	KR150161	KR150210	*Echium* sp.	Boraginaceae	*Echium* sp.	*Echium* sp.	*Echium* sp.	*Echium* sp.	*Echium* sp.	-	*Echium* sp.	-	*Echium* sp.
Kh008	Leaves	Marzanjoosh	Lamiaceae	*Thymus* sp.	*Origanum vulgare* L.	Species	Species	KR150165	KR150215	*Thymus* sp.	*Thymus* sp.	*Thymus* sp.	*Thymus* sp.	*Thymus* sp.	*Thymus* sp.	*Thymus serpyllum* L.	DQ667242 (U)	*Thymus serpyllum* L.	AJ505544 (V)	*Thymus serpyllum* L.
Kh009	Flower & seeds	Khatmi sefid	Malvaceae	*Alcea* sp.	*Alcea lavateriflora* (DC.) Boiss., *Alcea digitata* Alef.	Species, Genus	Species, Genus	KR150167	KR150217	*Alcea* sp.	*Alcea* sp.	*Alcea* sp.	Unidentified	Unidentified	Unidentified	*Alcea* sp.	-	*Alcea* sp.	-	*Alcea* sp.
kh010	Flower & leaves	Badranjbooyeh	Lamiaceae	*Hymenocrater* sp.	*Dracocephalum moldavica* L., *Asperugo procumbens* L., *Clinopodium nepeta* subsp. *glandulosum* (Req.) Govaerts, *Melissa officinalis* L.	S, S, G, S	S, S, S, S	-	KR150211	-	Lamiaceae	Lamiaceae	-	*Hymenocrater bituminosus* Fisch. & C.A.Mey.	*Hymenocrater bituminosus* Fisch. & C.A.Mey.	-	-	*Hymenocrater bituminosus* Fisch. & C.A.Mey.	JQ669045 (V)	*Hymenocrater bituminosus* Fisch. & C.A.Mey.
Kh011	Crushed flowers	Babooneh	Asteraceae	*Anthemis* sp.	*Matricaria recutita* L., *Matricaria* sp.	Species	Genus	KR150162	KR150212	*Tanacetum* sp.	*Chrysanthemum indicum* L.	Asteraceae	*Anthemis* sp.	*Anthemis* sp.	*Anthemis* sp.	*Anthemis cotula* L.	EU179216 (U)	*Chrysanthemum* sp.	-	*Anthemis cotula* L.
Kh012	Aerial parts powder	Maryam goli	Malvaceae	Malvaceae	*Salvia sclarea* L., *Salvia officinalis* L.	Species, Species	Species, Species	KR150183	-	*Althaea* sp.	-	*Althaea* sp.	*Althaea cannabina* L.	-	*Althaea cannabina* L.	*Althaea* sp.	-	-	-	*Althaea cannabina* L.
Kh016	Leaves	Avishan	Lamiaceae	*Thymus* sp.	*Thymus vulgaris* L., *Thymus serpyllum* L.	Species, Species	Species, Species	KR150173	KR150229	*Thymus* sp.	*Thymus* sp.	*Thymus* sp.	*Thymus* sp.	*Thymus* sp.	*Thymus* sp.	*Thymus* sp.	-	*Thymus serpyllum* L.	AJ505544.1 (V)	*Thymus serpyllum* L.
Kh017	Flowers	Boomadaran	Asteraceae	*Achillea* sp.	*Achillea millefolium* L.	Species	Species	KR150185	KR150244	*Achillea* sp.	*Achillea* sp.	*Achillea* sp.	*Achillea* sp.	*Achillea* sp.	*Achillea* sp.	*Achillea millefolium* L.	EU796891.1 (U)	*Achillea millefolium* L.	EU385030.1 (U)	*Achillea millefolium* L.
Kh018	Crushed flowers	Ostokhodoos	Lamiaceae	*Stachys* sp.	*Lavandula* sp.	Genus	Genus	KR150189	KR150248	*Stachys* sp.	Lamiaceae	*Stachys* sp.	Lamiaceae	Lamiaceae	Lamiaceae	*Stachys* sp.	-	*Phlomis tuberosa* L.	211907851 (V)	*Stachys* sp.
Kh019	Flower & leaves	Kalpooreh	Lamiaceae	*Teucrium* sp.	*Teucrium polium* L.	Species	Species	-	KR150230	-	*Teucrium polium* L.	*Teucrium polium* L.	-	*Teucrium* sp.	*Teucrium* sp.	-	-	*Teucrium polium* L.	JQ044780.1 (U)	*Teucrium polium* L.
Kh020	Leaves	Kakooti	Lamiaceae	*Ziziphora* sp.	*Ziziphora tenuior* L.	Species	Species	-	KR150243	-	*Ziziphora* sp.	*Ziziphora* sp.	-	Lamiaceae	Lamiaceae	-	-	*Ziziphora tenuior* L.	GU381507 (V)	*Ziziphora tenuior* L.
Kh021	Root powder	Charme giyah	Malvaceae	Malvaceae	*Alcea lavateriflora* (DC.) Boiss., *Alcea digitata* Alef.	Species, Genus	Species, Genus	KR150174	-	Althaea sp.	-	*Althaea* sp.	*Althaea armeniaca* Ten.	-	*Althaea armeniaca* Ten.	*Althaea cannabina* L.	EF419540 (U)	-	-	*Althaea cannabina* L.
Kh023	Flower & leaves	Darmaneh, Kalpooreh	Lamiaceae	*Teucrium* sp.	*Teucrium polium* L.	Species	Species	-	KR150224	-	*Teucrium polium* L.	*Teucrium polium* L.	-	*Teucrium* sp.	*Teucrium* sp.	-	-	*Teucrium polium* L.	JQ044780 (U)	*Teucrium polium* L.
Kh024	Leaves	Avishan	Lamiaceae	*Thymus* sp.	*Thymus vulgaris* L., *Thymus serpyllum* L.	Species, Species	Species, Species	KR150171	KR150225	*Thymus* sp.	*Thymus* sp.	*Thymus* sp.	*Thymus* sp.	*Thymus* sp.	*Thymus* sp.	*Thymus* sp.	-	*Thymus serpyllum* L.	AJ505544 (V)	*Thymus serpyllum* L.
Kh025	Seed	Kachireh	Asteraceae	Asteraceae	*Carthamus tinctorius* L.	Species	Species	KR150143	-	*Carthamus tinctorius* L.	-	*Carthamus tinctorius* L.	*Carthamus* sp.	-	*Carthamus* sp.	*Carthamus tinctorius* L.	EF483946 (V)	-	-	*Carthamus tinctorius* L.
Kh028	Crushed flowers	Hofarighoon	Hypericaceae	*Hypericum* sp.	*Hypericum perforatum* L.	Species	Species	KR150172	KR150226	*Hypericum scabrum* L.	*Hypericum* sp.	*Hypericum scabrum* L.	*Hypericum scabrum* L.	Unidentified	*Hypericum scabrum* L.	*Hypericum scabrum* L.	HE653624 (V)	*Hypericum scabrum* L.	KC709053 (U)	*Hypericum scabrum* L.
Kh031	Fruits	Kaganj	Solanaceae	*Physalis* sp.	Unidentified	-	-	KR150190	KR150250	*Physalis alkekengi* L.	*Physalis* sp.	*Physalis alkekengi* L.	Unidentified	*Physalis alkekengi* L.	*Physalis alkekengi* L.	*Physalis alkekengi* L.	AM503883 (U)	*Physalis alkekengi* L.	DQ180420 (U)	*Physalis alkekengi* L.
Kh032	Stem	Homolmajoos	Ephedraceae	*Ephedra* sp.	*Ephedra pachyclada* Boiss.	Species	Family	-	KR150228	-	*Ephedra* sp.	*Ephedra* sp.	-	*Ephedra* sp.	*Ephedra* sp.	-	-	*Ephedra intermedia* Schrenk & C.A.Mey.	AY423430 (U)	*Ephedra intermedia* Schrenk & C.A.Mey.
Kh033	Fruits	Panj angosht	Lamiaceae	*Vitex* sp.	*Vitex* sp.	Genus	Genus	-	KR150227	-	*Verbena officinalis* L.	*Verbena officinalis* L.	-	Unidentified	Unidentified	-	-	*Vitex agnus-castus* L.	HQ412926 (U)	*Vitex agnus-castus* L.
Kh034	Seed	Zaban gonjeshk	Oleaceae	*Fraxinus* sp.	Fraxinus spp.	Genus	Genus	-	KR150249	-	*Fraxinus angustifolia* Vahl.	*Fraxinus angustifolia* Vahl.	-	*Fraxinus* sp.	*Fraxinus* sp.	-	-	*Fraxinus excelsior* L.	LN515485 (V)	*Fraxinus excelsior* L.
Kh036	Flowers	Banafsheh	Violaceae	*Viola* sp.	*Viola odorata* L.	Species	Species	KR150157	KR150205	*Viola alba* Besser	*Viola* sp.	*Viola alba* Besser	*Viola alba* Besser	*Viola* sp.	*Viola alba* Besser	*Viola alba* Besser	HM486499 (V)	*Viola* sp.	-	*Viola alba* Besser
Kh038	Seed	Zireh sabz	Apiaceae	*Cuminum* sp.	*Cuminum cyminum* L.	Species	Family	KR150150	-	*Cuminum cyminum* L.	-	*Cuminum cyminum* L.	*Cuminum cyminum* L.	-	*Cuminum cyminum* L.	*Cuminum cyminum* L.	KF160677 (V)	-	-	*Cuminum cyminum* L.
Kh039	Seed	Barhang	Plantaginaceae	*Plantago* sp.	*Plantago major* L.	Species	Species	KR150184	KR150242	*Plantago* sp.	*Plantago* sp.	*Plantago* sp.	Plantaginaceae	*Plantago* sp.	*Plantago* sp.	*Plantago major* L.	AY101861 (U)	*Plantago major* L.	AY101917 (U)	*Plantago major* L.
Kh044	Fruits	Zereshk koohi	Berberidaceae	*Berberis* sp.	*Berberis vulgaris* L.	Species	Species	KR150149	-	*Berberis weiningensis* T.S.Ying	-	*Berberis weiningensis* T.S.Ying	*Berberis* sp.	-	*Berberis* sp.	*Berberis integerrima* Bunge	JN228267 (U)	-	-	*Berberis integerrima* Bunge
Kh045	Seed	Zireh siyah	Apiaceae	*Bunium* sp.	*Bunium persicum* (Boiss.) B.Fedtsch.	Species	Family	KR150187	KR150247	Apiaceae	Apiaceae	Apiaceae	*Bunium* sp.	Apiaceae	*Bunium* sp.	*Bunium* sp.	-	Apiaceae	-	*Bunium* sp.
Kh048	Seed	Gole roomi	Apiaceae	*Prangos* sp.	*Levisticum officinale* W.D. J Koch	Species	Family	KR150148	KR150197	*Zozima* sp.	Apiaceae	*Zozima* sp.	*Zosima absinthifolia* Link	*Zosima absinthifolia* Link	*Zosima absinthifolia* Link	*Zosima absinthifolia* Link	EU185647 (U)	Apiaceae	-	*Zosima absinthifolia* Link
Kh051	Flower & leaves	Badranjbooyeh	Lamiaceae	*Hymenocrater* sp.	*Dracocephalum moldavica* L., *Asperugo procumbens* L., *Clinopodium nepeta* subsp. *glandulosum* (Req.) Govaerts, *Melissa officinalis* L.	S, S, G, S	S, S, S, S	-	KR150192	-	Lamiaceae	Lamiaceae	-	*Hymenocrater bituminosus* Fisch. & C.A.Mey.	*Hymenocrater bituminosus* Fisch. & C.A.Mey.	-	-	*Hymenocrater bituminosus* Fisch. & C.A.Mey.	JQ669045 (V)	*Hymenocrater bituminosus* Fisch. & C.A.Mey.
Kh053	Leaves	Nana koohi	Lamiaceae	*Mentha* sp.	*Mentha* sp.	Genus	Genus	KR150144	KR150193	*Mentha arvensis* L.	*Mentha* sp.	*Mentha arvensis* L.	Lamiaceae	*Mentha arvensis* L.	*Mentha arvensis* L.	*Mentha arvensis* L.	JQ669115 (V)	*Mentha arvensis* L.	DQ667514 (U)	*Mentha arvensis* L.
Kh055	Inflorescence	Gole zoofa	Lamiaceae	*Nepeta* sp.	*Hyssopus officinalis* L.	Species	Species	-	KR150191	-	*Nepeta menthoides* Boiss. & Buhse	*Nepeta menthoides* Boiss. & Buhse	-	*Nepeta menthoides* Boiss. & Buhse	*Nepeta menthoides* Boiss. & Buhse	-	-	*Nepeta menthoides* Boiss. & Buhse	AJ505431 (V)	*Nepeta menthoides* Boiss. & Buhse

Kh056	Crushed leaves	Piyaze koohi	Alliaceae	Alliaceae	*Allium schoenoprasum* L.	Species	Species	KR150145	KR150194	*Allium atroviolaceum* Boiss.	*Allium* sp.	*Allium atroviolaceum* Boiss.	*Allium atroviolaceum* Boiss.	*Allium* sp.	*Allium atroviolaceum* Boiss.	*Allium atroviolaceum* Boiss.	EU626324 (V)	*Allium* sp.	-	*Allium atroviolaceum* Boiss.
Kh057	Leaves	Avishan, Saetar	Lamiaceae	*Thymus* sp.	*Zataria multiflora* Boiss., *Thymus vulgaris* L., *Thymus serpyllum* L.	S, S, S	S, S, S	KR150146	KR150195	*Thymus daenensis* Celak.	*Origanum* sp.	Lamiaceae	*Thymus* sp.	*Lamiaceae*	Lamiaceae	*Thymus serpyllum* L.	DQ667242 (U)	*Thymus* sp.	-	*Thymus serpyllum* L.
Kh058	Flowers	Boomadaran	Asteraceae	*Achillea* sp.	*Achillea millefolium* L.	Species	Species	KR150147	KR150196	*Achillea* sp.	*Achillea* sp.	*Achillea* sp.	*Achillea* sp.	*Achillea* sp.	*Achillea* sp.	*Achillea biebersteinii* Hub.-Mor.	AY603218 (U)	*Achillea* sp.	-	*Achillea biebersteinii* Hub.-Mor.
kh059	Root powder	Khatmi sefid	Malvaceae	*Alcea* sp.	*Alcea lavateriflora* (DC.) Boiss., *Alcea digitata* Alef.	Species, Genus	Species, Genus	KR150188	-	*Alcea* sp.	-	*Alcea* sp.	*Alcea acaulis* (Cav.) Alef.	-	*Alcea acaulis* (Cav.) Alef.	*Alcea* sp.	-	-	-	*Alcea* sp.
Kh062	Leaves powder	Nana	Lamiaceae	Lamiaceae	*Mentha spicata* L.	Species	Species	-	KR150223	-	*Mentha* sp.	*Mentha* sp.	-	*Mentha* sp.	*Mentha* sp.	-	-	*Mentha spicata* L.	GU381518 (V)	*Mentha spicata* L.
Kh063	Flower & seeds	Babooneh	Asteraceae	*Tripleurospermum* sp.	*Matricaria recutita* L., *Matricaria* sp.	Species	Genus	KR150169	KR150219	*Tripleurospermum* sp.	*Chrysanthemum indicum* L.	*Asteraceae*	*Tanacetum turcomanicum* (Krasch.) Tzvelev	*Artemisia lagocephala* (Fisch. ex Besser) DC.	Asteraceae	*Tripleurospermum* sp.	-	*Chrysanthemum* sp.	-	*Tripleurospermum* sp.
Kh065	Leaves	Marzanjoosh	Lamiaceae	*Thymus* sp.	*Thymus* ssp., *Origanum vulgare* L.	Genus, Species	Genus, Species	-	KR150222	-	*Thymus* sp.	*Thymus* sp.	-	*Thymus* sp.	*Thymus* sp.	-	-	*Thymus serpyllum* L.	AJ505544 (V)	*Thymus serpyllum* L.
Kh066	Flower & leaves	Kalpooreh	Lamiaceae	*Teucrium* sp.	*Teucrium polium* L.	Species	Species	-	KR150221	-	*Teucrium polium* L.	*Teucrium polium* L.	-	*Teucrium* sp.	*Teucrium* sp.	-	-	*Teucrium polium* L.	JQ044780 (U)	*Teucrium polium* L.
Kh067	Flower & leaves	Badranjbooyeh	Lamiaceae	*Hymenocrater* sp.	*Dracocephalum moldavica* L., *Asperugo procumbens* L., *Clinopodium nepeta* subsp. *glandulosum* (Req.) Govaerts, *Melissa officinalis* L.	S, S, G, S	S, S, S, S	-	KR150220	-	Lamiaceae	Lamiaceae	-	Lamiaceae	Lamiaceae	-	-	*Hymenocrater bituminosus* Fisch. & C.A.Mey.	JQ669045 (V)	*Hymenocrater bituminosus* Fisch. & C.A.Mey.
Kh068	Flowers	Gol khatmi	Malvaceae	*Alcea* sp.	*Alcea lavateriflora* (DC.) Boiss., *Alcea digitata* Alef.	Species, Genus	Species, Genus	KR150170	-	*Alcea* sp.	-	*Alcea* sp.	*Alcea* sp.	-	*Alcea* sp.	*Alcea* sp.	-	-	-	*Alcea* sp.
Kh069	Leaves	Avishan	Lamiaceae	*Thymus* sp.	*Thymus vulgaris* L., *Thymus serpyllum* L.	Species, Species	Species, Species	KR150168	KR150218	*Thymus* sp.	*Thymus* sp.	*Thymus* sp.	*Thymus* sp.	*Thymus* sp.	*Thymus* sp.	*Thymus* sp.	-	*Thymus serpyllum* L.	AJ505544 (V)	*Thymus serpyllum* L.
Kh070	Crushed leaves	Marzeh	Lamiaceae	*Satureja* sp.	*Satureja laxiflora* C.Koch	Genus	Genus	KR150181	KR150240	*Satureja* sp.	*Satureja* sp.	*Satureja* sp.	*Satureja hortensis* L.	*Satureja hortensis* L.	*Satureja hortensis* L.	*Satureja hortensis* L.	AY227143 (U)	*Satureja hortensis* L.	GU381620 (V)	*Satureja hortensis* L.
Kh071	Seed	Zireh koohi	Apiaceae	Apiaceae	*Bunium* sp.	Genus	Family	KR150180	KR150239	Apiaceae	Apiaceae	Apiaceae	*Bunium capillifolium* (Guss.) Bertol.	Apiaceae	*Bunium capillifolium* (Guss.) Bertol.	*Bunium* sp.	-	Apiaceae	-	*Bunium* sp.
Kh073	Crushed flowers	Kalpooreh	Lamiaceae	*Teucrium* sp.	*Teucrium polium* L.	Species	Species	-	KR150237	-	*Teucrium polium* L.	*Teucrium polium* L.	-	*Teucrium polium* L.	*Teucrium polium* L.	-	-	*Teucrium polium* L.	JQ044780 (U)	*Teucrium polium* L.
Kh074	Crushed flowers	Gol kooyid	Lamiaceae	*Perovskia* sp.	Unidentified	-	-	KR150182	KR150241	*Perovskia* sp.	Lamiaceae	*Perovskia* sp.	Unidentified	*Perovskia abrotanoides* Kar.	*Perovskia abrotanoides* Kar.	*Perovskia atriplicifolia* Benth.	KJ584242 (U)	*Perovskia atriplicifolia* Benth.	AY570464 (V)	*Perovskia atriplicifolia* Benth.
Kh075	Leaves	Kakooti, Annokh	Lamiaceae	*Ziziphora* sp.	*Ziziphora tenuior* L.	Species	Species	-	KR150238	-	Lamiaceae	Lamiaceae	-	Lamiaceae	Lamiaceae	-	-	*Ziziphora tenuior* L.	GU381507 (V)	*Ziziphora tenuior* L.
Kh076	Crushed flowers	Babooneh	Asteraceae	*Tanacetum* sp.	*Matricaria recutita* L., *Matricaria* sp.	Species	Genus	KR150179	KR150236	*Tripleurospermum* sp.	*Chrysanthemum* sp.	Asteraceae	*Tripleurospermum maritimum* (L.) W.D.J.Koch	*Artemisia* sp.	Asteraceae	*Tanacetum parthenium* (L.) Sch.Bip.	EF577320 (U)	*Chrysanthemum* sp.	-	*Tanacetum parthenium* (L.) Sch.Bip.
Kh082	Flower & leaves	Badranjbooyeh	Lamiaceae	*Hymenocrater* sp.	*Dracocephalum moldavica* L., *Asperugo procumbens* L., *Clinopodium nepeta* subsp. *glandulosum* (Req.) Govaerts, *Melissa officinalis* L.	S, S, G, S	S, S, S, S	-	KR150208	-	Lamiaceae	Lamiaceae	-	*Hymenocrater bituminosus* Fisch. & C.A.Mey.	*Hymenocrater bituminosus* Fisch. & C.A.Mey.	-	-	*Hymenocrater bituminosus* Fisch. & C.A.Mey.	JQ669045 (V)	*Hymenocrater bituminosus* Fisch. & C.A.Mey.
Kh090	Leaves	Merzeh	Lamiaceae	*Satureja* sp.	*Satureja laxiflora* C.Koch	Genus	Genus	KR150160	KR150209	*Satureja montana* L.	*Satureja* sp.	*Satureja montana* L.	*Satureja* sp.	*Satureja* sp.	*Satureja* sp.	*Satureja* sp.	-	*Satureja mutica* Fisch. & C.A.Mey.	GU381619 (V)	*Satureja mutica* Fisch. & C.A.Mey.
Kh095	Flowers	Babooneh	Asteraceae	*Anthemis* sp.	*Matricaria recutita* L., *Matricaria* sp.	Species	Genus	KR150159	KR150207	*Tanacetum* sp.	*Chrysanthemum* sp.	Asteraceae	*Tanacetum* sp.	*Artemisia lagocephala* (Fisch. ex Besser) DC.	*Asteraceae*	*Tanacetum parthenium* (L.) Sch.Bip.	EF577320 (U)	*Chrysanthemum* sp.	-	*Tanacetum* sp.
Kh102	Flowers (petals)	Shaghayegh	Papaveraceae	*Papaver* sp.	*Glaucium* ssp., *Papaver rhoeas* L.	Genus, Species	Family, Species	KR150158	KR150206	*Papaver bracteatum* Lindl.	*Papaver* sp.	*Papaver bracteatum* Lindl.	*Papaver bracteatum* Lindl.	*Papaver* sp.	*Papaver bracteatum* Lindl.	*Papaver bracteatum* Lindl.	DQ912881 (U)	*Papaver* sp.	-	*Papaver bracteatum* Lindl.
Kh108	Flower & leaves	Kalpooreh	Lamiaceae	*Teucrium* sp.	*Teucrium polium* L.	Species	Species	-	KR150202	-	*Teucrium* sp.	*Teucrium* sp.	-	*Teucrium* sp.	*Teucrium* sp.	-	-	*Teucrium polium* L.	JQ044780 (U)	*Teucrium polium* L.
Kh110	Crushed flowers	Boomadaran	Asteraceae	*Achillea* sp.	*Achillea millefolium* L.	Species	Species	-	KR150245	-	*Achillea* sp.	*Achillea* sp.	-	*Achillea santolinoides* subsp. *wilhelmsii* (K.Koch) Greuter	*Achillea santolinoides* subsp. *wilhelmsii* (K.Koch) Greuter	-	-	*Achillea vermicularis* Trin.	DQ267618 (U)	*Achillea vermicularis* Trin.
Kh111	Inflorescence & seed	Taj khoroos	Amaranthaceae	Amaranthus sp.	*Amaranthus caudatus* L.	Species	Genus	KR150154	KR150203	*Amaranthus hybridus* L.	*Amaranthus spinosus* L.	*Amaranthus sp.*	*Amaranthus sp.*	*Amaranthus sp.*	*Amaranthus sp.*	*Amaranthus hybridus* L.	DQ005960 (V)	*Amaranthus sp.*	-	*Amaranthus hybridus* L.
Kh112	Leaves	Kakooti, Annokh	Lamiaceae	*Ziziphora* sp.	*Ziziphora tenuior* L.	Species	Species	-	KR150198	-	*Ziziphora* sp.	*Ziziphora* sp.	-	Lamiaceae	Lamiaceae	-	-	*Ziziphora tenuior* L.	GU381507 (V)	*Ziziphora tenuior* L.
Kh113	Flowers (petals)	Gole gavzaban	Boraginaceae	*Echium* sp.	*Echium amoenum* Fisch. & C. A. Mey., *Borago officinalis* L.	Genus, Species	Genus, Species	KR150155	KR150204	*Echium* sp.	Boraginaceae	*Echium* sp.	*Echium* sp.	*Lobostemon fruticosus* (L.) H.Buek	Boraginaceae	*Echium* sp.	-	*Echium* sp.	-	*Echium* sp.
Kh114	Flowers	Khatmi	Malvaceae	*Alcea* sp.	*Alcea lavateriflora* (DC.) Boiss., *Alcea digitata* Alef.	Species, Genus	Species, Genus	KR150156	-	*Alcea* sp.	-	*Alcea* sp.	*Alcea koelzii* Riedl	-	*Alcea koelzii* Riedl	*Alcea* sp.	-	-	-	*Alcea* sp.
Kh115	Seed	Zireh siyah	Apiaceae	*Bunium* sp.	*Bunium persicum* (Boiss.) B.Fedtsch.	Species	Family	KR150151	KR150199	*Ligusticum* sp.	Apiaceae	*Ligusticum* sp.	*Bunium* sp.	Apiaceae	*Bunium* sp.	*Bunium* sp.	-	Apiaceae	-	*Bunium* sp.
Kh116	Fruits	Zereshk koohi	Berberidaceae	*Berberis* sp.	*Berberis vulgaris* L.	Species	Species	KR150186	KR150246	*Berberis* sp.	*Berberis* sp.	*Berberis* sp.	*Berberis* sp.	*Berberis* sp.	*Berberis* sp.	*Berberis integerrima* Bunge	JN228267 (U)	*Berberis* sp.	-	*Berberis integerrima* Bunge
Kh117	Crushed leaves	Marzeh	Urticaceae	Urticaceae	*Satureja laxiflora* C.Koch, *Satureja hortensis* L.	Genus, Species	Genus, Species	KR150153	KR150201	*Urtica* sp.	*Urtica dioica* L.	*Urtica dioica* L.	*Urtica dioica* L.	*Urtica dioica* L.	*Urtica dioica* L.	*Urtica dioica* L.	KF137936 (U)	*Urtica dioica* L.	KF138424 (U)	*Urtica dioica* L.

Kh118	Seed pods	Nakhonak	Fabaceae	*Astragalus* sp.	*Melilotus officinalis* L., *Astragalus hamosus* L.	Species, Species	Species, Species	KR150152	KR150200	*Oxytropis nigrescens* var. *uniflora* (Hook.) Barneby	*Astragalus* sp.	Fabaceae	*Astragalus commixtus* Bunge	*Astragalus* sp.	*Astragalus commixtus* Bunge	*Oxytropis* sp.	-	*Astragalus* sp.	-	*Astragalus* sp.
Kh132	Gum	Katira	Fabaceae	Fabaceae	*Astragalus* sp.	Genus	Genus	KR150178	KR150235	*Astragalus* sp.	*Astragalus* sp.	*Astragalus* sp.	*Astragalus* sp.	*Astragalus edulis* Bunge	*Astragalus edulis* Bunge	*Astracantha floccosa* (Boiss.) Podlech	AB231144 (V)	*Astragalus verus* Olivier	AB485937 (V)	*Astragalus* sp.
Kh133	Leaves	Avishan	Lamiaceae	*Thymus* sp.	*Thymus vulgaris* L., *Thymus serpyllum* L.	Species, Species	Species, Species	KR150176	KR150233	*Zataria multiflora* Boiss.	Lamiaceae	*Zataria multiflora* Boiss.	*Zataria multiflora* Boiss.	*Pentapleura subulifera* Hand.-Mazz.	Lamiaceae	*Zataria multiflora* Boiss.	JQ669139 (V)	*Zataria multiflora* Boiss.	JQ669071 (V)	*Zataria multiflora* Boiss.
Kh135	Flowers	Gol khatmi	Malvaceae	*Malva* sp.	*Alcea lavateriflora* (DC.) Boiss., *Alcea digitata* Alef.	Species, Genus	Species, Genus	-	KR150232	-	*Malva* sp.	*Malva* sp.	-	*Pentaplaris doroteae* L.O.Williams & Standl.	*Pentaplaris doroteae* L.O.Williams & Standl.	-	-	*Malva nicaeensis* All.		*Malva nicaeensis* All.
Kh138	Seed	Zireh siyah	Apiaceae	Apiaceae	*Bunium persicum* (Boiss.) B.Fedtsch.	Species	Family	KR150175	KR150231	Apiaceae	Apiaceae	Apiaceae	*Bunium* sp.	Apiaceae	*Bunium* sp.	*Bunium* sp.	-	Apiaceae	-	*Bunium* sp.
Kh141	Seed & stem	Shirin badyan	Apiaceae	Apiaceae	*Foeniculum vulgare* Mill.	Species	Species	KR150177	KR150234	*Pimpinella anisum* L.	Apiaceae	*Pimpinella anisum* L.	*Pimpinella sp.*	*Heteromorpha* sp.	Apiaceae	*Pimpinella anisum* L.	EU785940 (U)	*Pimpinella* sp.	-	*Pimpinella anisum* L.
