# Supplementary material for: Unidentifiable by morphology: DNA barcoding of plant material in local markets in Iran
Source: PLoS One. 2017 Apr 18;12(4):e0175722. doi: 10.1371/journal.pone.0175722 (PMC5395179; doi:10.1371/journal.pone.0175722)
Supplement: S4 Table — BLAST hits sorted by max score*(identity/cover). Colored by deviation (d) from highest hit: d = < 1%: green, 1% < d = < 2%: orange, 2% < d = < 3%: red, 3% < d: no color. (PDF) [file pone.0175722.s004.pdf]

Select for downloading or viewing reports	Kh001_ITS Description	Max score	Total score	Query cover	E value	Ident	Accession	(Ident/Cover)* Max score	Deviation from top hit
Select seq gb|HM176650.1|	Cuminum cyminum cultivar GC-2 18S ribosomal RNA gene, partial sequence; internal transcribed spacer 1, 5.8S ribosomal RNA gene, and internal transcribed spacer 2, complete sequence; and 28S ribosomal RNA gene, partial sequence	998	998	95%	0.0	96%	HM176650.1	1008,51	100,0%
Select seq gb|HM176653.1|	Cuminum cyminum cultivar RZ-223 18S ribosomal RNA gene, partial sequence; internal transcribed spacer 1, 5.8S ribosomal RNA gene, and internal transcribed spacer 2, complete sequence; and 28S ribosomal RNA gene, partial sequence	1007	1007	96%	0.0	96%	HM176653.1	1007,00	99,9%
Select seq gb|HM176652.1|	Cuminum cyminum cultivar RZ-19 18S ribosomal RNA gene, partial sequence; internal transcribed spacer 1, 5.8S ribosomal RNA gene, and internal transcribed spacer 2, complete sequence; and 28S ribosomal RNA gene, partial sequence	1016	1016	97%	0.0	96%	HM176652.1	1005,53	99,7%
Select seq gb|HM176651.1|	Cuminum cyminum cultivar GC-4 18S ribosomal RNA gene, partial sequence; internal transcribed spacer 1, 5.8S ribosomal RNA gene, and internal transcribed spacer 2, complete sequence; and 28S ribosomal RNA gene, partial sequence	1014	1014	97%	0.0	96%	HM176651.1	1003,55	99,5%
Select seq gb|HM176655.1|	Cuminum cyminum cultivar RZ-341 18S ribosomal RNA gene, partial sequence; internal transcribed spacer 1, 5.8S ribosomal RNA gene, and internal transcribed spacer 2, complete sequence; and 28S ribosomal RNA gene, partial sequence	1013	1013	97%	0.0	96%	HM176655.1	1002,56	99,4%
Select seq gb|HM176654.1|	Cuminum cyminum cultivar RZ-209 18S ribosomal RNA gene, partial sequence; internal transcribed spacer 1, 5.8S ribosomal RNA gene, and internal transcribed spacer 2, complete sequence; and 28S ribosomal RNA gene, partial sequence	994	994	96%	0.0	95%	HM176654.1	983,65	97,5%
Select seq gb|KF160677.1|	Cuminum cyminum voucher C:Simonsen 2013-8 internal transcribed spacer 1, partial sequence; 5.8S ribosomal RNA gene, complete sequence; and internal transcribed spacer 2, partial sequence	1013	1013	98%	0.0	95%	KF160677.1	981,99	97,4%
Select seq gb|U78362.2|CCU78362	Cuminum cyminum internal transcribed spacer 1, 5.8S ribosomal RNA gene, and internal transcribed spacer 2, complete sequence	909	909	90%	0.0	95%	U78362.2	959,50	95,1%
Select seq gb|KF160679.1|	Laserpitium siler voucher C:Simonsen 2013-5 internal transcribed spacer 1, partial sequence; 5.8S ribosomal RNA gene, complete sequence; and internal transcribed spacer 2, partial sequence	758	758	98%	0.0	88%	KF160679.1	680,65	67,5%
Select seq gb|FJ415113.1|	Laserpitium siler voucher Gardner & Gardner 2455 30-VII-1984 (E 00043177) internal transcribed spacer 1, 5.8S ribosomal RNA gene, and internal transcribed spacer 2, complete sequence	673	673	90%	0.0	88%	FJ415113.1	658,04	65,2%
Select seq gb|FJ415112.1|	Laserpitium siler voucher Gardner & Gardner 781 13-VII-1980 (E 00043183) internal transcribed spacer 1, 5.8S ribosomal RNA gene, and internal transcribed spacer 2, complete sequence	671	671	90%	0.0	87%	FJ415112.1	648,63	64,3%
Select seq gb|FJ415132.1|	Laserpitium nitidum voucher Cobau 2715 5-VI-1920 (KRA) internal transcribed spacer 1, 5.8S ribosomal RNA gene, and internal transcribed spacer 2, complete sequence	667	667	90%	0.0	87%	FJ415132.1	644,77	63,9%
Select seq gb|KF160678.1|	Laser trilobum voucher C:Simonsen 2013-6 internal transcribed spacer 1, partial sequence; 5.8S ribosomal RNA gene, complete sequence; and internal transcribed spacer 2, partial sequence	708	708	97%	0.0	87%	KF160678.1	635,01	63,0%
Select seq gb|FJ415133.1|	Laserpitium peucedanoides voucher Zarzycki 2-VIII-1957 (KRAM) internal transcribed spacer 1, 5.8S ribosomal RNA gene, and internal transcribed spacer 2, complete sequence	656	656	90%	0.0	87%	FJ415133.1	634,13	62,9%
Select seq gb|FJ415136.1|	Thapsia meoides voucher cult., Con. bot. de la Ville de Mulhouse 98138 7-IX-2000 internal transcribed spacer 1, 5.8S ribosomal RNA gene, and internal transcribed spacer 2, complete sequence	654	654	90%	0.0	87%	FJ415136.1	632,20	62,7%
Select seq gb|FJ415134.1|	Laserpitium pseudomeum voucher Gustavsson 9672 10-VI-1985 (G) internal transcribed spacer 1, 5.8S ribosomal RNA gene, and internal transcribed spacer 2, complete sequence	652	652	90%	0.0	87%	FJ415134.1	630,27	62,5%
Select seq gb|FJ415152.1|	Laserpitium stevenii voucher Muibaniani & al. 12-V-1979 (LE) internal transcribed spacer 1, 5.8S ribosomal RNA gene, and internal transcribed spacer 2, complete sequence	651	651	90%	0.0	87%	FJ415152.1	629,30	62,4%
Select seq gb|JQ305147.1|	Polylophium involucratum voucher TARI-s.n. internal transcribed spacer 1, 5.8S ribosomal RNA gene, and internal transcribed spacer 2, complete sequence	640	640	89%	#######	87%	JQ305147.1	625,62	62,0%
Select seq gb|FJ415125.1|	Laserpitium krapfii subsp. gaudinii voucher Charpin 17-VII-1969 (G) internal transcribed spacer 1, 5.8S ribosomal RNA gene, and internal transcribed spacer 2, complete sequence	645	645	90%	0.0	87%	FJ415125.1	623,50	61,8%
Select seq gb|KJ004342.1|	Spergula fallax 18S ribosomal RNA gene, partial sequence; internal transcribed spacer 1, 5.8S ribosomal RNA gene, and internal transcribed spacer 2, complete sequence; and 26S ribosomal RNA gene, partial sequence	701	701	98%	0.0	87%	KJ004342.1	622,32	61,7%
Select seq gb|FJ415123.1|	Laserpitium nestleri voucher Fabregat & Udias 2007 1991 (BCC) internal transcribed spacer 1, 5.8S ribosomal RNA gene, and internal transcribed spacer 2, complete sequence	636	636	89%	#######	87%	FJ415123.1	621,71	61,6%
Select seq gb|FJ415111.1|	Laserpitium siler voucher Davis & Sutton D 65739 21-IV-1979 (E) internal transcribed spacer 1, 5.8S ribosomal RNA gene, and internal transcribed spacer 2, complete sequence	643	643	90%	#######	87%	FJ415111.1	621,57	61,6%
Select seq gb|KF850594.1|	Ferula communis voucher KSUFS821 18S ribosomal RNA gene, partial sequence; internal transcribed spacer 1, 5.8S ribosomal RNA gene, and internal transcribed spacer 2, complete sequence; and 28S ribosomal RNA gene, partial sequence	678	678	95%	0.0	86%	KF850594.1	613,77	60,9%
Select seq gb|DQ379386.1|	Ferula varia voucher Grubov 17-VI-1960 (LE) internal transcribed spacer 1, 5.8S ribosomal RNA gene, and internal transcribed spacer 2, complete sequence	628	628	88%	#######	86%	DQ379386.1	613,73	60,9%
Select seq gb|FJ415141.1|	Thapsia thapsioides voucher Davis & Lamond D 57768 12-V-1975 (E 00040997) internal transcribed spacer 1, 5.8S ribosomal RNA gene, and internal transcribed spacer 2, complete sequence	640	640	90%	#######	86%	FJ415141.1	611,56	60,6%
Select seq gb|FJ415121.1|	Laserpitium nestleri subsp. flabellatum voucher Montserrat 22-VIII-2000 (JACA R265475) internal transcribed spacer 1, 5.8S ribosomal RNA gene, and internal transcribed spacer 2, complete sequence	632	632	89%	#######	86%	FJ415121.1	610,70	60,6%

Select seq gb|KJ660796.1|	Ferula licentiana voucher ACE 900 (E) internal transcribed spacer 1, partial sequence; 5.8S ribosomal RNA gene, complete sequence; and internal transcribed spacer 2, partial sequence	638	638	90%	#######	86%	KJ660796.1	609,64	60,5%
Select seq gb|JQ305146.1|	Laserpitium stevenii voucher MW-886 internal transcribed spacer 1, 5.8S ribosomal RNA gene, and internal transcribed spacer 2, complete sequence	638	638	90%	#######	86%	JQ305146.1	609,64	60,5%
Select seq gb|EF560691.1|	Ferula olivacea internal transcribed spacer 1, 5.8S ribosomal RNA gene, and internal transcribed spacer 2, complete sequence	638	638	90%	#######	86%	EF560691.1	609,64	60,5%
Select seq gb|GQ165517.1|	Ferula communis 18S ribosomal RNA gene, partial sequence; internal transcribed spacer 1, 5.8S ribosomal RNA gene, and internal transcribed spacer 2, complete sequence; and 28S ribosomal RNA gene, partial sequence	673	673	95%	0.0	86%	GQ165517.1	609,24	60,4%
Select seq gb|KJ660811.1|	Ferula rubricaulis voucher 666 0023611 (W) internal transcribed spacer 1, partial sequence; 5.8S ribosomal RNA gene, complete sequence; and internal transcribed spacer 2, partial sequence	623	623	88%	#######	86%	KJ660811.1	608,84	60,4%
Select seq gb|KJ660765.1|	Ferula assa-foetida voucher Parishani 14017 (IUH) internal transcribed spacer 1, partial sequence; 5.8S ribosomal RNA gene, complete sequence; and internal transcribed spacer 2, partial sequence	623	623	88%	#######	86%	KJ660765.1	608,84	60,4%
Select seq gb|DQ379409.1|	Ferula karakalensis voucher Tarasov 25 22-V-1949 (LE) internal transcribed spacer 1, 5.8S ribosomal RNA gene, and internal transcribed spacer 2, complete sequence	623	623	88%	#######	86%	DQ379409.1	608,84	60,4%
Select seq gb|FJ415120.1|	Laserpitium eliasii subsp. thalictrifolium voucher Rico 11-VIII-1985 (SALA 41460) internal transcribed spacer 1, 5.8S ribosomal RNA gene, and internal transcribed spacer 2, complete sequence	630	630	89%	#######	86%	FJ415120.1	608,76	60,4%
Select seq gb|FJ415142.1|	Thapsia thapsioides voucher Davis 53419 9-VI-1971 (E) internal transcribed spacer 1, 5.8S ribosomal RNA gene, and internal transcribed spacer 2, complete sequence	636	636	90%	#######	86%	FJ415142.1	607,73	60,3%
Select seq gb|FJ415130.1|	Laserpitium halleri voucher Reverchon 3-VIII-1869 (KRAM) internal transcribed spacer 1, 5.8S ribosomal RNA gene, and internal transcribed spacer 2, complete sequence	636	636	90%	#######	86%	FJ415130.1	607,73	60,3%
Select seq gb|JQ305149.1|	Polylophium panjutinii voucher MW-s.n. internal transcribed spacer 1, 5.8S ribosomal RNA gene, and internal transcribed spacer 2, complete sequence	627	627	89%	#######	86%	JQ305149.1	605,87	60,1%
Select seq gb|KJ680158.1|	Ekimia bornmuelleri voucher MW-65 internal transcribed spacer 1 and 5.8S ribosomal RNA gene, complete sequence; and internal transcribed spacer 2, partial sequence	612	612	87%	#######	86%	KJ680158.1	604,97	60,0%
Select seq gb|FJ415119.1|	Laserpitium eliasii subsp. ordunae voucher Montserrat 30-VIII-1983 (JACA 733483) internal transcribed spacer 1, 5.8S ribosomal RNA gene, and internal transcribed spacer 2, complete sequence	625	625	89%	#######	86%	FJ415119.1	603,93	59,9%
Select seq gb|FJ415131.1|	Laserpitium latifolium voucher Sudnik 8-VIII-1975 (WA) internal transcribed spacer 1, 5.8S ribosomal RNA gene, and internal transcribed spacer 2, complete sequence	632	632	90%	#######	86%	FJ415131.1	603,91	59,9%
Select seq emb|HE602450.1|	Ferula communis genomic DNA containing ITS1, 5.8S rRNA gene, ITS2, specimen voucher Jury, SL. 16346	667	667	95%	0.0	86%	HE602450.1	603,81	59,9%
Select seq gb|GQ165518.1|	Ferula communis 18S ribosomal RNA gene, partial sequence; internal transcribed spacer 1, 5.8S ribosomal RNA gene, and internal transcribed spacer 2, complete sequence; and 28S ribosomal RNA gene, partial sequence	667	667	95%	0.0	86%	GQ165518.1	603,81	59,9%
Select seq gb|KJ660820.1|	Ferula szowitsiana voucher Feyzi 9316 internal transcribed spacer 1, partial sequence; 5.8S ribosomal RNA gene, complete sequence; and internal transcribed spacer 2, partial sequence	617	617	88%	#######	86%	KJ660820.1	602,98	59,8%
Select seq gb|KJ660807.1|	Ferula persica voucher Nowroozi 2833 internal transcribed spacer 1, partial sequence; 5.8S ribosomal RNA gene, complete sequence; and internal transcribed spacer 2, partial sequence	617	617	88%	#######	86%	KJ660807.1	602,98	59,8%
Select seq gb|KJ660782.1|	Ferula glabra voucher Hedge & Wendelbo W 3760 (E) internal transcribed spacer 1, partial sequence; 5.8S ribosomal RNA gene, complete sequence; and internal transcribed spacer 2, partial sequence	617	617	88%	#######	86%	KJ660782.1	602,98	59,8%
Select seq gb|KJ660777.1|	Ferula flabelliloba voucher Mozaffarian 87053 (TARI) internal transcribed spacer 1, partial sequence; 5.8S ribosomal RNA gene, complete sequence; and internal transcribed spacer 2, partial sequence	617	617	88%	#######	86%	KJ660777.1	602,98	59,8%
Select seq gb|KJ660776.1|	Ferula fedtschenkoana voucher Egorova 2231 (LE) internal transcribed spacer 1, partial sequence; 5.8S ribosomal RNA gene, complete sequence; and internal transcribed spacer 2, partial sequence	617	617	88%	#######	86%	KJ660776.1	602,98	59,8%
Select seq gb|KJ660753.1|	Ferula jaeschkeana voucher Kamelin 381 (LE) internal transcribed spacer 1, 5.8S ribosomal RNA gene, and internal transcribed spacer 2, complete sequence	617	617	88%	#######	86%	KJ660753.1	602,98	59,8%
Select seq gb|DQ379457.1|	Ferula moschata voucher Egorova 2235 31-VII-1960 (LE) internal transcribed spacer 1, 5.8S ribosomal RNA gene, and internal transcribed spacer 2, complete sequence	617	617	88%	#######	86%	DQ379457.1	602,98	59,8%
Select seq gb|DQ379427.1|	Ferula szowitsiana voucher Meshtsheriakov 14-VI-1969 (LE) internal transcribed spacer 1, 5.8S ribosomal RNA gene, and internal transcribed spacer 2, complete sequence	617	617	88%	#######	86%	DQ379427.1	602,98	59,8%
Select seq gb|DQ379391.1|	Ferula clematidifolia voucher Botschantzev & Egorova 1290 12-VI-1960 (LE) internal transcribed spacer 1, 5.8S ribosomal RNA gene, and internal transcribed spacer 2, complete sequence	617	617	88%	#######	86%	DQ379391.1	602,98	59,8%
Select seq gb|FJ415118.1|	Laserpitium eliasii voucher Alejandre 21-VIII-1983 (VIT 27070) internal transcribed spacer 1, 5.8S ribosomal RNA gene, and internal transcribed spacer 2, complete sequence	623	623	89%	#######	86%	FJ415118.1	602,00	59,7%
Select seq gb|AF008645.2|	Polylophium panjutinii internal transcribed spacer 1, 5.8S ribosomal RNA, and internal transcribed spacer 2, complete sequence	621	621	89%	#######	86%	AF008645.2	600,07	59,5%
Select seq gb|DQ379446.1|	Ferula schtschurowskiana voucher Botschantzev 103 12-X-1970 (LE) internal transcribed spacer 1, 5.8S ribosomal RNA gene, and internal transcribed spacer 2, complete sequence	614	614	88%	#######	86%	DQ379446.1	600,05	59,5%

Select seq emb|FN432918.1|	Ferula mervynii genomic DNA sequence containing ITS1, 5.8S rRNA gene and ITS2, specimen voucher GAZI:M. Sagiroglu 2262	669	669	96%	0.0	86%	FN432918.1	599,31	59,4%
Select seq emb|FN432921.1|	Ferula hermonis genomic DNA sequence containing ITS1, 5.8S rRNA gene and ITS2, specimen voucher GAZI:M. Sagiroglu 2246	662	662	95%	0.0	86%	FN432921.1	599,28	59,4%
Select seq gb|GQ165516.1|	Ferula communis 18S ribosomal RNA gene, partial sequence; internal transcribed spacer 1, 5.8S ribosomal RNA gene, and internal transcribed spacer 2, complete sequence; and 28S ribosomal RNA gene, partial sequence	662	662	95%	0.0	86%	GQ165516.1	599,28	59,4%
Select seq gb|KJ660837.1|	Leutea polyscias voucher Mozaffarian 64227 (TARI) internal transcribed spacer 1, partial sequence; 5.8S ribosomal RNA gene, complete sequence; and internal transcribed spacer 2, partial sequence	627	627	90%	#######	86%	KJ660837.1	599,13	59,4%
Select seq gb|KJ660832.1|	Leutea elbursensis voucher Assadi, Mozaffarian & Jamzad 33570 (TARI) internal transcribed spacer 1, partial sequence; 5.8S ribosomal RNA gene, complete sequence; and internal transcribed spacer 2, partial sequence	627	627	90%	#######	86%	KJ660832.1	599,13	59,4%
Select seq gb|EU169270.1|	Ferula olivacea voucher CLD Exp. 790 (cult. RBGE 19910663) internal transcribed spacer 1, partial sequence; 5.8S ribosomal RNA gene, complete sequence; and internal transcribed spacer 2, partial sequence	627	627	90%	#######	86%	EU169270.1	599,13	59,4%
Select seq gb|AY974773.1|	Laser rechingeri internal transcribed spacer 1, 5.8S ribosomal RNA gene, and internal transcribed spacer 2, complete sequence	627	627	90%	#######	86%	AY974773.1	599,13	59,4%
Select seq gb|KF160692.1|	Thapsia gummifera voucher C:Weitzel 2011-2 internal transcribed spacer 1, partial sequence; 5.8S ribosomal RNA gene, complete sequence; and internal transcribed spacer 2, partial sequence	682	682	98%	0.0	86%	KF160692.1	598,49	59,3%
Select seq gb|KF160672.1|	Ferula communis voucher C:Weitzel 2011-4 internal transcribed spacer 1, partial sequence; 5.8S ribosomal RNA gene, complete sequence; and internal transcribed spacer 2, partial sequence	682	682	98%	0.0	86%	KF160672.1	598,49	59,3%
Select seq gb|KJ660819.1|	Ferula stenoloba voucher Wendelbo & Ekberg 9790 (E) internal transcribed spacer 1, partial sequence; 5.8S ribosomal RNA gene, complete sequence; and internal transcribed spacer 2, partial sequence	612	612	88%	#######	86%	KJ660819.1	598,09	59,3%
Select seq gb|KJ660793.1|	Ferula kashanica voucher Rechinger 46849 (B) internal transcribed spacer 1, partial sequence; 5.8S ribosomal RNA gene, complete sequence; and internal transcribed spacer 2, partial sequence	612	612	88%	#######	86%	KJ660793.1	598,09	59,3%
Select seq emb|FN432920.1|	Ferula orientalis genomic DNA sequence containing ITS1, 5.8S rRNA gene, ITS2, specimen voucher GAZI:M. Sagiroglu 2170	667	667	96%	0.0	86%	FN432920.1	597,52	59,2%
Select seq emb|FN432917.1|	Ferula szowitsiana genomic DNA sequence containing ITS1, 5.8S rRNA gene and ITS2, specimen voucher GAZI:M. Sagiroglu 2147	660	660	95%	0.0	86%	FN432917.1	597,47	59,2%
Select seq emb|FN432815.1|	Ferula sp. GAZI 2181 genomic DNA sequence containing ITS1, 5.8S rRNA gene and ITS2, specimen voucher GAZI:2181	660	660	95%	0.0	86%	FN432815.1	597,47	59,2%
Select seq emb|FN432916.1|	Ferula haussknechtii genomic DNA sequence containing ITS1, 5.8S rRNA gene and ITS2, specimen voucher GAZI:M. Sagiroglu 2255	658	658	95%	0.0	86%	FN432916.1	595,66	59,1%
Select seq gb|FJ415153.1|	Laserpitium archangelica voucher Mayer 63416 12-VIII-1967 (KRAM) internal transcribed spacer 1, 5.8S ribosomal RNA gene, and internal transcribed spacer 2, complete sequence	623	623	90%	#######	86%	FJ415153.1	595,31	59,0%
Select seq gb|JQ305148.1|	Polylophium panjutinii voucher AA-s.n. internal transcribed spacer 1, 5.8S ribosomal RNA gene, and internal transcribed spacer 2, complete sequence	616	616	89%	#######	86%	JQ305148.1	595,24	59,0%
Select seq gb|GQ165515.1|	Ferula communis 18S ribosomal RNA gene, partial sequence; internal transcribed spacer 1, 5.8S ribosomal RNA gene, and internal transcribed spacer 2, complete sequence; and 28S ribosomal RNA gene, partial sequence	656	656	95%	0.0	86%	GQ165515.1	593,85	58,9%
Select seq gb|KJ660834.1|	Leutea gracillima voucher Akhani 12060 (W 1999-03655) internal transcribed spacer 1, partial sequence; 5.8S ribosomal RNA gene, complete sequence; and internal transcribed spacer 2, partial sequence	621	621	90%	#######	86%	KJ660834.1	593,40	58,8%
Select seq gb|FJ415139.1|	Thapsia gummifera voucher cult., Con. bot. de la Ville de Mulhouse 9309* 9-IX-2000 internal transcribed spacer 1, 5.8S ribosomal RNA gene, and internal transcribed spacer 2, complete sequence	621	621	90%	#######	86%	FJ415139.1	593,40	58,8%
Select seq gb|DQ379425.1|	Ferula loscosii voucher Perez-Collazos et al. 28-V-2004 (UZ) internal transcribed spacer 1, 5.8S ribosomal RNA gene, and internal transcribed spacer 2, complete sequence	621	621	90%	#######	86%	DQ379425.1	593,40	58,8%
Select seq emb|FN432914.1|	Ferula elaeochytris genomic DNA sequence containing ITS1, 5.8S rRNA gene and ITS2, specimen voucher GAZI:M. Sagiroglu 2227	662	662	96%	0.0	86%	FN432914.1	593,04	58,8%
Select seq gb|KM983398.1|	Ferula gummosa voucher ZNU313-Taham 18S ribosomal RNA gene, partial sequence; internal transcribed spacer 1, 5.8S ribosomal RNA gene, and internal transcribed spacer 2, complete sequence; and 28S ribosomal RNA gene, partial sequence	641	641	93%	#######	86%	KM983398.1	592,75	58,8%
Select seq emb|FN432915.1|	Ferula longipedunculata genomic DNA sequence containing ITS1, 5.8S rRNA gene and ITS2, specimen voucher GAZI:M. Sagiroglu 2235	654	654	95%	0.0	86%	FN432915.1	592,04	58,7%
Select seq gb|KJ660836.1|	Leutea petiolaris voucher Mozaffarian & Massoumi 78152 (TARI) internal transcribed spacer 1, partial sequence; 5.8S ribosomal RNA gene, complete sequence; and internal transcribed spacer 2, partial sequence	616	616	90%	#######	86%	KJ660836.1	588,62	58,4%
Select seq gb|KJ660833.1|	Leutea glaucopruinosa voucher Termeh & Zargari 040483-E (W 0023608) internal transcribed spacer 1, partial sequence; 5.8S ribosomal RNA gene, complete sequence; and internal transcribed spacer 2, partial sequence	616	616	90%	#######	86%	KJ660833.1	588,62	58,4%
Select seq gb|KJ660831.1|	Leutea cupularis voucher Assadi & Mozaffarian 31236 (TARI) internal transcribed spacer 1, partial sequence; 5.8S ribosomal RNA gene, complete sequence; and internal transcribed spacer 2, partial sequence	616	616	90%	#######	86%	KJ660831.1	588,62	58,4%
Select seq gb|KJ660817.1|	Ferula sinaica voucher Albert 22A (W 2009-02860) internal transcribed spacer 1, partial sequence; 5.8S ribosomal RNA gene, complete sequence; and internal transcribed spacer 2, partial sequence	616	616	90%	#######	86%	KJ660817.1	588,62	58,4%

Select seq gb|KJ660798.1|	Ferula marmarica voucher Davis 50085 internal transcribed spacer 1, partial sequence; 5.8S ribosomal RNA gene, complete sequence; and internal transcribed spacer 2, partial sequence	616	616	90%	#######	86% KJ660798.1	588,62	58,4%
Select seq gb|FJ415150.1|	Thapsia scabra voucher Aran & Toha 20-VIII-2000 (VAL 118242) internal transcribed spacer 1, 5.8S ribosomal RNA gene, and internal transcribed spacer 2, complete sequence	616	616	90%	#######	86% FJ415150.1	588,62	58,4%
Select seq gb|FJ415115.1|	Laserpitium glaucum voucher Darrah 552 10-VIII-1969 (E 00042002) internal transcribed spacer 1, 5.8S ribosomal RNA gene, and internal transcribed spacer 2, complete sequence	616	616	90%	#######	86% FJ415115.1	588,62	58,4%
Select seq gb|DQ379424.1|	Ferula loscosii voucher Perez-Collazos & Catalan 25-IV-2004 (UZ) internal transcribed spacer 1, 5.8S ribosomal RNA gene, and internal transcribed spacer 2, complete sequence	616	616	90%	#######	86% DQ379424.1	588,62	58,4%
Select seq gb|DQ379406.1|	Ferula glauca voucher Augustin 4821 6-VI-1923 (KRA) internal transcribed spacer 1, 5.8S ribosomal RNA gene, and internal transcribed spacer 2, complete sequence	616	616	90%	#######	86% DQ379406.1	588,62	58,4%
Select seq emb|FN432913.1|	Ferula halophila genomic DNA sequence containing ITS1, 5.8S rRNA gene and ITS2, specimen voucher GAZI:M. Sagiroglu 2146	665	665	99%	0.0	85% FN432913.1	570,96	56,6%
Select seq gb|KF160680.1|	Melanoselinum decipiens voucher C:Hansen 13407 internal transcribed spacer 1, partial sequence; 5.8S ribosomal RNA gene, complete sequence; and internal transcribed spacer 2, partial sequence	643	643	97%	#######	85% KF160680.1	563,45	55,9%
Select seq emb|FN432919.1|	Ferula coskunii genomic DNA sequence containing ITS1, 5.8S rRNA gene and ITS2, specimen voucher GAZI:M. Sagiroglu 2270	623	623	95%	#######	85% FN432919.1	557,42	55,3%
Select seq gb|KF160686.1|	Thapsia asclepium voucher C:Constantidinis 6835 internal transcribed spacer 1, partial sequence; 5.8S ribosomal RNA gene, complete sequence; and internal transcribed spacer 2, partial sequence	640	640	98%	#######	85% KF160686.1	555,10	55,0%
Select seq gb|KF160681.1|	Monizia edulis voucher C:Hansen 2478 internal transcribed spacer 1, partial sequence; 5.8S ribosomal RNA gene, complete sequence; and internal transcribed spacer 2, partial sequence	621	621	97%	#######	85% KF160681.1	544,18	54,0%
Select seq gb|KF160711.1|	Thapsia smittii voucher C:Weitzel 2011-5 internal transcribed spacer 1, partial sequence; 5.8S ribosomal RNA gene, complete sequence; and internal transcribed spacer 2, partial sequence	625	625	98%	#######	85% KF160711.1	542,09	53,8%
Select seq gb|KF160708.1|	Thapsia smittii voucher C:Smitt 81-v-11 internal transcribed spacer 1, partial sequence; 5.8S ribosomal RNA gene, complete sequence; and internal transcribed spacer 2, partial sequence	625	625	98%	#######	85% KF160708.1	542,09	53,8%
Select seq gb|KF160709.1|	Thapsia smittii voucher C:Smitt 87-08 internal transcribed spacer 1, partial sequence; 5.8S ribosomal RNA gene, complete sequence; and internal transcribed spacer 2, partial sequence	619	619	98%	#######	85% KF160709.1	536,89	53,2%
Select seq gb|KF160706.1|	Thapsia smittii voucher C:FC 9169 internal transcribed spacer 1, partial sequence; 5.8S ribosomal RNA gene, complete sequence; and internal transcribed spacer 2, partial sequence	619	619	98%	#######	85% KF160706.1	536,89	53,2%
Select seq gb|KF160707.1|	Thapsia smittii voucher C:Smitt 88-36 internal transcribed spacer 1, partial sequence; 5.8S ribosomal RNA gene, complete sequence; and internal transcribed spacer 2, partial sequence	614	614	98%	#######	85% KF160707.1	532,55	52,8%
Select seq gb|KF160693.1|	Thapsia gymnesica voucher C:Smitt 94-01 internal transcribed spacer 1, partial sequence; 5.8S ribosomal RNA gene, complete sequence; and internal transcribed spacer 2, partial sequence	612	612	97%	#######	84% KF160693.1	529,98	52,6%
							0,00	0,0%

Select for downloading or viewing reports	Kh001_trnL Description	Max score	Total score	Query cover	E value	Ident	Accession	(Ident/Cover)* Max score	Deviation from top hit
Select seq gb|KJ157756.1|	Osmorhiza bipatriata voucher J. Wen 7265 tRNA-Leu (trnL) gene and trnL-trnF intergenic spacer, partial sequence; chloroplast	1208	1208	99%	0.0	98%	KJ157756.1	1195,80	100,0%
Select seq gb|KJ157736.1|	Osmorhiza berteroi voucher J. Wen 7397 tRNA-Leu (trnL) gene and trnL-trnF intergenic spacer, partial sequence; chloroplast	1199	1199	99%	0.0	97%	KJ157736.1	1174,78	98,2%
Select seq gb|KJ157727.1|	Myrrhis odorata voucher J. Wen 10377 tRNA-Leu (trnL) gene and trnL-trnF intergenic spacer, partial sequence; chloroplast	1197	1197	99%	0.0	97%	KJ157727.1	1172,82	98,1%
Select seq gb|KJ157766.1|	Osmorhiza occidentalis voucher R. R. Halse 5560 tRNA-Leu (trnL) gene and trnL-trnF intergenic spacer, partial sequence; chloroplast	1194	1194	99%	0.0	97%	KJ157766.1	1169,88	97,8%
Select seq gb|KJ157762.1|	Osmorhiza occidentalis voucher J. Wen 7116 tRNA-Leu (trnL) gene and trnL-trnF intergenic spacer, partial sequence; chloroplast	1194	1194	99%	0.0	97%	KJ157762.1	1169,88	97,8%
Select seq gb|KJ157729.1|	Osmorhiza aristata voucher J. Wen 8500 tRNA-Leu (trnL) gene and trnL-trnF intergenic spacer, partial sequence; chloroplast	1190	1190	99%	0.0	97%	KJ157729.1	1165,96	97,5%
Select seq gb|KJ157761.1|	Osmorhiza occidentalis voucher P. P. Lowry II 4633 tRNA-Leu (trnL) gene and trnL-trnF intergenic spacer, partial sequence; chloroplast	1188	1188	99%	0.0	97%	KJ157761.1	1164,00	97,3%
Select seq gb|KJ157744.1|	Osmorhiza depauperata voucher J. Wen 10456 tRNA-Leu (trnL) gene and trnL-trnF intergenic spacer, partial sequence; chloroplast	1188	1188	99%	0.0	97%	KJ157744.1	1164,00	97,3%
Select seq gb|KJ157752.1|	Osmorhiza glabrata voucher J. Wen 7456 tRNA-Leu (trnL) gene and trnL-trnF intergenic spacer, partial sequence; chloroplast	1184	1184	99%	0.0	97%	KJ157752.1	1160,08	97,0%
Select seq gb|KJ157750.1|	Osmorhiza glabrata voucher J. Wen 7315 tRNA-Leu (trnL) gene and trnL-trnF intergenic spacer, partial sequence; chloroplast	1184	1184	99%	0.0	97%	KJ157750.1	1160,08	97,0%
Select seq gb|KJ157728.1|	Osmorhiza aristata voucher J. Wen 5722 tRNA-Leu (trnL) gene and trnL-trnF intergenic spacer, partial sequence; chloroplast	1184	1184	99%	0.0	97%	KJ157728.1	1160,08	97,0%
Select seq gb|KJ157757.1|	Osmorhiza bipatriata voucher P. P. Lowry II & M. J. Warnock 3182 tRNA-Leu (trnL) gene and trnL-trnF intergenic spacer, partial sequence; chloroplast	1182	1182	99%	0.0	97%	KJ157757.1	1158,12	96,8%
Select seq gb|KJ157748.1|	Osmorhiza depauperata voucher J. Wen 7415 tRNA-Leu (trnL) gene and trnL-trnF intergenic spacer, partial sequence; chloroplast	1182	1182	99%	0.0	97%	KJ157748.1	1158,12	96,8%
Select seq gb|KJ157747.1|	Osmorhiza depauperata voucher J. Wen 7387 tRNA-Leu (trnL) gene and trnL-trnF intergenic spacer, partial sequence; chloroplast	1182	1182	99%	0.0	97%	KJ157747.1	1158,12	96,8%
Select seq dbj|AB219666.1|	Tilingia ajanensis chloroplast trnL(UAA), trnF(GAA) genes for tRNA-Leu, tRNA-Phe, partial sequence, trnL-trnF spacer region, specimen_voucher: MAK:K. Senni S1372	1175	1175	100%	0.0	97%	AB219666.1	1139,75	95,3%
Select seq gb|FJ475193.1|	Ligusticum delavayi tRNA-Leu (trnL) gene and trnL-trnF intergenic spacer, partial sequence; chloroplast	1170	1170	100%	0.0	96%	FJ475193.1	1123,20	93,9%
Select seq gb|FJ475196.1|	Hymenidium heterosciadium tRNA-Leu (trnL) gene and trnL-trnF intergenic spacer, partial sequence; chloroplast	1170	1170	100%	0.0	96%	FJ475196.1	1123,20	93,9%
Select seq gb|FJ475190.1|	Hymenidium chloroleucum tRNA-Leu (trnL) gene and trnL-trnF intergenic spacer, partial sequence; chloroplast	1170	1170	100%	0.0	96%	FJ475190.1	1123,20	93,9%
Select seq gb|GU456628.1|	Anthriscus cerefolium chloroplast, complete genome	1146	1146	99%	0.0	96%	GU456628.1	1111,27	92,9%
Select seq gb|AF432022.1|AF432022	Anthriscus cerefolium chloroplast tRNA-Leu (trnL) gene and trnL-trnF intergenic spacer region, partial sequence	1146	1146	99%	0.0	96%	AF432022.1	1111,27	92,9%
Select seq gb|KJ157724.1|	Chaerophyllum roseum voucher J. Wen 10332 tRNA-Leu (trnL) gene and trnL-trnF intergenic spacer, partial sequence; chloroplast	1107	1107	96%	0.0	96%	KJ157724.1	1107,00	92,6%
Select seq gb|KJ157725.1|	Chaerophyllum roseum voucher J. Wen 10334 tRNA-Leu (trnL) gene and trnL-trnF intergenic spacer, partial sequence; chloroplast	1118	1118	97%	0.0	96%	KJ157725.1	1106,47	92,5%
Select seq gb|KJ157723.1|	Anthriscus velutinus voucher J. Wen 10336 tRNA-Leu (trnL) gene and trnL-trnF intergenic spacer, partial sequence; chloroplast	1136	1136	99%	0.0	96%	KJ157723.1	1101,58	92,1%
Select seq gb|KP665025.1|	Anthriscus sylvestris isolate 13S90 tRNA-Leu (trnL) gene and trnL-trnF intergenic spacer, partial sequence; chloroplast	1134	1134	99%	0.0	96%	KP665025.1	1099,64	92,0%
Select seq gb|KP665024.1|	Anthriscus sylvestris isolate 13S89 tRNA-Leu (trnL) gene and trnL-trnF intergenic spacer, partial sequence; chloroplast	1134	1134	99%	0.0	96%	KP665024.1	1099,64	92,0%
Select seq gb|KP665023.1|	Anthriscus sylvestris isolate 13S88 tRNA-Leu (trnL) gene and trnL-trnF intergenic spacer, partial sequence; chloroplast	1134	1134	99%	0.0	96%	KP665023.1	1099,64	92,0%

Select seq gb|KP665022.1|	Anthriscus sylvestris isolate 13S14 tRNA-Leu (trnL) gene and trnL-trnF intergenic spacer, partial sequence; chloroplast	1134	1134	99% 0.0	96% KP665022.1	1099,64	92,0%
Select seq gb|KP665021.1|	Anthriscus sylvestris isolate 13S13 tRNA-Leu (trnL) gene and trnL-trnF intergenic spacer, partial sequence; chloroplast	1134	1134	99% 0.0	96% KP665021.1	1099,64	92,0%
Select seq gb|KP665020.1|	Anthriscus sylvestris isolate 13S107 tRNA-Leu (trnL) gene and trnL-trnF intergenic spacer, partial sequence; chloroplast	1134	1134	99% 0.0	96% KP665020.1	1099,64	92,0%
Select seq gb|KP665019.1|	Anthriscus sylvestris isolate 13S106 tRNA-Leu (trnL) gene and trnL-trnF intergenic spacer, partial sequence; chloroplast	1134	1134	99% 0.0	96% KP665019.1	1099,64	92,0%
Select seq gb|KP665017.1|	Anthriscus sylvestris f. hirtifructus isolate 13S06-1 tRNA-Leu (trnL) gene and trnL-trnF intergenic spacer, partial sequence; chloroplast	1134	1134	99% 0.0	96% KP665017.1	1099,64	92,0%
Select seq gb|KP665016.1|	Anthriscus sylvestris isolate 13S05-2 tRNA-Leu (trnL) gene and trnL-trnF intergenic spacer, partial sequence; chloroplast	1134	1134	99% 0.0	96% KP665016.1	1099,64	92,0%
Select seq gb|KP665015.1|	Anthriscus sylvestris isolate 13S05-1 tRNA-Leu (trnL) gene and trnL-trnF intergenic spacer, partial sequence; chloroplast	1134	1134	99% 0.0	96% KP665015.1	1099,64	92,0%
Select seq gb|KP665014.1|	Anthriscus sylvestris isolate 13S04-5 tRNA-Leu (trnL) gene and trnL-trnF intergenic spacer, partial sequence; chloroplast	1134	1134	99% 0.0	96% KP665014.1	1099,64	92,0%
Select seq gb|KP665013.1|	Anthriscus sylvestris isolate 13S04-4 tRNA-Leu (trnL) gene and trnL-trnF intergenic spacer, partial sequence; chloroplast	1134	1134	99% 0.0	96% KP665013.1	1099,64	92,0%
Select seq gb|KP665012.1|	Anthriscus sylvestris isolate 13S04-3 tRNA-Leu (trnL) gene and trnL-trnF intergenic spacer, partial sequence; chloroplast	1134	1134	99% 0.0	96% KP665012.1	1099,64	92,0%
Select seq gb|KP665011.1|	Anthriscus sylvestris isolate 13S04-2 tRNA-Leu (trnL) gene and trnL-trnF intergenic spacer, partial sequence; chloroplast	1134	1134	99% 0.0	96% KP665011.1	1099,64	92,0%
Select seq gb|KP665010.1|	Anthriscus sylvestris isolate 13S04-1 tRNA-Leu (trnL) gene and trnL-trnF intergenic spacer, partial sequence; chloroplast	1134	1134	99% 0.0	96% KP665010.1	1099,64	92,0%
Select seq gb|KP665018.1|	Anthriscus sylvestris f. hirtifructus isolate 13S06-2 tRNA-Leu (trnL) gene and trnL-trnF intergenic spacer, partial sequence; chloroplast	1133	1133	99% 0.0	96% KP665018.1	1098,67	91,9%
Select seq gb|AF444011.1|	Cymopterus multinervatus tRNA-Phe gene, partial sequence; tRNA-Leu gene, complete sequence; and tRNA-Thr gene, partial sequence; chloroplast genes for chloroplast products	1144	1144	100% 0.0	96% AF444011.1	1098,24	91,8%
Select seq gb|KT963039.1|	Ligusticum tenuissimum voucher 13I-08 chloroplast, complete genome	1140	1140	100% 0.0	96% KT963039.1	1094,40	91,5%
Select seq gb|KP665036.1|	Apiaceae sp. 13R31re1 tRNA-Leu (trnL) gene and trnL-trnF intergenic spacer, partial sequence; chloroplast	1127	1127	99% 0.0	96% KP665036.1	1092,85	91,4%
Select seq gb|KP665035.1|	Apiaceae sp. 13R29re3 tRNA-Leu (trnL) gene and trnL-trnF intergenic spacer, partial sequence; chloroplast	1127	1127	99% 0.0	96% KP665035.1	1092,85	91,4%
Select seq gb|FJ475189.1|	Hymenidium brunonis tRNA-Leu (trnL) gene and trnL-trnF intergenic spacer, partial sequence; chloroplast	1138	1138	100% 0.0	96% FJ475189.1	1092,48	91,4%
Select seq gb|AF444009.1|	Cymopterus globosus tRNA-Phe gene, partial sequence; tRNA-Leu gene, complete sequence; and tRNA-Thr gene, partial sequence; chloroplast genes for chloroplast products	1138	1138	100% 0.0	96% AF444009.1	1092,48	91,4%
Select seq gb|AF444015.1|	Harbouria trachypleura tRNA-Phe gene, partial sequence; tRNA-Leu gene, complete sequence; and tRNA-Thr gene, partial sequence; chloroplast genes for chloroplast products	1134	1134	100% 0.0	96% AF444015.1	1088,64	91,0%
Select seq gb|DQ898156.1|	Daucus carota chloroplast, complete genome	1131	1131	100% 0.0	96% DQ898156.1	1085,76	90,8%
Select seq gb|AF432023.1|AF432023	Myrrhis odorata chloroplast tRNA-Leu (trnL) gene and trnL-trnF intergenic spacer region, partial sequence	1127	1127	99% 0.0	95% AF432023.1	1081,46	90,4%
Select seq gb|FJ475191.1|	Hymenidium davidii tRNA-Leu (trnL) gene and trnL-trnF intergenic spacer, partial sequence; chloroplast	1129	1129	100% 0.0	95% FJ475191.1	1072,55	89,7%
Select seq gb|FJ475215.1|	Pterocyclus angelicoides tRNA-Leu (trnL) gene and trnL-trnF intergenic spacer, partial sequence; chloroplast	1116	1116	99% 0.0	95% FJ475215.1	1070,91	89,6%
Select seq gb|KR011054.1|	Foeniculum vulgare chloroplast, complete genome	1127	1127	100% 0.0	95% KR011054.1	1070,65	89,5%
Select seq gb|KR011055.1|	Anethum graveolens chloroplast, complete genome	1125	1125	100% 0.0	95% KR011055.1	1068,75	89,4%
Select seq gb|HQ246199.1|	Pseudotrachydium vesiculosoalatum tRNA-Leu (trnL) gene, partial sequence; trnL-trnF intergenic spacer, complete sequence; and tRNA-Phe (trnF) gene, partial sequence; chloroplast	1123	1123	100% 0.0	95% HQ246199.1	1066,85	89,2%
Select seq gb|AF444025.1|	Musineon tenuifolium tRNA-Phe gene, partial sequence; tRNA-Leu gene, complete sequence; and tRNA-Thr gene, partial sequence; chloroplast genes for chloroplast products	1123	1123	100% 0.0	95% AF444025.1	1066,85	89,2%

Select seq gb|FJ475212.1|	Physospermum cornubiense tRNA-Leu (trnL) gene and trnL-trnF intergenic spacer, partial sequence; chloroplast	1122	1122	100% 0.0	95%	FJ475212.1	1065,90	89,1%
Select seq gb|AF444028.1|	Pteryxia hendersonii tRNA-Phe gene, partial sequence; tRNA-Leu gene, complete sequence; and tRNA-Thr gene, partial sequence; chloroplast genes for chloroplast products	1120	1120	100% 0.0	95%	AF444028.1	1064,00	89,0%
Select seq gb|AF432024.1|AF432024	Osmorhiza aristata chloroplast tRNA-Leu (trnL) gene and trnL-trnF intergenic spacer region, partial sequence	1120	1120	99% 0.0	94%	AF432024.1	1063,43	88,9%
Select seq gb|AF432002.1|AF432002	Osmorhiza aristata chloroplast tRNA-Leu (trnL) gene and trnL-trnF intergenic spacer region, partial sequence	1120	1120	99% 0.0	94%	AF432002.1	1063,43	88,9%
Select seq gb|KT963036.1|	Angelica acutiloba voucher 13F-28 chloroplast, complete genome	1118	1118	100% 0.0	95%	KT963036.1	1062,10	88,8%
Select seq gb|AF444030.1|	Shoshonea pulvinata tRNA-Phe gene, partial sequence; tRNA-Leu gene, complete sequence; and tRNA-Thr gene, partial sequence; chloroplast genes for chloroplast products	1118	1118	100% 0.0	95%	AF444030.1	1062,10	88,8%
Select seq gb|AF432014.1|AF432014	Osmorhiza depauperata chloroplast tRNA-Leu (trnL) gene and trnL-trnF intergenic spacer region, partial sequence	1118	1118	99% 0.0	94%	AF432014.1	1061,54	88,8%
Select seq gb|FJ475192.1|	Hymenidium decurrens tRNA-Leu (trnL) gene and trnL-trnF intergenic spacer, partial sequence; chloroplast	1114	1114	100% 0.0	95%	FJ475192.1	1058,30	88,5%
Select seq gb|AF432013.1|AF432013	Osmorhiza depauperata chloroplast tRNA-Leu (trnL) gene and trnL-trnF intergenic spacer region, partial sequence	1114	1114	99% 0.0	94%	AF432013.1	1057,74	88,5%
Select seq gb|KP665033.1|	Angelica sinensis isolate 13R17 tRNA-Leu (trnL) gene and trnL-trnF intergenic spacer, partial sequence; chloroplast	1101	1101	99% 0.0	95%	KP665033.1	1056,52	88,4%
Select seq dbj|LC072692.1|	Libanotis buchtormensis chloroplast DNA, trnL-trnF intergenic spacer, partial sequence, isolate: LJJ13	1112	1112	100% 0.0	95%	LC072692.1	1056,40	88,3%
Select seq gb|HQ246203.1|	Trachydium roylei tRNA-Leu (trnL) gene, partial sequence; trnL-trnF intergenic spacer, complete sequence; and tRNA-Phe (trnF) gene, partial sequence; chloroplast	1112	1112	100% 0.0	95%	HQ246203.1	1056,40	88,3%
Select seq gb|FJ475205.1|	Hymenidium wrightianum tRNA-Leu (trnL) gene and trnL-trnF intergenic spacer, partial sequence; chloroplast	1112	1112	100% 0.0	95%	FJ475205.1	1056,40	88,3%
Select seq gb|AF444029.1|	Pteryxia terebinthina tRNA-Phe gene, partial sequence; and tRNA-Leu gene, complete sequence; chloroplast genes for chloroplast products	1112	1112	100% 0.0	95%	AF444029.1	1056,40	88,3%
Select seq gb|AF444026.1|	Neoparrya lithophila tRNA-Phe gene, partial sequence; tRNA-Leu gene, complete sequence; and tRNA-Thr gene, partial sequence; chloroplast genes for chloroplast products	1112	1112	100% 0.0	95%	AF444026.1	1056,40	88,3%
Select seq gb|AF444008.1|	Aletes anisatus tRNA-Phe gene, partial sequence; tRNA-Leu gene, complete sequence; and tRNA-Thr gene, partial sequence; chloroplast genes for chloroplast products	1112	1112	100% 0.0	95%	AF444008.1	1056,40	88,3%
Select seq emb|AM109908.1|	Cnidium officinale plastid partial tRNA-Leu gene and partial IGS	1112	1112	100% 0.0	95%	AM109908.1	1056,40	88,3%
Select seq gb|FJ475202.1|	Hymenidium mieheanum tRNA-Leu (trnL) gene and trnL-trnF intergenic spacer, partial sequence; chloroplast	1109	1109	100% 0.0	95%	FJ475202.1	1053,55	88,1%
Select seq gb|AF444033.1|	Zizia aurea tRNA-Phe gene, partial sequence; tRNA-Leu gene, complete sequence; and tRNA-Thr gene, partial sequence; chloroplast genes for chloroplast products	1109	1109	100% 0.0	95%	AF444033.1	1053,55	88,1%
Select seq dbj|LC072690.1|	Libanotis buchtormensis chloroplast DNA, trnL-trnF intergenic spacer, partial sequence, isolate: LJJ11	1107	1107	100% 0.0	95%	LC072690.1	1051,65	87,9%
Select seq gb|KM035850.1|	Pastinaca pimpinellifolia voucher M.G. Pimenov et al., 117 (MW) plastid, complete genome	1107	1107	100% 0.0	95%	KM035850.1	1051,65	87,9%
Select seq gb|FJ475216.1|	Pterocyclus rivulorum tRNA-Leu (trnL) gene and trnL-trnF intergenic spacer, partial sequence; chloroplast	1107	1107	100% 0.0	95%	FJ475216.1	1051,65	87,9%
Select seq gb|FJ475188.1|	Hymenidium bicolor tRNA-Leu (trnL) gene and trnL-trnF intergenic spacer, partial sequence; chloroplast	1107	1107	100% 0.0	95%	FJ475188.1	1051,65	87,9%
Select seq gb|FJ475182.1|	Aulacospermum simplex tRNA-Leu (trnL) gene and trnL-trnF intergenic spacer, partial sequence; chloroplast	1107	1107	100% 0.0	95%	FJ475182.1	1051,65	87,9%
Select seq gb|FJ475181.1|	Aulacospermum popovii tRNA-Leu (trnL) gene and trnL-trnF intergenic spacer, partial sequence; chloroplast	1107	1107	100% 0.0	95%	FJ475181.1	1051,65	87,9%
Select seq gb|FJ475180.1|	Aulacospermum anomalum tRNA-Leu (trnL) gene and trnL-trnF intergenic spacer, partial sequence; chloroplast	1107	1107	100% 0.0	95%	FJ475180.1	1051,65	87,9%

Select seq gb|EU001357.1|	Heracleum candicans clone trn-yy06 tRNA-Leu (trnL) gene, partial sequence; and trnL-trnF intergenic spacer, complete sequence; chloroplast	1107	1107	100%	0.0	95%	EU001357.1	1051,65	87,9%
Select seq gb|AF444024.1|	Musineon divaricatum tRNA-Phe gene, partial sequence; tRNA-Leu gene, complete sequence; and tRNA-Thr gene, partial sequence; chloroplast genes for chloroplast products	1107	1107	100%	0.0	95%	AF444024.1	1051,65	87,9%
Select seq gb|AF444014.1|	Cymopterus williamsii tRNA-Phe gene, partial sequence; tRNA-Leu gene, complete sequence; and tRNA-Thr gene, partial sequence; chloroplast genes for chloroplast products	1107	1107	100%	0.0	95%	AF444014.1	1051,65	87,9%
Select seq gb|AF444007.1|	Angelica archangelica tRNA-Phe gene, partial sequence; tRNA-Leu gene, complete sequence; and tRNA-Thr gene, partial sequence; chloroplast genes for chloroplast products	1107	1107	100%	0.0	95%	AF444007.1	1051,65	87,9%
Select seq gb|KJ157767.1|	Pastinaca sativa voucher J. Wen 9783 tRNA-Leu (trnL) gene and trnL-trnF intergenic spacer, partial sequence; chloroplast	1105	1105	100%	0.0	95%	KJ157767.1	1049,75	87,8%
Select seq gb|EU001351.1|	Heracleum millefolium clone trn-yy16 tRNA-Leu (trnL) gene, partial sequence; and trnL-trnF intergenic spacer, complete sequence; chloroplast	1105	1105	100%	0.0	95%	EU001351.1	1049,75	87,8%
Select seq gb|JQ041844.1|	Heracleum sphondylium voucher HSP3 tRNA-Leu (trnL) gene, complete sequence; and trnL-trnF intergenic spacer, partial sequence; plastid	1103	1103	100%	0.0	95%	JQ041844.1	1047,85	87,6%
Select seq gb|JQ041843.1|	Heracleum sphondylium voucher HSP1 tRNA-Leu (trnL) gene, complete sequence; and trnL-trnF intergenic spacer, partial sequence; plastid	1103	1103	100%	0.0	95%	JQ041843.1	1047,85	87,6%
Select seq gb|EU076599.1|	Seseli mairei tRNA-Leu (trnL) gene and trnF-trnL intergenic spacer, partial sequence; chromoplast	1103	1103	100%	0.0	95%	EU076599.1	1047,85	87,6%
Select seq gb|EU001356.1|	Heracleum oreocharis clone trn-yy08 tRNA-Leu (trnL) gene, partial sequence; and trnL-trnF intergenic spacer, complete sequence; chloroplast	1103	1103	100%	0.0	95%	EU001356.1	1047,85	87,6%
Select seq gb|AF444032.1|	Zizia aptera tRNA-Phe gene, partial sequence; tRNA-Leu gene, complete sequence; and tRNA-Thr gene, partial sequence; chloroplast genes for chloroplast products	1103	1103	100%	0.0	95%	AF444032.1	1047,85	87,6%
Select seq gb|AF444031.1|	Thaspium trifoliatum tRNA-Phe gene, partial sequence; and tRNA-Leu gene, complete sequence; chloroplast genes for chloroplast products	1103	1103	100%	0.0	95%	AF444031.1	1047,85	87,6%
Select seq gb|AF444013.1|	Cymopterus purpureus tRNA-Phe gene, partial sequence; tRNA-Leu gene, complete sequence; and tRNA-Thr gene, partial sequence; chloroplast genes for chloroplast products	1103	1103	100%	0.0	95%	AF444013.1	1047,85	87,6%
Select seq gb|AF444012.1|	Cymopterus nivalis tRNA-Phe gene, partial sequence; tRNA-Leu gene, complete sequence; and tRNA-Thr gene, partial sequence; chloroplast genes for chloroplast products	1103	1103	100%	0.0	95%	AF444012.1	1047,85	87,6%
Select seq gb|KT963038.1|	Angelica gigas voucher 13E-39-3 chloroplast, complete genome	1101	1101	100%	0.0	95%	KT963038.1	1045,95	87,5%
Select seq gb|KT852844.1|	Ostericum grosseserratum voucher 13A-14-2 chloroplast, complete genome	1101	1101	100%	0.0	95%	KT852844.1	1045,95	87,5%
Select seq gb|KT781591.1|	Angelica decursiva voucher 13Q-02-1 chloroplast, complete genome	1101	1101	100%	0.0	95%	KT781591.1	1045,95	87,5%
Select seq gb|AF432025.1|AF432025	Osmorhiza mexicana subsp. bipatriata chloroplast tRNA-Leu (trnL) gene and trnL-trnF intergenic spacer region, partial sequence	1101	1101	99%	0.0	94%	AF432025.1	1045,39	87,4%
Select seq gb|AF432017.1|	Osmorhiza occidentalis chloroplast tRNA-Leu (trnL) gene and trnL-trnF intergenic spacer region, partial sequence	601	601	49%	8,00E-168	98%	AF432017.1	0,00	0,0%

Select for downloading or viewing reports	Kh003_ITS Description	Max score	Total score	Query cover	E value	Ident	Accession	(Ident/Cover)* Max score	Deviation from top hit
Select seq emb|AJ515606.1|	Nepeta balouchestanica ITS1, 5.8S rRNA gene and ITS2	1009	1009	100%	0.0	99%	AJ515606.1	998,91	100,0%
Select seq emb|AJ515317.1|	Nepeta glomerulosa 5.8S rRNA gene, ITS1 and ITS2	1009	1009	100%	0.0	99%	AJ515317.1	998,91	100,0%
Select seq gb|KP294370.1|	Nepeta sp. Kharazian & Shiran s.n. 18S ribosomal RNA gene, partial sequence; internal transcribed spacer 1, 5.8S ribosomal RNA gene, and internal transcribed spacer 2, complete sequence; and 28S ribosomal RNA gene, partial sequence	1003	1003	100%	0.0	99%	KP294370.1	992,97	99,4%
Select seq emb|AJ515309.1|	Nepeta mirzayanii 5.8S rRNA gene, ITS1 and ITS2	902	902	100%	0.0	96%	AJ515309.1	865,92	86,7%
Select seq emb|AJ515319.1|	Nepeta scrophularioides 5.8S rRNA gene, ITS1 and ITS2	854	854	100%	0.0	94%	AJ515319.1	802,76	80,4%
Select seq emb|AJ515320.1|	Nepeta kurdica 5.8S rRNA gene, ITS1 and ITS2	848	848	100%	0.0	94%	AJ515320.1	797,12	79,8%
Select seq gb|JQ669126.1|	Nepeta cataria voucher B. Drew 72 18S ribosomal RNA gene, internal transcribed spacer 1, 5.8S ribosomal RNA gene, internal transcribed spacer 2, and 26S ribosomal RNA gene, region	833	833	99%	0.0	94%	JQ669126.1	790,93	79,2%
Select seq emb|AJ515306.1|	Nepeta isaurica 5.8S rRNA gene, ITS1 and ITS2	839	839	100%	0.0	94%	AJ515306.1	788,66	79,0%
Select seq gb|KF765442.1|	Nepeta deflersiana 18S ribosomal RNA gene, partial sequence; internal transcribed spacer 1, 5.8S ribosomal RNA gene, and internal transcribed spacer 2, complete sequence; and 28S ribosomal RNA gene, partial sequence	835	835	100%	0.0	94%	KF765442.1	784,90	78,6%
Select seq emb|AJ515312.1|	Nepeta heliotropifolia 5.8S rRNA gene, ITS1 and ITS2	835	835	100%	0.0	94%	AJ515312.1	784,90	78,6%
Select seq gb|JQ669097.1|	Dracocephalum parviflorum voucher Thomas s.n. 18S ribosomal RNA gene, internal transcribed spacer 1, 5.8S ribosomal RNA gene, internal transcribed spacer 2, and 26S ribosomal RNA gene, region	761	761	91%	0.0	93%	JQ669097.1	777,73	77,9%
Select seq gb|DQ667301.1|	Nepeta cataria isolate x220 18S ribosomal RNA gene, partial sequence; internal transcribed spacer 1, 5.8S ribosomal RNA gene, and internal transcribed spacer 2, complete sequence; and 28S ribosomal RNA gene, partial sequence	826	826	99%	0.0	93%	DQ667301.1	775,94	77,7%
Select seq emb|AJ515161.1|	Nepeta congesta var. cryptantha 5.8S rRNA gene, ITS1 and ITS2	832	832	100%	0.0	93%	AJ515161.1	773,76	77,5%
Select seq emb|AJ515313.1|	Nepeta cataria 5.8S rRNA gene, ITS1 and ITS2	832	832	100%	0.0	93%	AJ515313.1	773,76	77,5%
Select seq gb|GQ456153.1|	Nepeta glutinosa internal transcribed spacer 1, partial sequence; 5.8S ribosomal RNA gene, complete sequence; and internal transcribed spacer 2, partial sequence	771	771	93%	0.0	93%	GQ456153.1	771,00	77,2%
Select seq emb|AJ421035.1|	Nepeta fissa 5S rRNA gene, ITS 1 and 2	826	826	100%	0.0	93%	AJ421035.1	768,18	76,9%
Select seq gb|GQ456140.1|	Dracocephalum argunense internal transcribed spacer 1, partial sequence; 5.8S ribosomal RNA gene, complete sequence; and internal transcribed spacer 2, partial sequence	747	747	91%	0.0	93%	GQ456140.1	763,42	76,4%
Select seq gb|JQ669109.1|	Lophanthus lipskyanus voucher Vassiljeva s.n. 18S ribosomal RNA gene, internal transcribed spacer 1, 5.8S ribosomal RNA gene, internal transcribed spacer 2, and 26S ribosomal RNA gene, region	771	771	94%	0.0	93%	JQ669109.1	762,80	76,4%
Select seq emb|AJ420996.1|	Nepeta oxyodonta 5.8S rRNA gene, internal transcribed spacer 1 (ITS1) and internal transcribed spacer 2 (ITS2)	787	787	96%	0.0	93%	AJ420996.1	762,41	76,3%
Select seq gb|GQ456154.1|	Nepeta longibracteata internal transcribed spacer 1, partial sequence; 5.8S ribosomal RNA gene, complete sequence; and internal transcribed spacer 2, partial sequence	793	793	97%	0.0	93%	GQ456154.1	760,30	76,1%
Select seq emb|AJ421039.1|	Nepeta sibirica 5.8S rRNA gene, ITS 1 and 2	815	815	100%	0.0	93%	AJ421039.1	757,95	75,9%
Select seq emb|AJ421034.1|	Nepeta schiraziana 23S rRNA gene, ITS 1 and 2	815	815	100%	0.0	93%	AJ421034.1	757,95	75,9%
Select seq gb|JQ669105.1|	Hymenocrater bituminosus voucher K. Tamanyan & George Fayvush 18S ribosomal RNA gene, internal transcribed spacer 1, 5.8S ribosomal RNA gene, internal transcribed spacer 2, and 26S ribosomal RNA gene, region	765	765	94%	0.0	93%	JQ669105.1	756,86	75,8%
Select seq emb|AJ515305.1|	Nepeta mussinii 5.8S rRNA gene, ITS1 and ITS2	813	813	100%	0.0	93%	AJ515305.1	756,09	75,7%
Select seq emb|AJ421038.1|	Nepeta wettsteinii 5.8S rRNA gene, ITS 1 and 2	813	813	100%	0.0	93%	AJ421038.1	756,09	75,7%
Select seq emb|AJ420995.1|	Nepeta laxiflora 5.8S rRNA gene, internal transcribed spacer 1 (ITS1) and internal transcribed spacer 2 (ITS2)	780	780	96%	0.0	93%	AJ420995.1	755,63	75,6%

Select seq gb|GQ456138.1|	Agastache scrophulariifolia internal transcribed spacer 1, partial sequence; 5.8S ribosomal RNA gene, complete sequence; and internal transcribed spacer 2, partial sequence	737	737	91%	0.0	93%	GQ456138.1	753,20	75,4%
Select seq emb|AJ421001.1|	Agastache barberi 5.8S rRNA gene, internal transcribed spacer 1 (ITS1) and internal transcribed spacer 2 (ITS2)	737	737	91%	0.0	93%	AJ421001.1	753,20	75,4%
Select seq gb|DQ667245.1|	Glechoma hederacea isolate x079 18S ribosomal RNA gene, partial sequence; internal transcribed spacer 1, 5.8S ribosomal RNA gene, and internal transcribed spacer 2, complete sequence; and 28S ribosomal RNA gene, partial sequence	745	745	92%	0.0	93%	DQ667245.1	753,10	75,4%
Select seq emb|AJ515314.1|	Nepeta saccharata 5.8S rRNA gene, ITS1 and ITS2	808	808	100%	0.0	93%	AJ515314.1	751,44	75,2%
Select seq gb|GQ456142.1|	Glechoma hederacea clone 1 internal transcribed spacer 1, partial sequence; 5.8S ribosomal RNA gene, complete sequence; and internal transcribed spacer 2, partial sequence	739	739	92%	0.0	93%	GQ456142.1	747,03	74,8%
Select seq gb|JQ669076.1|	Agastache rugosa voucher H. Kanai, K Hasagawa, K. Ohkubo 8916 18S ribosomal RNA gene, internal transcribed spacer 1, 5.8S ribosomal RNA gene, internal transcribed spacer 2, and 26S ribosomal RNA gene, region	734	734	91%	0.0	92%	JQ669076.1	742,07	74,3%
Select seq gb|GQ456150.1|	Marmoritis complanatum internal transcribed spacer 1, partial sequence; 5.8S ribosomal RNA gene, complete sequence; and internal transcribed spacer 2, partial sequence	749	749	93%	0.0	92%	GQ456150.1	740,95	74,2%
Select seq gb|KP058324.1|	Agastache rugosa voucher JKTM-1-000077 18S ribosomal RNA gene, partial sequence; internal transcribed spacer 1, 5.8S ribosomal RNA gene, and internal transcribed spacer 2, complete sequence; and 26S ribosomal RNA gene, partial sequence	732	732	91%	0.0	92%	KP058324.1	740,04	74,1%
Select seq gb|KM051459.1|	Schizonepeta tenuifolia isolate TKM201404 18S ribosomal RNA gene, partial sequence; internal transcribed spacer 1, 5.8S ribosomal RNA gene, and internal transcribed spacer 2, complete sequence; and 26S ribosomal RNA gene, partial sequence	732	732	91%	0.0	92%	KM051459.1	740,04	74,1%
Select seq gb|JN802670.1|	Schizonepeta tenuifolia clone HB11 18S ribosomal RNA gene, partial sequence; internal transcribed spacer 1, 5.8S ribosomal RNA gene, and internal transcribed spacer 2, complete sequence; and 28S ribosomal RNA gene, partial sequence	732	732	91%	0.0	92%	JN802670.1	740,04	74,1%
Select seq dbj|AB557591.1|	Schizonepeta tenuifolia genes for 18S rRNA, ITS1, 5.8S rRNA, ITS2, 26S rRNA, partial and complete sequence	732	732	91%	0.0	92%	AB557591.1	740,04	74,1%
Select seq gb|GQ456137.1|	Agastache rugosa internal transcribed spacer 1, partial sequence; 5.8S ribosomal RNA gene, complete sequence; and internal transcribed spacer 2, partial sequence	732	732	91%	0.0	92%	GQ456137.1	740,04	74,1%
Select seq gb|GQ456135.1|	Agastache nepetoides internal transcribed spacer 1, partial sequence; 5.8S ribosomal RNA gene, complete sequence; and internal transcribed spacer 2, partial sequence	732	732	91%	0.0	92%	GQ456135.1	740,04	74,1%
Select seq gb|EU383034.1|	Schizonepeta tenuifolia internal transcribed spacer 1, partial sequence; 5.8S ribosomal RNA gene, complete sequence; and internal transcribed spacer 2, partial sequence	732	732	91%	0.0	92%	EU383034.1	740,04	74,1%
Select seq gb|DQ667247.1|	Agastache urticifolia isolate x090 18S ribosomal RNA gene, partial sequence; internal transcribed spacer 1, 5.8S ribosomal RNA gene, and internal transcribed spacer 2, complete sequence; and 28S ribosomal RNA gene, partial sequence	732	732	91%	0.0	92%	DQ667247.1	740,04	74,1%
Select seq gb|AY506660.1|	Agastache foeniculum internal transcribed spacer 1, partial sequence; 5.8S ribosomal RNA gene, complete sequence; and internal transcribed spacer 2, partial sequence	730	730	91%	0.0	92%	AY506660.1	738,02	73,9%
Select seq gb|KM886739.1|	Meehania fargesii voucher D. G. Zhang 6502 (KUN) internal transcribed spacer 1, partial sequence; 5.8S ribosomal RNA gene, complete sequence; and internal transcribed spacer 2, partial sequence	721	721	90%	0.0	92%	KM886739.1	737,02	73,8%
Select seq gb|GQ456149.1|	Glechoma sardoa internal transcribed spacer 1, partial sequence; 5.8S ribosomal RNA gene, complete sequence; and internal transcribed spacer 2, partial sequence	736	736	92%	0.0	92%	GQ456149.1	736,00	73,7%
Select seq gb|GQ456139.1|	Agastache urticifolia internal transcribed spacer 1, partial sequence; 5.8S ribosomal RNA gene, complete sequence; and internal transcribed spacer 2, partial sequence	726	726	91%	0.0	92%	GQ456139.1	733,98	73,5%
Select seq gb|GQ456133.1|	Agastache anethiodora internal transcribed spacer 1, partial sequence; 5.8S ribosomal RNA gene, complete sequence; and internal transcribed spacer 2, partial sequence	726	726	91%	0.0	92%	GQ456133.1	733,98	73,5%
Select seq gb|KM886735.1|	Meehania fargesii var. pedunculata voucher D. G. Zhang 6091 (KUN) internal transcribed spacer 1, partial sequence; 5.8S ribosomal RNA gene, complete sequence; and internal transcribed spacer 2, partial sequence	749	749	94%	0.0	92%	KM886735.1	733,06	73,4%
Select seq gb|KM886738.1|	Meehania fargesii var. pinetorum voucher C. L.Xiang 357 (KUN) internal transcribed spacer 1, partial sequence; 5.8S ribosomal RNA gene, complete sequence; and internal transcribed spacer 2, partial sequence	756	756	95%	0.0	92%	KM886738.1	732,13	73,3%
Select seq gb|KM886731.1|	Marmoritis complanata voucher T. Deng 2359 (KUN) internal transcribed spacer 1, partial sequence; 5.8S ribosomal RNA gene, complete sequence; and internal transcribed spacer 2, partial sequence	756	756	95%	0.0	92%	KM886731.1	732,13	73,3%
Select seq gb|JF708195.1|	Agastache rugosa internal transcribed spacer 1, partial sequence; 5.8S ribosomal RNA gene, complete sequence; and internal transcribed spacer 2, partial sequence	723	723	91%	0.0	92%	JF708195.1	730,95	73,2%
Select seq gb|GQ456136.1|	Agastache pallidiflora var. neomexicana internal transcribed spacer 1, partial sequence; 5.8S ribosomal RNA gene, complete sequence; and internal transcribed spacer 2, partial sequence	723	723	91%	0.0	92%	GQ456136.1	730,95	73,2%
Select seq gb|KM886734.1|	Meehania fargesii var. fargesii voucher C. L. Xiang 057 (KUN) internal transcribed spacer 1, partial sequence; 5.8S ribosomal RNA gene, complete sequence; and internal transcribed spacer 2, partial sequence	752	752	95%	0.0	92%	KM886734.1	728,25	72,9%
Select seq gb|GQ456148.1|	Glechoma hirsuta internal transcribed spacer 1, partial sequence; 5.8S ribosomal RNA gene, complete sequence; and internal transcribed spacer 2, partial sequence	728	728	92%	0.0	92%	GQ456148.1	728,00	72,9%
Select seq gb|JX073986.1|	Glechoma hederacea isolate 215 18S ribosomal RNA gene, partial sequence; internal transcribed spacer 1, 5.8S ribosomal RNA gene, and internal transcribed spacer 2, complete sequence; and 28S ribosomal RNA gene, partial sequence	758	758	96%	0.0	92%	JX073986.1	726,42	72,7%

Select seq gb|KM886747.1|	Meehania urticifolia voucher # 39114 (AJOU) internal transcribed spacer 1, partial sequence; 5.8S ribosomal RNA gene, complete sequence; and internal transcribed spacer 2, partial sequence	750	750	95% 0.0	92% KM886747.1	726,32	72,7%
Select seq gb|KM886737.1|	Meehania fargesii var. pinetorum voucher C. L.Xiang 056 (KUN) internal transcribed spacer 1, partial sequence; 5.8S ribosomal RNA gene, complete sequence; and internal transcribed spacer 2, partial sequence	750	750	95% 0.0	92% KM886737.1	726,32	72,7%
Select seq gb|JQ669111.1|	Marmoritis complanatum voucher D.E. Boufford et al., 32012 18S ribosomal RNA gene, internal transcribed spacer 1, 5.8S ribosomal RNA gene, internal transcribed spacer 2, and 26S ribosomal RNA gene, region	750	750	95% 0.0	92% JQ669111.1	726,32	72,7%
Select seq emb|AJ515307.1|	Nepeta crassifolia 5.8S rRNA gene, ITS1 and ITS2	789	789	100% 0.0	92% AJ515307.1	725,88	72,7%
Select seq emb|AJ421042.1|	Nepeta meyeri 5.8S rRNA gene, ITS 1 and 2	789	789	100% 0.0	92% AJ421042.1	725,88	72,7%
Select seq gb|GQ456152.1|	Meehania urticifolia internal transcribed spacer 1, partial sequence; 5.8S ribosomal RNA gene, complete sequence; and internal transcribed spacer 2, partial sequence	732	732	93% 0.0	92% GQ456152.1	724,13	72,5%
Select seq gb|KM886740.1|	Meehania henryi voucher D. G. Zhang 4596 (KUN) internal transcribed spacer 1, partial sequence; 5.8S ribosomal RNA gene, complete sequence; and internal transcribed spacer 2, partial sequence	723	723	92% 0.0	92% KM886740.1	723,00	72,4%
Select seq gb|JQ669099.1|	Glechoma hederacea voucher B. Drew 69 18S ribosomal RNA gene, internal transcribed spacer 1, 5.8S ribosomal RNA gene, internal transcribed spacer 2, and 26S ribosomal RNA gene, region	754	754	96% 0.0	92% JQ669099.1	722,58	72,3%
Select seq gb|JF301404.1|	Nepeta straussii isolate K10030 18S ribosomal RNA gene, partial sequence; internal transcribed spacer 1, 5.8S ribosomal RNA gene, and internal transcribed spacer 2, complete sequence; and 26S ribosomal RNA gene, partial sequence	785	785	100% 0.0	92% JF301404.1	722,20	72,3%
Select seq emb|AJ421040.1|	Nepeta straussii 5.8S rRNA gene, ITS 1 and 2	785	785	100% 0.0	92% AJ421040.1	722,20	72,3%
Select seq gb|KF800643.1|	Uncultured eukaryote clone CMH554 18S ribosomal RNA gene, partial sequence; internal transcribed spacer 1, 5.8S ribosomal RNA gene, and internal transcribed spacer 2, complete sequence; and 28S ribosomal RNA gene, partial sequence	761	761	97% 0.0	92% KF800643.1	721,77	72,3%
Select seq gb|DQ006014.1|	Glechoma hederacea voucher Beyersdorfer 14 US 18S ribosomal RNA gene, partial sequence; internal transcribed spacer 1, 5.8S ribosomal RNA gene, and internal transcribed spacer 2, complete sequence; and 28S ribosomal RNA gene, partial sequence	761	761	97% 0.0	92% DQ006014.1	721,77	72,3%
Select seq gb|GQ456151.1|	Marmoritis decolorans internal transcribed spacer 1, partial sequence; 5.8S ribosomal RNA gene, complete sequence; and internal transcribed spacer 2, partial sequence	725	725	93% 0.0	92% GQ456151.1	717,20	71,8%
Select seq gb|EU591983.1|	Schizonepeta tenuifolia internal transcribed spacer 1, partial sequence; 5.8S ribosomal RNA gene, complete sequence; and internal transcribed spacer 2, partial sequence	725	725	93% 0.0	92% EU591983.1	717,20	71,8%
Select seq gb|KM886722.1|	Glechoma longituba voucher T. Deng 415 (KUN) internal transcribed spacer 1, partial sequence; 5.8S ribosomal RNA gene, complete sequence; and internal transcribed spacer 2, partial sequence	732	732	94% 0.0	92% KM886722.1	716,43	71,7%
Select seq gb|KM886746.1|	Meehania sp. Qiu & Su 20091002 internal transcribed spacer 1, partial sequence; 5.8S ribosomal RNA gene, complete sequence; and internal transcribed spacer 2, partial sequence	739	739	95% 0.0	92% KM886746.1	715,66	71,6%
Select seq gb|KM886742.1|	Meehania henryi var. stachydifolia voucher T. Deng 2358 (KUN) internal transcribed spacer 1, partial sequence; 5.8S ribosomal RNA gene, complete sequence; and internal transcribed spacer 2, partial sequence	739	739	95% 0.0	92% KM886742.1	715,66	71,6%
Select seq gb|KM886721.1|	Glechoma longituba voucher dt 178 (KUN) internal transcribed spacer 1, partial sequence; 5.8S ribosomal RNA gene, complete sequence; and internal transcribed spacer 2, partial sequence	739	739	95% 0.0	92% KM886721.1	715,66	71,6%
Select seq gb|KM886720.1|	Glechoma biondiana var. angustituba voucher D. G. Zhang 4583 (KUN) internal transcribed spacer 1, partial sequence; 5.8S ribosomal RNA gene, complete sequence; and internal transcribed spacer 2, partial sequence	739	739	95% 0.0	92% KM886720.1	715,66	71,6%
Select seq gb|JQ669113.1|	Meehania urticifolia voucher Lai Shushen & Shan Hanrong s.n. 18S ribosomal RNA gene, internal transcribed spacer 1, 5.8S ribosomal RNA gene, internal transcribed spacer 2, and 26S ribosomal RNA gene, region	739	739	95% 0.0	92% JQ669113.1	715,66	71,6%
Select seq gb|JQ669079.1|	Cedronella canariensis voucher UCBG #2004.0788 Royl 6859 18S ribosomal RNA gene, internal transcribed spacer 1, 5.8S ribosomal RNA gene, internal transcribed spacer 2, and 26S ribosomal RNA gene, region	730	730	94% 0.0	92% JQ669079.1	714,47	71,5%
Select seq gb|AY551290.1|	Bupleurum falcatum isolate um24 18S ribosomal RNA gene, partial sequence; internal transcribed spacer 1, 5.8S ribosomal RNA gene, and internal transcribed spacer 2, complete sequence; and 26S ribosomal RNA gene, partial sequence	737	737	95% 0.0	92% AY551290.1	713,73	71,5%
Select seq gb|KM886736.1|	Meehania fargesii var. pedunculata voucher D. G. Zhang 6391 (KUN) internal transcribed spacer 1, partial sequence; 5.8S ribosomal RNA gene, complete sequence; and internal transcribed spacer 2, partial sequence	728	728	94% 0.0	92% KM886736.1	712,51	71,3%
Select seq emb|AJ515160.1|	Nepeta hormozganica 5.8S rRNA gene, ITS1 and ITS2	773	773	100% 0.0	92% AJ515160.1	711,16	71,2%
Select seq gb|KM886744.1|	Meehania montis-koyae voucher T. Deng 2356 (KUN) internal transcribed spacer 1, partial sequence; 5.8S ribosomal RNA gene, complete sequence; and internal transcribed spacer 2, partial sequence	734	734	95% 0.0	92% KM886744.1	710,82	71,2%
Select seq gb|KM886732.1|	Meehania cordata voucher dt 101 (KUN) internal transcribed spacer 1, partial sequence; 5.8S ribosomal RNA gene, complete sequence; and internal transcribed spacer 2, partial sequence	734	734	95% 0.0	92% KM886732.1	710,82	71,2%
Select seq gb|KM886725.1|	Glechoma longituba voucher dt167 (AJOU) internal transcribed spacer 1, partial sequence; 5.8S ribosomal RNA gene, complete sequence; and internal transcribed spacer 2, partial sequence	734	734	95% 0.0	92% KM886725.1	710,82	71,2%
Select seq gb|KM886723.1|	Glechoma longituba voucher T. Deng 416 (KUN) internal transcribed spacer 1, partial sequence; 5.8S ribosomal RNA gene, complete sequence; and internal transcribed spacer 2, partial sequence	734	734	95% 0.0	92% KM886723.1	710,82	71,2%

Select seq emb|AJ515308.1|	Nepeta gloeocephala 5.8S rRNA gene, ITS1 and ITS2	767	767	100% 0.0	92% AJ515308.1	705,64	70,6%
Select seq emb|AJ515315.1|	Nepeta eremophila 5.8S rRNA gene, ITS1 and ITS2	765	765	100% 0.0	92% AJ515315.1	703,80	70,5%
Select seq gb|JQ669103.1|	Meehania cf. henryi Zhiduan 960093 18S ribosomal RNA gene, internal transcribed spacer 1, 5.8S ribosomal RNA gene, internal transcribed spacer 2, and 26S ribosomal RNA gene, region	734	734	95% 0.0	91% JQ669103.1	703,09	70,4%
Select seq gb|KM886724.1|	Glechoma longituba voucher T. Deng 433 (KUN) internal transcribed spacer 1, partial sequence; 5.8S ribosomal RNA gene, complete sequence; and internal transcribed spacer 2, partial sequence	730	730	95% 0.0	91% KM886724.1	699,26	70,0%
Select seq gb|AY506655.1|	Nepeta grandiflora internal transcribed spacer 1, partial sequence; 5.8S ribosomal RNA gene, complete sequence; and internal transcribed spacer 2, partial sequence	736	736	96% 0.0	91% AY506655.1	697,67	69,8%
Select seq gb|JQ669112.1|	Meehania cordata voucher A. E. Radford 45379 18S ribosomal RNA gene, internal transcribed spacer 1, 5.8S ribosomal RNA gene, internal transcribed spacer 2, and 26S ribosomal RNA gene, region	728	728	95% 0.0	91% JQ669112.1	697,35	69,8%
Select seq gb|DQ667328.1|	Drepanocaryum sewerzowii isolate x523 18S ribosomal RNA gene, partial sequence; internal transcribed spacer 1, 5.8S ribosomal RNA gene, and internal transcribed spacer 2, complete sequence; and 28S ribosomal RNA gene, partial sequence	763	763	100% 0.0	91% DQ667328.1	694,33	69,5%
Select seq gb|KM886743.1|	Meehania montis-koyae voucher G. H. Xia 215 (KUN) internal transcribed spacer 1, partial sequence; 5.8S ribosomal RNA gene, complete sequence; and internal transcribed spacer 2, partial sequence	723	723	95% 0.0	91% KM886743.1	692,56	69,3%
Select seq emb|AJ515318.1|	Nepeta ispahanica 5.8S rRNA gene, ITS1 and ITS2	761	761	100% 0.0	91% AJ515318.1	692,51	69,3%
Select seq emb|AJ515310.1|	Nepeta bornmuelleri 5.8S rRNA gene, ITS1 and ITS2	761	761	100% 0.0	91% AJ515310.1	692,51	69,3%
Select seq emb|AJ421003.1|	Nepeta sp. Assadi 23249 5.8S rRNA gene, internal transcribed spacer 1 (ITS1) and internal transcribed spacer 2 (ITS2)	756	756	100% 0.0	91% AJ421003.1	687,96	68,9%
Select seq emb|AJ515159.1|	Nepeta crispa 5.8S rRNA gene, ITS1 and ITS2	754	754	100% 0.0	91% AJ515159.1	686,14	68,7%
Select seq emb|AJ515316.1|	Nepeta assurgens 5.8S rRNA gene, ITS1 and ITS2	750	750	100% 0.0	91% AJ515316.1	682,50	68,3%
Select seq emb|AJ421037.1|	Nepeta cephalotes 5.8S rRNA gene, ITS 1 and 2	750	750	100% 0.0	91% AJ421037.1	682,50	68,3%
Select seq emb|AJ421036.1|	Nepeta pungens 5.8S rRNA gene, ITS 1 and 2	737	737	100% 0.0	91% AJ421036.1	670,67	67,1%
Select seq emb|AJ515304.1|	Nepeta denudata 5.8S rRNA gene, ITS1 and ITS2	732	732	100% 0.0	91% AJ515304.1	666,12	66,7%
Select seq emb|AJ515311.1|	Nepeta binaloudensis 5.8S rRNA gene, ITS1 and ITS2	736	736	100% 0.0	90% AJ515311.1	662,40	66,3%

Select for downloading or viewing reports	Kh003_trnL Description	Max score	Total score	Query cover	E value	Ident	Accession	(Ident/Cover)* Max score	Deviation from top hit
Select seq emb|AJ505432.1|	Nepeta racemosa plastid trnL-trnF intergenic spacer, specimen voucher Jamzad s.n. (TARI)	1083	1083	100%	0.0	99%	AJ505432.1	1072,17	100,0%
Select seq gb|JF301391.1|	Nepeta cataria voucher B. Drew 72 tRNA-Leu (trnL) gene and trnL-trnF intergenic spacer, partial sequence; chloroplast	1066	1066	100%	0.0	99%	JF301391.1	1055,34	98,4%
Select seq gb|DQ667487.1|	Nepeta cataria isolate x220 tRNA-Leu (trnL) gene and trnL-trnF intergenic spacer, partial sequence; chloroplast	1066	1066	100%	0.0	99%	DQ667487.1	1055,34	98,4%
Select seq gb|GU381479.1|	Nepeta supina voucher M:Groeger & Schewardnadse 1466 tRNA-Leu (trnL) gene and trnL-trnF intergenic spacer, partial sequence; chloroplast	1055	1055	100%	0.0	99%	GU381479.1	1044,45	97,4%
Select seq emb|AJ505433.1|	Nepeta straussii plastid trnL-trnF intergenic spacer, specimen voucher Jamzad etal 76846 (TARI)	1044	1044	100%	0.0	98%	AJ505433.1	1023,12	95,4%
Select seq gb|AY506621.1|	Nepeta grandiflora tRNA-Leu (trnL) gene and trnL-trnF intergenic spacer, partial sequence; chloroplast	1014	1014	100%	0.0	97%	AY506621.1	983,58	91,7%
Select seq gb|JQ669045.1|	Hymenocrater bituminosus voucher K. Tamanyan & George Fayvush tRNA-Leu (trnL) gene and trnL-trnF intergenic spacer, partial sequence; plastid	913	913	99%	0.0	95%	JQ669045.1	876,11	81,7%
Select seq emb|AJ505431.1|	Nepeta menthoides plastid trnL-trnF intergenic spacer, specimen voucher Jamzad s.n. (K)	913	913	100%	0.0	95%	AJ505431.1	867,35	80,9%
Select seq gb|KM886627.1|	Marmoritis complanata voucher T. Deng 2359 (KUN) tRNA-Leu (trnL) gene, partial sequence; trnL-trnF intergenic spacer, complete sequence; and tRNA-Phe (trnF) gene, partial sequence; plastid	911	911	100%	0.0	95%	KM886627.1	865,45	80,7%
Select seq gb|JQ669049.1|	Marmoritis complanatum voucher D.E. Boufford et al., 32012 tRNA-Leu (trnL) gene and trnL-trnF intergenic spacer, partial sequence; plastid	907	907	100%	0.0	95%	JQ669049.1	861,65	80,4%
Select seq gb|FJ593457.1|	Nepeta stewartiana tRNA-Leu (trnL) gene and trnL-trnF intergenic spacer, partial sequence; chloroplast	881	881	100%	0.0	94%	FJ593457.1	828,14	77,2%
Select seq emb|AJ505430.1|	Nepeta fissa plastid trnL-trnF intergenic spacer, specimen voucher Jamzad & Nikchehreh 80486 TARI	852	1021	100%	0.0	97%	AJ505430.1	826,44	77,1%
Select seq gb|KF307433.1|	Lepechinia urbanii voucher B. Drew 135 tRNA-Leu (trnL) gene and trnL-trnF intergenic spacer, partial sequence; chloroplast	861	861	100%	0.0	93%	KF307433.1	800,73	74,7%
Select seq gb|KF307432.1|	Lepechinia speciosa voucher Cordeno 3060 tRNA-Leu (trnL) gene and trnL-trnF intergenic spacer, partial sequence; chloroplast	856	856	100%	0.0	93%	KF307432.1	796,08	74,2%
Select seq gb|KF307411.1|	Lepechinia bella voucher Rachel Jabaily s.n. tRNA-Leu (trnL) gene and trnL-trnF intergenic spacer, partial sequence; chloroplast	856	856	100%	0.0	93%	KF307411.1	796,08	74,2%
Select seq gb|JF301383.1|	Lepechinia salviifolia voucher R. Jabaily s.n. tRNA-Leu (trnL) gene and trnL-trnF intergenic spacer, partial sequence; chloroplast	856	856	100%	0.0	93%	JF301383.1	796,08	74,2%
Select seq gb|JF301382.1|	Lepechinia radula voucher B. Drew 185 tRNA-Leu (trnL) gene and trnL-trnF intergenic spacer, partial sequence; chloroplast	856	856	100%	0.0	93%	JF301382.1	796,08	74,2%
Select seq gb|DQ667492.1|	Lepechinia lancifolia isolate x232 tRNA-Leu (trnL) gene and trnL-trnF intergenic spacer, partial sequence; chloroplast	856	856	100%	0.0	93%	DQ667492.1	796,08	74,2%
Select seq gb|KF307435.1|	Lepechinia yecorana voucher Henrickson 24691 tRNA-Leu (trnL) gene and trnL-trnF intergenic spacer, partial sequence; chloroplast	850	850	100%	0.0	93%	KF307435.1	790,50	73,7%
Select seq gb|KF307430.1|	Lepechinia schiedeana voucher B. Drew 157 tRNA-Leu (trnL) gene and trnL-trnF intergenic spacer, partial sequence; chloroplast	850	850	100%	0.0	93%	KF307430.1	790,50	73,7%
Select seq gb|KF307421.1|	Lepechinia heteromorpha voucher B. Drew 192 tRNA-Leu (trnL) gene and trnL-trnF intergenic spacer, partial sequence; chloroplast	850	850	100%	0.0	93%	KF307421.1	790,50	73,7%
Select seq gb|KF307420.1|	Lepechinia graveolens voucher Fuentes 10351 tRNA-Leu (trnL) gene and trnL-trnF intergenic spacer, partial sequence; chloroplast	850	850	100%	0.0	93%	KF307420.1	790,50	73,7%
Select seq gb|KF307418.1|	Lepechinia floribunda voucher B. Drew 172 tRNA-Leu (trnL) gene and trnL-trnF intergenic spacer, partial sequence; chloroplast	850	850	100%	0.0	93%	KF307418.1	790,50	73,7%
Select seq gb|JF301361.1|	Chaunostoma mecistandrum voucher J.A. Monterrosa & R.A. Carballo 213 tRNA-Leu (trnL) gene and trnL-trnF intergenic spacer, partial sequence; chloroplast	850	850	100%	0.0	93%	JF301361.1	790,50	73,7%
Select seq gb|KF307422.1|	Lepechinia mexicana voucher B. Drew 130 tRNA-Leu (trnL) gene and trnL-trnF intergenic spacer, partial sequence; chloroplast	845	845	100%	0.0	93%	KF307422.1	785,85	73,3%
Select seq gb|JF301380.1|	Lepechinia mexicana voucher B. Drew 127 tRNA-Leu (trnL) gene and trnL-trnF intergenic spacer, partial sequence; chloroplast	845	845	100%	0.0	93%	JF301380.1	785,85	73,3%
Select seq gb|KF307416.1|	Lepechinia codon voucher B. Drew 177 tRNA-Leu (trnL) gene and trnL-trnF intergenic spacer, partial sequence; chloroplast	843	843	100%	0.0	93%	KF307416.1	783,99	73,1%

Select seq gb|JF301381.1|	Lepechinia mexicana voucher B. Drew 164 tRNA-Leu (trnL) gene and trnL-trnF intergenic spacer, partial sequence; chloroplast	839	839	100% 0.0	93% JF301381.1	780,27	72,8%
Select seq gb|JF301386.1|	Melissa officinalis voucher B. Drew 70 tRNA-Leu (trnL) gene and trnL-trnF intergenic spacer, partial sequence; chloroplast	832	832	100% 0.0	93% JF301386.1	773,76	72,2%
Select seq gb|DQ667477.1|	Melissa officinalis isolate x193 tRNA-Leu (trnL) gene and trnL-trnF intergenic spacer, partial sequence; chloroplast	832	832	100% 0.0	93% DQ667477.1	773,76	72,2%
Select seq emb|AJ505529.1|	Melissa officinalis plastid trnL-trnF intergenic spacer, specimen voucher Catino	832	832	100% 0.0	93% AJ505529.1	773,76	72,2%
Select seq gb|KF307419.1|	Lepechinia ganderi voucher B. Drew 24 tRNA-Leu (trnL) gene and trnL-trnF intergenic spacer, partial sequence; chloroplast	837	837	100% 0.0	92% KF307419.1	770,04	71,8%
Select seq gb|KF307426.1|	Lepechinia paniculata voucher B. Drew 241 tRNA-Leu (trnL) gene and trnL-trnF intergenic spacer, partial sequence; chloroplast	835	835	100% 0.0	92% KF307426.1	768,20	71,6%
Select seq gb|KM886649.1|	Prunella vulgaris voucher SNJ Exped. 20110719005 (KUN) tRNA-Leu (trnL) gene, partial sequence; trnL-trnF intergenic spacer, complete sequence; and tRNA-Phe (trnF) gene, partial sequence; plastid	832	832	100% 0.0	92% KM886649.1	765,44	71,4%
Select seq gb|EF153679.1|	Prunella grandiflora tRNA-Leu (trnL) and trnL-trnF intergenic spacer, partial sequence; chloroplast	832	832	100% 0.0	92% EF153679.1	765,44	71,4%
Select seq gb|DQ667508.1|	Prunella vulgaris isolate x314 tRNA-Leu (trnL) gene and trnL-trnF intergenic spacer, partial sequence; chloroplast	832	832	100% 0.0	92% DQ667508.1	765,44	71,4%
Select seq gb|AY506619.1|	Prunella vulgaris tRNA-Leu (trnL) gene and trnL-trnF intergenic spacer, partial sequence; chloroplast	832	832	100% 0.0	92% AY506619.1	765,44	71,4%
Select seq gb|KF307414.1|	Lepechinia calycina voucher B. Drew 20 tRNA-Leu (trnL) gene and trnL-trnF intergenic spacer, partial sequence; chloroplast	828	828	100% 0.0	92% KF307414.1	761,76	71,0%
Select seq gb|JF301375.1|	Lepechinia calycina voucher B. Drew 197 tRNA-Leu (trnL) gene and trnL-trnF intergenic spacer, partial sequence; chloroplast	828	828	100% 0.0	92% JF301375.1	761,76	71,0%
Select seq gb|DQ667494.1|	Lepechinia calycina isolate x246 tRNA-Leu (trnL) gene and trnL-trnF intergenic spacer, partial sequence; chloroplast	828	828	100% 0.0	92% DQ667494.1	761,76	71,0%
Select seq gb|AY570460.1|	Lepechinia fragrans voucher JBW 1333 tRNA-Leu and trnL-trnF intergenic spacer, partial sequence; chloroplast	828	828	100% 0.0	92% AY570460.1	761,76	71,0%
Select seq gb|EF153681.1|	Prunella asiatica tRNA-Leu (trnL) and trnL-trnF intergenic spacer, partial sequence; chloroplast	826	826	100% 0.0	92% EF153681.1	759,92	70,9%
Select seq gb|KF307429.1|	Lepechinia salviae voucher R. Jabaily s.n. tRNA-Leu (trnL) gene and trnL-trnF intergenic spacer, partial sequence; chloroplast	822	822	100% 0.0	92% KF307429.1	756,24	70,5%
Select seq gb|KF307415.1|	Lepechinia caulescens voucher B. Drew 149 tRNA-Leu (trnL) gene and trnL-trnF intergenic spacer, partial sequence; chloroplast	822	822	100% 0.0	92% KF307415.1	756,24	70,5%
Select seq gb|JF301376.1|	Lepechinia caulescens voucher B. Drew 106 tRNA-Leu (trnL) gene and trnL-trnF intergenic spacer, partial sequence; chloroplast	822	822	100% 0.0	92% JF301376.1	756,24	70,5%
Select seq gb|AY570459.1|	Lepechinia chamaedryoides voucher JBW 2537 tRNA-Leu and trnL-trnF intergenic spacer, partial sequence; chloroplast	822	822	100% 0.0	92% AY570459.1	756,24	70,5%
Select seq gb|JF301360.1|	Cedronella canariensis voucher UCBG 2004.0788 tRNA-Leu (trnL) gene and trnL-trnF intergenic spacer, partial sequence; chloroplast	821	821	100% 0.0	92% JF301360.1	755,32	70,4%
Select seq gb|GU381484.1|	Micromeria flagellaris voucher E:van der Werff & McPherson 13570 tRNA-Leu (trnL) gene and trnL-trnF intergenic spacer, partial sequence; chloroplast	821	821	100% 0.0	92% GU381484.1	755,32	70,4%
Select seq gb|GU381483.1|	Micromeria flagellaris voucher E:Clement et al 2140 tRNA-Leu (trnL) gene and trnL-trnF intergenic spacer, partial sequence; chloroplast	821	821	100% 0.0	92% GU381483.1	755,32	70,4%
Select seq gb|AY506622.1|	Cedronella canariensis tRNA-Leu (trnL) gene and trnL-trnF intergenic spacer, partial sequence; chloroplast	821	821	100% 0.0	92% AY506622.1	755,32	70,4%
Select seq gb|AY570458.1|	Lepechinia calycina voucher JBW 1344 tRNA-Leu and trnL-trnF intergenic spacer, partial sequence; chloroplast	821	821	100% 0.0	92% AY570458.1	755,32	70,4%
Select seq gb|KF307417.1|	Lepechinia dioica voucher B. Drew 232 tRNA-Leu (trnL) gene and trnL-trnF intergenic spacer, partial sequence; chloroplast	817	817	100% 0.0	92% KF307417.1	751,64	70,1%
Select seq gb|JF301378.1|	Lepechinia hastata voucher B. Drew 44 tRNA-Leu (trnL) gene and trnL-trnF intergenic spacer, partial sequence; chloroplast	817	817	100% 0.0	92% JF301378.1	751,64	70,1%
Select seq gb|DQ667495.1|	Cleonia lusitanica isolate x255 tRNA-Leu (trnL) gene and trnL-trnF intergenic spacer, partial sequence; chloroplast	817	817	100% 0.0	92% DQ667495.1	751,64	70,1%
Select seq gb|JF301377.1|	Lepechinia flammea tRNA-Leu (trnL) gene and trnL-trnF intergenic spacer, partial sequence; chloroplast	819	819	100% 0.0	91% JF301377.1	745,29	69,5%

Select seq gb|AY840207.1|	Thymbra spicata tRNA-Leu (trnL) gene and trnL-trnF intergenic spacer, partial sequence; chloroplast	809	809	100% 0.0	92% AY840207.1	744,28	69,4%
Select seq gb|EF153682.1|	Prunella hispida tRNA-Leu (trnL) and trnL-trnF intergenic spacer, partial sequence; chloroplast	808	808	100% 0.0	92% EF153682.1	743,36	69,3%
Select seq gb|GU381496.1|	Killickia pilosa voucher M:Braeuchler 3832 tRNA-Leu (trnL) gene and trnL-trnF intergenic spacer, partial sequence; chloroplast	806	806	100% 0.0	92% GU381496.1	741,52	69,2%
Select seq gb|GU381495.1|	Killickia pilosa voucher M:Braeuchler 3810 tRNA-Leu (trnL) gene and trnL-trnF intergenic spacer, partial sequence; chloroplast	806	806	100% 0.0	92% GU381495.1	741,52	69,2%
Select seq gb|GU381494.1|	Killickia pilosa voucher NU<ZAF>:Potgieter & Thompson 739 tRNA-Leu (trnL) gene and trnL-trnF intergenic spacer, partial sequence; chloroplast	806	806	100% 0.0	92% GU381494.1	741,52	69,2%
Select seq gb|GU381493.1|	Killickia grandiflora voucher M:Braeuchler 3811 tRNA-Leu (trnL) gene and trnL-trnF intergenic spacer, partial sequence; chloroplast	806	806	100% 0.0	92% GU381493.1	741,52	69,2%
Select seq gb|GU381492.1|	Killickia grandiflora voucher NU<ZAF>:Hilliard & Burtt 18579 tRNA-Leu (trnL) gene and trnL-trnF intergenic spacer, partial sequence; chloroplast	806	806	100% 0.0	92% GU381492.1	741,52	69,2%
Select seq gb|GU381489.1|	Killickia lutea voucher NU<ZAF>:Hilliard & Burtt 9876 tRNA-Leu (trnL) gene and trnL-trnF intergenic spacer, partial sequence; chloroplast	806	806	100% 0.0	92% GU381489.1	741,52	69,2%
Select seq gb|GU381488.1|	Killickia compacta voucher M:Braeuchler 3816 tRNA-Leu (trnL) gene and trnL-trnF intergenic spacer, partial sequence; chloroplast	806	806	100% 0.0	92% GU381488.1	741,52	69,2%
Select seq gb|KF307428.1|	Lepechinia rufocampii voucher B. Drew 244 tRNA-Leu (trnL) gene and trnL-trnF intergenic spacer, partial sequence; chloroplast	804	804	100% 0.0	92% KF307428.1	739,68	69,0%
Select seq gb|JF301379.1|	Lepechinia lamiifolia voucher B. Drew 178 tRNA-Leu (trnL) gene and trnL-trnF intergenic spacer, partial sequence; chloroplast	804	804	100% 0.0	92% JF301379.1	739,68	69,0%
Select seq gb|GU381632.1|	Thymbra spicata voucher M:Braeuchler 4548 tRNA-Leu (trnL) gene and trnL-trnF intergenic spacer, partial sequence; chloroplast	806	806	100% 0.0	91% GU381632.1	733,46	68,4%
Select seq gb|JF301401.1|	Thymbra capitata voucher UCBG 96.0817 tRNA-Leu (trnL) gene and trnL-trnF intergenic spacer, partial sequence; chloroplast	804	804	100% 0.0	91% JF301401.1	731,64	68,2%
Select seq gb|GU381629.1|	Thymbra capitata voucher M:Braeuchler 2518 tRNA-Leu (trnL) gene and trnL-trnF intergenic spacer, partial sequence; chloroplast	804	804	100% 0.0	91% GU381629.1	731,64	68,2%
Select seq gb|AY570481.1|	Salvia greatai voucher JBW 2511 tRNA-Leu and trnL-trnF intergenic spacer, partial sequence; chloroplast	802	802	100% 0.0	91% AY570481.1	729,82	68,1%
Select seq gb|GU381627.1|	Thymbra sintenisii subsp. isaurica voucher E:Goener 12628 tRNA-Leu (trnL) gene and trnL-trnF intergenic spacer, partial sequence; chloroplast	798	798	100% 0.0	91% GU381627.1	726,18	67,7%
Select seq gb|GU381485.1|	Micromeria sphaerophylla voucher E:Lewis et al 1064 tRNA-Leu (trnL) gene and trnL-trnF intergenic spacer, partial sequence; chloroplast	797	797	100% 0.0	91% GU381485.1	725,27	67,6%
Select seq gb|KR063656.1|	Satureja pilosa subsp. origanita tRNA-Leu (trnL) gene, partial sequence; trnL-trnF intergenic spacer, complete sequence; and tRNA-Phe (trnF) gene, partial sequence; chloroplast	795	795	100% 0.0	91% KR063656.1	723,45	67,5%
Select seq gb|KM886626.1|	Lycopus cavaleriei voucher SNJ Exped. 20110807071 (KUN) tRNA-Leu (trnL) gene, partial sequence; trnL-trnF intergenic spacer, complete sequence; and tRNA-Phe (trnF) gene, partial sequence; plastid	795	795	100% 0.0	91% KM886626.1	723,45	67,5%
Select seq gb|JQ669067.1|	Satureja montana voucher UCBG 2002.0593, Forbes s.n tRNA-Leu (trnL) gene and trnL-trnF intergenic spacer, partial sequence; plastid	795	795	100% 0.0	91% JQ669067.1	723,45	67,5%
Select seq gb|JQ669022.1|	Agastache rugosa voucher H. Kanai, K Hasagawa, K. Ohkubo 8916 tRNA-Leu (trnL) gene and trnL-trnF intergenic spacer, partial sequence; plastid	795	795	100% 0.0	91% JQ669022.1	723,45	67,5%
Select seq gb|GU381621.1|	Satureja cuneifolia voucher M:Rechinger 11142 tRNA-Leu (trnL) gene and trnL-trnF intergenic spacer, partial sequence; chloroplast	795	795	100% 0.0	91% GU381621.1	723,45	67,5%
Select seq gb|GU381612.1|	Gontscharovia popovii voucher BM<GBR-LONDON>:Schmid 2419 tRNA-Leu (trnL) gene and trnL-trnF intergenic spacer, partial sequence; chloroplast	795	795	100% 0.0	91% GU381612.1	723,45	67,5%
Select seq gb|GU381480.1|	Agastache foeniculum voucher M:Braeuchler 2513 tRNA-Leu (trnL) gene and trnL-trnF intergenic spacer, partial sequence; chloroplast	795	795	100% 0.0	91% GU381480.1	723,45	67,5%
Select seq gb|GU381478.1|	Lycopus europaeus voucher M:Braeuchler 2505 tRNA-Leu (trnL) gene and trnL-trnF intergenic spacer, partial sequence; chloroplast	795	795	100% 0.0	91% GU381478.1	723,45	67,5%
Select seq gb|EU244612.1|	Agastache rugosa tRNA-Leu (trnL) gene and trnL-trnF intergenic spacer, partial sequence	795	795	100% 0.0	91% EU244612.1	723,45	67,5%
Select seq gb|AY840179.1|	Satureja montana tRNA-Leu (trnL) gene and trnL-trnF intergenic spacer, partial sequence; chloroplast	795	795	100% 0.0	91% AY840179.1	723,45	67,5%
Select seq gb|KF307412.1|	Lepechinia betonicifolia voucher B. Drew 224 tRNA-Leu (trnL) gene and trnL-trnF intergenic spacer, partial sequence; chloroplast	793	793	100% 0.0	91% KF307412.1	721,63	67,3%

Select seq gb|GU381517.1|	Clinopodium barosmum voucher BM<GBR-LONDON>:McLaren N193 tRNA-Leu (trnL) gene and trnL-trnF intergenic spacer, partial sequence; chloroplast	793	793	100%	0.0	91%	GU381517.1	721,63	67,3%
Select seq gb|GU381516.1|	Clinopodium wardii voucher BM<GBR-LONDON>:Ludlow et al. 14234 tRNA-Leu (trnL) gene and trnL-trnF intergenic spacer, partial sequence; chloroplast	793	793	100%	0.0	91%	GU381516.1	721,63	67,3%
Select seq gb|GU381515.1|	Clinopodium hydaspidis voucher BM<GBR-LONDON>:Mohd 133 tRNA-Leu (trnL) gene and trnL-trnF intergenic spacer, partial sequence; chloroplast	793	793	100%	0.0	91%	GU381515.1	721,63	67,3%
Select seq gb|GU381514.1|	Clinopodium nepalense voucher FR:Stainton 6024 tRNA-Leu (trnL) gene and trnL-trnF intergenic spacer, partial sequence; chloroplast	793	793	100%	0.0	91%	GU381514.1	721,63	67,3%
Select seq gb|GU381513.1|	Clinopodium nepalense voucher BM<GBR-LONDON>:Mikage et al. 9550294 tRNA-Leu (trnL) gene and trnL-trnF intergenic spacer, partial sequence; chloroplast	793	793	100%	0.0	91%	GU381513.1	721,63	67,3%
Select seq gb|GU381511.1|	Clinopodium piperitum voucher BM<GBR-LONDON>:Vickery 454 tRNA-Leu (trnL) gene and trnL-trnF intergenic spacer, partial sequence; chloroplast	793	793	100%	0.0	91%	GU381511.1	721,63	67,3%
Select seq gb|GU381481.1|	Micromeria cf. madagascariensis Morawetz 205 tRNA-Leu (trnL) gene and trnL-trnF intergenic spacer, partial sequence; chloroplast	793	793	100%	0.0	91%	GU381481.1	721,63	67,3%
Select seq gb|DQ667493.1|	Lepechinia conferta isolate x234 tRNA-Leu (trnL) gene and trnL-trnF intergenic spacer, partial sequence; chloroplast	793	793	100%	0.0	91%	DQ667493.1	721,63	67,3%
Select seq gb|DQ667488.1|	Lycopus uniflorus isolate x221 tRNA-Leu (trnL) gene and trnL-trnF intergenic spacer, partial sequence; chloroplast	793	793	100%	0.0	91%	DQ667488.1	721,63	67,3%
Select seq gb|AY506611.1|	Satureja hortensis tRNA-Leu (trnL) gene and trnL-trnF intergenic spacer, partial sequence; chloroplast	791	791	100%	0.0	91%	AY506611.1	719,81	67,1%
Select seq gb|JF301384.1|	Lophanthus lipskyanus voucher Vassiljeva s.n. tRNA-Leu (trnL) gene and trnL-trnF intergenic spacer, partial sequence; chloroplast	745	745	100%	0.0	88%	JF301384.1	655,60	61,1%
								0,00	0,0%
								0,00	0,0%
								0,00	0,0%
								0,00	0,0%
								0,00	0,0%

Select for downloading or viewing reports	Kh004_ITS Description	Max score	Total score	Query cover	E value	Ident	Accession	(Ident/Cover)* Max score	Deviation from top hit
Select seq gb|GU381444.1|	Satureja pilosa voucher M:Braeuchler 4448 internal transcribed spacer 1, partial sequence; 5.8S ribosomal RNA gene, complete sequence; and internal transcribed spacer 2, partial sequence	769	769	86%	0.0	91%	GU381444.1	813,71	100,0%
Select seq gb|GU381442.1|	Satureja montana voucher M:Braeuchler 2509 internal transcribed spacer 1, partial sequence; 5.8S ribosomal RNA gene, complete sequence; and internal transcribed spacer 2, partial sequence	763	763	86%	0.0	91%	GU381442.1	807,36	99,2%
Select seq gb|GU381445.1|	Satureja spinosa voucher M:Braeuchler 4449 internal transcribed spacer 1, partial sequence; 5.8S ribosomal RNA gene, complete sequence; and internal transcribed spacer 2, partial sequence	758	758	86%	0.0	91%	GU381445.1	802,07	98,6%
Select seq gb|GU381443.1|	Satureja parnassica voucher M:Braeuchler 4453 internal transcribed spacer 1, partial sequence; 5.8S ribosomal RNA gene, complete sequence; and internal transcribed spacer 2, partial sequence	752	752	86%	0.0	91%	GU381443.1	795,72	97,8%
Select seq gb|EU823288.1|	Satureja subspicata internal transcribed spacer 1, partial sequence; 5.8S ribosomal RNA gene, complete sequence; and internal transcribed spacer 2, partial sequence	776	776	90%	0.0	91%	EU823288.1	784,62	96,4%
Select seq gb|JQ669136.1|	Satureja thymbra voucher UCBG 2002.0540, Forbes s.n. 18S ribosomal RNA gene, internal transcribed spacer 1, 5.8S ribosomal RNA gene, internal transcribed spacer 2, and 26S ribosomal RNA gene, region	749	749	88%	0.0	90%	JQ669136.1	766,02	94,1%
Select seq gb|AY227143.1|	Satureja hortensis internal transcribed spacer 1, partial sequence; 5.8S ribosomal RNA, complete sequence; and internal transcribed spacer 2, partial sequence	767	767	91%	0.0	90%	AY227143.1	758,57	93,2%
Select seq gb|DQ017569.1|	Minthostachys dimorpha voucher Humbert 30679 (US) internal transcribed spacer 1, partial sequence; 5.8S ribosomal RNA gene and internal transcribed spacer 2, complete sequence; and 28S ribosomal RNA gene, partial sequence	588	588	71%	5,00E-164	90%	DQ017569.1	745,35	91,6%
Select seq gb|EU823289.1|	Satureja visianii internal transcribed spacer 1, partial sequence; 5.8S ribosomal RNA gene, complete sequence; and internal transcribed spacer 2, partial sequence	743	743	90%	0.0	90%	EU823289.1	743,00	91,3%
Select seq gb|DQ017570.1|	Minthostachys elongata voucher Beck 26146 (LPB) internal transcribed spacer 1, partial sequence; 5.8S ribosomal RNA gene, complete sequence; and internal transcribed spacer 2, partial sequence	586	586	71%	2,00E-163	90%	DQ017570.1	742,82	91,3%
Select seq gb|JQ669135.1|	Satureja montana voucher UCBG 2002.0593, Forbes s.n 18S ribosomal RNA gene, internal transcribed spacer 1, 5.8S ribosomal RNA gene, internal transcribed spacer 2, and 26S ribosomal RNA gene, region	815	815	99%	0.0	90%	JQ669135.1	740,91	91,1%
Select seq gb|GU381438.1|	Gontscharovia popovii voucher M:Vvedensky s.n. internal transcribed spacer 1, partial sequence; 5.8S ribosomal RNA gene, complete sequence; and internal transcribed spacer 2, partial sequence	719	719	88%	0.0	89%	GU381438.1	727,17	89,4%
Select seq gb|GU381441.1|	Satureja thymbra voucher M:Braeuchler 2896 internal transcribed spacer 1, partial sequence; 5.8S ribosomal RNA gene, complete sequence; and internal transcribed spacer 2, partial sequence	691	691	86%	0.0	89%	GU381441.1	715,10	87,9%
Select seq gb|KC591661.1|	Mentha arvensis voucher UC1862058 internal transcribed spacer 1, partial sequence; 5.8S ribosomal RNA gene and internal transcribed spacer 2, complete sequence; and 28S ribosomal RNA gene, partial sequence	625	625	78%	4,00E-175	89%	KC591661.1	713,14	87,6%
Select seq gb|DQ017572.1|	Minthostachys verticillata voucher Schmidt-Lebuhn 294 (GOET) internal transcribed spacer 1, partial sequence; 5.8S ribosomal RNA gene and internal transcribed spacer 2, complete sequence; and 28S ribosomal RNA gene, partial sequence	623	623	78%	1,00E-174	89%	DQ017572.1	710,86	87,4%
Select seq gb|KF735671.1|	Mentha aquatica strain YS internal transcribed spacer 1, partial sequence; 5.8S ribosomal RNA gene, complete sequence; and internal transcribed spacer 2, partial sequence	612	612	77%	3,00E-171	89%	KF735671.1	707,38	86,9%
Select seq gb|KF735670.1|	Mentha aquatica strain TT internal transcribed spacer 1, partial sequence; 5.8S ribosomal RNA gene, complete sequence; and internal transcribed spacer 2, partial sequence	612	612	77%	3,00E-171	89%	KF735670.1	707,38	86,9%
Select seq gb|KC591663.1|	Acanthomintha lanceolata voucher SDSU17310 internal transcribed spacer 1, partial sequence; 5.8S ribosomal RNA gene and internal transcribed spacer 2, complete sequence; and 28S ribosomal RNA gene, partial sequence	619	619	78%	2,00E-173	89%	KC591663.1	706,29	86,8%
Select seq gb|JQ230966.1|	Mentha x piperita voucher SBB-1151 internal transcribed spacer 1, partial sequence; 5.8S ribosomal RNA gene and internal transcribed spacer 2, complete sequence; and 28S ribosomal RNA gene, partial sequence	619	619	78%	2,00E-173	89%	JQ230966.1	706,29	86,8%
Select seq gb|DQ667325.1|	Mentha arvensis isolate x424 18S ribosomal RNA gene, partial sequence; internal transcribed spacer 1, 5.8S ribosomal RNA gene, and internal transcribed spacer 2, complete sequence; and 28S ribosomal RNA gene, partial sequence	619	619	78%	2,00E-173	89%	DQ667325.1	706,29	86,8%
Select seq gb|DQ017576.1|	Minthostachys setosa voucher Schmidt-Lebuhn 551 (GOET) internal transcribed spacer 1, partial sequence; 5.8S ribosomal RNA gene and internal transcribed spacer 2, complete sequence; and 28S ribosomal RNA gene, partial sequence	619	619	78%	2,00E-173	89%	DQ017576.1	706,29	86,8%
Select seq gb|AY506638.1|	Minthostachys mollis internal transcribed spacer 1, partial sequence; 5.8S ribosomal RNA gene, complete sequence; and internal transcribed spacer 2, partial sequence	619	619	78%	2,00E-173	89%	AY506638.1	706,29	86,8%
Select seq gb|JQ669073.1|	Acanthomintha lanceolata voucher Crosby & Morin 14383 18S ribosomal RNA gene, internal transcribed spacer 1, 5.8S ribosomal RNA gene, internal transcribed spacer 2, and 26S ribosomal RNA gene, region	610	610	77%	1,00E-170	89%	JQ669073.1	705,06	86,6%
Select seq gb|DQ017575.1|	Minthostachys acutifolia voucher Schmidt-Lebuhn 268 (GOET) internal transcribed spacer 1, partial sequence; 5.8S ribosomal RNA gene and internal transcribed spacer 2, complete sequence; and 28S ribosomal RNA gene, partial sequence	617	617	78%	7,00E-173	89%	DQ017575.1	704,01	86,5%
Select seq gb|DQ017578.1|	Minthostachys spicata voucher Schmidt-Lebuhn 496 (GOET) internal transcribed spacer 1, partial sequence; 5.8S ribosomal RNA gene and internal transcribed spacer 2, complete sequence; and 28S ribosomal RNA gene, partial sequence	616	616	78%	2,00E-172	89%	DQ017578.1	702,87	86,4%
Select seq gb|DQ017573.1|	Minthostachys andina voucher Schmidt-Lebuhn 277 (GOET) internal transcribed spacer 1, partial sequence; 5.8S ribosomal RNA gene and internal transcribed spacer 2, complete sequence; and 28S ribosomal RNA gene, partial sequence	616	616	78%	2,00E-172	89%	DQ017573.1	702,87	86,4%

Select seq gb|DQ017557.1|	Clinopodium jacquelinae voucher Schmidt-Lebuhn 445 (GOET) internal transcribed spacer 1, partial sequence; 5.8S ribosomal RNA gene and internal transcribed spacer 2, complete sequence; and 28S ribosomal RNA gene, partial sequence	616	616	78%	2,00E-172	89%	DQ017557.1	702,87	86,4%
Select seq gb|FJ593401.1|	Mentha sp. JSZ-2009a internal transcribed spacer 1, partial sequence; 5.8S ribosomal RNA gene, complete sequence; and internal transcribed spacer 2, partial sequence	599	599	76%	2,00E-167	89%	FJ593401.1	701,46	86,2%
Select seq gb|DQ667333.1|	Acanthomintha lanceolata isolate x545 18S ribosomal RNA gene, partial sequence; internal transcribed spacer 1, 5.8S ribosomal RNA gene, and internal transcribed spacer 2, complete sequence; and 28S ribosomal RNA gene, partial sequence	614	614	78%	9,00E-172	89%	DQ667333.1	700,59	86,1%
Select seq gb|DQ017579.1|	Minthostachys acris voucher Schmidt-Lebuhn 564 (GOET) internal transcribed spacer 1, partial sequence; 5.8S ribosomal RNA gene and internal transcribed spacer 2, complete sequence; and 28S ribosomal RNA gene, partial sequence	614	614	78%	9,00E-172	89%	DQ017579.1	700,59	86,1%
Select seq gb|DQ017561.1|	Clinopodium sericeum voucher Schmidt-Lebuhn 465 (GOET) internal transcribed spacer 1, partial sequence; 5.8S ribosomal RNA gene and internal transcribed spacer 2, complete sequence; and 28S ribosomal RNA gene, partial sequence	614	614	78%	9,00E-172	89%	DQ017561.1	700,59	86,1%
Select seq gb|KF735672.1|	Mentha aquatica strain NH 18S ribosomal RNA gene, partial sequence; internal transcribed spacer 1 and 5.8S ribosomal RNA gene, complete sequence; and internal transcribed spacer 2, partial sequence	606	606	77%	1,00E-169	89%	KF735672.1	700,44	86,1%
Select seq gb|JQ669115.1|	Mentha arvensis voucher B. Drew 82 18S ribosomal RNA gene, internal transcribed spacer 1, 5.8S ribosomal RNA gene, internal transcribed spacer 2, and 26S ribosomal RNA gene, region	606	606	77%	1,00E-169	89%	JQ669115.1	700,44	86,1%
Select seq gb|DQ017571.1|	Minthostachys sp. Hart 1274 internal transcribed spacer 1, partial sequence; 5.8S ribosomal RNA gene and internal transcribed spacer 2, complete sequence; and 28S ribosomal RNA gene, partial sequence	612	612	78%	3,00E-171	89%	DQ017571.1	698,31	85,8%
Select seq gb|JQ669133.1|	Rhabdocaulon strictum voucher Singhurst s.n 18S ribosomal RNA gene, internal transcribed spacer 1, 5.8S ribosomal RNA gene, internal transcribed spacer 2, and 26S ribosomal RNA gene, region	603	603	77%	2,00E-168	89%	JQ669133.1	696,97	85,7%
Select seq gb|JQ669083.1|	Clinopodium macrostemum voucher B. Drew 147 18S ribosomal RNA gene, internal transcribed spacer 1, 5.8S ribosomal RNA gene, internal transcribed spacer 2, and 26S ribosomal RNA gene, region	603	603	77%	2,00E-168	89%	JQ669083.1	696,97	85,7%
Select seq gb|DQ667311.1|	Poliomintha palmeri isolate x259 18S ribosomal RNA gene, partial sequence; internal transcribed spacer 1, 5.8S ribosomal RNA gene, and internal transcribed spacer 2, complete sequence; and 28S ribosomal RNA gene, partial sequence	610	610	78%	1,00E-170	89%	DQ667311.1	696,03	85,5%
Select seq gb|KC591662.1|	Acanthomintha ilicifolia voucher SDSU12198 internal transcribed spacer 1, partial sequence; 5.8S ribosomal RNA gene and internal transcribed spacer 2, complete sequence; and 28S ribosomal RNA gene, partial sequence	608	608	78%	4,00E-170	89%	KC591662.1	693,74	85,3%
Select seq gb|AF369167.1|	Blephilia ciliata isolate BleCili1786 internal transcribed spacer 1, 5.8S ribosomal RNA gene, and internal transcribed spacer 2, complete sequence	592	592	76%	4,00E-165	89%	AF369167.1	693,26	85,2%
Select seq gb|DQ017574.1|	Minthostachys mollis voucher Schmidt-Lebuhn 410 (GOET) internal transcribed spacer 1, partial sequence; 5.8S ribosomal RNA gene and internal transcribed spacer 2, complete sequence; and 28S ribosomal RNA gene, partial sequence	612	612	78%	3,00E-171	88%	DQ017574.1	690,46	84,9%
Select seq gb|JQ669123.1|	Minthostachys mollis voucher B. Drew 349 18S ribosomal RNA gene, internal transcribed spacer 1, 5.8S ribosomal RNA gene, internal transcribed spacer 2, and 26S ribosomal RNA gene, region	603	603	77%	2,00E-168	88%	JQ669123.1	689,14	84,7%
Select seq gb|EU823287.1|	Satureja montana internal transcribed spacer 1, partial sequence; 5.8S ribosomal RNA gene, complete sequence; and internal transcribed spacer 2, partial sequence	688	688	89%	0.0	89%	EU823287.1	688,00	84,6%
Select seq gb|JQ669122.1|	Minthostachys mollis voucher B. Drew 345 18S ribosomal RNA gene, internal transcribed spacer 1, 5.8S ribosomal RNA gene, internal transcribed spacer 2, and 26S ribosomal RNA gene, region	601	601	77%	7,00E-168	88%	JQ669122.1	686,86	84,4%
Select seq gb|JQ669084.1|	Clinopodium taxifolium voucher B. Drew 228 18S ribosomal RNA gene, internal transcribed spacer 1, 5.8S ribosomal RNA gene, internal transcribed spacer 2, and 26S ribosomal RNA gene, region	601	601	77%	7,00E-168	88%	JQ669084.1	686,86	84,4%
Select seq gb|JN407476.1|	Mentha canadensis isolate shawpc0994I 18S ribosomal RNA gene, partial sequence; internal transcribed spacer 1, 5.8S ribosomal RNA gene, and internal transcribed spacer 2, complete sequence; and 28S ribosomal RNA gene, partial sequence	608	608	78%	4,00E-170	88%	JN407476.1	685,95	84,3%
Select seq gb|DQ017560.1|	Clinopodium speciosum voucher Schmidt-Lebuhn 503 (GOET) internal transcribed spacer 1, partial sequence; 5.8S ribosomal RNA gene and internal transcribed spacer 2, complete sequence; and 28S ribosomal RNA gene, partial sequence	608	608	78%	4,00E-170	88%	DQ017560.1	685,95	84,3%
Select seq gb|JQ669089.1|	Cuminia fernandezia voucher Stuessy et al., 11580 18S ribosomal RNA gene, internal transcribed spacer 1, 5.8S ribosomal RNA gene, internal transcribed spacer 2, and 26S ribosomal RNA gene, region	597	597	77%	9,00E-167	88%	JQ669089.1	682,29	83,8%
Select seq gb|DQ017565.1|	Clinopodium axillare voucher Wester 9 (GOET) internal transcribed spacer 1, partial sequence; 5.8S ribosomal RNA gene and internal transcribed spacer 2, complete sequence; and 28S ribosomal RNA gene, partial sequence	604	604	78%	5,00E-169	88%	DQ017565.1	681,44	83,7%
Select seq gb|DQ667305.1|	Cunila microcephala isolate x226 18S ribosomal RNA gene, partial sequence; internal transcribed spacer 1, 5.8S ribosomal RNA gene, and internal transcribed spacer 2, complete sequence; and 28S ribosomal RNA gene, partial sequence	603	603	78%	2,00E-168	88%	DQ667305.1	680,31	83,6%
Select seq gb|AF369166.1|	Hesperozygis spathulata isolate HesSpat1743 internal transcribed spacer 1, 5.8S ribosomal RNA gene, and internal transcribed spacer 2, complete sequence	586	586	76%	2,00E-163	88%	AF369166.1	678,53	83,4%
Select seq gb|DQ017564.1|	Clinopodium bolivianum voucher Schmidt-Lebuhn 729 (GOET) internal transcribed spacer 1, partial sequence; 5.8S ribosomal RNA gene and internal transcribed spacer 2, complete sequence; and 28S ribosomal RNA gene, partial sequence	601	601	78%	7,00E-168	88%	DQ017564.1	678,05	83,3%
Select seq gb|JQ669125.1|	Monardella villosa voucher B. Drew 66 18S ribosomal RNA gene, internal transcribed spacer 1, 5.8S ribosomal RNA gene, internal transcribed spacer 2, and 26S ribosomal RNA gene, region	592	592	77%	4,00E-165	88%	JQ669125.1	676,57	83,1%
Select seq gb|JQ669091.1|	Cunila microcephala voucher Sytsma 7247 18S ribosomal RNA gene, internal transcribed spacer 1, 5.8S ribosomal RNA gene, internal transcribed spacer 2, and 26S ribosomal RNA gene, region	592	592	77%	4,00E-165	88%	JQ669091.1	676,57	83,1%
Select seq gb|DQ667319.1|	Pycnanthemum virginianum isolate x313 18S ribosomal RNA gene, partial sequence; internal transcribed spacer 1, 5.8S ribosomal RNA gene, and internal transcribed spacer 2, complete sequence; and 28S ribosomal RNA gene, partial sequence	599	599	78%	2,00E-167	88%	DQ667319.1	675,79	83,1%

Select seq gb|DQ017566.1|	Clinopodium gilliesii voucher Hensen 2313 (LPB) internal transcribed spacer 1, partial sequence; 5.8S ribosomal RNA gene and internal transcribed spacer 2, complete sequence; and 28S ribosomal RNA gene, partial sequence	599	599	78%	2,00E-167	88%	DQ017566.1	675,79	83,1%
Select seq gb|KF735667.1|	Mentha aquatica strain HY1 18S ribosomal RNA gene, partial sequence; internal transcribed spacer 1 and 5.8S ribosomal RNA gene, complete sequence; and internal transcribed spacer 2, partial sequence	590	590	77%	1,00E-164	88%	KF735667.1	674,29	82,9%
Select seq gb|DQ667236.1|	Hedeoma costata isolate x067 18S ribosomal RNA gene, partial sequence; internal transcribed spacer 1, 5.8S ribosomal RNA gene, and internal transcribed spacer 2, complete sequence; and 28S ribosomal RNA gene, partial sequence	597	597	78%	9,00E-167	88%	DQ667236.1	673,54	82,8%
Select seq gb|DQ017563.1|	Clinopodium nubigenum voucher Schmidt-Lebuhn 376 (GOET) internal transcribed spacer 1, partial sequence; 5.8S ribosomal RNA gene and internal transcribed spacer 2, complete sequence; and 28S ribosomal RNA gene, partial sequence	597	597	78%	9,00E-167	88%	DQ017563.1	673,54	82,8%
Select seq gb|DQ017559.1|	Clinopodium tomentosum voucher Schmidt-Lebuhn 327 (GOET) internal transcribed spacer 1, partial sequence; 5.8S ribosomal RNA gene and internal transcribed spacer 2, complete sequence; and 28S ribosomal RNA gene, partial sequence	597	597	78%	9,00E-167	88%	DQ017559.1	673,54	82,8%
Select seq gb|JQ669137.1|	Thymbra capitata voucher UCBG 96.0817 s.n. 18S ribosomal RNA gene, internal transcribed spacer 1, 5.8S ribosomal RNA gene, internal transcribed spacer 2, and 26S ribosomal RNA gene, region	588	588	77%	5,00E-164	88%	JQ669137.1	672,00	82,6%
Select seq gb|DQ667318.1|	Monarda fistulosa isolate x312 18S ribosomal RNA gene, partial sequence; internal transcribed spacer 1, 5.8S ribosomal RNA gene, and internal transcribed spacer 2, complete sequence; and 28S ribosomal RNA gene, partial sequence	595	595	78%	3,00E-166	88%	DQ667318.1	671,28	82,5%
Select seq gb|DQ667242.1|	Thymus serpyllum isolate x075 18S ribosomal RNA gene, partial sequence; internal transcribed spacer 1, 5.8S ribosomal RNA gene, and internal transcribed spacer 2, complete sequence; and 28S ribosomal RNA gene, partial sequence	595	595	78%	3,00E-166	88%	DQ667242.1	671,28	82,5%
Select seq gb|KF735666.1|	Mentha aquatica strain HY 18S ribosomal RNA gene, partial sequence; internal transcribed spacer 1 and 5.8S ribosomal RNA gene, complete sequence; and internal transcribed spacer 2, partial sequence	586	586	77%	2,00E-163	88%	KF735666.1	669,71	82,3%
Select seq gb|JQ669118.1|	Clinopodium dalmaticum voucher M. Kintgen s.n. 18S ribosomal RNA gene, internal transcribed spacer 1, 5.8S ribosomal RNA gene, internal transcribed spacer 2, and 26S ribosomal RNA gene, region	586	586	77%	2,00E-163	88%	JQ669118.1	669,71	82,3%
Select seq gb|DQ667316.1|	Cunila incana isolate x296 18S ribosomal RNA gene, partial sequence; internal transcribed spacer 1, 5.8S ribosomal RNA gene, and internal transcribed spacer 2, complete sequence; and 28S ribosomal RNA gene, partial sequence	586	586	77%	2,00E-163	88%	DQ667316.1	669,71	82,3%
Select seq gb|FJ236468.1|	Thymus persicus isolate Tabriz 18S ribosomal RNA gene, partial sequence; internal transcribed spacer 1, 5.8S ribosomal RNA gene, and internal transcribed spacer 2, complete sequence; and 28S ribosomal RNA gene, partial sequence	593	593	78%	1,00E-165	88%	FJ236468.1	669,03	82,2%
Select seq gb|JF301343.1|	Hedeoma piperita voucher B. Drew 92 internal transcribed spacer 1, partial sequence; 5.8S ribosomal RNA gene and internal transcribed spacer 2, complete sequence; and 28S ribosomal RNA gene, partial sequence	592	592	78%	4,00E-165	88%	JF301343.1	667,90	82,1%
Select seq gb|DQ667237.1|	Clinopodium ashei isolate x068 18S ribosomal RNA gene, partial sequence; internal transcribed spacer 1, 5.8S ribosomal RNA gene, and internal transcribed spacer 2, complete sequence; and 28S ribosomal RNA gene, partial sequence	592	592	78%	4,00E-165	88%	DQ667237.1	667,90	82,1%
Select seq gb|DQ017562.1|	Clinopodium cylindristachys voucher Schmidt-Lebuhn 486 (GOET) internal transcribed spacer 1, partial sequence; 5.8S ribosomal RNA gene and internal transcribed spacer 2, complete sequence; and 28S ribosomal RNA gene, partial sequence	592	592	78%	4,00E-165	88%	DQ017562.1	667,90	82,1%
Select seq gb|JQ669077.1|	Blephilia hirsuta voucher T. Cochrane 13609 18S ribosomal RNA gene, internal transcribed spacer 1, 5.8S ribosomal RNA gene, internal transcribed spacer 2, and 26S ribosomal RNA gene, region	599	599	79%	2,00E-167	88%	JQ669077.1	667,24	82,0%
Select seq gb|GU381440.1|	Satureja pallaryi voucher TUB:Frey & Kuerschner 83-747 internal transcribed spacer 1, partial sequence; 5.8S ribosomal RNA gene, complete sequence; and internal transcribed spacer 2, partial sequence	702	702	93%	0.0	88%	GU381440.1	664,26	81,6%
Select seq gb|EU556524.1|	Thymus quinquecostatus isolate ZY internal transcribed spacer 1, partial sequence; 5.8S ribosomal RNA gene, complete sequence; and internal transcribed spacer 2, partial sequence	588	588	78%	5,00E-164	88%	EU556524.1	663,38	81,5%
Select seq gb|EU556522.1|	Thymus quinquecostatus isolate ZJ internal transcribed spacer 1, partial sequence; 5.8S ribosomal RNA gene, complete sequence; and internal transcribed spacer 2, partial sequence	588	588	78%	5,00E-164	88%	EU556522.1	663,38	81,5%
Select seq gb|EU556521.1|	Thymus mongolicus isolate XM internal transcribed spacer 1, partial sequence; 5.8S ribosomal RNA gene, complete sequence; and internal transcribed spacer 2, partial sequence	588	588	78%	5,00E-164	88%	EU556521.1	663,38	81,5%
Select seq gb|EU556519.1|	Thymus mongolicus isolate S2 internal transcribed spacer 1, partial sequence; 5.8S ribosomal RNA gene, complete sequence; and internal transcribed spacer 2, partial sequence	588	588	78%	5,00E-164	88%	EU556519.1	663,38	81,5%
Select seq gb|EU556518.1|	Thymus mongolicus isolate S1 internal transcribed spacer 1, partial sequence; 5.8S ribosomal RNA gene, complete sequence; and internal transcribed spacer 2, partial sequence	588	588	78%	5,00E-164	88%	EU556518.1	663,38	81,5%
Select seq gb|EU556517.1|	Thymus quinquecostatus isolate N2 internal transcribed spacer 1, partial sequence; 5.8S ribosomal RNA gene, complete sequence; and internal transcribed spacer 2, partial sequence	588	588	78%	5,00E-164	88%	EU556517.1	663,38	81,5%
Select seq gb|EU556516.1|	Thymus quinquecostatus isolate N1 internal transcribed spacer 1, partial sequence; 5.8S ribosomal RNA gene, complete sequence; and internal transcribed spacer 2, partial sequence	588	588	78%	5,00E-164	88%	EU556516.1	663,38	81,5%
Select seq gb|EU556511.1|	Thymus dahuricus isolate HD internal transcribed spacer 1, partial sequence; 5.8S ribosomal RNA gene, complete sequence; and internal transcribed spacer 2, partial sequence	588	588	78%	5,00E-164	88%	EU556511.1	663,38	81,5%
Select seq gb|EU556510.1|	Thymus mongolicus isolate G3 internal transcribed spacer 1, partial sequence; 5.8S ribosomal RNA gene, complete sequence; and internal transcribed spacer 2, partial sequence	588	588	78%	5,00E-164	88%	EU556510.1	663,38	81,5%
Select seq gb|EU556509.1|	Thymus mongolicus isolate G2 internal transcribed spacer 1, partial sequence; 5.8S ribosomal RNA gene, complete sequence; and internal transcribed spacer 2, partial sequence	588	588	78%	5,00E-164	88%	EU556509.1	663,38	81,5%
Select seq gb|EU556508.1|	Thymus mongolicus isolate G1 internal transcribed spacer 1, partial sequence; 5.8S ribosomal RNA gene, complete sequence; and internal transcribed spacer 2, partial sequence	588	588	78%	5,00E-164	88%	EU556508.1	663,38	81,5%

Select seq gb|EU556506.1|	Thymus quinquecostatus isolate D1 internal transcribed spacer 1, partial sequence; 5.8S ribosomal RNA gene, complete sequence; and internal transcribed spacer 2, partial sequence	588	588	78%	5,00E-164	88%	EU556506.1	663,38	81,5%
Select seq gb|JQ669090.1|	Cunila lythrifolia voucher Rzedowski 251 18S ribosomal RNA gene, internal transcribed spacer 1, 5.8S ribosomal RNA gene, internal transcribed spacer 2, and 26S ribosomal RNA gene, region	580	580	77%	9,00E-162	88%	JQ669090.1	662,86	81,5%
Select seq gb|EU556520.1|	Thymus quinquecostatus isolate XZ internal transcribed spacer 1, partial sequence; 5.8S ribosomal RNA gene, complete sequence; and internal transcribed spacer 2, partial sequence	586	586	78%	2,00E-163	88%	EU556520.1	661,13	81,2%
Select seq gb|EU823290.1|	Satureja cuneifolia internal transcribed spacer 1, partial sequence; 5.8S ribosomal RNA gene, complete sequence; and internal transcribed spacer 2, partial sequence	658	658	88%	0.0	88%	EU823290.1	658,00	80,9%
Select seq gb|GU381397.1|	Cyclotrichium niveum voucher B:McNeill 450 internal transcribed spacer 1, partial sequence; 5.8S ribosomal RNA gene, complete sequence; and internal transcribed spacer 2, partial sequence	612	612	83%	3,00E-171	87%	GU381397.1	641,49	78,8%
Select seq gb|GU381439.1|	Gontscharovia popovii voucher BM<GBR-LONDON>:Schmid 2419 internal transcribed spacer 1, partial sequence; 5.8S ribosomal RNA gene, complete sequence; and internal transcribed spacer 2, partial sequence	676	676	93%	0.0	87%	GU381439.1	632,39	77,7%
Select seq gb|GU381399.1|	Cyclotrichium origanifolium voucher M:Nydegger 46865 internal transcribed spacer 1, partial sequence; 5.8S ribosomal RNA gene, complete sequence; and internal transcribed spacer 2, partial sequence	595	595	83%	3,00E-166	87%	GU381399.1	623,67	76,6%
Select seq gb|AY506641.1|	Blephilia hirsuta internal transcribed spacer 1, partial sequence; 5.8S ribosomal RNA gene, complete sequence; and internal transcribed spacer 2, partial sequence	604	604	88%	5,00E-169	86%	AY506641.1	590,27	72,5%
Select seq gb|JQ669092.1|	Cunila origanoides voucher Stoots s.n. 18S ribosomal RNA gene, internal transcribed spacer 1, 5.8S ribosomal RNA gene, internal transcribed spacer 2, and 26S ribosomal RNA gene, region	601	601	88%	7,00E-168	86%	JQ669092.1	587,34	72,2%
Select seq gb|JQ669081.1|	Micromeria douglasii voucher B. Drew 206 18S ribosomal RNA gene, internal transcribed spacer 1, 5.8S ribosomal RNA gene, internal transcribed spacer 2, and 26S ribosomal RNA gene, region	588	588	87%	5,00E-164	86%	JQ669081.1	581,24	71,4%
Select seq gb|KC473228.1|	Mentha canadensis isolate 511190201 18S ribosomal RNA gene, partial sequence; internal transcribed spacer 1, 5.8S ribosomal RNA gene, and internal transcribed spacer 2, complete sequence; and 28S ribosomal RNA gene, partial sequence	599	599	89%	2,00E-167	86%	KC473228.1	578,81	71,1%
Select seq gb|KR150165.1|	Thymus sp. Kh8 internal transcribed spacer 1, partial sequence; 5.8S ribosomal RNA gene gene, complete sequence; and internal transcribed spacer 2 region, partial sequence	595	595	89%	3,00E-166	85%	KR150165.1	568,26	69,8%
Select seq gb|KR150146.1|	Thymus sp. Kh57 internal transcribed spacer 1, partial sequence; 5.8S ribosomal RNA gene gene, complete sequence; and internal transcribed spacer 2 region, partial sequence	595	595	89%	3,00E-166	85%	KR150146.1	568,26	69,8%
Select seq gb|EU735059.1|	Thymus trautvetteri 18S ribosomal RNA gene, partial sequence; internal transcribed spacer 1, 5.8S ribosomal RNA gene, and internal transcribed spacer 2, complete sequence; and 28S ribosomal RNA gene, partial sequence	595	595	89%	3,00E-166	85%	EU735059.1	568,26	69,8%
Select seq gb|EU735058.1|	Thymus persicus isolate Ardabil 18S ribosomal RNA gene, partial sequence; internal transcribed spacer 1, 5.8S ribosomal RNA gene, and internal transcribed spacer 2, complete sequence; and 28S ribosomal RNA gene, partial sequence	595	595	89%	3,00E-166	85%	EU735058.1	568,26	69,8%
Select seq gb|DQ017558.1|	Clinopodium fasciculatum voucher Schmidt-Lebuhn 325 (GOET) internal transcribed spacer 1, partial sequence; 5.8S ribosomal RNA gene and internal transcribed spacer 2, complete sequence; and 28S ribosomal RNA gene, partial sequence	593	593	89%	1,00E-165	85%	DQ017558.1	566,35	69,6%
								0,00	0,0%

Select for downloading or viewing reports	Kh004_trnL Description	Max score	Total score	Query cover	E value	Ident	Accession	(Ident/Cover) *Max score	Deviation from top hit
Select seq gb|GU381619.1|	Satureja mutica voucher M:Akhani 12362 tRNA-Leu (trnL) gene and trnL-trnF intergenic spacer, partial sequence; chloroplast	1195	1195	98%	0.0	100%	GU381619.1	1219,39	100,0%
Select seq gb|JQ669067.1|	Satureja montana voucher UCBG 2002.0593, Forbes s.n tRNA-Leu (trnL) gene and trnL-trnF intergenic spacer, partial sequence; plastid	1184	1184	97%	0.0	99%	JQ669067.1	1208,41	99,1%
Select seq gb|GU381621.1|	Satureja cuneifolia voucher M:Rechinger 11142 tRNA-Leu (trnL) gene and trnL-trnF intergenic spacer, partial sequence; chloroplast	1190	1190	98%	0.0	99%	GU381621.1	1202,14	98,6%
Select seq gb|GU381615.1|	Satureja innota voucher M:Barra et al. 2484GL tRNA-Leu (trnL) gene and trnL-trnF intergenic spacer, partial sequence; chloroplast	1190	1190	98%	0.0	99%	GU381615.1	1202,14	98,6%
Select seq gb|GU381612.1|	Gontscharovia popovii voucher BM<GBR-LONDON>:Schmid 2419 tRNA-Leu (trnL) gene and trnL-trnF intergenic spacer, partial sequence; chloroplast	1190	1190	98%	0.0	99%	GU381612.1	1202,14	98,6%
Select seq gb|AY840179.1|	Satureja montana tRNA-Leu (trnL) gene and trnL-trnF intergenic spacer, partial sequence; chloroplast	1190	1190	98%	0.0	99%	AY840179.1	1202,14	98,6%
Select seq gb|KR063656.1|	Satureja pilosa subsp. origanita tRNA-Leu (trnL) gene, partial sequence; trnL-trnF intergenic spacer, complete sequence; and tRNA-Phe (trnF) gene, partial sequence; chloroplast	1212	1212	100%	0.0	99%	KR063656.1	1199,88	98,4%
Select seq gb|JQ669068.1|	Satureja thymbra voucher UCBG 2002.0540, Forbes s.n. tRNA-Leu (trnL) gene and trnL-trnF intergenic spacer, partial sequence; plastid	1173	1173	97%	0.0	99%	JQ669068.1	1197,19	98,2%
Select seq gb|GU381614.1|	Satureja thymbra voucher M:Braeuchler 2896 tRNA-Leu (trnL) gene and trnL-trnF intergenic spacer, partial sequence; chloroplast	1184	1184	98%	0.0	99%	GU381614.1	1196,08	98,1%
Select seq gb|GU381611.1|	Gontscharovia popovii voucher M:Vvedensky s.n. tRNA-Leu (trnL) gene and trnL-trnF intergenic spacer, partial sequence; chloroplast	1184	1184	98%	0.0	99%	GU381611.1	1196,08	98,1%
Select seq gb|AY506611.1|	Satureja hortensis tRNA-Leu (trnL) gene and trnL-trnF intergenic spacer, partial sequence; chloroplast	1142	1142	95%	0.0	99%	AY506611.1	1190,08	97,6%
Select seq gb|GU381623.1|	Satureja spicigera voucher M:Kartli et al. 2726 tRNA-Leu (trnL) gene and trnL-trnF intergenic spacer, partial sequence; chloroplast	1173	1173	98%	0.0	99%	GU381623.1	1184,97	97,2%
Select seq gb|GU381620.1|	Satureja hortensis voucher M:Braeuchler 2422 tRNA-Leu (trnL) gene and trnL-trnF intergenic spacer, partial sequence; chloroplast	1166	1166	98%	0.0	99%	GU381620.1	1177,90	96,6%
Select seq gb|GU381622.1|	Satureja intermedia voucher M:Rechinger 43441 tRNA-Leu (trnL) gene and trnL-trnF intergenic spacer, partial sequence; chloroplast	1162	1162	98%	0.0	99%	GU381622.1	1173,86	96,3%
Select seq gb|GU381629.1|	Thymbra capitata voucher M:Braeuchler 2518 tRNA-Leu (trnL) gene and trnL-trnF intergenic spacer, partial sequence; chloroplast	1114	1114	98%	0.0	98%	GU381629.1	1114,00	91,4%
Select seq gb|GU381484.1|	Micromeria flagellaris voucher E:van der Werff & McPherson 13570 tRNA-Leu (trnL) gene and trnL-trnF intergenic spacer, partial sequence; chloroplast	1114	1114	98%	0.0	98%	GU381484.1	1114,00	91,4%
Select seq gb|GU381483.1|	Micromeria flagellaris voucher E:Clement et al 2140 tRNA-Leu (trnL) gene and trnL-trnF intergenic spacer, partial sequence; chloroplast	1114	1114	98%	0.0	98%	GU381483.1	1114,00	91,4%
Select seq gb|JF301401.1|	Thymbra capitata voucher UCBG 96.0817 tRNA-Leu (trnL) gene and trnL-trnF intergenic spacer, partial sequence; chloroplast	1088	1088	96%	0.0	98%	JF301401.1	1110,67	91,1%
Select seq gb|AY840207.1|	Thymbra spicata tRNA-Leu (trnL) gene and trnL-trnF intergenic spacer, partial sequence; chloroplast	1110	1110	98%	0.0	98%	AY840207.1	1110,00	91,0%
Select seq gb|GU381632.1|	Thymbra spicata voucher M:Braeuchler 4548 tRNA-Leu (trnL) gene and trnL-trnF intergenic spacer, partial sequence; chloroplast	1105	1105	98%	0.0	98%	GU381632.1	1105,00	90,6%
Select seq gb|GU381627.1|	Thymbra sintenisii subsp. isaurica voucher E:Goener 12628 tRNA-Leu (trnL) gene and trnL-trnF intergenic spacer, partial sequence; chloroplast	1103	1103	98%	0.0	98%	GU381627.1	1103,00	90,5%
Select seq gb|GU381517.1|	Clinopodium barosmum voucher BM<GBR-LONDON>:McLaren N193 tRNA-Leu (trnL) gene and trnL-trnF intergenic spacer, partial sequence; chloroplast	1098	1098	98%	0.0	98%	GU381517.1	1098,00	90,0%
Select seq gb|GU381516.1|	Clinopodium wardii voucher BM<GBR-LONDON>:Ludlow et al. 14234 tRNA-Leu (trnL) gene and trnL-trnF intergenic spacer, partial sequence; chloroplast	1098	1098	98%	0.0	98%	GU381516.1	1098,00	90,0%
Select seq gb|GU381515.1|	Clinopodium hydaspidis voucher BM<GBR-LONDON>:Mohd 133 tRNA-Leu (trnL) gene and trnL-trnF intergenic spacer, partial sequence; chloroplast	1098	1098	98%	0.0	98%	GU381515.1	1098,00	90,0%
Select seq gb|GU381514.1|	Clinopodium nepalense voucher FR:Stainton 6024 tRNA-Leu (trnL) gene and trnL-trnF intergenic spacer, partial sequence; chloroplast	1098	1098	98%	0.0	98%	GU381514.1	1098,00	90,0%
Select seq gb|GU381511.1|	Clinopodium piperitum voucher BM<GBR-LONDON>:Vickery 454 tRNA-Leu (trnL) gene and trnL-trnF intergenic spacer, partial sequence; chloroplast	1098	1098	98%	0.0	98%	GU381511.1	1098,00	90,0%

Select seq gb|GU381513.1|	Clinopodium nepalense voucher BM<GBR-LONDON>:Mikage et al. 9550294 tRNA-Leu (trnL) gene and trnL-trnF intergenic spacer, partial sequence; chloroplast	1070	1070	95%	0.0	97%	GU381513.1	1092,53	89,6%
Select seq gb|GU381495.1|	Killickia pilosa voucher M:Braeuchler 3810 tRNA-Leu (trnL) gene and trnL-trnF intergenic spacer, partial sequence; chloroplast	1074	1074	96%	0.0	97%	GU381495.1	1085,19	89,0%
Select seq gb|GU381638.1|	Thymus haussknechtii voucher M:Nydegger 43838 tRNA-Leu (trnL) gene and trnL-trnF intergenic spacer, partial sequence; chloroplast	1059	1059	95%	0.0	97%	GU381638.1	1081,29	88,7%
Select seq gb|KR150193.1|	Mentha sp. 2 Kh53 trnL-trnF intergenic spacer region, partial sequence; chloroplast	1070	1070	96%	0.0	97%	KR150193.1	1081,15	88,7%
Select seq gb|GU381512.1|	Clinopodium piperitum voucher E:Stainton 7320 tRNA-Leu (trnL) gene and trnL-trnF intergenic spacer, partial sequence; chloroplast	1092	1092	98%	0.0	97%	GU381512.1	1080,86	88,6%
Select seq gb|GU381496.1|	Killickia pilosa voucher M:Braeuchler 3832 tRNA-Leu (trnL) gene and trnL-trnF intergenic spacer, partial sequence; chloroplast	1088	1088	98%	0.0	97%	GU381496.1	1076,90	88,3%
Select seq gb|GU381493.1|	Killickia grandiflora voucher M:Braeuchler 3811 tRNA-Leu (trnL) gene and trnL-trnF intergenic spacer, partial sequence; chloroplast	1088	1088	98%	0.0	97%	GU381493.1	1076,90	88,3%
Select seq gb|KR150222.1|	Thymus sp. 3 Kh65 trnL-trnF intergenic spacer region, partial sequence; chloroplast	1099	1099	99%	0.0	97%	KR150222.1	1076,80	88,3%
Select seq gb|KR150225.1|	Thymus sp. 4 Kh24 trnL-trnF intergenic spacer region, partial sequence; chloroplast	1098	1098	99%	0.0	97%	KR150225.1	1075,82	88,2%
Select seq gb|KR150215.1|	Thymus sp. 1 Kh08 trnL-trnF intergenic spacer region, partial sequence; chloroplast	1098	1098	99%	0.0	97%	KR150215.1	1075,82	88,2%
Select seq gb|KR150218.1|	Thymus sp. 2 Kh69 trnL-trnF intergenic spacer region, partial sequence; chloroplast	1109	1109	100%	0.0	97%	KR150218.1	1075,73	88,2%
Select seq gb|JQ669069.1|	Thymus pulegioides voucher Riina 1577 tRNA-Leu (trnL) gene and trnL-trnF intergenic spacer, partial sequence; plastid	1075	1075	97%	0.0	97%	JQ669069.1	1075,00	88,2%
Select seq gb|GU381633.1|	Thymus caespititius voucher M:Heubl s.n. tRNA-Leu (trnL) gene and trnL-trnF intergenic spacer, partial sequence; chloroplast	1086	1086	98%	0.0	97%	GU381633.1	1074,92	88,2%
Select seq gb|GU381485.1|	Micromeria sphaerophylla voucher E:Lewis et al 1064 tRNA-Leu (trnL) gene and trnL-trnF intergenic spacer, partial sequence; chloroplast	1086	1086	98%	0.0	97%	GU381485.1	1074,92	88,2%
Select seq gb|GU381481.1|	Micromeria cf. madagascariensis Morawetz 205 tRNA-Leu (trnL) gene and trnL-trnF intergenic spacer, partial sequence; chloroplast	1086	1086	98%	0.0	97%	GU381481.1	1074,92	88,2%
Select seq gb|KR150198.1|	Ziziphora sp. 3 Kh112 trnL-trnF intergenic spacer region, partial sequence; chloroplast	1096	1096	99%	0.0	97%	KR150198.1	1073,86	88,1%
Select seq gb|GU381631.1|	Satureja linearifolia voucher M:Brullo & Furnari s.n. tRNA-Leu (trnL) gene and trnL-trnF intergenic spacer, partial sequence; chloroplast	1083	1083	98%	0.0	97%	GU381631.1	1071,95	87,9%
Select seq gb|GU381640.1|	Argantoniella salzmannii voucher M:Barra et al. 2673GL tRNA-Leu (trnL) gene and trnL-trnF intergenic spacer, partial sequence; chloroplast	1081	1081	98%	0.0	97%	GU381640.1	1069,97	87,7%
Select seq gb|GU381635.1|	Thymus broussonetii subsp. hannonis voucher MSB:Podlech 142 tRNA-Leu (trnL) gene and trnL-trnF intergenic spacer, partial sequence; chloroplast	1081	1081	98%	0.0	97%	GU381635.1	1069,97	87,7%
Select seq gb|AY840202.1|	Origanum vulgare tRNA-Leu (trnL) gene and trnL-trnF intergenic spacer, partial sequence; chloroplast	1081	1081	98%	0.0	97%	AY840202.1	1069,97	87,7%
Select seq gb|KR063657.1|	Thymus sibthorpii tRNA-Leu (trnL) gene, partial sequence; trnL-trnF intergenic spacer, complete sequence; and tRNA-Phe (trnF) gene, partial sequence; chloroplast	1103	1103	100%	0.0	97%	KR063657.1	1069,91	87,7%
Select seq gb|JX880022.1|	Origanum vulgare subsp. vulgare chloroplast, complete genome	1103	1103	100%	0.0	97%	JX880022.1	1069,91	87,7%
Select seq gb|JQ690290.1|	Origanum elongatum isolate H5_O_elo trnL-trnF intergenic spacer, partial sequence; chloroplast	1103	1103	100%	0.0	97%	JQ690290.1	1069,91	87,7%
Select seq gb|JQ690289.1|	Origanum rotundifolium isolate DNA3_O_rot trnL-trnF intergenic spacer, partial sequence; chloroplast	1103	1103	100%	0.0	97%	JQ690289.1	1069,91	87,7%
Select seq gb|EU556532.1|	Thymus amurensis isolate HX tRNA-Leu (trnL) gene and trnL-trnF intergenic spacer, partial sequence; chloroplast	1103	1103	100%	0.0	97%	EU556532.1	1069,91	87,7%
Select seq gb|EU556525.1|	Thymus quinquecostatus isolate CL tRNA-Leu (trnL) gene and trnL-trnF intergenic spacer, partial sequence; chloroplast	1103	1103	100%	0.0	97%	EU556525.1	1069,91	87,7%
Select seq emb|AJ505543.1|	Origanum vulgare plastid trnL-trnF intergenic spacer, specimen voucher cult., K-000-69-19317, chase 13334 (K)	1103	1103	100%	0.0	97%	AJ505543.1	1069,91	87,7%
Select seq gb|GU381628.1|	Thymbra calostachya voucher M:Ulrich s.n. tRNA-Leu (trnL) gene and trnL-trnF intergenic spacer, partial sequence; chloroplast	1079	1079	98%	0.0	97%	GU381628.1	1067,99	87,6%

Select seq gb|GU381489.1|	Killickia lutea voucher NU<ZAF>:Hilliard & Burtt 9876 tRNA-Leu (trnL) gene and trnL-trnF intergenic spacer, partial sequence; chloroplast	1077	1077	98% 0.0	97% GU381489.1	1066,01	87,4%
Select seq gb|GU381488.1|	Killickia compacta voucher M:Braeuchler 3816 tRNA-Leu (trnL) gene and trnL-trnF intergenic spacer, partial sequence; chloroplast	1077	1077	98% 0.0	97% GU381488.1	1066,01	87,4%
Select seq gb|EU556530.1|	Thymus mandschuricus isolate HM tRNA-Leu (trnL) gene and trnL-trnF intergenic spacer, partial sequence; chloroplast	1077	1077	98% 0.0	97% EU556530.1	1066,01	87,4%
Select seq gb|DQ667501.1|	Ziziphora taurica isolate x262 tRNA-Leu (trnL) gene and trnL-trnF intergenic spacer, partial sequence; chloroplast	1077	1077	98% 0.0	97% DQ667501.1	1066,01	87,4%
Select seq gb|EU556536.1|	Thymus marschallianus isolate XY tRNA-Leu (trnL) gene and trnL-trnF intergenic spacer, partial sequence; chloroplast	1066	1066	97% 0.0	97% EU556536.1	1066,00	87,4%
Select seq gb|KR150243.1|	Ziziphora sp. 2 Kh20 trnL-trnF intergenic spacer region, partial sequence; chloroplast	1098	1098	100% 0.0	97% KR150243.1	1065,06	87,3%
Select seq gb|EU556539.1|	Thymus quinquecostatus isolate ZY tRNA-Leu (trnL) gene and trnL-trnF intergenic spacer, partial sequence; chloroplast	1098	1098	100% 0.0	97% EU556539.1	1065,06	87,3%
Select seq gb|EU556538.1|	Thymus quinquecostatus isolate ZJ tRNA-Leu (trnL) gene and trnL-trnF intergenic spacer, partial sequence; chloroplast	1098	1098	100% 0.0	97% EU556538.1	1065,06	87,3%
Select seq gb|EU556533.1|	Thymus quinquecostatus isolate HY tRNA-Leu (trnL) gene and trnL-trnF intergenic spacer, partial sequence; chloroplast	1098	1098	100% 0.0	97% EU556533.1	1065,06	87,3%
Select seq gb|EU556527.1|	Thymus mongolicus isolate G2 tRNA-Leu (trnL) gene and trnL-trnF intergenic spacer, partial sequence; chloroplast	1098	1098	100% 0.0	97% EU556527.1	1065,06	87,3%
Select seq gb|EU556526.1|	Thymus quinquecostatus isolate D2 tRNA-Leu (trnL) gene and trnL-trnF intergenic spacer, partial sequence; chloroplast	1098	1098	100% 0.0	97% EU556526.1	1065,06	87,3%
Select seq gb|GU381637.1|	Thymus serpyllum voucher M:Braeuchler 2514 tRNA-Leu (trnL) gene and trnL-trnF intergenic spacer, partial sequence; chloroplast	1075	1075	98% 0.0	97% GU381637.1	1064,03	87,3%
Select seq gb|GU381510.1|	Ziziphora pamiroalaica voucher C:Murray et al. 10090 tRNA-Leu (trnL) gene and trnL-trnF intergenic spacer, partial sequence; chloroplast	1075	1075	98% 0.0	97% GU381510.1	1064,03	87,3%
Select seq gb|GU381507.1|	Ziziphora tenuior voucher MSB:Fayvush et al. 03-1503 tRNA-Leu (trnL) gene and trnL-trnF intergenic spacer, partial sequence; chloroplast	1075	1075	98% 0.0	97% GU381507.1	1064,03	87,3%
Select seq gb|GU381505.1|	Clinopodium troodi voucher W:Davis 1856 tRNA-Leu (trnL) gene and trnL-trnF intergenic spacer, partial sequence; chloroplast	1075	1075	98% 0.0	97% GU381505.1	1064,03	87,3%
Select seq gb|GU381501.1|	Clinopodium graveolens subsp. rotundifolium voucher M:Podlech 47181 tRNA-Leu (trnL) gene and trnL-trnF intergenic spacer, partial sequence; chloroplast	1075	1075	98% 0.0	97% GU381501.1	1064,03	87,3%
Select seq gb|GU381499.1|	Clinopodium suaveolens voucher M:Erben s.n. tRNA-Leu (trnL) gene and trnL-trnF intergenic spacer, partial sequence; chloroplast	1075	1075	98% 0.0	97% GU381499.1	1064,03	87,3%
Select seq gb|AY570463.1|	Origanum vulgare voucher JBW 2567 tRNA-Leu and trnL-trnF intergenic spacer, partial sequence; chloroplast	1075	1075	98% 0.0	97% AY570463.1	1064,03	87,3%
Select seq gb|JQ669021.1|	Clinopodium acinos voucher Judziewicz 14160 tRNA-Leu (trnL) gene and trnL-trnF intergenic spacer, partial sequence; plastid	1064	1064	97% 0.0	97% JQ669021.1	1064,00	87,3%
Select seq gb|GU381500.1|	Clinopodium nanum voucher M:Braeuchler 2796 tRNA-Leu (trnL) gene and trnL-trnF intergenic spacer, partial sequence; chloroplast	1062	1062	97% 0.0	97% GU381500.1	1062,00	87,1%
Select seq gb|GU381634.1|	Thymus pulegioides voucher M:Braeuchler 3129 tRNA-Leu (trnL) gene and trnL-trnF intergenic spacer, partial sequence; chloroplast	1072	1072	98% 0.0	97% GU381634.1	1061,06	87,0%
Select seq gb|KR150238.1|	Ziziphora sp. 1 Kh75 trnL-trnF intergenic spacer region, partial sequence; chloroplast	1092	1092	100% 0.0	97% KR150238.1	1059,24	86,9%
Select seq gb|JQ690293.1|	Origanum dayi isolate H43_O_day trnL-trnF intergenic spacer, partial sequence; chloroplast	1092	1092	100% 0.0	97% JQ690293.1	1059,24	86,9%
Select seq gb|EU556531.1|	Thymus amurensis isolate HW tRNA-Leu (trnL) gene and trnL-trnF intergenic spacer, partial sequence; chloroplast	1092	1092	100% 0.0	97% EU556531.1	1059,24	86,9%
Select seq gb|GU381636.1|	Thymus vulgaris voucher M:Braeuchler 3683 tRNA-Leu (trnL) gene and trnL-trnF intergenic spacer, partial sequence; chloroplast	1070	1070	98% 0.0	97% GU381636.1	1059,08	86,9%
Select seq gb|GU381497.1|	Clinopodium acinos voucher M:Podlech 50287 tRNA-Leu (trnL) gene and trnL-trnF intergenic spacer, partial sequence; chloroplast	1070	1070	98% 0.0	97% GU381497.1	1059,08	86,9%
Select seq gb|EU556528.1|	Thymus mongolicus isolate G3 tRNA-Leu (trnL) gene and trnL-trnF intergenic spacer, partial sequence; chloroplast	1070	1070	98% 0.0	97% EU556528.1	1059,08	86,9%
Select seq gb|AY618513.1|	Mentha arvensis tRNA-Leu (trnL) gene and trnL-trnF intergenic spacer, partial sequence; chloroplast	1057	1057	97% 0.0	97% AY618513.1	1057,00	86,7%

Select seq gb|FJ593456.1|	Mentha sp. JSZ-2009a tRNA-Leu (trnL) gene and trnL-trnF intergenic spacer, partial sequence; chloroplast	1086	1086	100%	0.0	97%	FJ593456.1	1053,42	86,4%
Select seq emb|AJ505544.1|	Thymus serpyllum var. citriodorum plastid trnL-trnF intergenic spacer, specimen voucher cult., K-1975-1177, Chase 13331 (K)	1086	1086	100%	0.0	97%	AJ505544.1	1053,42	86,4%
Select seq gb|DQ667492.1|	Lepechinia lancifolia isolate x232 tRNA-Leu (trnL) gene and trnL-trnF intergenic spacer, partial sequence; chloroplast	1079	1079	100%	0.0	97%	DQ667492.1	1046,63	85,8%
Select seq gb|EU556529.1|	Thymus dahuricus isolate HD tRNA-Leu (trnL) gene and trnL-trnF intergenic spacer, partial sequence; chloroplast	1061	1061	98%	0.0	96%	EU556529.1	1039,35	85,2%
Select seq gb|DQ667514.1|	Mentha arvensis isolate x424 tRNA-Leu (trnL) gene and trnL-trnF intergenic spacer, partial sequence; chloroplast	1079	1079	100%	0.0	96%	DQ667514.1	1035,84	84,9%
Select seq gb|GU381521.1|	Mentha pulegium voucher M:Braeuchler 2300 tRNA-Leu (trnL) gene and trnL-trnF intergenic spacer, partial sequence; chloroplast	1057	1057	98%	0.0	96%	GU381521.1	1035,43	84,9%
Select seq gb|GU381630.1|	Satureja thymbrifolia voucher M:Danin s.n. tRNA-Leu (trnL) gene and trnL-trnF intergenic spacer, partial sequence; chloroplast	1055	1055	98%	0.0	96%	GU381630.1	1033,47	84,8%
Select seq gb|GU381522.1|	Mentha cervina voucher M:Braeuchler 2394 tRNA-Leu (trnL) gene and trnL-trnF intergenic spacer, partial sequence; chloroplast	1055	1055	98%	0.0	96%	GU381522.1	1033,47	84,8%
Select seq gb|GU381613.1|	Satureja pallaryi voucher TUB:Frey & Kuerschner 83-747 tRNA-Leu (trnL) gene and trnL-trnF intergenic spacer, partial sequence; chloroplast	1066	1066	98%	0.0	95%	GU381613.1	1033,37	84,7%
Select seq gb|EU556537.1|	Thymus quinquecostatus isolate XZ tRNA-Leu (trnL) gene and trnL-trnF intergenic spacer, partial sequence; chloroplast	1072	1072	100%	0.0	96%	EU556537.1	1029,12	84,4%
Select seq gb|KR150233.1|	Thymus sp. 6 Kh133 trnL-trnF intergenic spacer region, partial sequence; chloroplast	1061	1061	99%	0.0	96%	KR150233.1	1028,85	84,4%
Select seq gb|KC414276.1|	Mentha canadensis isolate 511190001 tRNA-Leu (trnL) gene and trnL-trnF intergenic spacer, partial sequence; chloroplast	1070	1070	100%	0.0	96%	KC414276.1	1027,20	84,2%
Select seq emb|AJ505541.1|	Mentha suaveolens plastid trnL-trnF intergenic spacer, specimen voucher cult., K-1970-3169 (K)	1070	1070	100%	0.0	96%	AJ505541.1	1027,20	84,2%
Select seq gb|KR150223.1|	Mentha sp. 1 Kh62 trnL-trnF intergenic spacer region, partial sequence; chloroplast	1053	1053	99%	0.0	96%	KR150223.1	1021,09	83,7%
								0,00	0,0%
								0,00	0,0%
								0,00	0,0%

Select for downloading or viewing reports	Kh007_ITS Description	Max score	Total score	Query cover	E value	Ident	Accession	(Ident/Cover)* Max score	Deviation from top hit
Select seq gb|KF287973.1|	Echium wildpretii internal transcribed spacer 1, partial sequence; 5.8S ribosomal RNA gene, complete sequence; and internal transcribed spacer 2, partial sequence	641	641	73%	4,00E-180	92%	KF287973.1	807,84	100,0%
Select seq gb|EU048860.1|	Echium stenosiphon from Cape Verde: San Vicente 18S ribosomal RNA gene, partial sequence; and internal transcribed spacer 1, 5.8S ribosomal RNA gene, internal transcribed spacer 2, and 26S ribosomal RNA gene, complete sequence	776	776	92%	0.0	91%	EU048860.1	767,57	95,0%
Select seq gb|EU048851.1|	Echium simplex 18S ribosomal RNA gene, partial sequence; and internal transcribed spacer 1, 5.8S ribosomal RNA gene, internal transcribed spacer 2, and 26S ribosomal RNA gene, complete sequence	776	776	92%	0.0	91%	EU048851.1	767,57	95,0%
Select seq gb|KF287992.1|	Lobostemon fruticosus internal transcribed spacer 1, partial sequence; 5.8S ribosomal RNA gene, complete sequence; and internal transcribed spacer 2, partial sequence	632	632	75%	2,00E-177	91%	KF287992.1	766,83	94,9%
Select seq gb|EU048859.1|	Echium stenosiphon from Cape Verde: San Nicolau 18S ribosomal RNA gene, partial sequence; and internal transcribed spacer 1, 5.8S ribosomal RNA gene, internal transcribed spacer 2, and 26S ribosomal RNA gene, complete sequence	771	771	92%	0.0	91%	EU048859.1	762,62	94,4%
Select seq gb|EU048855.1|	Echium nervosum 18S ribosomal RNA gene, partial sequence; and internal transcribed spacer 1, 5.8S ribosomal RNA gene, internal transcribed spacer 2, and 26S ribosomal RNA gene, complete sequence	771	771	92%	0.0	91%	EU048855.1	762,62	94,4%
Select seq gb|EU048854.1|	Echium webbii 18S ribosomal RNA gene, partial sequence; and internal transcribed spacer 1, 5.8S ribosomal RNA gene, internal transcribed spacer 2, and 26S ribosomal RNA gene, complete sequence	771	771	92%	0.0	91%	EU048854.1	762,62	94,4%
Select seq gb|EU048848.1|	Echium hierrense 18S ribosomal RNA gene, partial sequence; and internal transcribed spacer 1, 5.8S ribosomal RNA gene, internal transcribed spacer 2, and 26S ribosomal RNA gene, complete sequence	771	771	92%	0.0	91%	EU048848.1	762,62	94,4%
Select seq gb|EU048853.1|	Echium acanthocarpum 18S ribosomal RNA gene, partial sequence; and internal transcribed spacer 1, 5.8S ribosomal RNA gene, internal transcribed spacer 2, and 26S ribosomal RNA gene, complete sequence	765	765	92%	0.0	91%	EU048853.1	756,68	93,7%
Select seq gb|EU048850.1|	Echium virescens 18S ribosomal RNA gene, partial sequence; and internal transcribed spacer 1, 5.8S ribosomal RNA gene, internal transcribed spacer 2, and 26S ribosomal RNA gene, complete sequence	765	765	92%	0.0	91%	EU048850.1	756,68	93,7%
Select seq gb|EU048849.1|	Echium aculeatum 18S ribosomal RNA gene, partial sequence; and internal transcribed spacer 1, 5.8S ribosomal RNA gene, internal transcribed spacer 2, and 26S ribosomal RNA gene, complete sequence	765	765	92%	0.0	91%	EU048849.1	756,68	93,7%
Select seq gb|EU048852.1|	Echium decaisnei 18S ribosomal RNA gene, partial sequence; and internal transcribed spacer 1, 5.8S ribosomal RNA gene, internal transcribed spacer 2, and 26S ribosomal RNA gene, complete sequence	750	750	91%	0.0	91%	EU048852.1	750,00	92,8%
Select seq gb|EU048857.1|	Echium vulcanorum 18S ribosomal RNA gene, partial sequence; and internal transcribed spacer 1, 5.8S ribosomal RNA gene, internal transcribed spacer 2, and 26S ribosomal RNA gene, complete sequence	749	749	92%	0.0	91%	EU048857.1	740,86	91,7%
Select seq gb|EU048847.1|	Echium lusitanicum 18S ribosomal RNA gene, partial sequence; internal transcribed spacer 1, 5.8S ribosomal RNA gene, and internal transcribed spacer 2, complete sequence; and 26S ribosomal RNA gene, partial sequence	739	739	91%	0.0	90%	EU048847.1	730,88	90,5%
Select seq gb|FJ763247.1|	Echium vulgare isolate 2063 internal transcribed spacer 1, partial sequence; 5.8S ribosomal RNA gene, complete sequence; and internal transcribed spacer 2, partial sequence	754	754	95%	0.0	90%	FJ763247.1	714,32	88,4%
Select seq gb|FJ763249.1|	Echium creticum isolate 2074 internal transcribed spacer 1, partial sequence; 5.8S ribosomal RNA gene, complete sequence; and internal transcribed spacer 2, partial sequence	750	750	95%	0.0	90%	FJ763249.1	710,53	88,0%
Select seq emb|AJ555896.1|	Echium vulgare 5.8S rRNA gene, ITS1 and ITS2	717	717	91%	0.0	90%	AJ555896.1	709,12	87,8%
Select seq gb|KP027097.1|	Echium rosulatum internal transcribed spacer 1, partial sequence; 5.8S ribosomal RNA gene, complete sequence; and internal transcribed spacer 2, partial sequence	706	706	90%	0.0	90%	KP027097.1	706,00	87,4%
Select seq gb|FJ789876.1|	Lobostemon trigonus internal transcribed spacer 1, partial sequence; 5.8S ribosomal RNA gene, complete sequence; and internal transcribed spacer 2, partial sequence	693	693	89%	0.0	90%	FJ789876.1	700,79	86,7%
Select seq gb|FJ789862.1|	Echium vulgare internal transcribed spacer 1, partial sequence; 5.8S ribosomal RNA gene, complete sequence; and internal transcribed spacer 2, partial sequence	699	699	90%	0.0	90%	FJ789862.1	699,00	86,5%
Select seq gb|AY092900.1|	Echium vulgare 18S ribosomal RNA gene, partial sequence; internal transcribed spacer 1, 5.8S ribosomal RNA gene and internal transcribed spacer 2, complete sequence; and 28S ribosomal RNA gene, partial sequence	712	712	92%	0.0	89%	AY092900.1	688,78	85,3%
Select seq gb|EU919589.1|	Huynhia pulchra voucher FI-HB 02.77 internal transcribed spacer 1, 5.8S ribosomal RNA gene, and internal transcribed spacer 2, complete sequence	599	599	78%	2,00E-167	89%	EU919589.1	683,47	84,6%
Select seq gb|EF199859.1|	Onosma paniculatum 18S ribosomal RNA gene, partial sequence; internal transcribed spacer 1, 5.8S ribosomal RNA gene, and internal transcribed spacer 2, complete sequence; and 28S ribosomal RNA gene, partial sequence	662	662	87%	0.0	89%	EF199859.1	677,22	83,8%
Select seq gb|GU827142.1|	Onosma viridis isolate DEV-15/7 internal transcribed spacer 1, partial sequence; 5.8S ribosomal RNA gene, complete sequence; and internal transcribed spacer 2, partial sequence	630	630	83%	8,00E-177	89%	GU827142.1	675,54	83,6%
Select seq gb|GU827137.1|	Onosma heterophylla isolate ALI4 internal transcribed spacer 1, partial sequence; 5.8S ribosomal RNA gene, complete sequence; and internal transcribed spacer 2, partial sequence	630	630	83%	8,00E-177	89%	GU827137.1	675,54	83,6%
Select seq gb|GU827133.1|	Onosma echioides subsp. angustifolia isolate ANG2 internal transcribed spacer 1, partial sequence; 5.8S ribosomal RNA gene, complete sequence; and internal transcribed spacer 2, partial sequence	630	630	83%	8,00E-177	89%	GU827133.1	675,54	83,6%
Select seq gb|GU827132.1|	Onosma echioides subsp. angustifolia isolate MOR13 internal transcribed spacer 1, partial sequence; 5.8S ribosomal RNA gene, complete sequence; and internal transcribed spacer 2, partial sequence	630	630	83%	8,00E-177	89%	GU827132.1	675,54	83,6%

Select seq gb|GU827147.1|	Onosma viridis isolate BAZ5 internal transcribed spacer 1, partial sequence; 5.8S ribosomal RNA gene, complete sequence; and internal transcribed spacer 2, partial sequence	628	628	83%	3,00E-176	89%	GU827147.1	673,40	83,4%
Select seq gb|GU827140.1|	Onosma heterophylla isolate BJL12 internal transcribed spacer 1, partial sequence; 5.8S ribosomal RNA gene, complete sequence; and internal transcribed spacer 2, partial sequence	628	628	83%	3,00E-176	89%	GU827140.1	673,40	83,4%
Select seq emb|FR718853.1|	Onosma mattirolii genomic DNA containing ITS1, 5.8S rRNA gene, ITS2, 28S rRNA gene, isolate Oma, specimen voucher Bartolini et al. 09.26	658	658	87%	0.0	89%	FR718853.1	673,13	83,3%
Select seq gb|GU827148.1|	Onosma viridis isolate VAR7 internal transcribed spacer 1, partial sequence; 5.8S ribosomal RNA gene, complete sequence; and internal transcribed spacer 2, partial sequence	627	627	83%	1,00E-175	89%	GU827148.1	672,33	83,2%
Select seq gb|GU827145.1|	Onosma viridis isolate SVI1 internal transcribed spacer 1, partial sequence; 5.8S ribosomal RNA gene, complete sequence; and internal transcribed spacer 2, partial sequence	627	627	83%	1,00E-175	89%	GU827145.1	672,33	83,2%
Select seq gb|GU827134.1|	Onosma echioides subsp. echioides isolate CAS3 internal transcribed spacer 1, partial sequence; 5.8S ribosomal RNA gene, complete sequence; and internal transcribed spacer 2, partial sequence	627	627	83%	1,00E-175	89%	GU827134.1	672,33	83,2%
Select seq gb|GU827139.1|	Onosma heterophylla isolate RLA7 internal transcribed spacer 1, partial sequence; 5.8S ribosomal RNA gene, complete sequence; and internal transcribed spacer 2, partial sequence	625	625	83%	4,00E-175	89%	GU827139.1	670,18	83,0%
Select seq gb|GU827135.1|	Onosma tornensis isolate TOR-T1 internal transcribed spacer 1, partial sequence; 5.8S ribosomal RNA gene, complete sequence; and internal transcribed spacer 2, partial sequence	625	625	83%	4,00E-175	89%	GU827135.1	670,18	83,0%
Select seq emb|FR718854.1|	Onosma paradoxa genomic DNA containing ITS1, 5.8S rRNA gene, ITS2, isolate Opa2, specimen voucher Strid & Papanicolau 15973	608	608	81%	4,00E-170	89%	FR718854.1	668,05	82,7%
Select seq gb|GU827131.1|	Onosma echioides subsp. echioides isolate ELP7 internal transcribed spacer 1, partial sequence; 5.8S ribosomal RNA gene, complete sequence; and internal transcribed spacer 2, partial sequence	623	623	83%	1,00E-174	89%	GU827131.1	668,04	82,7%
Select seq gb|GU827196.1|	Onosma echioides subsp. dalmatica isolate MON10 internal transcribed spacer 1, partial sequence; 5.8S ribosomal RNA gene, complete sequence; and internal transcribed spacer 2, partial sequence	621	621	83%	5,00E-174	89%	GU827196.1	665,89	82,4%
Select seq gb|GU827195.1|	Onosma echioides subsp. dalmatica isolate LOR1 internal transcribed spacer 1, partial sequence; 5.8S ribosomal RNA gene, complete sequence; and internal transcribed spacer 2, partial sequence	621	621	83%	5,00E-174	89%	GU827195.1	665,89	82,4%
Select seq gb|GU827189.1|	Onosma echioides subsp. dalmatica isolate OBR7 internal transcribed spacer 1, partial sequence; 5.8S ribosomal RNA gene, complete sequence; and internal transcribed spacer 2, partial sequence	621	621	83%	5,00E-174	89%	GU827189.1	665,89	82,4%
Select seq gb|GU827151.1|	Onosma taurica isolate HAG4 internal transcribed spacer 1, partial sequence; 5.8S ribosomal RNA gene, complete sequence; and internal transcribed spacer 2, partial sequence	619	619	83%	2,00E-173	89%	GU827151.1	663,75	82,2%
Select seq gb|GU827150.1|	Onosma taurica isolate HAG7 internal transcribed spacer 1, partial sequence; 5.8S ribosomal RNA gene, complete sequence; and internal transcribed spacer 2, partial sequence	619	619	83%	2,00E-173	89%	GU827150.1	663,75	82,2%
Select seq emb|FR718841.1|	Onosma bracteosa genomic DNA containing ITS1, 5.8S rRNA gene, ITS2, isolate Ono8, specimen voucher Cecchi & Selvi 10.69	603	603	81%	2,00E-168	89%	FR718841.1	662,56	82,0%
Select seq gb|EU919608.1|	Pontechium maculatum voucher FI-HB 06.07 internal transcribed spacer 1, 5.8S ribosomal RNA gene, and internal transcribed spacer 2, complete sequence	601	601	81%	6,00E-168	89%	EU919608.1	660,36	81,7%
Select seq gb|EU919584.1|	Echium arenarium internal transcribed spacer 1, 5.8S ribosomal RNA gene, and internal transcribed spacer 2, complete sequence	675	675	91%	0.0	89%	EU919584.1	660,16	81,7%
Select seq gb|GU827138.1|	Onosma heterophylla isolate KON1 internal transcribed spacer 1, partial sequence; 5.8S ribosomal RNA gene, complete sequence; and internal transcribed spacer 2, partial sequence	619	619	83%	2,00E-173	88%	GU827138.1	656,29	81,2%
Select seq gb|GU827191.1|	Onosma echioides subsp. echioides isolate GAB1 internal transcribed spacer 1, partial sequence; 5.8S ribosomal RNA gene, complete sequence; and internal transcribed spacer 2, partial sequence	617	617	83%	6,00E-173	88%	GU827191.1	654,17	81,0%
Select seq gb|GU827193.1|	Onosma echioides subsp. dalmatica isolate SIN10 internal transcribed spacer 1, partial sequence; 5.8S ribosomal RNA gene, complete sequence; and internal transcribed spacer 2, partial sequence	616	616	83%	2,00E-172	88%	GU827193.1	653,11	80,8%
Select seq gb|GU827182.1|	Onosma thracica isolate BIN2 internal transcribed spacer 1, partial sequence; 5.8S ribosomal RNA gene, complete sequence; and internal transcribed spacer 2, partial sequence	616	616	83%	2,00E-172	88%	GU827182.1	653,11	80,8%
Select seq gb|GU827179.1|	Onosma thracica isolate MAD1 internal transcribed spacer 1, partial sequence; 5.8S ribosomal RNA gene, complete sequence; and internal transcribed spacer 2, partial sequence	616	616	83%	2,00E-172	88%	GU827179.1	653,11	80,8%
Select seq gb|GU827197.1|	Onosma echioides subsp. dalmatica isolate MUC3 internal transcribed spacer 1, partial sequence; 5.8S ribosomal RNA gene, complete sequence; and internal transcribed spacer 2, partial sequence	610	610	83%	1,00E-170	88%	GU827197.1	646,75	80,1%
Select seq gb|GU827185.1|	Onosma echioides subsp. dalmatica isolate ZAV4 internal transcribed spacer 1, partial sequence; 5.8S ribosomal RNA gene, complete sequence; and internal transcribed spacer 2, partial sequence	610	610	83%	1,00E-170	88%	GU827185.1	646,75	80,1%
Select seq gb|GU827183.1|	Onosma echioides subsp. dalmatica isolate ZAV1 internal transcribed spacer 1, partial sequence; 5.8S ribosomal RNA gene, complete sequence; and internal transcribed spacer 2, partial sequence	610	610	83%	1,00E-170	88%	GU827183.1	646,75	80,1%
Select seq gb|GU827149.1|	Onosma malkarmayorum isolate ASE4 internal transcribed spacer 1, partial sequence; 5.8S ribosomal RNA gene, complete sequence; and internal transcribed spacer 2, partial sequence	610	610	83%	1,00E-170	88%	GU827149.1	646,75	80,1%
Select seq gb|FJ763198.1|	Neatostema apulum isolate 0639 internal transcribed spacer 1, partial sequence; 5.8S ribosomal RNA gene, complete sequence; and internal transcribed spacer 2, partial sequence	623	623	85%	1,00E-174	88%	FJ763198.1	644,99	79,8%

Select seq gb|EF199856.1|	Onosma waltonii isolate OWS7A 18S ribosomal RNA gene, partial sequence; internal transcribed spacer 1, 5.8S ribosomal RNA gene, and internal transcribed spacer 2, complete sequence; and 28S ribosomal RNA gene, partial sequence	636	636	87%	2,00E-178	88%	EF199856.1	643,31	79,6%
Select seq gb|EF199847.1|	Onosma hookerii 18S ribosomal RNA gene, partial sequence; internal transcribed spacer 1, 5.8S ribosomal RNA gene, and internal transcribed spacer 2, complete sequence; and 28S ribosomal RNA gene, partial sequence	636	636	87%	2,00E-178	88%	EF199847.1	643,31	79,6%
Select seq gb|GU827178.1|	Onosma thracica isolate KAV3 internal transcribed spacer 1, partial sequence; 5.8S ribosomal RNA gene, complete sequence; and internal transcribed spacer 2, partial sequence	606	606	83%	1,00E-169	88%	GU827178.1	642,51	79,5%
Select seq gb|GU827187.1|	Onosma rigida isolate KAL5 internal transcribed spacer 1, partial sequence; 5.8S ribosomal RNA gene, complete sequence; and internal transcribed spacer 2, partial sequence	604	604	83%	5,00E-169	88%	GU827187.1	640,39	79,3%
Select seq emb|FR718846.1|	Onosma erecta genomic DNA containing ITS1, 5.8S rRNA gene, ITS2, 28S rRNA gene, isolate Ono1, specimen voucher Bigazzi & Selvi 94.16	630	630	87%	8,00E-177	88%	FR718846.1	637,24	78,9%
Select seq gb|GU827181.1|	Onosma thracica isolate GAL2 internal transcribed spacer 1, partial sequence; 5.8S ribosomal RNA gene, complete sequence; and internal transcribed spacer 2, partial sequence	601	601	83%	6,00E-168	88%	GU827181.1	637,20	78,9%
Select seq gb|GU827180.1|	Onosma thracica isolate VLA10 internal transcribed spacer 1, partial sequence; 5.8S ribosomal RNA gene, complete sequence; and internal transcribed spacer 2, partial sequence	601	601	83%	6,00E-168	88%	GU827180.1	637,20	78,9%
Select seq gb|FJ763209.1|	Lithospermum viride isolate 1019 internal transcribed spacer 1, partial sequence; 5.8S ribosomal RNA gene, complete sequence; and internal transcribed spacer 2, partial sequence	614	614	85%	8,00E-172	88%	FJ763209.1	635,67	78,7%
Select seq gb|EF199857.1|	Onosma sp. YNH-2007 isolate OSPPS7A 18S ribosomal RNA gene, partial sequence; internal transcribed spacer 1, 5.8S ribosomal RNA gene, and internal transcribed spacer 2, complete sequence; and 28S ribosomal RNA gene, partial sequence	640	640	89%	1,00E-179	88%	EF199857.1	632,81	78,3%
Select seq gb|EF199846.1|	Onosma waltonii 18S ribosomal RNA gene, partial sequence; internal transcribed spacer 1, 5.8S ribosomal RNA gene, and internal transcribed spacer 2, complete sequence; and 28S ribosomal RNA gene, partial sequence	640	640	89%	1,00E-179	88%	EF199846.1	632,81	78,3%
Select seq gb|GU190212.1|	Lithospermum distichum voucher Keith Roe et al. 269 (NY) internal transcribed spacer 1, partial sequence; 5.8S ribosomal RNA gene, complete sequence; and internal transcribed spacer 2, partial sequence	603	603	85%	2,00E-168	88%	GU190212.1	624,28	77,3%
Select seq gb|FJ763210.1|	Lithospermum tuberosum isolate 1028 internal transcribed spacer 1, partial sequence; 5.8S ribosomal RNA gene, complete sequence; and internal transcribed spacer 2, partial sequence	603	603	85%	2,00E-168	88%	FJ763210.1	624,28	77,3%
Select seq gb|EF199858.1|	Onosma hookerii isolate OHS7A 18S ribosomal RNA gene, partial sequence; internal transcribed spacer 1, 5.8S ribosomal RNA gene, and internal transcribed spacer 2, complete sequence; and 28S ribosomal RNA gene, partial sequence	627	627	89%	1,00E-175	87%	EF199858.1	612,91	75,9%
Select seq gb|FJ763219.1|	Huynhia pulchra isolate 1085 internal transcribed spacer 1, partial sequence; 5.8S ribosomal RNA gene, complete sequence; and internal transcribed spacer 2, partial sequence	628	628	90%	3,00E-176	87%	FJ763219.1	607,07	75,1%
Select seq gb|EF199860.1|	Arnebia euchroma isolate AES7A 18S ribosomal RNA gene, partial sequence; internal transcribed spacer 1, 5.8S ribosomal RNA gene, and internal transcribed spacer 2, complete sequence; and 28S ribosomal RNA gene, partial sequence	665	665	97%	0.0	87%	EF199860.1	596,44	73,8%
Select seq gb|EF199861.1|	Lithospermum erythrorhizon isolate LES7A 18S ribosomal RNA gene, partial sequence; internal transcribed spacer 1, 5.8S ribosomal RNA gene, and internal transcribed spacer 2, complete sequence; and 28S ribosomal RNA gene, partial sequence	643	643	94%	1,00E-180	87%	EF199861.1	595,12	73,7%
Select seq gb|FJ763197.1|	Onosmodium virginianum isolate 0635 internal transcribed spacer 1, partial sequence; 5.8S ribosomal RNA gene, complete sequence; and internal transcribed spacer 2, partial sequence	628	628	92%	3,00E-176	87%	FJ763197.1	593,87	73,5%
Select seq gb|EF199848.1|	Arnebia euchroma 18S ribosomal RNA gene, partial sequence; internal transcribed spacer 1, 5.8S ribosomal RNA gene, and internal transcribed spacer 2, complete sequence; and 28S ribosomal RNA gene, partial sequence	669	669	99%	0.0	87%	EF199848.1	587,91	72,8%
Select seq gb|FJ763232.1|	Lithospermum longiflorum isolate 1596 internal transcribed spacer 1, partial sequence; 5.8S ribosomal RNA gene, complete sequence; and internal transcribed spacer 2, partial sequence	617	617	92%	6,00E-173	87%	FJ763232.1	583,47	72,2%
Select seq gb|GU827153.1|	Onosma stellulata isolate GRB1 internal transcribed spacer 1, partial sequence; 5.8S ribosomal RNA gene, complete sequence; and internal transcribed spacer 2, partial sequence	616	616	92%	2,00E-172	87%	GU827153.1	582,52	72,1%
Select seq gb|GU827152.1|	Onosma stellulata isolate GRB5 internal transcribed spacer 1, partial sequence; 5.8S ribosomal RNA gene, complete sequence; and internal transcribed spacer 2, partial sequence	616	616	92%	2,00E-172	87%	GU827152.1	582,52	72,1%
Select seq gb|FJ763220.1|	Ulugbekia tschimganica isolate 1088 internal transcribed spacer 1, partial sequence; 5.8S ribosomal RNA gene, complete sequence; and internal transcribed spacer 2, partial sequence	614	614	92%	8,00E-172	87%	FJ763220.1	580,63	71,9%
Select seq gb|FJ763206.1|	Lithospermum exsertum isolate 1001 internal transcribed spacer 1, partial sequence; 5.8S ribosomal RNA gene, complete sequence; and internal transcribed spacer 2, partial sequence	614	614	92%	8,00E-172	87%	FJ763206.1	580,63	71,9%
Select seq gb|JQ388515.1|	Myosotis krylovii voucher Rolfsmeier 1199 (KSC) internal transcribed spacer 1, partial sequence; 5.8S ribosomal RNA gene, complete sequence; and internal transcribed spacer 2, partial sequence	603	603	90%	2,00E-168	86%	JQ388515.1	576,20	71,3%
Select seq gb|FJ763211.1|	Lithospermum parksii isolate 1029 internal transcribed spacer 1, partial sequence; 5.8S ribosomal RNA gene, complete sequence; and internal transcribed spacer 2, partial sequence	612	612	92%	3,00E-171	86%	FJ763211.1	572,09	70,8%
Select seq gb|FJ763189.1|	Lithospermum officinale isolate 0409 internal transcribed spacer 1, partial sequence; 5.8S ribosomal RNA gene, complete sequence; and internal transcribed spacer 2, partial sequence	612	612	92%	3,00E-171	86%	FJ763189.1	572,09	70,8%
Select seq gb|GU190213.1|	Lithospermum mediale voucher B. & F. Oberwinkler 14257 (M) internal transcribed spacer 1, partial sequence; 5.8S ribosomal RNA gene, complete sequence; and internal transcribed spacer 2, partial sequence	606	606	92%	1,00E-169	86%	GU190213.1	566,48	70,1%
Select seq gb|FJ763221.1|	Lithospermum rosei isolate 1209 internal transcribed spacer 1, partial sequence; 5.8S ribosomal RNA gene, complete sequence; and internal transcribed spacer 2, partial sequence	601	601	92%	6,00E-168	86%	FJ763221.1	561,80	69,5%

Select seq gb|FJ763205.1|	Lithospermum rzedowskii isolate 1000 internal transcribed spacer 1, partial sequence; 5.8S ribosomal RNA gene, complete sequence; and internal transcribed spacer 2, partial sequence	601	601	92%	6,00E-168	86%	FJ763205.1	561,80	69,5%
Select seq gb|FJ763237.1|	Lithospermum papillosum isolate 1637 internal transcribed spacer 1, partial sequence; 5.8S ribosomal RNA gene, complete sequence; and internal transcribed spacer 2, partial sequence	599	599	92%	2,00E-167	86%	FJ763237.1	559,93	69,3%
Select seq gb|FJ763234.1|	Lithospermum onosmodium isolate 1599 internal transcribed spacer 1, partial sequence; 5.8S ribosomal RNA gene, complete sequence; and internal transcribed spacer 2, partial sequence	597	597	92%	8,00E-167	86%	FJ763234.1	558,07	69,1%
Select seq gb|FJ763225.1|	Lithospermum incisum isolate 1580 internal transcribed spacer 1, partial sequence; 5.8S ribosomal RNA gene, complete sequence; and internal transcribed spacer 2, partial sequence	597	597	92%	8,00E-167	86%	FJ763225.1	558,07	69,1%
Select seq gb|FJ763203.1|	Lithospermum molle isolate 0998 internal transcribed spacer 1, partial sequence; 5.8S ribosomal RNA gene, complete sequence; and internal transcribed spacer 2, partial sequence	597	597	92%	8,00E-167	86%	FJ763203.1	558,07	69,1%
Select seq gb|FJ763202.1|	Onosmodium occidentale isolate 0997 internal transcribed spacer 1, partial sequence; 5.8S ribosomal RNA gene, complete sequence; and internal transcribed spacer 2, partial sequence	597	597	92%	8,00E-167	86%	FJ763202.1	558,07	69,1%
Select seq gb|FJ763208.1|	Lithospermum californicum isolate 1018 internal transcribed spacer 1, partial sequence; 5.8S ribosomal RNA gene, complete sequence; and internal transcribed spacer 2, partial sequence	595	595	92%	3,00E-166	86%	FJ763208.1	556,20	68,9%
Select seq gb|KF849122.1|	Mertensia maritima voucher J.A.Churchill 7572013(RSA) internal transcribed spacer 1, partial sequence; 5.8S ribosomal RNA gene, complete sequence; and internal transcribed spacer 2, partial sequence	606	606	94%	1,00E-169	86%	KF849122.1	554,43	68,6%
Select seq gb|JQ388510.1|	Mertensia maritima voucher Kines s.n. (WS) internal transcribed spacer 1, partial sequence; 5.8S ribosomal RNA gene, complete sequence; and internal transcribed spacer 2, partial sequence	610	610	95%	1,00E-170	86%	JQ388510.1	552,21	68,4%
Select seq gb|JQ388512.1|	Mertensia paniculata var. borealis voucher Nazaire 1752 (WS) internal transcribed spacer 1, partial sequence; 5.8S ribosomal RNA gene, complete sequence; and internal transcribed spacer 2, partial sequence	599	599	95%	2,00E-167	85%	JQ388512.1	535,95	66,3%
Select seq gb|EF199863.1|	Arnebia szechenyi isolate ASS7A 18S ribosomal RNA gene, partial sequence; internal transcribed spacer 1, 5.8S ribosomal RNA gene, and internal transcribed spacer 2, complete sequence; and 28S ribosomal RNA gene, partial sequence	621	621	99%	5,00E-174	85%	EF199863.1	533,18	66,0%
Select seq gb|JQ388513.1|	Mertensia sibirica voucher Nazaire 1892 (WS) internal transcribed spacer 1, partial sequence; 5.8S ribosomal RNA gene, complete sequence; and internal transcribed spacer 2, partial sequence	593	593	95%	1,00E-165	85%	JQ388513.1	530,58	65,7%
Select seq gb|JQ388511.1|	Mertensia oblongifolia subsp. nevadensis voucher Nazaire 1748 (WS) internal transcribed spacer 1, partial sequence; 5.8S ribosomal RNA gene, complete sequence; and internal transcribed spacer 2, partial sequence	593	593	95%	1,00E-165	85%	JQ388511.1	530,58	65,7%
Select seq gb|JQ388509.1|	Mertensia davurica voucher Nazaire 1889 (WS) internal transcribed spacer 1, partial sequence; 5.8S ribosomal RNA gene, complete sequence; and internal transcribed spacer 2, partial sequence	593	593	95%	1,00E-165	85%	JQ388509.1	530,58	65,7%
Select seq gb|JQ388507.1|	Mertensia alpina voucher Nazaire 1810 (WS) internal transcribed spacer 1, partial sequence; 5.8S ribosomal RNA gene, complete sequence; and internal transcribed spacer 2, partial sequence	593	593	95%	1,00E-165	85%	JQ388507.1	530,58	65,7%
								0,00	0,0%

Select for downloading or viewing reports	Kh007_trnL Description	Max score	Total score	Query cover	E value	Ident	Accession	(Ident/Cover)* Max score	Deviation from top hit
Select seq gb|FJ763301.1|	Echium vulgare isolate 2063 tRNA-Leu (trnL) gene and trnL-trnF intergenic spacer, partial sequence; chloroplast	811	811	86%	0.0	92%	FJ763301.1	867,58	100,0%
Select seq gb|FJ763303.1|	Echium creticum isolate 2074 tRNA-Leu (trnL) gene and trnL-trnF intergenic spacer, partial sequence; chloroplast	808	808	86%	0.0	91%	FJ763303.1	854,98	98,5%
Select seq gb|FJ763294.1|	Arnebia decumbens isolate 1641 tRNA-Leu (trnL) gene and trnL-trnF intergenic spacer, partial sequence; chloroplast	806	806	86%	0.0	91%	FJ763294.1	852,86	98,3%
Select seq gb|KC542561.1|	Arnebia linearifolia isolate 2340 tRNA-Leu (trnL) gene, partial sequence; trnL-trnF intergenic spacer, complete sequence; and tRNA-Phe (trnF) gene, partial sequence; chloroplast	798	798	86%	0.0	91%	KC542561.1	844,40	97,3%
Select seq gb|JX976910.1|	Arnebia guttata tRNA-Leu (trnL) gene, partial sequence; trnL-trnF intergenic spacer, complete sequence; and tRNA-Phe (trnF) gene, partial sequence; chloroplast	798	798	86%	0.0	91%	JX976910.1	844,40	97,3%
Select seq gb|FJ763269.1|	Maharanga emodi isolate 1010 tRNA-Leu (trnL) gene and trnL-trnF intergenic spacer, partial sequence; chloroplast	791	791	86%	0.0	91%	FJ763269.1	836,99	96,5%
Select seq gb|KC542533.1|	Huynhia pulchra isolate 2082 tRNA-Leu (trnL) gene, partial sequence; trnL-trnF intergenic spacer, complete sequence; and tRNA-Phe (trnF) gene, partial sequence; chloroplast	787	787	86%	0.0	91%	KC542533.1	832,76	96,0%
Select seq gb|FJ763278.1|	Huynhia pulchra isolate 1085 tRNA-Leu (trnL) gene and trnL-trnF intergenic spacer, partial sequence; chloroplast	787	787	86%	0.0	91%	FJ763278.1	832,76	96,0%
Select seq gb|FJ788928.1|	Lithospermum tuberosum tRNA-Leu (trnL) gene, partial sequence; trnL-trnF intergenic spacer, complete sequence; and tRNA-Phe (trnF) gene, partial sequence; chloroplast	785	785	86%	0.0	91%	FJ788928.1	830,64	95,7%
Select seq gb|FJ763286.1|	Lithospermum canescens isolate 1592 tRNA-Leu (trnL) gene and trnL-trnF intergenic spacer, partial sequence; chloroplast	785	785	86%	0.0	91%	FJ763286.1	830,64	95,7%
Select seq gb|FJ763280.1|	Lithospermum rosei isolate 1206 tRNA-Leu (trnL) gene and trnL-trnF intergenic spacer, partial sequence; chloroplast	785	785	86%	0.0	91%	FJ763280.1	830,64	95,7%
Select seq emb|HG939437.1|	Buglossoides gastonii genomic DNA containing partial trnL gene, trnL-trnF IGS, specimen voucher FI-HB 07.58	693	693	76%	0.0	91%	HG939437.1	829,78	95,6%
Select seq emb|LN626707.1|	Arnebia purpurea genomic DNA containing partial trnL gene, trnL-trnF IGS, specimen voucher FI-HB 13.24	784	784	86%	0.0	91%	LN626707.1	829,58	95,6%
Select seq gb|FJ763277.1|	Glandora prostrata isolate 1083 tRNA-Leu (trnL) gene and trnL-trnF intergenic spacer, partial sequence; chloroplast	773	773	85%	0.0	91%	FJ763277.1	827,56	95,4%
Select seq gb|FJ788929.1|	Buglossoides gastonii tRNA-Leu (trnL) gene, partial sequence; trnL-trnF intergenic spacer, complete sequence; and tRNA-Phe (trnF) gene, partial sequence; chloroplast	782	782	86%	0.0	91%	FJ788929.1	827,47	95,4%
Select seq gb|FJ763305.1|	Buglossoides calabra isolate 2302 tRNA-Leu (trnL) gene and trnL-trnF intergenic spacer, partial sequence; chloroplast	782	782	86%	0.0	91%	FJ763305.1	827,47	95,4%
Select seq gb|FJ763297.1|	Lithospermum gayanum isolate 1684 tRNA-Leu (trnL) gene and trnL-trnF intergenic spacer, partial sequence; chloroplast	780	780	86%	0.0	91%	FJ763297.1	825,35	95,1%
Select seq gb|FJ763288.1|	Lithospermum carolinense isolate 1594 tRNA-Leu (trnL) gene and trnL-trnF intergenic spacer, partial sequence; chloroplast	780	780	86%	0.0	91%	FJ763288.1	825,35	95,1%
Select seq gb|FJ763276.1|	Lithospermum peruvianum isolate 1070 tRNA-Leu (trnL) gene and trnL-trnF intergenic spacer, partial sequence; chloroplast	780	780	86%	0.0	91%	FJ763276.1	825,35	95,1%
Select seq gb|FJ763275.1|	Lithospermum peruvianum isolate 1068 tRNA-Leu (trnL) gene and trnL-trnF intergenic spacer, partial sequence; chloroplast	780	780	86%	0.0	91%	FJ763275.1	825,35	95,1%
Select seq gb|FJ763273.1|	Lithospermum macbridei isolate 1032 tRNA-Leu (trnL) gene and trnL-trnF intergenic spacer, partial sequence; chloroplast	780	780	86%	0.0	91%	FJ763273.1	825,35	95,1%
Select seq emb|HG939444.1|	Arnebia decumbens genomic DNA containing partial trnL gene, trnL-trnF IGS, specimen voucher FI-HB 07.05	689	689	76%	0.0	91%	HG939444.1	824,99	95,1%
Select seq gb|KF288068.1|	Lithodora hispidula tRNA-Leu (trnL) gene and trnL-trnF intergenic spacer, partial sequence; chloroplast	769	769	85%	0.0	91%	KF288068.1	823,28	94,9%
Select seq gb|KC542597.1|	Cystostemon ethiopicus isolate 2731 tRNA-Leu (trnL) gene, partial sequence; trnL-trnF intergenic spacer, complete sequence; and tRNA-Phe (trnF) gene, partial sequence; chloroplast	778	778	86%	0.0	90%	KC542597.1	814,19	93,8%
Select seq gb|FJ763300.1|	Glandora diffusa isolate 1781 tRNA-Leu (trnL) gene and trnL-trnF intergenic spacer, partial sequence; chloroplast	767	767	85%	0.0	90%	FJ763300.1	812,12	93,6%
Select seq gb|KF288062.1|	Huynhia pulchra tRNA-Leu (trnL) gene and trnL-trnF intergenic spacer, partial sequence; chloroplast	776	776	86%	0.0	90%	KF288062.1	812,09	93,6%

Select seq gb|KF288032.1|	Arnebia benthamii tRNA-Leu (trnL) gene and trnL-trnF intergenic spacer, partial sequence; chloroplast	774	774	86%	0.0	90%	KF288032.1	810,00	93,4%
Select seq gb|FJ763256.1|	Buglossoides arvensis isolate 0473 tRNA-Leu (trnL) gene and trnL-trnF intergenic spacer, partial sequence; chloroplast	774	774	86%	0.0	90%	FJ763256.1	810,00	93,4%
Select seq gb|FJ763291.1|	Glandora rosmarinifolia isolate 1617 tRNA-Leu (trnL) gene and trnL-trnF intergenic spacer, partial sequence; chloroplast	763	763	85%	0.0	90%	FJ763291.1	807,88	93,1%
Select seq emb|HG939438.1|	Buglossoides zollingeri genomic DNA containing partial trnL gene, trnL-trnF IGS, specimen voucher HCT TFRI 8017	682	682	76%	0.0	90%	HG939438.1	807,63	93,1%
Select seq emb|LN626708.1|	Arnebia tubata genomic DNA containing partial trnL gene, trnL-trnF IGS, specimen voucher FI-HB 07.10	769	769	86%	0.0	90%	LN626708.1	804,77	92,8%
Select seq gb|KC542532.1|	Echium callithyrsum isolate 2076 tRNA-Leu (trnL) gene, partial sequence; trnL-trnF intergenic spacer, complete sequence; and tRNA-Phe (trnF) gene, partial sequence; chloroplast	760	760	85%	0.0	90%	KC542532.1	804,71	92,8%
Select seq gb|KF288051.1|	Cystostemon heliocharis tRNA-Leu (trnL) gene and trnL-trnF intergenic spacer, partial sequence; chloroplast	767	767	86%	0.0	90%	KF288051.1	802,67	92,5%
Select seq gb|KF288071.1|	Lithospermum distichum tRNA-Leu (trnL) gene and trnL-trnF intergenic spacer, partial sequence; chloroplast	730	730	82%	0.0	90%	KF288071.1	801,22	92,4%
Select seq gb|KF288037.1|	Buglossoides arvensis tRNA-Leu (trnL) gene and trnL-trnF intergenic spacer, partial sequence; chloroplast	763	763	86%	0.0	90%	KF288037.1	798,49	92,0%
Select seq gb|FJ763255.1|	Buglossoides incrassata isolate 0472 tRNA-Leu (trnL) gene and trnL-trnF intergenic spacer, partial sequence; chloroplast	761	761	86%	0.0	90%	FJ763255.1	796,40	91,8%
Select seq gb|FJ763284.1|	Lithospermum latifolium isolate 1582 tRNA-Leu (trnL) gene and trnL-trnF intergenic spacer, partial sequence; chloroplast	654	654	74%	0.0	90%	FJ763284.1	795,41	91,7%
Select seq gb|FJ763257.1|	Mairetis microsperma isolate 0512 tRNA-Leu (trnL) gene and trnL-trnF intergenic spacer, partial sequence; chloroplast	750	750	85%	0.0	90%	FJ763257.1	794,12	91,5%
Select seq gb|FJ763283.1|	Lithospermum incisum isolate 1581 tRNA-Leu (trnL) gene and trnL-trnF intergenic spacer, partial sequence; chloroplast	758	758	86%	0.0	90%	FJ763283.1	793,26	91,4%
Select seq gb|KF288055.1|	Pontechium maculatum tRNA-Leu (trnL) gene and trnL-trnF intergenic spacer, partial sequence; chloroplast	756	756	86%	0.0	90%	KF288055.1	791,16	91,2%
Select seq emb|HG939439.1|	Buglossoides arvensis subsp. arvensis genomic DNA containing partial trnL gene, trnL-trnF IGS, specimen voucher FI-HB 07.64	667	667	76%	0.0	90%	HG939439.1	789,87	91,0%
Select seq emb|HG939445.1|	Arnebia linearifolia genomic DNA containing partial trnL gene, trnL-trnF IGS, specimen voucher FI-HB 07.03	665	665	76%	0.0	90%	HG939445.1	787,50	90,8%
Select seq gb|KC542541.1|	Onosma tricerosperma isolate 2125 tRNA-Leu (trnL) gene, partial sequence; trnL-trnF intergenic spacer, complete sequence; and tRNA-Phe (trnF) gene, partial sequence; chloroplast	664	664	76%	0.0	90%	KC542541.1	786,32	90,6%
Select seq gb|FJ763260.1|	Onosma fruticosa isolate 0620 tRNA-Leu (trnL) gene and trnL-trnF intergenic spacer, partial sequence; chloroplast	664	664	76%	0.0	90%	FJ763260.1	786,32	90,6%
Select seq gb|FJ763259.1|	Onosma rhodopea isolate 0582 tRNA-Leu (trnL) gene and trnL-trnF intergenic spacer, partial sequence; chloroplast	664	664	76%	0.0	90%	FJ763259.1	786,32	90,6%
Select seq gb|KC542553.1|	Buglossoides incrassata isolate 2304 tRNA-Leu (trnL) gene, partial sequence; trnL-trnF intergenic spacer, complete sequence; and tRNA-Phe (trnF) gene, partial sequence; chloroplast	750	750	86%	0.0	90%	KC542553.1	784,88	90,5%
Select seq gb|FJ763299.1|	Glandora nitida isolate 1706 tRNA-Leu (trnL) gene and trnL-trnF intergenic spacer, partial sequence; chloroplast	741	741	85%	0.0	90%	FJ763299.1	784,59	90,4%
Select seq gb|KF288094.1|	Onosma stellulata tRNA-Leu (trnL) gene and trnL-trnF intergenic spacer, partial sequence; chloroplast	658	658	76%	0.0	90%	KF288094.1	779,21	89,8%
Select seq gb|FJ763293.1|	Lithospermum scabrum isolate 1638 tRNA-Leu (trnL) gene and trnL-trnF intergenic spacer, partial sequence; chloroplast	747	747	86%	0.0	89%	FJ763293.1	773,06	89,1%
Select seq gb|FJ763272.1|	Lithospermum parksii isolate 1029 tRNA-Leu (trnL) gene and trnL-trnF intergenic spacer, partial sequence; chloroplast	747	747	86%	0.0	89%	FJ763272.1	773,06	89,1%
Select seq gb|FJ763270.1|	Lithospermum californicum isolate 1018 tRNA-Leu (trnL) gene and trnL-trnF intergenic spacer, partial sequence; chloroplast	747	747	86%	0.0	89%	FJ763270.1	773,06	89,1%
Select seq emb|HG939442.1|	Buglossoides permixta genomic DNA containing partial trnL gene, trnL-trnF IGS, specimen voucher FI-HB 13.63	658	658	76%	0.0	89%	HG939442.1	770,55	88,8%
Select seq emb|HG939440.1|	Buglossoides incrassata subsp. splitgerberi genomic DNA containing partial trnL gene, trnL-trnF IGS, specimen voucher FI-HB 10.44	640	640	74%	2,00E-179	89%	HG939440.1	769,73	88,7%
Select seq gb|FJ763292.1|	Lithospermum papillosum isolate 1637 tRNA-Leu (trnL) gene and trnL-trnF intergenic spacer, partial sequence; chloroplast	741	741	86%	0.0	89%	FJ763292.1	766,85	88,4%

Select seq gb|FJ763282.1|	Lithospermum cobrense isolate 1576 tRNA-Leu (trnL) gene and trnL-trnF intergenic spacer, partial sequence; chloroplast	741	741	86% 0.0		89% FJ763282.1	766,85	88,4%
Select seq gb|FJ763279.1|	Ulugbekia tschimganica isolate 1088 tRNA-Leu (trnL) gene and trnL-trnF intergenic spacer, partial sequence; chloroplast	741	741	86% 0.0		89% FJ763279.1	766,85	88,4%
Select seq gb|FJ763274.1|	Lithospermum cinerascens isolate 1036 tRNA-Leu (trnL) gene and trnL-trnF intergenic spacer, partial sequence; chloroplast	741	741	86% 0.0		89% FJ763274.1	766,85	88,4%
Select seq gb|FJ763268.1|	Lithospermum rzedowskii isolate 1000 tRNA-Leu (trnL) gene and trnL-trnF intergenic spacer, partial sequence; chloroplast	741	741	86% 0.0		89% FJ763268.1	766,85	88,4%
Select seq gb|FJ763261.1|	Onosmodium virginianum isolate 0635 tRNA-Leu (trnL) gene and trnL-trnF intergenic spacer, partial sequence; chloroplast	741	741	86% 0.0		89% FJ763261.1	766,85	88,4%
Select seq gb|FJ763254.1|	Lithospermum officinale isolate 0409 tRNA-Leu (trnL) gene and trnL-trnF intergenic spacer, partial sequence; chloroplast	741	741	86% 0.0		89% FJ763254.1	766,85	88,4%
Select seq gb|FJ763281.1|	Cerinthe minor isolate 1521 tRNA-Leu (trnL) gene and trnL-trnF intergenic spacer, partial sequence; chloroplast	737	737	86% 0.0		89% FJ763281.1	762,71	87,9%
Select seq emb|HG939443.1|	Buglossoides goulandriorum genomic DNA containing partial trnL gene, trnL-trnF IGS, specimen voucher FI-HB 08.38	651	651	76% 0.0		89% HG939443.1	762,36	87,9%
Select seq gb|FJ763309.1|	Lithospermum erythrorhizon isolate 2368 tRNA-Leu (trnL) gene and trnL-trnF intergenic spacer, partial sequence; chloroplast	736	736	86% 0.0		89% FJ763309.1	761,67	87,8%
Select seq emb|HG939441.1|	Buglossoides minima genomic DNA containing partial trnL gene, trnL-trnF IGS, specimen voucher FI-HB 09.22	630	630	74%	9,00E-177	89% HG939441.1	757,70	87,3%
Select seq gb|FJ763308.1|	Buglossoides purpurocaerulea isolate 2351 tRNA-Leu (trnL) gene and trnL-trnF intergenic spacer, partial sequence; chloroplast	732	732	86% 0.0		89% FJ763308.1	757,53	87,3%
Select seq gb|FJ763267.1|	Lithospermum molle isolate 0998 tRNA-Leu (trnL) gene and trnL-trnF intergenic spacer, partial sequence; chloroplast	728	728	86% 0.0		89% FJ763267.1	753,40	86,8%
Select seq gb|KF288070.1|	Lithospermum bejariense tRNA-Leu (trnL) gene and trnL-trnF intergenic spacer, partial sequence; chloroplast	721	721	86% 0.0		89% KF288070.1	746,15	86,0%
Select seq gb|KF288073.1|	Lithospermum macromeria tRNA-Leu (trnL) gene and trnL-trnF intergenic spacer, partial sequence; chloroplast	719	719	86% 0.0		89% KF288073.1	744,08	85,8%
Select seq gb|KC542594.1|	Cystostemon hispidus isolate 2719 tRNA-Leu (trnL) gene, partial sequence; trnL-trnF intergenic spacer, complete sequence; and tRNA-Phe (trnF) gene, partial sequence; chloroplast	719	719	86% 0.0		89% KC542594.1	744,08	85,8%
Select seq gb|FJ763289.1|	Lithospermum macromeria isolate 1597 tRNA-Leu (trnL) gene and trnL-trnF intergenic spacer, partial sequence; chloroplast	719	719	86% 0.0		89% FJ763289.1	744,08	85,8%
Select seq gb|KF288077.1|	Lobostemon fruticosus tRNA-Leu (trnL) gene and trnL-trnF intergenic spacer, partial sequence; chloroplast	686	872	86% 0.0		92% KF288077.1	733,86	84,6%
Select seq gb|FJ763304.1|	Alkanna tinctoria isolate 2123 tRNA-Leu (trnL) gene and trnL-trnF intergenic spacer, partial sequence; chloroplast	708	708	86% 0.0		88% FJ763304.1	724,47	83,5%
Select seq gb|FJ763263.1|	Alkanna sieberi isolate 0643 tRNA-Leu (trnL) gene and trnL-trnF intergenic spacer, partial sequence; chloroplast	708	708	86% 0.0		88% FJ763263.1	724,47	83,5%
Select seq gb|FJ763298.1|	Cerinthe major isolate 1702 tRNA-Leu (trnL) gene and trnL-trnF intergenic spacer, partial sequence; chloroplast	704	704	86% 0.0		88% FJ763298.1	720,37	83,0%
Select seq gb|FJ763307.1|	Podonosma orientalis isolate 2309 tRNA-Leu (trnL) gene and trnL-trnF intergenic spacer, partial sequence; chloroplast	702	702	86% 0.0		88% FJ763307.1	718,33	82,8%
Select seq gb|KF288040.1|	Cerinthe major tRNA-Leu (trnL) gene and trnL-trnF intergenic spacer, partial sequence; chloroplast	697	697	86% 0.0		88% KF288040.1	713,21	82,2%
Select seq gb|FJ763262.1|	Neatostema apulum isolate 0639 tRNA-Leu (trnL) gene and trnL-trnF intergenic spacer, partial sequence; chloroplast	686	686	85% 0.0		88% FJ763262.1	710,21	81,9%
Select seq gb|KF849219.1|	Trichodesma calcaratum voucher T.Buira & J.Calvo JC0447(MA) tRNA-Leu (trnL) gene, partial sequence; trnL-trnF intergenic spacer, complete sequence; and tRNA-Phe (trnF) gene, partial sequence; plastid	665	665	86% 0.0		87% KF849219.1	672,73	77,5%
Select seq gb|KC542567.1|	Trichodesma calcaratum isolate 2362 tRNA-Leu (trnL) gene, partial sequence; trnL-trnF intergenic spacer, complete sequence; and tRNA-Phe (trnF) gene, partial sequence; chloroplast	665	665	86% 0.0		87% KC542567.1	672,73	77,5%
Select seq gb|KF849218.1|	Trichodesma calycosum voucher Y.-Y.Huang 234(MO) tRNA-Leu (trnL) gene, partial sequence; trnL-trnF intergenic spacer, complete sequence; and tRNA-Phe (trnF) gene, partial sequence; plastid	664	664	86% 0.0		87% KF849218.1	671,72	77,4%
Select seq gb|KC542569.1|	Trichodesma physaloides isolate 2364 tRNA-Leu (trnL) gene, partial sequence; trnL-trnF intergenic spacer, complete sequence; and tRNA-Phe (trnF) gene, partial sequence; chloroplast	660	660	86% 0.0		87% KC542569.1	667,67	77,0%
Select seq gb|KF849215.1|	Caccinia macranthera voucher Fayvush et al.05-0683790641(MA) tRNA-Leu (trnL) gene, partial sequence; trnL-trnF intergenic spacer, complete sequence; and tRNA-Phe (trnF) gene, partial sequence; plastid	656	656	86% 0.0		87% KF849215.1	663,63	76,5%

Select seq gb|KF288039.1|	Caccinia strigosa tRNA-Leu (trnL) gene and trnL-trnF intergenic spacer, partial sequence; chloroplast	656	656	86%	0.0	87%	KF288039.1	663,63	76,5%
Select seq gb|GQ285241.1|	Caccinia strigosa tRNA-Leu (trnL) gene, partial sequence; trnL-trnF intergenic spacer, complete sequence; and tRNA-Phe (trnF) gene, partial sequence; chloroplast	656	656	86%	0.0	87%	GQ285241.1	663,63	76,5%
Select seq gb|GQ285240.1|	Trichodesma zeylanicum tRNA-Leu (trnL) gene, partial sequence; trnL-trnF intergenic spacer, complete sequence; and tRNA-Phe (trnF) gene, partial sequence; chloroplast	654	654	86%	0.0	87%	GQ285240.1	661,60	76,3%
Select seq gb|KF849217.1|	Trichodesma zeylanicum voucher F.J.Badman 2084(MA) tRNA-Leu (trnL) gene, partial sequence; trnL-trnF intergenic spacer, complete sequence; and tRNA-Phe (trnF) gene, partial sequence; plastid	651	651	86%	0.0	87%	KF849217.1	658,57	75,9%
Select seq gb|GQ285255.1|	Moritzia lindenii tRNA-Leu (trnL) gene, partial sequence; trnL-trnF intergenic spacer, complete sequence; and tRNA-Phe (trnF) gene, partial sequence; chloroplast	647	647	86%	0.0	87%	GQ285255.1	654,52	75,4%
Select seq dbj|AB758347.1|	Trichodesma aucheri chloroplast gene for tRNA-Leu (trnL) and trnL-trnF intergenic spacer, partial and complete sequence, specimen_voucher: TARI:57195	641	641	86%	4,00E-180	86%	AB758347.1	641,00	73,9%
Select seq gb|GQ285260.1|	Thaumatocaryon tetraquetrum tRNA-Leu (trnL) gene, partial sequence; trnL-trnF intergenic spacer, complete sequence; and tRNA-Phe (trnF) gene, partial sequence; chloroplast	636	636	86%	2,00E-178	86%	GQ285260.1	636,00	73,3%
Select seq gb|GQ285249.1|	Trachystemon orientalis tRNA-Leu (trnL) gene, partial sequence; trnL-trnF intergenic spacer, complete sequence; and tRNA-Phe (trnF) gene, partial sequence; chloroplast	636	636	86%	2,00E-178	86%	GQ285249.1	636,00	73,3%
Select seq gb|KF416951.1|	Mertensia davurica isolate Mdav_R1205 tRNA-Leu (trnL) gene, partial sequence; trnL-trnF intergenic spacer, complete sequence; and tRNA-Phe (trnF) gene, partial sequence; chloroplast	627	627	85%	1,00E-175	86%	KF416951.1	634,38	73,1%
Select seq gb|KC542592.1|	Mertensia dahurica isolate 2695 tRNA-Leu (trnL) gene, partial sequence; trnL-trnF intergenic spacer, complete sequence; and tRNA-Phe (trnF) gene, partial sequence; chloroplast	625	625	85%	4,00E-175	86%	KC542592.1	632,35	72,9%
Select seq gb|FJ763310.1|	Echiochilon fruticosum isolate 2542 tRNA-Leu (trnL) gene and trnL-trnF intergenic spacer, partial sequence; chloroplast	632	632	86%	3,00E-177	86%	FJ763310.1	632,00	72,8%
Select seq gb|KF288113.1|	Trachystemon orientalis tRNA-Leu (trnL) gene and trnL-trnF intergenic spacer, partial sequence; chloroplast	630	630	86%	9,00E-177	86%	KF288113.1	630,00	72,6%
Select seq gb|KF417011.1|	Mertensia pratensis isolate Mpra_N1785 tRNA-Leu (trnL) gene, partial sequence; trnL-trnF intergenic spacer, complete sequence; and tRNA-Phe (trnF) gene, partial sequence; chloroplast	621	621	85%	6,00E-174	86%	KF417011.1	628,31	72,4%
Select seq gb|JQ388593.1|	Mertensia sibirica voucher Nazaire 1892 (WS) tRNA-Leu (trnL) gene, partial sequence; trnL-trnF intergenic spacer, complete sequence; and tRNA-Phe (trnF) gene, partial sequence; chloroplast	621	621	85%	6,00E-174	86%	JQ388593.1	628,31	72,4%
Select seq gb|KC542586.1|	Mertensia sibirica isolate 2622 tRNA-Leu (trnL) gene, partial sequence; trnL-trnF intergenic spacer, complete sequence; and tRNA-Phe (trnF) gene, partial sequence; chloroplast	619	619	85%	2,00E-173	86%	KC542586.1	626,28	72,2%
Select seq gb|JQ388589.1|	Mertensia davurica voucher Nazaire 1889 (WS) tRNA-Leu (trnL) gene, partial sequence; trnL-trnF intergenic spacer, complete sequence; and tRNA-Phe (trnF) gene, partial sequence; chloroplast	619	619	85%	2,00E-173	86%	JQ388589.1	626,28	72,2%
								0,00	0,0%

Select for downloading or viewing reports	Kh008_ITS Description	Max score	Total score	Query cover	E value	Ident	Accession	(Ident/Cover)* Max score	Deviation from top hit
Select seq gb|EU735059.1|	Thymus trautvetteri 18S ribosomal RNA gene, partial sequence; internal transcribed spacer 1, 5.8S ribosomal RNA gene, and internal transcribed spacer 2, complete sequence; and 28S ribosomal RNA gene, partial sequence	1267	1267	92%	0.0	100%	EU735059.1	1377,17	100,0%
Select seq gb|EU735058.1|	Thymus persicus isolate Ardabil 18S ribosomal RNA gene, partial sequence; internal transcribed spacer 1, 5.8S ribosomal RNA gene, and internal transcribed spacer 2, complete sequence; and 28S ribosomal RNA gene, partial sequence	1267	1267	92%	0.0	100%	EU735058.1	1377,17	100,0%
Select seq gb|EU374715.1|	Thymus pubescens 18S ribosomal RNA gene, partial sequence; internal transcribed spacer 1, 5.8S ribosomal RNA gene, and internal transcribed spacer 2, complete sequence; and 25/28S ribosomal RNA gene, partial sequence	1142	1142	83%	0.0	100%	EU374715.1	1375,90	99,9%
Select seq gb|AY443448.1|	Thymus magnus JUNG-TM04 internal transcribed spacer 1, 5.8S ribosomal RNA and internal transcribed spcer 2 genes, complete sequence	1131	1131	83%	0.0	99%	AY443448.1	1349,02	98,0%
Select seq gb|AY443434.1|	Thymus quinquecostatus JUNG-TQ01 internal transcribed spacer 1, 5.8S ribosomal RNA and internal transcribed spcer 2 genes, complete sequence	1131	1131	83%	0.0	99%	AY443434.1	1349,02	98,0%
Select seq gb|EU556520.1|	Thymus quinquecostatus isolate XZ internal transcribed spacer 1, partial sequence; 5.8S ribosomal RNA gene, complete sequence; and internal transcribed spacer 2, partial sequence	1240	1240	91%	0.0	99%	EU556520.1	1349,01	98,0%
Select seq gb|EU556524.1|	Thymus quinquecostatus isolate ZY internal transcribed spacer 1, partial sequence; 5.8S ribosomal RNA gene, complete sequence; and internal transcribed spacer 2, partial sequence	1251	1251	92%	0.0	99%	EU556524.1	1346,18	97,7%
Select seq gb|EU556511.1|	Thymus dahuricus isolate HD internal transcribed spacer 1, partial sequence; 5.8S ribosomal RNA gene, complete sequence; and internal transcribed spacer 2, partial sequence	1251	1251	92%	0.0	99%	EU556511.1	1346,18	97,7%
Select seq gb|EU556522.1|	Thymus quinquecostatus isolate ZJ internal transcribed spacer 1, partial sequence; 5.8S ribosomal RNA gene, complete sequence; and internal transcribed spacer 2, partial sequence	1249	1249	92%	0.0	99%	EU556522.1	1344,03	97,6%
Select seq gb|DQ667242.1|	Thymus serpyllum isolate x075 18S ribosomal RNA gene, partial sequence; internal transcribed spacer 1, 5.8S ribosomal RNA gene, and internal transcribed spacer 2, complete sequence; and 28S ribosomal RNA gene, partial sequence	1288	1288	95%	0.0	99%	DQ667242.1	1342,23	97,5%
Select seq gb|AY443445.1|	Thymus magnus JUNG-TM01 internal transcribed spacer 1, 5.8S ribosomal RNA and internal transcribed spcer 2 genes, complete sequence	1125	1125	83%	0.0	99%	AY443445.1	1341,87	97,4%
Select seq gb|FJ236468.1|	Thymus persicus isolate Tabriz 18S ribosomal RNA gene, partial sequence; internal transcribed spacer 1, 5.8S ribosomal RNA gene, and internal transcribed spacer 2, complete sequence; and 28S ribosomal RNA gene, partial sequence	1260	1260	93%	0.0	99%	FJ236468.1	1341,29	97,4%
Select seq gb|EU556510.1|	Thymus mongolicus isolate G3 internal transcribed spacer 1, partial sequence; 5.8S ribosomal RNA gene, complete sequence; and internal transcribed spacer 2, partial sequence	1138	1138	84%	0.0	99%	EU556510.1	1341,21	97,4%
Select seq gb|EU556518.1|	Thymus mongolicus isolate S1 internal transcribed spacer 1, partial sequence; 5.8S ribosomal RNA gene, complete sequence; and internal transcribed spacer 2, partial sequence	1192	1192	88%	0.0	99%	EU556518.1	1341,00	97,4%
Select seq gb|EU556517.1|	Thymus quinquecostatus isolate N2 internal transcribed spacer 1, partial sequence; 5.8S ribosomal RNA gene, complete sequence; and internal transcribed spacer 2, partial sequence	1219	1219	90%	0.0	99%	EU556517.1	1340,90	97,4%
Select seq gb|GU381459.1|	Thymus serpyllum voucher M:Braeuchler 2514 internal transcribed spacer 1, partial sequence; 5.8S ribosomal RNA gene, complete sequence; and internal transcribed spacer 2, partial sequence	1110	1110	82%	0.0	99%	GU381459.1	1340,12	97,3%
Select seq gb|EU556507.1|	Thymus quinquecostatus isolate CL internal transcribed spacer 1, partial sequence; 5.8S ribosomal RNA gene, complete sequence; and internal transcribed spacer 2, partial sequence	1245	1245	92%	0.0	99%	EU556507.1	1339,73	97,3%
Select seq gb|AY029168.1|	Thymus mastichina internal transcribed spacer 1, 5.8S ribosomal RNA gene, and internal transcribed spacer 2, complete sequence	1123	1123	83%	0.0	99%	AY029168.1	1339,48	97,3%
Select seq gb|EU556506.1|	Thymus quinquecostatus isolate D1 internal transcribed spacer 1, partial sequence; 5.8S ribosomal RNA gene, complete sequence; and internal transcribed spacer 2, partial sequence	1203	1203	89%	0.0	99%	EU556506.1	1338,17	97,2%
Select seq gb|EU556519.1|	Thymus mongolicus isolate S2 internal transcribed spacer 1, partial sequence; 5.8S ribosomal RNA gene, complete sequence; and internal transcribed spacer 2, partial sequence	1188	1188	88%	0.0	99%	EU556519.1	1336,50	97,0%
Select seq gb|AY443447.1|	Thymus magnus JUNG-TM03 internal transcribed spacer 1, 5.8S ribosomal RNA and internal transcribed spcer 2 genes, complete sequence	1120	1120	83%	0.0	99%	AY443447.1	1335,90	97,0%
Select seq gb|AY443438.1|	Thymus quinquecostatus JUNG-TQ05 internal transcribed spacer 1, 5.8S ribosomal RNA and internal transcribed spcer 2 genes, complete sequence	1120	1120	83%	0.0	99%	AY443438.1	1335,90	97,0%
Select seq gb|EU556521.1|	Thymus mongolicus isolate XM internal transcribed spacer 1, partial sequence; 5.8S ribosomal RNA gene, complete sequence; and internal transcribed spacer 2, partial sequence	1133	1133	84%	0.0	99%	EU556521.1	1335,32	97,0%
Select seq gb|JQ669138.1|	Thymus pulegioides voucher Riina 1577 18S ribosomal RNA gene, internal transcribed spacer 1, 5.8S ribosomal RNA gene, internal transcribed spacer 2, and 26S ribosomal RNA gene, region	1254	1254	93%	0.0	99%	JQ669138.1	1334,90	96,9%
Select seq gb|EU556509.1|	Thymus mongolicus isolate G2 internal transcribed spacer 1, partial sequence; 5.8S ribosomal RNA gene, complete sequence; and internal transcribed spacer 2, partial sequence	1186	1186	88%	0.0	99%	EU556509.1	1334,25	96,9%
Select seq gb|EU556516.1|	Thymus quinquecostatus isolate N1 internal transcribed spacer 1, partial sequence; 5.8S ribosomal RNA gene, complete sequence; and internal transcribed spacer 2, partial sequence	1197	1197	89%	0.0	99%	EU556516.1	1331,49	96,7%

Select seq gb|EU556508.1|	Thymus mongolicus isolate G1 internal transcribed spacer 1, partial sequence; 5.8S ribosomal RNA gene, complete sequence; and internal transcribed spacer 2, partial sequence	1170	1170	87% 0.0	99% EU556508.1	1331,38	96,7%
Select seq gb|AY443444.1|	Thymus quinquecostatus JUNG-TQ11 internal transcribed spacer 1, 5.8S ribosomal RNA and internal transcribed spcer 2 genes, complete sequence	1114	1114	83% 0.0	99% AY443444.1	1328,75	96,5%
Select seq gb|AY443443.1|	Thymus quinquecostatus JUNG-TQ10 internal transcribed spacer 1, 5.8S ribosomal RNA and internal transcribed spcer 2 genes, complete sequence	1114	1114	83% 0.0	99% AY443443.1	1328,75	96,5%
Select seq gb|AY443442.1|	Thymus quinquecostatus JUNG-TQ09 internal transcribed spacer 1, 5.8S ribosomal RNA and internal transcribed spcer 2 genes, complete sequence	1114	1114	83% 0.0	99% AY443442.1	1328,75	96,5%
Select seq gb|AY443441.1|	Thymus quinquecostatus JUNG-TQ08 internal transcribed spacer 1, 5.8S ribosomal RNA and internal transcribed spcer 2 genes, complete sequence	1114	1114	83% 0.0	99% AY443441.1	1328,75	96,5%
Select seq gb|AY443437.1|	Thymus quinquecostatus JUNG-TQ04 internal transcribed spacer 1, 5.8S ribosomal RNA and internal transcribed spcer 2 genes, complete sequence	1114	1114	83% 0.0	99% AY443437.1	1328,75	96,5%
Select seq gb|AY443436.1|	Thymus quinquecostatus JUNG-TQ03 internal transcribed spacer 1, 5.8S ribosomal RNA and internal transcribed spcer 2 genes, complete sequence	1114	1114	83% 0.0	99% AY443436.1	1328,75	96,5%
Select seq gb|EU796890.1|	Thymus serpyllum 18S ribosomal RNA gene, partial sequence; internal transcribed spacer 1, 5.8S ribosomal RNA gene, and internal transcribed spacer 2, complete sequence; and 26S ribosomal RNA gene, partial sequence	1206	1206	90% 0.0	99% EU796890.1	1326,60	96,3%
Select seq gb|EU785939.1|	Thymus vulgaris 18S ribosomal RNA gene, partial sequence; internal transcribed spacer 1, 5.8S ribosomal RNA gene, and internal transcribed spacer 2, complete sequence; and 26S ribosomal RNA gene, partial sequence	1203	1203	90% 0.0	99% EU785939.1	1323,30	96,1%
Select seq gb|AY443446.1|	Thymus magnus JUNG-TM02 internal transcribed spacer 1, 5.8S ribosomal RNA and internal transcribed spcer 2 genes, complete sequence	1103	1103	83% 0.0	99% AY443446.1	1315,63	95,5%
Select seq gb|AY443440.1|	Thymus quinquecostatus JUNG-TQ07 internal transcribed spacer 1, 5.8S ribosomal RNA and internal transcribed spcer 2 genes, complete sequence	1103	1103	83% 0.0	99% AY443440.1	1315,63	95,5%
Select seq gb|EU556523.1|	Thymus marschallianus isolate XY internal transcribed spacer 1, partial sequence; 5.8S ribosomal RNA gene, complete sequence; and internal transcribed spacer 2, partial sequence	1192	1192	90% 0.0	99% EU556523.1	1311,20	95,2%
Select seq gb|GU381458.1|	Thymus broussonetii subsp. hannonis voucher MSB:Podlech 142 internal transcribed spacer 1, partial sequence; 5.8S ribosomal RNA gene, complete sequence; and internal transcribed spacer 2, partial sequence	1086	1086	82% 0.0	99% GU381458.1	1311,15	95,2%
Select seq gb|AY443435.1|	Thymus quinquecostatus JUNG-TQ02 internal transcribed spacer 1, 5.8S ribosomal RNA and internal transcribed spcer 2 genes, complete sequence	1092	1092	83% 0.0	98% AY443435.1	1289,35	93,6%
Select seq gb|EU556512.1|	Thymus mandschuricus isolate HM internal transcribed spacer 1, partial sequence; 5.8S ribosomal RNA gene, complete sequence; and internal transcribed spacer 2, partial sequence	1179	1179	92% 0.0	98% EU556512.1	1255,89	91,2%
Select seq gb|AY329369.1|	Thymus vulgaris internal transcribed spacer 1, partial sequence; 5.8S ribosomal RNA gene, complete sequence; and internal transcribed spacer 2, partial sequence	1098	1098	86% 0.0	97% AY329369.1	1238,44	89,9%
Select seq gb|AY506646.1|	Thymus vulgaris internal transcribed spacer 1, partial sequence; 5.8S ribosomal RNA gene, complete sequence; and internal transcribed spacer 2, partial sequence	1092	1092	86% 0.0	97% AY506646.1	1231,67	89,4%
Select seq gb|EU556515.1|	Thymus amurensis isolate HW internal transcribed spacer 1, partial sequence; 5.8S ribosomal RNA gene, complete sequence; and internal transcribed spacer 2, partial sequence	1142	1142	92% 0.0	97% EU556515.1	1204,07	87,4%
Select seq gb|EU556514.1|	Thymus quinquecostatus isolate HY internal transcribed spacer 1, partial sequence; 5.8S ribosomal RNA gene, complete sequence; and internal transcribed spacer 2, partial sequence	1026	1026	83% 0.0	97% EU556514.1	1199,06	87,1%
Select seq gb|EU556513.1|	Thymus przewalskii isolate HX internal transcribed spacer 1, partial sequence; 5.8S ribosomal RNA gene, complete sequence; and internal transcribed spacer 2, partial sequence	1033	1033	86% 0.0	96% EU556513.1	1153,12	83,7%
Select seq gb|GU381460.1|	Thymus saturejoides subsp. commutatus voucher M:Podlech 47906 internal transcribed spacer 1, partial sequence; 5.8S ribosomal RNA gene, complete sequence; and internal transcribed spacer 2, partial sequence	965	965	82% 0.0	95% GU381460.1	1117,99	81,2%
Select seq gb|GU381462.1|	Saccocalyx saturejoides voucher MSB:Faurel 5650 internal transcribed spacer 1, partial sequence; 5.8S ribosomal RNA gene, complete sequence; and internal transcribed spacer 2, partial sequence	953	953	82% 0.0	95% GU381462.1	1104,09	80,2%
Select seq gb|GU381457.1|	Thymus caespititius voucher M:Heubl s.n. internal transcribed spacer 1, partial sequence; 5.8S ribosomal RNA gene, complete sequence; and internal transcribed spacer 2, partial sequence	953	953	82% 0.0	95% GU381457.1	1104,09	80,2%
Select seq gb|GU381461.1|	Argantoniella salzmannii voucher M:Barra et al. 2673GL internal transcribed spacer 1, partial sequence; 5.8S ribosomal RNA gene, complete sequence; and internal transcribed spacer 2, partial sequence	948	948	82% 0.0	95% GU381461.1	1098,29	79,7%
Select seq gb|JQ669137.1|	Thymbra capitata voucher UCBG 96.0817 s.n. 18S ribosomal RNA gene, internal transcribed spacer 1, 5.8S ribosomal RNA gene, internal transcribed spacer 2, and 26S ribosomal RNA gene, region	1053	1053	93% 0.0	94% JQ669137.1	1064,32	77,3%
Select seq gb|GU381451.1|	Thymbra sintenisii subsp. isaurica voucher E:Goener 12628 internal transcribed spacer 1, partial sequence; 5.8S ribosomal RNA gene, complete sequence; and internal transcribed spacer 2, partial sequence	915	915	81% 0.0	94% GU381451.1	1061,85	77,1%
Select seq gb|GU381452.1|	Thymbra calostachya voucher M:Ulrich s.n. internal transcribed spacer 1, partial sequence; 5.8S ribosomal RNA gene, complete sequence; and internal transcribed spacer 2, partial sequence	909	909	81% 0.0	94% GU381452.1	1054,89	76,6%
Select seq gb|GU381455.1|	Satureja linearifolia voucher M:Brullo & Furnari s.n. internal transcribed spacer 1, partial sequence; 5.8S ribosomal RNA gene, complete sequence; and internal transcribed spacer 2, partial sequence	907	907	81% 0.0	94% GU381455.1	1052,57	76,4%

Select seq gb|JQ669127.1|	Origanum vulgare voucher B. Drew 77 18S ribosomal RNA gene, internal transcribed spacer 1, 5.8S ribosomal RNA gene, internal transcribed spacer 2, and 26S ribosomal RNA gene, region	1020	1020	93% 0.0	93% JQ669127.1	1020,00	74,1%
Select seq gb|DQ667243.1|	Origanum vulgare isolate x076 18S ribosomal RNA gene, partial sequence; internal transcribed spacer 1, 5.8S ribosomal RNA gene, and internal transcribed spacer 2, complete sequence; and 28S ribosomal RNA gene, partial sequence	1018	1018	94% 0.0	93% DQ667243.1	1007,17	73,1%
Select seq gb|AY506647.1|	Origanum vulgare internal transcribed spacer 1, partial sequence; 5.8S ribosomal RNA gene, complete sequence; and internal transcribed spacer 2, partial sequence	928	928	87% 0.0	92% AY506647.1	981,33	71,3%
Select seq gb|JX162834.1|	Origanum majorana voucher OBI clone 3 internal transcribed spacer 1, partial sequence; 5.8S ribosomal RNA gene, complete sequence; and internal transcribed spacer 2, partial sequence	917	917	86% 0.0	92% JX162834.1	980,98	71,2%
Select seq gb|JX162831.1|	Origanum majorana voucher Mira2-3 clone 10 internal transcribed spacer 1, partial sequence; 5.8S ribosomal RNA gene, complete sequence; and internal transcribed spacer 2, partial sequence	917	917	86% 0.0	92% JX162831.1	980,98	71,2%
Select seq gb|JX163036.1|	Origanum syriacum voucher LC7 clone 8 internal transcribed spacer 1, partial sequence; 5.8S ribosomal RNA gene, complete sequence; and internal transcribed spacer 2, partial sequence	913	913	86% 0.0	92% JX163036.1	976,70	70,9%
Select seq gb|JX162836.1|	Origanum majorana voucher OBI clone 5 internal transcribed spacer 1, partial sequence; 5.8S ribosomal RNA gene, complete sequence; and internal transcribed spacer 2, partial sequence	913	913	86% 0.0	92% JX162836.1	976,70	70,9%
Select seq gb|JX162980.1|	Origanum onites voucher SR777 clone 14 internal transcribed spacer 1, partial sequence; 5.8S ribosomal RNA gene, complete sequence; and internal transcribed spacer 2, partial sequence	911	911	86% 0.0	92% JX162980.1	974,56	70,8%
Select seq gb|JX162837.1|	Origanum majorana voucher OBI clone 6 internal transcribed spacer 1, partial sequence; 5.8S ribosomal RNA gene, complete sequence; and internal transcribed spacer 2, partial sequence	911	911	86% 0.0	92% JX162837.1	974,56	70,8%
Select seq gb|JX162826.1|	Origanum majorana voucher Mira2-3 clone 4 internal transcribed spacer 1, partial sequence; 5.8S ribosomal RNA gene, complete sequence; and internal transcribed spacer 2, partial sequence	911	911	86% 0.0	92% JX162826.1	974,56	70,8%
Select seq gb|JX162814.1|	Origanum majorana voucher SR562 clone 1 internal transcribed spacer 1, partial sequence; 5.8S ribosomal RNA gene, complete sequence; and internal transcribed spacer 2, partial sequence	911	911	86% 0.0	92% JX162814.1	974,56	70,8%
Select seq gb|JX162807.1|	Origanum majorana voucher LC10 clone 4 internal transcribed spacer 1, partial sequence; 5.8S ribosomal RNA gene, complete sequence; and internal transcribed spacer 2, partial sequence	911	911	86% 0.0	92% JX162807.1	974,56	70,8%
Select seq gb|JX162805.1|	Origanum majorana voucher LC10 clone 2 internal transcribed spacer 1, partial sequence; 5.8S ribosomal RNA gene, complete sequence; and internal transcribed spacer 2, partial sequence	911	911	86% 0.0	92% JX162805.1	974,56	70,8%
Select seq gb|JX162830.1|	Origanum majorana voucher Mira2-3 clone 9 internal transcribed spacer 1, partial sequence; 5.8S ribosomal RNA gene, complete sequence; and internal transcribed spacer 2, partial sequence	907	907	86% 0.0	92% JX162830.1	970,28	70,5%
Select seq gb|JX163016.1|	Origanum syriacum voucher RNG:H50 clone 8 internal transcribed spacer 1, partial sequence; 5.8S ribosomal RNA gene, complete sequence; and internal transcribed spacer 2, partial sequence	905	905	86% 0.0	92% JX163016.1	968,14	70,3%
Select seq gb|JX162978.1|	Origanum onites voucher SR777 clone 11 internal transcribed spacer 1, partial sequence; 5.8S ribosomal RNA gene, complete sequence; and internal transcribed spacer 2, partial sequence	905	905	86% 0.0	92% JX162978.1	968,14	70,3%
Select seq gb|JX162966.1|	Origanum onites voucher SR530 clone 8 internal transcribed spacer 1, partial sequence; 5.8S ribosomal RNA gene, complete sequence; and internal transcribed spacer 2, partial sequence	905	905	86% 0.0	92% JX162966.1	968,14	70,3%
Select seq gb|JX162832.1|	Origanum majorana voucher OBI clone 1 internal transcribed spacer 1, partial sequence; 5.8S ribosomal RNA gene, complete sequence; and internal transcribed spacer 2, partial sequence	905	905	86% 0.0	92% JX162832.1	968,14	70,3%
Select seq gb|JX162829.1|	Origanum majorana voucher Mira2-3 clone 8 internal transcribed spacer 1, partial sequence; 5.8S ribosomal RNA gene, complete sequence; and internal transcribed spacer 2, partial sequence	905	905	86% 0.0	92% JX162829.1	968,14	70,3%
Select seq gb|JX162823.1|	Origanum majorana voucher Mira2-3 clone 1 internal transcribed spacer 1, partial sequence; 5.8S ribosomal RNA gene, complete sequence; and internal transcribed spacer 2, partial sequence	905	905	86% 0.0	92% JX162823.1	968,14	70,3%
Select seq gb|JX162818.1|	Origanum majorana voucher SR562 clone 5 internal transcribed spacer 1, partial sequence; 5.8S ribosomal RNA gene, complete sequence; and internal transcribed spacer 2, partial sequence	905	905	86% 0.0	92% JX162818.1	968,14	70,3%
Select seq gb|JX162816.1|	Origanum majorana voucher SR562 clone 3 internal transcribed spacer 1, partial sequence; 5.8S ribosomal RNA gene, complete sequence; and internal transcribed spacer 2, partial sequence	905	905	86% 0.0	92% JX162816.1	968,14	70,3%
Select seq gb|JX162815.1|	Origanum majorana voucher SR562 clone 2 internal transcribed spacer 1, partial sequence; 5.8S ribosomal RNA gene, complete sequence; and internal transcribed spacer 2, partial sequence	905	905	86% 0.0	92% JX162815.1	968,14	70,3%
Select seq gb|JX163024.1|	Origanum syriacum voucher RNG:H50 clone 16 internal transcribed spacer 1, partial sequence; 5.8S ribosomal RNA gene, complete sequence; and internal transcribed spacer 2, partial sequence	902	902	86% 0.0	92% JX163024.1	964,93	70,1%
Select seq gb|JX163022.1|	Origanum syriacum voucher RNG:H50 clone 14 internal transcribed spacer 1, partial sequence; 5.8S ribosomal RNA gene, complete sequence; and internal transcribed spacer 2, partial sequence	902	902	86% 0.0	92% JX163022.1	964,93	70,1%
Select seq gb|JX163021.1|	Origanum syriacum voucher RNG:H50 clone 13 internal transcribed spacer 1, partial sequence; 5.8S ribosomal RNA gene, complete sequence; and internal transcribed spacer 2, partial sequence	902	902	86% 0.0	92% JX163021.1	964,93	70,1%
Select seq gb|JX163014.1|	Origanum syriacum voucher RNG:H50 clone 6 internal transcribed spacer 1, partial sequence; 5.8S ribosomal RNA gene, complete sequence; and internal transcribed spacer 2, partial sequence	902	902	86% 0.0	92% JX163014.1	964,93	70,1%
Select seq gb|JX163010.1|	Origanum syriacum voucher RNG:H50 clone 2 internal transcribed spacer 1, partial sequence; 5.8S ribosomal RNA gene, complete sequence; and internal transcribed spacer 2, partial sequence	902	902	86% 0.0	92% JX163010.1	964,93	70,1%

Select seq gb|JX163004.1|	Origanum onites voucher SR1372 clone 8 internal transcribed spacer 1, partial sequence; 5.8S ribosomal RNA gene, complete sequence; and internal transcribed spacer 2, partial sequence	902	902	86%	0.0	92% JX163004.1	964,93	70,1%
Select seq gb|JX163019.1|	Origanum syriacum voucher RNG:H50 clone 11 internal transcribed spacer 1, partial sequence; 5.8S ribosomal RNA gene, complete sequence; and internal transcribed spacer 2, partial sequence	900	900	86%	0.0	92% JX163019.1	962,79	69,9%
Select seq gb|JX163018.1|	Origanum syriacum voucher RNG:H50 clone 10 internal transcribed spacer 1, partial sequence; 5.8S ribosomal RNA gene, complete sequence; and internal transcribed spacer 2, partial sequence	900	900	86%	0.0	92% JX163018.1	962,79	69,9%
Select seq gb|JX163017.1|	Origanum syriacum voucher RNG:H50 clone 9 internal transcribed spacer 1, partial sequence; 5.8S ribosomal RNA gene, complete sequence; and internal transcribed spacer 2, partial sequence	900	900	86%	0.0	92% JX163017.1	962,79	69,9%
Select seq gb|JX163054.1|	Origanum onites voucher SR810 internal transcribed spacer 1, partial sequence; 5.8S ribosomal RNA gene, complete sequence; and internal transcribed spacer 2, partial sequence	891	891	86%	0.0	91% JX163054.1	942,80	68,5%
Select seq gb|JQ669139.1|	Zataria multiflora voucher Rechinger 51885 18S ribosomal RNA gene, internal transcribed spacer 1, 5.8S ribosomal RNA gene, internal transcribed spacer 2, and 26S ribosomal RNA gene, region	928	928	93%	0.0	91% JQ669139.1	908,04	65,9%
Select seq gb|DQ667333.1|	Acanthomintha lanceolata isolate x545 18S ribosomal RNA gene, partial sequence; internal transcribed spacer 1, 5.8S ribosomal RNA gene, and internal transcribed spacer 2, complete sequence; and 28S ribosomal RNA gene, partial sequence	950	950	96%	0.0	91% DQ667333.1	900,52	65,4%
Select seq gb|DQ667311.1|	Poliomintha palmeri isolate x259 18S ribosomal RNA gene, partial sequence; internal transcribed spacer 1, 5.8S ribosomal RNA gene, and internal transcribed spacer 2, complete sequence; and 28S ribosomal RNA gene, partial sequence	907	907	97%	0.0	89% DQ667311.1	832,20	60,4%
Select seq gb|DQ667310.1|	Glechon thymoides isolate x256 18S ribosomal RNA gene, partial sequence; internal transcribed spacer 1, 5.8S ribosomal RNA gene, and internal transcribed spacer 2, complete sequence; and 28S ribosomal RNA gene, partial sequence	907	907	97%	0.0	89% DQ667310.1	832,20	60,4%
Select seq gb|DQ667305.1|	Cunila microcephala isolate x226 18S ribosomal RNA gene, partial sequence; internal transcribed spacer 1, 5.8S ribosomal RNA gene, and internal transcribed spacer 2, complete sequence; and 28S ribosomal RNA gene, partial sequence	907	907	97%	0.0	89% DQ667305.1	832,20	60,4%
Select seq gb|DQ667303.1|	Glechon marifolia isolate x223 18S ribosomal RNA gene, partial sequence; internal transcribed spacer 1, 5.8S ribosomal RNA gene, and internal transcribed spacer 2, complete sequence; and 28S ribosomal RNA gene, partial sequence	907	907	97%	0.0	89% DQ667303.1	832,20	60,4%
Select seq gb|DQ667244.1|	Mentha spicata isolate x077 18S ribosomal RNA gene, partial sequence; internal transcribed spacer 1, 5.8S ribosomal RNA gene, and internal transcribed spacer 2, complete sequence; and 28S ribosomal RNA gene, partial sequence	905	905	97%	0.0	89% DQ667244.1	830,36	60,3%
Select seq gb|DQ667325.1|	Mentha arvensis isolate x424 18S ribosomal RNA gene, partial sequence; internal transcribed spacer 1, 5.8S ribosomal RNA gene, and internal transcribed spacer 2, complete sequence; and 28S ribosomal RNA gene, partial sequence	902	902	97%	0.0	89% DQ667325.1	827,61	60,1%
							0,00	0,0%
							0,00	0,0%
							0,00	0,0%
							0,00	0,0%

Select for downloading or viewing reports	Kh008_trnL Description	Max score	Total score	Query cover	E value	Ident	Accession	(Ident/Cover)* Max score	Deviation from top hit
Select seq gb|GU381638.1|	Thymus haussknechtii voucher M:Nydegger 43838 tRNA-Leu (trnL) gene and trnL-trnF intergenic spacer, partial sequence; chloroplast	1140	1140	93%	0.0	99%	GU381638.1	1213,55	100,0%
Select seq gb|GU381635.1|	Thymus broussonetii subsp. hannonis voucher MSB:Podlech 142 tRNA-Leu (trnL) gene and trnL-trnF intergenic spacer, partial sequence; chloroplast	1162	1162	95%	0.0	99%	GU381635.1	1210,93	99,8%
Select seq gb|JQ669069.1|	Thymus pulegioides voucher Riina 1577 tRNA-Leu (trnL) gene and trnL-trnF intergenic spacer, partial sequence; plastid	1157	1157	95%	0.0	99%	JQ669069.1	1205,72	99,4%
Select seq gb|GU381637.1|	Thymus serpyllum voucher M:Braeuchler 2514 tRNA-Leu (trnL) gene and trnL-trnF intergenic spacer, partial sequence; chloroplast	1157	1157	95%	0.0	99%	GU381637.1	1205,72	99,4%
Select seq gb|EU556535.1|	Thymus proximus isolate XN tRNA-Leu (trnL) gene and trnL-trnF intergenic spacer, partial sequence; chloroplast	1118	1118	92%	0.0	99%	EU556535.1	1203,07	99,1%
Select seq gb|AY506613.1|	Thymus vulgaris tRNA-Leu (trnL) gene and trnL-trnF intergenic spacer, partial sequence; chloroplast	1129	1129	93%	0.0	99%	AY506613.1	1201,84	99,0%
Select seq gb|GU381634.1|	Thymus pulegioides voucher M:Braeuchler 3129 tRNA-Leu (trnL) gene and trnL-trnF intergenic spacer, partial sequence; chloroplast	1153	1153	95%	0.0	99%	GU381634.1	1201,55	99,0%
Select seq gb|KR063657.1|	Thymus sibthorpii tRNA-Leu (trnL) gene, partial sequence; trnL-trnF intergenic spacer, complete sequence; and tRNA-Phe (trnF) gene, partial sequence; chloroplast	1212	1212	100%	0.0	99%	KR063657.1	1199,88	98,9%
Select seq gb|EU556532.1|	Thymus amurensis isolate HX tRNA-Leu (trnL) gene and trnL-trnF intergenic spacer, partial sequence; chloroplast	1212	1212	100%	0.0	99%	EU556532.1	1199,88	98,9%
Select seq gb|GU381636.1|	Thymus vulgaris voucher M:Braeuchler 3683 tRNA-Leu (trnL) gene and trnL-trnF intergenic spacer, partial sequence; chloroplast	1151	1151	95%	0.0	99%	GU381636.1	1199,46	98,8%
Select seq gb|EU556534.1|	Thymus mongolicus isolate S1 tRNA-Leu (trnL) gene and trnL-trnF intergenic spacer, partial sequence; chloroplast	1112	1112	92%	0.0	99%	EU556534.1	1196,61	98,6%
Select seq gb|EU556536.1|	Thymus marschallianus isolate XY tRNA-Leu (trnL) gene and trnL-trnF intergenic spacer, partial sequence; chloroplast	1147	1147	95%	0.0	99%	EU556536.1	1195,29	98,5%
Select seq gb|GU381633.1|	Thymus caespititius voucher M:Heubl s.n. tRNA-Leu (trnL) gene and trnL-trnF intergenic spacer, partial sequence; chloroplast	1146	1146	95%	0.0	99%	GU381633.1	1194,25	98,4%
Select seq gb|EU556525.1|	Thymus quinquecostatus isolate CL tRNA-Leu (trnL) gene and trnL-trnF intergenic spacer, partial sequence; chloroplast	1206	1206	100%	0.0	99%	EU556525.1	1193,94	98,4%
Select seq gb|EU556530.1|	Thymus mandschuricus isolate HM tRNA-Leu (trnL) gene and trnL-trnF intergenic spacer, partial sequence; chloroplast	1157	1157	96%	0.0	99%	EU556530.1	1193,16	98,3%
Select seq gb|EU556539.1|	Thymus quinquecostatus isolate ZY tRNA-Leu (trnL) gene and trnL-trnF intergenic spacer, partial sequence; chloroplast	1205	1205	100%	0.0	99%	EU556539.1	1192,95	98,3%
Select seq gb|EU556538.1|	Thymus quinquecostatus isolate ZJ tRNA-Leu (trnL) gene and trnL-trnF intergenic spacer, partial sequence; chloroplast	1205	1205	100%	0.0	99%	EU556538.1	1192,95	98,3%
Select seq gb|EU556533.1|	Thymus quinquecostatus isolate HY tRNA-Leu (trnL) gene and trnL-trnF intergenic spacer, partial sequence; chloroplast	1205	1205	100%	0.0	99%	EU556533.1	1192,95	98,3%
Select seq gb|EU556527.1|	Thymus mongolicus isolate G2 tRNA-Leu (trnL) gene and trnL-trnF intergenic spacer, partial sequence; chloroplast	1205	1205	100%	0.0	99%	EU556527.1	1192,95	98,3%
Select seq gb|EU556526.1|	Thymus quinquecostatus isolate D2 tRNA-Leu (trnL) gene and trnL-trnF intergenic spacer, partial sequence; chloroplast	1205	1205	100%	0.0	99%	EU556526.1	1192,95	98,3%
Select seq gb|JQ690290.1|	Origanum elongatum isolate H5_O_elo trnL-trnF intergenic spacer, partial sequence; chloroplast	1177	1177	98%	0.0	99%	JQ690290.1	1189,01	98,0%
Select seq gb|JQ690289.1|	Origanum rotundifolium isolate DNA3_O_rot trnL-trnF intergenic spacer, partial sequence; chloroplast	1177	1177	98%	0.0	99%	JQ690289.1	1189,01	98,0%
Select seq gb|GU381640.1|	Argantoniella salzmannii voucher M:Barra et al. 2673GL tRNA-Leu (trnL) gene and trnL-trnF intergenic spacer, partial sequence; chloroplast	1140	1140	95%	0.0	99%	GU381640.1	1188,00	97,9%
Select seq gb|AY840202.1|	Origanum vulgare tRNA-Leu (trnL) gene and trnL-trnF intergenic spacer, partial sequence; chloroplast	1140	1140	95%	0.0	99%	AY840202.1	1188,00	97,9%
Select seq gb|EU556531.1|	Thymus amurensis isolate HW tRNA-Leu (trnL) gene and trnL-trnF intergenic spacer, partial sequence; chloroplast	1199	1199	100%	0.0	99%	EU556531.1	1187,01	97,8%
Select seq gb|EU556528.1|	Thymus mongolicus isolate G3 tRNA-Leu (trnL) gene and trnL-trnF intergenic spacer, partial sequence; chloroplast	1151	1151	96%	0.0	99%	EU556528.1	1186,97	97,8%

Select seq emb|AJ505544.1|	Thymus serpyllum var. citriodorum plastid trnL-trnF intergenic spacer, specimen voucher cult., K-1975-1177, Chase 13331 (K)	1170	1170	98%	0.0	99%	AJ505544.1	1181,94	97,4%
Select seq gb|AY570463.1|	Origanum vulgare voucher JBW 2567 tRNA-Leu and trnL-trnF intergenic spacer, partial sequence; chloroplast	1133	1133	95%	0.0	99%	AY570463.1	1180,71	97,3%
Select seq emb|AJ505543.1|	Origanum vulgare plastid trnL-trnF intergenic spacer, specimen voucher cult., K-000-69-19317, chase 13334 (K)	1168	1168	98%	0.0	99%	AJ505543.1	1179,92	97,2%
Select seq gb|JF301392.1|	Origanum vulgare voucher B. Drew 77 tRNA-Leu (trnL) gene and trnL-trnF intergenic spacer, partial sequence; chloroplast	1107	1107	93%	0.0	99%	JF301392.1	1178,42	97,1%
Select seq gb|JX880022.1|	Origanum vulgare subsp. vulgare chloroplast, complete genome	1190	1190	100%	0.0	99%	JX880022.1	1178,10	97,1%
Select seq gb|JQ690293.1|	Origanum dayi isolate H43_O_day trnL-trnF intergenic spacer, partial sequence; chloroplast	1166	1166	98%	0.0	99%	JQ690293.1	1177,90	97,1%
Select seq gb|EU556529.1|	Thymus dahuricus isolate HD tRNA-Leu (trnL) gene and trnL-trnF intergenic spacer, partial sequence; chloroplast	1142	1142	96%	0.0	99%	EU556529.1	1177,69	97,0%
Select seq gb|GU381629.1|	Thymbra capitata voucher M:Braeuchler 2518 tRNA-Leu (trnL) gene and trnL-trnF intergenic spacer, partial sequence; chloroplast	1123	1123	95%	0.0	99%	GU381629.1	1170,28	96,4%
Select seq gb|AY506614.1|	Origanum vulgare tRNA-Leu (trnL) gene and trnL-trnF intergenic spacer, partial sequence; chloroplast	1099	1099	93%	0.0	99%	AY506614.1	1169,90	96,4%
Select seq gb|JF301401.1|	Thymbra capitata voucher UCBG 96.0817 tRNA-Leu (trnL) gene and trnL-trnF intergenic spacer, partial sequence; chloroplast	1098	1098	93%	0.0	99%	JF301401.1	1168,84	96,3%
Select seq gb|EU556537.1|	Thymus quinquecostatus isolate XZ tRNA-Leu (trnL) gene and trnL-trnF intergenic spacer, partial sequence; chloroplast	1175	1175	100%	0.0	99%	EU556537.1	1163,25	95,9%
Select seq gb|GU381484.1|	Micromeria flagellaris voucher E:van der Werff & McPherson 13570 tRNA-Leu (trnL) gene and trnL-trnF intergenic spacer, partial sequence; chloroplast	1118	1118	95%	0.0	98%	GU381484.1	1153,31	95,0%
Select seq gb|GU381483.1|	Micromeria flagellaris voucher E:Clement et al 2140 tRNA-Leu (trnL) gene and trnL-trnF intergenic spacer, partial sequence; chloroplast	1118	1118	95%	0.0	98%	GU381483.1	1153,31	95,0%
Select seq gb|AY840207.1|	Thymbra spicata tRNA-Leu (trnL) gene and trnL-trnF intergenic spacer, partial sequence; chloroplast	1118	1118	95%	0.0	98%	AY840207.1	1153,31	95,0%
Select seq gb|GU381481.1|	Micromeria cf. madagascariensis Morawetz 205 tRNA-Leu (trnL) gene and trnL-trnF intergenic spacer, partial sequence; chloroplast	1094	1094	93%	0.0	98%	GU381481.1	1152,82	95,0%
Select seq gb|JQ669070.1|	Thymus serpyllum voucher J. Walker 2564 tRNA-Leu (trnL) gene and trnL-trnF intergenic spacer, partial sequence; plastid	1116	1116	95%	0.0	98%	JQ669070.1	1151,24	94,9%
Select seq gb|GU381632.1|	Thymbra spicata voucher M:Braeuchler 4548 tRNA-Leu (trnL) gene and trnL-trnF intergenic spacer, partial sequence; chloroplast	1114	1114	95%	0.0	98%	GU381632.1	1149,18	94,7%
Select seq gb|GU381627.1|	Thymbra sintenisii subsp. isaurica voucher E:Goener 12628 tRNA-Leu (trnL) gene and trnL-trnF intergenic spacer, partial sequence; chloroplast	1112	1112	95%	0.0	98%	GU381627.1	1147,12	94,5%
Select seq gb|AY570502.1|	Thymus serpyllum voucher JBW 2564 tRNA-Leu and trnL-trnF intergenic spacer, partial sequence; chloroplast	1123	1123	96%	0.0	98%	AY570502.1	1146,40	94,5%
Select seq gb|GU381517.1|	Clinopodium barosmum voucher BM<GBR-LONDON>:McLaren N193 tRNA-Leu (trnL) gene and trnL-trnF intergenic spacer, partial sequence; chloroplast	1101	1101	95%	0.0	98%	GU381517.1	1135,77	93,6%
Select seq gb|GU381516.1|	Clinopodium wardii voucher BM<GBR-LONDON>:Ludlow et al. 14234 tRNA-Leu (trnL) gene and trnL-trnF intergenic spacer, partial sequence; chloroplast	1101	1101	95%	0.0	98%	GU381516.1	1135,77	93,6%
Select seq gb|GU381515.1|	Clinopodium hydaspidis voucher BM<GBR-LONDON>:Mohd 133 tRNA-Leu (trnL) gene and trnL-trnF intergenic spacer, partial sequence; chloroplast	1101	1101	95%	0.0	98%	GU381515.1	1135,77	93,6%
Select seq gb|GU381514.1|	Clinopodium nepalense voucher FR:Stainton 6024 tRNA-Leu (trnL) gene and trnL-trnF intergenic spacer, partial sequence; chloroplast	1101	1101	95%	0.0	98%	GU381514.1	1135,77	93,6%
Select seq gb|GU381511.1|	Clinopodium piperitum voucher BM<GBR-LONDON>:Vickery 454 tRNA-Leu (trnL) gene and trnL-trnF intergenic spacer, partial sequence; chloroplast	1101	1101	95%	0.0	98%	GU381511.1	1135,77	93,6%
Select seq gb|GU381513.1|	Clinopodium nepalense voucher BM<GBR-LONDON>:Mikage et al. 9550294 tRNA-Leu (trnL) gene and trnL-trnF intergenic spacer, partial sequence; chloroplast	1074	1074	93%	0.0	98%	GU381513.1	1131,74	93,3%
Select seq gb|GU381512.1|	Clinopodium piperitum voucher E:Stainton 7320 tRNA-Leu (trnL) gene and trnL-trnF intergenic spacer, partial sequence; chloroplast	1096	1096	95%	0.0	98%	GU381512.1	1130,61	93,2%
Select seq gb|GU381496.1|	Killickia pilosa voucher M:Braeuchler 3832 tRNA-Leu (trnL) gene and trnL-trnF intergenic spacer, partial sequence; chloroplast	1092	1092	95%	0.0	98%	GU381496.1	1126,48	92,8%
Select seq gb|GU381493.1|	Killickia grandiflora voucher M:Braeuchler 3811 tRNA-Leu (trnL) gene and trnL-trnF intergenic spacer, partial sequence; chloroplast	1092	1092	95%	0.0	98%	GU381493.1	1126,48	92,8%

Select seq gb|GU381631.1|	Satureja linearifolia voucher M:Brullo & Furnari s.n. tRNA-Leu (trnL) gene and trnL-trnF intergenic spacer, partial sequence; chloroplast	1090	1090	95%	0.0	98%	GU381631.1	1124,42	92,7%
Select seq gb|GU381495.1|	Killickia pilosa voucher M:Braeuchler 3810 tRNA-Leu (trnL) gene and trnL-trnF intergenic spacer, partial sequence; chloroplast	1077	1077	94%	0.0	98%	GU381495.1	1122,83	92,5%
Select seq gb|GU381485.1|	Micromeria sphaerophylla voucher E:Lewis et al 1064 tRNA-Leu (trnL) gene and trnL-trnF intergenic spacer, partial sequence; chloroplast	1088	1088	95%	0.0	98%	GU381485.1	1122,36	92,5%
Select seq gb|GU381628.1|	Thymbra calostachya voucher M:Ulrich s.n. tRNA-Leu (trnL) gene and trnL-trnF intergenic spacer, partial sequence; chloroplast	1086	1086	95%	0.0	98%	GU381628.1	1120,29	92,3%
Select seq gb|GU381621.1|	Satureja cuneifolia voucher M:Rechinger 11142 tRNA-Leu (trnL) gene and trnL-trnF intergenic spacer, partial sequence; chloroplast	1081	1081	95%	0.0	97%	GU381621.1	1103,76	91,0%
Select seq gb|GU381612.1|	Gontscharovia popovii voucher BM<GBR-LONDON>:Schmid 2419 tRNA-Leu (trnL) gene and trnL-trnF intergenic spacer, partial sequence; chloroplast	1081	1081	95%	0.0	97%	GU381612.1	1103,76	91,0%
Select seq gb|GU381489.1|	Killickia lutea voucher NU<ZAF>:Hilliard & Burtt 9876 tRNA-Leu (trnL) gene and trnL-trnF intergenic spacer, partial sequence; chloroplast	1081	1081	95%	0.0	97%	GU381489.1	1103,76	91,0%
Select seq gb|GU381488.1|	Killickia compacta voucher M:Braeuchler 3816 tRNA-Leu (trnL) gene and trnL-trnF intergenic spacer, partial sequence; chloroplast	1081	1081	95%	0.0	97%	GU381488.1	1103,76	91,0%
Select seq gb|AY840179.1|	Satureja montana tRNA-Leu (trnL) gene and trnL-trnF intergenic spacer, partial sequence; chloroplast	1081	1081	95%	0.0	97%	AY840179.1	1103,76	91,0%
Select seq gb|GU381510.1|	Ziziphora pamiroalaica voucher C:Murray et al. 10090 tRNA-Leu (trnL) gene and trnL-trnF intergenic spacer, partial sequence; chloroplast	1079	1079	95%	0.0	97%	GU381510.1	1101,72	90,8%
Select seq gb|GU381507.1|	Ziziphora tenuior voucher MSB:Fayvush et al. 03-1503 tRNA-Leu (trnL) gene and trnL-trnF intergenic spacer, partial sequence; chloroplast	1079	1079	95%	0.0	97%	GU381507.1	1101,72	90,8%
Select seq gb|GU381505.1|	Clinopodium troodi voucher W:Davis 1856 tRNA-Leu (trnL) gene and trnL-trnF intergenic spacer, partial sequence; chloroplast	1079	1079	95%	0.0	97%	GU381505.1	1101,72	90,8%
Select seq gb|GU381501.1|	Clinopodium graveolens subsp. rotundifolium voucher M:Podlech 47181 tRNA-Leu (trnL) gene and trnL-trnF intergenic spacer, partial sequence; chloroplast	1079	1079	95%	0.0	97%	GU381501.1	1101,72	90,8%
Select seq gb|GU381499.1|	Clinopodium suaveolens voucher M:Erben s.n. tRNA-Leu (trnL) gene and trnL-trnF intergenic spacer, partial sequence; chloroplast	1079	1079	95%	0.0	97%	GU381499.1	1101,72	90,8%
Select seq gb|JQ669067.1|	Satureja montana voucher UCBG 2002.0593, Forbes s.n tRNA-Leu (trnL) gene and trnL-trnF intergenic spacer, partial sequence; plastid	1075	1075	95%	0.0	97%	JQ669067.1	1097,63	90,4%
Select seq gb|GU381619.1|	Satureja mutica voucher M:Akhani 12362 tRNA-Leu (trnL) gene and trnL-trnF intergenic spacer, partial sequence; chloroplast	1075	1075	95%	0.0	97%	GU381619.1	1097,63	90,4%
Select seq gb|GU381614.1|	Satureja thymbra voucher M:Braeuchler 2896 tRNA-Leu (trnL) gene and trnL-trnF intergenic spacer, partial sequence; chloroplast	1075	1075	95%	0.0	97%	GU381614.1	1097,63	90,4%
Select seq gb|GU381611.1|	Gontscharovia popovii voucher M:Vvedensky s.n. tRNA-Leu (trnL) gene and trnL-trnF intergenic spacer, partial sequence; chloroplast	1075	1075	95%	0.0	97%	GU381611.1	1097,63	90,4%
Select seq gb|KR063656.1|	Satureja pilosa subsp. origanita tRNA-Leu (trnL) gene, partial sequence; trnL-trnF intergenic spacer, complete sequence; and tRNA-Phe (trnF) gene, partial sequence; chloroplast	1131	1131	100%	0.0	97%	KR063656.1	1097,07	90,4%
Select seq gb|GU381497.1|	Clinopodium acinos voucher M:Podlech 50287 tRNA-Leu (trnL) gene and trnL-trnF intergenic spacer, partial sequence; chloroplast	1074	1074	95%	0.0	97%	GU381497.1	1096,61	90,4%
Select seq gb|GU381615.1|	Satureja innota voucher M:Barra et al. 2484GL tRNA-Leu (trnL) gene and trnL-trnF intergenic spacer, partial sequence; chloroplast	1070	1070	95%	0.0	97%	GU381615.1	1092,53	90,0%
Select seq gb|GU381521.1|	Mentha pulegium voucher M:Braeuchler 2300 tRNA-Leu (trnL) gene and trnL-trnF intergenic spacer, partial sequence; chloroplast	1070	1070	95%	0.0	97%	GU381521.1	1092,53	90,0%
Select seq gb|DQ667501.1|	Ziziphora taurica isolate x262 tRNA-Leu (trnL) gene and trnL-trnF intergenic spacer, partial sequence; chloroplast	1081	1081	96%	0.0	97%	DQ667501.1	1092,26	90,0%
Select seq gb|JQ669021.1|	Clinopodium acinos voucher Judziewicz 14160 tRNA-Leu (trnL) gene and trnL-trnF intergenic spacer, partial sequence; plastid	1068	1068	95%	0.0	97%	JQ669021.1	1090,48	89,9%
Select seq gb|DQ667492.1|	Lepechinia lancifolia isolate x232 tRNA-Leu (trnL) gene and trnL-trnF intergenic spacer, partial sequence; chloroplast	1103	1103	100%	0.0	97%	DQ667492.1	1069,91	88,2%
Select seq emb|AJ505541.1|	Mentha suaveolens plastid trnL-trnF intergenic spacer, specimen voucher cult., K-1970-3169 (K)	1079	1079	98%	0.0	97%	AJ505541.1	1067,99	88,0%
Select seq gb|KC414276.1|	Mentha canadensis isolate 511190001 tRNA-Leu (trnL) gene and trnL-trnF intergenic spacer, partial sequence; chloroplast	1101	1101	100%	0.0	97%	KC414276.1	1067,97	88,0%
Select seq gb|KR150240.1|	Satureja sp. 2 Kh70 trnL-trnF intergenic spacer region, partial sequence; chloroplast	1066	1066	97%	0.0	96%	KR150240.1	1055,01	86,9%

Select seq gb|FJ593456.1|	Mentha sp. JSZ-2009a tRNA-Leu (trnL) gene and trnL-trnF intergenic spacer, partial sequence; chloroplast	1094	1094	100%	0.0	96%	FJ593456.1	1050,24	86,5%
Select seq gb|DQ667514.1|	Mentha arvensis isolate x424 tRNA-Leu (trnL) gene and trnL-trnF intergenic spacer, partial sequence; chloroplast	1081	1081	99%	0.0	96%	DQ667514.1	1048,24	86,4%
Select seq gb|DQ667513.1|	Clinopodium vulgare isolate x423 tRNA-Leu (trnL) gene and trnL-trnF intergenic spacer, partial sequence; chloroplast	1081	1081	100%	0.0	96%	DQ667513.1	1037,76	85,5%
Select seq gb|DQ667504.1|	Cunila incana isolate x296 tRNA-Leu (trnL) gene and trnL-trnF intergenic spacer, partial sequence; chloroplast	1075	1075	100%	0.0	96%	DQ667504.1	1032,00	85,0%
Select seq gb|DQ667507.1|	Pycnanthemum virginianum isolate x313 tRNA-Leu (trnL) gene and trnL-trnF intergenic spacer, partial sequence; chloroplast	1070	1070	100%	0.0	96%	DQ667507.1	1027,20	84,6%
Select seq gb|DQ667497.1|	Hoehnea epilobioides isolate x258 tRNA-Leu (trnL) gene and trnL-trnF intergenic spacer, partial sequence; chloroplast	1070	1070	100%	0.0	96%	DQ667497.1	1027,20	84,6%
Select seq gb|AY570459.1|	Lepechinia chamaedryoides voucher JBW 2537 tRNA-Leu and trnL-trnF intergenic spacer, partial sequence; chloroplast	1070	1070	100%	0.0	96%	AY570459.1	1027,20	84,6%
								0,00	0,0%
								0,00	0,0%
								0,00	0,0%
								0,00	0,0%
								0,00	0,0%
								0,00	0,0%
								0,00	0,0%
								0,00	0,0%
								0,00	0,0%
								0,00	0,0%

Select for downloading or viewing reports	Kh009_ITS Description	Max score	Total score	Query cover	E value	Ident	Accession	(Ident/Cover)* Max score	Deviation from top hit
Select seq gb|GU166769.1|	Alcea striata isolate Istr01 internal transcribed spacer 1, partial sequence; 5.8S ribosomal RNA gene, complete sequence; and internal transcribed spacer 2, partial sequence	217	217	17%	3,00E-52	98%	GU166769.1	0,00	#DIV/0!
Select seq gb|GU166768.1|	Alcea rufescens isolate Iruf01 internal transcribed spacer 1, partial sequence; 5.8S ribosomal RNA gene, complete sequence; and internal transcribed spacer 2, partial sequence	217	217	17%	3,00E-52	98%	GU166768.1	0,00	#DIV/0!
Select seq gb|GU166765.1|	Alcea lavateriflora isolate Ilav02 internal transcribed spacer 1, partial sequence; 5.8S ribosomal RNA gene, complete sequence; and internal transcribed spacer 2, partial sequence	217	217	17%	3,00E-52	98%	GU166765.1	0,00	#DIV/0!
Select seq gb|GU166764.1|	Alcea lavateriflora isolate Ilav01 internal transcribed spacer 1, partial sequence; 5.8S ribosomal RNA gene, complete sequence; and internal transcribed spacer 2, partial sequence	217	217	17%	3,00E-52	98%	GU166764.1	0,00	#DIV/0!
Select seq gb|GU166761.1|	Alcea heldreichii isolate Ihel03 internal transcribed spacer 1, partial sequence; 5.8S ribosomal RNA gene, complete sequence; and internal transcribed spacer 2, partial sequence	217	217	17%	3,00E-52	98%	GU166761.1	0,00	#DIV/0!
Select seq gb|GU166758.1|	Alcea glabrata isolate Igla01 internal transcribed spacer 1, partial sequence; 5.8S ribosomal RNA gene, complete sequence; and internal transcribed spacer 2, partial sequence	217	217	17%	3,00E-52	98%	GU166758.1	0,00	#DIV/0!
Select seq gb|GU166755.1|	Alcea apterocarpa isolate Iapt01 internal transcribed spacer 1, partial sequence; 5.8S ribosomal RNA gene, complete sequence; and internal transcribed spacer 2, partial sequence	217	217	17%	3,00E-52	98%	GU166755.1	0,00	#DIV/0!
Select seq gb|EF679729.1|	Alcea sulphurea voucher PE610 internal transcribed spacer 1, partial sequence; 5.8S ribosomal RNA gene, complete sequence; and internal transcribed spacer 2, partial sequence	217	217	17%	3,00E-52	98%	EF679729.1	0,00	#DIV/0!
Select seq gb|EF679728.1|	Alcea schirazana voucher PE618 internal transcribed spacer 1, partial sequence; 5.8S ribosomal RNA gene, complete sequence; and internal transcribed spacer 2, partial sequence	217	217	17%	3,00E-52	98%	EF679728.1	0,00	#DIV/0!
Select seq gb|EF679725.1|	Alcea rechingeri voucher PE621 internal transcribed spacer 1, partial sequence; 5.8S ribosomal RNA gene, complete sequence; and internal transcribed spacer 2, partial sequence	217	217	17%	3,00E-52	98%	EF679725.1	0,00	#DIV/0!
Select seq gb|EF679722.1|	Alcea koelzii voucher PE609 internal transcribed spacer 1, partial sequence; 5.8S ribosomal RNA gene, complete sequence; and internal transcribed spacer 2, partial sequence	217	217	17%	3,00E-52	98%	EF679722.1	0,00	#DIV/0!
Select seq gb|EF679716.1|	Alcea arbelensis voucher PE624 internal transcribed spacer 1, partial sequence; 5.8S ribosomal RNA gene, complete sequence; and internal transcribed spacer 2, partial sequence	217	217	17%	3,00E-52	98%	EF679716.1	0,00	#DIV/0!
Select seq gb|EF679715.1|	Alcea pallida voucher PE140 internal transcribed spacer 1, partial sequence; 5.8S ribosomal RNA gene, complete sequence; and internal transcribed spacer 2, partial sequence	217	217	17%	3,00E-52	98%	EF679715.1	0,00	#DIV/0!
Select seq emb|AJ251164.1|	Alcea rugosa internal transcribed spacer 1 (ITS1)	126	126	10%	5,00E-25	97%	AJ251164.1	0,00	#DIV/0!
Select seq gb|GU166762.1|	Alcea hohenackeri isolate Ihoh01 internal transcribed spacer 1, partial sequence; 5.8S ribosomal RNA gene, complete sequence; and internal transcribed spacer 2, partial sequence	213	213	17%	4,00E-51	97%	GU166762.1	0,00	#DIV/0!
Select seq gb|GU166760.1|	Alcea heldreichii isolate Ihel01 internal transcribed spacer 1, partial sequence; 5.8S ribosomal RNA gene, complete sequence; and internal transcribed spacer 2, partial sequence	213	213	17%	4,00E-51	97%	GU166760.1	0,00	#DIV/0!
Select seq gb|GU166754.1|	Alcea angulata isolate Iang01 internal transcribed spacer 1, partial sequence; 5.8S ribosomal RNA gene, complete sequence; and internal transcribed spacer 2, partial sequence	213	213	17%	4,00E-51	97%	GU166754.1	0,00	#DIV/0!
Select seq gb|AF303022.1|AF303022S1	Alcea rosea internal transcribed spacer 1 and 5.8S ribosomal RNA gene, partial sequence	174	174	14%	2,00E-39	97%	AF303022.1	0,00	#DIV/0!
Select seq gb|GU166770.1|	Alcea acaulis isolate Iaca01 internal transcribed spacer 1, partial sequence; 5.8S ribosomal RNA gene, complete sequence; and internal transcribed spacer 2, partial sequence	211	211	17%	1,00E-50	97%	GU166770.1	0,00	#DIV/0!
Select seq gb|GU166767.1|	Alcea peduncularis isolate Iped01 internal transcribed spacer 1, partial sequence; 5.8S ribosomal RNA gene, complete sequence; and internal transcribed spacer 2, partial sequence	211	211	17%	1,00E-50	97%	GU166767.1	0,00	#DIV/0!
Select seq gb|GU166763.1|	Alcea hohenackeri isolate Ihoh02 internal transcribed spacer 1, partial sequence; 5.8S ribosomal RNA gene, complete sequence; and internal transcribed spacer 2, partial sequence	211	211	17%	1,00E-50	97%	GU166763.1	0,00	#DIV/0!
Select seq gb|GU166759.1|	Alcea glabrata isolate Igla03 internal transcribed spacer 1, partial sequence; 5.8S ribosomal RNA gene, complete sequence; and internal transcribed spacer 2, partial sequence	211	211	17%	1,00E-50	97%	GU166759.1	0,00	#DIV/0!
Select seq gb|GU166756.1|	Alcea apterocarpa isolate Iapt02 internal transcribed spacer 1, partial sequence; 5.8S ribosomal RNA gene, complete sequence; and internal transcribed spacer 2, partial sequence	211	211	17%	1,00E-50	97%	GU166756.1	0,00	#DIV/0!
Select seq gb|EF679727.1|	Alcea setosa voucher PE424 internal transcribed spacer 1, partial sequence; 5.8S ribosomal RNA gene, complete sequence; and internal transcribed spacer 2, partial sequence	211	211	17%	1,00E-50	97%	EF679727.1	0,00	#DIV/0!
Select seq gb|EF679726.1|	Alcea sachsachanica voucher PE620 internal transcribed spacer 1, partial sequence; 5.8S ribosomal RNA gene, complete sequence; and internal transcribed spacer 2, partial sequence	211	211	17%	1,00E-50	97%	EF679726.1	0,00	#DIV/0!

Select seq gb|EF679720.1|	Alcea gorganica voucher PE611 internal transcribed spacer 1, partial sequence; 5.8S ribosomal RNA gene, complete sequence; and internal transcribed spacer 2, partial sequence	211	211	17%	1,00E-50	97% EF679720.1	0,00	#DIV/0!
Select seq gb|EF679714.1|	Alcea rosea voucher PE422 internal transcribed spacer 1, partial sequence; 5.8S ribosomal RNA gene, complete sequence; and internal transcribed spacer 2, partial sequence	211	211	17%	1,00E-50	97% EF679714.1	0,00	#DIV/0!
Select seq gb|EF419545.1|	Alcea pallida isolate IAsp0140 internal transcribed spacer 1, partial sequence; 5.8S ribosomal RNA gene, complete sequence; and internal transcribed spacer 2, partial sequence	193	193	16%	5,00E-45	97% EF419545.1	0,00	#DIV/0!
Select seq gb|EF419543.1|	Alcea angulata isolate Iang615 internal transcribed spacer 1, partial sequence; 5.8S ribosomal RNA gene, complete sequence; and internal transcribed spacer 2, partial sequence	193	193	16%	5,00E-45	97% EF419543.1	0,00	#DIV/0!
Select seq gb|EF679721.1|	Alcea glabrata voucher PE607 internal transcribed spacer 1, partial sequence; 5.8S ribosomal RNA gene, complete sequence; and internal transcribed spacer 2, partial sequence	206	206	17%	7,00E-49	96% EF679721.1	0,00	#DIV/0!
Select seq gb|EF679718.1|	Alcea excubita voucher PE417 internal transcribed spacer 1, partial sequence; 5.8S ribosomal RNA gene, complete sequence; and internal transcribed spacer 2, partial sequence	206	206	17%	7,00E-49	96% EF679718.1	0,00	#DIV/0!
Select seq gb|JX017319.1|	Althaea rosea voucher 01 internal transcribed spacer 1, partial sequence; 5.8S ribosomal RNA and internal transcribed spacer 2, complete sequence; and 26S ribosomal RNA, partial sequence	193	193	16%	5,00E-45	96% JX017319.1	0,00	#DIV/0!
Select seq gb|EF679717.1|	Alcea aucheri voucher PE617 internal transcribed spacer 1, partial sequence; 5.8S ribosomal RNA gene, complete sequence; and internal transcribed spacer 2, partial sequence	204	204	17%	2,00E-48	96% EF679717.1	0,00	#DIV/0!
Select seq gb|EF419544.1|	Alcea rosea isolate Iros422 internal transcribed spacer 1, partial sequence; 5.8S ribosomal RNA gene, complete sequence; and internal transcribed spacer 2, partial sequence	187	187	16%	2,00E-43	96% EF419544.1	0,00	#DIV/0!
Select seq emb|AJ274972.1|	Kitaibela vitifolia internal transcribed spacer 1 (ITS1)	141	141	12%	2,00E-29	95% AJ274972.1	0,00	#DIV/0!
Select seq gb|AF303026.1|	Althaea officinalis internal transcribed spacer 1, partial sequence; 5.8S ribosomal RNA gene, complete sequence; and internal transcribed spacer 2, partial sequence	163	163	14%	4,00E-36	95% AF303026.1	0,00	#DIV/0!
Select seq gb|FJ527607.1|	Hibiscus cannabinus 18S ribosomal RNA gene, partial sequence; internal transcribed spacer 1, 5.8S ribosomal RNA gene, and internal transcribed spacer 2, complete sequence; and 28S ribosomal RNA gene, partial sequence	128	128	11%	2,00E-25	94% FJ527607.1	0,00	#DIV/0!
Select seq gb|EF419540.1|	Althaea cannabina isolate IAca0345 internal transcribed spacer 1, partial sequence; 5.8S ribosomal RNA gene, complete sequence; and internal transcribed spacer 2, partial sequence	176	176	16%	5,00E-40	95% EF419540.1	0,00	#DIV/0!
Select seq gb|EF419539.1|	Althaea cannabina isolate IAca594 internal transcribed spacer 1, partial sequence; 5.8S ribosomal RNA gene, complete sequence; and internal transcribed spacer 2, partial sequence	176	176	16%	5,00E-40	95% EF419539.1	0,00	#DIV/0!
Select seq gb|EF679733.1|	Althaea officinalis voucher PE512 internal transcribed spacer 1, partial sequence; 5.8S ribosomal RNA gene, complete sequence; and internal transcribed spacer 2, partial sequence	187	187	17%	2,00E-43	94% EF679733.1	0,00	#DIV/0!
Select seq gb|DQ006018.1|	Sida spinosa voucher Wiser 45 US 18S ribosomal RNA gene, partial sequence; internal transcribed spacer 1, 5.8S ribosomal RNA gene, and internal transcribed spacer 2, complete sequence; and 28S ribosomal RNA gene, partial sequence	130	130	12%	4,00E-26	94% DQ006018.1	0,00	#DIV/0!
Select seq gb|EF419537.1|	Althaea officinalis isolate IAof0330 internal transcribed spacer 1, partial sequence; 5.8S ribosomal RNA gene, complete sequence; and internal transcribed spacer 2, partial sequence	171	171	16%	2,00E-38	94% EF419537.1	0,00	#DIV/0!
Select seq gb|EF419536.1|	Althaea officinalis isolate IAof604 internal transcribed spacer 1, partial sequence; 5.8S ribosomal RNA gene, complete sequence; and internal transcribed spacer 2, partial sequence	171	171	16%	2,00E-38	94% EF419536.1	0,00	#DIV/0!
Select seq gb|EF419510.1|	Althaea hirsuta isolate IAhi0270 internal transcribed spacer 1, partial sequence; 5.8S ribosomal RNA gene, complete sequence; and internal transcribed spacer 2, partial sequence	132	132	13%	1,00E-26	92% EF419510.1	0,00	#DIV/0!
Select seq gb|EF419509.1|	Althaea hirsuta isolate IAhi0356 internal transcribed spacer 1, partial sequence; 5.8S ribosomal RNA gene, complete sequence; and internal transcribed spacer 2, partial sequence	132	132	13%	1,00E-26	92% EF419509.1	0,00	#DIV/0!
Select seq gb|EF419508.1|	Althaea hirsuta isolate IAhi454 internal transcribed spacer 1, partial sequence; 5.8S ribosomal RNA gene, complete sequence; and internal transcribed spacer 2, partial sequence	132	132	13%	1,00E-26	92% EF419508.1	0,00	#DIV/0!
Select seq gb|EF419547.1|	Anisodontea malvastroides internal transcribed spacer 1, partial sequence; 5.8S ribosomal RNA gene, complete sequence; and internal transcribed spacer 2, partial sequence	141	141	14%	2,00E-29	91% EF419547.1	0,00	#DIV/0!
Select seq gb|AF303024.1|AF303024S1	Anisodontea capensis internal transcribed spacer 1 and 5.8S ribosomal RNA gene, partial sequence	141	141	14%	2,00E-29	91% AF303024.1	0,00	#DIV/0!
Select seq gb|EF419526.1|	Lavatera phoenicea isolate ILph0002 internal transcribed spacer 1, partial sequence; 5.8S ribosomal RNA gene, complete sequence; and internal transcribed spacer 2, partial sequence	148	148	16%	1,00E-31	90% EF419526.1	0,00	#DIV/0!
Select seq gb|EF679732.1|	Malope trifida voucher PE601 internal transcribed spacer 1, partial sequence; 5.8S ribosomal RNA gene, complete sequence; and internal transcribed spacer 2, partial sequence	147	147	17%	4,00E-31	88% EF679732.1	0,00	#DIV/0!
Select seq gb|EF419507.1|	Althaea hirsuta isolate IAhi455 internal transcribed spacer 1, partial sequence; 5.8S ribosomal RNA gene, complete sequence; and internal transcribed spacer 2, partial sequence	135	135	16%	9,00E-28	88% EF419507.1	0,00	#DIV/0!
Select seq gb|EF419502.1|	Althaea longiflora isolate IAlo461 internal transcribed spacer 1, partial sequence; 5.8S ribosomal RNA gene, complete sequence; and internal transcribed spacer 2, partial sequence	135	135	16%	9,00E-28	88% EF419502.1	0,00	#DIV/0!

Select seq gb|EF419501.1|	Althaea longiflora isolate IAlo596 internal transcribed spacer 1, partial sequence; 5.8S ribosomal RNA gene, complete sequence; and internal transcribed spacer 2, partial sequence	135	135	16%	9,00E-28	88% EF419501.1	0,00	#DIV/0!
Select seq gb|EF419500.1|	Althaea longiflora isolate IAlo0362 internal transcribed spacer 1, partial sequence; 5.8S ribosomal RNA gene, complete sequence; and internal transcribed spacer 2, partial sequence	135	135	16%	9,00E-28	88% EF419500.1	0,00	#DIV/0!
Select seq gb|KR734289.1|	Pavonia sp. TRK-2015 18S ribosomal RNA gene, partial sequence; internal transcribed spacer 1, complete sequence; and 5.8S ribosomal RNA gene, partial sequence	134	134	16%	3,00E-27	87% KR734289.1	0,00	#DIV/0!
Select seq gb|KR734281.1|	Sida sp. TRK-2015 18S ribosomal RNA gene, partial sequence; internal transcribed spacer 1, complete sequence; and 5.8S ribosomal RNA gene, partial sequence	134	134	16%	3,00E-27	87% KR734281.1	0,00	#DIV/0!
Select seq gb|KR734272.1|	Sida sp. TRK-2015 18S ribosomal RNA gene, partial sequence; internal transcribed spacer 1, complete sequence; and 5.8S ribosomal RNA gene, partial sequence	134	134	16%	3,00E-27	87% KR734272.1	0,00	#DIV/0!
Select seq gb|KR734256.1|	Pavonia sp. TRK-2015 18S ribosomal RNA gene, partial sequence; internal transcribed spacer 1, complete sequence; and 5.8S ribosomal RNA gene, partial sequence	134	134	16%	3,00E-27	87% KR734256.1	0,00	
Select seq gb|KR734097.1|	Pavonia sp. TRK-2015 18S ribosomal RNA gene, partial sequence; internal transcribed spacer 1, complete sequence; and 5.8S ribosomal RNA gene, partial sequence	134	134	16%	3,00E-27	87% KR734097.1	0,00	
Select seq gb|KR733990.1|	Abutilon sp. TRK-2015 18S ribosomal RNA gene, partial sequence; internal transcribed spacer 1, complete sequence; and 5.8S ribosomal RNA gene, partial sequence	134	134	16%	3,00E-27	87% KR733990.1	0,00	
Select seq gb|KR733981.1|	Sida sp. TRK-2015 18S ribosomal RNA gene, partial sequence; internal transcribed spacer 1, complete sequence; and 5.8S ribosomal RNA gene, partial sequence	134	134	16%	3,00E-27	87% KR733981.1	0,00	
Select seq gb|KR733929.1|	Abutilon sp. TRK-2015 18S ribosomal RNA gene, partial sequence; internal transcribed spacer 1, complete sequence; and 5.8S ribosomal RNA gene, partial sequence	134	134	16%	3,00E-27	87% KR733929.1	0,00	
Select seq gb|KR733896.1|	Sida sp. TRK-2015 18S ribosomal RNA gene, partial sequence; internal transcribed spacer 1, complete sequence; and 5.8S ribosomal RNA gene, partial sequence	134	134	16%	3,00E-27	87% KR733896.1	0,00	
Select seq gb|KR733809.1|	Pavonia sp. TRK-2015 18S ribosomal RNA gene, partial sequence; internal transcribed spacer 1, complete sequence; and 5.8S ribosomal RNA gene, partial sequence	134	134	16%	3,00E-27	87% KR733809.1	0,00	
Select seq gb|KR733710.1|	Abutilon sp. TRK-2015 18S ribosomal RNA gene, partial sequence; internal transcribed spacer 1, complete sequence; and 5.8S ribosomal RNA gene, partial sequence	134	134	16%	3,00E-27	87% KR733710.1	0,00	
Select seq gb|EF419459.1|	Malva canariensis isolate ILac0135 internal transcribed spacer 1, partial sequence; 5.8S ribosomal RNA gene, complete sequence; and internal transcribed spacer 2, partial sequence	132	132	16%	1,00E-26	88% EF419459.1	0,00	
Select seq gb|KP092989.1|	Abutilon indicum isolate SCBGP200_1 18S ribosomal RNA gene, partial sequence; internal transcribed spacer 1, 5.8S ribosomal RNA gene, and internal transcribed spacer 2, complete sequence; and 28S ribosomal RNA gene, partial sequence	141	141	17%	2,00E-29	87% KP092989.1	0,00	
Select seq gb|DQ287984.1|	Abutilon theophrasti internal transcribed spacer 1, partial sequence; 5.8S ribosomal RNA gene, complete sequence; and internal transcribed spacer 2, partial sequence	141	141	17%	2,00E-29	87% DQ287984.1	0,00	
Select seq gb|DQ006017.1|	Abutilon theophrasti voucher Wells 5165 US 18S ribosomal RNA gene, partial sequence; internal transcribed spacer 1, 5.8S ribosomal RNA gene, and internal transcribed spacer 2, complete sequence; and 28S ribosomal RNA gene, partial sequence	141	141	17%	2,00E-29	87% DQ006017.1	0,00	
Select seq gb|KJ999382.1|	Malva pusilla voucher JK1 18S ribosomal RNA gene, partial sequence; internal transcribed spacer 1, 5.8S ribosomal RNA gene, and internal transcribed spacer 2, complete sequence; and 28S ribosomal RNA gene, partial sequence	132	132	16%	1,00E-26	87% KJ999382.1	0,00	
Select seq gb|EF419535.1|	Malope malacoides isolate IMma415 internal transcribed spacer 1, partial sequence; 5.8S ribosomal RNA gene, complete sequence; and internal transcribed spacer 2, partial sequence	130	130	16%	4,00E-26	88% EF419535.1	0,00	
Select seq gb|EF419534.1|	Malope malacoides isolate IMma600 internal transcribed spacer 1, partial sequence; 5.8S ribosomal RNA gene, complete sequence; and internal transcribed spacer 2, partial sequence	130	130	16%	4,00E-26	88% EF419534.1	0,00	
Select seq gb|EF419532.1|	Malope trifida isolate IMtf070 internal transcribed spacer 1, partial sequence; 5.8S ribosomal RNA gene, complete sequence; and internal transcribed spacer 2, partial sequence	130	130	16%	4,00E-26	88% EF419532.1	0,00	
Select seq gb|EF419529.1|	Malope trifida isolate IMtf394 internal transcribed spacer 1, partial sequence; 5.8S ribosomal RNA gene, complete sequence; and internal transcribed spacer 2, partial sequence	130	130	16%	4,00E-26	88% EF419529.1	0,00	
Select seq gb|HM116982.1|	Hibiscus heterophyllus internal transcribed spacer 1, partial sequence; and 5.8S ribosomal RNA gene and internal transcribed spacer 2, complete sequence	130	130	16%	4,00E-26	87% HM116982.1	0,00	
Select seq gb|EF419506.1|	Althaea ludwigii isolate IAlu616 internal transcribed spacer 1, partial sequence; 5.8S ribosomal RNA gene, complete sequence; and internal transcribed spacer 2, partial sequence	130	130	16%	4,00E-26	87% EF419506.1	0,00	
Select seq gb|EF419505.1|	Althaea ludwigii isolate IAlu460 internal transcribed spacer 1, partial sequence; 5.8S ribosomal RNA gene, complete sequence; and internal transcribed spacer 2, partial sequence	130	130	16%	4,00E-26	87% EF419505.1	0,00	
Select seq gb|EF419504.1|	Althaea ludwigii isolate IAlu459 internal transcribed spacer 1, partial sequence; 5.8S ribosomal RNA gene, complete sequence; and internal transcribed spacer 2, partial sequence	130	130	16%	4,00E-26	87% EF419504.1	0,00	
Select seq gb|EF660543.1|	Plagianthus regius var. chathamicus 18S ribosomal RNA gene, partial sequence; internal transcribed spacer 1, 5.8S ribosomal RNA gene, and internal transcribed spacer 2, complete sequence; and 28S ribosomal RNA gene, partial sequence	130	130	16%	4,00E-26	87% EF660543.1	0,00	

Select seq gb|EF419451.1|	Lavatera thuringiaca isolate ILth559 internal transcribed spacer 1, partial sequence; 5.8S ribosomal RNA gene, complete sequence; and internal transcribed spacer 2, partial sequence	128	128	16%	2,00E-25	87%	EF419451.1	0,00
Select seq gb|EF419430.1|	Lavatera triloba subsp. agrigentina isolate ILag308 internal transcribed spacer 1, partial sequence; 5.8S ribosomal RNA gene, complete sequence; and internal transcribed spacer 2, partial sequence	128	128	16%	2,00E-25	87%	EF419430.1	0,00
Select seq gb|EF419458.1|	Malva wigandii isolate ILma404 internal transcribed spacer 1, partial sequence; 5.8S ribosomal RNA gene, complete sequence; and internal transcribed spacer 2, partial sequence	126	126	16%	5,00E-25	87%	EF419458.1	0,00
Select seq gb|EF419457.1|	Malva wigandii isolate ILma0200 internal transcribed spacer 1, partial sequence; 5.8S ribosomal RNA gene, complete sequence; and internal transcribed spacer 2, partial sequence	126	126	16%	5,00E-25	87%	EF419457.1	0,00
Select seq gb|EF419456.1|	Malva wigandii isolate ILma0329 internal transcribed spacer 1, partial sequence; 5.8S ribosomal RNA gene, complete sequence; and internal transcribed spacer 2, partial sequence	126	126	16%	5,00E-25	87%	EF419456.1	0,00
Select seq gb|EF419455.1|	Malva wigandii isolate ILma598 internal transcribed spacer 1, partial sequence; 5.8S ribosomal RNA gene, complete sequence; and internal transcribed spacer 2, partial sequence	126	126	16%	5,00E-25	87%	EF419455.1	0,00
Select seq gb|KC952014.1|	Sida acuta 18S ribosomal RNA gene, partial sequence; internal transcribed spacer 1, 5.8S ribosomal RNA gene, and internal transcribed spacer 2, complete sequence; and 28S ribosomal RNA gene, partial sequence	135	135	17%	9,00E-28	86%	KC952014.1	0,00
Select seq gb|JN542429.1|	Sida acuta strain 13009 18S ribosomal RNA gene, partial sequence; internal transcribed spacer 1, 5.8S ribosomal RNA gene, and internal transcribed spacer 2, complete sequence; and 28S ribosomal RNA gene, partial sequence	135	135	17%	9,00E-28	86%	JN542429.1	0,00
Select seq gb|JN542432.1|	Sida cordata strain 13013 18S ribosomal RNA gene, partial sequence; internal transcribed spacer 1, 5.8S ribosomal RNA gene, and internal transcribed spacer 2, complete sequence; and 28S ribosomal RNA gene, partial sequence	134	134	17%	3,00E-27	86%	JN542432.1	0,00
Select seq gb|JN542431.1|	Sida cordifolia strain 13014 18S ribosomal RNA gene, partial sequence; internal transcribed spacer 1, 5.8S ribosomal RNA gene, and internal transcribed spacer 2, complete sequence; and 28S ribosomal RNA gene, partial sequence	134	134	17%	3,00E-27	86%	JN542431.1	0,00
Select seq gb|JN542430.1|	Sida spinosa strain 13012 18S ribosomal RNA gene, partial sequence; internal transcribed spacer 1, 5.8S ribosomal RNA gene, and internal transcribed spacer 2, complete sequence; and 28S ribosomal RNA gene, partial sequence	134	134	17%	3,00E-27	86%	JN542430.1	0,00
Select seq gb|KR734283.1|	Malva parviflora 18S ribosomal RNA gene, partial sequence; internal transcribed spacer 1, complete sequence; and 5.8S ribosomal RNA gene, partial sequence	126	126	16%	5,00E-25	86%	KR734283.1	0,00
Select seq gb|KR734025.1|	Malva parviflora 18S ribosomal RNA gene, partial sequence; internal transcribed spacer 1, complete sequence; and 5.8S ribosomal RNA gene, partial sequence	126	126	16%	5,00E-25	86%	KR734025.1	0,00
Select seq gb|KR733908.1|	Malva parviflora 18S ribosomal RNA gene, partial sequence; internal transcribed spacer 1, complete sequence; and 5.8S ribosomal RNA gene, partial sequence	126	126	16%	5,00E-25	86%	KR733908.1	0,00
Select seq gb|KJ999384.1|	Malva pusilla voucher JK2 18S ribosomal RNA gene, partial sequence; internal transcribed spacer 1, 5.8S ribosomal RNA gene, and internal transcribed spacer 2, complete sequence; and 28S ribosomal RNA gene, partial sequence	126	126	16%	5,00E-25	86%	KJ999384.1	0,00
Select seq gb|FJ527608.1|	Hibiscus sabdariffa var. sabdariffa 18S ribosomal RNA gene, partial sequence; internal transcribed spacer 1, 5.8S ribosomal RNA gene, and internal transcribed spacer 2, complete sequence; and 28S ribosomal RNA gene, partial sequence	124	124	16%	2,00E-24	86%	FJ527608.1	0,00
Select seq gb|EF419524.1|	Malva trifida isolate IMtr0352 internal transcribed spacer 1, partial sequence; 5.8S ribosomal RNA gene, complete sequence; and internal transcribed spacer 2, partial sequence	124	124	16%	2,00E-24	86%	EF419524.1	0,00
Select seq gb|EF635464.1|	Hoheria populnea 18S ribosomal RNA gene, partial sequence; internal transcribed spacer 1, 5.8S ribosomal RNA gene, and internal transcribed spacer 2, complete sequence; and 28S ribosomal RNA gene, partial sequence	130	130	17%	4,00E-26	85%	EF635464.1	0,00
Select seq gb|EF152583.1|	Cristaria insularis 18S ribosomal RNA gene, partial sequence; internal transcribed spacer 1 and 5.8S ribosomal RNA gene, complete sequence; and internal transcribed spacer 2, partial sequence	128	128	17%	2,00E-25	85%	EF152583.1	0,00
								0,00

Select for downloading or viewing reports	Kh009_trnL Description	Max score	Total score	Query cover	E value	Ident	Accession	(Ident/Cover)* Max score	Deviation from top hit
Select seq gb|EF419700.1|	Malva nicaeensis isolate TMni097 trnL-trnF intergenic spacer, partial sequence; chloroplast	56,5	56.5	4%	6,00E-04	100%	EF419700.1	0,00	#DIV/0!
Select seq gb|EF419699.1|	Malva nicaeensis isolate TMni032 trnL-trnF intergenic spacer, partial sequence; chloroplast	56,5	56.5	4%	6,00E-04	100%	EF419699.1	0,00	#DIV/0!
Select seq gb|JN676078.1|	Sterculia nobilis tRNA-Leu (trnL) gene, partial sequence; trnL-trnF intergenic spacer, complete sequence; and tRNA-Phe (trnF) gene, partial sequence; chloroplast	134	134	14%	3,00E-27	93%	JN676078.1	0,00	#DIV/0!
Select seq gb|AY328152.1|	Sterculia nobilis specimen-voucher Ye 99120501 tRNA-Leu (trnL) gene, partial sequence; trnL-trnF intergenic spacer, complete sequence; and tRNA-Phe (trnF) gene, partial sequence; chloroplast	134	134	14%	3,00E-27	93%	AY328152.1	0,00	#DIV/0!
Select seq gb|AY328151.1|	Sterculia lanceolata specimen-voucher Ye 99120504 tRNA-Leu (trnL) gene, partial sequence; trnL-trnF intergenic spacer, complete sequence; and tRNA-Phe (trnF) gene, partial sequence; chloroplast	134	134	14%	3,00E-27	93%	AY328151.1	0,00	#DIV/0!
Select seq gb|EF679772.1|	Malope trifida voucher PE601 trnL-trnF intergenic spacer and tRNA-Phe (trnF) gene, partial sequence; chloroplast	165	165	18%	1,00E-36	94%	EF679772.1	0,00	#DIV/0!
Select seq gb|EF419757.1|	Malope trifida isolate TMtf601 trnL-trnF intergenic spacer, partial sequence; chloroplast	165	165	18%	1,00E-36	94%	EF419757.1	0,00	#DIV/0!
Select seq gb|EF419761.1|	Malope malacoides isolate TMma600 trnL-trnF intergenic spacer, partial sequence; chloroplast	189	189	25%	6,00E-44	89%	EF419761.1	0,00	#DIV/0!
Select seq gb|EF419727.1|	Althaea officinalis isolate TAof330 trnL-trnF intergenic spacer, partial sequence; chloroplast	189	189	25%	6,00E-44	89%	EF419727.1	0,00	#DIV/0!
Select seq gb|EF679770.1|	Althaea officinalis voucher PE512 trnL-trnF intergenic spacer and tRNA-Phe (trnF) gene, partial sequence; chloroplast	182	182	25%	1,00E-41	88%	EF679770.1	0,00	#DIV/0!
Select seq gb|EF419768.1|	Malvella sherardiana isolate TMsh325 trnL-trnF intergenic spacer, partial sequence; chloroplast	182	182	25%	1,00E-41	88%	EF419768.1	0,00	#DIV/0!
Select seq gb|EF419728.1|	Althaea officinalis isolate TAof604 trnL-trnF intergenic spacer, partial sequence; chloroplast	182	182	25%	1,00E-41	88%	EF419728.1	0,00	#DIV/0!
Select seq gb|EF419760.1|	Malope malacoides isolate TMma415 trnL-trnF intergenic spacer, partial sequence; chloroplast	176	176	25%	5,00E-40	87%	EF419760.1	0,00	#DIV/0!
Select seq gb|EF419763.1|	Lavatera phoenicea isolate TLph002 trnL-trnF intergenic spacer, partial sequence; chloroplast	165	165	25%	1,00E-36	86%	EF419763.1	0,00	#DIV/0!
Select seq gb|EF419744.1|	Malva cretica subsp. althaeoides isolate TMca463 trnL-trnF intergenic spacer, partial sequence; chloroplast	165	165	25%	1,00E-36	86%	EF419744.1	0,00	#DIV/0!
Select seq gb|EF419697.1|	Malva parviflora isolate TMpa296 trnL-trnF intergenic spacer, partial sequence; chloroplast	165	165	25%	1,00E-36	86%	EF419697.1	0,00	#DIV/0!
Select seq gb|EF419687.1|	Malva canariensis isolate TLac134 trnL-trnF intergenic spacer, partial sequence; chloroplast	165	165	25%	1,00E-36	86%	EF419687.1	0,00	#DIV/0!
Select seq gb|EF419665.1|	Lavatera oblongifolia isolate TLob144 trnL-trnF intergenic spacer, partial sequence; chloroplast	165	165	25%	1,00E-36	86%	EF419665.1	0,00	#DIV/0!
Select seq gb|EF419754.1|	Malva tournefortiana isolate TMto479 trnL-trnF intergenic spacer, partial sequence; chloroplast	159	159	25%	5,00E-35	86%	EF419754.1	0,00	#DIV/0!
Select seq gb|EF419747.1|	Malva alcea isolate TMal338 trnL-trnF intergenic spacer, partial sequence; chloroplast	159	159	25%	5,00E-35	86%	EF419747.1	0,00	#DIV/0!
Select seq gb|EF419745.1|	Malva alcea isolate TMal440 trnL-trnF intergenic spacer, partial sequence; chloroplast	159	159	25%	5,00E-35	86%	EF419745.1	0,00	#DIV/0!
Select seq gb|EF419743.1|	Malva cretica subsp. althaeoides isolate TMcc361 trnL-trnF intergenic spacer, partial sequence; chloroplast	159	159	25%	5,00E-35	86%	EF419743.1	0,00	#DIV/0!
Select seq gb|EF419736.1|	Malva trifida isolate TMtr452 trnL-trnF intergenic spacer, partial sequence; chloroplast	159	159	25%	5,00E-35	86%	EF419736.1	0,00	#DIV/0!
Select seq gb|EF419722.1|	Althaea ludwigii isolate TAlu460 trnL-trnF intergenic spacer, partial sequence; chloroplast	159	159	25%	5,00E-35	86%	EF419722.1	0,00	#DIV/0!
Select seq gb|EF419718.1|	Althaea hirsuta isolate TAhi454 trnL-trnF intergenic spacer, partial sequence; chloroplast	159	159	25%	5,00E-35	86%	EF419718.1	0,00	#DIV/0!
Select seq gb|EF419717.1|	Althaea hirsuta isolate TAhi270 trnL-trnF intergenic spacer, partial sequence; chloroplast	159	159	25%	5,00E-35	86%	EF419717.1	0,00	#DIV/0!

Select seq gb|EF419716.1|	Althaea hirsuta isolate TAhi356 trnL-trnF intergenic spacer, partial sequence; chloroplast	159	159	25%	5,00E-35	86%	EF419716.1	0,00	#DIV/0!
Select seq gb|EF419672.1|	Lavatera triloba subsp. pallescens isolate TLtp354 trnL-trnF intergenic spacer, partial sequence; chloroplast	159	159	25%	5,00E-35	86%	EF419672.1	0,00	#DIV/0!
Select seq gb|EF419666.1|	Lavatera bryoniifolia isolate TLbr141 trnL-trnF intergenic spacer, partial sequence; chloroplast	159	159	25%	5,00E-35	86%	EF419666.1	0,00	#DIV/0!
Select seq gb|EF419725.1|	Althaea longiflora isolate TAlo461 trnL-trnF intergenic spacer, partial sequence; chloroplast	158	158	25%	2,00E-34	85%	EF419725.1	0,00	#DIV/0!
Select seq gb|EF419724.1|	Althaea longiflora isolate TAlo596 trnL-trnF intergenic spacer, partial sequence; chloroplast	158	158	25%	2,00E-34	85%	EF419724.1	0,00	#DIV/0!
Select seq gb|EF419758.1|	Malope trifida isolate TMtf070 trnL-trnF intergenic spacer, partial sequence; chloroplast	154	154	25%	2,00E-33	85%	EF419758.1	0,00	#DIV/0!
Select seq gb|EF419714.1|	Malva hispanica isolate TMhi602 trnL-trnF intergenic spacer, partial sequence; chloroplast	154	154	25%	2,00E-33	85%	EF419714.1	0,00	#DIV/0!
Select seq gb|EF419710.1|	Malva wigandii isolate TLma598 trnL-trnF intergenic spacer, partial sequence; chloroplast	154	154	25%	2,00E-33	85%	EF419710.1	0,00	#DIV/0!
Select seq gb|EF419680.1|	Lavatera thuringiaca isolate TLth353 trnL-trnF intergenic spacer, partial sequence; chloroplast	154	154	25%	2,00E-33	85%	EF419680.1	0,00	#DIV/0!
Select seq gb|EF419682.1|	Lavatera maroccana isolate TLmo515 trnL-trnF intergenic spacer, partial sequence; chloroplast	152	152	25%	8,00E-33	85%	EF419682.1	0,00	#DIV/0!
Select seq gb|EF419681.1|	Lavatera maroccana isolate TLmo346 trnL-trnF intergenic spacer, partial sequence; chloroplast	152	152	25%	8,00E-33	85%	EF419681.1	0,00	#DIV/0!
Select seq gb|EF419671.1|	Lavatera triloba subsp. pallescens isolate TLtp564 trnL-trnF intergenic spacer, partial sequence; chloroplast	152	152	25%	8,00E-33	85%	EF419671.1	0,00	#DIV/0!
Select seq gb|EF419664.1|	Lavatera plazzae isolate TLpl285 trnL-trnF intergenic spacer, partial sequence; chloroplast	152	152	25%	8,00E-33	85%	EF419664.1	0,00	#DIV/0!
Select seq gb|EF419746.1|	Malva alcea isolate TMal539 trnL-trnF intergenic spacer, partial sequence; chloroplast	148	148	25%	1,00E-31	84%	EF419746.1	0,00	#DIV/0!
Select seq gb|EF419708.1|	Malva assurgentiflora isolate TLas570 trnL-trnF intergenic spacer, partial sequence; chloroplast	147	147	25%	4,00E-31	84%	EF419708.1	0,00	#DIV/0!
Select seq gb|EF419702.1|	Malva neglecta isolate TMne349 trnL-trnF intergenic spacer, partial sequence; chloroplast	145	145	25%	1,00E-30	84%	EF419702.1	0,00	#DIV/0!
Select seq gb|EF419695.1|	Malva parviflora isolate TMpa249 trnL-trnF intergenic spacer, partial sequence; chloroplast	145	145	25%	1,00E-30	84%	EF419695.1	0,00	#DIV/0!
Select seq gb|EF419694.1|	Malva parviflora isolate TMpa005 trnL-trnF intergenic spacer, partial sequence; chloroplast	145	145	25%	1,00E-30	84%	EF419694.1	0,00	#DIV/0!
Select seq gb|EF419693.1|	Lavatera mauritanica isolate TLmu630 trnL-trnF intergenic spacer, partial sequence; chloroplast	145	145	25%	1,00E-30	84%	EF419693.1	0,00	#DIV/0!
Select seq gb|EF419692.1|	Lavatera mauritanica isolate TLmu319 trnL-trnF intergenic spacer, partial sequence; chloroplast	145	145	25%	1,00E-30	84%	EF419692.1	0,00	#DIV/0!
Select seq gb|EF419691.1|	Lavatera mauritanica isolate TLmu137 trnL-trnF intergenic spacer, partial sequence; chloroplast	145	145	25%	1,00E-30	84%	EF419691.1	0,00	#DIV/0!
Select seq gb|EF419690.1|	Malva linnaei isolate TLcr235 trnL-trnF intergenic spacer, partial sequence; chloroplast	145	145	25%	1,00E-30	84%	EF419690.1	0,00	#DIV/0!
Select seq gb|EF419689.1|	Malva linnaei isolate TLcr599 trnL-trnF intergenic spacer, partial sequence; chloroplast	145	145	25%	1,00E-30	84%	EF419689.1	0,00	#DIV/0!
Select seq gb|EF419688.1|	Malva linnaei isolate TLcr031 trnL-trnF intergenic spacer, partial sequence; chloroplast	145	145	25%	1,00E-30	84%	EF419688.1	0,00	#DIV/0!
Select seq gb|EF679759.1|	Alcea pallida voucher PE140 trnL-trnF intergenic spacer and tRNA-Phe (trnF) gene, partial sequence; chloroplast	206	206	37%	6,00E-49	83%	EF679759.1	0,00	#DIV/0!
Select seq gb|EF679758.1|	Alcea longipedicellata voucher PE612 trnL-trnF intergenic spacer and tRNA-Phe (trnF) gene, partial sequence; chloroplast	206	206	37%	6,00E-49	83%	EF679758.1	0,00	#DIV/0!
Select seq gb|EF679757.1|	Alcea kurdica voucher PE619 trnL-trnF intergenic spacer and tRNA-Phe (trnF) gene, partial sequence; chloroplast	206	206	37%	6,00E-49	83%	EF679757.1	0,00	#DIV/0!
Select seq gb|EF679754.1|	Alcea aucheri voucher PE617 trnL-trnF intergenic spacer and tRNA-Phe (trnF) gene, partial sequence; chloroplast	206	206	37%	6,00E-49	83%	EF679754.1	0,00	#DIV/0!

Select seq gb|EF419766.1|	Alcea rosea isolate Tros422 trnL-trnF intergenic spacer, partial sequence; chloroplast	206	206	37%	6,00E-49	83%	EF419766.1	0,00	#DIV/0!
Select seq gb|EF679766.1|	Alcea schirazana voucher PE618 trnL-trnF intergenic spacer and tRNA-Phe (trnF) gene, partial sequence; chloroplast	200	200	37%	3,00E-47	82%	EF679766.1	0,00	#DIV/0!
Select seq gb|EF679756.1|	Alcea koelzii voucher PE609 trnL-trnF intergenic spacer and tRNA-Phe (trnF) gene, partial sequence; chloroplast	195	195	37%	1,00E-45	82%	EF679756.1	0,00	#DIV/0!

Select for downloading or viewing reports	Kh010_trnL Description	Max score	Total score	Query cover	E value	Ident	Accession	(Ident/Cover)* Max score	Deviation from top hit
Select seq gb|JQ669045.1|	Hymenocrater bituminosus voucher K. Tamanyan & George Fayvush tRNA-Leu (trnL) gene and trnL-trnF intergenic spacer, partial sequence; plastid	1197	1197	98%	0.0	99%	JQ669045.1	1209,21	100,0%
Select seq gb|JQ669049.1|	Marmoritis complanatum voucher D.E. Boufford et al., 32012 tRNA-Leu (trnL) gene and trnL-trnF intergenic spacer, partial sequence; plastid	1149	1149	96%	0.0	99%	JQ669049.1	1184,91	98,0%
Select seq gb|KM886627.1|	Marmoritis complanata voucher T. Deng 2359 (KUN) tRNA-Leu (trnL) gene, partial sequence; trnL-trnF intergenic spacer, complete sequence; and tRNA-Phe (trnF) gene, partial sequence; plastid	1140	1140	96%	0.0	99%	KM886627.1	1175,63	97,2%
Select seq emb|AJ505431.1|	Nepeta menthoides plastid trnL-trnF intergenic spacer, specimen voucher Jamzad s.n. (K)	1092	1092	99%	0.0	97%	AJ505431.1	1069,94	88,5%
Select seq gb|KF307433.1|	Lepechinia urbanii voucher B. Drew 135 tRNA-Leu (trnL) gene and trnL-trnF intergenic spacer, partial sequence; chloroplast	1031	1031	96%	0.0	96%	KF307433.1	1031,00	85,3%
Select seq gb|FJ593457.1|	Nepeta stewartiana tRNA-Leu (trnL) gene and trnL-trnF intergenic spacer, partial sequence; chloroplast	1070	1070	100%	0.0	96%	FJ593457.1	1027,20	84,9%
Select seq gb|DQ667492.1|	Lepechinia lancifolia isolate x232 tRNA-Leu (trnL) gene and trnL-trnF intergenic spacer, partial sequence; chloroplast	1064	1064	100%	0.0	96%	DQ667492.1	1021,44	84,5%
Select seq gb|JF301382.1|	Lepechinia radula voucher B. Drew 185 tRNA-Leu (trnL) gene and trnL-trnF intergenic spacer, partial sequence; chloroplast	1031	1031	97%	0.0	96%	JF301382.1	1020,37	84,4%
Select seq gb|KF307430.1|	Lepechinia schiedeana voucher B. Drew 157 tRNA-Leu (trnL) gene and trnL-trnF intergenic spacer, partial sequence; chloroplast	1020	1020	96%	0.0	96%	KF307430.1	1020,00	84,4%
Select seq gb|KF307411.1|	Lepechinia bella voucher Rachel Jabaily s.n. tRNA-Leu (trnL) gene and trnL-trnF intergenic spacer, partial sequence; chloroplast	1020	1020	96%	0.0	96%	KF307411.1	1020,00	84,4%
Select seq gb|JF301383.1|	Lepechinia salviifolia voucher R. Jabaily s.n. tRNA-Leu (trnL) gene and trnL-trnF intergenic spacer, partial sequence; chloroplast	1029	1029	97%	0.0	96%	JF301383.1	1018,39	84,2%
Select seq gb|JF301361.1|	Chaunostoma mecistandrum voucher J.A. Monterrosa & R.A. Carballo 213 tRNA-Leu (trnL) gene and trnL-trnF intergenic spacer, partial sequence; chloroplast	1026	1026	97%	0.0	96%	JF301361.1	1015,42	84,0%
Select seq gb|JF301386.1|	Melissa officinalis voucher B. Drew 70 tRNA-Leu (trnL) gene and trnL-trnF intergenic spacer, partial sequence; chloroplast	1018	1018	97%	0.0	96%	JF301386.1	1007,51	83,3%
Select seq gb|DQ667477.1|	Melissa officinalis isolate x193 tRNA-Leu (trnL) gene and trnL-trnF intergenic spacer, partial sequence; chloroplast	1046	1046	100%	0.0	96%	DQ667477.1	1004,16	83,0%
Select seq emb|AJ505529.1|	Melissa officinalis plastid trnL-trnF intergenic spacer, specimen voucher Catino	1046	1046	100%	0.0	96%	AJ505529.1	1004,16	83,0%
Select seq gb|DQ667487.1|	Nepeta cataria isolate x220 tRNA-Leu (trnL) gene and trnL-trnF intergenic spacer, partial sequence; chloroplast	1057	1057	100%	0.0	95%	DQ667487.1	1004,15	83,0%
Select seq gb|KF307435.1|	Lepechinia yecorana voucher Henrickson 24691 tRNA-Leu (trnL) gene and trnL-trnF intergenic spacer, partial sequence; chloroplast	1014	1014	96%	0.0	95%	KF307435.1	1003,44	83,0%
Select seq gb|KF307432.1|	Lepechinia speciosa voucher Cordeno 3060 tRNA-Leu (trnL) gene and trnL-trnF intergenic spacer, partial sequence; chloroplast	1014	1014	96%	0.0	95%	KF307432.1	1003,44	83,0%
Select seq gb|KF307421.1|	Lepechinia heteromorpha voucher B. Drew 192 tRNA-Leu (trnL) gene and trnL-trnF intergenic spacer, partial sequence; chloroplast	1014	1014	96%	0.0	95%	KF307421.1	1003,44	83,0%
Select seq gb|KF307420.1|	Lepechinia graveolens voucher Fuentes 10351 tRNA-Leu (trnL) gene and trnL-trnF intergenic spacer, partial sequence; chloroplast	1014	1014	96%	0.0	95%	KF307420.1	1003,44	83,0%
Select seq gb|KF307418.1|	Lepechinia floribunda voucher B. Drew 172 tRNA-Leu (trnL) gene and trnL-trnF intergenic spacer, partial sequence; chloroplast	1014	1014	96%	0.0	95%	KF307418.1	1003,44	83,0%
Select seq gb|JF301391.1|	Nepeta cataria voucher B. Drew 72 tRNA-Leu (trnL) gene and trnL-trnF intergenic spacer, partial sequence; chloroplast	1024	1024	97%	0.0	95%	JF301391.1	1002,89	82,9%
Select seq emb|AJ505432.1|	Nepeta racemosa plastid trnL-trnF intergenic spacer, specimen voucher Jamzad s.n. (TARI)	1053	1053	100%	0.0	95%	AJ505432.1	1000,35	82,7%
Select seq gb|JF301380.1|	Lepechinia mexicana voucher B. Drew 127 tRNA-Leu (trnL) gene and trnL-trnF intergenic spacer, partial sequence; chloroplast	1020	1020	97%	0.0	95%	JF301380.1	998,97	82,6%
Select seq gb|KF307422.1|	Lepechinia mexicana voucher B. Drew 130 tRNA-Leu (trnL) gene and trnL-trnF intergenic spacer, partial sequence; chloroplast	1009	1009	96%	0.0	95%	KF307422.1	998,49	82,6%
Select seq gb|KF307416.1|	Lepechinia codon voucher B. Drew 177 tRNA-Leu (trnL) gene and trnL-trnF intergenic spacer, partial sequence; chloroplast	1007	1007	96%	0.0	95%	KF307416.1	996,51	82,4%

Select seq gb|GU381479.1|	Nepeta supina voucher M:Groeger & Schewardnadse 1466 tRNA-Leu (trnL) gene and trnL-trnF intergenic spacer, partial sequence; chloroplast	1038	1038	99% 0.0	95% GU381479.1	996,06	82,4%
Select seq gb|DQ667517.1|	Drepanocaryum sewerzowii isolate x523 tRNA-Leu (trnL) gene and trnL-trnF intergenic spacer, partial sequence; chloroplast	1005	1005	96% 0.0	95% DQ667517.1	994,53	82,2%
Select seq gb|JF301381.1|	Lepechinia mexicana voucher B. Drew 164 tRNA-Leu (trnL) gene and trnL-trnF intergenic spacer, partial sequence; chloroplast	1013	1013	97% 0.0	95% JF301381.1	992,11	82,0%
Select seq gb|GU381496.1|	Killickia pilosa voucher M:Braeuchler 3832 tRNA-Leu (trnL) gene and trnL-trnF intergenic spacer, partial sequence; chloroplast	1033	1033	99% 0.0	95% GU381496.1	991,26	82,0%
Select seq gb|GU381493.1|	Killickia grandiflora voucher M:Braeuchler 3811 tRNA-Leu (trnL) gene and trnL-trnF intergenic spacer, partial sequence; chloroplast	1033	1033	99% 0.0	95% GU381493.1	991,26	82,0%
Select seq emb|AJ505433.1|	Nepeta straussii plastid trnL-trnF intergenic spacer, specimen voucher Jamzad etal 76846 (TARI)	1040	1040	100% 0.0	95% AJ505433.1	988,00	81,7%
Select seq gb|GU381495.1|	Killickia pilosa voucher M:Braeuchler 3810 tRNA-Leu (trnL) gene and trnL-trnF intergenic spacer, partial sequence; chloroplast	1018	1018	98% 0.0	95% GU381495.1	986,84	81,6%
Select seq gb|KF307419.1|	Lepechinia ganderi voucher B. Drew 24 tRNA-Leu (trnL) gene and trnL-trnF intergenic spacer, partial sequence; chloroplast	996	996	96% 0.0	95% KF307419.1	985,63	81,5%
Select seq gb|AY570459.1|	Lepechinia chamaedryoides voucher JBW 2537 tRNA-Leu and trnL-trnF intergenic spacer, partial sequence; chloroplast	1037	1037	100% 0.0	95% AY570459.1	985,15	81,5%
Select seq gb|KR150191.1|	Nepeta sp. Kh55 trnL-trnF intergenic spacer region, partial sequence; chloroplast	994	994	96% 0.0	95% KR150191.1	983,65	81,3%
Select seq gb|AY570460.1|	Lepechinia fragrans voucher JBW 1333 tRNA-Leu and trnL-trnF intergenic spacer, partial sequence; chloroplast	1024	1024	99% 0.0	95% AY570460.1	982,63	81,3%
Select seq gb|KF307429.1|	Lepechinia salviae voucher R. Jabaily s.n. tRNA-Leu (trnL) gene and trnL-trnF intergenic spacer, partial sequence; chloroplast	992	992	96% 0.0	95% KF307429.1	981,67	81,2%
Select seq gb|AY840207.1|	Thymbra spicata tRNA-Leu (trnL) gene and trnL-trnF intergenic spacer, partial sequence; chloroplast	1022	1022	99% 0.0	95% AY840207.1	980,71	81,1%
Select seq gb|DQ667494.1|	Lepechinia calycina isolate x246 tRNA-Leu (trnL) gene and trnL-trnF intergenic spacer, partial sequence; chloroplast	1031	1031	100% 0.0	95% DQ667494.1	979,45	81,0%
Select seq gb|GU381484.1|	Micromeria flagellaris voucher E:van der Werff & McPherson 13570 tRNA-Leu (trnL) gene and trnL-trnF intergenic spacer, partial sequence; chloroplast	1020	1020	99% 0.0	95% GU381484.1	978,79	80,9%
Select seq gb|GU381483.1|	Micromeria flagellaris voucher E:Clement et al 2140 tRNA-Leu (trnL) gene and trnL-trnF intergenic spacer, partial sequence; chloroplast	1020	1020	99% 0.0	95% GU381483.1	978,79	80,9%
Select seq gb|AY506622.1|	Cedronella canariensis tRNA-Leu (trnL) gene and trnL-trnF intergenic spacer, partial sequence; chloroplast	989	989	96% 0.0	95% AY506622.1	978,70	80,9%
Select seq gb|JF301375.1|	Lepechinia calycina voucher B. Drew 197 tRNA-Leu (trnL) gene and trnL-trnF intergenic spacer, partial sequence; chloroplast	998	998	97% 0.0	95% JF301375.1	977,42	80,8%
Select seq gb|KF307414.1|	Lepechinia calycina voucher B. Drew 20 tRNA-Leu (trnL) gene and trnL-trnF intergenic spacer, partial sequence; chloroplast	987	987	96% 0.0	95% KF307414.1	976,72	80,8%
Select seq gb|JF301376.1|	Lepechinia caulescens voucher B. Drew 106 tRNA-Leu (trnL) gene and trnL-trnF intergenic spacer, partial sequence; chloroplast	996	996	97% 0.0	95% JF301376.1	975,46	80,7%
Select seq gb|JF301360.1|	Cedronella canariensis voucher UCBG 2004.0788 tRNA-Leu (trnL) gene and trnL-trnF intergenic spacer, partial sequence; chloroplast	996	996	97% 0.0	95% JF301360.1	975,46	80,7%
Select seq gb|GU381632.1|	Thymbra spicata voucher M:Braeuchler 4548 tRNA-Leu (trnL) gene and trnL-trnF intergenic spacer, partial sequence; chloroplast	1016	1016	99% 0.0	95% GU381632.1	974,95	80,6%
Select seq gb|GU381629.1|	Thymbra capitata voucher M:Braeuchler 2518 tRNA-Leu (trnL) gene and trnL-trnF intergenic spacer, partial sequence; chloroplast	1014	1014	99% 0.0	95% GU381629.1	973,03	80,5%
Select seq gb|AY570458.1|	Lepechinia calycina voucher JBW 1344 tRNA-Leu and trnL-trnF intergenic spacer, partial sequence; chloroplast	1024	1024	100% 0.0	95% AY570458.1	972,80	80,4%
Select seq gb|GU381489.1|	Killickia lutea voucher NU<ZAF>:Hilliard & Burtt 9876 tRNA-Leu (trnL) gene and trnL-trnF intergenic spacer, partial sequence; chloroplast	1011	1011	99% 0.0	95% GU381489.1	970,15	80,2%
Select seq gb|GU381488.1|	Killickia compacta voucher M:Braeuchler 3816 tRNA-Leu (trnL) gene and trnL-trnF intergenic spacer, partial sequence; chloroplast	1011	1011	99% 0.0	95% GU381488.1	970,15	80,2%
Select seq gb|JF301401.1|	Thymbra capitata voucher UCBG 96.0817 tRNA-Leu (trnL) gene and trnL-trnF intergenic spacer, partial sequence; chloroplast	989	989	97% 0.0	94% JF301401.1	958,41	79,3%
Select seq gb|GU381627.1|	Thymbra sintenisii subsp. isaurica voucher E:Goener 12628 tRNA-Leu (trnL) gene and trnL-trnF intergenic spacer, partial sequence; chloroplast	1009	1009	99% 0.0	94% GU381627.1	958,04	79,2%

Select seq gb|JF301384.1|	Lophanthus lipskyanus voucher Vassiljeva s.n. tRNA-Leu (trnL) gene and trnL-trnF intergenic spacer, partial sequence; chloroplast	998	998	97%	0.0	93%	JF301384.1	956,85	79,1%
Select seq gb|GU381517.1|	Clinopodium barosmum voucher BM<GBR-LONDON>:McLaren N193 tRNA-Leu (trnL) gene and trnL-trnF intergenic spacer, partial sequence; chloroplast	1003	1003	99%	0.0	94%	GU381517.1	952,34	78,8%
Select seq gb|GU381516.1|	Clinopodium wardii voucher BM<GBR-LONDON>:Ludlow et al. 14234 tRNA-Leu (trnL) gene and trnL-trnF intergenic spacer, partial sequence; chloroplast	1003	1003	99%	0.0	94%	GU381516.1	952,34	78,8%
Select seq gb|GU381515.1|	Clinopodium hydaspidis voucher BM<GBR-LONDON>:Mohd 133 tRNA-Leu (trnL) gene and trnL-trnF intergenic spacer, partial sequence; chloroplast	1003	1003	99%	0.0	94%	GU381515.1	952,34	78,8%
Select seq gb|GU381514.1|	Clinopodium nepalense voucher FR:Stainton 6024 tRNA-Leu (trnL) gene and trnL-trnF intergenic spacer, partial sequence; chloroplast	1003	1003	99%	0.0	94%	GU381514.1	952,34	78,8%
Select seq gb|GU381511.1|	Clinopodium piperitum voucher BM<GBR-LONDON>:Vickery 454 tRNA-Leu (trnL) gene and trnL-trnF intergenic spacer, partial sequence; chloroplast	1003	1003	99%	0.0	94%	GU381511.1	952,34	78,8%
Select seq gb|DQ667501.1|	Ziziphora taurica isolate x262 tRNA-Leu (trnL) gene and trnL-trnF intergenic spacer, partial sequence; chloroplast	1000	1000	99%	0.0	94%	DQ667501.1	949,49	78,5%
Select seq gb|GU381512.1|	Clinopodium piperitum voucher E:Stainton 7320 tRNA-Leu (trnL) gene and trnL-trnF intergenic spacer, partial sequence; chloroplast	998	998	99%	0.0	94%	GU381512.1	947,60	78,4%
Select seq gb|GU381510.1|	Ziziphora pamiroalaica voucher C:Murray et al. 10090 tRNA-Leu (trnL) gene and trnL-trnF intergenic spacer, partial sequence; chloroplast	998	998	99%	0.0	94%	GU381510.1	947,60	78,4%
Select seq gb|GU381505.1|	Clinopodium troodi voucher W:Davis 1856 tRNA-Leu (trnL) gene and trnL-trnF intergenic spacer, partial sequence; chloroplast	998	998	99%	0.0	94%	GU381505.1	947,60	78,4%
Select seq gb|GU381501.1|	Clinopodium graveolens subsp. rotundifolium voucher M:Podlech 47181 tRNA-Leu (trnL) gene and trnL-trnF intergenic spacer, partial sequence; chloroplast	998	998	99%	0.0	94%	GU381501.1	947,60	78,4%
Select seq gb|GU381499.1|	Clinopodium suaveolens voucher M:Erben s.n. tRNA-Leu (trnL) gene and trnL-trnF intergenic spacer, partial sequence; chloroplast	998	998	99%	0.0	94%	GU381499.1	947,60	78,4%
Select seq gb|GU381485.1|	Micromeria sphaerophylla voucher E:Lewis et al 1064 tRNA-Leu (trnL) gene and trnL-trnF intergenic spacer, partial sequence; chloroplast	998	998	99%	0.0	94%	GU381485.1	947,60	78,4%
Select seq gb|KR150198.1|	Ziziphora sp. 3 Kh112 trnL-trnF intergenic spacer region, partial sequence; chloroplast	987	987	98%	0.0	94%	KR150198.1	946,71	78,3%
Select seq gb|JQ669021.1|	Clinopodium acinos voucher Judziewicz 14160 tRNA-Leu (trnL) gene and trnL-trnF intergenic spacer, partial sequence; plastid	987	987	98%	0.0	94%	JQ669021.1	946,71	78,3%
Select seq gb|DQ667493.1|	Lepechinia conferta isolate x234 tRNA-Leu (trnL) gene and trnL-trnF intergenic spacer, partial sequence; chloroplast	1007	1007	100%	0.0	94%	DQ667493.1	946,58	78,3%
Select seq gb|KR150238.1|	Ziziphora sp. 1 Kh75 trnL-trnF intergenic spacer region, partial sequence; chloroplast	1005	1005	100%	0.0	94%	KR150238.1	944,70	78,1%
Select seq gb|GU381480.1|	Agastache foeniculum voucher M:Braeuchler 2513 tRNA-Leu (trnL) gene and trnL-trnF intergenic spacer, partial sequence; chloroplast	994	994	99%	0.0	94%	GU381480.1	943,80	78,1%
Select seq gb|GU381633.1|	Thymus caespititius voucher M:Heubl s.n. tRNA-Leu (trnL) gene and trnL-trnF intergenic spacer, partial sequence; chloroplast	992	992	99%	0.0	94%	GU381633.1	941,90	77,9%
Select seq gb|GU381507.1|	Ziziphora tenuior voucher MSB:Fayvush et al. 03-1503 tRNA-Leu (trnL) gene and trnL-trnF intergenic spacer, partial sequence; chloroplast	992	992	99%	0.0	94%	GU381507.1	941,90	77,9%
Select seq gb|GU381497.1|	Clinopodium acinos voucher M:Podlech 50287 tRNA-Leu (trnL) gene and trnL-trnF intergenic spacer, partial sequence; chloroplast	992	992	99%	0.0	94%	GU381497.1	941,90	77,9%
Select seq gb|GU381481.1|	Micromeria cf. madagascariensis Morawetz 205 tRNA-Leu (trnL) gene and trnL-trnF intergenic spacer, partial sequence; chloroplast	992	992	99%	0.0	94%	GU381481.1	941,90	77,9%
Select seq gb|AY840202.1|	Origanum vulgare tRNA-Leu (trnL) gene and trnL-trnF intergenic spacer, partial sequence; chloroplast	992	992	99%	0.0	94%	AY840202.1	941,90	77,9%
Select seq gb|EU244612.1|	Agastache rugosa tRNA-Leu (trnL) gene and trnL-trnF intergenic spacer, partial sequence	1002	1002	100%	0.0	94%	EU244612.1	941,88	77,9%
Select seq gb|JX880022.1|	Origanum vulgare subsp. vulgare chloroplast, complete genome	1000	1000	100%	0.0	94%	JX880022.1	940,00	77,7%
Select seq gb|JQ690290.1|	Origanum elongatum isolate H5_O_elo trnL-trnF intergenic spacer, partial sequence; chloroplast	1000	1000	100%	0.0	94%	JQ690290.1	940,00	77,7%
Select seq gb|JQ690289.1|	Origanum rotundifolium isolate DNA3_O_rot trnL-trnF intergenic spacer, partial sequence; chloroplast	1000	1000	100%	0.0	94%	JQ690289.1	940,00	77,7%
Select seq emb|AJ505543.1|	Origanum vulgare plastid trnL-trnF intergenic spacer, specimen voucher cult., K-000-69-19317, chase 13334 (K)	1000	1000	100%	0.0	94%	AJ505543.1	940,00	77,7%

Select seq gb|KR150218.1|	Thymus sp. 2 Kh69 trnL-trnF intergenic spacer region, partial sequence; chloroplast	989	989	99%	0.0	94%	KR150218.1	939,05	77,7%
Select seq gb|KR150222.1|	Thymus sp. 3 Kh65 trnL-trnF intergenic spacer region, partial sequence; chloroplast	987	987	99%	0.0	94%	KR150222.1	937,15	77,5%
Select seq gb|GU381640.1|	Argantoniella salzmannii voucher M:Barra et al. 2673GL tRNA-Leu (trnL) gene and trnL-trnF intergenic spacer, partial sequence; chloroplast	987	987	99%	0.0	94%	GU381640.1	937,15	77,5%
Select seq gb|AY570452.1|	Agastache urticifolia voucher JBW 815 tRNA-Leu and trnL-trnF intergenic spacer, partial sequence; chloroplast	996	996	100%	0.0	94%	AY570452.1	936,24	77,4%
Select seq gb|KR063656.1|	Satureja pilosa subsp. origanita tRNA-Leu (trnL) gene, partial sequence; trnL-trnF intergenic spacer, complete sequence; and tRNA-Phe (trnF) gene, partial sequence; chloroplast	992	992	100%	0.0	94%	KR063656.1	932,48	77,1%
Select seq gb|KR063657.1|	Thymus sibthorpii tRNA-Leu (trnL) gene, partial sequence; trnL-trnF intergenic spacer, complete sequence; and tRNA-Phe (trnF) gene, partial sequence; chloroplast	989	989	100%	0.0	94%	KR063657.1	929,66	76,9%
Select seq gb|KR150229.1|	Thymus sp. 5 Kh16 trnL-trnF intergenic spacer region, partial sequence; chloroplast	989	989	100%	0.0	94%	KR150229.1	929,66	76,9%
Select seq gb|KR150225.1|	Thymus sp. 4 Kh24 trnL-trnF intergenic spacer region, partial sequence; chloroplast	989	989	100%	0.0	94%	KR150225.1	929,66	76,9%
Select seq gb|JQ690293.1|	Origanum dayi isolate H43_O_day trnL-trnF intergenic spacer, partial sequence; chloroplast	989	989	100%	0.0	94%	JQ690293.1	929,66	76,9%
Select seq gb|EU556532.1|	Thymus amurensis isolate HX tRNA-Leu (trnL) gene and trnL-trnF intergenic spacer, partial sequence; chloroplast	989	989	100%	0.0	94%	EU556532.1	929,66	76,9%
Select seq gb|EU556525.1|	Thymus quinquecostatus isolate CL tRNA-Leu (trnL) gene and trnL-trnF intergenic spacer, partial sequence; chloroplast	989	989	100%	0.0	94%	EU556525.1	929,66	76,9%
Select seq gb|KR150209.1|	Satureja sp. 3 Kh90 trnL-trnF intergenic spacer region, partial sequence; chloroplast	987	987	100%	0.0	94%	KR150209.1	927,78	76,7%
Select seq gb|EF153679.1|	Prunella grandiflora tRNA-Leu (trnL) and trnL-trnF intergenic spacer, partial sequence; chloroplast	987	987	100%	0.0	93%	EF153679.1	917,91	75,9%
Select seq gb|DQ667508.1|	Prunella vulgaris isolate x314 tRNA-Leu (trnL) gene and trnL-trnF intergenic spacer, partial sequence; chloroplast	987	987	100%	0.0	93%	DQ667508.1	917,91	75,9%
								0,00	0,0%
								0,00	0,0%
								0,00	0,0%

Select for downloading or viewing reports	Kh011_ITS Description	Max score	Total score	Query cover	E value	Ident	Accession	(Ident/Cover)* Max score	Deviation from top hit
Select seq gb|EU179216.1|	Anthemis cotula 18S ribosomal RNA gene, partial sequence; internal transcribed spacer 1, 5.8S ribosomal RNA gene, and internal transcribed spacer 2, complete sequence; and 26S ribosomal RNA gene, partial sequence	957	957	98%	0.0	94%	EU179216.1	917,94	100,0%
Select seq dbj|AB683287.1|	Tanacetum haradjanii gene for ITS1, 5.8S rRNA and ITS2, complete sequence, specimen_voucher: 16366 (M)	928	928	97%	0.0	94%	AB683287.1	899,30	98,0%
Select seq dbj|AB683278.1|	Tanacetum demetrii gene for ITS1, 5.8S rRNA and ITS2, complete sequence, specimen_voucher: (W, 2001-10597)	918	918	97%	0.0	93%	AB683278.1	880,14	95,9%
Select seq dbj|AB683289.1|	Tanacetum hololeucum gene for ITS1, 5.8S rRNA and ITS2, complete sequence, specimen_voucher: 6472 (S)	917	917	97%	0.0	93%	AB683289.1	879,19	95,8%
Select seq dbj|AB683260.1|	Tanacetum abrotanifolium gene for ITS1, 5.8S rRNA and ITS2, complete sequence, specimen_voucher: 68865 (TARI)	917	917	97%	0.0	93%	AB683260.1	879,19	95,8%
Select seq emb|FN823080.1|	Tanacetum parthenium genomic DNA containing ITS1, 5.8S rRNA gene, ITS2, specimen voucher MPH:1119	917	917	97%	0.0	93%	FN823080.1	879,19	95,8%
Select seq dbj|AB683270.1|	Tanacetum balsamita gene for ITS1, 5.8S rRNA and ITS2, complete sequence, specimen_voucher: 1126 (MPH)	915	915	97%	0.0	93%	AB683270.1	877,27	95,6%
Select seq dbj|AB683268.1|	Tanacetum aucherianum gene for ITS1, 5.8S rRNA and ITS2, complete sequence, specimen_voucher: 44441 (HUB)	915	915	97%	0.0	93%	AB683268.1	877,27	95,6%
Select seq gb|EF577322.1|	Tanacetum ptarmiciflorum 18S ribosomal RNA gene, partial sequence; internal transcribed spacer 1, 5.8S ribosomal RNA gene, and internal transcribed spacer 2, complete sequence; and 28S ribosomal RNA gene, partial sequence	942	942	100%	0.0	93%	EF577322.1	876,06	95,4%
Select seq gb|EF577320.1|	Tanacetum parthenium 18S ribosomal RNA gene, partial sequence; internal transcribed spacer 1, 5.8S ribosomal RNA gene, and internal transcribed spacer 2, complete sequence; and 28S ribosomal RNA gene, partial sequence	942	942	100%	0.0	93%	EF577320.1	876,06	95,4%
Select seq dbj|AB683303.1|	Tanacetum parthenifolium gene for ITS1, 5.8S rRNA and ITS2, complete sequence, specimen_voucher: 1127 (MPH)	911	911	97%	0.0	93%	AB683303.1	873,43	95,2%
Select seq dbj|AB683271.1|	Tanacetum bamianicum gene for ITS1, 5.8S rRNA and ITS2, complete sequence, specimen_voucher: 18238 (B)	911	911	97%	0.0	93%	AB683271.1	873,43	95,2%
Select seq gb|AY603264.1|	Tanacetum vulgare internal transcribed spacer 1, 5.8S ribosomal RNA gene, and internal transcribed spacer 2, complete sequence	911	911	97%	0.0	93%	AY603264.1	873,43	95,2%
Select seq dbj|AB683290.1|	Tanacetum khorassanicum gene for ITS1, 5.8S rRNA and ITS2, complete sequence, specimen_voucher: 4937 (S)	909	909	97%	0.0	93%	AB683290.1	871,52	94,9%
Select seq dbj|AB683285.1|	Tanacetum germanicopolitanum gene for ITS1, 5.8S rRNA and ITS2, complete sequence, specimen_voucher: 14254 (S)	905	905	97%	0.0	93%	AB683285.1	867,68	94,5%
Select seq emb|FN823079.1|	Tanacetum chiliophyllum genomic DNA containing ITS1, 5.8S rRNA gene, ITS2, specimen voucher MPH:1124	905	905	97%	0.0	93%	FN823079.1	867,68	94,5%
Select seq dbj|AB683286.1|	Tanacetum griffithii gene for ITS1, 5.8S rRNA and ITS2, complete sequence, specimen_voucher: 37485 (B)	904	904	97%	0.0	93%	AB683286.1	866,72	94,4%
Select seq gb|EF577323.1|	Tanacetum vulgare 18S ribosomal RNA gene, partial sequence; internal transcribed spacer 1, 5.8S ribosomal RNA gene, and internal transcribed spacer 2, complete sequence; and 28S ribosomal RNA gene, partial sequence	931	931	100%	0.0	93%	EF577323.1	865,83	94,3%
Select seq dbj|AB608336.1|	Tanacetum polycephalum genes for ITS1, 5.8S rRNA, ITS2, complete sequence	902	902	97%	0.0	93%	AB608336.1	864,80	94,2%
Select seq dbj|AB683304.1|	Tanacetum pinnatum gene for ITS1, 5.8S rRNA and ITS2, complete sequence, specimen_voucher: 1118 (MPH)	900	900	97%	0.0	93%	AB683304.1	862,89	94,0%
Select seq dbj|AB608333.1|	Tanacetum coccineum genes for ITS1, 5.8S rRNA, ITS2, complete sequence	900	900	97%	0.0	93%	AB608333.1	862,89	94,0%
Select seq dbj|AB683282.1|	Tanacetum elbursense gene for ITS1, 5.8S rRNA and ITS2, complete sequence, specimen_voucher: 1109 (MPH)	898	898	97%	0.0	93%	AB683282.1	860,97	93,8%
Select seq dbj|AB683267.1|	Tanacetum armenum gene for ITS1, 5.8S rRNA and ITS2, complete sequence, specimen_voucher: (W, 1972-03416)	898	898	97%	0.0	93%	AB683267.1	860,97	93,8%
Select seq dbj|AB608331.1|	Tanacetum canescens genes for ITS1, 5.8S rRNA, ITS2, complete sequence	898	898	97%	0.0	93%	AB608331.1	860,97	93,8%
Select seq gb|EU179214.1|	Anthemis arvensis 18S ribosomal RNA gene, partial sequence; internal transcribed spacer 1, 5.8S ribosomal RNA gene, and internal transcribed spacer 2, complete sequence; and 26S ribosomal RNA gene, partial sequence	904	904	98%	0.0	93%	EU179214.1	857,88	93,5%
Select seq dbj|AB683308.1|	Tanacetum polycephalum subsp. farsicum gene for ITS1, 5.8S rRNA and ITS2, complete sequence, specimen_voucher: 1167 (MPH)	894	894	97%	0.0	93%	AB683308.1	857,13	93,4%

Select seq dbj|AB683288.1|	Tanacetum haussknechtii gene for ITS1, 5.8S rRNA and ITS2, complete sequence, specimen_voucher: 1193 (HUB)	894	894	97% 0.0	93% AB683288.1	857,13	93,4%
Select seq dbj|AB683269.1|	Tanacetum bachtiaricum gene for ITS1, 5.8S rRNA and ITS2, complete sequence, specimen_voucher: 1174 (MPH)	894	894	97% 0.0	93% AB683269.1	857,13	93,4%
Select seq dbj|AB608337.1|	Tanacetum tabrisianum genes for ITS1, 5.8S rRNA, ITS2, complete sequence	894	894	97% 0.0	93% AB608337.1	857,13	93,4%
Select seq emb|FN823081.1|	Tanacetum coccineum genomic DNA containing ITS1, 5.8S rRNA gene, ITS2, specimen voucher MPH:1144	894	894	97% 0.0	93% FN823081.1	857,13	93,4%
Select seq gb|AY603263.1|	Tanacetum millefolium internal transcribed spacer 1, 5.8S ribosomal RNA gene, and internal transcribed spacer 2, complete sequence	894	894	97% 0.0	93% AY603263.1	857,13	93,4%
Select seq dbj|AB683323.1|	Tanacetum sonbolii gene for ITS1, 5.8S rRNA and ITS2, complete sequence, specimen_voucher: 998 (MPH)	893	893	97% 0.0	93% AB683323.1	856,18	93,3%
Select seq dbj|AB683301.1|	Tanacetum nitens gene for ITS1, 5.8S rRNA and ITS2, complete sequence, specimen_voucher: (W, 1977-16564)	893	893	97% 0.0	93% AB683301.1	856,18	93,3%
Select seq dbj|AB683265.1|	Tanacetum argenteum subsp. canum gene for ITS1, 5.8S rRNA and ITS2, complete sequence, specimen_voucher: 46856 (B)	893	893	97% 0.0	93% AB683265.1	856,18	93,3%
Select seq dbj|AB683281.1|	Tanacetum dumosum gene for ITS1, 5.8S rRNA and ITS2, complete sequence, specimen_voucher: 1168 (MPH)	891	891	97% 0.0	93% AB683281.1	854,26	93,1%
Select seq dbj|AB683302.1|	Tanacetum paleaceum gene for ITS1, 5.8S rRNA and ITS2, complete sequence, specimen_voucher: 5828 (B)	889	889	97% 0.0	93% AB683302.1	852,34	92,9%
Select seq gb|KR150159.1|	Anthemis sp. Kh95 internal transcribed spacer 1, partial sequence; 5.8S ribosomal RNA gene gene, complete sequence; and internal transcribed spacer 2 region, partial sequence	915	915	100% 0.0	93% KR150159.1	850,95	92,7%
Select seq dbj|AB523749.1|	Tanacetum kotschyi genes for ITS1, 5.8S rRNA, ITS2, complete sequence, specimen_voucher: MPH:1129	887	887	97% 0.0	93% AB523749.1	850,42	92,6%
Select seq dbj|AB523751.1|	Tanacetum porphyrostephanum genes for ITS1, 5.8S rRNA, ITS2, complete sequence, specimen_voucher: W:1986-05841	883	883	97% 0.0	93% AB523751.1	846,59	92,2%
Select seq dbj|AB683305.1|	Tanacetum polycephalum subsp. argyrophyllum gene for ITS1, 5.8S rRNA and ITS2, complete sequence, specimen_voucher: 1125 (MPH)	891	891	97% 0.0	92% AB683305.1	845,07	92,1%
Select seq dbj|AB608332.1|	Tanacetum chiliophyllum genes for ITS1, 5.8S rRNA, ITS2, complete sequence	891	891	97% 0.0	92% AB608332.1	845,07	92,1%
Select seq dbj|AB683327.1|	Tanacetum turcomanicum gene for ITS1, 5.8S rRNA and ITS2, complete sequence, specimen_voucher: 20867 (FUMH)	889	889	97% 0.0	92% AB683327.1	843,18	91,9%
Select seq dbj|AB683316.1|	Tanacetum salsugineum gene for ITS1, 5.8S rRNA and ITS2, complete sequence, specimen_voucher: 1407 (MPH)	887	887	97% 0.0	92% AB683316.1	841,28	91,6%
Select seq dbj|AB683274.1|	Tanacetum budjnurdense gene for ITS1, 5.8S rRNA and ITS2, complete sequence, specimen_voucher: 38119 (FUMH)	887	887	97% 0.0	92% AB683274.1	841,28	91,6%
Select seq dbj|AB683339.1|	Xylanthemum paghmanense gene for ITS1, 5.8S rRNA and ITS2, complete sequence, specimen_voucher: (W-1986-05509)	883	883	97% 0.0	92% AB683339.1	837,48	91,2%
Select seq dbj|AB683336.1|	Tanacetum zahlbruckneri gene for ITS1, 5.8S rRNA and ITS2, complete sequence, specimen_voucher: 602 (MPH)	883	883	97% 0.0	92% AB683336.1	837,48	91,2%
Select seq dbj|AB683313.1|	Tanacetum punctatum gene for ITS1, 5.8S rRNA and ITS2, complete sequence, specimen_voucher: 49833 (IRAN)	883	883	97% 0.0	92% AB683313.1	837,48	91,2%
Select seq dbj|AB523746.1|	Tanacetum joharchii genes for ITS1, 5.8S rRNA, ITS2, complete sequence, specimen_voucher: FUMH:29141	883	883	97% 0.0	92% AB523746.1	837,48	91,2%
Select seq gb|JF907423.1|	Tripleurospermum maritimum subsp. inodorum voucher OSBU:2005 DE-0-OSN-2005-169 18S ribosomal RNA gene, partial sequence; internal transcribed spacer 1, 5.8S ribosomal RNA gene, and internal transcribed spacer 2, complete sequence; and 26S ribosomal RNA gene, partial sequence	909	909	100% 0.0	92% JF907423.1	836,28	91,1%
Select seq dbj|AB608330.1|	Tanacetum budjnurdense genes for ITS1, 5.8S rRNA, ITS2, complete sequence	880	880	97% 0.0	92% AB608330.1	834,64	90,9%
Select seq dbj|AB523750.1|	Tanacetum kotschyi genes for ITS1, 5.8S rRNA, ITS2, complete sequence, specimen_voucher: HUB:5534	878	878	97% 0.0	92% AB523750.1	832,74	90,7%
Select seq gb|AY603262.1|	Tanacetum macrophyllum internal transcribed spacer 1, 5.8S ribosomal RNA gene, and internal transcribed spacer 2, complete sequence	878	878	97% 0.0	92% AY603262.1	832,74	90,7%
Select seq gb|KM887400.1|	Anacyclus pyrethrum isolate SBB-1400 internal transcribed spacer 1, partial sequence; 5.8S ribosomal RNA gene, complete sequence; and internal transcribed spacer 2, partial sequence	867	867	96% 0.0	92% KM887400.1	830,88	90,5%
Select seq emb|HE602385.1|	Anacyclus homogamos genomic DNA containing ITS1, 5.8S rRNA gene, ITS2, specimen voucher Jury, SL. 20904	867	867	96% 0.0	92% HE602385.1	830,88	90,5%

Select seq dbj|AB523747.1|	Tanacetum tenuisectum genes for ITS1, 5.8S rRNA, ITS2, complete sequence, specimen_voucher: MPH:1087	874	874	97%	0.0	92%	AB523747.1	828,95	90,3%
Select seq gb|AY603229.1|	Achillea clavennae internal transcribed spacer 1, 5.8S ribosomal RNA gene, and internal transcribed spacer 2, complete sequence	874	874	97%	0.0	92%	AY603229.1	828,95	90,3%
Select seq gb|AY603191.1|	Achillea setacea internal transcribed spacer 1, 5.8S ribosomal RNA gene, and internal transcribed spacer 2, complete sequence	874	874	97%	0.0	92%	AY603191.1	828,95	90,3%
Select seq gb|AY603184.1|	Achillea pannonica internal transcribed spacer 1, 5.8S ribosomal RNA gene, and internal transcribed spacer 2, complete sequence	874	874	97%	0.0	92%	AY603184.1	828,95	90,3%
Select seq dbj|AB683266.1|	Tanacetum argenteum subsp. flabellifolium gene for ITS1, 5.8S rRNA and ITS2, complete sequence, specimen_voucher: 1976 (HUB)	870	870	97%	0.0	92%	AB683266.1	825,15	89,9%
Select seq gb|AY603190.1|	Achillea styriaca internal transcribed spacer 1, 5.8S ribosomal RNA gene, and internal transcribed spacer 2, complete sequence	870	870	97%	0.0	92%	AY603190.1	825,15	89,9%
Select seq dbj|AB683340.1|	Xylanthemum pamiricum gene for ITS1, 5.8S rRNA and ITS2, complete sequence, specimen_voucher: 34987-E (IRAN)	869	869	97%	0.0	92%	AB683340.1	824,21	89,8%
Select seq dbj|AB523748.1|	Tanacetum kotschyi genes for ITS1, 5.8S rRNA, ITS2, complete sequence, specimen_voucher: MPH:1423	869	869	97%	0.0	92%	AB523748.1	824,21	89,8%
Select seq gb|AY603249.1|	Achillea ptarmicifolia internal transcribed spacer 1, 5.8S ribosomal RNA gene, and internal transcribed spacer 2, complete sequence	869	869	97%	0.0	92%	AY603249.1	824,21	89,8%
Select seq gb|AY603240.1|	Achillea schurii internal transcribed spacer 1, 5.8S ribosomal RNA gene, and internal transcribed spacer 2, complete sequence	869	869	97%	0.0	92%	AY603240.1	824,21	89,8%
Select seq gb|AY603212.1|	Achillea nobilis internal transcribed spacer 1, 5.8S ribosomal RNA gene, and internal transcribed spacer 2, complete sequence	869	869	97%	0.0	92%	AY603212.1	824,21	89,8%
Select seq gb|AY603202.1|	Achillea euxina internal transcribed spacer 1, 5.8S ribosomal RNA gene, and internal transcribed spacer 2, complete sequence	869	869	97%	0.0	92%	AY603202.1	824,21	89,8%
Select seq gb|AY603187.1|	Achillea millefolium subsp. sudetica internal transcribed spacer 1, 5.8S ribosomal RNA gene, and internal transcribed spacer 2, complete sequence	869	869	97%	0.0	92%	AY603187.1	824,21	89,8%
Select seq gb|AY603185.1|	Achillea millefolium internal transcribed spacer 1, 5.8S ribosomal RNA gene, and internal transcribed spacer 2, complete sequence	869	869	97%	0.0	92%	AY603185.1	824,21	89,8%
Select seq emb|HE602382.1|	Anacyclus pyrethrum genomic DNA containing ITS1, 5.8S rRNA gene, ITS2, specimen voucher Jury, SL. 17869	859	859	96%	0.0	92%	HE602382.1	823,21	89,7%
Select seq gb|AY603230.1|	Achillea clavennae internal transcribed spacer 1, 5.8S ribosomal RNA gene, and internal transcribed spacer 2, complete sequence	865	865	97%	0.0	92%	AY603230.1	820,41	89,4%
Select seq gb|AY603228.1|	Achillea clusiana internal transcribed spacer 1, 5.8S ribosomal RNA gene, and internal transcribed spacer 2, complete sequence	865	865	97%	0.0	92%	AY603228.1	820,41	89,4%
Select seq gb|AY603205.1|	Achillea lanulosa subsp. alpicola internal transcribed spacer 1, 5.8S ribosomal RNA gene, and internal transcribed spacer 2, complete sequence	865	865	97%	0.0	92%	AY603205.1	820,41	89,4%
Select seq gb|AY603186.1|	Achillea millefolium internal transcribed spacer 1, 5.8S ribosomal RNA gene, and internal transcribed spacer 2, complete sequence	865	865	97%	0.0	92%	AY603186.1	820,41	89,4%
Select seq gb|KM887397.1|	Anacyclus pyrethrum isolate SBB-1407 18S ribosomal RNA gene, partial sequence; internal transcribed spacer 1, 5.8S ribosomal RNA gene, and internal transcribed spacer 2, complete sequence; and 28S ribosomal RNA gene, partial sequence	891	891	100%	0.0	92%	KM887397.1	819,72	89,3%
Select seq gb|KM887396.1|	Anacyclus pyrethrum isolate SBB-1364 18S ribosomal RNA gene, partial sequence; internal transcribed spacer 1, 5.8S ribosomal RNA gene, and internal transcribed spacer 2, complete sequence; and 28S ribosomal RNA gene, partial sequence	891	891	100%	0.0	92%	KM887396.1	819,72	89,3%
Select seq gb|AY603241.1|	Achillea schurii internal transcribed spacer 1, 5.8S ribosomal RNA gene, and internal transcribed spacer 2, complete sequence	863	863	97%	0.0	92%	AY603241.1	818,52	89,2%
Select seq gb|AY603211.1|	Achillea virescens internal transcribed spacer 1, 5.8S ribosomal RNA gene, and internal transcribed spacer 2, complete sequence	863	863	97%	0.0	92%	AY603211.1	818,52	89,2%
Select seq gb|AY603197.1|	Achillea setacea internal transcribed spacer 1, 5.8S ribosomal RNA gene, and internal transcribed spacer 2, complete sequence	863	863	97%	0.0	92%	AY603197.1	818,52	89,2%
Select seq gb|AY603219.1|	Achillea filipendulina internal transcribed spacer 1, 5.8S ribosomal RNA gene, and internal transcribed spacer 2, complete sequence	861	861	97%	0.0	92%	AY603219.1	816,62	89,0%
Select seq gb|AY603254.1|	Achillea wilhelmsii internal transcribed spacer 1, 5.8S ribosomal RNA gene, and internal transcribed spacer 2, complete sequence	859	859	97%	0.0	92%	AY603254.1	814,72	88,8%
Select seq gb|AY603204.1|	Achillea ceretanica internal transcribed spacer 1, 5.8S ribosomal RNA gene, and internal transcribed spacer 2, complete sequence	859	859	97%	0.0	92%	AY603204.1	814,72	88,8%
Select seq gb|AY603200.1|	Achillea roseoalba internal transcribed spacer 1, 5.8S ribosomal RNA gene, and internal transcribed spacer 2, complete sequence	859	859	97%	0.0	92%	AY603200.1	814,72	88,8%

Select seq gb|AY603251.1|	Achillea salicifolia internal transcribed spacer 1, 5.8S ribosomal RNA gene, and internal transcribed spacer 2, complete sequence	857	857	97%	0.0	92%	AY603251.1	812,82	88,5%
Select seq gb|AY603250.1|	Achillea biserrata internal transcribed spacer 1, 5.8S ribosomal RNA gene, and internal transcribed spacer 2, complete sequence	857	857	97%	0.0	92%	AY603250.1	812,82	88,5%
Select seq gb|AY603248.1|	Achillea ptarmica internal transcribed spacer 1, 5.8S ribosomal RNA gene, and internal transcribed spacer 2, complete sequence	857	857	97%	0.0	92%	AY603248.1	812,82	88,5%
Select seq gb|AY603247.1|	Achillea pyrenaica internal transcribed spacer 1, 5.8S ribosomal RNA gene, and internal transcribed spacer 2, complete sequence	857	857	97%	0.0	92%	AY603247.1	812,82	88,5%
Select seq gb|AY603246.1|	Achillea impatiens internal transcribed spacer 1, 5.8S ribosomal RNA gene, and internal transcribed spacer 2, complete sequence	857	857	97%	0.0	92%	AY603246.1	812,82	88,5%
Select seq gb|AY603232.1|	Achillea fraasii internal transcribed spacer 1, 5.8S ribosomal RNA gene, and internal transcribed spacer 2, complete sequence	857	857	97%	0.0	92%	AY603232.1	812,82	88,5%
Select seq gb|AY603223.1|	Achillea micrantha internal transcribed spacer 1, 5.8S ribosomal RNA gene, and internal transcribed spacer 2, complete sequence	857	857	97%	0.0	92%	AY603223.1	812,82	88,5%
Select seq gb|AY603222.1|	Achillea holosericea internal transcribed spacer 1, 5.8S ribosomal RNA gene, and internal transcribed spacer 2, complete sequence	857	857	97%	0.0	92%	AY603222.1	812,82	88,5%
Select seq gb|AY603216.1|	Achillea clypeolata internal transcribed spacer 1, 5.8S ribosomal RNA gene, and internal transcribed spacer 2, complete sequence	857	857	97%	0.0	92%	AY603216.1	812,82	88,5%
Select seq gb|EU796891.1|	Achillea millefolium 18S ribosomal RNA gene, partial sequence; internal transcribed spacer 1, 5.8S ribosomal RNA gene, and internal transcribed spacer 2, complete sequence; and 26S ribosomal RNA gene, partial sequence	881	881	100%	0.0	92%	EU796891.1	810,52	88,3%
Select seq gb|FJ553518.1|	Angiosperm environmental sample clone LTSP_EUKA_P3K07 18S ribosomal RNA, 18S-25/28S ribosomal RNA intergenic spacer, partial sequence	878	878	100%	0.0	92%	FJ553518.1	807,76	88,0%
Select seq gb|EF577285.1|	Achillea wilsoniana 18S ribosomal RNA gene, partial sequence; internal transcribed spacer 1, 5.8S ribosomal RNA gene, and internal transcribed spacer 2, complete sequence; and 28S ribosomal RNA gene, partial sequence	878	878	100%	0.0	92%	EF577285.1	807,76	88,0%
Select seq gb|JQ230971.1|	Achillea millefolium voucher SBB-1156 18S ribosomal RNA gene, partial sequence; internal transcribed spacer 1, 5.8S ribosomal RNA gene, and internal transcribed spacer 2, complete sequence; and 28S ribosomal RNA gene, partial sequence	872	872	100%	0.0	91%	JQ230971.1	793,52	86,4%
Select seq gb|KC816562.1|	Matricaria chamomilla var. recutita 18S ribosomal RNA gene, partial sequence; internal transcribed spacer 1, 5.8S ribosomal RNA gene, and internal transcribed spacer 2, complete sequence; and 28S ribosomal RNA gene, partial sequence	869	869	100%	0.0	91%	KC816562.1	790,79	86,1%
Select seq gb|FJ980358.1|	Achillea wilsoniana voucher PS0715MT02 18S ribosomal RNA gene, partial sequence; internal transcribed spacer 1, 5.8S ribosomal RNA gene, and internal transcribed spacer 2, complete sequence; and 28S ribosomal RNA gene, partial sequence	865	865	100%	0.0	91%	FJ980358.1	787,15	85,8%
Select seq gb|FJ980357.1|	Achillea wilsoniana voucher PS0715MT01 18S ribosomal RNA gene, partial sequence; internal transcribed spacer 1, 5.8S ribosomal RNA gene, and internal transcribed spacer 2, complete sequence; and 28S ribosomal RNA gene, partial sequence	863	863	100%	0.0	91%	FJ980357.1	785,33	85,6%
Select seq gb|GU818490.1|	Anacyclus valentinus isolate L1186 18S ribosomal RNA gene, partial sequence; internal transcribed spacer 1, 5.8S ribosomal RNA gene, and internal transcribed spacer 2, complete sequence; and 25S ribosomal RNA gene, partial sequence	846	846	100%	0.0	91%	GU818490.1	769,86	83,9%

Select for downloading or viewing reports	Kh011_trnL Description	Max score	Total score	Query cover	E value	Ident	Accession	(Ident/Cover)* Max score	Deviation from top hit
Select seq gb|JX073760.1|	Artemisia lagocephala tRNA-Leu (trnL) gene and trnL-trnF intergenic spacer, partial sequence; chloroplast	1114	1114	100%	0.0	99%	JX073760.1	1102,86	100,0%
Select seq gb|JX073787.1|	Artemisia ludoviciana tRNA-Leu (trnL) gene and trnL-trnF intergenic spacer, partial sequence; chloroplast	1103	1103	100%	0.0	99%	JX073787.1	1091,97	99,0%
Select seq gb|HM000099.1|	Chrysanthemum indicum isolate HEBSJZ tRNA-Leu (trnL) gene, partial sequence; trnL-trnF intergenic spacer, complete sequence; and tRNA-Phe (trnF) gene, partial sequence; chloroplast	1098	1098	100%	0.0	98%	HM000099.1	1076,04	97,6%
Select seq gb|HM000094.1|	Chrysanthemum indicum isolate HUBJZ tRNA-Leu (trnL) gene, partial sequence; trnL-trnF intergenic spacer, complete sequence; and tRNA-Phe (trnF) gene, partial sequence; chloroplast	1098	1098	100%	0.0	98%	HM000094.1	1076,04	97,6%
Select seq gb|KF736807.1|	Artemisia turcomanica tRNA-Leu (trnL) gene and trnL-trnF intergenic spacer, partial sequence; chloroplast	1096	1096	100%	0.0	98%	KF736807.1	1074,08	97,4%
Select seq gb|KF736803.1|	Artemisia fragrans tRNA-Leu (trnL) gene and trnL-trnF intergenic spacer, partial sequence; chloroplast	1096	1096	100%	0.0	98%	KF736803.1	1074,08	97,4%
Select seq gb|KF736802.1|	Artemisia incana tRNA-Leu (trnL) gene and trnL-trnF intergenic spacer, partial sequence; chloroplast	1096	1096	100%	0.0	98%	KF736802.1	1074,08	97,4%
Select seq gb|JX073815.1|	Artemisia afra tRNA-Leu (trnL) gene and trnL-trnF intergenic spacer, partial sequence; chloroplast	1096	1096	100%	0.0	98%	JX073815.1	1074,08	97,4%
Select seq gb|JX073814.1|	Mausolea eriocarpa tRNA-Leu (trnL) gene and trnL-trnF intergenic spacer, partial sequence; chloroplast	1096	1096	100%	0.0	98%	JX073814.1	1074,08	97,4%
Select seq gb|JX073812.1|	Artemisia subdigitata tRNA-Leu (trnL) gene and trnL-trnF intergenic spacer, partial sequence; chloroplast	1096	1096	100%	0.0	98%	JX073812.1	1074,08	97,4%
Select seq gb|JX073748.1|	Artemisia santonicum tRNA-Leu (trnL) gene and trnL-trnF intergenic spacer, partial sequence; chloroplast	1096	1096	100%	0.0	98%	JX073748.1	1074,08	97,4%
Select seq gb|JX073805.1|	Artemisia gracilescens tRNA-Leu (trnL) gene and trnL-trnF intergenic spacer, partial sequence; chloroplast	1096	1096	100%	0.0	98%	JX073805.1	1074,08	97,4%
Select seq gb|JX073796.1|	Artemisia scoparia tRNA-Leu (trnL) gene and trnL-trnF intergenic spacer, partial sequence; chloroplast	1096	1096	100%	0.0	98%	JX073796.1	1074,08	97,4%
Select seq gb|JX073792.1|	Artemisia japonica tRNA-Leu (trnL) gene and trnL-trnF intergenic spacer, partial sequence; chloroplast	1096	1096	100%	0.0	98%	JX073792.1	1074,08	97,4%
Select seq gb|JX073790.1|	Artemisia pycnocephala tRNA-Leu (trnL) gene and trnL-trnF intergenic spacer, partial sequence; chloroplast	1096	1096	100%	0.0	98%	JX073790.1	1074,08	97,4%
Select seq gb|JX073783.1|	Artemisia schmidtiana tRNA-Leu (trnL) gene and trnL-trnF intergenic spacer, partial sequence; chloroplast	1096	1096	100%	0.0	98%	JX073783.1	1074,08	97,4%
Select seq gb|JX073777.1|	Artemisia armeniaca tRNA-Leu (trnL) gene and trnL-trnF intergenic spacer, partial sequence; chloroplast	1096	1096	100%	0.0	98%	JX073777.1	1074,08	97,4%
Select seq gb|JX073747.1|	Artemisia nitrosa tRNA-Leu (trnL) gene and trnL-trnF intergenic spacer, partial sequence; chloroplast	1096	1096	100%	0.0	98%	JX073747.1	1074,08	97,4%
Select seq gb|JX073749.1|	Artemisia gmelinii subsp. scheludjakoviae tRNA-Leu (trnL) gene and trnL-trnF intergenic spacer, partial sequence; chloroplast	1096	1096	100%	0.0	98%	JX073749.1	1074,08	97,4%
Select seq gb|JN867592.1|	Chrysanthemum indicum voucher NJ026 chloroplast, complete genome	1096	1096	100%	0.0	98%	JN867592.1	1074,08	97,4%
Select seq dbj|AB551235.1|	Artemisia capillaris chloroplast genes for tRNA-Leu intron and 3' exon, trnL-trnF intergenic spacer and tRNA-Phe	1096	1096	100%	0.0	98%	AB551235.1	1074,08	97,4%
Select seq gb|FJ842617.1|	Artemisia fukudo voucher MPS001124 (KMH) tRNA-Leu (trnL) gene and trnL-trnF intergenic spacer, partial sequence; chloroplast	1096	1096	100%	0.0	98%	FJ842617.1	1074,08	97,4%
Select seq gb|FJ842615.1|	Artemisia capillaris voucher MPS001196 (KMH) tRNA-Leu (trnL) gene and trnL-trnF intergenic spacer, partial sequence; chloroplast	1096	1096	100%	0.0	98%	FJ842615.1	1074,08	97,4%
Select seq gb|JX073816.1|	Artemisia elongata tRNA-Leu (trnL) gene and trnL-trnF intergenic spacer, partial sequence; chloroplast	1092	1092	100%	0.0	98%	JX073816.1	1070,16	97,0%
Select seq gb|KF736827.1|	Artemisia ciniformis tRNA-Leu (trnL) gene and trnL-trnF intergenic spacer, partial sequence; chloroplast	1090	1090	100%	0.0	98%	KF736827.1	1068,20	96,9%
Select seq gb|KF736826.1|	Artemisia aucheri tRNA-Leu (trnL) gene and trnL-trnF intergenic spacer, partial sequence; chloroplast	1090	1090	100%	0.0	98%	KF736826.1	1068,20	96,9%
Select seq gb|KF736825.1|	Artemisia melanolepis tRNA-Leu (trnL) gene and trnL-trnF intergenic spacer, partial sequence; chloroplast	1090	1090	100%	0.0	98%	KF736825.1	1068,20	96,9%

Select seq gb|KF736805.1|	Artemisia spicigera tRNA-Leu (trnL) gene and trnL-trnF intergenic spacer, partial sequence; chloroplast	1090	1090	100% 0.0	98% KF736805.1	1068,20	96,9%
Select seq gb|KF669899.1|	Artemisia absinthium tRNA-Leu (trnL) gene and trnL-trnF intergenic spacer, partial sequence; chloroplast	1090	1090	100% 0.0	98% KF669899.1	1068,20	96,9%
Select seq gb|JX293720.1|	Artemisia frigida chloroplast, complete genome	1090	1090	100% 0.0	98% JX293720.1	1068,20	96,9%
Select seq gb|JX073811.1|	Artemisia glomerata tRNA-Leu (trnL) gene and trnL-trnF intergenic spacer, partial sequence; chloroplast	1090	1090	100% 0.0	98% JX073811.1	1068,20	96,9%
Select seq gb|JX073808.1|	Artemisia tilesii tRNA-Leu (trnL) gene and trnL-trnF intergenic spacer, partial sequence; chloroplast	1090	1090	100% 0.0	98% JX073808.1	1068,20	96,9%
Select seq gb|JX073786.1|	Artemisia suksdorfii tRNA-Leu (trnL) gene and trnL-trnF intergenic spacer, partial sequence; chloroplast	1090	1090	100% 0.0	98% JX073786.1	1068,20	96,9%
Select seq gb|JX073785.1|	Artemisia serrata tRNA-Leu (trnL) gene and trnL-trnF intergenic spacer, partial sequence; chloroplast	1090	1090	100% 0.0	98% JX073785.1	1068,20	96,9%
Select seq gb|JX073779.1|	Artemisia alba tRNA-Leu (trnL) gene and trnL-trnF intergenic spacer, partial sequence; chloroplast	1090	1090	100% 0.0	98% JX073779.1	1068,20	96,9%
Select seq gb|HM000091.1|	Chrysanthemum indicum isolate HENXY tRNA-Leu (trnL) gene, partial sequence; trnL-trnF intergenic spacer, complete sequence; and tRNA-Phe (trnF) gene, partial sequence; chloroplast	1090	1090	100% 0.0	98% HM000091.1	1068,20	96,9%
Select seq gb|FJ842621.1|	Artemisia keiskeana voucher MPS001234 (KMH) tRNA-Leu (trnL) gene and trnL-trnF intergenic spacer, partial sequence; chloroplast	1090	1090	100% 0.0	98% FJ842621.1	1068,20	96,9%
Select seq gb|FJ842619.1|	Artemisia japonica voucher MPS000177 (KMH) tRNA-Leu (trnL) gene and trnL-trnF intergenic spacer, partial sequence; chloroplast	1090	1090	100% 0.0	98% FJ842619.1	1068,20	96,9%
Select seq gb|FJ842618.1|	Artemisia gmelinii voucher MPS001216 (KMH) tRNA-Leu (trnL) gene and trnL-trnF intergenic spacer, partial sequence; chloroplast	1090	1090	100% 0.0	98% FJ842618.1	1068,20	96,9%
Select seq gb|FJ710526.1|	Artemisia absinthium tRNA-Leu (trnL) gene and trnL-trnF intergenic spacer, partial sequence; chloroplast	1090	1090	100% 0.0	98% FJ710526.1	1068,20	96,9%
Select seq gb|FJ692341.1|	Artemisia annua voucher MPS 001275 (KMH) tRNA-Leu (trnL) gene and trnL-trnF intergenic spacer, partial sequence; chloroplast	1090	1090	100% 0.0	98% FJ692341.1	1068,20	96,9%
Select seq dbj|AB219593.1|	Artemisia sinanensis chloroplast trnL(UAA), trnF(GAA) genes for tRNA-Leu, tRNA-Phe, partial sequence, trnL-trnF spacer region, specimen_voucher: MAK:N. Fujii F01782	1090	1090	100% 0.0	98% AB219593.1	1068,20	96,9%
Select seq gb|JX073804.1|	Artemisia chamaemelifolia tRNA-Leu (trnL) gene and trnL-trnF intergenic spacer, partial sequence; chloroplast	1086	1086	100% 0.0	98% JX073804.1	1064,28	96,5%
Select seq gb|KF736809.1|	Artemisia marschalliana tRNA-Leu (trnL) gene and trnL-trnF intergenic spacer, partial sequence; chloroplast	1085	1085	100% 0.0	98% KF736809.1	1063,30	96,4%
Select seq gb|KF736808.1|	Artemisia chamaemelifolia tRNA-Leu (trnL) gene and trnL-trnF intergenic spacer, partial sequence; chloroplast	1085	1085	100% 0.0	98% KF736808.1	1063,30	96,4%
Select seq gb|KF736806.1|	Artemisia haussknechtii tRNA-Leu (trnL) gene and trnL-trnF intergenic spacer, partial sequence; chloroplast	1085	1085	100% 0.0	98% KF736806.1	1063,30	96,4%
Select seq gb|JX073828.1|	Kaschgaria komarovii tRNA-Leu (trnL) gene and trnL-trnF intergenic spacer, partial sequence; chloroplast	1085	1085	100% 0.0	98% JX073828.1	1063,30	96,4%
Select seq gb|JX073827.1|	Artemisia absinthium tRNA-Leu (trnL) gene and trnL-trnF intergenic spacer, partial sequence; chloroplast	1085	1085	100% 0.0	98% JX073827.1	1063,30	96,4%
Select seq gb|JX073821.1|	Artemisia mauiensis tRNA-Leu (trnL) gene and trnL-trnF intergenic spacer, partial sequence; chloroplast	1085	1085	100% 0.0	98% JX073821.1	1063,30	96,4%
Select seq gb|JX073819.1|	Artemisia australis tRNA-Leu (trnL) gene and trnL-trnF intergenic spacer, partial sequence; chloroplast	1085	1085	100% 0.0	98% JX073819.1	1063,30	96,4%
Select seq gb|JX073791.1|	Artemisia tomentella tRNA-Leu (trnL) gene and trnL-trnF intergenic spacer, partial sequence; chloroplast	1085	1085	100% 0.0	98% JX073791.1	1063,30	96,4%
Select seq gb|JX073800.1|	Artemisia carruthii tRNA-Leu (trnL) gene and trnL-trnF intergenic spacer, partial sequence; chloroplast	1085	1085	100% 0.0	98% JX073800.1	1063,30	96,4%
Select seq gb|JX073799.1|	Artemisia gorgonum tRNA-Leu (trnL) gene and trnL-trnF intergenic spacer, partial sequence; chloroplast	1085	1085	100% 0.0	98% JX073799.1	1063,30	96,4%
Select seq gb|JX073782.1|	Artemisia nitida tRNA-Leu (trnL) gene and trnL-trnF intergenic spacer, partial sequence; chloroplast	1085	1085	100% 0.0	98% JX073782.1	1063,30	96,4%
Select seq gb|JX073778.1|	Artemisia abrotanum tRNA-Leu (trnL) gene and trnL-trnF intergenic spacer, partial sequence; chloroplast	1085	1085	100% 0.0	98% JX073778.1	1063,30	96,4%

Select seq gb|JX073774.1|	Artemisia molinieri tRNA-Leu (trnL) gene and trnL-trnF intergenic spacer, partial sequence; chloroplast	1085	1085	100% 0.0	98% JX073774.1	1063,30	96,4%
Select seq gb|JX073761.1|	Artemisia anomala tRNA-Leu (trnL) gene and trnL-trnF intergenic spacer, partial sequence; chloroplast	1085	1085	100% 0.0	98% JX073761.1	1063,30	96,4%
Select seq gb|HM000085.1|	Chrysanthemum indicum isolate JSNJ tRNA-Leu (trnL) gene, partial sequence; trnL-trnF intergenic spacer, complete sequence; and tRNA-Phe (trnF) gene, partial sequence; chloroplast	1085	1085	100% 0.0	98% HM000085.1	1063,30	96,4%
Select seq gb|FJ842620.1|	Artemisia manshurica voucher MPS00123 (KMH) tRNA-Leu (trnL) gene and trnL-trnF intergenic spacer, partial sequence; chloroplast	1085	1085	100% 0.0	98% FJ842620.1	1063,30	96,4%
Select seq gb|FJ710524.1|	Artemisia caruifolia var. apiacea tRNA-Leu (trnL) gene and trnL-trnF intergenic spacer, partial sequence; chloroplast	1085	1085	100% 0.0	98% FJ710524.1	1063,30	96,4%
Select seq gb|JX073797.1|	Artemisia dracunculus tRNA-Leu (trnL) gene and trnL-trnF intergenic spacer, partial sequence; chloroplast	1083	1083	100% 0.0	98% JX073797.1	1061,34	96,2%
Select seq gb|JN867589.1|	Chrysanthemum indicum voucher HeN001 chloroplast, complete genome	1083	1083	100% 0.0	98% JN867589.1	1061,34	96,2%
Select seq gb|JN315916.1|	Chrysanthemum indicum tRNA-Leu (trnL) gene and trnL-trnF intergenic spacer, partial sequence; chloroplast	1083	1083	100% 0.0	98% JN315916.1	1061,34	96,2%
Select seq dbj|AB685322.1|	Chrysanthemum indicum chloroplast DNA, contains tRNA-Leu(trnL) gene (intron and 3' exon), trnL-trnF intergenic spacer and tRNA-Phe(trnF) gene	1083	1083	100% 0.0	98% AB685322.1	1061,34	96,2%
Select seq gb|HM000097.1|	Chrysanthemum indicum isolate JXPX tRNA-Leu (trnL) gene, partial sequence; trnL-trnF intergenic spacer, complete sequence; and tRNA-Phe (trnF) gene, partial sequence; chloroplast	1083	1083	100% 0.0	98% HM000097.1	1061,34	96,2%
Select seq gb|HM000096.1|	Chrysanthemum indicum isolate YNKM tRNA-Leu (trnL) gene, partial sequence; trnL-trnF intergenic spacer, complete sequence; and tRNA-Phe (trnF) gene, partial sequence; chloroplast	1083	1083	100% 0.0	98% HM000096.1	1061,34	96,2%
Select seq gb|HM000095.1|	Chrysanthemum indicum isolate HUNZZ tRNA-Leu (trnL) gene, partial sequence; trnL-trnF intergenic spacer, complete sequence; and tRNA-Phe (trnF) gene, partial sequence; chloroplast	1083	1083	100% 0.0	98% HM000095.1	1061,34	96,2%
Select seq gb|HM000088.1|	Chrysanthemum indicum isolate GZGY tRNA-Leu (trnL) gene, partial sequence; trnL-trnF intergenic spacer, complete sequence; and tRNA-Phe (trnF) gene, partial sequence; chloroplast	1083	1083	100% 0.0	98% HM000088.1	1061,34	96,2%
Select seq gb|HM000087.1|	Chrysanthemum indicum isolate AHCZ tRNA-Leu (trnL) gene, partial sequence; trnL-trnF intergenic spacer, complete sequence; and tRNA-Phe (trnF) gene, partial sequence; chloroplast	1083	1083	100% 0.0	98% HM000087.1	1061,34	96,2%
Select seq gb|KF736821.1|	Artemisia capillaris tRNA-Leu (trnL) gene and trnL-trnF intergenic spacer, partial sequence; chloroplast	1079	1079	100% 0.0	98% KF736821.1	1057,42	95,9%
Select seq gb|JX073826.1|	Artemisia atrata tRNA-Leu (trnL) gene and trnL-trnF intergenic spacer, partial sequence; chloroplast	1079	1079	100% 0.0	98% JX073826.1	1057,42	95,9%
Select seq gb|JX073822.1|	Artemisia chinensis tRNA-Leu (trnL) gene and trnL-trnF intergenic spacer, partial sequence; chloroplast	1079	1079	100% 0.0	98% JX073822.1	1057,42	95,9%
Select seq gb|JX073820.1|	Artemisia kauaiensis tRNA-Leu (trnL) gene and trnL-trnF intergenic spacer, partial sequence; chloroplast	1079	1079	100% 0.0	98% JX073820.1	1057,42	95,9%
Select seq gb|JX073803.1|	Artemisia tournefortiana tRNA-Leu (trnL) gene and trnL-trnF intergenic spacer, partial sequence; chloroplast	1079	1079	100% 0.0	98% JX073803.1	1057,42	95,9%
Select seq gb|JX073753.1|	Artemisia arbuscula tRNA-Leu (trnL) gene and trnL-trnF intergenic spacer, partial sequence; chloroplast	1079	1079	100% 0.0	98% JX073753.1	1057,42	95,9%
Select seq gb|JX073798.1|	Artemisia rigida tRNA-Leu (trnL) gene and trnL-trnF intergenic spacer, partial sequence; chloroplast	1079	1079	100% 0.0	98% JX073798.1	1057,42	95,9%
Select seq gb|JX073788.1|	Artemisia scopulorum tRNA-Leu (trnL) gene and trnL-trnF intergenic spacer, partial sequence; chloroplast	1079	1079	100% 0.0	98% JX073788.1	1057,42	95,9%
Select seq gb|JX073775.1|	Artemisia laciniata tRNA-Leu (trnL) gene and trnL-trnF intergenic spacer, partial sequence; chloroplast	1079	1079	100% 0.0	98% JX073775.1	1057,42	95,9%
Select seq gb|JX073768.1|	Artemisia roxburghiana tRNA-Leu (trnL) gene and trnL-trnF intergenic spacer, partial sequence; chloroplast	1079	1079	100% 0.0	98% JX073768.1	1057,42	95,9%
Select seq gb|JQ362483.1|	Chrysanthemum x morifolium chloroplast, complete genome	1079	1079	100% 0.0	98% JQ362483.1	1057,42	95,9%
Select seq gb|HM000086.1|	Chrysanthemum indicum isolate JSWX tRNA-Leu (trnL) gene, partial sequence; trnL-trnF intergenic spacer, complete sequence; and tRNA-Phe (trnF) gene, partial sequence; chloroplast	1079	1079	100% 0.0	98% HM000086.1	1057,42	95,9%
Select seq gb|FJ842626.1|	Artemisia selengensis voucher MPS001262 (KMH) tRNA-Leu (trnL) gene and trnL-trnF intergenic spacer, partial sequence; chloroplast	1079	1079	100% 0.0	98% FJ842626.1	1057,42	95,9%
Select seq gb|JX073750.1|	Artemisia pontica tRNA-Leu (trnL) gene and trnL-trnF intergenic spacer, partial sequence; chloroplast	1077	1077	100% 0.0	98% JX073750.1	1055,46	95,7%

Select seq gb|HM000093.1|	Chrysanthemum indicum isolate SCMY tRNA-Leu (trnL) gene, partial sequence; trnL-trnF intergenic spacer, complete sequence; and tRNA-Phe (trnF) gene, partial sequence; chloroplast	1077	1077	100% 0.0	98% HM000093.1	1055,46	95,7%
Select seq gb|HM000092.1|	Chrysanthemum indicum isolate GXGL tRNA-Leu (trnL) gene, partial sequence; trnL-trnF intergenic spacer, complete sequence; and tRNA-Phe (trnF) gene, partial sequence; chloroplast	1077	1077	100% 0.0	98% HM000092.1	1055,46	95,7%
Select seq gb|JX073823.1|	Artemisia globularia tRNA-Leu (trnL) gene and trnL-trnF intergenic spacer, partial sequence; chloroplast	1075	1075	100% 0.0	98% JX073823.1	1053,50	95,5%
Select seq gb|DQ267617.1|	Achillea pseudoaleppica tRNA-Leu (trnL) gene, partial sequence; trnL-trnF intergenic spacer, complete sequence; and tRNA-Phe (trnF) gene, partial sequence; chloroplast	1075	1075	100% 0.0	98% DQ267617.1	1053,50	95,5%
Select seq gb|JX073825.1|	Artemisia potentilloides tRNA-Leu (trnL) gene and trnL-trnF intergenic spacer, partial sequence; chloroplast	1074	1074	100% 0.0	98% JX073825.1	1052,52	95,4%
Select seq gb|JX073824.1|	Artemisia copa tRNA-Leu (trnL) gene and trnL-trnF intergenic spacer, partial sequence; chloroplast	1074	1074	100% 0.0	98% JX073824.1	1052,52	95,4%
Select seq gb|JX073809.1|	Artemisia pattersonii tRNA-Leu (trnL) gene and trnL-trnF intergenic spacer, partial sequence; chloroplast	1074	1074	100% 0.0	98% JX073809.1	1052,52	95,4%
Select seq gb|JX073807.1|	Artemisia martirensis tRNA-Leu (trnL) gene and trnL-trnF intergenic spacer, partial sequence; chloroplast	1074	1074	100% 0.0	98% JX073807.1	1052,52	95,4%
Select seq gb|JX073793.1|	Artemisia campestris tRNA-Leu (trnL) gene and trnL-trnF intergenic spacer, partial sequence; chloroplast	1074	1074	100% 0.0	98% JX073793.1	1052,52	95,4%
Select seq gb|JX073773.1|	Artemisia tridentata tRNA-Leu (trnL) gene and trnL-trnF intergenic spacer, partial sequence; chloroplast	1074	1074	100% 0.0	98% JX073773.1	1052,52	95,4%
Select seq gb|JX073771.1|	Artemisia nesiotica tRNA-Leu (trnL) gene and trnL-trnF intergenic spacer, partial sequence; chloroplast	1074	1074	100% 0.0	98% JX073771.1	1052,52	95,4%
Select seq gb|JX073757.1|	Artemisia filifolia tRNA-Leu (trnL) gene and trnL-trnF intergenic spacer, partial sequence; chloroplast	1074	1074	100% 0.0	98% JX073757.1	1052,52	95,4%
Select seq gb|JX073751.1|	Artemisia tripartita tRNA-Leu (trnL) gene and trnL-trnF intergenic spacer, partial sequence; chloroplast	1074	1074	100% 0.0	98% JX073751.1	1052,52	95,4%
Select seq gb|HM000098.1|	Chrysanthemum indicum isolate FJFZ tRNA-Leu (trnL) gene, partial sequence; trnL-trnF intergenic spacer, complete sequence; and tRNA-Phe (trnF) gene, partial sequence; chloroplast	1074	1074	100% 0.0	98% HM000098.1	1052,52	95,4%
Select seq gb|HM000089.1|	Chrysanthemum indicum isolate AHHF tRNA-Leu (trnL) gene, partial sequence; trnL-trnF intergenic spacer, complete sequence; and tRNA-Phe (trnF) gene, partial sequence; chloroplast	1074	1074	100% 0.0	98% HM000089.1	1052,52	95,4%
Select seq gb|JX073756.1|	Artemisia franserioides tRNA-Leu (trnL) gene and trnL-trnF intergenic spacer, partial sequence; chloroplast	1072	1072	100% 0.0	98% JX073756.1	1050,56	95,3%

Select for downloading or viewing reports	Kh012_ITS Description	Max score	Total score	Query cover	E value	Ident	Accession	(Ident/Cover)* Max score	Deviation from top hit
Select seq gb|EF419539.1|	Althaea cannabina isolate IAca594 internal transcribed spacer 1, partial sequence; 5.8S ribosomal RNA gene, complete sequence; and internal transcribed spacer 2, partial sequence	970	970	95%	0.0	95%	EF419539.1	970,00	100,0%
Select seq gb|EF419540.1|	Althaea cannabina isolate IAca0345 internal transcribed spacer 1, partial sequence; 5.8S ribosomal RNA gene, complete sequence; and internal transcribed spacer 2, partial sequence	963	963	95%	0.0	94%	EF419540.1	952,86	98,2%
Select seq gb|EF419542.1|	Althaea armeniaca isolate IAar427 internal transcribed spacer 1, partial sequence; 5.8S ribosomal RNA gene, complete sequence; and internal transcribed spacer 2, partial sequence	961	961	95%	0.0	94%	EF419542.1	950,88	98,0%
Select seq gb|EF679734.1|	Althaea armeniaca voucher PE427 internal transcribed spacer 1, partial sequence; 5.8S ribosomal RNA gene, complete sequence; and internal transcribed spacer 2, partial sequence	957	957	95%	0.0	94%	EF679734.1	946,93	97,6%
Select seq gb|EF419536.1|	Althaea officinalis isolate IAof604 internal transcribed spacer 1, partial sequence; 5.8S ribosomal RNA gene, complete sequence; and internal transcribed spacer 2, partial sequence	942	942	95%	0.0	94%	EF419536.1	932,08	96,1%
Select seq gb|EF679733.1|	Althaea officinalis voucher PE512 internal transcribed spacer 1, partial sequence; 5.8S ribosomal RNA gene, complete sequence; and internal transcribed spacer 2, partial sequence	941	941	95%	0.0	94%	EF679733.1	931,09	96,0%
Select seq gb|EF419537.1|	Althaea officinalis isolate IAof0330 internal transcribed spacer 1, partial sequence; 5.8S ribosomal RNA gene, complete sequence; and internal transcribed spacer 2, partial sequence	937	937	95%	0.0	94%	EF419537.1	927,14	95,6%
Select seq gb|AF303026.1|	Althaea officinalis internal transcribed spacer 1, partial sequence; 5.8S ribosomal RNA gene, complete sequence; and internal transcribed spacer 2, partial sequence	965	965	98%	0.0	94%	AF303026.1	925,61	95,4%
Select seq gb|GU166770.1|	Alcea acaulis isolate Iaca01 internal transcribed spacer 1, partial sequence; 5.8S ribosomal RNA gene, complete sequence; and internal transcribed spacer 2, partial sequence	765	765	89%	0.0	90%	GU166770.1	773,60	79,8%
Select seq gb|GU166761.1|	Alcea heldreichii isolate Ihel03 internal transcribed spacer 1, partial sequence; 5.8S ribosomal RNA gene, complete sequence; and internal transcribed spacer 2, partial sequence	817	817	98%	0.0	90%	GU166761.1	750,31	77,4%
Select seq gb|GU166755.1|	Alcea apterocarpa isolate Iapt01 internal transcribed spacer 1, partial sequence; 5.8S ribosomal RNA gene, complete sequence; and internal transcribed spacer 2, partial sequence	817	817	98%	0.0	90%	GU166755.1	750,31	77,4%
Select seq gb|GU166760.1|	Alcea heldreichii isolate Ihel01 internal transcribed spacer 1, partial sequence; 5.8S ribosomal RNA gene, complete sequence; and internal transcribed spacer 2, partial sequence	815	815	98%	0.0	89%	GU166760.1	740,15	76,3%
Select seq gb|GU166768.1|	Alcea rufescens isolate Iruf01 internal transcribed spacer 1, partial sequence; 5.8S ribosomal RNA gene, complete sequence; and internal transcribed spacer 2, partial sequence	811	811	98%	0.0	89%	GU166768.1	736,52	75,9%
Select seq gb|GU166763.1|	Alcea hohenackeri isolate Ihoh02 internal transcribed spacer 1, partial sequence; 5.8S ribosomal RNA gene, complete sequence; and internal transcribed spacer 2, partial sequence	811	811	98%	0.0	89%	GU166763.1	736,52	75,9%
Select seq gb|GU166762.1|	Alcea hohenackeri isolate Ihoh01 internal transcribed spacer 1, partial sequence; 5.8S ribosomal RNA gene, complete sequence; and internal transcribed spacer 2, partial sequence	811	811	98%	0.0	89%	GU166762.1	736,52	75,9%
Select seq gb|EF679718.1|	Alcea excubita voucher PE417 internal transcribed spacer 1, partial sequence; 5.8S ribosomal RNA gene, complete sequence; and internal transcribed spacer 2, partial sequence	785	785	95%	0.0	89%	EF679718.1	735,42	75,8%
Select seq gb|EF679717.1|	Alcea aucheri voucher PE617 internal transcribed spacer 1, partial sequence; 5.8S ribosomal RNA gene, complete sequence; and internal transcribed spacer 2, partial sequence	785	785	95%	0.0	89%	EF679717.1	735,42	75,8%
Select seq gb|EF679716.1|	Alcea arbelensis voucher PE624 internal transcribed spacer 1, partial sequence; 5.8S ribosomal RNA gene, complete sequence; and internal transcribed spacer 2, partial sequence	785	785	95%	0.0	89%	EF679716.1	735,42	75,8%
Select seq gb|EF679714.1|	Alcea rosea voucher PE422 internal transcribed spacer 1, partial sequence; 5.8S ribosomal RNA gene, complete sequence; and internal transcribed spacer 2, partial sequence	785	785	95%	0.0	89%	EF679714.1	735,42	75,8%
Select seq gb|GU166767.1|	Alcea peduncularis isolate Iped01 internal transcribed spacer 1, partial sequence; 5.8S ribosomal RNA gene, complete sequence; and internal transcribed spacer 2, partial sequence	809	809	98%	0.0	89%	GU166767.1	734,70	75,7%
Select seq gb|EF419544.1|	Alcea rosea isolate Iros422 internal transcribed spacer 1, partial sequence; 5.8S ribosomal RNA gene, complete sequence; and internal transcribed spacer 2, partial sequence	784	784	95%	0.0	89%	EF419544.1	734,48	75,7%
Select seq gb|EF419543.1|	Alcea angulata isolate Iang615 internal transcribed spacer 1, partial sequence; 5.8S ribosomal RNA gene, complete sequence; and internal transcribed spacer 2, partial sequence	784	784	95%	0.0	89%	EF419543.1	734,48	75,7%
Select seq gb|GU166759.1|	Alcea glabrata isolate Igla03 internal transcribed spacer 1, partial sequence; 5.8S ribosomal RNA gene, complete sequence; and internal transcribed spacer 2, partial sequence	808	808	98%	0.0	89%	GU166759.1	733,80	75,6%
Select seq gb|GU166758.1|	Alcea glabrata isolate Igla01 internal transcribed spacer 1, partial sequence; 5.8S ribosomal RNA gene, complete sequence; and internal transcribed spacer 2, partial sequence	808	808	98%	0.0	89%	GU166758.1	733,80	75,6%
Select seq gb|GU166756.1|	Alcea apterocarpa isolate Iapt02 internal transcribed spacer 1, partial sequence; 5.8S ribosomal RNA gene, complete sequence; and internal transcribed spacer 2, partial sequence	808	808	98%	0.0	89%	GU166756.1	733,80	75,6%
Select seq gb|EF679727.1|	Alcea setosa voucher PE424 internal transcribed spacer 1, partial sequence; 5.8S ribosomal RNA gene, complete sequence; and internal transcribed spacer 2, partial sequence	782	782	95%	0.0	89%	EF679727.1	732,61	75,5%

Select seq gb|EF679725.1|	Alcea rechingeri voucher PE621 internal transcribed spacer 1, partial sequence; 5.8S ribosomal RNA gene, complete sequence; and internal transcribed spacer 2, partial sequence	782	782	95% 0.0	89% EF679725.1	732,61	75,5%
Select seq gb|GU166765.1|	Alcea lavateriflora isolate Ilav02 internal transcribed spacer 1, partial sequence; 5.8S ribosomal RNA gene, complete sequence; and internal transcribed spacer 2, partial sequence	806	806	98% 0.0	89% GU166765.1	731,98	75,5%
Select seq gb|GU166754.1|	Alcea angulata isolate Iang01 internal transcribed spacer 1, partial sequence; 5.8S ribosomal RNA gene, complete sequence; and internal transcribed spacer 2, partial sequence	806	806	98% 0.0	89% GU166754.1	731,98	75,5%
Select seq gb|EF679729.1|	Alcea sulphurea voucher PE610 internal transcribed spacer 1, partial sequence; 5.8S ribosomal RNA gene, complete sequence; and internal transcribed spacer 2, partial sequence	780	780	95% 0.0	89% EF679729.1	730,74	75,3%
Select seq gb|EF679726.1|	Alcea sachsachanica voucher PE620 internal transcribed spacer 1, partial sequence; 5.8S ribosomal RNA gene, complete sequence; and internal transcribed spacer 2, partial sequence	780	780	95% 0.0	89% EF679726.1	730,74	75,3%
Select seq gb|EF679722.1|	Alcea koelzii voucher PE609 internal transcribed spacer 1, partial sequence; 5.8S ribosomal RNA gene, complete sequence; and internal transcribed spacer 2, partial sequence	780	780	95% 0.0	89% EF679722.1	730,74	75,3%
Select seq gb|EF679721.1|	Alcea glabrata voucher PE607 internal transcribed spacer 1, partial sequence; 5.8S ribosomal RNA gene, complete sequence; and internal transcribed spacer 2, partial sequence	780	780	95% 0.0	89% EF679721.1	730,74	75,3%
Select seq gb|GU166764.1|	Alcea lavateriflora isolate Ilav01 internal transcribed spacer 1, partial sequence; 5.8S ribosomal RNA gene, complete sequence; and internal transcribed spacer 2, partial sequence	804	804	98% 0.0	89% GU166764.1	730,16	75,3%
Select seq gb|KR150170.1|	Alcea sp. Kh68 internal transcribed spacer 1, partial sequence; 5.8S ribosomal RNA gene gene, complete sequence; and internal transcribed spacer 2 region, partial sequence	745	745	91% 0.0	89% KR150170.1	728,63	75,1%
Select seq gb|EF679728.1|	Alcea schirazana voucher PE618 internal transcribed spacer 1, partial sequence; 5.8S ribosomal RNA gene, complete sequence; and internal transcribed spacer 2, partial sequence	776	776	95% 0.0	89% EF679728.1	726,99	74,9%
Select seq gb|EF419545.1|	Alcea pallida isolate IAsp0140 internal transcribed spacer 1, partial sequence; 5.8S ribosomal RNA gene, complete sequence; and internal transcribed spacer 2, partial sequence	776	776	95% 0.0	89% EF419545.1	726,99	74,9%
Select seq gb|GU166769.1|	Alcea striata isolate Istr01 internal transcribed spacer 1, partial sequence; 5.8S ribosomal RNA gene, complete sequence; and internal transcribed spacer 2, partial sequence	800	800	98% 0.0	89% GU166769.1	726,53	74,9%
Select seq gb|EF679720.1|	Alcea gorganica voucher PE611 internal transcribed spacer 1, partial sequence; 5.8S ribosomal RNA gene, complete sequence; and internal transcribed spacer 2, partial sequence	774	774	95% 0.0	89% EF679720.1	725,12	74,8%
Select seq gb|EF679715.1|	Alcea pallida voucher PE140 internal transcribed spacer 1, partial sequence; 5.8S ribosomal RNA gene, complete sequence; and internal transcribed spacer 2, partial sequence	774	774	95% 0.0	89% EF679715.1	725,12	74,8%
Select seq gb|JX017319.1|	Althaea rosea voucher 01 internal transcribed spacer 1, partial sequence; 5.8S ribosomal RNA and internal transcribed spacer 2, complete sequence; and 26S ribosomal RNA, partial sequence	754	754	93% 0.0	89% JX017319.1	721,57	74,4%
Select seq gb|KR150156.1|	Alcea sp. Kh114 internal transcribed spacer 1, partial sequence; 5.8S ribosomal RNA gene gene, complete sequence; and internal transcribed spacer 2 region, partial sequence	752	752	93% 0.0	89% KR150156.1	719,66	74,2%
Select seq gb|EF419547.1|	Anisodontea malvastroides internal transcribed spacer 1, partial sequence; 5.8S ribosomal RNA gene, complete sequence; and internal transcribed spacer 2, partial sequence	728	728	95% 0.0	88% EF419547.1	674,36	69,5%
Select seq gb|HQ632303.1|	Palaua sp. Schneider et al. 2777 voucher Schneider (et al.) 2777 (FR) clone 2777j internal transcribed spacer 1, partial sequence; 5.8S ribosomal RNA gene, complete sequence; and internal transcribed spacer 2, partial sequence	697	697	91% 0.0	88% HQ632303.1	674,02	69,5%
Select seq gb|AY172219.1|	Andeimalva machupicchensis internal transcribed spacer 1, 5.8S ribosomal RNA gene, and internal transcribed spacer 2, complete sequence	693	693	91% 0.0	88% AY172219.1	670,15	69,1%
Select seq gb|HQ632285.1|	Palaua dissecta voucher Schneider (et al.) 2978 (FR) clone 2978d internal transcribed spacer 1, partial sequence; 5.8S ribosomal RNA gene, complete sequence; and internal transcribed spacer 2, partial sequence	691	691	91% 0.0	88% HQ632285.1	668,22	68,9%
Select seq gb|HQ632284.1|	Palaua dissecta voucher Schneider (et al.) 2978 (FR) clone 2978b internal transcribed spacer 1, partial sequence; 5.8S ribosomal RNA gene, complete sequence; and internal transcribed spacer 2, partial sequence	691	691	91% 0.0	88% HQ632284.1	668,22	68,9%
Select seq gb|HQ632286.1|	Palaua sp. Schneider et al. 2777 voucher Schneider (et al.) 2777 (FR) clone 2777b internal transcribed spacer 1, partial sequence; 5.8S ribosomal RNA gene, complete sequence; and internal transcribed spacer 2, partial sequence	691	691	91% 0.0	87% HQ632286.1	660,63	68,1%
Select seq gb|DQ156306.1|	Palaua pusilla voucher Schneider & Huertas 2978 internal transcribed spacer 1, 5.8S ribosomal RNA gene, and internal transcribed spacer 2, complete sequence	691	691	91% 0.0	87% DQ156306.1	660,63	68,1%
Select seq gb|AY591833.1|	Kearnemalvastrum lacteum internal transcribed spacer 1, 5.8S ribosomal RNA gene, and internal transcribed spacer 2, complete sequence	691	691	91% 0.0	87% AY591833.1	660,63	68,1%
Select seq gb|AY172236.1|	Tarasa trisecta internal transcribed spacer 1, 5.8S ribosomal RNA gene, and internal transcribed spacer 2, complete sequence	688	688	91% 0.0	87% AY172236.1	657,76	67,8%
Select seq gb|AY172190.1|	Modiola caroliniana internal transcribed spacer 1, 5.8S ribosomal RNA gene, and internal transcribed spacer 2, complete sequence	688	688	91% 0.0	87% AY172190.1	657,76	67,8%
Select seq gb|HQ632302.1|	Palaua sp. Schneider et al. 2777 voucher Schneider (et al.) 2777 (FR) clone 2777e internal transcribed spacer 1, partial sequence; 5.8S ribosomal RNA gene, complete sequence; and internal transcribed spacer 2, partial sequence	686	686	91% 0.0	87% HQ632302.1	655,85	67,6%
Select seq gb|HQ632296.1|	Palaua dissecta voucher Schneider (et al.) 2807 (FR) internal transcribed spacer 1, partial sequence; 5.8S ribosomal RNA gene, complete sequence; and internal transcribed spacer 2, partial sequence	686	686	91% 0.0	87% HQ632296.1	655,85	67,6%

Select seq gb|HQ632292.1|	Palaua dissecta voucher Schneider (et al.) 2978 (FR) clone 2978e internal transcribed spacer 1, partial sequence; 5.8S ribosomal RNA gene, complete sequence; and internal transcribed spacer 2, partial sequence	686	686	91% 0.0	87%	HQ632292.1	655,85	67,6%
Select seq gb|DQ156308.1|	Palaua dissecta voucher Schneider & Huertas 2777 internal transcribed spacer 1, 5.8S ribosomal RNA gene, and internal transcribed spacer 2, complete sequence	686	686	91% 0.0	87%	DQ156308.1	655,85	67,6%
Select seq gb|HQ632306.1|	Palaua dissecta voucher Schneider (et al.) 2771 (FR) clone 2771e internal transcribed spacer 1, partial sequence; 5.8S ribosomal RNA gene, complete sequence; and internal transcribed spacer 2, partial sequence	684	684	91% 0.0	87%	HQ632306.1	653,93	67,4%
Select seq gb|AY172232.1|	Tarasa tenella internal transcribed spacer 1, 5.8S ribosomal RNA gene, and internal transcribed spacer 2, complete sequence	684	684	91% 0.0	87%	AY172232.1	653,93	67,4%
Select seq gb|AY172229.1|	Andeimalva spiciformis internal transcribed spacer 1, 5.8S ribosomal RNA gene, and internal transcribed spacer 2, complete sequence	684	684	91% 0.0	87%	AY172229.1	653,93	67,4%
Select seq gb|AY172191.1|	Modiolastrum lateritium internal transcribed spacer 1, 5.8S ribosomal RNA gene, and internal transcribed spacer 2, complete sequence	682	682	91% 0.0	87%	AY172191.1	652,02	67,2%
Select seq gb|HQ632369.1|	Palaua weberbaueri voucher Schneider (et al.) 3017 (FR) clone 3017j internal transcribed spacer 1, partial sequence; 5.8S ribosomal RNA gene, complete sequence; and internal transcribed spacer 2, partial sequence	680	680	91% 0.0	87%	HQ632369.1	650,11	67,0%
Select seq gb|HQ632358.1|	Palaua dissecta voucher Schneider (et al.) 2888 (FR) clone 2888g internal transcribed spacer 1, partial sequence; 5.8S ribosomal RNA gene, complete sequence; and internal transcribed spacer 2, partial sequence	680	680	91% 0.0	87%	HQ632358.1	650,11	67,0%
Select seq gb|HQ632351.1|	Palaua dissecta voucher Schneider (et al.) 2888 (FR) clone 2888a internal transcribed spacer 1, partial sequence; 5.8S ribosomal RNA gene, complete sequence; and internal transcribed spacer 2, partial sequence	680	680	91% 0.0	87%	HQ632351.1	650,11	67,0%
Select seq gb|HQ632324.1|	Palaua mollendoensis voucher Schneider (et al.) 2985 (FR) clone 2985a internal transcribed spacer 1, partial sequence; 5.8S ribosomal RNA gene, complete sequence; and internal transcribed spacer 2, partial sequence	680	680	91% 0.0	87%	HQ632324.1	650,11	67,0%
Select seq gb|HQ632308.1|	Palaua dissecta x Palaua tomentosa voucher Schneider (et al.) 2997 (FR) clone 2997e internal transcribed spacer 1, partial sequence; 5.8S ribosomal RNA gene, complete sequence; and internal transcribed spacer 2, partial sequence	680	680	91% 0.0	87%	HQ632308.1	650,11	67,0%
Select seq gb|HQ632311.1|	Palaua dissecta voucher Schneider (et al.) 2778 (FR) internal transcribed spacer 1, partial sequence; 5.8S ribosomal RNA gene, complete sequence; and internal transcribed spacer 2, partial sequence	680	680	91% 0.0	87%	HQ632311.1	650,11	67,0%
Select seq gb|HQ632300.1|	Palaua sp. Schneider et al. 2777 voucher Schneider (et al.) 2777 (FR) clone 2777c internal transcribed spacer 1, partial sequence; 5.8S ribosomal RNA gene, complete sequence; and internal transcribed spacer 2, partial sequence	680	680	91% 0.0	87%	HQ632300.1	650,11	67,0%
Select seq gb|HQ632269.1|	Palaua cf. dissecta Schneider et al. 2802 voucher Schneider (et al.) 2802 (FR) clone 2802a internal transcribed spacer 1, partial sequence; 5.8S ribosomal RNA gene, complete sequence; and internal transcribed spacer 2, partial sequence	680	680	91% 0.0	87%	HQ632269.1	650,11	67,0%
Select seq gb|EU784123.1|	Palaua dissecta voucher Schneider & Huertas 2888 internal transcribed spacer 1, 5.8S ribosomal RNA gene, and internal transcribed spacer 2, complete sequence	680	680	91% 0.0	87%	EU784123.1	650,11	67,0%
Select seq gb|DQ156315.1|	Palaua camanensis voucher FLSP 211 internal transcribed spacer 1, 5.8S ribosomal RNA gene, and internal transcribed spacer 2, complete sequence	680	680	91% 0.0	87%	DQ156315.1	650,11	67,0%
Select seq gb|AY217208.1|	Tarasa tenella internal transcribed spacer 1, 5.8S ribosomal RNA gene, and internal transcribed spacer 2, complete sequence	680	680	91% 0.0	87%	AY217208.1	650,11	67,0%
Select seq gb|AY172220.1|	Andeimalva mandonii internal transcribed spacer 1, 5.8S ribosomal RNA gene, and internal transcribed spacer 2, complete sequence	680	680	91% 0.0	87%	AY172220.1	650,11	67,0%
Select seq gb|AY591845.1|	Palaua camanensis internal transcribed spacer 1, 5.8S ribosomal RNA gene, and internal transcribed spacer 2, complete sequence	680	680	91% 0.0	87%	AY591845.1	650,11	67,0%
Select seq gb|AY172237.1|	Tarasa urbaniana internal transcribed spacer 1, 5.8S ribosomal RNA gene, and internal transcribed spacer 2, complete sequence	678	678	91% 0.0	87%	AY172237.1	648,20	66,8%
Select seq gb|AY172227.1|	Tarasa pediculata internal transcribed spacer 1, 5.8S ribosomal RNA gene, and internal transcribed spacer 2, complete sequence	678	678	91% 0.0	87%	AY172227.1	648,20	66,8%
Select seq gb|AY591820.1|	Calyculogygas uruguayensis internal transcribed spacer 1, 5.8S ribosomal RNA gene, and internal transcribed spacer 2, complete sequence	678	678	91% 0.0	87%	AY591820.1	648,20	66,8%
Select seq gb|HQ632359.1|	Palaua weberbaueri voucher Schneider (et al.) 3017 (FR) clone 3017g internal transcribed spacer 1, partial sequence; 5.8S ribosomal RNA gene, complete sequence; and internal transcribed spacer 2, partial sequence	675	675	91% 0.0	87%	HQ632359.1	645,33	66,5%
Select seq gb|HQ632356.1|	Palaua dissecta voucher Schneider (et al.) 2888 (FR) clone 2888d internal transcribed spacer 1, partial sequence; 5.8S ribosomal RNA gene, complete sequence; and internal transcribed spacer 2, partial sequence	675	675	91% 0.0	87%	HQ632356.1	645,33	66,5%
Select seq gb|HQ632355.1|	Palaua mollendoensis voucher Schneider (et al.) 2813 (FR) clone 2813d internal transcribed spacer 1, partial sequence; 5.8S ribosomal RNA gene, complete sequence; and internal transcribed spacer 2, partial sequence	675	675	91% 0.0	87%	HQ632355.1	645,33	66,5%
Select seq gb|HQ632348.1|	Palaua mollendoensis voucher Schneider (et al.) 2985 (FR) clone 2985j internal transcribed spacer 1, partial sequence; 5.8S ribosomal RNA gene, complete sequence; and internal transcribed spacer 2, partial sequence	675	675	91% 0.0	87%	HQ632348.1	645,33	66,5%
Select seq gb|HQ632344.1|	Palaua dissecta voucher Schneider (et al.) 2888 (FR) clone 2888e internal transcribed spacer 1, partial sequence; 5.8S ribosomal RNA gene, complete sequence; and internal transcribed spacer 2, partial sequence	675	675	91% 0.0	87%	HQ632344.1	645,33	66,5%
Select seq gb|HQ632343.1|	Palaua dissecta voucher Schneider (et al.) 2836 (FR) clone 2836d internal transcribed spacer 1, partial sequence; 5.8S ribosomal RNA gene, complete sequence; and internal transcribed spacer 2, partial sequence	675	675	91% 0.0	87%	HQ632343.1	645,33	66,5%

Select seq gb|HQ632342.1|	Palaua dissecta voucher Schneider (et al.) 2771 (FR) clone 2771h internal transcribed spacer 1, partial sequence; 5.8S ribosomal RNA gene, complete sequence; and internal transcribed spacer 2, partial sequence	675	675	91% 0.0	87% HQ632342.1	645,33	66,5%
Select seq gb|HQ632340.1|	Palaua mollendoensis voucher Schneider (et al.) 2985 (FR) clone 2985k internal transcribed spacer 1, partial sequence; 5.8S ribosomal RNA gene, complete sequence; and internal transcribed spacer 2, partial sequence	675	675	91% 0.0	87% HQ632340.1	645,33	66,5%
Select seq gb|HQ632315.1|	Palaua mollendoensis voucher Schneider (et al.) 2985 (FR) clone 2985g internal transcribed spacer 1, partial sequence; 5.8S ribosomal RNA gene, complete sequence; and internal transcribed spacer 2, partial sequence	675	675	91% 0.0	87% HQ632315.1	645,33	66,5%
Select seq gb|HQ632335.1|	Palaua dissecta voucher Schneider (et al.) 2836 (FR) clone 2836g internal transcribed spacer 1, partial sequence; 5.8S ribosomal RNA gene, complete sequence; and internal transcribed spacer 2, partial sequence	675	675	91% 0.0	87% HQ632335.1	645,33	66,5%
Select seq gb|HQ632333.1|	Palaua dissecta voucher Schneider (et al.) 2836 (FR) clone 2836c internal transcribed spacer 1, partial sequence; 5.8S ribosomal RNA gene, complete sequence; and internal transcribed spacer 2, partial sequence	675	675	91% 0.0	87% HQ632333.1	645,33	66,5%
Select seq gb|HQ632328.1|	Palaua dissecta voucher Schneider (et al.) 2771 (FR) clone 2771f internal transcribed spacer 1, partial sequence; 5.8S ribosomal RNA gene, complete sequence; and internal transcribed spacer 2, partial sequence	675	675	91% 0.0	87% HQ632328.1	645,33	66,5%
Select seq gb|HQ632322.1|	Palaua dissecta x Palaua tomentosa voucher Schneider (et al.) 2997 (FR) clone 2997g internal transcribed spacer 1, partial sequence; 5.8S ribosomal RNA gene, complete sequence; and internal transcribed spacer 2, partial sequence	675	675	91% 0.0	87% HQ632322.1	645,33	66,5%
Select seq gb|HQ632318.1|	Palaua mollendoensis voucher Schneider (et al.) 2813 (FR) clone 2813b internal transcribed spacer 1, partial sequence; 5.8S ribosomal RNA gene, complete sequence; and internal transcribed spacer 2, partial sequence	675	675	91% 0.0	87% HQ632318.1	645,33	66,5%
Select seq gb|HQ632295.1|	Palaua dissecta voucher Schneider (et al.) 2978 (FR) clone 2978j internal transcribed spacer 1, partial sequence; 5.8S ribosomal RNA gene, complete sequence; and internal transcribed spacer 2, partial sequence	675	675	91% 0.0	87% HQ632295.1	645,33	66,5%
Select seq gb|HQ632279.1|	Palaua camanensis voucher Schneider (et al.) FLSP 211 (FR) clone FLSP211-g internal transcribed spacer 1, partial sequence; 5.8S ribosomal RNA gene, complete sequence; and internal transcribed spacer 2, partial sequence	675	675	91% 0.0	87% HQ632279.1	645,33	66,5%
Select seq gb|HQ632271.1|	Palaua dissecta x Palaua tomentosa voucher Schneider (et al.) 3002 (FR) clone 3002e internal transcribed spacer 1, partial sequence; 5.8S ribosomal RNA gene, complete sequence; and internal transcribed spacer 2, partial sequence	675	675	91% 0.0	87% HQ632271.1	645,33	66,5%
Select seq gb|HQ632158.1|	Palaua cf. dissecta Schneider et al. 2802 voucher Schneider (et al.) 2802 (FR) clone 2802k internal transcribed spacer 1, partial sequence; 5.8S ribosomal RNA gene, complete sequence; and internal transcribed spacer 2, partial sequence	675	675	91% 0.0	87% HQ632158.1	645,33	66,5%
Select seq gb|HQ632122.1|	Palaua dissecta voucher Schneider (et al.) 3093 (FR) clone 3093e internal transcribed spacer 1, partial sequence; 5.8S ribosomal RNA gene, complete sequence; and internal transcribed spacer 2, partial sequence	675	675	91% 0.0	87% HQ632122.1	645,33	66,5%
Select seq gb|HQ632107.1|	Palaua dissecta voucher Schneider (et al.) 2836 (FR) clone 2836b internal transcribed spacer 1, partial sequence; 5.8S ribosomal RNA gene, complete sequence; and internal transcribed spacer 2, partial sequence	675	675	91% 0.0	87% HQ632107.1	645,33	66,5%
Select seq gb|HQ632310.1|	Palaua dissecta voucher Schneider (et al.) 2824a (FR) internal transcribed spacer 1, partial sequence; 5.8S ribosomal RNA gene, complete sequence; and internal transcribed spacer 2, partial sequence	673	673	91% 0.0	87% HQ632310.1	643,42	66,3%
Select seq gb|DQ826561.1|	Palaua mollendoensis voucher Schneider & Huertas 2985 internal transcribed spacer 1, 5.8S ribosomal RNA gene, and internal transcribed spacer 2, complete sequence	673	673	91% 0.0	87% DQ826561.1	643,42	66,3%
Select seq gb|FJ204686.1|	Tarasa tenella voucher Krapovickas and Seijo 47838 (TEX) internal transcribed spacer 1, 5.8S ribosomal RNA gene, and internal transcribed spacer 2, complete sequence	678	678	92% 0.0	87% FJ204686.1	641,15	66,1%

Select for downloading or viewing reports	Kh016_ITS Description	Max score	Total score	Query cover	E value	Ident	Accession	(Ident/Cover)* Max score	Deviation from top hit
Select seq gb|EU374715.1|	Thymus pubescens 18S ribosomal RNA gene, partial sequence; internal transcribed spacer 1, 5.8S ribosomal RNA gene, and internal transcribed spacer 2, complete sequence; and 25/28S ribosomal RNA gene, partial sequence	1140	1140	98%	0.0	100%	EU374715.1	1163,27	100,0%
Select seq gb|EU735059.1|	Thymus trautvetteri 18S ribosomal RNA gene, partial sequence; internal transcribed spacer 1, 5.8S ribosomal RNA gene, and internal transcribed spacer 2, complete sequence; and 28S ribosomal RNA gene, partial sequence	1157	1157	100%	0.0	100%	EU735059.1	1157,00	99,5%
Select seq gb|EU735058.1|	Thymus persicus isolate Ardabil 18S ribosomal RNA gene, partial sequence; internal transcribed spacer 1, 5.8S ribosomal RNA gene, and internal transcribed spacer 2, complete sequence; and 28S ribosomal RNA gene, partial sequence	1157	1157	100%	0.0	100%	EU735058.1	1157,00	99,5%
Select seq gb|AY029168.1|	Thymus mastichina internal transcribed spacer 1, 5.8S ribosomal RNA gene, and internal transcribed spacer 2, complete sequence	1122	1122	98%	0.0	99%	AY029168.1	1133,45	97,4%
Select seq gb|EU556509.1|	Thymus mongolicus isolate G2 internal transcribed spacer 1, partial sequence; 5.8S ribosomal RNA gene, complete sequence; and internal transcribed spacer 2, partial sequence	1133	1133	99%	0.0	99%	EU556509.1	1133,00	97,4%
Select seq gb|GU381459.1|	Thymus serpyllum voucher M:Braeuchler 2514 internal transcribed spacer 1, partial sequence; 5.8S ribosomal RNA gene, complete sequence; and internal transcribed spacer 2, partial sequence	1109	1109	97%	0.0	99%	GU381459.1	1131,87	97,3%
Select seq gb|EU556510.1|	Thymus mongolicus isolate G3 internal transcribed spacer 1, partial sequence; 5.8S ribosomal RNA gene, complete sequence; and internal transcribed spacer 2, partial sequence	1085	1085	95%	0.0	99%	EU556510.1	1130,68	97,2%
Select seq gb|EU556508.1|	Thymus mongolicus isolate G1 internal transcribed spacer 1, partial sequence; 5.8S ribosomal RNA gene, complete sequence; and internal transcribed spacer 2, partial sequence	1116	1116	98%	0.0	99%	EU556508.1	1127,39	96,9%
Select seq gb|EU556521.1|	Thymus mongolicus isolate XM internal transcribed spacer 1, partial sequence; 5.8S ribosomal RNA gene, complete sequence; and internal transcribed spacer 2, partial sequence	1079	1079	95%	0.0	99%	EU556521.1	1124,43	96,7%
Select seq gb|AY443448.1|	Thymus magnus JUNG-TM04 internal transcribed spacer 1, 5.8S ribosomal RNA and internal transcribed spcer 2 genes, complete sequence	1123	1123	99%	0.0	99%	AY443448.1	1123,00	96,5%
Select seq gb|AY443434.1|	Thymus quinquecostatus JUNG-TQ01 internal transcribed spacer 1, 5.8S ribosomal RNA and internal transcribed spcer 2 genes, complete sequence	1123	1123	99%	0.0	99%	AY443434.1	1123,00	96,5%
Select seq gb|EU556524.1|	Thymus quinquecostatus isolate ZY internal transcribed spacer 1, partial sequence; 5.8S ribosomal RNA gene, complete sequence; and internal transcribed spacer 2, partial sequence	1134	1134	100%	0.0	99%	EU556524.1	1122,66	96,5%
Select seq gb|EU556522.1|	Thymus quinquecostatus isolate ZJ internal transcribed spacer 1, partial sequence; 5.8S ribosomal RNA gene, complete sequence; and internal transcribed spacer 2, partial sequence	1134	1134	100%	0.0	99%	EU556522.1	1122,66	96,5%
Select seq gb|EU556520.1|	Thymus quinquecostatus isolate XZ internal transcribed spacer 1, partial sequence; 5.8S ribosomal RNA gene, complete sequence; and internal transcribed spacer 2, partial sequence	1134	1134	100%	0.0	99%	EU556520.1	1122,66	96,5%
Select seq gb|EU556519.1|	Thymus mongolicus isolate S2 internal transcribed spacer 1, partial sequence; 5.8S ribosomal RNA gene, complete sequence; and internal transcribed spacer 2, partial sequence	1134	1134	100%	0.0	99%	EU556519.1	1122,66	96,5%
Select seq gb|EU556518.1|	Thymus mongolicus isolate S1 internal transcribed spacer 1, partial sequence; 5.8S ribosomal RNA gene, complete sequence; and internal transcribed spacer 2, partial sequence	1134	1134	100%	0.0	99%	EU556518.1	1122,66	96,5%
Select seq gb|EU556517.1|	Thymus quinquecostatus isolate N2 internal transcribed spacer 1, partial sequence; 5.8S ribosomal RNA gene, complete sequence; and internal transcribed spacer 2, partial sequence	1134	1134	100%	0.0	99%	EU556517.1	1122,66	96,5%
Select seq gb|EU556516.1|	Thymus quinquecostatus isolate N1 internal transcribed spacer 1, partial sequence; 5.8S ribosomal RNA gene, complete sequence; and internal transcribed spacer 2, partial sequence	1134	1134	100%	0.0	99%	EU556516.1	1122,66	96,5%
Select seq gb|EU556511.1|	Thymus dahuricus isolate HD internal transcribed spacer 1, partial sequence; 5.8S ribosomal RNA gene, complete sequence; and internal transcribed spacer 2, partial sequence	1134	1134	100%	0.0	99%	EU556511.1	1122,66	96,5%
Select seq gb|EU556506.1|	Thymus quinquecostatus isolate D1 internal transcribed spacer 1, partial sequence; 5.8S ribosomal RNA gene, complete sequence; and internal transcribed spacer 2, partial sequence	1134	1134	100%	0.0	99%	EU556506.1	1122,66	96,5%
Select seq gb|FJ236468.1|	Thymus persicus isolate Tabriz 18S ribosomal RNA gene, partial sequence; internal transcribed spacer 1, 5.8S ribosomal RNA gene, and internal transcribed spacer 2, complete sequence; and 28S ribosomal RNA gene, partial sequence	1134	1134	100%	0.0	99%	FJ236468.1	1122,66	96,5%
Select seq gb|DQ667242.1|	Thymus serpyllum isolate x075 18S ribosomal RNA gene, partial sequence; internal transcribed spacer 1, 5.8S ribosomal RNA gene, and internal transcribed spacer 2, complete sequence; and 28S ribosomal RNA gene, partial sequence	1134	1134	100%	0.0	99%	DQ667242.1	1122,66	96,5%
Select seq gb|AY443441.1|	Thymus quinquecostatus JUNG-TQ08 internal transcribed spacer 1, 5.8S ribosomal RNA and internal transcribed spcer 2 genes, complete sequence	1107	1107	98%	0.0	99%	AY443441.1	1118,30	96,1%
Select seq gb|AY443445.1|	Thymus magnus JUNG-TM01 internal transcribed spacer 1, 5.8S ribosomal RNA and internal transcribed spcer 2 genes, complete sequence	1118	1118	99%	0.0	99%	AY443445.1	1118,00	96,1%
Select seq gb|EU556507.1|	Thymus quinquecostatus isolate CL internal transcribed spacer 1, partial sequence; 5.8S ribosomal RNA gene, complete sequence; and internal transcribed spacer 2, partial sequence	1129	1129	100%	0.0	99%	EU556507.1	1117,71	96,1%
Select seq gb|JQ669138.1|	Thymus pulegioides voucher Riina 1577 18S ribosomal RNA gene, internal transcribed spacer 1, 5.8S ribosomal RNA gene, internal transcribed spacer 2, and 26S ribosomal RNA gene, region	1125	1125	100%	0.0	99%	JQ669138.1	1113,75	95,7%

Select seq gb|AY443447.1|	Thymus magnus JUNG-TM03 internal transcribed spacer 1, 5.8S ribosomal RNA and internal transcribed spcer 2 genes, complete sequence	1112	1112	99%	0.0	99%	AY443447.1	1112,00	95,6%
Select seq gb|AY443438.1|	Thymus quinquecostatus JUNG-TQ05 internal transcribed spacer 1, 5.8S ribosomal RNA and internal transcribed spcer 2 genes, complete sequence	1112	1112	99%	0.0	99%	AY443438.1	1112,00	95,6%
Select seq gb|EU796890.1|	Thymus serpyllum 18S ribosomal RNA gene, partial sequence; internal transcribed spacer 1, 5.8S ribosomal RNA gene, and internal transcribed spacer 2, complete sequence; and 26S ribosomal RNA gene, partial sequence	1123	1123	100%	0.0	99%	EU796890.1	1111,77	95,6%
Select seq gb|EU785939.1|	Thymus vulgaris 18S ribosomal RNA gene, partial sequence; internal transcribed spacer 1, 5.8S ribosomal RNA gene, and internal transcribed spacer 2, complete sequence; and 26S ribosomal RNA gene, partial sequence	1120	1120	100%	0.0	99%	EU785939.1	1108,80	95,3%
Select seq gb|GU381458.1|	Thymus broussonetii subsp. hannonis voucher MSB:Podlech 142 internal transcribed spacer 1, partial sequence; 5.8S ribosomal RNA gene, complete sequence; and internal transcribed spacer 2, partial sequence	1085	1085	97%	0.0	99%	GU381458.1	1107,37	95,2%
Select seq gb|AY443444.1|	Thymus quinquecostatus JUNG-TQ11 internal transcribed spacer 1, 5.8S ribosomal RNA and internal transcribed spcer 2 genes, complete sequence	1107	1107	99%	0.0	99%	AY443444.1	1107,00	95,2%
Select seq gb|AY443443.1|	Thymus quinquecostatus JUNG-TQ10 internal transcribed spacer 1, 5.8S ribosomal RNA and internal transcribed spcer 2 genes, complete sequence	1107	1107	99%	0.0	99%	AY443443.1	1107,00	95,2%
Select seq gb|AY443442.1|	Thymus quinquecostatus JUNG-TQ09 internal transcribed spacer 1, 5.8S ribosomal RNA and internal transcribed spcer 2 genes, complete sequence	1107	1107	99%	0.0	99%	AY443442.1	1107,00	95,2%
Select seq gb|AY443437.1|	Thymus quinquecostatus JUNG-TQ04 internal transcribed spacer 1, 5.8S ribosomal RNA and internal transcribed spcer 2 genes, complete sequence	1107	1107	99%	0.0	99%	AY443437.1	1107,00	95,2%
Select seq gb|AY443436.1|	Thymus quinquecostatus JUNG-TQ03 internal transcribed spacer 1, 5.8S ribosomal RNA and internal transcribed spcer 2 genes, complete sequence	1107	1107	99%	0.0	99%	AY443436.1	1107,00	95,2%
Select seq gb|EU556523.1|	Thymus marschallianus isolate XY internal transcribed spacer 1, partial sequence; 5.8S ribosomal RNA gene, complete sequence; and internal transcribed spacer 2, partial sequence	1112	1112	100%	0.0	99%	EU556523.1	1100,88	94,6%
Select seq gb|AY443446.1|	Thymus magnus JUNG-TM02 internal transcribed spacer 1, 5.8S ribosomal RNA and internal transcribed spcer 2 genes, complete sequence	1096	1096	99%	0.0	99%	AY443446.1	1096,00	94,2%
Select seq gb|AY443440.1|	Thymus quinquecostatus JUNG-TQ07 internal transcribed spacer 1, 5.8S ribosomal RNA and internal transcribed spcer 2 genes, complete sequence	1096	1096	99%	0.0	99%	AY443440.1	1096,00	94,2%
Select seq gb|AY443435.1|	Thymus quinquecostatus JUNG-TQ02 internal transcribed spacer 1, 5.8S ribosomal RNA and internal transcribed spcer 2 genes, complete sequence	1090	1090	99%	0.0	98%	AY443435.1	1078,99	92,8%
Select seq gb|EU556512.1|	Thymus mandschuricus isolate HM internal transcribed spacer 1, partial sequence; 5.8S ribosomal RNA gene, complete sequence; and internal transcribed spacer 2, partial sequence	1070	1070	100%	0.0	98%	EU556512.1	1048,60	90,1%
Select seq gb|AY329369.1|	Thymus vulgaris internal transcribed spacer 1, partial sequence; 5.8S ribosomal RNA gene, complete sequence; and internal transcribed spacer 2, partial sequence	1064	1064	100%	0.0	97%	AY329369.1	1032,08	88,7%
Select seq gb|AY506646.1|	Thymus vulgaris internal transcribed spacer 1, partial sequence; 5.8S ribosomal RNA gene, complete sequence; and internal transcribed spacer 2, partial sequence	1061	1061	100%	0.0	97%	AY506646.1	1029,17	88,5%
Select seq gb|EU556515.1|	Thymus amurensis isolate HW internal transcribed spacer 1, partial sequence; 5.8S ribosomal RNA gene, complete sequence; and internal transcribed spacer 2, partial sequence	1053	1053	100%	0.0	97%	EU556515.1	1021,41	87,8%
Select seq gb|EU556514.1|	Thymus quinquecostatus isolate HY internal transcribed spacer 1, partial sequence; 5.8S ribosomal RNA gene, complete sequence; and internal transcribed spacer 2, partial sequence	939	939	91%	0.0	97%	EU556514.1	1000,91	86,0%
Select seq gb|EU556513.1|	Thymus przewalskii isolate HX internal transcribed spacer 1, partial sequence; 5.8S ribosomal RNA gene, complete sequence; and internal transcribed spacer 2, partial sequence	944	944	92%	0.0	96%	EU556513.1	985,04	84,7%
Select seq gb|GU381460.1|	Thymus saturejoides subsp. commutatus voucher M:Podlech 47906 internal transcribed spacer 1, partial sequence; 5.8S ribosomal RNA gene, complete sequence; and internal transcribed spacer 2, partial sequence	963	963	97%	0.0	95%	GU381460.1	943,14	81,1%
Select seq gb|GU381462.1|	Saccocalyx saturejoides voucher MSB:Faurel 5650 internal transcribed spacer 1, partial sequence; 5.8S ribosomal RNA gene, complete sequence; and internal transcribed spacer 2, partial sequence	952	952	97%	0.0	95%	GU381462.1	932,37	80,2%
Select seq gb|GU381457.1|	Thymus caespititius voucher M:Heubl s.n. internal transcribed spacer 1, partial sequence; 5.8S ribosomal RNA gene, complete sequence; and internal transcribed spacer 2, partial sequence	952	952	97%	0.0	95%	GU381457.1	932,37	80,2%
Select seq gb|GU381461.1|	Argantoniella salzmannii voucher M:Barra et al. 2673GL internal transcribed spacer 1, partial sequence; 5.8S ribosomal RNA gene, complete sequence; and internal transcribed spacer 2, partial sequence	946	946	97%	0.0	95%	GU381461.1	926,49	79,6%
Select seq gb|GU381451.1|	Thymbra sintenisii subsp. isaurica voucher E:Goener 12628 internal transcribed spacer 1, partial sequence; 5.8S ribosomal RNA gene, complete sequence; and internal transcribed spacer 2, partial sequence	913	913	97%	0.0	94%	GU381451.1	884,76	76,1%
Select seq gb|GU381452.1|	Thymbra calostachya voucher M:Ulrich s.n. internal transcribed spacer 1, partial sequence; 5.8S ribosomal RNA gene, complete sequence; and internal transcribed spacer 2, partial sequence	907	907	97%	0.0	94%	GU381452.1	878,95	75,6%
Select seq gb|GU381455.1|	Satureja linearifolia voucher M:Brullo & Furnari s.n. internal transcribed spacer 1, partial sequence; 5.8S ribosomal RNA gene, complete sequence; and internal transcribed spacer 2, partial sequence	905	905	97%	0.0	94%	GU381455.1	877,01	75,4%
Select seq gb|JQ669137.1|	Thymbra capitata voucher UCBG 96.0817 s.n. 18S ribosomal RNA gene, internal transcribed spacer 1, 5.8S ribosomal RNA gene, internal transcribed spacer 2, and 26S ribosomal RNA gene, region	924	924	100%	0.0	93%	JQ669137.1	859,32	73,9%

Select seq gb|GU381453.1|	Thymbra capitata voucher M:Braeuchler 2518 internal transcribed spacer 1, partial sequence; 5.8S ribosomal RNA gene, complete sequence; and internal transcribed spacer 2, partial sequence	894	894	97% 0.0	93% GU381453.1	857,13	73,7%
Select seq gb|GU381466.1|	Origanum dayi voucher MSB:Liston 7-85-3931 internal transcribed spacer 1, partial sequence; 5.8S ribosomal RNA gene, complete sequence; and internal transcribed spacer 2, partial sequence	874	874	95% 0.0	93% GU381466.1	855,60	73,6%
Select seq gb|GU381454.1|	Satureja thymbrifolia voucher M:Danin s.n. internal transcribed spacer 1, partial sequence; 5.8S ribosomal RNA gene, complete sequence; and internal transcribed spacer 2, partial sequence	889	889	97% 0.0	93% GU381454.1	852,34	73,3%
Select seq gb|JQ669127.1|	Origanum vulgare voucher B. Drew 77 18S ribosomal RNA gene, internal transcribed spacer 1, 5.8S ribosomal RNA gene, internal transcribed spacer 2, and 26S ribosomal RNA gene, region	894	894	98% 0.0	93% JQ669127.1	848,39	72,9%
Select seq gb|JX162834.1|	Origanum majorana voucher OBI clone 3 internal transcribed spacer 1, partial sequence; 5.8S ribosomal RNA gene, complete sequence; and internal transcribed spacer 2, partial sequence	883	883	98% 0.0	93% JX162834.1	837,95	72,0%
Select seq gb|JX162831.1|	Origanum majorana voucher Mira2-3 clone 10 internal transcribed spacer 1, partial sequence; 5.8S ribosomal RNA gene, complete sequence; and internal transcribed spacer 2, partial sequence	883	883	98% 0.0	93% JX162831.1	837,95	72,0%
Select seq gb|GU381463.1|	Origanum rotundifolium voucher M:Braeuchler 2517 internal transcribed spacer 1, partial sequence; 5.8S ribosomal RNA gene, complete sequence; and internal transcribed spacer 2, partial sequence	872	872	97% 0.0	93% GU381463.1	836,04	71,9%
Select seq gb|JX163036.1|	Origanum syriacum voucher LC7 clone 8 internal transcribed spacer 1, partial sequence; 5.8S ribosomal RNA gene, complete sequence; and internal transcribed spacer 2, partial sequence	880	880	98% 0.0	93% JX163036.1	835,10	71,8%
Select seq gb|JX162836.1|	Origanum majorana voucher OBI clone 5 internal transcribed spacer 1, partial sequence; 5.8S ribosomal RNA gene, complete sequence; and internal transcribed spacer 2, partial sequence	880	880	98% 0.0	93% JX162836.1	835,10	71,8%
Select seq gb|DQ667243.1|	Origanum vulgare isolate x076 18S ribosomal RNA gene, partial sequence; internal transcribed spacer 1, 5.8S ribosomal RNA gene, and internal transcribed spacer 2, complete sequence; and 28S ribosomal RNA gene, partial sequence	880	880	98% 0.0	93% DQ667243.1	835,10	71,8%
Select seq gb|JX162837.1|	Origanum majorana voucher OBI clone 6 internal transcribed spacer 1, partial sequence; 5.8S ribosomal RNA gene, complete sequence; and internal transcribed spacer 2, partial sequence	878	878	98% 0.0	93% JX162837.1	833,20	71,6%
Select seq gb|JX162826.1|	Origanum majorana voucher Mira2-3 clone 4 internal transcribed spacer 1, partial sequence; 5.8S ribosomal RNA gene, complete sequence; and internal transcribed spacer 2, partial sequence	878	878	98% 0.0	93% JX162826.1	833,20	71,6%
Select seq gb|JX162816.1|	Origanum majorana voucher SR562 clone 3 internal transcribed spacer 1, partial sequence; 5.8S ribosomal RNA gene, complete sequence; and internal transcribed spacer 2, partial sequence	878	878	98% 0.0	93% JX162816.1	833,20	71,6%
Select seq gb|JX162814.1|	Origanum majorana voucher SR562 clone 1 internal transcribed spacer 1, partial sequence; 5.8S ribosomal RNA gene, complete sequence; and internal transcribed spacer 2, partial sequence	878	878	98% 0.0	93% JX162814.1	833,20	71,6%
Select seq gb|JX162807.1|	Origanum majorana voucher LC10 clone 4 internal transcribed spacer 1, partial sequence; 5.8S ribosomal RNA gene, complete sequence; and internal transcribed spacer 2, partial sequence	878	878	98% 0.0	93% JX162807.1	833,20	71,6%
Select seq gb|JX162805.1|	Origanum majorana voucher LC10 clone 2 internal transcribed spacer 1, partial sequence; 5.8S ribosomal RNA gene, complete sequence; and internal transcribed spacer 2, partial sequence	878	878	98% 0.0	93% JX162805.1	833,20	71,6%
Select seq gb|JX162830.1|	Origanum majorana voucher Mira2-3 clone 9 internal transcribed spacer 1, partial sequence; 5.8S ribosomal RNA gene, complete sequence; and internal transcribed spacer 2, partial sequence	874	874	98% 0.0	92% JX162830.1	820,49	70,5%
Select seq gb|JX163016.1|	Origanum syriacum voucher RNG:H50 clone 8 internal transcribed spacer 1, partial sequence; 5.8S ribosomal RNA gene, complete sequence; and internal transcribed spacer 2, partial sequence	872	872	98% 0.0	92% JX163016.1	818,61	70,4%
Select seq gb|JX162832.1|	Origanum majorana voucher OBI clone 1 internal transcribed spacer 1, partial sequence; 5.8S ribosomal RNA gene, complete sequence; and internal transcribed spacer 2, partial sequence	872	872	98% 0.0	92% JX162832.1	818,61	70,4%
Select seq gb|JX162829.1|	Origanum majorana voucher Mira2-3 clone 8 internal transcribed spacer 1, partial sequence; 5.8S ribosomal RNA gene, complete sequence; and internal transcribed spacer 2, partial sequence	872	872	98% 0.0	92% JX162829.1	818,61	70,4%
Select seq gb|JX162823.1|	Origanum majorana voucher Mira2-3 clone 1 internal transcribed spacer 1, partial sequence; 5.8S ribosomal RNA gene, complete sequence; and internal transcribed spacer 2, partial sequence	872	872	98% 0.0	92% JX162823.1	818,61	70,4%
Select seq gb|JX162818.1|	Origanum majorana voucher SR562 clone 5 internal transcribed spacer 1, partial sequence; 5.8S ribosomal RNA gene, complete sequence; and internal transcribed spacer 2, partial sequence	872	872	98% 0.0	92% JX162818.1	818,61	70,4%
Select seq gb|JX162815.1|	Origanum majorana voucher SR562 clone 2 internal transcribed spacer 1, partial sequence; 5.8S ribosomal RNA gene, complete sequence; and internal transcribed spacer 2, partial sequence	872	872	98% 0.0	92% JX162815.1	818,61	70,4%
Select seq gb|JX163024.1|	Origanum syriacum voucher RNG:H50 clone 16 internal transcribed spacer 1, partial sequence; 5.8S ribosomal RNA gene, complete sequence; and internal transcribed spacer 2, partial sequence	869	869	98% 0.0	92% JX163024.1	815,80	70,1%
Select seq gb|JX163023.1|	Origanum syriacum voucher RNG:H50 clone 15 internal transcribed spacer 1, partial sequence; 5.8S ribosomal RNA gene, complete sequence; and internal transcribed spacer 2, partial sequence	869	869	98% 0.0	92% JX163023.1	815,80	70,1%
Select seq gb|JX163022.1|	Origanum syriacum voucher RNG:H50 clone 14 internal transcribed spacer 1, partial sequence; 5.8S ribosomal RNA gene, complete sequence; and internal transcribed spacer 2, partial sequence	869	869	98% 0.0	92% JX163022.1	815,80	70,1%
Select seq gb|JX163021.1|	Origanum syriacum voucher RNG:H50 clone 13 internal transcribed spacer 1, partial sequence; 5.8S ribosomal RNA gene, complete sequence; and internal transcribed spacer 2, partial sequence	869	869	98% 0.0	92% JX163021.1	815,80	70,1%
Select seq gb|JX163014.1|	Origanum syriacum voucher RNG:H50 clone 6 internal transcribed spacer 1, partial sequence; 5.8S ribosomal RNA gene, complete sequence; and internal transcribed spacer 2, partial sequence	869	869	98% 0.0	92% JX163014.1	815,80	70,1%

Select seq gb|JX163010.1|	Origanum syriacum voucher RNG:H50 clone 2 internal transcribed spacer 1, partial sequence; 5.8S ribosomal RNA gene, complete sequence; and internal transcribed spacer 2, partial sequence	869	869	98%	0.0	92%	JX163010.1	815,80	70,1%
Select seq gb|JX162824.1|	Origanum majorana voucher Mira2-3 clone 2 internal transcribed spacer 1, partial sequence; 5.8S ribosomal RNA gene, complete sequence; and internal transcribed spacer 2, partial sequence	869	869	98%	0.0	92%	JX162824.1	815,80	70,1%
Select seq gb|JX163019.1|	Origanum syriacum voucher RNG:H50 clone 11 internal transcribed spacer 1, partial sequence; 5.8S ribosomal RNA gene, complete sequence; and internal transcribed spacer 2, partial sequence	867	867	98%	0.0	92%	JX163019.1	813,92	70,0%
Select seq gb|JX163018.1|	Origanum syriacum voucher RNG:H50 clone 10 internal transcribed spacer 1, partial sequence; 5.8S ribosomal RNA gene, complete sequence; and internal transcribed spacer 2, partial sequence	867	867	98%	0.0	92%	JX163018.1	813,92	70,0%
Select seq gb|JX163017.1|	Origanum syriacum voucher RNG:H50 clone 9 internal transcribed spacer 1, partial sequence; 5.8S ribosomal RNA gene, complete sequence; and internal transcribed spacer 2, partial sequence	867	867	98%	0.0	92%	JX163017.1	813,92	70,0%
Select seq gb|AY506647.1|	Origanum vulgare internal transcribed spacer 1, partial sequence; 5.8S ribosomal RNA gene, complete sequence; and internal transcribed spacer 2, partial sequence	881	881	100%	0.0	92%	AY506647.1	810,52	69,7%
Select seq gb|JX162980.1|	Origanum onites voucher SR777 clone 14 internal transcribed spacer 1, partial sequence; 5.8S ribosomal RNA gene, complete sequence; and internal transcribed spacer 2, partial sequence	878	878	100%	0.0	92%	JX162980.1	807,76	69,4%
Select seq gb|JX163004.1|	Origanum onites voucher SR1372 clone 8 internal transcribed spacer 1, partial sequence; 5.8S ribosomal RNA gene, complete sequence; and internal transcribed spacer 2, partial sequence	872	872	100%	0.0	92%	JX163004.1	802,24	69,0%
Select seq gb|JX162978.1|	Origanum onites voucher SR777 clone 11 internal transcribed spacer 1, partial sequence; 5.8S ribosomal RNA gene, complete sequence; and internal transcribed spacer 2, partial sequence	872	872	100%	0.0	92%	JX162978.1	802,24	69,0%
Select seq gb|JX162966.1|	Origanum onites voucher SR530 clone 8 internal transcribed spacer 1, partial sequence; 5.8S ribosomal RNA gene, complete sequence; and internal transcribed spacer 2, partial sequence	872	872	100%	0.0	92%	JX162966.1	802,24	69,0%
Select seq gb|JX163003.1|	Origanum onites voucher SR1372 clone 7 internal transcribed spacer 1, partial sequence; 5.8S ribosomal RNA gene, complete sequence; and internal transcribed spacer 2, partial sequence	867	867	100%	0.0	92%	JX163003.1	797,64	68,6%
Select seq gb|JX162994.1|	Origanum onites voucher SR786 clone 13 internal transcribed spacer 1, partial sequence; 5.8S ribosomal RNA gene, complete sequence; and internal transcribed spacer 2, partial sequence	867	867	100%	0.0	92%	JX162994.1	797,64	68,6%
Select seq gb|JX163054.1|	Origanum onites voucher SR810 internal transcribed spacer 1, partial sequence; 5.8S ribosomal RNA gene, complete sequence; and internal transcribed spacer 2, partial sequence	857	857	100%	0.0	91%	JX163054.1	779,87	
								0,00	
								0,00	
								0,00	
								0,00	

Select for downloading or viewing reports	Kh016_trnL Description	Max score	Total score	Query cover	E value	Ident	Accession	(Ident/Cover)* Max score	Deviation from top hit
Select seq gb|GU381638.1|	Thymus haussknechtii voucher M:Nydegger 43838 tRNA-Leu (trnL) gene and trnL-trnF intergenic spacer, partial sequence; chloroplast	1182	1182	93%	0.0	99%	GU381638.1	1258,26	100,0%
Select seq gb|JQ669069.1|	Thymus pulegioides voucher Riina 1577 tRNA-Leu (trnL) gene and trnL-trnF intergenic spacer, partial sequence; plastid	1205	1205	95%	0.0	99%	JQ669069.1	1255,74	99,8%
Select seq gb|AY506613.1|	Thymus vulgaris tRNA-Leu (trnL) gene and trnL-trnF intergenic spacer, partial sequence; chloroplast	1177	1177	93%	0.0	99%	AY506613.1	1252,94	99,6%
Select seq gb|GU381635.1|	Thymus broussonetii subsp. hannonis voucher MSB:Podlech 142 tRNA-Leu (trnL) gene and trnL-trnF intergenic spacer, partial sequence; chloroplast	1210	1210	96%	0.0	99%	GU381635.1	1247,81	99,2%
Select seq gb|KR063657.1|	Thymus sibthorpii tRNA-Leu (trnL) gene, partial sequence; trnL-trnF intergenic spacer, complete sequence; and tRNA-Phe (trnF) gene, partial sequence; chloroplast	1260	1260	100%	0.0	99%	KR063657.1	1247,40	99,1%
Select seq gb|EU556532.1|	Thymus amurensis isolate HX tRNA-Leu (trnL) gene and trnL-trnF intergenic spacer, partial sequence; chloroplast	1260	1260	100%	0.0	99%	EU556532.1	1247,40	99,1%
Select seq gb|EU556536.1|	Thymus marschallianus isolate XY tRNA-Leu (trnL) gene and trnL-trnF intergenic spacer, partial sequence; chloroplast	1195	1195	95%	0.0	99%	EU556536.1	1245,32	99,0%
Select seq gb|GU381637.1|	Thymus serpyllum voucher M:Braeuchler 2514 tRNA-Leu (trnL) gene and trnL-trnF intergenic spacer, partial sequence; chloroplast	1205	1205	96%	0.0	99%	GU381637.1	1242,66	98,8%
Select seq gb|EU556530.1|	Thymus mandschuricus isolate HM tRNA-Leu (trnL) gene and trnL-trnF intergenic spacer, partial sequence; chloroplast	1205	1205	96%	0.0	99%	EU556530.1	1242,66	98,8%
Select seq gb|EU556525.1|	Thymus quinquecostatus isolate CL tRNA-Leu (trnL) gene and trnL-trnF intergenic spacer, partial sequence; chloroplast	1254	1254	100%	0.0	99%	EU556525.1	1241,46	98,7%
Select seq gb|EU556535.1|	Thymus proximus isolate XN tRNA-Leu (trnL) gene and trnL-trnF intergenic spacer, partial sequence; chloroplast	1166	1166	93%	0.0	99%	EU556535.1	1241,23	98,6%
Select seq gb|EU556539.1|	Thymus quinquecostatus isolate ZY tRNA-Leu (trnL) gene and trnL-trnF intergenic spacer, partial sequence; chloroplast	1253	1253	100%	0.0	99%	EU556539.1	1240,47	98,6%
Select seq gb|EU556538.1|	Thymus quinquecostatus isolate ZJ tRNA-Leu (trnL) gene and trnL-trnF intergenic spacer, partial sequence; chloroplast	1253	1253	100%	0.0	99%	EU556538.1	1240,47	98,6%
Select seq gb|EU556533.1|	Thymus quinquecostatus isolate HY tRNA-Leu (trnL) gene and trnL-trnF intergenic spacer, partial sequence; chloroplast	1253	1253	100%	0.0	99%	EU556533.1	1240,47	98,6%
Select seq gb|EU556527.1|	Thymus mongolicus isolate G2 tRNA-Leu (trnL) gene and trnL-trnF intergenic spacer, partial sequence; chloroplast	1253	1253	100%	0.0	99%	EU556527.1	1240,47	98,6%
Select seq gb|EU556526.1|	Thymus quinquecostatus isolate D2 tRNA-Leu (trnL) gene and trnL-trnF intergenic spacer, partial sequence; chloroplast	1253	1253	100%	0.0	99%	EU556526.1	1240,47	98,6%
Select seq gb|GU381634.1|	Thymus pulegioides voucher M:Braeuchler 3129 tRNA-Leu (trnL) gene and trnL-trnF intergenic spacer, partial sequence; chloroplast	1201	1201	96%	0.0	99%	GU381634.1	1238,53	98,4%
Select seq gb|GU381636.1|	Thymus vulgaris voucher M:Braeuchler 3683 tRNA-Leu (trnL) gene and trnL-trnF intergenic spacer, partial sequence; chloroplast	1199	1199	96%	0.0	99%	GU381636.1	1236,47	98,3%
Select seq gb|EU556528.1|	Thymus mongolicus isolate G3 tRNA-Leu (trnL) gene and trnL-trnF intergenic spacer, partial sequence; chloroplast	1199	1199	96%	0.0	99%	EU556528.1	1236,47	98,3%
Select seq gb|EU556534.1|	Thymus mongolicus isolate S1 tRNA-Leu (trnL) gene and trnL-trnF intergenic spacer, partial sequence; chloroplast	1160	1160	93%	0.0	99%	EU556534.1	1234,84	98,1%
Select seq gb|EU556531.1|	Thymus amurensis isolate HW tRNA-Leu (trnL) gene and trnL-trnF intergenic spacer, partial sequence; chloroplast	1247	1247	100%	0.0	99%	EU556531.1	1234,53	98,1%
Select seq gb|JQ690290.1|	Origanum elongatum isolate H5_O_elo trnL-trnF intergenic spacer, partial sequence; chloroplast	1219	1219	98%	0.0	99%	JQ690290.1	1231,44	97,9%
Select seq gb|JQ690289.1|	Origanum rotundifolium isolate DNA3_O_rot trnL-trnF intergenic spacer, partial sequence; chloroplast	1219	1219	98%	0.0	99%	JQ690289.1	1231,44	97,9%
Select seq emb|AJ505544.1|	Thymus serpyllum var. citriodorum plastid trnL-trnF intergenic spacer, specimen voucher cult., K-1975-1177, Chase 13331 (K)	1218	1218	98%	0.0	99%	AJ505544.1	1230,43	97,8%
Select seq gb|EU556529.1|	Thymus dahuricus isolate HD tRNA-Leu (trnL) gene and trnL-trnF intergenic spacer, partial sequence; chloroplast	1190	1190	96%	0.0	99%	EU556529.1	1227,19	97,5%
Select seq gb|GU381633.1|	Thymus caespititius voucher M:Heubl s.n. tRNA-Leu (trnL) gene and trnL-trnF intergenic spacer, partial sequence; chloroplast	1188	1188	96%	0.0	99%	GU381633.1	1225,13	97,4%

Select seq emb|AJ505543.1|	Origanum vulgare plastid trnL-trnF intergenic spacer, specimen voucher cult., K-000-69-19317, chase 13334 (K)	1210	1210	98%	0.0	99%	AJ505543.1	1222,35	97,1%
Select seq gb|JQ690293.1|	Origanum dayi isolate H43_O_day trnL-trnF intergenic spacer, partial sequence; chloroplast	1208	1208	98%	0.0	99%	JQ690293.1	1220,33	97,0%
Select seq gb|JX880022.1|	Origanum vulgare subsp. vulgare chloroplast, complete genome	1232	1232	100%	0.0	99%	JX880022.1	1219,68	96,9%
Select seq gb|GU381640.1|	Argantoniella salzmannii voucher M:Barra et al. 2673GL tRNA-Leu (trnL) gene and trnL-trnF intergenic spacer, partial sequence; chloroplast	1182	1182	96%	0.0	99%	GU381640.1	1218,94	96,9%
Select seq gb|AY840202.1|	Origanum vulgare tRNA-Leu (trnL) gene and trnL-trnF intergenic spacer, partial sequence; chloroplast	1182	1182	96%	0.0	99%	AY840202.1	1218,94	96,9%
Select seq gb|AY506614.1|	Origanum vulgare tRNA-Leu (trnL) gene and trnL-trnF intergenic spacer, partial sequence; chloroplast	1142	1142	93%	0.0	99%	AY506614.1	1215,68	96,6%
Select seq gb|AY570463.1|	Origanum vulgare voucher JBW 2567 tRNA-Leu and trnL-trnF intergenic spacer, partial sequence; chloroplast	1175	1175	96%	0.0	99%	AY570463.1	1211,72	96,3%
Select seq gb|EU556537.1|	Thymus quinquecostatus isolate XZ tRNA-Leu (trnL) gene and trnL-trnF intergenic spacer, partial sequence; chloroplast	1223	1223	100%	0.0	99%	EU556537.1	1210,77	96,2%
Select seq gb|JF301392.1|	Origanum vulgare voucher B. Drew 77 tRNA-Leu (trnL) gene and trnL-trnF intergenic spacer, partial sequence; chloroplast	1149	1149	94%	0.0	99%	JF301392.1	1210,12	96,2%
Select seq gb|AY570502.1|	Thymus serpyllum voucher JBW 2564 tRNA-Leu and trnL-trnF intergenic spacer, partial sequence; chloroplast	1171	1171	96%	0.0	99%	AY570502.1	1207,59	96,0%
Select seq gb|JQ669070.1|	Thymus serpyllum voucher J. Walker 2564 tRNA-Leu (trnL) gene and trnL-trnF intergenic spacer, partial sequence; plastid	1164	1164	95%	0.0	98%	JQ669070.1	1200,76	95,4%
Select seq gb|GU381629.1|	Thymbra capitata voucher M:Braeuchler 2518 tRNA-Leu (trnL) gene and trnL-trnF intergenic spacer, partial sequence; chloroplast	1166	1166	96%	0.0	98%	GU381629.1	1190,29	94,6%
Select seq gb|JF301401.1|	Thymbra capitata voucher UCBG 96.0817 tRNA-Leu (trnL) gene and trnL-trnF intergenic spacer, partial sequence; chloroplast	1140	1140	94%	0.0	98%	JF301401.1	1188,51	94,5%
Select seq gb|GU381484.1|	Micromeria flagellaris voucher E:van der Werff & McPherson 13570 tRNA-Leu (trnL) gene and trnL-trnF intergenic spacer, partial sequence; chloroplast	1160	1160	96%	0.0	98%	GU381484.1	1184,17	94,1%
Select seq gb|GU381483.1|	Micromeria flagellaris voucher E:Clement et al 2140 tRNA-Leu (trnL) gene and trnL-trnF intergenic spacer, partial sequence; chloroplast	1160	1160	96%	0.0	98%	GU381483.1	1184,17	94,1%
Select seq gb|AY840207.1|	Thymbra spicata tRNA-Leu (trnL) gene and trnL-trnF intergenic spacer, partial sequence; chloroplast	1160	1160	96%	0.0	98%	AY840207.1	1184,17	94,1%
Select seq gb|GU381632.1|	Thymbra spicata voucher M:Braeuchler 4548 tRNA-Leu (trnL) gene and trnL-trnF intergenic spacer, partial sequence; chloroplast	1157	1157	96%	0.0	98%	GU381632.1	1181,10	93,9%
Select seq gb|GU381627.1|	Thymbra sintenisii subsp. isaurica voucher E:Goener 12628 tRNA-Leu (trnL) gene and trnL-trnF intergenic spacer, partial sequence; chloroplast	1155	1155	96%	0.0	98%	GU381627.1	1179,06	93,7%
Select seq gb|GU381513.1|	Clinopodium nepalense voucher BM<GBR-LONDON>:Mikage et al. 9550294 tRNA-Leu (trnL) gene and trnL-trnF intergenic spacer, partial sequence; chloroplast	1116	1116	93%	0.0	98%	GU381513.1	1176,00	93,5%
Select seq gb|GU381517.1|	Clinopodium barosmum voucher BM<GBR-LONDON>:McLaren N193 tRNA-Leu (trnL) gene and trnL-trnF intergenic spacer, partial sequence; chloroplast	1144	1144	96%	0.0	98%	GU381517.1	1167,83	92,8%
Select seq gb|GU381516.1|	Clinopodium wardii voucher BM<GBR-LONDON>:Ludlow et al. 14234 tRNA-Leu (trnL) gene and trnL-trnF intergenic spacer, partial sequence; chloroplast	1144	1144	96%	0.0	98%	GU381516.1	1167,83	92,8%
Select seq gb|GU381515.1|	Clinopodium hydaspidis voucher BM<GBR-LONDON>:Mohd 133 tRNA-Leu (trnL) gene and trnL-trnF intergenic spacer, partial sequence; chloroplast	1144	1144	96%	0.0	98%	GU381515.1	1167,83	92,8%
Select seq gb|GU381514.1|	Clinopodium nepalense voucher FR:Stainton 6024 tRNA-Leu (trnL) gene and trnL-trnF intergenic spacer, partial sequence; chloroplast	1144	1144	96%	0.0	98%	GU381514.1	1167,83	92,8%
Select seq gb|GU381511.1|	Clinopodium piperitum voucher BM<GBR-LONDON>:Vickery 454 tRNA-Leu (trnL) gene and trnL-trnF intergenic spacer, partial sequence; chloroplast	1144	1144	96%	0.0	98%	GU381511.1	1167,83	92,8%
Select seq gb|GU381495.1|	Killickia pilosa voucher M:Braeuchler 3810 tRNA-Leu (trnL) gene and trnL-trnF intergenic spacer, partial sequence; chloroplast	1120	1120	94%	0.0	98%	GU381495.1	1167,66	92,8%
Select seq gb|GU381512.1|	Clinopodium piperitum voucher E:Stainton 7320 tRNA-Leu (trnL) gene and trnL-trnF intergenic spacer, partial sequence; chloroplast	1138	1138	96%	0.0	98%	GU381512.1	1161,71	92,3%
Select seq gb|GU381496.1|	Killickia pilosa voucher M:Braeuchler 3832 tRNA-Leu (trnL) gene and trnL-trnF intergenic spacer, partial sequence; chloroplast	1134	1134	96%	0.0	98%	GU381496.1	1157,63	92,0%

Select seq gb|GU381493.1|	Killickia grandiflora voucher M:Braeuchler 3811 tRNA-Leu (trnL) gene and trnL-trnF intergenic spacer, partial sequence; chloroplast	1134	1134	96% 0.0	98% GU381493.1	1157,63	92,0%
Select seq gb|GU381631.1|	Satureja linearifolia voucher M:Brullo & Furnari s.n. tRNA-Leu (trnL) gene and trnL-trnF intergenic spacer, partial sequence; chloroplast	1133	1133	96% 0.0	98% GU381631.1	1156,60	91,9%
Select seq gb|GU381628.1|	Thymbra calostachya voucher M:Ulrich s.n. tRNA-Leu (trnL) gene and trnL-trnF intergenic spacer, partial sequence; chloroplast	1129	1129	96% 0.0	98% GU381628.1	1152,52	91,6%
Select seq gb|KR150198.1|	Ziziphora sp. 3 Kh112 trnL-trnF intergenic spacer region, partial sequence; chloroplast	1122	1122	95% 0.0	97% KR150198.1	1145,62	91,0%
Select seq gb|GU381481.1|	Micromeria cf. madagascariensis Morawetz 205 tRNA-Leu (trnL) gene and trnL-trnF intergenic spacer, partial sequence; chloroplast	1133	1133	96% 0.0	97% GU381481.1	1144,80	91,0%
Select seq gb|GU381485.1|	Micromeria sphaerophylla voucher E:Lewis et al 1064 tRNA-Leu (trnL) gene and trnL-trnF intergenic spacer, partial sequence; chloroplast	1131	1131	96% 0.0	97% GU381485.1	1142,78	90,8%
Select seq gb|KR150243.1|	Ziziphora sp. 2 Kh20 trnL-trnF intergenic spacer region, partial sequence; chloroplast	1142	1142	97% 0.0	97% KR150243.1	1142,00	90,8%
Select seq gb|GU381507.1|	Ziziphora tenuior voucher MSB:Fayvush et al. 03-1503 tRNA-Leu (trnL) gene and trnL-trnF intergenic spacer, partial sequence; chloroplast	1127	1127	96% 0.0	97% GU381507.1	1138,74	90,5%
Select seq gb|KR150238.1|	Ziziphora sp. 1 Kh75 trnL-trnF intergenic spacer region, partial sequence; chloroplast	1138	1138	97% 0.0	97% KR150238.1	1138,00	90,4%
Select seq gb|JQ669067.1|	Satureja montana voucher UCBG 2002.0593, Forbes s.n tRNA-Leu (trnL) gene and trnL-trnF intergenic spacer, partial sequence; plastid	1112	1112	95% 0.0	97% JQ669067.1	1135,41	90,2%
Select seq gb|GU381489.1|	Killickia lutea voucher NU<ZAF>:Hilliard & Burtt 9876 tRNA-Leu (trnL) gene and trnL-trnF intergenic spacer, partial sequence; chloroplast	1123	1123	96% 0.0	97% GU381489.1	1134,70	90,2%
Select seq gb|GU381488.1|	Killickia compacta voucher M:Braeuchler 3816 tRNA-Leu (trnL) gene and trnL-trnF intergenic spacer, partial sequence; chloroplast	1123	1123	96% 0.0	97% GU381488.1	1134,70	90,2%
Select seq gb|DQ667501.1|	Ziziphora taurica isolate x262 tRNA-Leu (trnL) gene and trnL-trnF intergenic spacer, partial sequence; chloroplast	1123	1123	96% 0.0	97% DQ667501.1	1134,70	90,2%
Select seq gb|KR150209.1|	Satureja sp. 3 Kh90 trnL-trnF intergenic spacer region, partial sequence; chloroplast	1122	1122	96% 0.0	97% KR150209.1	1133,69	90,1%
Select seq gb|GU381510.1|	Ziziphora pamiroalaica voucher C:Murray et al. 10090 tRNA-Leu (trnL) gene and trnL-trnF intergenic spacer, partial sequence; chloroplast	1122	1122	96% 0.0	97% GU381510.1	1133,69	90,1%
Select seq gb|GU381505.1|	Clinopodium troodi voucher W:Davis 1856 tRNA-Leu (trnL) gene and trnL-trnF intergenic spacer, partial sequence; chloroplast	1122	1122	96% 0.0	97% GU381505.1	1133,69	90,1%
Select seq gb|GU381501.1|	Clinopodium graveolens subsp. rotundifolium voucher M:Podlech 47181 tRNA-Leu (trnL) gene and trnL-trnF intergenic spacer, partial sequence; chloroplast	1122	1122	96% 0.0	97% GU381501.1	1133,69	90,1%
Select seq gb|GU381499.1|	Clinopodium suaveolens voucher M:Erben s.n. tRNA-Leu (trnL) gene and trnL-trnF intergenic spacer, partial sequence; chloroplast	1122	1122	96% 0.0	97% GU381499.1	1133,69	90,1%
Select seq gb|JQ669021.1|	Clinopodium acinos voucher Judziewicz 14160 tRNA-Leu (trnL) gene and trnL-trnF intergenic spacer, partial sequence; plastid	1110	1110	95% 0.0	97% JQ669021.1	1133,37	90,1%
Select seq gb|KR063656.1|	Satureja pilosa subsp. origanita tRNA-Leu (trnL) gene, partial sequence; trnL-trnF intergenic spacer, complete sequence; and tRNA-Phe (trnF) gene, partial sequence; chloroplast	1168	1168	100% 0.0	97% KR063656.1	1132,96	90,0%
Select seq gb|GU381500.1|	Clinopodium nanum voucher M:Braeuchler 2796 tRNA-Leu (trnL) gene and trnL-trnF intergenic spacer, partial sequence; chloroplast	1109	1109	95% 0.0	97% GU381500.1	1132,35	90,0%
Select seq gb|KR150233.1|	Thymus sp. 6 Kh133 trnL-trnF intergenic spacer region, partial sequence; chloroplast	1120	1120	96% 0.0	97% KR150233.1	1131,67	89,9%
Select seq gb|GU381621.1|	Satureja cuneifolia voucher M:Rechinger 11142 tRNA-Leu (trnL) gene and trnL-trnF intergenic spacer, partial sequence; chloroplast	1118	1118	96% 0.0	97% GU381621.1	1129,65	89,8%
Select seq gb|GU381612.1|	Gontscharovia popovii voucher BM<GBR-LONDON>:Schmid 2419 tRNA-Leu (trnL) gene and trnL-trnF intergenic spacer, partial sequence; chloroplast	1118	1118	96% 0.0	97% GU381612.1	1129,65	89,8%
Select seq gb|AY840179.1|	Satureja montana tRNA-Leu (trnL) gene and trnL-trnF intergenic spacer, partial sequence; chloroplast	1118	1118	96% 0.0	97% AY840179.1	1129,65	89,8%
Select seq gb|GU381497.1|	Clinopodium acinos voucher M:Podlech 50287 tRNA-Leu (trnL) gene and trnL-trnF intergenic spacer, partial sequence; chloroplast	1116	1116	96% 0.0	97% GU381497.1	1127,63	89,6%
Select seq gb|GU381619.1|	Satureja mutica voucher M:Akhani 12362 tRNA-Leu (trnL) gene and trnL-trnF intergenic spacer, partial sequence; chloroplast	1112	1112	96% 0.0	97% GU381619.1	1123,58	89,3%

Select seq gb|GU381614.1|	Satureja thymbra voucher M:Braeuchler 2896 tRNA-Leu (trnL) gene and trnL-trnF intergenic spacer, partial sequence; chloroplast	1112	1112	96%	0.0	97%	GU381614.1	1123,58	89,3%
Select seq gb|GU381611.1|	Gontscharovia popovii voucher M:Vvedensky s.n. tRNA-Leu (trnL) gene and trnL-trnF intergenic spacer, partial sequence; chloroplast	1112	1112	96%	0.0	97%	GU381611.1	1123,58	89,3%
Select seq gb|GU381521.1|	Mentha pulegium voucher M:Braeuchler 2300 tRNA-Leu (trnL) gene and trnL-trnF intergenic spacer, partial sequence; chloroplast	1112	1112	96%	0.0	97%	GU381521.1	1123,58	89,3%
Select seq gb|GU381625.1|	Pentapleura subulifera voucher W:Rechinger 12085 tRNA-Leu (trnL) gene and trnL-trnF intergenic spacer, partial sequence; chloroplast	1109	1109	96%	0.0	97%	GU381625.1	1120,55	89,1%
Select seq gb|GU381615.1|	Satureja innota voucher M:Barra et al. 2484GL tRNA-Leu (trnL) gene and trnL-trnF intergenic spacer, partial sequence; chloroplast	1107	1107	96%	0.0	97%	GU381615.1	1118,53	88,9%
Select seq gb|KC414276.1|	Mentha canadensis isolate 511190001 tRNA-Leu (trnL) gene and trnL-trnF intergenic spacer, partial sequence; chloroplast	1144	1144	100%	0.0	97%	KC414276.1	1109,68	88,2%
Select seq gb|DQ667492.1|	Lepechinia lancifolia isolate x232 tRNA-Leu (trnL) gene and trnL-trnF intergenic spacer, partial sequence; chloroplast	1140	1140	100%	0.0	97%	DQ667492.1	1105,80	87,9%
Select seq emb|AJ505541.1|	Mentha suaveolens plastid trnL-trnF intergenic spacer, specimen voucher cult., K-1970-3169 (K)	1122	1122	98%	0.0	96%	AJ505541.1	1099,10	87,4%
Select seq gb|GU381630.1|	Satureja thymbrifolia voucher M:Danin s.n. tRNA-Leu (trnL) gene and trnL-trnF intergenic spacer, partial sequence; chloroplast	1099	1099	96%	0.0	96%	GU381630.1	1099,00	87,3%
Select seq gb|FJ593456.1|	Mentha sp. JSZ-2009a tRNA-Leu (trnL) gene and trnL-trnF intergenic spacer, partial sequence; chloroplast	1136	1136	100%	0.0	96%	FJ593456.1	1090,56	86,7%
Select seq gb|DQ667514.1|	Mentha arvensis isolate x424 tRNA-Leu (trnL) gene and trnL-trnF intergenic spacer, partial sequence; chloroplast	1123	1123	99%	0.0	96%	DQ667514.1	1088,97	86,5%
Select seq gb|DQ667513.1|	Clinopodium vulgare isolate x423 tRNA-Leu (trnL) gene and trnL-trnF intergenic spacer, partial sequence; chloroplast	1123	1123	100%	0.0	96%	DQ667513.1	1078,08	85,7%
Select seq gb|DQ667504.1|	Cunila incana isolate x296 tRNA-Leu (trnL) gene and trnL-trnF intergenic spacer, partial sequence; chloroplast	1118	1118	100%	0.0	96%	DQ667504.1	1073,28	85,3%
Select seq gb|DQ667507.1|	Pycnanthemum virginianum isolate x313 tRNA-Leu (trnL) gene and trnL-trnF intergenic spacer, partial sequence; chloroplast	1112	1112	100%	0.0	96%	DQ667507.1	1067,52	84,8%
Select seq gb|DQ667497.1|	Hoehnea epilobioides isolate x258 tRNA-Leu (trnL) gene and trnL-trnF intergenic spacer, partial sequence; chloroplast	1112	1112	100%	0.0	96%	DQ667497.1	1067,52	84,8%
								0,00	0,0%
								0,00	0,0%
								0,00	0,0%
								0,00	0,0%

Select for downloading or viewing reports	Kh017_ITS Description	Max score	Total score	Query cover	E value	Ident	Accession	(Ident/Cover)* Max score	Deviation from top hit
Select seq gb|AY603242.1|	Achillea pseudopectinata internal transcribed spacer 1, 5.8S ribosomal RNA gene, and internal transcribed spacer 2, complete sequence	1077	1077	92%	0.0	98%	AY603242.1	1147,24	100,0%
Select seq gb|AY603218.1|	Achillea biebersteinii internal transcribed spacer 1, 5.8S ribosomal RNA gene, and internal transcribed spacer 2, complete sequence	1075	1075	92%	0.0	98%	AY603218.1	1145,11	99,8%
Select seq gb|AY603220.1|	Achillea leptophylla internal transcribed spacer 1, 5.8S ribosomal RNA gene, and internal transcribed spacer 2, complete sequence	1062	1062	92%	0.0	97%	AY603220.1	1119,72	97,6%
Select seq gb|AY603229.1|	Achillea clavennae internal transcribed spacer 1, 5.8S ribosomal RNA gene, and internal transcribed spacer 2, complete sequence	1057	1057	92%	0.0	97%	AY603229.1	1114,45	97,1%
Select seq gb|AY603222.1|	Achillea holosericea internal transcribed spacer 1, 5.8S ribosomal RNA gene, and internal transcribed spacer 2, complete sequence	1053	1053	92%	0.0	97%	AY603222.1	1110,23	96,8%
Select seq gb|AY603245.1|	Achillea chamaemelifolia internal transcribed spacer 1, 5.8S ribosomal RNA gene, and internal transcribed spacer 2, complete sequence	1051	1051	92%	0.0	97%	AY603245.1	1108,12	96,6%
Select seq gb|AY603211.1|	Achillea virescens internal transcribed spacer 1, 5.8S ribosomal RNA gene, and internal transcribed spacer 2, complete sequence	1051	1051	92%	0.0	97%	AY603211.1	1108,12	96,6%
Select seq gb|AY603216.1|	Achillea clypeolata internal transcribed spacer 1, 5.8S ribosomal RNA gene, and internal transcribed spacer 2, complete sequence	1051	1051	92%	0.0	97%	AY603216.1	1108,12	96,6%
Select seq gb|AY603187.1|	Achillea millefolium subsp. sudetica internal transcribed spacer 1, 5.8S ribosomal RNA gene, and internal transcribed spacer 2, complete sequence	1051	1051	92%	0.0	97%	AY603187.1	1108,12	96,6%
Select seq gb|AY603190.1|	Achillea styriaca internal transcribed spacer 1, 5.8S ribosomal RNA gene, and internal transcribed spacer 2, complete sequence	1048	1048	92%	0.0	97%	AY603190.1	1104,96	96,3%
Select seq gb|AY603186.1|	Achillea millefolium internal transcribed spacer 1, 5.8S ribosomal RNA gene, and internal transcribed spacer 2, complete sequence	1048	1048	92%	0.0	97%	AY603186.1	1104,96	96,3%
Select seq gb|AY603221.1|	Achillea grandifolia internal transcribed spacer 1, 5.8S ribosomal RNA gene, and internal transcribed spacer 2, complete sequence	1046	1046	92%	0.0	97%	AY603221.1	1102,85	96,1%
Select seq gb|AY603215.1|	Achillea crithmifolia internal transcribed spacer 1, 5.8S ribosomal RNA gene, and internal transcribed spacer 2, complete sequence	1046	1046	92%	0.0	97%	AY603215.1	1102,85	96,1%
Select seq gb|AY603212.1|	Achillea nobilis internal transcribed spacer 1, 5.8S ribosomal RNA gene, and internal transcribed spacer 2, complete sequence	1046	1046	92%	0.0	97%	AY603212.1	1102,85	96,1%
Select seq gb|AY603202.1|	Achillea euxina internal transcribed spacer 1, 5.8S ribosomal RNA gene, and internal transcribed spacer 2, complete sequence	1046	1046	92%	0.0	97%	AY603202.1	1102,85	96,1%
Select seq gb|AY603197.1|	Achillea setacea internal transcribed spacer 1, 5.8S ribosomal RNA gene, and internal transcribed spacer 2, complete sequence	1046	1046	92%	0.0	97%	AY603197.1	1102,85	96,1%
Select seq gb|AY603185.1|	Achillea millefolium internal transcribed spacer 1, 5.8S ribosomal RNA gene, and internal transcribed spacer 2, complete sequence	1046	1046	92%	0.0	97%	AY603185.1	1102,85	96,1%
Select seq gb|AY603184.1|	Achillea pannonica internal transcribed spacer 1, 5.8S ribosomal RNA gene, and internal transcribed spacer 2, complete sequence	1046	1046	92%	0.0	97%	AY603184.1	1102,85	96,1%
Select seq gb|AY603228.1|	Achillea clusiana internal transcribed spacer 1, 5.8S ribosomal RNA gene, and internal transcribed spacer 2, complete sequence	1044	1044	92%	0.0	97%	AY603228.1	1100,74	95,9%
Select seq gb|AY603205.1|	Achillea lanulosa subsp. alpicola internal transcribed spacer 1, 5.8S ribosomal RNA gene, and internal transcribed spacer 2, complete sequence	1044	1044	92%	0.0	97%	AY603205.1	1100,74	95,9%
Select seq gb|EU796891.1|	Achillea millefolium 18S ribosomal RNA gene, partial sequence; internal transcribed spacer 1, 5.8S ribosomal RNA gene, and internal transcribed spacer 2, complete sequence; and 26S ribosomal RNA gene, partial sequence	1110	1110	98%	0.0	97%	EU796891.1	1098,67	95,8%
Select seq gb|AY603230.1|	Achillea clavennae internal transcribed spacer 1, 5.8S ribosomal RNA gene, and internal transcribed spacer 2, complete sequence	1042	1042	92%	0.0	97%	AY603230.1	1098,63	95,8%
Select seq gb|AY603204.1|	Achillea ceretanica internal transcribed spacer 1, 5.8S ribosomal RNA gene, and internal transcribed spacer 2, complete sequence	1042	1042	92%	0.0	97%	AY603204.1	1098,63	95,8%
Select seq gb|AY603200.1|	Achillea roseoalba internal transcribed spacer 1, 5.8S ribosomal RNA gene, and internal transcribed spacer 2, complete sequence	1042	1042	92%	0.0	97%	AY603200.1	1098,63	95,8%
Select seq gb|AY603195.1|	Achillea setacea internal transcribed spacer 1, 5.8S ribosomal RNA gene, and internal transcribed spacer 2, complete sequence	1042	1042	92%	0.0	97%	AY603195.1	1098,63	95,8%
Select seq gb|FJ553518.1|	Angiosperm environmental sample clone LTSP_EUKA_P3K07 18S ribosomal RNA, 18S-25/28S ribosomal RNA intergenic spacer, partial sequence	1120	1120	99%	0.0	97%	FJ553518.1	1097,37	95,7%

Select seq gb|AY603214.1|	Achillea ageratum internal transcribed spacer 1, 5.8S ribosomal RNA gene, and internal transcribed spacer 2, complete sequence	1040	1040	92% 0.0	97%	AY603214.1	1096,52	95,6%
Select seq gb|AY603213.1|	Achillea absinthoides internal transcribed spacer 1, 5.8S ribosomal RNA gene, and internal transcribed spacer 2, complete sequence	1040	1040	92% 0.0	97%	AY603213.1	1096,52	95,6%
Select seq gb|AY603207.1|	Achillea wilsoniana internal transcribed spacer 1, 5.8S ribosomal RNA gene, and internal transcribed spacer 2, complete sequence	1040	1040	92% 0.0	97%	AY603207.1	1096,52	95,6%
Select seq gb|AY603203.1|	Achillea ceretanica internal transcribed spacer 1, 5.8S ribosomal RNA gene, and internal transcribed spacer 2, complete sequence	1040	1040	92% 0.0	97%	AY603203.1	1096,52	95,6%
Select seq gb|AY603191.1|	Achillea setacea internal transcribed spacer 1, 5.8S ribosomal RNA gene, and internal transcribed spacer 2, complete sequence	1040	1040	92% 0.0	97%	AY603191.1	1096,52	95,6%
Select seq gb|AY603210.1|	Achillea monticola internal transcribed spacer 1, 5.8S ribosomal RNA gene, and internal transcribed spacer 2, complete sequence	1038	1038	92% 0.0	97%	AY603210.1	1094,41	95,4%
Select seq gb|AY603201.1|	Achillea asplenifolia internal transcribed spacer 1, 5.8S ribosomal RNA gene, and internal transcribed spacer 2, complete sequence	1038	1038	92% 0.0	97%	AY603201.1	1094,41	95,4%
Select seq gb|AY603232.1|	Achillea fraasii internal transcribed spacer 1, 5.8S ribosomal RNA gene, and internal transcribed spacer 2, complete sequence	1035	1035	92% 0.0	97%	AY603232.1	1091,25	95,1%
Select seq gb|AY603219.1|	Achillea filipendulina internal transcribed spacer 1, 5.8S ribosomal RNA gene, and internal transcribed spacer 2, complete sequence	1035	1035	92% 0.0	97%	AY603219.1	1091,25	95,1%
Select seq gb|FJ980357.1|	Achillea wilsoniana voucher PS0715MT01 18S ribosomal RNA gene, partial sequence; internal transcribed spacer 1, 5.8S ribosomal RNA gene, and internal transcribed spacer 2, complete sequence; and 28S ribosomal RNA gene, partial sequence	1110	1110	99% 0.0	97%	FJ980357.1	1087,58	94,8%
Select seq gb|JQ230971.1|	Achillea millefolium voucher SBB-1156 18S ribosomal RNA gene, partial sequence; internal transcribed spacer 1, 5.8S ribosomal RNA gene, and internal transcribed spacer 2, complete sequence; and 28S ribosomal RNA gene, partial sequence	1098	1098	98% 0.0	97%	JQ230971.1	1086,80	94,7%
Select seq gb|FJ980358.1|	Achillea wilsoniana voucher PS0715MT02 18S ribosomal RNA gene, partial sequence; internal transcribed spacer 1, 5.8S ribosomal RNA gene, and internal transcribed spacer 2, complete sequence; and 28S ribosomal RNA gene, partial sequence	1109	1109	99% 0.0	97%	FJ980358.1	1086,60	94,7%
Select seq gb|AY603223.1|	Achillea micrantha internal transcribed spacer 1, 5.8S ribosomal RNA gene, and internal transcribed spacer 2, complete sequence	1029	1029	92% 0.0	96%	AY603223.1	1073,74	93,6%
Select seq gb|EF577285.1|	Achillea wilsoniana 18S ribosomal RNA gene, partial sequence; internal transcribed spacer 1, 5.8S ribosomal RNA gene, and internal transcribed spacer 2, complete sequence; and 28S ribosomal RNA gene, partial sequence	1094	1094	98% 0.0	96%	EF577285.1	1071,67	93,4%
Select seq gb|AY603227.1|	Achillea atrata internal transcribed spacer 1, 5.8S ribosomal RNA gene, and internal transcribed spacer 2, complete sequence	1027	1027	92% 0.0	96%	AY603227.1	1071,65	93,4%
Select seq gb|AY603240.1|	Achillea schurii internal transcribed spacer 1, 5.8S ribosomal RNA gene, and internal transcribed spacer 2, complete sequence	1018	1018	92% 0.0	96%	AY603240.1	1062,26	92,6%
Select seq gb|AY603238.1|	Achillea oxyloba internal transcribed spacer 1, 5.8S ribosomal RNA gene, and internal transcribed spacer 2, complete sequence	1013	1013	92% 0.0	96%	AY603238.1	1057,04	92,1%
Select seq gb|AY603239.1|	Achillea pindicola subsp. integrifolia internal transcribed spacer 1, 5.8S ribosomal RNA gene, and internal transcribed spacer 2, complete sequence	1007	1007	92% 0.0	96%	AY603239.1	1050,78	91,6%
Select seq gb|AY603241.1|	Achillea schurii internal transcribed spacer 1, 5.8S ribosomal RNA gene, and internal transcribed spacer 2, complete sequence	1002	1002	92% 0.0	96%	AY603241.1	1045,57	91,1%
Select seq gb|AY603249.1|	Achillea ptarmicifolia internal transcribed spacer 1, 5.8S ribosomal RNA gene, and internal transcribed spacer 2, complete sequence	990	990	92% 0.0	95%	AY603249.1	1022,28	89,1%
Select seq gb|AY603224.1|	Achillea ochroleuca internal transcribed spacer 1, 5.8S ribosomal RNA gene, and internal transcribed spacer 2, complete sequence	989	989	92% 0.0	95%	AY603224.1	1021,25	89,0%
Select seq gb|AY603256.1|	Achillea fragrantissima internal transcribed spacer 1, 5.8S ribosomal RNA gene, and internal transcribed spacer 2, complete sequence	985	985	92% 0.0	95%	AY603256.1	1017,12	88,7%
Select seq gb|AY603255.1|	Achillea teretifolia internal transcribed spacer 1, 5.8S ribosomal RNA gene, and internal transcribed spacer 2, complete sequence	981	981	92% 0.0	95%	AY603255.1	1012,99	88,3%
Select seq gb|AY603251.1|	Achillea salicifolia internal transcribed spacer 1, 5.8S ribosomal RNA gene, and internal transcribed spacer 2, complete sequence	981	981	92% 0.0	95%	AY603251.1	1012,99	88,3%
Select seq gb|AY603248.1|	Achillea ptarmica internal transcribed spacer 1, 5.8S ribosomal RNA gene, and internal transcribed spacer 2, complete sequence	981	981	92% 0.0	95%	AY603248.1	1012,99	88,3%
Select seq gb|AY603247.1|	Achillea pyrenaica internal transcribed spacer 1, 5.8S ribosomal RNA gene, and internal transcribed spacer 2, complete sequence	981	981	92% 0.0	95%	AY603247.1	1012,99	88,3%
Select seq gb|AY603246.1|	Achillea impatiens internal transcribed spacer 1, 5.8S ribosomal RNA gene, and internal transcribed spacer 2, complete sequence	981	981	92% 0.0	95%	AY603246.1	1012,99	88,3%

Select seq gb|AY603237.1|	Achillea nana internal transcribed spacer 1, 5.8S ribosomal RNA gene, and internal transcribed spacer 2, complete sequence	979	979	92%	0.0	95%	AY603237.1	1010,92	88,1%
Select seq gb|AY603235.1|	Achillea moschata internal transcribed spacer 1, 5.8S ribosomal RNA gene, and internal transcribed spacer 2, complete sequence	979	979	92%	0.0	95%	AY603235.1	1010,92	88,1%
Select seq gb|AY603244.1|	Achillea salicifolia internal transcribed spacer 1, 5.8S ribosomal RNA gene, and internal transcribed spacer 2, complete sequence	976	976	92%	0.0	95%	AY603244.1	1007,83	87,8%
Select seq gb|AY603233.1|	Achillea macrophylla internal transcribed spacer 1, 5.8S ribosomal RNA gene, and internal transcribed spacer 2, complete sequence	976	976	92%	0.0	95%	AY603233.1	1007,83	87,8%
Select seq gb|AY603250.1|	Achillea biserrata internal transcribed spacer 1, 5.8S ribosomal RNA gene, and internal transcribed spacer 2, complete sequence	974	974	92%	0.0	95%	AY603250.1	1005,76	87,7%
Select seq gb|AY603226.1|	Achillea abrotanoides internal transcribed spacer 1, 5.8S ribosomal RNA gene, and internal transcribed spacer 2, complete sequence	974	974	92%	0.0	95%	AY603226.1	1005,76	87,7%
Select seq gb|AY603234.1|	Achillea x morisiana internal transcribed spacer 1, 5.8S ribosomal RNA gene, and internal transcribed spacer 2, complete sequence	972	972	92%	0.0	95%	AY603234.1	1003,70	87,5%
Select seq gb|AY603231.1|	Achillea erba-rotta internal transcribed spacer 1, 5.8S ribosomal RNA gene, and internal transcribed spacer 2, complete sequence	972	972	92%	0.0	95%	AY603231.1	1003,70	87,5%
Select seq gb|KC816562.1|	Matricaria chamomilla var. recutita 18S ribosomal RNA gene, partial sequence; internal transcribed spacer 1, 5.8S ribosomal RNA gene, and internal transcribed spacer 2, complete sequence; and 28S ribosomal RNA gene, partial sequence	1024	1024	97%	0.0	95%	KC816562.1	1002,89	87,4%
Select seq gb|AY603254.1|	Achillea wilhelmsii internal transcribed spacer 1, 5.8S ribosomal RNA gene, and internal transcribed spacer 2, complete sequence	970	970	92%	0.0	95%	AY603254.1	1001,63	87,3%
Select seq gb|AY603252.1|	Achillea cretica internal transcribed spacer 1, 5.8S ribosomal RNA gene, and internal transcribed spacer 2, complete sequence	968	968	92%	0.0	95%	AY603252.1	999,57	87,1%
Select seq gb|KC855670.1|	Achillea multifida isolate ACMt5 18S ribosomal RNA gene, partial sequence; internal transcribed spacer 1, 5.8S ribosomal RNA gene, and internal transcribed spacer 2, complete sequence; and 28S ribosomal RNA gene, partial sequence	955	955	91%	0.0	95%	KC855670.1	996,98	86,9%
Select seq gb|KC855667.1|	Achillea multifida isolate ACMt2 18S ribosomal RNA gene, partial sequence; internal transcribed spacer 1, 5.8S ribosomal RNA gene, and internal transcribed spacer 2, complete sequence; and 28S ribosomal RNA gene, partial sequence	955	955	91%	0.0	95%	KC855667.1	996,98	86,9%
Select seq gb|AY603253.1|	Achillea wilhelmsii internal transcribed spacer 1, 5.8S ribosomal RNA gene, and internal transcribed spacer 2, complete sequence	965	965	92%	0.0	95%	AY603253.1	996,47	86,9%
Select seq dbj|AB683260.1|	Tanacetum abrotanifolium gene for ITS1, 5.8S rRNA and ITS2, complete sequence, specimen_voucher: 68865 (TARI)	961	961	92%	0.0	95%	AB683260.1	992,34	86,5%
Select seq gb|KC855671.1|	Achillea multifida isolate ACMt6 18S ribosomal RNA gene, partial sequence; internal transcribed spacer 1, 5.8S ribosomal RNA gene, and internal transcribed spacer 2, complete sequence; and 28S ribosomal RNA gene, partial sequence	957	957	92%	0.0	95%	KC855671.1	988,21	86,1%
Select seq dbj|AB683267.1|	Tanacetum armenum gene for ITS1, 5.8S rRNA and ITS2, complete sequence, specimen_voucher: (W, 1972-03416)	959	959	92%	0.0	94%	AB683267.1	979,85	85,4%
Select seq gb|AY603243.1|	Achillea acuminata internal transcribed spacer 1, 5.8S ribosomal RNA gene, and internal transcribed spacer 2, complete sequence	959	959	92%	0.0	94%	AY603243.1	979,85	85,4%
Select seq gb|KC855673.1|	Achillea multifida isolate ACMt8 18S ribosomal RNA gene, partial sequence; internal transcribed spacer 1, 5.8S ribosomal RNA gene, and internal transcribed spacer 2, complete sequence; and 28S ribosomal RNA gene, partial sequence	944	944	91%	0.0	94%	KC855673.1	975,12	85,0%
Select seq gb|KC855666.1|	Achillea multifida isolate ACMt1 18S ribosomal RNA gene, partial sequence; internal transcribed spacer 1, 5.8S ribosomal RNA gene, and internal transcribed spacer 2, complete sequence; and 28S ribosomal RNA gene, partial sequence	944	944	91%	0.0	94%	KC855666.1	975,12	85,0%
Select seq dbj|AB683290.1|	Tanacetum khorassanicum gene for ITS1, 5.8S rRNA and ITS2, complete sequence, specimen_voucher: 4937 (S)	953	953	92%	0.0	94%	AB683290.1	973,72	84,9%
Select seq gb|DQ267612.1|	Achillea pseudoaleppica internal transcribed spacer 1, partial sequence; 5.8S ribosomal RNA gene, complete sequence; and internal transcribed spacer 2, partial sequence	952	952	92%	0.0	94%	DQ267612.1	972,70	84,8%
Select seq gb|EF577320.1|	Tanacetum parthenium 18S ribosomal RNA gene, partial sequence; internal transcribed spacer 1, 5.8S ribosomal RNA gene, and internal transcribed spacer 2, complete sequence; and 28S ribosomal RNA gene, partial sequence	1014	1014	98%	0.0	94%	EF577320.1	972,61	84,8%
Select seq dbj|AB683285.1|	Tanacetum germanicopolitanum gene for ITS1, 5.8S rRNA and ITS2, complete sequence, specimen_voucher: 14254 (S)	950	950	92%	0.0	94%	AB683285.1	970,65	84,6%
Select seq dbj|AB683271.1|	Tanacetum bamianicum gene for ITS1, 5.8S rRNA and ITS2, complete sequence, specimen_voucher: 18238 (B)	950	950	92%	0.0	94%	AB683271.1	970,65	84,6%
Select seq dbj|AB683265.1|	Tanacetum argenteum subsp. canum gene for ITS1, 5.8S rRNA and ITS2, complete sequence, specimen_voucher: 46856 (B)	950	950	92%	0.0	94%	AB683265.1	970,65	84,6%
Select seq emb|FN823081.1|	Tanacetum coccineum genomic DNA containing ITS1, 5.8S rRNA gene, ITS2, specimen voucher MPH:1144	950	950	92%	0.0	94%	FN823081.1	970,65	84,6%

Select seq gb|EF577323.1|	Tanacetum vulgare 18S ribosomal RNA gene, partial sequence; internal transcribed spacer 1, 5.8S ribosomal RNA gene, and internal transcribed spacer 2, complete sequence; and 28S ribosomal RNA gene, partial sequence	1009	1009	98%	0.0	94%	EF577323.1	967,82	84,4%
Select seq gb|EF577322.1|	Tanacetum ptarmiciflorum 18S ribosomal RNA gene, partial sequence; internal transcribed spacer 1, 5.8S ribosomal RNA gene, and internal transcribed spacer 2, complete sequence; and 28S ribosomal RNA gene, partial sequence	1009	1009	98%	0.0	94%	EF577322.1	967,82	84,4%
Select seq dbj|AB683289.1|	Tanacetum hololeucum gene for ITS1, 5.8S rRNA and ITS2, complete sequence, specimen_voucher: 6472 (S)	946	946	92%	0.0	94%	AB683289.1	966,57	84,3%
Select seq gb|AY603225.1|	Achillea ligustica internal transcribed spacer 1, 5.8S ribosomal RNA gene, and internal transcribed spacer 2, complete sequence	946	946	92%	0.0	94%	AY603225.1	966,57	84,3%
Select seq dbj|AB683304.1|	Tanacetum pinnatum gene for ITS1, 5.8S rRNA and ITS2, complete sequence, specimen_voucher: 1118 (MPH)	944	944	92%	0.0	94%	AB683304.1	964,52	84,1%
Select seq dbj|AB608333.1|	Tanacetum coccineum genes for ITS1, 5.8S rRNA, ITS2, complete sequence	944	944	92%	0.0	94%	AB608333.1	964,52	84,1%
Select seq gb|DQ267613.1|	Achillea vermicularis internal transcribed spacer 1, partial sequence; 5.8S ribosomal RNA gene, complete sequence; and internal transcribed spacer 2, partial sequence	942	942	92%	0.0	94%	DQ267613.1	962,48	83,9%
Select seq gb|DQ267610.1|	Achillea formosa subsp. formosa internal transcribed spacer 1, partial sequence; 5.8S ribosomal RNA gene, complete sequence; and internal transcribed spacer 2, partial sequence	941	941	92%	0.0	94%	DQ267610.1	961,46	83,8%
Select seq dbj|AB683288.1|	Tanacetum haussknechtii gene for ITS1, 5.8S rRNA and ITS2, complete sequence, specimen_voucher: 1193 (HUB)	939	939	92%	0.0	94%	AB683288.1	959,41	83,6%
Select seq dbj|AB683323.1|	Tanacetum sonbolii gene for ITS1, 5.8S rRNA and ITS2, complete sequence, specimen_voucher: 998 (MPH)	937	937	92%	0.0	94%	AB683323.1	957,37	83,4%
Select seq dbj|AB608331.1|	Tanacetum canescens genes for ITS1, 5.8S rRNA, ITS2, complete sequence	937	937	92%	0.0	94%	AB608331.1	957,37	83,4%
Select seq gb|EF577319.1|	Tanacetum cinerariifolium 18S ribosomal RNA gene, partial sequence; internal transcribed spacer 1, 5.8S ribosomal RNA gene, and internal transcribed spacer 2, complete sequence; and 28S ribosomal RNA gene, partial sequence	942	942	93%	0.0	94%	EF577319.1	952,13	83,0%
Select seq gb|KR150159.1|	Anthemis sp. Kh95 internal transcribed spacer 1, partial sequence; 5.8S ribosomal RNA gene gene, complete sequence; and internal transcribed spacer 2 region, partial sequence	996	996	99%	0.0	94%	KR150159.1	945,70	82,4%
Select seq dbj|AB683303.1|	Tanacetum parthenifolium gene for ITS1, 5.8S rRNA and ITS2, complete sequence, specimen_voucher: 1127 (MPH)	928	928	92%	0.0	93%	AB683303.1	938,09	81,8%
Select seq gb|KM887397.1|	Anacyclus pyrethrum isolate SBB-1407 18S ribosomal RNA gene, partial sequence; internal transcribed spacer 1, 5.8S ribosomal RNA gene, and internal transcribed spacer 2, complete sequence; and 28S ribosomal RNA gene, partial sequence	989	989	99%	0.0	93%	KM887397.1	929,06	81,0%
Select seq gb|KM887396.1|	Anacyclus pyrethrum isolate SBB-1364 18S ribosomal RNA gene, partial sequence; internal transcribed spacer 1, 5.8S ribosomal RNA gene, and internal transcribed spacer 2, complete sequence; and 28S ribosomal RNA gene, partial sequence	966	966	98%	0.0	93%	KM887396.1	916,71	79,9%
Select seq gb|KC816561.1|	Achillea santolina 18S ribosomal RNA gene, partial sequence; internal transcribed spacer 1, 5.8S ribosomal RNA gene, and internal transcribed spacer 2, complete sequence; and 28S ribosomal RNA gene, partial sequence	941	941	97%	0.0	92%	KC816561.1	892,49	77,8%
Select seq gb|GU818490.1|	Anacyclus valentinus isolate L1186 18S ribosomal RNA gene, partial sequence; internal transcribed spacer 1, 5.8S ribosomal RNA gene, and internal transcribed spacer 2, complete sequence; and 25S ribosomal RNA gene, partial sequence	944	944	99%	0.0	92%	GU818490.1	877,25	76,5%
								0,00	0,0%

Select for downloading or viewing reports	Kh017_trnL Description	Max score	Total score	Query cover	E value	Ident	Accession	(Ident/Cover)* Max score	Deviation from top hit
Select seq gb|AY603302.1|	Achillea ligustica voucher Ehrendorfer, 2001.09.21 (WU) trnL gene, partial sequence; trnL-trnF intergenic spacer, complete sequence; and tRNA-Phe (trnF) gene, partial sequence; chloroplast	1184	1184	93%	0.0	99%	AY603302.1	1260,39	100,0%
Select seq gb|AY603312.1|	Achillea macrophylla voucher Ehrendorfer, 2000.08.17 (WU) trnL gene, partial sequence; trnL-trnF intergenic spacer, complete sequence; and tRNA-Phe (trnF) gene, partial sequence; chloroplast	1195	1195	94%	0.0	99%	AY603312.1	1258,56	99,9%
Select seq gb|AY603303.1|	Achillea coarctata voucher Ehrendorfer & Danihelka, 2002.07.12-13 (WU) trnL gene, partial sequence; trnL-trnF intergenic spacer, complete sequence; and tRNA-Phe (trnF) gene, partial sequence; chloroplast	1195	1195	94%	0.0	99%	AY603303.1	1258,56	99,9%
Select seq gb|AY603290.1|	Achillea crithmifolia voucher Saukel B035 (WU) trnL gene, partial sequence; trnL-trnF intergenic spacer, complete sequence; and tRNA-Phe (trnF) gene, partial sequence; chloroplast	1195	1195	94%	0.0	99%	AY603290.1	1258,56	99,9%
Select seq gb|AY603309.1|	Achillea clavennae voucher Schoenswetter & Tribsch 56, 2000.07.04 (WU) trnL gene, partial sequence; trnL-trnF intergenic spacer, complete sequence; and tRNA-Phe (trnF) gene, partial sequence; chloroplast	1182	1182	93%	0.0	99%	AY603309.1	1258,26	99,8%
Select seq gb|AY603281.1|	Achillea millefolium subsp. ceretanica voucher Ehrendorfer, 2001 (WU) trnL gene, partial sequence; trnL-trnF intergenic spacer, complete sequence; and tRNA-Phe (trnF) gene, partial sequence; chloroplast	1181	1181	93%	0.0	99%	AY603281.1	1257,19	99,7%
Select seq gb|AY603324.1|	Achillea impatiens voucher Tribsch 9491, 2003.07.26 (WU) trnL gene, partial sequence; trnL-trnF intergenic spacer, complete sequence; and tRNA-Phe (trnF) gene, partial sequence; chloroplast	1190	1190	94%	0.0	99%	AY603324.1	1253,30	99,4%
Select seq gb|AY603318.1|	Achillea erba-rotta voucher Schoenswetter & Tribsch, 1998 (WU) trnL gene, partial sequence; trnL-trnF intergenic spacer, complete sequence; and tRNA-Phe (trnF) gene, partial sequence; chloroplast	1190	1190	94%	0.0	99%	AY603318.1	1253,30	99,4%
Select seq gb|AY603313.1|	Achillea oxyloba voucher Ehrendorfer-Schratt, 2001.23.08 (WU) trnL gene, partial sequence; trnL-trnF intergenic spacer, complete sequence; and tRNA-Phe (trnF) gene, partial sequence; chloroplast	1190	1190	94%	0.0	99%	AY603313.1	1253,30	99,4%
Select seq gb|AY603305.1|	Achillea x morisiana voucher Schoenswetter & Tribsch, 1998.07.21 (WU) trnL gene, partial sequence; trnL-trnF intergenic spacer, complete sequence; and tRNA-Phe (trnF) gene, partial sequence; chloroplast	1190	1190	94%	0.0	99%	AY603305.1	1253,30	99,4%
Select seq gb|AY603292.1|	Achillea pseudopectinata voucher Ehrendorfer & Danihelka, 2002.07.12 (WU) trnL gene, partial sequence; trnL-trnF intergenic spacer, complete sequence; and tRNA-Phe (trnF) gene, partial sequence; chloroplast	1190	1190	94%	0.0	99%	AY603292.1	1253,30	99,4%
Select seq gb|AY603307.1|	Achillea atrata voucher Schneeweiss 8943/2, 2002.08.23 (WU) trnL gene and trnL-trnF intergenic spacer, partial sequence; chloroplast	1177	1177	93%	0.0	99%	AY603307.1	1252,94	99,4%
Select seq gb|AY603279.1|	Achillea asplenifolia voucher Tod, 2001.10.03 (WU) trnL gene, partial sequence; and trnL-trnF intergenic spacer, complete sequence; chloroplast	1177	1177	93%	0.0	99%	AY603279.1	1252,94	99,4%
Select seq gb|EU128988.1|	Achillea millefolium subsp. ceretanica isolate Pyrenees no. 1 trnL-trnF intergenic spacer, partial sequence; chloroplast	1175	1175	93%	0.0	99%	EU128988.1	1250,81	99,2%
Select seq gb|EU128982.1|	Achillea asiatica isolate Huilang no. 1 trnL-trnF intergenic spacer, partial sequence; chloroplast	1175	1175	93%	0.0	99%	EU128982.1	1250,81	99,2%
Select seq gb|EU385030.1|	Achillea millefolium tRNA-Leu (trnL) gene and trnL-trnF intergenic spacer, complete sequence; chloroplast	1197	1197	95%	0.0	99%	EU385030.1	1247,40	99,0%
Select seq gb|AY603322.1|	Achillea salicifolia voucher Ehrendorfer & Guo K14, 2003.07.28 (WU) trnL gene, partial sequence; trnL-trnF intergenic spacer, complete sequence; and tRNA-Phe (trnF) gene, partial sequence; chloroplast	1184	1184	94%	0.0	99%	AY603322.1	1246,98	98,9%
Select seq gb|AY603319.1|	Achillea pindicola subsp. integrifolia voucher Schneeweiss & Gutermann, 2000.07.01 (WU) trnL gene, partial sequence; trnL-trnF intergenic spacer, complete sequence; and tRNA-Phe (trnF) gene, partial sequence; chloroplast	1184	1184	94%	0.0	99%	AY603319.1	1246,98	98,9%
Select seq gb|AY603308.1|	Achillea clavennae voucher Saukel, 2003.06.21 (WU) trnL gene, partial sequence; trnL-trnF intergenic spacer, complete sequence; and tRNA-Phe (trnF) gene, partial sequence; chloroplast	1184	1184	94%	0.0	99%	AY603308.1	1246,98	98,9%
Select seq gb|AY603300.1|	Achillea filipendulina voucher Saukel GR105 (WU) trnL gene, partial sequence; trnL-trnF intergenic spacer, complete sequence; and tRNA-Phe (trnF) gene, partial sequence; chloroplast	1184	1184	94%	0.0	99%	AY603300.1	1246,98	98,9%
Select seq gb|AY603296.1|	Achillea ochroleuca voucher Ehrendorfer, 2003.06.07 (WU) trnL gene, partial sequence; trnL-trnF intergenic spacer, complete sequence; and tRNA-Phe (trnF) gene, partial sequence; chloroplast	1184	1184	94%	0.0	99%	AY603296.1	1246,98	98,9%
Select seq gb|AY603288.1|	Achillea virescens voucher Ehrendorfer, 2002.07.31 (WU) trnL gene, partial sequence; trnL-trnF intergenic spacer, complete sequence; and tRNA-Phe (trnF) gene, partial sequence; chloroplast	1184	1184	94%	0.0	99%	AY603288.1	1246,98	98,9%
Select seq gb|AY603269.1|	Achillea schmakovii voucher Staudinger 5304 (WU) trnL gene, partial sequence; trnL-trnF intergenic spacer, complete sequence; and tRNA-Phe (trnF) gene, partial sequence; chloroplast	1184	1184	94%	0.0	99%	AY603269.1	1246,98	98,9%
Select seq gb|AY603284.1|	Achillea asiatica voucher Tan, 2002.05.15 (WU) trnL gene, partial sequence; trnL-trnF intergenic spacer, complete sequence; and tRNA-Phe (trnF) gene, partial sequence; chloroplast	1184	1184	94%	0.0	99%	AY603284.1	1246,98	98,9%
Select seq gb|AY603283.1|	Achillea alpina voucher Guo & Rao BJ02-5 (WU) trnL gene, partial sequence; trnL-trnF intergenic spacer, complete sequence; and tRNA-Phe (trnF) gene, partial sequence; chloroplast	1184	1184	94%	0.0	99%	AY603283.1	1246,98	98,9%
Select seq gb|AY603282.1|	Achillea wilsoniana voucher Guo & Rao 0203 (WU) trnL gene, partial sequence; trnL-trnF intergenic spacer, complete sequence; and tRNA-Phe (trnF) gene, partial sequence; chloroplast	1184	1184	94%	0.0	99%	AY603282.1	1246,98	98,9%
Select seq gb|AY603280.1|	Achillea euxina voucher Schanzer Ach12 (WU) trnL gene, partial sequence; trnL-trnF intergenic spacer, complete sequence; and tRNA-Phe (trnF) gene, partial sequence; chloroplast	1184	1184	94%	0.0	99%	AY603280.1	1246,98	98,9%

Select seq gb|AY603278.1|	Achillea roseoalba voucher Ehrendorfer & Ehrendorfer-Schratt, 2001.07.21 (WU) trnL gene, partial sequence; trnL-trnF intergenic spacer, complete sequence; and tRNA-Phe (trnF) gene, partial sequence; chloroplast	1184	1184	94% 0.0	99% AY603278.1	1246,98	98,9%
Select seq gb|AY603276.1|	Achillea collina voucher Ehrendorfer, 2001.07.22 (WU) trnL gene, partial sequence; trnL-trnF intergenic spacer, complete sequence; and tRNA-Phe (trnF) gene, partial sequence; chloroplast	1184	1184	94% 0.0	99% AY603276.1	1246,98	98,9%
Select seq gb|AY603273.1|	Achillea setacea voucher Saukel GR246 (WU) trnL gene, partial sequence; trnL-trnF intergenic spacer, complete sequence; and tRNA-Phe (trnF) gene, partial sequence; chloroplast	1184	1184	94% 0.0	99% AY603273.1	1246,98	98,9%
Select seq gb|AY603271.1|	Achillea styriaca voucher Saukel, 2002.07 (WU) trnL gene, partial sequence; trnL-trnF intergenic spacer, complete sequence; and tRNA-Phe (trnF) gene, partial sequence; chloroplast	1184	1184	94% 0.0	99% AY603271.1	1246,98	98,9%
Select seq gb|AY603265.1|	Achillea pannonica voucher Saukel, 2002.06.04 (WU) trnL gene, partial sequence; trnL-trnF intergenic spacer, complete sequence; and tRNA-Phe (trnF) gene, partial sequence; chloroplast	1184	1184	94% 0.0	99% AY603265.1	1246,98	98,9%
Select seq gb|DQ267619.1|	Achillea latiloba tRNA-Leu (trnL) gene, partial sequence; trnL-trnF intergenic spacer, complete sequence; and tRNA-Phe (trnF) gene, partial sequence; chloroplast	1184	1184	94% 0.0	99% DQ267619.1	1246,98	98,9%
Select seq gb|EU129025.1|	Achillea millefolium var. gigantea isolate Selma no. 1 trnL-trnF intergenic spacer, partial sequence; chloroplast	1170	1170	93% 0.0	99% EU129025.1	1245,48	98,8%
Select seq gb|EU128991.1|	Achillea millefolium var. alpicola isolate Hurricane no. 1 trnL-trnF intergenic spacer, partial sequence; chloroplast	1170	1170	93% 0.0	99% EU128991.1	1245,48	98,8%
Select seq gb|AY603306.1|	Achillea multifida voucher Ehrendorfer, 1993.07.24 (WU) trnL gene, partial sequence; trnL-trnF intergenic spacer, complete sequence; and tRNA-Phe (trnF) gene, partial sequence; chloroplast	1179	1179	94% 0.0	99% AY603306.1	1241,71	98,5%
Select seq gb|AY603310.1|	Achillea clusiana voucher Saukel, 2003.06.21 (WU) trnL gene, partial sequence; trnL-trnF intergenic spacer, complete sequence; and tRNA-Phe (trnF) gene, partial sequence; chloroplast	1179	1179	94% 0.0	99% AY603310.1	1241,71	98,5%
Select seq gb|AY603301.1|	Achillea micrantha voucher Ehrendorfer & Danihelka, 2002.07.12-13 (WU) trnL gene, partial sequence; trnL-trnF intergenic spacer, complete sequence; and tRNA-Phe (trnF) gene, partial sequence; chloroplast	1179	1179	94% 0.0	99% AY603301.1	1241,71	98,5%
Select seq gb|AY603287.1|	Achillea monticola voucher Schneeweiss et al. 6396, 2001.07.14 (WU) trnL gene, partial sequence; trnL-trnF intergenic spacer, complete sequence; and tRNA-Phe (trnF) gene, partial sequence; chloroplast	1179	1179	94% 0.0	99% AY603287.1	1241,71	98,5%
Select seq gb|AY603285.1|	Achillea asiatica voucher Staudinger 5893 (WU) trnL gene, partial sequence; trnL-trnF intergenic spacer, complete sequence; and tRNA-Phe (trnF) gene, partial sequence; chloroplast	1179	1179	94% 0.0	99% AY603285.1	1241,71	98,5%
Select seq gb|AY603297.1|	Achillea holosericea voucher Schneeweiss & Gutermann, 2000.06.28 (WU) trnL gene, partial sequence; trnL-trnF intergenic spacer, complete sequence; and tRNA-Phe (trnF) gene, partial sequence; chloroplast	1177	1177	94% 0.0	99% AY603297.1	1239,61	98,4%
Select seq gb|AY603293.1|	Achillea absinthoides voucher Schneeweiss & Gutermann, 2000.07.01 (WU) trnL gene, partial sequence; trnL-trnF intergenic spacer, complete sequence; and tRNA-Phe (trnF) gene, partial sequence; chloroplast	1177	1177	94% 0.0	99% AY603293.1	1239,61	98,4%
Select seq gb|EU128984.1|	Achillea asiatica isolate Altai 2x no. 1 trnL-trnF intergenic spacer, partial sequence; chloroplast	1164	1164	93% 0.0	99% EU128984.1	1239,10	98,3%
Select seq gb|AY603326.1|	Achillea pyrenaica voucher Schoenswetter et al. 6395 (WU) trnL gene, partial sequence; trnL-trnF intergenic spacer, complete sequence; and tRNA-Phe (trnF) gene, partial sequence; chloroplast	1164	1164	94% 0.0	99% AY603326.1	1225,91	97,3%
Select seq gb|AY603323.1|	Achillea ptarmica voucher Ehrendorfer, 2003.09.26 (WU) trnL gene, partial sequence; trnL-trnF intergenic spacer, complete sequence; and tRNA-Phe (trnF) gene, partial sequence; chloroplast	1164	1164	94% 0.0	99% AY603323.1	1225,91	97,3%
Select seq gb|AY603320.1|	Achillea acuminata voucher Guo & Rao 0201 (WU) trnL gene, partial sequence; trnL-trnF intergenic spacer, complete sequence; and tRNA-Phe (trnF) gene, partial sequence; chloroplast	1158	1158	94% 0.0	99% AY603320.1	1219,60	96,8%
Select seq gb|AY603316.1|	Achillea schurii voucher Ehrendorfer & Ehrendorfer-Schratt, 2002.07.12 (WU) trnL gene, partial sequence; trnL-trnF intergenic spacer, complete sequence; and tRNA-Phe (trnF) gene, partial sequence; chloroplast	1164	1164	94% 0.0	98% AY603316.1	1213,53	96,3%
Select seq gb|AY603270.1|	Achillea distans voucher Saukel 96010 (WU) trnL gene, partial sequence; trnL-trnF intergenic spacer, complete sequence; and tRNA-Phe (trnF) gene, partial sequence; chloroplast	1162	1162	94% 0.0	98% AY603270.1	1211,45	96,1%
Select seq gb|AY603274.1|	Achillea setacea voucher Ehrendorfer, 2002.03.24 (WU) trnL gene, partial sequence; trnL-trnF intergenic spacer, complete sequence; and tRNA-Phe (trnF) gene, partial sequence; chloroplast	1162	1162	94% 0.0	98% AY603274.1	1211,45	96,1%
Select seq gb|AY603325.1|	Achillea chamaemelifolia voucher Ehrendorfer & Danihelka, 2002.07.13 (WU) trnL gene, partial sequence; trnL-trnF intergenic spacer, complete sequence; and tRNA-Phe (trnF) gene, partial sequence; chloroplast	1157	1157	94% 0.0	98% AY603325.1	1206,23	95,7%
Select seq gb|AY603315.1|	Achillea schurii voucher Ehrendorfer, 1998.07.08 (WU) trnL gene, partial sequence; trnL-trnF intergenic spacer, complete sequence; and tRNA-Phe (trnF) gene, partial sequence; chloroplast	1153	1153	94% 0.0	98% AY603315.1	1202,06	95,4%
Select seq gb|AY603275.1|	Achillea setacea voucher Tod, 1999.11.01 (WU) trnL gene, partial sequence; trnL-trnF intergenic spacer, complete sequence; and tRNA-Phe (trnF) gene, partial sequence; chloroplast	1134	1134	94% 0.0	98% AY603275.1	1182,26	93,8%
Select seq gb|AY603272.1|	Achillea setacea voucher Saukel, 2002.06.04 (WU) trnL gene, partial sequence; trnL-trnF intergenic spacer, complete sequence; and tRNA-Phe (trnF) gene, partial sequence; chloroplast	1134	1134	94% 0.0	98% AY603272.1	1182,26	93,8%
Select seq gb|JX073780.1|	Artemisia argentea tRNA-Leu (trnL) gene and trnL-trnF intergenic spacer, partial sequence; chloroplast	1138	1138	97% 0.0	97% JX073780.1	1138,00	90,3%
Select seq gb|JN867592.1|	Chrysanthemum indicum voucher NJ026 chloroplast, complete genome	1162	1162	100% 0.0	96% JN867592.1	1115,52	88,5%

Select seq gb|KF736805.1|	Artemisia spicigera tRNA-Leu (trnL) gene and trnL-trnF intergenic spacer, partial sequence; chloroplast	1096	1096	95%	0.0	96%	KF736805.1	1107,54	87,9%
Select seq gb|JX073812.1|	Artemisia subdigitata tRNA-Leu (trnL) gene and trnL-trnF intergenic spacer, partial sequence; chloroplast	1118	1118	97%	0.0	96%	JX073812.1	1106,47	87,8%
Select seq gb|JX073808.1|	Artemisia tilesii tRNA-Leu (trnL) gene and trnL-trnF intergenic spacer, partial sequence; chloroplast	1118	1118	97%	0.0	96%	JX073808.1	1106,47	87,8%
Select seq gb|JX073748.1|	Artemisia santonicum tRNA-Leu (trnL) gene and trnL-trnF intergenic spacer, partial sequence; chloroplast	1118	1118	97%	0.0	96%	JX073748.1	1106,47	87,8%
Select seq gb|HM000099.1|	Chrysanthemum indicum isolate HEBSJZ tRNA-Leu (trnL) gene, partial sequence; trnL-trnF intergenic spacer, complete sequence; and tRNA-Phe (trnF) gene, partial sequence; chloroplast	1151	1151	100%	0.0	96%	HM000099.1	1104,96	87,7%
Select seq gb|HM000094.1|	Chrysanthemum indicum isolate HUBJZ tRNA-Leu (trnL) gene, partial sequence; trnL-trnF intergenic spacer, complete sequence; and tRNA-Phe (trnF) gene, partial sequence; chloroplast	1151	1151	100%	0.0	96%	HM000094.1	1104,96	87,7%
Select seq gb|JX073816.1|	Artemisia elongata tRNA-Leu (trnL) gene and trnL-trnF intergenic spacer, partial sequence; chloroplast	1114	1114	97%	0.0	96%	JX073816.1	1102,52	87,5%
Select seq gb|KF736807.1|	Artemisia turcomanica tRNA-Leu (trnL) gene and trnL-trnF intergenic spacer, partial sequence; chloroplast	1090	1090	95%	0.0	96%	KF736807.1	1101,47	87,4%
Select seq gb|KF736803.1|	Artemisia fragrans tRNA-Leu (trnL) gene and trnL-trnF intergenic spacer, partial sequence; chloroplast	1090	1090	95%	0.0	96%	KF736803.1	1101,47	87,4%
Select seq gb|JX073811.1|	Artemisia glomerata tRNA-Leu (trnL) gene and trnL-trnF intergenic spacer, partial sequence; chloroplast	1112	1112	97%	0.0	96%	JX073811.1	1100,54	87,3%
Select seq dbj|AB551235.1|	Artemisia capillaris chloroplast genes for tRNA-Leu intron and 3' exon, trnL-trnF intergenic spacer and tRNA-Phe	1133	1133	99%	0.0	96%	AB551235.1	1098,67	87,2%
Select seq gb|JX293720.1|	Artemisia frigida chloroplast, complete genome	1144	1144	100%	0.0	96%	JX293720.1	1098,24	87,1%
Select seq gb|HM000093.1|	Chrysanthemum indicum isolate SCMY tRNA-Leu (trnL) gene, partial sequence; trnL-trnF intergenic spacer, complete sequence; and tRNA-Phe (trnF) gene, partial sequence; chloroplast	1144	1144	100%	0.0	96%	HM000093.1	1098,24	87,1%
Select seq gb|HM000091.1|	Chrysanthemum indicum isolate HENXY tRNA-Leu (trnL) gene, partial sequence; trnL-trnF intergenic spacer, complete sequence; and tRNA-Phe (trnF) gene, partial sequence; chloroplast	1144	1144	100%	0.0	96%	HM000091.1	1098,24	87,1%
Select seq gb|JX073805.1|	Artemisia gracilescens tRNA-Leu (trnL) gene and trnL-trnF intergenic spacer, partial sequence; chloroplast	1096	1096	96%	0.0	96%	JX073805.1	1096,00	87,0%
Select seq gb|JX073828.1|	Kaschgaria komarovii tRNA-Leu (trnL) gene and trnL-trnF intergenic spacer, partial sequence; chloroplast	1107	1107	97%	0.0	96%	JX073828.1	1095,59	86,9%
Select seq gb|JX073827.1|	Artemisia absinthium tRNA-Leu (trnL) gene and trnL-trnF intergenic spacer, partial sequence; chloroplast	1107	1107	97%	0.0	96%	JX073827.1	1095,59	86,9%
Select seq gb|JX073814.1|	Mausolea eriocarpa tRNA-Leu (trnL) gene and trnL-trnF intergenic spacer, partial sequence; chloroplast	1107	1107	97%	0.0	96%	JX073814.1	1095,59	86,9%
Select seq gb|JX073791.1|	Artemisia tomentella tRNA-Leu (trnL) gene and trnL-trnF intergenic spacer, partial sequence; chloroplast	1107	1107	97%	0.0	96%	JX073791.1	1095,59	86,9%
Select seq dbj|AB685322.1|	Chrysanthemum indicum chloroplast DNA, contains tRNA-Leu(trnL) gene (intron and 3' exon), trnL-trnF intergenic spacer and tRNA-Phe(trnF) gene	1127	1127	99%	0.0	96%	AB685322.1	1092,85	86,7%
Select seq gb|JN867589.1|	Chrysanthemum indicum voucher HeN001 chloroplast, complete genome	1138	1138	100%	0.0	96%	JN867589.1	1092,48	86,7%
Select seq gb|HM000097.1|	Chrysanthemum indicum isolate JXPX tRNA-Leu (trnL) gene, partial sequence; trnL-trnF intergenic spacer, complete sequence; and tRNA-Phe (trnF) gene, partial sequence; chloroplast	1138	1138	100%	0.0	96%	HM000097.1	1092,48	86,7%
Select seq gb|HM000096.1|	Chrysanthemum indicum isolate YNKM tRNA-Leu (trnL) gene, partial sequence; trnL-trnF intergenic spacer, complete sequence; and tRNA-Phe (trnF) gene, partial sequence; chloroplast	1138	1138	100%	0.0	96%	HM000096.1	1092,48	86,7%
Select seq gb|HM000095.1|	Chrysanthemum indicum isolate HUNZZ tRNA-Leu (trnL) gene, partial sequence; trnL-trnF intergenic spacer, complete sequence; and tRNA-Phe (trnF) gene, partial sequence; chloroplast	1138	1138	100%	0.0	96%	HM000095.1	1092,48	86,7%
Select seq gb|HM000088.1|	Chrysanthemum indicum isolate GZGY tRNA-Leu (trnL) gene, partial sequence; trnL-trnF intergenic spacer, complete sequence; and tRNA-Phe (trnF) gene, partial sequence; chloroplast	1138	1138	100%	0.0	96%	HM000088.1	1092,48	86,7%
Select seq gb|HM000087.1|	Chrysanthemum indicum isolate AHCZ tRNA-Leu (trnL) gene, partial sequence; trnL-trnF intergenic spacer, complete sequence; and tRNA-Phe (trnF) gene, partial sequence; chloroplast	1138	1138	100%	0.0	96%	HM000087.1	1092,48	86,7%
Select seq gb|HM000085.1|	Chrysanthemum indicum isolate JSNJ tRNA-Leu (trnL) gene, partial sequence; trnL-trnF intergenic spacer, complete sequence; and tRNA-Phe (trnF) gene, partial sequence; chloroplast	1138	1138	100%	0.0	96%	HM000085.1	1092,48	86,7%
Select seq gb|JX073804.1|	Artemisia chamaemelifolia tRNA-Leu (trnL) gene and trnL-trnF intergenic spacer, partial sequence; chloroplast	1103	1103	97%	0.0	96%	JX073804.1	1091,63	86,6%

Select seq gb|JX073820.1|	Artemisia kauaiensis tRNA-Leu (trnL) gene and trnL-trnF intergenic spacer, partial sequence; chloroplast	1101	1101	97%	0.0	96%	JX073820.1	1089,65	86,5%
Select seq gb|JX073803.1|	Artemisia tournefortiana tRNA-Leu (trnL) gene and trnL-trnF intergenic spacer, partial sequence; chloroplast	1101	1101	97%	0.0	96%	JX073803.1	1089,65	86,5%
Select seq gb|JQ362483.1|	Chrysanthemum x morifolium chloroplast, complete genome	1133	1133	100%	0.0	96%	JQ362483.1	1087,68	86,3%
Select seq gb|HM000092.1|	Chrysanthemum indicum isolate GXGL tRNA-Leu (trnL) gene, partial sequence; trnL-trnF intergenic spacer, complete sequence; and tRNA-Phe (trnF) gene, partial sequence; chloroplast	1133	1133	100%	0.0	96%	HM000092.1	1087,68	86,3%
Select seq gb|HM000086.1|	Chrysanthemum indicum isolate JSWX tRNA-Leu (trnL) gene, partial sequence; trnL-trnF intergenic spacer, complete sequence; and tRNA-Phe (trnF) gene, partial sequence; chloroplast	1133	1133	100%	0.0	96%	HM000086.1	1087,68	86,3%
Select seq gb|HM000098.1|	Chrysanthemum indicum isolate FJFZ tRNA-Leu (trnL) gene, partial sequence; trnL-trnF intergenic spacer, complete sequence; and tRNA-Phe (trnF) gene, partial sequence; chloroplast	1127	1127	100%	0.0	96%	HM000098.1	1081,92	85,8%
Select seq gb|HM000089.1|	Chrysanthemum indicum isolate AHHF tRNA-Leu (trnL) gene, partial sequence; trnL-trnF intergenic spacer, complete sequence; and tRNA-Phe (trnF) gene, partial sequence; chloroplast	1127	1127	100%	0.0	96%	HM000089.1	1081,92	85,8%
Select seq gb|JX073823.1|	Artemisia globularia tRNA-Leu (trnL) gene and trnL-trnF intergenic spacer, partial sequence; chloroplast	1098	1098	97%	0.0	95%	JX073823.1	1075,36	85,3%
Select seq gb|JX073809.1|	Artemisia pattersonii tRNA-Leu (trnL) gene and trnL-trnF intergenic spacer, partial sequence; chloroplast	1096	1096	97%	0.0	95%	JX073809.1	1073,40	85,2%
Select seq gb|JX073807.1|	Artemisia martirensis tRNA-Leu (trnL) gene and trnL-trnF intergenic spacer, partial sequence; chloroplast	1096	1096	97%	0.0	95%	JX073807.1	1073,40	85,2%
Select seq gb|JX073825.1|	Artemisia potentilloides tRNA-Leu (trnL) gene and trnL-trnF intergenic spacer, partial sequence; chloroplast	1092	1092	97%	0.0	95%	JX073825.1	1069,48	84,9%
Select seq gb|JX073752.1|	Artemisia rothrockii tRNA-Leu (trnL) gene and trnL-trnF intergenic spacer, partial sequence; chloroplast	1092	1092	97%	0.0	95%	JX073752.1	1069,48	84,9%
Select seq gb|HM000090.1|	Chrysanthemum indicum isolate ZJWZ tRNA-Leu (trnL) gene, partial sequence; trnL-trnF intergenic spacer, complete sequence; and tRNA-Phe (trnF) gene, partial sequence; chloroplast	1122	1122	100%	0.0	95%	HM000090.1	1065,90	84,6%
Select seq gb|GU817959.1|	Anacyclus valentinus isolate L1186 tRNA-Leu (trnL) gene, partial sequence; trnL-trnF intergenic spacer, complete sequence; and tRNA-Phe (trnF) gene, partial sequence; plastid	1101	1101	100%	0.0	95%	GU817959.1	1045,95	83,0%
Select seq gb|KF887960.1|	Artemisia montana chloroplast, complete genome	1099	1099	100%	0.0	95%	KF887960.1	1044,05	82,8%
								0,00	

Select for downloading or viewing reports	Kh018_ITS Description	Max score	Total score	Query cover	E value	Ident	Accession	(Ident/Cover)* Max score	Deviation from top hit
Select seq gb|DQ900772.1|	Sideritis sventenii voucher JB 195 18S ribosomal RNA gene, partial sequence; internal transcribed spacer 1, 5.8S ribosomal RNA gene, and internal transcribed spacer 2, complete sequence; and 26S ribosomal RNA gene, partial sequence	224	345	87%	1,00E-54	89%	DQ900772.1	229,15	100,0%
Select seq gb|DQ900759.1|	Sideritis discolor voucher 8424 ORT 18S ribosomal RNA gene, partial sequence; internal transcribed spacer 1, 5.8S ribosomal RNA gene, and internal transcribed spacer 2, complete sequence; and 26S ribosomal RNA gene, partial sequence	224	345	87%	1,00E-54	89%	DQ900759.1	229,15	100,0%
Select seq gb|AF335640.1|	Sideritis tragoriganum JB 247 (TEX) 18S ribosomal RNA gene, partial sequence; internal transcribed spacer 1, 5.8S ribosomal RNA gene, and internal transcribed spacer 2, complete sequence; and 26S ribosomal RNA gene, partial sequence	224	351	87%	1,00E-54	89%	AF335640.1	229,15	100,0%
Select seq gb|AF335632.1|	Sideritis hirta from Portugal 18S ribosomal RNA gene, partial sequence; internal transcribed spacer 1, 5.8S ribosomal RNA gene, and internal transcribed spacer 2, complete sequence; and 26S ribosomal RNA gene, partial sequence	224	354	87%	1,00E-54	89%	AF335632.1	229,15	100,0%
Select seq gb|AF335624.1|	Sideritis antiatlantica 18S ribosomal RNA gene, partial sequence; internal transcribed spacer 1, 5.8S ribosomal RNA gene, and internal transcribed spacer 2, complete sequence; and 26S ribosomal RNA gene, partial sequence	226	356	88%	3,00E-55	89%	AF335624.1	228,57	99,7%
Select seq gb|AF335635.1|	Sideritis marmorinensis 18S ribosomal RNA gene, partial sequence; internal transcribed spacer 1, 5.8S ribosomal RNA gene, and internal transcribed spacer 2, complete sequence; and 26S ribosomal RNA gene, partial sequence	226	369	89%	3,00E-55	89%	AF335635.1	226,00	98,6%
Select seq gb|AF335633.1|	Sideritis hyssopifolia 18S ribosomal RNA gene, partial sequence; internal transcribed spacer 1, 5.8S ribosomal RNA gene, and internal transcribed spacer 2, complete sequence; and 26S ribosomal RNA gene, partial sequence	226	369	89%	3,00E-55	89%	AF335633.1	226,00	98,6%
Select seq gb|AF335621.1|	Sideritis syriaca from Turkey 18S ribosomal RNA gene, partial sequence; internal transcribed spacer 1, 5.8S ribosomal RNA gene, and internal transcribed spacer 2, complete sequence; and 26S ribosomal RNA gene, partial sequence	226	366	89%	3,00E-55	89%	AF335621.1	226,00	98,6%
Select seq gb|AF335620.1|	Sideritis syriaca from Greece 18S ribosomal RNA gene, partial sequence; internal transcribed spacer 1, 5.8S ribosomal RNA gene, and internal transcribed spacer 2, complete sequence; and 26S ribosomal RNA gene, partial sequence	226	364	89%	3,00E-55	89%	AF335620.1	226,00	98,6%
Select seq gb|AF335639.1|	Sideritis tragoriganum JB 241 (TEX) 18S ribosomal RNA gene, partial sequence; internal transcribed spacer 1, 5.8S ribosomal RNA gene, and internal transcribed spacer 2, complete sequence; and 26S ribosomal RNA gene, partial sequence	224	367	89%	1,00E-54	89%	AF335639.1	224,00	97,8%
Select seq gb|AF335636.1|	Sideritis murgetana 18S ribosomal RNA gene, partial sequence; internal transcribed spacer 1, 5.8S ribosomal RNA gene, and internal transcribed spacer 2, complete sequence; and 26S ribosomal RNA gene, partial sequence	224	366	89%	1,00E-54	89%	AF335636.1	224,00	97,8%
Select seq gb|AF335629.1|	Sideritis glacialis 18S ribosomal RNA gene, partial sequence; internal transcribed spacer 1, 5.8S ribosomal RNA gene, and internal transcribed spacer 2, complete sequence; and 26S ribosomal RNA gene, partial sequence	224	367	89%	1,00E-54	89%	AF335629.1	224,00	97,8%
Select seq gb|AF335637.1|	Sideritis pungens subsp. javalambrensis 18S ribosomal RNA gene, partial sequence; internal transcribed spacer 1, 5.8S ribosomal RNA gene, and internal transcribed spacer 2, complete sequence; and 26S ribosomal RNA gene, partial sequence	220	347	87%	1,00E-53	88%	AF335637.1	222,53	97,1%
Select seq gb|KF577378.1|	Stachys multicaulis voucher SKU:116 18S ribosomal RNA gene, partial sequence; internal transcribed spacer 1, 5.8S ribosomal RNA gene, and internal transcribed spacer 2, complete sequence; and 28S ribosomal RNA gene, partial sequence	233	430	100%	2,00E-57	86%	KF577378.1	200,38	87,4%
Select seq gb|JF301507.1|	Hyptis lorentziana isolate FS6968 18S ribosomal RNA gene, partial sequence; internal transcribed spacer 1, 5.8S ribosomal RNA gene, and internal transcribed spacer 2, complete sequence; and 26S ribosomal RNA gene, partial sequence	226	226	44%	3,00E-55	90%	JF301507.1	0,00	0,0%
Select seq gb|KF529649.1|	Stachys turcomanica isolate SN-117 internal transcribed spacer 1, partial sequence; 5.8S ribosomal RNA gene, complete sequence; and internal transcribed spacer 2, partial sequence	228	228	45%	8,00E-56	90%	KF529649.1	0,00	0,0%
Select seq gb|KF529637.1|	Stachys schtschegleevii isolate SN-105 internal transcribed spacer 1, partial sequence; 5.8S ribosomal RNA gene, complete sequence; and internal transcribed spacer 2, partial sequence	228	228	45%	8,00E-56	90%	KF529637.1	0,00	0,0%
Select seq gb|KF529616.1|	Stachys mollissima isolate SN-84 internal transcribed spacer 1, partial sequence; 5.8S ribosomal RNA gene, complete sequence; and internal transcribed spacer 2, partial sequence	228	228	45%	8,00E-56	90%	KF529616.1	0,00	0,0%
Select seq gb|KF529610.1|	Stachys lavandulifolia isolate SN-78 internal transcribed spacer 1, partial sequence; 5.8S ribosomal RNA gene, complete sequence; and internal transcribed spacer 2, partial sequence	228	228	45%	8,00E-56	90%	KF529610.1	0,00	0,0%
Select seq gb|KF529609.1|	Stachys lavandulifolia isolate SN-77 internal transcribed spacer 1, partial sequence; 5.8S ribosomal RNA gene, complete sequence; and internal transcribed spacer 2, partial sequence	228	228	45%	8,00E-56	90%	KF529609.1	0,00	0,0%
Select seq gb|KF529601.1|	Stachys inflata isolate SN-69 internal transcribed spacer 1, partial sequence; 5.8S ribosomal RNA gene, complete sequence; and internal transcribed spacer 2, partial sequence	228	228	45%	8,00E-56	90%	KF529601.1	0,00	0,0%
Select seq gb|AY792833.1|	Valeriana aretioides internal transcribed spacer 1, partial sequence; 5.8S ribosomal RNA gene, complete sequence; and internal transcribed spacer 2, partial sequence	228	228	45%	8,00E-56	90%	AY792833.1	0,00	0,0%
Select seq gb|JQ730026.1|	Stachys lavandulifolia voucher Akcicek 5335 & Dirmenci internal transcribed spacer 1, partial sequence; 5.8S ribosomal RNA gene, complete sequence; and internal transcribed spacer 2, partial sequence	231	231	46%	6,00E-57	90%	JQ730026.1	0,00	0,0%
Select seq gb|KU203459.1|	Phyllarthron sp. 2 SB-2016 voucher Nusbaumer 2584 internal transcribed spacer 1, partial sequence; 5.8S ribosomal RNA gene, complete sequence; and internal transcribed spacer 2, partial sequence	226	226	45%	3,00E-55	89%	KU203459.1	0,00	0,0%
Select seq gb|KP267800.1|	Deprea sylvarum internal transcribed spacer 1, partial sequence; 5.8S ribosomal RNA gene, complete sequence; and internal transcribed spacer 2, partial sequence	224	224	45%	1,00E-54	89%	KP267800.1	0,00	0,0%
Select seq gb|KP267799.1|	Deprea glabra internal transcribed spacer 1, partial sequence; 5.8S ribosomal RNA gene, complete sequence; and internal transcribed spacer 2, partial sequence	224	224	45%	1,00E-54	89%	KP267799.1	0,00	0,0%

Select seq gb|KP267796.1|	Deprea nubicola internal transcribed spacer 1, partial sequence; 5.8S ribosomal RNA gene, complete sequence; and internal transcribed spacer 2, partial sequence	224	224	45%	1,00E-54	89%	KP267796.1	0,00	0,0%
Select seq gb|KP267771.1|	Deprea purpurea internal transcribed spacer 1, partial sequence; 5.8S ribosomal RNA gene, complete sequence; and internal transcribed spacer 2, partial sequence	224	224	45%	1,00E-54	89%	KP267771.1	0,00	0,0%
Select seq gb|KM270522.1|	Withania sp. 'ashwagandha' clone SBB-WA025(3) internal transcribed spacer 1, partial sequence; 5.8S ribosomal RNA gene, complete sequence; and internal transcribed spacer 2, partial sequence	224	224	45%	1,00E-54	89%	KM270522.1	0,00	0,0%
Select seq gb|KM270521.1|	Withania sp. 'ashwagandha' clone SBB-WA025(2) internal transcribed spacer 1, partial sequence; 5.8S ribosomal RNA gene, complete sequence; and internal transcribed spacer 2, partial sequence	224	224	45%	1,00E-54	89%	KM270521.1	0,00	0,0%
Select seq gb|KC768878.1|	Physaliastrum heterophyllum voucher Lihq0435 18S ribosomal RNA gene, partial sequence; internal transcribed spacer 1, 5.8S ribosomal RNA and genes, complete sequence; and 26S ribosomal RNA gene, partial sequence	224	224	45%	1,00E-54	89%	KC768878.1	0,00	0,0%
Select seq dbj|AB295101.1|	Salvia nipponica genes for ITS1, 5.8S rRNA, ITS2, partial and complete sequence, specimen_voucher: Sudarmono et al Jap03/66 BO (Herbarium Bogoriense)	224	224	45%	1,00E-54	89%	AB295101.1	0,00	0,0%
Select seq gb|JF330297.1|	Stachys cretica subsp. smyrnea voucher 4732 internal transcribed spacer 1, partial sequence; 5.8S ribosomal RNA gene, complete sequence; and internal transcribed spacer 2, partial sequence	226	226	46%	3,00E-55	89%	JF330297.1	0,00	0,0%
Select seq gb|JF330293.1|	Stachys cretica subsp. garana voucher EA5185 internal transcribed spacer 1, partial sequence; 5.8S ribosomal RNA gene, complete sequence; and internal transcribed spacer 2, partial sequence	226	226	46%	3,00E-55	89%	JF330293.1	0,00	0,0%
Select seq gb|DQ900771.1|	Sideritis soluta subsp. gueimaris voucher JB 221 18S ribosomal RNA gene, partial sequence; internal transcribed spacer 1, 5.8S ribosomal RNA gene, and internal transcribed spacer 2, complete sequence; and 26S ribosomal RNA gene, partial sequence	226	226	46%	3,00E-55	89%	DQ900771.1	0,00	0,0%
Select seq gb|DQ900769.1|	Sideritis oroteneriffae var. oroteneriffae voucher JB 207 18S ribosomal RNA gene, partial sequence; internal transcribed spacer 1, 5.8S ribosomal RNA gene, and internal transcribed spacer 2, complete sequence; and 26S ribosomal RNA gene, partial sequence	226	226	46%	3,00E-55	89%	DQ900769.1	0,00	0,0%
Select seq gb|DQ900765.1|	Sideritis marmorea voucher 32073 ORT 18S ribosomal RNA gene, partial sequence; internal transcribed spacer 1, 5.8S ribosomal RNA gene, and internal transcribed spacer 2, complete sequence; and 26S ribosomal RNA gene, partial sequence	226	226	46%	3,00E-55	89%	DQ900765.1	0,00	0,0%
Select seq gb|DQ900764.1|	Sideritis lotsyi voucher JB 206 18S ribosomal RNA gene, partial sequence; internal transcribed spacer 1, 5.8S ribosomal RNA gene, and internal transcribed spacer 2, complete sequence; and 26S ribosomal RNA gene, partial sequence	226	226	46%	3,00E-55	89%	DQ900764.1	0,00	0,0%
Select seq gb|DQ900763.1|	Sideritis kuegleriana voucher 16767 ORT 18S ribosomal RNA gene, partial sequence; internal transcribed spacer 1, 5.8S ribosomal RNA gene, and internal transcribed spacer 2, complete sequence; and 26S ribosomal RNA gene, partial sequence	226	226	46%	3,00E-55	89%	DQ900763.1	0,00	0,0%
Select seq gb|DQ900762.1|	Sideritis infernalis voucher JB 225 18S ribosomal RNA gene, partial sequence; internal transcribed spacer 1, 5.8S ribosomal RNA gene, and internal transcribed spacer 2, complete sequence; and 26S ribosomal RNA gene, partial sequence	226	226	46%	3,00E-55	89%	DQ900762.1	0,00	0,0%
Select seq gb|DQ900760.1|	Sideritis ferrensis voucher JB 204 18S ribosomal RNA gene, partial sequence; internal transcribed spacer 1, 5.8S ribosomal RNA gene, and internal transcribed spacer 2, complete sequence; and 26S ribosomal RNA gene, partial sequence	226	226	46%	3,00E-55	89%	DQ900760.1	0,00	0,0%
Select seq gb|DQ900757.1|	Sideritis dasygnaphala voucher 18691 LPA 18S ribosomal RNA gene, partial sequence; internal transcribed spacer 1, 5.8S ribosomal RNA gene, and internal transcribed spacer 2, complete sequence; and 26S ribosomal RNA gene, partial sequence	226	226	46%	3,00E-55	89%	DQ900757.1	0,00	0,0%
Select seq gb|DQ900756.1|	Sideritis cystosiphon voucher JB 222 18S ribosomal RNA gene, partial sequence; internal transcribed spacer 1, 5.8S ribosomal RNA gene, and internal transcribed spacer 2, complete sequence; and 26S ribosomal RNA gene, partial sequence	226	226	46%	3,00E-55	89%	DQ900756.1	0,00	0,0%
Select seq gb|DQ900751.1|	Sideritis brevicaulis voucher JB 226 18S ribosomal RNA gene, partial sequence; internal transcribed spacer 1, 5.8S ribosomal RNA gene, and internal transcribed spacer 2, complete sequence; and 26S ribosomal RNA gene, partial sequence	226	226	46%	3,00E-55	89%	DQ900751.1	0,00	0,0%
Select seq gb|DQ900750.1|	Sideritis barbellata voucher JB 262 18S ribosomal RNA gene, partial sequence; internal transcribed spacer 1, 5.8S ribosomal RNA gene, and internal transcribed spacer 2, complete sequence; and 26S ribosomal RNA gene, partial sequence	226	226	46%	3,00E-55	89%	DQ900750.1	0,00	0,0%
Select seq gb|AF335611.1|	Sideritis soluta subsp. soluta 18S ribosomal RNA gene, partial sequence; internal transcribed spacer 1, 5.8S ribosomal RNA gene, and internal transcribed spacer 2, complete sequence; and 26S ribosomal RNA gene, partial sequence	226	226	46%	3,00E-55	89%	AF335611.1	0,00	0,0%
Select seq gb|KF805088.1|	Stachys aegyptiaca voucher KSUFS10 18S ribosomal RNA gene, partial sequence; internal transcribed spacer 1, 5.8S ribosomal RNA gene, and internal transcribed spacer 2, complete sequence; and 28S ribosomal RNA gene, partial sequence	224	224	46%	1,00E-54	89%	KF805088.1	0,00	0,0%
Select seq gb|KF529642.1|	Stachys swainsonii isolate SN-110 internal transcribed spacer 1, partial sequence; 5.8S ribosomal RNA gene, complete sequence; and internal transcribed spacer 2, partial sequence	224	224	46%	1,00E-54	89%	KF529642.1	0,00	0,0%
Select seq gb|KF529639.1|	Stachys spinosa isolate SN-107 internal transcribed spacer 1, partial sequence; 5.8S ribosomal RNA gene, complete sequence; and internal transcribed spacer 2, partial sequence	224	224	46%	1,00E-54	89%	KF529639.1	0,00	0,0%
Select seq gb|KF529635.1|	Stachys setifera isolate SN-103 internal transcribed spacer 1, partial sequence; 5.8S ribosomal RNA gene, complete sequence; and internal transcribed spacer 2, partial sequence	224	224	46%	1,00E-54	89%	KF529635.1	0,00	0,0%
Select seq gb|KF529629.1|	Stachys pubescens isolate SN-97 internal transcribed spacer 1, partial sequence; 5.8S ribosomal RNA gene, complete sequence; and internal transcribed spacer 2, partial sequence	224	224	46%	1,00E-54	89%	KF529629.1	0,00	0,0%
Select seq gb|KF529618.1|	Stachys mucronata isolate SN-86 internal transcribed spacer 1, partial sequence; 5.8S ribosomal RNA gene, complete sequence; and internal transcribed spacer 2, partial sequence	224	224	46%	1,00E-54	89%	KF529618.1	0,00	0,0%
Select seq gb|KF529617.1|	Stachys mucronata isolate SN-85 internal transcribed spacer 1, partial sequence; 5.8S ribosomal RNA gene, complete sequence; and internal transcribed spacer 2, partial sequence	224	224	46%	1,00E-54	89%	KF529617.1	0,00	0,0%

Select seq gb|KF529615.1|	Stachys menthoides isolate SN-83 internal transcribed spacer 1, partial sequence; 5.8S ribosomal RNA gene, complete sequence; and internal transcribed spacer 2, partial sequence	224	224	46%	1,00E-54	89% KF529615.1	0,00	0,0%
Select seq gb|KF529595.1|	Stachys graeca isolate SN-63 internal transcribed spacer 1, partial sequence; 5.8S ribosomal RNA gene, complete sequence; and internal transcribed spacer 2, partial sequence	224	224	46%	1,00E-54	89% KF529595.1	0,00	0,0%
Select seq gb|KF529580.1|	Stachys chrysantha isolate SN-48 internal transcribed spacer 1, partial sequence; 5.8S ribosomal RNA gene, complete sequence; and internal transcribed spacer 2, partial sequence	224	224	46%	1,00E-54	89% KF529580.1	0,00	0,0%
Select seq gb|KF529557.1|	Stachys aegyptiaca isolate SN-25 internal transcribed spacer 1, partial sequence; 5.8S ribosomal RNA gene, complete sequence; and internal transcribed spacer 2, partial sequence	224	224	46%	1,00E-54	89% KF529557.1	0,00	0,0%
Select seq gb|JF330316.1|	Stachys viticina voucher 4746 internal transcribed spacer 1, partial sequence; 5.8S ribosomal RNA gene, complete sequence; and internal transcribed spacer 2, partial sequence	224	224	46%	1,00E-54	89% JF330316.1	0,00	0,0%
Select seq gb|JF330310.1|	Stachys suricantha voucher 4624 internal transcribed spacer 1, partial sequence; 5.8S ribosomal RNA gene, complete sequence; and internal transcribed spacer 2, partial sequence	224	224	46%	1,00E-54	89% JF330310.1	0,00	0,0%
Select seq gb|JF330303.1|	Stachys huber-morathii voucher 5715a internal transcribed spacer 1, partial sequence; 5.8S ribosomal RNA gene, complete sequence; and internal transcribed spacer 2, partial sequence	224	224	46%	1,00E-54	89% JF330303.1	0,00	0,0%
Select seq gb|JF330301.1|	Stachys germanica subsp. heldreichii voucher 4629 internal transcribed spacer 1, partial sequence; 5.8S ribosomal RNA gene, complete sequence; and internal transcribed spacer 2, partial sequence	224	224	46%	1,00E-54	89% JF330301.1	0,00	0,0%
Select seq gb|JF330296.1|	Stachys cretica subsp. mersinae internal transcribed spacer 1, partial sequence; 5.8S ribosomal RNA gene, complete sequence; and internal transcribed spacer 2, partial sequence	224	224	46%	1,00E-54	89% JF330296.1	0,00	0,0%
Select seq gb|JF330290.1|	Stachys byzantina voucher 4158 internal transcribed spacer 1, partial sequence; 5.8S ribosomal RNA gene, complete sequence; and internal transcribed spacer 2, partial sequence	224	224	46%	1,00E-54	89% JF330290.1	0,00	0,0%
Select seq gb|JF330289.1|	Stachys cretica subsp. bulgarica voucher 4558 internal transcribed spacer 1, partial sequence; 5.8S ribosomal RNA gene, complete sequence; and internal transcribed spacer 2, partial sequence	224	224	46%	1,00E-54	89% JF330289.1	0,00	0,0%
Select seq gb|JQ730030.1|	Stachys diversifolia voucher A. Duran 6318 internal transcribed spacer 1, partial sequence; 5.8S ribosomal RNA gene, complete sequence; and internal transcribed spacer 2, partial sequence	224	224	46%	1,00E-54	89% JQ730030.1	0,00	0,0%
Select seq gb|JQ730028.1|	Stachys iberica voucher Akcicek 2848 internal transcribed spacer 1, partial sequence; 5.8S ribosomal RNA gene, complete sequence; and internal transcribed spacer 2, partial sequence	224	224	46%	1,00E-54	89% JQ730028.1	0,00	0,0%
Select seq gb|JQ730025.1|	Stachys bombycina voucher Akcicek 5127 & Dirmenci internal transcribed spacer 1, partial sequence; 5.8S ribosomal RNA gene, complete sequence; and internal transcribed spacer 2, partial sequence	224	224	46%	1,00E-54	89% JQ730025.1	0,00	0,0%
Select seq gb|DQ860351.1|	Dicerandra cornutissima isolate COR17 internal transcribed spacer 1, partial sequence; 5.8S ribosomal RNA gene, complete sequence; and internal transcribed spacer 2, partial sequence	224	224	46%	1,00E-54	89% DQ860351.1	0,00	0,0%
Select seq gb|DQ900767.1|	Sideritis nutans voucher JB 201 18S ribosomal RNA gene, partial sequence; internal transcribed spacer 1, 5.8S ribosomal RNA gene, and internal transcribed spacer 2, complete sequence; and 26S ribosomal RNA gene, partial sequence	224	224	46%	1,00E-54	89% DQ900767.1	0,00	0,0%
Select seq gb|DQ900758.1|	Sideritis dendro-chahorra voucher JB 224 18S ribosomal RNA gene, partial sequence; internal transcribed spacer 1, 5.8S ribosomal RNA gene, and internal transcribed spacer 2, complete sequence; and 26S ribosomal RNA gene, partial sequence	224	224	46%	1,00E-54	89% DQ900758.1	0,00	0,0%
Select seq gb|DQ900755.1|	Sideritis cretica subsp. spicata voucher JB 213 18S ribosomal RNA gene, partial sequence; internal transcribed spacer 1, 5.8S ribosomal RNA gene, and internal transcribed spacer 2, complete sequence; and 26S ribosomal RNA gene, partial sequence	224	224	46%	1,00E-54	89% DQ900755.1	0,00	0,0%
Select seq gb|DQ900754.1|	Sideritis cretica subsp. cretica voucher JB 218 18S ribosomal RNA gene, partial sequence; internal transcribed spacer 1, 5.8S ribosomal RNA gene, and internal transcribed spacer 2, complete sequence; and 26S ribosomal RNA gene, partial sequence	224	224	46%	1,00E-54	89% DQ900754.1	0,00	0,0%
Select seq gb|DQ900753.1|	Sideritis canariensis voucher JB 227 18S ribosomal RNA gene, partial sequence; internal transcribed spacer 1, 5.8S ribosomal RNA gene, and internal transcribed spacer 2, complete sequence; and 26S ribosomal RNA gene, partial sequence	224	224	46%	1,00E-54	89% DQ900753.1	0,00	0,0%
Select seq gb|DQ900752.1|	Sideritis canariensis voucher JB 228 18S ribosomal RNA gene, partial sequence; internal transcribed spacer 1, 5.8S ribosomal RNA gene, and internal transcribed spacer 2, complete sequence; and 26S ribosomal RNA gene, partial sequence	224	224	46%	1,00E-54	89% DQ900752.1	0,00	0,0%
Select seq gb|KF848298.1|	Sesuvium portulacastrum voucher AUFMS260 18S ribosomal RNA gene, partial sequence; internal transcribed spacer 1, 5.8S ribosomal RNA gene, and internal transcribed spacer 2, complete sequence; and 28S ribosomal RNA gene, partial sequence	226	226	47%	3,00E-55	89% KF848298.1	0,00	0,0%
Select seq gb|KC185421.1|	Sesuvium portulacastrum isolate Halo6 internal transcribed spacer 1 and 5.8S ribosomal RNA gene, partial sequence	226	226	47%	3,00E-55	89% KC185421.1	0,00	0,0%
Select seq gb|FJ784241.1|	Sesuvium portulacastrum voucher RK 402 18S ribosomal RNA gene, partial sequence; internal transcribed spacer 1, 5.8S ribosomal RNA gene, and internal transcribed spacer 2, complete sequence; and 28S ribosomal RNA gene, partial sequence	226	226	47%	3,00E-55	89% FJ784241.1	0,00	0,0%
Select seq gb|DQ900770.1|	Sideritis pumila voucher 22226 ORT 18S ribosomal RNA gene, partial sequence; internal transcribed spacer 1, 5.8S ribosomal RNA gene, and internal transcribed spacer 2, complete sequence; and 26S ribosomal RNA gene, partial sequence	222	222	46%	4,00E-54	88% DQ900770.1	0,00	0,0%
Select seq gb|JQ669095.1|	Dicerandra christmanii voucher Bok Tower Gardens, B. Drew s.n. 18S ribosomal RNA gene, internal transcribed spacer 1, 5.8S ribosomal RNA gene, internal transcribed spacer 2, and 26S ribosomal RNA gene, region	224	224	47%	1,00E-54	89% JQ669095.1	0,00	0,0%
Select seq gb|AY943489.1|	Dicerandra thinicola internal transcribed spacer 1, partial sequence; 5.8S ribosomal RNA gene, complete sequence; and internal transcribed spacer 2, partial sequence	224	224	47%	1,00E-54	89% AY943489.1	0,00	0,0%

Select seq gb|DQ860375.1|	Dicerandra immaculata var. savannarum isolate SAV27 internal transcribed spacer 1, partial sequence; 5.8S ribosomal RNA gene, complete sequence; and internal transcribed spacer 2, partial sequence	224	224	47%	1,00E-54	89% DQ860375.1	0,00	0,0%
Select seq gb|DQ860372.1|	Dicerandra linearifolia var. robustior isolate ROB12 internal transcribed spacer 1, partial sequence; 5.8S ribosomal RNA gene, complete sequence; and internal transcribed spacer 2, partial sequence	224	224	47%	1,00E-54	89% DQ860372.1	0,00	0,0%
Select seq gb|DQ860366.1|	Dicerandra frutescens subsp. modesta isolate MOD19 internal transcribed spacer 1, partial sequence; 5.8S ribosomal RNA gene, complete sequence; and internal transcribed spacer 2, partial sequence	224	224	47%	1,00E-54	89% DQ860366.1	0,00	0,0%
Select seq gb|DQ860365.1|	Dicerandra linearifolia var. linearifolia isolate LIN7 internal transcribed spacer 1, partial sequence; 5.8S ribosomal RNA gene, complete sequence; and internal transcribed spacer 2, partial sequence	224	224	47%	1,00E-54	89% DQ860365.1	0,00	0,0%
Select seq gb|DQ860363.1|	Dicerandra linearifolia var. linearifolia isolate LIN4 internal transcribed spacer 1, partial sequence; 5.8S ribosomal RNA gene, complete sequence; and internal transcribed spacer 2, partial sequence	224	224	47%	1,00E-54	89% DQ860363.1	0,00	0,0%
Select seq gb|DQ860362.1|	Dicerandra linearifolia var. linearifolia isolate LIN3 internal transcribed spacer 1, partial sequence; 5.8S ribosomal RNA gene, complete sequence; and internal transcribed spacer 2, partial sequence	224	224	47%	1,00E-54	89% DQ860362.1	0,00	0,0%
Select seq gb|DQ860360.1|	Dicerandra linearifolia var. linearifolia isolate LIN2 internal transcribed spacer 1, partial sequence; 5.8S ribosomal RNA gene, complete sequence; and internal transcribed spacer 2, partial sequence	224	224	47%	1,00E-54	89% DQ860360.1	0,00	0,0%
Select seq gb|DQ860359.1|	Dicerandra linearifolia var. linearifolia isolate LIN1 internal transcribed spacer 1, partial sequence; 5.8S ribosomal RNA gene, complete sequence; and internal transcribed spacer 2, partial sequence	224	224	47%	1,00E-54	89% DQ860359.1	0,00	0,0%
Select seq gb|DQ860358.1|	Dicerandra immaculata var. immaculata isolate IMA26 internal transcribed spacer 1, partial sequence; 5.8S ribosomal RNA gene, complete sequence; and internal transcribed spacer 2, partial sequence	224	224	47%	1,00E-54	89% DQ860358.1	0,00	0,0%
Select seq gb|DQ860357.1|	Dicerandra sp. Huck 5628-30 internal transcribed spacer 1, partial sequence; 5.8S ribosomal RNA gene, complete sequence; and internal transcribed spacer 2, partial sequence	224	224	47%	1,00E-54	89% DQ860357.1	0,00	0,0%
Select seq gb|DQ860355.1|	Dicerandra frutescens subsp. frutescens isolate FRU24 internal transcribed spacer 1, partial sequence; 5.8S ribosomal RNA gene, complete sequence; and internal transcribed spacer 2, partial sequence	224	224	47%	1,00E-54	89% DQ860355.1	0,00	0,0%
Select seq gb|AY943488.1|	Dicerandra densiflora internal transcribed spacer 1, partial sequence; 5.8S ribosomal RNA gene, complete sequence; and internal transcribed spacer 2, partial sequence	224	224	47%	1,00E-54	89% AY943488.1	0,00	0,0%
Select seq gb|DQ860350.1|	Dicerandra christmanii isolate CHR23 internal transcribed spacer 1, partial sequence; 5.8S ribosomal RNA gene, complete sequence; and internal transcribed spacer 2, partial sequence	224	224	47%	1,00E-54	89% DQ860350.1	0,00	0,0%
Select seq gb|DQ667234.1|	Dicerandra odoratissima isolate x059 18S ribosomal RNA gene, partial sequence; internal transcribed spacer 1, 5.8S ribosomal RNA gene, and internal transcribed spacer 2, complete sequence; and 28S ribosomal RNA gene, partial sequence	224	224	47%	1,00E-54	89% DQ667234.1	0,00	0,0%
Select seq gb|DQ900761.1|	Sideritis gomeraea subsp. perezii voucher JB 200 18S ribosomal RNA gene, partial sequence; internal transcribed spacer 1, 5.8S ribosomal RNA gene, and internal transcribed spacer 2, complete sequence; and 26S ribosomal RNA gene, partial sequence	220	220	46%	1,00E-53	88% DQ900761.1	0,00	0,0%
Select seq gb|DQ314164.1|	Witheringia solanacea voucher D'Arcy 16399 (MO) internal transcribed spacer 1, partial sequence; 5.8S ribosomal RNA gene and internal transcribed spacer 2, complete sequence; and 25S ribosomal RNA gene, partial sequence	224	332	61%	1,00E-54	89% DQ314164.1	0,00	0,0%
Select seq gb|DQ314162.1|	Leucophysalis grandiflora voucher Olmstead S-30 (WTU) internal transcribed spacer 1, partial sequence; 5.8S ribosomal RNA gene and internal transcribed spacer 2, complete sequence; and 25S ribosomal RNA gene, partial sequence	224	332	61%	1,00E-54	89% DQ314162.1	0,00	0,0%
Select seq gb|DQ314166.1|	Larnax sachapapa voucher Smith 205 (WIS) internal transcribed spacer 1, partial sequence; 5.8S ribosomal RNA gene and internal transcribed spacer 2, complete sequence; and 25S ribosomal RNA gene, partial sequence	220	329	60%	1,00E-53	89% DQ314166.1	0,00	0,0%
Select seq gb|KC591674.1|	Pogogyne douglasii voucher SDSU19285 internal transcribed spacer 1, partial sequence; 5.8S ribosomal RNA gene and internal transcribed spacer 2, complete sequence; and 28S ribosomal RNA gene, partial sequence	220	329	61%	1,00E-53	88% KC591674.1	0,00	0,0%

Select for downloading or viewing reports	Kh018_trnL Description	Max score	Total score	Query cover	E value	Ident	Accession	(Ident/Cover)* Max score	Deviation from top hit
Select seq gb|EU827136.1|	Paraphlomis hispida voucher Fang091066 (KUN) tRNA-Leu (trnL) gene, partial sequence; trnL-trnF intergenic spacer, complete sequence; and tRNA-Phe (trnF) gene, partial sequence; chloroplast	747	969	74%	0.0	98%	EU827136.1	989,27	100,0%
Select seq gb|EU827132.1|	Paraphlomis hirsutissima voucher Fang091048 (KUN) tRNA-Leu (trnL) gene, partial sequence; trnL-trnF intergenic spacer, complete sequence; and tRNA-Phe (trnF) gene, partial sequence; chloroplast	747	969	74%	0.0	98%	EU827132.1	989,27	100,0%
Select seq gb|EU827131.1|	Paraphlomisjavanica voucher Fang091058 (KUN) tRNA-Leu (trnL) gene, partial sequence; trnL-trnF intergenic spacer, complete sequence; and tRNA-Phe (trnF) gene, partial sequence; chloroplast	747	969	74%	0.0	98%	EU827131.1	989,27	100,0%
Select seq gb|EU827119.1|	Phlomis betonicoides voucher Fang091045 (KUN) tRNA-Leu (trnL) gene, partial sequence; trnL-trnF intergenic spacer, complete sequence; and tRNA-Phe (trnF) gene, partial sequence; chloroplast	747	953	74%	0.0	98%	EU827119.1	989,27	100,0%
Select seq gb|EU827137.1|	Phlomis tuberosa voucher Fang091052 (KUN) tRNA-Leu (trnL) gene, partial sequence; trnL-trnF intergenic spacer, complete sequence; and tRNA-Phe (trnF) gene, partial sequence; chloroplast	743	953	74%	0.0	98%	EU827137.1	983,97	99,5%
Select seq gb|EU827129.1|	Phlomis alpina voucher Fang091051 (KUN) tRNA-Leu (trnL) gene, partial sequence; trnL-trnF intergenic spacer, complete sequence; and tRNA-Phe (trnF) gene, partial sequence; chloroplast	732	942	74%	0.0	98%	EU827129.1	969,41	98,0%
Select seq gb|KM408859.1|	Eriophyton wallichii haplotype H15 tRNA-Leu (trnL) gene, partial sequence; trnL-trnF intergenic spacer, complete sequence; and tRNA-Phe (trnF) gene, partial sequence; chloroplast	736	962	75%	0.0	98%	KM408859.1	961,71	97,2%
Select seq gb|KM408853.1|	Eriophyton wallichii haplotype H9 tRNA-Leu (trnL) gene, partial sequence; trnL-trnF intergenic spacer, complete sequence; and tRNA-Phe (trnF) gene, partial sequence; chloroplast	736	962	75%	0.0	98%	KM408853.1	961,71	97,2%
Select seq gb|KM408852.1|	Eriophyton wallichii haplotype H8 tRNA-Leu (trnL) gene, partial sequence; trnL-trnF intergenic spacer, complete sequence; and tRNA-Phe (trnF) gene, partial sequence; chloroplast	736	962	75%	0.0	98%	KM408852.1	961,71	97,2%
Select seq gb|KM408851.1|	Eriophyton wallichii haplotype H7 tRNA-Leu (trnL) gene, partial sequence; trnL-trnF intergenic spacer, complete sequence; and tRNA-Phe (trnF) gene, partial sequence; chloroplast	736	962	75%	0.0	98%	KM408851.1	961,71	97,2%
Select seq gb|KM408850.1|	Eriophyton wallichii haplotype H6 tRNA-Leu (trnL) gene, partial sequence; trnL-trnF intergenic spacer, complete sequence; and tRNA-Phe (trnF) gene, partial sequence; chloroplast	736	964	75%	0.0	98%	KM408850.1	961,71	97,2%
Select seq gb|KM408846.1|	Eriophyton wallichii haplotype H2 tRNA-Leu (trnL) gene, partial sequence; trnL-trnF intergenic spacer, complete sequence; and tRNA-Phe (trnF) gene, partial sequence; chloroplast	736	962	75%	0.0	98%	KM408846.1	961,71	97,2%
Select seq gb|EU827122.1|	Paraphlomis membranacea voucher Fang091057 (KUN) tRNA-Leu (trnL) gene, partial sequence; trnL-trnF intergenic spacer, complete sequence; and tRNA-Phe (trnF) gene, partial sequence; chloroplast	730	921	74%	0.0	97%	EU827122.1	956,89	96,7%
Select seq gb|EU827138.1|	Galeopsis bifida voucher Fang091077 (KUN) tRNA-Leu (trnL) gene, partial sequence; trnL-trnF intergenic spacer, complete sequence; and tRNA-Phe (trnF) gene, partial sequence; chloroplast	725	936	74%	0.0	97%	EU827138.1	950,34	96,1%
Select seq gb|KM408863.1|	Eriophyton wallichii haplotype H19 tRNA-Leu (trnL) gene, partial sequence; trnL-trnF intergenic spacer, complete sequence; and tRNA-Phe (trnF) gene, partial sequence; chloroplast	730	956	75%	0.0	97%	KM408863.1	944,13	95,4%
Select seq gb|KM408856.1|	Eriophyton wallichii haplotype H12 tRNA-Leu (trnL) gene, partial sequence; trnL-trnF intergenic spacer, complete sequence; and tRNA-Phe (trnF) gene, partial sequence; chloroplast	730	956	75%	0.0	97%	KM408856.1	944,13	95,4%
Select seq gb|KM408849.1|	Eriophyton wallichii haplotype H5 tRNA-Leu (trnL) gene, partial sequence; trnL-trnF intergenic spacer, complete sequence; and tRNA-Phe (trnF) gene, partial sequence; chloroplast	730	956	75%	0.0	97%	KM408849.1	944,13	95,4%
Select seq gb|KM408848.1|	Eriophyton wallichii haplotype H4 tRNA-Leu (trnL) gene, partial sequence; trnL-trnF intergenic spacer, complete sequence; and tRNA-Phe (trnF) gene, partial sequence; chloroplast	730	938	75%	0.0	97%	KM408848.1	944,13	95,4%
Select seq gb|KM408847.1|	Eriophyton wallichii haplotype H3 tRNA-Leu (trnL) gene, partial sequence; trnL-trnF intergenic spacer, complete sequence; and tRNA-Phe (trnF) gene, partial sequence; chloroplast	730	932	75%	0.0	97%	KM408847.1	944,13	95,4%
Select seq gb|KR608675.1|	Gomphostemma sp. GY-2016 tRNA-Leu (trnL) gene, partial sequence; trnL-trnF intergenic spacer, complete sequence; and tRNA-Phe (trnF) gene, partial sequence; chloroplast	741	982	77%	0.0	98%	KR608675.1	943,09	95,3%
Select seq gb|KR608674.1|	Gomphostemma sp. GY-2016 tRNA-Leu (trnL) gene, partial sequence; trnL-trnF intergenic spacer, complete sequence; and tRNA-Phe (trnF) gene, partial sequence; chloroplast	741	982	77%	0.0	98%	KR608674.1	943,09	95,3%
Select seq gb|KM408858.1|	Eriophyton wallichii haplotype H14 tRNA-Leu (trnL) gene, partial sequence; trnL-trnF intergenic spacer, complete sequence; and tRNA-Phe (trnF) gene, partial sequence; chloroplast	725	951	75%	0.0	97%	KM408858.1	937,67	94,8%
Select seq gb|KM408845.1|	Eriophyton wallichii haplotype H1 tRNA-Leu (trnL) gene, partial sequence; trnL-trnF intergenic spacer, complete sequence; and tRNA-Phe (trnF) gene, partial sequence; chloroplast	725	951	75%	0.0	97%	KM408845.1	937,67	94,8%
Select seq gb|KM408857.1|	Eriophyton wallichii haplotype H13 tRNA-Leu (trnL) gene, partial sequence; trnL-trnF intergenic spacer, complete sequence; and tRNA-Phe (trnF) gene, partial sequence; chloroplast	721	947	75%	0.0	97%	KM408857.1	932,49	94,3%
Select seq gb|KM408861.1|	Eriophyton wallichii haplotype H17 tRNA-Leu (trnL) gene, partial sequence; trnL-trnF intergenic spacer, complete sequence; and tRNA-Phe (trnF) gene, partial sequence; chloroplast	719	940	75%	0.0	97%	KM408861.1	929,91	94,0%
Select seq gb|KM408855.1|	Eriophyton wallichii haplotype H11 tRNA-Leu (trnL) gene, partial sequence; trnL-trnF intergenic spacer, complete sequence; and tRNA-Phe (trnF) gene, partial sequence; chloroplast	719	945	75%	0.0	97%	KM408855.1	929,91	94,0%

Select seq gb|EU827139.1|	Phlomis ambigua voucher Fang091055 (KUN) tRNA-Leu (trnL) gene, partial sequence; trnL-trnF intergenic spacer, complete sequence; and tRNA-Phe (trnF) gene, partial sequence; chloroplast	706	923	74% 0.0	97% EU827139.1	925,43	93,5%
Select seq gb|EU827135.1|	Phlomis melanantha voucher Fang091040 (KUN) tRNA-Leu (trnL) gene, partial sequence; trnL-trnF intergenic spacer, complete sequence; and tRNA-Phe (trnF) gene, partial sequence; chloroplast	706	923	74% 0.0	97% EU827135.1	925,43	93,5%
Select seq gb|EU827126.1|	Phlomis ornata voucher Fang091027 (KUN) tRNA-Leu (trnL) gene, partial sequence; trnL-trnF intergenic spacer, complete sequence; and tRNA-Phe (trnF) gene, partial sequence; chloroplast	706	918	74% 0.0	97% EU827126.1	925,43	93,5%
Select seq gb|EU827127.1|	Phlomis megalantha voucher Fang091029 (KUN) tRNA-Leu (trnL) gene, partial sequence; trnL-trnF intergenic spacer, complete sequence; and tRNA-Phe (trnF) gene, partial sequence; chloroplast	701	918	74% 0.0	97% EU827127.1	918,88	92,9%
Select seq gb|JQ041839.1|	Galeopsis tetrahit voucher GTE1 tRNA-Leu (trnL) gene, complete sequence; and trnL-trnF intergenic spacer, partial sequence; plastid	697	899	73% 0.0	96% JQ041839.1	916,60	92,7%
Select seq gb|KM408862.1|	Eriophyton wallichii haplotype H18 tRNA-Leu (trnL) gene, partial sequence; trnL-trnF intergenic spacer, complete sequence; and tRNA-Phe (trnF) gene, partial sequence; chloroplast	704	921	75% 0.0	97% KM408862.1	910,51	92,0%
Select seq gb|KM408860.1|	Eriophyton wallichii haplotype H16 tRNA-Leu (trnL) gene, partial sequence; trnL-trnF intergenic spacer, complete sequence; and tRNA-Phe (trnF) gene, partial sequence; chloroplast	704	931	75% 0.0	97% KM408860.1	910,51	92,0%
Select seq gb|JF780025.1|	Lamium orvala voucher N. Lundqvist 7702 (UPS) tRNA-Leu (trnL) gene, partial sequence; trnL-trnF intergenic spacer, complete sequence; and tRNA-Phe (trnF) gene, partial sequence; plastid	686	888	73% 0.0	96% JF780025.1	902,14	91,2%
Select seq gb|EU827130.1|	Phlomis umbrosa voucher Fang091030 (KUN) tRNA-Leu (trnL) gene, partial sequence; trnL-trnF intergenic spacer, complete sequence; and tRNA-Phe (trnF) gene, partial sequence; chloroplast	695	912	74% 0.0	96% EU827130.1	901,62	91,1%
Select seq gb|EU827123.1|	Phlomis tatsiensis voucher Fang091028 (KUN) tRNA-Leu (trnL) gene, partial sequence; trnL-trnF intergenic spacer, complete sequence; and tRNA-Phe (trnF) gene, partial sequence; chloroplast	695	912	74% 0.0	96% EU827123.1	901,62	91,1%
Select seq gb|EU827118.1|	Phlomis atropurpurea voucher Fang091053 (KUN) tRNA-Leu (trnL) gene, partial sequence; trnL-trnF intergenic spacer, complete sequence; and tRNA-Phe (trnF) gene, partial sequence; chloroplast	695	912	74% 0.0	96% EU827118.1	901,62	91,1%
Select seq gb|KR608664.1|	Craniotome furcata tRNA-Leu (trnL) gene, partial sequence; trnL-trnF intergenic spacer, complete sequence; and tRNA-Phe (trnF) gene, partial sequence; chloroplast	702	897	75% 0.0	96% KR608664.1	898,56	90,8%
Select seq gb|KR608673.1|	Colquhounia coccinea tRNA-Leu (trnL) gene, partial sequence; trnL-trnF intergenic spacer, complete sequence; and tRNA-Phe (trnF) gene, partial sequence; chloroplast	713	958	77% 0.0	97% KR608673.1	898,19	90,8%
Select seq gb|EU827120.1|	Phlomis fruticosa voucher Fang091026 (KUN) tRNA-Leu (trnL) gene, partial sequence; trnL-trnF intergenic spacer, complete sequence; and tRNA-Phe (trnF) gene, partial sequence; chloroplast	691	901	74% 0.0	96% EU827120.1	896,43	90,6%
Select seq gb|KR608663.1|	Microtoena sp. GY-2016 tRNA-Leu (trnL) gene, partial sequence; trnL-trnF intergenic spacer, complete sequence; and tRNA-Phe (trnF) gene, partial sequence; chloroplast	699	903	75% 0.0	96% KR608663.1	894,72	90,4%
Select seq gb|KM408854.1|	Eriophyton wallichii haplotype H10 tRNA-Leu (trnL) gene, partial sequence; trnL-trnF intergenic spacer, complete sequence; and tRNA-Phe (trnF) gene, partial sequence; chloroplast	699	925	75% 0.0	96% KM408854.1	894,72	90,4%
Select seq gb|EU827125.1|	Phlomis strigosa voucher Fang091046 (KUN) tRNA-Leu (trnL) gene, partial sequence; trnL-trnF intergenic spacer, complete sequence; and tRNA-Phe (trnF) gene, partial sequence; chloroplast	689	907	74% 0.0	96% EU827125.1	893,84	90,4%
Select seq gb|EU827134.1|	Phlomis purpurea voucher Fang091025 (KUN) tRNA-Leu (trnL) gene, partial sequence; trnL-trnF intergenic spacer, complete sequence; and tRNA-Phe (trnF) gene, partial sequence; chloroplast	688	892	74% 0.0	96% EU827134.1	892,54	90,2%
Select seq gb|EU827133.1|	Phlomis flocossa voucher Fang091024 (KUN) tRNA-Leu (trnL) gene, partial sequence; trnL-trnF intergenic spacer, complete sequence; and tRNA-Phe (trnF) gene, partial sequence; chloroplast	688	892	74% 0.0	96% EU827133.1	892,54	90,2%
Select seq gb|EU827121.1|	Lamiophlomis rotata voucher Fang091011 (KUN) tRNA-Leu (trnL) gene, partial sequence; trnL-trnF intergenic spacer, complete sequence; and tRNA-Phe (trnF) gene, partial sequence; chloroplast	684	901	74% 0.0	96% EU827121.1	887,35	89,7%
Select seq gb|KR608665.1|	Craniotome furcata tRNA-Leu (trnL) gene, partial sequence; trnL-trnF intergenic spacer, complete sequence; and tRNA-Phe (trnF) gene, partial sequence; chloroplast	702	903	76% 0.0	96% KR608665.1	886,74	89,6%
Select seq gb|JF779995.1|	Lamium galeobdolon subsp. galeobdolon voucher N. Orderud 236911 (O) tRNA-Leu (trnL) gene, partial sequence; trnL-trnF intergenic spacer, complete sequence; and tRNA-Phe (trnF) gene, partial sequence; plastid	686	918	75% 0.0	96% JF779995.1	878,08	88,8%
Select seq gb|JF779990.1|	Lamium galeobdolon subsp. argentatum voucher H. Nielsen s.n., 22.7.1989 (C) tRNA-Leu (trnL) gene, partial sequence; trnL-trnF intergenic spacer, complete sequence; and tRNA-Phe (trnF) gene, partial sequence; plastid	686	918	75% 0.0	96% JF779990.1	878,08	88,8%
Select seq gb|KR608662.1|	Microtoena sp. GY-2016 tRNA-Leu (trnL) gene, partial sequence; trnL-trnF intergenic spacer, complete sequence; and tRNA-Phe (trnF) gene, partial sequence; chloroplast	702	932	77% 0.0	96% KR608662.1	875,22	88,5%
Select seq gb|JF779996.1|	Lamium galeobdolon subsp. montanum voucher M. Bendiksby 05-015 (O) tRNA-Leu (trnL) gene, partial sequence; trnL-trnF intergenic spacer, complete sequence; and tRNA-Phe (trnF) gene, partial sequence; plastid	686	934	76% 0.0	96% JF779996.1	866,53	87,6%
Select seq gb|JF779994.1|	Lamium galeobdolon subsp. galeobdolon voucher M. Bendiksby 05-016 (O) tRNA-Leu (trnL) gene, partial sequence; trnL-trnF intergenic spacer, complete sequence; and tRNA-Phe (trnF) gene, partial sequence; plastid	686	934	76% 0.0	96% JF779994.1	866,53	87,6%
Select seq gb|KR608670.1|	Eurysolen gracilis tRNA-Leu (trnL) gene, partial sequence; trnL-trnF intergenic spacer, complete sequence; and tRNA-Phe (trnF) gene, partial sequence; chloroplast	693	916	77% 0.0	96% KR608670.1	864,00	87,3%
Select seq gb|KR608669.1|	Eurysolen gracilis tRNA-Leu (trnL) gene, partial sequence; trnL-trnF intergenic spacer, complete sequence; and tRNA-Phe (trnF) gene, partial sequence; chloroplast	693	916	77% 0.0	96% KR608669.1	864,00	87,3%

Select seq gb|KC890943.1|	Lagochilus ilicifolius voucher LE SEQ012010005; chloroplast	702	949	78%	0.0	96%	KC890943.1	864,00	87,3%
Select seq gb|KC890941.1|	Lagochilus ilicifolius voucher LE SEQ012010003; chloroplast	702	944	78%	0.0	96%	KC890941.1	864,00	87,3%
Select seq gb|KC890937.1|	Leonurus glaucescens voucher XJBI:200907121; chloroplast	697	947	78%	0.0	96%	KC890937.1	857,85	86,7%
Select seq gb|JF779992.1|	Lamium galeobdolon subsp. flavidum voucher G. & E. Golles 365 (WU) tRNA-Leu (trnL) gene, partial sequence; trnL-trnF intergenic spacer, complete sequence; and tRNA-Phe (trnF) gene, partial sequence; plastid	686	956	77%	0.0	96%	JF779992.1	855,27	86,5%
Select seq gb|KC890938.1|	Panzerina lanata var. alaschanica voucher XJBI:200907011; chloroplast	691	964	78%	0.0	96%	KC890938.1	850,46	86,0%
Select seq gb|KC890936.1|	Leonurus turkestanicus voucher XJBI:200907041 tRNA-Leu (trnL) gene, partial sequence; trnL-trnF intergenic spacer, complete sequence; and tRNA-Phe (trnF) gene, partial sequence; chloroplast	691	944	78%	0.0	96%	KC890936.1	850,46	86,0%
Select seq gb|KC890944.1|	Lagochilus ilicifolius voucher LE SEQ012010006; chloroplast	697	955	79%	0.0	96%	KC890944.1	846,99	85,6%
Select seq gb|KC890950.1|	Lagochilus ilicifolius voucher XJBI:200907094; chloroplast	691	958	79%	0.0	96%	KC890950.1	839,70	84,9%
Select seq gb|KC890945.1|	Lagochilus ilicifolius voucher LE SEQ012010007; chloroplast	691	962	79%	0.0	96%	KC890945.1	839,70	84,9%
Select seq gb|JF779987.1|	Lamium flexuosum voucher I. Segelberg s.n., 13.5.1962 (S) tRNA-Leu (trnL) gene, partial sequence; trnL-trnF intergenic spacer, complete sequence; and tRNA-Phe (trnF) gene, partial sequence; plastid	686	979	79%	0.0	96%	JF779987.1	833,62	84,3%
Select seq gb|JF779986.1|	Lamium flexuosum voucher H. Lindberg 3722 (S) tRNA-Leu (trnL) gene, partial sequence; trnL-trnF intergenic spacer, complete sequence; and tRNA-Phe (trnF) gene, partial sequence; plastid	686	977	79%	0.0	96%	JF779986.1	833,62	84,3%
Select seq gb|JF779985.1|	Lamium flexuosum voucher Grobner 7 (C) tRNA-Leu (trnL) gene, partial sequence; trnL-trnF intergenic spacer, complete sequence; and tRNA-Phe (trnF) gene, partial sequence; plastid	686	979	79%	0.0	96%	JF779985.1	833,62	84,3%
Select seq gb|KP338099.1|	Stachys persepolitana tRNA-Leu (trnL) gene, partial sequence; trnL-trnF intergenic spacer, complete sequence; and tRNA- Phe (trnF) gene, partial sequence; chloroplast	675	936	77%	0.0	95%	KP338099.1	832,79	84,2%
Select seq gb|JF780008.1|	Lamium purpureum var. hybridum voucher K.A. Lye 23936 (O) tRNA-Leu (trnL) gene, partial sequence; trnL-trnF intergenic spacer, complete sequence; and tRNA-Phe (trnF) gene, partial sequence; plastid	675	945	77%	0.0	95%	JF780008.1	832,79	84,2%
Select seq gb|KC890956.1|	Lagochilus ilicifolius voucher XJBI:200908112; chloroplast	686	958	79%	0.0	95%	KC890956.1	824,94	83,4%
Select seq gb|KC890954.1|	Lagochilus ilicifolius voucher XJBI:200908030; chloroplast	686	964	79%	0.0	95%	KC890954.1	824,94	83,4%
Select seq gb|KC890948.1|	Lagochilus ilicifolius voucher XJBI:200908047; chloroplast	686	958	79%	0.0	95%	KC890948.1	824,94	83,4%
Select seq gb|KC890946.1|	Lagochilus ilicifolius voucher XJBI:200907056; chloroplast	686	958	79%	0.0	95%	KC890946.1	824,94	83,4%
Select seq gb|JF779977.1|	Lamium bifidum voucher M. Bendiksby 05-021 (O) tRNA-Leu (trnL) gene, partial sequence; trnL-trnF intergenic spacer, complete sequence; and tRNA-Phe (trnF) gene, partial sequence; plastid	675	953	78%	0.0	95%	JF779977.1	822,12	83,1%
Select seq gb|KC890959.1|	Lagochilus ilicifolius voucher XJBI:200908107; chloroplast	680	953	79%	0.0	95%	KC890959.1	817,72	82,7%
Select seq gb|KC890957.1|	Lagochilus ilicifolius voucher XJBI:200908051; chloroplast	680	947	79%	0.0	95%	KC890957.1	817,72	82,7%
Select seq gb|KC890955.1|	Lagochilus ilicifolius voucher XJBI:200907024; chloroplast	680	958	79%	0.0	95%	KC890955.1	817,72	82,7%
Select seq gb|KC890953.1|	Lagochilus ilicifolius voucher XJBI:200907091; chloroplast	680	944	79%	0.0	95%	KC890953.1	817,72	82,7%
Select seq gb|KC890952.1|	Lagochilus ilicifolius voucher XJBI:200907220; chloroplast	680	953	79%	0.0	95%	KC890952.1	817,72	82,7%
Select seq gb|KC890951.1|	Lagochilus ilicifolius voucher XJBI:200908021; chloroplast	680	942	79%	0.0	95%	KC890951.1	817,72	82,7%
Select seq gb|KC890949.1|	Lagochilus ilicifolius voucher XJBI:200908001; chloroplast	680	932	79%	0.0	95%	KC890949.1	817,72	82,7%
Select seq gb|KC890947.1|	Lagochilus ilicifolius voucher XJBI:200907014; chloroplast	680	958	79%	0.0	95%	KC890947.1	817,72	82,7%
Select seq gb|KC890958.1|	Lagochilus ilicifolius voucher XJBI:200908061; chloroplast	675	918	79%	0.0	95%	KC890958.1	811,71	82,1%

Select seq gb|JF780006.1|	Lamium purpureum var. hybridum voucher J.E. Eriksen s.n., 2.7.2000 (O) tRNA-Leu (trnL) gene, partial sequence; trnL-trnF intergenic spacer, complete sequence; and tRNA-Phe (trnF) gene, partial sequence; plastid	675	968	79%	0.0	95% JF780006.1	811,71	82,1%
Select seq gb|JF779976.1|	Lamium bifidum voucher A. Latzel s.n., 29.3.1909 (UPS) tRNA-Leu (trnL) gene, partial sequence; trnL-trnF intergenic spacer, complete sequence; and tRNA-Phe (trnF) gene, partial sequence; plastid	675	968	79%	0.0	95% JF779976.1	811,71	82,1%
Select seq gb|AF502053.1|	Stachys lavandulifolia tRNA-Leu (trnL) gene, partial sequence; chloroplast gene for chloroplast product	693	693	49%	0.0	99% AF502053.1	0,00	0,0%
Select seq gb|AF502044.1|	Stachys argillicola tRNA-Leu (trnL) gene, partial sequence; chloroplast gene for chloroplast product	682	682	49%	0.0	99% AF502044.1	0,00	0,0%
Select seq gb|AF502035.1|	Prasium majus tRNA-Leu (trnL) gene, partial sequence; chloroplast gene for chloroplast product	682	682	49%	0.0	99% AF502035.1	0,00	0,0%
Select seq gb|AF502062.1|	Stachys swainsonii tRNA-Leu (trnL) gene, partial sequence; chloroplast gene for chloroplast product	676	676	49%	0.0	98% AF502062.1	0,00	0,0%
Select seq gb|AF502046.1|	Stachys byzantina tRNA-Leu (trnL) gene, partial sequence; chloroplast gene for chloroplast product	676	676	49%	0.0	98% AF502046.1	0,00	0,0%
Select seq gb|AF502038.1|	Sideritis macrostachys tRNA-Leu (trnL) gene, partial sequence; chloroplast gene for chloroplast product	676	676	49%	0.0	98% AF502038.1	0,00	0,0%
Select seq gb|AF502037.1|	Sideritis hyssopifolia tRNA-Leu (trnL) gene, partial sequence; chloroplast gene for chloroplast product	676	676	49%	0.0	98% AF502037.1	0,00	0,0%
Select seq gb|AF502036.1|	Sideritis gomeraea tRNA-Leu (trnL) gene, partial sequence; chloroplast gene for chloroplast product	676	676	49%	0.0	98% AF502036.1	0,00	0,0%
Select seq gb|AF335647.1|	Sideritis gomeraea subsp. gomeraea voucher JB 256 tRNA-Leu (trnL) gene, partial sequence; chloroplast	676	676	49%	0.0	98% AF335647.1	0,00	0,0%
Select seq gb|AF502064.1|	Stachys tymphaea tRNA-Leu (trnL) gene, partial sequence; chloroplast gene for chloroplast product	671	671	49%	0.0	98% AF502064.1	0,00	0,0%
Select seq gb|AF502060.1|	Stachys radicans tRNA-Leu (trnL) gene, partial sequence; chloroplast gene for chloroplast product	671	671	49%	0.0	98% AF502060.1	0,00	0,0%
Select seq gb|KC890939.1|	Lagochilus ilicifolius voucher LE SEQ012010001; chloroplast	719	719	55%	0.0	97% KC890939.1	0,00	0,0%
Select seq gb|KM886612.1|	Eriophyton wallichii voucher SNJ Exped. 20110814032 (KUN) tRNA-Leu (trnL) gene, partial sequence; trnL-trnF intergenic spacer, complete sequence; and tRNA-Phe (trnF) gene, partial sequence; plastid	702	702	55%	0.0	97% KM886612.1	0,00	0,0%
Select seq gb|KC890940.1|	Lagochilus ilicifolius voucher LE SEQ012010002; chloroplast	691	691	55%	0.0	96% KC890940.1	0,00	0,0%
Select seq gb|KC890942.1|	Lagochilus ilicifolius voucher LE SEQ012010004; chloroplast	686	686	55%	0.0	95% KC890942.1	0,00	0,0%

Select for downloading or viewing reports	Kh019_trnL Description	Max score	Total score	Query cover	E value	Ident	Accession	(Ident/Cover)* Max score	Deviation from top hit
Select seq gb|JN408660.1|	Teucrium pyrenaicum tRNA-Leu (trnL) gene, partial sequence; and trnL-trnF intergenic spacer, complete sequence; chloroplast	1170	1170	96%	0.0	99%	JN408660.1	1206,56	100,0%
Select seq gb|JN408625.1|	Teucrium capitatum tRNA-Leu (trnL) gene, partial sequence; and trnL-trnF intergenic spacer, complete sequence; chloroplast	1133	1133	93%	0.0	99%	JN408625.1	1206,10	100,0%
Select seq gb|JN408624.1|	Teucrium capitatum tRNA-Leu (trnL) gene, partial sequence; and trnL-trnF intergenic spacer, complete sequence; chloroplast	1133	1133	93%	0.0	99%	JN408624.1	1206,10	100,0%
Select seq gb|JN408623.1|	Teucrium aureum tRNA-Leu (trnL) gene, partial sequence; and trnL-trnF intergenic spacer, complete sequence; chloroplast	1133	1133	93%	0.0	99%	JN408623.1	1206,10	100,0%
Select seq gb|JN408621.1|	Teucrium pumilum tRNA-Leu (trnL) gene, partial sequence; and trnL-trnF intergenic spacer, complete sequence; chloroplast	1133	1133	93%	0.0	99%	JN408621.1	1206,10	100,0%
Select seq gb|JQ044780.1|	Teucrium polium subsp. polium isolate TP12 tRNA-Leu (trnL) gene, partial sequence; trnL-trnF intergenic spacer, complete sequence; and tRNA-Phe (trnF) gene, partial sequence; chloroplast	1205	1205	99%	0.0	99%	JQ044780.1	1205,00	99,9%
Select seq gb|JQ044779.1|	Teucrium polium subsp. polium isolate TP11 tRNA-Leu (trnL) gene, partial sequence; trnL-trnF intergenic spacer, complete sequence; and tRNA-Phe (trnF) gene, partial sequence; chloroplast	1205	1205	99%	0.0	99%	JQ044779.1	1205,00	99,9%
Select seq gb|JQ044778.1|	Teucrium polium subsp. polium isolate TP10 tRNA-Leu (trnL) gene, partial sequence; trnL-trnF intergenic spacer, complete sequence; and tRNA-Phe (trnF) gene, partial sequence; chloroplast	1205	1205	99%	0.0	99%	JQ044778.1	1205,00	99,9%
Select seq gb|JN408657.1|	Teucrium polium tRNA-Leu (trnL) gene, partial sequence; and trnL-trnF intergenic spacer, complete sequence; chloroplast	1131	1131	93%	0.0	99%	JN408657.1	1203,97	99,8%
Select seq gb|JN408646.1|	Teucrium montanum tRNA-Leu (trnL) gene, partial sequence; and trnL-trnF intergenic spacer, complete sequence; chloroplast	1131	1131	93%	0.0	99%	JN408646.1	1203,97	99,8%
Select seq gb|JN408620.1|	Teucrium aureum tRNA-Leu (trnL) gene, partial sequence; and trnL-trnF intergenic spacer, complete sequence; chloroplast	1179	1179	97%	0.0	99%	JN408620.1	1203,31	99,7%
Select seq gb|JN408619.1|	Teucrium capitatum tRNA-Leu (trnL) gene, partial sequence; and trnL-trnF intergenic spacer, complete sequence; chloroplast	1177	1177	97%	0.0	99%	JN408619.1	1201,27	99,6%
Select seq gb|JN408597.1|	Teucrium alpestre tRNA-Leu (trnL) gene, partial sequence; trnL-trnF intergenic spacer, complete sequence; and tRNA-Phe (trnF) gene, partial sequence; chloroplast	1212	1212	100%	0.0	99%	JN408597.1	1199,88	99,4%
Select seq gb|FJ952080.1|	Teucrium pyrenaicum tRNA-Leu (trnL) gene, partial sequence; trnL-trnF intergenic spacer, complete sequence; and tRNA-Phe (trnF) gene, partial sequence; chloroplast	1212	1212	100%	0.0	99%	FJ952080.1	1199,88	99,4%
Select seq gb|JN408616.1|	Teucrium cyprium subsp. cyprium tRNA-Leu (trnL) gene, partial sequence; and trnL-trnF intergenic spacer, complete sequence; chloroplast	1175	1175	97%	0.0	99%	JN408616.1	1199,23	99,4%
Select seq gb|JQ044781.1|	Teucrium polium subsp. polium isolate TP19 tRNA-Leu (trnL) gene, partial sequence; trnL-trnF intergenic spacer, complete sequence; and tRNA-Phe (trnF) gene, partial sequence; chloroplast	1199	1199	99%	0.0	99%	JQ044781.1	1199,00	99,4%
Select seq gb|JQ044774.1|	Teucrium polium subsp. polium isolate TP20 tRNA-Leu (trnL) gene, partial sequence; trnL-trnF intergenic spacer, complete sequence; and tRNA-Phe (trnF) gene, partial sequence; chloroplast	1199	1199	99%	0.0	99%	JQ044774.1	1199,00	99,4%
Select seq gb|JQ044782.1|	Teucrium polium subsp. polium isolate TP13 tRNA-Leu (trnL) gene, partial sequence; trnL-trnF intergenic spacer, complete sequence; and tRNA-Phe (trnF) gene, partial sequence; chloroplast	1194	1194	99%	0.0	98%	JQ044782.1	1181,94	98,0%
Select seq gb|JQ044777.1|	Teucrium polium subsp. polium isolate TP5 tRNA-Leu (trnL) gene, partial sequence; trnL-trnF intergenic spacer, complete sequence; and tRNA-Phe (trnF) gene, partial sequence; chloroplast	1194	1194	99%	0.0	98%	JQ044777.1	1181,94	98,0%
Select seq gb|JQ044776.1|	Teucrium polium subsp. polium isolate TP4 tRNA-Leu (trnL) gene, partial sequence; trnL-trnF intergenic spacer, complete sequence; and tRNA-Phe (trnF) gene, partial sequence; chloroplast	1194	1194	99%	0.0	98%	JQ044776.1	1181,94	98,0%
Select seq gb|JQ044775.1|	Teucrium polium subsp. polium isolate TP3 tRNA-Leu (trnL) gene, partial sequence; trnL-trnF intergenic spacer, complete sequence; and tRNA-Phe (trnF) gene, partial sequence; chloroplast	1194	1194	99%	0.0	98%	JQ044775.1	1181,94	98,0%
Select seq gb|JQ044771.1|	Teucrium polium subsp. capitatum isolate TPC2 tRNA-Leu (trnL) gene, partial sequence; trnL-trnF intergenic spacer, complete sequence; and tRNA-Phe (trnF) gene, partial sequence; chloroplast	1181	1181	99%	0.0	98%	JQ044771.1	1169,07	96,9%
Select seq gb|JQ044773.1|	Teucrium polium subsp. capitatum isolate TPC1 tRNA-Leu (trnL) gene, partial sequence; trnL-trnF intergenic spacer, complete sequence; and tRNA-Phe (trnF) gene, partial sequence; chloroplast	1175	1175	99%	0.0	98%	JQ044773.1	1163,13	96,4%
Select seq gb|JQ044772.1|	Teucrium polium subsp. capitatum isolate TPC4 tRNA-Leu (trnL) gene, partial sequence; trnL-trnF intergenic spacer, complete sequence; and tRNA-Phe (trnF) gene, partial sequence; chloroplast	1175	1175	99%	0.0	98%	JQ044772.1	1163,13	96,4%
Select seq gb|JN408632.1|	Teucrium heterophyllum tRNA-Leu (trnL) gene, partial sequence; and trnL-trnF intergenic spacer, complete sequence; chloroplast	1140	1140	97%	0.0	97%	JN408632.1	1140,00	94,5%
Select seq gb|JN408594.1|	Teucrium antitauricum tRNA-Leu (trnL) gene, partial sequence; and trnL-trnF intergenic spacer, complete sequence; chloroplast	1120	1120	97%	0.0	97%	JN408594.1	1120,00	92,8%

Select seq gb|JN408631.1|	Teucrium halacsyanum tRNA-Leu (trnL) gene, partial sequence; and trnL-trnF intergenic spacer, complete sequence; chloroplast	1079	1079	94% 0.0	97%	JN408631.1	1113,44	92,3%
Select seq gb|JN408647.1|	Teucrium montbretii subsp. heliotropiifolium tRNA-Leu (trnL) gene, partial sequence; and trnL-trnF intergenic spacer, complete sequence; chloroplast	1048	1048	92% 0.0	97%	JN408647.1	1104,96	91,6%
Select seq gb|JN408676.1|	Teucrium viscidum var. miquelianum tRNA-Leu (trnL) gene, partial sequence; and trnL-trnF intergenic spacer, complete sequence; chloroplast	1070	1070	94% 0.0	97%	JN408676.1	1104,15	91,5%
Select seq gb|JN408672.1|	Teucrium lamiifolium subsp. stachyophyllum tRNA-Leu (trnL) gene, partial sequence; and trnL-trnF intergenic spacer, complete sequence; chloroplast	1101	1101	97% 0.0	97%	JN408672.1	1101,00	91,3%
Select seq gb|JN408618.1|	Teucrium demnatense tRNA-Leu (trnL) gene, partial sequence; and trnL-trnF intergenic spacer, complete sequence; chloroplast	1088	1088	96% 0.0	97%	JN408618.1	1099,33	91,1%
Select seq gb|JN408636.1|	Teucrium japonicum tRNA-Leu (trnL) gene, partial sequence; and trnL-trnF intergenic spacer, complete sequence; chloroplast	1099	1099	97% 0.0	97%	JN408636.1	1099,00	91,1%
Select seq gb|JN408661.1|	Teucrium quadrifarium tRNA-Leu (trnL) gene, partial sequence; and trnL-trnF intergenic spacer, complete sequence; chloroplast	1051	1051	93% 0.0	97%	JN408661.1	1096,20	90,9%
Select seq gb|JN408664.1|	Teucrium royleanum tRNA-Leu (trnL) gene, partial sequence; and trnL-trnF intergenic spacer, complete sequence; chloroplast	1094	1094	97% 0.0	97%	JN408664.1	1094,00	90,7%
Select seq gb|JN408665.1|	Teucrium rupestre tRNA-Leu (trnL) gene, partial sequence; and trnL-trnF intergenic spacer, complete sequence; chloroplast	1037	1037	92% 0.0	97%	JN408665.1	1093,36	90,6%
Select seq gb|JN408628.1|	Teucrium edelbergii tRNA-Leu (trnL) gene, partial sequence; and trnL-trnF intergenic spacer, complete sequence; chloroplast	1079	1079	96% 0.0	97%	JN408628.1	1090,24	90,4%
Select seq gb|JN408649.1|	Teucrium odontites tRNA-Leu (trnL) gene, partial sequence; and trnL-trnF intergenic spacer, complete sequence; chloroplast	1098	1098	97% 0.0	96%	JN408649.1	1086,68	90,1%
Select seq gb|JN408598.1|	Teucrium arduinoi tRNA-Leu (trnL) gene, partial sequence; and trnL-trnF intergenic spacer, complete sequence; chloroplast	1090	1090	97% 0.0	96%	JN408598.1	1078,76	89,4%
Select seq gb|JN408633.1|	Teucrium hircanicum tRNA-Leu (trnL) gene, partial sequence; and trnL-trnF intergenic spacer, complete sequence; chloroplast	1033	1033	92% 0.0	96%	JN408633.1	1077,91	89,3%
Select seq gb|JN408674.1|	Teucridium parvifolium tRNA-Leu (trnL) gene, partial sequence; trnL-trnF intergenic spacer, complete sequence; and tRNA-Phe (trnF) gene, partial sequence; chloroplast	1109	1109	99% 0.0	96%	JN408674.1	1075,39	89,1%
Select seq gb|JN408640.1|	Teucrium lamiifolium subsp. lamiifolium tRNA-Leu (trnL) gene, partial sequence; and trnL-trnF intergenic spacer, complete sequence; chloroplast	1077	1077	97% 0.0	96%	JN408640.1	1065,90	88,3%
Select seq gb|JN408669.1|	Teucrium scorodonia tRNA-Leu (trnL) gene, partial sequence; and trnL-trnF intergenic spacer, complete sequence; chloroplast	1064	1064	96% 0.0	96%	JN408669.1	1064,00	88,2%
Select seq gb|JN408666.1|	Teucrium salviastrum tRNA-Leu (trnL) gene, partial sequence; and trnL-trnF intergenic spacer, complete sequence; chloroplast	1064	1064	96% 0.0	96%	JN408666.1	1064,00	88,2%
Select seq gb|JN408603.1|	Teucrium asiaticum tRNA-Leu (trnL) gene, partial sequence; and trnL-trnF intergenic spacer, complete sequence; chloroplast	1064	1064	96% 0.0	96%	JN408603.1	1064,00	88,2%
Select seq gb|JN408648.1|	Teucrium nudicaule tRNA-Leu (trnL) gene, partial sequence; and trnL-trnF intergenic spacer, complete sequence; chloroplast	1061	1061	96% 0.0	96%	JN408648.1	1061,00	87,9%
Select seq gb|JN408627.1|	Teucrium scorodonia tRNA-Leu (trnL) gene, partial sequence; and trnL-trnF intergenic spacer, complete sequence; chloroplast	1026	1026	93% 0.0	96%	JN408627.1	1059,10	87,8%
Select seq gb|JF694866.1|	Teucrium scorodonia isolate TSC8 tRNA-Leu (trnL) gene and trnL-trnF intergenic spacer, complete sequence; and tRNA-Phe (trnF) gene, partial sequence; plastid	1092	1092	99% 0.0	96%	JF694866.1	1058,91	87,8%
Select seq gb|JF694861.1|	Teucrium scorodonia isolate TSC3 tRNA-Leu (trnL) gene and trnL-trnF intergenic spacer, complete sequence; and tRNA-Phe (trnF) gene, partial sequence; plastid	1092	1092	99% 0.0	96%	JF694861.1	1058,91	87,8%
Select seq gb|JF694859.1|	Teucrium scorodonia isolate TSC1 tRNA-Leu (trnL) gene and trnL-trnF intergenic spacer, complete sequence; and tRNA-Phe (trnF) gene, partial sequence; plastid	1092	1092	99% 0.0	96%	JF694859.1	1058,91	87,8%
Select seq gb|JN408600.1|	Teucrium cubense tRNA-Leu (trnL) gene, partial sequence; trnL-trnF intergenic spacer, complete sequence; and tRNA-Phe (trnF) gene, partial sequence; chloroplast	1103	1103	100% 0.0	96%	JN408600.1	1058,88	87,8%
Select seq gb|JN408607.1|	Teucrium bracteatum tRNA-Leu (trnL) gene, partial sequence; and trnL-trnF intergenic spacer, complete sequence; chloroplast	1068	1068	97% 0.0	96%	JN408607.1	1056,99	87,6%
Select seq gb|JN408595.1|	Teucrium abutiloides tRNA-Leu (trnL) gene, partial sequence; trnL-trnF intergenic spacer, complete sequence; and tRNA-Phe (trnF) gene, partial sequence; chloroplast	1090	1090	99% 0.0	96%	JN408595.1	1056,97	87,6%
Select seq gb|JN408667.1|	Teucrium francoi tRNA-Leu (trnL) gene, partial sequence; and trnL-trnF intergenic spacer, complete sequence; chloroplast	1066	1066	97% 0.0	96%	JN408667.1	1055,01	87,4%

Select seq gb|JN408643.1|	Teucrium massiliense tRNA-Leu (trnL) gene, partial sequence; and trnL-trnF intergenic spacer, complete sequence; chloroplast	1066	1066	97% 0.0	96% JN408643.1	1055,01	87,4%
Select seq gb|JN408610.1|	Teucrium trifidum tRNA-Leu (trnL) gene, partial sequence; and trnL-trnF intergenic spacer, complete sequence; chloroplast	1044	1044	95% 0.0	96% JN408610.1	1054,99	87,4%
Select seq gb|JF694864.1|	Teucrium scorodonia isolate TSC6 tRNA-Leu (trnL) gene and trnL-trnF intergenic spacer, complete sequence; and tRNA-Phe (trnF) gene, partial sequence; plastid	1086	1086	99% 0.0	96% JF694864.1	1053,09	87,3%
Select seq gb|JN408605.1|	Teucrium bicolor tRNA-Leu (trnL) gene, partial sequence; and trnL-trnF intergenic spacer, complete sequence; chloroplast	1064	1064	97% 0.0	96% JN408605.1	1053,03	87,3%
Select seq gb|JN408675.1|	Teucrium trifidum tRNA-Leu (trnL) gene, partial sequence; and trnL-trnF intergenic spacer, complete sequence; chloroplast	1053	1053	96% 0.0	96% JN408675.1	1053,00	87,3%
Select seq gb|JN408591.1|	Spartothamnella puberula tRNA-Leu (trnL) gene, partial sequence; trnL-trnF intergenic spacer, complete sequence; and tRNA-Phe (trnF) gene, partial sequence; chloroplast	1038	1038	95% 0.0	96% JN408591.1	1048,93	86,9%
Select seq gb|JN408639.1|	Teucrium laciniatum tRNA-Leu (trnL) gene, partial sequence; and trnL-trnF intergenic spacer, complete sequence; chloroplast	1024	1024	94% 0.0	96% JN408639.1	1045,79	86,7%
Select seq gb|JN408590.1|	Oncinocalyx betchei tRNA-Leu (trnL) gene, partial sequence; trnL-trnF intergenic spacer, complete sequence; and tRNA-Phe (trnF) gene, partial sequence; chloroplast	1033	1033	95% 0.0	96% JN408590.1	1043,87	86,5%
Select seq gb|JN408658.1|	Teucrium pruinosum tRNA-Leu (trnL) gene, partial sequence; and trnL-trnF intergenic spacer, complete sequence; chloroplast	1048	1048	96% 0.0	95% JN408658.1	1037,08	86,0%
Select seq gb|JN408650.1|	Teucrium oliverianum tRNA-Leu (trnL) gene, partial sequence; and trnL-trnF intergenic spacer, complete sequence; chloroplast	1042	1042	96% 0.0	95% JN408650.1	1031,15	85,5%
Select seq gb|JN408670.1|	Teucrium sessiliflorum tRNA-Leu (trnL) gene, partial sequence; and trnL-trnF intergenic spacer, complete sequence; chloroplast	994	994	92% 0.0	95% JN408670.1	1026,41	85,1%
Select seq gb|JN408677.1|	Teucrium webbianum tRNA-Leu (trnL) gene, partial sequence; and trnL-trnF intergenic spacer, complete sequence; chloroplast	1046	1046	97% 0.0	95% JN408677.1	1024,43	84,9%
Select seq gb|JN408655.1|	Teucrium chamaedrys tRNA-Leu (trnL) gene, partial sequence; and trnL-trnF intergenic spacer, complete sequence; chloroplast	990	990	92% 0.0	95% JN408655.1	1022,28	84,7%
Select seq gb|JN408641.1|	Teucrium lucidum tRNA-Leu (trnL) gene, partial sequence; and trnL-trnF intergenic spacer, complete sequence; chloroplast	1033	1033	96% 0.0	95% JN408641.1	1022,24	84,7%
Select seq gb|HQ646990.1|	Teucrium flavum subsp. glaucum isolate TFG64 tRNA-Leu (trnL) gene and trnL-trnF intergenic spacer, complete sequence; and tRNA-Phe (trnF) gene, partial sequence; chloroplast	1064	1064	99% 0.0	95% HQ646990.1	1021,01	84,6%
Select seq gb|HQ646981.1|	Teucrium flavum subsp. glaucum isolate TFG54 tRNA-Leu (trnL) gene and trnL-trnF intergenic spacer, complete sequence; and tRNA-Phe (trnF) gene, partial sequence; chloroplast	1064	1064	99% 0.0	95% HQ646981.1	1021,01	84,6%
Select seq gb|JN408644.1|	Teucrium melissoides tRNA-Leu (trnL) gene, partial sequence; and trnL-trnF intergenic spacer, complete sequence; chloroplast	1031	1031	96% 0.0	95% JN408644.1	1020,26	84,6%
Select seq gb|JN408626.1|	Teucrium chamaedrys tRNA-Leu (trnL) gene, partial sequence; and trnL-trnF intergenic spacer, complete sequence; chloroplast	998	998	93% 0.0	95% JN408626.1	1019,46	84,5%
Select seq gb|JN408612.1|	Teucrium chamaedrys tRNA-Leu (trnL) gene, partial sequence; and trnL-trnF intergenic spacer, complete sequence; chloroplast	1038	1038	97% 0.0	95% JN408612.1	1016,60	84,3%
Select seq gb|HQ646991.1|	Teucrium flavum subsp. flavum isolate TFF56 tRNA-Leu (trnL) gene and trnL-trnF intergenic spacer, complete sequence; and tRNA-Phe (trnF) gene, partial sequence; chloroplast	1059	1059	99% 0.0	95% HQ646991.1	1016,21	84,2%
Select seq gb|JN408668.1|	Teucrium scordium tRNA-Leu (trnL) gene, partial sequence; and trnL-trnF intergenic spacer, complete sequence; chloroplast	1037	1037	97% 0.0	95% JN408668.1	1015,62	84,2%
Select seq gb|JN408602.1|	Teucrium aroanium tRNA-Leu (trnL) gene, partial sequence; and trnL-trnF intergenic spacer, complete sequence; chloroplast	1037	1037	97% 0.0	95% JN408602.1	1015,62	84,2%
Select seq gb|JN408635.1|	Teucrium intricatum tRNA-Leu (trnL) gene, partial sequence; and trnL-trnF intergenic spacer, complete sequence; chloroplast	1026	1026	96% 0.0	95% JN408635.1	1015,31	84,1%
Select seq gb|JN408606.1|	Teucrium botrys tRNA-Leu (trnL) gene, partial sequence; and trnL-trnF intergenic spacer, complete sequence; chloroplast	1026	1026	96% 0.0	95% JN408606.1	1015,31	84,1%
Select seq gb|JN408608.1|	Teucrium brevifolium tRNA-Leu (trnL) gene, partial sequence; and trnL-trnF intergenic spacer, complete sequence; chloroplast	1014	1014	95% 0.0	95% JN408608.1	1014,00	84,0%
Select seq gb|JN408645.1|	Teucrium microphyllum tRNA-Leu (trnL) gene, partial sequence; and trnL-trnF intergenic spacer, complete sequence; chloroplast	1033	1033	97% 0.0	95% JN408645.1	1011,70	83,8%
Select seq gb|JN408593.1|	Teucrium flavum subsp. glaucum tRNA-Leu (trnL) gene, partial sequence; and trnL-trnF intergenic spacer, complete sequence; chloroplast	1033	1033	97% 0.0	95% JN408593.1	1011,70	83,8%

Select seq gb|JN408637.1|	Teucrium jolyi tRNA-Leu (trnL) gene, partial sequence; and trnL-trnF intergenic spacer, complete sequence; chloroplast	1022	1022	96%	0.0	95%	JN408637.1	1011,35	83,8%
Select seq gb|GU331787.1|	Teucrium marum isolate TM12N tRNA-Leu (trnL) gene, partial sequence; trnL-trnF intergenic spacer, complete sequence; and tRNA-Phe (trnF) gene, partial sequence; chloroplast	1053	1053	99%	0.0	95%	GU331787.1	1010,45	83,7%
Select seq gb|GU331782.1|	Teucrium marum isolate TM1C tRNA-Leu (trnL) gene, partial sequence; trnL-trnF intergenic spacer, complete sequence; and tRNA-Phe (trnF) gene, partial sequence; chloroplast	1053	1053	99%	0.0	95%	GU331782.1	1010,45	83,7%
Select seq gb|JN408642.1|	Teucrium marum tRNA-Leu (trnL) gene, partial sequence; and trnL-trnF intergenic spacer, complete sequence; chloroplast	1020	1020	96%	0.0	95%	JN408642.1	1009,38	83,7%
Select seq gb|JN408673.1|	Teucrium subspinosum tRNA-Leu (trnL) gene, partial sequence; and trnL-trnF intergenic spacer, complete sequence; chloroplast	1018	1018	96%	0.0	95%	JN408673.1	1007,40	83,5%
Select seq gb|GU331791.1|	Teucrium marum isolate TM16W tRNA-Leu (trnL) gene, partial sequence; trnL-trnF intergenic spacer, complete sequence; and tRNA-Phe (trnF) gene, partial sequence; chloroplast	1048	1048	99%	0.0	95%	GU331791.1	1005,66	83,3%
Select seq gb|GU331790.1|	Teucrium marum isolate TM23W tRNA-Leu (trnL) gene, partial sequence; trnL-trnF intergenic spacer, complete sequence; and tRNA-Phe (trnF) gene, partial sequence; chloroplast	1042	1042	99%	0.0	95%	GU331790.1	999,90	82,9%
Select seq gb|GU331789.1|	Teucrium marum isolate TM15S tRNA-Leu (trnL) gene, partial sequence; trnL-trnF intergenic spacer, complete sequence; and tRNA-Phe (trnF) gene, partial sequence; chloroplast	1042	1042	99%	0.0	95%	GU331789.1	999,90	82,9%
Select seq gb|GU331786.1|	Teucrium marum isolate TM5N tRNA-Leu (trnL) gene, partial sequence; trnL-trnF intergenic spacer, complete sequence; and tRNA-Phe (trnF) gene, partial sequence; chloroplast	1042	1042	99%	0.0	95%	GU331786.1	999,90	82,9%
Select seq gb|GU331785.1|	Teucrium marum isolate TM20C tRNA-Leu (trnL) gene, partial sequence; trnL-trnF intergenic spacer, complete sequence; and tRNA-Phe (trnF) gene, partial sequence; chloroplast	1042	1042	99%	0.0	95%	GU331785.1	999,90	82,9%
Select seq gb|GU331784.1|	Teucrium marum isolate TM8C tRNA-Leu (trnL) gene, partial sequence; trnL-trnF intergenic spacer, complete sequence; and tRNA-Phe (trnF) gene, partial sequence; chloroplast	1042	1042	99%	0.0	95%	GU331784.1	999,90	82,9%
Select seq gb|GU331783.1|	Teucrium marum isolate TM6C tRNA-Leu (trnL) gene, partial sequence; trnL-trnF intergenic spacer, complete sequence; and tRNA-Phe (trnF) gene, partial sequence; chloroplast	1042	1042	99%	0.0	95%	GU331783.1	999,90	82,9%
Select seq gb|JN408611.1|	Teucrium chamaedrys tRNA-Leu (trnL) gene, partial sequence; and trnL-trnF intergenic spacer, complete sequence; chloroplast	1027	1027	97%	0.0	94%	JN408611.1	995,24	82,5%
Select seq gb|JN408604.1|	Teucrium betonicum tRNA-Leu (trnL) gene, partial sequence; trnL-trnF intergenic spacer, complete sequence; and tRNA-Phe (trnF) gene, partial sequence; chloroplast	1024	1024	97%	0.0	94%	JN408604.1	992,33	82,2%
Select seq gb|JN408652.1|	Teucrium oxylepis tRNA-Leu (trnL) gene, partial sequence; and trnL-trnF intergenic spacer, complete sequence; chloroplast	1022	1022	97%	0.0	94%	JN408652.1	990,39	82,1%
Select seq gb|GU331788.1|	Teucrium marum isolate TM25S tRNA-Leu (trnL) gene, partial sequence; trnL-trnF intergenic spacer, complete sequence; and tRNA-Phe (trnF) gene, partial sequence; chloroplast	1037	1037	99%	0.0	94%	GU331788.1	984,63	81,6%
Select seq gb|JN408653.1|	Teucrium pseudochamaepitys tRNA-Leu (trnL) gene, partial sequence; and trnL-trnF intergenic spacer, complete sequence; chloroplast	1016	1016	97%	0.0	94%	JN408653.1	984,58	81,6%
								0,00	0,0%
								0,00	

Select for downloading or viewing reports	Kh020_trnL Description	Max score	Total score	Query cover	E value	Ident	Accession	(Ident/Cover)* Max score	Deviation from top hit
Select seq gb|DQ667501.1|	Ziziphora taurica isolate x262 tRNA-Leu (trnL) gene and trnL-trnF intergenic spacer, partial sequence; chloroplast	1188	1188	94%	0.0	100%	DQ667501.1	1263,83	100,0%
Select seq gb|GU381510.1|	Ziziphora pamiroalaica voucher C:Murray et al. 10090 tRNA-Leu (trnL) gene and trnL-trnF intergenic spacer, partial sequence; chloroplast	1186	1186	94%	0.0	100%	GU381510.1	1261,70	99,8%
Select seq gb|GU381507.1|	Ziziphora tenuior voucher MSB:Fayvush et al. 03-1503 tRNA-Leu (trnL) gene and trnL-trnF intergenic spacer, partial sequence; chloroplast	1186	1186	94%	0.0	100%	GU381507.1	1261,70	99,8%
Select seq gb|GU381505.1|	Clinopodium troodi voucher W:Davis 1856 tRNA-Leu (trnL) gene and trnL-trnF intergenic spacer, partial sequence; chloroplast	1186	1186	94%	0.0	100%	GU381505.1	1261,70	99,8%
Select seq gb|GU381501.1|	Clinopodium graveolens subsp. rotundifolium voucher M:Podlech 47181 tRNA-Leu (trnL) gene and trnL-trnF intergenic spacer, partial sequence; chloroplast	1186	1186	94%	0.0	100%	GU381501.1	1261,70	99,8%
Select seq gb|GU381499.1|	Clinopodium suaveolens voucher M:Erben s.n. tRNA-Leu (trnL) gene and trnL-trnF intergenic spacer, partial sequence; chloroplast	1186	1186	94%	0.0	100%	GU381499.1	1261,70	99,8%
Select seq gb|GU381506.1|	Ziziphora tenuior voucher MSB:Nydegger 43557 tRNA-Leu (trnL) gene and trnL-trnF intergenic spacer, partial sequence; chloroplast	1158	1158	92%	0.0	100%	GU381506.1	1258,70	99,6%
Select seq gb|GU381502.1|	Ziziphora hispanica subsp. aragonensis voucher M:Podlech & Lippert 2492 tRNA-Leu (trnL) gene and trnL-trnF intergenic spacer, partial sequence; chloroplast	1158	1158	92%	0.0	100%	GU381502.1	1258,70	99,6%
Select seq gb|KP265673.1|	Ziziphora taurica subsp. cleonioides tRNA-Leu (trnL) gene and trnL-trnF intergenic spacer, partial sequence; chloroplast	1114	1114	89%	0.0	100%	KP265673.1	1251,69	99,0%
Select seq gb|KP265671.1|	Ziziphora serpyllacea tRNA-Leu (trnL) gene and trnL-trnF intergenic spacer, partial sequence; chloroplast	1114	1114	89%	0.0	100%	KP265671.1	1251,69	99,0%
Select seq gb|KP265670.1|	Ziziphora rigida tRNA-Leu (trnL) gene and trnL-trnF intergenic spacer, partial sequence; chloroplast	1114	1114	89%	0.0	100%	KP265670.1	1251,69	99,0%
Select seq gb|KP265669.1|	Ziziphora raddei tRNA-Leu (trnL) gene and trnL-trnF intergenic spacer, partial sequence; chloroplast	1114	1114	89%	0.0	100%	KP265669.1	1251,69	99,0%
Select seq gb|KP265666.1|	Ziziphora clinopodioides subsp. ronningeri tRNA-Leu (trnL) gene, partial sequence; trnL-trnF intergenic spacer, complete sequence; and tRNA-Phe (trnF) gene, partial sequence; chloroplast	1114	1114	89%	0.0	100%	KP265666.1	1251,69	99,0%
Select seq gb|KP265662.1|	Ziziphora clinopodioides subsp. filicaulis tRNA-Leu (trnL) gene, partial sequence; trnL-trnF intergenic spacer, complete sequence; and tRNA-Phe (trnF) gene, partial sequence; chloroplast	1114	1114	89%	0.0	100%	KP265662.1	1251,69	99,0%
Select seq gb|KP265661.1|	Ziziphora clinopodioides subsp. elbursensis tRNA-Leu (trnL) gene and trnL-trnF intergenic spacer, partial sequence; chloroplast	1114	1114	89%	0.0	100%	KP265661.1	1251,69	99,0%
Select seq gb|KP265659.1|	Ziziphora clinopodioides subsp. afghanica tRNA-Leu (trnL) gene, partial sequence; trnL-trnF intergenic spacer, complete sequence; and tRNA-Phe (trnF) gene, partial sequence; chloroplast	1114	1114	89%	0.0	100%	KP265659.1	1251,69	99,0%
Select seq gb|KP265658.1|	Ziziphora biebersteiniana trnL-trnF intergenic spacer, partial sequence; chloroplast	1114	1114	89%	0.0	100%	KP265658.1	1251,69	99,0%
Select seq gb|GU381497.1|	Clinopodium acinos voucher M:Podlech 50287 tRNA-Leu (trnL) gene and trnL-trnF intergenic spacer, partial sequence; chloroplast	1181	1181	94%	0.0	99%	GU381497.1	1243,82	98,4%
Select seq gb|GU381503.1|	Ziziphora hispanica voucher M:Lippert 24827 tRNA-Leu (trnL) gene and trnL-trnF intergenic spacer, partial sequence; chloroplast	1153	1153	92%	0.0	99%	GU381503.1	1240,73	98,2%
Select seq gb|JQ669021.1|	Clinopodium acinos voucher Judziewicz 14160 tRNA-Leu (trnL) gene and trnL-trnF intergenic spacer, partial sequence; plastid	1175	1175	94%	0.0	99%	JQ669021.1	1237,50	97,9%
Select seq gb|JF301402.1|	Ziziphora clinopodioides voucher DBG 980177 tRNA-Leu (trnL) gene and trnL-trnF intergenic spacer, partial sequence; chloroplast	1149	1149	92%	0.0	99%	JF301402.1	1236,42	97,8%
Select seq gb|AY840180.1|	Clinopodium alpinum tRNA-Leu (trnL) gene and trnL-trnF intergenic spacer, partial sequence; chloroplast	1149	1149	92%	0.0	99%	AY840180.1	1236,42	97,8%
Select seq gb|GU381500.1|	Clinopodium nanum voucher M:Braeuchler 2796 tRNA-Leu (trnL) gene and trnL-trnF intergenic spacer, partial sequence; chloroplast	1173	1173	94%	0.0	99%	GU381500.1	1235,39	97,8%
Select seq gb|AY506595.1|	Ziziphora hispanica tRNA-Leu (trnL) gene and trnL-trnF intergenic spacer, partial sequence; chloroplast	1147	1147	92%	0.0	99%	AY506595.1	1234,27	97,7%
Select seq gb|KP265668.1|	Ziziphora puschkinii tRNA-Leu (trnL) gene and trnL-trnF intergenic spacer, partial sequence; chloroplast	1109	1109	89%	0.0	99%	KP265668.1	1233,61	97,6%
Select seq gb|KP265667.1|	Ziziphora clinopodioides tRNA-Leu (trnL) gene and trnL-trnF intergenic spacer, partial sequence; chloroplast	1109	1109	89%	0.0	99%	KP265667.1	1233,61	97,6%
Select seq gb|KP265665.1|	Ziziphora clinopodioides subsp. rigida tRNA-Leu (trnL) gene, partial sequence; trnL-trnF intergenic spacer, complete sequence; and tRNA-Phe (trnF) gene, partial sequence; chloroplast	1109	1109	89%	0.0	99%	KP265665.1	1233,61	97,6%

Select seq gb|KP265664.1|	Ziphora clinopodioides subsp. pseudodasyantha tRNA-Leu (trnL) gene and trnL-trnF intergenic spacer, partial sequence; chloroplast	1109	1109	89%	0.0	99%	KP265664.1	1233,61	97,6%
Select seq gb|KP265663.1|	Ziphora clinopodioides subsp. kurdica tRNA-Leu (trnL) gene, partial sequence; trnL-trnF intergenic spacer, complete sequence; and tRNA-Phe (trnF) gene, partial sequence; chloroplast	1109	1109	89%	0.0	99%	KP265663.1	1233,61	97,6%
Select seq gb|KP265672.1|	Ziphora taurica subsp. taurica tRNA-Leu (trnL) gene and trnL-trnF intergenic spacer, partial sequence; chloroplast	1103	1103	89%	0.0	99%	KP265672.1	1226,93	97,1%
Select seq gb|KP265660.1|	Ziphora clinopodioides subsp. bungeana tRNA-Leu (trnL) gene, partial sequence; trnL-trnF intergenic spacer, complete sequence; and tRNA-Phe (trnF) gene, partial sequence; chloroplast	1103	1103	89%	0.0	99%	KP265660.1	1226,93	97,1%
Select seq gb|GU381517.1|	Clinopodium barosmum voucher BM<GBR-LONDON>:McLaren N193 tRNA-Leu (trnL) gene and trnL-trnF intergenic spacer, partial sequence; chloroplast	1164	1164	94%	0.0	99%	GU381517.1	1225,91	97,0%
Select seq gb|GU381516.1|	Clinopodium wardii voucher BM<GBR-LONDON>:Ludlow et al. 14234 tRNA-Leu (trnL) gene and trnL-trnF intergenic spacer, partial sequence; chloroplast	1164	1164	94%	0.0	99%	GU381516.1	1225,91	97,0%
Select seq gb|GU381515.1|	Clinopodium hydaspidis voucher BM<GBR-LONDON>:Mohd 133 tRNA-Leu (trnL) gene and trnL-trnF intergenic spacer, partial sequence; chloroplast	1164	1164	94%	0.0	99%	GU381515.1	1225,91	97,0%
Select seq gb|GU381514.1|	Clinopodium nepalense voucher FR:Stainton 6024 tRNA-Leu (trnL) gene and trnL-trnF intergenic spacer, partial sequence; chloroplast	1164	1164	94%	0.0	99%	GU381514.1	1225,91	97,0%
Select seq gb|GU381511.1|	Clinopodium piperitum voucher BM<GBR-LONDON>:Vickery 454 tRNA-Leu (trnL) gene and trnL-trnF intergenic spacer, partial sequence; chloroplast	1164	1164	94%	0.0	99%	GU381511.1	1225,91	97,0%
Select seq gb|GU381513.1|	Clinopodium nepalense voucher BM<GBR-LONDON>:Mikage et al. 9550294 tRNA-Leu (trnL) gene and trnL-trnF intergenic spacer, partial sequence; chloroplast	1136	1136	92%	0.0	99%	GU381513.1	1222,43	96,7%
Select seq gb|GU381512.1|	Clinopodium piperitum voucher E:Stainton 7320 tRNA-Leu (trnL) gene and trnL-trnF intergenic spacer, partial sequence; chloroplast	1158	1158	94%	0.0	99%	GU381512.1	1219,60	96,5%
Select seq gb|GU381484.1|	Micromeria flagellaris voucher E:van der Werff & McPherson 13570 tRNA-Leu (trnL) gene and trnL-trnF intergenic spacer, partial sequence; chloroplast	1136	1136	94%	0.0	99%	GU381484.1	1196,43	94,7%
Select seq gb|GU381483.1|	Micromeria flagellaris voucher E:Clement et al 2140 tRNA-Leu (trnL) gene and trnL-trnF intergenic spacer, partial sequence; chloroplast	1136	1136	94%	0.0	99%	GU381483.1	1196,43	94,7%
Select seq gb|GU381509.1|	Ziziphora capitata voucher M:Braeuchler 3142 tRNA-Leu (trnL) gene and trnL-trnF intergenic spacer, partial sequence; chloroplast	1134	1134	94%	0.0	99%	GU381509.1	1194,32	94,5%
Select seq gb|AY506594.1|	Acinos alpinus tRNA-Leu (trnL) gene and trnL-trnF intergenic spacer, partial sequence; chloroplast	1094	1094	92%	0.0	99%	AY506594.1	1177,24	93,1%
Select seq gb|GU381629.1|	Thymbra capitata voucher M:Braeuchler 2518 tRNA-Leu (trnL) gene and trnL-trnF intergenic spacer, partial sequence; chloroplast	1120	1120	94%	0.0	98%	GU381629.1	1167,66	92,4%
Select seq gb|JF301401.1|	Thymbra capitata voucher UCBG 96.0817 tRNA-Leu (trnL) gene and trnL-trnF intergenic spacer, partial sequence; chloroplast	1094	1094	92%	0.0	98%	JF301401.1	1165,35	92,2%
Select seq gb|GU381496.1|	Killickia pilosa voucher M:Braeuchler 3832 tRNA-Leu (trnL) gene and trnL-trnF intergenic spacer, partial sequence; chloroplast	1116	1116	94%	0.0	98%	GU381496.1	1163,49	92,1%
Select seq gb|GU381493.1|	Killickia grandiflora voucher M:Braeuchler 3811 tRNA-Leu (trnL) gene and trnL-trnF intergenic spacer, partial sequence; chloroplast	1116	1116	94%	0.0	98%	GU381493.1	1163,49	92,1%
Select seq gb|GU381481.1|	Micromeria cf. madagascariensis Morawetz 205 tRNA-Leu (trnL) gene and trnL-trnF intergenic spacer, partial sequence; chloroplast	1114	1114	94%	0.0	98%	GU381481.1	1161,40	91,9%
Select seq gb|AY840207.1|	Thymbra spicata tRNA-Leu (trnL) gene and trnL-trnF intergenic spacer, partial sequence; chloroplast	1114	1114	94%	0.0	98%	AY840207.1	1161,40	91,9%
Select seq gb|GU381495.1|	Killickia pilosa voucher M:Braeuchler 3810 tRNA-Leu (trnL) gene and trnL-trnF intergenic spacer, partial sequence; chloroplast	1101	1101	93%	0.0	98%	GU381495.1	1160,19	91,8%
Select seq gb|GU381485.1|	Micromeria sphaerophylla voucher E:Lewis et al 1064 tRNA-Leu (trnL) gene and trnL-trnF intergenic spacer, partial sequence; chloroplast	1112	1112	94%	0.0	98%	GU381485.1	1159,32	91,7%
Select seq gb|GU381632.1|	Thymbra spicata voucher M:Braeuchler 4548 tRNA-Leu (trnL) gene and trnL-trnF intergenic spacer, partial sequence; chloroplast	1110	1110	94%	0.0	98%	GU381632.1	1157,23	91,6%
Select seq gb|GU381627.1|	Thymbra sintenisii subsp. isaurica voucher E:Goener 12628 tRNA-Leu (trnL) gene and trnL-trnF intergenic spacer, partial sequence; chloroplast	1109	1109	94%	0.0	98%	GU381627.1	1156,19	91,5%
Select seq gb|GU381489.1|	Killickia lutea voucher NU<ZAF>:Hilliard & Burtt 9876 tRNA-Leu (trnL) gene and trnL-trnF intergenic spacer, partial sequence; chloroplast	1105	1105	94%	0.0	98%	GU381489.1	1152,02	91,2%
Select seq gb|GU381488.1|	Killickia compacta voucher M:Braeuchler 3816 tRNA-Leu (trnL) gene and trnL-trnF intergenic spacer, partial sequence; chloroplast	1105	1105	94%	0.0	98%	GU381488.1	1152,02	91,2%
Select seq gb|JQ690290.1|	Origanum elongatum isolate H5_O_elo trnL-trnF intergenic spacer, partial sequence; chloroplast	1134	1134	97%	0.0	98%	JQ690290.1	1145,69	90,7%
Select seq gb|JQ690289.1|	Origanum rotundifolium isolate DNA3_O_rot trnL-trnF intergenic spacer, partial sequence; chloroplast	1134	1134	97%	0.0	98%	JQ690289.1	1145,69	90,7%

Select seq gb|GU381633.1|	Thymus caespititius voucher M:Heubl s.n. tRNA-Leu (trnL) gene and trnL-trnF intergenic spacer, partial sequence; chloroplast	1098	1098	94% 0.0	98% GU381633.1	1144,72	90,6%
Select seq gb|AY840202.1|	Origanum vulgare tRNA-Leu (trnL) gene and trnL-trnF intergenic spacer, partial sequence; chloroplast	1098	1098	94% 0.0	98% AY840202.1	1144,72	90,6%
Select seq gb|KR150218.1|	Thymus sp. 2 Kh69 trnL-trnF intergenic spacer region, partial sequence; chloroplast	1140	1140	98% 0.0	98% KR150218.1	1140,00	90,2%
Select seq gb|JX880022.1|	Origanum vulgare subsp. vulgare chloroplast, complete genome	1162	1162	100% 0.0	98% JX880022.1	1138,76	90,1%
Select seq gb|KR063657.1|	Thymus sibthorpii tRNA-Leu (trnL) gene, partial sequence; trnL-trnF intergenic spacer, complete sequence; and tRNA-Phe (trnF) gene, partial sequence; chloroplast	1155	1155	99% 0.0	97% KR063657.1	1131,67	89,5%
Select seq gb|KR150229.1|	Thymus sp. 5 Kh16 trnL-trnF intergenic spacer region, partial sequence; chloroplast	1142	1142	98% 0.0	97% KR150229.1	1130,35	89,4%
Select seq gb|KR150215.1|	Thymus sp. 1 Kh08 trnL-trnF intergenic spacer region, partial sequence; chloroplast	1129	1129	97% 0.0	97% KR150215.1	1129,00	89,3%
Select seq gb|GU381640.1|	Argantoniella salzmannii voucher M:Barra et al. 2673GL tRNA-Leu (trnL) gene and trnL-trnF intergenic spacer, partial sequence; chloroplast	1092	1092	94% 0.0	97% GU381640.1	1126,85	89,2%
Select seq gb|GU381635.1|	Thymus broussonetii subsp. hannonis voucher MSB:Podlech 142 tRNA-Leu (trnL) gene and trnL-trnF intergenic spacer, partial sequence; chloroplast	1092	1092	94% 0.0	97% GU381635.1	1126,85	89,2%
Select seq emb|AJ505543.1|	Origanum vulgare plastid trnL-trnF intergenic spacer, specimen voucher cult., K-000-69-19317, chase 13334 (K)	1125	1125	97% 0.0	97% AJ505543.1	1125,00	89,0%
Select seq gb|JQ690293.1|	Origanum dayi isolate H43_O_day trnL-trnF intergenic spacer, partial sequence; chloroplast	1123	1123	97% 0.0	97% JQ690293.1	1123,00	88,9%
Select seq gb|KR063656.1|	Satureja pilosa subsp. origanita tRNA-Leu (trnL) gene, partial sequence; trnL-trnF intergenic spacer, complete sequence; and tRNA-Phe (trnF) gene, partial sequence; chloroplast	1146	1146	99% 0.0	97% KR063656.1	1122,85	88,8%
Select seq gb|KR150222.1|	Thymus sp. 3 Kh65 trnL-trnF intergenic spacer region, partial sequence; chloroplast	1110	1110	96% 0.0	97% KR150222.1	1121,56	88,7%
Select seq gb|KR150225.1|	Thymus sp. 4 Kh24 trnL-trnF intergenic spacer region, partial sequence; chloroplast	1109	1109	96% 0.0	97% KR150225.1	1120,55	88,7%
Select seq gb|EU556525.1|	Thymus quinquecostatus isolate CL tRNA-Leu (trnL) gene and trnL-trnF intergenic spacer, partial sequence; chloroplast	1151	1151	100% 0.0	97% EU556525.1	1116,47	88,3%
Select seq gb|KR150209.1|	Satureja sp. 3 Kh90 trnL-trnF intergenic spacer region, partial sequence; chloroplast	1092	1092	95% 0.0	97% KR150209.1	1114,99	88,2%
Select seq gb|EU556539.1|	Thymus quinquecostatus isolate ZY tRNA-Leu (trnL) gene and trnL-trnF intergenic spacer, partial sequence; chloroplast	1149	1149	100% 0.0	97% EU556539.1	1114,53	88,2%
Select seq gb|EU556538.1|	Thymus quinquecostatus isolate ZJ tRNA-Leu (trnL) gene and trnL-trnF intergenic spacer, partial sequence; chloroplast	1149	1149	100% 0.0	97% EU556538.1	1114,53	88,2%
Select seq gb|EU556533.1|	Thymus quinquecostatus isolate HY tRNA-Leu (trnL) gene and trnL-trnF intergenic spacer, partial sequence; chloroplast	1149	1149	100% 0.0	97% EU556533.1	1114,53	88,2%
Select seq gb|EU556532.1|	Thymus amurensis isolate HX tRNA-Leu (trnL) gene and trnL-trnF intergenic spacer, partial sequence; chloroplast	1149	1149	100% 0.0	97% EU556532.1	1114,53	88,2%
Select seq gb|EU556531.1|	Thymus amurensis isolate HW tRNA-Leu (trnL) gene and trnL-trnF intergenic spacer, partial sequence; chloroplast	1149	1149	100% 0.0	97% EU556531.1	1114,53	88,2%
Select seq gb|EU556527.1|	Thymus mongolicus isolate G2 tRNA-Leu (trnL) gene and trnL-trnF intergenic spacer, partial sequence; chloroplast	1149	1149	100% 0.0	97% EU556527.1	1114,53	88,2%
Select seq gb|EU556526.1|	Thymus quinquecostatus isolate D2 tRNA-Leu (trnL) gene and trnL-trnF intergenic spacer, partial sequence; chloroplast	1149	1149	100% 0.0	97% EU556526.1	1114,53	88,2%
Select seq gb|KR150214.1|	Satureja sp. 1 Kh04 trnL-trnF intergenic spacer region, partial sequence; chloroplast	1098	1098	96% 0.0	97% KR150214.1	1109,44	87,8%
Select seq gb|DQ667513.1|	Clinopodium vulgare isolate x423 tRNA-Leu (trnL) gene and trnL-trnF intergenic spacer, partial sequence; chloroplast	1131	1131	99% 0.0	97% DQ667513.1	1108,15	87,7%
Select seq gb|DQ667504.1|	Cunila incana isolate x296 tRNA-Leu (trnL) gene and trnL-trnF intergenic spacer, partial sequence; chloroplast	1125	1125	99% 0.0	97% DQ667504.1	1102,27	87,2%
Select seq gb|KC414276.1|	Mentha canadensis isolate 511190001 tRNA-Leu (trnL) gene and trnL-trnF intergenic spacer, partial sequence; chloroplast	1134	1134	100% 0.0	97% KC414276.1	1099,98	87,0%
Select seq emb|AJ505544.1|	Thymus serpyllum var. citriodorum plastid trnL-trnF intergenic spacer, specimen voucher cult., K-1975-1177, Chase 13331 (K)	1099	1099	97% 0.0	97% AJ505544.1	1099,00	87,0%
Select seq emb|AJ505541.1|	Mentha suaveolens plastid trnL-trnF intergenic spacer, specimen voucher cult., K-1970-3169 (K)	1098	1098	97% 0.0	97% AJ505541.1	1098,00	86,9%

Select seq gb|DQ667492.1|	Lepechinia lancifolia isolate x232 tRNA-Leu (trnL) gene and trnL-trnF intergenic spacer, partial sequence; chloroplast	1120	1120	99% 0.0	97% DQ667492.1	1097,37	86,8%
Select seq gb|DQ667507.1|	Pycnanthemum virginianum isolate x313 tRNA-Leu (trnL) gene and trnL-trnF intergenic spacer, partial sequence; chloroplast	1131	1131	100% 0.0	97% DQ667507.1	1097,07	86,8%
Select seq gb|DQ667497.1|	Hoehnea epilobioides isolate x258 tRNA-Leu (trnL) gene and trnL-trnF intergenic spacer, partial sequence; chloroplast	1131	1131	100% 0.0	97% DQ667497.1	1097,07	86,8%
Select seq gb|FJ593456.1|	Mentha sp. JSZ-2009a tRNA-Leu (trnL) gene and trnL-trnF intergenic spacer, partial sequence; chloroplast	1127	1127	100% 0.0	97% FJ593456.1	1093,19	86,5%
Select seq gb|DQ667436.1|	Hedeoma costata isolate x067 tRNA-Leu (trnL) gene and trnL-trnF intergenic spacer, partial sequence; chloroplast	1125	1125	100% 0.0	97% DQ667436.1	1091,25	86,3%
Select seq gb|DQ667496.1|	Glechon thymoides isolate x256 tRNA-Leu (trnL) gene and trnL-trnF intergenic spacer, partial sequence; chloroplast	1112	1112	99% 0.0	97% DQ667496.1	1089,54	86,2%
Select seq gb|DQ667514.1|	Mentha arvensis isolate x424 tRNA-Leu (trnL) gene and trnL-trnF intergenic spacer, partial sequence; chloroplast	1099	1099	98% 0.0	96% DQ667514.1	1076,57	85,2%
Select seq gb|EU556537.1|	Thymus quinquecostatus isolate XZ tRNA-Leu (trnL) gene and trnL-trnF intergenic spacer, partial sequence; chloroplast	1112	1112	100% 0.0	96% EU556537.1	1067,52	84,5%
Select seq gb|DQ667520.1|	Pogogyne floribunda isolate x534 tRNA-Leu (trnL) gene and trnL-trnF intergenic spacer, partial sequence; chloroplast	1109	1109	100% 0.0	96% DQ667520.1	1064,64	84,2%
Select seq gb|DQ667522.1|	Acanthomintha lanceolata isolate x545 tRNA-Leu (trnL) gene and trnL-trnF intergenic spacer, partial sequence; chloroplast	1096	1096	99% 0.0	96% DQ667522.1	1062,79	84,1%
Select seq gb|DQ667438.1|	Conradina canescens isolate x069 tRNA-Leu (trnL) gene and trnL-trnF intergenic spacer, partial sequence; chloroplast	1101	1101	100% 0.0	96% DQ667438.1	1056,96	83,6%
Select seq gb|AY570459.1|	Lepechinia chamaedryoides voucher JBW 2537 tRNA-Leu and trnL-trnF intergenic spacer, partial sequence; chloroplast	1098	1098	100% 0.0	96% AY570459.1	1054,08	
						0,00	
						0,00	

Select for downloading or viewing reports	Kh021_ITS Description	Max score	Total score	Query cover	E value	Ident	Accession	(Ident/Cover)* Max score	Deviation from top hit
Select seq gb|EF679734.1|	Althaea armeniaca voucher PE427 internal transcribed spacer 1, partial sequence; 5.8S ribosomal RNA gene, complete sequence; and internal transcribed spacer 2, partial sequence	457	457	93%	2,00E-124	79%	EF679734.1	388,20	100,0%
Select seq gb|EF419540.1|	Althaea cannabina isolate IAca0345 internal transcribed spacer 1, partial sequence; 5.8S ribosomal RNA gene, complete sequence; and internal transcribed spacer 2, partial sequence	442	442	92%	5,00E-120	79%	EF419540.1	379,54	97,8%
Select seq gb|EF419539.1|	Althaea cannabina isolate IAca594 internal transcribed spacer 1, partial sequence; 5.8S ribosomal RNA gene, complete sequence; and internal transcribed spacer 2, partial sequence	442	442	92%	5,00E-120	79%	EF419539.1	379,54	97,8%
Select seq gb|EF679733.1|	Althaea officinalis voucher PE512 internal transcribed spacer 1, partial sequence; 5.8S ribosomal RNA gene, complete sequence; and internal transcribed spacer 2, partial sequence	436	436	93%	2,00E-118	78%	EF679733.1	365,68	94,2%
Select seq gb|EF419542.1|	Althaea armeniaca isolate IAar427 internal transcribed spacer 1, partial sequence; 5.8S ribosomal RNA gene, complete sequence; and internal transcribed spacer 2, partial sequence	435	435	90%	9,00E-118	79%	EF419542.1	381,83	98,4%
Select seq gb|EF419536.1|	Althaea officinalis isolate IAof604 internal transcribed spacer 1, partial sequence; 5.8S ribosomal RNA gene, complete sequence; and internal transcribed spacer 2, partial sequence	427	427	92%	1,00E-115	78%	EF419536.1	362,02	93,3%
Select seq gb|EF419537.1|	Althaea officinalis isolate IAof0330 internal transcribed spacer 1, partial sequence; 5.8S ribosomal RNA gene, complete sequence; and internal transcribed spacer 2, partial sequence	425	425	92%	5,00E-115	78%	EF419537.1	360,33	92,8%
Select seq gb|AF303026.1|	Althaea officinalis internal transcribed spacer 1, partial sequence; 5.8S ribosomal RNA gene, complete sequence; and internal transcribed spacer 2, partial sequence	411	411	90%	1,00E-110	78%	AF303026.1	356,20	91,8%
Select seq gb|EF419547.1|	Anisodontea malvastroides internal transcribed spacer 1, partial sequence; 5.8S ribosomal RNA gene, complete sequence; and internal transcribed spacer 2, partial sequence	228	228	90%	2,00E-55	73%	EF419547.1	184,93	47,6%
Select seq gb|AY172219.1|	Andeimalva machupicchensis internal transcribed spacer 1, 5.8S ribosomal RNA gene, and internal transcribed spacer 2, complete sequence	230	230	85%	4,00E-56	74%	AY172219.1	200,24	51,6%
Select seq gb|AY172229.1|	Andeimalva spiciformis internal transcribed spacer 1, 5.8S ribosomal RNA gene, and internal transcribed spacer 2, complete sequence	222	222	85%	7,00E-54	73%	AY172229.1	190,66	49,1%
Select seq gb|AY591833.1|	Kearnemalvastrum lacteum internal transcribed spacer 1, 5.8S ribosomal RNA gene, and internal transcribed spacer 2, complete sequence	219	219	85%	9,00E-53	73%	AY591833.1	188,08	48,4%
Select seq gb|AY172220.1|	Andeimalva mandonii internal transcribed spacer 1, 5.8S ribosomal RNA gene, and internal transcribed spacer 2, complete sequence	213	213	85%	4,00E-51	73%	AY172220.1	182,93	47,1%
Select seq gb|AY172199.1|	Andeimalva chilensis internal transcribed spacer 1, 5.8S ribosomal RNA gene, and internal transcribed spacer 2, complete sequence	204	204	88%	3,00E-48	73%	AY172199.1	169,23	43,6%
Select seq gb|AY591834.1|	Kearnemalvastrum sp. Ventura A. 816 internal transcribed spacer 1, 5.8S ribosomal RNA gene, and internal transcribed spacer 2, complete sequence	198	198	85%	1,00E-46	73%	AY591834.1	170,05	43,8%
Select seq gb|AF271166.1|AF271166	Iliamna bakeri isolate 2McCloud internal transcribed spacer 1, 5.8S ribosomal RNA gene, and internal transcribed spacer 2, complete sequence	178	178	85%	2,00E-40	72%	AF271166.1	150,78	38,8%
Select seq gb|AY591809.1|	Acaulimalva alismatifolia internal transcribed spacer 1, 5.8S ribosomal RNA gene, and internal transcribed spacer 2, complete sequence	172	172	85%	7,00E-39	72%	AY591809.1	145,69	37,5%
Select seq gb|AY591843.1|	Monteiroa glomerata internal transcribed spacer 1, 5.8S ribosomal RNA gene, and internal transcribed spacer 2, complete sequence	165	165	85%	1,00E-36	72%	AY591843.1	139,76	36,0%
Select seq gb|AF271183.1|AF271183	Iliamna latibracteata isolate 1 internal transcribed spacer 1, 5.8S ribosomal RNA gene, and internal transcribed spacer 2, complete sequence	147	147	85%	4,00E-31	71%	AF271183.1	122,79	31,6%
Select seq gb|AF271185.1|AF271185	Iliamna latibracteata isolate 2 internal transcribed spacer 1, 5.8S ribosomal RNA gene, and internal transcribed spacer 2, complete sequence	141	141	85%	2,00E-29	71%	AF271185.1	117,78	30,3%
Select seq gb|AF271169.1|AF271169	Iliamna bakeri isolate 3McBryde internal transcribed spacer 1, 5.8S ribosomal RNA gene, and internal transcribed spacer 2, complete sequence	139	139	85%	8,00E-29	71%	AF271169.1	116,11	29,9%
Select seq emb|AJ520075.1|	Sidalcea sp. Oswald 9466 ITS1, 5.8S rRNA gene and ITS2, clone C4	139	139	85%	8,00E-29	71%	AJ520075.1	116,11	29,9%
Select seq gb|AF271184.1|AF271184	Iliamna latibracteata isolate 1B internal transcribed spacer 1, 5.8S ribosomal RNA gene, and internal transcribed spacer 2, complete sequence	135	135	85%	1,00E-27	71%	AF271184.1	112,76	29,0%

Select seq gb|AF271168.1|AF271168	Iliamna bakeri isolate 4McBryde internal transcribed spacer 1, 5.8S ribosomal RNA gene, and internal transcribed spacer 2, complete sequence	134	134	85%	3,00E-27	71%	AF271168.1	111,93	28,8%
Select seq gb|AF271165.1|AF271165	Iliamna bakeri isolate 1McCloud internal transcribed spacer 1, 5.8S ribosomal RNA gene, and internal transcribed spacer 2, complete sequence	134	134	85%	3,00E-27	71%	AF271165.1	111,93	28,8%
Select seq emb|AJ304936.1|	Callirhoe digitata 5.8S rRNA gene, internal transcribed spacer 1 (ITS1) and internal transcribed spacer 2 (ITS2)	126	126	87%	6,00E-25	71%	AJ304936.1	102,83	26,5%
Select seq gb|AF271167.1|AF271167	Iliamna bakeri isolate 4McCloud internal transcribed spacer 1, 5.8S ribosomal RNA gene, and internal transcribed spacer 2, complete sequence	122	122	85%	8,00E-24	71%	AF271167.1	101,91	26,3%
Select seq gb|AF303024.1|AF303024S1	Anisodontea capensis internal transcribed spacer 1 and 5.8S ribosomal RNA gene, partial sequence	189	189	42%	7,00E-44	78%	AF303024.1	0,00	0,0%
Select seq emb|AJ274972.1|	Kitaibela vitifolia internal transcribed spacer 1 (ITS1)	224	224	32%	2,00E-54	83%	AJ274972.1	0,00	0,0%
Select seq gb|AY172196.1|	Sphaeralcea philippiana internal transcribed spacer 1, 5.8S ribosomal RNA gene, and internal transcribed spacer 2, complete sequence	137	137	54%	3,00E-28	73%	AY172196.1	0,00	0,0%
Select seq emb|AJ251165.1|	Anisodontea capensis internal transcribed spacer 1 (ITS1)	158	158	32%	2,00E-34	78%	AJ251165.1	0,00	0,0%
Select seq emb|AJ849677.1|	Eremalche rotundifolia ITS1, 5.8S rRNA gene and ITS2 (partial), specimen voucher Baldwin & Martens 120 (JEPS)	119	119	13%	1,00E-22	88%	AJ849677.1	0,00	0,0%
Select seq gb|AY517650.1|	Minuartia moehringioides internal transcribed spacer 1, partial sequence; 5.8S ribosomal RNA gene, complete sequence; and internal transcribed spacer 2, partial sequence	52,8	52.8	3%	0.010	100%	AY517650.1	0,00	0,0%
								0,00	
								0,00	
								0,00	
								0,00	
								0,00	
								0,00	
								0,00	
								0,00	
								0,00	
								0,00	
								0,00	
								0,00	
								0,00	
								0,00	
								0,00	
								0,00	
								0,00	
								0,00	
								0,00	
								0,00	
								0,00	
								0,00	
								0,00	
								0,00	
								0,00	
								0,00	
								0,00	
								0,00	
								0,00	
								0,00	
								0,00	
								0,00	

0,00
0,00
0,00
0,00
0,00
0,00
0,00
0,00
0,00
0,00
0,00
0,00
0,00
0,00
0,00
0,00
0,00
0,00
0,00
0,00
0,00
0,00
0,00
0,00
0,00
0,00
0,00
0,00
0,00
0,00
0,00
0,00
0,00
0,00

Select for downloading or viewing reports	Kh023_trnL Description	Max score	Total score	Query cover	E value	Ident	Accession	(Ident/Cover)* Max score	Deviation from top hit
Select seq gb|JN408629.1|	Teucrium eriocephalum subsp. almeriense tRNA-Leu (trnL) gene, partial sequence; and trnL-trnF intergenic spacer, complete sequence; chloroplast	800	800	79%	0.0	94%	JN408629.1	951,90	100,0%
Select seq gb|JN408656.1|	Teucrium capitatum tRNA-Leu (trnL) gene, partial sequence; and trnL-trnF intergenic spacer, complete sequence; chloroplast	798	798	79%	0.0	94%	JN408656.1	949,52	99,8%
Select seq gb|JN408634.1|	Teucrium stocksianum subsp. incanum tRNA-Leu (trnL) gene, partial sequence; and trnL-trnF intergenic spacer, complete sequence; chloroplast	797	797	79%	0.0	94%	JN408634.1	948,33	99,6%
Select seq gb|JQ044771.1|	Teucrium polium subsp. capitatum isolate TPC2 tRNA-Leu (trnL) gene, partial sequence; trnL-trnF intergenic spacer, complete sequence; and tRNA-Phe (trnF) gene, partial sequence; chloroplast	815	815	90%	0.0	91%	JQ044771.1	824,06	86,6%
Select seq gb|JQ044773.1|	Teucrium polium subsp. capitatum isolate TPC1 tRNA-Leu (trnL) gene, partial sequence; trnL-trnF intergenic spacer, complete sequence; and tRNA-Phe (trnF) gene, partial sequence; chloroplast	809	809	90%	0.0	91%	JQ044773.1	817,99	85,9%
Select seq gb|JQ044772.1|	Teucrium polium subsp. capitatum isolate TPC4 tRNA-Leu (trnL) gene, partial sequence; trnL-trnF intergenic spacer, complete sequence; and tRNA-Phe (trnF) gene, partial sequence; chloroplast	809	809	90%	0.0	91%	JQ044772.1	817,99	85,9%
Select seq gb|JQ044780.1|	Teucrium polium subsp. polium isolate TP12 tRNA-Leu (trnL) gene, partial sequence; trnL-trnF intergenic spacer, complete sequence; and tRNA-Phe (trnF) gene, partial sequence; chloroplast	828	828	94%	0.0	91%	JQ044780.1	801,57	84,2%
Select seq gb|JQ044779.1|	Teucrium polium subsp. polium isolate TP11 tRNA-Leu (trnL) gene, partial sequence; trnL-trnF intergenic spacer, complete sequence; and tRNA-Phe (trnF) gene, partial sequence; chloroplast	828	828	94%	0.0	91%	JQ044779.1	801,57	84,2%
Select seq gb|JQ044778.1|	Teucrium polium subsp. polium isolate TP10 tRNA-Leu (trnL) gene, partial sequence; trnL-trnF intergenic spacer, complete sequence; and tRNA-Phe (trnF) gene, partial sequence; chloroplast	828	828	94%	0.0	91%	JQ044778.1	801,57	84,2%
Select seq gb|JN408607.1|	Teucrium bracteatum tRNA-Leu (trnL) gene, partial sequence; and trnL-trnF intergenic spacer, complete sequence; chloroplast	728	728	83%	0.0	91%	JN408607.1	798,17	83,9%
Select seq gb|JN408632.1|	Teucrium heterophyllum tRNA-Leu (trnL) gene, partial sequence; and trnL-trnF intergenic spacer, complete sequence; chloroplast	798	798	91%	0.0	91%	JN408632.1	798,00	83,8%
Select seq gb|JN408655.1|	Teucrium chamaedrys tRNA-Leu (trnL) gene, partial sequence; and trnL-trnF intergenic spacer, complete sequence; chloroplast	701	701	80%	0.0	91%	JN408655.1	797,39	83,8%
Select seq gb|JN408626.1|	Teucrium chamaedrys tRNA-Leu (trnL) gene, partial sequence; and trnL-trnF intergenic spacer, complete sequence; chloroplast	701	701	80%	0.0	91%	JN408626.1	797,39	83,8%
Select seq gb|KR150202.1|	Teucrium sp. 5 Kh108 trnL-trnF intergenic spacer region, partial sequence; chloroplast	782	782	90%	0.0	90%	KR150202.1	782,00	82,2%
Select seq gb|JN408657.1|	Teucrium polium tRNA-Leu (trnL) gene, partial sequence; and trnL-trnF intergenic spacer, complete sequence; chloroplast	817	817	95%	0.0	90%	JN408657.1	774,00	81,3%
Select seq gb|JN408646.1|	Teucrium montanum tRNA-Leu (trnL) gene, partial sequence; and trnL-trnF intergenic spacer, complete sequence; chloroplast	817	817	95%	0.0	90%	JN408646.1	774,00	81,3%
Select seq gb|JN408625.1|	Teucrium capitatum tRNA-Leu (trnL) gene, partial sequence; and trnL-trnF intergenic spacer, complete sequence; chloroplast	817	817	95%	0.0	90%	JN408625.1	774,00	81,3%
Select seq gb|JN408624.1|	Teucrium capitatum tRNA-Leu (trnL) gene, partial sequence; and trnL-trnF intergenic spacer, complete sequence; chloroplast	817	817	95%	0.0	90%	JN408624.1	774,00	81,3%
Select seq gb|JN408623.1|	Teucrium aureum tRNA-Leu (trnL) gene, partial sequence; and trnL-trnF intergenic spacer, complete sequence; chloroplast	817	817	95%	0.0	90%	JN408623.1	774,00	81,3%
Select seq gb|JN408621.1|	Teucrium pumilum tRNA-Leu (trnL) gene, partial sequence; and trnL-trnF intergenic spacer, complete sequence; chloroplast	817	817	95%	0.0	90%	JN408621.1	774,00	81,3%
Select seq gb|JN408677.1|	Teucrium webbianum tRNA-Leu (trnL) gene, partial sequence; and trnL-trnF intergenic spacer, complete sequence; chloroplast	713	713	83%	0.0	90%	JN408677.1	773,13	81,2%
Select seq gb|JN408645.1|	Teucrium microphyllum tRNA-Leu (trnL) gene, partial sequence; and trnL-trnF intergenic spacer, complete sequence; chloroplast	708	708	83%	0.0	90%	JN408645.1	767,71	80,7%
Select seq gb|JN408641.1|	Teucrium lucidum tRNA-Leu (trnL) gene, partial sequence; and trnL-trnF intergenic spacer, complete sequence; chloroplast	708	708	83%	0.0	90%	JN408641.1	767,71	80,7%
Select seq gb|JN408612.1|	Teucrium chamaedrys tRNA-Leu (trnL) gene, partial sequence; and trnL-trnF intergenic spacer, complete sequence; chloroplast	708	708	83%	0.0	90%	JN408612.1	767,71	80,7%
Select seq gb|HQ646990.1|	Teucrium flavum subsp. glaucum isolate TFG64 tRNA-Leu (trnL) gene and trnL-trnF intergenic spacer, complete sequence; and tRNA-Phe (trnF) gene, partial sequence; chloroplast	708	708	83%	0.0	90%	HQ646990.1	767,71	80,7%
Select seq gb|HQ646981.1|	Teucrium flavum subsp. glaucum isolate TFG54 tRNA-Leu (trnL) gene and trnL-trnF intergenic spacer, complete sequence; and tRNA-Phe (trnF) gene, partial sequence; chloroplast	708	708	83%	0.0	90%	HQ646981.1	767,71	80,7%

Select seq gb|GU331789.1|	Teucrium marum isolate TM15S tRNA-Leu (trnL) gene, partial sequence; trnL-trnF intergenic spacer, complete sequence; and tRNA-Phe (trnF) gene, partial sequence; chloroplast	708	708	83% 0.0	90%	GU331789.1	767,71	80,7%
Select seq gb|JN408611.1|	Teucrium chamaedrys tRNA-Leu (trnL) gene, partial sequence; and trnL-trnF intergenic spacer, complete sequence; chloroplast	706	706	83% 0.0	90%	JN408611.1	765,54	80,4%
Select seq gb|JN408660.1|	Teucrium pyrenaicum tRNA-Leu (trnL) gene, partial sequence; and trnL-trnF intergenic spacer, complete sequence; chloroplast	832	832	98% 0.0	90%	JN408660.1	764,08	80,3%
Select seq gb|JN408619.1|	Teucrium capitatum tRNA-Leu (trnL) gene, partial sequence; and trnL-trnF intergenic spacer, complete sequence; chloroplast	832	832	98% 0.0	90%	JN408619.1	764,08	80,3%
Select seq gb|JN408616.1|	Teucrium cyprium subsp. cyprium tRNA-Leu (trnL) gene, partial sequence; and trnL-trnF intergenic spacer, complete sequence; chloroplast	832	832	98% 0.0	90%	JN408616.1	764,08	80,3%
Select seq gb|JN408597.1|	Teucrium alpestre tRNA-Leu (trnL) gene, partial sequence; trnL-trnF intergenic spacer, complete sequence; and tRNA-Phe (trnF) gene, partial sequence; chloroplast	832	832	98% 0.0	90%	JN408597.1	764,08	80,3%
Select seq gb|FJ952080.1|	Teucrium pyrenaicum tRNA-Leu (trnL) gene, partial sequence; trnL-trnF intergenic spacer, complete sequence; and tRNA-Phe (trnF) gene, partial sequence; chloroplast	832	832	98% 0.0	90%	FJ952080.1	764,08	80,3%
Select seq gb|JN408635.1|	Teucrium intricatum tRNA-Leu (trnL) gene, partial sequence; and trnL-trnF intergenic spacer, complete sequence; chloroplast	702	702	83% 0.0	90%	JN408635.1	761,20	80,0%
Select seq gb|JN408593.1|	Teucrium flavum subsp. glaucum tRNA-Leu (trnL) gene, partial sequence; and trnL-trnF intergenic spacer, complete sequence; chloroplast	702	702	83% 0.0	90%	JN408593.1	761,20	80,0%
Select seq gb|HQ646991.1|	Teucrium flavum subsp. flavum isolate TFF56 tRNA-Leu (trnL) gene and trnL-trnF intergenic spacer, complete sequence; and tRNA-Phe (trnF) gene, partial sequence; chloroplast	702	702	83% 0.0	90%	HQ646991.1	761,20	80,0%
Select seq gb|GU331788.1|	Teucrium marum isolate TM25S tRNA-Leu (trnL) gene, partial sequence; trnL-trnF intergenic spacer, complete sequence; and tRNA-Phe (trnF) gene, partial sequence; chloroplast	702	702	83% 0.0	90%	GU331788.1	761,20	80,0%
Select seq gb|GU331783.1|	Teucrium marum isolate TM6C tRNA-Leu (trnL) gene, partial sequence; trnL-trnF intergenic spacer, complete sequence; and tRNA-Phe (trnF) gene, partial sequence; chloroplast	702	702	83% 0.0	90%	GU331783.1	761,20	80,0%
Select seq gb|JQ044776.1|	Teucrium polium subsp. polium isolate TP4 tRNA-Leu (trnL) gene, partial sequence; trnL-trnF intergenic spacer, complete sequence; and tRNA-Phe (trnF) gene, partial sequence; chloroplast	821	821	97% 0.0	89%	JQ044776.1	753,29	79,1%
Select seq gb|JN408617.1|	Teucrium decipiens tRNA-Leu (trnL) gene, partial sequence; and trnL-trnF intergenic spacer, complete sequence; chloroplast	710	710	85% 0.0	90%	JN408617.1	751,76	79,0%
Select seq gb|JN408620.1|	Teucrium aureum tRNA-Leu (trnL) gene, partial sequence; and trnL-trnF intergenic spacer, complete sequence; chloroplast	826	826	98% 0.0	89%	JN408620.1	750,14	78,8%
Select seq gb|JQ044781.1|	Teucrium polium subsp. polium isolate TP19 tRNA-Leu (trnL) gene, partial sequence; trnL-trnF intergenic spacer, complete sequence; and tRNA-Phe (trnF) gene, partial sequence; chloroplast	826	826	98% 0.0	89%	JQ044781.1	750,14	78,8%
Select seq gb|JQ044774.1|	Teucrium polium subsp. polium isolate TP20 tRNA-Leu (trnL) gene, partial sequence; trnL-trnF intergenic spacer, complete sequence; and tRNA-Phe (trnF) gene, partial sequence; chloroplast	826	826	98% 0.0	89%	JQ044774.1	750,14	78,8%
Select seq gb|KR150230.1|	Teucrium sp. 3 Kh19 trnL-trnF intergenic spacer region, partial sequence; chloroplast	791	791	94% 0.0	89%	KR150230.1	748,93	78,7%
Select seq gb|KR150221.1|	Teucrium sp. 1 Kh66 trnL-trnF intergenic spacer region, partial sequence; chloroplast	791	791	94% 0.0	89%	KR150221.1	748,93	78,7%
Select seq gb|JQ044782.1|	Teucrium polium subsp. polium isolate TP13 tRNA-Leu (trnL) gene, partial sequence; trnL-trnF intergenic spacer, complete sequence; and tRNA-Phe (trnF) gene, partial sequence; chloroplast	824	824	98% 0.0	89%	JQ044782.1	748,33	78,6%
Select seq gb|JQ044777.1|	Teucrium polium subsp. polium isolate TP5 tRNA-Leu (trnL) gene, partial sequence; trnL-trnF intergenic spacer, complete sequence; and tRNA-Phe (trnF) gene, partial sequence; chloroplast	821	821	98% 0.0	89%	JQ044777.1	745,60	78,3%
Select seq gb|JQ044775.1|	Teucrium polium subsp. polium isolate TP3 tRNA-Leu (trnL) gene, partial sequence; trnL-trnF intergenic spacer, complete sequence; and tRNA-Phe (trnF) gene, partial sequence; chloroplast	821	821	98% 0.0	89%	JQ044775.1	745,60	78,3%
Select seq gb|JN408663.1|	Teucrium resupinatum tRNA-Leu (trnL) gene, partial sequence; and trnL-trnF intergenic spacer, complete sequence; chloroplast	726	726	88% 0.0	89%	JN408663.1	734,25	77,1%
Select seq gb|JN408661.1|	Teucrium quadrifarium tRNA-Leu (trnL) gene, partial sequence; and trnL-trnF intergenic spacer, complete sequence; chloroplast	747	747	91% 0.0	89%	JN408661.1	730,58	76,8%
Select seq gb|JN408594.1|	Teucrium antitauricum tRNA-Leu (trnL) gene, partial sequence; and trnL-trnF intergenic spacer, complete sequence; chloroplast	771	771	94% 0.0	89%	JN408594.1	729,99	76,7%
Select seq gb|JN408647.1|	Teucrium montbretii subsp. heliotropiifolium tRNA-Leu (trnL) gene, partial sequence; and trnL-trnF intergenic spacer, complete sequence; chloroplast	743	743	91% 0.0	89%	JN408647.1	726,67	76,3%
Select seq gb|JN408665.1|	Teucrium rupestre tRNA-Leu (trnL) gene, partial sequence; and trnL-trnF intergenic spacer, complete sequence; chloroplast	737	737	91% 0.0	89%	JN408665.1	720,80	75,7%
Select seq gb|JN408631.1|	Teucrium halacsyanum tRNA-Leu (trnL) gene, partial sequence; and trnL-trnF intergenic spacer, complete sequence; chloroplast	752	752	93% 0.0	89%	JN408631.1	719,66	75,6%

Select seq gb|JN408633.1|	Teucrium hircanicum tRNA-Leu (trnL) gene, partial sequence; and trnL-trnF intergenic spacer, complete sequence; chloroplast	734	734	91% 0.0	89%	JN408633.1	717,87	75,4%
Select seq gb|JN408664.1|	Teucrium royleanum tRNA-Leu (trnL) gene, partial sequence; and trnL-trnF intergenic spacer, complete sequence; chloroplast	758	758	94% 0.0	89%	JN408664.1	717,68	75,4%
Select seq gb|JN408676.1|	Teucrium viscidum var. miquelianum tRNA-Leu (trnL) gene, partial sequence; and trnL-trnF intergenic spacer, complete sequence; chloroplast	749	749	93% 0.0	89%	JN408676.1	716,78	75,3%
Select seq gb|JN408636.1|	Teucrium japonicum tRNA-Leu (trnL) gene, partial sequence; and trnL-trnF intergenic spacer, complete sequence; chloroplast	756	756	94% 0.0	89%	JN408636.1	715,79	75,2%
Select seq gb|JN408672.1|	Teucrium lamiifolium subsp. stachyophyllum tRNA-Leu (trnL) gene, partial sequence; and trnL-trnF intergenic spacer, complete sequence; chloroplast	752	752	94% 0.0	89%	JN408672.1	712,00	74,8%
Select seq gb|JN408628.1|	Teucrium edelbergii tRNA-Leu (trnL) gene, partial sequence; and trnL-trnF intergenic spacer, complete sequence; chloroplast	752	752	94% 0.0	89%	JN408628.1	712,00	74,8%
Select seq gb|JN408618.1|	Teucrium demnatense tRNA-Leu (trnL) gene, partial sequence; and trnL-trnF intergenic spacer, complete sequence; chloroplast	750	750	94% 0.0	89%	JN408618.1	710,11	74,6%
Select seq gb|JN408649.1|	Teucrium odontites tRNA-Leu (trnL) gene, partial sequence; and trnL-trnF intergenic spacer, complete sequence; chloroplast	747	747	94% 0.0	88%	JN408649.1	699,32	73,5%
Select seq gb|JN408674.1|	Teucridium parvifolium tRNA-Leu (trnL) gene, partial sequence; trnL-trnF intergenic spacer, complete sequence; and tRNA-Phe (trnF) gene, partial sequence; chloroplast	741	741	94% 0.0	88%	JN408674.1	693,70	72,9%
Select seq gb|JN408598.1|	Teucrium arduinoi tRNA-Leu (trnL) gene, partial sequence; and trnL-trnF intergenic spacer, complete sequence; chloroplast	739	739	94% 0.0	88%	JN408598.1	691,83	72,7%
Select seq gb|JN408627.1|	Teucrium scorodonia tRNA-Leu (trnL) gene, partial sequence; and trnL-trnF intergenic spacer, complete sequence; chloroplast	713	713	91% 0.0	88%	JN408627.1	689,49	72,4%
Select seq gb|JN408600.1|	Teucrium cubense tRNA-Leu (trnL) gene, partial sequence; trnL-trnF intergenic spacer, complete sequence; and tRNA-Phe (trnF) gene, partial sequence; chloroplast	736	736	94% 0.0	88%	JN408600.1	689,02	72,4%
Select seq gb|JN408639.1|	Teucrium laciniatum tRNA-Leu (trnL) gene, partial sequence; and trnL-trnF intergenic spacer, complete sequence; chloroplast	719	719	92% 0.0	88%	JN408639.1	687,74	72,2%
Select seq gb|JN408640.1|	Teucrium lamiifolium subsp. lamiifolium tRNA-Leu (trnL) gene, partial sequence; and trnL-trnF intergenic spacer, complete sequence; chloroplast	734	734	94% 0.0	88%	JN408640.1	687,15	72,2%
Select seq gb|JN408602.1|	Teucrium aroanium tRNA-Leu (trnL) gene, partial sequence; and trnL-trnF intergenic spacer, complete sequence; chloroplast	701	701	90% 0.0	88%	JN408602.1	685,42	72,0%
Select seq gb|JN408595.1|	Teucrium abutiloides tRNA-Leu (trnL) gene, partial sequence; trnL-trnF intergenic spacer, complete sequence; and tRNA-Phe (trnF) gene, partial sequence; chloroplast	732	732	94% 0.0	88%	JN408595.1	685,28	72,0%
Select seq gb|JN408648.1|	Teucrium nudicaule tRNA-Leu (trnL) gene, partial sequence; and trnL-trnF intergenic spacer, complete sequence; chloroplast	730	730	94% 0.0	88%	JN408648.1	683,40	71,8%
Select seq gb|JN408605.1|	Teucrium bicolor tRNA-Leu (trnL) gene, partial sequence; and trnL-trnF intergenic spacer, complete sequence; chloroplast	730	730	94% 0.0	88%	JN408605.1	683,40	71,8%
Select seq gb|JN408651.1|	Teucrium orientale subsp. gloeotrichum tRNA-Leu (trnL) gene, partial sequence; and trnL-trnF intergenic spacer, complete sequence; chloroplast	706	706	91% 0.0	88%	JN408651.1	682,73	71,7%
Select seq gb|JN408669.1|	Teucrium scorodonia tRNA-Leu (trnL) gene, partial sequence; and trnL-trnF intergenic spacer, complete sequence; chloroplast	728	728	94% 0.0	88%	JN408669.1	681,53	71,6%
Select seq gb|JN408667.1|	Teucrium francoi tRNA-Leu (trnL) gene, partial sequence; and trnL-trnF intergenic spacer, complete sequence; chloroplast	728	728	94% 0.0	88%	JN408667.1	681,53	71,6%
Select seq gb|JN408666.1|	Teucrium salviastrum tRNA-Leu (trnL) gene, partial sequence; and trnL-trnF intergenic spacer, complete sequence; chloroplast	728	728	94% 0.0	88%	JN408666.1	681,53	71,6%
Select seq gb|JN408643.1|	Teucrium massiliense tRNA-Leu (trnL) gene, partial sequence; and trnL-trnF intergenic spacer, complete sequence; chloroplast	728	728	94% 0.0	88%	JN408643.1	681,53	71,6%
Select seq gb|JN408603.1|	Teucrium asiaticum tRNA-Leu (trnL) gene, partial sequence; and trnL-trnF intergenic spacer, complete sequence; chloroplast	728	728	94% 0.0	88%	JN408603.1	681,53	71,6%
Select seq gb|JF694866.1|	Teucrium scorodonia isolate TSC8 tRNA-Leu (trnL) gene and trnL-trnF intergenic spacer, complete sequence; and tRNA-Phe (trnF) gene, partial sequence; plastid	728	728	94% 0.0	88%	JF694866.1	681,53	71,6%
Select seq gb|JF694864.1|	Teucrium scorodonia isolate TSC6 tRNA-Leu (trnL) gene and trnL-trnF intergenic spacer, complete sequence; and tRNA-Phe (trnF) gene, partial sequence; plastid	728	728	94% 0.0	88%	JF694864.1	681,53	71,6%
Select seq gb|JF694861.1|	Teucrium scorodonia isolate TSC3 tRNA-Leu (trnL) gene and trnL-trnF intergenic spacer, complete sequence; and tRNA-Phe (trnF) gene, partial sequence; plastid	728	728	94% 0.0	88%	JF694861.1	681,53	71,6%
Select seq gb|JF694859.1|	Teucrium scorodonia isolate TSC1 tRNA-Leu (trnL) gene and trnL-trnF intergenic spacer, complete sequence; and tRNA-Phe (trnF) gene, partial sequence; plastid	728	728	94% 0.0	88%	JF694859.1	681,53	71,6%

Select seq gb|JN408675.1|	Teucrium trifidum tRNA-Leu (trnL) gene, partial sequence; and trnL-trnF intergenic spacer, complete sequence; chloroplast	725	725	94%	0.0	88% JN408675.1	678,72	71,3%
Select seq gb|JN408610.1|	Teucrium trifidum tRNA-Leu (trnL) gene, partial sequence; and trnL-trnF intergenic spacer, complete sequence; chloroplast	725	725	94%	0.0	88% JN408610.1	678,72	71,3%
Select seq gb|JN408650.1|	Teucrium oliverianum tRNA-Leu (trnL) gene, partial sequence; and trnL-trnF intergenic spacer, complete sequence; chloroplast	715	715	93%	0.0	88% JN408650.1	676,56	71,1%
Select seq gb|JN408658.1|	Teucrium pruinosum tRNA-Leu (trnL) gene, partial sequence; and trnL-trnF intergenic spacer, complete sequence; chloroplast	719	719	94%	0.0	88% JN408658.1	673,11	70,7%
Select seq gb|GU331791.1|	Teucrium marum isolate TM16W tRNA-Leu (trnL) gene, partial sequence; trnL-trnF intergenic spacer, complete sequence; and tRNA-Phe (trnF) gene, partial sequence; chloroplast	717	717	94%	0.0	88% GU331791.1	671,23	70,5%
Select seq gb|JN408591.1|	Spartothamnella puberula tRNA-Leu (trnL) gene, partial sequence; trnL-trnF intergenic spacer, complete sequence; and tRNA-Phe (trnF) gene, partial sequence; chloroplast	715	715	94%	0.0	88% JN408591.1	669,36	70,3%
Select seq gb|JN408673.1|	Teucrium subspinosum tRNA-Leu (trnL) gene, partial sequence; and trnL-trnF intergenic spacer, complete sequence; chloroplast	712	712	94%	0.0	88% JN408673.1	666,55	70,0%
Select seq gb|JN408642.1|	Teucrium marum tRNA-Leu (trnL) gene, partial sequence; and trnL-trnF intergenic spacer, complete sequence; chloroplast	712	712	94%	0.0	88% JN408642.1	666,55	70,0%
Select seq gb|GU331790.1|	Teucrium marum isolate TM23W tRNA-Leu (trnL) gene, partial sequence; trnL-trnF intergenic spacer, complete sequence; and tRNA-Phe (trnF) gene, partial sequence; chloroplast	712	712	94%	0.0	88% GU331790.1	666,55	70,0%
Select seq gb|GU331787.1|	Teucrium marum isolate TM12N tRNA-Leu (trnL) gene, partial sequence; trnL-trnF intergenic spacer, complete sequence; and tRNA-Phe (trnF) gene, partial sequence; chloroplast	712	712	94%	0.0	88% GU331787.1	666,55	70,0%
Select seq gb|GU331782.1|	Teucrium marum isolate TM1C tRNA-Leu (trnL) gene, partial sequence; trnL-trnF intergenic spacer, complete sequence; and tRNA-Phe (trnF) gene, partial sequence; chloroplast	712	712	94%	0.0	88% GU331782.1	666,55	70,0%
Select seq gb|JN408590.1|	Oncinocalyx betchei tRNA-Leu (trnL) gene, partial sequence; trnL-trnF intergenic spacer, complete sequence; and tRNA-Phe (trnF) gene, partial sequence; chloroplast	710	710	94%	0.0	88% JN408590.1	664,68	69,8%
Select seq gb|GU331786.1|	Teucrium marum isolate TM5N tRNA-Leu (trnL) gene, partial sequence; trnL-trnF intergenic spacer, complete sequence; and tRNA-Phe (trnF) gene, partial sequence; chloroplast	701	701	94%	0.0	87% GU331786.1	648,80	68,2%
Select seq gb|GU331785.1|	Teucrium marum isolate TM20C tRNA-Leu (trnL) gene, partial sequence; trnL-trnF intergenic spacer, complete sequence; and tRNA-Phe (trnF) gene, partial sequence; chloroplast	701	701	94%	0.0	87% GU331785.1	648,80	68,2%
Select seq gb|GU331784.1|	Teucrium marum isolate TM8C tRNA-Leu (trnL) gene, partial sequence; trnL-trnF intergenic spacer, complete sequence; and tRNA-Phe (trnF) gene, partial sequence; chloroplast	701	701	94%	0.0	87% GU331784.1	648,80	68,2%
Select seq gb|JN408637.1|	Teucrium jolyi tRNA-Leu (trnL) gene, partial sequence; and trnL-trnF intergenic spacer, complete sequence; chloroplast	701	701	96%	0.0	87% JN408637.1	635,28	66,7%
							0,00	

Select for downloading or viewing reports	Kh024_ITS Description	Max score	Total score	Query cover	E value	Ident	Accession	(Ident/Cover)* Max score	Deviation from top hit
Select seq gb|EU735059.1|	Thymus trautvetteri 18S ribosomal RNA gene, partial sequence; internal transcribed spacer 1, 5.8S ribosomal RNA gene, and internal transcribed spacer 2, complete sequence; and 28S ribosomal RNA gene, partial sequence	1110	1110	100%	0.0	100%	EU735059.1	1110,00	100,0%
Select seq gb|EU735058.1|	Thymus persicus isolate Ardabil 18S ribosomal RNA gene, partial sequence; internal transcribed spacer 1, 5.8S ribosomal RNA gene, and internal transcribed spacer 2, complete sequence; and 28S ribosomal RNA gene, partial sequence	1110	1110	100%	0.0	100%	EU735058.1	1110,00	100,0%
Select seq gb|EU374715.1|	Thymus pubescens 18S ribosomal RNA gene, partial sequence; internal transcribed spacer 1, 5.8S ribosomal RNA gene, and internal transcribed spacer 2, complete sequence; and 25/28S ribosomal RNA gene, partial sequence	1110	1110	100%	0.0	100%	EU374715.1	1110,00	100,0%
Select seq gb|GU381459.1|	Thymus serpyllum voucher M:Braeuchler 2514 internal transcribed spacer 1, partial sequence; 5.8S ribosomal RNA gene, complete sequence; and internal transcribed spacer 2, partial sequence	1088	1088	99%	0.0	99%	GU381459.1	1088,00	98,0%
Select seq gb|EU556510.1|	Thymus mongolicus isolate G3 internal transcribed spacer 1, partial sequence; 5.8S ribosomal RNA gene, complete sequence; and internal transcribed spacer 2, partial sequence	1062	1062	97%	0.0	99%	EU556510.1	1083,90	97,6%
Select seq gb|EU556521.1|	Thymus mongolicus isolate XM internal transcribed spacer 1, partial sequence; 5.8S ribosomal RNA gene, complete sequence; and internal transcribed spacer 2, partial sequence	1057	1057	97%	0.0	99%	EU556521.1	1078,79	97,2%
Select seq gb|EU556524.1|	Thymus quinquecostatus isolate ZY internal transcribed spacer 1, partial sequence; 5.8S ribosomal RNA gene, complete sequence; and internal transcribed spacer 2, partial sequence	1088	1088	100%	0.0	99%	EU556524.1	1077,12	97,0%
Select seq gb|EU556522.1|	Thymus quinquecostatus isolate ZJ internal transcribed spacer 1, partial sequence; 5.8S ribosomal RNA gene, complete sequence; and internal transcribed spacer 2, partial sequence	1088	1088	100%	0.0	99%	EU556522.1	1077,12	97,0%
Select seq gb|EU556520.1|	Thymus quinquecostatus isolate XZ internal transcribed spacer 1, partial sequence; 5.8S ribosomal RNA gene, complete sequence; and internal transcribed spacer 2, partial sequence	1088	1088	100%	0.0	99%	EU556520.1	1077,12	97,0%
Select seq gb|EU556519.1|	Thymus mongolicus isolate S2 internal transcribed spacer 1, partial sequence; 5.8S ribosomal RNA gene, complete sequence; and internal transcribed spacer 2, partial sequence	1088	1088	100%	0.0	99%	EU556519.1	1077,12	97,0%
Select seq gb|EU556518.1|	Thymus mongolicus isolate S1 internal transcribed spacer 1, partial sequence; 5.8S ribosomal RNA gene, complete sequence; and internal transcribed spacer 2, partial sequence	1088	1088	100%	0.0	99%	EU556518.1	1077,12	97,0%
Select seq gb|EU556517.1|	Thymus quinquecostatus isolate N2 internal transcribed spacer 1, partial sequence; 5.8S ribosomal RNA gene, complete sequence; and internal transcribed spacer 2, partial sequence	1088	1088	100%	0.0	99%	EU556517.1	1077,12	97,0%
Select seq gb|EU556516.1|	Thymus quinquecostatus isolate N1 internal transcribed spacer 1, partial sequence; 5.8S ribosomal RNA gene, complete sequence; and internal transcribed spacer 2, partial sequence	1088	1088	100%	0.0	99%	EU556516.1	1077,12	97,0%
Select seq gb|EU556511.1|	Thymus dahuricus isolate HD internal transcribed spacer 1, partial sequence; 5.8S ribosomal RNA gene, complete sequence; and internal transcribed spacer 2, partial sequence	1088	1088	100%	0.0	99%	EU556511.1	1077,12	97,0%
Select seq gb|EU556509.1|	Thymus mongolicus isolate G2 internal transcribed spacer 1, partial sequence; 5.8S ribosomal RNA gene, complete sequence; and internal transcribed spacer 2, partial sequence	1088	1088	100%	0.0	99%	EU556509.1	1077,12	97,0%
Select seq gb|EU556508.1|	Thymus mongolicus isolate G1 internal transcribed spacer 1, partial sequence; 5.8S ribosomal RNA gene, complete sequence; and internal transcribed spacer 2, partial sequence	1088	1088	100%	0.0	99%	EU556508.1	1077,12	97,0%
Select seq gb|EU556506.1|	Thymus quinquecostatus isolate D1 internal transcribed spacer 1, partial sequence; 5.8S ribosomal RNA gene, complete sequence; and internal transcribed spacer 2, partial sequence	1088	1088	100%	0.0	99%	EU556506.1	1077,12	97,0%
Select seq gb|FJ236468.1|	Thymus persicus isolate Tabriz 18S ribosomal RNA gene, partial sequence; internal transcribed spacer 1, 5.8S ribosomal RNA gene, and internal transcribed spacer 2, complete sequence; and 28S ribosomal RNA gene, partial sequence	1088	1088	100%	0.0	99%	FJ236468.1	1077,12	97,0%
Select seq gb|DQ667242.1|	Thymus serpyllum isolate x075 18S ribosomal RNA gene, partial sequence; internal transcribed spacer 1, 5.8S ribosomal RNA gene, and internal transcribed spacer 2, complete sequence; and 28S ribosomal RNA gene, partial sequence	1088	1088	100%	0.0	99%	DQ667242.1	1077,12	97,0%
Select seq gb|AY443448.1|	Thymus magnus JUNG-TM04 internal transcribed spacer 1, 5.8S ribosomal RNA and internal transcribed spcer 2 genes, complete sequence	1088	1088	100%	0.0	99%	AY443448.1	1077,12	97,0%
Select seq gb|AY443434.1|	Thymus quinquecostatus JUNG-TQ01 internal transcribed spacer 1, 5.8S ribosomal RNA and internal transcribed spcer 2 genes, complete sequence	1088	1088	100%	0.0	99%	AY443434.1	1077,12	97,0%
Select seq gb|AY029168.1|	Thymus mastichina internal transcribed spacer 1, 5.8S ribosomal RNA gene, and internal transcribed spacer 2, complete sequence	1088	1088	100%	0.0	99%	AY029168.1	1077,12	97,0%
Select seq gb|EU556507.1|	Thymus quinquecostatus isolate CL internal transcribed spacer 1, partial sequence; 5.8S ribosomal RNA gene, complete sequence; and internal transcribed spacer 2, partial sequence	1083	1083	100%	0.0	99%	EU556507.1	1072,17	96,6%
Select seq gb|AY443445.1|	Thymus magnus JUNG-TM01 internal transcribed spacer 1, 5.8S ribosomal RNA and internal transcribed spcer 2 genes, complete sequence	1083	1083	100%	0.0	99%	AY443445.1	1072,17	96,6%
Select seq gb|AY443442.1|	Thymus quinquecostatus JUNG-TQ09 internal transcribed spacer 1, 5.8S ribosomal RNA and internal transcribed spcer 2 genes, complete sequence	1072	1072	99%	0.0	99%	AY443442.1	1072,00	96,6%
Select seq gb|EU796890.1|	Thymus serpyllum 18S ribosomal RNA gene, partial sequence; internal transcribed spacer 1, 5.8S ribosomal RNA gene, and internal transcribed spacer 2, complete sequence; and 26S ribosomal RNA gene, partial sequence	1081	1081	100%	0.0	99%	EU796890.1	1070,19	96,4%

Select seq gb|EU556523.1|	Thymus marschallianus isolate XY internal transcribed spacer 1, partial sequence; 5.8S ribosomal RNA gene, complete sequence; and internal transcribed spacer 2, partial sequence	1070	1070	99% 0.0	99%	EU556523.1	1070,00	96,4%
Select seq gb|JQ669138.1|	Thymus pulegioides voucher Riina 1577 18S ribosomal RNA gene, internal transcribed spacer 1, 5.8S ribosomal RNA gene, internal transcribed spacer 2, and 26S ribosomal RNA gene, region	1079	1079	100% 0.0	99%	JQ669138.1	1068,21	96,2%
Select seq gb|AY443447.1|	Thymus magnus JUNG-TM03 internal transcribed spacer 1, 5.8S ribosomal RNA and internal transcribed spcer 2 genes, complete sequence	1077	1077	100% 0.0	99%	AY443447.1	1066,23	96,1%
Select seq gb|AY443441.1|	Thymus quinquecostatus JUNG-TQ08 internal transcribed spacer 1, 5.8S ribosomal RNA and internal transcribed spcer 2 genes, complete sequence	1077	1077	100% 0.0	99%	AY443441.1	1066,23	96,1%
Select seq gb|AY443438.1|	Thymus quinquecostatus JUNG-TQ05 internal transcribed spacer 1, 5.8S ribosomal RNA and internal transcribed spcer 2 genes, complete sequence	1077	1077	100% 0.0	99%	AY443438.1	1066,23	96,1%
Select seq gb|EU785939.1|	Thymus vulgaris 18S ribosomal RNA gene, partial sequence; internal transcribed spacer 1, 5.8S ribosomal RNA gene, and internal transcribed spacer 2, complete sequence; and 26S ribosomal RNA gene, partial sequence	1074	1074	100% 0.0	99%	EU785939.1	1063,26	95,8%
Select seq gb|AY443444.1|	Thymus quinquecostatus JUNG-TQ11 internal transcribed spacer 1, 5.8S ribosomal RNA and internal transcribed spcer 2 genes, complete sequence	1072	1072	100% 0.0	99%	AY443444.1	1061,28	95,6%
Select seq gb|AY443443.1|	Thymus quinquecostatus JUNG-TQ10 internal transcribed spacer 1, 5.8S ribosomal RNA and internal transcribed spcer 2 genes, complete sequence	1072	1072	100% 0.0	99%	AY443443.1	1061,28	95,6%
Select seq gb|AY443437.1|	Thymus quinquecostatus JUNG-TQ04 internal transcribed spacer 1, 5.8S ribosomal RNA and internal transcribed spcer 2 genes, complete sequence	1072	1072	100% 0.0	99%	AY443437.1	1061,28	95,6%
Select seq gb|AY443436.1|	Thymus quinquecostatus JUNG-TQ03 internal transcribed spacer 1, 5.8S ribosomal RNA and internal transcribed spcer 2 genes, complete sequence	1072	1072	100% 0.0	99%	AY443436.1	1061,28	95,6%
Select seq gb|GU381458.1|	Thymus broussonetii subsp. hannonis voucher MSB:Podlech 142 internal transcribed spacer 1, partial sequence; 5.8S ribosomal RNA gene, complete sequence; and internal transcribed spacer 2, partial sequence	1061	1061	99% 0.0	99%	GU381458.1	1061,00	95,6%
Select seq gb|AY443446.1|	Thymus magnus JUNG-TM02 internal transcribed spacer 1, 5.8S ribosomal RNA and internal transcribed spcer 2 genes, complete sequence	1061	1061	100% 0.0	99%	AY443446.1	1050,39	94,6%
Select seq gb|AY443440.1|	Thymus quinquecostatus JUNG-TQ07 internal transcribed spacer 1, 5.8S ribosomal RNA and internal transcribed spcer 2 genes, complete sequence	1061	1061	100% 0.0	99%	AY443440.1	1050,39	94,6%
Select seq gb|AY443435.1|	Thymus quinquecostatus JUNG-TQ02 internal transcribed spacer 1, 5.8S ribosomal RNA and internal transcribed spcer 2 genes, complete sequence	1055	1055	100% 0.0	98%	AY443435.1	1033,90	93,1%
Select seq gb|EU556515.1|	Thymus amurensis isolate HW internal transcribed spacer 1, partial sequence; 5.8S ribosomal RNA gene, complete sequence; and internal transcribed spacer 2, partial sequence	1016	1016	99% 0.0	98%	EU556515.1	1005,74	90,6%
Select seq gb|EU556512.1|	Thymus mandschuricus isolate HM internal transcribed spacer 1, partial sequence; 5.8S ribosomal RNA gene, complete sequence; and internal transcribed spacer 2, partial sequence	1024	1024	100% 0.0	98%	EU556512.1	1003,52	90,4%
Select seq gb|AY329369.1|	Thymus vulgaris internal transcribed spacer 1, partial sequence; 5.8S ribosomal RNA gene, complete sequence; and internal transcribed spacer 2, partial sequence	1018	1018	100% 0.0	97%	AY329369.1	987,46	89,0%
Select seq gb|AY506646.1|	Thymus vulgaris internal transcribed spacer 1, partial sequence; 5.8S ribosomal RNA gene, complete sequence; and internal transcribed spacer 2, partial sequence	1014	1014	100% 0.0	97%	AY506646.1	983,58	88,6%
Select seq gb|EU556514.1|	Thymus quinquecostatus isolate HY internal transcribed spacer 1, partial sequence; 5.8S ribosomal RNA gene, complete sequence; and internal transcribed spacer 2, partial sequence	915	915	93% 0.0	96%	EU556514.1	944,52	85,1%
Select seq gb|EU556513.1|	Thymus przewalskii isolate HX internal transcribed spacer 1, partial sequence; 5.8S ribosomal RNA gene, complete sequence; and internal transcribed spacer 2, partial sequence	920	920	94% 0.0	96%	EU556513.1	939,57	84,6%
Select seq gb|GU381460.1|	Thymus saturejoides subsp. commutatus voucher M:Podlech 47906 internal transcribed spacer 1, partial sequence; 5.8S ribosomal RNA gene, complete sequence; and internal transcribed spacer 2, partial sequence	944	944	99% 0.0	95%	GU381460.1	905,86	81,6%
Select seq gb|GU381462.1|	Saccocalyx saturejoides voucher MSB:Faurel 5650 internal transcribed spacer 1, partial sequence; 5.8S ribosomal RNA gene, complete sequence; and internal transcribed spacer 2, partial sequence	933	933	99% 0.0	95%	GU381462.1	895,30	80,7%
Select seq gb|GU381457.1|	Thymus caespititius voucher M:Heubl s.n. internal transcribed spacer 1, partial sequence; 5.8S ribosomal RNA gene, complete sequence; and internal transcribed spacer 2, partial sequence	933	933	99% 0.0	95%	GU381457.1	895,30	80,7%
Select seq gb|GU381461.1|	Argantoniella salzmannii voucher M:Barra et al. 2673GL internal transcribed spacer 1, partial sequence; 5.8S ribosomal RNA gene, complete sequence; and internal transcribed spacer 2, partial sequence	928	928	99% 0.0	95%	GU381461.1	890,51	80,2%
Select seq gb|GU381451.1|	Thymbra sintenisii subsp. isaurica voucher E:Goener 12628 internal transcribed spacer 1, partial sequence; 5.8S ribosomal RNA gene, complete sequence; and internal transcribed spacer 2, partial sequence	900	900	99% 0.0	94%	GU381451.1	854,55	77,0%
Select seq gb|GU381452.1|	Thymbra calostachya voucher M:Ulrich s.n. internal transcribed spacer 1, partial sequence; 5.8S ribosomal RNA gene, complete sequence; and internal transcribed spacer 2, partial sequence	894	894	99% 0.0	94%	GU381452.1	848,85	76,5%
Select seq gb|GU381455.1|	Satureja linearifolia voucher M:Brullo & Furnari s.n. internal transcribed spacer 1, partial sequence; 5.8S ribosomal RNA gene, complete sequence; and internal transcribed spacer 2, partial sequence	893	893	99% 0.0	94%	GU381455.1	847,90	76,4%
Select seq gb|JQ669137.1|	Thymbra capitata voucher UCBG 96.0817 s.n. 18S ribosomal RNA gene, internal transcribed spacer 1, 5.8S ribosomal RNA gene, internal transcribed spacer 2, and 26S ribosomal RNA gene, region	889	889	99% 0.0	94%	JQ669137.1	844,10	76,0%

Select seq gb|GU381454.1|	Satureja thymbrifolia voucher M:Danin s.n. internal transcribed spacer 1, partial sequence; 5.8S ribosomal RNA gene, complete sequence; and internal transcribed spacer 2, partial sequence	887	887	99% 0.0	94% GU381454.1	842,20	75,9%
Select seq gb|GU381453.1|	Thymbra capitata voucher M:Braeuchler 2518 internal transcribed spacer 1, partial sequence; 5.8S ribosomal RNA gene, complete sequence; and internal transcribed spacer 2, partial sequence	881	881	99% 0.0	93% GU381453.1	827,61	74,6%
Select seq gb|GU381466.1|	Origanum dayi voucher MSB:Liston 7-85-3931 internal transcribed spacer 1, partial sequence; 5.8S ribosomal RNA gene, complete sequence; and internal transcribed spacer 2, partial sequence	872	872	99% 0.0	93% GU381466.1	819,15	73,8%
Select seq gb|JQ669127.1|	Origanum vulgare voucher B. Drew 77 18S ribosomal RNA gene, internal transcribed spacer 1, 5.8S ribosomal RNA gene, internal transcribed spacer 2, and 26S ribosomal RNA gene, region	870	870	99% 0.0	93% JQ669127.1	817,27	73,6%
Select seq gb|GU381469.1|	Origanum vulgare voucher M:Braeuchler 3131 internal transcribed spacer 1, partial sequence; 5.8S ribosomal RNA gene, complete sequence; and internal transcribed spacer 2, partial sequence	865	865	99% 0.0	93% GU381469.1	812,58	73,2%
Select seq gb|GU381463.1|	Origanum rotundifolium voucher M:Braeuchler 2517 internal transcribed spacer 1, partial sequence; 5.8S ribosomal RNA gene, complete sequence; and internal transcribed spacer 2, partial sequence	865	865	99% 0.0	93% GU381463.1	812,58	73,2%
Select seq gb|GU381468.1|	Origanum vulgare voucher M:Braeuchler 2512 internal transcribed spacer 1, partial sequence; 5.8S ribosomal RNA gene, complete sequence; and internal transcribed spacer 2, partial sequence	861	861	99% 0.0	93% GU381468.1	808,82	72,9%
Select seq gb|JX162834.1|	Origanum majorana voucher OBI clone 3 internal transcribed spacer 1, partial sequence; 5.8S ribosomal RNA gene, complete sequence; and internal transcribed spacer 2, partial sequence	859	859	99% 0.0	93% JX162834.1	806,94	72,7%
Select seq gb|JX162831.1|	Origanum majorana voucher Mira2-3 clone 10 internal transcribed spacer 1, partial sequence; 5.8S ribosomal RNA gene, complete sequence; and internal transcribed spacer 2, partial sequence	859	859	99% 0.0	93% JX162831.1	806,94	72,7%
Select seq gb|GU381464.1|	Origanum dictamnus voucher M:Braeuchler 2519 internal transcribed spacer 1, partial sequence; 5.8S ribosomal RNA gene, complete sequence; and internal transcribed spacer 2, partial sequence	859	859	99% 0.0	93% GU381464.1	806,94	72,7%
Select seq gb|JX162829.1|	Origanum majorana voucher Mira2-3 clone 8 internal transcribed spacer 1, partial sequence; 5.8S ribosomal RNA gene, complete sequence; and internal transcribed spacer 2, partial sequence	848	848	98% 0.0	93% JX162829.1	804,73	72,5%
Select seq gb|JX163036.1|	Origanum syriacum voucher LC7 clone 8 internal transcribed spacer 1, partial sequence; 5.8S ribosomal RNA gene, complete sequence; and internal transcribed spacer 2, partial sequence	856	856	99% 0.0	93% JX163036.1	804,12	72,4%
Select seq gb|JX162836.1|	Origanum majorana voucher OBI clone 5 internal transcribed spacer 1, partial sequence; 5.8S ribosomal RNA gene, complete sequence; and internal transcribed spacer 2, partial sequence	856	856	99% 0.0	93% JX162836.1	804,12	72,4%
Select seq gb|DQ667243.1|	Origanum vulgare isolate x076 18S ribosomal RNA gene, partial sequence; internal transcribed spacer 1, 5.8S ribosomal RNA gene, and internal transcribed spacer 2, complete sequence; and 28S ribosomal RNA gene, partial sequence	856	856	99% 0.0	93% DQ667243.1	804,12	72,4%
Select seq gb|JX162837.1|	Origanum majorana voucher OBI clone 6 internal transcribed spacer 1, partial sequence; 5.8S ribosomal RNA gene, complete sequence; and internal transcribed spacer 2, partial sequence	854	854	99% 0.0	93% JX162837.1	802,24	72,3%
Select seq gb|JX162826.1|	Origanum majorana voucher Mira2-3 clone 4 internal transcribed spacer 1, partial sequence; 5.8S ribosomal RNA gene, complete sequence; and internal transcribed spacer 2, partial sequence	854	854	99% 0.0	93% JX162826.1	802,24	72,3%
Select seq gb|JX162816.1|	Origanum majorana voucher SR562 clone 3 internal transcribed spacer 1, partial sequence; 5.8S ribosomal RNA gene, complete sequence; and internal transcribed spacer 2, partial sequence	854	854	99% 0.0	93% JX162816.1	802,24	72,3%
Select seq gb|JX162814.1|	Origanum majorana voucher SR562 clone 1 internal transcribed spacer 1, partial sequence; 5.8S ribosomal RNA gene, complete sequence; and internal transcribed spacer 2, partial sequence	854	854	99% 0.0	93% JX162814.1	802,24	72,3%
Select seq gb|JX162807.1|	Origanum majorana voucher LC10 clone 4 internal transcribed spacer 1, partial sequence; 5.8S ribosomal RNA gene, complete sequence; and internal transcribed spacer 2, partial sequence	854	854	99% 0.0	93% JX162807.1	802,24	72,3%
Select seq gb|JX162805.1|	Origanum majorana voucher LC10 clone 2 internal transcribed spacer 1, partial sequence; 5.8S ribosomal RNA gene, complete sequence; and internal transcribed spacer 2, partial sequence	854	854	99% 0.0	93% JX162805.1	802,24	72,3%
Select seq gb|GU381465.1|	Origanum elongatum voucher MSB:Podlech 46949 internal transcribed spacer 1, partial sequence; 5.8S ribosomal RNA gene, complete sequence; and internal transcribed spacer 2, partial sequence	854	854	99% 0.0	93% GU381465.1	802,24	72,3%
Select seq gb|JX163024.1|	Origanum syriacum voucher RNG:H50 clone 16 internal transcribed spacer 1, partial sequence; 5.8S ribosomal RNA gene, complete sequence; and internal transcribed spacer 2, partial sequence	850	850	99% 0.0	92% JX163024.1	789,90	71,2%
Select seq gb|JX163023.1|	Origanum syriacum voucher RNG:H50 clone 15 internal transcribed spacer 1, partial sequence; 5.8S ribosomal RNA gene, complete sequence; and internal transcribed spacer 2, partial sequence	850	850	99% 0.0	92% JX163023.1	789,90	71,2%
Select seq gb|JX163021.1|	Origanum syriacum voucher RNG:H50 clone 13 internal transcribed spacer 1, partial sequence; 5.8S ribosomal RNA gene, complete sequence; and internal transcribed spacer 2, partial sequence	850	850	99% 0.0	92% JX163021.1	789,90	71,2%
Select seq gb|JX163010.1|	Origanum syriacum voucher RNG:H50 clone 2 internal transcribed spacer 1, partial sequence; 5.8S ribosomal RNA gene, complete sequence; and internal transcribed spacer 2, partial sequence	850	850	99% 0.0	92% JX163010.1	789,90	71,2%
Select seq gb|JX162830.1|	Origanum majorana voucher Mira2-3 clone 9 internal transcribed spacer 1, partial sequence; 5.8S ribosomal RNA gene, complete sequence; and internal transcribed spacer 2, partial sequence	850	850	99% 0.0	92% JX162830.1	789,90	71,2%
Select seq gb|GU381467.1|	Origanum microphyllum voucher M:Braeuchler 3402 internal transcribed spacer 1, partial sequence; 5.8S ribosomal RNA gene, complete sequence; and internal transcribed spacer 2, partial sequence	850	850	99% 0.0	92% GU381467.1	789,90	71,2%
Select seq gb|JX163019.1|	Origanum syriacum voucher RNG:H50 clone 11 internal transcribed spacer 1, partial sequence; 5.8S ribosomal RNA gene, complete sequence; and internal transcribed spacer 2, partial sequence	848	848	99% 0.0	92% JX163019.1	788,04	71,0%

Select seq gb|JX163018.1|	Origanum syriacum voucher RNG:H50 clone 10 internal transcribed spacer 1, partial sequence; 5.8S ribosomal RNA gene, complete sequence; and internal transcribed spacer 2, partial sequence	848	848	99% 0.0	92% JX163018.1	788,04	71,0%
Select seq gb|JX163017.1|	Origanum syriacum voucher RNG:H50 clone 9 internal transcribed spacer 1, partial sequence; 5.8S ribosomal RNA gene, complete sequence; and internal transcribed spacer 2, partial sequence	848	848	99% 0.0	92% JX163017.1	788,04	71,0%
Select seq gb|JX163016.1|	Origanum syriacum voucher RNG:H50 clone 8 internal transcribed spacer 1, partial sequence; 5.8S ribosomal RNA gene, complete sequence; and internal transcribed spacer 2, partial sequence	848	848	99% 0.0	92% JX163016.1	788,04	71,0%
Select seq gb|JX162980.1|	Origanum onites voucher SR777 clone 14 internal transcribed spacer 1, partial sequence; 5.8S ribosomal RNA gene, complete sequence; and internal transcribed spacer 2, partial sequence	848	848	99% 0.0	92% JX162980.1	788,04	71,0%
Select seq gb|JX162832.1|	Origanum majorana voucher OBI clone 1 internal transcribed spacer 1, partial sequence; 5.8S ribosomal RNA gene, complete sequence; and internal transcribed spacer 2, partial sequence	848	848	99% 0.0	92% JX162832.1	788,04	71,0%
Select seq gb|JX162823.1|	Origanum majorana voucher Mira2-3 clone 1 internal transcribed spacer 1, partial sequence; 5.8S ribosomal RNA gene, complete sequence; and internal transcribed spacer 2, partial sequence	848	848	99% 0.0	92% JX162823.1	788,04	71,0%
Select seq gb|JX162818.1|	Origanum majorana voucher SR562 clone 5 internal transcribed spacer 1, partial sequence; 5.8S ribosomal RNA gene, complete sequence; and internal transcribed spacer 2, partial sequence	848	848	99% 0.0	92% JX162818.1	788,04	71,0%
Select seq gb|JX162815.1|	Origanum majorana voucher SR562 clone 2 internal transcribed spacer 1, partial sequence; 5.8S ribosomal RNA gene, complete sequence; and internal transcribed spacer 2, partial sequence	848	848	99% 0.0	92% JX162815.1	788,04	71,0%
Select seq gb|AY506647.1|	Origanum vulgare internal transcribed spacer 1, partial sequence; 5.8S ribosomal RNA gene, complete sequence; and internal transcribed spacer 2, partial sequence	846	846	99% 0.0	92% AY506647.1	786,18	70,8%
Select seq gb|JX163022.1|	Origanum syriacum voucher RNG:H50 clone 14 internal transcribed spacer 1, partial sequence; 5.8S ribosomal RNA gene, complete sequence; and internal transcribed spacer 2, partial sequence	845	845	99% 0.0	92% JX163022.1	785,25	70,7%
Select seq gb|JX163014.1|	Origanum syriacum voucher RNG:H50 clone 6 internal transcribed spacer 1, partial sequence; 5.8S ribosomal RNA gene, complete sequence; and internal transcribed spacer 2, partial sequence	845	845	99% 0.0	92% JX163014.1	785,25	70,7%
Select seq gb|JX162824.1|	Origanum majorana voucher Mira2-3 clone 2 internal transcribed spacer 1, partial sequence; 5.8S ribosomal RNA gene, complete sequence; and internal transcribed spacer 2, partial sequence	845	845	99% 0.0	92% JX162824.1	785,25	70,7%
Select seq gb|JX163012.1|	Origanum syriacum voucher RNG:H50 clone 4 internal transcribed spacer 1, partial sequence; 5.8S ribosomal RNA gene, complete sequence; and internal transcribed spacer 2, partial sequence	843	843	99% 0.0	92% JX163012.1	783,39	70,6%
						0,00	0,0%
						0,00	0,0%
						0,00	0,0%
						0,00	0,0%

Select for downloading or viewing reports	Kh024_trnL Description	Max score	Total score	Query cover	E value	Ident	Accession	(Ident/Cover) *Max score	Deviation from top hit
Select seq gb|AY506613.1|	Thymus vulgaris tRNA-Leu (trnL) gene and trnL-trnF intergenic spacer, partial sequence; chloroplast	1177	1177	95%	0.0	99%	AY506613.1	1226,56	100,0%
Select seq gb|GU381635.1|	Thymus broussonetii subsp. hannonis voucher MSB:Podlech 142 tRNA-Leu (trnL) gene and trnL-trnF intergenic spacer, partial sequence; chloroplast	1210	1210	98%	0.0	99%	GU381635.1	1222,35	99,7%
Select seq gb|EU556536.1|	Thymus marschallianus isolate XY tRNA-Leu (trnL) gene and trnL-trnF intergenic spacer, partial sequence; chloroplast	1195	1195	97%	0.0	99%	EU556536.1	1219,64	99,4%
Select seq gb|GU381638.1|	Thymus haussknechtii voucher M:Nydegger 43838 tRNA-Leu (trnL) gene and trnL-trnF intergenic spacer, partial sequence; chloroplast	1182	1182	96%	0.0	99%	GU381638.1	1218,94	99,4%
Select seq gb|JQ669069.1|	Thymus pulegioides voucher Riina 1577 tRNA-Leu (trnL) gene and trnL-trnF intergenic spacer, partial sequence; plastid	1205	1205	98%	0.0	99%	JQ669069.1	1217,30	99,2%
Select seq gb|GU381637.1|	Thymus serpyllum voucher M:Braeuchler 2514 tRNA-Leu (trnL) gene and trnL-trnF intergenic spacer, partial sequence; chloroplast	1205	1205	98%	0.0	99%	GU381637.1	1217,30	99,2%
Select seq gb|EU556530.1|	Thymus mandschuricus isolate HM tRNA-Leu (trnL) gene and trnL-trnF intergenic spacer, partial sequence; chloroplast	1205	1205	98%	0.0	99%	EU556530.1	1217,30	99,2%
Select seq gb|EU556535.1|	Thymus proximus isolate XN tRNA-Leu (trnL) gene and trnL-trnF intergenic spacer, partial sequence; chloroplast	1166	1166	95%	0.0	99%	EU556535.1	1215,09	99,1%
Select seq gb|KR063657.1|	Thymus sibthorpii tRNA-Leu (trnL) gene, partial sequence; trnL-trnF intergenic spacer, complete sequence; and tRNA-Phe (trnF) gene, partial sequence; chloroplast	1227	1227	100%	0.0	99%	KR063657.1	1214,73	99,0%
Select seq gb|EU556532.1|	Thymus amurensis isolate HX tRNA-Leu (trnL) gene and trnL-trnF intergenic spacer, partial sequence; chloroplast	1227	1227	100%	0.0	99%	EU556532.1	1214,73	99,0%
Select seq gb|EU556525.1|	Thymus quinquecostatus isolate CL tRNA-Leu (trnL) gene and trnL-trnF intergenic spacer, partial sequence; chloroplast	1227	1227	100%	0.0	99%	EU556525.1	1214,73	99,0%
Select seq gb|GU381634.1|	Thymus pulegioides voucher M:Braeuchler 3129 tRNA-Leu (trnL) gene and trnL-trnF intergenic spacer, partial sequence; chloroplast	1201	1201	98%	0.0	99%	GU381634.1	1213,26	98,9%
Select seq gb|GU381636.1|	Thymus vulgaris voucher M:Braeuchler 3683 tRNA-Leu (trnL) gene and trnL-trnF intergenic spacer, partial sequence; chloroplast	1199	1199	98%	0.0	99%	GU381636.1	1211,23	98,8%
Select seq gb|EU556534.1|	Thymus mongolicus isolate S1 tRNA-Leu (trnL) gene and trnL-trnF intergenic spacer, partial sequence; chloroplast	1160	1160	95%	0.0	99%	EU556534.1	1208,84	98,6%
Select seq gb|EU556539.1|	Thymus quinquecostatus isolate ZY tRNA-Leu (trnL) gene and trnL-trnF intergenic spacer, partial sequence; chloroplast	1219	1219	100%	0.0	99%	EU556539.1	1206,81	98,4%
Select seq gb|EU556538.1|	Thymus quinquecostatus isolate ZJ tRNA-Leu (trnL) gene and trnL-trnF intergenic spacer, partial sequence; chloroplast	1219	1219	100%	0.0	99%	EU556538.1	1206,81	98,4%
Select seq gb|EU556533.1|	Thymus quinquecostatus isolate HY tRNA-Leu (trnL) gene and trnL-trnF intergenic spacer, partial sequence; chloroplast	1219	1219	100%	0.0	99%	EU556533.1	1206,81	98,4%
Select seq gb|EU556527.1|	Thymus mongolicus isolate G2 tRNA-Leu (trnL) gene and trnL-trnF intergenic spacer, partial sequence; chloroplast	1219	1219	100%	0.0	99%	EU556527.1	1206,81	98,4%
Select seq gb|EU556526.1|	Thymus quinquecostatus isolate D2 tRNA-Leu (trnL) gene and trnL-trnF intergenic spacer, partial sequence; chloroplast	1219	1219	100%	0.0	99%	EU556526.1	1206,81	98,4%
Select seq gb|EU556529.1|	Thymus dahuricus isolate HD tRNA-Leu (trnL) gene and trnL-trnF intergenic spacer, partial sequence; chloroplast	1190	1190	98%	0.0	99%	EU556529.1	1202,14	98,0%
Select seq gb|EU556531.1|	Thymus amurensis isolate HW tRNA-Leu (trnL) gene and trnL-trnF intergenic spacer, partial sequence; chloroplast	1214	1214	100%	0.0	99%	EU556531.1	1201,86	98,0%
Select seq gb|GU381633.1|	Thymus caespititius voucher M:Heubl s.n. tRNA-Leu (trnL) gene and trnL-trnF intergenic spacer, partial sequence; chloroplast	1188	1188	98%	0.0	99%	GU381633.1	1200,12	97,8%
Select seq gb|EU556528.1|	Thymus mongolicus isolate G3 tRNA-Leu (trnL) gene and trnL-trnF intergenic spacer, partial sequence; chloroplast	1199	1199	99%	0.0	99%	EU556528.1	1199,00	97,8%
Select seq emb|AJ505544.1|	Thymus serpyllum var. citriodorum plastid trnL-trnF intergenic spacer, specimen voucher cult., K-1975-1177, Chase 13331 (K)	1208	1208	100%	0.0	99%	AJ505544.1	1195,92	97,5%
Select seq gb|GU381640.1|	Argantoniella salzmannii voucher M:Barra et al. 2673GL tRNA-Leu (trnL) gene and trnL-trnF intergenic spacer, partial sequence; chloroplast	1182	1182	98%	0.0	99%	GU381640.1	1194,06	97,4%
Select seq gb|AY840202.1|	Origanum vulgare tRNA-Leu (trnL) gene and trnL-trnF intergenic spacer, partial sequence; chloroplast	1182	1182	98%	0.0	99%	AY840202.1	1194,06	97,4%

Select seq gb|AY506614.1|	Origanum vulgare tRNA-Leu (trnL) gene and trnL-trnF intergenic spacer, partial sequence; chloroplast	1142	1142	95%	0.0	99%	AY506614.1	1190,08	97,0%
Select seq gb|JX880022.1|	Origanum vulgare subsp. vulgare chloroplast, complete genome	1199	1199	100%	0.0	99%	JX880022.1	1187,01	96,8%
Select seq gb|JQ690290.1|	Origanum elongatum isolate H5_O_elo trnL-trnF intergenic spacer, partial sequence; chloroplast	1199	1199	100%	0.0	99%	JQ690290.1	1187,01	96,8%
Select seq gb|JQ690289.1|	Origanum rotundifolium isolate DNA3_O_rot trnL-trnF intergenic spacer, partial sequence; chloroplast	1199	1199	100%	0.0	99%	JQ690289.1	1187,01	96,8%
Select seq emb|AJ505543.1|	Origanum vulgare plastid trnL-trnF intergenic spacer, specimen voucher cult., K-000-69-19317, chase 13334 (K)	1199	1199	100%	0.0	99%	AJ505543.1	1187,01	96,8%
Select seq gb|AY570463.1|	Origanum vulgare voucher JBW 2567 tRNA-Leu and trnL-trnF intergenic spacer, partial sequence; chloroplast	1175	1175	98%	0.0	99%	AY570463.1	1186,99	96,8%
Select seq gb|JF301392.1|	Origanum vulgare voucher B. Drew 77 tRNA-Leu (trnL) gene and trnL-trnF intergenic spacer, partial sequence; chloroplast	1149	1149	96%	0.0	99%	JF301392.1	1184,91	96,6%
Select seq gb|EU556537.1|	Thymus quinquecostatus isolate XZ tRNA-Leu (trnL) gene and trnL-trnF intergenic spacer, partial sequence; chloroplast	1195	1195	100%	0.0	99%	EU556537.1	1183,05	96,5%
Select seq gb|AY570502.1|	Thymus serpyllum voucher JBW 2564 tRNA-Leu and trnL-trnF intergenic spacer, partial sequence; chloroplast	1171	1171	98%	0.0	99%	AY570502.1	1182,95	96,4%
Select seq gb|JQ690293.1|	Origanum dayi isolate H43_O_day trnL-trnF intergenic spacer, partial sequence; chloroplast	1188	1188	100%	0.0	99%	JQ690293.1	1176,12	95,9%
Select seq gb|GU381629.1|	Thymbra capitata voucher M:Braeuchler 2518 tRNA-Leu (trnL) gene and trnL-trnF intergenic spacer, partial sequence; chloroplast	1166	1166	98%	0.0	98%	GU381629.1	1166,00	95,1%
Select seq gb|JQ669070.1|	Thymus serpyllum voucher J. Walker 2564 tRNA-Leu (trnL) gene and trnL-trnF intergenic spacer, partial sequence; plastid	1164	1164	98%	0.0	98%	JQ669070.1	1164,00	94,9%
Select seq gb|JF301401.1|	Thymbra capitata voucher UCBG 96.0817 tRNA-Leu (trnL) gene and trnL-trnF intergenic spacer, partial sequence; chloroplast	1140	1140	96%	0.0	98%	JF301401.1	1163,75	94,9%
Select seq gb|GU381484.1|	Micromeria flagellaris voucher E:van der Werff & McPherson 13570 tRNA-Leu (trnL) gene and trnL-trnF intergenic spacer, partial sequence; chloroplast	1160	1160	98%	0.0	98%	GU381484.1	1160,00	94,6%
Select seq gb|GU381483.1|	Micromeria flagellaris voucher E:Clement et al 2140 tRNA-Leu (trnL) gene and trnL-trnF intergenic spacer, partial sequence; chloroplast	1160	1160	98%	0.0	98%	GU381483.1	1160,00	94,6%
Select seq gb|AY840207.1|	Thymbra spicata tRNA-Leu (trnL) gene and trnL-trnF intergenic spacer, partial sequence; chloroplast	1160	1160	98%	0.0	98%	AY840207.1	1160,00	94,6%
Select seq gb|GU381632.1|	Thymbra spicata voucher M:Braeuchler 4548 tRNA-Leu (trnL) gene and trnL-trnF intergenic spacer, partial sequence; chloroplast	1157	1157	98%	0.0	98%	GU381632.1	1157,00	94,3%
Select seq gb|GU381627.1|	Thymbra sintenisii subsp. isaurica voucher E:Goener 12628 tRNA-Leu (trnL) gene and trnL-trnF intergenic spacer, partial sequence; chloroplast	1155	1155	98%	0.0	98%	GU381627.1	1155,00	94,2%
Select seq gb|GU381517.1|	Clinopodium barosmum voucher BM<GBR-LONDON>:McLaren N193 tRNA-Leu (trnL) gene and trnL-trnF intergenic spacer, partial sequence; chloroplast	1144	1144	98%	0.0	98%	GU381517.1	1144,00	93,3%
Select seq gb|GU381516.1|	Clinopodium wardii voucher BM<GBR-LONDON>:Ludlow et al. 14234 tRNA-Leu (trnL) gene and trnL-trnF intergenic spacer, partial sequence; chloroplast	1144	1144	98%	0.0	98%	GU381516.1	1144,00	93,3%
Select seq gb|GU381515.1|	Clinopodium hydaspidis voucher BM<GBR-LONDON>:Mohd 133 tRNA-Leu (trnL) gene and trnL-trnF intergenic spacer, partial sequence; chloroplast	1144	1144	98%	0.0	98%	GU381515.1	1144,00	93,3%
Select seq gb|GU381514.1|	Clinopodium nepalense voucher FR:Stainton 6024 tRNA-Leu (trnL) gene and trnL-trnF intergenic spacer, partial sequence; chloroplast	1144	1144	98%	0.0	98%	GU381514.1	1144,00	93,3%
Select seq gb|GU381511.1|	Clinopodium piperitum voucher BM<GBR-LONDON>:Vickery 454 tRNA-Leu (trnL) gene and trnL-trnF intergenic spacer, partial sequence; chloroplast	1144	1144	98%	0.0	98%	GU381511.1	1144,00	93,3%
Select seq gb|GU381513.1|	Clinopodium nepalense voucher BM<GBR-LONDON>:Mikage et al. 9550294 tRNA-Leu (trnL) gene and trnL-trnF intergenic spacer, partial sequence; chloroplast	1116	1116	96%	0.0	98%	GU381513.1	1139,25	92,9%
Select seq gb|GU381512.1|	Clinopodium piperitum voucher E:Stainton 7320 tRNA-Leu (trnL) gene and trnL-trnF intergenic spacer, partial sequence; chloroplast	1138	1138	98%	0.0	98%	GU381512.1	1138,00	92,8%
Select seq gb|GU381496.1|	Killickia pilosa voucher M:Braeuchler 3832 tRNA-Leu (trnL) gene and trnL-trnF intergenic spacer, partial sequence; chloroplast	1134	1134	98%	0.0	98%	GU381496.1	1134,00	92,5%
Select seq gb|GU381493.1|	Killickia grandiflora voucher M:Braeuchler 3811 tRNA-Leu (trnL) gene and trnL-trnF intergenic spacer, partial sequence; chloroplast	1134	1134	98%	0.0	98%	GU381493.1	1134,00	92,5%
Select seq gb|GU381631.1|	Satureja linearifolia voucher M:Brullo & Furnari s.n. tRNA-Leu (trnL) gene and trnL-trnF intergenic spacer, partial sequence; chloroplast	1133	1133	98%	0.0	98%	GU381631.1	1133,00	92,4%

Select seq gb|GU381495.1|	Killickia pilosa voucher M:Braeuchler 3810 tRNA-Leu (trnL) gene and trnL-trnF intergenic spacer, partial sequence; chloroplast	1120	1120	97% 0.0	98% GU381495.1	1131,55	92,3%
Select seq gb|GU381628.1|	Thymbra calostachya voucher M:Ulrich s.n. tRNA-Leu (trnL) gene and trnL-trnF intergenic spacer, partial sequence; chloroplast	1129	1129	98% 0.0	98% GU381628.1	1129,00	92,0%
Select seq gb|GU381481.1|	Micromeria cf. madagascariensis Morawetz 205 tRNA-Leu (trnL) gene and trnL-trnF intergenic spacer, partial sequence; chloroplast	1133	1133	98% 0.0	97% GU381481.1	1121,44	91,4%
Select seq gb|GU381485.1|	Micromeria sphaerophylla voucher E:Lewis et al 1064 tRNA-Leu (trnL) gene and trnL-trnF intergenic spacer, partial sequence; chloroplast	1131	1131	98% 0.0	97% GU381485.1	1119,46	91,3%
Select seq gb|KR150198.1|	Ziziphora sp. 3 Kh112 trnL-trnF intergenic spacer region, partial sequence; chloroplast	1118	1118	97% 0.0	97% KR150198.1	1118,00	91,1%
Select seq gb|GU381507.1|	Ziziphora tenuior voucher MSB:Fayvush et al. 03-1503 tRNA-Leu (trnL) gene and trnL-trnF intergenic spacer, partial sequence; chloroplast	1127	1127	98% 0.0	97% GU381507.1	1115,50	90,9%
Select seq gb|GU381489.1|	Killickia lutea voucher NU<ZAF>:Hilliard & Burtt 9876 tRNA-Leu (trnL) gene and trnL-trnF intergenic spacer, partial sequence; chloroplast	1123	1123	98% 0.0	97% GU381489.1	1111,54	90,6%
Select seq gb|GU381488.1|	Killickia compacta voucher M:Braeuchler 3816 tRNA-Leu (trnL) gene and trnL-trnF intergenic spacer, partial sequence; chloroplast	1123	1123	98% 0.0	97% GU381488.1	1111,54	90,6%
Select seq gb|DQ667501.1|	Ziziphora taurica isolate x262 tRNA-Leu (trnL) gene and trnL-trnF intergenic spacer, partial sequence; chloroplast	1123	1123	98% 0.0	97% DQ667501.1	1111,54	90,6%
Select seq gb|GU381510.1|	Ziziphora pamiroalaica voucher C:Murray et al. 10090 tRNA-Leu (trnL) gene and trnL-trnF intergenic spacer, partial sequence; chloroplast	1122	1122	98% 0.0	97% GU381510.1	1110,55	90,5%
Select seq gb|GU381505.1|	Clinopodium troodi voucher W:Davis 1856 tRNA-Leu (trnL) gene and trnL-trnF intergenic spacer, partial sequence; chloroplast	1122	1122	98% 0.0	97% GU381505.1	1110,55	90,5%
Select seq gb|GU381501.1|	Clinopodium graveolens subsp. rotundifolium voucher M:Podlech 47181 tRNA-Leu (trnL) gene and trnL-trnF intergenic spacer, partial sequence; chloroplast	1122	1122	98% 0.0	97% GU381501.1	1110,55	90,5%
Select seq gb|GU381499.1|	Clinopodium suaveolens voucher M:Erben s.n. tRNA-Leu (trnL) gene and trnL-trnF intergenic spacer, partial sequence; chloroplast	1122	1122	98% 0.0	97% GU381499.1	1110,55	90,5%
Select seq gb|GU381506.1|	Ziziphora tenuior voucher MSB:Nydegger 43557 tRNA-Leu (trnL) gene and trnL-trnF intergenic spacer, partial sequence; chloroplast	1099	1099	96% 0.0	97% GU381506.1	1110,45	90,5%
Select seq gb|KR150243.1|	Ziziphora sp. 2 Kh20 trnL-trnF intergenic spacer region, partial sequence; chloroplast	1109	1109	97% 0.0	97% KR150243.1	1109,00	90,4%
Select seq gb|GU381621.1|	Satureja cuneifolia voucher M:Rechinger 11142 tRNA-Leu (trnL) gene and trnL-trnF intergenic spacer, partial sequence; chloroplast	1118	1118	98% 0.0	97% GU381621.1	1106,59	90,2%
Select seq gb|GU381612.1|	Gontscharovia popovii voucher BM<GBR-LONDON>:Schmid 2419 tRNA-Leu (trnL) gene and trnL-trnF intergenic spacer, partial sequence; chloroplast	1118	1118	98% 0.0	97% GU381612.1	1106,59	90,2%
Select seq gb|AY840179.1|	Satureja montana tRNA-Leu (trnL) gene and trnL-trnF intergenic spacer, partial sequence; chloroplast	1118	1118	98% 0.0	97% AY840179.1	1106,59	90,2%
Select seq gb|GU381497.1|	Clinopodium acinos voucher M:Podlech 50287 tRNA-Leu (trnL) gene and trnL-trnF intergenic spacer, partial sequence; chloroplast	1116	1116	98% 0.0	97% GU381497.1	1104,61	90,1%
Select seq gb|JQ669067.1|	Satureja montana voucher UCBG 2002.0593, Forbes s.n tRNA-Leu (trnL) gene and trnL-trnF intergenic spacer, partial sequence; plastid	1112	1112	98% 0.0	97% JQ669067.1	1100,65	89,7%
Select seq gb|GU381619.1|	Satureja mutica voucher M:Akhani 12362 tRNA-Leu (trnL) gene and trnL-trnF intergenic spacer, partial sequence; chloroplast	1112	1112	98% 0.0	97% GU381619.1	1100,65	89,7%
Select seq gb|GU381614.1|	Satureja thymbra voucher M:Braeuchler 2896 tRNA-Leu (trnL) gene and trnL-trnF intergenic spacer, partial sequence; chloroplast	1112	1112	98% 0.0	97% GU381614.1	1100,65	89,7%
Select seq gb|GU381611.1|	Gontscharovia popovii voucher M:Vvedensky s.n. tRNA-Leu (trnL) gene and trnL-trnF intergenic spacer, partial sequence; chloroplast	1112	1112	98% 0.0	97% GU381611.1	1100,65	89,7%
Select seq gb|GU381521.1|	Mentha pulegium voucher M:Braeuchler 2300 tRNA-Leu (trnL) gene and trnL-trnF intergenic spacer, partial sequence; chloroplast	1112	1112	98% 0.0	97% GU381521.1	1100,65	89,7%
Select seq gb|KR063656.1|	Satureja pilosa subsp. origanita tRNA-Leu (trnL) gene, partial sequence; trnL-trnF intergenic spacer, complete sequence; and tRNA-Phe (trnF) gene, partial sequence; chloroplast	1134	1134	100% 0.0	97% KR063656.1	1099,98	89,7%
Select seq gb|KR150209.1|	Satureja sp. 3 Kh90 trnL-trnF intergenic spacer region, partial sequence; chloroplast	1122	1122	99% 0.0	97% KR150209.1	1099,33	89,6%
Select seq gb|KR150238.1|	Ziziphora sp. 1 Kh75 trnL-trnF intergenic spacer region, partial sequence; chloroplast	1133	1133	100% 0.0	97% KR150238.1	1099,01	89,6%
Select seq gb|JQ669021.1|	Clinopodium acinos voucher Judziewicz 14160 tRNA-Leu (trnL) gene and trnL-trnF intergenic spacer, partial sequence; plastid	1110	1110	98% 0.0	97% JQ669021.1	1098,67	89,6%

Select seq gb|GU381625.1|	Pentapleura subulifera voucher W:Rechinger 12085 tRNA-Leu (trnL) gene and trnL-trnF intergenic spacer, partial sequence; chloroplast	1109	1109	98%	0.0	97%	GU381625.1	1097,68	89,5%
Select seq gb|GU381500.1|	Clinopodium nanum voucher M:Braeuchler 2796 tRNA-Leu (trnL) gene and trnL-trnF intergenic spacer, partial sequence; chloroplast	1109	1109	98%	0.0	97%	GU381500.1	1097,68	89,5%
Select seq gb|GU381615.1|	Satureja innota voucher M:Barra et al. 2484GL tRNA-Leu (trnL) gene and trnL-trnF intergenic spacer, partial sequence; chloroplast	1107	1107	98%	0.0	97%	GU381615.1	1095,70	89,3%
Select seq gb|KR150233.1|	Thymus sp. 6 Kh133 trnL-trnF intergenic spacer region, partial sequence; chloroplast	1118	1118	99%	0.0	97%	KR150233.1	1095,41	89,3%
Select seq gb|JQ669068.1|	Satureja thymbra voucher UCBG 2002.0540, Forbes s.n. tRNA-Leu (trnL) gene and trnL-trnF intergenic spacer, partial sequence; plastid	1101	1101	98%	0.0	97%	JQ669068.1	1089,77	88,8%
Select seq gb|GU381623.1|	Satureja spicigera voucher M:Kartli et al. 2726 tRNA-Leu (trnL) gene and trnL-trnF intergenic spacer, partial sequence; chloroplast	1101	1101	98%	0.0	97%	GU381623.1	1089,77	88,8%
Select seq gb|GU381522.1|	Mentha cervina voucher M:Braeuchler 2394 tRNA-Leu (trnL) gene and trnL-trnF intergenic spacer, partial sequence; chloroplast	1099	1099	98%	0.0	97%	GU381522.1	1087,79	88,7%
Select seq gb|KC414276.1|	Mentha canadensis isolate 511190001 tRNA-Leu (trnL) gene and trnL-trnF intergenic spacer, partial sequence; chloroplast	1110	1110	100%	0.0	97%	KC414276.1	1076,70	87,8%
Select seq gb|GU381630.1|	Satureja thymbrifolia voucher M:Danin s.n. tRNA-Leu (trnL) gene and trnL-trnF intergenic spacer, partial sequence; chloroplast	1099	1099	98%	0.0	96%	GU381630.1	1076,57	87,8%
Select seq emb|AJ505541.1|	Mentha suaveolens plastid trnL-trnF intergenic spacer, specimen voucher cult., K-1970-3169 (K)	1109	1109	100%	0.0	97%	AJ505541.1	1075,73	87,7%
Select seq gb|DQ667492.1|	Lepechinia lancifolia isolate x232 tRNA-Leu (trnL) gene and trnL-trnF intergenic spacer, partial sequence; chloroplast	1107	1107	100%	0.0	97%	DQ667492.1	1073,79	87,5%
Select seq gb|KR150240.1|	Satureja sp. 2 Kh70 trnL-trnF intergenic spacer region, partial sequence; chloroplast	1099	1099	99%	0.0	96%	KR150240.1	1065,70	86,9%
Select seq gb|FJ593456.1|	Mentha sp. JSZ-2009a tRNA-Leu (trnL) gene and trnL-trnF intergenic spacer, partial sequence; chloroplast	1103	1103	100%	0.0	96%	FJ593456.1	1058,88	86,3%
								0,00	0,0%
								0,00	0,0%
								0,00	0,0%
								0,00	0,0%

Select for downloading or viewing reports	Kh025_ITS Description	Max score	Total score	Query cover	E value	Ident	Accession	(Ident/Cover)* Max score	Deviation from top hit
Select seq gb|AY826248.1|	Carthamus oxyacanthus internal transcribed spacer 1, partial sequence; 5.8S ribosomal RNA gene, complete sequence; and internal transcribed spacer 2, partial sequence	1171	1171	83%	0.0	100%	AY826248.1	1410,84	100,0%
Select seq gb|HQ407426.1|	Carthamus palaestinus internal transcribed spacer 1, 5.8S ribosomal RNA gene, and internal transcribed spacer 2, complete sequence	1179	1179	84%	0.0	100%	HQ407426.1	1403,57	99,5%
Select seq gb|AY504689.1|	Carthamus oxyacanthus internal transcribed spacer 1, 5.8S ribosomal RNA gene, and internal transcribed spacer 2, complete sequence	1164	1164	83%	0.0	99%	AY504689.1	1388,39	98,4%
Select seq gb|JQ230977.1|	Carthamus tinctorius voucher SBB-1162 internal transcribed spacer 1, partial sequence; 5.8S ribosomal RNA gene, complete sequence; and internal transcribed spacer 2, partial sequence	1286	1286	92%	0.0	99%	JQ230977.1	1383,85	98,1%
Select seq gb|EF483946.1|	Carthamus tinctorius cultivar BJ1063 internal transcribed spacer 1, 5.8S ribosomal RNA gene, and internal transcribed spacer 2, complete sequence	1325	1325	95%	0.0	99%	EF483946.1	1380,79	97,9%
Select seq gb|EF483943.1|	Carthamus tinctorius cultivar W66730 internal transcribed spacer 1, 5.8S ribosomal RNA gene, and internal transcribed spacer 2, complete sequence	1325	1325	95%	0.0	99%	EF483943.1	1380,79	97,9%
Select seq gb|GU969644.1|	Carthamus persicus voucher K:31517 internal transcribed spacer 1, partial sequence; 5.8S ribosomal RNA gene, complete sequence; and internal transcribed spacer 2, partial sequence	1297	1359	93%	0.0	99%	GU969644.1	1380,68	97,9%
Select seq gb|GU969639.1|	Carthamus oxyacanthus voucher GH:244792 internal transcribed spacer 1, partial sequence; 5.8S ribosomal RNA gene, complete sequence; and internal transcribed spacer 2, partial sequence	1297	1297	93%	0.0	99%	GU969639.1	1380,68	97,9%
Select seq gb|GU969640.1|	Carthamus oxyacanthus bio-material USDA seedstock PI426185 internal transcribed spacer 1, partial sequence; 5.8S ribosomal RNA gene, complete sequence; and internal transcribed spacer 2, partial sequence	1297	1297	93%	0.0	99%	GU969640.1	1380,68	97,9%
Select seq gb|HM921410.1|	Carthamus tinctorius voucher CAUTI-19 internal transcribed spacer 1, partial sequence; 5.8S ribosomal RNA gene, complete sequence; and internal transcribed spacer 2, partial sequence	1240	1240	89%	0.0	99%	HM921410.1	1379,33	97,8%
Select seq gb|GU969641.1|	Carthamus oxyacanthus bio-material USDA seedstock PI426428 internal transcribed spacer 1, partial sequence; 5.8S ribosomal RNA gene, complete sequence; and internal transcribed spacer 2, partial sequence	1295	1295	93%	0.0	99%	GU969641.1	1378,55	97,7%
Select seq gb|EF483948.1|	Carthamus tinctorius cultivar BJ1067 internal transcribed spacer 1, 5.8S ribosomal RNA gene, and internal transcribed spacer 2, complete sequence	1267	1267	91%	0.0	99%	EF483948.1	1378,38	97,7%
Select seq gb|EF483950.1|	Carthamus tinctorius cultivar ToziSpiny internal transcribed spacer 1, 5.8S ribosomal RNA gene, and internal transcribed spacer 2, complete sequence	1321	1321	95%	0.0	99%	EF483950.1	1376,62	97,6%
Select seq gb|EF483949.1|	Carthamus tinctorius cultivar BJ2701 internal transcribed spacer 1, 5.8S ribosomal RNA gene, and internal transcribed spacer 2, complete sequence	1321	1321	95%	0.0	99%	EF483949.1	1376,62	97,6%
Select seq gb|EF483947.1|	Carthamus tinctorius cultivar BJ673 internal transcribed spacer 1, 5.8S ribosomal RNA gene, and internal transcribed spacer 2, complete sequence	1321	1321	95%	0.0	99%	EF483947.1	1376,62	97,6%
Select seq gb|EF483944.1|	Carthamus tinctorius cultivar LESAF internal transcribed spacer 1, 5.8S ribosomal RNA gene, and internal transcribed spacer 2, complete sequence	1321	1321	95%	0.0	99%	EF483944.1	1376,62	97,6%
Select seq gb|GU969647.1|	Carthamus tinctorius bio-material Good Lab seedstock Centennial internal transcribed spacer 1, partial sequence; 5.8S ribosomal RNA gene, complete sequence; and internal transcribed spacer 2, partial sequence	1279	1279	92%	0.0	99%	GU969647.1	1376,32	97,6%
Select seq gb|GU969648.1|	Carthamus tinctorius bio-material Good Lab seedstock NP12 internal transcribed spacer 1, partial sequence; 5.8S ribosomal RNA gene, complete sequence; and internal transcribed spacer 2, partial sequence	1291	1291	93%	0.0	99%	GU969648.1	1374,29	97,4%
Select seq gb|EF483945.1|	Carthamus tinctorius cultivar ENANA internal transcribed spacer 1, 5.8S ribosomal RNA gene, and internal transcribed spacer 2, complete sequence	1317	1317	95%	0.0	99%	EF483945.1	1372,45	97,3%
Select seq gb|GU969643.1|	Carthamus palaestinus bio-material USDA seedstock PI235663 internal transcribed spacer 1, partial sequence; 5.8S ribosomal RNA gene, complete sequence; and internal transcribed spacer 2, partial sequence	1288	1288	93%	0.0	99%	GU969643.1	1371,10	97,2%
Select seq gb|GU724280.1|	Carthamus tinctorius voucher PS0629MT02 18S ribosomal RNA gene, partial sequence; internal transcribed spacer 1, 5.8S ribosomal RNA gene, and internal transcribed spacer 2, complete sequence; and 26S ribosomal RNA gene, partial sequence	1315	1315	95%	0.0	99%	GU724280.1	1370,37	97,1%
Select seq gb|HQ112176.1|	Carthamus glaucus isolate HW-023 internal transcribed spacer 1, partial sequence; 5.8S ribosomal RNA gene, complete sequence; and internal transcribed spacer 2, partial sequence	1273	1273	92%	0.0	99%	HQ112176.1	1369,86	97,1%
Select seq gb|HQ112154.1|	Carthamus glaucus isolate HW-001 internal transcribed spacer 1, partial sequence; 5.8S ribosomal RNA gene, complete sequence; and internal transcribed spacer 2, partial sequence	1273	1273	92%	0.0	99%	HQ112154.1	1369,86	97,1%
Select seq gb|GU969649.1|	Carthamus tinctorius bio-material Good Lab seedstock S317 internal transcribed spacer 1, partial sequence; 5.8S ribosomal RNA gene, complete sequence; and internal transcribed spacer 2, partial sequence	1273	1273	92%	0.0	99%	GU969649.1	1369,86	97,1%
Select seq gb|FJ539127.1|	Carthamus tinctorius 18S ribosomal RNA gene, partial sequence; internal transcribed spacer 1, 5.8S ribosomal RNA gene, and internal transcribed spacer 2, complete sequence; and 26S ribosomal RNA gene, partial sequence	1273	1273	92%	0.0	99%	FJ539127.1	1369,86	97,1%
Select seq gb|GU969650.1|	Carthamus tinctorius voucher DAV:27591 internal transcribed spacer 1, partial sequence; 5.8S ribosomal RNA gene, complete sequence; and internal transcribed spacer 2, partial sequence	1286	1286	93%	0.0	99%	GU969650.1	1368,97	97,0%

Select seq gb|HQ112166.1|	Carthamus glaucus isolate HW-013 internal transcribed spacer 1, partial sequence; 5.8S ribosomal RNA gene, complete sequence; and internal transcribed spacer 2, partial sequence	1269	1269	92% 0.0	99% HQ112166.1	1365,55	96,8%
Select seq gb|HQ112155.1|	Carthamus glaucus isolate HW-002 internal transcribed spacer 1, partial sequence; 5.8S ribosomal RNA gene, complete sequence; and internal transcribed spacer 2, partial sequence	1269	1269	92% 0.0	99% HQ112155.1	1365,55	96,8%
Select seq gb|GU969651.1|	Carthamus tinctorius voucher AHUC:33256 internal transcribed spacer 1, partial sequence; 5.8S ribosomal RNA gene, complete sequence; and internal transcribed spacer 2, partial sequence	1282	1282	93% 0.0	99% GU969651.1	1364,71	96,7%
Select seq gb|HQ112168.1|	Carthamus glaucus isolate HW-015 internal transcribed spacer 1, partial sequence; 5.8S ribosomal RNA gene, complete sequence; and internal transcribed spacer 2, partial sequence	1267	1267	92% 0.0	99% HQ112168.1	1363,40	96,6%
Select seq gb|HQ112161.1|	Carthamus glaucus isolate HW-008 internal transcribed spacer 1, partial sequence; 5.8S ribosomal RNA gene, complete sequence; and internal transcribed spacer 2, partial sequence	1267	1267	92% 0.0	99% HQ112161.1	1363,40	96,6%
Select seq gb|HQ112172.1|	Carthamus glaucus isolate HW-019 internal transcribed spacer 1, partial sequence; 5.8S ribosomal RNA gene, complete sequence; and internal transcribed spacer 2, partial sequence	1266	1266	92% 0.0	99% HQ112172.1	1362,33	96,6%
Select seq gb|HQ112156.1|	Carthamus glaucus isolate HW-003 internal transcribed spacer 1, partial sequence; 5.8S ribosomal RNA gene, complete sequence; and internal transcribed spacer 2, partial sequence	1266	1266	92% 0.0	99% HQ112156.1	1362,33	96,6%
Select seq gb|HQ112171.1|	Carthamus glaucus isolate HW-018 internal transcribed spacer 1, partial sequence; 5.8S ribosomal RNA gene, complete sequence; and internal transcribed spacer 2, partial sequence	1262	1262	92% 0.0	99% HQ112171.1	1358,02	96,3%
Select seq gb|HQ112157.1|	Carthamus glaucus isolate HW-004 internal transcribed spacer 1, partial sequence; 5.8S ribosomal RNA gene, complete sequence; and internal transcribed spacer 2, partial sequence	1262	1262	92% 0.0	99% HQ112157.1	1358,02	96,3%
Select seq gb|EU592011.1|	Carthamus tinctorius internal transcribed spacer 1, partial sequence; 5.8S ribosomal RNA gene, complete sequence; and internal transcribed spacer 2, partial sequence	1123	1123	84% 0.0	98% EU592011.1	1310,17	92,9%
Select seq gb|GU969631.1|	Carthamus lanatus voucher GH:244778 internal transcribed spacer 1, partial sequence; 5.8S ribosomal RNA gene, complete sequence; and internal transcribed spacer 2, partial sequence	1203	1203	93% 0.0	97% GU969631.1	1254,74	88,9%
Select seq gb|AY826249.1|	Carthamus turkestanicus internal transcribed spacer 1, partial sequence; 5.8S ribosomal RNA gene, complete sequence; and internal transcribed spacer 2, partial sequence	1072	1072	83% 0.0	97% AY826249.1	1252,82	88,8%
Select seq gb|GU969624.1|	Carthamus glaucus voucher AHUC:26223 internal transcribed spacer 1, partial sequence; 5.8S ribosomal RNA gene, complete sequence; and internal transcribed spacer 2, partial sequence	1147	1147	89% 0.0	97% GU969624.1	1250,10	88,6%
Select seq gb|HM921409.1|	Carthamus lanatus voucher CAULA-7 internal transcribed spacer 1, partial sequence; 5.8S ribosomal RNA gene, complete sequence; and internal transcribed spacer 2, partial sequence	1066	1066	83% 0.0	97% HM921409.1	1245,81	88,3%
Select seq gb|AY826247.2|	Carthamus lanatus subsp. baeticus internal transcribed spacer 1, 5.8S ribosomal RNA gene, and internal transcribed spacer 2, complete sequence	1066	1066	83% 0.0	97% AY826247.2	1245,81	88,3%
Select seq gb|GU969653.1|	Carthamus turkestanicus bio-material USDA seedstock PI426180 internal transcribed spacer 1, partial sequence; 5.8S ribosomal RNA gene, complete sequence; and internal transcribed spacer 2, partial sequence	1192	1192	93% 0.0	97% GU969653.1	1243,27	88,1%
Select seq gb|GU969625.1|	Carthamus glaucus voucher GH:244779 internal transcribed spacer 1, partial sequence; 5.8S ribosomal RNA gene, complete sequence; and internal transcribed spacer 2, partial sequence	1192	1254	93% 0.0	97% GU969625.1	1243,27	88,1%
Select seq gb|GU969622.1|	Carthamus dentatus voucher AHUC:26216 internal transcribed spacer 1, partial sequence; 5.8S ribosomal RNA gene, complete sequence; and internal transcribed spacer 2, partial sequence	1192	1192	93% 0.0	97% GU969622.1	1243,27	88,1%
Select seq gb|GU969627.1|	Carthamus glaucus voucher AHUC:27774 internal transcribed spacer 1, partial sequence; 5.8S ribosomal RNA gene, complete sequence; and internal transcribed spacer 2, partial sequence	1166	1166	91% 0.0	97% GU969627.1	1242,88	88,1%
Select seq gb|GU969654.1|	Carthamus turkestanicus bio-material USDA seedstock PI426426 internal transcribed spacer 1, partial sequence; 5.8S ribosomal RNA gene, complete sequence; and internal transcribed spacer 2, partial sequence	1190	1190	93% 0.0	97% GU969654.1	1241,18	88,0%
Select seq gb|GU969626.1|	Carthamus glaucus voucher AHUC:27771 internal transcribed spacer 1, partial sequence; 5.8S ribosomal RNA gene, complete sequence; and internal transcribed spacer 2, partial sequence	1190	1190	93% 0.0	97% GU969626.1	1241,18	88,0%
Select seq gb|GU969629.1|	Carthamus lanatus voucher DAV:34452 internal transcribed spacer 1, partial sequence; 5.8S ribosomal RNA gene, complete sequence; and internal transcribed spacer 2, partial sequence	1188	1188	93% 0.0	97% GU969629.1	1239,10	87,8%
Select seq gb|GU969619.1|	Carthamus lanatus subsp. baeticus bio-material SemBioSys Inc seedstock internal transcribed spacer 1, partial sequence; 5.8S ribosomal RNA gene, complete sequence; and internal transcribed spacer 2, partial sequence	1175	1175	92% 0.0	97% GU969619.1	1238,86	87,8%
Select seq gb|HQ407425.1|	Carthamus glaucus internal transcribed spacer 1, 5.8S ribosomal RNA gene, and internal transcribed spacer 2, complete sequence	1134	1134	89% 0.0	97% HQ407425.1	1235,93	87,6%
Select seq gb|GU969633.1|	Carthamus lanatus bio-material USDA seedstock PI326364 internal transcribed spacer 1, partial sequence; 5.8S ribosomal RNA gene, complete sequence; and internal transcribed spacer 2, partial sequence	1184	1184	93% 0.0	97% GU969633.1	1234,92	87,5%
Select seq gb|GU969632.1|	Carthamus lanatus bio-material USDA seedstock PI235668 internal transcribed spacer 1, partial sequence; 5.8S ribosomal RNA gene, complete sequence; and internal transcribed spacer 2, partial sequence	1184	1184	93% 0.0	97% GU969632.1	1234,92	87,5%
Select seq gb|GU969645.1|	Carthamus tenuis voucher DAV:24730 internal transcribed spacer 1, partial sequence; 5.8S ribosomal RNA gene, complete sequence; and internal transcribed spacer 2, partial sequence	1182	1182	93% 0.0	97% GU969645.1	1232,84	87,4%
Select seq gb|GU969637.1|	Carthamus nitidus voucher AHUC:26224 internal transcribed spacer 1, partial sequence; 5.8S ribosomal RNA gene, complete sequence; and internal transcribed spacer 2, partial sequence	1179	1179	93% 0.0	97% GU969637.1	1229,71	87,2%

Select seq gb|GU969616.1|	Carthamus alexandrinus voucher AHUC:23230 internal transcribed spacer 1, partial sequence; 5.8S ribosomal RNA gene, complete sequence; and internal transcribed spacer 2, partial sequence	1177	1177	93% 0.0	97% GU969616.1	1227,62	87,0%
Select seq gb|GU969635.1|	Carthamus leucocaulos bio-material USDA seedstock PI54-53 internal transcribed spacer 1, partial sequence; 5.8S ribosomal RNA gene, complete sequence; and internal transcribed spacer 2, partial sequence	1162	1162	92% 0.0	97% GU969635.1	1225,15	86,8%
Select seq gb|GU969617.1|	Carthamus alexandrinus voucher AHUC:27773 internal transcribed spacer 1, partial sequence; 5.8S ribosomal RNA gene, complete sequence; and internal transcribed spacer 2, partial sequence	1173	1173	93% 0.0	97% GU969617.1	1223,45	86,7%
Select seq gb|GU969634.1|	Carthamus leucocaulos voucher DAV:52307 internal transcribed spacer 1, partial sequence; 5.8S ribosomal RNA gene, complete sequence; and internal transcribed spacer 2, partial sequence	1171	1171	93% 0.0	97% GU969634.1	1221,37	86,6%
Select seq gb|GU969628.1|	Carthamus lanatus voucher DAV:24885 internal transcribed spacer 1, partial sequence; 5.8S ribosomal RNA gene, complete sequence; and internal transcribed spacer 2, partial sequence	1175	1175	93% 0.0	96% GU969628.1	1212,90	86,0%
Select seq gb|GU969623.1|	Carthamus divaricatus voucher AHUC:35617 internal transcribed spacer 1, partial sequence; 5.8S ribosomal RNA gene, complete sequence; and internal transcribed spacer 2, partial sequence	1170	1170	93% 0.0	96% GU969623.1	1207,74	85,6%
Select seq emb|HE602470.1|	Carthamus pinnatus genomic DNA containing ITS1, 5.8S rRNA gene, ITS2, specimen voucher Kool, A. 1018	1053	1053	85% 0.0	96% HE602470.1	1189,27	84,3%
Select seq gb|AY829445.1|	Psephellus xanthocephalus internal transcribed spacer 1, partial sequence; 5.8S ribosomal RNA gene, complete sequence; and internal transcribed spacer 2, partial sequence	1027	1027	83% 0.0	96% AY829445.1	1187,86	84,2%
Select seq gb|AY826315.1|	Psephellus gilanicus internal transcribed spacer 1, partial sequence; 5.8S ribosomal RNA gene, complete sequence; and internal transcribed spacer 2, partial sequence	1027	1027	83% 0.0	96% AY826315.1	1187,86	84,2%
Select seq gb|AY826317.1|	Psephellus pulcherrimus internal transcribed spacer 1, partial sequence; 5.8S ribosomal RNA gene, complete sequence; and internal transcribed spacer 2, partial sequence	1016	1016	83% 0.0	96% AY826317.1	1175,13	83,3%
Select seq gb|GU969638.1|	Carthamus oxyacanthus voucher GH:244786 internal transcribed spacer 1, partial sequence; 5.8S ribosomal RNA gene, complete sequence; and internal transcribed spacer 2, partial sequence	1142	1142	93% 0.0	95% GU969638.1	1166,56	82,7%
Select seq gb|GU969630.1|	Carthamus lanatus voucher GH:244776 internal transcribed spacer 1, partial sequence; 5.8S ribosomal RNA gene, complete sequence; and internal transcribed spacer 2, partial sequence	1138	1138	93% 0.0	95% GU969630.1	1162,47	82,4%
Select seq gb|AY826295.1|	Klasea serratuloides internal transcribed spacer 1, partial sequence; 5.8S ribosomal RNA gene, complete sequence; and internal transcribed spacer 2, partial sequence	1013	1013	83% 0.0	95% AY826295.1	1159,46	82,2%
Select seq gb|AY826316.1|	Psephellus persicus internal transcribed spacer 1, partial sequence; 5.8S ribosomal RNA gene, complete sequence; and internal transcribed spacer 2, partial sequence	1011	1011	83% 0.0	95% AY826316.1	1157,17	82,0%
Select seq gb|DQ310928.1|	Klasea biebersteiniana from Armenia internal transcribed spacer 1, 5.8S ribosomal RNA gene, and internal transcribed spacer 2, complete sequence	1042	1042	86% 0.0	95% DQ310928.1	1151,05	81,6%
Select seq gb|DQ310927.1|	Klasea chartacea from Tajikistan internal transcribed spacer 1, 5.8S ribosomal RNA gene, and internal transcribed spacer 2, complete sequence	1042	1042	86% 0.0	95% DQ310927.1	1151,05	81,6%
Select seq gb|DQ310926.1|	Klasea coriacea from Armenia internal transcribed spacer 1, 5.8S ribosomal RNA gene, and internal transcribed spacer 2, complete sequence	1037	1037	86% 0.0	95% DQ310926.1	1145,52	81,2%
Select seq gb|AY914830.1|	Serratula strangulata internal transcribed spacer 1, 5.8 S ribosomal RNA gene, and internal transcribed spacer 2, complete sequence	1011	1011	84% 0.0	95% AY914830.1	1143,39	81,0%
Select seq gb|GU969620.1|	Carthamus caeruleus voucher AHUC:27785 internal transcribed spacer 1, partial sequence; 5.8S ribosomal RNA gene, complete sequence; and internal transcribed spacer 2, partial sequence	1118	1118	93% 0.0	95% GU969620.1	1142,04	80,9%
Select seq gb|DQ310929.1|	Klasea algida from Tajikistan internal transcribed spacer 1, 5.8S ribosomal RNA gene, and internal transcribed spacer 2, complete sequence	1031	1031	86% 0.0	95% DQ310929.1	1138,90	80,7%
Select seq gb|DQ310943.1|	Rhaponticum pulchrum from Russia internal transcribed spacer 1, 5.8S ribosomal RNA gene, and internal transcribed spacer 2, complete sequence	1029	1029	86% 0.0	95% DQ310943.1	1136,69	80,6%
Select seq gb|DQ310935.1|	Rhaponticum lyratum from Tajikistan internal transcribed spacer 1, 5.8S ribosomal RNA gene, and internal transcribed spacer 2, complete sequence	1029	1029	86% 0.0	95% DQ310935.1	1136,69	80,6%
Select seq gb|DQ310948.1|	Leuzea berardioides from Morocco internal transcribed spacer 1, 5.8S ribosomal RNA gene, and internal transcribed spacer 2, complete sequence	1027	1027	86% 0.0	95% DQ310948.1	1134,48	80,4%
Select seq gb|DQ310934.1|	Rhaponticum integrifolium from Tajikistan internal transcribed spacer 1, 5.8S ribosomal RNA gene, and internal transcribed spacer 2, complete sequence	1024	1024	86% 0.0	95% DQ310934.1	1131,16	80,2%
Select seq gb|DQ310951.1|	Rhaponticum scariosum from Russia internal transcribed spacer 1, 5.8S ribosomal RNA gene, and internal transcribed spacer 2, complete sequence	1022	1022	86% 0.0	95% DQ310951.1	1128,95	80,0%
Select seq gb|DQ310944.1|	Rhaponticum insigne from Iran internal transcribed spacer 1, 5.8S ribosomal RNA gene, and internal transcribed spacer 2, complete sequence	1022	1022	86% 0.0	95% DQ310944.1	1128,95	80,0%
Select seq gb|KJ961607.1|	Centaurea breviceps voucher KWU-59569 18S ribosomal RNA gene, partial sequence; internal transcribed spacer 1, 5.8S ribosomal RNA gene, and internal transcribed spacer 2, complete sequence; and 28S ribosomal RNA gene, partial sequence	1081	1081	91% 0.0	95% KJ961607.1	1128,52	80,0%
Select seq gb|DQ310952.1|	Rhaponticum scariosum from Slovenia internal transcribed spacer 1, 5.8S ribosomal RNA gene, and internal transcribed spacer 2, complete sequence	1018	1018	86% 0.0	95% DQ310952.1	1124,53	79,7%

Select seq gb|DQ310924.1|	Klasea cerinthifolia from Iran internal transcribed spacer 1, 5.8S ribosomal RNA gene, and internal transcribed spacer 2, complete sequence	1018	1018	86% 0.0	95% DQ310924.1	1124,53	79,7%
Select seq gb|KJ961606.1|	Centaurea breviceps voucher KHER-10001 18S ribosomal RNA gene, partial sequence; internal transcribed spacer 1, 5.8S ribosomal RNA gene, and internal transcribed spacer 2, complete sequence; and 28S ribosomal RNA gene, partial sequence	1077	1077	91% 0.0	95% KJ961606.1	1124,34	79,7%
Select seq gb|KJ486335.1|	Klasea leptoclada 18S ribosomal RNA gene, partial sequence; internal transcribed spacer 1, 5.8S ribosomal RNA gene, and internal transcribed spacer 2, complete sequence; and 26S ribosomal RNA gene, partial sequence	1064	1064	90% 0.0	95% KJ486335.1	1123,11	79,6%
Select seq gb|GU969636.1|	Centaurea montana voucher ALTA:G.W. & G.G. Douglas s.n. internal transcribed spacer 1, partial sequence; 5.8S ribosomal RNA gene, complete sequence; and internal transcribed spacer 2, partial sequence	1099	1099	93% 0.0	95% GU969636.1	1122,63	79,6%
Select seq gb|DQ310950.1|	Rhaponticum longifolium from Portugal internal transcribed spacer 1, 5.8S ribosomal RNA gene, and internal transcribed spacer 2, complete sequence	1014	1014	86% 0.0	95% DQ310950.1	1120,12	79,4%
Select seq gb|GU969618.1|	Carthamus arborescens voucher AHUC:31960 internal transcribed spacer 1, partial sequence; 5.8S ribosomal RNA gene, complete sequence; and internal transcribed spacer 2, partial sequence	1092	1092	93% 0.0	95% GU969618.1	1115,48	79,1%
Select seq gb|DQ310930.1|	Klasea grandifolia from Iran internal transcribed spacer 1, 5.8S ribosomal RNA gene, and internal transcribed spacer 2, complete sequence	1016	1016	86% 0.0	94% DQ310930.1	1110,51	78,7%
Select seq emb|HE687358.1|	Rhaponticum sp. EM413 genomic DNA containing ITS1, 5.8S rRNA gene, ITS2, specimen voucher EM413	1016	1016	87% 0.0	95% HE687358.1	1109,43	78,6%
Select seq gb|DQ310945.1|	Rhaponticum heleniifolium internal transcribed spacer 1, 5.8S ribosomal RNA gene, and internal transcribed spacer 2, complete sequence	1009	1009	86% 0.0	94% DQ310945.1	1102,86	78,2%
Select seq gb|DQ310925.1|	Klasea khuzistanica from Iran internal transcribed spacer 1, 5.8S ribosomal RNA gene, and internal transcribed spacer 2, complete sequence	1009	1009	86% 0.0	94% DQ310925.1	1102,86	78,2%
Select seq gb|DQ310946.1|	Rhaponticum cynaroides from Spain internal transcribed spacer 1, 5.8S ribosomal RNA gene, and internal transcribed spacer 2, complete sequence	1007	1007	86% 0.0	94% DQ310946.1	1100,67	78,0%
Select seq gb|DQ310936.1|	Rhaponticum aulieatense from Kyrgyzstan internal transcribed spacer 1, 5.8S ribosomal RNA gene, and internal transcribed spacer 2, complete sequence	1007	1007	86% 0.0	94% DQ310936.1	1100,67	78,0%
Select seq gb|DQ310922.1|	Rhaponticoides hajastana from Armenia internal transcribed spacer 1, 5.8S ribosomal RNA gene, and internal transcribed spacer 2, complete sequence	1000	1000	86% 0.0	94% DQ310922.1	1093,02	77,5%
Select seq gb|FJ969854.1|	Plectocephalus rothrockii 18S ribosomal RNA gene, partial sequence; internal transcribed spacer 1, 5.8S ribosomal RNA gene, and internal transcribed spacer 2, complete sequence; and 28S ribosomal RNA gene, partial sequence	1027	1027	90% 0.0	94% FJ969854.1	1072,64	76,0%
Select seq gb|FJ980340.1|	Rhaponticum uniflorum voucher PS0674MT01 18S ribosomal RNA gene, partial sequence; internal transcribed spacer 1, 5.8S ribosomal RNA gene, and internal transcribed spacer 2, complete sequence; and 28S ribosomal RNA gene, partial sequence	1083	1083	95% 0.0	94% FJ980340.1	1071,60	76,0%
Select seq gb|FJ969855.1|	Centaurea stoebe subsp. micranthos 18S ribosomal RNA gene, partial sequence; internal transcribed spacer 1, 5.8S ribosomal RNA gene, and internal transcribed spacer 2, complete sequence; and 28S ribosomal RNA gene, partial sequence	1094	1094	96% 0.0	94% FJ969855.1	1071,21	75,9%
Select seq gb|GU969621.1|	Centaurea cyanus voucher ALTA:M. Gwizdala s.n. internal transcribed spacer 1, partial sequence; 5.8S ribosomal RNA gene, complete sequence; and internal transcribed spacer 2, partial sequence	1029	1029	92% 0.0	93% GU969621.1	1040,18	73,7%

Select for downloading or viewing reports	Kh028_ITS Description	Max score	Total score	Query cover	E value	Ident	Accession	(Ident/Cover)* Max score	Deviation from top hit
Select seq gb|KC709353.1|	Hypericum scabrum isolate C91 internal transcribed spacer 1, partial sequence; 5.8S ribosomal RNA gene, complete sequence; and internal transcribed spacer 2, partial sequence	1136	1136	94%	0.0	100%	KC709353.1	1208,51	100,0%
Select seq emb|HE653624.1|	Hypericum scabrum genomic DNA containing ITS1, 5.8S rRNA gene, ITS2, specimen voucher K. Sutory 110 (BM)	1168	1168	98%	0.0	99%	HE653624.1	1179,92	97,6%
Select seq emb|HE653625.1|	Hypericum scabrum genomic DNA containing ITS1, 5.8S rRNA gene, ITS2, specimen voucher Cultivated 1995-3560 (Kew-Wakehurst)	1162	1162	98%	0.0	99%	HE653625.1	1173,86	97,1%
Select seq emb|HE653594.1|	Hypericum pseudolaeve genomic DNA containing ITS1, 5.8S rRNA gene, ITS2, specimen voucher J. Roper 68 (BM)	1151	1151	98%	0.0	99%	HE653594.1	1162,74	96,2%
Select seq emb|HE653645.1|	Hypericum thymbrifolium genomic DNA containing ITS1, 5.8S rRNA gene, ITS2, specimen voucher A. Cubukcu 3 (BM)	1136	1136	98%	0.0	99%	HE653645.1	1147,59	95,0%
Select seq emb|HE653463.1|	Hypericum elongatum genomic DNA containing ITS1, 5.8S rRNA gene, ITS2, specimen voucher K. Sutory 112 (BM)	1134	1134	98%	0.0	99%	HE653463.1	1145,57	94,8%
Select seq emb|HE653443.1|	Hypericum confertum genomic DNA containing ITS1, 5.8S rRNA gene, ITS2, specimen voucher Himmetoglu H22 (BM)	1133	1133	98%	0.0	99%	HE653443.1	1144,56	94,7%
Select seq gb|KC709352.1|	Hypericum linarioides isolate C88 internal transcribed spacer 1, partial sequence; 5.8S ribosomal RNA gene, complete sequence; and internal transcribed spacer 2, partial sequence	1086	1086	94%	0.0	99%	KC709352.1	1143,77	94,6%
Select seq gb|KC709344.1|	Hypericum helianthemoides isolate C77 internal transcribed spacer 1, partial sequence; 5.8S ribosomal RNA gene, complete sequence; and internal transcribed spacer 2, partial sequence	1085	1085	94%	0.0	99%	KC709344.1	1142,71	94,6%
Select seq emb|HE653533.1|	Hypericum linarioides genomic DNA containing ITS1, 5.8S rRNA gene, ITS2, specimen voucher P. Hein 64 (BM)	1129	1129	98%	0.0	99%	HE653533.1	1140,52	94,4%
Select seq gb|KC709381.1|	Hypericum confertum isolate C138 internal transcribed spacer 1, partial sequence; 5.8S ribosomal RNA gene, complete sequence; and internal transcribed spacer 2, partial sequence	1079	1079	94%	0.0	98%	KC709381.1	1124,91	93,1%
Select seq gb|KC709336.1|	Hypericum hirsutum isolate C59 internal transcribed spacer 1, partial sequence; 5.8S ribosomal RNA gene, complete sequence; and internal transcribed spacer 2, partial sequence	1079	1079	94%	0.0	98%	KC709336.1	1124,91	93,1%
Select seq emb|HE662738.1|	Hypericum scabroides genomic DNA containing ITS1, 5.8S rRNA gene and ITS2, specimen voucher C. Scheriau HEID-808412 (HEID), clone #1	1123	1123	98%	0.0	98%	HE662738.1	1123,00	92,9%
Select seq emb|HE662737.1|	Hypericum davisii genomic DNA containing ITS1, 5.8S rRNA gene and ITS2, specimen voucher A. Cubukcu s.n. (1978) (BM), clone #8	1120	1120	98%	0.0	98%	HE662737.1	1120,00	92,7%
Select seq emb|HE662736.1|	Hypericum davisii genomic DNA containing ITS1, 5.8S rRNA gene and ITS2, specimen voucher A. Cubukcu s.n. (1978) (BM), clone #7	1120	1120	98%	0.0	98%	HE662736.1	1120,00	92,7%
Select seq emb|HE662732.1|	Hypericum davisii genomic DNA containing ITS1, 5.8S rRNA gene and ITS2, specimen voucher A. Cubukcu s.n. (1978) (BM), clone #3	1120	1120	98%	0.0	98%	HE662732.1	1120,00	92,7%
Select seq emb|HE662739.1|	Hypericum scabroides genomic DNA containing ITS1, 5.8S rRNA gene and ITS2, specimen voucher C. Scheriau HEID-808412 (HEID), clone #2	1118	1118	98%	0.0	98%	HE662739.1	1118,00	92,5%
Select seq emb|HE653622.1|	Hypericum scabroides genomic DNA containing ITS1, 5.8S rRNA gene, ITS2, specimen voucher C. Scheriau HEID-808410 (HEID)	1116	1116	98%	0.0	98%	HE653622.1	1116,00	92,3%
Select seq emb|HE653499.1|	Hypericum hirsutum genomic DNA containing ITS1, 5.8S rRNA gene, ITS2, specimen voucher F. Dvorak 1648 (HEL)	1116	1116	98%	0.0	98%	HE653499.1	1116,00	92,3%
Select seq emb|HE662731.1|	Hypericum davisii genomic DNA containing ITS1, 5.8S rRNA gene and ITS2, specimen voucher A. Cubukcu s.n. (1978) (BM), clone #1	1114	1114	98%	0.0	98%	HE662731.1	1114,00	92,2%
Select seq gb|KC709347.1|	Hypericum hyssopifolium isolate C81 internal transcribed spacer 1, partial sequence; 5.8S ribosomal RNA gene, complete sequence; and internal transcribed spacer 2, partial sequence	1068	1068	94%	0.0	98%	KC709347.1	1113,45	92,1%
Select seq emb|HE662740.1|	Hypericum scabroides genomic DNA containing ITS1, 5.8S rRNA gene and ITS2, specimen voucher C. Scheriau HEID-808412 (HEID), clone #3	1112	1112	98%	0.0	98%	HE662740.1	1112,00	92,0%
Select seq emb|HE653448.1|	Hypericum davisii genomic DNA containing ITS1, 5.8S rRNA gene, ITS2, specimen voucher A. Cubukcu s.n. (1978) (BM)	1112	1112	98%	0.0	98%	HE653448.1	1112,00	92,0%
Select seq emb|HE662733.1|	Hypericum davisii genomic DNA containing ITS1, 5.8S rRNA gene and ITS2, specimen voucher A. Cubukcu s.n. (1978) (BM), clone #4	1110	1110	98%	0.0	98%	HE662733.1	1110,00	91,8%
Select seq emb|HE653510.1|	Hypericum hyssopifolium genomic DNA containing ITS1, 5.8S rRNA gene, ITS2, specimen voucher B. de Retz 67577 (BM)	1110	1110	98%	0.0	98%	HE653510.1	1110,00	91,8%
Select seq emb|HE662734.1|	Hypericum davisii genomic DNA containing ITS1, 5.8S rRNA gene and ITS2, specimen voucher A. Cubukcu s.n. (1978) (BM), clone #5	1107	1107	98%	0.0	98%	HE662734.1	1107,00	91,6%

Select seq emb|HE653623.1|	Hypericum scabroides genomic DNA containing ITS1, 5.8S rRNA gene, ITS2, specimen voucher C. Scheriau HEID-808412 (HEID)	1107	1107	98% 0.0	98% HE653623.1	1107,00	91,6%
Select seq emb|HE653498.1|	Hypericum hirsutum genomic DNA containing ITS1, 5.8S rRNA gene, ITS2, specimen voucher N.M. Nurk 390 (GAT)	1105	1105	98% 0.0	98% HE653498.1	1105,00	91,4%
Select seq emb|HE653500.1|	Hypericum hirsutum genomic DNA containing ITS1, 5.8S rRNA gene, ITS2, specimen voucher M.F. Fay 350 (K)	1103	1103	98% 0.0	98% HE653500.1	1103,00	91,3%
Select seq gb|KC709342.1|	Hypericum callithyrsum isolate C74 internal transcribed spacer 1, partial sequence; 5.8S ribosomal RNA gene, complete sequence; and internal transcribed spacer 2, partial sequence	1057	1057	94% 0.0	98% KC709342.1	1101,98	91,2%
Select seq emb|HE662735.1|	Hypericum davisii genomic DNA containing ITS1, 5.8S rRNA gene and ITS2, specimen voucher A. Cubukcu s.n. (1978) (BM), clone #6	1098	1098	98% 0.0	98% HE662735.1	1098,00	90,9%
Select seq emb|HE653539.1|	Hypericum marginatum genomic DNA containing ITS1, 5.8S rRNA gene, ITS2, specimen voucher Davis Hedge D.32436 (BM)	1040	1040	98% 0.0	96% HE653539.1	1018,78	84,3%
Select seq emb|HE653556.1|	Hypericum nummularioides genomic DNA containing ITS1, 5.8S rRNA gene, ITS2, specimen voucher C.R. Lancaster s.n. 1.viii.1979 (BM)	1031	1031	98% 0.0	96% HE653556.1	1009,96	83,6%
Select seq gb|FJ694219.1|	Hypericum pulchrum voucher Caen FB 1257 18S ribosomal RNA gene, partial sequence; internal transcribed spacer 1, 5.8S ribosomal RNA gene, and internal transcribed spacer 2, complete sequence; and 26S ribosomal RNA gene, partial sequence	1037	1037	99% 0.0	96% FJ694219.1	1005,58	83,2%
Select seq emb|HE653567.1|	Hypericum pallens genomic DNA containing ITS1, 5.8S rRNA gene, ITS2, specimen voucher C. Scheriau HEID-801626 (HEID)	1024	1024	98% 0.0	96% HE653567.1	1003,10	83,0%
Select seq gb|KC709417.1|	Hypericum humifusum isolate C201 internal transcribed spacer 1, partial sequence; 5.8S ribosomal RNA gene, complete sequence; and internal transcribed spacer 2, partial sequence	1044	1044	100% 0.0	96% KC709417.1	1002,24	82,9%
Select seq emb|HE653568.1|	Hypericum pallens genomic DNA containing ITS1, 5.8S rRNA gene, ITS2, specimen voucher Cultivated 1945-31202 (Kew-Wakehurst)	1018	1018	98% 0.0	96% HE653568.1	997,22	82,5%
Select seq emb|HE653598.1|	Hypericum pulchrum genomic DNA containing ITS1, 5.8S rRNA gene, ITS2, specimen voucher S.L. Jury M.F. Watson 6219 (BM)	1014	1014	97% 0.0	95% HE653598.1	993,09	82,2%
Select seq emb|HE653505.1|	Hypericum humifusum genomic DNA containing ITS1, 5.8S rRNA gene, ITS2, specimen voucher Carine Ait Lafkih Rumsey Rutherford 262 (BM)	1022	1022	98% 0.0	95% HE653505.1	990,71	82,0%
Select seq emb|HE653600.1|	Hypericum pulchrum genomic DNA containing ITS1, 5.8S rRNA gene, ITS2, specimen voucher M.F. Fay 298 (K)	1011	1011	97% 0.0	95% HE653600.1	990,15	81,9%
Select seq emb|HE653532.1|	Hypericum linariifolium genomic DNA containing ITS1, 5.8S rRNA gene, ITS2, specimen voucher N.M. Nurk 379 (GAT)	1020	1020	98% 0.0	95% HE653532.1	988,78	81,8%
Select seq gb|FJ694212.1|	Hypericum olympicum voucher FB 1237 18S ribosomal RNA gene, partial sequence; internal transcribed spacer 1, 5.8S ribosomal RNA gene, and internal transcribed spacer 2, complete sequence; and 26S ribosomal RNA gene, partial sequence	1029	1029	99% 0.0	95% FJ694212.1	987,42	81,7%
Select seq emb|HE662744.1|	Hypericum reflexum genomic DNA containing ITS1, 5.8S rRNA gene and ITS2, specimen voucher F. Blattner FRB-2008-002 (GAT), clone #4	1018	1018	98% 0.0	95% HE662744.1	986,84	81,7%
Select seq gb|KC709420.1|	Hypericum humifusum isolate C204 internal transcribed spacer 1, partial sequence; 5.8S ribosomal RNA gene, complete sequence; and internal transcribed spacer 2, partial sequence	1038	1038	100% 0.0	95% KC709420.1	986,10	81,6%
Select seq gb|FJ694206.1|	Hypericum humifusum voucher Nan FB 1245 18S ribosomal RNA gene, partial sequence; internal transcribed spacer 1, 5.8S ribosomal RNA gene, and internal transcribed spacer 2, complete sequence; and 26S ribosomal RNA gene, partial sequence	1038	1038	100% 0.0	95% FJ694206.1	986,10	81,6%
Select seq gb|AF455674.1|AF455674	Hypericum perforatum 18S ribosomal RNA gene, partial sequence; internal transcribed spacer 1, 5.8S ribosomal RNA gene and internal transcribed spacer 2, complete sequence; and 26S ribosomal RNA gene, partial sequence	1038	1038	100% 0.0	95% AF455674.1	986,10	81,6%
Select seq emb|HE653509.1|	Hypericum humifusum genomic DNA containing ITS1, 5.8S rRNA gene, ITS2, specimen voucher N.M. Nurk 381 (GAT)	1016	1016	98% 0.0	95% HE653509.1	984,90	81,5%
Select seq emb|HE653506.1|	Hypericum humifusum genomic DNA containing ITS1, 5.8S rRNA gene, ITS2, specimen voucher K. Harris s.n. 1988 (BM)	1016	1016	98% 0.0	95% HE653506.1	984,90	81,5%
Select seq gb|FJ694196.1|	Hypericum coris voucher Boch FB 1252 18S ribosomal RNA gene, partial sequence; internal transcribed spacer 1, 5.8S ribosomal RNA gene, and internal transcribed spacer 2, complete sequence; and 26S ribosomal RNA gene, partial sequence	1035	1035	100% 0.0	95% FJ694196.1	983,25	81,4%
Select seq emb|HE653490.1|	Hypericum haplophylloides genomic DNA containing ITS1, 5.8S rRNA gene, ITS2, specimen voucher F.K. Meyer 5973 (BM)	1003	1003	97% 0.0	95% HE653490.1	982,32	81,3%
Select seq emb|HE662729.1|	Hypericum polyphyllum genomic DNA containing ITS1, 5.8S rRNA gene and ITS2, specimen voucher N.M. Nurk 407 (GAT), clone #6	1013	1013	98% 0.0	95% HE662729.1	981,99	81,3%
Select seq emb|HE653621.1|	Hypericum saxifragum genomic DNA containing ITS1, 5.8S rRNA gene, ITS2, specimen voucher R. Ulrich s.n. 6.10.1997 (BM)	1013	1013	98% 0.0	95% HE653621.1	981,99	81,3%
Select seq emb|HE653445.1|	Hypericum coris genomic DNA containing ITS1, 5.8S rRNA gene, ITS2, specimen voucher S.L. Jury M.F. Watson D.A. Webb M. B. Wyse Jackson 6415 (BM)	1013	1013	98% 0.0	95% HE653445.1	981,99	81,3%

Select seq gb|JN811130.1|	Hypericum perforatum voucher MSB<DEU>:002597 internal transcribed spacer 1, partial sequence; 5.8S ribosomal RNA gene and internal transcribed spacer 2, complete sequence; and 26S ribosomal RNA gene, partial sequence	1011	1011	98%	0.0	95%	JN811130.1	980,05	81,1%
Select seq emb|HE653583.1|	Hypericum petiolulatum subsp. yunnanense genomic DNA containing ITS1, 5.8S rRNA gene, ITS2, specimen voucher B. Bartholomew D.E. Boufford Q.H. Chen et al. 2163 (BM)	1011	1011	98%	0.0	95%	HE653583.1	980,05	81,1%
Select seq emb|HE653507.1|	Hypericum humifusum genomic DNA containing ITS1, 5.8S rRNA gene, ITS2, specimen voucher J.F. Veldkamp 8837 (BM)	1011	1011	98%	0.0	95%	HE653507.1	980,05	81,1%
Select seq emb|HE653420.1|	Hypericum barbatum genomic DNA containing ITS1, 5.8S rRNA gene, ITS2, specimen voucher N.M. Nurk 410 (GAT)	1011	1011	98%	0.0	95%	HE653420.1	980,05	81,1%
Select seq emb|HE653446.1|	Hypericum coris genomic DNA containing ITS1, 5.8S rRNA gene, ITS2, specimen voucher R.E. Longton 4436 (BM)	1009	1009	98%	0.0	95%	HE653446.1	978,11	80,9%
Select seq emb|HE662726.1|	Hypericum polyphyllum genomic DNA containing ITS1, 5.8S rRNA gene and ITS2, specimen voucher N.M. Nurk 407 (GAT), clone #1	1007	1007	98%	0.0	95%	HE662726.1	976,17	80,8%
Select seq emb|HE662724.1|	Hypericum undulatum subsp. undulatum genomic DNA containing ITS1, 5.8S rRNA gene and ITS2, specimen voucher D.J. Goyer S.L. Jury 545 (BM), clone #7	1007	1007	98%	0.0	95%	HE662724.1	976,17	80,8%
Select seq emb|HE653561.1|	Hypericum olympicum genomic DNA containing ITS1, 5.8S rRNA gene, ITS2, specimen voucher D. McClintock s.n. 1983 (BM)	1007	1007	98%	0.0	95%	HE653561.1	976,17	80,8%
Select seq emb|HE653471.1|	Hypericum ericoides genomic DNA containing ITS1, 5.8S rRNA gene, ITS2, specimen voucher P.F. Cannon P.R. Crane S.R. Jury D.M. Moore (R.U. Botany Dept. Exped.) 475 (BM)	1007	1007	98%	0.0	95%	HE653471.1	976,17	80,8%
Select seq emb|HE653464.1|	Hypericum empetrifolium genomic DNA containing ITS1, 5.8S rRNA gene, ITS2, specimen voucher R.C. Lancaster 1118 (BM)	1007	1007	98%	0.0	95%	HE653464.1	976,17	80,8%
Select seq gb|FJ694192.1|	Hypericum barbatum voucher Kew FB 1248 18S ribosomal RNA gene, partial sequence; internal transcribed spacer 1, 5.8S ribosomal RNA gene, and internal transcribed spacer 2, complete sequence; and 26S ribosomal RNA gene, partial sequence	1027	1027	100%	0.0	95%	FJ694192.1	975,65	80,7%
Select seq gb|KC709415.1|	Hypericum olympicum isolate C199 internal transcribed spacer 1, partial sequence; 5.8S ribosomal RNA gene, complete sequence; and internal transcribed spacer 2, partial sequence	1016	1016	99%	0.0	95%	KC709415.1	974,95	80,7%
Select seq gb|JN811139.1|	Hypericum undulatum voucher HEID:808335 internal transcribed spacer 1, partial sequence; 5.8S ribosomal RNA gene and internal transcribed spacer 2, complete sequence; and 26S ribosomal RNA gene, partial sequence	1005	1005	98%	0.0	95%	JN811139.1	974,23	80,6%
Select seq emb|HE653589.1|	Hypericum polyphyllum genomic DNA containing ITS1, 5.8S rRNA gene, ITS2, specimen voucher N.M. Nurk 391 (GAT)	1005	1005	98%	0.0	95%	HE653589.1	974,23	80,6%
Select seq emb|HE653582.1|	Hypericum petiolulatum genomic DNA containing ITS1, 5.8S rRNA gene, ITS2, specimen voucher A.J.C. Grierson D.G. Long 2549 (BM)	1005	1005	98%	0.0	95%	HE653582.1	974,23	80,6%
Select seq gb|JN811125.1|	Hypericum perforatum voucher HEID:704624 internal transcribed spacer 1, partial sequence; 5.8S ribosomal RNA gene and internal transcribed spacer 2, complete sequence; and 26S ribosomal RNA gene, partial sequence	1005	1005	98%	0.0	95%	JN811125.1	974,23	80,6%
Select seq gb|EU796888.1|	Hypericum perforatum 18S ribosomal RNA gene, partial sequence; internal transcribed spacer 1, 5.8S ribosomal RNA gene, and internal transcribed spacer 2, complete sequence; and 26S ribosomal RNA gene, partial sequence	1024	1024	100%	0.0	95%	EU796888.1	972,80	80,5%
Select seq emb|HE653564.1|	Hypericum olympicum genomic DNA containing ITS1, 5.8S rRNA gene, ITS2, specimen voucher N.M. Nurk 387 (GAT)	1003	1003	98%	0.0	95%	HE653564.1	972,30	80,5%
Select seq gb|KC709424.1|	Hypericum montanum isolate C208 internal transcribed spacer 1, partial sequence; 5.8S ribosomal RNA gene, complete sequence; and internal transcribed spacer 2, partial sequence	1013	1013	99%	0.0	95%	KC709424.1	972,07	80,4%
Select seq gb|FJ694211.1|	Hypericum montanum voucher FB 1229 18S ribosomal RNA gene, partial sequence; internal transcribed spacer 1, 5.8S ribosomal RNA gene, and internal transcribed spacer 2, complete sequence; and 26S ribosomal RNA gene, partial sequence	1013	1013	99%	0.0	95%	FJ694211.1	972,07	80,4%
Select seq gb|FJ694203.1|	Hypericum hirsutum voucher FB 1228 18S ribosomal RNA gene, partial sequence; internal transcribed spacer 1, 5.8S ribosomal RNA gene, and internal transcribed spacer 2, complete sequence; and 26S ribosomal RNA gene, partial sequence	1013	1013	99%	0.0	95%	FJ694203.1	972,07	80,4%
Select seq gb|JN811126.1|	Hypericum perforatum voucher HEID:803959 internal transcribed spacer 1, partial sequence; 5.8S ribosomal RNA gene and internal transcribed spacer 2, complete sequence; and 26S ribosomal RNA gene, partial sequence	1002	1002	98%	0.0	95%	JN811126.1	971,33	80,4%
Select seq emb|HE653654.1|	Hypericum undulatum genomic DNA containing ITS1, 5.8S rRNA gene, ITS2, specimen voucher C. Scheriau HEID-808335 (HEID)	1002	1002	98%	0.0	95%	HE653654.1	971,33	80,4%
Select seq gb|JN984883.1|	Hypericum erectum var. caespitosum isolate LeeH8 18S ribosomal RNA gene, partial sequence; internal transcribed spacer 1, 5.8S ribosomal RNA gene, and internal transcribed spacer 2, complete sequence; and 26S ribosomal RNA gene, partial sequence	1022	1022	100%	0.0	95%	JN984883.1	970,90	80,3%
Select seq gb|FJ793046.1|	Hypericum yezoense voucher TSU FB 1265 18S ribosomal RNA gene, partial sequence; internal transcribed spacer 1, 5.8S ribosomal RNA gene, and internal transcribed spacer 2, complete sequence; and 26S ribosomal RNA gene, partial sequence	1022	1022	100%	0.0	95%	FJ793046.1	970,90	80,3%
Select seq gb|FJ694199.1|	Hypericum dubium voucher Mei FB 1253 18S ribosomal RNA gene, partial sequence; internal transcribed spacer 1, 5.8S ribosomal RNA gene, and internal transcribed spacer 2, complete sequence; and 26S ribosomal RNA gene, partial sequence	1022	1022	100%	0.0	95%	FJ694199.1	970,90	80,3%
Select seq gb|FJ694222.1|	Hypericum richeri subsp. grisebachii voucher FB 1230 18S ribosomal RNA gene, partial sequence; internal transcribed spacer 1, 5.8S ribosomal RNA gene, and internal transcribed spacer 2, complete sequence; and 26S ribosomal RNA gene, partial sequence	1022	1022	100%	0.0	95%	FJ694222.1	970,90	80,3%
Select seq gb|FJ694216.1|	Hypericum polyphyllum voucher Sia FB 1234 18S ribosomal RNA gene, partial sequence; internal transcribed spacer 1, 5.8S ribosomal RNA gene, and internal transcribed spacer 2, complete sequence; and 26S ribosomal RNA gene, partial sequence	1020	1020	100%	0.0	95%	FJ694216.1	969,00	80,2%

Select seq gb|FJ793043.1|	Hypericum attenuatum voucher Gat FB 1263 18S ribosomal RNA gene, partial sequence; internal transcribed spacer 1, 5.8S ribosomal RNA gene, and internal transcribed spacer 2, complete sequence; and 26S ribosomal RNA gene, partial sequence	1016	1016	100% 0.0	95% FJ793043.1	965,20	79,9%
Select seq gb|FJ694220.1|	Hypericum reptans voucher Ber FB 1258 18S ribosomal RNA gene, partial sequence; internal transcribed spacer 1, 5.8S ribosomal RNA gene, and internal transcribed spacer 2, complete sequence; and 26S ribosomal RNA gene, partial sequence	1016	1016	100% 0.0	95% FJ694220.1	965,20	79,9%
Select seq gb|FJ694215.1|	Hypericum perforatum voucher FB 1225 18S ribosomal RNA gene, partial sequence; internal transcribed spacer 1, 5.8S ribosomal RNA gene, and internal transcribed spacer 2, complete sequence; and 26S ribosomal RNA gene, partial sequence	1016	1016	100% 0.0	95% FJ694215.1	965,20	79,9%
Select seq emb|LK871661.1|	Hypericum perforatum subsp. veronense genomic DNA containing ITS1, 5.8S rRNA gene and ITS2, specimen voucher S.L. Jury and M. Ait Lafkih & B. Tahiri-14865-BM	994	994	98% 0.0	95% LK871661.1	963,57	79,7%
Select seq gb|KC709422.1|	Hypericum maculatum subsp. maculatum isolate C206 internal transcribed spacer 1, partial sequence; 5.8S ribosomal RNA gene, complete sequence; and internal transcribed spacer 2, partial sequence	1011	1011	100% 0.0	95% KC709422.1	960,45	79,5%
Select seq gb|KC709421.1|	Hypericum nummularium isolate C205 internal transcribed spacer 1, partial sequence; 5.8S ribosomal RNA gene, complete sequence; and internal transcribed spacer 2, partial sequence	1011	1011	100% 0.0	95% KC709421.1	960,45	79,5%
Select seq gb|GU596502.1|	Hypericum perforatum 18S ribosomal RNA gene, partial sequence; internal transcribed spacer 1, 5.8S ribosomal RNA gene, and internal transcribed spacer 2, complete sequence; and 26S ribosomal RNA gene, partial sequence	1011	1011	100% 0.0	95% GU596502.1	960,45	79,5%
Select seq gb|FJ694198.1|	Hypericum drummondii voucher Sp FB 1261 18S ribosomal RNA gene, partial sequence; internal transcribed spacer 1, 5.8S ribosomal RNA gene, and internal transcribed spacer 2, complete sequence; and 26S ribosomal RNA gene, partial sequence	1011	1011	100% 0.0	95% FJ694198.1	960,45	79,5%
Select seq gb|FJ694197.1|	Hypericum delphicum voucher M-L FB 1232 18S ribosomal RNA gene, partial sequence; internal transcribed spacer 1, 5.8S ribosomal RNA gene, and internal transcribed spacer 2, complete sequence; and 26S ribosomal RNA gene, partial sequence	1011	1011	100% 0.0	95% FJ694197.1	960,45	79,5%
Select seq gb|GQ868706.1|	Hypericum perforatum 18S ribosomal RNA gene, partial sequence; internal transcribed spacer 1, 5.8S ribosomal RNA gene, and internal transcribed spacer 2, complete sequence; and 26S ribosomal RNA gene, partial sequence	1011	1011	100% 0.0	95% GQ868706.1	960,45	79,5%
Select seq gb|KJ123872.1|	Hypericum peplidifolium isolate C28 18S ribosomal RNA gene, partial sequence; internal transcribed spacer 1, 5.8S ribosomal RNA gene, and internal transcribed spacer 2, complete sequence; and 28S ribosomal RNA gene, partial sequence	1007	1007	100% 0.0	95% KJ123872.1	956,65	79,2%
Select seq gb|KC709463.1|	Hypericum scioanum isolate C29c16 internal transcribed spacer 1, partial sequence; 5.8S ribosomal RNA gene, complete sequence; and internal transcribed spacer 2, partial sequence	1007	1007	100% 0.0	95% KC709463.1	956,65	79,2%
Select seq gb|KC709460.1|	Hypericum scioanum isolate C29c13 internal transcribed spacer 1, partial sequence; 5.8S ribosomal RNA gene, complete sequence; and internal transcribed spacer 2, partial sequence	1007	1007	100% 0.0	95% KC709460.1	956,65	79,2%
Select seq gb|FJ694223.1|	Hypericum stragulum voucher Sp FB 1259 18S ribosomal RNA gene, partial sequence; internal transcribed spacer 1, 5.8S ribosomal RNA gene, and internal transcribed spacer 2, complete sequence; and 26S ribosomal RNA gene, partial sequence	1005	1005	100% 0.0	95% FJ694223.1	954,75	79,0%
Select seq gb|FJ694207.1|	Hypericum hypericoides voucher Sp FB 1260 18S ribosomal RNA gene, partial sequence; internal transcribed spacer 1, 5.8S ribosomal RNA gene, and internal transcribed spacer 2, complete sequence; and 26S ribosomal RNA gene, partial sequence	1005	1005	100% 0.0	95% FJ694207.1	954,75	79,0%
Select seq gb|JN984884.1|	Hypericum oliganthum isolate LeeH9 18S ribosomal RNA gene, partial sequence; internal transcribed spacer 1, 5.8S ribosomal RNA gene, and internal transcribed spacer 2, complete sequence; and 26S ribosomal RNA gene, partial sequence	1005	1005	100% 0.0	94% JN984884.1	944,70	78,2%
Select seq gb|KC709461.1|	Hypericum scioanum isolate C29c14 internal transcribed spacer 1, partial sequence; 5.8S ribosomal RNA gene, complete sequence; and internal transcribed spacer 2, partial sequence	1002	1002	100% 0.0	94% KC709461.1	941,88	77,9%
Select seq gb|KC709458.1|	Hypericum scioanum isolate C29c11 internal transcribed spacer 1, partial sequence; 5.8S ribosomal RNA gene, complete sequence; and internal transcribed spacer 2, partial sequence	1002	1002	100% 0.0	94% KC709458.1	941,88	77,9%

Select for downloading or viewing reports	Kh028_trnL Description	Max score	Total score	Query cover	E value	Ident	Accession	(Ident/Cover)* Max score	Deviation from top hit
Select seq gb|KC709040.1|	Hypericum callithyrsum isolate C74 trnL-trnF intergenic spacer, partial sequence; chloroplast	318	318	27%	8,00E-83	97%	KC709040.1	0,00	#DIV/0!
Select seq gb|JF734851.1|	Hypericum oliganthum isolate Hyp4348 tRNA-Leu (trnL-UAA) gene, partial sequence; chloroplast	361	361	31%	1,00E-95	97%	JF734851.1	0,00	#DIV/0!
Select seq gb|JF734847.1|	Hypericum erectum isolate Hyp4331 tRNA-Leu (trnL-UAA) gene, partial sequence; chloroplast	361	361	31%	1,00E-95	97%	JF734847.1	0,00	#DIV/0!
Select seq gb|JF734849.1|	Hypericum pibairense isolate Hyp4341 tRNA-Leu (trnL-UAA) gene, partial sequence; chloroplast	355	355	31%	6,00E-94	97%	JF734849.1	0,00	#DIV/0!
Select seq gb|JF734848.1|	Hypericum gracillimum isolate Hyp4330 tRNA-Leu (trnL-UAA) gene, partial sequence; chloroplast	355	355	31%	6,00E-94	97%	JF734848.1	0,00	#DIV/0!
Select seq emb|LK871771.1|	Hypericum nagasawai chloroplast DNA containing trnL(UAA)-trnF(GAA) IGS, specimen voucher C.-K. Liou et al.-821-PE	387	387	34%	2,00E-103	97%	LK871771.1	0,00	#DIV/0!
Select seq emb|LK871749.1|	Hypericum bequaertii chloroplast DNA containing trnL(UAA)-trnF(GAA) IGS, specimen voucher B. Gehrke-BG367-Z	383	383	34%	3,00E-102	96%	LK871749.1	0,00	#DIV/0!
Select seq gb|JF734853.1|	Hypericum perforatum isolate Hyp0215 tRNA-Leu (trnL-UAA) gene, partial sequence; chloroplast	344	344	31%	1,00E-90	96%	JF734853.1	0,00	#DIV/0!
Select seq gb|JF734852.1|	Hypericum perforatum isolate Hyp0065 tRNA-Leu (trnL-UAA) gene, partial sequence; chloroplast	344	344	31%	1,00E-90	96%	JF734852.1	0,00	#DIV/0!
Select seq gb|JF734846.1|	Hypericum attenuatum isolate Hyp4260 tRNA-Leu (trnL-UAA) gene, partial sequence; chloroplast	344	344	31%	1,00E-90	96%	JF734846.1	0,00	#DIV/0!
Select seq emb|LK871765.1|	Hypericum kiboense chloroplast DNA containing trnL(UAA)-trnF(GAA) IGS, specimen voucher B. Gehrke-BG149C-Z	355	355	32%	6,00E-94	96%	LK871765.1	0,00	#DIV/0!
Select seq gb|KC709046.1|	Hypericum revolutum isolate C82 trnL-trnF intergenic spacer, partial sequence; chloroplast	298	298	27%	1,00E-76	95%	KC709046.1	0,00	#DIV/0!
Select seq gb|KC709131.1|	Hypericum roeperianum isolate C233 trnL-trnF intergenic spacer, partial sequence; chloroplast	305	305	28%	6,00E-79	96%	KC709131.1	0,00	#DIV/0!
Select seq gb|KC709125.1|	Hypericum revolutum subsp. revolutum isolate C213 trnL-trnF intergenic spacer, partial sequence; chloroplast	305	305	28%	6,00E-79	96%	KC709125.1	0,00	#DIV/0!
Select seq gb|KC709023.1|	Hypericum perforatum isolate C47 trnL-trnF intergenic spacer, partial sequence; chloroplast	296	296	27%	4,00E-76	95%	KC709023.1	0,00	#DIV/0!
Select seq gb|JF734854.1|	Hypericum tetrapterum isolate Hyp0538 tRNA-Leu (trnL-UAA) gene, partial sequence; chloroplast	339	339	31%	6,00E-89	95%	JF734854.1	0,00	#DIV/0!
Select seq emb|LK871747.1|	Hypericum ascyron subsp. pyramidatum chloroplast DNA containing trnL(UAA)-trnF(GAA) IGS, specimen voucher Ames-28349-ISU	346	346	32%	4,00E-91	95%	LK871747.1	0,00	#DIV/0!
Select seq gb|JF734850.1|	Hypericum maculatum isolate Hyp0222 tRNA-Leu (trnL-UAA) gene, partial sequence; chloroplast	333	333	31%	3,00E-87	95%	JF734850.1	0,00	#DIV/0!
Select seq gb|HM590302.1|	Hypericum maculatum voucher personal collection:I. Hiiesalu 37 tRNA-Leu (trnL) gene, partial sequence; chloroplast	333	333	31%	3,00E-87	95%	HM590302.1	0,00	#DIV/0!
Select seq gb|KC709129.1|	Hypericum quartinianum isolate C224 trnL-trnF intergenic spacer, partial sequence; chloroplast	300	300	28%	3,00E-77	95%	KC709129.1	0,00	#DIV/0!
Select seq gb|KC709016.1|	Hypericum quartinianum isolate C32 trnL-trnF intergenic spacer, partial sequence; chloroplast	300	300	28%	3,00E-77	95%	KC709016.1	0,00	#DIV/0!
Select seq dbj|AB219639.1|	Hypericum kamtschaticum chloroplast trnL(UAA), trnF(GAA) genes for tRNA-Leu, tRNA-Phe, partial sequence, trnL-trnF spacer region, specimen_voucher: MAK:K. Senni S2558	675	675	64%	0.0	95%	AB219639.1	0,00	#DIV/0!
Select seq gb|KC709155.1|	Hypericum curvisepalum isolate C303 trnL-trnF intergenic spacer, partial sequence; chloroplast	294	294	28%	1,00E-75	95%	KC709155.1	0,00	#DIV/0!
Select seq gb|KC709154.1|	Hypericum acmosepalum isolate C302 trnL-trnF intergenic spacer, partial sequence; chloroplast	294	294	28%	1,00E-75	95%	KC709154.1	0,00	#DIV/0!
Select seq emb|LK871752.1|	Hypericum calcicola chloroplast DNA containing trnL(UAA)-trnF(GAA) IGS, specimen voucher D.E. Breedlove & R.F. Thorne-21104-BM	346	346	33%	4,00E-91	95%	LK871752.1	0,00	#DIV/0!
Select seq dbj|AB219637.1|	Hypericum kamtschaticum chloroplast trnL(UAA), trnF(GAA) genes for tRNA-Leu, tRNA-Phe, partial sequence, trnL-trnF spacer region, specimen_voucher: MAK:N. Fujii F01715	669	669	64%	0.0	95%	AB219637.1	0,00	#DIV/0!

Select seq dbj|AB219638.1|	Hypericum kamtschaticum chloroplast trnL(UAA), trnF(GAA) genes for tRNA-Leu, tRNA-Phe, partial sequence, trnL-trnF spacer region, specimen_voucher: MAK:N. Fujii F01777	667	667	64%	0.0	95%	AB219638.1	0,00	#DIV/0!
Select seq gb|KC709143.1|	Hypericum pseudolaeve isolate C276 trnL-trnF intergenic spacer, partial sequence; chloroplast	333	397	33%	3,00E-87	98%	KC709143.1	0,00	#DIV/0!
Select seq emb|LK871748.1|	Hypericum athoum chloroplast DNA containing trnL(UAA)-trnF(GAA) IGS, specimen voucher C. Scheriau-HEID801636-HEID	675	675	65%	0.0	94%	LK871748.1	0,00	#DIV/0!
Select seq emb|LK871754.1|	Hypericum connatum chloroplast DNA containing trnL(UAA)-trnF(GAA) IGS, specimen voucher M. Serrano et al.-6893-BM	353	353	34%	2,00E-93	94%	LK871754.1	0,00	#DIV/0!
Select seq emb|LK871750.1|	Hypericum brasiliense chloroplast DNA containing trnL(UAA)-trnF(GAA) IGS, specimen voucher Gentry & Solomon-44755-BM	353	353	34%	2,00E-93	94%	LK871750.1	0,00	#DIV/0!
Select seq emb|LK871755.1|	Hypericum delphicum chloroplast DNA containing trnL(UAA)-trnF(GAA) IGS, specimen voucher C. Scheriau-HEID808395-HEID	671	671	65%	0.0	94%	LK871755.1	0,00	#DIV/0!
Select seq dbj|AB219636.1|	Hypericum kamtschaticum chloroplast trnL(UAA), trnF(GAA) genes for tRNA-Leu, tRNA-Phe, partial sequence, trnL-trnF spacer region, specimen_voucher: MAK:K. Senni S1187	658	658	64%	0.0	94%	AB219636.1	0,00	#DIV/0!
Select seq emb|LK871759.1|	Hypericum globuliferum chloroplast DNA containing trnL(UAA)-trnF(GAA) IGS, specimen voucher B. Gehrke-BG246-Z	348	348	34%	1,00E-91	94%	LK871759.1	0,00	#DIV/0!
Select seq emb|LK871772.1|	Hypericum oliganthum chloroplast DNA containing trnL(UAA)-trnF(GAA) IGS, specimen voucher T. Sawada-236-KYO	665	665	65%	0.0	94%	LK871772.1	0,00	#DIV/0!
Select seq emb|LK871762.1|	Hypericum humifusum chloroplast DNA containing trnL(UAA)-trnF(GAA) IGS, specimen voucher N.M. Nurk-381-GAT	525	525	52%	4,00E-145	94%	LK871762.1	0,00	#DIV/0!
Select seq gb|KF591260.1|	Hypericum androsaemum voucher Allan Herbarium CHR 630393 tRNA-Leu (trnL) gene and trnL-trnF intergenic spacer, partial sequence; chloroplast	333	333	33%	3,00E-87	94%	KF591260.1	0,00	#DIV/0!
Select seq emb|LK871780.1|	Hypericum silenoides chloroplast DNA containing trnL(UAA)-trnF(GAA) IGS, specimen voucher J. C. Solomon-16431-BM	342	342	34%	5,00E-90	94%	LK871780.1	0,00	#DIV/0!
Select seq emb|LK871764.1|	Hypericum kalmianum chloroplast DNA containing trnL(UAA)-trnF(GAA) IGS, specimen voucher N.M. Nurk-397-GAT	320	320	32%	2,00E-83	93%	LK871764.1	0,00	#DIV/0!
Select seq emb|LK871757.1|	Hypericum erectum chloroplast DNA containing trnL(UAA)-trnF(GAA) IGS, specimen voucher N.M. Nurk-383-BM	645	645	65%	0.0	93%	LK871757.1	0,00	#DIV/0!
Select seq emb|LK871781.1|	Hypericum tosaense chloroplast DNA containing trnL(UAA)-trnF(GAA) IGS, specimen voucher Tamiki Kobayashi-41978-KYO	643	643	65%	1,00E-180	93%	LK871781.1	0,00	#DIV/0!
Select seq emb|LK871776.1|	Hypericum perforatum subsp. veronense chloroplast DNA containing trnL(UAA)-trnF(GAA) IGS, specimen voucher S.L. Jury and M. Ait Lafkih & B. Tahiri-14865-BM	638	638	65%	5,00E-179	93%	LK871776.1	0,00	#DIV/0!
Select seq emb|LK871777.1|	Hypericum reflexum chloroplast DNA containing trnL(UAA)-trnF(GAA) IGS, specimen voucher F. Blattner-FB2008/004-GAT	623	623	64%	2,00E-174	93%	LK871777.1	0,00	#DIV/0!
Select seq emb|LK871773.1|	Hypericum pamphylicum chloroplast DNA containing trnL(UAA)-trnF(GAA) IGS, specimen voucher R. Ulrich-s.n. [1998]-BM	553	553	57%	2,00E-153	93%	LK871773.1	0,00	#DIV/0!
Select seq emb|LK871746.1|	Hypericum androsaemum chloroplast DNA containing trnL(UAA)-trnF(GAA) IGS, specimen voucher C. Scheriau-HEID808382-HEID	545	545	56%	3,00E-151	92%	LK871746.1	0,00	#DIV/0!
Select seq emb|LK871775.1|	Hypericum perforatum subsp. chinense chloroplast DNA containing trnL(UAA)-trnF(GAA) IGS, specimen voucher N.M. Nurk-353-GAT	627	627	65%	1,00E-175	92%	LK871775.1	0,00	#DIV/0!
Select seq emb|LK871774.1|	Hypericum peplidifolium chloroplast DNA containing trnL(UAA)-trnF(GAA) IGS, specimen voucher B. Gehrke-BG078-Z	307	307	32%	2,00E-79	92%	LK871774.1	0,00	#DIV/0!
Select seq emb|LK871766.1|	Hypericum lalandii chloroplast DNA containing trnL(UAA)-trnF(GAA) IGS, specimen voucher B. Gehrke-BG207-Z	536	536	56%	2,00E-148	92%	LK871766.1	0,00	#DIV/0!
Select seq emb|LK871769.1|	Hypericum maculatum chloroplast DNA containing trnL(UAA)-trnF(GAA) IGS, specimen voucher C. Scheriau-HEID808356-HEID	614	614	65%	9,00E-172	92%	LK871769.1	0,00	#DIV/0!
Select seq emb|LK871760.1|	Hypericum hircinum chloroplast DNA containing trnL(UAA)-trnF(GAA) IGS, specimen voucher C. Scheriau-HEID808404-HEID	588	588	64%	6,00E-164	91%	LK871760.1	0,00	#DIV/0!
Select seq emb|LK871768.1|	Hypericum linariifolium chloroplast DNA containing trnL(UAA)-trnF(GAA) IGS, specimen voucher N.M. Nurk-379-GAT	477	477	52%	1,00E-130	91%	LK871768.1	0,00	#DIV/0!
Select seq emb|LK871779.1|	Hypericum scioanum chloroplast DNA containing trnL(UAA)-trnF(GAA) IGS, specimen voucher B. Gehrke-BG081-Z	508	508	56%	4,00E-140	91%	LK871779.1	0,00	#DIV/0!
Select seq emb|LK871778.1|	Hypericum revolutum chloroplast DNA containing trnL(UAA)-trnF(GAA) IGS, specimen voucher B. Gehrke-BG100-Z	551	551	62%	7,00E-153	91%	LK871778.1	0,00	#DIV/0!
Select seq emb|LK871770.1|	Hypericum x mitchellianum chloroplast DNA containing trnL(UAA)-trnF(GAA) IGS, specimen voucher N.M. Nurk-382-GAT	582	582	65%	3,00E-162	90%	LK871770.1	0,00	#DIV/0!

Select seq gb|KC709006.1|	Hypericum tomentosum isolate C19 trnL-trnF intergenic spacer, partial sequence; chloroplast	311	432	38%	1,00E-80	96%	KC709006.1	0,00	#DIV/0!
Select seq emb|LK871782.1|	Hypericum triquetrifolium chloroplast DNA containing trnL(UAA)-trnF(GAA) IGS, specimen voucher J,R. Akeroyd, S.L. Jury & F.J.Rumsey-3572-BM	566	566	65%	3,00E-157	90%	LK871782.1	0,00	#DIV/0!
Select seq gb|KF267872.1|	Hypericum olympicum voucher Schneider s.n. tRNA-Leu (trnL) gene and trnL-trnF intergenic spacer, partial sequence; chloroplast	579	579	68%	3,00E-161	90%	KF267872.1	0,00	#DIV/0!
Select seq gb|KC709102.1|	Hypericum formosum isolate C175 trnL-trnF intergenic spacer, partial sequence; chloroplast	316	458	40%	3,00E-82	97%	KC709102.1	0,00	#DIV/0!
Select seq emb|LK871761.1|	Hypericum hookerianum chloroplast DNA containing trnL(UAA)-trnF(GAA) IGS, specimen voucher N.M. Nurk-413-GAT	549	549	65%	3,00E-152	90%	LK871761.1	0,00	#DIV/0!
Select seq emb|LK871753.1|	Hypericum calycinum chloroplast DNA containing trnL(UAA)-trnF(GAA) IGS, specimen voucher D. McClintock-s.n. [1993]-BM	547	547	65%	9,00E-152	90%	LK871753.1	0,00	#DIV/0!
Select seq emb|LK871763.1|	Hypericum japonicum chloroplast DNA containing trnL(UAA)-trnF(GAA) IGS, specimen voucher K. Masuda-3360-KYO	551	551	65%	7,00E-153	89%	LK871763.1	0,00	#DIV/0!
Select seq gb|KC709011.1|	Hypericum coris isolate C24 trnL-trnF intergenic spacer, partial sequence; chloroplast	300	397	38%	3,00E-77	95%	KC709011.1	0,00	#DIV/0!
Select seq gb|KC709010.1|	Hypericum coris isolate C23 trnL-trnF intergenic spacer, partial sequence; chloroplast	300	397	38%	3,00E-77	95%	KC709010.1	0,00	#DIV/0!
Select seq gb|KC709090.1|	Hypericum naudinianum isolate C157 trnL-trnF intergenic spacer, partial sequence; chloroplast	311	447	40%	1,00E-80	96%	KC709090.1	0,00	#DIV/0!
Select seq gb|KC709083.1|	Hypericum glandulosum isolate C145 trnL-trnF intergenic spacer, partial sequence; chloroplast	311	452	40%	1,00E-80	96%	KC709083.1	0,00	#DIV/0!
Select seq gb|KC709082.1|	Hypericum coadunatum isolate C144 trnL-trnF intergenic spacer, partial sequence; chloroplast	311	452	40%	1,00E-80	96%	KC709082.1	0,00	#DIV/0!
Select seq gb|KC709081.1|	Hypericum reflexum isolate C143 trnL-trnF intergenic spacer, partial sequence; chloroplast	311	452	40%	1,00E-80	96%	KC709081.1	0,00	#DIV/0!
Select seq gb|KC709005.1|	Hypericum caprifolium isolate C18 trnL-trnF intergenic spacer, partial sequence; chloroplast	311	452	40%	1,00E-80	96%	KC709005.1	0,00	#DIV/0!
Select seq gb|KC709053.1|	Hypericum scabrum isolate C91 trnL-trnF intergenic spacer, partial sequence; chloroplast	339	493	45%	6,00E-89	99%	KC709053.1	0,00	#DIV/0!
Select seq emb|LK871758.1|	Hypericum fauriei chloroplast DNA containing trnL(UAA)-trnF(GAA) IGS, specimen voucher N.M. Nurk-414-GAT	536	536	65%	2,00E-148	89%	LK871758.1	0,00	#DIV/0!
Select seq gb|KC709068.1|	Hypericum reflexum var. reflexum isolate C112 trnL-trnF intergenic spacer, partial sequence; chloroplast	305	447	40%	6,00E-79	96%	KC709068.1	0,00	#DIV/0!
Select seq gb|KC709048.1|	Hypericum montbretii isolate C84 trnL-trnF intergenic spacer, partial sequence; chloroplast	305	454	40%	6,00E-79	96%	KC709048.1	0,00	#DIV/0!
Select seq gb|KC708995.1|	Hypericum somaliense isolate C4 trnL-trnF intergenic spacer, partial sequence; chloroplast	305	447	40%	6,00E-79	96%	KC708995.1	0,00	#DIV/0!
Select seq gb|KC709018.1|	Hypericum psilophytum isolate C38 trnL-trnF intergenic spacer, partial sequence; chloroplast	311	454	41%	1,00E-80	96%	KC709018.1	0,00	#DIV/0!
Select seq gb|KC709042.1|	Hypericum helianthemoides isolate C77 trnL-trnF intergenic spacer, partial sequence; chloroplast	311	465	41%	1,00E-80	96%	KC709042.1	0,00	#DIV/0!
Select seq gb|KC709027.1|	Hypericum hirsutum isolate C59 trnL-trnF intergenic spacer, partial sequence; chloroplast	329	467	45%	4,00E-86	98%	KC709027.1	0,00	#DIV/0!
Select seq gb|KC709133.1|	Hypericum nummularioides isolate C243 trnL-trnF intergenic spacer, partial sequence; chloroplast	300	441	40%	3,00E-77	95%	KC709133.1	0,00	#DIV/0!
Select seq emb|LK871767.1|	Hypericum laricifolium chloroplast DNA containing trnL(UAA)-trnF(GAA) IGS, specimen voucher M. Weigend-910-B	521	521	65%	6,00E-144	88%	LK871767.1	0,00	#DIV/0!
Select seq gb|KC709137.1|	Hypericum empetrifolium isolate C255 trnL-trnF intergenic spacer, partial sequence; chloroplast	300	443	41%	3,00E-77	95%	KC709137.1	0,00	#DIV/0!
Select seq gb|KC708993.1|	Hypericum kiboense isolate C1 trnL-trnF intergenic spacer, partial sequence; chloroplast	300	449	41%	3,00E-77	95%	KC708993.1	0,00	#DIV/0!
Select seq gb|KC709059.1|	Hypericum nummularium isolate C101 trnL-trnF intergenic spacer, partial sequence; chloroplast	298	419	41%	1,00E-76	95%	KC709059.1	0,00	#DIV/0!
Select seq gb|KC709119.1|	Hypericum erectum isolate C202 trnL-trnF intergenic spacer, partial sequence; chloroplast	316	465	45%	3,00E-82	97%	KC709119.1	0,00	#DIV/0!

Select seq gb|KC709025.1|	Hypericum olympicum isolate C57 trnL-trnF intergenic spacer, partial sequence; chloroplast	316	471	45%	3,00E-82	97%	KC709025.1	0,00	#DIV/0!
Select seq gb|KC709080.1|	Hypericum confertum isolate C138 trnL-trnF intergenic spacer, partial sequence; chloroplast	316	465	45%	3,00E-82	97%	KC709080.1	0,00	#DIV/0!
Select seq gb|KC708998.1|	Hypericum annulatum isolate C7 trnL-trnF intergenic spacer, partial sequence; chloroplast	316	454	45%	3,00E-82	97%	KC708998.1	0,00	#DIV/0!
Select seq gb|KC709142.1|	Hypericum papuanum isolate C275 trnL-trnF intergenic spacer, partial sequence; chloroplast	311	445	45%	1,00E-80	96%	KC709142.1	0,00	#DIV/0!
Select seq gb|KC709136.1|	Hypericum athoum isolate C251 trnL-trnF intergenic spacer, partial sequence; chloroplast	311	460	45%	1,00E-80	96%	KC709136.1	0,00	#DIV/0!
Select seq gb|KC709045.1|	Hypericum hyssopifolium isolate C81 trnL-trnF intergenic spacer, partial sequence; chloroplast	311	465	45%	1,00E-80	96%	KC709045.1	0,00	#DIV/0!
Select seq gb|KC709017.1|	Hypericum aucheri isolate C37 trnL-trnF intergenic spacer, partial sequence; chloroplast	311	454	45%	1,00E-80	96%	KC709017.1	0,00	#DIV/0!
Select seq gb|KC709147.1|	Hypericum monanthemum isolate C283 trnL-trnF intergenic spacer, partial sequence; chloroplast	309	449	45%	5,00E-80	96%	KC709147.1	0,00	#DIV/0!
Select seq gb|KC709075.1|	Hypericum aethiopicum subsp. sonderi isolate C131 trnL-trnF intergenic spacer, partial sequence; chloroplast	305	449	45%	6,00E-79	96%	KC709075.1	0,00	#DIV/0!
Select seq gb|KC709067.1|	Hypericum aethiopicum subsp. sonderi isolate C110 trnL-trnF intergenic spacer, partial sequence; chloroplast	305	443	45%	6,00E-79	96%	KC709067.1	0,00	#DIV/0!
Select seq gb|KC709044.1|	Hypericum scouleri isolate C80 trnL-trnF intergenic spacer, partial sequence; chloroplast	305	454	45%	6,00E-79	96%	KC709044.1	0,00	#DIV/0!
Select seq gb|KC709113.1|	Hypericum conjungens isolate C194 trnL-trnF intergenic spacer, partial sequence; chloroplast	300	449	45%	3,00E-77	95%	KC709113.1	0,00	#DIV/0!
Select seq gb|KC709009.1|	Hypericum perforatum isolate C22 trnL-trnF intergenic spacer, partial sequence; chloroplast	300	449	45%	3,00E-77	95%	KC709009.1	0,00	#DIV/0!
Select seq gb|KC709089.1|	Hypericum undulatum isolate C156 trnL-trnF intergenic spacer, partial sequence; chloroplast	300	449	45%	3,00E-77	95%	KC709089.1	0,00	#DIV/0!
Select seq gb|KC709043.1|	Hypericum heterophyllum isolate C78 trnL-trnF intergenic spacer, partial sequence; chloroplast	298	430	45%	1,00E-76	95%	KC709043.1	0,00	#DIV/0!
Select seq emb|LK871756.1|	Hypericum elodes chloroplast DNA containing trnL(UAA)-trnF(GAA) IGS, specimen voucher C. Scheriau-HEID808396-HEID	411	411	62%	1,00E-110	85%	LK871756.1	0,00	#DIV/0!
Select seq gb|KC709051.1|	Hypericum linarioides isolate C88 trnL-trnF intergenic spacer, partial sequence; chloroplast	302	430	53%	8,00E-78	95%	KC709051.1	0,00	#DIV/0!

Select for downloading or viewing reports	Kh031_ITS Description	Max score	Total score	Query cover	E value	Ident	Accession	(Ident/Cover)* Max score	Deviation from top hit
Select seq emb|AM503883.2|	Physalis alkekengi 18S rRNA pseudogene (partial), ITS1, 5.8S rRNA pseudogene, ITS2 and 28S rRNA pseudogene (partial), clone Pa-a	399	399	49%	3,00E-107	90%	AM503883.2	0,00	#DIV/0!
Select seq emb|AM503884.2|	Physalis alkekengi 18S rRNA pseudogene (partial), ITS1, 5.8S rRNA pseudogene, ITS2 and 28S rRNA pseudogene (partial), clone Pa-b	383	383	50%	3,00E-102	89%	AM503884.2	0,00	#DIV/0!
Select seq gb|DQ314189.1|	Iochroma australe voucher Smith 390 (WIS) internal transcribed spacer 1, partial sequence; 5.8S ribosomal RNA gene and internal transcribed spacer 2, complete sequence; and 25S ribosomal RNA gene, partial sequence	281	281	44%	1,00E-71	85%	DQ314189.1	0,00	#DIV/0!
Select seq gb|DQ314199.1|	Iochroma ellipticum voucher Jager 622 (CDRS) internal transcribed spacer 1, partial sequence; 5.8S ribosomal RNA gene and internal transcribed spacer 2, complete sequence; and 25S ribosomal RNA gene, partial sequence	267	267	44%	3,00E-67	84%	DQ314199.1	0,00	#DIV/0!
Select seq emb|AJ492441.1|	Nicotiana solanifolia 5.8S rRNA gene, ITS1 (partial) and ITS2 (partial)	309	309	52%	4,00E-80	84%	AJ492441.1	0,00	#DIV/0!
Select seq gb|DQ314162.1|	Leucophysalis grandiflora voucher Olmstead S-30 (WTU) internal transcribed spacer 1, partial sequence; 5.8S ribosomal RNA gene and internal transcribed spacer 2, complete sequence; and 25S ribosomal RNA gene, partial sequence	303	303	51%	2,00E-78	84%	DQ314162.1	0,00	#DIV/0!
Select seq emb|AJ492440.1|	Nicotiana cordifolia 5.8S rRNA gene, ITS1 (partial) and ITS2 (partial)	303	303	52%	2,00E-78	84%	AJ492440.1	0,00	#DIV/0!
Select seq gb|AY665853.1|	Tzeltalia amphitricha isolate TZA 18S ribosomal RNA gene, partial sequence; internal transcribed spacer 1, 5.8S ribosomal RNA gene gene, and internal transcribed spacer 2, complete sequence; and 26S ribosomal RNA gene, partial sequence	243	243	43%	5,00E-60	83%	AY665853.1	0,00	#DIV/0!
Select seq emb|AJ492451.1|	Nicotiana palmeri 5.8S rRNA gene, ITS1 (partial) and ITS2 (partial)	292	292	52%	4,00E-75	83%	AJ492451.1	0,00	#DIV/0!
Select seq emb|AJ492435.1|	Nicotiana wigandioides 5.8S rRNA gene, ITS1 (partial) and ITS2 (partial)	292	292	52%	4,00E-75	83%	AJ492435.1	0,00	#DIV/0!
Select seq gb|KM514683.1|	Iochroma sp. SDS-2014 voucher Smith 542 (HOXA) internal transcribed spacer 1, partial sequence; 5.8S ribosomal RNA gene and internal transcribed spacer 2, complete sequence; and 28S ribosomal RNA gene, partial sequence	296	296	53%	3,00E-76	83%	KM514683.1	0,00	#DIV/0!
Select seq emb|AJ492454.1|	Nicotiana otophora 5.8S rRNA gene, ITS1 (partial) and ITS2 (partial), specimen Nee at al. 51739 BM	289	289	52%	6,00E-74	83%	AJ492454.1	0,00	#DIV/0!
Select seq emb|AJ492449.1|	Nicotiana tomentosa 5.8S rRNA gene, ITS1 (partial) and ITS2 (partial)	289	289	52%	6,00E-74	83%	AJ492449.1	0,00	#DIV/0!
Select seq emb|AJ492445.1|	Nicotiana kawakamii 5.8S rRNA gene, ITS1 (partial) and ITS2 (partial)	289	289	52%	6,00E-74	83%	AJ492445.1	0,00	#DIV/0!
Select seq emb|AJ492439.1|	Nicotiana debneyi 5.8S rRNA gene, ITS1 (partial) and ITS2 (partial)	289	289	52%	6,00E-74	83%	AJ492439.1	0,00	#DIV/0!
Select seq emb|AJ492427.1|	Nicotiana attenuata 5.8S rRNA gene, ITS1 (partial) and ITS2 (partial)	289	289	52%	6,00E-74	83%	AJ492427.1	0,00	#DIV/0!
Select seq emb|AJ492426.1|	Nicotiana acuminata 5.8S rRNA gene, ITS1 (partial) and ITS2 (partial)	289	289	52%	6,00E-74	83%	AJ492426.1	0,00	#DIV/0!
Select seq emb|AJ492436.1|	Nicotiana thyrsiflora 5.8S rRNA gene, ITS1 (partial) and ITS2 (partial)	287	287	52%	2,00E-73	83%	AJ492436.1	0,00	#DIV/0!
Select seq emb|AJ492430.1|	Nicotiana obtusifolia 5.8S rRNA gene, ITS1 (partial) and ITS2 (partial)	287	287	52%	2,00E-73	83%	AJ492430.1	0,00	#DIV/0!
Select seq gb|DQ314192.1|	Dunalia obovata voucher Smith 458 (WIS) internal transcribed spacer 1, partial sequence; 5.8S ribosomal RNA gene and internal transcribed spacer 2, complete sequence; and 25S ribosomal RNA gene, partial sequence	292	292	53%	4,00E-75	83%	DQ314192.1	0,00	#DIV/0!
Select seq gb|DQ314167.1|	Iochroma tingoanum voucher Smith 370 (WIS) internal transcribed spacer 1, partial sequence; 5.8S ribosomal RNA gene and internal transcribed spacer 2, complete sequence; and 25S ribosomal RNA gene, partial sequence	291	291	53%	2,00E-74	83%	DQ314167.1	0,00	#DIV/0!
Select seq emb|AJ492447.1|	Nicotiana tabacum 5.8S rRNA gene, ITS1 (partial) and ITS2 (partial), cultivar Big Cuban	283	283	52%	3,00E-72	83%	AJ492447.1	0,00	#DIV/0!
Select seq emb|AJ492450.1|	Nicotiana tomentosiformis 5.8S rRNA gene, ITS1 (partial) and ITS2 (partial), specimen Clarkson JC001 BM	283	283	52%	3,00E-72	83%	AJ492450.1	0,00	#DIV/0!
Select seq emb|AJ492444.1|	Nicotiana clevelandii 5.8S rRNA gene, ITS1 (partial) and ITS2 (partial)	283	283	52%	3,00E-72	83%	AJ492444.1	0,00	#DIV/0!
Select seq emb|AJ492442.1|	Nicotiana nesophila 5.8S rRNA gene, ITS1 (partial) and ITS2 (partial)	283	283	52%	3,00E-72	83%	AJ492442.1	0,00	#DIV/0!
Select seq emb|AJ492428.1|	Nicotiana pauciflora 5.8S rRNA gene, ITS1 (partial) and ITS2 (partial)	283	283	52%	3,00E-72	83%	AJ492428.1	0,00	#DIV/0!

Select seq emb|AJ492425.1|	Nicotiana linearis 5.8S rRNA gene, ITS1 (partial) and ITS2 (partial)	283	283	52%	3,00E-72	83%	AJ492425.1	0,00	#DIV/0!
Select seq emb|AJ492424.1|	Nicotiana alata 5.8S rRNA gene, ITS1 (partial) and ITS2 (partial)	283	283	52%	3,00E-72	83%	AJ492424.1	0,00	#DIV/0!
Select seq emb|AJ492423.1|	Nicotiana sylvestris 5.8S rRNA gene, ITS1 (partial) and ITS2 (partial)	283	283	52%	3,00E-72	83%	AJ492423.1	0,00	#DIV/0!
Select seq gb|DQ314163.1|	Tubocapsicum anomalum voucher Chen 231 (MO) internal transcribed spacer 1, partial sequence; 5.8S ribosomal RNA gene and internal transcribed spacer 2, complete sequence; and 25S ribosomal RNA gene, partial sequence	281	281	52%	1,00E-71	83%	DQ314163.1	0,00	#DIV/0!
Select seq gb|DQ314201.1|	Iochroma calycinum voucher Smith 471 (WIS) internal transcribed spacer 1, partial sequence; 5.8S ribosomal RNA gene and internal transcribed spacer 2, complete sequence; and 25S ribosomal RNA gene, partial sequence	285	285	53%	7,00E-73	83%	DQ314201.1	0,00	#DIV/0!
Select seq gb|DQ314200.1|	Iochroma lehmannii voucher Smith 484 (WIS) internal transcribed spacer 1, partial sequence; 5.8S ribosomal RNA gene and internal transcribed spacer 2, complete sequence; and 25S ribosomal RNA gene, partial sequence	285	285	53%	7,00E-73	83%	DQ314200.1	0,00	#DIV/0!
Select seq gb|DQ314198.1|	Dunalia spathulata voucher Smith 452 (WIS) internal transcribed spacer 1, partial sequence; 5.8S ribosomal RNA gene and internal transcribed spacer 2, complete sequence; and 25S ribosomal RNA gene, partial sequence	285	285	53%	7,00E-73	83%	DQ314198.1	0,00	#DIV/0!
Select seq gb|DQ314195.1|	Iochroma parvifolium voucher Smith 303 (WIS) internal transcribed spacer 1, partial sequence; 5.8S ribosomal RNA gene and internal transcribed spacer 2, complete sequence; and 25S ribosomal RNA gene, partial sequence	285	285	53%	7,00E-73	83%	DQ314195.1	0,00	#DIV/0!
Select seq gb|DQ314188.1|	Dunalia spinosa voucher Smith 379 (WIS) internal transcribed spacer 1, partial sequence; 5.8S ribosomal RNA gene and internal transcribed spacer 2, complete sequence; and 25S ribosomal RNA gene, partial sequence	285	285	53%	7,00E-73	83%	DQ314188.1	0,00	#DIV/0!
Select seq gb|DQ314186.1|	Iochroma squamosum voucher Smith 330 (WIS) internal transcribed spacer 1, partial sequence; 5.8S ribosomal RNA gene and internal transcribed spacer 2, complete sequence; and 25S ribosomal RNA gene, partial sequence	285	285	53%	7,00E-73	83%	DQ314186.1	0,00	#DIV/0!
Select seq gb|DQ314181.1|	Acnistus arborescens voucher Smith 209 (WIS) internal transcribed spacer 1, partial sequence; 5.8S ribosomal RNA gene and internal transcribed spacer 2, complete sequence; and 25S ribosomal RNA gene, partial sequence	285	285	53%	7,00E-73	83%	DQ314181.1	0,00	#DIV/0!
Select seq gb|DQ314176.1|	Iochroma confertiflorum voucher Smith 237 (WIS) internal transcribed spacer 1, partial sequence; 5.8S ribosomal RNA gene and internal transcribed spacer 2, complete sequence; and 25S ribosomal RNA gene, partial sequence	285	285	53%	7,00E-73	83%	DQ314176.1	0,00	#DIV/0!
Select seq gb|DQ314170.1|	Iochroma grandiflorum voucher Smith 320 (WIS) internal transcribed spacer 1, partial sequence; 5.8S ribosomal RNA gene and internal transcribed spacer 2, complete sequence; and 25S ribosomal RNA gene, partial sequence	285	285	53%	7,00E-73	83%	DQ314170.1	0,00	#DIV/0!
Select seq gb|AY665855.1|	Tzeltalia calidaria isolate TZC 18S ribosomal RNA gene, partial sequence; internal transcribed spacer 1, 5.8S ribosomal RNA gene gene, and internal transcribed spacer 2, complete sequence; and 26S ribosomal RNA gene, partial sequence	233	233	43%	3,00E-57	82%	AY665855.1	0,00	#DIV/0!
Select seq gb|DQ314203.1|	Iochroma fuchsioides voucher Smith 488 (WIS) internal transcribed spacer 1, partial sequence; 5.8S ribosomal RNA gene and internal transcribed spacer 2, complete sequence; and 25S ribosomal RNA gene, partial sequence	287	287	53%	2,00E-73	82%	DQ314203.1	0,00	#DIV/0!
Select seq emb|AJ492438.1|	Nicotiana suaveolens 5.8S rRNA gene, ITS1 (partial) and ITS2 (partial)	278	278	52%	1,00E-70	83%	AJ492438.1	0,00	#DIV/0!
Select seq gb|DQ314196.1|	Eriolarynx fasciculata voucher Smith 432 (WIS) internal transcribed spacer 1, partial sequence; 5.8S ribosomal RNA gene and internal transcribed spacer 2, complete sequence; and 25S ribosomal RNA gene, partial sequence	285	285	53%	7,00E-73	82%	DQ314196.1	0,00	#DIV/0!
Select seq gb|DQ314182.1|	Saracha punctata voucher Nee 51804 (NY) internal transcribed spacer 1, partial sequence; 5.8S ribosomal RNA gene and internal transcribed spacer 2, complete sequence; and 25S ribosomal RNA gene, partial sequence	285	285	53%	7,00E-73	82%	DQ314182.1	0,00	#DIV/0!
Select seq gb|DQ314171.1|	Eriolarynx lorentzii voucher Hawkes et al. 3452 (WIS) internal transcribed spacer 1, partial sequence; 5.8S ribosomal RNA gene and internal transcribed spacer 2, complete sequence; and 25S ribosomal RNA gene, partial sequence	285	285	53%	7,00E-73	82%	DQ314171.1	0,00	#DIV/0!
Select seq gb|AY665848.1|	Leucophysalis viscosa isolate LV 18S ribosomal RNA gene, partial sequence; internal transcribed spacer 1, 5.8S ribosomal RNA gene gene, and internal transcribed spacer 2, complete sequence; and 26S ribosomal RNA gene, partial sequence	231	231	43%	1,00E-56	82%	AY665848.1	0,00	#DIV/0!
Select seq gb|KP824745.1|	Nicotiana benthamiana external transcribed spacer, 18S ribosomal RNA gene, internal transcribed spacer 1, 5.8S ribosomal RNA gene, internal transcribed spacer 2, and 25S ribosomal RNA gene, complete sequence; and external transcribed spacer, partial sequence	278	278	52%	1,00E-70	82%	KP824745.1	0,00	#DIV/0!
Select seq gb|DQ314185.1|	Iochroma sp. Smith 317 internal transcribed spacer 1, partial sequence; 5.8S ribosomal RNA gene and internal transcribed spacer 2, complete sequence; and 25S ribosomal RNA gene, partial sequence	278	278	52%	1,00E-70	82%	DQ314185.1	0,00	#DIV/0!
Select seq emb|AJ492446.1|	Nicotiana digluta 5.8S rRNA gene, ITS1 (partial) and ITS2 (partial)	278	278	52%	1,00E-70	82%	AJ492446.1	0,00	#DIV/0!
Select seq gb|DQ314178.1|	Saracha quitensis voucher Smith 257 (WIS) internal transcribed spacer 1, partial sequence; 5.8S ribosomal RNA gene and internal transcribed spacer 2, complete sequence; and 25S ribosomal RNA gene, partial sequence	283	283	53%	3,00E-72	82%	DQ314178.1	0,00	#DIV/0!
Select seq emb|AJ012367.1|	Nicotiana tabacum 5.8S rRNA gene and internal transcribed spacers 1 and 2, cultivar Xanti Yaka	272	272	51%	6,00E-69	82%	AJ012367.1	0,00	#DIV/0!
Select seq emb|AJ012364.1|	Nicotiana tabacum 5.8S rRNA gene and internal transcribed spacers 1 and 2, cultivar Kentucky	272	272	51%	6,00E-69	82%	AJ012364.1	0,00	#DIV/0!
Select seq emb|AJ012363.1|	Nicotiana tabacum 5.8S rRNA gene and internal transcribed spacers 1 and 2, cultivar Paraguay	272	272	51%	6,00E-69	82%	AJ012363.1	0,00	#DIV/0!
Select seq emb|AJ012359.1|	Nicotiana tabacum 5.8S rRNA gene and internal transcribed spacers 1 and 2, cultivar Burley	272	272	51%	6,00E-69	82%	AJ012359.1	0,00	#DIV/0!

Select seq emb|AJ012358.1|	Nicotiana tabacum 5.8S rRNA gene and internal transcribed spacers 1 and 2, cultivar Bright Virginia	272	272	51%	6,00E-69	82% AJ012358.1	0,00	#DIV/0!
Select seq emb|AJ492437.1|	Nicotiana arentsii 5.8S rRNA gene, ITS1 (partial) and ITS2 (partial)	276	276	52%	4,00E-70	82% AJ492437.1	0,00	#DIV/0!
Select seq emb|AJ492433.1|	Nicotiana glutinosa 5.8S rRNA gene, ITS1 (partial) and ITS2 (partial)	276	276	52%	4,00E-70	82% AJ492433.1	0,00	#DIV/0!
Select seq gb|DQ314190.1|	Vassobia breviflora voucher Smith 412 (WIS) internal transcribed spacer 1, partial sequence; 5.8S ribosomal RNA gene and internal transcribed spacer 2, complete sequence; and 25S ribosomal RNA gene, partial sequence	281	281	53%	1,00E-71	82% DQ314190.1	0,00	#DIV/0!
Select seq gb|DQ314191.1|	Vassobia dichotoma voucher Smith 440 (WIS) internal transcribed spacer 1, partial sequence; 5.8S ribosomal RNA gene and internal transcribed spacer 2, complete sequence; and 25S ribosomal RNA gene, partial sequence	274	274	52%	2,00E-69	82% DQ314191.1	0,00	#DIV/0!
Select seq gb|DQ314202.1|	Iochroma baumii voucher Smith 476 (WIS) internal transcribed spacer 1, partial sequence; 5.8S ribosomal RNA gene and internal transcribed spacer 2, complete sequence; and 25S ribosomal RNA gene, partial sequence	279	279	53%	3,00E-71	82% DQ314202.1	0,00	#DIV/0!
Select seq gb|DQ314180.1|	Iochroma cyaneum voucher Smith 223 (WIS) internal transcribed spacer 1, partial sequence; 5.8S ribosomal RNA gene and internal transcribed spacer 2, complete sequence; and 25S ribosomal RNA gene, partial sequence	279	279	53%	3,00E-71	82% DQ314180.1	0,00	#DIV/0!
Select seq gb|DQ314179.1|	Iochroma gesnerioides voucher Smith 200 (WIS) internal transcribed spacer 1, partial sequence; 5.8S ribosomal RNA gene and internal transcribed spacer 2, complete sequence; and 25S ribosomal RNA gene, partial sequence	279	279	53%	3,00E-71	82% DQ314179.1	0,00	#DIV/0!
Select seq gb|DQ314175.1|	Iochroma loxense voucher Smith 220 (WIS) internal transcribed spacer 1, partial sequence; 5.8S ribosomal RNA gene and internal transcribed spacer 2, complete sequence; and 25S ribosomal RNA gene, partial sequence	279	279	53%	3,00E-71	82% DQ314175.1	0,00	#DIV/0!
Select seq gb|DQ314173.1|	Acnistus arborescens voucher Bohs 2428 (UT) internal transcribed spacer 1, partial sequence; 5.8S ribosomal RNA gene and internal transcribed spacer 2, complete sequence; and 25S ribosomal RNA gene, partial sequence	279	279	53%	3,00E-71	82% DQ314173.1	0,00	#DIV/0!
Select seq gb|KP100283.1|	Browallia eludens internal transcribed spacer 1, partial sequence; 5.8S ribosomal RNA gene and internal transcribed spacer 2, complete sequence; and 25S ribosomal RNA gene, partial sequence	263	263	50%	3,00E-66	82% KP100283.1	0,00	#DIV/0!
Select seq emb|AJ012366.1|	Nicotiana tabacum 5.8S rRNA gene and internal transcribed spacers 1 and 2, cultivar Perustitza	268	268	51%	7,00E-68	82% AJ012366.1	0,00	#DIV/0!
Select seq gb|DQ277646.1|	Iris pseudacorus strain NEFUIris0011 18S ribosomal RNA gene, partial sequence; internal transcribed spacer 1, 5.8S ribosomal RNA gene, and internal transcribed spacer 2, complete sequence; and 28S ribosomal RNA gene, partial sequence	278	278	53%	1,00E-70	82% DQ277646.1	0,00	#DIV/0!
Select seq gb|DQ277642.1|	Iris mandshurica strain NEFUIris0007 18S ribosomal RNA gene, partial sequence; internal transcribed spacer 1, 5.8S ribosomal RNA gene, and internal transcribed spacer 2, complete sequence; and 28S ribosomal RNA gene, partial sequence	278	278	53%	1,00E-70	82% DQ277642.1	0,00	#DIV/0!
Select seq emb|AJ012360.1|	Nicotiana tabacum 5.8S rRNA gene and internal transcribed spacers 1 and 2, cultivar Maryland	267	267	51%	3,00E-67	82% AJ012360.1	0,00	#DIV/0!
Select seq emb|AJ492456.1|	Symonanthus bancroftii 5.8S rRNA gene, ITS1 (partial) and ITS2 (partial)	272	272	52%	6,00E-69	82% AJ492456.1	0,00	#DIV/0!
Select seq emb|AJ492453.1|	Nicotiana picilla 5.8S rRNA gene, ITS1 (partial) and ITS2 (partial)	272	272	52%	6,00E-69	82% AJ492453.1	0,00	#DIV/0!
Select seq emb|AJ492429.1|	Nicotiana miersii 5.8S rRNA gene, ITS1 (partial) and ITS2 (partial)	272	272	52%	6,00E-69	82% AJ492429.1	0,00	#DIV/0!
Select seq emb|AJ492434.1|	Nicotiana undulata 5.8S rRNA gene, ITS1 (partial) and ITS2 (partial)	270	270	52%	2,00E-68	82% AJ492434.1	0,00	#DIV/0!
Select seq gb|DQ314177.1|	Iochroma cornifolium voucher Smith 242 (WIS) internal transcribed spacer 1, partial sequence; 5.8S ribosomal RNA gene and internal transcribed spacer 2, complete sequence; and 25S ribosomal RNA gene, partial sequence	274	274	53%	2,00E-69	82% DQ314177.1	0,00	#DIV/0!
Select seq emb|AJ492452.1|	Nicotiana bigelovii 5.8S rRNA gene, ITS1 (partial) and ITS2 (partial)	267	267	52%	3,00E-67	82% AJ492452.1	0,00	#DIV/0!
Select seq emb|X59789.1|	N.rustica genes for 5.8S rRNA and 18S and 28S rRNA	267	267	52%	3,00E-67	82% X59789.1	0,00	#DIV/0!
Select seq gb|DQ314193.1|	Iochroma edule voucher Smith 300 (WIS) internal transcribed spacer 1, partial sequence; 5.8S ribosomal RNA gene and internal transcribed spacer 2, complete sequence; and 25S ribosomal RNA gene, partial sequence	270	270	53%	2,00E-68	82% DQ314193.1	0,00	#DIV/0!
Select seq gb|DQ314169.1|	Iochroma umbellatum voucher Smith 301 (WIS) internal transcribed spacer 1, partial sequence; 5.8S ribosomal RNA gene and internal transcribed spacer 2, complete sequence; and 25S ribosomal RNA gene, partial sequence	268	268	53%	7,00E-68	82% DQ314169.1	0,00	#DIV/0!
Select seq gb|DQ314156.1|	Iochroma cardenasianum voucher Smith 385 (WIS) internal transcribed spacer 1, partial sequence; 5.8S ribosomal RNA gene and internal transcribed spacer 2, complete sequence; and 25S ribosomal RNA gene, partial sequence	268	268	53%	7,00E-68	82% DQ314156.1	0,00	#DIV/0!
Select seq gb|EF190038.1|	Nicotiana obtusifolia 18S ribosomal RNA gene, partial sequence; internal transcribed spacer 1, 5.8S ribosomal RNA gene, and internal transcribed spacer 2, complete sequence; and 28S ribosomal RNA gene, partial sequence	257	257	51%	2,00E-64	82% EF190038.1	0,00	#DIV/0!
Select seq gb|DQ006041.1|	Datura stramonium voucher Beyersdorfer 126 US 18S ribosomal RNA gene, partial sequence; internal transcribed spacer 1, 5.8S ribosomal RNA gene, and internal transcribed spacer 2, complete sequence; and 28S ribosomal RNA gene, partial sequence	241	241	48%	2,00E-59	81% DQ006041.1	0,00	#DIV/0!
Select seq gb|DQ314187.1|	Iochroma salpoanum voucher Smith 364 (WIS) internal transcribed spacer 1, partial sequence; 5.8S ribosomal RNA gene and internal transcribed spacer 2, complete sequence; and 25S ribosomal RNA gene, partial sequence	265	265	53%	1,00E-66	81% DQ314187.1	0,00	#DIV/0!

Select seq gb|KF022335.1|	Datura stramonium isolate 01 18S ribosomal RNA gene, partial sequence; internal transcribed spacer 1, 5.8S ribosomal RNA gene, and internal transcribed spacer 2, complete sequence; and 28S ribosomal RNA gene, partial sequence	231	231	47%	1,00E-56	81%	KF022335.1	0,00	#DIV/0!
Select seq emb|AJ492432.1|	Nicotiana noctiflora 5.8S rRNA gene, ITS1 (partial) and ITS2 (partial)	255	255	52%	6,00E-64	81%	AJ492432.1	0,00	#DIV/0!
Select seq emb|AJ492431.1|	Nicotiana petunioides 5.8S rRNA gene, ITS1 (partial) and ITS2 (partial)	255	255	52%	6,00E-64	81%	AJ492431.1	0,00	#DIV/0!
Select seq gb|DQ314164.1|	Witheringia solanacea voucher D'Arcy 16399 (MO) internal transcribed spacer 1, partial sequence; 5.8S ribosomal RNA gene and internal transcribed spacer 2, complete sequence; and 25S ribosomal RNA gene, partial sequence	250	250	51%	3,00E-62	81%	DQ314164.1	0,00	#DIV/0!
Select seq gb|DQ314165.1|	Cuatresia colombiana voucher Smith 204 (WIS) internal transcribed spacer 1, partial sequence; 5.8S ribosomal RNA gene and internal transcribed spacer 2, complete sequence; and 25S ribosomal RNA gene, partial sequence	257	257	53%	2,00E-64	81%	DQ314165.1	0,00	#DIV/0!
Select seq gb|DQ314184.1|	Iochroma stenanthum voucher Smith 313 (WIS) internal transcribed spacer 1, partial sequence; 5.8S ribosomal RNA gene and internal transcribed spacer 2, complete sequence; and 25S ribosomal RNA gene, partial sequence	255	255	53%	6,00E-64	80%	DQ314184.1	0,00	#DIV/0!
Select seq gb|DQ314194.1|	Iochroma ayabacense voucher Smith 337 (WIS) internal transcribed spacer 1, partial sequence; 5.8S ribosomal RNA gene and internal transcribed spacer 2, complete sequence; and 25S ribosomal RNA gene, partial sequence	233	233	49%	3,00E-57	80%	DQ314194.1	0,00	#DIV/0!
Select seq gb|KP100298.1|	Schultesianthus leucanthus internal transcribed spacer 1, partial sequence; 5.8S ribosomal RNA gene and internal transcribed spacer 2, complete sequence; and 25S ribosomal RNA gene, partial sequence	248	248	53%	1,00E-61	80%	KP100298.1	0,00	#DIV/0!
Select seq gb|KP100295.1|	Markea lopezii internal transcribed spacer 1, partial sequence; 5.8S ribosomal RNA gene and internal transcribed spacer 2, complete sequence; and 25S ribosomal RNA gene, partial sequence	233	233	50%	3,00E-57	80%	KP100295.1	0,00	#DIV/0!
Select seq gb|KP100285.1|	Nicotiana suaveolens internal transcribed spacer 1, partial sequence; 5.8S ribosomal RNA gene and internal transcribed spacer 2, complete sequence; and 25S ribosomal RNA gene, partial sequence	233	233	50%	3,00E-57	80%	KP100285.1	0,00	#DIV/0!
Select seq gb|KP100288.1|	Cestrum brevifolium internal transcribed spacer 1, partial sequence; 5.8S ribosomal RNA gene and internal transcribed spacer 2, complete sequence; and 25S ribosomal RNA gene, partial sequence	233	233	52%	3,00E-57	80%	KP100288.1	0,00	#DIV/0!
Select seq gb|DQ314183.1|	Acnistus arborescens voucher Smith 312 (WIS) internal transcribed spacer 1, partial sequence; 5.8S ribosomal RNA gene and internal transcribed spacer 2, complete sequence; and 25S ribosomal RNA gene, partial sequence	231	231	51%	1,00E-56	79%	DQ314183.1	0,00	#DIV/0!
Select seq gb|DQ314159.1|	Lycianthes inaequilatera voucher Smith 210 (WIS) internal transcribed spacer 1, partial sequence; 5.8S ribosomal RNA gene and internal transcribed spacer 2, complete sequence; and 25S ribosomal RNA gene, partial sequence	237	237	53%	2,00E-58	80%	DQ314159.1	0,00	#DIV/0!
Select seq gb|DQ314160.1|	Salpichroa tristis voucher Smith 382 (WIS) internal transcribed spacer 1, partial sequence; 5.8S ribosomal RNA gene and internal transcribed spacer 2, complete sequence; and 25S ribosomal RNA gene, partial sequence	235	235	53%	8,00E-58	80%	DQ314160.1	0,00	#DIV/0!
Select seq emb|AJ492457.1|	Anthocercis gracilis 5.8S rRNA gene, ITS1 (partial) and ITS2 (partial)	230	230	52%	4,00E-56	80%	AJ492457.1	0,00	#DIV/0!
Select seq gb|DQ314155.1|	Nicandra physalodes voucher Smith 369 (WIS) internal transcribed spacer 1, partial sequence; 5.8S ribosomal RNA gene and internal transcribed spacer 2, complete sequence; and 25S ribosomal RNA gene, partial sequence	230	230	53%	4,00E-56	80%	DQ314155.1	0,00	#DIV/0!
Select seq gb|DQ314168.1|	Iochroma nitidum voucher Smith 371 (WIS) internal transcribed spacer 1, partial sequence; 5.8S ribosomal RNA gene and internal transcribed spacer 2, complete sequence; and 25S ribosomal RNA gene, partial sequence	228	228	53%	1,00E-55	78%	DQ314168.1	0,00	

Alignments

Select for downloading or viewing reports	Kh031_trnL Description	Max score	Total score	Query cover	E value	Ident	Accession	(Ident/Cover)* Max score	Deviation from top hit
Select seq gb|HM006824.1|	Physalis alkekengi isolate UPG0081 tRNA-Thr (trnT) gene, partial sequence; trnT-trnL intergenic spacer and tRNA-Leu (trnL) gene, complete sequence; and trnL-trnF intergenic spacer, partial sequence; chloroplast	1520	1520	97%	0.0	100%	HM006824.1	1567,01	100,0%
Select seq gb|DQ180420.1|	Physalis alkekengi isolate RGO 277 tRNA-Thr (trnT) gene, partial sequence; trnT-trnL intergenic spacer, tRNA-Leu (trnL) gene, and trnL-trnF intergenic spacer, complete sequence; and tRNA-Phe (trnF) gene, partial sequence; chloroplast	1555	1555	100%	0.0	100%	DQ180420.1	1555,00	99,2%
Select seq gb|EU581042.1|	Physalis carpenteri voucher Whitson 1133 (DUKE) tRNA-Leu (trnL) gene and trnL-trnF intergenic spacer, partial sequence; chloroplast	1533	1533	100%	0.0	99%	EU581042.1	1517,67	96,9%
Select seq gb|KP756703.1|	Iochroma edule isolate R034 trnT-trnL intergenic spacer, partial sequence; tRNA-Leu (trnL) gene and trnL-trnF intergenic spacer, complete sequence; and tRNA-Phe (trnF) gene, partial sequence; chloroplast	1332	1332	91%	0.0	98%	KP756703.1	1434,46	91,5%
Select seq gb|KP756702.1|	Iochroma cyaneum isolate R045 trnT-trnL intergenic spacer, partial sequence; tRNA-Leu (trnL) gene and trnL-trnF intergenic spacer, complete sequence; and tRNA-Phe (trnF) gene, partial sequence; chloroplast	1332	1332	91%	0.0	98%	KP756702.1	1434,46	91,5%
Select seq gb|EU581039.1|	Oryctes nevadensis voucher Tiehm 11982 (COLO) tRNA-Leu (trnL) gene and trnL-trnF intergenic spacer, partial sequence; chloroplast	1454	1605	100%	0.0	98%	EU581039.1	1424,92	90,9%
Select seq gb|EU581000.1|	Iochroma cardenasianum voucher Smith et al. 383 (WIS) tRNA-Leu (trnL) gene and trnL-trnF intergenic spacer, partial sequence; chloroplast	1155	1155	80%	0.0	97%	EU581000.1	1400,44	89,4%
Select seq gb|KP756707.1|	Iochroma cardenasianum isolate R046 trnT-trnL intergenic spacer, partial sequence; tRNA-Leu (trnL) gene and trnL-trnF intergenic spacer, complete sequence; and tRNA-Phe (trnF) gene, partial sequence; chloroplast	1151	1151	80%	0.0	97%	KP756707.1	1395,59	89,1%
Select seq gb|KF720762.1|	Larnax sylvarum tRNA-Leu (trnL) gene and trnL-trnF intergenic spacer, partial sequence; chloroplast	1206	1206	84%	0.0	97%	KF720762.1	1392,64	88,9%
Select seq gb|EU580961.1|	Aureliana fasciculata voucher Brown s.n. (IAC) tRNA-Leu (trnL) gene and trnL-trnF intergenic spacer, partial sequence; chloroplast	1290	1290	90%	0.0	97%	EU580961.1	1390,33	88,7%
Select seq gb|KF720760.1|	Athenaea pogogena isolate LB2690 tRNA-Leu (trnL) gene and trnL-trnF intergenic spacer, partial sequence; chloroplast	1197	1197	84%	0.0	97%	KF720760.1	1382,25	88,2%
Select seq gb|DQ124592.1|	Lycium macrodon voucher Miller 97-21 ARIZ tRNA-Leu (trnL) gene, partial sequence; trnL-trnF intergenic spacer, complete sequence; and tRNA-Phe (trnF) gene, partial sequence; chloroplast	1153	1153	81%	0.0	97%	DQ124592.1	1380,75	88,1%
Select seq gb|DQ124563.1|	Lycium arenicola voucher Venter 647 BLFU tRNA-Leu (trnL) gene, partial sequence; trnL-trnF intergenic spacer, complete sequence; and tRNA-Phe (trnF) gene, partial sequence; chloroplast	1153	1153	81%	0.0	97%	DQ124563.1	1380,75	88,1%
Select seq gb|DQ124596.1|	Lycium pallidum voucher Miller 97-20 ARIZ tRNA-Leu (trnL) gene, partial sequence; trnL-trnF intergenic spacer, complete sequence; and tRNA-Phe (trnF) gene, partial sequence; chloroplast	1151	1151	81%	0.0	97%	DQ124596.1	1378,36	88,0%
Select seq gb|EU581034.1|	Nolana linearifolia voucher Dillon & Dillon 5727 (F) tRNA-Leu (trnL) gene and trnL-trnF intergenic spacer, partial sequence; chloroplast	1147	1147	81%	0.0	97%	EU581034.1	1373,57	87,7%
Select seq gb|EU581022.1|	Lycium sandwicense voucher Olmstead 92-224 (WTU) tRNA-Leu (trnL) gene and trnL-trnF intergenic spacer, partial sequence; chloroplast	1147	1147	81%	0.0	97%	EU581022.1	1373,57	87,7%
Select seq gb|DQ124603.1|	Lycium shawii voucher Olmstead S-36 WTU tRNA-Leu (trnL) gene, partial sequence; trnL-trnF intergenic spacer, complete sequence; and tRNA-Phe (trnF) gene, partial sequence; chloroplast	1147	1147	81%	0.0	97%	DQ124603.1	1373,57	87,7%
Select seq gb|DQ124580.1|	Lycium cuneatum voucher Bernardello & Vesprini 897 CORD tRNA-Leu (trnL) gene, partial sequence; trnL-trnF intergenic spacer, complete sequence; and tRNA-Phe (trnF) gene, partial sequence; chloroplast	1147	1147	81%	0.0	97%	DQ124580.1	1373,57	87,7%
Select seq gb|DQ124560.1|	Lycium ameghinoi voucher Forcone 790 CORD tRNA-Leu (trnL) gene, partial sequence; trnL-trnF intergenic spacer, complete sequence; and tRNA-Phe (trnF) gene, partial sequence; chloroplast	1147	1147	81%	0.0	97%	DQ124560.1	1373,57	87,7%
Select seq gb|DQ124569.1|	Lycium brevipes voucher Miller 97-19 ARIZ tRNA-Leu (trnL) gene and trnL-trnF intergenic spacer, partial sequence; chloroplast	1147	1147	81%	0.0	97%	DQ124569.1	1373,57	87,7%
Select seq gb|DQ124562.1|	Lycium andersonii voucher Miller 97-12 ARIZ tRNA-Leu (trnL) gene, partial sequence; trnL-trnF intergenic spacer, complete sequence; and tRNA-Phe (trnF) gene, partial sequence; chloroplast	1147	1147	81%	0.0	97%	DQ124562.1	1373,57	87,7%
Select seq gb|DQ124561.1|	Lycium americanum voucher Barboza 525 CORD tRNA-Leu (trnL) gene, partial sequence; trnL-trnF intergenic spacer, complete sequence; and tRNA-Phe (trnF) gene, partial sequence; chloroplast	1147	1147	81%	0.0	97%	DQ124561.1	1373,57	87,7%
Select seq gb|DQ124554.1|	Grabowskia boerhaaviifolia voucher Bernardello 894 CORD tRNA-Leu (trnL) gene, partial sequence; trnL-trnF intergenic spacer, complete sequence; and tRNA-Phe (trnF) gene, partial sequence; chloroplast	1147	1147	81%	0.0	97%	DQ124554.1	1373,57	87,7%
Select seq gb|EU581055.1|	Schultesianthus leucanthus voucher Wendt 6784 (TEX) tRNA-Leu (trnL) gene and trnL-trnF intergenic spacer, partial sequence; chloroplast	1146	1146	81%	0.0	97%	EU581055.1	1372,37	87,6%
Select seq gb|EU581051.1|	Quincula lobata voucher Olmstead 93-74 (WTU) tRNA-Leu (trnL) gene and trnL-trnF intergenic spacer, partial sequence; chloroplast	1242	1242	88%	0.0	97%	EU581051.1	1369,02	87,4%
Select seq gb|FJ189721.1|	Lycium oxycarpum isolate 506796 tRNA-Leu (trnL) gene, partial sequence; trnL-trnF intergenic spacer, complete sequence; and tRNA-Phe (trnF) gene, partial sequence; chloroplast	1142	1142	81%	0.0	97%	FJ189721.1	1367,58	87,3%

Select seq gb|EU581061.1|	Scopolia japonica tRNA-Leu (trnL) gene and trnL-trnF intergenic spacer, partial sequence; chloroplast	1142	1142	81%	0.0	97%	EU581061.1	1367,58	87,3%
Select seq gb|EU581060.1|	Scopolia carniolica tRNA-Leu (trnL) gene and trnL-trnF intergenic spacer, partial sequence; chloroplast	1142	1142	81%	0.0	97%	EU581060.1	1367,58	87,3%
Select seq gb|EU581032.1|	Nicandra physalodes voucher Olmstead S-38 (WTU) tRNA-Leu (trnL) gene and trnL-trnF intergenic spacer, partial sequence; chloroplast	1142	1142	81%	0.0	97%	EU581032.1	1367,58	87,3%
Select seq gb|EU581010.1|	Latua pubiflora voucher Gardner et al. DCI 20 (E) tRNA-Leu (trnL) gene and trnL-trnF intergenic spacer, partial sequence; chloroplast	1142	1142	81%	0.0	97%	EU581010.1	1367,58	87,3%
Select seq gb|DQ124614.1|	Lycium vimineum voucher Bernardello & Vesprini 896 CORD tRNA-Leu (trnL) gene, partial sequence; trnL-trnF intergenic spacer, complete sequence; and tRNA-Phe (trnF) gene, partial sequence; chloroplast	1142	1142	81%	0.0	97%	DQ124614.1	1367,58	87,3%
Select seq gb|DQ124582.1|	Lycium exsertum voucher Miller 01-3 ARIZ tRNA-Leu (trnL) gene, partial sequence; trnL-trnF intergenic spacer, complete sequence; and tRNA-Phe (trnF) gene, partial sequence; chloroplast	1142	1142	81%	0.0	97%	DQ124582.1	1367,58	87,3%
Select seq gb|EU580982.1|	Cuatresia riparia voucher Bohs 2551 (UT) tRNA-Leu (trnL) gene and trnL-trnF intergenic spacer, partial sequence; chloroplast	1280	1280	91%	0.0	97%	EU580982.1	1364,40	87,1%
Select seq gb|EU581073.1|	Witheringia mexicana voucher BIRM S.1199 (BIRM) tRNA-Leu (trnL) gene and trnL-trnF intergenic spacer, partial sequence; chloroplast	1406	1592	100%	0.0	97%	EU581073.1	1363,82	87,0%
Select seq gb|FJ189725.1|	Phrodus microphyllus isolate 415 tRNA-Leu (trnL) gene, partial sequence; trnL-trnF intergenic spacer, complete sequence; and tRNA-Phe (trnF) gene, partial sequence; chloroplast	1136	1136	81%	0.0	97%	FJ189725.1	1360,40	86,8%
Select seq gb|EU581008.1|	Juanulloa mexicana voucher BIRM S.0411/69 (BIRM) tRNA-Leu (trnL) gene and trnL-trnF intergenic spacer, partial sequence; chloroplast	1136	1136	81%	0.0	97%	EU581008.1	1360,40	86,8%
Select seq gb|EU580955.1|	Anisodus luridus voucher BIRM S.0215/71 (BIRM) tRNA-Leu (trnL) gene and trnL-trnF intergenic spacer, partial sequence; chloroplast	1136	1136	81%	0.0	97%	EU580955.1	1360,40	86,8%
Select seq gb|DQ124587.1|	Lycium gilliesianum voucher Forcone 789 CORD tRNA-Leu (trnL) gene, partial sequence; trnL-trnF intergenic spacer, complete sequence; and tRNA-Phe (trnF) gene, partial sequence; chloroplast	1136	1136	81%	0.0	97%	DQ124587.1	1360,40	86,8%
Select seq gb|EU581072.1|	Witheringia meiantha voucher Bohs 3015 (UT) tRNA-Leu (trnL) gene and trnL-trnF intergenic spacer, partial sequence; chloroplast	1399	1568	100%	0.0	97%	EU581072.1	1357,03	86,6%
Select seq gb|EU581071.1|	Witheringia macrantha voucher Bohs 2512 (UT) tRNA-Leu (trnL) gene and trnL-trnF intergenic spacer, partial sequence; chloroplast	1387	1573	100%	0.0	97%	EU581071.1	1345,39	85,9%
Select seq gb|KP756706.1|	Iochroma peruvianum isolate R035 trnT-trnL intergenic spacer, partial sequence; tRNA-Leu (trnL) gene and trnL-trnF intergenic spacer, complete sequence; and tRNA-Phe (trnF) gene, partial sequence; chloroplast	1400	1400	100%	0.0	96%	KP756706.1	1344,00	85,8%
Select seq gb|KP280175.1|	Iochroma tingoanum chloroplast, complete genome	1400	1400	100%	0.0	96%	KP280175.1	1344,00	85,8%
Select seq gb|KP296185.1|	Iochroma loxense chloroplast, complete genome	1400	1400	100%	0.0	96%	KP296185.1	1344,00	85,8%
Select seq gb|KP262399.1|	Iochroma stenanthum chloroplast, complete genome	1400	1400	100%	0.0	96%	KP262399.1	1344,00	85,8%
Select seq gb|EU581014.1|	Leucophysalis nana voucher Bartholomew 5994 (MO) tRNA-Leu (trnL) gene and trnL-trnF intergenic spacer, partial sequence; chloroplast	1400	1400	100%	0.0	96%	EU581014.1	1344,00	85,8%
Select seq gb|EU581002.1|	Iochroma umbellatum voucher Hutchison et al. 6240 (UC) tRNA-Leu (trnL) gene and trnL-trnF intergenic spacer, partial sequence; chloroplast	1400	1400	100%	0.0	96%	EU581002.1	1344,00	85,8%
Select seq gb|EU581001.1|	Iochroma fuchsioides voucher Olmstead S-29 (WTU) tRNA-Leu (trnL) gene and trnL-trnF intergenic spacer, partial sequence; chloroplast	1400	1400	100%	0.0	96%	EU581001.1	1344,00	85,8%
Select seq gb|KP756719.1|	Solandra longiflora isolate R096 trnT-trnL intergenic spacer, partial sequence; tRNA-Leu (trnL) gene and trnL-trnF intergenic spacer, complete sequence; and tRNA-Phe (trnF) gene, partial sequence; chloroplast	1131	1131	81%	0.0	96%	KP756719.1	1340,44	85,5%
Select seq gb|EU581063.1|	Solandra grandiflora voucher Cultivated Matthei Bot. Gard., #840415 (no voucher) tRNA-Leu (trnL) gene and trnL-trnF intergenic spacer, partial sequence; chloroplast	1131	1131	81%	0.0	96%	EU581063.1	1340,44	85,5%
Select seq gb|EU580991.1|	Exodeconus miersii voucher BIRM S.1223.73 (BIRM) tRNA-Leu (trnL) gene and trnL-trnF intergenic spacer, partial sequence; chloroplast	1131	1131	81%	0.0	96%	EU580991.1	1340,44	85,5%
Select seq gb|KP294386.1|	Iochroma nitidum chloroplast, complete genome	1395	1395	100%	0.0	96%	KP294386.1	1339,20	85,5%
Select seq gb|EU580954.1|	Acnistus arborescens voucher Bohs 2577 (UT) tRNA-Leu (trnL) gene and trnL-trnF intergenic spacer, partial sequence; chloroplast	1395	1395	100%	0.0	96%	EU580954.1	1339,20	85,5%
Select seq gb|EU580978.1|	Chamaesaracha coronopus voucher Turner 15854 (TEX) tRNA-Leu (trnL) gene and trnL-trnF intergenic spacer, partial sequence; chloroplast	1378	1546	100%	0.0	97%	EU580978.1	1336,66	85,3%
Select seq gb|KP756709.1|	Saracha punctata isolate R042 tRNA-Leu (trnL) gene, partial sequence; trnL-trnF intergenic spacer, complete sequence; and tRNA-Phe (trnF) gene, partial sequence; chloroplast	1365	1509	99%	0.0	96%	KP756709.1	1323,64	84,5%

Select seq gb|KP756704.1|	Iochroma calycinum isolate R043 tRNA-Thr (trnT) gene, partial sequence; trnT-trnL intergenic spacer, tRNA-Leu (trnL) gene, and trnL-trnF intergenic spacer, complete sequence; and tRNA-Phe (trnF) gene, partial sequence; chloroplast	1365	1641	99% 0.0	96% KP756704.1	1323,64	84,5%
Select seq gb|EU581013.1|	Leucophysalis grandiflora voucher Olmstead S-30 (WTU) tRNA-Leu (trnL) gene and trnL-trnF intergenic spacer, partial sequence; chloroplast	1376	1376	100% 0.0	96% EU581013.1	1320,96	84,3%
Select seq gb|KP998157.1|	Dunalia solanacea chloroplast, complete genome	1371	1534	100% 0.0	96% KP998157.1	1316,16	84,0%
Select seq gb|KP280050.1|	Saracha punctata chloroplast, complete genome	1371	1534	100% 0.0	96% KP280050.1	1316,16	84,0%
Select seq gb|KP280057.1|	Dunalia obovata chloroplast, complete genome	1371	1534	100% 0.0	96% KP280057.1	1316,16	84,0%
Select seq gb|KP756705.1|	Iochroma gesnerioides isolate R037 trnT-trnL intergenic spacer, partial sequence; tRNA-Leu (trnL) gene and trnL-trnF intergenic spacer, complete sequence; and tRNA-Phe (trnF) gene, partial sequence; chloroplast	1365	1529	100% 0.0	96% KP756705.1	1310,40	83,6%
Select seq gb|KP308151.1|	Dunalia brachyacantha chloroplast, complete genome	1365	1529	100% 0.0	96% KP308151.1	1310,40	83,6%
Select seq gb|EU581053.1|	Saracha punctata voucher Plowman 4651 (UC) tRNA-Leu (trnL) gene and trnL-trnF intergenic spacer, partial sequence; chloroplast	1365	1529	100% 0.0	96% EU581053.1	1310,40	83,6%
Select seq gb|EU580999.1|	Iochroma australe voucher Olmstead S-17 (WTU) tRNA-Leu (trnL) gene and trnL-trnF intergenic spacer, partial sequence; chloroplast	1365	1529	100% 0.0	96% EU580999.1	1310,40	83,6%
Select seq gb|KP756711.1|	Iochroma fuchsioides isolate R036 trnT-trnL intergenic spacer, partial sequence; tRNA-Leu (trnL) gene and trnL-trnF intergenic spacer, complete sequence; and tRNA-Phe (trnF) gene, partial sequence; chloroplast	1269	1324	94% 0.0	97% KP756711.1	1309,50	83,6%
Select seq gb|EU581074.1|	Witheringia solanacea voucher Bohs 2416 (UT) tRNA-Leu (trnL) gene and trnL-trnF intergenic spacer, partial sequence; chloroplast	1362	1536	100% 0.0	96% EU581074.1	1307,52	83,4%
Select seq gb|KP756712.1|	Iochroma australe isolate R039 trnT-trnL intergenic spacer, partial sequence; tRNA-Leu (trnL) gene and trnL-trnF intergenic spacer, complete sequence; and tRNA-Phe (trnF) gene, partial sequence; chloroplast	1266	1320	94% 0.0	97% KP756712.1	1306,40	83,4%
Select seq gb|KP294521.1|	Vassobia dichotoma chloroplast, complete genome	1360	1523	100% 0.0	96% KP294521.1	1305,60	83,3%
Select seq gb|EU581067.1|	Vassobia dichotoma voucher Nee et al. 51797 (NY) tRNA-Leu (trnL) gene and trnL-trnF intergenic spacer, partial sequence; chloroplast	1360	1523	100% 0.0	96% EU581067.1	1305,60	83,3%
Select seq gb|KP756700.1|	Acnistus arborescens isolate R038 trnT-trnL intergenic spacer, partial sequence; tRNA-Leu (trnL) gene and trnL-trnF intergenic spacer, complete sequence; and tRNA-Phe (trnF) gene, partial sequence; chloroplast	1264	1318	94% 0.0	97% KP756700.1	1304,34	83,2%
Select seq gb|EU580990.1|	Eriolarynx lorentzii voucher Olmstead S-18 (WTU), (BIRM S.0376) tRNA-Leu (trnL) gene and trnL-trnF intergenic spacer, partial sequence; chloroplast	1358	1522	100% 0.0	96% EU580990.1	1303,68	83,2%
Select seq gb|EU581066.1|	Tubocapsicum anomalum voucher Chen 231 (MO) tRNA-Leu (trnL) gene and trnL-trnF intergenic spacer, partial sequence; chloroplast	1339	1464	100% 0.0	97% EU581066.1	1298,83	82,9%
Select seq gb|EU580986.1|	Discopodium penninervium voucher Knapp 9808 (BM) tRNA-Leu (trnL) gene and trnL-trnF intergenic spacer, partial sequence; chloroplast	1352	1437	100% 0.0	96% EU580986.1	1297,92	82,8%
Select seq gb|KC768880.1|	Physaliastrum heterophyllum voucher Lihq0435 tRNA-Leu (trnL) gene, partial sequence; trnL-trnF intergenic spacer, complete sequence; and tRNA-Phe (trnF) gene, partial sequence; chloroplast	1360	1360	100% 0.0	95% KC768880.1	1292,00	82,5%
Select seq gb|KC768879.1|	Physaliastrum chamaesarachoides voucher Lihq0393 tRNA-Leu (trnL) gene, partial sequence; trnL-trnF intergenic spacer, complete sequence; and tRNA-Phe (trnF) gene, partial sequence; chloroplast	1343	1451	100% 0.0	96% KC768879.1	1289,28	82,3%
Select seq gb|KP756713.1|	Eriolarynx lorentzii isolate R040 trnT-trnL intergenic spacer, partial sequence; tRNA-Leu (trnL) gene and trnL-trnF intergenic spacer, complete sequence; and tRNA-Phe (trnF) gene, partial sequence; chloroplast	1262	1317	94% 0.0	96% KP756713.1	1288,85	82,2%
Select seq gb|EU581037.1|	Nothocestrum latifolium voucher Herbst et al. 725 (COLO) tRNA-Leu (trnL) gene and trnL-trnF intergenic spacer, partial sequence; chloroplast	1347	1347	100% 0.0	95% EU581037.1	1279,65	81,7%
Select seq gb|KM200029.1|	Cuatresia colombiana tRNA-Leu (trnL) gene and trnL-trnF intergenic spacer, partial sequence; chloroplast	1295	1365	99% 0.0	97% KM200029.1	1268,84	81,0%
Select seq gb|EU581009.1|	Larnax subtriflora voucher Sawyer 777 (CONN) tRNA-Leu (trnL) gene and trnL-trnF intergenic spacer, partial sequence; chloroplast	1280	1280	96% 0.0	95% EU581009.1	1266,67	80,8%
Select seq gb|EU580981.1|	Cuatresia exiguiflora voucher Bohs 2454 (UT) tRNA-Leu (trnL) gene and trnL-trnF intergenic spacer, partial sequence; chloroplast	1290	1361	99% 0.0	97% EU580981.1	1263,94	80,7%
Select seq gb|EU580985.1|	Deprea sylvarum voucher Almeda 2226 (DUKE) tRNA-Leu (trnL) gene and trnL-trnF intergenic spacer, partial sequence; chloroplast	1273	1273	96% 0.0	95% EU580985.1	1259,74	80,4%
Select seq gb|EU581070.1|	Witheringia cuneata voucher Bohs 2394 (UT) tRNA-Leu (trnL) gene and trnL-trnF intergenic spacer, partial sequence; chloroplast	1284	1353	99% 0.0	97% EU581070.1	1258,06	80,3%
Select seq gb|EU580963.1|	Brachistus stramonifolius voucher Sousa-Pena 738a (CONN) tRNA-Leu (trnL) gene and trnL-trnF intergenic spacer, partial sequence; chloroplast	1301	1301	98% 0.0	94% EU580963.1	1247,90	79,6%

Select seq gb|EU581038.1|	Nothocestrum longifolium voucher Oppenheimer s.n.#(BISH) tRNA-Leu (trnL) gene and trnL-trnF intergenic spacer, partial sequence; chloroplast	1308	1398	100% 0.0	95% EU581038.1	1242,60	79,3%
Select seq gb|EU581025.1|	Margaranthus solanaceus voucher Olmstead S-37 (WTU) tRNA-Leu (trnL) gene and trnL-trnF intergenic spacer, partial sequence; chloroplast	1279	1279	98% 0.0	95% EU581025.1	1239,85	79,1%
Select seq gb|EU580979.1|	Chamaesaracha sordida voucher Olmstead 92-245 (WTU) tRNA-Leu (trnL) gene and trnL-trnF intergenic spacer, partial sequence; chloroplast	1301	1462	100% 0.0	94% EU580979.1	1222,94	78,0%
Select seq gb|EU581043.1|	Physalis heterophylla voucher Olmstead S-64 (WTU) tRNA-Leu (trnL) gene and trnL-trnF intergenic spacer, partial sequence; chloroplast	1273	1273	100% 0.0	94% EU581043.1	1196,62	76,4%
Select seq gb|KP295964.1|	Physalis peruviana plastid, complete genome	1253	1361	100% 0.0	94% KP295964.1	1177,82	75,2%
Select seq gb|DQ180417.1|	Lycianthes rantonnei isolate RGO 364 tRNA-Thr (trnT) gene, partial sequence; trnT-trnL intergenic spacer, tRNA-Leu (trnL) gene, and trnL-trnF intergenic spacer, complete sequence; and tRNA-Phe (trnF) gene, partial sequence; chloroplast	1194	1194	95% 0.0	93% DQ180417.1	1168,86	74,6%
Select seq gb|EU581020.1|	Lycianthes peduncularis voucher Dean 283 (DAV) tRNA-Leu (trnL) gene and trnL-trnF intergenic spacer, partial sequence; chloroplast	1162	1162	94% 0.0	93% EU581020.1	1149,64	73,4%
Select seq gb|EU581016.1|	Lycianthes ciliolata voucher Dean 206 (DAV) tRNA-Leu (trnL) gene and trnL-trnF intergenic spacer, partial sequence; chloroplast	1162	1162	94% 0.0	93% EU581016.1	1149,64	73,4%
Select seq gb|DQ914924.1|	Lycianthes rantonnei tRNA-Leu (trnL) gene, partial sequence; trnL-trnF intergenic spacer, complete sequence; and tRNA-Phe (trnF) gene, partial sequence; chloroplast	1188	1245	99% 0.0	93% DQ914924.1	1116,00	71,2%
Select seq gb|EU581018.1|	Lycianthes inaequilatera voucher Bohs 3089 (UT) tRNA-Leu (trnL) gene and trnL-trnF intergenic spacer, partial sequence; chloroplast	1147	1147	95% 0.0	92% EU581018.1	1110,78	70,9%
Select seq gb|EU581044.1|	Physalis peruviana voucher Olmstead S-69 (WTU) tRNA-Leu (trnL) gene and trnL-trnF intergenic spacer, partial sequence; chloroplast	1175	1175	98% 0.0	91% EU581044.1	1091,07	69,6%
Select seq gb|EU581045.1|	Physalis philadelphica voucher Bohs 2433 (UT) tRNA-Leu (trnL) gene and trnL-trnF intergenic spacer, partial sequence; chloroplast	1151	1235	98% 0.0	91% EU581045.1	1068,79	68,2%
Select seq gb|DQ180441.1|	Solanum aligerum isolate LB 797 tRNA-Thr (trnT) gene, partial sequence; trnT-trnL intergenic spacer, tRNA-Leu (trnL) gene, and trnL-trnF intergenic spacer, complete sequence; and tRNA-Phe (trnF) gene, partial sequence; chloroplast	1144	1144	100% 0.0	91% DQ180441.1	1041,04	66,4%
Select seq gb|DQ180451.1|	Solanum nitidum isolate LB 125 tRNA-Thr (trnT) gene, partial sequence; trnT-trnL intergenic spacer, tRNA-Leu (trnL) gene, and trnL-trnF intergenic spacer, complete sequence; and tRNA-Phe (trnF) gene, partial sequence; chloroplast	1134	1134	100% 0.0	91% DQ180451.1	1031,94	65,9%
Select seq gb|DQ855062.1|	Solanum symonii isolate ASP075 tRNA-Leu (trnL) gene and trnL-trnF intergenic spacer, partial sequence; chloroplast	1133	1133	100% 0.0	91% DQ855062.1	1031,03	65,8%
Select seq gb|DQ855060.1|	Solanum aviculare isolate ASP003 tRNA-Leu (trnL) gene and trnL-trnF intergenic spacer, partial sequence; chloroplast	1133	1133	100% 0.0	91% DQ855060.1	1031,03	65,8%
Select seq gb|AY559238.1|	Solanum aviculare tRNA-Thr (trnT) gene, partial sequence; trnT-trnL intergenic spacer, tRNA-Leu (trnL) gene, and trnL-trnF intergenic spacer, complete sequence; and tRNA-Phe (trnF) gene, partial sequence; chloroplast	1133	1133	100% 0.0	91% AY559238.1	1031,03	

Select for downloading or viewing reports	Kh032_trnL Description	Max score	Total score	Query cover	E value	Ident	Accession	(Ident/Cover)* Max score	Deviation from top hit
Select seq gb|GU111506.1|	Ephedra distachya voucher H. Freitag 33.259 (KAS) tRNA-Leu (trnL) gene, partial sequence; trnL-trnF intergenic spacer, complete sequence; and tRNA-Phe (trnF) gene, partial sequence; chloroplast	654	654	71%	0.0	99%	GU111506.1	911,92	100,0%
Select seq gb|AF479869.1|	Ephedra viridis tRNA-Leu (trnL) gene, intron sequence; chloroplast gene for chloroplast product	688	688	75%	0.0	99%	AF479869.1	908,16	99,6%
Select seq gb|AY423433.1|	Ephedra equisetina tRNA-Leu (trnL) gene, partial sequence; trnL-trnF intergenic spacer, complete sequence; and tRNA-Phe (trnF) gene, partial sequence; chloroplast	669	669	73%	0.0	99%	AY423433.1	907,27	99,5%
Select seq gb|AY423430.1|	Ephedra intermedia tRNA-Leu (trnL) gene, partial sequence; trnL-trnF intergenic spacer, complete sequence; and tRNA-Phe (trnF) gene, partial sequence; chloroplast	669	669	73%	0.0	99%	AY423430.1	907,27	99,5%
Select seq gb|GU111514.1|	Ephedra distachya voucher H. Freitag 28.834 (KAS) tRNA-Leu (trnL) gene, partial sequence; trnL-trnF intergenic spacer, complete sequence; and tRNA-Phe (trnF) gene, partial sequence; chloroplast	649	649	71%	0.0	99%	GU111514.1	904,94	99,2%
Select seq dbj|AP010819.1|	Ephedra equisetina chloroplast DNA, complete sequence	684	684	75%	0.0	99%	AP010819.1	902,88	99,0%
Select seq gb|AY423434.1|	Ephedra equisetina voucher 60821 tRNA-Leu (trnL) gene, partial sequence; trnL-trnF intergenic spacer, complete sequence; and tRNA-Phe (trnF) gene, partial sequence; chloroplast	664	664	73%	0.0	99%	AY423434.1	900,49	98,7%
Select seq gb|GU111507.1|	Ephedra dahurica voucher H. Freitag 33.130 (KAS) tRNA-Leu (trnL) gene, partial sequence; trnL-trnF intergenic spacer, complete sequence; and tRNA-Phe (trnF) gene, partial sequence; chloroplast	641	641	71%	3,00E-180	99%	GU111507.1	893,79	98,0%
Select seq gb|AY423438.1|	Ephedra minuta tRNA-Leu (trnL) gene, partial sequence; trnL-trnF intergenic spacer, complete sequence; and tRNA-Phe (trnF) gene, partial sequence; chloroplast	658	658	73%	0.0	99%	AY423438.1	892,36	97,9%
Select seq gb|AY423431.1|	Ephedra sinica tRNA-Leu (trnL) gene, partial sequence; trnL-trnF intergenic spacer, complete sequence; and tRNA-Phe (trnF) gene, partial sequence; chloroplast	656	656	73%	0.0	99%	AY423431.1	889,64	97,6%
Select seq gb|AY423439.1|	Ephedra minuta voucher 1017 tRNA-Leu (trnL) gene, partial sequence; trnL-trnF intergenic spacer, complete sequence; and tRNA-Phe (trnF) gene, partial sequence; chloroplast	652	652	73%	0.0	99%	AY423439.1	884,22	97,0%
Select seq gb|KT934791.1|	Ephedra foeminea voucher K. Bolinder 542 chloroplast, complete genome	604	604	75%	4,00E-169	95%	KT934791.1	765,07	83,9%
Select seq gb|GU111523.1|	Ephedra foeminea voucher H. Freitag 19.807 (KAS) tRNA-Leu (trnL) gene, partial sequence; trnL-trnF intergenic spacer, complete sequence; and tRNA-Phe (trnF) gene, partial sequence; chloroplast	569	569	71%	1,00E-158	95%	GU111523.1	761,34	83,5%
Select seq gb|EU571226.1|	Aglaomorpha coronans voucher WZQXHG076021 trnL-trnF intergenic spacer, partial sequence; chloroplast	285	285	31%	6,00E-73	100%	EU571226.1	0,00	0,0%
Select seq gb|EU571224.1|	Drynaria quercifolia voucher WQZXHG062021 trnL-trnF intergenic spacer, partial sequence; chloroplast	285	285	31%	6,00E-73	100%	EU571224.1	0,00	0,0%
Select seq gb|EU571223.1|	Drynaria rigidula voucher XHGXCY061010 trnL-trnF intergenic spacer, partial sequence; chloroplast	285	285	31%	6,00E-73	100%	EU571223.1	0,00	0,0%
Select seq gb|EU571221.1|	Drynaria fortunei voucher XHGXCY071016 trnL-trnF intergenic spacer, partial sequence; chloroplast	285	285	31%	6,00E-73	100%	EU571221.1	0,00	0,0%
Select seq gb|KT033391.1|	Ephedra monosperma haplotype H14 tRNA-Thr (trnT) gene, partial sequence; trnT-trnL intergenic spacer and tRNA-Leu (trnL) gene, complete sequence; and trnL-trnF intergenic spacer, partial sequence; chloroplast	569	569	62%	1,00E-158	99%	KT033391.1	0,00	0,0%
Select seq gb|KC407800.1|	Ephedra equisetina isolate H23 tRNA-Thr (trnT) gene, partial sequence; trnT-trnL intergenic spacer and tRNA-Leu (trnL) gene, complete sequence; and trnL-trnF intergenic spacer, partial sequence; chloroplast	569	569	62%	1,00E-158	99%	KC407800.1	0,00	0,0%
Select seq gb|KC407798.1|	Ephedra przewalskii isolate H21 tRNA-Thr (trnT) gene, partial sequence; trnT-trnL intergenic spacer and tRNA-Leu (trnL) gene, complete sequence; and trnL-trnF intergenic spacer, partial sequence; chloroplast	569	569	62%	1,00E-158	99%	KC407798.1	0,00	0,0%
Select seq gb|KC407796.1|	Ephedra intermedia isolate H19 tRNA-Thr (trnT) gene, partial sequence; trnT-trnL intergenic spacer and tRNA-Leu (trnL) gene, complete sequence; and trnL-trnF intergenic spacer, partial sequence; chloroplast	569	569	62%	1,00E-158	99%	KC407796.1	0,00	0,0%
Select seq gb|KC407793.1|	Ephedra intermedia isolate H16 tRNA-Thr (trnT) gene, partial sequence; trnT-trnL intergenic spacer and tRNA-Leu (trnL) gene, complete sequence; and trnL-trnF intergenic spacer, partial sequence; chloroplast	569	569	62%	1,00E-158	99%	KC407793.1	0,00	0,0%
Select seq gb|KC407792.1|	Ephedra likiangensis isolate H15 tRNA-Thr (trnT) gene, partial sequence; trnT-trnL intergenic spacer and tRNA-Leu (trnL) gene, complete sequence; and trnL-trnF intergenic spacer, partial sequence; chloroplast	569	569	62%	1,00E-158	99%	KC407792.1	0,00	0,0%
Select seq gb|KC407784.1|	Ephedra rituensis isolate H7 tRNA-Thr (trnT) gene, partial sequence; trnT-trnL intergenic spacer and tRNA-Leu (trnL) gene, complete sequence; and trnL-trnF intergenic spacer, partial sequence; chloroplast	569	569	62%	1,00E-158	99%	KC407784.1	0,00	0,0%
Select seq gb|KC407783.1|	Ephedra minuta isolate H6 tRNA-Thr (trnT) gene, partial sequence; trnT-trnL intergenic spacer and tRNA-Leu (trnL) gene, complete sequence; and trnL-trnF intergenic spacer, partial sequence; chloroplast	569	569	62%	1,00E-158	99%	KC407783.1	0,00	0,0%
Select seq gb|EU326056.1|	Ephedra gerardiana tRNA-Leu (trnL) gene, intron; chloroplast	329	329	36%	3,00E-86	99%	EU326056.1	0,00	0,0%

Select seq gb|JX217730.1|	Ephedra distachya voucher s.n. (MEXU) tRNA-Thr (trnT) gene, partial sequence; trnT-trnL intergenic spacer, complete sequence; and tRNA-Leu (trnL) gene, partial sequence; chloroplast	292	292	32%	4,00E-75	99%	JX217730.1	0,00	0,0%
Select seq gb|JX217719.1|	Ephedra antisyphilitica voucher Loera 22 (XAL) tRNA-Thr (trnT) gene, partial sequence; trnT-trnL intergenic spacer, complete sequence; and tRNA-Leu (trnL) gene, partial sequence; chloroplast	292	292	32%	4,00E-75	99%	JX217719.1	0,00	0,0%
Select seq gb|AY730600.1|	Ephedra przewalskii voucher Kamp03072010 tRNA-Leu (trnL) gene and trnL-trnF intergenic spacer, partial sequence; chloroplast	601	601	66%	5,00E-168	99%	AY730600.1	0,00	0,0%
Select seq gb|KT033395.1|	Ephedra sinica haplotype H8 tRNA-Thr (trnT) gene, partial sequence; trnT-trnL intergenic spacer and tRNA-Leu (trnL) gene, complete sequence; and trnL-trnF intergenic spacer, partial sequence; chloroplast	564	564	62%	7,00E-157	99%	KT033395.1	0,00	0,0%
Select seq gb|KT033392.1|	Ephedra equisetina haplotype H15 tRNA-Thr (trnT) gene, partial sequence; trnT-trnL intergenic spacer and tRNA-Leu (trnL) gene, complete sequence; and trnL-trnF intergenic spacer, partial sequence; chloroplast	564	564	62%	7,00E-157	99%	KT033392.1	0,00	0,0%
Select seq gb|KC407797.1|	Ephedra przewalskii isolate H20 tRNA-Thr (trnT) gene, partial sequence; trnT-trnL intergenic spacer and tRNA-Leu (trnL) gene, complete sequence; and trnL-trnF intergenic spacer, partial sequence; chloroplast	564	564	62%	7,00E-157	99%	KC407797.1	0,00	0,0%
Select seq gb|KC407787.1|	Ephedra minuta isolate H10 tRNA-Thr (trnT) gene, partial sequence; trnT-trnL intergenic spacer and tRNA-Leu (trnL) gene, complete sequence; and trnL-trnF intergenic spacer, partial sequence; chloroplast	564	564	62%	7,00E-157	99%	KC407787.1	0,00	0,0%
Select seq gb|KC407780.1|	Ephedra gerardiana isolate H3 tRNA-Thr (trnT) gene, partial sequence; trnT-trnL intergenic spacer and tRNA-Leu (trnL) gene, complete sequence; and trnL-trnF intergenic spacer, partial sequence; chloroplast	564	564	62%	7,00E-157	99%	KC407780.1	0,00	0,0%
Select seq gb|KC407778.1|	Ephedra gerardiana isolate H1 tRNA-Thr (trnT) gene, partial sequence; trnT-trnL intergenic spacer and tRNA-Leu (trnL) gene, complete sequence; and trnL-trnF intergenic spacer, partial sequence; chloroplast	564	564	62%	7,00E-157	99%	KC407778.1	0,00	0,0%
Select seq gb|AY513736.1|	Ephedra torreyana tRNA-Thr (trnT) gene, partial sequence; and trnT-trnL intergenic spacer and tRNA-Leu (trnL) gene, complete sequence; chloroplast	363	363	40%	3,00E-96	99%	AY513736.1	0,00	0,0%
Select seq gb|KP788796.1|	Ephedra minuta voucher JBM1106 00 tRNA-Leu (trnL) gene, partial sequence; chloroplast	335	335	37%	6,00E-88	99%	KP788796.1	0,00	0,0%
Select seq gb|KP788806.1|	Ephedra tweediana voucher Ickert-Bond 1225 (ASU) tRNA-Thr (trnT) gene, partial sequence; trnT-trnL intergenic spacer, complete sequence; and tRNA-Leu (trnL) gene, partial sequence; chloroplast	344	344	38%	1,00E-90	99%	KP788806.1	0,00	0,0%
Select seq gb|KP788805.1|	Ephedra triandra voucher Ickert-Bond 1227 (ASU) tRNA-Thr (trnT) gene, partial sequence; trnT-trnL intergenic spacer, complete sequence; and tRNA-Leu (trnL) gene, partial sequence; chloroplast	344	344	38%	1,00E-90	99%	KP788805.1	0,00	0,0%
Select seq gb|KP788803.1|	Ephedra sinica voucher 986 tRNA-Thr (trnT) gene, partial sequence; trnT-trnL intergenic spacer, complete sequence; and tRNA-Leu (trnL) gene, partial sequence; chloroplast	344	344	38%	1,00E-90	99%	KP788803.1	0,00	0,0%
Select seq gb|KP788801.1|	Ephedra rupestris voucher Ickert-Bond 1110 (ASU) tRNA-Thr (trnT) gene, partial sequence; trnT-trnL intergenic spacer, complete sequence; and tRNA-Leu (trnL) gene, partial sequence; chloroplast	344	344	38%	1,00E-90	99%	KP788801.1	0,00	0,0%
Select seq gb|KP788800.1|	Ephedra pseudodistachya voucher Freitag 33002 (KAS) tRNA-Thr (trnT) gene, partial sequence; trnT-trnL intergenic spacer, complete sequence; and tRNA-Leu (trnL) gene, partial sequence; chloroplast	344	344	38%	1,00E-90	99%	KP788800.1	0,00	0,0%
Select seq gb|KP788797.1|	Ephedra monosperma voucher Freitag 33031 (KAS) tRNA-Thr (trnT) gene, partial sequence; trnT-trnL intergenic spacer, complete sequence; and tRNA-Leu (trnL) gene, partial sequence; chloroplast	344	344	38%	1,00E-90	99%	KP788797.1	0,00	0,0%
Select seq gb|KP788795.1|	Ephedra likiangensis voucher Boufford 30871 (GH) tRNA-Leu (trnL) gene, partial sequence; chloroplast	344	344	38%	1,00E-90	99%	KP788795.1	0,00	0,0%
Select seq gb|KP788794.1|	Ephedra intermedia voucher XJ 08070801 tRNA-Thr (trnT) gene, partial sequence; trnT-trnL intergenic spacer, complete sequence; and tRNA-Leu (trnL) gene, partial sequence; chloroplast	344	344	38%	1,00E-90	99%	KP788794.1	0,00	0,0%
Select seq gb|KP788793.1|	Ephedra gracilis voucher Ickert-Bond 1201 (ASU) tRNA-Thr (trnT) gene, partial sequence; trnT-trnL intergenic spacer, complete sequence; and tRNA-Leu (trnL) gene, partial sequence; chloroplast	344	344	38%	1,00E-90	99%	KP788793.1	0,00	0,0%
Select seq gb|KP788792.1|	Ephedra intermedia voucher YYNH08070502 tRNA-Thr (trnT) gene, partial sequence; trnT-trnL intergenic spacer, complete sequence; and tRNA-Leu (trnL) gene, partial sequence; chloroplast	344	344	38%	1,00E-90	99%	KP788792.1	0,00	0,0%
Select seq gb|KP788784.1|	Ephedra americana voucher Ickert-Bond 1105 (ASU) tRNA-Thr (trnT) gene, partial sequence; trnT-trnL intergenic spacer, complete sequence; and tRNA-Leu (trnL) gene, partial sequence; chloroplast	344	344	38%	1,00E-90	99%	KP788784.1	0,00	0,0%
Select seq gb|AY730598.1|	Ephedra przewalskii voucher Kamp03071340 tRNA-Leu (trnL) gene and trnL-trnF intergenic spacer, partial sequence; chloroplast	595	595	66%	2,00E-166	99%	AY730598.1	0,00	0,0%
Select seq gb|KT033390.1|	Ephedra foeminea haplotype H24 tRNA-Thr (trnT) gene, partial sequence; trnT-trnL intergenic spacer and tRNA-Leu (trnL) gene, complete sequence; and trnL-trnF intergenic spacer, partial sequence; chloroplast	558	558	62%	3,00E-155	99%	KT033390.1	0,00	0,0%
Select seq gb|KC407786.1|	Ephedra saxatilis var. mairei isolate H9 tRNA-Thr (trnT) gene, partial sequence; trnT-trnL intergenic spacer and tRNA-Leu (trnL) gene, complete sequence; and trnL-trnF intergenic spacer, partial sequence; chloroplast	558	558	62%	3,00E-155	99%	KC407786.1	0,00	0,0%
Select seq gb|KC407781.1|	Ephedra saxatilis isolate H4 tRNA-Thr (trnT) gene, partial sequence; trnT-trnL intergenic spacer and tRNA-Leu (trnL) gene, complete sequence; and trnL-trnF intergenic spacer, partial sequence; chloroplast	558	558	62%	3,00E-155	99%	KC407781.1	0,00	0,0%
Select seq gb|EU571225.1|	Drynaria mollis voucher XHGXCY051011 trnL-trnF intergenic spacer, partial sequence; chloroplast	279	279	31%	3,00E-71	99%	EU571225.1	0,00	0,0%
Select seq gb|KP788807.1|	Ephedra viridis voucher Elias & Morefield 10376 (MEXU) tRNA-Thr (trnT) gene, partial sequence; trnT-trnL intergenic spacer, complete sequence; and tRNA-Leu (trnL) gene, partial sequence; chloroplast	287	287	32%	2,00E-73	99%	KP788807.1	0,00	0,0%

Select seq gb|KP788804.1|	Ephedra torreyana voucher Gentry & Engard 23198 (MEXU) tRNA-Thr (trnT) gene, partial sequence; trnT-trnL intergenic spacer, complete sequence; and tRNA-Leu (trnL) gene, partial sequence; chloroplast	287	287	32%	2,00E-73	99%	KP788804.1	0,00	0,0%
Select seq gb|KP788791.1|	Ephedra funerea voucher Ickert-Bond 473 (ASU) tRNA-Thr (trnT) gene, partial sequence; trnT-trnL intergenic spacer, complete sequence; and tRNA-Leu (trnL) gene, partial sequence; chloroplast	287	287	32%	2,00E-73	99%	KP788791.1	0,00	0,0%
Select seq gb|KP788790.1|	Ephedra funerea voucher Ickert-Bond 964 (ASU) tRNA-Thr (trnT) gene, partial sequence; trnT-trnL intergenic spacer, complete sequence; and tRNA-Leu (trnL) gene, partial sequence; chloroplast	287	287	32%	2,00E-73	99%	KP788790.1	0,00	0,0%
Select seq gb|JX217737.1|	Ephedra trifurca voucher Reina-G. & Van Devender 96-208 (MEXU) tRNA-Thr (trnT) gene, partial sequence; trnT-trnL intergenic spacer, complete sequence; and tRNA-Leu (trnL) gene, partial sequence; chloroplast	287	287	32%	2,00E-73	99%	JX217737.1	0,00	0,0%
Select seq gb|JX217734.1|	Ephedra nevadensis voucher Felger & Dimmitt 85-724 (MEXU) tRNA-Thr (trnT) gene, partial sequence; trnT-trnL intergenic spacer, complete sequence; and tRNA-Leu (trnL) gene, partial sequence; chloroplast	287	287	32%	2,00E-73	99%	JX217734.1	0,00	0,0%
Select seq gb|JX217733.1|	Ephedra nevadensis voucher Phillips & Phillips 93141 (MEXU) tRNA-Thr (trnT) gene, partial sequence; trnT-trnL intergenic spacer, complete sequence; and tRNA-Leu (trnL) gene, partial sequence; chloroplast	287	287	32%	2,00E-73	99%	JX217733.1	0,00	0,0%
Select seq gb|JX217721.1|	Ephedra aspera voucher Spellenberg & Bacon s.n. (MEXU) tRNA-Thr (trnT) gene, partial sequence; trnT-trnL intergenic spacer, complete sequence; and tRNA-Leu (trnL) gene, partial sequence; chloroplast	287	287	32%	2,00E-73	99%	JX217721.1	0,00	0,0%
Select seq gb|KC407788.1|	Ephedra saxatilis var. mairei isolate H11 tRNA-Thr (trnT) gene, partial sequence; trnT-trnL intergenic spacer and tRNA-Leu (trnL) gene, complete sequence; and trnL-trnF intergenic spacer, partial sequence; chloroplast	555	555	62%	4,00E-154	99%	KC407788.1	0,00	0,0%
Select seq gb|AY513735.1|	Ephedra viridis tRNA-Thr (trnT) gene, partial sequence; and tRNA-Leu (trnL) gene, complete sequence; chloroplast	357	357	40%	1,00E-94	99%	AY513735.1	0,00	0,0%
Select seq gb|AY513734.1|	Ephedra trifurca tRNA-Thr (trnT) gene, partial sequence; and trnT-trnL intergenic spacer and tRNA-Leu (trnL) gene, complete sequence; chloroplast	357	357	40%	1,00E-94	99%	AY513734.1	0,00	0,0%
Select seq gb|KP788802.1|	Ephedra rupestris voucher Ickert-Bond 1220 (ASU) tRNA-Thr (trnT) gene, partial sequence; trnT-trnL intergenic spacer, complete sequence; and tRNA-Leu (trnL) gene, partial sequence; chloroplast	339	339	38%	4,00E-89	99%	KP788802.1	0,00	0,0%
Select seq gb|KP788799.1|	Ephedra ochreata voucher Muhlen s.n. tRNA-Thr (trnT) gene, partial sequence; trnT-trnL intergenic spacer, complete sequence; and tRNA-Leu (trnL) gene, partial sequence; chloroplast	339	339	38%	4,00E-89	99%	KP788799.1	0,00	0,0%
Select seq gb|KP788798.1|	Ephedra multiflora voucher Ickert-Bond 1211 (ASU) tRNA-Thr (trnT) gene, partial sequence; trnT-trnL intergenic spacer, complete sequence; and tRNA-Leu (trnL) gene, partial sequence; chloroplast	339	339	38%	4,00E-89	99%	KP788798.1	0,00	0,0%
Select seq gb|KP788789.1|	Ephedra frustillata voucher Ickert-Bond 1247 (ASU) tRNA-Thr (trnT) gene, partial sequence; trnT-trnL intergenic spacer, complete sequence; and tRNA-Leu (trnL) gene, partial sequence; chloroplast	339	339	38%	4,00E-89	99%	KP788789.1	0,00	0,0%
Select seq gb|KP788788.1|	Ephedra breana voucher Ickert-Bond 1234 (ASU) tRNA-Thr (trnT) gene, partial sequence; trnT-trnL intergenic spacer, complete sequence; and tRNA-Leu (trnL) gene, partial sequence; chloroplast	339	339	38%	4,00E-89	99%	KP788788.1	0,00	0,0%
Select seq gb|KP788787.1|	Ephedra boelckei voucher Ickert-Bond 1252 (ASU) tRNA-Thr (trnT) gene, partial sequence; trnT-trnL intergenic spacer, complete sequence; and tRNA-Leu (trnL) gene, partial sequence; chloroplast	339	339	38%	4,00E-89	99%	KP788787.1	0,00	0,0%
Select seq gb|KP788786.1|	Ephedra boelckei voucher Ickert-Bond 1251 (ASU) trnT-trnL intergenic spacer and tRNA-Leu (trnL) gene, partial sequence; chloroplast	339	339	38%	4,00E-89	99%	KP788786.1	0,00	0,0%
Select seq gb|KP788785.1|	Ephedra aphylla voucher Freitag 30181 (KAS) tRNA-Thr (trnT) gene, partial sequence; trnT-trnL intergenic spacer, complete sequence; and tRNA-Leu (trnL) gene, partial sequence; chloroplast	339	339	38%	4,00E-89	99%	KP788785.1	0,00	0,0%
Select seq gb|KC407803.1|	Ephedra major isolate H26 tRNA-Thr (trnT) gene, partial sequence; trnT-trnL intergenic spacer and tRNA-Leu (trnL) gene, complete sequence; and trnL-trnF intergenic spacer, partial sequence; chloroplast	553	553	62%	1,00E-153	99%	KC407803.1	0,00	0,0%
Select seq gb|KC407790.1|	Ephedra saxatilis var. mairei isolate H13 tRNA-Thr (trnT) gene, partial sequence; and trnT-trnL intergenic spacer and tRNA-Leu (trnL) gene, complete sequence; chloroplast	553	553	62%	1,00E-153	99%	KC407790.1	0,00	0,0%
Select seq gb|KC407789.1|	Ephedra saxatilis var. mairei isolate H12 tRNA-Thr (trnT) gene, partial sequence; trnT-trnL intergenic spacer and tRNA-Leu (trnL) gene, complete sequence; and trnL-trnF intergenic spacer, partial sequence; chloroplast	553	553	62%	1,00E-153	99%	KC407789.1	0,00	0,0%
Select seq gb|EU571222.1|	Drynaria sparsisora voucher WQZXHG056021 trnL-trnF intergenic spacer, partial sequence; chloroplast	272	272	31%	5,00E-69	99%	EU571222.1	0,00	0,0%
Select seq gb|KP099649.1|	Gnetum gnemon plastid, complete genome	102	102	15%	6,00E-18	90%	KP099649.1	0,00	0,0%
Select seq gb|AY296521.1|	Gnetum ula tRNA-Thr (trnT) gene, partial sequence; trnT-trnL intergenic spacer, tRNA-Leu (trnL) gene, and trnL-trnF intergenic spacer, complete sequence; and tRNA-Phe (trnF) gene, partial sequence; chloroplast	102	102	15%	6,00E-18	90%	AY296521.1	0,00	0,0%
Select seq gb|AY296517.1|	Gnetum parvifolium tRNA-Thr (trnT) gene, partial sequence; trnT-trnL intergenic spacer, tRNA-Leu (trnL) gene, and trnL-trnF intergenic spacer, complete sequence; and tRNA-Phe (trnF) gene, partial sequence; chloroplast	102	102	15%	6,00E-18	90%	AY296517.1	0,00	0,0%
Select seq gb|AY296509.1|	Gnetum latifolium tRNA-Thr (trnT) gene, partial sequence; trnT-trnL intergenic spacer, tRNA-Leu (trnL) gene, and trnL-trnF intergenic spacer, complete sequence; and tRNA-Phe (trnF) gene, partial sequence; chloroplast	102	102	15%	6,00E-18	90%	AY296509.1	0,00	0,0%
Select seq gb|AY296505.1|	Gnetum latifolium tRNA-Thr (trnT) gene, partial sequence; trnT-trnL intergenic spacer, tRNA-Leu (trnL) gene, and trnL-trnF intergenic spacer, complete sequence; and tRNA-Phe (trnF) gene, partial sequence; chloroplast	102	102	15%	6,00E-18	90%	AY296505.1	0,00	0,0%
Select seq gb|AY296494.1|	Gnetum gnemon tRNA-Thr (trnT) gene, partial sequence; trnT-trnL intergenic spacer, tRNA-Leu (trnL) gene, and trnL-trnF intergenic spacer, complete sequence; and tRNA-Phe (trnF) gene, partial sequence; chloroplast	102	102	15%	6,00E-18	90%	AY296494.1	0,00	0,0%

Select seq gb|AY296488.1|	Gnetum costatum tRNA-Thr (trnT) gene, partial sequence; trnT-trnL intergenic spacer, tRNA-Leu (trnL) gene, and trnL-trnF intergenic spacer, complete sequence; and tRNA-Phe (trnF) gene, partial sequence; chloroplast	102	102	15%	6,00E-18	90% AY296488.1	0,00	0,0%
Select seq gb|AY445621.1|	Gnetum leptostachyum tRNA-Leu (trnL) gene and trnL-trnF intergenic spacer, partial sequence	102	102	15%	6,00E-18	90% AY445621.1	0,00	0,0%
Select seq gb|AY304546.1|	Gnetum gnemon trnT-trnL spacer, partial sequence; trnL gene, complete sequence; and trnL-trnF-spacer, partial sequence	102	102	15%	6,00E-18	90% AY304546.1	0,00	0,0%
Select seq gb|AY296506.1|	Gnetum latifolium tRNA-Thr (trnT) gene, partial sequence; trnT-trnL intergenic spacer, tRNA-Leu (trnL) gene, and trnL-trnF intergenic spacer, complete sequence; and tRNA-Phe (trnF) gene, partial sequence; chloroplast	102	157	21%	6,00E-18	90% AY296506.1	0,00	0,0%
Select seq gb|AY296525.1|	Gnetum sp. Takeuchi et al. 7049 tRNA-Thr (trnT) gene, partial sequence; trnT-trnL intergenic spacer, tRNA-Leu (trnL) gene, and trnL-trnF intergenic spacer, complete sequence; and tRNA-Phe (trnF) gene, partial sequence; chloroplast	102	175	23%	6,00E-18	90% AY296525.1	0,00	0,0%
Select seq gb|AY296520.1|	Gnetum tenuifolium tRNA-Thr (trnT) gene, partial sequence; trnT-trnL intergenic spacer, tRNA-Leu (trnL) gene, and trnL-trnF intergenic spacer, complete sequence; and tRNA-Phe (trnF) gene, partial sequence; chloroplast	102	175	23%	6,00E-18	90% AY296520.1	0,00	0,0%
Select seq gb|AY296511.1|	Gnetum macrostachyum tRNA-Thr (trnT) gene, partial sequence; trnT-trnL intergenic spacer, tRNA-Leu (trnL) gene, and trnL-trnF intergenic spacer, complete sequence; and tRNA-Phe (trnF) gene, partial sequence; chloroplast	102	175	23%	6,00E-18	90% AY296511.1	0,00	0,0%
Select seq gb|AY296503.1|	Gnetum klossii tRNA-Thr (trnT) gene, partial sequence; trnT-trnL intergenic spacer, tRNA-Leu (trnL) gene, and trnL-trnF intergenic spacer, complete sequence; and tRNA-Phe (trnF) gene, partial sequence; chloroplast	102	175	23%	6,00E-18	90% AY296503.1	0,00	0,0%
Select seq gb|AY296485.1|	Gnetum acutum tRNA-Thr (trnT) gene, partial sequence; trnT-trnL intergenic spacer, tRNA-Leu (trnL) gene, and trnL-trnF intergenic spacer, complete sequence; and tRNA-Phe (trnF) gene, partial sequence; chloroplast	102	175	23%	6,00E-18	90% AY296485.1	0,00	0,0%
Select seq dbj|AP014923.1|	Gnetum ula chloroplast DNA, complete genome	102	186	25%	6,00E-18	90% AP014923.1	0,00	0,0%
Select seq gb|KC427271.1|	Gnetum montanum chloroplast, complete genome	102	186	25%	6,00E-18	90% KC427271.1	0,00	0,0%
Select seq dbj|AP009569.1|	Gnetum parvifolium chloroplast DNA, complete genome	102	186	25%	6,00E-18	90% AP009569.1	0,00	0,0%
Select seq gb|AY296508.1|	Gnetum aff. latifolium SAN151116 tRNA-Thr (trnT) gene, partial sequence; trnT-trnL intergenic spacer, tRNA-Leu (trnL) gene, and trnL-trnF intergenic spacer, complete sequence; and tRNA-Phe (trnF) gene, partial sequence; chloroplast	102	186	25%	6,00E-18	90% AY296508.1	0,00	0,0%
Select seq gb|AY296487.1|	Gnetum africanum tRNA-Thr (trnT) gene, partial sequence; trnT-trnL intergenic spacer, tRNA-Leu (trnL) gene, and trnL-trnF intergenic spacer, complete sequence; and tRNA-Phe (trnF) gene, partial sequence; chloroplast	102	186	25%	6,00E-18	90% AY296487.1	0,00	0,0%
Select seq gb|AY296486.1|	Gnetum africanum tRNA-Thr (trnT) gene, partial sequence; trnT-trnL intergenic spacer, tRNA-Leu (trnL) gene, and trnL-trnF intergenic spacer, complete sequence; and tRNA-Phe (trnF) gene, partial sequence; chloroplast	102	186	25%	6,00E-18	90% AY296486.1	0,00	0,0%
Select seq gb|AY296522.1|	Gnetum urens tRNA-Thr (trnT) gene, partial sequence; trnT-trnL intergenic spacer, tRNA-Leu (trnL) gene, and trnL-trnF intergenic spacer, complete sequence; and tRNA-Phe (trnF) gene, partial sequence; chloroplast	97,10	181	25%	3,00E-16	89% AY296522.1	0,00	0,0%
Select seq gb|AY296515.1|	Gnetum nodiflorum tRNA-Thr (trnT) gene, partial sequence; trnT-trnL intergenic spacer, tRNA-Leu (trnL) gene, and trnL-trnF intergenic spacer, complete sequence; and tRNA-Phe (trnF) gene, partial sequence; chloroplast	97,10	179	25%	3,00E-16	89% AY296515.1	0,00	0,0%

Select for downloading or viewing reports	Kh033_trnL Description	Max score	Total score	Query cover	E value	Ident	Accession	(Ident/Cover)* Max score	Deviation from top hit
Select seq emb|AJ505539.1|	Vitex trifolia plastid trnL-trnF intergenic spacer, specimen voucher TCMK 15, Chase 8757 (K)	1230	1230	94%	0.0	99%	AJ505539.1	1295,43	100,0%
Select seq gb|HQ412926.1|	Vitex agnus-castus tRNA-Leu (trnL) gene and trnL-trnF intergenic spacer, partial sequence; chloroplast	1249	1249	96%	0.0	99%	HQ412926.1	1288,03	99,4%
Select seq gb|JN408589.1|	Verbena officinalis tRNA-Leu (trnL) gene, partial sequence; trnL-trnF intergenic spacer, complete sequence; and tRNA-Phe (trnF) gene, partial sequence; chloroplast	1260	1260	97%	0.0	99%	JN408589.1	1285,98	99,3%
Select seq gb|FJ952070.1|	Aegiphila alba tRNA-Leu (trnL) gene, partial sequence; trnL-trnF intergenic spacer, complete sequence; and tRNA-Phe (trnF) gene, partial sequence; chloroplast	942	942	99%	0.0	91%	FJ952070.1	865,88	66,8%
Select seq gb|HQ412925.1|	Premna odorata tRNA-Leu (trnL) gene and trnL-trnF intergenic spacer, partial sequence; chloroplast	909	909	96%	0.0	91%	HQ412925.1	861,66	66,5%
Select seq gb|FJ952071.1|	Aegiphila anomala tRNA-Leu (trnL) gene, partial sequence; trnL-trnF intergenic spacer, complete sequence; and tRNA-Phe (trnF) gene, partial sequence; chloroplast	937	937	99%	0.0	91%	FJ952071.1	861,28	66,5%
Select seq gb|EU827122.1|	Paraphlomis membranacea voucher Fang091057 (KUN) tRNA-Leu (trnL) gene, partial sequence; trnL-trnF intergenic spacer, complete sequence; and tRNA-Phe (trnF) gene, partial sequence; chloroplast	898	898	95%	0.0	91%	EU827122.1	860,19	66,4%
Select seq gb|JN408638.1|	Teucrium kotschyanum tRNA-Leu (trnL) gene, partial sequence; and trnL-trnF intergenic spacer, complete sequence; chloroplast	850	850	90%	0.0	91%	JN408638.1	859,44	66,3%
Select seq gb|FJ952039.1|	Clerodendrum hildebrandtii var. puberula tRNA-Leu (trnL) gene, partial sequence; trnL-trnF intergenic spacer, complete sequence; and tRNA-Phe (trnF) gene, partial sequence; chloroplast	933	933	99%	0.0	91%	FJ952039.1	857,61	66,2%
Select seq gb|FJ952073.1|	Aegiphila martinicensis tRNA-Leu (trnL) gene, partial sequence; trnL-trnF intergenic spacer, complete sequence; and tRNA-Phe (trnF) gene, partial sequence; chloroplast	931	931	99%	0.0	91%	FJ952073.1	855,77	66,1%
Select seq gb|FJ952069.1|	Aegiphila multiflora tRNA-Leu (trnL) gene, partial sequence; trnL-trnF intergenic spacer, complete sequence; and tRNA-Phe (trnF) gene, partial sequence; chloroplast	931	931	99%	0.0	91%	FJ952069.1	855,77	66,1%
Select seq gb|FJ952072.1|	Aegiphila elata tRNA-Leu (trnL) gene, partial sequence; trnL-trnF intergenic spacer, complete sequence; and tRNA-Phe (trnF) gene, partial sequence; chloroplast	926	926	99%	0.0	91%	FJ952072.1	851,17	65,7%
Select seq gb|FJ952058.1|	Clerodendrum inerme tRNA-Leu (trnL) gene, partial sequence; trnL-trnF intergenic spacer, complete sequence; and tRNA-Phe (trnF) gene, partial sequence; chloroplast	926	926	99%	0.0	91%	FJ952058.1	851,17	65,7%
Select seq gb|FJ952052.1|	Clerodendrum tomentosum tRNA-Leu (trnL) gene, partial sequence; trnL-trnF intergenic spacer, complete sequence; and tRNA-Phe (trnF) gene, partial sequence; chloroplast	922	922	99%	0.0	91%	FJ952052.1	847,49	65,4%
Select seq gb|FJ952075.1|	Aegiphila brachiata tRNA-Leu (trnL) gene, partial sequence; trnL-trnF intergenic spacer, complete sequence; and tRNA-Phe (trnF) gene, partial sequence; chloroplast	920	920	99%	0.0	91%	FJ952075.1	845,66	65,3%
Select seq gb|FJ952074.1|	Aegiphila hassleri tRNA-Leu (trnL) gene, partial sequence; trnL-trnF intergenic spacer, complete sequence; and tRNA-Phe (trnF) gene, partial sequence; chloroplast	920	920	99%	0.0	91%	FJ952074.1	845,66	65,3%
Select seq gb|FJ952065.1|	Clerodendrum spinosum tRNA-Leu (trnL) gene, partial sequence; trnL-trnF intergenic spacer, complete sequence; and tRNA-Phe (trnF) gene, partial sequence; chloroplast	920	920	99%	0.0	91%	FJ952065.1	845,66	65,3%
Select seq gb|FJ870062.1|	Tectona grandis tRNA-Leu (trnL) gene, partial sequence; trnL-trnF intergenic spacer, complete sequence; and tRNA-Phe (trnF) gene, partial sequence; chloroplast	920	920	99%	0.0	91%	FJ870062.1	845,66	65,3%
Select seq gb|FJ952050.1|	Clerodendrum fortunatum tRNA-Leu (trnL) gene, partial sequence; trnL-trnF intergenic spacer, complete sequence; and tRNA-Phe (trnF) gene, partial sequence; chloroplast	917	917	99%	0.0	91%	FJ952050.1	842,90	65,1%
Select seq gb|FJ952047.1|	Clerodendrum canescens tRNA-Leu (trnL) gene, partial sequence; trnL-trnF intergenic spacer, complete sequence; and tRNA-Phe (trnF) gene, partial sequence; chloroplast	917	917	99%	0.0	91%	FJ952047.1	842,90	65,1%
Select seq gb|FJ952037.1|	Clerodendrum cephalanthum subsp. mashariki tRNA-Leu (trnL) gene, partial sequence; trnL-trnF intergenic spacer, complete sequence; and tRNA-Phe (trnF) gene, partial sequence; chloroplast	917	917	99%	0.0	91%	FJ952037.1	842,90	65,1%
Select seq emb|HF567871.1|	Tectona grandis complete chloroplast genome, haplotype 3	922	922	100%	0.0	91%	HF567871.1	839,02	64,8%
Select seq emb|HF567870.1|	Tectona grandis complete chloroplast genome, haplotype 2	922	922	100%	0.0	91%	HF567870.1	839,02	64,8%
Select seq emb|HF567869.1|	Tectona grandis complete chloroplast genome, haplotype 1	922	922	100%	0.0	91%	HF567869.1	839,02	64,8%
Select seq gb|JN408604.1|	Teucrium betonicum tRNA-Leu (trnL) gene, partial sequence; trnL-trnF intergenic spacer, complete sequence; and tRNA-Phe (trnF) gene, partial sequence; chloroplast	870	870	94%	0.0	90%	JN408604.1	832,98	64,3%
Select seq gb|JN408652.1|	Teucrium oxylepis tRNA-Leu (trnL) gene, partial sequence; and trnL-trnF intergenic spacer, complete sequence; chloroplast	869	869	94%	0.0	90%	JN408652.1	832,02	64,2%

Select seq gb|FJ952064.1|	Clerodendrum ligustrinum tRNA-Leu (trnL) gene, partial sequence; trnL-trnF intergenic spacer, complete sequence; and tRNA-Phe (trnF) gene, partial sequence; chloroplast	915	915	99% 0.0	90% FJ952064.1	831,82	64,2%
Select seq gb|FJ952056.1|	Clerodendrum indicum tRNA-Leu (trnL) gene, partial sequence; trnL-trnF intergenic spacer, complete sequence; and tRNA-Phe (trnF) gene, partial sequence; chloroplast	915	915	99% 0.0	90% FJ952056.1	831,82	64,2%
Select seq gb|FJ952036.1|	Clerodendrum capitatum tRNA-Leu (trnL) gene, partial sequence; trnL-trnF intergenic spacer, complete sequence; and tRNA-Phe (trnF) gene, partial sequence; chloroplast	915	915	99% 0.0	90% FJ952036.1	831,82	64,2%
Select seq gb|FJ952051.1|	Clerodendrum trichotomum tRNA-Leu (trnL) gene, partial sequence; trnL-trnF intergenic spacer, complete sequence; and tRNA-Phe (trnF) gene, partial sequence; chloroplast	911	911	99% 0.0	90% FJ952051.1	828,18	63,9%
Select seq gb|FJ952048.1|	Clerodendrum cyrtophyllum tRNA-Leu (trnL) gene, partial sequence; trnL-trnF intergenic spacer, complete sequence; and tRNA-Phe (trnF) gene, partial sequence; chloroplast	911	911	99% 0.0	90% FJ952048.1	828,18	63,9%
Select seq gb|FJ952038.1|	Clerodendrum cephalanthum subsp. montanum tRNA-Leu (trnL) gene, partial sequence; trnL-trnF intergenic spacer, complete sequence; and tRNA-Phe (trnF) gene, partial sequence; chloroplast	911	911	99% 0.0	90% FJ952038.1	828,18	63,9%
Select seq gb|FJ952035.1|	Clerodendrum rotundifolium tRNA-Leu (trnL) gene, partial sequence; trnL-trnF intergenic spacer, complete sequence; and tRNA-Phe (trnF) gene, partial sequence; chloroplast	911	911	99% 0.0	90% FJ952035.1	828,18	63,9%
Select seq gb|FJ952060.1|	Clerodendrum aculeatum var. gracile tRNA-Leu (trnL) gene, partial sequence; trnL-trnF intergenic spacer, complete sequence; and tRNA-Phe (trnF) gene, partial sequence; chloroplast	909	909	99% 0.0	90% FJ952060.1	826,36	63,8%
Select seq gb|FJ952059.1|	Clerodendrum pittieri tRNA-Leu (trnL) gene, partial sequence; trnL-trnF intergenic spacer, complete sequence; and tRNA-Phe (trnF) gene, partial sequence; chloroplast	909	909	99% 0.0	90% FJ952059.1	826,36	63,8%
Select seq gb|FJ952055.1|	Clerodendrum quadriloculare tRNA-Leu (trnL) gene, partial sequence; trnL-trnF intergenic spacer, complete sequence; and tRNA-Phe (trnF) gene, partial sequence; chloroplast	905	905	99% 0.0	90% FJ952055.1	822,73	63,5%
Select seq gb|FJ952053.1|	Clerodendrum floribundum tRNA-Leu (trnL) gene, partial sequence; trnL-trnF intergenic spacer, complete sequence; and tRNA-Phe (trnF) gene, partial sequence; chloroplast	905	905	99% 0.0	90% FJ952053.1	822,73	63,5%
Select seq gb|FJ952049.1|	Clerodendrum chinense var. simplex tRNA-Leu (trnL) gene, partial sequence; trnL-trnF intergenic spacer, complete sequence; and tRNA-Phe (trnF) gene, partial sequence; chloroplast	905	905	99% 0.0	90% FJ952049.1	822,73	63,5%
Select seq gb|JN102144.1|	Clerodendrum trichotomum isolate P157 tRNA-Leu (trnL) gene, partial sequence; trnL-trnF intergenic spacer, complete sequence; and tRNA-Phe (trnF) gene, partial sequence; chloroplast	913	913	100% 0.0	90% JN102144.1	821,70	63,4%
Select seq emb|AJ505527.1|	Gmelina hystrix plastid trnL-trnF intergenic spacer, specimen voucher Cult., K-381-74-02999 (K)	865	865	95% 0.0	90% AJ505527.1	819,47	63,3%
Select seq gb|FJ952041.1|	Clerodendrum speciosissimum tRNA-Leu (trnL) gene, partial sequence; trnL-trnF intergenic spacer, complete sequence; and tRNA-Phe (trnF) gene, partial sequence; chloroplast	900	900	99% 0.0	90% FJ952041.1	818,18	63,2%
Select seq gb|FJ952040.1|	Clerodendrum buchananii tRNA-Leu (trnL) gene, partial sequence; trnL-trnF intergenic spacer, complete sequence; and tRNA-Phe (trnF) gene, partial sequence; chloroplast	900	900	99% 0.0	90% FJ952040.1	818,18	63,2%
Select seq gb|FJ952079.1|	Ajuga chamaepitys tRNA-Leu (trnL) gene, partial sequence; trnL-trnF intergenic spacer, complete sequence; and tRNA-Phe (trnF) gene, partial sequence; chloroplast	894	894	99% 0.0	90% FJ952079.1	812,73	62,7%
Select seq gb|FJ952027.1|	Clerodendrum splendens tRNA-Leu (trnL) gene, partial sequence; trnL-trnF intergenic spacer, complete sequence; and tRNA-Phe (trnF) gene, partial sequence; chloroplast	894	894	99% 0.0	90% FJ952027.1	812,73	62,7%
Select seq gb|FJ952076.1|	Kalaharia uncinata tRNA-Leu (trnL) gene, partial sequence; trnL-trnF intergenic spacer, complete sequence; and tRNA-Phe (trnF) gene, partial sequence; chloroplast	893	893	99% 0.0	90% FJ952076.1	811,82	62,7%
Select seq gb|EF153683.1|	Ajuga decumbens tRNA-Leu (trnL) and trnL-trnF intergenic spacer, partial sequence; chloroplast	902	902	100% 0.0	90% EF153683.1	811,80	62,7%
Select seq gb|FJ952033.1|	Clerodendrum bipindense tRNA-Leu (trnL) gene, partial sequence; trnL-trnF intergenic spacer, complete sequence; and tRNA-Phe (trnF) gene, partial sequence; chloroplast	889	889	99% 0.0	90% FJ952033.1	808,18	62,4%
Select seq gb|FJ952030.1|	Clerodendrum volubile tRNA-Leu (trnL) gene, partial sequence; trnL-trnF intergenic spacer, complete sequence; and tRNA-Phe (trnF) gene, partial sequence; chloroplast	889	889	99% 0.0	90% FJ952030.1	808,18	62,4%
Select seq dbj|AB894373.1|	Leucosceptrum japonicum chloroplast DNA, tRNA-Leu (trnL), trnL-trnF intergenic spacer, tRNA-Phe (trnF), partial and complete sequence, isolate: J1J20102-1	861	861	96% 0.0	90% AB894373.1	807,19	62,3%
Select seq gb|KF709391.1|	Ajuga reptans plastid, complete genome	896	896	100% 0.0	90% KF709391.1	806,40	62,2%
Select seq gb|FJ952026.1|	Clerodendrum polycephalum tRNA-Leu (trnL) gene, partial sequence; trnL-trnF intergenic spacer, complete sequence; and tRNA-Phe (trnF) gene, partial sequence; chloroplast	887	887	99% 0.0	90% FJ952026.1	806,36	62,2%
Select seq gb|FJ952031.1|	Clerodendrum johnstonii tRNA-Leu (trnL) gene, partial sequence; trnL-trnF intergenic spacer, complete sequence; and tRNA-Phe (trnF) gene, partial sequence; chloroplast	883	883	99% 0.0	90% FJ952031.1	802,73	62,0%
Select seq dbj|AB894374.1|	Leucosceptrum stellipilum chloroplast DNA, tRNA-Leu (trnL), trnL-trnF intergenic spacer, tRNA-Phe (trnF), partial and complete sequence, isolate: S1S20102-1	856	856	96% 0.0	90% AB894374.1	802,50	61,9%

Select seq gb|JN408674.1|	Teucridium parvifolium tRNA-Leu (trnL) gene, partial sequence; trnL-trnF intergenic spacer, complete sequence; and tRNA-Phe (trnF) gene, partial sequence; chloroplast	856	856	96% 0.0	90% JN408674.1	802,50	61,9%
Select seq emb|LM651038.1|	Clerodendrum colebrookianum genomic DNA containing partial trnL gene, trnL-trnF IGS and partial trnF gene	869	869	98% 0.0	90% LM651038.1	798,06	61,6%
Select seq gb|FJ952062.1|	Clerodendrum aggregatum tRNA-Leu (trnL) gene, partial sequence; trnL-trnF intergenic spacer, complete sequence; and tRNA-Phe (trnF) gene, partial sequence; chloroplast	874	874	99% 0.0	90% FJ952062.1	794,55	61,3%
Select seq gb|FJ952061.1|	Clerodendrum emirnense tRNA-Leu (trnL) gene, partial sequence; trnL-trnF intergenic spacer, complete sequence; and tRNA-Phe (trnF) gene, partial sequence; chloroplast	874	874	99% 0.0	90% FJ952061.1	794,55	61,3%
Select seq gb|FJ952080.1|	Teucrium pyrenaicum tRNA-Leu (trnL) gene, partial sequence; trnL-trnF intergenic spacer, complete sequence; and tRNA-Phe (trnF) gene, partial sequence; chloroplast	883	883	99% 0.0	89% FJ952080.1	793,81	61,3%
Select seq gb|FJ952057.1|	Clerodendrum linifolium tRNA-Leu (trnL) gene, partial sequence; trnL-trnF intergenic spacer, complete sequence; and tRNA-Phe (trnF) gene, partial sequence; chloroplast	883	883	99% 0.0	89% FJ952057.1	793,81	61,3%
Select seq gb|FJ952063.1|	Clerodendrum glabrum tRNA-Leu (trnL) gene, partial sequence; trnL-trnF intergenic spacer, complete sequence; and tRNA-Phe (trnF) gene, partial sequence; chloroplast	869	869	99% 0.0	90% FJ952063.1	790,00	61,0%
Select seq gb|FJ952068.1|	Tetraclea coulteri tRNA-Leu (trnL) gene, partial sequence; trnL-trnF intergenic spacer, complete sequence; and tRNA-Phe (trnF) gene, partial sequence; chloroplast	878	878	99% 0.0	89% FJ952068.1	789,31	60,9%
Select seq gb|JN408600.1|	Teucrium cubense tRNA-Leu (trnL) gene, partial sequence; trnL-trnF intergenic spacer, complete sequence; and tRNA-Phe (trnF) gene, partial sequence; chloroplast	869	869	98% 0.0	89% JN408600.1	789,19	60,9%
Select seq gb|JN408599.1|	Teucrium vesicarium tRNA-Leu (trnL) gene, partial sequence; trnL-trnF intergenic spacer, complete sequence; and tRNA-Phe (trnF) gene, partial sequence; chloroplast	869	869	98% 0.0	89% JN408599.1	789,19	60,9%
Select seq gb|JN408587.1|	Ajuga reptans tRNA-Leu (trnL) gene, partial sequence; trnL-trnF intergenic spacer, complete sequence; and tRNA-Phe (trnF) gene, partial sequence; chloroplast	863	863	98% 0.0	89% JN408587.1	783,74	60,5%
Select seq gb|HQ646990.1|	Teucrium flavum subsp. glaucum isolate TFG64 tRNA-Leu (trnL) gene and trnL-trnF intergenic spacer, complete sequence; and tRNA-Phe (trnF) gene, partial sequence; chloroplast	845	845	96% 0.0	89% HQ646990.1	783,39	60,5%
Select seq gb|HQ646981.1|	Teucrium flavum subsp. glaucum isolate TFG54 tRNA-Leu (trnL) gene and trnL-trnF intergenic spacer, complete sequence; and tRNA-Phe (trnF) gene, partial sequence; chloroplast	845	845	96% 0.0	89% HQ646981.1	783,39	60,5%
Select seq emb|LN823952.1|	Clerodendrum fragrans chloroplast DNA containing partial trnL gene, trnL-trnF IGS and partial trnF gene	870	870	99% 0.0	89% LN823952.1	782,12	60,4%
Select seq gb|JN408595.1|	Teucrium abutiloides tRNA-Leu (trnL) gene, partial sequence; trnL-trnF intergenic spacer, complete sequence; and tRNA-Phe (trnF) gene, partial sequence; chloroplast	843	843	96% 0.0	89% JN408595.1	781,53	60,3%
Select seq gb|JN408592.1|	Teucrium argutum var. incisum tRNA-Leu (trnL) gene, partial sequence; trnL-trnF intergenic spacer, complete sequence; and tRNA-Phe (trnF) gene, partial sequence; chloroplast	843	843	96% 0.0	89% JN408592.1	781,53	60,3%
Select seq gb|FJ952054.1|	Clerodendrum minahassae tRNA-Leu (trnL) gene, partial sequence; trnL-trnF intergenic spacer, complete sequence; and tRNA-Phe (trnF) gene, partial sequence; chloroplast	867	867	99% 0.0	89% FJ952054.1	779,42	60,2%
Select seq gb|FJ952032.1|	Clerodendrum umbellatum tRNA-Leu (trnL) gene, partial sequence; trnL-trnF intergenic spacer, complete sequence; and tRNA-Phe (trnF) gene, partial sequence; chloroplast	867	867	99% 0.0	89% FJ952032.1	779,42	60,2%
Select seq gb|HQ412927.1|	Prostanthera calycina tRNA-Leu (trnL) gene and trnL-trnF intergenic spacer, partial sequence; chloroplast	839	839	96% 0.0	89% HQ412927.1	777,82	60,0%
Select seq gb|JF694866.1|	Teucrium scorodonia isolate TSC8 tRNA-Leu (trnL) gene and trnL-trnF intergenic spacer, complete sequence; and tRNA-Phe (trnF) gene, partial sequence; plastid	839	839	96% 0.0	89% JF694866.1	777,82	60,0%
Select seq gb|JF694861.1|	Teucrium scorodonia isolate TSC3 tRNA-Leu (trnL) gene and trnL-trnF intergenic spacer, complete sequence; and tRNA-Phe (trnF) gene, partial sequence; plastid	839	839	96% 0.0	89% JF694861.1	777,82	60,0%
Select seq gb|JF694859.1|	Teucrium scorodonia isolate TSC1 tRNA-Leu (trnL) gene and trnL-trnF intergenic spacer, complete sequence; and tRNA-Phe (trnF) gene, partial sequence; plastid	839	839	96% 0.0	89% JF694859.1	777,82	60,0%
Select seq gb|HQ646991.1|	Teucrium flavum subsp. flavum isolate TFF56 tRNA-Leu (trnL) gene and trnL-trnF intergenic spacer, complete sequence; and tRNA-Phe (trnF) gene, partial sequence; chloroplast	839	839	96% 0.0	89% HQ646991.1	777,82	60,0%
Select seq gb|EU827136.1|	Paraphlomis hispida voucher Fang091066 (KUN) tRNA-Leu (trnL) gene, partial sequence; trnL-trnF intergenic spacer, complete sequence; and tRNA-Phe (trnF) gene, partial sequence; chloroplast	830	830	95% 0.0	89% EU827136.1	777,58	60,0%
Select seq gb|JN408597.1|	Teucrium alpestre tRNA-Leu (trnL) gene, partial sequence; trnL-trnF intergenic spacer, complete sequence; and tRNA-Phe (trnF) gene, partial sequence; chloroplast	846	846	97% 0.0	89% JN408597.1	776,23	59,9%
Select seq gb|JQ044780.1|	Teucrium polium subsp. polium isolate TP12 tRNA-Leu (trnL) gene, partial sequence; trnL-trnF intergenic spacer, complete sequence; and tRNA-Phe (trnF) gene, partial sequence; chloroplast	835	835	96% 0.0	89% JQ044780.1	774,11	59,8%
Select seq gb|JQ044779.1|	Teucrium polium subsp. polium isolate TP11 tRNA-Leu (trnL) gene, partial sequence; trnL-trnF intergenic spacer, complete sequence; and tRNA-Phe (trnF) gene, partial sequence; chloroplast	835	835	96% 0.0	89% JQ044779.1	774,11	59,8%

Select seq gb|JQ044778.1|	Teucrium polium subsp. polium isolate TP10 tRNA-Leu (trnL) gene, partial sequence; trnL-trnF intergenic spacer, complete sequence; and tRNA-Phe (trnF) gene, partial sequence; chloroplast	835	835	96% 0.0	89%	JQ044778.1	774,11	59,8%
Select seq gb|JF694864.1|	Teucrium scorodonia isolate TSC6 tRNA-Leu (trnL) gene and trnL-trnF intergenic spacer, complete sequence; and tRNA-Phe (trnF) gene, partial sequence; plastid	833	833	96% 0.0	89%	JF694864.1	772,26	59,6%
Select seq gb|FJ952078.1|	Faradaya splendida tRNA-Leu (trnL) gene, partial sequence; trnL-trnF intergenic spacer, complete sequence; and tRNA-Phe (trnF) gene, partial sequence; chloroplast	856	856	99% 0.0	89%	FJ952078.1	769,54	59,4%
Select seq gb|JQ044781.1|	Teucrium polium subsp. polium isolate TP19 tRNA-Leu (trnL) gene, partial sequence; trnL-trnF intergenic spacer, complete sequence; and tRNA-Phe (trnF) gene, partial sequence; chloroplast	830	830	96% 0.0	89%	JQ044781.1	769,48	59,4%
Select seq gb|JQ044776.1|	Teucrium polium subsp. polium isolate TP4 tRNA-Leu (trnL) gene, partial sequence; trnL-trnF intergenic spacer, complete sequence; and tRNA-Phe (trnF) gene, partial sequence; chloroplast	830	830	96% 0.0	89%	JQ044776.1	769,48	59,4%
Select seq gb|JQ044775.1|	Teucrium polium subsp. polium isolate TP3 tRNA-Leu (trnL) gene, partial sequence; trnL-trnF intergenic spacer, complete sequence; and tRNA-Phe (trnF) gene, partial sequence; chloroplast	830	830	96% 0.0	89%	JQ044775.1	769,48	59,4%
Select seq gb|JQ044774.1|	Teucrium polium subsp. polium isolate TP20 tRNA-Leu (trnL) gene, partial sequence; trnL-trnF intergenic spacer, complete sequence; and tRNA-Phe (trnF) gene, partial sequence; chloroplast	830	830	96% 0.0	89%	JQ044774.1	769,48	59,4%
Select seq gb|KT750009.1|	Scutellaria insignis chloroplast, complete genome	859	859	100% 0.0	89%	KT750009.1	764,51	59,0%
Select seq gb|FJ952077.1|	Oxera pulchella tRNA-Leu (trnL) gene, partial sequence; trnL-trnF intergenic spacer, complete sequence; and tRNA-Phe (trnF) gene, partial sequence; chloroplast	850	850	99% 0.0	89%	FJ952077.1	764,14	59,0%
Select seq gb|FJ952028.1|	Clerodendrum schweinfurthii tRNA-Leu (trnL) gene, partial sequence; trnL-trnF intergenic spacer, complete sequence; and tRNA-Phe (trnF) gene, partial sequence; chloroplast	848	848	99% 0.0	89%	FJ952028.1	762,34	58,8%
Select seq gb|AY570453.1|	Collinsonia canadensis voucher JBW 958 tRNA-Leu and trnL-trnF intergenic spacer, partial sequence; chloroplast	845	845	99% 0.0	89%	AY570453.1	759,65	58,6%
Select seq gb|FJ952045.1|	Clerodendrum bungei tRNA-Leu (trnL) gene, partial sequence; trnL-trnF intergenic spacer, complete sequence; and tRNA-Phe (trnF) gene, partial sequence; chloroplast	841	841	99% 0.0	89%	FJ952045.1	756,05	58,4%
Select seq gb|FJ952029.1|	Clerodendrum thomsoniae tRNA-Leu (trnL) gene, partial sequence; trnL-trnF intergenic spacer, complete sequence; and tRNA-Phe (trnF) gene, partial sequence; chloroplast	841	841	99% 0.0	89%	FJ952029.1	756,05	58,4%
Select seq gb|FJ952042.1|	Clerodendrum sp. Ramadhanil et al. 461 tRNA-Leu (trnL) gene, partial sequence; trnL-trnF intergenic spacer, complete sequence; and tRNA-Phe (trnF) gene, partial sequence; chloroplast	837	837	99% 0.0	89%	FJ952042.1	752,45	58,1%
Select seq gb|FJ952046.1|	Clerodendrum lindleyi tRNA-Leu (trnL) gene, partial sequence; trnL-trnF intergenic spacer, complete sequence; and tRNA-Phe (trnF) gene, partial sequence; chloroplast	835	835	99% 0.0	89%	FJ952046.1	750,66	57,9%
Select seq gb|DQ667439.1|	Perilla frutescens isolate x089 tRNA-Leu (trnL) gene and trnL-trnF intergenic spacer, partial sequence; chloroplast	833	833	99% 0.0	89%	DQ667439.1	748,86	57,8%
Select seq dbj|AB685327.1|	Perilla frutescens var. acuta chloroplast DNA, contains tRNA-Leu(trnL) gene (intron and 3' exon), trnL-trnF intergenic spacer and tRNA-Phe(trnF) gene	832	832	99% 0.0	89%	AB685327.1	747,96	57,7%
Select seq gb|FJ952043.1|	Clerodendrum japonicum tRNA-Leu (trnL) gene, partial sequence; trnL-trnF intergenic spacer, complete sequence; and tRNA-Phe (trnF) gene, partial sequence; chloroplast	832	832	99% 0.0	89%	FJ952043.1	747,96	57,7%
Select seq emb|LM651035.1|	Clerodendrum speciosum genomic DNA containing partial trnL gene, trnL-trnF IGS and partial trnF gene	830	830	99% 0.0	89%	LM651035.1	746,16	57,6%

Select for downloading or viewing reports	Kh034_trnL Description	Max score	Total score	Query cover	E value	Ident	Accession	(Ident/Cover)* Max score	Deviation from top hit
Select seq emb|LN515485.1|	Fraxinus angustifolia plastid DNA containing tRNA-Leu (trnL) intron and 3' exon, and trnL-F IGS, specimen voucher Besnard 41-2014	1288	1288	99%	0.0	100%	LN515485.1	1301,01	100,0%
Select seq gb|AF231830.1|	Fraxinus excelsior tRNA-Leu (trnL) gene, partial intron and 3' exon, and trnL-trnF spacer region; chloroplast gene for chloroplast product	1247	1247	96%	0.0	100%	AF231830.1	1298,96	99,8%
Select seq gb|JN591011.1|	Fraxinus excelsior voucher Li 3613 tRNA-Leu (trnL) gene and trnL-trnF intergenic spacer, partial sequence; plastid	1179	1179	91%	0.0	99%	JN591011.1	1282,65	98,6%
Select seq gb|EU281162.1|	Fraxinus sieboldiana tRNA-Leu (trnL) gene and trnL-trnF intergenic spacer, partial sequence; chloroplast	1242	1242	96%	0.0	99%	EU281162.1	1280,81	98,4%
Select seq gb|AF231827.1|AF231827	Fraxinus chinensis var. rhynchophylla tRNA-Leu (trnL) gene, partial intron and 3' exon, and trnL-trnF spacer region; chloroplast gene for chloroplast product	1242	1242	96%	0.0	99%	AF231827.1	1280,81	98,4%
Select seq gb|AY911650.1|	Fraxinus excelsior isolate frax9 tRNA-Leu gene, partial sequence; trnL-trnF intergenic spacer, complete sequence; and tRNA-Phe gene, partial sequence; chloroplast	1236	1236	96%	0.0	99%	AY911650.1	1274,63	98,0%
Select seq gb|AF231832.1|AF231832	Fraxinus ornus tRNA-Leu (trnL) gene, partial intron and 3' exon, and trnL-trnF spacer region; chloroplast gene for chloroplast product	1236	1236	96%	0.0	99%	AF231832.1	1274,63	98,0%
Select seq gb|EU284160.1|	Fraxinus raibocarpa tRNA-Leu (trnL) gene and trnL-trnF intergenic spacer, partial sequence; chloroplast	1166	1166	91%	0.0	99%	EU284160.1	1268,51	97,5%
Select seq gb|AF231831.1|AF231831	Fraxinus greggii tRNA-Leu (trnL) gene, partial intron and 3' exon, and trnL-trnF spacer region; chloroplast gene for chloroplast product	1230	1230	96%	0.0	99%	AF231831.1	1268,44	97,5%
Select seq gb|AF231825.1|AF231825	Fraxinus americana tRNA-Leu (trnL) gene, partial intron and 3' exon, and trnL-trnF spacer region; chloroplast gene for chloroplast product	1225	1225	96%	0.0	99%	AF231825.1	1263,28	97,1%
Select seq gb|EU284157.1|	Fraxinus chiisanensis tRNA-Leu (trnL) gene and trnL-trnF intergenic spacer, partial sequence; chloroplast	1219	1219	96%	0.0	99%	EU284157.1	1257,09	96,6%
Select seq gb|EU284161.1|	Fraxinus spaethiana tRNA-Leu (trnL) gene and trnL-trnF intergenic spacer, partial sequence; chloroplast	1179	1179	93%	0.0	99%	EU284161.1	1255,06	96,5%
Select seq emb|LN515489.1|	Hesperelaea palmeri complete chloroplast genome, specimen voucher E. Palmer 81 (MO)	1254	1254	99%	0.0	99%	LN515489.1	1254,00	96,4%
Select seq emb|LN515478.1|	Osmanthus decorus plastid DNA containing tRNA-Leu (trnL) intron and 3' exon, and trnL-F IGS, specimen voucher Merello 2324 (MO)	1254	1254	99%	0.0	99%	LN515478.1	1254,00	96,4%
Select seq emb|LN515476.1|	Olea paniculata plastid DNA containing tRNA-Leu (trnL) intron and 3' exon, and trnL-F IGS, specimen voucher Lambrides 1 (MPU)	1254	1254	99%	0.0	99%	LN515476.1	1254,00	96,4%
Select seq emb|FN998901.1|	Olea woodiana subsp. woodiana complete chloroplast genome, specimen voucher A. Costa 02 (MPU)	1254	1254	99%	0.0	99%	FN998901.1	1254,00	96,4%
Select seq gb|AY911649.1|	Fraxinus excelsior isolate frax5 tRNA-Leu gene, partial sequence; trnL-trnF intergenic spacer, complete sequence; and tRNA-Phe gene, partial sequence; chloroplast	1190	1190	94%	0.0	99%	AY911649.1	1253,30	96,3%
Select seq gb|GU450320.1|	Osmanthus hainanensis tRNA-Leu (trnL) gene and trnL-trnF intergenic spacer, partial sequence; chloroplast	1214	1214	96%	0.0	99%	GU450320.1	1251,94	96,2%
Select seq gb|AF231873.1|	Phillyrea angustifolia tRNA-Leu (trnL) gene, partial intron and 3' exon, and trnL-trnF spacer region; chloroplast gene for chloroplast product	1214	1214	96%	0.0	99%	AF231873.1	1251,94	96,2%
Select seq gb|AF231875.1|AF231875	Phillyrea media tRNA-Leu (trnL) gene, partial intron and 3' exon, and trnL-trnF spacer region; chloroplast gene for chloroplast product	1214	1214	96%	0.0	99%	AF231875.1	1251,94	96,2%
Select seq gb|AY911648.1|	Fraxinus excelsior isolate frax100 tRNA-Leu gene, partial sequence; trnL-trnF intergenic spacer, complete sequence; and tRNA-Phe gene, partial sequence; chloroplast	1188	1188	94%	0.0	99%	AY911648.1	1251,19	96,2%
Select seq emb|LN515482.1|	Picconia azorica plastid DNA containing tRNA-Leu (trnL) intron and 3' exon, and trnL-F IGS, specimen voucher Schaefer 2008-323 (BM)	1249	1249	99%	0.0	99%	LN515482.1	1249,00	96,0%
Select seq emb|LN515481.1|	Osmanthus fragrans plastid DNA containing tRNA-Leu (trnL) intron and 3' exon, and trnL-F IGS, specimen voucher Hong-Wa SN3 (MO)	1249	1249	99%	0.0	99%	LN515481.1	1249,00	96,0%
Select seq emb|LN515465.1|	Chionanthus virginicus plastid DNA containing tRNA-Leu (trnL) intron and 3' exon, and trnL-F IGS, bio-material Kew 1976-292	1249	1249	99%	0.0	99%	LN515465.1	1249,00	96,0%
Select seq emb|LN515461.1|	Chionanthus panamensis plastid DNA containing tRNA-Leu (trnL) intron and 3' exon, and trnL-F IGS, specimen voucher Martinez & Ramos 26308 (K)	1249	1249	99%	0.0	99%	LN515461.1	1249,00	96,0%
Select seq gb|AF231872.1|AF231872	Osmanthus rigidus tRNA-Leu (trnL) gene, partial intron and 3' exon, and trnL-trnF spacer region; chloroplast gene for chloroplast product	1171	1171	93%	0.0	99%	AF231872.1	1246,55	95,8%
Select seq emb|LN515475.1|	Olea capensis subsp. capensis plastid DNA containing tRNA-Leu (trnL) intron and 3' exon, and trnL-F IGS, specimen voucher Costa 3 (MPU)	1208	1208	96%	0.0	99%	LN515475.1	1245,75	95,8%
Select seq gb|GU450324.1|	Osmanthus delavayi tRNA-Leu (trnL) gene and trnL-trnF intergenic spacer, partial sequence; chloroplast	1208	1208	96%	0.0	99%	GU450324.1	1245,75	95,8%
Select seq gb|GU450318.1|	Osmanthus yunnanensis tRNA-Leu (trnL) gene and trnL-trnF intergenic spacer, partial sequence; chloroplast	1208	1208	96%	0.0	99%	GU450318.1	1245,75	95,8%
Select seq gb|AF231869.1|	Osmanthus fragrans tRNA-Leu (trnL) gene, partial intron and 3' exon, and trnL-trnF spacer region; chloroplast gene for chloroplast product	1208	1208	96%	0.0	99%	AF231869.1	1245,75	95,8%
Select seq gb|EU659987.1|	Osmanthus henryi tRNA-Leu (trnL) gene and trnL-trnF intergenic spacer, partial sequence; chloroplast	1208	1208	96%	0.0	99%	EU659987.1	1245,75	95,8%
Select seq gb|EU659986.1|	Osmanthus x fortunei tRNA-Leu (trnL) gene and trnL-trnF intergenic spacer, partial sequence; chloroplast	1208	1208	96%	0.0	99%	EU659986.1	1245,75	95,8%
Select seq gb|AF231876.1|AF231876	Picconia excelsa tRNA-Leu (trnL) gene, partial intron and 3' exon, and trnL-trnF spacer region; chloroplast gene for chloroplast product	1208	1208	96%	0.0	99%	AF231876.1	1245,75	95,8%
Select seq gb|AF231874.1|AF231874	Phillyrea latifolia tRNA-Leu (trnL) gene, partial intron and 3' exon, and trnL-trnF spacer region; chloroplast gene for chloroplast product	1208	1208	96%	0.0	99%	AF231874.1	1245,75	95,8%
Select seq gb|AY911651.1|	Fraxinus excelsior isolate frax42 tRNA-Leu gene, partial sequence; trnL-trnF intergenic spacer, complete sequence; and tRNA-Phe gene, partial sequence; chloroplast	1181	1181	94%	0.0	99%	AY911651.1	1243,82	95,6%
Select seq emb|LN515473.1|	Notelaea microcarpa plastid DNA containing tRNA-Leu (trnL) intron and 3' exon, and trnL-F IGS, specimen voucher Streiman 731 (K)	1243	1243	99%	0.0	99%	LN515473.1	1243,00	95,5%
Select seq emb|LN515472.1|	Nestegis sandwicensis plastid DNA containing tRNA-Leu (trnL) intron and 3' exon, and trnL-F IGS, specimen voucher Flynn 6329 (MPU)	1243	1243	99%	0.0	99%	LN515472.1	1243,00	95,5%
Select seq gb|EU284159.1|	Fraxinus pennsylvanica tRNA-Leu (trnL) gene and trnL-trnF intergenic spacer, partial sequence; chloroplast	1205	1205	96%	0.0	99%	EU284159.1	1242,66	95,5%
Select seq gb|AF231870.1|AF231870	Osmanthus heterophyllus tRNA-Leu (trnL) gene, partial intron and 3' exon, and trnL-trnF spacer region; chloroplast gene for chloroplast product	1166	1166	93%	0.0	99%	AF231870.1	1241,23	95,4%
Select seq gb|AF231867.1|AF231867	Olea paniculata tRNA-Leu (trnL) gene, partial intron and 3' exon, and trnL-trnF spacer region; chloroplast gene for chloroplast product	1203	1203	96%	0.0	99%	AF231867.1	1240,59	95,4%
Select seq gb|AF231859.1|AF231859	Notelaea longifolia tRNA-Leu (trnL) gene, partial intron and 3' exon, and trnL-trnF spacer region; chloroplast gene for chloroplast product	1203	1203	96%	0.0	99%	AF231859.1	1240,59	95,4%
Select seq emb|LN515479.1|	Cartrema americana plastid DNA containing tRNA-Leu (trnL) intron and 3' exon, and trnL-F IGS, specimen voucher Hong-Wa OA (MO)	1238	1238	99%	0.0	99%	LN515479.1	1238,00	95,2%
Select seq emb|LN515470.1|	Forestiera segregata var. segregata plastid DNA containing tRNA-Leu (trnL) intron and 3' exon, and trnL-F IGS, specimen voucher Wagner 1330 (P)	1238	1238	99%	0.0	99%	LN515470.1	1238,00	95,2%
Select seq emb|LN515467.1|	Forestiera ligustrina plastid DNA containing tRNA-Leu (trnL) intron and 3' exon, and trnL-F IGS	1238	1238	99%	0.0	99%	LN515467.1	1238,00	95,2%

Select seq gb|AF231871.1|AF231871	Osmanthus insularis tRNA-Leu (trnL) gene, partial intron and 3' exon, and trnL-trnF spacer region; chloroplast gene for chloroplast product	1160	1160	93%	0.0	99%	AF231871.1	1234,84	94,9%
Select seq gb|HQ117892.1|	Chionanthus ramiflorus trnL-trnF intergenic spacer, partial sequence; chloroplast	1197	1197	96%	0.0	99%	HQ117892.1	1234,41	94,9%
Select seq gb|GU450317.1|	Osmanthus matsumuranus tRNA-Leu (trnL) gene and trnL-trnF intergenic spacer, partial sequence; chloroplast	1197	1197	96%	0.0	99%	GU450317.1	1234,41	94,9%
Select seq gb|AF231868.1|AF231868	Osmanthus americanus tRNA-Leu (trnL) gene, partial intron and 3' exon, and trnL-trnF spacer region; chloroplast gene for chloroplast product	1197	1197	96%	0.0	99%	AF231868.1	1234,41	94,9%
Select seq gb|AF231861.1|AF231861	Notelaea punctata tRNA-Leu (trnL) gene, partial intron and 3' exon, and trnL-trnF spacer region; chloroplast gene for chloroplast product	1197	1197	96%	0.0	99%	AF231861.1	1234,41	94,9%
Select seq gb|AF231860.1|AF231860	Notelaea microcarpa tRNA-Leu (trnL) gene, partial intron and 3' exon, and trnL-trnF spacer region; chloroplast gene for chloroplast product	1197	1197	96%	0.0	99%	AF231860.1	1234,41	94,9%
Select seq gb|AF231835.1|AF231835	Haenianthus incrassatus tRNA-Leu (trnL) gene, partial intron and 3' exon, and trnL-trnF spacer region; chloroplast gene for chloroplast product	1197	1197	96%	0.0	99%	AF231835.1	1234,41	94,9%
Select seq gb|AF231822.1|AF231822	Forestiera segregata var. pinetorum tRNA-Leu (trnL) gene, partial intron and 3' exon, and trnL-trnF spacer region; chloroplast gene for chloroplast product	1197	1197	96%	0.0	99%	AF231822.1	1234,41	94,9%
Select seq gb|AF231812.1|AF231812	Chionanthus virginicus tRNA-Leu (trnL) gene, partial intron and 3' exon, and trnL-trnF spacer region; chloroplast gene for chloroplast product	1197	1197	96%	0.0	99%	AF231812.1	1234,41	94,9%
Select seq emb|LN515466.1|	Chionanthus sp. Munzinger 245 plastid DNA containing tRNA-Leu (trnL) intron and 3' exon, and trnL-F IGS, specimen voucher Munzinger 245 (P)	1232	1232	99%	0.0	99%	LN515466.1	1232,00	94,7%
Select seq emb|LN515463.1|	Chionanthus ramiflorus plastid DNA containing tRNA-Leu (trnL) intron and 3' exon, and trnL-F IGS, specimen voucher Flynn 6332 (MPU)	1232	1232	99%	0.0	99%	LN515463.1	1232,00	94,7%
Select seq gb|AF231856.1|AF231856	Nestegis lanceolata tRNA-Leu (trnL) gene, partial intron and 3' exon, and trnL-trnF spacer region; chloroplast gene for chloroplast product	1192	1192	96%	0.0	99%	AF231856.1	1229,25	94,5%
Select seq gb|AF231821.1|AF231821	Forestiera neo-mexicana tRNA-Leu (trnL) gene, partial intron and 3' exon, and trnL-trnF spacer region; chloroplast gene for chloroplast product	1192	1192	96%	0.0	99%	AF231821.1	1229,25	94,5%
Select seq gb|AF231819.1|AF231819	Forestiera acuminata tRNA-Leu (trnL) gene, partial intron and 3' exon, and trnL-trnF spacer region; chloroplast gene for chloroplast product	1192	1192	96%	0.0	99%	AF231819.1	1229,25	94,5%
Select seq emb|HF558645.1|	Olea europaea subsp. europaea complete chloroplast genome, haplotype E1.4, isolate Stavrovouni 11	1232	1232	99%	0.0	98%	HF558645.1	1219,56	93,7%
Select seq gb|GU228899.2|	Olea europaea cultivar Bianchera chloroplast, complete genome	1232	1232	99%	0.0	98%	GU228899.2	1219,56	93,7%
Select seq emb|FN998900.2|	Olea europaea subsp. maroccana complete chloroplast genome, isolate Immouzzer S1	1232	1232	99%	0.0	98%	FN998900.2	1219,56	93,7%
Select seq emb|FN997651.1|	Olea europaea subsp. europaea complete chloroplast genome, isolate Gue de Constantine 20	1232	1232	99%	0.0	98%	FN997651.1	1219,56	93,7%
Select seq emb|FN996972.1|	Olea europaea subsp. europaea complete chloroplast genome, cultivar Manzanilla	1232	1232	99%	0.0	98%	FN996972.1	1219,56	93,7%
Select seq emb|FN997650.2|	Olea europaea subsp. europaea complete chloroplast genome, isolate Haut Atlas 1	1232	1232	99%	0.0	98%	FN997650.2	1219,56	93,7%
Select seq gb|GU931818.1|	Olea europaea cultivar Frantoio chloroplast, complete genome	1232	1232	99%	0.0	98%	GU931818.1	1219,56	93,7%
Select seq gb|DQ423790.1|	Zelcova sicula tRNA-Leu (trnL) gene, partial sequence, and trnL-trnF intergenic spacer; chloroplast gene for chloroplast product	1166	1166	94%	0.0	98%	DQ423790.1	1215,62	93,4%
Select seq emb|LN515486.1|	Comoranthus minor plastid DNA containing tRNA-Leu (trnL) intron and 3' exon, and trnL-F IGS, specimen voucher Randrianaivo 1757 (MO)	1227	1227	99%	0.0	98%	LN515486.1	1214,61	93,4%
Select seq emb|FN996943.2|	Olea europaea subsp. cuspidata complete chloroplast genome, isolate Almihwit 5.1	1227	1227	99%	0.0	98%	FN996943.2	1214,61	93,4%
Select seq emb|FN650747.2|	Olea europaea subsp. cuspidata complete chloroplast genome, isolate Maui 1	1227	1227	99%	0.0	98%	FN650747.2	1214,61	93,4%
Select seq emb|FN996944.1|	Olea europaea subsp. cuspidata complete chloroplast genome, isolate Guanghzou 1	1227	1227	99%	0.0	98%	FN996944.1	1214,61	93,4%
Select seq emb|AJ430922.1|	Olea europaea chloroplast partial tRNA-Leu gene for transfer RNA-Leu and partial tRNA-Phe gene for transfer RNA-Phe	1227	1227	99%	0.0	98%	AJ430922.1	1214,61	93,4%
Select seq gb|AF231828.1|AF231828	Fraxinus cuspidata var. macropetala tRNA-Leu (trnL) gene, partial intron and 3' exon, and trnL-trnF spacer region; chloroplast gene for chloroplast product	1188	1188	96%	0.0	98%	AF231828.1	1212,75	93,2%
Select seq emb|LN515471.1|	Haenianthus salicifolius plastid DNA containing tRNA-Leu (trnL) intron and 3' exon, and trnL-F IGS, specimen voucher Terranova 15 (MO)	1225	1225	99%	0.0	98%	LN515471.1	1212,63	93,2%
Select seq gb|AF231865.1|	Olea capensis tRNA-Leu (trnL) gene, partial intron and 3' exon, and trnL-trnF spacer region; chloroplast gene for chloroplast product	1186	1186	96%	0.0	98%	AF231865.1	1210,71	93,1%
Select seq gb|AF231854.1|AF231854	Nestegis apetala tRNA-Leu (trnL) gene, partial intron and 3' exon, and trnL-trnF spacer region; chloroplast gene for chloroplast product	1186	1186	96%	0.0	98%	AF231854.1	1210,71	93,1%
Select seq gb|AF231814.1|AF231814	Comoranthus minor tRNA-Leu (trnL) gene, partial intron and 3' exon, and trnL-trnF spacer region; chloroplast gene for chloroplast product	1186	1186	96%	0.0	98%	AF231814.1	1210,71	93,1%
Select seq gb|AF231813.1|AF231813	Comoranthus madagascariensis tRNA-Leu (trnL) gene, partial intron and 3' exon, and trnL-trnF spacer region; chloroplast gene for chloroplast product	1186	1186	96%	0.0	98%	AF231813.1	1210,71	93,1%
Select seq gb|AF231811.1|AF231811	Chionanthus retusus tRNA-Leu (trnL) gene, partial intron and 3' exon, and trnL-trnF spacer region; chloroplast gene for chloroplast product	1186	1186	96%	0.0	98%	AF231811.1	1210,71	93,1%
Select seq emb|LN515464.1|	Chionanthus retusus plastid DNA containing tRNA-Leu (trnL) intron and 3' exon, and trnL-F IGS, bio-material Kew 13008	1221	1221	99%	0.0	98%	LN515464.1	1208,67	92,9%
Select seq gb|EU281165.1|	Chionanthus retusus tRNA-Leu (trnL) gene and trnL-trnF intergenic spacer, partial sequence; chloroplast	1221	1221	99%	0.0	98%	EU281165.1	1208,67	92,9%
Select seq gb|AF231836.1|AF231836	Haenianthus salicifolius var. obovatus tRNA-Leu (trnL) gene, partial intron and 3' exon, and trnL-trnF spacer region; chloroplast gene for chloroplast product	1184	1184	96%	0.0	98%	AF231836.1	1208,67	92,9%
Select seq gb|AY911646.1|	Fraxinus excelsior isolate frax8 tRNA-Leu gene, partial sequence; trnL-trnF intergenic spacer, complete sequence; and tRNA-Phe gene, partial sequence; chloroplast	1171	1171	95%	0.0	98%	AY911646.1	1207,98	92,8%
Select seq gb|JN591027.1|	Syringa vulgaris voucher Li 2457 tRNA-Leu (trnL) gene and trnL-trnF intergenic spacer, partial sequence; plastid	1170	1170	95%	0.0	98%	JN591027.1	1206,95	92,8%
Select seq gb|AF231882.1|AF231882	Syringa vulgaris tRNA-Leu (trnL) gene, partial intron and 3' exon, and trnL-trnF spacer region; chloroplast gene for chloroplast product	1170	1170	95%	0.0	98%	AF231882.1	1206,95	92,8%
Select seq gb|HQ117893.1|	Olea europaea subsp. cuspidata trnL-trnF intergenic spacer, partial sequence; chloroplast	1181	1181	96%	0.0	98%	HQ117893.1	1205,60	92,7%
Select seq gb|GU450315.1|	Osmanthus marginatus tRNA-Leu (trnL) gene and trnL-trnF intergenic spacer, partial sequence; chloroplast	1179	1179	96%	0.0	98%	GU450315.1	1203,56	92,5%

Select seq gb|AF231878.1|	Schrebera alata tRNA-Leu (trnL) gene, partial intron and 3' exon, and trnL-trnF spacer region; chloroplast gene for chloroplast product	1177	1177	96%	0.0	98%	AF231878.1	1201,52	92,4%
Select seq emb|LN515487.1|	Schrebera alata plastid DNA containing tRNA-Leu (trnL) intron and 3' exon, and trnL-F IGS	1210	1210	99%	0.0	98%	LN515487.1	1197,78	92,1%
Select seq gb|GU450325.1|	Osmanthus didymopetalus tRNA-Leu (trnL) gene and trnL-trnF intergenic spacer, partial sequence; chloroplast	1170	1170	96%	0.0	98%	GU450325.1	1194,38	91,8%
Select seq gb|EU281163.1|	Syringa dilatata tRNA-Leu (trnL) gene and trnL-trnF intergenic spacer, partial sequence; chloroplast	1190	1190	98%	0.0	98%	EU281163.1	1190,00	91,5%
Select seq gb|AF231883.1|AF231883	Syringa yunnanensis tRNA-Leu (trnL) gene, partial intron and 3' exon, and trnL-trnF spacer region; chloroplast gene for chloroplast product	1164	1164	96%	0.0	98%	AF231883.1	1188,25	91,3%
Select seq emb|LN515460.1|	Chionanthus filiformis plastid DNA containing tRNA-Leu (trnL) intron and 3' exon, and trnL-F IGS, specimen voucher Frey 52705	1188	1291	99%	0.0	99%	LN515460.1	1188,00	91,3%
Select seq emb|LN515477.1|	Olea tsoongii plastid DNA containing tRNA-Leu (trnL) intron and 3' exon, and trnL-F IGS, specimen voucher Besnard sn (MPU)	1199	1199	99%	0.0	98%	LN515477.1	1186,89	91,2%
Select seq gb|JN591013.1|	Syringa emodi voucher Li 2465 tRNA-Leu (trnL) gene and trnL-trnF intergenic spacer, partial sequence; plastid	1160	1160	96%	0.0	98%	JN591013.1	1184,17	91,0%
Select seq gb|AF231833.1|AF231833	Fraxinus quadrangulata tRNA-Leu (trnL) gene, partial intron and 3' exon, and trnL-trnF spacer region; chloroplast gene for chloroplast product	1160	1160	96%	0.0	98%	AF231833.1	1184,17	91,0%
Select seq gb|JN102159.1|	Ligustrum obtusifolium isolate P286 tRNA-Leu (trnL) gene, partial sequence; trnL-trnF intergenic spacer, complete sequence; and tRNA-Phe (trnF) gene, partial sequence; chloroplast	1179	1179	99%	0.0	97%	JN102159.1	1155,18	88,8%
Select seq emb|LN515462.1|	Chionanthus quadristamineus plastid DNA containing tRNA-Leu (trnL) intron and 3' exon, and trnL-F IGS, specimen voucher Papadopoulos 366	1173	1173	99%	0.0	97%	LN515462.1	1149,30	88,3%
Select seq gb|DQ673256.1|	Forsythia europaea psaA-psbB fragment, chloroplast	1173	1173	99%	0.0	97%	DQ673256.1	1149,30	88,3%
Select seq emb|LN515474.1|	Noronhia emarginata plastid DNA containing tRNA-Leu (trnL) intron and 3' exon, and trnL-F IGS, specimen voucher Flynn 6331 (MPU)	1168	1168	99%	0.0	97%	LN515474.1	1144,40	

Select for downloading or viewing reports	Kh036_ITS Description	Max score	Total score	Query cover	E value	Ident	Accession	(Ident/Cover)* Max score	Deviation from top hit
Select seq gb|EU413916.1|	Viola alba isolate ALB184 (V6) internal transcribed spacer 1, partial sequence; 5.8S ribosomal RNA gene, complete sequence; and internal transcribed spacer 2, partial sequence	1035	1035	100%	0.0	99%	EU413916.1	1024,65	100,0%
Select seq gb|HM486499.1|	Viola alba subsp. alba voucher GUH-13684 18S ribosomal RNA gene, partial sequence; internal transcribed spacer 1, 5.8S ribosomal RNA gene, and internal transcribed spacer 2, complete sequence; and 28S ribosomal RNA gene, partial sequence	1033	1033	100%	0.0	99%	HM486499.1	1022,67	99,8%
Select seq gb|EU413913.1|	Viola alba isolate ALB190 (V12) internal transcribed spacer 1, partial sequence; 5.8S ribosomal RNA gene, complete sequence; and internal transcribed spacer 2, partial sequence	1031	1031	100%	0.0	99%	EU413913.1	1020,69	99,6%
Select seq gb|EU413914.1|	Viola alba isolate ALB176 (V1) internal transcribed spacer 1, partial sequence; 5.8S ribosomal RNA gene, complete sequence; and internal transcribed spacer 2, partial sequence	1029	1029	100%	0.0	99%	EU413914.1	1018,71	99,4%
Select seq gb|HM851449.1|	Viola alba voucher GUH-13655 18S ribosomal RNA gene, partial sequence; internal transcribed spacer 1, 5.8S ribosomal RNA gene, and internal transcribed spacer 2, complete sequence; and 28S ribosomal RNA gene, partial sequence	1027	1027	100%	0.0	98%	HM851449.1	1006,46	98,2%
Select seq gb|HM851450.1|	Viola alba voucher GUH-13688 18S ribosomal RNA gene, partial sequence; internal transcribed spacer 1, 5.8S ribosomal RNA gene, and internal transcribed spacer 2, complete sequence; and 28S ribosomal RNA gene, partial sequence	1026	1026	100%	0.0	98%	HM851450.1	1005,48	98,1%
Select seq gb|EU413945.1|	Viola hirta isolate HIR142 (V91) internal transcribed spacer 1, partial sequence; 5.8S ribosomal RNA gene, complete sequence; and internal transcribed spacer 2, partial sequence	1000	1000	99%	0.0	98%	EU413945.1	989,90	96,6%
Select seq gb|EU413947.1|	Viola hirta isolate HIR158 (V94) internal transcribed spacer 1, partial sequence; 5.8S ribosomal RNA gene, complete sequence; and internal transcribed spacer 2, partial sequence	990	990	99%	0.0	97%	EU413947.1	970,00	94,7%
Select seq gb|AY928296.1|	Viola hondoensis voucher Yoo1124 internal transcribed spacer 1, 5.8S ribosomal RNA gene, and internal transcribed spacer 2, complete sequence	845	845	85%	0.0	97%	AY928296.1	964,29	94,1%
Select seq gb|EU413922.1|	Viola odorata isolate ODO178 (V11) internal transcribed spacer 1, partial sequence; 5.8S ribosomal RNA gene, complete sequence; and internal transcribed spacer 2, partial sequence	992	992	100%	0.0	97%	EU413922.1	962,24	93,9%
Select seq gb|EU413948.1|	Viola hirta isolate HIR156 (V93) internal transcribed spacer 1, partial sequence; 5.8S ribosomal RNA gene, complete sequence; and internal transcribed spacer 2, partial sequence	981	981	99%	0.0	97%	EU413948.1	961,18	93,8%
Select seq gb|JF683822.1|	Viola pyrenaica isolate 272e (V434) internal transcribed spacer 1, partial sequence; 5.8S ribosomal RNA gene, complete sequence; and internal transcribed spacer 2, partial sequence	987	987	100%	0.0	97%	JF683822.1	957,39	93,4%
Select seq gb|EU430656.1|	Viola alba subsp. dehnhardtii clone 200781 internal transcribed spacer 1, partial sequence; 5.8S ribosomal RNA gene, complete sequence; and internal transcribed spacer 2, partial sequence	987	987	100%	0.0	97%	EU430656.1	957,39	93,4%
Select seq gb|EU413919.1|	Viola odorata isolate ODO214 (V8) internal transcribed spacer 1, partial sequence; 5.8S ribosomal RNA gene, complete sequence; and internal transcribed spacer 2, partial sequence	987	987	100%	0.0	97%	EU413919.1	957,39	93,4%
Select seq gb|EU413918.1|	Viola odorata isolate ODO182 (V3) internal transcribed spacer 1, partial sequence; 5.8S ribosomal RNA gene, complete sequence; and internal transcribed spacer 2, partial sequence	987	987	100%	0.0	97%	EU413918.1	957,39	93,4%
Select seq gb|EU413921.1|	Viola odorata isolate ODO215 (V13) internal transcribed spacer 1, partial sequence; 5.8S ribosomal RNA gene, complete sequence; and internal transcribed spacer 2, partial sequence	985	985	100%	0.0	97%	EU413921.1	955,45	93,2%
Select seq gb|JF683842.1|	Viola thomasiana isolate 273c (V461) internal transcribed spacer 1, partial sequence; 5.8S ribosomal RNA gene, complete sequence; and internal transcribed spacer 2, partial sequence	972	972	99%	0.0	97%	JF683842.1	952,36	92,9%
Select seq gb|EU430661.1|	Viola x balearica clone 200786 internal transcribed spacer 1, partial sequence; 5.8S ribosomal RNA gene, complete sequence; and internal transcribed spacer 2, partial sequence	981	981	100%	0.0	97%	EU430661.1	951,57	92,9%
Select seq gb|EU430659.1|	Viola jaubertiana clone 200784 internal transcribed spacer 1, partial sequence; 5.8S ribosomal RNA gene, complete sequence; and internal transcribed spacer 2, partial sequence	981	981	100%	0.0	97%	EU430659.1	951,57	92,9%
Select seq gb|EU413923.1|	Viola suavis isolate SUB211 (V7) internal transcribed spacer 1, partial sequence; 5.8S ribosomal RNA gene, complete sequence; and internal transcribed spacer 2, partial sequence	981	981	100%	0.0	97%	EU413923.1	951,57	92,9%
Select seq gb|JF683831.1|	Viola suavis subsp. adriatica isolate 249c (V264) internal transcribed spacer 1, partial sequence; 5.8S ribosomal RNA gene, complete sequence; and internal transcribed spacer 2, partial sequence	979	979	100%	0.0	97%	JF683831.1	949,63	92,7%
Select seq gb|JF683843.1|	Viola thomasiana isolate 273e (V427) internal transcribed spacer 1, partial sequence; 5.8S ribosomal RNA gene, complete sequence; and internal transcribed spacer 2, partial sequence	968	968	99%	0.0	97%	JF683843.1	948,44	92,6%
Select seq gb|HM851451.1|	Viola odorata voucher GUH-13657 18S ribosomal RNA gene, partial sequence; internal transcribed spacer 1, 5.8S ribosomal RNA gene, and internal transcribed spacer 2, complete sequence; and 28S ribosomal RNA gene, partial sequence	977	977	100%	0.0	97%	HM851451.1	947,69	92,5%
Select seq gb|FJ002880.1|	Viola collina internal transcribed spacer 1, partial sequence; 5.8S ribosomal RNA gene, complete sequence; and internal transcribed spacer 2, partial sequence	977	977	100%	0.0	97%	FJ002880.1	947,69	92,5%
Select seq gb|EU413930.1|	Viola suavis isolate SUW170 (V17) internal transcribed spacer 1, partial sequence; 5.8S ribosomal RNA gene, complete sequence; and internal transcribed spacer 2, partial sequence	976	976	100%	0.0	97%	EU413930.1	946,72	92,4%
Select seq gb|EU413924.1|	Viola suavis isolate SUB174 (V16) internal transcribed spacer 1, partial sequence; 5.8S ribosomal RNA gene, complete sequence; and internal transcribed spacer 2, partial sequence	976	976	100%	0.0	97%	EU413924.1	946,72	92,4%

Select seq gb|AY928274.1|	Viola websteri voucher Chon030430 internal transcribed spacer 1, 5.8S ribosomal RNA gene, and internal transcribed spacer 2, complete sequence	976	976	100% 0.0	97% AY928274.1	946,72	92,4%
Select seq gb|JF683834.1|	Viola suavis subsp. adriatica isolate 228a (V314) internal transcribed spacer 1, partial sequence; 5.8S ribosomal RNA gene, complete sequence; and internal transcribed spacer 2, partial sequence	974	974	100% 0.0	97% JF683834.1	944,78	92,2%
Select seq gb|JF683821.1|	Viola pyrenaica isolate 271h (V431B) internal transcribed spacer 1, partial sequence; 5.8S ribosomal RNA gene, complete sequence; and internal transcribed spacer 2, partial sequence	974	974	100% 0.0	97% JF683821.1	944,78	92,2%
Select seq gb|AY928272.1|	Viola hondoensis voucher Yoo837 internal transcribed spacer 1, 5.8S ribosomal RNA gene, and internal transcribed spacer 2, complete sequence	974	974	100% 0.0	97% AY928272.1	944,78	92,2%
Select seq gb|JF683835.1|	Viola suavis ssp. PM-2011b isolate 241h (V316) internal transcribed spacer 1, partial sequence; 5.8S ribosomal RNA gene, complete sequence; and internal transcribed spacer 2, partial sequence	970	970	100% 0.0	97% JF683835.1	940,90	91,8%
Select seq gb|HM851448.1|	Viola rupestris voucher GUH-13485-2 18S ribosomal RNA gene, partial sequence; internal transcribed spacer 1, 5.8S ribosomal RNA gene, and internal transcribed spacer 2, complete sequence; and 28S ribosomal RNA gene, partial sequence	970	970	100% 0.0	97% HM851448.1	940,90	91,8%
Select seq gb|DQ521291.1|	Viola sp. Hearn cult33 18S ribosomal RNA gene, partial sequence; internal transcribed spacer 1, 5.8S ribosomal RNA gene, and internal transcribed spacer 2, complete sequence; and 28S ribosomal RNA gene, partial sequence	970	970	100% 0.0	97% DQ521291.1	940,90	91,8%
Select seq gb|AY928282.1|	Viola grypoceras var. exilis voucher Yoo833 internal transcribed spacer 1, 5.8S ribosomal RNA gene, and internal transcribed spacer 2, complete sequence	970	970	100% 0.0	97% AY928282.1	940,90	91,8%
Select seq gb|AY928281.1|	Viola grypoceras f. albiflora voucher Yoo868 internal transcribed spacer 1, 5.8S ribosomal RNA gene, and internal transcribed spacer 2, complete sequence	970	970	100% 0.0	97% AY928281.1	940,90	91,8%
Select seq gb|EU413910.1|	Viola reichenbachiana isolate REI220 (V106) internal transcribed spacer 1, partial sequence; 5.8S ribosomal RNA gene, complete sequence; and internal transcribed spacer 2, partial sequence	968	968	100% 0.0	97% EU413910.1	938,96	91,6%
Select seq gb|DQ055382.1|	Viola reichenbachiana clone g1 from Germany 18S ribosomal RNA gene, partial sequence; internal transcribed spacer 1, 5.8S ribosomal RNA gene, and internal transcribed spacer 2, complete sequence; and 26S ribosomal RNA gene, partial sequence	968	968	100% 0.0	97% DQ055382.1	938,96	91,6%
Select seq gb|JF683836.1|	Viola suavis ssp. PM-2011b isolate 235d (V320) internal transcribed spacer 1, partial sequence; 5.8S ribosomal RNA gene, complete sequence; and internal transcribed spacer 2, partial sequence	966	966	100% 0.0	97% JF683836.1	937,02	91,4%
Select seq gb|EU413936.1|	Viola ambigua isolate AMB149 (V27) internal transcribed spacer 1, partial sequence; 5.8S ribosomal RNA gene, complete sequence; and internal transcribed spacer 2, partial sequence	966	966	100% 0.0	97% EU413936.1	937,02	91,4%
Select seq gb|EU413934.1|	Viola ambigua isolate AMB21 (V36) internal transcribed spacer 1, partial sequence; 5.8S ribosomal RNA gene, complete sequence; and internal transcribed spacer 2, partial sequence	966	966	100% 0.0	97% EU413934.1	937,02	91,4%
Select seq gb|EU413933.1|	Viola ambigua isolate AMB21 (V38) internal transcribed spacer 1, partial sequence; 5.8S ribosomal RNA gene, complete sequence; and internal transcribed spacer 2, partial sequence	966	966	100% 0.0	97% EU413933.1	937,02	91,4%
Select seq gb|FJ002889.1|	Viola labradorica internal transcribed spacer 1, partial sequence; 5.8S ribosomal RNA gene, complete sequence; and internal transcribed spacer 2, partial sequence	965	965	100% 0.0	97% FJ002889.1	936,05	91,4%
Select seq gb|AY928280.1|	Viola grypoceras voucher Yoo826 internal transcribed spacer 1, 5.8S ribosomal RNA gene, and internal transcribed spacer 2, complete sequence	965	965	100% 0.0	97% AY928280.1	936,05	91,4%
Select seq gb|AY928278.1|	Viola kusanoana voucher Yoo863 internal transcribed spacer 1, 5.8S ribosomal RNA gene, and internal transcribed spacer 2, complete sequence	965	965	100% 0.0	97% AY928278.1	936,05	91,4%
Select seq gb|DQ055389.1|	Viola reichenbachiana clone b6 from Germany 18S ribosomal RNA gene, partial sequence; internal transcribed spacer 1, 5.8S ribosomal RNA gene, and internal transcribed spacer 2, complete sequence; and 26S ribosomal RNA gene, partial sequence	963	963	100% 0.0	97% DQ055389.1	934,11	91,2%
Select seq gb|DQ055385.1|	Viola reichenbachiana clone a4 from Germany 18S ribosomal RNA gene, partial sequence; internal transcribed spacer 1, 5.8S ribosomal RNA gene, and internal transcribed spacer 2, complete sequence; and 26S ribosomal RNA gene, partial sequence	963	963	100% 0.0	97% DQ055385.1	934,11	91,2%
Select seq gb|DQ055384.1|	Viola reichenbachiana clone a1 from Germany 18S ribosomal RNA gene, partial sequence; internal transcribed spacer 1, 5.8S ribosomal RNA gene, and internal transcribed spacer 2, complete sequence; and 26S ribosomal RNA gene, partial sequence	963	963	100% 0.0	97% DQ055384.1	934,11	91,2%
Select seq gb|DQ055383.1|	Viola reichenbachiana clone g3 from Germany 18S ribosomal RNA gene, partial sequence; internal transcribed spacer 1, 5.8S ribosomal RNA gene, and internal transcribed spacer 2, complete sequence; and 26S ribosomal RNA gene, partial sequence	963	963	100% 0.0	97% DQ055383.1	934,11	91,2%
Select seq gb|AY928276.1|	Viola sacchalinensis voucher Kim040507 internal transcribed spacer 1, 5.8S ribosomal RNA gene, and internal transcribed spacer 2, complete sequence	963	963	100% 0.0	97% AY928276.1	934,11	91,2%
Select seq gb|KF977431.1|	Viola labradorica internal transcribed spacer 1, partial sequence; 5.8S ribosomal RNA gene, complete sequence; and internal transcribed spacer 2, partial sequence	959	959	100% 0.0	96% KF977431.1	920,64	89,8%
Select seq gb|JF683838.1|	Viola suavis ssp. PM-2011b isolate 238e (V300) internal transcribed spacer 1, partial sequence; 5.8S ribosomal RNA gene, complete sequence; and internal transcribed spacer 2, partial sequence	959	959	100% 0.0	96% JF683838.1	920,64	89,8%
Select seq gb|FJ002887.1|	Viola serrula internal transcribed spacer 1, partial sequence; 5.8S ribosomal RNA gene, complete sequence; and internal transcribed spacer 2, partial sequence	959	959	100% 0.0	96% FJ002887.1	920,64	89,8%
Select seq gb|FJ002884.1|	Viola acuminata internal transcribed spacer 1, partial sequence; 5.8S ribosomal RNA gene, complete sequence; and internal transcribed spacer 2, partial sequence	959	959	100% 0.0	96% FJ002884.1	920,64	89,8%

Select seq gb|AY928273.1|	Viola acuminata voucher Yoo799 internal transcribed spacer 1, 5.8S ribosomal RNA gene, and internal transcribed spacer 2, complete sequence	959	959	100%	0.0	96%	AY928273.1	920,64	89,8%
Select seq dbj|LC079044.1|	Viola rostrata var. japonica genes for 18S rRNA, ITS1, 5.8S rRNA, ITS2, 28S rRNA, partial and complete sequence, isolate: rosAkt	955	955	100%	0.0	96%	LC079044.1	916,80	89,5%
Select seq gb|FJ002888.1|	Viola rupestris internal transcribed spacer 1, partial sequence; 5.8S ribosomal RNA gene, complete sequence; and internal transcribed spacer 2, partial sequence	953	953	100%	0.0	96%	FJ002888.1	914,88	89,3%
Select seq gb|AY928277.1|	Viola ovato-oblonga voucher Yoo931 internal transcribed spacer 1, 5.8S ribosomal RNA gene, and internal transcribed spacer 2, complete sequence	953	953	100%	0.0	96%	AY928277.1	914,88	89,3%
Select seq gb|JN682058.1|	Viola lanaiensis internal transcribed spacer 1, partial sequence; 5.8S ribosomal RNA gene, complete sequence; and internal transcribed spacer 2, partial sequence	819	819	86%	0.0	96%	JN682058.1	914,23	89,2%
Select seq dbj|LC079047.1|	Viola rostrata var. japonica x Viola violacea var. makinoi genes for 18S rRNA, ITS1, 5.8S rRNA, ITS2, 28S rRNA, partial and complete sequence, isolate: RxMHana	950	950	100%	0.0	96%	LC079047.1	912,00	89,0%
Select seq dbj|LC079043.1|	Viola rostrata var. japonica genes for 18S rRNA, ITS1, 5.8S rRNA, ITS2, 28S rRNA, partial and complete sequence, isolate: rosNish	950	950	100%	0.0	96%	LC079043.1	912,00	89,0%
Select seq dbj|LC079042.1|	Viola rostrata var. japonica genes for 18S rRNA, ITS1, 5.8S rRNA, ITS2, 28S rRNA, partial and complete sequence, isolate: rosHana2	950	950	100%	0.0	96%	LC079042.1	912,00	89,0%
Select seq dbj|LC079041.1|	Viola rostrata var. japonica genes for 18S rRNA, ITS1, 5.8S rRNA, ITS2, 28S rRNA, partial and complete sequence, isolate: rosHana1	950	950	100%	0.0	96%	LC079041.1	912,00	89,0%
Select seq gb|FJ002883.1|	Viola stewardiana internal transcribed spacer 1, partial sequence; 5.8S ribosomal RNA gene, complete sequence; and internal transcribed spacer 2, partial sequence	948	948	100%	0.0	96%	FJ002883.1	910,08	88,8%
Select seq gb|FJ002881.1|	Viola grypoceras internal transcribed spacer 1, partial sequence; 5.8S ribosomal RNA gene, complete sequence; and internal transcribed spacer 2, partial sequence	948	948	100%	0.0	96%	FJ002881.1	910,08	88,8%
Select seq gb|FJ553042.1|	Angiosperm environmental sample clone LTSP_EUKA_P2C02 18S ribosomal RNA, 18S-25/28S ribosomal RNA intergenic spacer, partial sequence	935	935	99%	0.0	96%	FJ553042.1	906,67	88,5%
Select seq gb|FJ002885.1|	Viola shinchikuensis internal transcribed spacer 1, partial sequence; 5.8S ribosomal RNA gene, complete sequence; and internal transcribed spacer 2, partial sequence	942	942	100%	0.0	96%	FJ002885.1	904,32	88,3%
Select seq gb|HM486500.1|	Viola caspia voucher GUH-13686 18S ribosomal RNA gene, partial sequence; internal transcribed spacer 1, 5.8S ribosomal RNA gene, and internal transcribed spacer 2, complete sequence; and 28S ribosomal RNA gene, partial sequence	918	918	100%	0.0	95%	HM486500.1	872,10	85,1%
Select seq gb|HM851447.1|	Viola reichenbachiana voucher GUH-13652 18S ribosomal RNA gene, partial sequence; internal transcribed spacer 1 and 5.8S ribosomal RNA gene, complete sequence; and internal transcribed spacer 2, partial sequence	824	824	89%	0.0	94%	HM851447.1	870,29	84,9%
Select seq gb|FJ002886.1|	Viola shinchikuensis internal transcribed spacer 1, partial sequence; 5.8S ribosomal RNA gene, complete sequence; and internal transcribed spacer 2, partial sequence	909	909	100%	0.0	95%	FJ002886.1	863,55	84,3%
Select seq gb|HM851446.1|	Viola caspia x Viola reichenbachiana voucher GUH-13687 18S ribosomal RNA gene, partial sequence; internal transcribed spacer 1, 5.8S ribosomal RNA gene, and internal transcribed spacer 2, complete sequence; and 28S ribosomal RNA gene, partial sequence	867	867	100%	0.0	93%	HM851446.1	806,31	78,7%
Select seq gb|HM851445.1|	Viola caspia voucher GUH-13653 18S ribosomal RNA gene, partial sequence; internal transcribed spacer 1, 5.8S ribosomal RNA gene, and internal transcribed spacer 2, complete sequence; and 28S ribosomal RNA gene, partial sequence	854	854	100%	0.0	93%	HM851445.1	794,22	77,5%
Select seq gb|HM756250.1|	Viola caspia voucher GUH-13661 internal transcribed spacer 1, partial sequence; 5.8S ribosomal RNA gene and internal transcribed spacer 2, complete sequence; and 28S ribosomal RNA gene, partial sequence	837	837	100%	0.0	93%	HM756250.1	778,41	76,0%
Select seq emb|HG315513.1|	Viola raunsiensis genomic DNA containing 18S rRNA gene, ITS1, 5.8S rRNA gene, ITS2 and 28S rRNA gene, individual no. 3	737	737	100%	0.0	90%	HG315513.1	663,30	64,7%
Select seq emb|HG315512.1|	Viola raunsiensis genomic DNA containing 18S rRNA gene, ITS1, 5.8S rRNA gene, ITS2 and 28S rRNA gene, individual no. 2	737	737	100%	0.0	90%	HG315512.1	663,30	64,7%
Select seq gb|DQ055359.1|	Viola lutea subsp. calaminaria clone d3 from Germany 18S ribosomal RNA gene, partial sequence; internal transcribed spacer 1, 5.8S ribosomal RNA gene, and internal transcribed spacer 2, complete sequence; and 26S ribosomal RNA gene, partial sequence	737	737	100%	0.0	90%	DQ055359.1	663,30	64,7%
Select seq emb|HG315517.1|	Viola cf. macedonica KHE-2013 genomic DNA containing ITS1, 5.8S rRNA gene, ITS2 and 28S rRNA gene, individual no. 1	734	734	100%	0.0	90%	HG315517.1	660,60	64,5%
Select seq emb|HG315515.1|	Viola raunsiensis genomic DNA containing 18S rRNA gene, ITS1, 5.8S rRNA gene, ITS2 and 28S rRNA gene, individual no. 5	734	734	100%	0.0	90%	HG315515.1	660,60	64,5%
Select seq emb|HG315503.1|	Viola aetolica genomic DNA containing ITS1, 5.8S rRNA gene, ITS2 and 28S rRNA gene, individual no. 1	734	734	100%	0.0	90%	HG315503.1	660,60	64,5%
Select seq emb|HG315516.1|	Viola macedonica genomic DNA containing 18S rRNA gene, ITS1, 5.8S rRNA gene, ITS2 and 28S rRNA gene, individual no. 1 Munella A	732	732	100%	0.0	90%	HG315516.1	658,80	64,3%
Select seq emb|HG315510.1|	Viola macedonica genomic DNA containing 18S rRNA gene, ITS1, 5.8S rRNA gene, ITS2 and 28S rRNA gene, individual no. 1 Munella B	732	732	100%	0.0	90%	HG315510.1	658,80	64,3%

Select seq emb|HG315504.1|	Viola dukadjinica genomic DNA containing 18S rRNA gene, ITS1, 5.8S rRNA gene, ITS2 and 28S rRNA gene, individual no. 1	732	732	100% 0.0	90%	HG315504.1	658,80	64,3%
Select seq gb|HM851454.1|	Viola arvensis voucher TARI-81800 18S ribosomal RNA gene, partial sequence; internal transcribed spacer 1, 5.8S ribosomal RNA gene, and internal transcribed spacer 2, complete sequence; and 28S ribosomal RNA gene, partial sequence	732	732	100% 0.0	90%	HM851454.1	658,80	64,3%
Select seq gb|FJ002879.1|	Viola sp. 15983 internal transcribed spacer 1, partial sequence; 5.8S ribosomal RNA gene, complete sequence; and internal transcribed spacer 2, partial sequence	732	732	100% 0.0	90%	FJ002879.1	658,80	64,3%
Select seq gb|FJ002878.1|	Viola atroviolacea internal transcribed spacer 1, partial sequence; 5.8S ribosomal RNA gene, complete sequence; and internal transcribed spacer 2, partial sequence	732	732	100% 0.0	90%	FJ002878.1	658,80	64,3%
Select seq gb|DQ055404.1|	Viola tricolor subsp. curtisii clone d3 from Netherlands 18S ribosomal RNA gene, partial sequence; internal transcribed spacer 1, 5.8S ribosomal RNA gene, and internal transcribed spacer 2, complete sequence; and 26S ribosomal RNA gene, partial sequence	732	732	100% 0.0	90%	DQ055404.1	658,80	64,3%
Select seq gb|DQ055400.1|	Viola tricolor subsp. curtisii clone b3 from Netherlands 18S ribosomal RNA gene, partial sequence; internal transcribed spacer 1, 5.8S ribosomal RNA gene, and internal transcribed spacer 2, complete sequence; and 26S ribosomal RNA gene, partial sequence	732	732	100% 0.0	90%	DQ055400.1	658,80	64,3%
Select seq gb|DQ055393.1|	Viola tricolor subsp. tricolor clone b1 from Germany 18S ribosomal RNA gene, partial sequence; internal transcribed spacer 1, 5.8S ribosomal RNA gene, and internal transcribed spacer 2, complete sequence; and 26S ribosomal RNA gene, partial sequence	732	732	100% 0.0	90%	DQ055393.1	658,80	64,3%
Select seq gb|DQ055391.1|	Viola tricolor subsp. tricolor clone a3 from Germany 18S ribosomal RNA gene, partial sequence; internal transcribed spacer 1, 5.8S ribosomal RNA gene, and internal transcribed spacer 2, complete sequence; and 26S ribosomal RNA gene, partial sequence	732	732	100% 0.0	90%	DQ055391.1	658,80	64,3%
Select seq gb|DQ055366.1|	Viola lutea clone f4 from France 18S ribosomal RNA gene, partial sequence; internal transcribed spacer 1, 5.8S ribosomal RNA gene, and internal transcribed spacer 2, complete sequence; and 26S ribosomal RNA gene, partial sequence	732	732	100% 0.0	90%	DQ055366.1	658,80	64,3%
Select seq emb|HG315514.1|	Viola raunsiensis genomic DNA containing 18S rRNA gene, ITS1, 5.8S rRNA gene, ITS2 and 28S rRNA gene, individual no. 4	730	730	100% 0.0	90%	HG315514.1	657,00	64,1%
Select seq emb|HG315511.1|	Viola raunsiensis genomic DNA containing 18S rRNA gene, ITS1, 5.8S rRNA gene, ITS2 and 28S rRNA gene, individual no. 1	728	728	100% 0.0	90%	HG315511.1	655,20	63,9%
Select seq emb|HG315508.1|	Viola albanica genomic DNA containing 18S rRNA gene, ITS1, 5.8S rRNA gene, ITS2 and 28S rRNA gene, individual no. 4	728	728	100% 0.0	90%	HG315508.1	655,20	63,9%
Select seq emb|HG315507.1|	Viola albanica genomic DNA containing ITS1, 5.8S rRNA gene, ITS2 and 28S rRNA gene, individual no. 3	728	728	100% 0.0	90%	HG315507.1	655,20	63,9%
Select seq emb|HG315506.1|	Viola albanica genomic DNA containing 18S rRNA gene, ITS1, 5.8S rRNA gene, ITS2 and 28S rRNA gene, individual no. 2	728	728	100% 0.0	90%	HG315506.1	655,20	63,9%
Select seq emb|HG315505.1|	Viola albanica genomic DNA containing 18S rRNA gene, ITS1, 5.8S rRNA gene, ITS2 and 28S rRNA gene, individual no. 1	728	728	100% 0.0	90%	HG315505.1	655,20	63,9%
Select seq emb|HG315518.1|	Viola lutea subsp. sudetica genomic DNA containing 18S rRNA gene, ITS1, 5.8S rRNA gene, ITS2 and 28S rRNA gene, individual no. 1	726	726	100% 0.0	90%	HG315518.1	653,40	63,8%
Select seq emb|HG315509.1|	Viola albanica genomic DNA containing 18S rRNA gene, ITS1, 5.8S rRNA gene, ITS2 and 28S rRNA gene, individual no. 5	726	726	100% 0.0	90%	HG315509.1	653,40	63,8%
Select seq gb|HM851455.1|	Viola tricolor voucher GUH-13656 18S ribosomal RNA gene, partial sequence; internal transcribed spacer 1, 5.8S ribosomal RNA gene, and internal transcribed spacer 2, complete sequence; and 28S ribosomal RNA gene, partial sequence	726	726	100% 0.0	90%	HM851455.1	653,40	63,8%
Select seq gb|DQ055413.1|	Viola tricolor subsp. tricolor clone c5 from Poland 18S ribosomal RNA gene, partial sequence; internal transcribed spacer 1, 5.8S ribosomal RNA gene, and internal transcribed spacer 2, complete sequence; and 26S ribosomal RNA gene, partial sequence	726	726	100% 0.0	90%	DQ055413.1	653,40	

Select for downloading or viewing reports	Kh036_trnL Description	Max score	Total score	Query cover	E value	Ident	Accession	(Ident/Cover)* Max score	Deviation from top hit
Select seq gb|JF767169.1|	Viola chelmea voucher TM352 tRNA-Leu (trnL) gene, partial sequence; trnL-trnF intergenic spacer, complete sequence; and tRNA-Phe (trnF) gene, partial sequence; chloroplast	1360	1360	100%	0.0	99%	JF767169.1	1346,40	100,0%
Select seq gb|GQ262537.1|	Viola hondoensis voucher KWNU Sun s.n. tRNA-Leu (trnL) gene, partial sequence; trnL-trnF intergenic spacer, complete sequence; and tRNA-Phe (trnF) gene, partial sequence; chloroplast	1360	1360	100%	0.0	99%	GQ262537.1	1346,40	100,0%
Select seq gb|DQ085888.1|	Viola hondoensis voucher Yoo837 tRNA-Leu (trnL) gene, partial sequence; trnL-trnF intergenic spacer, complete sequence; and tRNA-Phe (trnF) gene, partial sequence; chloroplast	1354	1354	100%	0.0	99%	DQ085888.1	1340,46	99,6%
Select seq gb|DQ085892.1|	Viola websteri voucher Chon030430 tRNA-Leu (trnL) gene, partial sequence; trnL-trnF intergenic spacer, complete sequence; and tRNA-Phe (trnF) gene, partial sequence; chloroplast	1338	1338	99%	0.0	99%	DQ085892.1	1338,00	99,4%
Select seq gb|HM483555.1|	Viola hondoensis voucher 0106 tRNA-Leu (trnL) gene, partial sequence; and trnL-trnF intergenic spacer, complete sequence; chloroplast	1323	1323	98%	0.0	99%	HM483555.1	1336,50	99,3%
Select seq gb|DQ085909.1|	Viola hondoensis voucher Yoo1124 tRNA-Leu (trnL) gene, partial sequence; trnL-trnF intergenic spacer, complete sequence; and tRNA-Phe (trnF) gene, partial sequence; chloroplast	1349	1349	100%	0.0	99%	DQ085909.1	1335,51	99,2%
Select seq gb|JF767171.1|	Viola collina voucher BRNU-590933 tRNA-Leu (trnL) gene, partial sequence; trnL-trnF intergenic spacer, complete sequence; and tRNA-Phe (trnF) gene, partial sequence; chloroplast	1347	1347	100%	0.0	99%	JF767171.1	1333,53	99,0%
Select seq gb|JF767172.1|	Viola mirabilis voucher TM675 tRNA-Leu (trnL) gene, partial sequence; trnL-trnF intergenic spacer, complete sequence; and tRNA-Phe (trnF) gene, partial sequence; chloroplast	1343	1343	100%	0.0	99%	JF767172.1	1329,57	98,8%
Select seq gb|DQ085894.1|	Viola grypoceras f. albiflora voucher Yoo868 tRNA-Leu (trnL) gene, partial sequence; trnL-trnF intergenic spacer, complete sequence; and tRNA-Phe (trnF) gene, partial sequence; chloroplast	1343	1343	100%	0.0	99%	DQ085894.1	1329,57	98,8%
Select seq gb|DQ085893.1|	Viola kusanoana voucher Yoo863 tRNA-Leu (trnL) gene, partial sequence; trnL-trnF intergenic spacer, complete sequence; and tRNA-Phe (trnF) gene, partial sequence; chloroplast	1343	1343	100%	0.0	99%	DQ085893.1	1329,57	98,8%
Select seq gb|DQ085887.1|	Viola collina voucher Yoo814 tRNA-Leu (trnL) gene, partial sequence; trnL-trnF intergenic spacer, complete sequence; and tRNA-Phe (trnF) gene, partial sequence; chloroplast	1343	1343	100%	0.0	99%	DQ085887.1	1329,57	98,8%
Select seq gb|DQ085895.1|	Viola grypoceras var. exilis voucher Yoo833 tRNA-Leu (trnL) gene, partial sequence; trnL-trnF intergenic spacer, complete sequence; and tRNA-Phe (trnF) gene, partial sequence; chloroplast	1338	1338	100%	0.0	99%	DQ085895.1	1324,62	98,4%
Select seq gb|DQ085891.1|	Viola grypoceras voucher Yoo826 tRNA-Leu (trnL) gene, partial sequence; trnL-trnF intergenic spacer, complete sequence; and tRNA-Phe (trnF) gene, partial sequence; chloroplast	1338	1338	100%	0.0	99%	DQ085891.1	1324,62	98,4%
Select seq gb|HM483564.1|	Viola shinchikuensis voucher 128 tRNA-Leu (trnL) gene, partial sequence; and trnL-trnF intergenic spacer, complete sequence; chloroplast	1306	1306	98%	0.0	99%	HM483564.1	1319,33	98,0%
Select seq gb|GQ262534.1|	Viola mirabilis voucher KWNU 65496 tRNA-Leu (trnL) gene, partial sequence; trnL-trnF intergenic spacer, complete sequence; and tRNA-Phe (trnF) gene, partial sequence; chloroplast	1325	1325	100%	0.0	99%	GQ262534.1	1311,75	97,4%
Select seq gb|DQ085923.1|	Viola sacchalinensis voucher Kim s.n. tRNA-Leu (trnL) gene, partial sequence; trnL-trnF intergenic spacer, complete sequence; and tRNA-Phe (trnF) gene, partial sequence; chloroplast	1321	1321	100%	0.0	99%	DQ085923.1	1307,79	97,1%
Select seq gb|JF767167.1|	Viola brevistipulata voucher TM745 tRNA-Leu (trnL) gene, partial sequence; trnL-trnF intergenic spacer, complete sequence; and tRNA-Phe (trnF) gene, partial sequence; chloroplast	1310	1310	100%	0.0	99%	JF767167.1	1296,90	96,3%
Select seq gb|JF767174.1|	Viola uliginosa voucher TM662 tRNA-Leu (trnL) gene, partial sequence; trnL-trnF intergenic spacer, complete sequence; and tRNA-Phe (trnF) gene, partial sequence; chloroplast	1306	1306	100%	0.0	99%	JF767174.1	1292,94	96,0%
Select seq gb|DQ085929.1|	Viola orientalis voucher Yoo924 tRNA-Leu (trnL) gene, partial sequence; trnL-trnF intergenic spacer, complete sequence; and tRNA-Phe (trnF) gene, partial sequence; chloroplast	1304	1304	100%	0.0	99%	DQ085929.1	1290,96	95,9%
Select seq gb|JF767165.1|	Viola biflora voucher TM775 tRNA-Leu (trnL) gene, partial sequence; trnL-trnF intergenic spacer, complete sequence; and tRNA-Phe (trnF) gene, partial sequence; chloroplast	1303	1303	100%	0.0	98%	JF767165.1	1276,94	94,8%
Select seq gb|DQ085886.1|	Viola brevistipulata var. minor voucher Yoo810 tRNA-Leu (trnL) gene, partial sequence; trnL-trnF intergenic spacer, complete sequence; and tRNA-Phe (trnF) gene, partial sequence; chloroplast	1299	1299	100%	0.0	98%	DQ085886.1	1273,02	94,5%
Select seq gb|JF767168.1|	Viola rotundifolia voucher TM734 tRNA-Leu (trnL) gene, partial sequence; trnL-trnF intergenic spacer, complete sequence; and tRNA-Phe (trnF) gene, partial sequence; chloroplast	1297	1297	100%	0.0	98%	JF767168.1	1271,06	94,4%
Select seq gb|DQ085922.1|	Viola biflora voucher Paik s.n. tRNA-Leu (trnL) gene, partial sequence; trnL-trnF intergenic spacer, complete sequence; and tRNA-Phe (trnF) gene, partial sequence; chloroplast	1297	1297	100%	0.0	98%	DQ085922.1	1271,06	94,4%
Select seq gb|HM483567.1|	Viola serrula voucher 320 tRNA-Leu (trnL) gene, partial sequence; and trnL-trnF intergenic spacer, complete sequence; chloroplast	1267	1267	98%	0.0	98%	HM483567.1	1267,00	94,1%
Select seq gb|JF767166.1|	Viola uniflora voucher BRNU-580353 tRNA-Leu (trnL) gene, partial sequence; trnL-trnF intergenic spacer, complete sequence; and tRNA-Phe (trnF) gene, partial sequence; chloroplast	1275	1275	100%	0.0	98%	JF767166.1	1249,50	92,8%
Select seq gb|DQ085890.1|	Viola acuminata voucher Yoo799 tRNA-Leu (trnL) gene, partial sequence; trnL-trnF intergenic spacer, complete sequence; and tRNA-Phe (trnF) gene, partial sequence; chloroplast	1266	1266	100%	0.0	97%	DQ085890.1	1228,02	91,2%

Select seq gb|HM483568.1|	Viola stewardiana voucher 211 tRNA-Leu (trnL) gene, partial sequence; and trnL-trnF intergenic spacer, complete sequence; chloroplast	1240	1240	98%	0.0	97%	HM483568.1	1227,35	91,2%
Select seq gb|JF767161.1|	Viola lobata voucher TM762 tRNA-Leu (trnL) gene, partial sequence; trnL-trnF intergenic spacer, complete sequence; and tRNA-Phe (trnF) gene, partial sequence; chloroplast	1262	1262	100%	0.0	97%	JF767161.1	1224,14	90,9%
Select seq gb|JF767173.1|	Viola striata voucher TM731 tRNA-Leu (trnL) gene, partial sequence; trnL-trnF intergenic spacer, complete sequence; and tRNA-Phe (trnF) gene, partial sequence; chloroplast	1253	1253	100%	0.0	97%	JF767173.1	1215,41	90,3%
Select seq gb|JF767162.1|	Viola pubescens voucher TM637 tRNA-Leu (trnL) gene, partial sequence; trnL-trnF intergenic spacer, complete sequence; and tRNA-Phe (trnF) gene, partial sequence; chloroplast	1251	1251	100%	0.0	97%	JF767162.1	1213,47	90,1%
Select seq gb|JF767175.1|	Viola laricicola voucher TM509 tRNA-Leu (trnL) gene, partial sequence; trnL-trnF intergenic spacer, complete sequence; and tRNA-Phe (trnF) gene, partial sequence; chloroplast	1247	1247	100%	0.0	97%	JF767175.1	1209,59	89,8%
Select seq gb|JF767170.1|	Viola hirta voucher TM682 tRNA-Leu (trnL) gene, partial sequence; trnL-trnF intergenic spacer, complete sequence; and tRNA-Phe (trnF) gene, partial sequence; chloroplast	1247	1247	100%	0.0	97%	JF767170.1	1209,59	89,8%
Select seq gb|JN620200.1|	Viola guadalupensis voucher VIGU1 tRNA-Leu (trnL) gene, partial sequence; trnL-trnF intergenic spacer, complete sequence; and tRNA-Phe (trnF) gene, partial sequence; chloroplast	1243	1243	100%	0.0	97%	JN620200.1	1205,71	89,6%
Select seq gb|JF767180.1|	Viola bulbosa subsp. tuberifera voucher TM948 tRNA-Leu (trnL) gene, partial sequence; trnL-trnF intergenic spacer, complete sequence; and tRNA-Phe (trnF) gene, partial sequence; chloroplast	1242	1242	100%	0.0	97%	JF767180.1	1204,74	89,5%
Select seq gb|JN620205.1|	Viola tomentosa voucher O:TM768 tRNA-Leu (trnL) gene, partial sequence; trnL-trnF intergenic spacer, complete sequence; and tRNA-Phe (trnF) gene, partial sequence; chloroplast	1232	1232	100%	0.0	97%	JN620205.1	1195,04	88,8%
Select seq gb|JN620204.1|	Viola bakeri voucher O:TM769 tRNA-Leu (trnL) gene, partial sequence; trnL-trnF intergenic spacer, complete sequence; and tRNA-Phe (trnF) gene, partial sequence; chloroplast	1232	1232	100%	0.0	97%	JN620204.1	1195,04	88,8%
Select seq gb|JN620201.1|	Viola guadalupensis voucher VIGU2 tRNA-Leu (trnL) gene, partial sequence; trnL-trnF intergenic spacer, complete sequence; and tRNA-Phe (trnF) gene, partial sequence; chloroplast	1232	1232	100%	0.0	97%	JN620201.1	1195,04	88,8%
Select seq gb|JF767160.1|	Viola purpurea voucher TM758 tRNA-Leu (trnL) gene, partial sequence; trnL-trnF intergenic spacer, complete sequence; and tRNA-Phe (trnF) gene, partial sequence; chloroplast	1232	1232	100%	0.0	97%	JF767160.1	1195,04	88,8%
Select seq gb|HM483566.1|	Viola rupestris voucher 1511 tRNA-Leu (trnL) gene, partial sequence; and trnL-trnF intergenic spacer, complete sequence; chloroplast	1214	1214	98%	0.0	96%	HM483566.1	1189,22	88,3%
Select seq gb|KJ138158.1|	Viola cinerea tRNA-Leu (trnL) gene, partial sequence; trnL-trnF intergenic spacer, complete sequence; and tRNA-Phe (trnF) gene, partial sequence	1214	1214	100%	0.0	96%	KJ138158.1	1165,44	86,6%
Select seq gb|JN620203.1|	Viola douglasii voucher O:TM764 tRNA-Leu (trnL) gene, partial sequence; trnL-trnF intergenic spacer, complete sequence; and tRNA-Phe (trnF) gene, partial sequence; chloroplast	1214	1214	100%	0.0	96%	JN620203.1	1165,44	86,6%
Select seq gb|JN620207.1|	Viola maculata voucher O:TM1000 tRNA-Leu (trnL) gene, partial sequence; trnL-trnF intergenic spacer, complete sequence; and tRNA-Phe (trnF) gene, partial sequence; chloroplast	1212	1212	100%	0.0	96%	JN620207.1	1163,52	86,4%
Select seq gb|JF767158.1|	Viola maculata voucher TM947 tRNA-Leu (trnL) gene, partial sequence; trnL-trnF intergenic spacer, complete sequence; and tRNA-Phe (trnF) gene, partial sequence; chloroplast	1212	1212	100%	0.0	96%	JF767158.1	1163,52	86,4%
Select seq gb|DQ085928.1|	Melicytus ramiflorus voucher KEW47461 tRNA-Leu (trnL) gene, partial sequence; trnL-trnF intergenic spacer, complete sequence; and tRNA-Phe (trnF) gene, partial sequence; chloroplast	1199	1199	99%	0.0	96%	DQ085928.1	1162,67	86,4%
Select seq gb|JF767159.1|	Viola sheltonii voucher TM757 tRNA-Leu (trnL) gene, partial sequence; trnL-trnF intergenic spacer, complete sequence; and tRNA-Phe (trnF) gene, partial sequence; chloroplast	1206	1206	100%	0.0	96%	JF767159.1	1157,76	86,0%
Select seq gb|KJ138162.1|	Viola reichei tRNA-Leu (trnL) gene, partial sequence; trnL-trnF intergenic spacer, complete sequence; and tRNA-Phe (trnF) gene, partial sequence	1201	1201	100%	0.0	96%	KJ138162.1	1152,96	85,6%
Select seq gb|KJ138166.1|	Viola tridentata tRNA-Leu (trnL) gene, partial sequence; trnL-trnF intergenic spacer, complete sequence; and tRNA-Phe (trnF) gene, partial sequence	1199	1199	100%	0.0	96%	KJ138166.1	1151,04	85,5%
Select seq gb|JF767157.1|	Viola rubella voucher TM671 tRNA-Leu (trnL) gene, partial sequence; trnL-trnF intergenic spacer, complete sequence; and tRNA-Phe (trnF) gene, partial sequence; chloroplast	1184	1184	100%	0.0	95%	JF767157.1	1124,80	83,5%
Select seq gb|JF767155.1|	Viola arguta voucher HEB02-309 tRNA-Leu (trnL) gene, partial sequence; trnL-trnF intergenic spacer, complete sequence; and tRNA-Phe (trnF) gene, partial sequence; chloroplast	1171	1171	100%	0.0	95%	JF767155.1	1112,45	82,6%
Select seq gb|JF767156.1|	Viola capillaris voucher TM748 tRNA-Leu (trnL) gene, partial sequence; trnL-trnF intergenic spacer, complete sequence; and tRNA-Phe (trnF) gene, partial sequence; chloroplast	1168	1168	100%	0.0	95%	JF767156.1	1109,60	82,4%
Select seq gb|KJ138165.1|	Viola stipularis tRNA-Leu (trnL) gene, partial sequence; trnL-trnF intergenic spacer, complete sequence; and tRNA-Phe (trnF) gene, partial sequence	1166	1166	100%	0.0	95%	KJ138165.1	1107,70	82,3%
Select seq gb|JF767184.1|	Viola epipsila subsp. epipsila voucher TM661 tRNA-Leu (trnL) gene, partial sequence; trnL-trnF intergenic spacer, complete sequence; and tRNA-Phe (trnF) gene, partial sequence; chloroplast	1059	1278	96%	0.0	99%	JF767184.1	1092,09	81,1%
Select seq gb|JF767182.1|	Viola vaginata voucher TM946 tRNA-Leu (trnL) gene, partial sequence; trnL-trnF intergenic spacer, complete sequence; and tRNA-Phe (trnF) gene, partial sequence; chloroplast	1059	1278	96%	0.0	99%	JF767182.1	1092,09	81,1%
Select seq gb|DQ085900.1|	Viola diamantiaca voucher Yoo030513 tRNA-Leu (trnL) gene, partial sequence; trnL-trnF intergenic spacer, complete sequence; and tRNA-Phe (trnF) gene, partial sequence; chloroplast	1059	1272	96%	0.0	99%	DQ085900.1	1092,09	81,1%

Select seq gb|HM483565.1|	Viola rossii voucher 0024 tRNA-Leu (trnL) gene, partial sequence; and trnL-trnF intergenic spacer, complete sequence; chloroplast	1033	1252	94%	0.0	99%	HM483565.1	1087,95	80,8%
Select seq gb|HM483560.1|	Viola moupinensis voucher 152 tRNA-Leu (trnL) gene, partial sequence; and trnL-trnF intergenic spacer, complete sequence; chloroplast	1033	1246	94%	0.0	99%	HM483560.1	1087,95	80,8%
Select seq gb|KC699707.1|	Viola subdimidiata voucher Paula-Souza et al. 5853 (ESA) tRNA-Thr (trnT) gene and trnL-trnF intergenic spacer, partial sequence; chloroplast	1122	1122	97%	0.0	94%	KC699707.1	1087,30	80,8%
Select seq gb|JF767186.1|	Viola epipsila subsp. repens voucher TM928 tRNA-Leu (trnL) gene, partial sequence; trnL-trnF intergenic spacer, complete sequence; and tRNA-Phe (trnF) gene, partial sequence; chloroplast	1053	1272	96%	0.0	99%	JF767186.1	1085,91	80,7%
Select seq gb|JF767185.1|	Viola epipsila subsp. repens voucher TM927 tRNA-Leu (trnL) gene, partial sequence; trnL-trnF intergenic spacer, complete sequence; and tRNA-Phe (trnF) gene, partial sequence; chloroplast	1053	1272	96%	0.0	99%	JF767185.1	1085,91	80,7%
Select seq gb|JF767183.1|	Viola principis voucher TM795 tRNA-Leu (trnL) gene, partial sequence; trnL-trnF intergenic spacer, complete sequence; and tRNA-Phe (trnF) gene, partial sequence; chloroplast	1053	1272	96%	0.0	99%	JF767183.1	1085,91	80,7%
Select seq gb|JF767181.1|	Viola verecunda voucher TM697 tRNA-Leu (trnL) gene, partial sequence; trnL-trnF intergenic spacer, complete sequence; and tRNA-Phe (trnF) gene, partial sequence; chloroplast	1053	1267	96%	0.0	99%	JF767181.1	1085,91	80,7%
Select seq gb|DQ085924.1|	Viola verecunda var. semilunaris voucher Yoo040507 tRNA-Leu (trnL) gene, partial sequence; trnL-trnF intergenic spacer, complete sequence; and tRNA-Phe (trnF) gene, partial sequence; chloroplast	1053	1267	96%	0.0	99%	DQ085924.1	1085,91	80,7%
Select seq gb|DQ085896.1|	Viola verecunda voucher Yoo975 tRNA-Leu (trnL) gene, partial sequence; trnL-trnF intergenic spacer, complete sequence; and tRNA-Phe (trnF) gene, partial sequence; chloroplast	1053	1267	96%	0.0	99%	DQ085896.1	1085,91	80,7%
Select seq gb|HM483571.1|	Viola arcuata voucher 1553 tRNA-Leu (trnL) gene, partial sequence; and trnL-trnF intergenic spacer, complete sequence; chloroplast	1027	1235	94%	0.0	99%	HM483571.1	1081,63	80,3%
Select seq gb|JF767199.1|	Viola occidentalis voucher TM753 tRNA-Leu (trnL) gene, partial sequence; trnL-trnF intergenic spacer, complete sequence; and tRNA-Phe (trnF) gene, partial sequence; chloroplast	1048	1267	96%	0.0	99%	JF767199.1	1080,75	80,3%
Select seq gb|JF767197.1|	Viola jalapaensis voucher HEB045 tRNA-Leu (trnL) gene, partial sequence; trnL-trnF intergenic spacer, complete sequence; and tRNA-Phe (trnF) gene, partial sequence; chloroplast	1048	1261	96%	0.0	99%	JF767197.1	1080,75	80,3%
Select seq gb|JF767189.1|	Viola lanceolata voucher TM739 tRNA-Leu (trnL) gene, partial sequence; trnL-trnF intergenic spacer, complete sequence; and tRNA-Phe (trnF) gene, partial sequence; chloroplast	1048	1267	96%	0.0	99%	JF767189.1	1080,75	80,3%
Select seq gb|JF767188.1|	Viola renifolia voucher TM932 tRNA-Leu (trnL) gene, partial sequence; trnL-trnF intergenic spacer, complete sequence; and tRNA-Phe (trnF) gene, partial sequence; chloroplast	1048	1202	96%	0.0	99%	JF767188.1	1080,75	80,3%
Select seq gb|DQ787752.1|	Viola dissecta voucher KWNU56518 tRNA-Leu (trnL) gene, partial sequence; trnL-trnF intergenic spacer, complete sequence; and tRNA-Phe (trnF) gene, partial sequence; chloroplast	1048	1228	96%	0.0	99%	DQ787752.1	1080,75	80,3%
Select seq gb|DQ085925.1|	Viola raddeana voucher Yoo030603 tRNA-Leu (trnL) gene, partial sequence; trnL-trnF intergenic spacer, complete sequence; and tRNA-Phe (trnF) gene, partial sequence; chloroplast	1048	1267	96%	0.0	99%	DQ085925.1	1080,75	80,3%
Select seq gb|DQ085897.1|	Viola verecunda voucher Yoo1129 tRNA-Leu (trnL) gene, partial sequence; trnL-trnF intergenic spacer, complete sequence; and tRNA-Phe (trnF) gene, partial sequence; chloroplast	1048	1261	96%	0.0	99%	DQ085897.1	1080,75	80,3%
Select seq gb|JF767205.1|	Viola clauseniana voucher TM707 tRNA-Leu (trnL) gene, partial sequence; trnL-trnF intergenic spacer, complete sequence; and tRNA-Phe (trnF) gene, partial sequence; chloroplast	1042	1256	96%	0.0	99%	JF767205.1	1074,56	79,8%
Select seq gb|JF767202.1|	Viola chamissoniana subsp. tracheliifolia voucher UC-1598182 tRNA-Leu (trnL) gene, partial sequence; trnL-trnF intergenic spacer, complete sequence; and tRNA-Phe (trnF) gene, partial sequence; chloroplast	1042	1261	96%	0.0	99%	JF767202.1	1074,56	79,8%
Select seq gb|JF767201.1|	Viola langsdorffii voucher TM618 tRNA-Leu (trnL) gene, partial sequence; trnL-trnF intergenic spacer, complete sequence; and tRNA-Phe (trnF) gene, partial sequence; chloroplast	1042	1261	96%	0.0	99%	JF767201.1	1074,56	79,8%
Select seq gb|JF767200.1|	Viola langsdorffii voucher TM617 tRNA-Leu (trnL) gene, partial sequence; trnL-trnF intergenic spacer, complete sequence; and tRNA-Phe (trnF) gene, partial sequence; chloroplast	1042	1261	96%	0.0	99%	JF767200.1	1074,56	79,8%
Select seq gb|JF767198.1|	Viola macloskeyi voucher TM761 tRNA-Leu (trnL) gene, partial sequence; trnL-trnF intergenic spacer, complete sequence; and tRNA-Phe (trnF) gene, partial sequence; chloroplast	1042	1256	96%	0.0	99%	JF767198.1	1074,56	79,8%
Select seq gb|JF767195.1|	Viola primulifolia voucher TM911 tRNA-Leu (trnL) gene, partial sequence; trnL-trnF intergenic spacer, complete sequence; and tRNA-Phe (trnF) gene, partial sequence; chloroplast	1042	1261	96%	0.0	99%	JF767195.1	1074,56	79,8%
Select seq gb|JF767190.1|	Viola blanda voucher TM727 tRNA-Leu (trnL) gene, partial sequence; trnL-trnF intergenic spacer, complete sequence; and tRNA-Phe (trnF) gene, partial sequence; chloroplast	1042	1261	96%	0.0	99%	JF767190.1	1074,56	79,8%
Select seq gb|JF767187.1|	Viola palustris voucher TM933 tRNA-Leu (trnL) gene, partial sequence; trnL-trnF intergenic spacer, complete sequence; and tRNA-Phe (trnF) gene, partial sequence; chloroplast	1042	1257	96%	0.0	99%	JF767187.1	1074,56	79,8%
Select seq gb|DQ085898.1|	Viola rossii voucher Yoo030514 tRNA-Leu (trnL) gene, partial sequence; trnL-trnF intergenic spacer, complete sequence; and tRNA-Phe (trnF) gene, partial sequence; chloroplast	1042	1261	96%	0.0	99%	DQ085898.1	1074,56	79,8%
Select seq gb|KC699706.1|	Viola cerasifolia voucher Paula-Souza et al. 5800 (ESA) tRNA-Thr (trnT) gene and trnL-trnF intergenic spacer, partial sequence; chloroplast	1099	1099	97%	0.0	94%	KC699706.1	1065,01	79,1%
Select seq gb|JQ950636.1|	Viola dactyloides voucher MHA S. Dshloezuor 80 tRNA-Leu (trnL) gene, partial sequence; trnL-trnF intergenic spacer, complete sequence; and tRNA-Phe (trnF) gene, partial sequence; chloroplast	1037	1245	96%	0.0	98%	JQ950636.1	1058,60	78,6%

Select seq gb|JQ950635.1|	Viola dactyloides voucher KWNU80906 tRNA-Leu (trnL) gene, partial sequence; trnL-trnF intergenic spacer, complete sequence; and tRNA-Phe (trnF) gene, partial sequence; chloroplast	1037	1245	96% 0.0	98% JQ950635.1	1058,60	78,6%
Select seq gb|JF767204.1|	Viola pedata voucher TM729 tRNA-Leu (trnL) gene, partial sequence; trnL-trnF intergenic spacer, complete sequence; and tRNA-Phe (trnF) gene, partial sequence; chloroplast	1037	1250	96% 0.0	98% JF767204.1	1058,60	78,6%
Select seq gb|JF767194.1|	Viola primulifolia voucher TM730 tRNA-Leu (trnL) gene, partial sequence; trnL-trnF intergenic spacer, complete sequence; and tRNA-Phe (trnF) gene, partial sequence; chloroplast	1037	1256	96% 0.0	98% JF767194.1	1058,60	78,6%
Select seq gb|GQ262535.1|	Viola tokubuchiana var. takedana voucher KWNU 65497 tRNA-Leu (trnL) gene, partial sequence; trnL-trnF intergenic spacer, complete sequence; and tRNA-Phe (trnF) gene, partial sequence; chloroplast	1037	1222	96% 0.0	98% GQ262535.1	1058,60	78,6%
Select seq gb|JQ950634.1|	Viola chaerophylloides var. sieboldiana voucher KWNU78676 tRNA-Leu (trnL) gene, partial sequence; trnL-trnF intergenic spacer, complete sequence; and tRNA-Phe (trnF) gene, partial sequence; chloroplast	1035	1248	96% 0.0	98% JQ950634.1	1056,56	78,5%
Select seq gb|JF767178.1|	Viola somchetica voucher TM636 tRNA-Leu (trnL) gene, partial sequence; trnL-trnF intergenic spacer, complete sequence; and tRNA-Phe (trnF) gene, partial sequence; chloroplast	1035	1248	96% 0.0	98% JF767178.1	1056,56	78,5%
Select seq gb|JF767177.1|	Viola diffusa voucher TM711 tRNA-Leu (trnL) gene, partial sequence; trnL-trnF intergenic spacer, complete sequence; and tRNA-Phe (trnF) gene, partial sequence; chloroplast	1035	1248	96% 0.0	98% JF767177.1	1056,56	78,5%
Select seq gb|DQ787748.1|	Viola chaerophylloides voucher KWNU57081 tRNA-Leu (trnL) gene, partial sequence; trnL-trnF intergenic spacer, complete sequence; and tRNA-Phe (trnF) gene, partial sequence; chloroplast	1035	1248	96% 0.0	98% DQ787748.1	1056,56	78,5%
Select seq gb|DQ787744.1|	Viola chaerophylloides voucher KWNU57064 tRNA-Leu (trnL) gene, partial sequence; trnL-trnF intergenic spacer, complete sequence; and tRNA-Phe (trnF) gene, partial sequence; chloroplast	1035	1248	96% 0.0	98% DQ787744.1	1056,56	78,5%
Select seq gb|DQ787742.1|	Viola chaerophylloides voucher KWNU56516 tRNA-Leu (trnL) gene, partial sequence; trnL-trnF intergenic spacer, complete sequence; and tRNA-Phe (trnF) gene, partial sequence; chloroplast	1035	1248	96% 0.0	98% DQ787742.1	1056,56	78,5%
Select seq gb|DQ085905.1|	Viola x takahashii voucher Yoo030520 tRNA-Leu (trnL) gene, partial sequence; trnL-trnF intergenic spacer, complete sequence; and tRNA-Phe (trnF) gene, partial sequence; chloroplast	1035	1248	96% 0.0	98% DQ085905.1	1056,56	78,5%
Select seq gb|JF767203.1|	Viola grahamii voucher A-T1-KK tRNA-Leu (trnL) gene, partial sequence; trnL-trnF intergenic spacer, complete sequence; and tRNA-Phe (trnF) gene, partial sequence; chloroplast	1031	1245	96% 0.0	98% JF767203.1	1052,48	78,2%
Select seq gb|GQ262536.1|	Viola tokubuchiana f. variegata voucher KWNU 65498 tRNA-Leu (trnL) gene, partial sequence; trnL-trnF intergenic spacer, complete sequence; and tRNA-Phe (trnF) gene, partial sequence; chloroplast	1031	1217	96% 0.0	98% GQ262536.1	1052,48	78,2%
Select seq gb|JQ950646.1|	Viola pinnata voucher MHA I. Krasnoborov et V. Hanminchun 151 tRNA-Leu (trnL) gene, partial sequence; trnL-trnF intergenic spacer, complete sequence; and tRNA-Phe (trnF) gene, partial sequence; chloroplast	1029	1243	96% 0.0	98% JQ950646.1	1050,44	78,0%
Select seq gb|JQ950645.1|	Viola pinnata voucher US3011392 tRNA-Leu (trnL) gene, partial sequence; trnL-trnF intergenic spacer, complete sequence; and tRNA-Phe (trnF) gene, partial sequence; chloroplast	1029	1243	96% 0.0	98% JQ950645.1	1050,44	78,0%
Select seq gb|KJ138156.1|	Viola banksii tRNA-Leu (trnL) gene, partial sequence; trnL-trnF intergenic spacer, complete sequence; and tRNA-Phe (trnF) gene, partial sequence	1112	1112	100% 0.0	94% KJ138156.1	1045,28	77,6%
Select seq gb|JF767153.1|	Viola pusilla voucher TM749 tRNA-Leu (trnL) gene, partial sequence; trnL-trnF intergenic spacer, complete sequence; and tRNA-Phe (trnF) gene, partial sequence; chloroplast	1074	1074	100% 0.0	93% JF767153.1	998,82	

Select for downloading or viewing reports	Kh038_ITS Description	Max score	Total score	Query cover	E value	Ident	Accession	(Ident/Cover)* Max score	Deviation from top hit
Select seq gb|HM176652.1|	Cuminum cyminum cultivar RZ-19 18S ribosomal RNA gene, partial sequence; internal transcribed spacer 1, 5.8S ribosomal RNA gene, and internal transcribed spacer 2, complete sequence; and 28S ribosomal RNA gene, partial sequence	1232	1232	88%	0.0	100%	HM176652.1	1400,00	100,0%
Select seq gb|HM176655.1|	Cuminum cyminum cultivar RZ-341 18S ribosomal RNA gene, partial sequence; internal transcribed spacer 1, 5.8S ribosomal RNA gene, and internal transcribed spacer 2, complete sequence; and 28S ribosomal RNA gene, partial sequence	1221	1221	88%	0.0	99%	HM176655.1	1373,63	98,1%
Select seq gb|HM176654.1|	Cuminum cyminum cultivar RZ-209 18S ribosomal RNA gene, partial sequence; internal transcribed spacer 1, 5.8S ribosomal RNA gene, and internal transcribed spacer 2, complete sequence; and 28S ribosomal RNA gene, partial sequence	1221	1221	88%	0.0	99%	HM176654.1	1373,63	98,1%
Select seq gb|HM176653.1|	Cuminum cyminum cultivar RZ-223 18S ribosomal RNA gene, partial sequence; internal transcribed spacer 1, 5.8S ribosomal RNA gene, and internal transcribed spacer 2, complete sequence; and 28S ribosomal RNA gene, partial sequence	1216	1216	88%	0.0	99%	HM176653.1	1368,00	97,7%
Select seq gb|HM176650.1|	Cuminum cyminum cultivar GC-2 18S ribosomal RNA gene, partial sequence; internal transcribed spacer 1, 5.8S ribosomal RNA gene, and internal transcribed spacer 2, complete sequence; and 28S ribosomal RNA gene, partial sequence	1201	1201	87%	0.0	99%	HM176650.1	1366,66	97,6%
Select seq gb|KF160677.1|	Cuminum cyminum voucher C:Simonsen 2013-8 internal transcribed spacer 1, partial sequence; 5.8S ribosomal RNA gene, complete sequence; and internal transcribed spacer 2, partial sequence	1297	1297	94%	0.0	99%	KF160677.1	1365,99	97,6%
Select seq gb|HM176651.1|	Cuminum cyminum cultivar GC-4 18S ribosomal RNA gene, partial sequence; internal transcribed spacer 1, 5.8S ribosomal RNA gene, and internal transcribed spacer 2, complete sequence; and 28S ribosomal RNA gene, partial sequence	1210	1210	88%	0.0	99%	HM176651.1	1361,25	97,2%
Select seq gb|U78362.2|CCU78362	Cuminum cyminum internal transcribed spacer 1, 5.8S ribosomal RNA gene, and internal transcribed spacer 2, complete sequence	1072	1072	79%	0.0	99%	U78362.2	1343,39	96,0%
Select seq gb|KR150163.1|	Cuminum sp. Kh1 internal transcribed spacer 1, partial sequence; 5.8S ribosomal RNA gene gene, complete sequence; and internal transcribed spacer 2 region, partial sequence	1018	1018	85%	0.0	96%	KR150163.1	1149,74	82,1%
Select seq gb|KF160679.1|	Laserpitium siler voucher C:Simonsen 2013-5 internal transcribed spacer 1, partial sequence; 5.8S ribosomal RNA gene, complete sequence; and internal transcribed spacer 2, partial sequence	996	996	93%	0.0	92%	KF160679.1	985,29	70,4%
Select seq gb|FJ415113.1|	Laserpitium siler voucher Gardner & Gardner 2455 30-VII-1984 (E 00043177) internal transcribed spacer 1, 5.8S ribosomal RNA gene, and internal transcribed spacer 2, complete sequence	804	804	79%	0.0	91%	FJ415113.1	926,13	66,2%
Select seq gb|KF160678.1|	Laser trilobum voucher C:Simonsen 2013-6 internal transcribed spacer 1, partial sequence; 5.8S ribosomal RNA gene, complete sequence; and internal transcribed spacer 2, partial sequence	965	965	95%	0.0	91%	KF160678.1	924,37	66,0%
Select seq gb|FJ415112.1|	Laserpitium siler voucher Gardner & Gardner 781 13-VII-1980 (E 00043183) internal transcribed spacer 1, 5.8S ribosomal RNA gene, and internal transcribed spacer 2, complete sequence	802	802	79%	0.0	91%	FJ415112.1	923,82	66,0%
Select seq gb|FJ415136.1|	Thapsia meoides voucher cult., Con. bot. de la Ville de Mulhouse 98138 7-IX-2000 internal transcribed spacer 1, 5.8S ribosomal RNA gene, and internal transcribed spacer 2, complete sequence	800	800	79%	0.0	91%	FJ415136.1	921,52	65,8%
Select seq gb|FJ415132.1|	Laserpitium nitidum voucher Cobau 2715 5-VI-1920 (KRA) internal transcribed spacer 1, 5.8S ribosomal RNA gene, and internal transcribed spacer 2, complete sequence	797	797	79%	0.0	91%	FJ415132.1	918,06	65,6%
Select seq gb|FJ415123.1|	Laserpitium nestleri voucher Fabregat & Udias 2007 1991 (BCC) internal transcribed spacer 1, 5.8S ribosomal RNA gene, and internal transcribed spacer 2, complete sequence	784	784	78%	0.0	91%	FJ415123.1	914,67	65,3%
Select seq gb|KJ004342.1|	Spergula fallax 18S ribosomal RNA gene, partial sequence; internal transcribed spacer 1, 5.8S ribosomal RNA gene, and internal transcribed spacer 2, complete sequence; and 26S ribosomal RNA gene, partial sequence	913	913	91%	0.0	91%	KJ004342.1	913,00	65,2%
Select seq gb|FJ415152.1|	Laserpitium stevenii voucher Muibaniani & al. 12-V-1979 (LE) internal transcribed spacer 1, 5.8S ribosomal RNA gene, and internal transcribed spacer 2, complete sequence	791	791	79%	0.0	91%	FJ415152.1	911,15	65,1%
Select seq gb|KF850594.1|	Ferula communis voucher KSUFS821 18S ribosomal RNA gene, partial sequence; internal transcribed spacer 1, 5.8S ribosomal RNA gene, and internal transcribed spacer 2, complete sequence; and 28S ribosomal RNA gene, partial sequence	889	889	89%	0.0	91%	KF850594.1	908,98	64,9%
Select seq gb|FJ415134.1|	Laserpitium pseudomeum voucher Gustavsson 9672 10-VI-1985 (G) internal transcribed spacer 1, 5.8S ribosomal RNA gene, and internal transcribed spacer 2, complete sequence	787	787	79%	0.0	91%	FJ415134.1	906,54	64,8%
Select seq gb|KF160692.1|	Thapsia gummifera voucher C:Weitzel 2011-2 internal transcribed spacer 1, partial sequence; 5.8S ribosomal RNA gene, complete sequence; and internal transcribed spacer 2, partial sequence	946	946	95%	0.0	91%	KF160692.1	906,17	64,7%
Select seq gb|KF160672.1|	Ferula communis voucher C:Weitzel 2011-4 internal transcribed spacer 1, partial sequence; 5.8S ribosomal RNA gene, complete sequence; and internal transcribed spacer 2, partial sequence	941	941	95%	0.0	91%	KF160672.1	901,38	64,4%
Select seq gb|FJ415121.1|	Laserpitium nestleri subsp. flabellatum voucher Montserrat 22-VIII-2000 (JACA R265475) internal transcribed spacer 1, 5.8S ribosomal RNA gene, and internal transcribed spacer 2, complete sequence	780	780	78%	0.0	90%	FJ415121.1	900,00	64,3%
Select seq gb|FJ415120.1|	Laserpitium eliasii subsp. thalictrifolium voucher Rico 11-VIII-1985 (SALA 41460) internal transcribed spacer 1, 5.8S ribosomal RNA gene, and internal transcribed spacer 2, complete sequence	778	778	78%	0.0	90%	FJ415120.1	897,69	64,1%
Select seq gb|FJ415133.1|	Laserpitium peucedanoides voucher Zarzycki 2-VIII-1957 (KRAM) internal transcribed spacer 1, 5.8S ribosomal RNA gene, and internal transcribed spacer 2, complete sequence	785	785	79%	0.0	90%	FJ415133.1	894,30	63,9%
Select seq gb|GQ165517.1|	Ferula communis 18S ribosomal RNA gene, partial sequence; internal transcribed spacer 1, 5.8S ribosomal RNA gene, and internal transcribed spacer 2, complete sequence; and 28S ribosomal RNA gene, partial sequence	883	883	89%	0.0	90%	GQ165517.1	892,92	63,8%
Select seq gb|JQ305147.1|	Polylophium involucratum voucher TARI-s.n. internal transcribed spacer 1, 5.8S ribosomal RNA gene, and internal transcribed spacer 2, complete sequence	780	780	79%	0.0	90%	JQ305147.1	888,61	63,5%

Select seq gb|JQ305146.1|	Laserpitium stevenii voucher MW-886 internal transcribed spacer 1, 5.8S ribosomal RNA gene, and internal transcribed spacer 2, complete sequence	780	780	79% 0.0	90% JQ305146.1	888,61	63,5%
Select seq gb|GQ165518.1|	Ferula communis 18S ribosomal RNA gene, partial sequence; internal transcribed spacer 1, 5.8S ribosomal RNA gene, and internal transcribed spacer 2, complete sequence; and 28S ribosomal RNA gene, partial sequence	878	878	89% 0.0	90% GQ165518.1	887,87	63,4%
Select seq gb|KJ660796.1|	Ferula licentiana voucher ACE 900 (E) internal transcribed spacer 1, partial sequence; 5.8S ribosomal RNA gene, complete sequence; and internal transcribed spacer 2, partial sequence	778	778	79% 0.0	90% KJ660796.1	886,33	63,3%
Select seq gb|EF560691.1|	Ferula olivacea internal transcribed spacer 1, 5.8S ribosomal RNA gene, and internal transcribed spacer 2, complete sequence	778	778	79% 0.0	90% EF560691.1	886,33	63,3%
Select seq gb|GQ165516.1|	Ferula communis 18S ribosomal RNA gene, partial sequence; internal transcribed spacer 1, 5.8S ribosomal RNA gene, and internal transcribed spacer 2, complete sequence; and 28S ribosomal RNA gene, partial sequence	872	872	89% 0.0	90% GQ165516.1	881,80	63,0%
Select seq gb|FJ415141.1|	Thapsia thapsioides voucher Davis & Lamond D 57768 12-V-1975 (E 00040997) internal transcribed spacer 1, 5.8S ribosomal RNA gene, and internal transcribed spacer 2, complete sequence	774	774	79% 0.0	90% FJ415141.1	881,77	63,0%
Select seq gb|FJ415125.1|	Laserpitium krapfii subsp. gaudinii voucher Charpin 17-VII-1969 (G) internal transcribed spacer 1, 5.8S ribosomal RNA gene, and internal transcribed spacer 2, complete sequence	774	774	79% 0.0	90% FJ415125.1	881,77	63,0%
Select seq gb|FJ415142.1|	Thapsia thapsioides voucher Davis 53419 9-VI-1971 (E) internal transcribed spacer 1, 5.8S ribosomal RNA gene, and internal transcribed spacer 2, complete sequence	771	771	79% 0.0	90% FJ415142.1	878,35	62,7%
Select seq gb|GQ165515.1|	Ferula communis 18S ribosomal RNA gene, partial sequence; internal transcribed spacer 1, 5.8S ribosomal RNA gene, and internal transcribed spacer 2, complete sequence; and 28S ribosomal RNA gene, partial sequence	867	867	89% 0.0	90% GQ165515.1	876,74	62,6%
Select seq emb|HE602450.1|	Ferula communis genomic DNA containing ITS1, 5.8S rRNA gene, ITS2, specimen voucher Jury, SL. 16346	828	828	85% 0.0	90% HE602450.1	876,71	62,6%
Select seq gb|KM983398.1|	Ferula gummosa voucher ZNU313-Taham 18S ribosomal RNA gene, partial sequence; internal transcribed spacer 1, 5.8S ribosomal RNA gene, and internal transcribed spacer 2, complete sequence; and 28S ribosomal RNA gene, partial sequence	874	874	90% 0.0	90% KM983398.1	874,00	62,4%
Select seq emb|HE687370.1|	Ferula communis genomic DNA containing ITS1, 5.8S rRNA gene, ITS2, specimen voucher EM433	811	811	84% 0.0	90% HE687370.1	868,93	62,1%
Select seq emb|FN432916.1|	Ferula haussknechtii genomic DNA sequence containing ITS1, 5.8S rRNA gene and ITS2, specimen voucher GAZI:M. Sagiroglu 2255	846	846	88% 0.0	90% FN432916.1	865,23	61,8%
Select seq emb|FN432913.1|	Ferula halophila genomic DNA sequence containing ITS1, 5.8S rRNA gene and ITS2, specimen voucher GAZI:M. Sagiroglu 2146	824	824	87% 0.0	89% FN432913.1	842,94	60,2%
Select seq gb|KF160711.1|	Thapsia smittii voucher C:Weitzel 2011-5 internal transcribed spacer 1, partial sequence; 5.8S ribosomal RNA gene, complete sequence; and internal transcribed spacer 2, partial sequence	889	889	95% 0.0	90% KF160711.1	842,21	60,2%
Select seq gb|KF160686.1|	Thapsia asclepium voucher C:Constantidinis 6835 internal transcribed spacer 1, partial sequence; 5.8S ribosomal RNA gene, complete sequence; and internal transcribed spacer 2, partial sequence	889	889	94% 0.0	89% KF160686.1	841,71	60,1%
Select seq emb|FN432920.1|	Ferula orientalis genomic DNA sequence containing ITS1, 5.8S rRNA gene, ITS2, specimen voucher GAZI:M. Sagiroglu 2170	832	832	88% 0.0	89% FN432920.1	841,45	60,1%
Select seq emb|FN432917.1|	Ferula szowitsiana genomic DNA sequence containing ITS1, 5.8S rRNA gene and ITS2, specimen voucher GAZI:M. Sagiroglu 2147	822	822	87% 0.0	89% FN432917.1	840,90	60,1%
Select seq emb|FN432914.1|	Ferula elaeochytris genomic DNA sequence containing ITS1, 5.8S rRNA gene and ITS2, specimen voucher GAZI:M. Sagiroglu 2227	822	822	87% 0.0	89% FN432914.1	840,90	60,1%
Select seq emb|FN432815.1|	Ferula sp. GAZI 2181 genomic DNA sequence containing ITS1, 5.8S rRNA gene and ITS2, specimen voucher GAZI:2181	822	822	87% 0.0	89% FN432815.1	840,90	60,1%
Select seq gb|KF160680.1|	Melanoselinum decipiens voucher C:Hansen 13407 internal transcribed spacer 1, partial sequence; 5.8S ribosomal RNA gene, complete sequence; and internal transcribed spacer 2, partial sequence	883	883	94% 0.0	89% KF160680.1	836,03	59,7%
Select seq gb|KF160706.1|	Thapsia smittii voucher C:FC 9169 internal transcribed spacer 1, partial sequence; 5.8S ribosomal RNA gene, complete sequence; and internal transcribed spacer 2, partial sequence	881	881	94% 0.0	89% KF160706.1	834,14	59,6%
Select seq emb|FN432915.1|	Ferula longipedunculata genomic DNA sequence containing ITS1, 5.8S rRNA gene and ITS2, specimen voucher GAZI:M. Sagiroglu 2235	811	811	87% 0.0	89% FN432915.1	829,64	59,3%
Select seq gb|KF160709.1|	Thapsia smittii voucher C:Smitt 87-08 internal transcribed spacer 1, partial sequence; 5.8S ribosomal RNA gene, complete sequence; and internal transcribed spacer 2, partial sequence	883	883	95% 0.0	89% KF160709.1	827,23	59,1%
Select seq gb|KF160708.1|	Thapsia smittii voucher C:Smitt 81-v-11 internal transcribed spacer 1, partial sequence; 5.8S ribosomal RNA gene, complete sequence; and internal transcribed spacer 2, partial sequence	883	883	95% 0.0	89% KF160708.1	827,23	59,1%
Select seq gb|KF160681.1|	Monizia edulis voucher C:Hansen 2478 internal transcribed spacer 1, partial sequence; 5.8S ribosomal RNA gene, complete sequence; and internal transcribed spacer 2, partial sequence	869	869	94% 0.0	89% KF160681.1	822,78	58,8%
Select seq gb|KF160707.1|	Thapsia smittii voucher C:Smitt 88-36 internal transcribed spacer 1, partial sequence; 5.8S ribosomal RNA gene, complete sequence; and internal transcribed spacer 2, partial sequence	878	878	95% 0.0	89% KF160707.1	822,55	58,8%
Select seq gb|KF160698.1|	Thapsia maxima voucher C:Smitt 87-31 internal transcribed spacer 1, partial sequence; 5.8S ribosomal RNA gene, complete sequence; and internal transcribed spacer 2, partial sequence	867	867	94% 0.0	89% KF160698.1	820,88	58,6%

Select seq gb|KF160693.1|	Thapsia gymnesica voucher C:Smitt 94-01 internal transcribed spacer 1, partial sequence; 5.8S ribosomal RNA gene, complete sequence; and internal transcribed spacer 2, partial sequence	854	854	93% 0.0	89% KF160693.1	817,27	58,4%
Select seq gb|KF160699.1|	Thapsia maxima voucher C:Smitt 88-18 internal transcribed spacer 1, partial sequence; 5.8S ribosomal RNA gene, complete sequence; and internal transcribed spacer 2, partial sequence	870	870	95% 0.0	89% KF160699.1	815,05	58,2%
Select seq emb|FN432918.1|	Ferula mervynii genomic DNA sequence containing ITS1, 5.8S rRNA gene and ITS2, specimen voucher GAZI:M. Sagiroglu 2262	832	832	91% 0.0	89% FN432918.1	813,71	58,1%
Select seq emb|HE602456.1|	Elaeoselinum meoides genomic DNA containing ITS1, 5.8S rRNA gene, ITS2, specimen voucher Jury, SL. 20572	804	804	88% 0.0	89% HE602456.1	813,14	58,1%
Select seq emb|FN432921.1|	Ferula hermonis genomic DNA sequence containing ITS1, 5.8S rRNA gene and ITS2, specimen voucher GAZI:M. Sagiroglu 2246	813	813	89% 0.0	89% FN432921.1	813,00	58,1%
Select seq gb|KF160705.1|	Thapsia minor voucher C:Smitt 88-26 internal transcribed spacer 1, partial sequence; 5.8S ribosomal RNA gene, complete sequence; and internal transcribed spacer 2, partial sequence	850	850	94% 0.0	88% KF160705.1	795,74	56,8%
Select seq gb|KF160703.1|	Thapsia minor voucher C:Smitt 88-25 internal transcribed spacer 1, partial sequence; 5.8S ribosomal RNA gene, complete sequence; and internal transcribed spacer 2, partial sequence	854	854	95% 0.0	88% KF160703.1	791,07	56,5%
Select seq gb|EU331133.1|	Chaerophyllum colensoi var. delicatulum voucher AK286407 18S ribosomal RNA gene, partial sequence; internal transcribed spacer 1, 5.8S ribosomal RNA gene, and internal transcribed spacer 2, complete sequence; and 28S ribosomal RNA gene, partial sequence	887	887	99% 0.0	88% EU331133.1	788,44	56,3%
Select seq gb|KF160704.1|	Thapsia minor voucher C:Smitt 88-30 internal transcribed spacer 1, partial sequence; 5.8S ribosomal RNA gene, complete sequence; and internal transcribed spacer 2, partial sequence	824	824	92% 0.0	88% KF160704.1	788,17	56,3%
Select seq gb|KF160696.1|	Thapsia villosa var. laciniata voucher C:Smitt 87-12 internal transcribed spacer 1, partial sequence; 5.8S ribosomal RNA gene, complete sequence; and internal transcribed spacer 2, partial sequence	841	841	94% 0.0	88% KF160696.1	787,32	56,2%
Select seq gb|KF160726.1|	Thapsia villosa voucher C:Smitt 87-32 internal transcribed spacer 1, partial sequence; 5.8S ribosomal RNA gene, complete sequence; and internal transcribed spacer 2, partial sequence	843	843	95% 0.0	88% KF160726.1	780,88	55,8%
Select seq gb|KF160723.1|	Thapsia villosa voucher C:Smitt 88-33 internal transcribed spacer 1, partial sequence; 5.8S ribosomal RNA gene, complete sequence; and internal transcribed spacer 2, partial sequence	843	843	95% 0.0	88% KF160723.1	780,88	55,8%
Select seq gb|KF160720.1|	Thapsia villosa voucher C:Smitt 81-02 internal transcribed spacer 1, partial sequence; 5.8S ribosomal RNA gene, complete sequence; and internal transcribed spacer 2, partial sequence	843	843	95% 0.0	88% KF160720.1	780,88	55,8%
Select seq gb|KF160701.1|	Thapsia minor voucher C:Smitt 81-v-10 internal transcribed spacer 1, partial sequence; 5.8S ribosomal RNA gene, complete sequence; and internal transcribed spacer 2, partial sequence	843	843	95% 0.0	88% KF160701.1	780,88	55,8%
Select seq gb|KF160694.1|	Thapsia villosa var. laciniata voucher C:Smitt 90-01 internal transcribed spacer 1, partial sequence; 5.8S ribosomal RNA gene, complete sequence; and internal transcribed spacer 2, partial sequence	843	843	95% 0.0	88% KF160694.1	780,88	55,8%
Select seq gb|KF160721.1|	Thapsia villosa voucher C:Smitt 87-19 internal transcribed spacer 1, partial sequence; 5.8S ribosomal RNA gene, complete sequence; and internal transcribed spacer 2, partial sequence	833	833	94% 0.0	88% KF160721.1	779,83	55,7%
Select seq gb|KF160722.1|	Thapsia villosa voucher C:Smitt 87-21 internal transcribed spacer 1, partial sequence; 5.8S ribosomal RNA gene, complete sequence; and internal transcribed spacer 2, partial sequence	837	837	95% 0.0	88% KF160722.1	775,33	55,4%
Select seq gb|KF160702.1|	Thapsia minor voucher C:Smitt 88-17 internal transcribed spacer 1, partial sequence; 5.8S ribosomal RNA gene, complete sequence; and internal transcribed spacer 2, partial sequence	837	837	95% 0.0	88% KF160702.1	775,33	55,4%
Select seq gb|KF160682.1|	Orlaya daucoides voucher C:Strid 42072 internal transcribed spacer 1, partial sequence; 5.8S ribosomal RNA gene, complete sequence; and internal transcribed spacer 2, partial sequence	826	826	94% 0.0	88% KF160682.1	773,28	55,2%
Select seq gb|KF160733.1|	Thapsia villosa voucher C:Weitzel 2011-8 internal transcribed spacer 1, partial sequence; 5.8S ribosomal RNA gene, complete sequence; and internal transcribed spacer 2, partial sequence	832	832	95% 0.0	88% KF160733.1	770,69	55,0%
Select seq gb|KF160683.1|	Pseudorlaya pumila voucher C:Strid 38276 internal transcribed spacer 1, partial sequence; 5.8S ribosomal RNA gene, complete sequence; and internal transcribed spacer 2, partial sequence	832	832	95% 0.0	88% KF160683.1	770,69	55,0%
Select seq gb|KF160718.1|	Thapsia transtagana voucher C:Smitt 81-t-20 internal transcribed spacer 1, partial sequence; 5.8S ribosomal RNA gene, complete sequence; and internal transcribed spacer 2, partial sequence	821	821	94% 0.0	88% KF160718.1	768,60	54,9%
Select seq gb|KF160714.1|	Thapsia transtagana voucher C:Smitt 81-t-16 internal transcribed spacer 1, partial sequence; 5.8S ribosomal RNA gene, complete sequence; and internal transcribed spacer 2, partial sequence	821	821	94% 0.0	88% KF160714.1	768,60	54,9%
Select seq gb|KF160728.1|	Thapsia villosa voucher C:Smitt 88-09 internal transcribed spacer 1, partial sequence; 5.8S ribosomal RNA gene, complete sequence; and internal transcribed spacer 2, partial sequence	826	826	95% 0.0	88% KF160728.1	765,14	54,7%
Select seq gb|KF160689.1|	Thapsia garganica voucher C:Sandberg s/n internal transcribed spacer 1, partial sequence; 5.8S ribosomal RNA gene, complete sequence; and internal transcribed spacer 2, partial sequence	815	815	94% 0.0	88% KF160689.1	762,98	54,5%
Select seq gb|AY552527.1|	Daucus carota 18S ribosomal RNA gene, partial sequence; internal transcribed spacer 1, 5.8S ribosomal RNA gene, and internal transcribed spacer 2, complete sequence; and 25S ribosomal RNA gene, partial sequence	867	867	100% 0.0	88% AY552527.1	762,96	54,5%
Select seq gb|KF160717.1|	Thapsia transtagana voucher C:Smitt 81-t-6 internal transcribed spacer 1, partial sequence; 5.8S ribosomal RNA gene, complete sequence; and internal transcribed spacer 2, partial sequence	822	822	95% 0.0	88% KF160717.1	761,43	54,4%
Select seq gb|KF160697.1|	Thapsia villosa var. laciniata voucher C:Simonsen 2010-01 internal transcribed spacer 1, partial sequence; 5.8S ribosomal RNA gene, complete sequence; and internal transcribed spacer 2, partial sequence	822	822	95% 0.0	88% KF160697.1	761,43	54,4%

Select seq gb|KF160690.1|	Thapsia garganica voucher C:Smitt 92-1 internal transcribed spacer 1, partial sequence; 5.8S ribosomal RNA gene, complete sequence; and internal transcribed spacer 2, partial sequence	804	804	93% 0.0	88% KF160690.1	760,77	54,3%
Select seq gb|KF160716.1|	Thapsia transtagana voucher C:Smitt 88-07 internal transcribed spacer 1, partial sequence; 5.8S ribosomal RNA gene, complete sequence; and internal transcribed spacer 2, partial sequence	819	819	95% 0.0	88% KF160716.1	758,65	54,2%
Select seq gb|KF160687.1|	Thapsia garganica voucher C:Living Collection DNA Ibiza internal transcribed spacer 1, partial sequence; 5.8S ribosomal RNA gene, complete sequence; and internal transcribed spacer 2, partial sequence	817	817	95% 0.0	88% KF160687.1	756,80	54,1%
Select seq gb|KF160684.1|	Rouya polygama voucher C:Greuter 9736 internal transcribed spacer 1, partial sequence; 5.8S ribosomal RNA gene, complete sequence; and internal transcribed spacer 2, partial sequence	817	817	95% 0.0	87% KF160684.1	748,20	53,4%
Select seq emb|X17534.1|	D.carota 5.8S, 18S and 25S ribosomal RNA genes and ITS regions	859	859	100% 0.0	87% X17534.1	747,33	53,4%
Select seq gb|U72363.1|GMU72363	Gingidia montana 18S ribosomal RNA and 25S ribosomal RNA genes, partial sequence, and internal transcribed spacer 1, 5.8S ribosomal RNA gene and internal transcribed spacer 2, complete sequence	791	791	93% 0.0	87% U72363.1	739,97	52,9%
Select seq gb|U72365.1|GBU72365	Gingidia baxterae 18S ribosomal RNA and 25S ribosomal RNA genes, partial sequence, and internal transcribed spacer 1, 5.8S ribosomal RNA gene and internal transcribed spacer 2, complete sequence	791	791	93% 0.0	87% U72365.1	739,97	52,9%
Select seq gb|KF160719.1|	Thapsia transtagana voucher C:Smitt 87-15 internal transcribed spacer 1, partial sequence; 5.8S ribosomal RNA gene, complete sequence; and internal transcribed spacer 2, partial sequence	806	806	95% 0.0	87% KF160719.1	738,13	52,7%
Select seq gb|KF160713.1|	Thapsia smittii voucher C:Weitzel 2011-7 internal transcribed spacer 1, partial sequence; 5.8S ribosomal RNA gene, complete sequence; and internal transcribed spacer 2, partial sequence	806	806	95% 0.0	87% KF160713.1	738,13	52,7%
Select seq gb|U72379.1|ASU72379	Aciphylla squarrosa 18S ribosomal RNA and 25S ribosomal RNA genes, partial sequence, and internal transcribed spacer 1, 5.8S ribosomal RNA gene and internal transcribed spacer 2, complete sequence	789	789	93% 0.0	87% U72379.1	738,10	52,7%
Select seq gb|EU331132.1|	Daucus glochidiatus voucher AK297601 18S ribosomal RNA gene, partial sequence; internal transcribed spacer 1, 5.8S ribosomal RNA gene, and internal transcribed spacer 2, complete sequence; and 28S ribosomal RNA gene, partial sequence	835	835	99% 0.0	87% EU331132.1	733,79	52,4%
Select seq gb|U72369.1|GFU72369	Gingidia flabellata 18S ribosomal RNA and 25S ribosomal RNA genes, partial sequence, and internal transcribed spacer 1, 5.8S ribosomal RNA gene and internal transcribed spacer 2, complete sequence	784	784	93% 0.0	87% U72369.1	733,42	52,4%
Select seq gb|U72374.1|AAU72374	Anisotome aromatica 18S ribosomal RNA and 25S ribosomal RNA genes, partial sequence, and internal transcribed spacer 1, 5.8S ribosomal RNA gene and internal transcribed spacer 2, complete sequence	784	784	93% 0.0	87% U72374.1	733,42	52,4%
Select seq gb|KF160671.1|	Aciphylla glacialis voucher C:Strid 22007 internal transcribed spacer 1, partial sequence; 5.8S ribosomal RNA gene, complete sequence; and internal transcribed spacer 2, partial sequence	793	793	95% 0.0	87% KF160671.1	726,22	51,9%
Select seq gb|U72381.1|ADU72381	Aciphylla dieffenbachii 18S ribosomal RNA and 25S ribosomal RNA genes, partial sequence, and internal transcribed spacer 1, 5.8S ribosomal RNA gene and internal transcribed spacer 2, complete sequence	774	774	93% 0.0	87% U72381.1	724,06	51,7%
Select seq gb|U72364.1|GEU72364	Gingidia enysii 18S ribosomal RNA and 25S ribosomal RNA genes, partial sequence, and internal transcribed spacer 1, 5.8S ribosomal RNA gene and internal transcribed spacer 2, complete sequence	774	774	93% 0.0	86% U72364.1	715,74	51,1%

Select for downloading or viewing reports	Kh039_ITS Description	Max score	Total score	Query cover	E value	Ident	Accession	(Ident/Cover)* Max score	Deviation from top hit
Select seq gb|JN407504.1|	Plantago major isolate shawpc0945I 18S ribosomal RNA gene, partial sequence; internal transcribed spacer 1, 5.8S ribosomal RNA gene, and internal transcribed spacer 2, complete sequence; and 28S ribosomal RNA gene, partial sequence	676	676	86%	0.0	89%	JN407504.1	699,58	100,0%
Select seq dbj|AB296068.1|	Plantago asiatica genes for ITS1, 5.8S rRNA, ITS2, haplotype: A2	676	676	86%	0.0	89%	AB296068.1	699,58	100,0%
Select seq dbj|LC076498.1|	Plantago major gene for ITS1, 5.8S rRNA and ITS2, complete sequence, isolate: Sp21	671	671	86%	0.0	88%	LC076498.1	686,60	98,1%
Select seq gb|JN407501.1|	Plantago major isolate shawpc0873I 18S ribosomal RNA gene, partial sequence; internal transcribed spacer 1, 5.8S ribosomal RNA gene, and internal transcribed spacer 2, complete sequence; and 28S ribosomal RNA gene, partial sequence	671	671	86%	0.0	88%	JN407501.1	686,60	98,1%
Select seq dbj|AB296071.1|	Plantago major genes for ITS1, 5.8S rRNA, ITS2, haplotype: M2	671	671	86%	0.0	88%	AB296071.1	686,60	98,1%
Select seq dbj|AB281165.1|	Plantago major genes for ITS1, 5.8S rRNA, ITS2, complete sequence	660	660	86%	0.0	88%	AB281165.1	675,35	96,5%
Select seq dbj|AB558160.1|	Plantago asiatica genes for 18S rRNA, ITS1, 5.8S rRNA, ITS2, 26S rRNA, partial and complete sequence	680	680	90%	0.0	88%	AB558160.1	664,89	95,0%
Select seq gb|FJ024620.1|	Plantago sp. Garnock-Jones & Tay 2566 18S ribosomal RNA gene, partial sequence; internal transcribed spacer 1, 5.8S ribosomal RNA gene, and internal transcribed spacer 2, complete sequence; and 28S ribosomal RNA gene, partial sequence	680	680	90%	0.0	88%	FJ024620.1	664,89	95,0%
Select seq dbj|AB281166.1|	Plantago hostifolia genes for 18S rRNA, ITS1, 5.8S rRNA, ITS2, 28S rRNA, partial and complete sequence	699	699	93%	0.0	87%	AB281166.1	653,90	93,5%
Select seq gb|AY101862.1|	Plantago asiatica 18S ribosomal RNA gene, partial sequence; internal transcribed spacer 1, 5.8S ribosomal RNA gene, and internal transcribed spacer 2, complete sequence; and 28S ribosomal RNA gene, partial sequence	699	699	93%	0.0	87%	AY101862.1	653,90	93,5%
Select seq emb|AJ548977.1|	Plantago asiatica partial 18S rRNA gene, 5.8S rRNA gene, partial 28S rRNA gene, ITS1 and ITS2, specimen voucher Hoggard RK #406	699	699	93%	0.0	87%	AJ548977.1	653,90	93,5%
Select seq gb|JN407500.1|	Plantago major isolate shawpc0871I 18S ribosomal RNA gene, partial sequence; internal transcribed spacer 1, 5.8S ribosomal RNA gene, and internal transcribed spacer 2, complete sequence; and 28S ribosomal RNA gene, partial sequence	641	641	86%	4,00E-180	87%	JN407500.1	648,45	92,7%
Select seq dbj|AB281164.1|	Plantago asiatica genes for ITS1, 5.8S rRNA, ITS2, complete sequence	641	641	86%	4,00E-180	87%	AB281164.1	648,45	92,7%
Select seq gb|AY101861.1|	Plantago major 18S ribosomal RNA gene, partial sequence; internal transcribed spacer 1, 5.8S ribosomal RNA gene, and internal transcribed spacer 2, complete sequence; and 28S ribosomal RNA gene, partial sequence	689	689	93%	0.0	87%	AY101861.1	644,55	92,1%
Select seq gb|FJ024619.1|	Plantago major voucher PJ Garnock-Jones 2550, WELTU 20180 18S ribosomal RNA gene, partial sequence; internal transcribed spacer 1, 5.8S ribosomal RNA gene, and internal transcribed spacer 2, complete sequence; and 28S ribosomal RNA gene, partial sequence	664	664	90%	0.0	87%	FJ024619.1	641,87	91,8%
Select seq gb|AF313038.1|AF31303 8	Plantago australis 18S ribosomal RNA gene, partial sequence; internal transcribed spacer 1, 5.8S ribosomal RNA gene and internal transcribed spacer 2, complete sequence; and 26S ribosomal RNA gene, partial sequence	627	627	86%	1,00E-175	87%	AF313038.1	634,29	90,7%
Select seq dbj|AB281167.1|	Plantago erosa genes for 18S rRNA, ITS1, 5.8S rRNA, ITS2, 28S rRNA, partial and complete sequence	678	678	93%	0.0	87%	AB281167.1	634,26	90,7%
Select seq gb|HM368731.1|	Plantago lanigera isolate 69Blani internal transcribed spacer 1, partial sequence; 5.8S ribosomal RNA gene, complete sequence; and internal transcribed spacer 2, partial sequence	619	619	85%	2,00E-173	87%	HM368731.1	633,56	90,6%
Select seq gb|AY101875.1|	Plantago uniglumis 18S ribosomal RNA gene, partial sequence; internal transcribed spacer 1, 5.8S ribosomal RNA gene, and internal transcribed spacer 2, complete sequence; and 28S ribosomal RNA gene, partial sequence	675	675	93%	0.0	87%	AY101875.1	631,45	90,3%
Select seq gb|FJ024618.1|	Plantago australis voucher ML Tay 019 & PJ Garnock-Jones, WELTU 20181 18S ribosomal RNA gene, partial sequence; internal transcribed spacer 1, 5.8S ribosomal RNA gene, and internal transcribed spacer 2, complete sequence; and 28S ribosomal RNA gene, partial sequence	647	647	90%	0.0	87%	FJ024618.1	625,43	89,4%
Select seq gb|EU602323.1|	Plantago pachyphylla voucher Dunbar 140 PTBG internal transcribed spacer 1, 5.8S ribosomal RNA gene, and internal transcribed spacer 2, complete sequence	617	617	86%	7,00E-173	87%	EU602323.1	624,17	89,2%
Select seq gb|EU602325.1|	Plantago princeps var. anomala voucher Lorence 5134 PTBG internal transcribed spacer 1, 5.8S ribosomal RNA gene, and internal transcribed spacer 2, complete sequence	616	616	86%	2,00E-172	87%	EU602325.1	623,16	89,1%
Select seq gb|EU602319.1|	Plantago hawaiensis voucher Dunbar 19 PTBG internal transcribed spacer 1, 5.8S ribosomal RNA gene, and internal transcribed spacer 2, complete sequence	616	616	86%	2,00E-172	87%	EU602319.1	623,16	89,1%
Select seq gb|HM368759.1|	Plantago unibracteata isolate 55Cunib internal transcribed spacer 1, partial sequence; 5.8S ribosomal RNA gene and internal transcribed spacer 2, complete sequence; and 28S ribosomal RNA gene, partial sequence	612	612	86%	3,00E-171	87%	HM368759.1	619,12	88,5%
Select seq gb|FJ024601.1|	Plantago unibracteata voucher ML Tay 024 & PJ Garnock-Jones, WELTU 2017[illegible] 18S ribosomal RNA gene, partial sequence; internal transcribed spacer 1, 5.8S ribosomal RNA gene, and internal transcribed spacer 2, complete sequence; and 28S ribosomal RNA gene, partial sequence	612	612	86%	3,00E-171	87%	FJ024601.1	619,12	88,5%
Select seq gb|GQ396669.1|	Plantago asiatica var. densiuscula voucher SCMR9412018 18S ribosomal RNA gene, partial sequence; internal transcribed spacer 1, 5.8S ribosomal RNA gene, and internal transcribed spacer 2, complete sequence; and 28S ribosomal RNA gene, partial sequence	664	664	93%	0.0	86%	GQ396669.1	614,02	87,8%

Select seq dbj|AB223157.1|	Plantago asiatica var. densiuscula genes for 18S rRNA, ITS1, 5.8S rRNA, ITS2, 28S rRNA, partial and complete sequence, haplotype: No. 7	664	664	93%	0.0	86%	AB223157.1	614,02	87,8%
Select seq dbj|AB223156.1|	Plantago asiatica var. densiuscula genes for 18S rRNA, ITS1, 5.8S rRNA, ITS2, 28S rRNA, partial and complete sequence, haplotype: No. 6	664	664	93%	0.0	86%	AB223156.1	614,02	87,8%
Select seq dbj|AB223151.1|	Plantago asiatica var. densiuscula genes for 18S rRNA, ITS1, 5.8S rRNA, ITS2, 28S rRNA, partial and complete sequence, haplotype: No. 1	664	664	93%	0.0	86%	AB223151.1	614,02	87,8%
Select seq gb|FJ024607.1|	Plantago euryphylla voucher BG Briggs 9743, NSW 742956 18S ribosomal RNA gene, partial sequence; internal transcribed spacer 1, 5.8S ribosomal RNA gene, and internal transcribed spacer 2, complete sequence; and 28S ribosomal RNA gene, partial sequence	641	641	90%	4,00E-180	86%	FJ024607.1	612,51	87,6%
Select seq dbj|AB223161.1|	Plantago asiatica var. densiuscula genes for 18S rRNA, ITS1, 5.8S rRNA, ITS2, 28S rRNA, partial and complete sequence, haplotype: No. 11	660	660	93%	0.0	86%	AB223161.1	610,32	87,2%
Select seq dbj|AB223159.1|	Plantago asiatica var. densiuscula genes for 18S rRNA, ITS1, 5.8S rRNA, ITS2, 28S rRNA, partial and complete sequence, haplotype: No. 9	660	660	93%	0.0	86%	AB223159.1	610,32	87,2%
Select seq dbj|AB223158.1|	Plantago asiatica var. densiuscula genes for 18S rRNA, ITS1, 5.8S rRNA, ITS2, 28S rRNA, partial and complete sequence, haplotype: No. 8	660	660	93%	0.0	86%	AB223158.1	610,32	87,2%
Select seq dbj|AB223152.1|	Plantago asiatica var. densiuscula genes for 18S rRNA, ITS1, 5.8S rRNA, ITS2, 28S rRNA, partial and complete sequence, haplotype: No. 2	660	660	93%	0.0	86%	AB223152.1	610,32	87,2%
Select seq gb|AY101873.1|	Plantago myosuros 18S ribosomal RNA gene, partial sequence; internal transcribed spacer 1, 5.8S ribosomal RNA gene, and internal transcribed spacer 2, complete sequence; and 28S ribosomal RNA gene, partial sequence	660	660	93%	0.0	86%	AY101873.1	610,32	87,2%
Select seq gb|HM368734.1|	Plantago lanigera isolate 54lani internal transcribed spacer 1, partial sequence; 5.8S ribosomal RNA gene and internal transcribed spacer 2, complete sequence; and 28S ribosomal RNA gene, partial sequence	630	630	89%	9,00E-177	86%	HM368734.1	608,76	87,0%
Select seq gb|HM368726.1|	Plantago lanigera isolate 66Alani internal transcribed spacer 1, partial sequence; 5.8S ribosomal RNA gene and internal transcribed spacer 2, complete sequence; and 28S ribosomal RNA gene, partial sequence	630	630	89%	9,00E-177	86%	HM368726.1	608,76	87,0%
Select seq dbj|AB223154.1|	Plantago asiatica var. densiuscula genes for 18S rRNA, ITS1, 5.8S rRNA, ITS2, 28S rRNA, partial and complete sequence, haplotype: No. 4	658	658	93%	0.0	86%	AB223154.1	608,47	87,0%
Select seq dbj|AB223153.1|	Plantago asiatica var. densiuscula genes for 18S rRNA, ITS1, 5.8S rRNA, ITS2, 28S rRNA, partial and complete sequence, haplotype: No. 3	658	658	93%	0.0	86%	AB223153.1	608,47	87,0%
Select seq gb|HM368721.1|	Plantago lanigera isolate 24Blani internal transcribed spacer 1, partial sequence; 5.8S ribosomal RNA gene and internal transcribed spacer 2, complete sequence; and 28S ribosomal RNA gene, partial sequence	636	636	90%	2,00E-178	86%	HM368721.1	607,73	86,9%
Select seq gb|FJ024613.1|	Plantago cunninghamii voucher JR Hosking 2752, WELTU 20186 18S ribosomal RNA gene, partial sequence; internal transcribed spacer 1, 5.8S ribosomal RNA gene, and internal transcribed spacer 2, complete sequence; and 28S ribosomal RNA gene, partial sequence	636	636	90%	2,00E-178	86%	FJ024613.1	607,73	86,9%
Select seq gb|FJ024610.1|	Plantago glacialis voucher BG Briggs 9753, NSW 743813 18S ribosomal RNA gene, partial sequence; internal transcribed spacer 1, 5.8S ribosomal RNA gene, and internal transcribed spacer 2, complete sequence; and 28S ribosomal RNA gene, partial sequence	636	636	90%	2,00E-178	86%	FJ024610.1	607,73	86,9%
Select seq dbj|AB223160.1|	Plantago asiatica var. densiuscula genes for 18S rRNA, ITS1, 5.8S rRNA, ITS2, 28S rRNA, partial and complete sequence, haplotype: No. 10	656	656	93%	0.0	86%	AB223160.1	606,62	86,7%
Select seq dbj|AB223155.1|	Plantago asiatica var. densiuscula genes for 18S rRNA, ITS1, 5.8S rRNA, ITS2, 28S rRNA, partial and complete sequence, haplotype: No. 5	656	656	93%	0.0	86%	AB223155.1	606,62	86,7%
Select seq gb|HM368738.1|	Plantago lanigera isolate 77Alani internal transcribed spacer 1, partial sequence; 5.8S ribosomal RNA gene and internal transcribed spacer 2, complete sequence; and 28S ribosomal RNA gene, partial sequence	627	627	89%	1,00E-175	86%	HM368738.1	605,87	86,6%
Select seq gb|HM368733.1|	Plantago lanigera isolate 63Elani internal transcribed spacer 1, partial sequence; 5.8S ribosomal RNA gene and internal transcribed spacer 2, complete sequence; and 28S ribosomal RNA gene, partial sequence	627	627	89%	1,00E-175	86%	HM368733.1	605,87	86,6%
Select seq gb|HM368720.1|	Plantago lanigera isolate 20Blani internal transcribed spacer 1, partial sequence; 5.8S ribosomal RNA gene and internal transcribed spacer 2, complete sequence; and 28S ribosomal RNA gene, partial sequence	627	627	89%	1,00E-175	86%	HM368720.1	605,87	86,6%
Select seq emb|AJ548966.1|	Plantago euryphylla partial 18S rRNA gene, 5.8S rRNA gene, partial 28S rRNA gene, ITS1 and ITS2, specimen voucher Craven & Craven #10157	654	654	93%	0.0	86%	AJ548966.1	604,77	86,4%
Select seq gb|FJ024593.1|	Plantago lanigera voucher PB Heenan s.n., WELTU 20143 18S ribosomal RNA gene, partial sequence; internal transcribed spacer 1, 5.8S ribosomal RNA gene, and internal transcribed spacer 2, complete sequence; and 28S ribosomal RNA gene, partial sequence	632	632	90%	2,00E-177	86%	FJ024593.1	603,91	86,3%
Select seq dbj|AB223162.1|	Plantago asiatica var. densiuscula genes for 18S rRNA, ITS1, 5.8S rRNA, ITS2, 28S rRNA, partial and complete sequence, haplotype: No. 12	651	651	93%	0.0	86%	AB223162.1	602,00	86,1%
Select seq gb|FJ024605.1|	Plantago aucklandica voucher J Wright s.n., WELTU 20185 18S ribosomal RNA gene, partial sequence; internal transcribed spacer 1, 5.8S ribosomal RNA gene, and internal transcribed spacer 2, complete sequence; and 28S ribosomal RNA gene, partial sequence	630	630	90%	9,00E-177	86%	FJ024605.1	602,00	86,1%
Select seq gb|HM368737.1|	Plantago lanigera isolate 62Dlani internal transcribed spacer 1, partial sequence; 5.8S ribosomal RNA gene and internal transcribed spacer 2, complete sequence; and 28S ribosomal RNA gene, partial sequence	623	623	89%	1,00E-174	86%	HM368737.1	602,00	86,1%
Select seq gb|HM368728.1|	Plantago lanigera isolate 52Elani internal transcribed spacer 1, partial sequence; 5.8S ribosomal RNA gene and internal transcribed spacer 2, complete sequence; and 28S ribosomal RNA gene, partial sequence	623	623	89%	1,00E-174	86%	HM368728.1	602,00	86,1%
Select seq dbj|AB281170.1|	Plantago virginica genes for 18S rRNA, ITS1, 5.8S rRNA, ITS2, 28S rRNA, partial and complete sequence	649	649	93%	0.0	86%	AB281170.1	600,15	85,8%

Select seq emb|AJ548970.1|	Plantago tasmanica partial 18S rRNA gene, 5.8S rRNA gene, partial 28S rRNA gene, ITS1 and ITS2, specimen voucher Burns D #2135	649	649	93%	0.0	86%	AJ548970.1	600,15	85,8%
Select seq emb|AJ548968.1|	Plantago daltonii partial 18S rRNA gene, 5.8S rRNA gene, partial 28S rRNA gene, ITS1 and ITS2, specimen voucher Burns D #2137	649	649	93%	0.0	86%	AJ548968.1	600,15	85,8%
Select seq gb|AY101871.1|	Plantago trinitatis 18S ribosomal RNA gene, partial sequence; internal transcribed spacer 1, 5.8S ribosomal RNA gene, and internal transcribed spacer 2, complete sequence; and 28S ribosomal RNA gene, partial sequence	634	634	91%	7,00E-178	86%	AY101871.1	599,16	85,6%
Select seq gb|FJ024617.1|	Plantago daltonii voucher BG Briggs 9782, NSW 743874 18S ribosomal RNA gene, partial sequence; internal transcribed spacer 1, 5.8S ribosomal RNA gene, and internal transcribed spacer 2, complete sequence; and 28S ribosomal RNA gene, partial sequence	627	627	90%	1,00E-175	86%	FJ024617.1	599,13	85,6%
Select seq gb|FJ024606.1|	Plantago muelleri voucher BG Briggs 9752, NSW 743812 18S ribosomal RNA gene, partial sequence; internal transcribed spacer 1, 5.8S ribosomal RNA gene, and internal transcribed spacer 2, complete sequence; and 28S ribosomal RNA gene, partial sequence	627	627	90%	1,00E-175	86%	FJ024606.1	599,13	85,6%
Select seq gb|FJ024602.1|	Plantago unibracteata voucher ML Tay 053 & PJ Garnock-Jones, WELTU 20173 18S ribosomal RNA gene, partial sequence; internal transcribed spacer 1, 5.8S ribosomal RNA gene, and internal transcribed spacer 2, complete sequence; and 28S ribosomal RNA gene, partial sequence	627	627	90%	1,00E-175	86%	FJ024602.1	599,13	85,6%
Select seq gb|FJ024596.1|	Plantago lanigera voucher M Thorsen s.n., WELTU20133 18S ribosomal RNA gene, partial sequence; internal transcribed spacer 1, 5.8S ribosomal RNA gene, and internal transcribed spacer 2, complete sequence; and 28S ribosomal RNA gene, partial sequence	627	627	90%	1,00E-175	86%	FJ024596.1	599,13	85,6%
Select seq gb|FJ024594.1|	Plantago lanigera voucher ML Tay 027 & al., WELTU 20147 18S ribosomal RNA gene, partial sequence; internal transcribed spacer 1, 5.8S ribosomal RNA gene, and internal transcribed spacer 2, complete sequence; and 28S ribosomal RNA gene, partial sequence	619	619	89%	2,00E-173	86%	FJ024594.1	598,13	85,5%
Select seq gb|FJ024611.1|	Plantago alpestris voucher BG Briggs 9748, NSW 742962 18S ribosomal RNA gene, partial sequence; internal transcribed spacer 1, 5.8S ribosomal RNA gene, and internal transcribed spacer 2, complete sequence; and 28S ribosomal RNA gene, partial sequence	625	625	90%	4,00E-175	86%	FJ024611.1	597,22	85,4%
Select seq gb|FJ024595.1|	Plantago lanigera voucher ML Tay 027 & al., WELTU 20147 18S ribosomal RNA gene, partial sequence; internal transcribed spacer 1, 5.8S ribosomal RNA gene, and internal transcribed spacer 2, complete sequence; and 28S ribosomal RNA gene, partial sequence	625	625	90%	4,00E-175	86%	FJ024595.1	597,22	85,4%
Select seq gb|FJ024615.1|	Plantago tasmanica var. tasmanica voucher BG Briggs 9780, WELTU 20188 18S ribosomal RNA gene, partial sequence; internal transcribed spacer 1, 5.8S ribosomal RNA gene, and internal transcribed spacer 2, complete sequence; and 28S ribosomal RNA gene, partial sequence	623	623	90%	1,00E-174	86%	FJ024615.1	595,31	85,1%
Select seq gb|HM368730.1|	Plantago lanigera isolate 53Clani internal transcribed spacer 1, partial sequence; 5.8S ribosomal RNA gene and internal transcribed spacer 2, complete sequence; and 28S ribosomal RNA gene, partial sequence	616	616	89%	2,00E-172	86%	HM368730.1	595,24	85,1%
Select seq gb|HM368764.1|	Plantago unibracteata isolate 43Aunib internal transcribed spacer 1, partial sequence; 5.8S ribosomal RNA gene and internal transcribed spacer 2, complete sequence; and 28S ribosomal RNA gene, partial sequence	621	621	90%	5,00E-174	86%	HM368764.1	593,40	84,8%
Select seq gb|HM368761.1|	Plantago unibracteata isolate 44Bunib internal transcribed spacer 1, partial sequence; 5.8S ribosomal RNA gene and internal transcribed spacer 2, complete sequence; and 28S ribosomal RNA gene, partial sequence	621	621	90%	5,00E-174	86%	HM368761.1	593,40	84,8%
Select seq gb|HM368748.1|	Plantago triandra subsp. triandra isolate 56Btrid internal transcribed spacer 1, partial sequence; 5.8S ribosomal RNA gene and internal transcribed spacer 2, complete sequence; and 28S ribosomal RNA gene, partial sequence	621	621	90%	5,00E-174	86%	HM368748.1	593,40	84,8%
Select seq gb|HM368746.1|	Plantago triandra subsp. masoniae isolate 29AtridM internal transcribed spacer 1, partial sequence; 5.8S ribosomal RNA gene and internal transcribed spacer 2, complete sequence; and 28S ribosomal RNA gene, partial sequence	621	621	90%	5,00E-174	86%	HM368746.1	593,40	84,8%
Select seq gb|HM368724.1|	Plantago lanigera isolate 27lani internal transcribed spacer 1, partial sequence; 5.8S ribosomal RNA gene and internal transcribed spacer 2, complete sequence; and 28S ribosomal RNA gene, partial sequence	614	614	89%	9,00E-172	86%	HM368724.1	593,30	84,8%
Select seq gb|AY101872.1|	Plantago tomentosa 18S ribosomal RNA gene, partial sequence; internal transcribed spacer 1, 5.8S ribosomal RNA gene, and internal transcribed spacer 2, complete sequence; and 28S ribosomal RNA gene, partial sequence	641	641	93%	4,00E-180	86%	AY101872.1	592,75	84,7%
Select seq gb|HM368739.1|	Plantago obconica isolate 42Aobco internal transcribed spacer 1, partial sequence; 5.8S ribosomal RNA gene and internal transcribed spacer 2, complete sequence; and 28S ribosomal RNA gene, partial sequence	619	619	90%	2,00E-173	86%	HM368739.1	591,49	84,5%
Select seq gb|FJ024604.1|	Plantago obconica voucher PJ Garnock-Jones 2600 & al., WELTU 20121 18S ribosomal RNA gene, partial sequence; internal transcribed spacer 1, 5.8S ribosomal RNA gene, and internal transcribed spacer 2, complete sequence; and 28S ribosomal RNA gene, partial sequence	619	619	90%	2,00E-173	86%	FJ024604.1	591,49	84,5%
Select seq gb|FJ024599.1|	Plantago triandra subsp. triandra voucher ML Tay 021 & PJ Garnock-Jones, WELTU 20163 18S ribosomal RNA gene, partial sequence; internal transcribed spacer 1, 5.8S ribosomal RNA gene, and internal transcribed spacer 2, complete sequence; and 28S ribosomal RNA gene, partial sequence	617	617	90%	7,00E-173	86%	FJ024599.1	589,58	84,3%
Select seq gb|HM368765.1|	Plantago unibracteata isolate 57Bunib internal transcribed spacer 1, partial sequence; 5.8S ribosomal RNA gene and internal transcribed spacer 2, complete sequence; and 28S ribosomal RNA gene, partial sequence	616	616	90%	2,00E-172	86%	HM368765.1	588,62	84,1%
Select seq gb|AY101874.1|	Plantago australis voucher IOK 6/94 (C) 18S ribosomal RNA gene, partial sequence; internal transcribed spacer 1, 5.8S ribosomal RNA gene, and internal transcribed spacer 2, complete sequence; and 28S ribosomal RNA gene, partial sequence	636	636	93%	2,00E-178	86%	AY101874.1	588,13	84,1%
Select seq gb|HM368762.1|	Plantago unibracteata isolate 60Aunib internal transcribed spacer 1, partial sequence; 5.8S ribosomal RNA gene and internal transcribed spacer 2, complete sequence; and 28S ribosomal RNA gene, partial sequence	614	614	90%	9,00E-172	86%	HM368762.1	586,71	83,9%
Select seq gb|AY101858.1|	Plantago reniformis 18S ribosomal RNA gene, partial sequence; internal transcribed spacer 1, 5.8S ribosomal RNA gene, and internal transcribed spacer 2, complete sequence; and 28S ribosomal RNA gene, partial sequence	634	634	93%	7,00E-178	86%	AY101858.1	586,28	83,8%
Select seq gb|HM368760.1|	Plantago unibracteata isolate 44Aunib internal transcribed spacer 1, partial sequence; 5.8S ribosomal RNA gene and internal transcribed spacer 2, complete sequence; and 28S ribosomal RNA gene, partial sequence	612	612	90%	3,00E-171	86%	HM368760.1	584,80	83,6%
Select seq emb|AJ548976.1|	Plantago rhodosperma partial 18S rRNA gene, 5.8S rRNA gene, partial 28S rRNA gene, ITS1 and ITS2, specimen voucher Hoggard RK #268	632	632	93%	2,00E-177	86%	AJ548976.1	584,43	83,5%

Select seq gb|HM368687.1|	Plantago raoulii isolate 32Araou internal transcribed spacer 1, partial sequence; 5.8S ribosomal RNA gene and internal transcribed spacer 2, complete sequence; and 28S ribosomal RNA gene, partial sequence	610	610	90%	1,00E-170	86%	HM368687.1	582,89	83,3%
Select seq gb|HM368679.1|	Plantago raoulii isolate 31Fraou internal transcribed spacer 1, partial sequence; 5.8S ribosomal RNA gene and internal transcribed spacer 2, complete sequence; and 28S ribosomal RNA gene, partial sequence	610	610	90%	1,00E-170	86%	HM368679.1	582,89	83,3%
Select seq gb|HM368743.1|	Plantago obconica isolate 80Aobco internal transcribed spacer 1, partial sequence; 5.8S ribosomal RNA gene and internal transcribed spacer 2, complete sequence; and 28S ribosomal RNA gene, partial sequence	614	614	90%	9,00E-172	85%	HM368743.1	579,89	82,9%
Select seq gb|FJ024603.1|	Plantago obconica voucher J Barkla s.n. & N Simpson, CHR573261 18S ribosomal RNA gene, partial sequence; internal transcribed spacer 1, 5.8S ribosomal RNA gene, and internal transcribed spacer 2, complete sequence; and 28S ribosomal RNA gene, partial sequence	614	614	90%	9,00E-172	85%	FJ024603.1	579,89	82,9%
Select seq gb|HM368744.1|	Plantago triandra subsp. masoniae isolate 89AtridM internal transcribed spacer 1, partial sequence; 5.8S ribosomal RNA gene and internal transcribed spacer 2, complete sequence; and 28S ribosomal RNA gene, partial sequence	610	610	90%	1,00E-170	85%	HM368744.1	576,11	82,4%
Select seq gb|AY101860.1|	Plantago palmata 18S ribosomal RNA gene, partial sequence; internal transcribed spacer 1, 5.8S ribosomal RNA gene, and internal transcribed spacer 2, complete sequence; and 28S ribosomal RNA gene, partial sequence	627	627	93%	1,00E-175	85%	AY101860.1	573,06	81,9%
Select seq emb|AJ548979.1|	Plantago sparsiflora partial 18S rRNA gene, 5.8S rRNA gene, partial 28S rRNA gene, ITS1 and ITS2, specimen voucher LeBlond R. #5305	627	627	93%	1,00E-175	85%	AJ548979.1	573,06	81,9%
Select seq emb|AJ548978.1|	Plantago reniformis partial 18S rRNA gene, 5.8S rRNA gene, partial 28S rRNA gene, ITS1 and ITS2, specimen voucher Hoggard RK #416	627	627	93%	1,00E-175	85%	AJ548978.1	573,06	81,9%
Select seq gb|HM368681.1|	Plantago raoulii isolate 33Eraou internal transcribed spacer 1, partial sequence; 5.8S ribosomal RNA gene and internal transcribed spacer 2, complete sequence; and 28S ribosomal RNA gene, partial sequence	606	606	90%	1,00E-169	85%	HM368681.1	572,33	81,8%
Select seq gb|AY101870.1|	Plantago stauntonii 18S ribosomal RNA gene, partial sequence; internal transcribed spacer 1, 5.8S ribosomal RNA gene, and internal transcribed spacer 2, complete sequence; and 28S ribosomal RNA gene, partial sequence	623	623	93%	1,00E-174	85%	AY101870.1	569,41	81,4%
Select seq gb|AY101863.1|	Plantago rugelii 18S ribosomal RNA gene, partial sequence; internal transcribed spacer 1, 5.8S ribosomal RNA gene, and internal transcribed spacer 2, complete sequence; and 28S ribosomal RNA gene, partial sequence	621	621	93%	5,00E-174	85%	AY101863.1	567,58	81,1%
Select seq gb|AY101869.1|	Plantago spathulata 18S ribosomal RNA gene, partial sequence; internal transcribed spacer 1, 5.8S ribosomal RNA gene, and internal transcribed spacer 2, complete sequence; and 28S ribosomal RNA gene, partial sequence	617	617	93%	7,00E-173	85%	AY101869.1	563,92	80,6%
Select seq gb|AY101867.1|	Plantago raoulii 18S ribosomal RNA gene, partial sequence; internal transcribed spacer 1, 5.8S ribosomal RNA gene, and internal transcribed spacer 2, complete sequence; and 28S ribosomal RNA gene, partial sequence	617	617	93%	7,00E-173	85%	AY101867.1	563,92	80,6%
Select seq emb|AJ548967.1|	Plantago hispida partial 18S rRNA gene, 5.8S rRNA gene, partial 28S rRNA gene, ITS1 and ITS2, specimen voucher Craven & Brubaker #10146	617	617	93%	7,00E-173	85%	AJ548967.1	563,92	80,6%
Select seq emb|AJ548965.1|	Plantago triandra partial 18S rRNA gene, 5.8S rRNA gene, partial 28S rRNA gene, ITS1 and ITS2, specimen voucher Burnett D. #052887	614	614	93%	9,00E-172	85%	AJ548965.1	561,18	80,2%
Select seq dbj|AB281169.1|	Plantago camtschatica genes for 18S rRNA, ITS1, 5.8S rRNA, ITS2, 28S rRNA, partial and complete sequence	612	612	93%	3,00E-171	85%	AB281169.1	559,35	80,0%
Select seq gb|AY101868.1|	Plantago debilis 18S ribosomal RNA gene, partial sequence; internal transcribed spacer 1, 5.8S ribosomal RNA gene, and internal transcribed spacer 2, complete sequence; and 28S ribosomal RNA gene, partial sequence	612	612	93%	3,00E-171	85%	AY101868.1	559,35	80,0%
Select seq emb|AJ548969.1|	Plantago paradoxa partial 18S rRNA gene, 5.8S rRNA gene, partial 28S rRNA gene, ITS1 and ITS2, specimen voucher Burns D #2136	612	612	93%	3,00E-171	85%	AJ548969.1	559,35	

Select for downloading or viewing reports	Kh039_trnL Description	Max score	Total score	Query cover	E value	Ident	Accession	(Ident/Cover)* Max score	Deviation from top hit
Select seq gb|AY101917.1|	Plantago major tRNA-Leu (trnL) gene, partial sequence; and trnL-trnF intergenic spacer region; chloroplast genes for chloroplast products	1339	1339	99%	0.0	99%	AY101917.1	1339,00	100,0%
Select seq gb|AY101915.1|	Plantago cornuti tRNA-Leu (trnL) gene, partial sequence; and trnL-trnF intergenic spacer region; chloroplast genes for chloroplast products	1336	1336	99%	0.0	99%	AY101915.1	1336,00	99,8%
Select seq gb|AY101916.1|	Plantago palmata tRNA-Leu (trnL) gene, partial sequence; and trnL-trnF intergenic spacer region; chloroplast genes for chloroplast products	1319	1319	99%	0.0	99%	AY101916.1	1319,00	98,5%
Select seq gb|AY101923.1|	Plantago raoulii tRNA-Leu (trnL) gene, partial sequence; and trnL-trnF intergenic spacer region; chloroplast genes for chloroplast products	1317	1317	99%	0.0	99%	AY101923.1	1317,00	98,4%
Select seq gb|AY101919.1|	Plantago rugelii tRNA-Leu (trnL) gene, partial sequence; and trnL-trnF intergenic spacer region; chloroplast genes for chloroplast products	1317	1317	99%	0.0	99%	AY101919.1	1317,00	98,4%
Select seq gb|AY101925.1|	Plantago stauntonii tRNA-Leu (trnL) gene, partial sequence; and trnL-trnF intergenic spacer region; chloroplast genes for chloroplast products	1314	1314	99%	0.0	99%	AY101925.1	1314,00	98,1%
Select seq gb|AY101930.1|	Plantago uniglumis tRNA-Leu (trnL) gene, partial sequence; and trnL-trnF intergenic spacer region; chloroplast genes for chloroplast products	1306	1306	99%	0.0	99%	AY101930.1	1306,00	97,5%
Select seq gb|AY101922.1|	Plantago debilis tRNA-Leu (trnL) gene, partial sequence; and trnL-trnF intergenic spacer region; chloroplast genes for chloroplast products	1306	1306	99%	0.0	99%	AY101922.1	1306,00	97,5%
Select seq gb|AY101924.1|	Plantago spathulata tRNA-Leu (trnL) gene, partial sequence; and trnL-trnF intergenic spacer region; chloroplast genes for chloroplast products	1301	1301	99%	0.0	98%	AY101924.1	1287,86	96,2%
Select seq gb|AY101929.1|	Plantago australis tRNA-Leu (trnL) gene, partial sequence; and trnL-trnF intergenic spacer region; chloroplast genes for chloroplast products	1293	1293	99%	0.0	98%	AY101929.1	1279,94	95,6%
Select seq gb|AY101928.1|	Plantago myosuros tRNA-Leu (trnL) gene, partial sequence; and trnL-trnF intergenic spacer region; chloroplast genes for chloroplast products	1288	1288	99%	0.0	98%	AY101928.1	1274,99	95,2%
Select seq gb|AY101927.1|	Plantago tomentosa tRNA-Leu (trnL) gene, partial sequence; and trnL-trnF intergenic spacer region; chloroplast genes for chloroplast products	1288	1288	99%	0.0	98%	AY101927.1	1274,99	95,2%
Select seq gb|AY101926.1|	Plantago trinitatis tRNA-Leu (trnL) gene, partial sequence; and trnL-trnF intergenic spacer region; chloroplast genes for chloroplast products	1288	1288	99%	0.0	98%	AY101926.1	1274,99	95,2%
Select seq gb|AY101918.1|	Plantago asiatica tRNA-Leu (trnL) gene, partial sequence; and trnL-trnF intergenic spacer region; chloroplast genes for chloroplast products	1288	1288	99%	0.0	98%	AY101918.1	1274,99	95,2%
Select seq gb|AY101931.1|	Plantago rigida tRNA-Leu (trnL) gene, partial sequence; and trnL-trnF intergenic spacer region; chloroplast genes for chloroplast products	1273	1273	99%	0.0	98%	AY101931.1	1260,14	94,1%
Select seq gb|KR297245.1|	Plantago media plastid, complete genome	1256	1256	99%	0.0	97%	KR297245.1	1230,63	91,9%
Select seq gb|AY101920.1|	Plantago media tRNA-Leu (trnL) gene, partial sequence; and trnL-trnF intergenic spacer region; chloroplast genes for chloroplast products	1253	1253	99%	0.0	97%	AY101920.1	1227,69	91,7%
Select seq gb|AY101921.1|	Plantago tenuiflora tRNA-Leu (trnL) gene, partial sequence; and trnL-trnF intergenic spacer region; chloroplast genes for chloroplast products	1210	1210	99%	0.0	96%	AY101921.1	1173,33	87,6%
Select seq gb|KJ579148.1|	Plantago algarbiensis tRNA-Leu (trnL) gene and trnL-F intergenic spacer, partial sequence; chloroplast	1098	1098	99%	0.0	94%	KJ579148.1	1042,55	77,9%
Select seq gb|KJ579147.1|	Plantago radicata subsp. monticola tRNA-Leu (trnL) gene, partial sequence; and trnL-F intergenic spacer, complete sequence; chloroplast	1098	1098	99%	0.0	94%	KJ579147.1	1042,55	77,9%
Select seq gb|AY101933.1|	Plantago subulata tRNA-Leu (trnL) gene, partial sequence; and trnL-trnF intergenic spacer region; chloroplast genes for chloroplast products	1094	1094	99%	0.0	94%	AY101933.1	1038,75	77,6%
Select seq gb|KJ579149.1|	Plantago almogravensis tRNA-Leu (trnL) gene and trnL-F intergenic spacer, partial sequence; chloroplast	1092	1092	99%	0.0	94%	KJ579149.1	1036,85	77,4%
Select seq gb|KJ579146.1|	Plantago maritima subsp. maritima tRNA-Leu (trnL) gene and trnL-F intergenic spacer, partial sequence; chloroplast	1090	1090	99%	0.0	94%	KJ579146.1	1034,95	77,3%
Select seq gb|KR297244.1|	Plantago maritima plastid, complete genome	1090	1090	99%	0.0	94%	KR297244.1	1034,95	77,3%
Select seq gb|AY101932.1|	Plantago alpina tRNA-Leu (trnL) gene, partial sequence; and trnL-trnF intergenic spacer region; chloroplast genes for chloroplast products	1090	1090	99%	0.0	94%	AY101932.1	1034,95	77,3%
Select seq gb|AY101934.1|	Plantago maritima tRNA-Leu (trnL) gene, partial sequence; and trnL-trnF intergenic spacer region; chloroplast genes for chloroplast products	1085	1085	99%	0.0	94%	AY101934.1	1030,20	76,9%

Select seq gb|AY101952.1|	Plantago lanceolata tRNA-Leu (trnL) gene, partial sequence; and trnL-trnF intergenic spacer region; chloroplast genes for chloroplast products	990	990	98%	0.0	91%	AY101952.1	919,29	68,7%
Select seq gb|AY101953.1|	Plantago leiopetala tRNA-Leu (trnL) gene, partial sequence; and trnL-trnF intergenic spacer region; chloroplast genes for chloroplast products	985	985	98%	0.0	91%	AY101953.1	914,64	68,3%
Select seq emb|AJ430931.1|	Plantago argentea chloroplast tRNA-Leu gene for transfer RNA-Leu and partial tRNA-Phe gene for transfer RNA-Phe	985	985	98%	0.0	91%	AJ430931.1	914,64	68,3%
Select seq gb|AY101950.1|	Plantago nivalis tRNA-Leu (trnL) gene, partial sequence; and trnL-trnF intergenic spacer region; chloroplast genes for chloroplast products	959	959	99%	0.0	91%	AY101950.1	881,51	65,8%
Select seq gb|AY101948.1|	Plantago nubicola tRNA-Leu (trnL) gene, partial sequence; and trnL-trnF intergenic spacer region; chloroplast genes for chloroplast products	957	957	99%	0.0	90%	AY101948.1	870,00	65,0%
Select seq gb|AY101951.1|	Plantago lagopus tRNA-Leu (trnL) gene, partial sequence; and trnL-trnF intergenic spacer region; chloroplast genes for chloroplast products	942	942	98%	0.0	90%	AY101951.1	865,10	64,6%
Select seq gb|AY101957.1|	Plantago ovata tRNA-Leu (trnL) gene, partial sequence; and trnL-trnF intergenic spacer region; chloroplast genes for chloroplast products	917	917	99%	0.0	90%	AY101957.1	833,64	62,3%
Select seq gb|AY101941.1|	Plantago webbii tRNA-Leu (trnL) gene, partial sequence; and trnL-trnF intergenic spacer region; chloroplast genes for chloroplast products	913	913	99%	0.0	89%	AY101941.1	820,78	61,3%
Select seq gb|KJ579145.1|	Plantago arborescens tRNA-Leu (trnL) gene and trnL-F intergenic spacer, partial sequence; chloroplast	885	885	99%	0.0	89%	KJ579145.1	795,61	59,4%
Select seq emb|FN794064.1|	Littorella uniflora chloroplast partial tRNA-Leu gene for transfer RNA-Leu and partial trnL-trnF intergenic spacer, isolate LE162	881	881	99%	0.0	89%	FN794064.1	792,01	59,1%
Select seq gb|AY101940.1|	Plantago uniflora tRNA-Leu (trnL) gene, partial sequence; and trnL-trnF intergenic spacer region; chloroplast genes for chloroplast products	881	881	99%	0.0	89%	AY101940.1	792,01	59,1%
Select seq gb|AY101914.1|	Plantago reniformis tRNA-Leu (trnL) gene, partial sequence; and trnL-trnF intergenic spacer region; chloroplast genes for chloroplast products	778	1259	97%	0.0	98%	AY101914.1	786,02	58,7%
Select seq gb|AY101958.1|	Plantago albicans tRNA-Leu (trnL) gene, partial sequence; and trnL-trnF intergenic spacer region; chloroplast genes for chloroplast products	865	865	99%	0.0	89%	AY101958.1	777,63	58,1%
Select seq gb|AY101946.1|	Plantago sarcophylla tRNA-Leu (trnL) gene, partial sequence; and trnL-trnF intergenic spacer region; chloroplast genes for chloroplast products	874	874	99%	0.0	88%	AY101946.1	776,89	58,0%
Select seq gb|AY101947.1|	Plantago squarrosa tRNA-Leu (trnL) gene, partial sequence; and trnL-trnF intergenic spacer region; chloroplast genes for chloroplast products	869	869	99%	0.0	88%	AY101947.1	772,44	57,7%
Select seq gb|AY101943.1|	Plantago mauritanica tRNA-Leu (trnL) gene, partial sequence; and trnL-trnF intergenic spacer region; chloroplast genes for chloroplast products	865	865	99%	0.0	88%	AY101943.1	768,89	57,4%
Select seq gb|AY101949.1|	Plantago atrata tRNA-Leu (trnL) gene, partial sequence; and trnL-trnF intergenic spacer region; chloroplast genes for chloroplast products	859	859	99%	0.0	88%	AY101949.1	763,56	57,0%
Select seq gb|AY101942.1|	Plantago sempervirens tRNA-Leu (trnL) gene, partial sequence; and trnL-trnF intergenic spacer region; chloroplast genes for chloroplast products	850	850	99%	0.0	88%	AY101942.1	755,56	56,4%
Select seq gb|AY101944.1|	Plantago arenaria tRNA-Leu (trnL) gene, partial sequence; and trnL-trnF intergenic spacer region; chloroplast genes for chloroplast products	830	830	99%	0.0	88%	AY101944.1	737,78	55,1%
Select seq gb|AY101956.1|	Plantago bellardii tRNA-Leu (trnL) gene, partial sequence; and trnL-trnF intergenic spacer region; chloroplast genes for chloroplast products	839	839	99%	0.0	87%	AY101956.1	737,30	55,1%
Select seq gb|AY101955.1|	Plantago cretica tRNA-Leu (trnL) gene, partial sequence; and trnL-trnF intergenic spacer region; chloroplast genes for chloroplast products	839	839	99%	0.0	87%	AY101955.1	737,30	55,1%
Select seq gb|AY101945.1|	Plantago afra tRNA-Leu (trnL) gene, partial sequence; and trnL-trnF intergenic spacer region; chloroplast genes for chloroplast products	821	821	99%	0.0	87%	AY101945.1	721,48	53,9%
Select seq gb|AF486412.1|	Veronicastrum virginicum tRNA-Leu (trnL) gene and trnL-trnF intergenic spacer, partial sequence; chloroplast	795	795	99%	0.0	87%	AF486412.1	698,64	52,2%
Select seq gb|EU233624.1|	Veronicastrum axillare tRNA-Leu (trnL) gene and trnL-trnF intergenic spacer, partial sequence; chloroplast	791	791	99%	0.0	87%	EU233624.1	695,12	51,9%
Select seq gb|AF513354.1|	Veronicastrum stenostachyum chloroplast tRNA-Leu (trnL) gene and trnL-trnF intergenic spacer, partial sequence	791	791	99%	0.0	87%	AF513354.1	695,12	51,9%
Select seq gb|AF486413.1|	Veronicastrum liukiuense tRNA-Leu (trnL) gene and trnL-trnF intergenic spacer, partial sequence; chloroplast	789	789	99%	0.0	87%	AF486413.1	693,36	51,8%
Select seq gb|AF486418.1|	Digitalis obscura tRNA-Leu (trnL) gene and trnL-trnF intergenic spacer, partial sequence; chloroplast	780	780	99%	0.0	86%	AF486418.1	677,58	50,6%

Select seq gb|KC413606.1|	Neopicrorhiza scrophulariiflora voucher SunH11131 (KUN) tRNA-Leu (trnL) gene and trnL-trnF intergenic spacer, partial sequence; chloroplast	776	776	99% 0.0	86% KC413606.1	674,10	50,3%
Select seq gb|AF486414.1|	Picrorhiza kurrooa tRNA-Leu (trnL) gene and trnL-trnF intergenic spacer, partial sequence; chloroplast	776	776	99% 0.0	86% AF486414.1	674,10	50,3%
Select seq gb|AY591308.1|	Digitalis obscura subsp. laciniata tRNA-Leu (trnL) gene and trnL-F intergenic spacer, partial sequence; chloroplast	774	774	99% 0.0	86% AY591308.1	672,36	50,2%
Select seq gb|AY591316.1|	Digitalis cariensis subsp. trojana tRNA-Leu (trnL) gene and trnL-F intergenic spacer, partial sequence; chloroplast	769	769	99% 0.0	86% AY591316.1	668,02	49,9%
Select seq gb|AY591302.1|	Isoplexis sceptrum tRNA-Leu (trnL) gene and trnL-F intergenic spacer, partial sequence; chloroplast	769	769	99% 0.0	86% AY591302.1	668,02	49,9%
Select seq gb|AY591313.1|	Digitalis ferruginea subsp. ferruginea tRNA-Leu (trnL) gene and trnL-F intergenic spacer, partial sequence; chloroplast	763	763	99% 0.0	86% AY591313.1	662,81	49,5%
Select seq gb|AY591309.1|	Digitalis subalpina var. subalpina tRNA-Leu (trnL) gene and trnL-F intergenic spacer, partial sequence; chloroplast	763	763	99% 0.0	86% AY591309.1	662,81	49,5%
Select seq gb|AY591291.1|	Digitalis purpurea subsp. purpurea tRNA-Leu (trnL) gene and trnL-F intergenic spacer, partial sequence; chloroplast	763	763	99% 0.0	86% AY591291.1	662,81	49,5%
Select seq gb|KC413607.1|	Scrofella chinensis voucher 39522 (KUN) tRNA-Leu (trnL) gene and trnL-trnF intergenic spacer, partial sequence; chloroplast	760	760	99% 0.0	86% KC413607.1	660,20	49,3%
Select seq gb|AY591296.1|	Digitalis viridiflora tRNA-Leu (trnL) gene and trnL-F intergenic spacer, partial sequence; chloroplast	758	758	99% 0.0	86% AY591296.1	658,46	49,2%
Select seq gb|AY591290.1|	Digitalis thapsi tRNA-Leu (trnL) gene and trnL-F intergenic spacer, partial sequence; chloroplast	758	758	99% 0.0	86% AY591290.1	658,46	49,2%
Select seq gb|AY591307.1|	Digitalis obscura subsp. obscura tRNA-Leu (trnL) gene and trnL-F intergenic spacer, partial sequence; chloroplast	719	719	94% 0.0	86% AY591307.1	657,81	49,1%
Select seq gb|AY316711.1|	Digitalis purpurea chloroplast tRNA-Leu (trnL) gene and trnL-trnF intergenic spacer region, partial sequence	756	756	99% 0.0	86% AY316711.1	656,73	49,0%
Select seq gb|AY591303.1|	Isoplexis chalcantha tRNA-Leu (trnL) gene and trnL-F intergenic spacer, partial sequence; chloroplast	754	754	99% 0.0	86% AY591303.1	654,99	48,9%
Select seq gb|AY591297.1|	Digitalis atlantica tRNA-Leu (trnL) gene and trnL-F intergenic spacer, partial sequence; chloroplast	754	754	99% 0.0	86% AY591297.1	654,99	48,9%
Select seq gb|AY591289.1|	Digitalis minor tRNA-Leu (trnL) gene and trnL-F intergenic spacer, partial sequence; chloroplast	754	754	99% 0.0	86% AY591289.1	654,99	48,9%
Select seq emb|AJ608589.1|	Isoplexis canariensis partial chloroplast tRNA-Leu (UAA) gene for transfer RNA-Leu (UAA), specimen voucher Thulin 9945 (UPS)	754	754	99% 0.0	86% AJ608589.1	654,99	48,9%
Select seq gb|AF486410.1|	Wulfenia orientalis tRNA-Leu (trnL) gene and trnL-trnF intergenic spacer, partial sequence; chloroplast	749	749	99% 0.0	86% AF486410.1	650,65	48,6%
Select seq gb|AY591298.1|	Digitalis ciliata tRNA-Leu (trnL) gene and trnL-F intergenic spacer, partial sequence; chloroplast	749	749	99% 0.0	86% AY591298.1	650,65	48,6%
Select seq gb|KC413583.1|	Lagotis glauca voucher US1132629 (US) tRNA-Leu (trnL) gene and trnL-trnF intergenic spacer, partial sequence; chloroplast	747	747	99% 0.0	86% KC413583.1	648,91	48,5%
Select seq gb|KC413568.1|	Lagotis alutacea voucher SunH11160 (KUN) tRNA-Leu (trnL) gene and trnL-trnF intergenic spacer, partial sequence; chloroplast	741	741	99% 0.0	86% KC413568.1	643,70	48,1%
Select seq gb|AY591301.1|	Digitalis davisiana tRNA-Leu (trnL) gene and trnL-F intergenic spacer, partial sequence; chloroplast	737	737	99% 0.0	86% AY591301.1	640,22	47,8%
Select seq gb|KC413595.1|	Lagotis integrifolia voucher SunH11166 (KUN) tRNA-Leu (trnL) gene and trnL-trnF intergenic spacer, partial sequence; chloroplast	736	736	99% 0.0	86% KC413595.1	639,35	47,7%
Select seq gb|KC413588.1|	Lagotis integra voucher SunH11045 (KUN) tRNA-Leu (trnL) gene and trnL-trnF intergenic spacer, partial sequence; chloroplast	736	736	99% 0.0	86% KC413588.1	639,35	47,7%
Select seq gb|KC413569.1|	Lagotis alutacea voucher SunH11161 (KUN) tRNA-Leu (trnL) gene and trnL-trnF intergenic spacer, partial sequence; chloroplast	736	736	99% 0.0	86% KC413569.1	639,35	47,7%
Select seq gb|EU233626.1|	Lagotis brevituba tRNA-Leu (trnL) gene and trnL-trnF intergenic spacer, partial sequence; chloroplast	736	736	99% 0.0	86% EU233626.1	639,35	47,7%
Select seq gb|KC413577.1|	Lagotis brevituba voucher SunH11029 (KUN) tRNA-Leu (trnL) gene and trnL-trnF intergenic spacer, partial sequence; chloroplast	732	732	99% 0.0	86% KC413577.1	635,88	47,5%

Select seq gb|KC413570.1|	Lagotis angustibracteata voucher SunH11048 (KUN) tRNA-Leu (trnL) gene and trnL-trnF intergenic spacer, partial sequence; chloroplast	732	732	99% 0.0	86% KC413570.1	635,88	47,5%
Select seq gb|KC413601.1|	Lagotis praecox voucher SunH11059 (KUN) tRNA-Leu (trnL) gene and trnL-trnF intergenic spacer, partial sequence; chloroplast	730	730	99% 0.0	85% KC413601.1	626,77	46,8%
Select seq gb|KC413597.1|	Lagotis kongboensis voucher HNWP31184 (HNWP) tRNA-Leu (trnL) gene and trnL-trnF intergenic spacer, partial sequence; chloroplast	730	730	99% 0.0	85% KC413597.1	626,77	46,8%
Select seq gb|KC413585.1|	Lagotis globosa voucher HNWP154258 (HNWP) tRNA-Leu (trnL) gene and trnL-trnF intergenic spacer, partial sequence; chloroplast	730	730	99% 0.0	85% KC413585.1	626,77	46,8%
Select seq gb|AF486408.1|	Paederota lutea tRNA-Leu (trnL) gene and trnL-trnF intergenic spacer, partial sequence; chloroplast	728	728	99% 0.0	85% AF486408.1	625,05	46,7%
Select seq gb|KC413582.1|	Lagotis decumbens voucher HNWP154256 (HNWP) tRNA-Leu (trnL) gene and trnL-trnF intergenic spacer, partial sequence; chloroplast	726	726	99% 0.0	85% KC413582.1	623,33	46,6%
Select seq gb|KC413576.1|	Lagotis brevituba voucher SunH11007 (KUN) tRNA-Leu (trnL) gene and trnL-trnF intergenic spacer, partial sequence; chloroplast	726	726	99% 0.0	85% KC413576.1	623,33	46,6%
Select seq gb|AF486416.1|	Lagotis angustibracteata tRNA-Leu (trnL) gene and trnL-trnF intergenic spacer, partial sequence; chloroplast	726	726	99% 0.0	85% AF486416.1	623,33	46,6%
Select seq gb|KC413603.1|	Lagotis ramalana voucher HNWP41825 (HNWP) tRNA-Leu (trnL) gene and trnL-trnF intergenic spacer, partial sequence; chloroplast	725	725	99% 0.0	85% KC413603.1	622,47	46,5%
Select seq gb|KC413580.1|	Lagotis clarkei voucher SunH11103 (KUN) tRNA-Leu (trnL) gene and trnL-trnF intergenic spacer, partial sequence; chloroplast	725	725	99% 0.0	85% KC413580.1	622,47	46,5%
Select seq gb|KC413579.1|	Lagotis cashmeriana voucher US1579974 (US) tRNA-Leu (trnL) gene and trnL-trnF intergenic spacer, partial sequence; chloroplast	725	725	99% 0.0	85% KC413579.1	622,47	46,5%
Select seq gb|AY591311.1|	Digitalis lutea subsp. australis tRNA-Leu (trnL) gene and trnL-F intergenic spacer, partial sequence; chloroplast	725	725	99% 0.0	85% AY591311.1	622,47	46,5%
Select seq gb|AF513356.1|	Lafuentea rotundifolia chloroplast tRNA-Leu (trnL) gene and trnL-trnF intergenic spacer, partial sequence	725	725	99% 0.0	85% AF513356.1	622,47	46,5%
Select seq gb|AF513353.1|	Paederota bonarota chloroplast tRNA-Leu (trnL) gene and trnL-trnF intergenic spacer, partial sequence	723	723	99% 0.0	85% AF513353.1	620,76	46,4%
Select seq gb|KC413578.1|	Lagotis brevituba voucher SunH11031 (KUN) tRNA-Leu (trnL) gene and trnL-trnF intergenic spacer, partial sequence; chloroplast	721	721	99% 0.0	85% KC413578.1	619,04	46,2%
Select seq gb|AY591295.1|	Digitalis grandiflora tRNA-Leu (trnL) gene and trnL-F intergenic spacer, partial sequence; chloroplast	721	721	99% 0.0	85% AY591295.1	619,04	46,2%
Select seq gb|KC413598.1|	Lagotis korolkowii voucher US2315506 (US) tRNA-Leu (trnL) gene and trnL-trnF intergenic spacer, partial sequence; chloroplast	719	719	99% 0.0	85% KC413598.1	617,32	46,1%
Select seq gb|AY591320.1|	Digitalis parviflora tRNA-Leu (trnL) gene and trnL-F intergenic spacer, partial sequence; chloroplast	713	713	99% 0.0	85% AY591320.1	612,17	45,7%
Select seq gb|AF486415.1|	Lagotis stolonifera tRNA-Leu (trnL) gene and trnL-trnF intergenic spacer, partial sequence; chloroplast	712	712	99% 0.0	85% AF486415.1	611,31	

Select for downloading or viewing reports	Kh044_ITS Description	Max score	Total score	Query cover	E value	Ident	Accession	(Ident/Cover)* Max score	Deviation from top hit
Select seq gb|KC575608.1|	Berberis vulgaris voucher Harber EU3 internal transcribed spacer 1, partial sequence; 5.8S ribosomal RNA gene and internal transcribed spacer 2, complete sequence; and 28S ribosomal RNA gene, partial sequence	1122	1122	89%	0.0	99%	KC575608.1	1248,07	100,0%
Select seq gb|KC575595.1|	Berberis maderensis voucher Harber EU5 internal transcribed spacer 1, partial sequence; 5.8S ribosomal RNA gene and internal transcribed spacer 2, complete sequence; and 28S ribosomal RNA gene, partial sequence	1138	1138	91%	0.0	99%	KC575595.1	1238,04	99,2%
Select seq gb|KC575581.1|	Berberis orthobotrys var. orthobotrys voucher RBGE-19810556 internal transcribed spacer 1, partial sequence; 5.8S ribosomal RNA gene and internal transcribed spacer 2, complete sequence; and 28S ribosomal RNA gene, partial sequence	1138	1138	91%	0.0	99%	KC575581.1	1238,04	99,2%
Select seq gb|EF488082.1|	Berberis vulgaris internal transcribed spacer 1, partial sequence; 5.8S ribosomal RNA gene, complete sequence; and internal transcribed spacer 2, partial sequence	1149	1149	92%	0.0	99%	EF488082.1	1236,42	99,1%
Select seq gb|KC575590.1|	Berberis canadensis voucher Chase 13196 (K) internal transcribed spacer 1, partial sequence; 5.8S ribosomal RNA gene and internal transcribed spacer 2, complete sequence; and 28S ribosomal RNA gene, partial sequence	1160	1160	93%	0.0	99%	KC575590.1	1234,84	98,9%
Select seq gb|JN228269.1|	Berberis sp. 3 ARB-2011 18S ribosomal RNA gene, partial sequence; internal transcribed spacer 1 and 5.8S ribosomal RNA gene, complete sequence; and internal transcribed spacer 2, partial sequence	1197	1197	96%	0.0	99%	JN228269.1	1234,41	98,9%
Select seq gb|KC575607.1|	Berberis amurensis var. japonica voucher RBGE-19920784A internal transcribed spacer 1, partial sequence; 5.8S ribosomal RNA gene and internal transcribed spacer 2, complete sequence; and 28S ribosomal RNA gene, partial sequence	1134	1134	91%	0.0	99%	KC575607.1	1233,69	98,8%
Select seq gb|KC575601.1|	Berberis integerrima voucher RBGE-19912970 internal transcribed spacer 1, partial sequence; 5.8S ribosomal RNA gene and internal transcribed spacer 2, complete sequence; and 28S ribosomal RNA gene, partial sequence	1134	1134	91%	0.0	99%	KC575601.1	1233,69	98,8%
Select seq gb|KC575593.1|	Berberis aetnensis voucher Harber EU2 internal transcribed spacer 1, partial sequence; 5.8S ribosomal RNA gene and internal transcribed spacer 2, complete sequence; and 28S ribosomal RNA gene, partial sequence	1134	1134	91%	0.0	99%	KC575593.1	1233,69	98,8%
Select seq gb|AF328969.1|AF328969	Berberis silva-taroucana internal transcribed spacer 1, 5.8S ribosomal RNA, internal transcribed spacer 2, complete sequence	1133	1133	91%	0.0	99%	AF328969.1	1232,60	98,8%
Select seq gb|KC575600.1|	Berberis heteropoda voucher Harber AS95 18S ribosomal RNA gene, partial sequence; internal transcribed spacer 1, 5.8S ribosomal RNA gene, and internal transcribed spacer 2, complete sequence; and 28S ribosomal RNA gene, partial sequence	1153	1153	93%	0.0	99%	KC575600.1	1227,39	98,3%
Select seq gb|KC575592.1|	Berberis subsessiliflora voucher Harber AS86 internal transcribed spacer 1, partial sequence; 5.8S ribosomal RNA gene and internal transcribed spacer 2, complete sequence; and 28S ribosomal RNA gene, partial sequence	1153	1153	93%	0.0	99%	KC575592.1	1227,39	98,3%
Select seq gb|KC575594.1|	Berberis garciae voucher Harber EU4 internal transcribed spacer 1, partial sequence; 5.8S ribosomal RNA gene and internal transcribed spacer 2, complete sequence; and 28S ribosomal RNA gene, partial sequence	1127	1127	91%	0.0	99%	KC575594.1	1226,08	98,2%
Select seq gb|KC575588.1|	Berberis petiolaris voucher JRS A122 (E) internal transcribed spacer 1, partial sequence; 5.8S ribosomal RNA gene and internal transcribed spacer 2, complete sequence; and 28S ribosomal RNA gene, partial sequence	1099	1099	89%	0.0	99%	KC575588.1	1222,48	98,0%
Select seq gb|KC575602.1|	Berberis integerrima voucher Rae, Gardner & Ogar, 11 (E) internal transcribed spacer 1, partial sequence; 5.8S ribosomal RNA gene and internal transcribed spacer 2, complete sequence; and 28S ribosomal RNA gene, partial sequence	1123	1123	91%	0.0	99%	KC575602.1	1221,73	97,9%
Select seq gb|KR150186.1|	Berberis sp. Kh116 internal transcribed spacer 1, partial sequence; 5.8S ribosomal RNA gene gene, complete sequence; and internal transcribed spacer 2 region, partial sequence	1195	1195	97%	0.0	99%	KR150186.1	1219,64	97,7%
Select seq gb|JN228268.1|	Berberis integerrima x Berberis vulgaris 18S ribosomal RNA gene, partial sequence; internal transcribed spacer 1 and 5.8S ribosomal RNA gene, complete sequence; and internal transcribed spacer 2, partial sequence	1144	1144	93%	0.0	99%	JN228268.1	1217,81	97,6%
Select seq gb|GU934703.1|	Berberis pachyacantha isolate B180 internal transcribed spacer 1, partial sequence; 5.8S ribosomal RNA gene, complete sequence; and internal transcribed spacer 2, partial sequence	1092	1092	89%	0.0	99%	GU934703.1	1214,70	97,3%
Select seq gb|JN228267.1|	Berberis integerrima 18S ribosomal RNA gene, partial sequence; internal transcribed spacer 1, 5.8S ribosomal RNA gene, and internal transcribed spacer 2, complete sequence; and 26S ribosomal RNA gene, partial sequence	1177	1177	96%	0.0	99%	JN228267.1	1213,78	97,3%
Select seq gb|KC575598.1|	Berberis minutiflora voucher Harber AS62 18S ribosomal RNA gene, partial sequence; internal transcribed spacer 1, 5.8S ribosomal RNA gene, and internal transcribed spacer 2, complete sequence; and 28S ribosomal RNA gene, partial sequence	1140	1140	93%	0.0	99%	KC575598.1	1213,55	97,2%
Select seq gb|KC575604.1|	Berberis gyalaica voucher Harber AS6 18S ribosomal RNA gene, partial sequence; internal transcribed spacer 1, 5.8S ribosomal RNA gene, and internal transcribed spacer 2, complete sequence; and 28S ribosomal RNA gene, partial sequence	1164	1164	95%	0.0	99%	KC575604.1	1213,01	97,2%
Select seq gb|KC575603.1|	Berberis prattii voucher RBGE-19687172 18S ribosomal RNA gene, partial sequence; internal transcribed spacer 1, 5.8S ribosomal RNA gene, and internal transcribed spacer 2, complete sequence; and 28S ribosomal RNA gene, partial sequence	1175	1175	96%	0.0	99%	KC575603.1	1211,72	97,1%
Select seq gb|JN012238.1|	Berberis weiningensis voucher WN2-12 18S ribosomal RNA gene, partial sequence; internal transcribed spacer 1, 5.8S ribosomal RNA gene, and internal transcribed spacer, complete sequence; and 28S ribosomal RNA gene, partial sequence	1199	1199	98%	0.0	99%	JN012238.1	1211,23	97,0%
Select seq gb|JN012237.1|	Berberis weiningensis voucher WN1-1 18S ribosomal RNA gene, partial sequence; internal transcribed spacer 1, 5.8S ribosomal RNA gene, and internal transcribed spacer, complete sequence; and 28S ribosomal RNA gene, partial sequence	1199	1199	98%	0.0	99%	JN012237.1	1211,23	97,0%
Select seq gb|GU934610.1|	Berberis angulosa isolate B121 internal transcribed spacer 1, partial sequence; 5.8S ribosomal RNA gene, complete sequence; and internal transcribed spacer 2, partial sequence	1086	1086	89%	0.0	99%	GU934610.1	1208,02	96,8%
Select seq gb|GU934707.1|	Berberis pachyacantha isolate B184 internal transcribed spacer 1, partial sequence; 5.8S ribosomal RNA gene, complete sequence; and internal transcribed spacer 2, partial sequence	1085	1085	89%	0.0	99%	GU934707.1	1206,91	96,7%

Select seq gb|KC575609.1|	Berberis kawakamii voucher RBGE-19933979B internal transcribed spacer 1, partial sequence; 5.8S ribosomal RNA gene and internal transcribed spacer 2, complete sequence; and 28S ribosomal RNA gene, partial sequence	1107	1107	91%	0.0	99%	KC575609.1	1204,32	96,5%
Select seq gb|KC575596.1|	Berberis tsarongensis voucher RBGE-19310185 18S ribosomal RNA gene, partial sequence; internal transcribed spacer 1, 5.8S ribosomal RNA gene, and internal transcribed spacer 2, complete sequence; and 28S ribosomal RNA gene, partial sequence	1155	1155	95%	0.0	99%	KC575596.1	1203,63	96,4%
Select seq gb|HM347895.1|	Berberis angulosa clone 4 internal transcribed spacer 1, 5.8S ribosomal RNA gene, and internal transcribed spacer 2, complete sequence	1081	1081	89%	0.0	99%	HM347895.1	1202,46	96,3%
Select seq gb|HM347899.1|	Berberis angulosa clone 8 internal transcribed spacer 1, 5.8S ribosomal RNA gene, and internal transcribed spacer 2, complete sequence	1081	1081	89%	0.0	99%	HM347899.1	1202,46	96,3%
Select seq gb|HM347898.1|	Berberis angulosa clone 7 internal transcribed spacer 1, 5.8S ribosomal RNA gene, and internal transcribed spacer 2, complete sequence	1081	1081	89%	0.0	99%	HM347898.1	1202,46	96,3%
Select seq gb|HM347892.1|	Berberis angulosa clone 1 internal transcribed spacer 1, 5.8S ribosomal RNA gene, and internal transcribed spacer 2, complete sequence	1081	1081	89%	0.0	99%	HM347892.1	1202,46	96,3%
Select seq gb|KC575614.1|	Berberis coxii voucher RBGE-19251027 internal transcribed spacer 1, partial sequence; 5.8S ribosomal RNA gene and internal transcribed spacer 2, complete sequence; and 28S ribosomal RNA gene, partial sequence	1079	1079	89%	0.0	99%	KC575614.1	1200,24	96,2%
Select seq gb|KC575580.1|	Berberis sibirica voucher Harber AS81 18S ribosomal RNA gene, partial sequence; internal transcribed spacer 1, 5.8S ribosomal RNA gene, and internal transcribed spacer 2, complete sequence; and 28S ribosomal RNA gene, partial sequence	1127	1127	93%	0.0	99%	KC575580.1	1199,71	96,1%
Select seq gb|KC575597.1|	Berberis tsarongensis voucher RBGE-19381165 18S ribosomal RNA gene, partial sequence; internal transcribed spacer 1, 5.8S ribosomal RNA gene, and internal transcribed spacer 2, complete sequence; and 28S ribosomal RNA gene, partial sequence	1173	1173	97%	0.0	99%	KC575597.1	1197,19	95,9%
Select seq gb|KC575573.1|	Berberis kumaonensis voucher JRSA 176 (E) 18S ribosomal RNA gene, partial sequence; internal transcribed spacer 1, 5.8S ribosomal RNA gene, and internal transcribed spacer 2, complete sequence; and 28S ribosomal RNA gene, partial sequence	1160	1160	96%	0.0	99%	KC575573.1	1196,25	95,8%
Select seq gb|HM347897.1|	Berberis angulosa clone 6 internal transcribed spacer 1, 5.8S ribosomal RNA gene, and internal transcribed spacer 2, complete sequence	1075	1075	89%	0.0	99%	HM347897.1	1195,79	95,8%
Select seq gb|GU934658.1|	Berberis chitria isolate B73 internal transcribed spacer 1, partial sequence; 5.8S ribosomal RNA gene, complete sequence; and internal transcribed spacer 2, partial sequence	1075	1075	89%	0.0	99%	GU934658.1	1195,79	95,8%
Select seq gb|KC575610.1|	Berberis bergmanniae voucher RBGE-19754095A internal transcribed spacer 1, partial sequence; 5.8S ribosomal RNA gene and internal transcribed spacer 2, complete sequence; and 28S ribosomal RNA gene, partial sequence	1098	1098	91%	0.0	99%	KC575610.1	1194,53	95,7%
Select seq gb|KC575587.1|	Berberis aristata voucher Adhikari, B. G19 (E) internal transcribed spacer 1, partial sequence; 5.8S ribosomal RNA gene and internal transcribed spacer 2, complete sequence; and 28S ribosomal RNA gene, partial sequence	1098	1098	91%	0.0	99%	KC575587.1	1194,53	95,7%
Select seq gb|KC575613.1|	Berberis hookeri voucher LKSRB 47 (E) internal transcribed spacer 1, partial sequence; 5.8S ribosomal RNA gene and internal transcribed spacer 2, complete sequence; and 28S ribosomal RNA gene, partial sequence	1122	1122	93%	0.0	99%	KC575613.1	1194,39	95,7%
Select seq gb|KC575612.1|	Berberis sargentiana voucher RBGE-19784169 18S ribosomal RNA gene, partial sequence; internal transcribed spacer 1, 5.8S ribosomal RNA gene, and internal transcribed spacer 2, complete sequence; and 28S ribosomal RNA gene, partial sequence	1158	1158	96%	0.0	99%	KC575612.1	1194,19	95,7%
Select seq gb|JN012234.1|	Berberis tsienii voucher YS1-6 18S ribosomal RNA gene, partial sequence; internal transcribed spacer 1, 5.8S ribosomal RNA gene, and internal transcribed spacer, complete sequence; and 28S ribosomal RNA gene, partial sequence	1182	1182	98%	0.0	99%	JN012234.1	1194,06	95,7%
Select seq gb|KC575606.1|	Berberis koreana voucher RBGE-19731131A internal transcribed spacer 1, partial sequence; 5.8S ribosomal RNA gene and internal transcribed spacer 2, complete sequence; and 28S ribosomal RNA gene, partial sequence	1096	1096	91%	0.0	99%	KC575606.1	1192,35	95,5%
Select seq gb|KC575582.1|	Berberis orthobotrys var. rubicunda voucher Adhikari, B. BL2 39 (E) internal transcribed spacer 1, partial sequence; 5.8S ribosomal RNA gene and internal transcribed spacer 2, complete sequence; and 28S ribosomal RNA gene, partial sequence	1107	1107	92%	0.0	99%	KC575582.1	1191,23	95,4%
Select seq gb|HM347914.1|	Berberis umbellata clone 5 internal transcribed spacer 1, 5.8S ribosomal RNA gene, and internal transcribed spacer 2, complete sequence	1070	1070	89%	0.0	99%	HM347914.1	1190,22	95,4%
Select seq gb|GU934669.1|	Berberis glaucocarpa isolate B169 internal transcribed spacer 1, partial sequence; 5.8S ribosomal RNA gene, complete sequence; and internal transcribed spacer 2, partial sequence	1070	1070	89%	0.0	99%	GU934669.1	1190,22	95,4%
Select seq gb|GU934673.1|	Berberis griffithiana isolate B141 internal transcribed spacer 1, partial sequence; 5.8S ribosomal RNA gene, complete sequence; and internal transcribed spacer 2, partial sequence	1070	1070	89%	0.0	99%	GU934673.1	1190,22	95,4%
Select seq gb|GU934682.1|	Berberis insignis isolate B127 internal transcribed spacer 1, partial sequence; 5.8S ribosomal RNA gene, complete sequence; and internal transcribed spacer 2, partial sequence	1070	1070	89%	0.0	99%	GU934682.1	1190,22	95,4%
Select seq gb|GU934624.1|	Berberis asiatica isolate B30 internal transcribed spacer 1, partial sequence; 5.8S ribosomal RNA gene, complete sequence; and internal transcribed spacer 2, partial sequence	1070	1070	89%	0.0	99%	GU934624.1	1190,22	95,4%
Select seq gb|GU934614.1|	Berberis aristata isolate B53 internal transcribed spacer 1, partial sequence; 5.8S ribosomal RNA gene, complete sequence; and internal transcribed spacer 2, partial sequence	1070	1070	89%	0.0	99%	GU934614.1	1190,22	95,4%
Select seq gb|KC575611.1|	Berberis wallichiana voucher Adhikari, B. BL2 20 (E) internal transcribed spacer 1, partial sequence; 5.8S ribosomal RNA gene and internal transcribed spacer 2, complete sequence; and 28S ribosomal RNA gene, partial sequence	1090	1090	91%	0.0	99%	KC575611.1	1185,82	95,0%
Select seq gb|KC575591.1|	Berberis fendleri voucher Ross McCauley 640 (E) 18S ribosomal RNA gene, partial sequence; internal transcribed spacer 1, 5.8S ribosomal RNA gene, and internal transcribed spacer 2, complete sequence; and 28S ribosomal RNA gene, partial sequence	1134	1134	94%	0.0	98%	KC575591.1	1182,26	94,7%
Select seq gb|KC575579.1|	Berberis karkaralensis voucher JRS A59 (E) 18S ribosomal RNA gene, partial sequence; internal transcribed spacer 1, 5.8S ribosomal RNA gene, and internal transcribed spacer 2, complete sequence; and 28S ribosomal RNA gene, partial sequence	1155	1155	96%	0.0	98%	KC575579.1	1179,06	94,5%

Select seq gb|KC575605.1|	Berberis tschonoskyana voucher RBGE-19940193B internal transcribed spacer 1, partial sequence; 5.8S ribosomal RNA gene and internal transcribed spacer 2, complete sequence; and 28S ribosomal RNA gene, partial sequence	1092	1092	91% 0.0	98% KC575605.1	1176,00	94,2%
Select seq gb|KC575589.1|	Berberis thomsoniana voucher Adhikari, B. G4 (E) internal transcribed spacer 1, partial sequence; 5.8S ribosomal RNA gene and internal transcribed spacer 2, complete sequence; and 28S ribosomal RNA gene, partial sequence	1114	1114	93% 0.0	98% KC575589.1	1173,89	94,1%
Select seq gb|KC575621.1|	Berberis ilicifolia voucher RBGE-19880919A internal transcribed spacer 1, partial sequence; 5.8S ribosomal RNA gene and internal transcribed spacer 2, complete sequence; and 28S ribosomal RNA gene, partial sequence	1090	1090	91% 0.0	98% KC575621.1	1173,85	94,1%
Select seq gb|KC575578.1|	Berberis pendryi voucher Adhikari, B. EA2 5 (E) internal transcribed spacer 1, partial sequence; 5.8S ribosomal RNA gene and internal transcribed spacer 2, complete sequence; and 28S ribosomal RNA gene, partial sequence	1090	1090	91% 0.0	98% KC575578.1	1173,85	94,1%
Select seq gb|KC575584.1|	Berberis asiatica voucher JRS A88 (E) internal transcribed spacer 1, partial sequence; 5.8S ribosomal RNA gene and internal transcribed spacer 2, complete sequence; and 28S ribosomal RNA gene, partial sequence	1088	1088	91% 0.0	98% KC575584.1	1171,69	93,9%
Select seq gb|KC575576.1|	Berberis mucrifolia voucher Adhikari, B. EA2 29 (E) internal transcribed spacer 1, partial sequence; 5.8S ribosomal RNA gene and internal transcribed spacer 2, complete sequence; and 28S ribosomal RNA gene, partial sequence	1088	1088	91% 0.0	98% KC575576.1	1171,69	93,9%
Select seq gb|HM347924.1|	Berberis jaeschkeana clone 6 internal transcribed spacer 1, 5.8S ribosomal RNA gene, and internal transcribed spacer 2, complete sequence	1064	1064	89% 0.0	98% HM347924.1	1171,60	93,9%
Select seq gb|HM347923.1|	Berberis jaeschkeana clone 5 internal transcribed spacer 1, 5.8S ribosomal RNA gene, and internal transcribed spacer 2, complete sequence	1064	1064	89% 0.0	98% HM347923.1	1171,60	93,9%
Select seq gb|HM347921.1|	Berberis jaeschkeana clone 3 internal transcribed spacer 1, 5.8S ribosomal RNA gene, and internal transcribed spacer 2, complete sequence	1064	1064	89% 0.0	98% HM347921.1	1171,60	93,9%
Select seq gb|HM347917.1|	Berberis umbellata clone 8 internal transcribed spacer 1, 5.8S ribosomal RNA gene, and internal transcribed spacer 2, complete sequence	1064	1064	89% 0.0	98% HM347917.1	1171,60	93,9%
Select seq gb|HM347915.1|	Berberis umbellata clone 6 internal transcribed spacer 1, 5.8S ribosomal RNA gene, and internal transcribed spacer 2, complete sequence	1064	1064	89% 0.0	98% HM347915.1	1171,60	93,9%
Select seq gb|GU934625.1|	Berberis asiatica isolate B26 internal transcribed spacer 1, partial sequence; 5.8S ribosomal RNA gene, complete sequence; and internal transcribed spacer 2, partial sequence	1064	1064	89% 0.0	98% GU934625.1	1171,60	93,9%
Select seq gb|HM347882.1|	Berberis lycium clone 6 internal transcribed spacer 1, 5.8S ribosomal RNA gene, and internal transcribed spacer 2, complete sequence	1064	1064	89% 0.0	98% HM347882.1	1171,60	93,9%
Select seq gb|HM347881.1|	Berberis lycium clone 5 internal transcribed spacer 1, 5.8S ribosomal RNA gene, and internal transcribed spacer 2, complete sequence	1064	1064	89% 0.0	98% HM347881.1	1171,60	93,9%
Select seq gb|HM347880.1|	Berberis lycium clone 4 internal transcribed spacer 1, 5.8S ribosomal RNA gene, and internal transcribed spacer 2, complete sequence	1064	1064	89% 0.0	98% HM347880.1	1171,60	93,9%
Select seq gb|GU934709.1|	Berberis replicata isolate B115 internal transcribed spacer 1, partial sequence; 5.8S ribosomal RNA gene, complete sequence; and internal transcribed spacer 2, partial sequence	1064	1064	89% 0.0	98% GU934709.1	1171,60	93,9%
Select seq gb|GU934684.1|	Berberis insignis isolate B129 internal transcribed spacer 1, partial sequence; 5.8S ribosomal RNA gene, complete sequence; and internal transcribed spacer 2, partial sequence	1064	1064	89% 0.0	98% GU934684.1	1171,60	93,9%
Select seq gb|KC575577.1|	Berberis jaeschkeana var. usteriana voucher Adhikari, B. EA2 40 (E) internal transcribed spacer 1, partial sequence; 5.8S ribosomal RNA gene and internal transcribed spacer 2, complete sequence; and 28S ribosomal RNA gene, partial sequence	1099	1099	92% 0.0	98% KC575577.1	1170,67	93,8%
Select seq gb|KC575572.1|	Berberis angulosa var. fasciculata voucher Adhikari, EL 127 (E) internal transcribed spacer 1, partial sequence; 5.8S ribosomal RNA gene and internal transcribed spacer 2, complete sequence; and 28S ribosomal RNA gene, partial sequence	1099	1099	92% 0.0	98% KC575572.1	1170,67	93,8%
Select seq gb|KC575570.1|	Berberis angulosa var. angulosa voucher Adhikari, B. BL2 38 (E) internal transcribed spacer 1, partial sequence; 5.8S ribosomal RNA gene and internal transcribed spacer 2, complete sequence; and 28S ribosomal RNA gene, partial sequence	1099	1099	92% 0.0	98% KC575570.1	1170,67	93,8%
Select seq gb|KC575585.1|	Berberis koehneana voucher Adhikari, B. BL2 55 (E) 18S ribosomal RNA gene, partial sequence; internal transcribed spacer 1, 5.8S ribosomal RNA gene, and internal transcribed spacer 2, complete sequence; and 28S ribosomal RNA gene, partial sequence	1109	1109	93% 0.0	98% KC575585.1	1168,62	93,6%
Select seq gb|KC575624.1|	Berberis microphylla voucher RBGE-19670638B internal transcribed spacer 1, partial sequence; 5.8S ribosomal RNA gene and internal transcribed spacer 2, complete sequence; and 28S ribosomal RNA gene, partial sequence	1085	1085	91% 0.0	98% KC575624.1	1168,46	93,6%
Select seq gb|KC575571.1|	Berberis angulosa var. angulosa voucher Adhikari, EL 126 (E) internal transcribed spacer 1, partial sequence; 5.8S ribosomal RNA gene and internal transcribed spacer 2, complete sequence; and 28S ribosomal RNA gene, partial sequence	1105	1105	93% 0.0	98% KC575571.1	1164,41	93,3%
Select seq gb|KC575586.1|	Berberis koehneana voucher Adhikari, B. BL2 26 (E) internal transcribed spacer 1, partial sequence; 5.8S ribosomal RNA gene and internal transcribed spacer 2, complete sequence; and 28S ribosomal RNA gene, partial sequence	1081	1081	91% 0.0	98% KC575586.1	1164,15	93,3%
Select seq gb|KC575626.1|	Berberis trigona voucher Harber LA3 18S ribosomal RNA gene, partial sequence; internal transcribed spacer 1, 5.8S ribosomal RNA gene, and internal transcribed spacer 2, complete sequence; and 28S ribosomal RNA gene, partial sequence	1140	1140	96% 0.0	98% KC575626.1	1163,75	93,2%
Select seq gb|KC575625.1|	Berberis montana voucher RBGE-19921060A internal transcribed spacer 1, partial sequence; 5.8S ribosomal RNA gene and internal transcribed spacer 2, complete sequence; and 28S ribosomal RNA gene, partial sequence	1079	1079	91% 0.0	98% KC575625.1	1162,00	93,1%
Select seq gb|KC575615.1|	Berberis congestiflora voucher RBGE-19892971B internal transcribed spacer 1, partial sequence; 5.8S ribosomal RNA gene and internal transcribed spacer 2, complete sequence; and 28S ribosomal RNA gene, partial sequence	1079	1079	91% 0.0	98% KC575615.1	1162,00	93,1%
Select seq gb|KC575617.1|	Berberis empetrifolia voucher RBGE-19761088A internal transcribed spacer 1, partial sequence; 5.8S ribosomal RNA gene and internal transcribed spacer 2, complete sequence; and 28S ribosomal RNA gene, partial sequence	1101	1101	93% 0.0	98% KC575617.1	1160,19	93,0%

Select seq gb|KC575619.1|	Berberis rotundifolia voucher RBGE-20080789 C internal transcribed spacer 1, partial sequence; 5.8S ribosomal RNA gene and internal transcribed spacer 2, complete sequence; and 28S ribosomal RNA gene, partial sequence	1077	1077	91%	0.0	98%	KC575619.1	1159,85	92,9%
Select seq gb|KC575583.1|	Berberis asiatica voucher Adhikari, B. G 2 (E) internal transcribed spacer 1, partial sequence; 5.8S ribosomal RNA gene and internal transcribed spacer 2, complete sequence; and 28S ribosomal RNA gene, partial sequence	1064	1064	90%	0.0	98%	KC575583.1	1158,58	92,8%
Select seq gb|KC575623.1|	Berberis valdiviana voucher RBGE-19900563C internal transcribed spacer 1, partial sequence; 5.8S ribosomal RNA gene and internal transcribed spacer 2, complete sequence; and 28S ribosomal RNA gene, partial sequence	1075	1075	91%	0.0	98%	KC575623.1	1157,69	92,8%
Select seq gb|KJ557011.1|	Berberis napaulensis isolate MH9 18S ribosomal RNA gene, partial sequence; internal transcribed spacer 1 and 5.8S ribosomal RNA gene, complete sequence; and internal transcribed spacer 2, partial sequence	1098	1098	93%	0.0	98%	KJ557011.1	1157,03	92,7%
Select seq gb|KC575616.1|	Berberis actinacantha voucher Gardner & Knees 8421 (E) internal transcribed spacer 1, partial sequence; 5.8S ribosomal RNA gene and internal transcribed spacer 2, complete sequence; and 28S ribosomal RNA gene, partial sequence	1074	1074	91%	0.0	98%	KC575616.1	1156,62	92,7%
Select seq gb|AY858638.1|	Magnolia heptapetala 18S ribosomal RNA gene, partial sequence; internal transcribed spacer 1, 5.8S ribosomal RNA gene, and internal transcribed spacer 2, complete sequence; and 26S ribosomal RNA gene, partial sequence	1118	1118	95%	0.0	98%	AY858638.1	1153,31	92,4%
Select seq gb|KJ557009.1|	Berberis napaulensis isolate MH7 18S ribosomal RNA gene, partial sequence; internal transcribed spacer 1 and 5.8S ribosomal RNA gene, complete sequence; and internal transcribed spacer 2, partial sequence	1103	1103	94%	0.0	98%	KJ557009.1	1149,94	92,1%
Select seq gb|KC575630.1|	Berberis napaulensis voucher LKSRB 1 (E) internal transcribed spacer 1, partial sequence; 5.8S ribosomal RNA gene and internal transcribed spacer 2, complete sequence; and 28S ribosomal RNA gene, partial sequence	1072	1072	92%	0.0	98%	KC575630.1	1141,91	91,5%
Select seq gb|KJ557005.1|	Berberis napaulensis isolate MH2 internal transcribed spacer 1, partial sequence; 5.8S ribosomal RNA gene, complete sequence; and internal transcribed spacer 2, partial sequence	1092	1092	94%	0.0	98%	KJ557005.1	1138,47	91,2%
Select seq gb|KC575633.1|	Berberis aquifolium voucher RBGE-19912984A internal transcribed spacer 1, partial sequence; 5.8S ribosomal RNA gene and internal transcribed spacer 2, complete sequence; and 28S ribosomal RNA gene, partial sequence	1064	1064	91%	0.0	97%	KC575633.1	1134,15	90,9%
Select seq gb|KJ557006.1|	Berberis napaulensis isolate MH4 internal transcribed spacer 1, partial sequence; 5.8S ribosomal RNA gene, complete sequence; and internal transcribed spacer 2, partial sequence	1079	1079	93%	0.0	97%	KJ557006.1	1125,41	90,2%
Select seq gb|FJ980428.1|	Berberis fortunei voucher PS1509MT01 18S ribosomal RNA gene, partial sequence; internal transcribed spacer 1, 5.8S ribosomal RNA gene, and internal transcribed spacer 2, complete sequence; and 28S ribosomal RNA gene, partial sequence	1118	1118	97%	0.0	97%	FJ980428.1	1118,00	89,6%
Select seq gb|KJ557004.1|	Berberis napaulensis isolate MH1 internal transcribed spacer 1, partial sequence; 5.8S ribosomal RNA gene, complete sequence; and internal transcribed spacer 2, partial sequence	1079	1079	94%	0.0	97%	KJ557004.1	1113,44	89,2%
Select seq gb|KJ557007.1|	Berberis napaulensis isolate MH5 18S ribosomal RNA gene, partial sequence; internal transcribed spacer 1 and 5.8S ribosomal RNA gene, complete sequence; and internal transcribed spacer 2, partial sequence	1075	1075	94%	0.0	97%	KJ557007.1	1109,31	88,9%
Select seq gb|KC575632.1|	Berberis repens voucher RBGE-19800553A internal transcribed spacer 1, partial sequence; 5.8S ribosomal RNA gene and internal transcribed spacer 2, complete sequence; and 28S ribosomal RNA gene, partial sequence	1062	1062	92%	0.0	96%	KC575632.1	1108,17	88,8%
Select seq gb|KJ557008.1|	Berberis napaulensis isolate MH6 18S ribosomal RNA gene, partial sequence; internal transcribed spacer 1 and 5.8S ribosomal RNA gene, complete sequence; and internal transcribed spacer 2, partial sequence	1070	1070	94%	0.0	97%	KJ557008.1	1104,15	88,5%
Select seq gb|KC575634.1|	Berberis fremontii voucher RBGE-19716619A internal transcribed spacer 1, partial sequence; 5.8S ribosomal RNA gene and internal transcribed spacer 2, complete sequence; and 28S ribosomal RNA gene, partial sequence	1057	1057	92%	0.0	96%	KC575634.1	1102,96	88,4%

Select for downloading or viewing reports	Kh045_ITS Description	Max score	Total score	Query cover	E value	Ident	Accession	(Ident/Cover)* Max score	Deviation from top hit
Select seq gb|GQ379304.1|	Bunium capillifolium voucher Kamelin 1564 6-VII-1974 (LE) internal transcribed spacer 1, 5.8S ribosomal RNA gene, and internal transcribed spacer 2, complete sequence	865	865	89%	0.0	93%	GQ379304.1	903,88	100,0%
Select seq gb|AF008631.2|	Pyramidoptera cabulica internal transcribed spacer 1, 5.8S ribosomal RNA gene, and internal transcribed spacer 2, complete sequence	850	850	89%	0.0	93%	AF008631.2	888,20	98,3%
Select seq gb|HM229354.1|	Elaeosticta allioides voucher M.G. Pimenov & E.V. Kljuykov 622 (MW) internal transcribed spacer 1, 5.8S ribosomal RNA gene, and internal transcribed spacer 2, complete sequence	843	843	89%	0.0	92%	HM229354.1	871,42	96,4%
Select seq gb|HM229403.1|	Oedibasis tamerlanii voucher M.G. Pimenov & E.V. Kljuykov 48 (MW) internal transcribed spacer 1, 5.8S ribosomal RNA gene, and internal transcribed spacer 2, complete sequence	841	841	89%	0.0	92%	HM229403.1	869,35	96,2%
Select seq gb|HM229373.1|	Elaeosticta platyphylla voucher M.G. Pimenov et al. 285 (MW) internal transcribed spacer 1, 5.8S ribosomal RNA gene, and internal transcribed spacer 2, complete sequence	830	830	88%	0.0	92%	HM229373.1	867,73	96,0%
Select seq gb|HM229398.1|	Hyalolaena melanorrhiza voucher M.G. Pimenov et al. 1004 (MW) internal transcribed spacer 1, 5.8S ribosomal RNA gene, and internal transcribed spacer 2, complete sequence	835	835	89%	0.0	92%	HM229398.1	863,15	95,5%
Select seq gb|KC784681.1|	Schrenkia kultiassovii voucher MW 330 internal transcribed spacer 1, 5.8S ribosomal RNA gene, and internal transcribed spacer 2, complete sequence	821	821	88%	0.0	92%	KC784681.1	858,32	95,0%
Select seq gb|HM229362.1|	Elaeosticta glaucescens voucher M.G. Pimenov et al. 348 (MW) internal transcribed spacer 1, 5.8S ribosomal RNA gene, and internal transcribed spacer 2, complete sequence	830	830	89%	0.0	92%	HM229362.1	857,98	94,9%
Select seq gb|DQ516359.1|	Muretia lutea isolate K69 internal transcribed spacer 1, 5.8S ribosomal RNA gene, and internal transcribed spacer 2, complete sequence	830	830	89%	0.0	92%	DQ516359.1	857,98	94,9%
Select seq gb|KC784687.1|	Schrenkia ugamica voucher MW 1649 internal transcribed spacer 1, 5.8S ribosomal RNA gene, and internal transcribed spacer 2, complete sequence	828	828	89%	0.0	92%	KC784687.1	855,91	94,7%
Select seq gb|KC784675.1|	Schrenkia golickeana voucher MW 112a internal transcribed spacer 1, 5.8S ribosomal RNA gene, and internal transcribed spacer 2, complete sequence	828	828	89%	0.0	92%	KC784675.1	855,91	94,7%
Select seq gb|HM229357.1|	Elaeosticta conica voucher M.G. Pimenov et al. 426 (MW) internal transcribed spacer 1, 5.8S ribosomal RNA gene, and internal transcribed spacer 2, complete sequence	826	826	89%	0.0	92%	HM229357.1	853,84	94,5%
Select seq gb|HM229356.1|	Elaeosticta bucharica voucher M.G. Pimenov & E.V. Kljuykov 409 (MW) internal transcribed spacer 1, 5.8S ribosomal RNA gene, and internal transcribed spacer 2, complete sequence	826	826	89%	0.0	92%	HM229356.1	853,84	94,5%
Select seq gb|HM229359.1|	Elaeosticta meifolia voucher 54987 (TARI) internal transcribed spacer 1, 5.8S ribosomal RNA gene, and internal transcribed spacer 2, complete sequence	824	824	89%	0.0	92%	HM229359.1	851,78	94,2%
Select seq gb|HM229367.1|	Elaeosticta korovinii voucher M.G. Pimenov & E.V. Kljuykov 129 (MW) internal transcribed spacer 1, 5.8S ribosomal RNA gene, and internal transcribed spacer 2, complete sequence	824	824	89%	0.0	92%	HM229367.1	851,78	94,2%
Select seq gb|GQ379316.1|	Schulzia albiflora voucher Sapozhnikov s.n. 24-VII-1913 (LE) internal transcribed spacer 1, 5.8S ribosomal RNA gene, and internal transcribed spacer 2, complete sequence	824	824	89%	0.0	92%	GQ379316.1	851,78	94,2%
Select seq gb|KC784685.1|	Schrenkia pulverulenta voucher MW K96-229 internal transcribed spacer 1, 5.8S ribosomal RNA gene, and internal transcribed spacer 2, complete sequence	822	822	89%	0.0	92%	KC784685.1	849,71	94,0%
Select seq gb|KC784677.1|	Schrenkia golickeana voucher MW 135 internal transcribed spacer 1, 5.8S ribosomal RNA gene, and internal transcribed spacer 2, complete sequence	822	822	89%	0.0	92%	KC784677.1	849,71	94,0%
Select seq gb|HM229389.1|	Galagania gracilis voucher M.G. Pimenov & E.V. Kljuykov 190 (MW) internal transcribed spacer 1, 5.8S ribosomal RNA gene, and internal transcribed spacer 2, complete sequence	822	822	89%	0.0	92%	HM229389.1	849,71	94,0%
Select seq gb|HM229386.1|	Elaeosticta tschimganica voucher E.V. Kljuykov 29 (MW) internal transcribed spacer 1, 5.8S ribosomal RNA gene, and internal transcribed spacer 2, complete sequence	822	822	89%	0.0	92%	HM229386.1	849,71	94,0%
Select seq gb|GQ379310.1|	Indoschulzia hameliana voucher Farille 78-79 26-X-1978 (G) internal transcribed spacer 1, 5.8S ribosomal RNA gene, and internal transcribed spacer 2, complete sequence	822	822	89%	0.0	92%	GQ379310.1	849,71	94,0%
Select seq gb|HM229388.1|	Elaeosticta vvedenskyi voucher M.G. Pimenov et al. 749 (MW) internal transcribed spacer 1, 5.8S ribosomal RNA gene, and internal transcribed spacer 2, complete sequence	821	821	89%	0.0	92%	HM229388.1	848,67	93,9%
Select seq gb|HM229395.1|	Hyalolaena intermedia voucher M.G. Pimenov 828 (MW) internal transcribed spacer 1, 5.8S ribosomal RNA gene, and internal transcribed spacer 2, complete sequence	819	819	89%	0.0	92%	HM229395.1	846,61	93,7%
Select seq gb|HM229390.1|	Galagania margiana voucher M.G. Pimenov & E.V. Kljuykov 267 (MW) internal transcribed spacer 1, 5.8S ribosomal RNA gene, and internal transcribed spacer 2, complete sequence	819	819	89%	0.0	92%	HM229390.1	846,61	93,7%
Select seq gb|HM229368.1|	Elaeosticta lutea voucher Ju.V. Baranova & T.V. Lavrova 1037 (MW) internal transcribed spacer 1, 5.8S ribosomal RNA gene, and internal transcribed spacer 2, complete sequence	819	819	89%	0.0	92%	HM229368.1	846,61	93,7%
Select seq gb|HM229365.1|	Elaeosticta knorringiana voucher M.G. Pimenov et al. 378 (MW) internal transcribed spacer 1, 5.8S ribosomal RNA gene, and internal transcribed spacer 2, complete sequence	819	819	89%	0.0	92%	HM229365.1	846,61	93,7%
Select seq gb|GQ379315.1|	Schtschurowskia margaritae voucher Kamelin 1325 17-VI-1974 (LE) internal transcribed spacer 1, 5.8S ribosomal RNA gene, and internal transcribed spacer 2, complete sequence	819	819	89%	0.0	92%	GQ379315.1	846,61	93,7%

<table>
<tr><td>Select seq gb|HM229401.1|</td><td>Mogoltavia narynensis voucher Aidarova et al. s.n. (MW) internal transcribed spacer 1, 5.8S ribosomal RNA gene, and internal transcribed spacer 2, complete sequence</td><td>808</td><td>808</td><td>88% 0.0</td><td>92% HM229401.1</td><td>844,73</td><td>93,5%</td></tr>
<tr><td>Select seq gb|KC784692.1|</td><td>Schrenkia vaginata voucher LE sn internal transcribed spacer 1, 5.8S ribosomal RNA gene, and internal transcribed spacer 2, complete sequence</td><td>817</td><td>817</td><td>89% 0.0</td><td>92% KC784692.1</td><td>844,54</td><td>93,4%</td></tr>
<tr><td>Select seq gb|KC784676.1|</td><td>Schrenkia golickeana voucher MW 1214 internal transcribed spacer 1, 5.8S ribosomal RNA gene, and internal transcribed spacer 2, complete sequence</td><td>817</td><td>817</td><td>89% 0.0</td><td>92% KC784676.1</td><td>844,54</td><td>93,4%</td></tr>
<tr><td>Select seq gb|HM229394.1|</td><td>Hyalolaena intermedia voucher M.G. Pimenov et al. K96-123 (MW) internal transcribed spacer 1, 5.8S ribosomal RNA gene, and internal transcribed spacer 2, complete sequence</td><td>817</td><td>817</td><td>89% 0.0</td><td>92% HM229394.1</td><td>844,54</td><td>93,4%</td></tr>
<tr><td>Select seq gb|GQ379311.1|</td><td>Kosopoljanskia turkestanica voucher Kamelin s.n. 5-VII-1973 (LE) internal transcribed spacer 1, 5.8S ribosomal RNA gene, and internal transcribed spacer 2, complete sequence</td><td>817</td><td>817</td><td>89% 0.0</td><td>92% GQ379311.1</td><td>844,54</td><td>93,4%</td></tr>
<tr><td>Select seq gb|HM229392.1|</td><td>Galagania platypoda voucher M.G. Pimenov et al. 440 (MW) internal transcribed spacer 1, 5.8S ribosomal RNA gene, and internal transcribed spacer 2, complete sequence</td><td>804</td><td>804</td><td>88% 0.0</td><td>91% HM229392.1</td><td>831,41</td><td>92,0%</td></tr>
<tr><td>Select seq gb|KC784690.1|</td><td>Schrenkia vaginata voucher MW K96-154 internal transcribed spacer 1, 5.8S ribosomal RNA gene, and internal transcribed spacer 2, complete sequence</td><td>811</td><td>811</td><td>89% 0.0</td><td>91% KC784690.1</td><td>829,22</td><td>91,7%</td></tr>
<tr><td>Select seq gb|KC784682.1|</td><td>Schrenkia sp. 'minkwitzae' voucher LE sn internal transcribed spacer 1, 5.8S ribosomal RNA gene, and internal transcribed spacer 2, complete sequence</td><td>811</td><td>811</td><td>89% 0.0</td><td>91% KC784682.1</td><td>829,22</td><td>91,7%</td></tr>
<tr><td>Select seq gb|HM229391.1|</td><td>Galagania neglecta voucher M.G. Pimenov et al. 320 (MW) internal transcribed spacer 1, 5.8S ribosomal RNA gene, and internal transcribed spacer 2, complete sequence</td><td>811</td><td>811</td><td>89% 0.0</td><td>91% HM229391.1</td><td>829,22</td><td>91,7%</td></tr>
<tr><td>Select seq gb|KC784695.1|</td><td>Schrenkia vaginata voucher MW 276 internal transcribed spacer 1, 5.8S ribosomal RNA gene, and internal transcribed spacer 2, complete sequence</td><td>806</td><td>806</td><td>89% 0.0</td><td>91% KC784695.1</td><td>824,11</td><td>91,2%</td></tr>
<tr><td>Select seq gb|KC784691.1|</td><td>Schrenkia vaginata voucher MW 270 internal transcribed spacer 1, 5.8S ribosomal RNA gene, and internal transcribed spacer 2, complete sequence</td><td>806</td><td>806</td><td>89% 0.0</td><td>91% KC784691.1</td><td>824,11</td><td>91,2%</td></tr>
<tr><td>Select seq gb|KC784688.1|</td><td>Schrenkia vaginata voucher MW 74 internal transcribed spacer 1, 5.8S ribosomal RNA gene, and internal transcribed spacer 2, complete sequence</td><td>806</td><td>806</td><td>89% 0.0</td><td>91% KC784688.1</td><td>824,11</td><td>91,2%</td></tr>
<tr><td>Select seq gb|KC784684.1|</td><td>Schrenkia papillaris voucher LE sn internal transcribed spacer 1, 5.8S ribosomal RNA gene, and internal transcribed spacer 2, complete sequence</td><td>806</td><td>806</td><td>89% 0.0</td><td>91% KC784684.1</td><td>824,11</td><td>91,2%</td></tr>
<tr><td>Select seq gb|KC784673.1|</td><td>Schrenkia fasciculata voucher MW 708 internal transcribed spacer 1, 5.8S ribosomal RNA gene, and internal transcribed spacer 2, complete sequence</td><td>806</td><td>806</td><td>89% 0.0</td><td>91% KC784673.1</td><td>824,11</td><td>91,2%</td></tr>
<tr><td>Select seq gb|HM229396.1|</td><td>Hyalolaena issykkulensis voucher M.G. Pimenov & E.V. Kljuykov 907 (MW) internal transcribed spacer 1, 5.8S ribosomal RNA gene, and internal transcribed spacer 2, complete sequence</td><td>806</td><td>806</td><td>89% 0.0</td><td>91% HM229396.1</td><td>824,11</td><td>91,2%</td></tr>
<tr><td>Select seq gb|HM229360.1|</td><td>Elaeosticta ferganensis voucher E.V. Kljuykov & Ju.V. Baranova 1179 (MW) internal transcribed spacer 1, 5.8S ribosomal RNA gene, and internal transcribed spacer 2, complete sequence</td><td>806</td><td>806</td><td>89% 0.0</td><td>91% HM229360.1</td><td>824,11</td><td>91,2%</td></tr>
<tr><td>Select seq gb|GQ379312.1|</td><td>Kosopoljanskia turkestanica voucher Kamelin 762 21-VII-1970 (LE) internal transcribed spacer 1, 5.8S ribosomal RNA gene, and internal transcribed spacer 2, complete sequence</td><td>806</td><td>806</td><td>89% 0.0</td><td>91% GQ379312.1</td><td>824,11</td><td>91,2%</td></tr>
<tr><td>Select seq gb|FJ385059.1|</td><td>Schulzia albiflora voucher J106 (XAU) internal transcribed spacer 1, partial sequence; 5.8S ribosomal RNA gene, complete sequence; and internal transcribed spacer 2, partial sequence</td><td>806</td><td>806</td><td>89% 0.0</td><td>91% FJ385059.1</td><td>824,11</td><td>91,2%</td></tr>
<tr><td>Select seq gb|KC784668.1|</td><td>Kosopoljanskia turkestanica voucher MW 249 internal transcribed spacer 1, 5.8S ribosomal RNA gene, and internal transcribed spacer 2, complete sequence</td><td>804</td><td>804</td><td>89% 0.0</td><td>91% KC784668.1</td><td>822,07</td><td>90,9%</td></tr>
<tr><td>Select seq gb|HM229361.1|</td><td>Elaeosticta ferganensis voucher E.V. Kljuykov et al. 404 (MW) internal transcribed spacer 1, 5.8S ribosomal RNA gene, and internal transcribed spacer 2, complete sequence</td><td>804</td><td>804</td><td>89% 0.0</td><td>91% HM229361.1</td><td>822,07</td><td>90,9%</td></tr>
<tr><td>Select seq gb|GQ379305.1|</td><td>Hyalolaena bupleuroides voucher Ikonnikov & Ladygina 9856 15-VIII-1972 (LE) internal transcribed spacer 1, 5.8S ribosomal RNA gene, and internal transcribed spacer 2, complete sequence</td><td>804</td><td>804</td><td>89% 0.0</td><td>91% GQ379305.1</td><td>822,07</td><td>90,9%</td></tr>
<tr><td>Select seq gb|HM229375.1|</td><td>Elaeosticta polycarpa voucher E.V. Kljuykov & M.G. Pimenov 561 (MW) internal transcribed spacer 1, 5.8S ribosomal RNA gene, and internal transcribed spacer 2, complete sequence</td><td>802</td><td>802</td><td>89% 0.0</td><td>91% HM229375.1</td><td>820,02</td><td>90,7%</td></tr>
<tr><td>Select seq gb|KC784696.1|</td><td>Schrenkia vaginata voucher MW sn internal transcribed spacer 1, 5.8S ribosomal RNA gene, and internal transcribed spacer 2, complete sequence</td><td>800</td><td>800</td><td>89% 0.0</td><td>91% KC784696.1</td><td>817,98</td><td>90,5%</td></tr>
<tr><td>Select seq gb|HM229406.1|</td><td>Schulzia prostrata voucher M.G. Pimenov & E.V. Kljuykov 790 (MW) internal transcribed spacer 1, 5.8S ribosomal RNA gene, and internal transcribed spacer 2, complete sequence</td><td>800</td><td>800</td><td>89% 0.0</td><td>91% HM229406.1</td><td>817,98</td><td>90,5%</td></tr>
<tr><td>Select seq gb|GQ379307.1|</td><td>Hyalolaena jaxartica voucher Botchantsev & Takhtajan 23 20-VI-1964 (LE) internal transcribed spacer 1, 5.8S ribosomal RNA gene, and internal transcribed spacer 2, complete sequence</td><td>798</td><td>798</td><td>89% 0.0</td><td>91% GQ379307.1</td><td>815,93</td><td>90,3%</td></tr>
<tr><td>Select seq gb|KF974538.1|</td><td>Bunium elegans voucher MW<RUS>:Pimenov et al. 353 internal transcribed spacer 1, 5.8S ribosomal RNA gene, and internal transcribed spacer 2, complete sequence</td><td>789</td><td>789</td><td>88% 0.0</td><td>91% KF974538.1</td><td>815,90</td><td>90,3%</td></tr>
<tr><td>Select seq gb|HM229402.1|</td><td>Oedibasis apiculata voucher M.G. Pimenov et al. 166 (MW) internal transcribed spacer 1, 5.8S ribosomal RNA gene, and internal transcribed spacer 2, complete sequence</td><td>793</td><td>793</td><td>89% 0.0</td><td>91% HM229402.1</td><td>810,82</td><td>89,7%</td></tr>
<tr><td>Select seq gb|HM229397.1|</td><td>Hyalolaena lipskyi voucher M.G. Pimenov et al. 316 (MW) internal transcribed spacer 1, 5.8S ribosomal RNA gene, and internal transcribed spacer 2, complete sequence</td><td>793</td><td>793</td><td>89% 0.0</td><td>91% HM229397.1</td><td>810,82</td><td>89,7%</td></tr>
</table>

Select seq gb|HM229351.1|	Elaeosticta alaica voucher M.G. Pimenov et al. 71 (MW) internal transcribed spacer 1, 5.8S ribosomal RNA gene, and internal transcribed spacer 2, complete sequence	793	793	89% 0.0	91% HM229351.1	810,82	89,7%
Select seq gb|GQ379309.1|	Hyalolaena jaxartica voucher Novikova s.n. 17-V-1951 (LE) internal transcribed spacer 1, 5.8S ribosomal RNA gene, and internal transcribed spacer 2, complete sequence	793	793	89% 0.0	91% GQ379309.1	810,82	89,7%
Select seq emb|HE602447.1|	Bunium alpinum genomic DNA containing ITS1, 5.8S rRNA gene, ITS2, specimen voucher OPTIMA ITER V. 1064	765	765	86% 0.0	91% HE602447.1	809,48	89,6%
Select seq gb|HM229350.1|	Elaeosticta aitchisonii voucher Ju. Gubanov et al. 444a (MW) internal transcribed spacer 1, 5.8S ribosomal RNA gene, and internal transcribed spacer 2, complete sequence	791	791	89% 0.0	91% HM229350.1	808,78	89,5%
Select seq gb|KC784669.1|	Schrenkia sp. ET-2014 voucher MW 503 internal transcribed spacer 1, 5.8S ribosomal RNA gene, and internal transcribed spacer 2, complete sequence	789	789	89% 0.0	91% KC784669.1	806,73	89,3%
Select seq gb|JQ792213.1|	Carum furcatum voucher Smith 5838 (UPS) internal transcribed spacer 1, 5.8S ribosomal RNA gene, and internal transcribed spacer 2, complete sequence	789	789	89% 0.0	91% JQ792213.1	806,73	89,3%
Select seq gb|KC784693.1|	Schrenkia vaginata voucher MW 177 internal transcribed spacer 1, 5.8S ribosomal RNA gene, and internal transcribed spacer 2, complete sequence	787	787	89% 0.0	91% KC784693.1	804,69	89,0%
Select seq gb|JQ792206.1|	Carum buriaticum voucher Pimenov & Vassilieva 97 (MW) internal transcribed spacer 1, 5.8S ribosomal RNA gene, and internal transcribed spacer 2, complete sequence	787	787	89% 0.0	91% JQ792206.1	804,69	89,0%
Select seq gb|HM229381.1|	Elaeosticta transcaspica voucher E.V. Kljuykov & Ju.V. Baranova 595 (MW) internal transcribed spacer 1, 5.8S ribosomal RNA gene, and internal transcribed spacer 2, complete sequence	787	787	89% 0.0	91% HM229381.1	804,69	89,0%
Select seq emb|HE687357.1|	Bunium sp. EM410 genomic DNA containing ITS1, 5.8S rRNA gene, ITS2, specimen voucher EM410	741	741	84% 0.0	91% HE687357.1	802,75	88,8%
Select seq gb|HM229363.1|	Elaeosticta hirtula voucher I. Hedge & P. Wendelbo W9358 (MW) internal transcribed spacer 1, 5.8S ribosomal RNA gene, and internal transcribed spacer 2, complete sequence	785	785	89% 0.0	91% HM229363.1	802,64	88,8%
Select seq gb|GQ379314.1|	Schtschurowskia margaritae voucher Mikeshin 453 19-VI-1937 (LE) internal transcribed spacer 1, 5.8S ribosomal RNA gene, and internal transcribed spacer 2, complete sequence	784	784	89% 0.0	91% GQ379314.1	801,62	88,7%
Select seq gb|DQ516363.1|	Pimpinella siifolia isolate K171 internal transcribed spacer 1, 5.8S ribosomal RNA gene, and internal transcribed spacer 2, complete sequence	774	774	88% 0.0	90% DQ516363.1	791,59	87,6%
Select seq gb|DQ516358.1|	Gongylotaxis rechingeri isolate K18 internal transcribed spacer 1, 5.8S ribosomal RNA gene, and internal transcribed spacer 2, complete sequence	782	782	89% 0.0	90% DQ516358.1	790,79	87,5%
Select seq gb|JX312805.1|	Bunium allioides voucher Bani 6580 internal transcribed spacer 1, 5.8S ribosomal RNA gene, and internal transcribed spacer 2, complete sequence	773	773	88% 0.0	90% JX312805.1	790,57	87,5%
Select seq gb|GQ379313.1|	Ormopterum turcomanicum voucher Kamelin et al. 597 28-V-1982 (LE) internal transcribed spacer 1, 5.8S ribosomal RNA gene, and internal transcribed spacer 2, complete sequence	776	776	89% 0.0	90% GQ379313.1	784,72	86,8%
Select seq gb|KF974533.1|	Carum depressum voucher C:Kit Tan & Strid 20416 internal transcribed spacer 1, 5.8S ribosomal RNA gene, and internal transcribed spacer 2, complete sequence	763	763	88% 0.0	90% KF974533.1	780,34	86,3%
Select seq gb|EU169246.1|	Astomaea seselifolia voucher Danin & al. 8-80 (RNG) internal transcribed spacer 1, partial sequence; 5.8S ribosomal RNA gene, complete sequence; and internal transcribed spacer 2, partial sequence	763	763	88% 0.0	90% EU169246.1	780,34	86,3%
Select seq gb|DQ516361.1|	Oreoschimperella verrucosa isolate K129 internal transcribed spacer 1, 5.8S ribosomal RNA gene, and internal transcribed spacer 2, complete sequence	754	754	88% 0.0	90% DQ516361.1	771,14	85,3%
Select seq gb|KF974537.1|	Hellenocarum multiflorum voucher MW<RUS>:Zakharova & Ukrainskaya 2 internal transcribed spacer 1, 5.8S ribosomal RNA gene, and internal transcribed spacer 2, complete sequence	745	745	88% 0.0	90% KF974537.1	761,93	84,3%
Select seq gb|KF974534.1|	Hellenocarum strictum voucher MHA:Greuter 16058 internal transcribed spacer 1, 5.8S ribosomal RNA gene, and internal transcribed spacer 2, complete sequence	745	745	88% 0.0	90% KF974534.1	761,93	84,3%
Select seq gb|JQ792227.1|	Tamamschjanella cruciata voucher Sintenis 7255 (LD) internal transcribed spacer 1, 5.8S ribosomal RNA gene, and internal transcribed spacer 2, complete sequence	745	745	88% 0.0	90% JQ792227.1	761,93	84,3%
Select seq emb|HE602464.1|	Bunium bulbocastanum genomic DNA containing ITS1, 5.8S rRNA gene, ITS2, specimen voucher Montserrat, JM. s.n	809	809	96% 0.0	90% HE602464.1	758,44	83,9%
Select seq emb|HE602463.1|	Bunium incrassatum genomic DNA containing ITS1, 5.8S rRNA gene, ITS2, specimen voucher Davis. 52337	809	809	96% 0.0	90% HE602463.1	758,44	83,9%
Select seq gb|HM229364.1|	Elaeosticta hirtula voucher M.G. Pimenov et al. 587 (MW) internal transcribed spacer 1, 5.8S ribosomal RNA gene, and internal transcribed spacer 2, complete sequence	750	750	89% 0.0	89% HM229364.1	750,00	83,0%
Select seq gb|KF974536.1|	Hellenocarum pisidicum voucher E:Ayasligil 1378B internal transcribed spacer 1, 5.8S ribosomal RNA gene, and internal transcribed spacer 2, complete sequence	741	741	88% 0.0	89% KF974536.1	749,42	82,9%
Select seq gb|HM229405.1|	Scaligeria moreana voucher H. Runemark & S. Snogerup 20727 (LD) internal transcribed spacer 1, 5.8S ribosomal RNA gene, and internal transcribed spacer 2, complete sequence	734	734	88% 0.0	89% HM229405.1	742,34	82,1%
Select seq emb|HE602462.1|	Bunium pachypodum genomic DNA containing ITS1, 5.8S rRNA gene, ITS2, specimen voucher Montserrat, JM. 5835/3	774	774	93% 0.0	89% HE602462.1	740,71	81,9%

Select seq gb|HM229404.1|	Scaligeria halophila voucher B. Bentzer & H. Runemark 28236 (LD) internal transcribed spacer 1, 5.8S ribosomal RNA gene, and internal transcribed spacer 2, complete sequence	739	739	89% 0.0	89% HM229404.1	739,00	81,8%
Select seq emb|HE985079.1|	Scaligeria alziarii genomic DNA containing ITS1, 5.8S rRNA gene and ITS2, specimen voucher B:GT 0003944	745	745	90% 0.0	89% HE985079.1	736,72	81,5%
Select seq gb|FJ481926.1|	Ligusticum sp. DCY-02 18S ribosomal RNA gene, partial sequence; internal transcribed spacer 1, 5.8S ribosomal RNA gene, and internal transcribed spacer 2, complete sequence; and 28S ribosomal RNA gene, partial sequence	750	750	97% 0.0	88% FJ481926.1	680,41	75,3%
Select seq gb|HQ202139.1|	Ammi seubertianum voucher M. Carine et al. 121 (AZB, BM) clone 4 internal transcribed spacer 1, partial sequence; 5.8S ribosomal RNA gene, complete sequence; and internal transcribed spacer 2, partial sequence	743	743	97% 0.0	88% HQ202139.1	674,06	74,6%
Select seq gb|HQ202137.1|	Ammi seubertianum voucher M. Carine et al. 5 (AZB, BM) clone 1 internal transcribed spacer 1, partial sequence; 5.8S ribosomal RNA gene, complete sequence; and internal transcribed spacer 2, partial sequence	743	743	97% 0.0	88% HQ202137.1	674,06	74,6%
Select seq gb|KP058314.1|	Ligusticum tenuissimum voucher JKTM-1-000065 18S ribosomal RNA gene, partial sequence; internal transcribed spacer 1, 5.8S ribosomal RNA gene, and internal transcribed spacer 2, complete sequence; and 26S ribosomal RNA gene, partial sequence	747	747	98% 0.0	87% KP058314.1	663,15	73,4%
Select seq gb|FJ481925.1|	Ligusticum tenuissimum isolate DCY-01 18S ribosomal RNA gene, partial sequence; internal transcribed spacer 1, 5.8S ribosomal RNA gene, and internal transcribed spacer 2, complete sequence; and 28S ribosomal RNA gene, partial sequence	747	747	98% 0.0	87% FJ481925.1	663,15	73,4%
Select seq gb|AY548206.1|	Atractylodes macrocephala isolate at5 18S ribosomal RNA gene, partial sequence; internal transcribed spacer 1 and 5.8S ribosomal RNA gene, complete sequence; and internal transcribed spacer 2, partial sequence	747	747	98% 0.0	87% AY548206.1	663,15	73,4%
Select seq gb|KC995009.1|	Falcaria vulgaris voucher 281AW 18S ribosomal RNA gene, partial sequence; internal transcribed spacer 1, 5.8S ribosomal RNA gene, and internal transcribed spacer 2, complete sequence; and 28S ribosomal RNA gene, partial sequence	737	737	97% 0.0	87% KC995009.1	661,02	73,1%
Select seq gb|HQ202136.1|	Ammi seubertianum voucher H. Schaefer 2008-668 (BM) internal transcribed spacer 1, partial sequence; 5.8S ribosomal RNA gene, complete sequence; and internal transcribed spacer 2, partial sequence	737	737	97% 0.0	87% HQ202136.1	661,02	73,1%
Select seq gb|AY548215.1|	Angelica tenuissima isolate um04 18S ribosomal RNA gene, partial sequence; internal transcribed spacer 1, 5.8S ribosomal RNA gene, and internal transcribed spacer 2, complete sequence; and 26S ribosomal RNA gene, partial sequence	741	741	98% 0.0	87% AY548215.1	657,83	72,8%
Select seq gb|AF455750.1|AF455750	Angelica tenuissima 18S ribosomal RNA gene, partial sequence; internal transcribed spacer 1, 5.8S ribosomal RNA gene and internal transcribed spacer 2, complete sequence; and 26S ribosomal RNA gene, partial sequence	741	741	98% 0.0	87% AF455750.1	657,83	72,8%
Select seq gb|KJ025063.1|	Cnidium officinale 18S ribosomal RNA gene, partial sequence; internal transcribed spacer 1, 5.8S ribosomal RNA gene, and internal transcribed spacer 2, complete sequence; and 26S ribosomal RNA gene, partial sequence	734	734	98% 0.0	87% KJ025063.1	651,61	72,1%
						0,00	0,0%
						0,00	0,0%
						0,00	0,0%

Select for downloading or viewing reports	Kh045_trnL Description	Max score	Total score	Query cover	E value	Ident	Accession	(Ident/Cover)* Max score	Deviation from top hit
Select seq gb|AF444009.1|	Cymopterus globosus tRNA-Phe gene, partial sequence; tRNA-Leu gene, complete sequence; and tRNA-Thr gene, partial sequence; chloroplast genes for chloroplast products	459	459	100%	4,00E-125	80%	AF444009.1	367,20	100,0%
Select seq gb|FJ475189.1|	Hymenidium brunonis tRNA-Leu (trnL) gene and trnL-trnF intergenic spacer, partial sequence; chloroplast	444	444	98%	1,00E-120	80%	FJ475189.1	362,45	98,7%
Select seq gb|EU001351.1|	Heracleum millefolium clone trn-yy16 tRNA-Leu (trnL) gene, partial sequence; and trnL-trnF intergenic spacer, complete sequence; chloroplast	444	444	98%	1,00E-120	80%	EU001351.1	362,45	98,7%
Select seq gb|AF444012.1|	Cymopterus nivalis tRNA-Phe gene, partial sequence; tRNA-Leu gene, complete sequence; and tRNA-Thr gene, partial sequence; chloroplast genes for chloroplast products	451	451	100%	7,00E-123	80%	AF444012.1	360,80	98,3%
Select seq gb|AF444029.1|	Pteryxia terebinthina tRNA-Phe gene, partial sequence; and tRNA-Leu gene, complete sequence; chloroplast genes for chloroplast products	449	449	100%	3,00E-122	80%	AF444029.1	359,20	97,8%
Select seq gb|AF444013.1|	Cymopterus purpureus tRNA-Phe gene, partial sequence; tRNA-Leu gene, complete sequence; and tRNA-Thr gene, partial sequence; chloroplast genes for chloroplast products	449	449	100%	3,00E-122	80%	AF444013.1	359,20	97,8%
Select seq gb|AF444011.1|	Cymopterus multinervatus tRNA-Phe gene, partial sequence; tRNA-Leu gene, complete sequence; and tRNA-Thr gene, partial sequence; chloroplast genes for chloroplast products	438	438	98%	6,00E-119	80%	AF444011.1	357,55	97,4%
Select seq gb|KR011054.1|	Foeniculum vulgare chloroplast, complete genome	436	436	98%	2,00E-118	80%	KR011054.1	355,92	96,9%
Select seq gb|JQ041833.1|	Aegopodium podagraria voucher AEPO05 tRNA-Leu (trnL) gene, complete sequence; and trnL-trnF intergenic spacer, partial sequence; plastid	427	427	96%	1,00E-115	80%	JQ041833.1	355,83	96,9%
Select seq gb|HM596072.1|	Crithmum maritimum chloroplast, complete genome	444	444	100%	1,00E-120	80%	HM596072.1	355,20	96,7%
Select seq gb|KT963036.1|	Angelica acutiloba voucher 13F-28 chloroplast, complete genome	435	435	98%	7,00E-118	80%	KT963036.1	355,10	96,7%
Select seq gb|AF444030.1|	Shoshonea pulvinata tRNA-Phe gene, partial sequence; tRNA-Leu gene, complete sequence; and tRNA-Thr gene, partial sequence; chloroplast genes for chloroplast products	435	435	98%	7,00E-118	80%	AF444030.1	355,10	96,7%
Select seq gb|KP665036.1|	Apiaceae sp. 13R31re1 tRNA-Leu (trnL) gene and trnL-trnF intergenic spacer, partial sequence; chloroplast	424	424	96%	2,00E-114	80%	KP665036.1	353,33	96,2%
Select seq gb|KP665035.1|	Apiaceae sp. 13R29re3 tRNA-Leu (trnL) gene and trnL-trnF intergenic spacer, partial sequence; chloroplast	424	424	96%	2,00E-114	80%	KP665035.1	353,33	96,2%
Select seq gb|GQ984062.1|	Coriandrum sativum tRNA-Leu (trnL) gene, partial sequence; trnL-trnF intergenic spacer, complete sequence; and tRNA-Phe (trnF) gene, partial sequence; chloroplast	440	440	100%	2,00E-119	80%	GQ984062.1	352,00	95,9%
Select seq gb|KR011055.1|	Anethum graveolens chloroplast, complete genome	431	431	98%	9,00E-117	80%	KR011055.1	351,84	95,8%
Select seq gb|AF444033.1|	Zizia aurea tRNA-Phe gene, partial sequence; tRNA-Leu gene, complete sequence; and tRNA-Thr gene, partial sequence; chloroplast genes for chloroplast products	431	431	98%	9,00E-117	80%	AF444033.1	351,84	95,8%
Select seq gb|KT963038.1|	Angelica gigas voucher 13E-39-3 chloroplast, complete genome	429	429	98%	3,00E-116	80%	KT963038.1	350,20	95,4%
Select seq gb|KT963037.1|	Angelica dahurica voucher 13T-05 chloroplast, complete genome	429	429	98%	3,00E-116	80%	KT963037.1	350,20	95,4%
Select seq gb|KT852844.1|	Ostericum grosseserratum voucher 13A-14-2 chloroplast, complete genome	429	429	98%	3,00E-116	80%	KT852844.1	350,20	95,4%
Select seq gb|KM035851.1|	Seseli montanum plastid, complete genome	429	429	98%	3,00E-116	80%	KM035851.1	350,20	95,4%
Select seq gb|AF444028.1|	Pteryxia hendersonii tRNA-Phe gene, partial sequence; tRNA-Leu gene, complete sequence; and tRNA-Thr gene, partial sequence; chloroplast genes for chloroplast products	429	429	98%	3,00E-116	80%	AF444028.1	350,20	95,4%
Select seq gb|AF444026.1|	Neoparrya lithophila tRNA-Phe gene, partial sequence; tRNA-Leu gene, complete sequence; and tRNA-Thr gene, partial sequence; chloroplast genes for chloroplast products	429	429	98%	3,00E-116	80%	AF444026.1	350,20	95,4%
Select seq gb|AF444025.1|	Musineon tenuifolium tRNA-Phe gene, partial sequence; tRNA-Leu gene, complete sequence; and tRNA-Thr gene, partial sequence; chloroplast genes for chloroplast products	429	429	98%	3,00E-116	80%	AF444025.1	350,20	95,4%
Select seq gb|AF444010.1|	Cymopterus montanus tRNA-Phe gene, partial sequence; tRNA-Leu gene, complete sequence; and tRNA-Thr gene, partial sequence; chloroplast genes for chloroplast products	429	429	98%	3,00E-116	80%	AF444010.1	350,20	95,4%
Select seq gb|AF444008.1|	Aletes anisatus tRNA-Phe gene, partial sequence; tRNA-Leu gene, complete sequence; and tRNA-Thr gene, partial sequence; chloroplast genes for chloroplast products	429	429	98%	3,00E-116	80%	AF444008.1	350,20	95,4%

Select seq emb|AM109908.1|	Cnidium officinale plastid partial tRNA-Leu gene and partial IGS	429	429	98%	3,00E-116	80%	AM109908.1	350,20	95,4%
Select seq gb|KR150197.1|	Zosima sp. Kh48 trnL-trnF intergenic spacer region, partial sequence; chloroplast	427	427	98%	1,00E-115	80%	KR150197.1	348,57	94,9%
Select seq gb|AF444019.1|	Lomatium graveolens tRNA-Phe gene, partial sequence; tRNA-Leu gene, complete sequence; and tRNA-Thr gene, partial sequence; chloroplast genes for chloroplast products	435	435	100%	7,00E-118	80%	AF444019.1	348,00	94,8%
Select seq gb|AF444032.1|	Zizia aptera tRNA-Phe gene, partial sequence; tRNA-Leu gene, complete sequence; and tRNA-Thr gene, partial sequence; chloroplast genes for chloroplast products	425	425	98%	4,00E-115	80%	AF444032.1	346,94	94,5%
Select seq gb|AF444031.1|	Thaspium trifoliatum tRNA-Phe gene, partial sequence; and tRNA-Leu gene, complete sequence; chloroplast genes for chloroplast products	425	425	98%	4,00E-115	80%	AF444031.1	346,94	94,5%
Select seq gb|KT781591.1|	Angelica decursiva voucher 13Q-02-1 chloroplast, complete genome	424	424	98%	2,00E-114	80%	KT781591.1	346,12	94,3%
Select seq gb|FJ475216.1|	Pterocyclus rivulorum tRNA-Leu (trnL) gene and trnL-trnF intergenic spacer, partial sequence; chloroplast	424	424	98%	2,00E-114	80%	FJ475216.1	346,12	94,3%
Select seq gb|EU076598.1|	Seseli squarrulosum tRNA-Leu (trnL) gene and trnF-trnL intergenic spacer, partial sequence; chromoplast	424	424	98%	2,00E-114	80%	EU076598.1	346,12	94,3%
Select seq dbj|LC072694.1|	Peucedanum praeruptorum chloroplast DNA, trnL-trnF intergenic spacer, partial sequence, isolate: LJJ15	420	420	97%	2,00E-113	79%	LC072694.1	342,06	93,2%
Select seq dbj|LC072690.1|	Libanotis buchtormensis chloroplast DNA, trnL-trnF intergenic spacer, partial sequence, isolate: LJJ11	418	418	97%	7,00E-113	79%	LC072690.1	340,43	92,7%
Select seq gb|EU001357.1|	Heracleum candicans clone trn-yy06 tRNA-Leu (trnL) gene, partial sequence; and trnL-trnF intergenic spacer, complete sequence; chloroplast	422	422	98%	6,00E-114	79%	EU001357.1	340,18	92,6%
Select seq gb|AF444018.1|	Lomatium dasycarpum tRNA-Phe gene, partial sequence; tRNA-Leu gene, complete sequence; and tRNA-Thr gene, partial sequence; chloroplast genes for chloroplast products	429	429	100%	3,00E-116	79%	AF444018.1	338,91	92,3%
Select seq gb|JQ041844.1|	Heracleum sphondylium voucher HSP3 tRNA-Leu (trnL) gene, complete sequence; and trnL-trnF intergenic spacer, partial sequence; plastid	420	420	98%	2,00E-113	79%	JQ041844.1	338,57	92,2%
Select seq gb|JQ041843.1|	Heracleum sphondylium voucher HSP1 tRNA-Leu (trnL) gene, complete sequence; and trnL-trnF intergenic spacer, partial sequence; plastid	420	420	98%	2,00E-113	79%	JQ041843.1	338,57	92,2%
Select seq gb|EU001358.1|	Heracleum stenopterum clone trn-yy05 tRNA-Leu (trnL) gene, partial sequence; and trnL-trnF intergenic spacer, complete sequence; chloroplast	420	420	98%	2,00E-113	79%	EU001358.1	338,57	92,2%
Select seq gb|EU001356.1|	Heracleum oreocharis clone trn-yy08 tRNA-Leu (trnL) gene, partial sequence; and trnL-trnF intergenic spacer, complete sequence; chloroplast	420	420	98%	2,00E-113	79%	EU001356.1	338,57	92,2%
Select seq gb|KJ157767.1|	Pastinaca sativa voucher J. Wen 9783 tRNA-Leu (trnL) gene and trnL-trnF intergenic spacer, partial sequence; chloroplast	414	414	97%	9,00E-112	79%	KJ157767.1	337,18	91,8%
Select seq gb|HQ246204.1|	Trachydium subnudum tRNA-Leu (trnL) gene, partial sequence; trnL-trnF intergenic spacer, complete sequence; and tRNA-Phe (trnF) gene, partial sequence; chloroplast	418	418	98%	7,00E-113	79%	HQ246204.1	336,96	91,8%
Select seq gb|FJ475201.1|	Hymenidium linearilobum tRNA-Leu (trnL) gene and trnL-trnF intergenic spacer, partial sequence; chloroplast	418	418	98%	7,00E-113	79%	FJ475201.1	336,96	91,8%
Select seq gb|FJ475194.1|	Pleurospermum foetens tRNA-Leu (trnL) gene and trnL-trnF intergenic spacer, partial sequence; chloroplast	418	418	98%	7,00E-113	79%	FJ475194.1	336,96	91,8%
Select seq gb|FJ475186.1|	Hymenidium apiolens tRNA-Leu (trnL) gene and trnL-trnF intergenic spacer, partial sequence; chloroplast	418	418	98%	7,00E-113	79%	FJ475186.1	336,96	91,8%
Select seq gb|EU076599.1|	Seseli mairei tRNA-Leu (trnL) gene and trnF-trnL intergenic spacer, partial sequence; chromoplast	418	418	98%	7,00E-113	79%	EU076599.1	336,96	91,8%
Select seq gb|AF444024.1|	Musineon divaricatum tRNA-Phe gene, partial sequence; tRNA-Leu gene, complete sequence; and tRNA-Thr gene, partial sequence; chloroplast genes for chloroplast products	418	418	98%	7,00E-113	79%	AF444024.1	336,96	91,8%
Select seq gb|AF444020.1|	Lomatium junceum tRNA-Phe gene, partial sequence; tRNA-Leu gene, complete sequence; and tRNA-Thr gene, partial sequence; chloroplast genes for chloroplast products	418	418	98%	7,00E-113	79%	AF444020.1	336,96	91,8%
Select seq gb|AF444017.1|	Lomatium californicum tRNA-Phe gene, partial sequence; tRNA-Leu gene, complete sequence; and tRNA-Thr gene, partial sequence; chloroplast genes for chloroplast products	418	418	98%	7,00E-113	79%	AF444017.1	336,96	91,8%
Select seq gb|AF444014.1|	Cymopterus williamsii tRNA-Phe gene, partial sequence; tRNA-Leu gene, complete sequence; and tRNA-Thr gene, partial sequence; chloroplast genes for chloroplast products	418	418	98%	7,00E-113	79%	AF444014.1	336,96	91,8%
Select seq gb|AF444007.1|	Angelica archangelica tRNA-Phe gene, partial sequence; tRNA-Leu gene, complete sequence; and tRNA-Thr gene, partial sequence; chloroplast genes for chloroplast products	418	418	98%	7,00E-113	79%	AF444007.1	336,96	91,8%
Select seq emb|AM109910.1|	Dystaenia ibukiensis plastid partial tRNA-Leu gene and partial IGS, isolate 7	418	418	98%	7,00E-113	79%	AM109910.1	336,96	91,8%

Select seq gb|KP665033.1|	Angelica sinensis isolate 13R17 tRNA-Leu (trnL) gene and trnL-trnF intergenic spacer, partial sequence; chloroplast	409	409	96%	4,00E-110	79% KP665033.1	336,57	91,7%
Select seq gb|KP664978.1|	Angelica decursiva isolate 13Q18 tRNA-Leu (trnL) gene and trnL-trnF intergenic spacer, partial sequence; chloroplast	409	409	96%	4,00E-110	79% KP664978.1	336,57	91,7%
Select seq gb|KP664977.1|	Angelica decursiva isolate 13Q16 tRNA-Leu (trnL) gene and trnL-trnF intergenic spacer, partial sequence; chloroplast	409	409	96%	4,00E-110	79% KP664977.1	336,57	91,7%
Select seq gb|KP664976.1|	Angelica decursiva isolate 13Q15 tRNA-Leu (trnL) gene and trnL-trnF intergenic spacer, partial sequence; chloroplast	409	409	96%	4,00E-110	79% KP664976.1	336,57	91,7%
Select seq gb|KP664975.1|	Angelica decursiva isolate 13Q13 tRNA-Leu (trnL) gene and trnL-trnF intergenic spacer, partial sequence; chloroplast	409	409	96%	4,00E-110	79% KP664975.1	336,57	91,7%
Select seq gb|KP664974.1|	Angelica decursiva isolate 13Q12 tRNA-Leu (trnL) gene and trnL-trnF intergenic spacer, partial sequence; chloroplast	409	409	96%	4,00E-110	79% KP664974.1	336,57	91,7%
Select seq gb|KP664973.1|	Angelica decursiva isolate 13Q11 tRNA-Leu (trnL) gene and trnL-trnF intergenic spacer, partial sequence; chloroplast	409	409	96%	4,00E-110	79% KP664973.1	336,57	91,7%
Select seq gb|KP664972.1|	Angelica decursiva isolate 13Q10 tRNA-Leu (trnL) gene and trnL-trnF intergenic spacer, partial sequence; chloroplast	409	409	96%	4,00E-110	79% KP664972.1	336,57	91,7%
Select seq gb|KP664971.1|	Angelica decursiva isolate 13Q09 tRNA-Leu (trnL) gene and trnL-trnF intergenic spacer, partial sequence; chloroplast	409	409	96%	4,00E-110	79% KP664971.1	336,57	91,7%
Select seq gb|KP664970.1|	Angelica decursiva isolate 13Q08 tRNA-Leu (trnL) gene and trnL-trnF intergenic spacer, partial sequence; chloroplast	409	409	96%	4,00E-110	79% KP664970.1	336,57	91,7%
Select seq gb|KP664969.1|	Angelica decursiva isolate 13Q06 tRNA-Leu (trnL) gene and trnL-trnF intergenic spacer, partial sequence; chloroplast	409	409	96%	4,00E-110	79% KP664969.1	336,57	91,7%
Select seq gb|KP664968.1|	Angelica decursiva isolate 13Q05 tRNA-Leu (trnL) gene and trnL-trnF intergenic spacer, partial sequence; chloroplast	409	409	96%	4,00E-110	79% KP664968.1	336,57	91,7%
Select seq gb|KP664967.1|	Angelica decursiva isolate 13Q04 tRNA-Leu (trnL) gene and trnL-trnF intergenic spacer, partial sequence; chloroplast	409	409	96%	4,00E-110	79% KP664967.1	336,57	91,7%
Select seq gb|KP664966.1|	Angelica decursiva isolate 13Q03 tRNA-Leu (trnL) gene and trnL-trnF intergenic spacer, partial sequence; chloroplast	409	409	96%	4,00E-110	79% KP664966.1	336,57	91,7%
Select seq gb|KP664965.1|	Angelica decursiva isolate 13Q01 tRNA-Leu (trnL) gene and trnL-trnF intergenic spacer, partial sequence; chloroplast	409	409	96%	4,00E-110	79% KP664965.1	336,57	91,7%
Select seq gb|FJ475203.1|	Hymenidium stellatum tRNA-Leu (trnL) gene and trnL-trnF intergenic spacer, partial sequence; chloroplast	409	409	96%	4,00E-110	79% FJ475203.1	336,57	91,7%
Select seq dbj|LC072693.1|	Libanotis spodotrichoma chloroplast DNA, trnL-trnF intergenic spacer, partial sequence, isolate: LJJ14	412	412	97%	3,00E-111	79% LC072693.1	335,55	91,4%
Select seq dbj|LC072692.1|	Libanotis buchtormensis chloroplast DNA, trnL-trnF intergenic spacer, partial sequence, isolate: LJJ13	412	412	97%	3,00E-111	79% LC072692.1	335,55	91,4%
Select seq gb|KJ157768.1|	Pastinaca sp. Tibet 1326 tRNA-Leu (trnL) gene and trnL-trnF intergenic spacer, partial sequence; chloroplast	412	412	97%	3,00E-111	79% KJ157768.1	335,55	91,4%
Select seq gb|EU001361.1|	Heracleum forrestii clone trn-yy01 tRNA-Leu (trnL) gene, partial sequence; and trnL-trnF intergenic spacer, complete sequence; chloroplast	416	416	98%	3,00E-112	79% EU001361.1	335,35	91,3%
Select seq gb|AF444015.1|	Harbouria trachypleura tRNA-Phe gene, partial sequence; tRNA-Leu gene, complete sequence; and tRNA-Thr gene, partial sequence; chloroplast genes for chloroplast products	416	416	98%	3,00E-112	79% AF444015.1	335,35	91,3%
Select seq gb|EU001353.1|	Angelica apaensis clone trn-yy11 tRNA-Leu (trnL) gene, partial sequence; and trnL-trnF intergenic spacer, complete sequence; chloroplast	414	414	98%	9,00E-112	79% EU001353.1	333,73	90,9%
Select seq gb|GQ351360.1|	Peucedanum japonicum tRNA-Leu (trnL) gene, partial sequence; trnL-trnF intergenic spacer, complete sequence; and tRNA-Phe (trnF) gene, partial sequence; chloroplast	409	409	97%	4,00E-110	79% GQ351360.1	333,10	90,7%
Select seq gb|HQ246201.1|	Sinodielsia thibetica tRNA-Leu (trnL) gene and trnL-trnF intergenic spacer, complete sequence; and tRNA-Phe (trnF) gene, partial sequence; chloroplast	412	412	98%	3,00E-111	79% HQ246201.1	332,12	90,4%
Select seq gb|AF444023.1|	Lomatium nudicaule tRNA-Phe gene, partial sequence; tRNA-Leu gene, complete sequence; and tRNA-Thr gene, partial sequence; chloroplast genes for chloroplast products	412	412	98%	3,00E-111	79% AF444023.1	332,12	90,4%
Select seq gb|FJ475206.1|	Hymenidium wilsonii tRNA-Leu (trnL) gene and trnL-trnF intergenic spacer, partial sequence; chloroplast	420	420	100%	2,00E-113	79% FJ475206.1	331,80	90,4%
Select seq gb|FJ475198.1|	Hymenidium huzhihaoi tRNA-Leu (trnL) gene and trnL-trnF intergenic spacer, partial sequence; chloroplast	420	420	100%	2,00E-113	79% FJ475198.1	331,80	90,4%
Select seq gb|KP665009.1|	Peucedanum praeruptorum isolate 13R37 tRNA-Leu (trnL) gene and trnL-trnF intergenic spacer, partial sequence; chloroplast	403	403	96%	2,00E-108	79% KP665009.1	331,64	90,3%

Select seq gb|KP665008.1|	Peucedanum praeruptorum isolate 13R36 tRNA-Leu (trnL) gene and trnL-trnF intergenic spacer, partial sequence; chloroplast	403	403	96%	2,00E-108	79%	KP665008.1	331,64	90,3%
Select seq gb|KP665007.1|	Peucedanum praeruptorum isolate 13R35 tRNA-Leu (trnL) gene and trnL-trnF intergenic spacer, partial sequence; chloroplast	403	403	96%	2,00E-108	79%	KP665007.1	331,64	90,3%
Select seq gb|KP665006.1|	Peucedanum praeruptorum isolate 13R34 tRNA-Leu (trnL) gene and trnL-trnF intergenic spacer, partial sequence; chloroplast	403	403	96%	2,00E-108	79%	KP665006.1	331,64	90,3%
Select seq gb|KP665005.1|	Peucedanum praeruptorum isolate 13R31re2 tRNA-Leu (trnL) gene and trnL-trnF intergenic spacer, partial sequence; chloroplast	403	403	96%	2,00E-108	79%	KP665005.1	331,64	90,3%
Select seq gb|KP665004.1|	Peucedanum praeruptorum isolate 13R29re2 tRNA-Leu (trnL) gene and trnL-trnF intergenic spacer, partial sequence; chloroplast	403	403	96%	2,00E-108	79%	KP665004.1	331,64	90,3%
Select seq gb|KP665003.1|	Peucedanum praeruptorum isolate 13R28 tRNA-Leu (trnL) gene and trnL-trnF intergenic spacer, partial sequence; chloroplast	403	403	96%	2,00E-108	79%	KP665003.1	331,64	90,3%
Select seq gb|EU001352.1|	Heracleum moellendorffii clone trn-yy15 tRNA-Leu (trnL) gene, partial sequence; and trnL-trnF intergenic spacer, complete sequence; chloroplast	411	411	98%	1,00E-110	79%	EU001352.1	331,32	90,2%
Select seq gb|KJ157726.1|	Heracleum maximum voucher J. Wen 10391 tRNA-Leu (trnL) gene and trnL-trnF intergenic spacer, partial sequence; chloroplast	405	405	97%	6,00E-109	79%	KJ157726.1	329,85	89,8%
Select seq gb|FJ475185.1|	Hymenidium amabile tRNA-Leu (trnL) gene and trnL-trnF intergenic spacer, partial sequence; chloroplast	409	409	98%	4,00E-110	79%	FJ475185.1	329,70	89,8%
Select seq gb|KM035850.1|	Pastinaca pimpinellifolia voucher M.G. Pimenov et al., 117 (MW) plastid, complete genome	407	407	98%	2,00E-109	79%	KM035850.1	328,09	89,3%
Select seq gb|FJ475215.1|	Pterocyclus angelicoides tRNA-Leu (trnL) gene and trnL-trnF intergenic spacer, partial sequence; chloroplast	407	407	98%	2,00E-109	79%	FJ475215.1	328,09	89,3%
Select seq gb|FJ475204.1|	Hymenidium virgatum tRNA-Leu (trnL) gene and trnL-trnF intergenic spacer, partial sequence; chloroplast	414	414	100%	9,00E-112	79%	FJ475204.1	327,06	89,1%
Select seq gb|HQ246202.1|	Trachydium involucellatum tRNA-Leu (trnL) gene, partial sequence; trnL-trnF intergenic spacer, complete sequence; and tRNA-Phe (trnF) gene, partial sequence; chloroplast	405	405	98%	6,00E-109	79%	HQ246202.1	326,48	88,9%
Select seq gb|AF444027.1|	Podistera eastwoodiae tRNA-Phe gene, partial sequence; tRNA-Leu gene, complete sequence; and tRNA-Thr gene, partial sequence; chloroplast genes for chloroplast products	405	405	98%	6,00E-109	79%	AF444027.1	326,48	88,9%
								0,00	0,0%
								0,00	0,0%
								0,00	0,0%

Select for downloading or viewing reports	Kh048_ITS Description	Max score	Total score	Query cover	E value	Ident	Accession	(Ident/Cover)* Max score	Deviation from top hit
Select seq gb|EU185647.1|	Zosima absinthifolia isolate EDNA06-4855 internal transcribed spacer 1, partial sequence; 5.8S ribosomal RNA gene, complete sequence; and internal transcribed spacer 2, partial sequence	1024	1024	84%	0.0	98%	EU185647.1	1194,67	100,0%
Select seq gb|EU185648.1|	Zosima radians isolate EDNA06-4854 internal transcribed spacer 1, partial sequence; 5.8S ribosomal RNA gene, complete sequence; and internal transcribed spacer 2, partial sequence	1013	1013	84%	0.0	97%	EU185648.1	1169,77	97,9%
Select seq gb|AF008628.2|	Zosima orientalis internal transcribed spacer 1, 5.8S ribosomal RNA gene, and internal transcribed spacer 2, complete sequence	1007	1007	84%	0.0	97%	AF008628.2	1162,85	97,3%
Select seq gb|EU169332.1|	Zosima absinthifolia voucher Constance C-635 (ISU 1143) internal transcribed spacer 1, partial sequence; 5.8S ribosomal RNA gene, complete sequence; and internal transcribed spacer 2, partial sequence	996	996	84%	0.0	97%	EU169332.1	1150,14	96,3%
Select seq gb|HQ686429.1|	Heracleum franchetii isolate I_HEXJ0106 internal transcribed spacer 1, 5.8S ribosomal RNA gene, and internal transcribed spacer 2, complete sequence	937	937	84%	0.0	95%	HQ686429.1	1059,70	88,7%
Select seq gb|FJ812130.1|	Heracleum millefolium var. longilobum isolate H25 internal transcribed spacer 1, 5.8S ribosomal RNA gene, and internal transcribed spacer 2, complete sequence	931	931	84%	0.0	95%	FJ812130.1	1052,92	88,1%
Select seq gb|DQ427052.1|	Kandaharia rechingerorum internal transcribed spacer 1, 5.8S ribosomal RNA gene, and internal transcribed spacer 2, complete sequence	931	931	84%	0.0	95%	DQ427052.1	1052,92	88,1%
Select seq gb|EU185642.1|	Tordyliopsis brunonis isolate EDNA06-4906 internal transcribed spacer 1, partial sequence; 5.8S ribosomal RNA gene, complete sequence; and internal transcribed spacer 2, partial sequence	931	931	84%	0.0	95%	EU185642.1	1052,92	88,1%
Select seq gb|EU236168.1|	Heracleum bivittatum voucher ZJ0611 (KUN) internal transcribed spacer 1, 5.8S ribosomal RNA gene, and internal transcribed spacer 2, complete sequence	929	929	84%	0.0	95%	EU236168.1	1050,65	87,9%
Select seq gb|EU185655.1|	Heracleum millefolium isolate EDNA06-4912 internal transcribed spacer 1, partial sequence; 5.8S ribosomal RNA gene, complete sequence; and internal transcribed spacer 2, partial sequence	917	917	83%	0.0	95%	EU185655.1	1049,58	87,9%
Select seq gb|DQ427039.1|	Semenovia frigida internal transcribed spacer 1, 5.8S ribosomal RNA gene, and internal transcribed spacer 2, complete sequence	928	928	84%	0.0	95%	DQ427039.1	1049,52	87,9%
Select seq gb|EU236170.1|	Heracleum millefolium voucher ZJ0569 (KUN) internal transcribed spacer 1, 5.8S ribosomal RNA gene, and internal transcribed spacer 2, complete sequence	926	926	84%	0.0	95%	EU236170.1	1047,26	87,7%
Select seq gb|HQ686489.1|	Heracleum candicans isolate I_HEXJ0168 internal transcribed spacer 1, 5.8S ribosomal RNA gene, and internal transcribed spacer 2, complete sequence	918	918	84%	0.0	94%	HQ686489.1	1027,29	86,0%
Select seq gb|FJ812152.1|	Semenovia tragioides isolate St internal transcribed spacer 1, 5.8S ribosomal RNA gene, and internal transcribed spacer 2, complete sequence	917	917	84%	0.0	94%	FJ812152.1	1026,17	85,9%
Select seq gb|HQ686450.1|	Heracleum yungningense isolate I_HEXJ0127 internal transcribed spacer 1, 5.8S ribosomal RNA gene, and internal transcribed spacer 2, complete sequence	915	915	84%	0.0	94%	HQ686450.1	1023,93	85,7%
Select seq gb|EU001362.1|	Heracleum millefolium clone its-yy16 internal transcribed spacer 1, 5.8S ribosomal RNA gene, and internal transcribed spacer 2, complete sequence	915	915	84%	0.0	94%	EU001362.1	1023,93	85,7%
Select seq gb|HQ686419.1|	Heracleum moellendorffii isolate I_HEXJ0096 internal transcribed spacer 1, 5.8S ribosomal RNA gene, and internal transcribed spacer 2, complete sequence	913	913	84%	0.0	94%	HQ686419.1	1021,69	85,5%
Select seq gb|EU185644.1|	Heracleum obtusifolium isolate EDNA06-4915 internal transcribed spacer 1, partial sequence; 5.8S ribosomal RNA gene, complete sequence; and internal transcribed spacer 2, partial sequence	913	913	84%	0.0	94%	EU185644.1	1021,69	85,5%
Select seq gb|FJ812117.1|	Heracleum millefolium var. millefolium isolate H07 internal transcribed spacer 1, 5.8S ribosomal RNA gene, and internal transcribed spacer 2, complete sequence	909	909	84%	0.0	94%	FJ812117.1	1017,21	85,1%
Select seq gb|EU185672.1|	Heracleum pinnatum isolate EDNA06-4885 internal transcribed spacer 1, partial sequence; 5.8S ribosomal RNA gene, complete sequence; and internal transcribed spacer 2, partial sequence	898	898	83%	0.0	94%	EU185672.1	1017,01	85,1%
Select seq gb|HQ686486.1|	Heracleum candicans isolate I_HEXJ0165 internal transcribed spacer 1, 5.8S ribosomal RNA gene, and internal transcribed spacer 2, complete sequence	907	907	84%	0.0	94%	HQ686486.1	1014,98	85,0%
Select seq gb|FJ986044.1|	Heracleum obtusifolium internal transcribed spacer 1, partial sequence; 5.8S ribosomal RNA gene, complete sequence; and internal transcribed spacer 2, partial sequence	907	907	84%	0.0	94%	FJ986044.1	1014,98	85,0%
Select seq gb|FJ812121.1|	Heracleum candicans var. candicans isolate H13 internal transcribed spacer 1, 5.8S ribosomal RNA gene, and internal transcribed spacer 2, complete sequence	907	907	84%	0.0	94%	FJ812121.1	1014,98	85,0%
Select seq gb|DQ427040.1|	Semenovia radiata internal transcribed spacer 1, 5.8S ribosomal RNA gene, and internal transcribed spacer 2, complete sequence	907	907	84%	0.0	94%	DQ427040.1	1014,98	85,0%
Select seq gb|EU185671.1|	Heracleum obtusifolium isolate EDNA06-4916 internal transcribed spacer 1, partial sequence; 5.8S ribosomal RNA gene, complete sequence; and internal transcribed spacer 2, partial sequence	907	907	84%	0.0	94%	EU185671.1	1014,98	85,0%
Select seq gb|DQ516378.1|	Heracleum candicans isolate 1312 internal transcribed spacer 1, 5.8S ribosomal RNA gene, and internal transcribed spacer 2, complete sequence	907	907	84%	0.0	94%	DQ516378.1	1014,98	85,0%
Select seq gb|DQ427034.1|	Tetrataenium pinnatum internal transcribed spacer 1, 5.8S ribosomal RNA gene, and internal transcribed spacer 2, complete sequence	905	905	84%	0.0	94%	DQ427034.1	1012,74	84,8%

Select seq gb|EU169276.1|	Heracleum candicans voucher cult. RBGE 19943858 internal transcribed spacer 1, partial sequence; 5.8S ribosomal RNA gene, complete sequence; and internal transcribed spacer 2, partial sequence	904	904	84% 0.0	94% EU169276.1	1011,62	84,7%
Select seq gb|HQ686484.1|	Heracleum candicans isolate I_HEXJ0163 internal transcribed spacer 1, 5.8S ribosomal RNA gene, and internal transcribed spacer 2, complete sequence	902	902	84% 0.0	94% HQ686484.1	1009,38	84,5%
Select seq gb|HQ686411.1|	Heracleum candicans isolate I_HEXJ0088 internal transcribed spacer 1, 5.8S ribosomal RNA gene, and internal transcribed spacer 2, complete sequence	902	902	84% 0.0	94% HQ686411.1	1009,38	84,5%
Select seq gb|HQ686409.1|	Heracleum candicans isolate I_HEXJ0086 internal transcribed spacer 1, 5.8S ribosomal RNA gene, and internal transcribed spacer 2, complete sequence	902	902	84% 0.0	94% HQ686409.1	1009,38	84,5%
Select seq gb|HQ686385.1|	Heracleum candicans isolate I_HEXJ0062 internal transcribed spacer 1, 5.8S ribosomal RNA gene, and internal transcribed spacer 2, complete sequence	902	902	84% 0.0	94% HQ686385.1	1009,38	84,5%
Select seq gb|EU185643.1|	Heracleum candicans isolate EDNA06-4824 internal transcribed spacer 1, partial sequence; 5.8S ribosomal RNA gene, complete sequence; and internal transcribed spacer 2, partial sequence	902	902	84% 0.0	94% EU185643.1	1009,38	84,5%
Select seq gb|EU001370.1|	Heracleum obtusifolium clone its-yy04 internal transcribed spacer 1, 5.8S ribosomal RNA gene, and internal transcribed spacer 2, complete sequence	902	902	84% 0.0	94% EU001370.1	1009,38	84,5%
Select seq gb|EU001366.1|	Heracleum dissectifolium clone its-yy09 internal transcribed spacer 1, 5.8S ribosomal RNA gene, and internal transcribed spacer 2, complete sequence	898	898	84% 0.0	94% EU001366.1	1004,90	84,1%
Select seq gb|HQ686442.1|	Heracleum candicans isolate I_HEXJ0119 internal transcribed spacer 1, 5.8S ribosomal RNA gene, and internal transcribed spacer 2, complete sequence	896	896	84% 0.0	94% HQ686442.1	1002,67	83,9%
Select seq gb|HQ686386.1|	Heracleum candicans isolate I_HEXJ0063 internal transcribed spacer 1, 5.8S ribosomal RNA gene, and internal transcribed spacer 2, complete sequence	896	896	84% 0.0	94% HQ686386.1	1002,67	83,9%
Select seq gb|GU967802.1|	Heracleum obtusifolium isolate 40 internal transcribed spacer 1, 5.8S ribosomal RNA gene, and internal transcribed spacer 2, complete sequence	896	896	84% 0.0	94% GU967802.1	1002,67	83,9%
Select seq gb|FJ812113.1|	Heracleum candicans var. candicans isolate H03 internal transcribed spacer 1, 5.8S ribosomal RNA gene, and internal transcribed spacer 2, complete sequence	896	896	84% 0.0	94% FJ812113.1	1002,67	83,9%
Select seq gb|EU625291.1|	Tetrataenium canescens internal transcribed spacer 1, 5.8S ribosomal RNA gene, and internal transcribed spacer 2, complete sequence	896	896	84% 0.0	94% EU625291.1	1002,67	83,9%
Select seq gb|EU185678.1|	Heracleum wallichii isolate EDNA06-4942 internal transcribed spacer 1, partial sequence; 5.8S ribosomal RNA gene, complete sequence; and internal transcribed spacer 2, partial sequence	896	896	84% 0.0	94% EU185678.1	1002,67	83,9%
Select seq gb|FJ812151.1|	Semenovia dichotoma isolate Sd internal transcribed spacer 1, 5.8S ribosomal RNA gene, and internal transcribed spacer 2, complete sequence	885	885	83% 0.0	94% FJ812151.1	1002,29	83,9%
Select seq gb|HQ686430.1|	Heracleum candicans isolate I_HEXJ0107 internal transcribed spacer 1, 5.8S ribosomal RNA gene, and internal transcribed spacer 2, complete sequence	894	894	84% 0.0	94% HQ686430.1	1000,43	83,7%
Select seq gb|HQ686410.1|	Heracleum candicans isolate I_HEXJ0087 internal transcribed spacer 1, 5.8S ribosomal RNA gene, and internal transcribed spacer 2, complete sequence	894	894	84% 0.0	94% HQ686410.1	1000,43	83,7%
Select seq gb|DQ427041.1|	Heracleum argaeum internal transcribed spacer 1, 5.8S ribosomal RNA gene, and internal transcribed spacer 2, complete sequence	894	894	84% 0.0	94% DQ427041.1	1000,43	83,7%
Select seq gb|EU169281.1|	Heracleum pastinacifolium subsp. incanum voucher Spalik s.n. (WA) internal transcribed spacer 1, partial sequence; 5.8S ribosomal RNA gene, complete sequence; and internal transcribed spacer 2, partial sequence	893	893	84% 0.0	94% EU169281.1	999,31	83,6%
Select seq gb|EU169275.1|	Heracleum apiifolium voucher Spalik s.n. (WA) internal transcribed spacer 1, partial sequence; 5.8S ribosomal RNA gene, complete sequence; and internal transcribed spacer 2, partial sequence	893	893	84% 0.0	94% EU169275.1	999,31	83,6%
Select seq gb|DQ468073.1|	Heracleum transcaucasicum internal transcribed spacer 1, 5.8S ribosomal RNA gene, and internal transcribed spacer 2, complete sequence	893	893	84% 0.0	94% DQ468073.1	999,31	83,6%
Select seq gb|HQ686482.1|	Tetrataenium canescens isolate I_HEXJ0161 internal transcribed spacer 1, 5.8S ribosomal RNA gene, and internal transcribed spacer 2, complete sequence	891	891	84% 0.0	94% HQ686482.1	997,07	83,5%
Select seq gb|GU967801.1|	Heracleum olgae isolate 183 internal transcribed spacer 1, 5.8S ribosomal RNA gene, and internal transcribed spacer 2, complete sequence	891	891	84% 0.0	94% GU967801.1	997,07	83,5%
Select seq gb|EU185677.1|	Heracleum wallichii isolate EDNA06-4844 internal transcribed spacer 1, partial sequence; 5.8S ribosomal RNA gene, complete sequence; and internal transcribed spacer 2, partial sequence	891	891	84% 0.0	94% EU185677.1	997,07	83,5%
Select seq gb|DQ427046.1|	Heracleum scabrum internal transcribed spacer 1, 5.8S ribosomal RNA gene, and internal transcribed spacer 2, complete sequence	889	889	84% 0.0	94% DQ427046.1	994,83	83,3%
Select seq gb|DQ468074.1|	Heracleum roseum internal transcribed spacer 1, 5.8S ribosomal RNA gene, and internal transcribed spacer 2, complete sequence	887	887	84% 0.0	93% DQ468074.1	982,04	82,2%
Select seq gb|EF043025.1|	Heracleum schelkovnikovii voucher MW 1217 internal transcribed spacer 1, 5.8S ribosomal RNA gene, and internal transcribed spacer 2, complete sequence	887	887	84% 0.0	93% EF043025.1	982,04	82,2%
Select seq gb|DQ427049.1|	Heracleum chorodanum internal transcribed spacer 1, 5.8S ribosomal RNA gene, and internal transcribed spacer 2, complete sequence	887	887	84% 0.0	93% DQ427049.1	982,04	82,2%

Select seq gb|HQ686432.1|	Heracleum franchetii isolate I_HEXJ0109 internal transcribed spacer 1, 5.8S ribosomal RNA gene, and internal transcribed spacer 2, complete sequence	885	885	84%	0.0	93%	HQ686432.1	979,82	82,0%
Select seq gb|HQ686417.1|	Heracleum candicans isolate I_HEXJ0094 internal transcribed spacer 1, 5.8S ribosomal RNA gene, and internal transcribed spacer 2, complete sequence	885	885	84%	0.0	93%	HQ686417.1	979,82	82,0%
Select seq gb|EU185673.1|	Heracleum canescens isolate EDNA06-4910 internal transcribed spacer 1, partial sequence; 5.8S ribosomal RNA gene, complete sequence; and internal transcribed spacer 2, partial sequence	885	885	84%	0.0	93%	EU185673.1	979,82	82,0%
Select seq gb|EU185645.1|	Semenovia dasycarpa isolate EDNA06-4882 internal transcribed spacer 1, partial sequence; 5.8S ribosomal RNA gene, complete sequence; and internal transcribed spacer 2, partial sequence	885	885	84%	0.0	93%	EU185645.1	979,82	82,0%
Select seq gb|HQ686491.1|	Tetrataenium canescens isolate I_HEXJ0170 internal transcribed spacer 1, 5.8S ribosomal RNA gene, and internal transcribed spacer 2, complete sequence	881	881	84%	0.0	93%	HQ686491.1	975,39	81,6%
Select seq gb|DQ427035.1|	Tetrataenium canescens internal transcribed spacer 1, 5.8S ribosomal RNA gene, and internal transcribed spacer 2, complete sequence	881	881	84%	0.0	93%	DQ427035.1	975,39	81,6%
Select seq gb|EU185646.1|	Semenovia transiliensis isolate EDNA06-4881 internal transcribed spacer 1, partial sequence; 5.8S ribosomal RNA gene, complete sequence; and internal transcribed spacer 2, partial sequence	881	881	84%	0.0	93%	EU185646.1	975,39	81,6%
Select seq gb|HQ686495.1|	Heracleum nyalamense isolate I_HEXJ0177 internal transcribed spacer 1, 5.8S ribosomal RNA gene, and internal transcribed spacer 2, complete sequence	880	880	84%	0.0	93%	HQ686495.1	974,29	81,6%
Select seq gb|HQ686448.1|	Tetrataenium canescens isolate I_HEXJ0125 internal transcribed spacer 1, 5.8S ribosomal RNA gene, and internal transcribed spacer 2, complete sequence	880	880	84%	0.0	93%	HQ686448.1	974,29	81,6%
Select seq gb|HQ686402.1|	Heracleum candicans isolate I_HEXJ0079 internal transcribed spacer 1, 5.8S ribosomal RNA gene, and internal transcribed spacer 2, complete sequence	880	880	84%	0.0	93%	HQ686402.1	974,29	81,6%
Select seq gb|EU185654.1|	Heracleum sublineare isolate EDNA06-4874 internal transcribed spacer 1, partial sequence; 5.8S ribosomal RNA gene, complete sequence; and internal transcribed spacer 2, partial sequence	880	880	84%	0.0	93%	EU185654.1	974,29	81,6%
Select seq gb|AY548226.1|	Heracleum moellendorffii isolate um15 18S ribosomal RNA gene, partial sequence; internal transcribed spacer 1, 5.8S ribosomal RNA gene, and internal transcribed spacer 2, complete sequence; and 26S ribosomal RNA gene, partial sequence	974	974	93%	0.0	93%	AY548226.1	974,00	81,5%
Select seq gb|HQ686485.1|	Tetrataenium canescens isolate I_HEXJ0164 internal transcribed spacer 1, 5.8S ribosomal RNA gene, and internal transcribed spacer 2, complete sequence	878	878	84%	0.0	93%	HQ686485.1	972,07	81,4%
Select seq gb|FJ812111.1|	Heracleum hemsleyanum isolate H01 internal transcribed spacer 1, 5.8S ribosomal RNA gene, and internal transcribed spacer 2, complete sequence	876	876	84%	0.0	93%	FJ812111.1	969,86	81,2%
Select seq gb|FJ385060.1|	Semenovia dasycarpa voucher J108 (XAU) internal transcribed spacer 1, partial sequence; and 5.8S ribosomal RNA gene and internal transcribed spacer 2, complete sequence	876	876	84%	0.0	93%	FJ385060.1	969,86	81,2%
Select seq gb|EU594897.1|	Heracleum ligusticifolium isolate 5-1 internal transcribed spacer 1, 5.8S ribosomal RNA gene, and internal transcribed spacer 2, complete sequence	876	876	84%	0.0	93%	EU594897.1	969,86	81,2%
Select seq gb|DQ427050.1|	Heracleum apiifolium internal transcribed spacer 1, 5.8S ribosomal RNA gene, and internal transcribed spacer 2, complete sequence	876	876	84%	0.0	93%	DQ427050.1	969,86	81,2%
Select seq gb|KP738154.1|	Verbascum macrocarpum voucher 2845 (VANF) 18S ribosomal RNA gene, partial sequence; internal transcribed spacer 1, 5.8S ribosomal RNA gene, and internal transcribed spacer 2, complete sequence; and 28S ribosomal RNA gene, partial sequence	1042	1042	100%	0.0	93%	KP738154.1	969,06	81,1%
Select seq gb|HQ686483.1|	Heracleum nyalamense isolate I_HEXJ0162 internal transcribed spacer 1, 5.8S ribosomal RNA gene, and internal transcribed spacer 2, complete sequence	874	874	84%	0.0	93%	HQ686483.1	967,64	81,0%
Select seq gb|EU185651.1|	Heracleum nepalense isolate EDNA06-4913 internal transcribed spacer 1, partial sequence; 5.8S ribosomal RNA gene, complete sequence; and internal transcribed spacer 2, partial sequence	874	874	84%	0.0	93%	EU185651.1	967,64	81,0%
Select seq gb|HQ686415.1|	Heracleum moellendorffii isolate I_HEXJ0092 internal transcribed spacer 1, 5.8S ribosomal RNA gene, and internal transcribed spacer 2, complete sequence	872	872	84%	0.0	93%	HQ686415.1	965,43	80,8%
Select seq gb|DQ427044.1|	Lalldhwojia acronemifolia internal transcribed spacer 1, 5.8S ribosomal RNA gene, and internal transcribed spacer 2, complete sequence	880	880	85%	0.0	93%	DQ427044.1	962,82	80,6%
Select seq gb|HQ686412.1|	Heracleum moellendorffii isolate I_HEXJ0089 internal transcribed spacer 1, 5.8S ribosomal RNA gene, and internal transcribed spacer 2, complete sequence	869	869	84%	0.0	93%	HQ686412.1	962,11	80,5%
Select seq gb|KJ157633.1|	Heracleum maximum voucher J. Wen 10391 18S ribosomal RNA gene, partial sequence; internal transcribed spacer 1, 5.8S ribosomal RNA gene, and internal transcribed spacer 2, complete sequence; and 28S ribosomal RNA gene, partial sequence	887	887	86%	0.0	93%	KJ157633.1	959,20	80,3%
Select seq gb|FJ481926.1|	Ligusticum sp. DCY-02 18S ribosomal RNA gene, partial sequence; internal transcribed spacer 1, 5.8S ribosomal RNA gene, and internal transcribed spacer 2, complete sequence; and 28S ribosomal RNA gene, partial sequence	965	965	100%	0.0	91%	FJ481926.1	878,15	73,5%
Select seq gb|KP058314.1|	Ligusticum tenuissimum voucher JKTM-1-000065 18S ribosomal RNA gene, partial sequence; internal transcribed spacer 1, 5.8S ribosomal RNA gene, and internal transcribed spacer 2, complete sequence; and 26S ribosomal RNA gene, partial sequence	939	939	98%	0.0	91%	KP058314.1	871,93	73,0%

Select seq gb|FJ481925.1|	Ligusticum tenuissimum isolate DCY-01 18S ribosomal RNA gene, partial sequence; internal transcribed spacer 1, 5.8S ribosomal RNA gene, and internal transcribed spacer 2, complete sequence; and 28S ribosomal RNA gene, partial sequence	953	953	100% 0.0	91% FJ481925.1	867,23	72,6%
Select seq gb|GU289653.1|	Angelica sinensis voucher ZX0811026 18S ribosomal RNA gene, partial sequence; internal transcribed spacer 1, 5.8S ribosomal RNA gene, and internal transcribed spacer 2, complete sequence; and 26S ribosomal RNA gene, partial sequence	905	905	95% 0.0	91% GU289653.1	866,89	72,6%
Select seq gb|AF393784.1|AF393784	Angelica sinensis 18S ribosomal RNA gene, partial sequence; internal transcribed spacer 1, 5.8S ribosomal RNA gene and internal transcribed spacer 2, complete sequence; and 26S ribosomal RNA gene, partial sequence	880	880	93% 0.0	91% AF393784.1	861,08	72,1%
Select seq gb|JX022936.1|	Angelica sinensis voucher HLQA10045-01(KUN) 18S ribosomal RNA gene, partial sequence; internal transcribed spacer 1, 5.8S ribosomal RNA gene, and internal transcribed spacer 2, complete sequence; and 26S ribosomal RNA gene, partial sequence	887	887	94% 0.0	91% JX022936.1	858,69	71,9%
Select seq gb|GU289654.1|	Angelica sinensis voucher ZX0811028 18S ribosomal RNA gene, partial sequence; internal transcribed spacer 1, 5.8S ribosomal RNA gene, and internal transcribed spacer 2, complete sequence; and 26S ribosomal RNA gene, partial sequence	915	915	97% 0.0	91% GU289654.1	858,40	71,9%
Select seq gb|KM051454.1|	Coriandrum sativum isolate TKM201428 18S ribosomal RNA gene, partial sequence; internal transcribed spacer 1, 5.8S ribosomal RNA gene, and internal transcribed spacer 2, complete sequence; and 26S ribosomal RNA gene, partial sequence	935	935	100% 0.0	91% KM051454.1	850,85	71,2%
Select seq gb|JN853781.1|	Ligusticum tenuissimum 18S ribosomal RNA gene, partial sequence; internal transcribed spacer 1, 5.8S ribosomal RNA gene, and internal transcribed spacer 2, complete sequence; and 26S ribosomal RNA gene, partial sequence	885	885	95% 0.0	90% JN853781.1	838,42	70,2%
Select seq gb|KJ025063.1|	Cnidium officinale 18S ribosomal RNA gene, partial sequence; internal transcribed spacer 1, 5.8S ribosomal RNA gene, and internal transcribed spacer 2, complete sequence; and 26S ribosomal RNA gene, partial sequence	931	931	100% 0.0	90% KJ025063.1	837,90	70,1%
Select seq gb|FJ481927.1|	Ligusticum jeholense isolate DCY-03 18S ribosomal RNA gene, partial sequence; internal transcribed spacer 1, 5.8S ribosomal RNA gene, and internal transcribed spacer 2, complete sequence; and 28S ribosomal RNA gene, partial sequence	931	931	100% 0.0	90% FJ481927.1	837,90	70,1%
Select seq gb|KP159316.1|	Glehnia littoralis isolate TKM201455 18S ribosomal RNA gene, partial sequence; internal transcribed spacer 1, 5.8S ribosomal RNA gene, and internal transcribed spacer 2, complete sequence; and 26S ribosomal RNA gene, partial sequence	911	911	98% 0.0	90% KP159316.1	836,63	70,0%
Select seq dbj|AB569093.1|	Angelica acutiloba genes for 18S rRNA, ITS1, 5.8S rRNA, ITS2, 26S rRNA, partial and complete sequence	883	883	95% 0.0	90% AB569093.1	836,53	70,0%
Select seq dbj|AB697608.1|	Angelica stenoloba genes for 18S rRNA, ITS1, 5.8S rRNA, ITS2, 26S rRNA, partial and complete sequence, strain: LH	878	878	95% 0.0	90% AB697608.1	831,79	69,6%
Select seq dbj|AB697606.1|	Angelica acutiloba var. iwatensis genes for 18S rRNA, ITS1, 5.8S rRNA, ITS2, 26S rRNA, partial and complete sequence, strain: MM	878	878	95% 0.0	90% AB697606.1	831,79	69,6%
Select seq dbj|AB697596.1|	Angelica acutiloba genes for 18S rRNA, ITS1, 5.8S rRNA, ITS2, 26S rRNA, partial and complete sequence, strain: TC	878	878	95% 0.0	90% AB697596.1	831,79	69,6%
Select seq dbj|AB697594.1|	Angelica acutiloba genes for 18S rRNA, ITS1, 5.8S rRNA, ITS2, 26S rRNA, partial and complete sequence, strain: TY	878	878	95% 0.0	90% AB697594.1	831,79	69,6%
Select seq gb|KF160674.1|	Peucedanum ostruthium voucher C:Simonsen 2013-1 internal transcribed spacer 1, partial sequence; 5.8S ribosomal RNA gene, complete sequence; and internal transcribed spacer 2, partial sequence	883	883	96% 0.0	90% KF160674.1	827,81	69,3%
Select seq gb|FJ593179.1|	Glehnia littoralis 18S ribosomal RNA gene, partial sequence; internal transcribed spacer 1, 5.8S ribosomal RNA gene, and internal transcribed spacer 2, complete sequence; and 26S ribosomal RNA gene, partial sequence	872	872	95% 0.0	90% FJ593179.1	826,11	69,1%
Select seq gb|KM051435.1|	Angelica gigas isolate TKM201410 18S ribosomal RNA gene, partial sequence; internal transcribed spacer 1, 5.8S ribosomal RNA gene, and internal transcribed spacer 2, complete sequence; and 26S ribosomal RNA gene, partial sequence	915	915	100% 0.0	90% KM051435.1	823,50	68,9%

Select for downloading or viewing reports	Kh048_trnL Description	Max score	Total score	Query cover	E value	Ident	Accession	(Ident/Cover)* Max score	Deviation from top hit
Select seq gb|EU001357.1|	Heracleum candicans clone trn-yy06 tRNA-Leu (trnL) gene, partial sequence; and trnL-trnF intergenic spacer, complete sequence; chloroplast	1314	1314	98%	0.0	99%	EU001357.1	1327,41	100,0%
Select seq gb|FJ475216.1|	Pterocyclus rivulorum tRNA-Leu (trnL) gene and trnL-trnF intergenic spacer, partial sequence; chloroplast	1293	1293	98%	0.0	99%	FJ475216.1	1306,19	98,4%
Select seq gb|KT963036.1|	Angelica acutiloba voucher 13F-28 chloroplast, complete genome	1319	1319	100%	0.0	99%	KT963036.1	1305,81	98,4%
Select seq gb|AF444030.1|	Shoshonea pulvinata tRNA-Phe gene, partial sequence; tRNA-Leu gene, complete sequence; and tRNA-Thr gene, partial sequence; chloroplast genes for chloroplast products	1319	1319	100%	0.0	99%	AF444030.1	1305,81	98,4%
Select seq gb|AF444029.1|	Pteryxia terebinthina tRNA-Phe gene, partial sequence; and tRNA-Leu gene, complete sequence; chloroplast genes for chloroplast products	1288	1288	98%	0.0	99%	AF444029.1	1301,14	98,0%
Select seq gb|AF444020.1|	Lomatium junceum tRNA-Phe gene, partial sequence; tRNA-Leu gene, complete sequence; and tRNA-Thr gene, partial sequence; chloroplast genes for chloroplast products	1288	1288	98%	0.0	99%	AF444020.1	1301,14	98,0%
Select seq gb|AF444026.1|	Neoparrya lithophila tRNA-Phe gene, partial sequence; tRNA-Leu gene, complete sequence; and tRNA-Thr gene, partial sequence; chloroplast genes for chloroplast products	1314	1314	100%	0.0	99%	AF444026.1	1300,86	98,0%
Select seq gb|AF444025.1|	Musineon tenuifolium tRNA-Phe gene, partial sequence; tRNA-Leu gene, complete sequence; and tRNA-Thr gene, partial sequence; chloroplast genes for chloroplast products	1314	1314	100%	0.0	99%	AF444025.1	1300,86	98,0%
Select seq gb|AF444008.1|	Aletes anisatus tRNA-Phe gene, partial sequence; tRNA-Leu gene, complete sequence; and tRNA-Thr gene, partial sequence; chloroplast genes for chloroplast products	1314	1314	100%	0.0	99%	AF444008.1	1300,86	98,0%
Select seq emb|AM109908.1|	Cnidium officinale plastid partial tRNA-Leu gene and partial IGS	1314	1314	100%	0.0	99%	AM109908.1	1300,86	98,0%
Select seq dbj|LC072690.1|	Libanotis buchtormensis chloroplast DNA, trnL-trnF intergenic spacer, partial sequence, isolate: LJJ11	1271	1271	97%	0.0	99%	LC072690.1	1297,21	97,7%
Select seq gb|AF444028.1|	Pteryxia hendersonii tRNA-Phe gene, partial sequence; tRNA-Leu gene, complete sequence; and tRNA-Thr gene, partial sequence; chloroplast genes for chloroplast products	1310	1310	100%	0.0	99%	AF444028.1	1296,90	97,7%
Select seq gb|AF444013.1|	Cymopterus purpureus tRNA-Phe gene, partial sequence; tRNA-Leu gene, complete sequence; and tRNA-Thr gene, partial sequence; chloroplast genes for chloroplast products	1279	1279	98%	0.0	99%	AF444013.1	1292,05	97,3%
Select seq gb|AF444012.1|	Cymopterus nivalis tRNA-Phe gene, partial sequence; tRNA-Leu gene, complete sequence; and tRNA-Thr gene, partial sequence; chloroplast genes for chloroplast products	1279	1279	98%	0.0	99%	AF444012.1	1292,05	97,3%
Select seq gb|KP665033.1|	Angelica sinensis isolate 13R17 tRNA-Leu (trnL) gene and trnL-trnF intergenic spacer, partial sequence; chloroplast	1264	1264	97%	0.0	99%	KP665033.1	1290,06	97,2%
Select seq gb|KT963038.1|	Angelica gigas voucher 13E-39-3 chloroplast, complete genome	1303	1303	100%	0.0	99%	KT963038.1	1289,97	97,2%
Select seq gb|KT852844.1|	Ostericum grosseserratum voucher 13A-14-2 chloroplast, complete genome	1303	1303	100%	0.0	99%	KT852844.1	1289,97	97,2%
Select seq gb|FJ475186.1|	Hymenidium apiolens tRNA-Leu (trnL) gene and trnL-trnF intergenic spacer, partial sequence; chloroplast	1303	1303	100%	0.0	99%	FJ475186.1	1289,97	97,2%
Select seq gb|AF444024.1|	Musineon divaricatum tRNA-Phe gene, partial sequence; tRNA-Leu gene, complete sequence; and tRNA-Thr gene, partial sequence; chloroplast genes for chloroplast products	1303	1303	100%	0.0	99%	AF444024.1	1289,97	97,2%
Select seq gb|KT963037.1|	Angelica dahurica voucher 13T-05 chloroplast, complete genome	1299	1299	100%	0.0	99%	KT963037.1	1286,01	96,9%
Select seq gb|AF444033.1|	Zizia aurea tRNA-Phe gene, partial sequence; tRNA-Leu gene, complete sequence; and tRNA-Thr gene, partial sequence; chloroplast genes for chloroplast products	1299	1299	100%	0.0	99%	AF444033.1	1286,01	96,9%
Select seq gb|AF444017.1|	Lomatium californicum tRNA-Phe gene, partial sequence; tRNA-Leu gene, complete sequence; and tRNA-Thr gene, partial sequence; chloroplast genes for chloroplast products	1299	1299	100%	0.0	99%	AF444017.1	1286,01	96,9%
Select seq gb|KT781591.1|	Angelica decursiva voucher 13Q-02-1 chloroplast, complete genome	1297	1297	100%	0.0	99%	KT781591.1	1284,03	96,7%
Select seq gb|AF444014.1|	Cymopterus williamsii tRNA-Phe gene, partial sequence; tRNA-Leu gene, complete sequence; and tRNA-Thr gene, partial sequence; chloroplast genes for chloroplast products	1297	1297	100%	0.0	99%	AF444014.1	1284,03	96,7%
Select seq gb|AF444007.1|	Angelica archangelica tRNA-Phe gene, partial sequence; tRNA-Leu gene, complete sequence; and tRNA-Thr gene, partial sequence; chloroplast genes for chloroplast products	1297	1297	100%	0.0	99%	AF444007.1	1284,03	96,7%
Select seq dbj|LC072693.1|	Libanotis spodotrichoma chloroplast DNA, trnL-trnF intergenic spacer, partial sequence, isolate: LJJ14	1266	1266	97%	0.0	98%	LC072693.1	1279,05	96,4%
Select seq dbj|LC072692.1|	Libanotis buchtormensis chloroplast DNA, trnL-trnF intergenic spacer, partial sequence, isolate: LJJ13	1266	1266	97%	0.0	98%	LC072692.1	1279,05	96,4%

Select seq gb|KJ157767.1|	Pastinaca sativa voucher J. Wen 9783 tRNA-Leu (trnL) gene and trnL-trnF intergenic spacer, partial sequence; chloroplast	1264	1264	97%	0.0	98%	KJ157767.1	1277,03	96,2%
Select seq gb|EU076599.1|	Seseli mairei tRNA-Leu (trnL) gene and trnF-trnL intergenic spacer, partial sequence; chromoplast	1277	1277	98%	0.0	98%	EU076599.1	1277,00	96,2%
Select seq gb|GQ351360.1|	Peucedanum japonicum tRNA-Leu (trnL) gene, partial sequence; trnL-trnF intergenic spacer, complete sequence; and tRNA-Phe (trnF) gene, partial sequence; chloroplast	1262	1262	97%	0.0	98%	GQ351360.1	1275,01	96,1%
Select seq gb|KP665009.1|	Peucedanum praeruptorum isolate 13R37 tRNA-Leu (trnL) gene and trnL-trnF intergenic spacer, partial sequence; chloroplast	1256	1256	97%	0.0	98%	KP665009.1	1268,95	95,6%
Select seq gb|KP665008.1|	Peucedanum praeruptorum isolate 13R36 tRNA-Leu (trnL) gene and trnL-trnF intergenic spacer, partial sequence; chloroplast	1256	1256	97%	0.0	98%	KP665008.1	1268,95	95,6%
Select seq gb|KP665007.1|	Peucedanum praeruptorum isolate 13R35 tRNA-Leu (trnL) gene and trnL-trnF intergenic spacer, partial sequence; chloroplast	1256	1256	97%	0.0	98%	KP665007.1	1268,95	95,6%
Select seq gb|KP665006.1|	Peucedanum praeruptorum isolate 13R34 tRNA-Leu (trnL) gene and trnL-trnF intergenic spacer, partial sequence; chloroplast	1256	1256	97%	0.0	98%	KP665006.1	1268,95	95,6%
Select seq gb|KP665005.1|	Peucedanum praeruptorum isolate 13R31re2 tRNA-Leu (trnL) gene and trnL-trnF intergenic spacer, partial sequence; chloroplast	1256	1256	97%	0.0	98%	KP665005.1	1268,95	95,6%
Select seq gb|KP665003.1|	Peucedanum praeruptorum isolate 13R28 tRNA-Leu (trnL) gene and trnL-trnF intergenic spacer, partial sequence; chloroplast	1256	1256	97%	0.0	98%	KP665003.1	1268,95	95,6%
Select seq gb|KP665002.1|	Peucedanum praeruptorum isolate 13R25 tRNA-Leu (trnL) gene and trnL-trnF intergenic spacer, partial sequence; chloroplast	1256	1256	97%	0.0	98%	KP665002.1	1268,95	95,6%
Select seq gb|KP665000.1|	Peucedanum praeruptorum isolate 13R23 tRNA-Leu (trnL) gene and trnL-trnF intergenic spacer, partial sequence; chloroplast	1256	1256	97%	0.0	98%	KP665000.1	1268,95	95,6%
Select seq gb|KP664999.1|	Peucedanum praeruptorum isolate 13R22 tRNA-Leu (trnL) gene and trnL-trnF intergenic spacer, partial sequence; chloroplast	1256	1256	97%	0.0	98%	KP664999.1	1268,95	95,6%
Select seq gb|KP664998.1|	Peucedanum praeruptorum isolate 13R21 tRNA-Leu (trnL) gene and trnL-trnF intergenic spacer, partial sequence; chloroplast	1256	1256	97%	0.0	98%	KP664998.1	1268,95	95,6%
Select seq gb|KP664997.1|	Peucedanum praeruptorum isolate 13R20 tRNA-Leu (trnL) gene and trnL-trnF intergenic spacer, partial sequence; chloroplast	1256	1256	97%	0.0	98%	KP664997.1	1268,95	95,6%
Select seq gb|KP664996.1|	Peucedanum praeruptorum isolate 13R19 tRNA-Leu (trnL) gene and trnL-trnF intergenic spacer, partial sequence; chloroplast	1256	1256	97%	0.0	98%	KP664996.1	1268,95	95,6%
Select seq gb|KP664995.1|	Peucedanum praeruptorum isolate 13R18 tRNA-Leu (trnL) gene and trnL-trnF intergenic spacer, partial sequence; chloroplast	1256	1256	97%	0.0	98%	KP664995.1	1268,95	95,6%
Select seq gb|KP664994.1|	Peucedanum praeruptorum isolate 13R15 tRNA-Leu (trnL) gene and trnL-trnF intergenic spacer, partial sequence; chloroplast	1256	1256	97%	0.0	98%	KP664994.1	1268,95	95,6%
Select seq gb|KP664993.1|	Peucedanum praeruptorum isolate 13R14 tRNA-Leu (trnL) gene and trnL-trnF intergenic spacer, partial sequence; chloroplast	1256	1256	97%	0.0	98%	KP664993.1	1268,95	95,6%
Select seq gb|KP664992.1|	Peucedanum praeruptorum isolate 13R13 tRNA-Leu (trnL) gene and trnL-trnF intergenic spacer, partial sequence; chloroplast	1256	1256	97%	0.0	98%	KP664992.1	1268,95	95,6%
Select seq gb|KP664991.1|	Peucedanum praeruptorum isolate 13R12 tRNA-Leu (trnL) gene and trnL-trnF intergenic spacer, partial sequence; chloroplast	1256	1256	97%	0.0	98%	KP664991.1	1268,95	95,6%
Select seq gb|KP664990.1|	Peucedanum praeruptorum isolate 13R11 tRNA-Leu (trnL) gene and trnL-trnF intergenic spacer, partial sequence; chloroplast	1256	1256	97%	0.0	98%	KP664990.1	1268,95	95,6%
Select seq gb|KP664989.1|	Peucedanum praeruptorum isolate 13R10 tRNA-Leu (trnL) gene and trnL-trnF intergenic spacer, partial sequence; chloroplast	1256	1256	97%	0.0	98%	KP664989.1	1268,95	95,6%
Select seq gb|KP664988.1|	Peucedanum praeruptorum isolate 13R09 tRNA-Leu (trnL) gene and trnL-trnF intergenic spacer, partial sequence; chloroplast	1256	1256	97%	0.0	98%	KP664988.1	1268,95	95,6%
Select seq gb|KP664987.1|	Peucedanum praeruptorum isolate 13R08 tRNA-Leu (trnL) gene and trnL-trnF intergenic spacer, partial sequence; chloroplast	1256	1256	97%	0.0	98%	KP664987.1	1268,95	95,6%
Select seq gb|KP664986.1|	Peucedanum praeruptorum isolate 13R07 tRNA-Leu (trnL) gene and trnL-trnF intergenic spacer, partial sequence; chloroplast	1256	1256	97%	0.0	98%	KP664986.1	1268,95	95,6%
Select seq gb|KP664985.1|	Peucedanum praeruptorum isolate 13R06 tRNA-Leu (trnL) gene and trnL-trnF intergenic spacer, partial sequence; chloroplast	1256	1256	97%	0.0	98%	KP664985.1	1268,95	95,6%
Select seq gb|KP664983.1|	Peucedanum praeruptorum isolate 13R04 tRNA-Leu (trnL) gene and trnL-trnF intergenic spacer, partial sequence; chloroplast	1256	1256	97%	0.0	98%	KP664983.1	1268,95	95,6%
Select seq gb|KP664982.1|	Peucedanum praeruptorum isolate 13R03 tRNA-Leu (trnL) gene and trnL-trnF intergenic spacer, partial sequence; chloroplast	1256	1256	97%	0.0	98%	KP664982.1	1268,95	95,6%

Select seq gb|KP664981.1|	Peucedanum praeruptorum isolate 13R02 tRNA-Leu (trnL) gene and trnL-trnF intergenic spacer, partial sequence; chloroplast	1256	1256	97%	0.0	98%	KP664981.1	1268,95	95,6%
Select seq gb|KP664980.1|	Peucedanum praeruptorum isolate 13R01 tRNA-Leu (trnL) gene and trnL-trnF intergenic spacer, partial sequence; chloroplast	1256	1256	97%	0.0	98%	KP664980.1	1268,95	95,6%
Select seq gb|KP664978.1|	Angelica decursiva isolate 13Q18 tRNA-Leu (trnL) gene and trnL-trnF intergenic spacer, partial sequence; chloroplast	1256	1256	97%	0.0	98%	KP664978.1	1268,95	95,6%
Select seq gb|KP664977.1|	Angelica decursiva isolate 13Q16 tRNA-Leu (trnL) gene and trnL-trnF intergenic spacer, partial sequence; chloroplast	1256	1256	97%	0.0	98%	KP664977.1	1268,95	95,6%
Select seq gb|KP664976.1|	Angelica decursiva isolate 13Q15 tRNA-Leu (trnL) gene and trnL-trnF intergenic spacer, partial sequence; chloroplast	1256	1256	97%	0.0	98%	KP664976.1	1268,95	95,6%
Select seq gb|KP664975.1|	Angelica decursiva isolate 13Q13 tRNA-Leu (trnL) gene and trnL-trnF intergenic spacer, partial sequence; chloroplast	1256	1256	97%	0.0	98%	KP664975.1	1268,95	95,6%
Select seq gb|KP664974.1|	Angelica decursiva isolate 13Q12 tRNA-Leu (trnL) gene and trnL-trnF intergenic spacer, partial sequence; chloroplast	1256	1256	97%	0.0	98%	KP664974.1	1268,95	95,6%
Select seq gb|KP664973.1|	Angelica decursiva isolate 13Q11 tRNA-Leu (trnL) gene and trnL-trnF intergenic spacer, partial sequence; chloroplast	1256	1256	97%	0.0	98%	KP664973.1	1268,95	95,6%
Select seq gb|KP664972.1|	Angelica decursiva isolate 13Q10 tRNA-Leu (trnL) gene and trnL-trnF intergenic spacer, partial sequence; chloroplast	1256	1256	97%	0.0	98%	KP664972.1	1268,95	95,6%
Select seq gb|KP664971.1|	Angelica decursiva isolate 13Q09 tRNA-Leu (trnL) gene and trnL-trnF intergenic spacer, partial sequence; chloroplast	1256	1256	97%	0.0	98%	KP664971.1	1268,95	95,6%
Select seq gb|KP664970.1|	Angelica decursiva isolate 13Q08 tRNA-Leu (trnL) gene and trnL-trnF intergenic spacer, partial sequence; chloroplast	1256	1256	97%	0.0	98%	KP664970.1	1268,95	95,6%
Select seq gb|KP664969.1|	Angelica decursiva isolate 13Q06 tRNA-Leu (trnL) gene and trnL-trnF intergenic spacer, partial sequence; chloroplast	1256	1256	97%	0.0	98%	KP664969.1	1268,95	95,6%
Select seq gb|KP664968.1|	Angelica decursiva isolate 13Q05 tRNA-Leu (trnL) gene and trnL-trnF intergenic spacer, partial sequence; chloroplast	1256	1256	97%	0.0	98%	KP664968.1	1268,95	95,6%
Select seq gb|KP664967.1|	Angelica decursiva isolate 13Q04 tRNA-Leu (trnL) gene and trnL-trnF intergenic spacer, partial sequence; chloroplast	1256	1256	97%	0.0	98%	KP664967.1	1268,95	95,6%
Select seq gb|KP664966.1|	Angelica decursiva isolate 13Q03 tRNA-Leu (trnL) gene and trnL-trnF intergenic spacer, partial sequence; chloroplast	1256	1256	97%	0.0	98%	KP664966.1	1268,95	95,6%
Select seq gb|KP664965.1|	Angelica decursiva isolate 13Q01 tRNA-Leu (trnL) gene and trnL-trnF intergenic spacer, partial sequence; chloroplast	1256	1256	97%	0.0	98%	KP664965.1	1268,95	95,6%
Select seq gb|KM035851.1|	Seseli montanum plastid, complete genome	1293	1293	100%	0.0	98%	KM035851.1	1267,14	95,5%
Select seq gb|AF444032.1|	Zizia aptera tRNA-Phe gene, partial sequence; tRNA-Leu gene, complete sequence; and tRNA-Thr gene, partial sequence; chloroplast genes for chloroplast products	1293	1293	100%	0.0	98%	AF444032.1	1267,14	95,5%
Select seq gb|AF444031.1|	Thaspium trifoliatum tRNA-Phe gene, partial sequence; and tRNA-Leu gene, complete sequence; chloroplast genes for chloroplast products	1293	1293	100%	0.0	98%	AF444031.1	1267,14	95,5%
Select seq gb|AF444023.1|	Lomatium nudicaule tRNA-Phe gene, partial sequence; tRNA-Leu gene, complete sequence; and tRNA-Thr gene, partial sequence; chloroplast genes for chloroplast products	1293	1293	100%	0.0	98%	AF444023.1	1267,14	95,5%
Select seq gb|EU076598.1|	Seseli squarrulosum tRNA-Leu (trnL) gene and trnF-trnL intergenic spacer, partial sequence; chromoplast	1267	1267	98%	0.0	98%	EU076598.1	1267,00	95,4%
Select seq gb|EU001356.1|	Heracleum oreocharis clone trn-yy08 tRNA-Leu (trnL) gene, partial sequence; and trnL-trnF intergenic spacer, complete sequence; chloroplast	1266	1266	98%	0.0	98%	EU001356.1	1266,00	95,4%
Select seq emb|AM109910.1|	Dystaenia ibukiensis plastid partial tRNA-Leu gene and partial IGS, isolate 7	1291	1291	100%	0.0	98%	AM109910.1	1265,18	95,3%
Select seq gb|JQ041843.1|	Heracleum sphondylium voucher HSP1 tRNA-Leu (trnL) gene, complete sequence; and trnL-trnF intergenic spacer, partial sequence; plastid	1264	1264	98%	0.0	98%	JQ041843.1	1264,00	95,2%
Select seq gb|KP665004.1|	Peucedanum praeruptorum isolate 13R29re2 tRNA-Leu (trnL) gene and trnL-trnF intergenic spacer, partial sequence; chloroplast	1251	1251	97%	0.0	98%	KP665004.1	1263,90	95,2%
Select seq gb|JQ041844.1|	Heracleum sphondylium voucher HSP3 tRNA-Leu (trnL) gene, complete sequence; and trnL-trnF intergenic spacer, partial sequence; plastid	1262	1262	98%	0.0	98%	JQ041844.1	1262,00	95,1%
Select seq gb|KT963039.1|	Ligusticum tenuissimum voucher 13I-08 chloroplast, complete genome	1286	1286	100%	0.0	98%	KT963039.1	1260,28	94,9%
Select seq gb|KM035850.1|	Pastinaca pimpinellifolia voucher M.G. Pimenov et al., 117 (MW) plastid, complete genome	1286	1286	100%	0.0	98%	KM035850.1	1260,28	94,9%

Select seq gb|HQ246204.1|	Trachydium subnudum tRNA-Leu (trnL) gene, partial sequence; trnL-trnF intergenic spacer, complete sequence; and tRNA-Phe (trnF) gene, partial sequence; chloroplast	1286	1286	100% 0.0	98% HQ246204.1	1260,28	94,9%
Select seq gb|AF444010.1|	Cymopterus montanus tRNA-Phe gene, partial sequence; tRNA-Leu gene, complete sequence; and tRNA-Thr gene, partial sequence; chloroplast genes for chloroplast products	1286	1286	100% 0.0	98% AF444010.1	1260,28	94,9%
Select seq gb|AF444019.1|	Lomatium graveolens tRNA-Phe gene, partial sequence; tRNA-Leu gene, complete sequence; and tRNA-Thr gene, partial sequence; chloroplast genes for chloroplast products	1258	1258	98% 0.0	98% AF444019.1	1258,00	94,8%
Select seq gb|EU001352.1|	Heracleum moellendorffii clone trn-yy15 tRNA-Leu (trnL) gene, partial sequence; and trnL-trnF intergenic spacer, complete sequence; chloroplast	1256	1256	98% 0.0	98% EU001352.1	1256,00	94,6%
Select seq gb|KJ157726.1|	Heracleum maximum voucher J. Wen 10391 tRNA-Leu (trnL) gene and trnL-trnF intergenic spacer, partial sequence; chloroplast	1242	1242	97% 0.0	98% KJ157726.1	1254,80	94,5%
Select seq gb|AF444018.1|	Lomatium dasycarpum tRNA-Phe gene, partial sequence; tRNA-Leu gene, complete sequence; and tRNA-Thr gene, partial sequence; chloroplast genes for chloroplast products	1253	1253	98% 0.0	98% AF444018.1	1253,00	94,4%
Select seq gb|HQ246201.1|	Sinodielsia thibetica tRNA-Leu (trnL) gene and trnL-trnF intergenic spacer, complete sequence; and tRNA-Phe (trnF) gene, partial sequence; chloroplast	1275	1275	100% 0.0	98% HQ246201.1	1249,50	94,1%
Select seq gb|EU001353.1|	Angelica apaensis clone trn-yy11 tRNA-Leu (trnL) gene, partial sequence; and trnL-trnF intergenic spacer, complete sequence; chloroplast	1249	1249	98% 0.0	98% EU001353.1	1249,00	94,1%
Select seq gb|AF444021.1|	Lomatium juniperinum tRNA-Phe gene, partial sequence; and tRNA-Leu gene, complete sequence; chloroplast genes for chloroplast products	1271	1271	100% 0.0	98% AF444021.1	1245,58	93,8%
Select seq gb|EU001361.1|	Heracleum forrestii clone trn-yy01 tRNA-Leu (trnL) gene, partial sequence; and trnL-trnF intergenic spacer, complete sequence; chloroplast	1245	1245	98% 0.0	98% EU001361.1	1245,00	93,8%
Select seq gb|EU001358.1|	Heracleum stenopterum clone trn-yy05 tRNA-Leu (trnL) gene, partial sequence; and trnL-trnF intergenic spacer, complete sequence; chloroplast	1245	1245	98% 0.0	98% EU001358.1	1245,00	93,8%
Select seq gb|FJ475189.1|	Hymenidium brunonis tRNA-Leu (trnL) gene and trnL-trnF intergenic spacer, partial sequence; chloroplast	1262	1262	100% 0.0	98% FJ475189.1	1236,76	93,2%
Select seq gb|AF444011.1|	Cymopterus multinervatus tRNA-Phe gene, partial sequence; tRNA-Leu gene, complete sequence; and tRNA-Thr gene, partial sequence; chloroplast genes for chloroplast products	1262	1262	100% 0.0	98% AF444011.1	1236,76	93,2%
Select seq gb|AF444027.1|	Podistera eastwoodiae tRNA-Phe gene, partial sequence; tRNA-Leu gene, complete sequence; and tRNA-Thr gene, partial sequence; chloroplast genes for chloroplast products	1258	1258	100% 0.0	98% AF444027.1	1232,84	92,9%
Select seq gb|AF444015.1|	Harbouria trachypleura tRNA-Phe gene, partial sequence; tRNA-Leu gene, complete sequence; and tRNA-Thr gene, partial sequence; chloroplast genes for chloroplast products	1247	1247	100% 0.0	97% AF444015.1	1209,59	91,1%
Select seq gb|KR011055.1|	Anethum graveolens chloroplast, complete genome	1243	1243	100% 0.0	97% KR011055.1	1205,71	90,8%

Select for downloading or viewing reports	Kh051_trnL Description	Max score	Total score	Query cover	E value	Ident	Accession	(Ident/Cover)* Max score	Deviation from top hit
Select seq gb|JQ669045.1|	Hymenocrater bituminosus voucher K. Tamanyan & George Fayvush tRNA-Leu (trnL) gene and trnL-trnF intergenic spacer, partial sequence; plastid	994	994	93%	0.0	98%	JQ669045.1	1047,44	100,0%
Select seq gb|JQ669049.1|	Marmoritis complanatum voucher D.E. Boufford et al., 32012 tRNA-Leu (trnL) gene and trnL-trnF intergenic spacer, partial sequence; plastid	976	976	93%	0.0	98%	JQ669049.1	1028,47	98,2%
Select seq gb|KM886627.1|	Marmoritis complanata voucher T. Deng 2359 (KUN) tRNA-Leu (trnL) gene, partial sequence; trnL-trnF intergenic spacer, complete sequence; and tRNA-Phe (trnF) gene, partial sequence; plastid	968	968	93%	0.0	98%	KM886627.1	1020,04	97,4%
Select seq gb|KR150216.1|	Nepeta sp. 1 Kh03 trnL-trnF intergenic spacer region, partial sequence; chloroplast	857	857	89%	0.0	95%	KR150216.1	914,78	87,3%
Select seq emb|AJ505431.1|	Nepeta menthoides plastid trnL-trnF intergenic spacer, specimen voucher Jamzad s.n. (K)	893	893	93%	0.0	95%	AJ505431.1	912,20	87,1%
Select seq gb|KF307433.1|	Lepechinia urbanii voucher B. Drew 135 tRNA-Leu (trnL) gene and trnL-trnF intergenic spacer, partial sequence; chloroplast	889	889	93%	0.0	95%	KF307433.1	908,12	86,7%
Select seq gb|JF301391.1|	Nepeta cataria voucher B. Drew 72 tRNA-Leu (trnL) gene and trnL-trnF intergenic spacer, partial sequence; chloroplast	948	948	100%	0.0	95%	JF301391.1	900,60	86,0%
Select seq gb|DQ667487.1|	Nepeta cataria isolate x220 tRNA-Leu (trnL) gene and trnL-trnF intergenic spacer, partial sequence; chloroplast	948	948	100%	0.0	95%	DQ667487.1	900,60	86,0%
Select seq emb|AJ505432.1|	Nepeta racemosa plastid trnL-trnF intergenic spacer, specimen voucher Jamzad s.n. (TARI)	948	948	100%	0.0	95%	AJ505432.1	900,60	86,0%
Select seq gb|JF301383.1|	Lepechinia salviifolia voucher R. Jabaily s.n. tRNA-Leu (trnL) gene and trnL-trnF intergenic spacer, partial sequence; chloroplast	880	880	93%	0.0	95%	JF301383.1	898,92	85,8%
Select seq gb|JF301382.1|	Lepechinia radula voucher B. Drew 185 tRNA-Leu (trnL) gene and trnL-trnF intergenic spacer, partial sequence; chloroplast	880	880	93%	0.0	95%	JF301382.1	898,92	85,8%
Select seq gb|JF301377.1|	Lepechinia flammea tRNA-Leu (trnL) gene and trnL-trnF intergenic spacer, partial sequence; chloroplast	880	880	93%	0.0	95%	JF301377.1	898,92	85,8%
Select seq gb|DQ667492.1|	Lepechinia lancifolia isolate x232 tRNA-Leu (trnL) gene and trnL-trnF intergenic spacer, partial sequence; chloroplast	880	880	93%	0.0	95%	DQ667492.1	898,92	85,8%
Select seq gb|KF307426.1|	Lepechinia paniculata voucher B. Drew 241 tRNA-Leu (trnL) gene and trnL-trnF intergenic spacer, partial sequence; chloroplast	870	870	92%	0.0	95%	KF307426.1	898,37	85,8%
Select seq gb|KF307430.1|	Lepechinia schiedeana voucher B. Drew 157 tRNA-Leu (trnL) gene and trnL-trnF intergenic spacer, partial sequence; chloroplast	878	878	93%	0.0	95%	KF307430.1	896,88	85,6%
Select seq gb|KF307411.1|	Lepechinia bella voucher Rachel Jabaily s.n. tRNA-Leu (trnL) gene and trnL-trnF intergenic spacer, partial sequence; chloroplast	878	878	93%	0.0	95%	KF307411.1	896,88	85,6%
Select seq gb|JF301361.1|	Chaunostoma mecistandrum voucher J.A. Monterrosa & R.A. Carballo 213 tRNA-Leu (trnL) gene and trnL-trnF intergenic spacer, partial sequence; chloroplast	874	874	93%	0.0	95%	JF301361.1	892,80	85,2%
Select seq gb|KF307435.1|	Lepechinia yecorana voucher Henrickson 24691 tRNA-Leu (trnL) gene and trnL-trnF intergenic spacer, partial sequence; chloroplast	872	872	93%	0.0	95%	KF307435.1	890,75	85,0%
Select seq gb|KF307432.1|	Lepechinia speciosa voucher Cordeno 3060 tRNA-Leu (trnL) gene and trnL-trnF intergenic spacer, partial sequence; chloroplast	872	872	93%	0.0	95%	KF307432.1	890,75	85,0%
Select seq gb|KF307421.1|	Lepechinia heteromorpha voucher B. Drew 192 tRNA-Leu (trnL) gene and trnL-trnF intergenic spacer, partial sequence; chloroplast	872	872	93%	0.0	95%	KF307421.1	890,75	85,0%
Select seq gb|KF307420.1|	Lepechinia graveolens voucher Fuentes 10351 tRNA-Leu (trnL) gene and trnL-trnF intergenic spacer, partial sequence; chloroplast	872	872	93%	0.0	95%	KF307420.1	890,75	85,0%
Select seq gb|KF307418.1|	Lepechinia floribunda voucher B. Drew 172 tRNA-Leu (trnL) gene and trnL-trnF intergenic spacer, partial sequence; chloroplast	872	872	93%	0.0	95%	KF307418.1	890,75	85,0%
Select seq gb|GU381494.1|	Killickia pilosa voucher NU<ZAF>:Potgieter & Thompson 739 tRNA-Leu (trnL) gene and trnL-trnF intergenic spacer, partial sequence; chloroplast	854	854	91%	0.0	94%	GU381494.1	882,15	84,2%
Select seq gb|GU381492.1|	Killickia grandiflora voucher NU<ZAF>:Hilliard & Burtt 18579 tRNA-Leu (trnL) gene and trnL-trnF intergenic spacer, partial sequence; chloroplast	854	854	91%	0.0	94%	GU381492.1	882,15	84,2%
Select seq gb|GU381496.1|	Killickia pilosa voucher M:Braeuchler 3832 tRNA-Leu (trnL) gene and trnL-trnF intergenic spacer, partial sequence; chloroplast	870	870	93%	0.0	94%	GU381496.1	879,35	84,0%
Select seq gb|GU381495.1|	Killickia pilosa voucher M:Braeuchler 3810 tRNA-Leu (trnL) gene and trnL-trnF intergenic spacer, partial sequence; chloroplast	870	870	93%	0.0	94%	GU381495.1	879,35	84,0%
Select seq gb|GU381493.1|	Killickia grandiflora voucher M:Braeuchler 3811 tRNA-Leu (trnL) gene and trnL-trnF intergenic spacer, partial sequence; chloroplast	870	870	93%	0.0	94%	GU381493.1	879,35	84,0%

Select seq gb|JF301381.1|	Lepechinia mexicana voucher B. Drew 164 tRNA-Leu (trnL) gene and trnL-trnF intergenic spacer, partial sequence; chloroplast	869	869	93% 0.0	94% JF301381.1	878,34	83,9%
Select seq gb|JF301380.1|	Lepechinia mexicana voucher B. Drew 127 tRNA-Leu (trnL) gene and trnL-trnF intergenic spacer, partial sequence; chloroplast	869	869	93% 0.0	94% JF301380.1	878,34	83,9%
Select seq gb|KF307422.1|	Lepechinia mexicana voucher B. Drew 130 tRNA-Leu (trnL) gene and trnL-trnF intergenic spacer, partial sequence; chloroplast	867	867	93% 0.0	94% KF307422.1	876,32	83,7%
Select seq gb|GU381479.1|	Nepeta supina voucher M:Groeger & Schewardnadse 1466 tRNA-Leu (trnL) gene and trnL-trnF intergenic spacer, partial sequence; chloroplast	931	931	100% 0.0	94% GU381479.1	875,14	83,6%
Select seq gb|KF307416.1|	Lepechinia codon voucher B. Drew 177 tRNA-Leu (trnL) gene and trnL-trnF intergenic spacer, partial sequence; chloroplast	865	865	93% 0.0	94% KF307416.1	874,30	83,5%
Select seq emb|AJ505433.1|	Nepeta straussii plastid trnL-trnF intergenic spacer, specimen voucher Jamzad etal 76846 (TARI)	926	926	100% 0.0	94% AJ505433.1	870,44	83,1%
Select seq gb|AY840207.1|	Thymbra spicata tRNA-Leu (trnL) gene and trnL-trnF intergenic spacer, partial sequence; chloroplast	861	861	93% 0.0	94% AY840207.1	870,26	83,1%
Select seq gb|GU381489.1|	Killickia lutea voucher NU<ZAF>:Hilliard & Burtt 9876 tRNA-Leu (trnL) gene and trnL-trnF intergenic spacer, partial sequence; chloroplast	850	850	92% 0.0	94% GU381489.1	868,48	82,9%
Select seq gb|GU381488.1|	Killickia compacta voucher M:Braeuchler 3816 tRNA-Leu (trnL) gene and trnL-trnF intergenic spacer, partial sequence; chloroplast	850	850	92% 0.0	94% GU381488.1	868,48	82,9%
Select seq gb|KF307419.1|	Lepechinia ganderi voucher B. Drew 24 tRNA-Leu (trnL) gene and trnL-trnF intergenic spacer, partial sequence; chloroplast	859	859	93% 0.0	94% KF307419.1	868,24	82,9%
Select seq gb|GU381485.1|	Micromeria sphaerophylla voucher E:Lewis et al 1064 tRNA-Leu (trnL) gene and trnL-trnF intergenic spacer, partial sequence; chloroplast	859	859	93% 0.0	94% GU381485.1	868,24	82,9%
Select seq gb|GU381484.1|	Micromeria flagellaris voucher E:van der Werff & McPherson 13570 tRNA-Leu (trnL) gene and trnL-trnF intergenic spacer, partial sequence; chloroplast	859	859	93% 0.0	94% GU381484.1	868,24	82,9%
Select seq gb|GU381483.1|	Micromeria flagellaris voucher E:Clement et al 2140 tRNA-Leu (trnL) gene and trnL-trnF intergenic spacer, partial sequence; chloroplast	859	859	93% 0.0	94% GU381483.1	868,24	82,9%
Select seq gb|GU381481.1|	Micromeria cf. madagascariensis Morawetz 205 tRNA-Leu (trnL) gene and trnL-trnF intergenic spacer, partial sequence; chloroplast	859	859	93% 0.0	94% GU381481.1	868,24	82,9%
Select seq gb|FJ593457.1|	Nepeta stewartiana tRNA-Leu (trnL) gene and trnL-trnF intergenic spacer, partial sequence; chloroplast	859	859	93% 0.0	94% FJ593457.1	868,24	82,9%
Select seq gb|GU381632.1|	Thymbra spicata voucher M:Braeuchler 4548 tRNA-Leu (trnL) gene and trnL-trnF intergenic spacer, partial sequence; chloroplast	856	856	93% 0.0	94% GU381632.1	865,20	82,6%
Select seq gb|JF301401.1|	Thymbra capitata voucher UCBG 96.0817 tRNA-Leu (trnL) gene and trnL-trnF intergenic spacer, partial sequence; chloroplast	854	854	93% 0.0	94% JF301401.1	863,18	82,4%
Select seq gb|GU381629.1|	Thymbra capitata voucher M:Braeuchler 2518 tRNA-Leu (trnL) gene and trnL-trnF intergenic spacer, partial sequence; chloroplast	854	854	93% 0.0	94% GU381629.1	863,18	82,4%
Select seq gb|JF301376.1|	Lepechinia caulescens voucher B. Drew 106 tRNA-Leu (trnL) gene and trnL-trnF intergenic spacer, partial sequence; chloroplast	852	852	93% 0.0	94% JF301376.1	861,16	82,2%
Select seq gb|AY570459.1|	Lepechinia chamaedryoides voucher JBW 2537 tRNA-Leu and trnL-trnF intergenic spacer, partial sequence; chloroplast	852	852	93% 0.0	94% AY570459.1	861,16	82,2%
Select seq gb|KF307429.1|	Lepechinia salviae voucher R. Jabaily s.n. tRNA-Leu (trnL) gene and trnL-trnF intergenic spacer, partial sequence; chloroplast	850	850	93% 0.0	94% KF307429.1	859,14	82,0%
Select seq gb|KF307415.1|	Lepechinia caulescens voucher B. Drew 149 tRNA-Leu (trnL) gene and trnL-trnF intergenic spacer, partial sequence; chloroplast	850	850	93% 0.0	94% KF307415.1	859,14	82,0%
Select seq gb|GU381627.1|	Thymbra sintenisii subsp. isaurica voucher E:Goener 12628 tRNA-Leu (trnL) gene and trnL-trnF intergenic spacer, partial sequence; chloroplast	848	848	93% 0.0	94% GU381627.1	857,12	81,8%
Select seq gb|JF301378.1|	Lepechinia hastata voucher B. Drew 44 tRNA-Leu (trnL) gene and trnL-trnF intergenic spacer, partial sequence; chloroplast	846	846	93% 0.0	94% JF301378.1	855,10	81,6%
Select seq gb|JF301360.1|	Cedronella canariensis voucher UCBG 2004.0788 tRNA-Leu (trnL) gene and trnL-trnF intergenic spacer, partial sequence; chloroplast	845	845	93% 0.0	94% JF301360.1	854,09	81,5%
Select seq gb|GU381628.1|	Thymbra calostachya voucher M:Ulrich s.n. tRNA-Leu (trnL) gene and trnL-trnF intergenic spacer, partial sequence; chloroplast	845	845	93% 0.0	94% GU381628.1	854,09	81,5%
Select seq gb|AY506622.1|	Cedronella canariensis tRNA-Leu (trnL) gene and trnL-trnF intergenic spacer, partial sequence; chloroplast	845	845	93% 0.0	94% AY506622.1	854,09	81,5%
Select seq gb|GU381517.1|	Clinopodium barosmum voucher BM<GBR-LONDON>:McLaren N193 tRNA-Leu (trnL) gene and trnL-trnF intergenic spacer, partial sequence; chloroplast	843	843	93% 0.0	94% GU381517.1	852,06	81,3%

Select seq gb|GU381516.1|	Clinopodium wardii voucher BM<GBR-LONDON>:Ludlow et al. 14234 tRNA-Leu (trnL) gene and trnL-trnF intergenic spacer, partial sequence; chloroplast	843	843	93% 0.0	94% GU381516.1	852,06	81,3%
Select seq gb|GU381515.1|	Clinopodium hydaspidis voucher BM<GBR-LONDON>:Mohd 133 tRNA-Leu (trnL) gene and trnL-trnF intergenic spacer, partial sequence; chloroplast	843	843	93% 0.0	94% GU381515.1	852,06	81,3%
Select seq gb|GU381514.1|	Clinopodium nepalense voucher FR:Stainton 6024 tRNA-Leu (trnL) gene and trnL-trnF intergenic spacer, partial sequence; chloroplast	843	843	93% 0.0	94% GU381514.1	852,06	81,3%
Select seq gb|GU381513.1|	Clinopodium nepalense voucher BM<GBR-LONDON>:Mikage et al. 9550294 tRNA-Leu (trnL) gene and trnL-trnF intergenic spacer, partial sequence; chloroplast	843	843	93% 0.0	94% GU381513.1	852,06	81,3%
Select seq gb|GU381511.1|	Clinopodium piperitum voucher BM<GBR-LONDON>:Vickery 454 tRNA-Leu (trnL) gene and trnL-trnF intergenic spacer, partial sequence; chloroplast	843	843	93% 0.0	94% GU381511.1	852,06	81,3%
Select seq gb|KF307417.1|	Lepechinia dioica voucher B. Drew 232 tRNA-Leu (trnL) gene and trnL-trnF intergenic spacer, partial sequence; chloroplast	839	839	93% 0.0	94% KF307417.1	848,02	81,0%
Select seq gb|JF301386.1|	Melissa officinalis voucher B. Drew 70 tRNA-Leu (trnL) gene and trnL-trnF intergenic spacer, partial sequence; chloroplast	839	839	93% 0.0	94% JF301386.1	848,02	81,0%
Select seq gb|DQ667477.1|	Melissa officinalis isolate x193 tRNA-Leu (trnL) gene and trnL-trnF intergenic spacer, partial sequence; chloroplast	839	839	93% 0.0	94% DQ667477.1	848,02	81,0%
Select seq emb|AJ505529.1|	Melissa officinalis plastid trnL-trnF intergenic spacer, specimen voucher Catino	839	839	93% 0.0	94% AJ505529.1	848,02	81,0%
Select seq gb|JF301379.1|	Lepechinia lamiifolia voucher B. Drew 178 tRNA-Leu (trnL) gene and trnL-trnF intergenic spacer, partial sequence; chloroplast	833	833	93% 0.0	94% JF301379.1	841,96	80,4%
Select seq gb|KR150243.1|	Ziziphora sp. 2 Kh20 trnL-trnF intergenic spacer region, partial sequence; chloroplast	837	837	93% 0.0	93% KR150243.1	837,00	79,9%
Select seq gb|KR150238.1|	Ziziphora sp. 1 Kh75 trnL-trnF intergenic spacer region, partial sequence; chloroplast	837	837	93% 0.0	93% KR150238.1	837,00	79,9%
Select seq gb|KR150198.1|	Ziziphora sp. 3 Kh112 trnL-trnF intergenic spacer region, partial sequence; chloroplast	837	837	93% 0.0	93% KR150198.1	837,00	79,9%
Select seq gb|GU381512.1|	Clinopodium piperitum voucher E:Stainton 7320 tRNA-Leu (trnL) gene and trnL-trnF intergenic spacer, partial sequence; chloroplast	837	837	93% 0.0	93% GU381512.1	837,00	79,9%
Select seq gb|GU381510.1|	Ziziphora pamiroalaica voucher C:Murray et al. 10090 tRNA-Leu (trnL) gene and trnL-trnF intergenic spacer, partial sequence; chloroplast	837	837	93% 0.0	93% GU381510.1	837,00	79,9%
Select seq gb|GU381507.1|	Ziziphora tenuior voucher MSB:Fayvush et al. 03-1503 tRNA-Leu (trnL) gene and trnL-trnF intergenic spacer, partial sequence; chloroplast	837	837	93% 0.0	93% GU381507.1	837,00	79,9%
Select seq gb|GU381506.1|	Ziziphora tenuior voucher MSB:Nydegger 43557 tRNA-Leu (trnL) gene and trnL-trnF intergenic spacer, partial sequence; chloroplast	837	837	93% 0.0	93% GU381506.1	837,00	79,9%
Select seq gb|GU381505.1|	Clinopodium troodi voucher W:Davis 1856 tRNA-Leu (trnL) gene and trnL-trnF intergenic spacer, partial sequence; chloroplast	837	837	93% 0.0	93% GU381505.1	837,00	79,9%
Select seq gb|GU381503.1|	Ziziphora hispanica voucher M:Lippert 24827 tRNA-Leu (trnL) gene and trnL-trnF intergenic spacer, partial sequence; chloroplast	837	837	93% 0.0	93% GU381503.1	837,00	79,9%
Select seq gb|GU381502.1|	Ziziphora hispanica subsp. aragonensis voucher M:Podlech & Lippert 2492 tRNA-Leu (trnL) gene and trnL-trnF intergenic spacer, partial sequence; chloroplast	837	837	93% 0.0	93% GU381502.1	837,00	79,9%
Select seq gb|GU381501.1|	Clinopodium graveolens subsp. rotundifolium voucher M:Podlech 47181 tRNA-Leu (trnL) gene and trnL-trnF intergenic spacer, partial sequence; chloroplast	837	837	93% 0.0	93% GU381501.1	837,00	79,9%
Select seq gb|GU381499.1|	Clinopodium suaveolens voucher M:Erben s.n. tRNA-Leu (trnL) gene and trnL-trnF intergenic spacer, partial sequence; chloroplast	837	837	93% 0.0	93% GU381499.1	837,00	79,9%
Select seq gb|DQ667501.1|	Ziziphora taurica isolate x262 tRNA-Leu (trnL) gene and trnL-trnF intergenic spacer, partial sequence; chloroplast	837	837	93% 0.0	93% DQ667501.1	837,00	79,9%
Select seq gb|AY506621.1|	Nepeta grandiflora tRNA-Leu (trnL) gene and trnL-trnF intergenic spacer, partial sequence; chloroplast	896	896	100% 0.0	93% AY506621.1	833,28	79,6%
Select seq gb|GU381522.1|	Mentha cervina voucher M:Braeuchler 2394 tRNA-Leu (trnL) gene and trnL-trnF intergenic spacer, partial sequence; chloroplast	833	833	93% 0.0	93% GU381522.1	833,00	79,5%
Select seq gb|AY618521.1|	Mentha longifolia tRNA-Leu (trnL) gene and trnL-trnF intergenic spacer, partial sequence; chloroplast	833	833	93% 0.0	93% AY618521.1	833,00	79,5%
Select seq gb|KR063657.1|	Thymus sibthorpii tRNA-Leu (trnL) gene, partial sequence; trnL-trnF intergenic spacer, complete sequence; and tRNA-Phe (trnF) gene, partial sequence; chloroplast	832	832	93% 0.0	93% KR063657.1	832,00	79,4%
Select seq gb|KR150218.1|	Thymus sp. 2 Kh69 trnL-trnF intergenic spacer region, partial sequence; chloroplast	832	832	93% 0.0	93% KR150218.1	832,00	79,4%

Select seq gb|KM886649.1|	Prunella vulgaris voucher SNJ Exped. 20110719005 (KUN) tRNA-Leu (trnL) gene, partial sequence; trnL-trnF intergenic spacer, complete sequence; and tRNA-Phe (trnF) gene, partial sequence; plastid	880	880	100% 0.0	93%	KM886649.1	818,40	78,1%
Select seq gb|EF153679.1|	Prunella grandiflora tRNA-Leu (trnL) and trnL-trnF intergenic spacer, partial sequence; chloroplast	880	880	100% 0.0	93%	EF153679.1	818,40	78,1%
Select seq gb|DQ667508.1|	Prunella vulgaris isolate x314 tRNA-Leu (trnL) gene and trnL-trnF intergenic spacer, partial sequence; chloroplast	880	880	100% 0.0	93%	DQ667508.1	818,40	78,1%
Select seq gb|AY506619.1|	Prunella vulgaris tRNA-Leu (trnL) gene and trnL-trnF intergenic spacer, partial sequence; chloroplast	880	880	100% 0.0	93%	AY506619.1	818,40	78,1%
Select seq gb|JF301384.1|	Lophanthus lipskyanus voucher Vassiljeva s.n. tRNA-Leu (trnL) gene and trnL-trnF intergenic spacer, partial sequence; chloroplast	833	833	93% 0.0	91%	JF301384.1	815,09	77,8%
Select seq gb|EF153681.1|	Prunella asiatica tRNA-Leu (trnL) and trnL-trnF intergenic spacer, partial sequence; chloroplast	874	874	100% 0.0	93%	EF153681.1	812,82	77,6%
Select seq gb|KR150191.1|	Nepeta sp. Kh55 trnL-trnF intergenic spacer region, partial sequence; chloroplast	870	870	100% 0.0	93%	KR150191.1	809,10	77,2%
Select seq gb|KF307414.1|	Lepechinia calycina voucher B. Drew 20 tRNA-Leu (trnL) gene and trnL-trnF intergenic spacer, partial sequence; chloroplast	850	925	99% 0.0	94%	KF307414.1	807,07	77,1%
Select seq gb|JF301375.1|	Lepechinia calycina voucher B. Drew 197 tRNA-Leu (trnL) gene and trnL-trnF intergenic spacer, partial sequence; chloroplast	852	927	100% 0.0	94%	JF301375.1	800,88	76,5%
Select seq gb|DQ667494.1|	Lepechinia calycina isolate x246 tRNA-Leu (trnL) gene and trnL-trnF intergenic spacer, partial sequence; chloroplast	852	927	100% 0.0	94%	DQ667494.1	800,88	76,5%
Select seq gb|AY570460.1|	Lepechinia fragrans voucher JBW 1333 tRNA-Leu and trnL-trnF intergenic spacer, partial sequence; chloroplast	852	927	100% 0.0	94%	AY570460.1	800,88	76,5%
Select seq gb|AY570458.1|	Lepechinia calycina voucher JBW 1344 tRNA-Leu and trnL-trnF intergenic spacer, partial sequence; chloroplast	845	920	100% 0.0	94%	AY570458.1	794,30	75,8%
Select seq gb|EF153682.1|	Prunella hispida tRNA-Leu (trnL) and trnL-trnF intergenic spacer, partial sequence; chloroplast	850	850	100% 0.0	92%	EF153682.1	782,00	74,7%
							0,00	0,0%
							0,00	0,0%
							0,00	0,0%

Select for downloading or viewing reports	Kh053_ITS Description	Max score	Total score	Query cover	E value	Ident	Accession	(Ident/Cover)* Max score	Deviation from top hit
Select seq gb|DQ667325.1|	Mentha arvensis isolate x424 18S ribosomal RNA gene, partial sequence; internal transcribed spacer 1, 5.8S ribosomal RNA gene, and internal transcribed spacer 2, complete sequence; and 28S ribosomal RNA gene, partial sequence	508	508	96%	4,00E-140	84%	DQ667325.1	444,50	100,0%
Select seq gb|JQ669115.1|	Mentha arvensis voucher B. Drew 82 18S ribosomal RNA gene, internal transcribed spacer 1, 5.8S ribosomal RNA gene, internal transcribed spacer 2, and 26S ribosomal RNA gene, region	508	508	96%	4,00E-140	83%	JQ669115.1	439,21	98,8%
Select seq gb|KF735666.1|	Mentha aquatica strain HY 18S ribosomal RNA gene, partial sequence; internal transcribed spacer 1 and 5.8S ribosomal RNA gene, complete sequence; and internal transcribed spacer 2, partial sequence	490	490	94%	1,00E-134	83%	KF735666.1	432,66	97,3%
Select seq gb|KF735667.1|	Mentha aquatica strain HY1 18S ribosomal RNA gene, partial sequence; internal transcribed spacer 1 and 5.8S ribosomal RNA gene, complete sequence; and internal transcribed spacer 2, partial sequence	479	479	94%	3,00E-131	83%	KF735667.1	422,95	95,2%
Select seq gb|DQ667333.1|	Acanthomintha lanceolata isolate x545 18S ribosomal RNA gene, partial sequence; internal transcribed spacer 1, 5.8S ribosomal RNA gene, and internal transcribed spacer 2, complete sequence; and 28S ribosomal RNA gene, partial sequence	484	484	95%	6,00E-133	83%	DQ667333.1	422,86	95,1%
Select seq gb|JQ669121.1|	Clinopodium thymifolium voucher M. Kintgen s.n. 18S ribosomal RNA gene, internal transcribed spacer 1, 5.8S ribosomal RNA gene, internal transcribed spacer 2, and 26S ribosomal RNA gene, region	486	486	96%	2,00E-133	83%	JQ669121.1	420,19	94,5%
Select seq gb|KF735668.1|	Mentha aquatica strain BS 18S ribosomal RNA gene, partial sequence; internal transcribed spacer 1 and 5.8S ribosomal RNA gene, complete sequence; and internal transcribed spacer 2, partial sequence	468	468	94%	6,00E-128	82%	KF735668.1	408,26	91,8%
Select seq gb|JQ230966.1|	Mentha x piperita voucher SBB-1151 internal transcribed spacer 1, partial sequence; 5.8S ribosomal RNA gene and internal transcribed spacer 2, complete sequence; and 28S ribosomal RNA gene, partial sequence	440	440	89%	1,00E-119	82%	JQ230966.1	405,39	91,2%
Select seq gb|KC591661.1|	Mentha arvensis voucher UC1862058 internal transcribed spacer 1, partial sequence; 5.8S ribosomal RNA gene and internal transcribed spacer 2, complete sequence; and 28S ribosomal RNA gene, partial sequence	425	425	86%	4,00E-115	82%	KC591661.1	405,23	91,2%
Select seq gb|JQ669122.1|	Minthostachys mollis voucher B. Drew 345 18S ribosomal RNA gene, internal transcribed spacer 1, 5.8S ribosomal RNA gene, internal transcribed spacer 2, and 26S ribosomal RNA gene, region	472	472	96%	5,00E-129	82%	JQ669122.1	403,17	90,7%
Select seq gb|JQ669093.1|	Cunila pycnantha voucher Ruiz 3150 18S ribosomal RNA gene, internal transcribed spacer 1, 5.8S ribosomal RNA gene, internal transcribed spacer 2, and 26S ribosomal RNA gene, region	470	470	96%	2,00E-128	82%	JQ669093.1	401,46	90,3%
Select seq gb|JQ669090.1|	Cunila lythrifolia voucher Rzedowski 251 18S ribosomal RNA gene, internal transcribed spacer 1, 5.8S ribosomal RNA gene, internal transcribed spacer 2, and 26S ribosomal RNA gene, region	470	470	96%	2,00E-128	82%	JQ669090.1	401,46	90,3%
Select seq gb|JQ669134.1|	Rhododon ciliatus voucher Singhurst s.n. 18S ribosomal RNA gene, internal transcribed spacer 1, 5.8S ribosomal RNA gene, internal transcribed spacer 2, and 26S ribosomal RNA gene, region	401	401	82%	6,00E-108	82%	JQ669134.1	401,00	90,2%
Select seq gb|JQ669118.1|	Clinopodium dalmaticum voucher M. Kintgen s.n. 18S ribosomal RNA gene, internal transcribed spacer 1, 5.8S ribosomal RNA gene, internal transcribed spacer 2, and 26S ribosomal RNA gene, region	433	433	90%	2,00E-117	82%	JQ669118.1	394,51	88,8%
Select seq gb|JQ669073.1|	Acanthomintha lanceolata voucher Crosby & Morin 14383 18S ribosomal RNA gene, internal transcribed spacer 1, 5.8S ribosomal RNA gene, internal transcribed spacer 2, and 26S ribosomal RNA gene, region	433	433	90%	2,00E-117	82%	JQ669073.1	394,51	88,8%
Select seq gb|DQ667310.1|	Glechon thymoides isolate x256 18S ribosomal RNA gene, partial sequence; internal transcribed spacer 1, 5.8S ribosomal RNA gene, and internal transcribed spacer 2, complete sequence; and 28S ribosomal RNA gene, partial sequence	459	459	96%	4,00E-125	82%	DQ667310.1	392,06	88,2%
Select seq gb|DQ667312.1|	Rhododon ciliatus isolate x260 18S ribosomal RNA gene, partial sequence; internal transcribed spacer 1, 5.8S ribosomal RNA gene, and internal transcribed spacer 2, complete sequence; and 28S ribosomal RNA gene, partial sequence	387	387	81%	2,00E-103	82%	DQ667312.1	391,78	88,1%
Select seq gb|JQ669081.1|	Micromeria douglasii voucher B. Drew 206 18S ribosomal RNA gene, internal transcribed spacer 1, 5.8S ribosomal RNA gene, internal transcribed spacer 2, and 26S ribosomal RNA gene, region	457	457	96%	1,00E-124	82%	JQ669081.1	390,35	87,8%
Select seq gb|KC591663.1|	Acanthomintha lanceolata voucher SDSU17310 internal transcribed spacer 1, partial sequence; 5.8S ribosomal RNA gene and internal transcribed spacer 2, complete sequence; and 28S ribosomal RNA gene, partial sequence	409	409	86%	4,00E-110	82%	KC591663.1	389,98	87,7%
Select seq gb|KR150160.1|	Satureja sp. Kh90 internal transcribed spacer 1, partial sequence; 5.8S ribosomal RNA gene gene, complete sequence; and internal transcribed spacer 2 region, partial sequence	346	346	73%	3,00E-91	82%	KR150160.1	388,66	87,4%
Select seq gb|JQ669125.1|	Monardella villosa voucher B. Drew 66 18S ribosomal RNA gene, internal transcribed spacer 1, 5.8S ribosomal RNA gene, internal transcribed spacer 2, and 26S ribosomal RNA gene, region	455	455	96%	5,00E-124	82%	JQ669125.1	388,65	87,4%
Select seq gb|JQ669083.1|	Clinopodium macrostemum voucher B. Drew 147 18S ribosomal RNA gene, internal transcribed spacer 1, 5.8S ribosomal RNA gene, internal transcribed spacer 2, and 26S ribosomal RNA gene, region	455	455	96%	5,00E-124	82%	JQ669083.1	388,65	87,4%
Select seq gb|DQ667311.1|	Poliomintha palmeri isolate x259 18S ribosomal RNA gene, partial sequence; internal transcribed spacer 1, 5.8S ribosomal RNA gene, and internal transcribed spacer 2, complete sequence; and 28S ribosomal RNA gene, partial sequence	453	453	96%	2,00E-123	82%	DQ667311.1	386,94	87,1%
Select seq gb|JQ669089.1|	Cuminia fernandezia voucher Stuessy et al., 11580 18S ribosomal RNA gene, internal transcribed spacer 1, 5.8S ribosomal RNA gene, internal transcribed spacer 2, and 26S ribosomal RNA gene, region	424	424	90%	1,00E-114	82%	JQ669089.1	386,31	86,9%
Select seq gb|DQ667244.1|	Mentha spicata isolate x077 18S ribosomal RNA gene, partial sequence; internal transcribed spacer 1, 5.8S ribosomal RNA gene, and internal transcribed spacer 2, complete sequence; and 28S ribosomal RNA gene, partial sequence	398	398	85%	8,00E-107	82%	DQ667244.1	383,95	86,4%
Select seq gb|DQ667303.1|	Glechon marifolia isolate x223 18S ribosomal RNA gene, partial sequence; internal transcribed spacer 1, 5.8S ribosomal RNA gene, and internal transcribed spacer 2, complete sequence; and 28S ribosomal RNA gene, partial sequence	448	448	96%	8,00E-122	82%	DQ667303.1	382,67	86,1%

Select seq emb|AJ421000.1|	Agastache cana 5.8S rRNA gene, internal transcribed spacer 1 (ITS1) and internal transcribed spacer 2 (ITS2)	396	396	86%	3,00E-106	82%	AJ421000.1	377,58	84,9%
Select seq gb|JQ669123.1|	Minthostachys mollis voucher B. Drew 349 18S ribosomal RNA gene, internal transcribed spacer 1, 5.8S ribosomal RNA gene, internal transcribed spacer 2, and 26S ribosomal RNA gene, region	416	416	90%	2,00E-112	81%	JQ669123.1	374,40	84,2%
Select seq gb|DQ667237.1|	Clinopodium ashei isolate x068 18S ribosomal RNA gene, partial sequence; internal transcribed spacer 1, 5.8S ribosomal RNA gene, and internal transcribed spacer 2, complete sequence; and 28S ribosomal RNA gene, partial sequence	396	396	86%	3,00E-106	81%	DQ667237.1	372,98	83,9%
Select seq gb|DQ667305.1|	Cunila microcephala isolate x226 18S ribosomal RNA gene, partial sequence; internal transcribed spacer 1, 5.8S ribosomal RNA gene, and internal transcribed spacer 2, complete sequence; and 28S ribosomal RNA gene, partial sequence	442	442	96%	4,00E-120	81%	DQ667305.1	372,94	83,9%
Select seq gb|JQ669094.1|	Cyclotrichium stamineum voucher Gillett 9444 18S ribosomal RNA gene, internal transcribed spacer 1, 5.8S ribosomal RNA gene, internal transcribed spacer 2, and 26S ribosomal RNA gene, region	385	385	85%	7,00E-103	81%	JQ669094.1	366,88	82,5%
Select seq gb|DQ667316.1|	Cunila incana isolate x296 18S ribosomal RNA gene, partial sequence; internal transcribed spacer 1, 5.8S ribosomal RNA gene, and internal transcribed spacer 2, complete sequence; and 28S ribosomal RNA gene, partial sequence	433	433	96%	2,00E-117	81%	DQ667316.1	365,34	82,2%
Select seq gb|JQ669072.1|	Acanthomintha duttonii voucher UCBG H. Forbes s.n. 18S ribosomal RNA gene, internal transcribed spacer 1, 5.8S ribosomal RNA gene, internal transcribed spacer 2, and 26S ribosomal RNA gene, region	425	425	95%	4,00E-115	81%	JQ669072.1	362,37	81,5%
Select seq gb|AY506635.1|	Bystropogon origanifolius internal transcribed spacer 1, partial sequence; 5.8S ribosomal RNA gene, complete sequence; and internal transcribed spacer 2, partial sequence	344	344	77%	1,00E-90	81%	AY506635.1	361,87	81,4%
Select seq emb|AJ421001.1|	Agastache barberi 5.8S rRNA gene, internal transcribed spacer 1 (ITS1) and internal transcribed spacer 2 (ITS2)	396	396	89%	3,00E-106	81%	AJ421001.1	360,40	81,1%
Select seq gb|DQ667233.1|	Clinopodium coccineum isolate x058 18S ribosomal RNA gene, partial sequence; internal transcribed spacer 1, 5.8S ribosomal RNA gene, and internal transcribed spacer 2, complete sequence; and 28S ribosomal RNA gene, partial sequence	427	427	96%	1,00E-115	81%	DQ667233.1	360,28	81,1%
Select seq gb|JQ669078.1|	Bystropogon origanifolius voucher B. Drew s.n. 18S ribosomal RNA gene, internal transcribed spacer 1, 5.8S ribosomal RNA gene, internal transcribed spacer 2, and 26S ribosomal RNA gene, region	418	418	94%	6,00E-113	81%	JQ669078.1	360,19	81,0%
Select seq gb|DQ667313.1|	Schizonepeta multifida isolate x261 18S ribosomal RNA gene, partial sequence; internal transcribed spacer 1, 5.8S ribosomal RNA gene, and internal transcribed spacer 2, complete sequence; and 28S ribosomal RNA gene, partial sequence	357	357	81%	1,00E-94	81%	DQ667313.1	357,00	80,3%
Select seq gb|KC473228.1|	Mentha canadensis isolate 511190201 18S ribosomal RNA gene, partial sequence; internal transcribed spacer 1, 5.8S ribosomal RNA gene, and internal transcribed spacer 2, complete sequence; and 28S ribosomal RNA gene, partial sequence	361	361	82%	1,00E-95	81%	KC473228.1	356,60	80,2%
Select seq gb|KC591662.1|	Acanthomintha ilicifolia voucher SDSU12198 internal transcribed spacer 1, partial sequence; 5.8S ribosomal RNA gene and internal transcribed spacer 2, complete sequence; and 28S ribosomal RNA gene, partial sequence	375	375	86%	4,00E-100	81%	KC591662.1	353,20	79,5%
Select seq gb|EU383034.1|	Schizonepeta tenuifolia internal transcribed spacer 1, partial sequence; 5.8S ribosomal RNA gene, complete sequence; and internal transcribed spacer 2, partial sequence	387	387	89%	2,00E-103	81%	EU383034.1	352,21	79,2%
Select seq emb|AJ420997.1|	Lallemantia peltata 5.8S rRNA gene, internal transcribed spacer 1 (ITS1) and internal transcribed spacer 2 (ITS2)	364	364	84%	8,00E-97	81%	AJ420997.1	351,00	79,0%
Select seq gb|AY506636.1|	Cuminia fernandezia internal transcribed spacer 1, partial sequence; 5.8S ribosomal RNA gene, complete sequence; and internal transcribed spacer 2, partial sequence	372	372	86%	5,00E-99	81%	AY506636.1	350,37	78,8%
Select seq emb|AJ420999.1|	Dracocephalum grandiflorum 5.8S rRNA gene, internal transcribed spacer 1 (ITS1) and internal transcribed spacer 2 (ITS2)	361	361	84%	1,00E-95	81%	AJ420999.1	348,11	78,3%
Select seq gb|KM051459.1|	Schizonepeta tenuifolia isolate TKM201404 18S ribosomal RNA gene, partial sequence; internal transcribed spacer 1, 5.8S ribosomal RNA gene, and internal transcribed spacer 2, complete sequence; and 26S ribosomal RNA gene, partial sequence	377	377	88%	1,00E-100	81%	KM051459.1	347,01	78,1%
Select seq gb|JQ669128.1|	Pogogyne douglasii voucher UCBG 91.1071 s.n. 18S ribosomal RNA gene, internal transcribed spacer 1, 5.8S ribosomal RNA gene, internal transcribed spacer 2, and 26S ribosomal RNA gene, region	412	412	96%	3,00E-111	80%	JQ669128.1	343,33	77,2%
Select seq emb|AJ420998.1|	Dracocephalum kotschyi 5.8S rRNA gene, internal transcribed spacer 1 (ITS1) and internal transcribed spacer 2 (ITS2)	359	359	84%	4,00E-95	80%	AJ420998.1	341,90	76,9%
Select seq gb|DQ667286.1|	Salvia aucheri var. canescens isolate x181 18S ribosomal RNA gene, partial sequence; internal transcribed spacer 1, 5.8S ribosomal RNA gene, and internal transcribed spacer 2, complete sequence; and 28S ribosomal RNA gene, partial sequence	346	346	81%	3,00E-91	80%	DQ667286.1	341,73	76,9%
Select seq gb|DQ667331.1|	Pogogyne floribunda isolate x534 18S ribosomal RNA gene, partial sequence; internal transcribed spacer 1, 5.8S ribosomal RNA gene, and internal transcribed spacer 2, complete sequence; and 28S ribosomal RNA gene, partial sequence	409	409	96%	4,00E-110	80%	DQ667331.1	340,83	76,7%
Select seq gb|DQ667332.1|	Salvia cynica isolate x537 18S ribosomal RNA gene, partial sequence; internal transcribed spacer 1, 5.8S ribosomal RNA gene, and internal transcribed spacer 2, complete sequence; and 28S ribosomal RNA gene, partial sequence	353	353	83%	2,00E-93	80%	DQ667332.1	340,24	76,5%
Select seq gb|JQ669076.1|	Agastache rugosa voucher H. Kanai, K Hasagawa, K. Ohkubo 8916 18S ribosomal RNA gene, internal transcribed spacer 1, 5.8S ribosomal RNA gene, internal transcribed spacer 2, and 26S ribosomal RNA gene, region	348	348	82%	9,00E-92	80%	JQ669076.1	339,51	76,4%
Select seq gb|JQ669080.1|	Clinopodium arkansanum voucher B. Drew 80 18S ribosomal RNA gene, internal transcribed spacer 1, 5.8S ribosomal RNA gene, internal transcribed spacer 2, and 26S ribosomal RNA gene, region	381	381	90%	8,00E-102	80%	JQ669080.1	338,67	76,2%
Select seq gb|JN802670.1|	Schizonepeta tenuifolia clone HB11 18S ribosomal RNA gene, partial sequence; internal transcribed spacer 1, 5.8S ribosomal RNA gene, and internal transcribed spacer 2, complete sequence; and 28S ribosomal RNA gene, partial sequence	364	364	86%	8,00E-97	80%	JN802670.1	338,60	76,2%
Select seq emb|AJ616321.1|	Halleria elliptica ITS1 (partial), 5.8S rRNA gene and ITS2 (partial)	359	359	85%	4,00E-95	80%	AJ616321.1	337,88	76,0%

Select seq gb|KF800643.1|	Uncultured eukaryote clone CMH554 18S ribosomal RNA gene, partial sequence; internal transcribed spacer 1, 5.8S ribosomal RNA gene, and internal transcribed spacer 2, complete sequence; and 28S ribosomal RNA gene, partial sequence	375	375	89%	4,00E-100	80%	KF800643.1	337,08	75,8%
Select seq gb|DQ667234.1|	Dicerandra odoratissima isolate x059 18S ribosomal RNA gene, partial sequence; internal transcribed spacer 1, 5.8S ribosomal RNA gene, and internal transcribed spacer 2, complete sequence; and 28S ribosomal RNA gene, partial sequence	403	403	96%	2,00E-108	80%	DQ667234.1	335,83	75,6%
Select seq gb|DQ667255.1|	Salvia digitaloides isolate x112 18S ribosomal RNA gene, partial sequence; internal transcribed spacer 1, 5.8S ribosomal RNA gene, and internal transcribed spacer 2, complete sequence; and 28S ribosomal RNA gene, partial sequence	339	339	81%	5,00E-89	80%	DQ667255.1	334,81	75,3%
Select seq gb|AY506637.1|	Monardella hypoleuca internal transcribed spacer 1, partial sequence; 5.8S ribosomal RNA gene, complete sequence; and internal transcribed spacer 2, partial sequence	363	363	87%	3,00E-96	80%	AY506637.1	333,79	75,1%
Select seq gb|KJ397260.1|	Salvia przewalskii voucher SP201301 18S ribosomal RNA gene, partial sequence; internal transcribed spacer 1, 5.8S ribosomal RNA gene, and internal transcribed spacer 2, complete sequence; and 26S ribosomal RNA gene, partial sequence	337	337	81%	2,00E-88	80%	KJ397260.1	332,84	74,9%
Select seq gb|KJ397259.1|	Salvia digitaloides voucher SD201301 18S ribosomal RNA gene, partial sequence; internal transcribed spacer 1, 5.8S ribosomal RNA gene, and internal transcribed spacer 2, complete sequence; and 26S ribosomal RNA gene, partial sequence	337	337	81%	2,00E-88	80%	KJ397259.1	332,84	74,9%
Select seq gb|DQ667330.1|	Perovskia scrophulariifolia isolate x532 18S ribosomal RNA gene, partial sequence; internal transcribed spacer 1, 5.8S ribosomal RNA gene, and internal transcribed spacer 2, complete sequence; and 28S ribosomal RNA gene, partial sequence	355	355	86%	5,00E-94	80%	DQ667330.1	330,23	74,3%
Select seq gb|DQ667247.1|	Agastache urticifolia isolate x090 18S ribosomal RNA gene, partial sequence; internal transcribed spacer 1, 5.8S ribosomal RNA gene, and internal transcribed spacer 2, complete sequence; and 28S ribosomal RNA gene, partial sequence	350	350	85%	2,00E-92	80%	DQ667247.1	329,41	74,1%
Select seq gb|JQ669117.1|	Mentha pulegium voucher Riina 1574 18S ribosomal RNA gene, internal transcribed spacer 1, 5.8S ribosomal RNA gene, internal transcribed spacer 2, and 26S ribosomal RNA gene, region	394	394	96%	1,00E-105	80%	JQ669117.1	328,33	73,9%
Select seq gb|DQ667289.1|	Salvia roborowskii isolate x189 18S ribosomal RNA gene, partial sequence; internal transcribed spacer 1, 5.8S ribosomal RNA gene, and internal transcribed spacer 2, complete sequence; and 28S ribosomal RNA gene, partial sequence	348	348	85%	9,00E-92	80%	DQ667289.1	327,53	73,7%
Select seq gb|JQ669096.1|	Dracocephalum bullatum voucher Boufford et al. 31785 18S ribosomal RNA gene, internal transcribed spacer 1, 5.8S ribosomal RNA gene, internal transcribed spacer 2, and 26S ribosomal RNA gene, region	331	331	81%	9,00E-87	80%	JQ669096.1	326,91	73,5%
Select seq gb|JQ669099.1|	Glechoma hederacea voucher B. Drew 69 18S ribosomal RNA gene, internal transcribed spacer 1, 5.8S ribosomal RNA gene, internal transcribed spacer 2, and 26S ribosomal RNA gene, region	351	351	86%	7,00E-93	80%	JQ669099.1	326,51	73,5%
Select seq gb|DQ667254.1|	Salvia przewalskii isolate x111 18S ribosomal RNA gene, partial sequence; internal transcribed spacer 1, 5.8S ribosomal RNA gene, and internal transcribed spacer 2, complete sequence; and 28S ribosomal RNA gene, partial sequence	333	333	82%	2,00E-87	80%	DQ667254.1	324,88	73,1%
Select seq gb|AY506634.1|	Bystropogon canariensis internal transcribed spacer 1, partial sequence; 5.8S ribosomal RNA gene, complete sequence; and internal transcribed spacer 2, partial sequence	353	353	87%	2,00E-93	80%	AY506634.1	324,60	73,0%
Select seq dbj|AB557591.1|	Schizonepeta tenuifolia genes for 18S rRNA, ITS1, 5.8S rRNA, ITS2, 26S rRNA, partial and complete sequence	340	340	84%	1,00E-89	80%	AB557591.1	323,81	72,8%
Select seq emb|AJ616322.1|	Halleria ovata ITS1 (partial), 5.8S rRNA gene and ITS2 (partial)	344	344	85%	1,00E-90	80%	AJ616322.1	323,76	72,8%
Select seq gb|DQ667302.1|	Lycopus uniflorus isolate x221 18S ribosomal RNA gene, partial sequence; internal transcribed spacer 1, 5.8S ribosomal RNA gene, and internal transcribed spacer 2, complete sequence; and 28S ribosomal RNA gene, partial sequence	346	346	86%	3,00E-91	80%	DQ667302.1	321,86	72,4%
Select seq gb|DQ667291.1|	Melissa officinalis isolate x193 18S ribosomal RNA gene, partial sequence; internal transcribed spacer 1, 5.8S ribosomal RNA gene, and internal transcribed spacer 2, complete sequence; and 28S ribosomal RNA gene, partial sequence	346	346	86%	3,00E-91	80%	DQ667291.1	321,86	72,4%
Select seq gb|KR150165.1|	Thymus sp. Kh8 internal transcribed spacer 1, partial sequence; 5.8S ribosomal RNA gene gene, complete sequence; and internal transcribed spacer 2 region, partial sequence	351	351	89%	7,00E-93	80%	KR150165.1	315,51	71,0%
Select seq gb|KR150146.1|	Thymus sp. Kh57 internal transcribed spacer 1, partial sequence; 5.8S ribosomal RNA gene gene, complete sequence; and internal transcribed spacer 2 region, partial sequence	351	351	89%	7,00E-93	80%	KR150146.1	315,51	71,0%
Select seq gb|JQ669114.1|	Melissa axillaris voucher D.E. Boufford et al., 24526 18S ribosomal RNA gene, internal transcribed spacer 1, 5.8S ribosomal RNA gene, internal transcribed spacer 2, and 26S ribosomal RNA gene, region	335	335	85%	7,00E-88	79%	JQ669114.1	311,35	70,0%
Select seq gb|JQ669079.1|	Cedronella canariensis voucher UCBG #2004.0788 Royl 6859 18S ribosomal RNA gene, internal transcribed spacer 1, 5.8S ribosomal RNA gene, internal transcribed spacer 2, and 26S ribosomal RNA gene, region	337	337	86%	2,00E-88	79%	JQ669079.1	309,57	69,6%
Select seq emb|AJ420995.1|	Nepeta laxiflora 5.8S rRNA gene, internal transcribed spacer 1 (ITS1) and internal transcribed spacer 2 (ITS2)	344	344	89%	1,00E-90	79%	AJ420995.1	305,35	68,7%
Select seq gb|KC591689.1|	Pogogyne zizyphoroides voucher SDSU19277 internal transcribed spacer 1, partial sequence; 5.8S ribosomal RNA gene and internal transcribed spacer 2, complete sequence; and 28S ribosomal RNA gene, partial sequence	331	331	86%	9,00E-87	79%	KC591689.1	304,06	68,4%
Select seq gb|KC591685.1|	Pogogyne serpylloides voucher SBBG119413 internal transcribed spacer 1, partial sequence; 5.8S ribosomal RNA gene and internal transcribed spacer 2, complete sequence; and 28S ribosomal RNA gene, partial sequence	331	331	86%	9,00E-87	79%	KC591685.1	304,06	68,4%
Select seq gb|KC591675.1|	Pogogyne douglasii voucher SDSU19309 internal transcribed spacer 1, partial sequence; 5.8S ribosomal RNA gene and internal transcribed spacer 2, complete sequence; and 28S ribosomal RNA gene, partial sequence	331	331	86%	9,00E-87	79%	KC591675.1	304,06	68,4%
Select seq emb|AJ616328.1|	Nuxia oppositifolia ITS1 (partial), 5.8S rRNA gene and ITS2 (partial)	327	327	85%	1,00E-85	79%	AJ616328.1	303,92	68,4%
Select seq dbj|AB763377.1|	Lycopus lucidus genes for 18S rRNA, ITS1, 5.8S rRNA, ITS2, and 28S rRNA	342	342	89%	4,00E-90	79%	AB763377.1	303,57	68,3%

Select seq gb|KC591668.1|	Pogogyne clareana voucher SBBG111196 internal transcribed spacer 1, partial sequence; 5.8S ribosomal RNA gene and internal transcribed spacer 2, complete sequence; and 28S ribosomal RNA gene, partial sequence	329	329	86%	3,00E-86	79%	KC591668.1	302,22	68,0%
Select seq gb|KC591666.1|	Pogogyne abramsii voucher SDSU19268 internal transcribed spacer 1, partial sequence; 5.8S ribosomal RNA gene and internal transcribed spacer 2, complete sequence; and 28S ribosomal RNA gene, partial sequence	329	329	86%	3,00E-86	79%	KC591666.1	302,22	68,0%
Select seq gb|DQ667306.1|	Lepechinia lancifolia isolate x232 18S ribosomal RNA gene, partial sequence; internal transcribed spacer 1, 5.8S ribosomal RNA gene, and internal transcribed spacer 2, complete sequence; and 28S ribosomal RNA gene, partial sequence	366	366	96%	2,00E-97	79%	DQ667306.1	301,19	67,8%
Select seq gb|KC591684.1|	Pogogyne floribunda voucher RSA551272 internal transcribed spacer 1, partial sequence; 5.8S ribosomal RNA gene and internal transcribed spacer 2, complete sequence; and 28S ribosomal RNA gene, partial sequence	327	327	86%	1,00E-85	79%	KC591684.1	300,38	67,6%
Select seq gb|KC591680.1|	Pogogyne nudiuscula voucher SDSU19271 internal transcribed spacer 1, partial sequence; 5.8S ribosomal RNA gene and internal transcribed spacer 2, complete sequence; and 28S ribosomal RNA gene, partial sequence	327	327	86%	1,00E-85	79%	KC591680.1	300,38	67,6%
Select seq gb|KC591673.1|	Pogogyne douglasii voucher RSA695764 internal transcribed spacer 1, partial sequence; 5.8S ribosomal RNA gene, complete sequence; and internal transcribed spacer 2, partial sequence	327	327	86%	1,00E-85	79%	KC591673.1	300,38	67,6%
Select seq gb|KC591686.1|	Pogogyne serpylloides voucher SDSU19288 internal transcribed spacer 1, partial sequence; 5.8S ribosomal RNA gene and internal transcribed spacer 2, complete sequence; and 28S ribosomal RNA gene, partial sequence	326	326	86%	4,00E-85	79%	KC591686.1	299,47	67,4%
Select seq gb|KC591679.1|	Pogogyne nudiuscula voucher SDSU19274 internal transcribed spacer 1, partial sequence; 5.8S ribosomal RNA gene and internal transcribed spacer 2, complete sequence; and 28S ribosomal RNA gene, partial sequence	326	326	86%	4,00E-85	79%	KC591679.1	299,47	67,4%
Select seq gb|KC591678.1|	Pogogyne nudiuscula voucher SDSU19269 internal transcribed spacer 1, partial sequence; 5.8S ribosomal RNA gene and internal transcribed spacer 2, complete sequence; and 28S ribosomal RNA gene, partial sequence	326	326	86%	4,00E-85	79%	KC591678.1	299,47	67,4%
Select seq gb|KC591677.1|	Pogogyne floribunda voucher UC1609361 internal transcribed spacer 1, partial sequence; 5.8S ribosomal RNA gene and internal transcribed spacer 2, complete sequence; and 28S ribosomal RNA gene, partial sequence	326	326	86%	4,00E-85	79%	KC591677.1	299,47	67,4%
Select seq emb|LN590706.1|	Cyprinus carpio genome assembly common carp genome, scaffold: LG36, chromosome: 36	375	375	99%	4,00E-100	79%	LN590706.1	299,24	67,3%
Select seq emb|AJ420996.1|	Nepeta oxyodonta 5.8S rRNA gene, internal transcribed spacer 1 (ITS1) and internal transcribed spacer 2 (ITS2)	337	337	89%	2,00E-88	79%	AJ420996.1	299,13	67,3%
Select seq gb|KC591670.1|	Pogogyne douglasii voucher JEPS100839 internal transcribed spacer 1, partial sequence; 5.8S ribosomal RNA gene and internal transcribed spacer 2, complete sequence; and 28S ribosomal RNA gene, partial sequence	324	324	86%	1,00E-84	79%	KC591670.1	297,63	67,0%
Select seq gb|DQ667334.1|	Salvia miltiorrhiza isolate x556 18S ribosomal RNA gene, partial sequence; internal transcribed spacer 1, 5.8S ribosomal RNA gene, and internal transcribed spacer 2, complete sequence; and 28S ribosomal RNA gene, partial sequence	348	348	96%	9,00E-92	79%	DQ667334.1	286,38	64,4%
Select seq gb|DQ667250.1|	Salvia glutinosa isolate x094 18S ribosomal RNA gene, partial sequence; internal transcribed spacer 1, 5.8S ribosomal RNA gene, and internal transcribed spacer 2, complete sequence; and 28S ribosomal RNA gene, partial sequence	329	329	93%	3,00E-86	78%	DQ667250.1	275,94	62,1%
Select seq gb|DQ667324.1|	Clinopodium vulgare isolate x423 18S ribosomal RNA gene, partial sequence; internal transcribed spacer 1, 5.8S ribosomal RNA gene, and internal transcribed spacer 2, complete sequence; and 28S ribosomal RNA gene, partial sequence	335	335	96%	7,00E-88	78%	DQ667324.1	272,19	61,2%
Select seq gb|DQ667290.1|	Salvia rugosa isolate x190 18S ribosomal RNA gene, partial sequence; internal transcribed spacer 1, 5.8S ribosomal RNA gene, and internal transcribed spacer 2, complete sequence; and 28S ribosomal RNA gene, partial sequence	327	327	96%	1,00E-85	78%	DQ667290.1	265,69	59,8%

Select for downloading or viewing reports	Kh053_trnL Description	Max score	Total score	Query cover	E value	Ident	Accession	(Ident/Cover)* Max score	Deviation from top hit
Select seq gb|AY618513.1|	Mentha arvensis tRNA-Leu (trnL) gene and trnL-trnF intergenic spacer, partial sequence; chloroplast	1157	1157	98%	0.0	100%	AY618513.1	1180,61	100,0%
Select seq gb|AY618515.1|	Mentha canadensis tRNA-Leu (trnL) gene and trnL-trnF intergenic spacer, partial sequence; chloroplast	1151	1151	98%	0.0	99%	AY618515.1	1162,74	98,5%
Select seq gb|AY618512.1|	Mentha aquatica tRNA-Leu (trnL) gene and trnL-trnF intergenic spacer, partial sequence; chloroplast	1151	1151	98%	0.0	99%	AY618512.1	1162,74	98,5%
Select seq gb|JF301387.1|	Mentha arvensis voucher B. Drew 82 tRNA-Leu (trnL) gene and trnL-trnF intergenic spacer, partial sequence; chloroplast	1138	1138	97%	0.0	99%	JF301387.1	1161,46	98,4%
Select seq gb|FJ593456.1|	Mentha sp. JSZ-2009a tRNA-Leu (trnL) gene and trnL-trnF intergenic spacer, partial sequence; chloroplast	1171	1171	100%	0.0	99%	FJ593456.1	1159,29	98,2%
Select seq gb|DQ667514.1|	Mentha arvensis isolate x424 tRNA-Leu (trnL) gene and trnL-trnF intergenic spacer, partial sequence; chloroplast	1164	1164	100%	0.0	99%	DQ667514.1	1152,36	97,6%
Select seq gb|GU381522.1|	Mentha cervina voucher M:Braeuchler 2394 tRNA-Leu (trnL) gene and trnL-trnF intergenic spacer, partial sequence; chloroplast	1133	1133	99%	0.0	99%	GU381522.1	1133,00	96,0%
Select seq gb|AY618526.1|	Mentha suaveolens tRNA-Leu (trnL) gene and trnL-trnF intergenic spacer, partial sequence; chloroplast	1120	1120	98%	0.0	99%	AY618526.1	1131,43	95,8%
Select seq gb|JQ669053.1|	Mentha pulegium voucher Riina 1574 tRNA-Leu (trnL) gene and trnL-trnF intergenic spacer, partial sequence; plastid	1116	1116	98%	0.0	99%	JQ669053.1	1127,39	95,5%
Select seq gb|JQ669054.1|	Mentha spicata voucher J. Walker 2566 tRNA-Leu (trnL) gene and trnL-trnF intergenic spacer, partial sequence; plastid	1114	1114	98%	0.0	99%	JQ669054.1	1125,37	95,3%
Select seq gb|JQ669052.1|	Mentha spicata subsp. condensata voucher Riina 1575 tRNA-Leu (trnL) gene and trnL-trnF intergenic spacer, partial sequence; plastid	1114	1114	98%	0.0	99%	JQ669052.1	1125,37	95,3%
Select seq gb|AY618521.1|	Mentha longifolia tRNA-Leu (trnL) gene and trnL-trnF intergenic spacer, partial sequence; chloroplast	1114	1114	98%	0.0	99%	AY618521.1	1125,37	95,3%
Select seq gb|AY618524.1|	Mentha satureioides tRNA-Leu (trnL) gene and trnL-trnF intergenic spacer, partial sequence; chloroplast	1114	1114	98%	0.0	99%	AY618524.1	1125,37	95,3%
Select seq gb|AY618517.1|	Mentha cunninghamii tRNA-Leu (trnL) gene and trnL-trnF intergenic spacer, partial sequence; chloroplast	1114	1114	98%	0.0	99%	AY618517.1	1125,37	95,3%
Select seq gb|AY618516.1|	Mentha cervina tRNA-Leu (trnL) gene and trnL-trnF intergenic spacer, partial sequence; chloroplast	1114	1114	98%	0.0	99%	AY618516.1	1125,37	95,3%
Select seq gb|AY618514.1|	Mentha australis tRNA-Leu (trnL) gene and trnL-trnF intergenic spacer, partial sequence; chloroplast	1114	1114	98%	0.0	99%	AY618514.1	1125,37	95,3%
Select seq gb|AY570461.1|	Mentha spicata voucher JBW 2566 tRNA-Leu and trnL-trnF intergenic spacer, partial sequence; chloroplast	1122	1122	99%	0.0	99%	AY570461.1	1122,00	95,0%
Select seq emb|AJ505541.1|	Mentha suaveolens plastid trnL-trnF intergenic spacer, specimen voucher cult., K-1970-3169 (K)	1133	1133	100%	0.0	99%	AJ505541.1	1121,67	95,0%
Select seq gb|GU381521.1|	Mentha pulegium voucher M:Braeuchler 2300 tRNA-Leu (trnL) gene and trnL-trnF intergenic spacer, partial sequence; chloroplast	1099	1099	97%	0.0	99%	GU381521.1	1121,66	95,0%
Select seq gb|AY618522.1|	Mentha pulegium tRNA-Leu (trnL) gene and trnL-trnF intergenic spacer, partial sequence; chloroplast	1109	1109	98%	0.0	99%	AY618522.1	1120,32	94,9%
Select seq gb|AY618518.1|	Mentha diemenica tRNA-Leu (trnL) gene and trnL-trnF intergenic spacer, partial sequence; chloroplast	1109	1109	98%	0.0	99%	AY618518.1	1120,32	94,9%
Select seq gb|GU381519.1|	Mentha suaveolens voucher M:Braeuchler 2380 tRNA-Leu (trnL) gene and trnL-trnF intergenic spacer, partial sequence; chloroplast	1120	1120	99%	0.0	99%	GU381519.1	1120,00	94,9%
Select seq gb|GU381518.1|	Mentha spicata voucher M:Braeuchler 2377 tRNA-Leu (trnL) gene and trnL-trnF intergenic spacer, partial sequence; chloroplast	1120	1120	99%	0.0	99%	GU381518.1	1120,00	94,9%
Select seq gb|KC414276.1|	Mentha canadensis isolate 511190001 tRNA-Leu (trnL) gene and trnL-trnF intergenic spacer, partial sequence; chloroplast	1127	1127	100%	0.0	99%	KC414276.1	1115,73	94,5%
Select seq gb|KR150223.1|	Mentha sp. 1 Kh62 trnL-trnF intergenic spacer region, partial sequence; chloroplast	1122	1122	100%	0.0	99%	KR150223.1	1110,78	94,1%
Select seq gb|GU381495.1|	Killickia pilosa voucher M:Braeuchler 3810 tRNA-Leu (trnL) gene and trnL-trnF intergenic spacer, partial sequence; chloroplast	1068	1068	95%	0.0	98%	GU381495.1	1101,73	93,3%

Select seq gb|AY618523.1|	Mentha requienii tRNA-Leu (trnL) gene and trnL-trnF intergenic spacer, partial sequence; chloroplast	1079	1079	96% 0.0	98% AY618523.1	1101,48	93,3%
Select seq gb|GU381484.1|	Micromeria flagellaris voucher E:van der Werff & McPherson 13570 tRNA-Leu (trnL) gene and trnL-trnF intergenic spacer, partial sequence; chloroplast	1088	1088	97% 0.0	98% GU381484.1	1099,22	93,1%
Select seq gb|GU381483.1|	Micromeria flagellaris voucher E:Clement et al 2140 tRNA-Leu (trnL) gene and trnL-trnF intergenic spacer, partial sequence; chloroplast	1088	1088	97% 0.0	98% GU381483.1	1099,22	93,1%
Select seq gb|GU381496.1|	Killickia pilosa voucher M:Braeuchler 3832 tRNA-Leu (trnL) gene and trnL-trnF intergenic spacer, partial sequence; chloroplast	1083	1083	97% 0.0	98% GU381496.1	1094,16	92,7%
Select seq gb|GU381493.1|	Killickia grandiflora voucher M:Braeuchler 3811 tRNA-Leu (trnL) gene and trnL-trnF intergenic spacer, partial sequence; chloroplast	1083	1083	97% 0.0	98% GU381493.1	1094,16	92,7%
Select seq gb|GU381485.1|	Micromeria sphaerophylla voucher E:Lewis et al 1064 tRNA-Leu (trnL) gene and trnL-trnF intergenic spacer, partial sequence; chloroplast	1103	1103	99% 0.0	98% GU381485.1	1091,86	92,5%
Select seq gb|GU381481.1|	Micromeria cf. madagascariensis Morawetz 205 tRNA-Leu (trnL) gene and trnL-trnF intergenic spacer, partial sequence; chloroplast	1103	1103	99% 0.0	98% GU381481.1	1091,86	92,5%
Select seq gb|GU381629.1|	Thymbra capitata voucher M:Braeuchler 2518 tRNA-Leu (trnL) gene and trnL-trnF intergenic spacer, partial sequence; chloroplast	1077	1077	97% 0.0	98% GU381629.1	1088,10	92,2%
Select seq gb|JF301401.1|	Thymbra capitata voucher UCBG 96.0817 tRNA-Leu (trnL) gene and trnL-trnF intergenic spacer, partial sequence; chloroplast	1051	1051	95% 0.0	98% JF301401.1	1084,19	91,8%
Select seq gb|GU381517.1|	Clinopodium barosmum voucher BM<GBR-LONDON>:McLaren N193 tRNA-Leu (trnL) gene and trnL-trnF intergenic spacer, partial sequence; chloroplast	1072	1072	97% 0.0	98% GU381517.1	1083,05	91,7%
Select seq gb|GU381516.1|	Clinopodium wardii voucher BM<GBR-LONDON>:Ludlow et al. 14234 tRNA-Leu (trnL) gene and trnL-trnF intergenic spacer, partial sequence; chloroplast	1072	1072	97% 0.0	98% GU381516.1	1083,05	91,7%
Select seq gb|GU381515.1|	Clinopodium hydaspidis voucher BM<GBR-LONDON>:Mohd 133 tRNA-Leu (trnL) gene and trnL-trnF intergenic spacer, partial sequence; chloroplast	1072	1072	97% 0.0	98% GU381515.1	1083,05	91,7%
Select seq gb|GU381514.1|	Clinopodium nepalense voucher FR:Stainton 6024 tRNA-Leu (trnL) gene and trnL-trnF intergenic spacer, partial sequence; chloroplast	1072	1072	97% 0.0	98% GU381514.1	1083,05	91,7%
Select seq gb|GU381511.1|	Clinopodium piperitum voucher BM<GBR-LONDON>:Vickery 454 tRNA-Leu (trnL) gene and trnL-trnF intergenic spacer, partial sequence; chloroplast	1072	1072	97% 0.0	98% GU381511.1	1083,05	91,7%
Select seq gb|GU381489.1|	Killickia lutea voucher NU<ZAF>:Hilliard & Burtt 9876 tRNA-Leu (trnL) gene and trnL-trnF intergenic spacer, partial sequence; chloroplast	1072	1072	97% 0.0	98% GU381489.1	1083,05	91,7%
Select seq gb|GU381488.1|	Killickia compacta voucher M:Braeuchler 3816 tRNA-Leu (trnL) gene and trnL-trnF intergenic spacer, partial sequence; chloroplast	1072	1072	97% 0.0	98% GU381488.1	1083,05	91,7%
Select seq gb|AY840207.1|	Thymbra spicata tRNA-Leu (trnL) gene and trnL-trnF intergenic spacer, partial sequence; chloroplast	1072	1072	97% 0.0	98% AY840207.1	1083,05	91,7%
Select seq gb|GU381632.1|	Thymbra spicata voucher M:Braeuchler 4548 tRNA-Leu (trnL) gene and trnL-trnF intergenic spacer, partial sequence; chloroplast	1068	1068	97% 0.0	98% GU381632.1	1079,01	91,4%
Select seq gb|GU381627.1|	Thymbra sintenisii subsp. isaurica voucher E:Goener 12628 tRNA-Leu (trnL) gene and trnL-trnF intergenic spacer, partial sequence; chloroplast	1066	1066	97% 0.0	98% GU381627.1	1076,99	91,2%
Select seq gb|GU381512.1|	Clinopodium piperitum voucher E:Stainton 7320 tRNA-Leu (trnL) gene and trnL-trnF intergenic spacer, partial sequence; chloroplast	1066	1066	97% 0.0	98% GU381512.1	1076,99	91,2%
Select seq gb|GU381621.1|	Satureja cuneifolia voucher M:Rechinger 11142 tRNA-Leu (trnL) gene and trnL-trnF intergenic spacer, partial sequence; chloroplast	1068	1068	97% 0.0	97% GU381621.1	1068,00	90,5%
Select seq gb|GU381612.1|	Gontscharovia popovii voucher BM<GBR-LONDON>:Schmid 2419 tRNA-Leu (trnL) gene and trnL-trnF intergenic spacer, partial sequence; chloroplast	1068	1068	97% 0.0	97% GU381612.1	1068,00	90,5%
Select seq gb|AY840179.1|	Satureja montana tRNA-Leu (trnL) gene and trnL-trnF intergenic spacer, partial sequence; chloroplast	1068	1068	97% 0.0	97% AY840179.1	1068,00	90,5%
Select seq gb|KR063656.1|	Satureja pilosa subsp. origanita tRNA-Leu (trnL) gene, partial sequence; trnL-trnF intergenic spacer, complete sequence; and tRNA-Phe (trnF) gene, partial sequence; chloroplast	1075	1075	98% 0.0	97% KR063656.1	1064,03	90,1%
Select seq gb|AY618519.1|	Mentha gattefossei tRNA-Leu (trnL) gene and trnL-trnF intergenic spacer, partial sequence; chloroplast	1074	1074	98% 0.0	97% AY618519.1	1063,04	90,0%
Select seq gb|JQ669067.1|	Satureja montana voucher UCBG 2002.0593, Forbes s.n tRNA-Leu (trnL) gene and trnL-trnF intergenic spacer, partial sequence; plastid	1062	1062	97% 0.0	97% JQ669067.1	1062,00	90,0%
Select seq gb|GU381619.1|	Satureja mutica voucher M:Akhani 12362 tRNA-Leu (trnL) gene and trnL-trnF intergenic spacer, partial sequence; chloroplast	1062	1062	97% 0.0	97% GU381619.1	1062,00	90,0%
Select seq gb|GU381614.1|	Satureja thymbra voucher M:Braeuchler 2896 tRNA-Leu (trnL) gene and trnL-trnF intergenic spacer, partial sequence; chloroplast	1062	1062	97% 0.0	97% GU381614.1	1062,00	90,0%

Select seq gb|GU381611.1|	Gontscharovia popovii voucher M:Vvedensky s.n. tRNA-Leu (trnL) gene and trnL-trnF intergenic spacer, partial sequence; chloroplast	1062	1062	97% 0.0	97% GU381611.1	1062,00	90,0%
Select seq gb|GU381510.1|	Ziziphora pamiroalaica voucher C:Murray et al. 10090 tRNA-Leu (trnL) gene and trnL-trnF intergenic spacer, partial sequence; chloroplast	1051	1051	96% 0.0	97% GU381510.1	1061,95	89,9%
Select seq gb|GU381507.1|	Ziziphora tenuior voucher MSB:Fayvush et al. 03-1503 tRNA-Leu (trnL) gene and trnL-trnF intergenic spacer, partial sequence; chloroplast	1051	1051	96% 0.0	97% GU381507.1	1061,95	89,9%
Select seq gb|GU381505.1|	Clinopodium troodi voucher W:Davis 1856 tRNA-Leu (trnL) gene and trnL-trnF intergenic spacer, partial sequence; chloroplast	1051	1051	96% 0.0	97% GU381505.1	1061,95	89,9%
Select seq gb|GU381501.1|	Clinopodium graveolens subsp. rotundifolium voucher M:Podlech 47181 tRNA-Leu (trnL) gene and trnL-trnF intergenic spacer, partial sequence; chloroplast	1051	1051	96% 0.0	97% GU381501.1	1061,95	89,9%
Select seq gb|GU381499.1|	Clinopodium suaveolens voucher M:Erben s.n. tRNA-Leu (trnL) gene and trnL-trnF intergenic spacer, partial sequence; chloroplast	1051	1051	96% 0.0	97% GU381499.1	1061,95	89,9%
Select seq gb|GU381635.1|	Thymus broussonetii subsp. hannonis voucher MSB:Podlech 142 tRNA-Leu (trnL) gene and trnL-trnF intergenic spacer, partial sequence; chloroplast	1061	1061	97% 0.0	97% GU381635.1	1061,00	89,9%
Select seq gb|KR150214.1|	Satureja sp. 1 Kh04 trnL-trnF intergenic spacer region, partial sequence; chloroplast	1070	1070	98% 0.0	97% KR150214.1	1059,08	89,7%
Select seq gb|KR150209.1|	Satureja sp. 3 Kh90 trnL-trnF intergenic spacer region, partial sequence; chloroplast	1070	1070	98% 0.0	97% KR150209.1	1059,08	89,7%
Select seq gb|KR150243.1|	Ziziphora sp. 2 Kh20 trnL-trnF intergenic spacer region, partial sequence; chloroplast	1059	1059	97% 0.0	97% KR150243.1	1059,00	89,7%
Select seq gb|KR150198.1|	Ziziphora sp. 3 Kh112 trnL-trnF intergenic spacer region, partial sequence; chloroplast	1059	1059	97% 0.0	97% KR150198.1	1059,00	89,7%
Select seq gb|GU381615.1|	Satureja innota voucher M:Barra et al. 2484GL tRNA-Leu (trnL) gene and trnL-trnF intergenic spacer, partial sequence; chloroplast	1057	1057	97% 0.0	97% GU381615.1	1057,00	89,5%
Select seq gb|GU381497.1|	Clinopodium acinos voucher M:Podlech 50287 tRNA-Leu (trnL) gene and trnL-trnF intergenic spacer, partial sequence; chloroplast	1046	1046	96% 0.0	97% GU381497.1	1056,90	89,5%
Select seq gb|GU381637.1|	Thymus serpyllum voucher M:Braeuchler 2514 tRNA-Leu (trnL) gene and trnL-trnF intergenic spacer, partial sequence; chloroplast	1055	1055	97% 0.0	97% GU381637.1	1055,00	89,4%
Select seq gb|GU381633.1|	Thymus caespititius voucher M:Heubl s.n. tRNA-Leu (trnL) gene and trnL-trnF intergenic spacer, partial sequence; chloroplast	1055	1055	97% 0.0	97% GU381633.1	1055,00	89,4%
Select seq gb|KR150238.1|	Ziziphora sp. 1 Kh75 trnL-trnF intergenic spacer region, partial sequence; chloroplast	1053	1053	97% 0.0	97% KR150238.1	1053,00	89,2%
Select seq gb|DQ667501.1|	Ziziphora taurica isolate x262 tRNA-Leu (trnL) gene and trnL-trnF intergenic spacer, partial sequence; chloroplast	1053	1053	97% 0.0	97% DQ667501.1	1053,00	89,2%
Select seq gb|KR063657.1|	Thymus sibthorpii tRNA-Leu (trnL) gene, partial sequence; trnL-trnF intergenic spacer, complete sequence; and tRNA-Phe (trnF) gene, partial sequence; chloroplast	1062	1062	98% 0.0	97% KR063657.1	1051,16	89,0%
Select seq gb|KR150218.1|	Thymus sp. 2 Kh69 trnL-trnF intergenic spacer region, partial sequence; chloroplast	1062	1062	98% 0.0	97% KR150218.1	1051,16	89,0%
Select seq gb|JQ669068.1|	Satureja thymbra voucher UCBG 2002.0540, Forbes s.n. tRNA-Leu (trnL) gene and trnL-trnF intergenic spacer, partial sequence; plastid	1051	1051	97% 0.0	97% JQ669068.1	1051,00	89,0%
Select seq gb|GU381623.1|	Satureja spicigera voucher M:Kartli et al. 2726 tRNA-Leu (trnL) gene and trnL-trnF intergenic spacer, partial sequence; chloroplast	1051	1051	97% 0.0	97% GU381623.1	1051,00	89,0%
Select seq gb|JQ669069.1|	Thymus pulegioides voucher Riina 1577 tRNA-Leu (trnL) gene and trnL-trnF intergenic spacer, partial sequence; plastid	1050	1050	97% 0.0	97% JQ669069.1	1050,00	88,9%
Select seq gb|GU381640.1|	Argantoniella salzmannii voucher M:Barra et al. 2673GL tRNA-Leu (trnL) gene and trnL-trnF intergenic spacer, partial sequence; chloroplast	1050	1050	97% 0.0	97% GU381640.1	1050,00	88,9%
Select seq gb|GU381636.1|	Thymus vulgaris voucher M:Braeuchler 3683 tRNA-Leu (trnL) gene and trnL-trnF intergenic spacer, partial sequence; chloroplast	1050	1050	97% 0.0	97% GU381636.1	1050,00	88,9%
Select seq gb|GU381620.1|	Satureja hortensis voucher M:Braeuchler 2422 tRNA-Leu (trnL) gene and trnL-trnF intergenic spacer, partial sequence; chloroplast	1050	1050	97% 0.0	97% GU381620.1	1050,00	88,9%
Select seq gb|AY840202.1|	Origanum vulgare tRNA-Leu (trnL) gene and trnL-trnF intergenic spacer, partial sequence; chloroplast	1050	1050	97% 0.0	97% AY840202.1	1050,00	88,9%
Select seq gb|KR150229.1|	Thymus sp. 5 Kh16 trnL-trnF intergenic spacer region, partial sequence; chloroplast	1057	1057	98% 0.0	97% KR150229.1	1046,21	88,6%
Select seq gb|KR150225.1|	Thymus sp. 4 Kh24 trnL-trnF intergenic spacer region, partial sequence; chloroplast	1057	1057	98% 0.0	97% KR150225.1	1046,21	88,6%

Select seq gb|KR150222.1|	Thymus sp. 3 Kh65 trnL-trnF intergenic spacer region, partial sequence; chloroplast	1057	1057	98% 0.0	97% KR150222.1	1046,21	88,6%
Select seq gb|KR150215.1|	Thymus sp. 1 Kh08 trnL-trnF intergenic spacer region, partial sequence; chloroplast	1057	1057	98% 0.0	97% KR150215.1	1046,21	88,6%
Select seq gb|JX880022.1|	Origanum vulgare subsp. vulgare chloroplast, complete genome	1057	1057	98% 0.0	97% JX880022.1	1046,21	88,6%
Select seq gb|JQ690290.1|	Origanum elongatum isolate H5_O_elo trnL-trnF intergenic spacer, partial sequence; chloroplast	1057	1057	98% 0.0	97% JQ690290.1	1046,21	88,6%
Select seq gb|JQ690289.1|	Origanum rotundifolium isolate DNA3_O_rot trnL-trnF intergenic spacer, partial sequence; chloroplast	1057	1057	98% 0.0	97% JQ690289.1	1046,21	88,6%
Select seq gb|EU556532.1|	Thymus amurensis isolate HX tRNA-Leu (trnL) gene and trnL-trnF intergenic spacer, partial sequence; chloroplast	1057	1057	98% 0.0	97% EU556532.1	1046,21	88,6%
Select seq gb|EU556525.1|	Thymus quinquecostatus isolate CL tRNA-Leu (trnL) gene and trnL-trnF intergenic spacer, partial sequence; chloroplast	1057	1057	98% 0.0	97% EU556525.1	1046,21	88,6%
Select seq emb|AJ505543.1|	Origanum vulgare plastid trnL-trnF intergenic spacer, specimen voucher cult., K-000-69-19317, chase 13334 (K)	1057	1057	98% 0.0	97% AJ505543.1	1046,21	88,6%
Select seq gb|GU381622.1|	Satureja intermedia voucher M:Rechinger 43441 tRNA-Leu (trnL) gene and trnL-trnF intergenic spacer, partial sequence; chloroplast	1046	1046	97% 0.0	97% GU381622.1	1046,00	88,6%
Select seq emb|AJ505544.1|	Thymus serpyllum var. citriodorum plastid trnL-trnF intergenic spacer, specimen voucher cult., K-1975-1177, Chase 13331 (K)	1051	1051	98% 0.0	97% AJ505544.1	1040,28	88,1%
Select seq gb|EU556539.1|	Thymus quinquecostatus isolate ZY tRNA-Leu (trnL) gene and trnL-trnF intergenic spacer, partial sequence; chloroplast	1050	1050	98% 0.0	97% EU556539.1	1039,29	88,0%
Select seq gb|EU556538.1|	Thymus quinquecostatus isolate ZJ tRNA-Leu (trnL) gene and trnL-trnF intergenic spacer, partial sequence; chloroplast	1050	1050	98% 0.0	97% EU556538.1	1039,29	88,0%
Select seq gb|EU556533.1|	Thymus quinquecostatus isolate HY tRNA-Leu (trnL) gene and trnL-trnF intergenic spacer, partial sequence; chloroplast	1050	1050	98% 0.0	97% EU556533.1	1039,29	88,0%
Select seq gb|EU556527.1|	Thymus mongolicus isolate G2 tRNA-Leu (trnL) gene and trnL-trnF intergenic spacer, partial sequence; chloroplast	1050	1050	98% 0.0	97% EU556527.1	1039,29	88,0%
Select seq gb|EU556526.1|	Thymus quinquecostatus isolate D2 tRNA-Leu (trnL) gene and trnL-trnF intergenic spacer, partial sequence; chloroplast	1050	1050	98% 0.0	97% EU556526.1	1039,29	88,0%
Select seq gb|KR150240.1|	Satureja sp. 2 Kh70 trnL-trnF intergenic spacer region, partial sequence; chloroplast	1048	1048	98% 0.0	97% KR150240.1	1037,31	87,9%
Select seq gb|JQ690293.1|	Origanum dayi isolate H43_O_day trnL-trnF intergenic spacer, partial sequence; chloroplast	1046	1046	98% 0.0	97% JQ690293.1	1035,33	87,7%

Select for downloading or viewing reports	Kh055_trnL Description	Max score	Total score	Query cover	E value	Ident	Accession	(Ident/Cover)* Max score	Deviation from top hit
Select seq emb|AJ505431.1|	Nepeta menthoides plastid trnL-trnF intergenic spacer, specimen voucher Jamzad s.n. (K)	1086	1086	100%	0.0	97%	AJ505431.1	1053,42	100,0%
Select seq gb|DQ667487.1|	Nepeta cataria isolate x220 tRNA-Leu (trnL) gene and trnL-trnF intergenic spacer, partial sequence; chloroplast	1027	1027	100%	0.0	95%	DQ667487.1	975,65	92,6%
Select seq emb|AJ505432.1|	Nepeta racemosa plastid trnL-trnF intergenic spacer, specimen voucher Jamzad s.n. (TARI)	1011	1011	99%	0.0	95%	AJ505432.1	970,15	92,1%
Select seq gb|JF301391.1|	Nepeta cataria voucher B. Drew 72 tRNA-Leu (trnL) gene and trnL-trnF intergenic spacer, partial sequence; chloroplast	979	979	96%	0.0	95%	JF301391.1	968,80	92,0%
Select seq gb|JQ669045.1|	Hymenocrater bituminosus voucher K. Tamanyan & George Fayvush tRNA-Leu (trnL) gene and trnL-trnF intergenic spacer, partial sequence; plastid	987	987	97%	0.0	95%	JQ669045.1	966,65	91,8%
Select seq gb|KR150208.1|	Hymenocrater sp. 3 Kh82 trnL-trnF intergenic spacer region, partial sequence; chloroplast	1005	1005	99%	0.0	95%	KR150208.1	964,39	91,5%
Select seq gb|KR150211.1|	Hymenocrater sp. Kh10 trnL-trnF intergenic spacer region, partial sequence; chloroplast	994	994	98%	0.0	95%	KR150211.1	963,57	91,5%
Select seq gb|KR150216.1|	Nepeta sp. 1 Kh03 trnL-trnF intergenic spacer region, partial sequence; chloroplast	870	870	86%	0.0	94%	KR150216.1	950,93	90,3%
Select seq gb|GU381479.1|	Nepeta supina voucher M:Groeger & Schewardnadse 1466 tRNA-Leu (trnL) gene and trnL-trnF intergenic spacer, partial sequence; chloroplast	983	983	98%	0.0	94%	GU381479.1	942,88	89,5%
Select seq gb|KR150220.1|	Hymenocrater sp. 1 Kh67 trnL-trnF intergenic spacer region, partial sequence; chloroplast	996	996	100%	0.0	94%	KR150220.1	936,24	88,9%
Select seq gb|JQ669049.1|	Marmoritis complanatum voucher D.E. Boufford et al., 32012 tRNA-Leu (trnL) gene and trnL-trnF intergenic spacer, partial sequence; plastid	939	939	95%	0.0	94%	JQ669049.1	929,12	88,2%
Select seq emb|AJ505433.1|	Nepeta straussii plastid trnL-trnF intergenic spacer, specimen voucher Jamzad etal 76846 (TARI)	977	977	99%	0.0	94%	AJ505433.1	927,66	88,1%
Select seq gb|KM886627.1|	Marmoritis complanata voucher T. Deng 2359 (KUN) tRNA-Leu (trnL) gene, partial sequence; trnL-trnF intergenic spacer, complete sequence; and tRNA-Phe (trnF) gene, partial sequence; plastid	929	929	95%	0.0	94%	KM886627.1	919,22	87,3%
Select seq gb|KF307426.1|	Lepechinia paniculata voucher B. Drew 241 tRNA-Leu (trnL) gene and trnL-trnF intergenic spacer, partial sequence; chloroplast	833	833	86%	0.0	93%	KF307426.1	900,80	85,5%
Select seq gb|AY506621.1|	Nepeta grandiflora tRNA-Leu (trnL) gene and trnL-trnF intergenic spacer, partial sequence; chloroplast	920	920	95%	0.0	93%	AY506621.1	900,63	85,5%
Select seq gb|KR150192.1|	Hymenocrater sp. 2 Kh51 trnL-trnF intergenic spacer region, partial sequence; chloroplast	870	870	91%	0.0	93%	KR150192.1	889,12	84,4%
Select seq gb|FJ593457.1|	Nepeta stewartiana tRNA-Leu (trnL) gene and trnL-trnF intergenic spacer, partial sequence; chloroplast	946	946	100%	0.0	93%	FJ593457.1	879,78	83,5%
Select seq gb|JF301377.1|	Lepechinia flammea tRNA-Leu (trnL) gene and trnL-trnF intergenic spacer, partial sequence; chloroplast	869	869	92%	0.0	93%	JF301377.1	878,45	83,4%
Select seq gb|KM886649.1|	Prunella vulgaris voucher SNJ Exped. 20110719005 (KUN) tRNA-Leu (trnL) gene, partial sequence; trnL-trnF intergenic spacer, complete sequence; and tRNA-Phe (trnF) gene, partial sequence; plastid	889	889	95%	0.0	92%	KM886649.1	860,93	81,7%
Select seq gb|AY506619.1|	Prunella vulgaris tRNA-Leu (trnL) gene and trnL-trnF intergenic spacer, partial sequence; chloroplast	889	889	95%	0.0	92%	AY506619.1	860,93	81,7%
Select seq gb|EF153681.1|	Prunella asiatica tRNA-Leu (trnL) and trnL-trnF intergenic spacer, partial sequence; chloroplast	893	893	96%	0.0	92%	EF153681.1	855,79	81,2%
Select seq gb|EF153679.1|	Prunella grandiflora tRNA-Leu (trnL) and trnL-trnF intergenic spacer, partial sequence; chloroplast	917	917	99%	0.0	92%	EF153679.1	852,16	80,9%
Select seq gb|DQ667508.1|	Prunella vulgaris isolate x314 tRNA-Leu (trnL) gene and trnL-trnF intergenic spacer, partial sequence; chloroplast	924	924	100%	0.0	92%	DQ667508.1	850,08	80,7%
Select seq gb|KR063656.1|	Satureja pilosa subsp. origanita tRNA-Leu (trnL) gene, partial sequence; trnL-trnF intergenic spacer, complete sequence; and tRNA-Phe (trnF) gene, partial sequence; chloroplast	885	885	96%	0.0	92%	KR063656.1	848,13	80,5%
Select seq gb|DQ667492.1|	Lepechinia lancifolia isolate x232 tRNA-Leu (trnL) gene and trnL-trnF intergenic spacer, partial sequence; chloroplast	918	918	100%	0.0	92%	DQ667492.1	844,56	80,2%
Select seq gb|AY840207.1|	Thymbra spicata tRNA-Leu (trnL) gene and trnL-trnF intergenic spacer, partial sequence; chloroplast	872	872	95%	0.0	92%	AY840207.1	844,46	80,2%
Select seq gb|KR150214.1|	Satureja sp. 1 Kh04 trnL-trnF intergenic spacer region, partial sequence; chloroplast	880	880	96%	0.0	92%	KR150214.1	843,33	80,1%

Select seq gb|KF307433.1|	Lepechinia urbanii voucher B. Drew 135 tRNA-Leu (trnL) gene and trnL-trnF intergenic spacer, partial sequence; chloroplast	870	870	95% 0.0	92% KF307433.1	842,53	80,0%
Select seq gb|GU381484.1|	Micromeria flagellaris voucher E:van der Werff & McPherson 13570 tRNA-Leu (trnL) gene and trnL-trnF intergenic spacer, partial sequence; chloroplast	870	870	95% 0.0	92% GU381484.1	842,53	80,0%
Select seq gb|GU381483.1|	Micromeria flagellaris voucher E:Clement et al 2140 tRNA-Leu (trnL) gene and trnL-trnF intergenic spacer, partial sequence; chloroplast	870	870	95% 0.0	92% GU381483.1	842,53	80,0%
Select seq gb|GU381481.1|	Micromeria cf. madagascariensis Morawetz 205 tRNA-Leu (trnL) gene and trnL-trnF intergenic spacer, partial sequence; chloroplast	870	870	95% 0.0	92% GU381481.1	842,53	80,0%
Select seq gb|JF301383.1|	Lepechinia salviifolia voucher R. Jabaily s.n. tRNA-Leu (trnL) gene and trnL-trnF intergenic spacer, partial sequence; chloroplast	869	869	95% 0.0	92% JF301383.1	841,56	79,9%
Select seq gb|KR150209.1|	Satureja sp. 3 Kh90 trnL-trnF intergenic spacer region, partial sequence; chloroplast	867	867	95% 0.0	92% KR150209.1	839,62	79,7%
Select seq gb|GU381632.1|	Thymbra spicata voucher M:Braeuchler 4548 tRNA-Leu (trnL) gene and trnL-trnF intergenic spacer, partial sequence; chloroplast	867	867	95% 0.0	92% GU381632.1	839,62	79,7%
Select seq gb|JQ669067.1|	Satureja montana voucher UCBG 2002.0593, Forbes s.n tRNA-Leu (trnL) gene and trnL-trnF intergenic spacer, partial sequence; plastid	857	857	94% 0.0	92% JQ669067.1	838,77	79,6%
Select seq gb|GU381629.1|	Thymbra capitata voucher M:Braeuchler 2518 tRNA-Leu (trnL) gene and trnL-trnF intergenic spacer, partial sequence; chloroplast	865	865	95% 0.0	92% GU381629.1	837,68	79,5%
Select seq gb|GU381485.1|	Micromeria sphaerophylla voucher E:Lewis et al 1064 tRNA-Leu (trnL) gene and trnL-trnF intergenic spacer, partial sequence; chloroplast	865	865	95% 0.0	92% GU381485.1	837,68	79,5%
Select seq gb|GU381621.1|	Satureja cuneifolia voucher M:Rechinger 11142 tRNA-Leu (trnL) gene and trnL-trnF intergenic spacer, partial sequence; chloroplast	863	863	95% 0.0	92% GU381621.1	835,75	79,3%
Select seq gb|GU381612.1|	Gontscharovia popovii voucher BM<GBR-LONDON>:Schmid 2419 tRNA-Leu (trnL) gene and trnL-trnF intergenic spacer, partial sequence; chloroplast	863	863	95% 0.0	92% GU381612.1	835,75	79,3%
Select seq gb|GU381611.1|	Gontscharovia popovii voucher M:Vvedensky s.n. tRNA-Leu (trnL) gene and trnL-trnF intergenic spacer, partial sequence; chloroplast	863	863	95% 0.0	92% GU381611.1	835,75	79,3%
Select seq gb|AY840179.1|	Satureja montana tRNA-Leu (trnL) gene and trnL-trnF intergenic spacer, partial sequence; chloroplast	863	863	95% 0.0	92% AY840179.1	835,75	79,3%
Select seq gb|JF301382.1|	Lepechinia radula voucher B. Drew 185 tRNA-Leu (trnL) gene and trnL-trnF intergenic spacer, partial sequence; chloroplast	870	870	96% 0.0	92% JF301382.1	833,75	79,1%
Select seq gb|DQ667495.1|	Cleonia lusitanica isolate x255 tRNA-Leu (trnL) gene and trnL-trnF intergenic spacer, partial sequence; chloroplast	869	869	96% 0.0	92% DQ667495.1	832,79	79,1%
Select seq gb|DQ667517.1|	Drepanocaryum sewerzowii isolate x523 tRNA-Leu (trnL) gene and trnL-trnF intergenic spacer, partial sequence; chloroplast	896	896	99% 0.0	92% DQ667517.1	832,65	79,0%
Select seq gb|DQ667477.1|	Melissa officinalis isolate x193 tRNA-Leu (trnL) gene and trnL-trnF intergenic spacer, partial sequence; chloroplast	896	896	99% 0.0	92% DQ667477.1	832,65	79,0%
Select seq gb|KF307430.1|	Lepechinia schiedeana voucher B. Drew 157 tRNA-Leu (trnL) gene and trnL-trnF intergenic spacer, partial sequence; chloroplast	859	859	95% 0.0	92% KF307430.1	831,87	79,0%
Select seq gb|KF307411.1|	Lepechinia bella voucher Rachel Jabaily s.n. tRNA-Leu (trnL) gene and trnL-trnF intergenic spacer, partial sequence; chloroplast	859	859	95% 0.0	92% KF307411.1	831,87	79,0%
Select seq gb|GU381627.1|	Thymbra sintenisii subsp. isaurica voucher E:Goener 12628 tRNA-Leu (trnL) gene and trnL-trnF intergenic spacer, partial sequence; chloroplast	859	859	95% 0.0	92% GU381627.1	831,87	79,0%
Select seq emb|AJ505529.1|	Melissa officinalis plastid trnL-trnF intergenic spacer, specimen voucher Catino	885	885	98% 0.0	92% AJ505529.1	830,82	78,9%
Select seq gb|GU381623.1|	Satureja spicigera voucher M:Kartli et al. 2726 tRNA-Leu (trnL) gene and trnL-trnF intergenic spacer, partial sequence; chloroplast	857	857	95% 0.0	92% GU381623.1	829,94	78,8%
Select seq gb|GU381619.1|	Satureja mutica voucher M:Akhani 12362 tRNA-Leu (trnL) gene and trnL-trnF intergenic spacer, partial sequence; chloroplast	857	857	95% 0.0	92% GU381619.1	829,94	78,8%
Select seq gb|GU381614.1|	Satureja thymbra voucher M:Braeuchler 2896 tRNA-Leu (trnL) gene and trnL-trnF intergenic spacer, partial sequence; chloroplast	857	857	95% 0.0	92% GU381614.1	829,94	78,8%
Select seq gb|JF301380.1|	Lepechinia mexicana voucher B. Drew 127 tRNA-Leu (trnL) gene and trnL-trnF intergenic spacer, partial sequence; chloroplast	865	865	96% 0.0	92% JF301380.1	828,96	78,7%
Select seq gb|JF301361.1|	Chaunostoma mecistandrum voucher J.A. Monterrosa & R.A. Carballo 213 tRNA-Leu (trnL) gene and trnL-trnF intergenic spacer, partial sequence; chloroplast	865	865	96% 0.0	92% JF301361.1	828,96	78,7%
Select seq gb|KF307435.1|	Lepechinia yecorana voucher Henrickson 24691 tRNA-Leu (trnL) gene and trnL-trnF intergenic spacer, partial sequence; chloroplast	854	854	95% 0.0	92% KF307435.1	827,03	78,5%

Select seq gb|KF307432.1|	Lepechinia speciosa voucher Cordeno 3060 tRNA-Leu (trnL) gene and trnL-trnF intergenic spacer, partial sequence; chloroplast	854	854	95%	0.0	92%	KF307432.1	827,03	78,5%
Select seq gb|KF307422.1|	Lepechinia mexicana voucher B. Drew 130 tRNA-Leu (trnL) gene and trnL-trnF intergenic spacer, partial sequence; chloroplast	854	854	95%	0.0	92%	KF307422.1	827,03	78,5%
Select seq gb|KF307421.1|	Lepechinia heteromorpha voucher B. Drew 192 tRNA-Leu (trnL) gene and trnL-trnF intergenic spacer, partial sequence; chloroplast	854	854	95%	0.0	92%	KF307421.1	827,03	78,5%
Select seq gb|KF307420.1|	Lepechinia graveolens voucher Fuentes 10351 tRNA-Leu (trnL) gene and trnL-trnF intergenic spacer, partial sequence; chloroplast	854	854	95%	0.0	92%	KF307420.1	827,03	78,5%
Select seq gb|KF307418.1|	Lepechinia floribunda voucher B. Drew 172 tRNA-Leu (trnL) gene and trnL-trnF intergenic spacer, partial sequence; chloroplast	854	854	95%	0.0	92%	KF307418.1	827,03	78,5%
Select seq gb|GU381615.1|	Satureja innota voucher M:Barra et al. 2484GL tRNA-Leu (trnL) gene and trnL-trnF intergenic spacer, partial sequence; chloroplast	852	852	95%	0.0	92%	GU381615.1	825,09	78,3%
Select seq gb|JF301386.1|	Melissa officinalis voucher B. Drew 70 tRNA-Leu (trnL) gene and trnL-trnF intergenic spacer, partial sequence; chloroplast	857	857	96%	0.0	92%	JF301386.1	821,29	78,0%
Select seq gb|JQ669068.1|	Satureja thymbra voucher UCBG 2002.0540, Forbes s.n. tRNA-Leu (trnL) gene and trnL-trnF intergenic spacer, partial sequence; plastid	846	846	94%	0.0	91%	JQ669068.1	819,00	77,7%
Select seq gb|JF301381.1|	Lepechinia mexicana voucher B. Drew 164 tRNA-Leu (trnL) gene and trnL-trnF intergenic spacer, partial sequence; chloroplast	852	852	95%	0.0	91%	JF301381.1	816,13	77,5%
Select seq gb|KR150243.1|	Ziziphora sp. 2 Kh20 trnL-trnF intergenic spacer region, partial sequence; chloroplast	859	859	96%	0.0	91%	KR150243.1	814,26	77,3%
Select seq gb|KR150229.1|	Thymus sp. 5 Kh16 trnL-trnF intergenic spacer region, partial sequence; chloroplast	859	859	96%	0.0	91%	KR150229.1	814,26	77,3%
Select seq gb|KR150215.1|	Thymus sp. 1 Kh08 trnL-trnF intergenic spacer region, partial sequence; chloroplast	859	859	96%	0.0	91%	KR150215.1	814,26	77,3%
Select seq gb|KR150198.1|	Ziziphora sp. 3 Kh112 trnL-trnF intergenic spacer region, partial sequence; chloroplast	857	857	96%	0.0	91%	KR150198.1	812,36	77,1%
Select seq gb|KR150222.1|	Thymus sp. 3 Kh65 trnL-trnF intergenic spacer region, partial sequence; chloroplast	856	856	96%	0.0	91%	KR150222.1	811,42	77,0%
Select seq gb|KF307416.1|	Lepechinia codon voucher B. Drew 177 tRNA-Leu (trnL) gene and trnL-trnF intergenic spacer, partial sequence; chloroplast	846	846	95%	0.0	91%	KF307416.1	810,38	76,9%
Select seq gb|GU381622.1|	Satureja intermedia voucher M:Rechinger 43441 tRNA-Leu (trnL) gene and trnL-trnF intergenic spacer, partial sequence; chloroplast	846	846	95%	0.0	91%	GU381622.1	810,38	76,9%
Select seq gb|KR063657.1|	Thymus sibthorpii tRNA-Leu (trnL) gene, partial sequence; trnL-trnF intergenic spacer, complete sequence; and tRNA-Phe (trnF) gene, partial sequence; chloroplast	854	854	96%	0.0	91%	KR063657.1	809,52	76,8%
Select seq gb|KR150238.1|	Ziziphora sp. 1 Kh75 trnL-trnF intergenic spacer region, partial sequence; chloroplast	854	854	96%	0.0	91%	KR150238.1	809,52	76,8%
Select seq gb|KR150225.1|	Thymus sp. 4 Kh24 trnL-trnF intergenic spacer region, partial sequence; chloroplast	854	854	96%	0.0	91%	KR150225.1	809,52	76,8%
Select seq gb|KR150218.1|	Thymus sp. 2 Kh69 trnL-trnF intergenic spacer region, partial sequence; chloroplast	854	854	96%	0.0	91%	KR150218.1	809,52	76,8%
Select seq gb|JX880022.1|	Origanum vulgare subsp. vulgare chloroplast, complete genome	854	854	96%	0.0	91%	JX880022.1	809,52	76,8%
Select seq gb|JQ690290.1|	Origanum elongatum isolate H5_O_elo trnL-trnF intergenic spacer, partial sequence; chloroplast	854	854	96%	0.0	91%	JQ690290.1	809,52	76,8%
Select seq gb|JQ690289.1|	Origanum rotundifolium isolate DNA3_O_rot trnL-trnF intergenic spacer, partial sequence; chloroplast	854	854	96%	0.0	91%	JQ690289.1	809,52	76,8%
Select seq emb|AJ505543.1|	Origanum vulgare plastid trnL-trnF intergenic spacer, specimen voucher cult., K-000-69-19317, chase 13334 (K)	854	854	96%	0.0	91%	AJ505543.1	809,52	76,8%
Select seq gb|GU381517.1|	Clinopodium barosmum voucher BM<GBR-LONDON>:McLaren N193 tRNA-Leu (trnL) gene and trnL-trnF intergenic spacer, partial sequence; chloroplast	843	843	95%	0.0	91%	GU381517.1	807,51	76,7%
Select seq gb|GU381516.1|	Clinopodium wardii voucher BM<GBR-LONDON>:Ludlow et al. 14234 tRNA-Leu (trnL) gene and trnL-trnF intergenic spacer, partial sequence; chloroplast	843	843	95%	0.0	91%	GU381516.1	807,51	76,7%
Select seq gb|DQ667494.1|	Lepechinia calycina isolate x246 tRNA-Leu (trnL) gene and trnL-trnF intergenic spacer, partial sequence; chloroplast	878	878	99%	0.0	91%	DQ667494.1	807,05	76,6%
Select seq gb|AY570459.1|	Lepechinia chamaedryoides voucher JBW 2537 tRNA-Leu and trnL-trnF intergenic spacer, partial sequence; chloroplast	885	885	100%	0.0	91%	AY570459.1	805,35	76,5%

Select seq gb|KR150240.1|	Satureja sp. 2 Kh70 trnL-trnF intergenic spacer region, partial sequence; chloroplast	848	848	96% 0.0	91%	KR150240.1	803,83	76,3%
Select seq gb|EU556532.1|	Thymus amurensis isolate HX tRNA-Leu (trnL) gene and trnL-trnF intergenic spacer, partial sequence; chloroplast	848	848	96% 0.0	91%	EU556532.1	803,83	76,3%
Select seq gb|EU556525.1|	Thymus quinquecostatus isolate CL tRNA-Leu (trnL) gene and trnL-trnF intergenic spacer, partial sequence; chloroplast	848	848	96% 0.0	91%	EU556525.1	803,83	76,3%
Select seq gb|GU381495.1|	Killickia pilosa voucher M:Braeuchler 3810 tRNA-Leu (trnL) gene and trnL-trnF intergenic spacer, partial sequence; chloroplast	846	846	96% 0.0	91%	GU381495.1	801,94	76,1%
Select seq gb|AY570458.1|	Lepechinia calycina voucher JBW 1344 tRNA-Leu and trnL-trnF intergenic spacer, partial sequence; chloroplast	872	872	99% 0.0	91%	AY570458.1	801,54	76,1%
Select seq gb|AY570460.1|	Lepechinia fragrans voucher JBW 1333 tRNA-Leu and trnL-trnF intergenic spacer, partial sequence; chloroplast	863	863	98% 0.0	91%	AY570460.1	801,36	76,1%
Select seq gb|KC414276.1|	Mentha canadensis isolate 511190001 tRNA-Leu (trnL) gene and trnL-trnF intergenic spacer, partial sequence; chloroplast	845	845	96% 0.0	91%	KC414276.1	800,99	76,0%
Select seq gb|EF153682.1|	Prunella hispida tRNA-Leu (trnL) and trnL-trnF intergenic spacer, partial sequence; chloroplast	870	870	99% 0.0	91%	EF153682.1	799,70	75,9%
Select seq gb|GU381496.1|	Killickia pilosa voucher M:Braeuchler 3832 tRNA-Leu (trnL) gene and trnL-trnF intergenic spacer, partial sequence; chloroplast	861	861	98% 0.0	91%	GU381496.1	799,50	75,9%
Select seq gb|GU381493.1|	Killickia grandiflora voucher M:Braeuchler 3811 tRNA-Leu (trnL) gene and trnL-trnF intergenic spacer, partial sequence; chloroplast	861	861	98% 0.0	91%	GU381493.1	799,50	75,9%
Select seq gb|JQ690293.1|	Origanum dayi isolate H43_O_day trnL-trnF intergenic spacer, partial sequence; chloroplast	843	843	96% 0.0	91%	JQ690293.1	799,09	75,9%
Select seq gb|GU381628.1|	Thymbra calostachya voucher M:Ulrich s.n. tRNA-Leu (trnL) gene and trnL-trnF intergenic spacer, partial sequence; chloroplast	857	857	98% 0.0	91%	GU381628.1	795,79	75,5%
Select seq gb|GU381489.1|	Killickia lutea voucher NU<ZAF>:Hilliard & Burtt 9876 tRNA-Leu (trnL) gene and trnL-trnF intergenic spacer, partial sequence; chloroplast	856	856	98% 0.0	91%	GU381489.1	794,86	75,5%
Select seq gb|GU381488.1|	Killickia compacta voucher M:Braeuchler 3816 tRNA-Leu (trnL) gene and trnL-trnF intergenic spacer, partial sequence; chloroplast	856	856	98% 0.0	91%	GU381488.1	794,86	75,5%
Select seq gb|DQ667488.1|	Lycopus uniflorus isolate x221 tRNA-Leu (trnL) gene and trnL-trnF intergenic spacer, partial sequence; chloroplast	856	856	100% 0.0	91%	DQ667488.1	778,96	73,9%
Select seq gb|DQ667493.1|	Lepechinia conferta isolate x234 tRNA-Leu (trnL) gene and trnL-trnF intergenic spacer, partial sequence; chloroplast	852	852	100% 0.0	91%	DQ667493.1	775,32	73,6%

Select for downloading or viewing reports	Kh056_ITS Description	Max score	Total score	Query cover	E value	Ident	Accession	(Ident/Cover)* Max score	Deviation from top hit
Select seq gb|EU626324.1|	Allium atroviolaceum voucher K:Chase 24452 small subunit ribosomal RNA gene, partial sequence; internal transcribed spacer 1, 5.8S ribosomal RNA gene, and internal transcribed spacer 2, complete sequence; and large subunit ribosomal RNA gene, partial sequence	1290	1290	96%	0.0	98%	EU626324.1	1316,88	100,0%
Select seq gb|FJ664302.1|	Allium polyanthum voucher BF-ALL-016 clone 1608 small subunit ribosomal RNA gene, partial sequence; internal transcribed spacer 1, 5.8S ribosomal RNA gene, and internal transcribed spacer 2, complete sequence; and large subunit ribosomal RNA gene, partial sequence	1273	1273	96%	0.0	98%	FJ664302.1	1299,52	98,7%
Select seq gb|FJ664301.1|	Allium polyanthum voucher BF-ALL-016 clone 1606 small subunit ribosomal RNA gene, partial sequence; internal transcribed spacer 1, 5.8S ribosomal RNA gene, and internal transcribed spacer 2, complete sequence; and large subunit ribosomal RNA gene, partial sequence	1267	1267	96%	0.0	98%	FJ664301.1	1293,40	98,2%
Select seq gb|EU626334.1|	Allium truncatum voucher K:Chase 24455 clone 280201 small subunit ribosomal RNA gene, partial sequence; internal transcribed spacer 1, 5.8S ribosomal RNA gene, and internal transcribed spacer 2, complete sequence; and large subunit ribosomal RNA gene, partial sequence	1267	1267	96%	0.0	98%	EU626334.1	1293,40	98,2%
Select seq gb|FJ664322.1|	Allium ampeloprasum voucher BF-ALL-022 clone 2226 small subunit ribosomal RNA gene, partial sequence; internal transcribed spacer 1, 5.8S ribosomal RNA gene, and internal transcribed spacer 2, complete sequence; and large subunit ribosomal RNA gene, partial sequence	1262	1262	96%	0.0	98%	FJ664322.1	1288,29	97,8%
Select seq gb|EU626319.1|	Allium ampeloprasum voucher BF-ALL-017 small subunit ribosomal RNA gene, partial sequence; internal transcribed spacer 1, 5.8S ribosomal RNA gene, and internal transcribed spacer 2, complete sequence; and large subunit ribosomal RNA gene, partial sequence	1262	1262	96%	0.0	98%	EU626319.1	1288,29	97,8%
Select seq gb|KF800370.1|	Uncultured eukaryote clone CMH279 18S ribosomal RNA gene, partial sequence; internal transcribed spacer 1, 5.8S ribosomal RNA gene, and internal transcribed spacer 2, complete sequence; and 28S ribosomal RNA gene, partial sequence	1310	1310	100%	0.0	98%	KF800370.1	1283,80	97,5%
Select seq gb|KU145494.1|	Allium ampeloprasum voucher Leek 2 18S ribosomal RNA gene, partial sequence; internal transcribed spacer 1, 5.8S ribosomal RNA gene, and internal transcribed spacer 2, complete sequence; and 28S ribosomal RNA gene, partial sequence	1181	1181	90%	0.0	97%	KU145494.1	1272,86	96,7%
Select seq gb|FJ664333.1|	Allium ampeloprasum voucher BF-ALL-022 clone 2219 small subunit ribosomal RNA gene, partial sequence; internal transcribed spacer 1, 5.8S ribosomal RNA gene, and internal transcribed spacer 2, complete sequence; and large subunit ribosomal RNA gene, partial sequence	1256	1256	96%	0.0	97%	FJ664333.1	1269,08	96,4%
Select seq gb|FJ664323.1|	Allium ampeloprasum voucher BF-ALL-046 clone 4631 small subunit ribosomal RNA gene, partial sequence; internal transcribed spacer 1, 5.8S ribosomal RNA gene, and internal transcribed spacer 2, complete sequence; and large subunit ribosomal RNA gene, partial sequence	1256	1256	96%	0.0	97%	FJ664323.1	1269,08	96,4%
Select seq gb|FJ664300.1|	Allium ampeloprasum voucher BF-ALL-046 clone 4620 small subunit ribosomal RNA gene, partial sequence; internal transcribed spacer 1, 5.8S ribosomal RNA gene, and internal transcribed spacer 2, complete sequence; and large subunit ribosomal RNA gene, partial sequence	1256	1256	96%	0.0	97%	FJ664300.1	1269,08	96,4%
Select seq gb|EU626323.1|	Allium iranicum voucher K:Chase 24454 clone 3108 small subunit ribosomal RNA gene, partial sequence; internal transcribed spacer 1, 5.8S ribosomal RNA gene, and internal transcribed spacer 2, complete sequence; and large subunit ribosomal RNA gene, partial sequence	1256	1256	96%	0.0	97%	EU626323.1	1269,08	96,4%
Select seq gb|EU626314.1|	Allium ampeloprasum voucher BF-ALL-022 small subunit ribosomal RNA gene, partial sequence; internal transcribed spacer 1, 5.8S ribosomal RNA gene, and internal transcribed spacer 2, complete sequence; and large subunit ribosomal RNA gene, partial sequence	1256	1256	96%	0.0	97%	EU626314.1	1269,08	96,4%
Select seq gb|FJ664340.1|	Allium ampeloprasum voucher BF-ALL-017 clone 1703 small subunit ribosomal RNA gene, partial sequence; internal transcribed spacer 1, 5.8S ribosomal RNA gene, and internal transcribed spacer 2, complete sequence; and large subunit ribosomal RNA gene, partial sequence	1251	1251	96%	0.0	97%	FJ664340.1	1264,03	96,0%
Select seq gb|FJ664337.1|	Allium ampeloprasum voucher BF-ALL-017 clone 1713 small subunit ribosomal RNA gene, partial sequence; internal transcribed spacer 1, 5.8S ribosomal RNA gene, and internal transcribed spacer 2, complete sequence; and large subunit ribosomal RNA gene, partial sequence	1251	1251	96%	0.0	97%	FJ664337.1	1264,03	96,0%
Select seq gb|FJ664332.1|	Allium ampeloprasum voucher BF-ALL-040 clone 4030 small subunit ribosomal RNA gene, partial sequence; internal transcribed spacer 1, 5.8S ribosomal RNA gene, and internal transcribed spacer 2, complete sequence; and large subunit ribosomal RNA gene, partial sequence	1251	1251	96%	0.0	97%	FJ664332.1	1264,03	96,0%
Select seq gb|FJ664326.1|	Allium ampeloprasum voucher BF-ALL-017 clone 1702 small subunit ribosomal RNA gene, partial sequence; internal transcribed spacer 1, 5.8S ribosomal RNA gene, and internal transcribed spacer 2, complete sequence; and large subunit ribosomal RNA gene, partial sequence	1251	1251	96%	0.0	97%	FJ664326.1	1264,03	96,0%
Select seq gb|FJ664320.1|	Allium ampeloprasum voucher BF-ALL-017 clone 1711 small subunit ribosomal RNA gene, partial sequence; internal transcribed spacer 1, 5.8S ribosomal RNA gene, and internal transcribed spacer 2, complete sequence; and large subunit ribosomal RNA gene, partial sequence	1251	1251	96%	0.0	97%	FJ664320.1	1264,03	96,0%
Select seq gb|FJ664305.1|	Allium ampeloprasum voucher BF-ALL-022 clone 2208 small subunit ribosomal RNA gene, partial sequence; internal transcribed spacer 1, 5.8S ribosomal RNA gene, and internal transcribed spacer 2, complete sequence; and large subunit ribosomal RNA gene, partial sequence	1251	1251	96%	0.0	97%	FJ664305.1	1264,03	96,0%
Select seq gb|EU626331.1|	Allium truncatum voucher K:Chase 24455 clone 2807 small subunit ribosomal RNA gene, partial sequence; internal transcribed spacer 1, 5.8S ribosomal RNA gene, and internal transcribed spacer 2, complete sequence; and large subunit ribosomal RNA gene, partial sequence	1251	1251	96%	0.0	97%	EU626331.1	1264,03	96,0%
Select seq gb|EU626318.1|	Allium iranicum voucher K:Chase 24454 clone 3101 small subunit ribosomal RNA gene, partial sequence; internal transcribed spacer 1, 5.8S ribosomal RNA gene, and internal transcribed spacer 2, complete sequence; and large subunit ribosomal RNA gene, partial sequence	1251	1251	96%	0.0	97%	EU626318.1	1264,03	96,0%
Select seq gb|EU626317.1|	Allium ampeloprasum voucher BF-ALL-006 clone 0606 small subunit ribosomal RNA gene, partial sequence; internal transcribed spacer 1, 5.8S ribosomal RNA gene, and internal transcribed spacer 2, complete sequence; and large subunit ribosomal RNA gene, partial sequence	1251	1251	96%	0.0	97%	EU626317.1	1264,03	96,0%
Select seq gb|EU626315.1|	Allium iranicum voucher K:Chase 24454 clone 310108 small subunit ribosomal RNA gene, partial sequence; internal transcribed spacer 1, 5.8S ribosomal RNA gene, and internal transcribed spacer 2, complete sequence; and large subunit ribosomal RNA gene, partial sequence	1251	1251	96%	0.0	97%	EU626315.1	1264,03	96,0%
Select seq gb|EU626312.1|	Allium iranicum voucher K:Chase 24454 clone 3104 small subunit ribosomal RNA gene, partial sequence; internal transcribed spacer 1, 5.8S ribosomal RNA gene, and internal transcribed spacer 2, complete sequence; and large subunit ribosomal RNA gene, partial sequence	1251	1251	96%	0.0	97%	EU626312.1	1264,03	96,0%
Select seq gb|FJ664339.1|	Allium ampeloprasum voucher BF-ALL-017 clone 1712 small subunit ribosomal RNA gene, partial sequence; internal transcribed spacer 1, 5.8S ribosomal RNA gene, and internal transcribed spacer 2, complete sequence; and large subunit ribosomal RNA gene, partial sequence	1245	1245	96%	0.0	97%	FJ664339.1	1257,97	95,5%
Select seq gb|FJ664331.1|	Allium ampeloprasum voucher BF-ALL-022 clone 2229 small subunit ribosomal RNA gene, partial sequence; internal transcribed spacer 1, 5.8S ribosomal RNA gene, and internal transcribed spacer 2, complete sequence; and large subunit ribosomal RNA gene, partial sequence	1245	1245	96%	0.0	97%	FJ664331.1	1257,97	95,5%

Select seq gb|FJ664330.1|	Allium ampeloprasum voucher BF-ALL-022 clone 2224 small subunit ribosomal RNA gene, partial sequence; internal transcribed spacer 1, 5.8S ribosomal RNA gene, and internal transcribed spacer 2, complete sequence; and large subunit ribosomal RNA gene, partial sequence	1245	1245	96% 0.0	97% FJ664330.1	1257,97	95,5%
Select seq gb|FJ664329.1|	Allium ampeloprasum voucher BF-ALL-022 clone 2218 small subunit ribosomal RNA gene, partial sequence; internal transcribed spacer 1, 5.8S ribosomal RNA gene, and internal transcribed spacer 2, complete sequence; and large subunit ribosomal RNA gene, partial sequence	1245	1245	96% 0.0	97% FJ664329.1	1257,97	95,5%
Select seq gb|FJ664328.1|	Allium ampeloprasum voucher BF-ALL-022 clone 2204 small subunit ribosomal RNA gene, partial sequence; internal transcribed spacer 1, 5.8S ribosomal RNA gene, and internal transcribed spacer 2, complete sequence; and large subunit ribosomal RNA gene, partial sequence	1245	1245	96% 0.0	97% FJ664328.1	1257,97	95,5%
Select seq gb|FJ664327.1|	Allium ampeloprasum voucher BF-ALL-017 clone 1715 small subunit ribosomal RNA gene, partial sequence; internal transcribed spacer 1, 5.8S ribosomal RNA gene, and internal transcribed spacer 2, complete sequence; and large subunit ribosomal RNA gene, partial sequence	1245	1245	96% 0.0	97% FJ664327.1	1257,97	95,5%
Select seq gb|FJ664325.1|	Allium ampeloprasum voucher BF-ALL-017 clone 1701 small subunit ribosomal RNA gene, partial sequence; internal transcribed spacer 1, 5.8S ribosomal RNA gene, and internal transcribed spacer 2, complete sequence; and large subunit ribosomal RNA gene, partial sequence	1245	1245	96% 0.0	97% FJ664325.1	1257,97	95,5%
Select seq gb|FJ664321.1|	Allium ampeloprasum voucher BF-ALL-046 clone 4627 small subunit ribosomal RNA gene, partial sequence; internal transcribed spacer 1, 5.8S ribosomal RNA gene, and internal transcribed spacer 2, complete sequence; and large subunit ribosomal RNA gene, partial sequence	1245	1245	96% 0.0	97% FJ664321.1	1257,97	95,5%
Select seq gb|FJ664319.1|	Allium ampeloprasum voucher BF-ALL-046 clone 4625 small subunit ribosomal RNA gene, partial sequence; internal transcribed spacer 1, 5.8S ribosomal RNA gene, and internal transcribed spacer 2, complete sequence; and large subunit ribosomal RNA gene, partial sequence	1245	1245	96% 0.0	97% FJ664319.1	1257,97	95,5%
Select seq gb|EU626330.1|	Allium truncatum voucher K:Chase 24455 clone 2813 small subunit ribosomal RNA gene, partial sequence; internal transcribed spacer 1, 5.8S ribosomal RNA gene, and internal transcribed spacer 2, complete sequence; and large subunit ribosomal RNA gene, partial sequence	1245	1245	96% 0.0	97% EU626330.1	1257,97	95,5%
Select seq gb|EU626328.1|	Allium truncatum voucher K:Chase 24455 clone 2806 small subunit ribosomal RNA gene, partial sequence; internal transcribed spacer 1, 5.8S ribosomal RNA gene, and internal transcribed spacer 2, complete sequence; and large subunit ribosomal RNA gene, partial sequence	1245	1245	96% 0.0	97% EU626328.1	1257,97	95,5%
Select seq gb|EU626326.1|	Allium truncatum voucher K:Chase 24455 clone 2801 small subunit ribosomal RNA gene, partial sequence; internal transcribed spacer 1, 5.8S ribosomal RNA gene, and internal transcribed spacer 2, complete sequence; and large subunit ribosomal RNA gene, partial sequence	1245	1245	96% 0.0	97% EU626326.1	1257,97	95,5%
Select seq gb|EU626310.1|	Allium ampeloprasum voucher BF-ALL-001 clone 0107 small subunit ribosomal RNA gene, partial sequence; internal transcribed spacer 1, 5.8S ribosomal RNA gene, and internal transcribed spacer 2, complete sequence; and large subunit ribosomal RNA gene, partial sequence	1245	1245	96% 0.0	97% EU626310.1	1257,97	95,5%
Select seq gb|EU626308.1|	Allium polyanthum voucher BF-ALL-016 small subunit ribosomal RNA gene, partial sequence; internal transcribed spacer 1, 5.8S ribosomal RNA gene, and internal transcribed spacer 2, complete sequence; and large subunit ribosomal RNA gene, partial sequence	1245	1245	96% 0.0	97% EU626308.1	1257,97	95,5%
Select seq gb|FJ664318.1|	Allium ampeloprasum voucher BF-ALL-046 clone 4622 small subunit ribosomal RNA gene, partial sequence; internal transcribed spacer 1, 5.8S ribosomal RNA gene, and internal transcribed spacer 2, complete sequence; and large subunit ribosomal RNA gene, partial sequence	1240	1240	96% 0.0	97% FJ664318.1	1252,92	95,1%
Select seq gb|FJ664316.1|	Allium ampeloprasum voucher BF-ALL-040 clone 4011 small subunit ribosomal RNA gene, partial sequence; internal transcribed spacer 1, 5.8S ribosomal RNA gene, and internal transcribed spacer 2, complete sequence; and large subunit ribosomal RNA gene, partial sequence	1240	1240	96% 0.0	97% FJ664316.1	1252,92	95,1%
Select seq gb|FJ664307.1|	Allium ampeloprasum voucher BF-ALL-040 clone 4026 small subunit ribosomal RNA gene, partial sequence; internal transcribed spacer 1, 5.8S ribosomal RNA gene, and internal transcribed spacer 2, complete sequence; and large subunit ribosomal RNA gene, partial sequence	1240	1240	96% 0.0	97% FJ664307.1	1252,92	95,1%
Select seq gb|FJ664296.1|	Allium polyanthum voucher BF-ALL-016 clone 1607 small subunit ribosomal RNA gene, partial sequence; internal transcribed spacer 1, 5.8S ribosomal RNA gene, and internal transcribed spacer 2, complete sequence; and large subunit ribosomal RNA gene, partial sequence	1240	1240	96% 0.0	97% FJ664296.1	1252,92	95,1%
Select seq gb|EU626332.1|	Allium truncatum voucher K:Chase 24455 clone 2830 small subunit ribosomal RNA gene, partial sequence; internal transcribed spacer 1, 5.8S ribosomal RNA gene, and internal transcribed spacer 2, complete sequence; and large subunit ribosomal RNA gene, partial sequence	1240	1240	96% 0.0	97% EU626332.1	1252,92	95,1%
Select seq gb|EU626329.1|	Allium truncatum voucher K:Chase 24455 clone 2832 small subunit ribosomal RNA gene, partial sequence; internal transcribed spacer 1, 5.8S ribosomal RNA gene, and internal transcribed spacer 2, complete sequence; and large subunit ribosomal RNA gene, partial sequence	1240	1240	96% 0.0	97% EU626329.1	1252,92	95,1%
Select seq gb|FJ664324.1|	Allium ampeloprasum voucher BF-ALL-046 clone 4623 small subunit ribosomal RNA gene, partial sequence; internal transcribed spacer 1, 5.8S ribosomal RNA gene, and internal transcribed spacer 2, complete sequence; and large subunit ribosomal RNA gene, partial sequence	1238	1238	96% 0.0	97% FJ664324.1	1250,90	95,0%
Select seq gb|FJ664315.1|	Allium ampeloprasum voucher BF-ALL-046 clone 4618 small subunit ribosomal RNA gene, partial sequence; internal transcribed spacer 1, 5.8S ribosomal RNA gene, and internal transcribed spacer 2, complete sequence; and large subunit ribosomal RNA gene, partial sequence	1234	1234	96% 0.0	97% FJ664315.1	1246,85	94,7%
Select seq gb|FJ664314.1|	Allium ampeloprasum voucher BF-ALL-046 clone 4617 small subunit ribosomal RNA gene, partial sequence; internal transcribed spacer 1, 5.8S ribosomal RNA gene, and internal transcribed spacer 2, complete sequence; and large subunit ribosomal RNA gene, partial sequence	1234	1234	96% 0.0	97% FJ664314.1	1246,85	94,7%
Select seq gb|FJ664313.1|	Allium ampeloprasum voucher BF-ALL-040 clone 4024 small subunit ribosomal RNA gene, partial sequence; internal transcribed spacer 1, 5.8S ribosomal RNA gene, and internal transcribed spacer 2, complete sequence; and large subunit ribosomal RNA gene, partial sequence	1234	1234	96% 0.0	97% FJ664313.1	1246,85	94,7%
Select seq gb|FJ664312.1|	Allium ampeloprasum voucher BF-ALL-040 clone 4018 small subunit ribosomal RNA gene, partial sequence; internal transcribed spacer 1, 5.8S ribosomal RNA gene, and internal transcribed spacer 2, complete sequence; and large subunit ribosomal RNA gene, partial sequence	1234	1234	96% 0.0	97% FJ664312.1	1246,85	94,7%
Select seq gb|FJ664311.1|	Allium ampeloprasum voucher BF-ALL-017 clone 1704 small subunit ribosomal RNA gene, partial sequence; internal transcribed spacer 1, 5.8S ribosomal RNA gene, and internal transcribed spacer 2, complete sequence; and large subunit ribosomal RNA gene, partial sequence	1234	1234	96% 0.0	97% FJ664311.1	1246,85	94,7%
Select seq gb|FJ664310.1|	Allium ampeloprasum voucher BF-ALL-046 clone 4626 small subunit ribosomal RNA gene, partial sequence; internal transcribed spacer 1, 5.8S ribosomal RNA gene, and internal transcribed spacer 2, complete sequence; and large subunit ribosomal RNA gene, partial sequence	1234	1234	96% 0.0	97% FJ664310.1	1246,85	94,7%
Select seq gb|FJ664295.1|	Allium polyanthum voucher BF-ALL-016 clone 1613 small subunit ribosomal RNA gene, partial sequence; internal transcribed spacer 1, 5.8S ribosomal RNA gene, and internal transcribed spacer 2, complete sequence; and large subunit ribosomal RNA gene, partial sequence	1234	1234	96% 0.0	97% FJ664295.1	1246,85	94,7%
Select seq gb|FJ664294.1|	Allium polyanthum voucher BF-ALL-016 clone 1612 small subunit ribosomal RNA gene, partial sequence; internal transcribed spacer 1, 5.8S ribosomal RNA gene, and internal transcribed spacer 2, complete sequence; and large subunit ribosomal RNA gene, partial sequence	1234	1234	96% 0.0	97% FJ664294.1	1246,85	94,7%

Select seq gb|FJ664292.1|	Allium polyanthum voucher BF-ALL-016 clone 1601 small subunit ribosomal RNA gene, partial sequence; internal transcribed spacer 1, 5.8S ribosomal RNA gene, and internal transcribed spacer 2, complete sequence; and large subunit ribosomal RNA gene, partial sequence	1234	1234	96%	0.0	97%	FJ664292.1	1246,85	94,7%
Select seq gb|EU626327.1|	Allium truncatum voucher K:Chase 24455 clone 2816 small subunit ribosomal RNA gene, partial sequence; internal transcribed spacer 1, 5.8S ribosomal RNA gene, and internal transcribed spacer 2, complete sequence; and large subunit ribosomal RNA gene, partial sequence	1234	1234	96%	0.0	97%	EU626327.1	1246,85	94,7%
Select seq gb|EU626322.1|	Allium ampeloprasum voucher BF-ALL-046 clone 4602 small subunit ribosomal RNA gene, partial sequence; internal transcribed spacer 1, 5.8S ribosomal RNA gene, and internal transcribed spacer 2, complete sequence; and large subunit ribosomal RNA gene, partial sequence	1234	1234	96%	0.0	97%	EU626322.1	1246,85	94,7%
Select seq gb|EU626321.1|	Allium ampeloprasum var. babingtonii voucher K:Chase 24451 clone 3029 small subunit ribosomal RNA gene, partial sequence; internal transcribed spacer 1, 5.8S ribosomal RNA gene, and internal transcribed spacer 2, complete sequence; and large subunit ribosomal RNA gene, partial sequence	1234	1234	96%	0.0	97%	EU626321.1	1246,85	94,7%
Select seq gb|EU626316.1|	Allium ampeloprasum var. babingtonii voucher K:Chase 24451 clone 3030 small subunit ribosomal RNA gene, partial sequence; internal transcribed spacer 1, 5.8S ribosomal RNA gene, and internal transcribed spacer 2, complete sequence; and large subunit ribosomal RNA gene, partial sequence	1234	1234	96%	0.0	97%	EU626316.1	1246,85	94,7%
Select seq gb|EU626309.1|	Allium ampeloprasum voucher BF-ALL-006 clone 0604 small subunit ribosomal RNA gene, partial sequence; internal transcribed spacer 1, 5.8S ribosomal RNA gene, and internal transcribed spacer 2, complete sequence; and large subunit ribosomal RNA gene, partial sequence	1234	1234	96%	0.0	97%	EU626309.1	1246,85	94,7%
Select seq gb|EU626307.1|	Allium ampeloprasum voucher BF-ALL-001 clone 0104 small subunit ribosomal RNA gene, partial sequence; internal transcribed spacer 1, 5.8S ribosomal RNA gene, and internal transcribed spacer 2, complete sequence; and large subunit ribosomal RNA gene, partial sequence	1234	1234	96%	0.0	97%	EU626307.1	1246,85	94,7%
Select seq gb|KT809296.1|	Allium ampeloprasum subsp. ampeloprasum clone a small subunit ribosomal RNA gene, partial sequence; internal transcribed spacer 1, 5.8S ribosomal RNA gene, and internal transcribed spacer 2, complete sequence; and large subunit ribosomal RNA gene, partial sequence	1219	1219	95%	0.0	97%	KT809296.1	1244,66	94,5%
Select seq gb|FJ664309.1|	Allium ampeloprasum voucher BF-ALL-046 clone 4628 small subunit ribosomal RNA gene, partial sequence; internal transcribed spacer 1, 5.8S ribosomal RNA gene, and internal transcribed spacer 2, complete sequence; and large subunit ribosomal RNA gene, partial sequence	1229	1229	96%	0.0	97%	FJ664309.1	1241,80	94,3%
Select seq gb|FJ664306.1|	Allium ampeloprasum voucher BF-ALL-040 clone 4007 small subunit ribosomal RNA gene, partial sequence; internal transcribed spacer 1, 5.8S ribosomal RNA gene, and internal transcribed spacer 2, complete sequence; and large subunit ribosomal RNA gene, partial sequence	1229	1229	96%	0.0	97%	FJ664306.1	1241,80	94,3%
Select seq gb|FJ664293.1|	Allium polyanthum voucher BF-ALL-016 clone 1604 small subunit ribosomal RNA gene, partial sequence; internal transcribed spacer 1, 5.8S ribosomal RNA gene, and internal transcribed spacer 2, complete sequence; and large subunit ribosomal RNA gene, partial sequence	1229	1229	96%	0.0	97%	FJ664293.1	1241,80	94,3%
Select seq gb|EU626333.1|	Allium truncatum voucher K:Chase 24455 clone 2834 small subunit ribosomal RNA gene, partial sequence; internal transcribed spacer 1, 5.8S ribosomal RNA gene, and internal transcribed spacer 2, complete sequence; and large subunit ribosomal RNA gene, partial sequence	1229	1229	96%	0.0	97%	EU626333.1	1241,80	94,3%
Select seq gb|EU626311.1|	Allium ampeloprasum voucher BF-ALL-006 clone 0601 small subunit ribosomal RNA gene, partial sequence; internal transcribed spacer 1, 5.8S ribosomal RNA gene, and internal transcribed spacer 2, complete sequence; and large subunit ribosomal RNA gene, partial sequence	1229	1229	96%	0.0	97%	EU626311.1	1241,80	94,3%
Select seq gb|FJ664299.1|	Allium polyanthum voucher BF-ALL-016 clone 1614 small subunit ribosomal RNA gene, partial sequence; internal transcribed spacer 1, 5.8S ribosomal RNA gene, and internal transcribed spacer 2, complete sequence; and large subunit ribosomal RNA gene, partial sequence	1223	1223	96%	0.0	97%	FJ664299.1	1235,74	93,8%
Select seq gb|EU626304.1|	Allium ampeloprasum subsp. ampeloprasum voucher IPK:ALL1333 clone 1310 small subunit ribosomal RNA gene, partial sequence; internal transcribed spacer 1, 5.8S ribosomal RNA gene, and internal transcribed spacer 2, complete sequence; and large subunit ribosomal RNA gene, partial sequence	1223	1223	96%	0.0	97%	EU626304.1	1235,74	93,8%
Select seq gb|EU626302.1|	Allium ampeloprasum voucher BF-ALL-010 clone 1004 small subunit ribosomal RNA gene, partial sequence; internal transcribed spacer 1, 5.8S ribosomal RNA gene, and internal transcribed spacer 2, complete sequence; and large subunit ribosomal RNA gene, partial sequence	1223	1223	96%	0.0	97%	EU626302.1	1235,74	93,8%
Select seq gb|EU626293.1|	Allium ampeloprasum voucher BF-ALL-001 clone 0102 small subunit ribosomal RNA gene, partial sequence; internal transcribed spacer 1, 5.8S ribosomal RNA gene, and internal transcribed spacer 2, complete sequence; and large subunit ribosomal RNA gene, partial sequence	1223	1223	96%	0.0	97%	EU626293.1	1235,74	93,8%
Select seq gb|EU626325.1|	Allium iranicum voucher K:Chase 24454 clone 3103 small subunit ribosomal RNA gene, partial sequence; internal transcribed spacer 1, 5.8S ribosomal RNA gene, and internal transcribed spacer 2, complete sequence; and large subunit ribosomal RNA gene, partial sequence	1218	1218	96%	0.0	96%	EU626325.1	1218,00	92,5%
Select seq gb|EU626306.1|	Allium ampeloprasum var. babingtonii voucher K:Chase 24451 clone 3010 small subunit ribosomal RNA gene, partial sequence; internal transcribed spacer 1, 5.8S ribosomal RNA gene, and internal transcribed spacer 2, complete sequence; and large subunit ribosomal RNA gene, partial sequence	1218	1218	96%	0.0	96%	EU626306.1	1218,00	92,5%
Select seq gb|EU626305.1|	Allium ampeloprasum voucher BF-ALL-006 clone 0607 small subunit ribosomal RNA gene, partial sequence; internal transcribed spacer 1, 5.8S ribosomal RNA gene, and internal transcribed spacer 2, complete sequence; and large subunit ribosomal RNA gene, partial sequence	1218	1218	96%	0.0	96%	EU626305.1	1218,00	92,5%
Select seq gb|EU626303.1|	Allium ampeloprasum voucher BF-ALL-010 clone 1007 small subunit ribosomal RNA gene, partial sequence; internal transcribed spacer 1, 5.8S ribosomal RNA gene, and internal transcribed spacer 2, complete sequence; and large subunit ribosomal RNA gene, partial sequence	1218	1218	96%	0.0	96%	EU626303.1	1218,00	92,5%
Select seq gb|EU626299.1|	Allium ampeloprasum subsp. ampeloprasum voucher IPK:ALL1333 clone 1301 small subunit ribosomal RNA gene, partial sequence; internal transcribed spacer 1, 5.8S ribosomal RNA gene, and internal transcribed spacer 2, complete sequence; and large subunit ribosomal RNA gene, partial sequence	1218	1218	96%	0.0	96%	EU626299.1	1218,00	92,5%
Select seq gb|EU626298.1|	Allium ampeloprasum voucher BF-ALL-001 clone 0103 small subunit ribosomal RNA gene, partial sequence; internal transcribed spacer 1, 5.8S ribosomal RNA gene, and internal transcribed spacer 2, complete sequence; and large subunit ribosomal RNA gene, partial sequence	1218	1218	96%	0.0	96%	EU626298.1	1218,00	92,5%
Select seq gb|EU626296.1|	Allium ampeloprasum voucher BF-ALL-015 clone 1503 small subunit ribosomal RNA gene, partial sequence; internal transcribed spacer 1, 5.8S ribosomal RNA gene, and internal transcribed spacer 2, complete sequence; and large subunit ribosomal RNA gene, partial sequence	1218	1218	96%	0.0	96%	EU626296.1	1218,00	92,5%
Select seq gb|EU626292.1|	Allium ampeloprasum voucher BF-ALL-015 clone 1501 small subunit ribosomal RNA gene, partial sequence; internal transcribed spacer 1, 5.8S ribosomal RNA gene, and internal transcribed spacer 2, complete sequence; and large subunit ribosomal RNA gene, partial sequence	1218	1218	96%	0.0	96%	EU626292.1	1218,00	92,5%
Select seq gb|EU626291.1|	Allium ampeloprasum voucher BF-ALL-010 clone 1009 small subunit ribosomal RNA gene, partial sequence; internal transcribed spacer 1, 5.8S ribosomal RNA gene, and internal transcribed spacer 2, complete sequence; and large subunit ribosomal RNA gene, partial sequence	1218	1218	96%	0.0	96%	EU626291.1	1218,00	92,5%
Select seq gb|EU626290.1|	Allium ampeloprasum voucher BF-ALL-006 clone 0605 small subunit ribosomal RNA gene, partial sequence; internal transcribed spacer 1, 5.8S ribosomal RNA gene, and internal transcribed spacer 2, complete sequence; and large subunit ribosomal RNA gene, partial sequence	1218	1218	96%	0.0	96%	EU626290.1	1218,00	92,5%

Select seq gb|FJ664298.1|	Allium polyanthum voucher BF-ALL-016 clone 1605 small subunit ribosomal RNA gene, partial sequence; internal transcribed spacer 1, 5.8S ribosomal RNA gene, and internal transcribed spacer 2, complete sequence; and large subunit ribosomal RNA gene, partial sequence	1212	1212	96%	0.0	96%	FJ664298.1	1212,00	92,0%
Select seq gb|EU626297.1|	Allium ampeloprasum voucher BF-ALL-010 clone 1001 small subunit ribosomal RNA gene, partial sequence; internal transcribed spacer 1, 5.8S ribosomal RNA gene, and internal transcribed spacer 2, complete sequence; and large subunit ribosomal RNA gene, partial sequence	1212	1212	96%	0.0	96%	EU626297.1	1212,00	92,0%
Select seq gb|EU626360.1|	Allium truncatum voucher K:Chase 24455 clone 2814 small subunit ribosomal RNA gene, partial sequence; internal transcribed spacer 1, 5.8S ribosomal RNA gene, and internal transcribed spacer 2, complete sequence; and large subunit ribosomal RNA gene, partial sequence	1195	1195	96%	0.0	96%	EU626360.1	1195,00	90,7%
Select seq gb|FJ664291.1|	Allium scorodoprasum voucher BF-ALL-044 small subunit ribosomal RNA gene, partial sequence; internal transcribed spacer 1, 5.8S ribosomal RNA gene, and internal transcribed spacer 2, complete sequence; and large subunit ribosomal RNA gene, partial sequence	1188	1188	96%	0.0	96%	FJ664291.1	1188,00	90,2%
Select seq gb|EU626377.1|	Allium pyrenaicum voucher K:Chase 24769 clone 3802 small subunit ribosomal RNA gene, partial sequence; internal transcribed spacer 1, 5.8S ribosomal RNA gene, and internal transcribed spacer 2, complete sequence; and large subunit ribosomal RNA gene, partial sequence	1184	1184	96%	0.0	96%	EU626377.1	1184,00	89,9%
Select seq gb|EU626375.1|	Allium sativum voucher BF-ALL-037 small subunit ribosomal RNA gene, partial sequence; internal transcribed spacer 1, 5.8S ribosomal RNA gene, and internal transcribed spacer 2, complete sequence; and large subunit ribosomal RNA gene, partial sequence	1184	1184	96%	0.0	96%	EU626375.1	1184,00	89,9%
Select seq gb|KF800585.1|	Uncultured eukaryote clone CMH494 18S ribosomal RNA gene, partial sequence; internal transcribed spacer 1, 5.8S ribosomal RNA gene, and internal transcribed spacer 2, complete sequence; and 28S ribosomal RNA gene, partial sequence	1232	1232	100%	0.0	96%	KF800585.1	1182,72	89,8%
Select seq gb|KT809295.1|	Allium ampeloprasum subsp. ampeloprasum clone b small subunit ribosomal RNA gene, partial sequence; internal transcribed spacer 1, 5.8S ribosomal RNA gene, and internal transcribed spacer 2, complete sequence; and large subunit ribosomal RNA gene, partial sequence	1170	1170	95%	0.0	96%	KT809295.1	1182,32	89,8%
Select seq gb|EU626395.1|	Allium scorodoprasum voucher K:Chase 26042 small subunit ribosomal RNA gene, partial sequence; internal transcribed spacer 1, 5.8S ribosomal RNA gene, and internal transcribed spacer 2, complete sequence; and large subunit ribosomal RNA gene, partial sequence	1182	1182	96%	0.0	96%	EU626395.1	1182,00	89,8%
Select seq gb|EU626383.1|	Allium pyrenaicum voucher K:Chase 24769 clone 3809 small subunit ribosomal RNA gene, partial sequence; internal transcribed spacer 1, 5.8S ribosomal RNA gene, and internal transcribed spacer 2, complete sequence; and large subunit ribosomal RNA gene, partial sequence	1179	1179	96%	0.0	96%	EU626383.1	1179,00	89,5%
Select seq gb|EU626381.1|	Allium pyrenaicum voucher K:Chase 24769 clone 3803 small subunit ribosomal RNA gene, partial sequence; internal transcribed spacer 1, 5.8S ribosomal RNA gene, and internal transcribed spacer 2, complete sequence; and large subunit ribosomal RNA gene, partial sequence	1179	1179	96%	0.0	96%	EU626381.1	1179,00	89,5%
Select seq gb|EU626380.1|	Allium pyrenaicum voucher K:Chase 24769 clone 3810 small subunit ribosomal RNA gene, partial sequence; internal transcribed spacer 1, 5.8S ribosomal RNA gene, and internal transcribed spacer 2, complete sequence; and large subunit ribosomal RNA gene, partial sequence	1179	1179	96%	0.0	96%	EU626380.1	1179,00	89,5%
Select seq gb|EU626382.1|	Allium pyrenaicum voucher K:Chase 24769 clone 3808 small subunit ribosomal RNA gene, partial sequence; internal transcribed spacer 1, 5.8S ribosomal RNA gene, and internal transcribed spacer 2, complete sequence; and large subunit ribosomal RNA gene, partial sequence	1173	1173	96%	0.0	95%	EU626382.1	1160,78	88,1%
Select seq gb|EU626379.1|	Allium pyrenaicum voucher K:Chase 24769 clone 3807 small subunit ribosomal RNA gene, partial sequence; internal transcribed spacer 1, 5.8S ribosomal RNA gene, and internal transcribed spacer 2, complete sequence; and large subunit ribosomal RNA gene, partial sequence	1173	1173	96%	0.0	95%	EU626379.1	1160,78	88,1%
Select seq gb|EU626376.1|	Allium pyrenaicum voucher K:Chase 24769 clone 3801 small subunit ribosomal RNA gene, partial sequence; internal transcribed spacer 1, 5.8S ribosomal RNA gene, and internal transcribed spacer 2, complete sequence; and large subunit ribosomal RNA gene, partial sequence	1173	1173	96%	0.0	95%	EU626376.1	1160,78	88,1%
Select seq gb|EU626374.1|	Allium pseudoampeloprasum voucher BF-ALL-024 small subunit ribosomal RNA gene, partial sequence; internal transcribed spacer 1, 5.8S ribosomal RNA gene, and internal transcribed spacer 2, complete sequence; and large subunit ribosomal RNA gene, partial sequence	1173	1173	96%	0.0	95%	EU626374.1	1160,78	88,1%
Select seq gb|EU626373.1|	Allium leucanthum voucher IPK:TAX6034 small subunit ribosomal RNA gene, partial sequence; internal transcribed spacer 1, 5.8S ribosomal RNA gene, and internal transcribed spacer 2, complete sequence; and large subunit ribosomal RNA gene, partial sequence	1173	1173	96%	0.0	95%	EU626373.1	1160,78	88,1%
Select seq gb|EU626338.1|	Allium ampeloprasum voucher BF-ALL-015 clone 1506 small subunit ribosomal RNA gene, partial sequence; internal transcribed spacer 1, 5.8S ribosomal RNA gene, and internal transcribed spacer 2, complete sequence; and large subunit ribosomal RNA gene, partial sequence	1173	1173	96%	0.0	95%	EU626338.1	1160,78	88,1%
Select seq gb|EU626353.1|	Allium ampeloprasum voucher BF-ALL-010 clone 1003 small subunit ribosomal RNA gene, partial sequence; internal transcribed spacer 1, 5.8S ribosomal RNA gene, and internal transcribed spacer 2, complete sequence; and large subunit ribosomal RNA gene, partial sequence	1171	1171	96%	0.0	95%	EU626353.1	1158,80	88,0%

Select for downloading or viewing reports	Kh056_trnL Description	Max score	Total score	Query cover	E value	Ident	Accession	(Ident/Cover)* Max score	Deviation from top hit
Select seq gb|EU626261.1|	Allium sativum voucher BF-ALL-037 tRNA-Leu (trnL) gene, partial sequence; trnL-trnF intergenic spacer, complete sequence; and tRNA-Phe (trnF) gene, partial sequence; chloroplast	913	913	97%	0.0	98%	EU626261.1	922,41	100,0%
Select seq gb|EU626258.1|	Allium pseudoampeloprasum voucher BF-ALL-024 tRNA-Leu (trnL) gene, partial sequence; trnL-trnF intergenic spacer, complete sequence; and tRNA-Phe (trnF) gene, partial sequence; chloroplast	913	913	97%	0.0	98%	EU626258.1	922,41	100,0%
Select seq gb|EU626262.1|	Allium tuncelianum voucher K:Chase 24456 tRNA-Leu (trnL) gene, partial sequence; trnL-trnF intergenic spacer, complete sequence; and tRNA-Phe (trnF) gene, partial sequence; chloroplast	907	907	97%	0.0	98%	EU626262.1	916,35	99,3%
Select seq gb|EU626256.1|	Allium acutiflorum voucher BF-ALL-025 tRNA-Leu (trnL) gene, partial sequence; trnL-trnF intergenic spacer, complete sequence; and tRNA-Phe (trnF) gene, partial sequence; chloroplast	907	907	97%	0.0	98%	EU626256.1	916,35	99,3%
Select seq gb|EU626238.1|	Allium ampeloprasum voucher BF-ALL-017 tRNA-Leu (trnL) gene, partial sequence; trnL-trnF intergenic spacer, complete sequence; and tRNA-Phe (trnF) gene, partial sequence; chloroplast	907	907	97%	0.0	98%	EU626238.1	916,35	99,3%
Select seq gb|FJ628601.1|	Allium scorodoprasum voucher BF-ALL-044 tRNA-Leu (trnL) gene, partial sequence; trnL-trnF intergenic spacer, complete sequence; and tRNA-Phe (trnF) gene, partial sequence; chloroplast	902	902	97%	0.0	98%	FJ628601.1	911,30	98,8%
Select seq gb|EU626263.1|	Allium scorodoprasum voucher K:Chase 26042 tRNA-Leu (trnL) gene, partial sequence; trnL-trnF intergenic spacer, complete sequence; and tRNA-Phe (trnF) gene, partial sequence; chloroplast	902	902	97%	0.0	98%	EU626263.1	911,30	98,8%
Select seq gb|EU626239.1|	Allium ampeloprasum voucher BF-ALL-040 tRNA-Leu (trnL) gene, partial sequence; trnL-trnF intergenic spacer, complete sequence; and tRNA-Phe (trnF) gene, partial sequence; chloroplast	902	902	97%	0.0	98%	EU626239.1	911,30	98,8%
Select seq gb|EU626244.1|	Allium truncatum voucher K:Chase 24455 tRNA-Leu (trnL) gene, partial sequence; trnL-trnF intergenic spacer, complete sequence; and tRNA-Phe (trnF) gene, partial sequence; chloroplast	900	900	97%	0.0	98%	EU626244.1	909,28	98,6%
Select seq gb|EU626252.1|	Allium bourgeaui subsp. bourgeaui voucher BF-ALL-021 tRNA-Leu (trnL) gene, partial sequence; trnL-trnF intergenic spacer, complete sequence; and tRNA-Phe (trnF) gene, partial sequence; chloroplast	896	896	97%	0.0	98%	EU626252.1	905,24	98,1%
Select seq gb|FJ628600.1|	Allium scorodoprasum voucher BF-ALL-042 tRNA-Leu (trnL) gene, partial sequence; trnL-trnF intergenic spacer, complete sequence; and tRNA-Phe (trnF) gene, partial sequence; chloroplast	885	885	97%	0.0	97%	FJ628600.1	885,00	95,9%
Select seq gb|KF143838.1|	Allium oreoprasum voucher H11070704 tRNA-Leu (trnL) gene, partial sequence; trnL-trnF intergenic spacer, complete sequence; and tRNA-Phe (trnF) gene, partial sequence; chloroplast	854	854	94%	0.0	97%	KF143838.1	881,26	95,5%
Select seq gb|EU626257.1|	Allium pyrenaicum voucher K:Chase 24769 tRNA-Leu (trnL) gene, partial sequence; trnL-trnF intergenic spacer, complete sequence; and tRNA-Phe (trnF) gene, partial sequence; chloroplast	881	881	97%	0.0	97%	EU626257.1	881,00	95,5%
Select seq gb|KF143840.1|	Allium tuberosum voucher H20120712 tRNA-Leu (trnL) gene, partial sequence; trnL-trnF intergenic spacer, complete sequence; and tRNA-Phe (trnF) gene, partial sequence; chloroplast	852	852	94%	0.0	97%	KF143840.1	879,19	95,3%
Select seq gb|KF143855.1|	Allium changduense voucher G10-1263 tRNA-Leu (trnL) gene, partial sequence; trnL-trnF intergenic spacer, complete sequence; and tRNA-Phe (trnF) gene, partial sequence; chloroplast	850	850	94%	0.0	97%	KF143855.1	877,13	95,1%
Select seq gb|JQ900598.1|	Allium tuberosum cultivar Chunjiu tRNA-Leu (trnL) gene, partial sequence; trnL-trnF intergenic spacer, complete sequence; and tRNA-Phe (trnF) gene, partial sequence; chloroplast	821	821	91%	0.0	97%	JQ900598.1	875,13	94,9%
Select seq gb|JF262647.1|	Allium tuberosum tRNA-Leu (trnL) gene, partial sequence; trnL-trnF intergenic spacer, complete sequence; and tRNA-Phe (trnF) gene, partial sequence	821	821	91%	0.0	97%	JF262647.1	875,13	94,9%
Select seq gb|JQ900591.1|	Allium tuberosum cultivar Pingjiuzayi tRNA-Leu (trnL) gene, partial sequence; trnL-trnF intergenic spacer, complete sequence; and tRNA-Phe (trnF) gene, partial sequence; chloroplast	815	815	91%	0.0	97%	JQ900591.1	868,74	94,2%
Select seq gb|JQ900590.1|	Allium tuberosum cultivar Guilinchuanshanjiu tRNA-Leu (trnL) gene, partial sequence; trnL-trnF intergenic spacer, complete sequence; and tRNA-Phe (trnF) gene, partial sequence; chloroplast	815	815	91%	0.0	97%	JQ900590.1	868,74	94,2%
Select seq gb|JQ900586.1|	Allium tuberosum cultivar Huanjiu tRNA-Leu (trnL) gene, partial sequence; trnL-trnF intergenic spacer, complete sequence; and tRNA-Phe (trnF) gene, partial sequence; chloroplast	815	815	91%	0.0	97%	JQ900586.1	868,74	94,2%
Select seq gb|JQ900584.1|	Allium tuberosum cultivar 791 tRNA-Leu (trnL) gene, partial sequence; trnL-trnF intergenic spacer, complete sequence; and tRNA-Phe (trnF) gene, partial sequence; chloroplast	815	815	91%	0.0	97%	JQ900584.1	868,74	94,2%
Select seq gb|JQ900597.1|	Allium tuberosum cultivar P3 tRNA-Leu (trnL) gene, partial sequence; trnL-trnF intergenic spacer, complete sequence; and tRNA-Phe (trnF) gene, partial sequence; chloroplast	809	809	91%	0.0	97%	JQ900597.1	862,34	93,5%
Select seq gb|JQ900601.1|	Allium tuberosum cultivar Heilongjiang2malan tRNA-Leu (trnL) gene, partial sequence; trnL-trnF intergenic spacer, complete sequence; and tRNA-Phe (trnF) gene, partial sequence; chloroplast	808	808	91%	0.0	97%	JQ900601.1	861,27	93,4%
Select seq gb|JQ900594.1|	Allium tuberosum cultivar Baimian tRNA-Leu (trnL) gene, partial sequence; trnL-trnF intergenic spacer, complete sequence; and tRNA-Phe (trnF) gene, partial sequence; chloroplast	808	808	91%	0.0	97%	JQ900594.1	861,27	93,4%
Select seq gb|JQ900592.1|	Allium tuberosum cultivar Haogen12 tRNA-Leu (trnL) gene, partial sequence; trnL-trnF intergenic spacer, complete sequence; and tRNA-Phe (trnF) gene, partial sequence; chloroplast	808	808	91%	0.0	97%	JQ900592.1	861,27	93,4%
Select seq gb|JQ900582.1|	Allium tuberosum cultivar Liaoningmalan tRNA-Leu (trnL) gene, partial sequence; trnL-trnF intergenic spacer, complete sequence; and tRNA-Phe (trnF) gene, partial sequence; chloroplast	808	808	91%	0.0	97%	JQ900582.1	861,27	93,4%

Select seq gb|GU570991.1|	Allium spicatum tRNA-Leu (trnL) gene, partial sequence; trnL-trnF intergenic spacer, complete sequence; and tRNA-Phe (trnF) gene, partial sequence; chloroplast	808	808	91% 0.0	97% GU570991.1	861,27	93,4%
Select seq gb|KF143844.1|	Allium spicatum voucher XZ2011082501 tRNA-Leu (trnL) gene, partial sequence; trnL-trnF intergenic spacer, complete sequence; and tRNA-Phe (trnF) gene, partial sequence; chloroplast	833	833	94% 0.0	97% KF143844.1	859,59	93,2%
Select seq gb|JQ900583.1|	Allium tuberosum cultivar Taijiu tRNA-Leu (trnL) gene, partial sequence; trnL-trnF intergenic spacer, complete sequence; and tRNA-Phe (trnF) gene, partial sequence; chloroplast	806	806	91% 0.0	97% JQ900583.1	859,14	93,1%
Select seq gb|EU626246.1|	Allium ampeloprasum var. babingtonii voucher K:Chase 24451 tRNA-Leu (trnL) gene, partial sequence; trnL-trnF intergenic spacer, complete sequence; and tRNA-Phe (trnF) gene, partial sequence; chloroplast	863	863	97% 0.0	96% EU626246.1	854,10	92,6%
Select seq gb|EU626245.1|	Allium ampeloprasum voucher BF-ALL-015 tRNA-Leu (trnL) gene, partial sequence; trnL-trnF intergenic spacer, complete sequence; and tRNA-Phe (trnF) gene, partial sequence; chloroplast	857	857	97% 0.0	96% EU626245.1	848,16	92,0%
Select seq gb|GU570985.1|	Allium cyathophorum tRNA-Leu (trnL) gene, partial sequence; trnL-trnF intergenic spacer, complete sequence; and tRNA-Phe (trnF) gene, partial sequence; chloroplast	797	797	91% 0.0	96% GU570985.1	840,79	91,2%
Select seq gb|JF262642.1|	Allium neriniflorum tRNA-Leu (trnL) gene, partial sequence; trnL-trnF intergenic spacer, complete sequence; and tRNA-Phe (trnF) gene, partial sequence	795	795	91% 0.0	96% JF262642.1	838,68	90,9%
Select seq gb|KF143837.1|	Allium tubiflorum voucher LQQ10081502 tRNA-Leu (trnL) gene, partial sequence; trnL-trnF intergenic spacer, complete sequence; and tRNA-Phe (trnF) gene, partial sequence; chloroplast	821	821	94% 0.0	96% KF143837.1	838,47	90,9%
Select seq gb|JF262652.1|	Allium tenuissimum tRNA-Leu (trnL) gene, partial sequence; trnL-trnF intergenic spacer, complete sequence; and tRNA-Phe (trnF) gene, partial sequence	793	793	91% 0.0	96% JF262652.1	836,57	90,7%
Select seq gb|JF262646.1|	Allium ramosum tRNA-Leu (trnL) gene, partial sequence; trnL-trnF intergenic spacer, complete sequence; and tRNA-Phe (trnF) gene, partial sequence	793	793	91% 0.0	96% JF262646.1	836,57	90,7%
Select seq emb|FN178319.1|	Allium viridiflorum chloroplast tRNA-Leu gene for transfer RNA-Leu (partial), IGS and tRNA-Phe gene for transfer RNA-Phe (partial), specimen voucher GAT 5699	793	793	91% 0.0	96% FN178319.1	836,57	90,7%
Select seq gb|KF143848.1|	Allium tenuissimum voucher zcj2012081908 tRNA-Leu (trnL) gene, partial sequence; trnL-trnF intergenic spacer, complete sequence; and tRNA-Phe (trnF) gene, partial sequence; chloroplast	819	819	94% 0.0	96% KF143848.1	836,43	90,7%
Select seq gb|JF262649.1|	Allium koreanum tRNA-Leu (trnL) gene, partial sequence; trnL-trnF intergenic spacer, complete sequence; and tRNA-Phe (trnF) gene, partial sequence	791	791	91% 0.0	96% JF262649.1	834,46	90,5%
Select seq emb|FN178320.1|	Allium viridulum chloroplast tRNA-Leu gene for transfer RNA-Leu (partial), IGS and tRNA-Phe gene for transfer RNA-Phe (partial), specimen voucher GAT 1I	791	791	91% 0.0	96% FN178320.1	834,46	90,5%
Select seq emb|FN178302.1|	Allium tulipifolium chloroplast tRNA-Leu gene for transfer RNA-Leu (partial), IGS and tRNA-Phe gene for transfer RNA-Phe (partial), specimen voucher GAT 6073	791	791	91% 0.0	96% FN178302.1	834,46	90,5%
Select seq gb|KF143861.1|	Allium galanthum voucher 97631 tRNA-Leu (trnL) gene, partial sequence; trnL-trnF intergenic spacer, complete sequence; and tRNA-Phe (trnF) gene, partial sequence; chloroplast	817	817	94% 0.0	96% KF143861.1	834,38	90,5%
Select seq gb|KF143845.1|	Allium cyathophorum var. farreri voucher zcj2011072301 tRNA-Leu (trnL) gene, partial sequence; trnL-trnF intergenic spacer, complete sequence; and tRNA-Phe (trnF) gene, partial sequence; chloroplast	817	817	94% 0.0	96% KF143845.1	834,38	90,5%
Select seq gb|JF262643.1|	Allium microdictyon tRNA-Leu (trnL) gene, partial sequence; trnL-trnF intergenic spacer, complete sequence; and tRNA-Phe (trnF) gene, partial sequence	789	789	91% 0.0	96% JF262643.1	832,35	90,2%
Select seq emb|FN178321.1|	Allium viridulum chloroplast tRNA-Leu gene for transfer RNA-Leu (partial), IGS and tRNA-Phe gene for transfer RNA-Phe (partial), specimen voucher GAT 5I	789	789	91% 0.0	96% FN178321.1	832,35	90,2%
Select seq emb|FN178160.1|	Allium robustum chloroplast tRNA-Leu gene for transfer RNA-Leu (partial), IGS and tRNA-Phe gene for transfer RNA-Phe (partial), specimen voucher GAT 4I	789	789	91% 0.0	96% FN178160.1	832,35	90,2%
Select seq gb|KF143849.1|	Allium polyrhizum voucher 97804 tRNA-Leu (trnL) gene, partial sequence; trnL-trnF intergenic spacer, complete sequence; and tRNA-Phe (trnF) gene, partial sequence; chloroplast	815	815	94% 0.0	96% KF143849.1	832,34	90,2%
Select seq gb|KF728079.1|	Allium cepa genotype male sterile (S) chloroplast, complete genome	839	839	97% 0.0	96% KF728079.1	830,35	90,0%
Select seq gb|JF262651.1|	Allium anisopodium tRNA-Leu (trnL) gene, partial sequence; trnL-trnF intergenic spacer, complete sequence; and tRNA-Phe (trnF) gene, partial sequence	787	787	91% 0.0	96% JF262651.1	830,24	90,0%
Select seq emb|FN178303.1|	Allium tulipifolium chloroplast tRNA-Leu gene for transfer RNA-Leu (partial), IGS and tRNA-Phe gene for transfer RNA-Phe (partial), specimen voucher GAT 6I	787	787	91% 0.0	96% FN178303.1	830,24	90,0%
Select seq gb|JF262650.1|	Allium strictum tRNA-Leu (trnL) gene, partial sequence; trnL-trnF intergenic spacer, complete sequence; and tRNA-Phe (trnF) gene, partial sequence	785	785	91% 0.0	96% JF262650.1	828,13	89,8%
Select seq gb|GU570987.1|	Allium galanthum tRNA-Leu (trnL) gene, partial sequence; trnL-trnF intergenic spacer, complete sequence; and tRNA-Phe (trnF) gene, partial sequence; chloroplast	785	785	91% 0.0	96% GU570987.1	828,13	89,8%
Select seq emb|FN177965.1|	Allium chelotum chloroplast tRNA-Leu gene for transfer RNA-Leu (partial), IGS and tRNA-Phe gene for transfer RNA-Phe (partial), specimen voucher GAT I323	784	784	91% 0.0	96% FN177965.1	827,08	89,7%
Select seq emb|FN177964.1|	Allium chelotum chloroplast tRNA-Leu gene for transfer RNA-Leu (partial), IGS and tRNA-Phe gene for transfer RNA-Phe (partial), specimen voucher GAT 6236	784	784	91% 0.0	96% FN177964.1	827,08	89,7%

Select seq emb|FN178135.1|	Allium nigrum chloroplast tRNA-Leu gene for transfer RNA-Leu (partial), IGS and tRNA-Phe gene for transfer RNA-Phe (partial), specimen voucher GAT 0515	784	784	91%	0.0	96%	FN178135.1	827,08	89,7%
Select seq emb|FN178080.1|	Allium koelzii chloroplast tRNA-Leu gene for transfer RNA-Leu (partial), IGS and tRNA-Phe gene for transfer RNA-Phe (partial), specimen voucher GAT 6644	784	784	91%	0.0	96%	FN178080.1	827,08	89,7%
Select seq gb|KF550177.1|	Allium victorialis voucher GAT:07-28-0001-20 tRNA-Leu (trnL) gene, partial sequence; trnL-trnF intergenic spacer, complete sequence; and tRNA-Phe (trnF) gene, partial sequence; chloroplast	808	808	94%	0.0	96%	KF550177.1	825,19	89,5%
Select seq gb|KF550168.1|	Allium ochotense voucher GAT:03-47-0004-20 tRNA-Leu (trnL) gene, partial sequence; trnL-trnF intergenic spacer, complete sequence; and tRNA-Phe (trnF) gene, partial sequence; chloroplast	808	808	94%	0.0	96%	KF550168.1	825,19	89,5%
Select seq gb|KF143850.1|	Allium bidentatum voucher 97813 tRNA-Leu (trnL) gene, partial sequence; trnL-trnF intergenic spacer, complete sequence; and tRNA-Phe (trnF) gene, partial sequence; chloroplast	811	811	94%	0.0	95%	KF143850.1	819,63	88,9%
Select seq gb|GU565918.1|	Allium polyrhizum tRNA-Leu (trnL) gene, partial sequence; trnL-trnF intergenic spacer, complete sequence; and tRNA-Phe (trnF) gene, partial sequence; chloroplast	784	784	91%	0.0	95%	GU565918.1	818,46	88,7%
Select seq gb|KF143851.1|	Allium mongolicum voucher H11072804 tRNA-Leu (trnL) gene, partial sequence; trnL-trnF intergenic spacer, complete sequence; and tRNA-Phe (trnF) gene, partial sequence; chloroplast	809	809	94%	0.0	95%	KF143851.1	817,61	88,6%
Select seq gb|KF143847.1|	Allium anisopodium voucher H11071403 tRNA-Leu (trnL) gene, partial sequence; trnL-trnF intergenic spacer, complete sequence; and tRNA-Phe (trnF) gene, partial sequence; chloroplast	808	808	94%	0.0	95%	KF143847.1	816,60	88,5%
Select seq gb|JF262662.1|	Allium schoenoprasum tRNA-Leu (trnL) gene, partial sequence; trnL-trnF intergenic spacer, complete sequence; and tRNA-Phe (trnF) gene, partial sequence	782	782	91%	0.0	95%	JF262662.1	816,37	88,5%
Select seq gb|KM088014.1|	Allium cepa strain CMS-S chloroplast, complete genome	833	833	97%	0.0	95%	KM088014.1	815,82	88,4%
Select seq gb|FJ628602.1|	Allium oleraceum voucher BF-ALL-043 tRNA-Leu (trnL) gene, partial sequence; trnL-trnF intergenic spacer, complete sequence; and tRNA-Phe (trnF) gene, partial sequence; chloroplast	828	828	97%	0.0	95%	FJ628602.1	810,93	87,9%
Select seq gb|KF143853.1|	Allium weschniakowii voucher XJ0072 tRNA-Leu (trnL) gene, partial sequence; trnL-trnF intergenic spacer, complete sequence; and tRNA-Phe (trnF) gene, partial sequence; chloroplast	802	802	94%	0.0	95%	KF143853.1	810,53	87,9%
Select seq gb|KF143852.1|	Allium weschniakowii voucher XJ0071 tRNA-Leu (trnL) gene, partial sequence; trnL-trnF intergenic spacer, complete sequence; and tRNA-Phe (trnF) gene, partial sequence; chloroplast	802	802	94%	0.0	95%	KF143852.1	810,53	87,9%
Select seq gb|KC139036.2|	Allium stellatum voucher MBG 2002-1694 tRNA-Leu (trnL) gene, partial sequence; trnL-trnF intergenic spacer, complete sequence; and tRNA-Phe (trnF) gene, partial sequence; chloroplast	802	802	94%	0.0	95%	KC139036.2	810,53	87,9%
Select seq gb|KC138973.2|	Allium acuminatum voucher UMO<USA-MO>:s.n. tRNA-Leu (trnL) gene, partial sequence; trnL-trnF intergenic spacer, complete sequence; and tRNA-Phe (trnF) gene, partial sequence; chloroplast	802	802	94%	0.0	95%	KC138973.2	810,53	87,9%
Select seq gb|KF143856.1|	Allium forrestii voucher m12101606 tRNA-Leu (trnL) gene, partial sequence; trnL-trnF intergenic spacer, complete sequence; and tRNA-Phe (trnF) gene, partial sequence; chloroplast	800	800	94%	0.0	95%	KF143856.1	808,51	87,7%
Select seq gb|KF550174.1|	Allium tricoccum voucher GAT:99-43-0005-10 tRNA-Leu (trnL) gene, partial sequence; trnL-trnF intergenic spacer, complete sequence; and tRNA-Phe (trnF) gene, partial sequence; chloroplast	800	800	94%	0.0	95%	KF550174.1	808,51	87,7%
Select seq gb|KF550171.1|	Allium schoenoprasum voucher RSA:043 tRNA-Leu (trnL) gene, partial sequence; trnL-trnF intergenic spacer, complete sequence; and tRNA-Phe (trnF) gene, partial sequence; chloroplast	800	800	94%	0.0	95%	KF550171.1	808,51	87,7%
Select seq gb|KC139047.2|	Allium tricoccum voucher UMO<USA-MO>:09-65 tRNA-Leu (trnL) gene, partial sequence; trnL-trnF intergenic spacer, complete sequence; and tRNA-Phe (trnF) gene, partial sequence; chloroplast	800	800	94%	0.0	95%	KC139047.2	808,51	87,7%
Select seq gb|KF143860.1|	Allium subtilissimum voucher H11072605 tRNA-Leu (trnL) gene, partial sequence; trnL-trnF intergenic spacer, complete sequence; and tRNA-Phe (trnF) gene, partial sequence; chloroplast	798	798	94%	0.0	95%	KF143860.1	806,49	87,4%
Select seq gb|KF143859.1|	Allium subtilissimum voucher H11071701 tRNA-Leu (trnL) gene, partial sequence; trnL-trnF intergenic spacer, complete sequence; and tRNA-Phe (trnF) gene, partial sequence; chloroplast	798	798	94%	0.0	95%	KF143859.1	806,49	87,4%
Select seq gb|KF143858.1|	Allium caespitosum voucher H11071401 tRNA-Leu (trnL) gene, partial sequence; trnL-trnF intergenic spacer, complete sequence; and tRNA-Phe (trnF) gene, partial sequence; chloroplast	798	798	94%	0.0	95%	KF143858.1	806,49	87,4%
Select seq gb|KF143857.1|	Allium caespitosum voucher H11071501 tRNA-Leu (trnL) gene, partial sequence; trnL-trnF intergenic spacer, complete sequence; and tRNA-Phe (trnF) gene, partial sequence; chloroplast	798	798	94%	0.0	95%	KF143857.1	806,49	87,4%
Select seq gb|KF143843.1|	Allium cyathophorum var. cyathophorum voucher y11100401 tRNA-Leu (trnL) gene, partial sequence; trnL-trnF intergenic spacer, complete sequence; and tRNA-Phe (trnF) gene, partial sequence; chloroplast	798	798	94%	0.0	95%	KF143843.1	806,49	87,4%
Select seq gb|KC139045.2|	Allium flavum voucher UCBG:81.0952 tRNA-Leu (trnL) gene, partial sequence; trnL-trnF intergenic spacer, complete sequence; and tRNA-Phe (trnF) gene, partial sequence; chloroplast	797	797	94%	0.0	95%	KC139045.2	805,48	87,3%
Select seq gb|KC139008.2|	Allium kunthii voucher RSA:001 tRNA-Leu (trnL) gene, partial sequence; trnL-trnF intergenic spacer, complete sequence; and tRNA-Phe (trnF) gene, partial sequence; chloroplast	797	797	94%	0.0	95%	KC139008.2	805,48	87,3%
Select seq gb|KC138997.2|	Allium eurotophilum voucher RSA:60887 tRNA-Leu (trnL) gene, partial sequence; trnL-trnF intergenic spacer, complete sequence; and tRNA-Phe (trnF) gene, partial sequence; chloroplast	797	797	94%	0.0	95%	KC138997.2	805,48	87,3%
Select seq gb|KC138990.2|	Allium cuthbertii tRNA-Leu (trnL) gene, partial sequence; trnL-trnF intergenic spacer, complete sequence; and tRNA-Phe (trnF) gene, partial sequence; chloroplast	797	797	94%	0.0	95%	KC138990.2	805,48	87,3%

Select seq gb|KF550166.1|	Allium monanthum voucher GAT:s.n. tRNA-Leu (trnL) gene, partial sequence; trnL-trnF intergenic spacer, complete sequence; and tRNA-Phe (trnF) gene, partial sequence; chloroplast	793	793	94% 0.0	95% KF550166.1	801,44	86,9%
Select seq gb|KC139037.2|	Allium textile voucher UMO<USA-MO>:08-31 tRNA-Leu (trnL) gene, partial sequence; trnL-trnF intergenic spacer, complete sequence; and tRNA-Phe (trnF) gene, partial sequence; chloroplast	791	791	94% 0.0	95% KC139037.2	799,41	86,7%
Select seq gb|KC139035.2|	Allium speculae tRNA-Leu (trnL) gene, partial sequence; trnL-trnF intergenic spacer, complete sequence; and tRNA-Phe (trnF) gene, partial sequence; chloroplast	791	791	94% 0.0	95% KC139035.2	799,41	86,7%
Select seq gb|KC139029.2|	Allium runyonii voucher UMO<USA-MO>:s.n. tRNA-Leu (trnL) gene, partial sequence; trnL-trnF intergenic spacer, complete sequence; and tRNA-Phe (trnF) gene, partial sequence; chloroplast	791	791	94% 0.0	95% KC139029.2	799,41	86,7%
Select seq gb|KC139011.2|	Allium macropetalum voucher UMO<USA-MO>:07-12 tRNA-Leu (trnL) gene, partial sequence; trnL-trnF intergenic spacer, complete sequence; and tRNA-Phe (trnF) gene, partial sequence; chloroplast	791	791	94% 0.0	95% KC139011.2	799,41	86,7%
Select seq gb|KC138996.2|	Allium elmendorfii voucher UMO<USA-MO>:26649 tRNA-Leu (trnL) gene, partial sequence; trnL-trnF intergenic spacer, complete sequence; and tRNA-Phe (trnF) gene, partial sequence; chloroplast	791	791	94% 0.0	95% KC138996.2	799,41	86,7%
Select seq gb|KC138995.2|	Allium drummondii voucher UMO<USA-MO>:07-04 tRNA-Leu (trnL) gene, partial sequence; trnL-trnF intergenic spacer, complete sequence; and tRNA-Phe (trnF) gene, partial sequence; chloroplast	791	791	94% 0.0	95% KC138995.2	799,41	86,7%
Select seq gb|KC138986.2|	Allium coryi voucher LL:7900 tRNA-Leu (trnL) gene, partial sequence; trnL-trnF intergenic spacer, complete sequence; and tRNA-Phe (trnF) gene, partial sequence; chloroplast	791	791	94% 0.0	95% KC138986.2	799,41	86,7%
Select seq gb|KC139002.2|	Allium haematochiton voucher UMO<USA-MO>:07-15 tRNA-Leu (trnL) gene, partial sequence; trnL-trnF intergenic spacer, complete sequence; and tRNA-Phe (trnF) gene, partial sequence; chloroplast	789	789	94% 0.0	95% KC139002.2	797,39	86,4%
Select seq gb|FJ628603.1|	Allium cepa voucher BF-ALL-047 tRNA-Leu (trnL) gene, partial sequence; trnL-trnF intergenic spacer, complete sequence; and tRNA-Phe (trnF) gene, partial sequence; chloroplast	813	813	97% 0.0	95% FJ628603.1	796,24	86,3%
Select seq gb|KF143862.1|	Allium atrosanguineum var. atrosanguineum voucher m12070601 tRNA-Leu (trnL) gene, partial sequence; trnL-trnF intergenic spacer, complete sequence; and tRNA-Phe (trnF) gene, partial sequence; chloroplast	787	787	94% 0.0	95% KF143862.1	795,37	86,2%
Select seq gb|FJ628604.1|	Allium fistulosum voucher BF-ALL-048 tRNA-Leu (trnL) gene, partial sequence; trnL-trnF intergenic spacer, complete sequence; and tRNA-Phe (trnF) gene, partial sequence; chloroplast	811	811	97% 0.0	95% FJ628604.1	794,28	86,1%
Select seq gb|KC139022.2|	Allium passeyi voucher UMO<USA-MO>:08-27 tRNA-Leu (trnL) gene, partial sequence; trnL-trnF intergenic spacer, complete sequence; and tRNA-Phe (trnF) gene, partial sequence; chloroplast	785	785	94% 0.0	95% KC139022.2	793,35	86,0%
Select seq gb|KC138980.2|	Allium brevistylum voucher UMO<USA-MO>:08-34 tRNA-Leu (trnL) gene, partial sequence; trnL-trnF intergenic spacer, complete sequence; and tRNA-Phe (trnF) gene, partial sequence; chloroplast	785	785	94% 0.0	95% KC138980.2	793,35	86,0%
Select seq gb|KF143841.1|	Allium trifurcatum voucher H2012080102 tRNA-Leu (trnL) gene, partial sequence; trnL-trnF intergenic spacer, complete sequence; and tRNA-Phe (trnF) gene, partial sequence; chloroplast	784	784	94% 0.0	95% KF143841.1	792,34	85,9%
Select seq gb|KC138976.2|	Allium atrorubens voucher UMO<USA-MO>:08-13 tRNA-Leu (trnL) gene, partial sequence; trnL-trnF intergenic spacer, complete sequence; and tRNA-Phe (trnF) gene, partial sequence; chloroplast	784	784	94% 0.0	95% KC138976.2	792,34	85,9%
Select seq gb|KC138972.2|	Allium abramsii voucher UMO<USA-MO>:07-39 tRNA-Leu (trnL) gene, partial sequence; trnL-trnF intergenic spacer, complete sequence; and tRNA-Phe (trnF) gene, partial sequence; chloroplast	784	784	94% 0.0	95% KC138972.2	792,34	85,9%

Select for downloading or viewing reports	Kh057_ITS Description	Max score	Total score	Query cover	E value	Ident	Accession	(Ident/Cover)* Max score	Deviation from top hit
Select seq gb|EU735059.1|	Thymus trautvetteri 18S ribosomal RNA gene, partial sequence; internal transcribed spacer 1, 5.8S ribosomal RNA gene, and internal transcribed spacer 2, complete sequence; and 28S ribosomal RNA gene, partial sequence	1267	1267	92%	0.0	100%	EU735059.1	1377,17	100,0%
Select seq gb|EU735058.1|	Thymus persicus isolate Ardabil 18S ribosomal RNA gene, partial sequence; internal transcribed spacer 1, 5.8S ribosomal RNA gene, and internal transcribed spacer 2, complete sequence; and 28S ribosomal RNA gene, partial sequence	1267	1267	92%	0.0	100%	EU735058.1	1377,17	100,0%
Select seq gb|EU374715.1|	Thymus pubescens 18S ribosomal RNA gene, partial sequence; internal transcribed spacer 1, 5.8S ribosomal RNA gene, and internal transcribed spacer 2, complete sequence; and 25/28S ribosomal RNA gene, partial sequence	1142	1142	83%	0.0	100%	EU374715.1	1375,90	99,9%
Select seq gb|AY443448.1|	Thymus magnus JUNG-TM04 internal transcribed spacer 1, 5.8S ribosomal RNA and internal transcribed spcer 2 genes, complete sequence	1131	1131	83%	0.0	99%	AY443448.1	1349,02	98,0%
Select seq gb|AY443434.1|	Thymus quinquecostatus JUNG-TQ01 internal transcribed spacer 1, 5.8S ribosomal RNA and internal transcribed spcer 2 genes, complete sequence	1131	1131	83%	0.0	99%	AY443434.1	1349,02	98,0%
Select seq gb|EU556520.1|	Thymus quinquecostatus isolate XZ internal transcribed spacer 1, partial sequence; 5.8S ribosomal RNA gene, complete sequence; and internal transcribed spacer 2, partial sequence	1240	1240	91%	0.0	99%	EU556520.1	1349,01	98,0%
Select seq gb|EU556524.1|	Thymus quinquecostatus isolate ZY internal transcribed spacer 1, partial sequence; 5.8S ribosomal RNA gene, complete sequence; and internal transcribed spacer 2, partial sequence	1251	1251	92%	0.0	99%	EU556524.1	1346,18	97,7%
Select seq gb|EU556511.1|	Thymus dahuricus isolate HD internal transcribed spacer 1, partial sequence; 5.8S ribosomal RNA gene, complete sequence; and internal transcribed spacer 2, partial sequence	1251	1251	92%	0.0	99%	EU556511.1	1346,18	97,7%
Select seq gb|EU556522.1|	Thymus quinquecostatus isolate ZJ internal transcribed spacer 1, partial sequence; 5.8S ribosomal RNA gene, complete sequence; and internal transcribed spacer 2, partial sequence	1249	1249	92%	0.0	99%	EU556522.1	1344,03	97,6%
Select seq gb|DQ667242.1|	Thymus serpyllum isolate x075 18S ribosomal RNA gene, partial sequence; internal transcribed spacer 1, 5.8S ribosomal RNA gene, and internal transcribed spacer 2, complete sequence; and 28S ribosomal RNA gene, partial sequence	1288	1288	95%	0.0	99%	DQ667242.1	1342,23	97,5%
Select seq gb|AY443445.1|	Thymus magnus JUNG-TM01 internal transcribed spacer 1, 5.8S ribosomal RNA and internal transcribed spcer 2 genes, complete sequence	1125	1125	83%	0.0	99%	AY443445.1	1341,87	97,4%
Select seq gb|FJ236468.1|	Thymus persicus isolate Tabriz 18S ribosomal RNA gene, partial sequence; internal transcribed spacer 1, 5.8S ribosomal RNA gene, and internal transcribed spacer 2, complete sequence; and 28S ribosomal RNA gene, partial sequence	1260	1260	93%	0.0	99%	FJ236468.1	1341,29	97,4%
Select seq gb|EU556510.1|	Thymus mongolicus isolate G3 internal transcribed spacer 1, partial sequence; 5.8S ribosomal RNA gene, complete sequence; and internal transcribed spacer 2, partial sequence	1138	1138	84%	0.0	99%	EU556510.1	1341,21	97,4%
Select seq gb|EU556518.1|	Thymus mongolicus isolate S1 internal transcribed spacer 1, partial sequence; 5.8S ribosomal RNA gene, complete sequence; and internal transcribed spacer 2, partial sequence	1192	1192	88%	0.0	99%	EU556518.1	1341,00	97,4%
Select seq gb|EU556517.1|	Thymus quinquecostatus isolate N2 internal transcribed spacer 1, partial sequence; 5.8S ribosomal RNA gene, complete sequence; and internal transcribed spacer 2, partial sequence	1219	1219	90%	0.0	99%	EU556517.1	1340,90	97,4%
Select seq gb|GU381459.1|	Thymus serpyllum voucher M:Braeuchler 2514 internal transcribed spacer 1, partial sequence; 5.8S ribosomal RNA gene, complete sequence; and internal transcribed spacer 2, partial sequence	1110	1110	82%	0.0	99%	GU381459.1	1340,12	97,3%
Select seq gb|EU556507.1|	Thymus quinquecostatus isolate CL internal transcribed spacer 1, partial sequence; 5.8S ribosomal RNA gene, complete sequence; and internal transcribed spacer 2, partial sequence	1245	1245	92%	0.0	99%	EU556507.1	1339,73	97,3%
Select seq gb|AY029168.1|	Thymus mastichina internal transcribed spacer 1, 5.8S ribosomal RNA gene, and internal transcribed spacer 2, complete sequence	1123	1123	83%	0.0	99%	AY029168.1	1339,48	97,3%
Select seq gb|EU556506.1|	Thymus quinquecostatus isolate D1 internal transcribed spacer 1, partial sequence; 5.8S ribosomal RNA gene, complete sequence; and internal transcribed spacer 2, partial sequence	1203	1203	89%	0.0	99%	EU556506.1	1338,17	97,2%
Select seq gb|EU556519.1|	Thymus mongolicus isolate S2 internal transcribed spacer 1, partial sequence; 5.8S ribosomal RNA gene, complete sequence; and internal transcribed spacer 2, partial sequence	1188	1188	88%	0.0	99%	EU556519.1	1336,50	97,0%
Select seq gb|AY443447.1|	Thymus magnus JUNG-TM03 internal transcribed spacer 1, 5.8S ribosomal RNA and internal transcribed spcer 2 genes, complete sequence	1120	1120	83%	0.0	99%	AY443447.1	1335,90	97,0%
Select seq gb|AY443438.1|	Thymus quinquecostatus JUNG-TQ05 internal transcribed spacer 1, 5.8S ribosomal RNA and internal transcribed spcer 2 genes, complete sequence	1120	1120	83%	0.0	99%	AY443438.1	1335,90	97,0%
Select seq gb|EU556521.1|	Thymus mongolicus isolate XM internal transcribed spacer 1, partial sequence; 5.8S ribosomal RNA gene, complete sequence; and internal transcribed spacer 2, partial sequence	1133	1133	84%	0.0	99%	EU556521.1	1335,32	97,0%
Select seq gb|JQ669138.1|	Thymus pulegioides voucher Riina 1577 18S ribosomal RNA gene, internal transcribed spacer 1, 5.8S ribosomal RNA gene, internal transcribed spacer 2, and 26S ribosomal RNA gene, region	1254	1254	93%	0.0	99%	JQ669138.1	1334,90	96,9%
Select seq gb|EU556509.1|	Thymus mongolicus isolate G2 internal transcribed spacer 1, partial sequence; 5.8S ribosomal RNA gene, complete sequence; and internal transcribed spacer 2, partial sequence	1186	1186	88%	0.0	99%	EU556509.1	1334,25	96,9%
Select seq gb|EU556516.1|	Thymus quinquecostatus isolate N1 internal transcribed spacer 1, partial sequence; 5.8S ribosomal RNA gene, complete sequence; and internal transcribed spacer 2, partial sequence	1197	1197	89%	0.0	99%	EU556516.1	1331,49	96,7%

Select seq gb|EU556508.1|	Thymus mongolicus isolate G1 internal transcribed spacer 1, partial sequence; 5.8S ribosomal RNA gene, complete sequence; and internal transcribed spacer 2, partial sequence	1170	1170	87% 0.0	99% EU556508.1	1331,38	96,7%
Select seq gb|AY443444.1|	Thymus quinquecostatus JUNG-TQ11 internal transcribed spacer 1, 5.8S ribosomal RNA and internal transcribed spcer 2 genes, complete sequence	1114	1114	83% 0.0	99% AY443444.1	1328,75	96,5%
Select seq gb|AY443443.1|	Thymus quinquecostatus JUNG-TQ10 internal transcribed spacer 1, 5.8S ribosomal RNA and internal transcribed spcer 2 genes, complete sequence	1114	1114	83% 0.0	99% AY443443.1	1328,75	96,5%
Select seq gb|AY443442.1|	Thymus quinquecostatus JUNG-TQ09 internal transcribed spacer 1, 5.8S ribosomal RNA and internal transcribed spcer 2 genes, complete sequence	1114	1114	83% 0.0	99% AY443442.1	1328,75	96,5%
Select seq gb|AY443441.1|	Thymus quinquecostatus JUNG-TQ08 internal transcribed spacer 1, 5.8S ribosomal RNA and internal transcribed spcer 2 genes, complete sequence	1114	1114	83% 0.0	99% AY443441.1	1328,75	96,5%
Select seq gb|AY443437.1|	Thymus quinquecostatus JUNG-TQ04 internal transcribed spacer 1, 5.8S ribosomal RNA and internal transcribed spcer 2 genes, complete sequence	1114	1114	83% 0.0	99% AY443437.1	1328,75	96,5%
Select seq gb|AY443436.1|	Thymus quinquecostatus JUNG-TQ03 internal transcribed spacer 1, 5.8S ribosomal RNA and internal transcribed spcer 2 genes, complete sequence	1114	1114	83% 0.0	99% AY443436.1	1328,75	96,5%
Select seq gb|EU796890.1|	Thymus serpyllum 18S ribosomal RNA gene, partial sequence; internal transcribed spacer 1, 5.8S ribosomal RNA gene, and internal transcribed spacer 2, complete sequence; and 26S ribosomal RNA gene, partial sequence	1206	1206	90% 0.0	99% EU796890.1	1326,60	96,3%
Select seq gb|EU785939.1|	Thymus vulgaris 18S ribosomal RNA gene, partial sequence; internal transcribed spacer 1, 5.8S ribosomal RNA gene, and internal transcribed spacer 2, complete sequence; and 26S ribosomal RNA gene, partial sequence	1203	1203	90% 0.0	99% EU785939.1	1323,30	96,1%
Select seq gb|AY443446.1|	Thymus magnus JUNG-TM02 internal transcribed spacer 1, 5.8S ribosomal RNA and internal transcribed spcer 2 genes, complete sequence	1103	1103	83% 0.0	99% AY443446.1	1315,63	95,5%
Select seq gb|AY443440.1|	Thymus quinquecostatus JUNG-TQ07 internal transcribed spacer 1, 5.8S ribosomal RNA and internal transcribed spcer 2 genes, complete sequence	1103	1103	83% 0.0	99% AY443440.1	1315,63	95,5%
Select seq gb|EU556523.1|	Thymus marschallianus isolate XY internal transcribed spacer 1, partial sequence; 5.8S ribosomal RNA gene, complete sequence; and internal transcribed spacer 2, partial sequence	1192	1192	90% 0.0	99% EU556523.1	1311,20	95,2%
Select seq gb|GU381458.1|	Thymus broussonetii subsp. hannonis voucher MSB:Podlech 142 internal transcribed spacer 1, partial sequence; 5.8S ribosomal RNA gene, complete sequence; and internal transcribed spacer 2, partial sequence	1086	1086	82% 0.0	99% GU381458.1	1311,15	95,2%
Select seq gb|AY443435.1|	Thymus quinquecostatus JUNG-TQ02 internal transcribed spacer 1, 5.8S ribosomal RNA and internal transcribed spcer 2 genes, complete sequence	1092	1092	83% 0.0	98% AY443435.1	1289,35	93,6%
Select seq gb|EU556512.1|	Thymus mandschuricus isolate HM internal transcribed spacer 1, partial sequence; 5.8S ribosomal RNA gene, complete sequence; and internal transcribed spacer 2, partial sequence	1179	1179	92% 0.0	98% EU556512.1	1255,89	91,2%
Select seq gb|AY329369.1|	Thymus vulgaris internal transcribed spacer 1, partial sequence; 5.8S ribosomal RNA gene, complete sequence; and internal transcribed spacer 2, partial sequence	1098	1098	86% 0.0	97% AY329369.1	1238,44	89,9%
Select seq gb|AY506646.1|	Thymus vulgaris internal transcribed spacer 1, partial sequence; 5.8S ribosomal RNA gene, complete sequence; and internal transcribed spacer 2, partial sequence	1092	1092	86% 0.0	97% AY506646.1	1231,67	89,4%
Select seq gb|EU556515.1|	Thymus amurensis isolate HW internal transcribed spacer 1, partial sequence; 5.8S ribosomal RNA gene, complete sequence; and internal transcribed spacer 2, partial sequence	1142	1142	92% 0.0	97% EU556515.1	1204,07	87,4%
Select seq gb|EU556514.1|	Thymus quinquecostatus isolate HY internal transcribed spacer 1, partial sequence; 5.8S ribosomal RNA gene, complete sequence; and internal transcribed spacer 2, partial sequence	1026	1026	83% 0.0	97% EU556514.1	1199,06	87,1%
Select seq gb|EU556513.1|	Thymus przewalskii isolate HX internal transcribed spacer 1, partial sequence; 5.8S ribosomal RNA gene, complete sequence; and internal transcribed spacer 2, partial sequence	1033	1033	86% 0.0	96% EU556513.1	1153,12	83,7%
Select seq gb|GU381460.1|	Thymus saturejoides subsp. commutatus voucher M:Podlech 47906 internal transcribed spacer 1, partial sequence; 5.8S ribosomal RNA gene, complete sequence; and internal transcribed spacer 2, partial sequence	965	965	82% 0.0	95% GU381460.1	1117,99	81,2%
Select seq gb|GU381462.1|	Saccocalyx saturejoides voucher MSB:Faurel 5650 internal transcribed spacer 1, partial sequence; 5.8S ribosomal RNA gene, complete sequence; and internal transcribed spacer 2, partial sequence	953	953	82% 0.0	95% GU381462.1	1104,09	80,2%
Select seq gb|GU381457.1|	Thymus caespititius voucher M:Heubl s.n. internal transcribed spacer 1, partial sequence; 5.8S ribosomal RNA gene, complete sequence; and internal transcribed spacer 2, partial sequence	953	953	82% 0.0	95% GU381457.1	1104,09	80,2%
Select seq gb|GU381461.1|	Argantoniella salzmannii voucher M:Barra et al. 2673GL internal transcribed spacer 1, partial sequence; 5.8S ribosomal RNA gene, complete sequence; and internal transcribed spacer 2, partial sequence	948	948	82% 0.0	95% GU381461.1	1098,29	79,7%
Select seq gb|JQ669137.1|	Thymbra capitata voucher UCBG 96.0817 s.n. 18S ribosomal RNA gene, internal transcribed spacer 1, 5.8S ribosomal RNA gene, internal transcribed spacer 2, and 26S ribosomal RNA gene, region	1053	1053	93% 0.0	94% JQ669137.1	1064,32	77,3%
Select seq gb|GU381451.1|	Thymbra sintenisii subsp. isaurica voucher E:Goener 12628 internal transcribed spacer 1, partial sequence; 5.8S ribosomal RNA gene, complete sequence; and internal transcribed spacer 2, partial sequence	915	915	81% 0.0	94% GU381451.1	1061,85	77,1%
Select seq gb|GU381452.1|	Thymbra calostachya voucher M:Ulrich s.n. internal transcribed spacer 1, partial sequence; 5.8S ribosomal RNA gene, complete sequence; and internal transcribed spacer 2, partial sequence	909	909	81% 0.0	94% GU381452.1	1054,89	76,6%
Select seq gb|GU381455.1|	Satureja linearifolia voucher M:Brullo & Furnari s.n. internal transcribed spacer 1, partial sequence; 5.8S ribosomal RNA gene, complete sequence; and internal transcribed spacer 2, partial sequence	907	907	81% 0.0	94% GU381455.1	1052,57	76,4%

Select seq gb|JQ669127.1|	Origanum vulgare voucher B. Drew 77 18S ribosomal RNA gene, internal transcribed spacer 1, 5.8S ribosomal RNA gene, internal transcribed spacer 2, and 26S ribosomal RNA gene, region	1020	1020	93%	0.0	93%	JQ669127.1	1020,00	74,1%
Select seq gb|DQ667243.1|	Origanum vulgare isolate x076 18S ribosomal RNA gene, partial sequence; internal transcribed spacer 1, 5.8S ribosomal RNA gene, and internal transcribed spacer 2, complete sequence; and 28S ribosomal RNA gene, partial sequence	1018	1018	94%	0.0	93%	DQ667243.1	1007,17	73,1%
Select seq gb|AY506647.1|	Origanum vulgare internal transcribed spacer 1, partial sequence; 5.8S ribosomal RNA gene, complete sequence; and internal transcribed spacer 2, partial sequence	928	928	87%	0.0	92%	AY506647.1	981,33	71,3%
Select seq gb|JX162834.1|	Origanum majorana voucher OBI clone 3 internal transcribed spacer 1, partial sequence; 5.8S ribosomal RNA gene, complete sequence; and internal transcribed spacer 2, partial sequence	917	917	86%	0.0	92%	JX162834.1	980,98	71,2%
Select seq gb|JX162831.1|	Origanum majorana voucher Mira2-3 clone 10 internal transcribed spacer 1, partial sequence; 5.8S ribosomal RNA gene, complete sequence; and internal transcribed spacer 2, partial sequence	917	917	86%	0.0	92%	JX162831.1	980,98	71,2%
Select seq gb|JX163036.1|	Origanum syriacum voucher LC7 clone 8 internal transcribed spacer 1, partial sequence; 5.8S ribosomal RNA gene, complete sequence; and internal transcribed spacer 2, partial sequence	913	913	86%	0.0	92%	JX163036.1	976,70	70,9%
Select seq gb|JX162836.1|	Origanum majorana voucher OBI clone 5 internal transcribed spacer 1, partial sequence; 5.8S ribosomal RNA gene, complete sequence; and internal transcribed spacer 2, partial sequence	913	913	86%	0.0	92%	JX162836.1	976,70	70,9%
Select seq gb|JX162980.1|	Origanum onites voucher SR777 clone 14 internal transcribed spacer 1, partial sequence; 5.8S ribosomal RNA gene, complete sequence; and internal transcribed spacer 2, partial sequence	911	911	86%	0.0	92%	JX162980.1	974,56	70,8%
Select seq gb|JX162837.1|	Origanum majorana voucher OBI clone 6 internal transcribed spacer 1, partial sequence; 5.8S ribosomal RNA gene, complete sequence; and internal transcribed spacer 2, partial sequence	911	911	86%	0.0	92%	JX162837.1	974,56	70,8%
Select seq gb|JX162826.1|	Origanum majorana voucher Mira2-3 clone 4 internal transcribed spacer 1, partial sequence; 5.8S ribosomal RNA gene, complete sequence; and internal transcribed spacer 2, partial sequence	911	911	86%	0.0	92%	JX162826.1	974,56	70,8%
Select seq gb|JX162814.1|	Origanum majorana voucher SR562 clone 1 internal transcribed spacer 1, partial sequence; 5.8S ribosomal RNA gene, complete sequence; and internal transcribed spacer 2, partial sequence	911	911	86%	0.0	92%	JX162814.1	974,56	70,8%
Select seq gb|JX162807.1|	Origanum majorana voucher LC10 clone 4 internal transcribed spacer 1, partial sequence; 5.8S ribosomal RNA gene, complete sequence; and internal transcribed spacer 2, partial sequence	911	911	86%	0.0	92%	JX162807.1	974,56	70,8%
Select seq gb|JX162805.1|	Origanum majorana voucher LC10 clone 2 internal transcribed spacer 1, partial sequence; 5.8S ribosomal RNA gene, complete sequence; and internal transcribed spacer 2, partial sequence	911	911	86%	0.0	92%	JX162805.1	974,56	70,8%
Select seq gb|JX162830.1|	Origanum majorana voucher Mira2-3 clone 9 internal transcribed spacer 1, partial sequence; 5.8S ribosomal RNA gene, complete sequence; and internal transcribed spacer 2, partial sequence	907	907	86%	0.0	92%	JX162830.1	970,28	70,5%
Select seq gb|JX163016.1|	Origanum syriacum voucher RNG:H50 clone 8 internal transcribed spacer 1, partial sequence; 5.8S ribosomal RNA gene, complete sequence; and internal transcribed spacer 2, partial sequence	905	905	86%	0.0	92%	JX163016.1	968,14	70,3%
Select seq gb|JX162978.1|	Origanum onites voucher SR777 clone 11 internal transcribed spacer 1, partial sequence; 5.8S ribosomal RNA gene, complete sequence; and internal transcribed spacer 2, partial sequence	905	905	86%	0.0	92%	JX162978.1	968,14	70,3%
Select seq gb|JX162966.1|	Origanum onites voucher SR530 clone 8 internal transcribed spacer 1, partial sequence; 5.8S ribosomal RNA gene, complete sequence; and internal transcribed spacer 2, partial sequence	905	905	86%	0.0	92%	JX162966.1	968,14	70,3%
Select seq gb|JX162832.1|	Origanum majorana voucher OBI clone 1 internal transcribed spacer 1, partial sequence; 5.8S ribosomal RNA gene, complete sequence; and internal transcribed spacer 2, partial sequence	905	905	86%	0.0	92%	JX162832.1	968,14	70,3%
Select seq gb|JX162829.1|	Origanum majorana voucher Mira2-3 clone 8 internal transcribed spacer 1, partial sequence; 5.8S ribosomal RNA gene, complete sequence; and internal transcribed spacer 2, partial sequence	905	905	86%	0.0	92%	JX162829.1	968,14	70,3%
Select seq gb|JX162823.1|	Origanum majorana voucher Mira2-3 clone 1 internal transcribed spacer 1, partial sequence; 5.8S ribosomal RNA gene, complete sequence; and internal transcribed spacer 2, partial sequence	905	905	86%	0.0	92%	JX162823.1	968,14	70,3%
Select seq gb|JX162818.1|	Origanum majorana voucher SR562 clone 5 internal transcribed spacer 1, partial sequence; 5.8S ribosomal RNA gene, complete sequence; and internal transcribed spacer 2, partial sequence	905	905	86%	0.0	92%	JX162818.1	968,14	70,3%
Select seq gb|JX162816.1|	Origanum majorana voucher SR562 clone 3 internal transcribed spacer 1, partial sequence; 5.8S ribosomal RNA gene, complete sequence; and internal transcribed spacer 2, partial sequence	905	905	86%	0.0	92%	JX162816.1	968,14	70,3%
Select seq gb|JX162815.1|	Origanum majorana voucher SR562 clone 2 internal transcribed spacer 1, partial sequence; 5.8S ribosomal RNA gene, complete sequence; and internal transcribed spacer 2, partial sequence	905	905	86%	0.0	92%	JX162815.1	968,14	70,3%
Select seq gb|JX163024.1|	Origanum syriacum voucher RNG:H50 clone 16 internal transcribed spacer 1, partial sequence; 5.8S ribosomal RNA gene, complete sequence; and internal transcribed spacer 2, partial sequence	902	902	86%	0.0	92%	JX163024.1	964,93	70,1%
Select seq gb|JX163022.1|	Origanum syriacum voucher RNG:H50 clone 14 internal transcribed spacer 1, partial sequence; 5.8S ribosomal RNA gene, complete sequence; and internal transcribed spacer 2, partial sequence	902	902	86%	0.0	92%	JX163022.1	964,93	70,1%
Select seq gb|JX163021.1|	Origanum syriacum voucher RNG:H50 clone 13 internal transcribed spacer 1, partial sequence; 5.8S ribosomal RNA gene, complete sequence; and internal transcribed spacer 2, partial sequence	902	902	86%	0.0	92%	JX163021.1	964,93	70,1%
Select seq gb|JX163014.1|	Origanum syriacum voucher RNG:H50 clone 6 internal transcribed spacer 1, partial sequence; 5.8S ribosomal RNA gene, complete sequence; and internal transcribed spacer 2, partial sequence	902	902	86%	0.0	92%	JX163014.1	964,93	70,1%
Select seq gb|JX163010.1|	Origanum syriacum voucher RNG:H50 clone 2 internal transcribed spacer 1, partial sequence; 5.8S ribosomal RNA gene, complete sequence; and internal transcribed spacer 2, partial sequence	902	902	86%	0.0	92%	JX163010.1	964,93	70,1%

Select seq gb|JX163004.1|	Origanum onites voucher SR1372 clone 8 internal transcribed spacer 1, partial sequence; 5.8S ribosomal RNA gene, complete sequence; and internal transcribed spacer 2, partial sequence	902	902	86%	0.0	92%	JX163004.1	964,93	70,1%
Select seq gb|JX163019.1|	Origanum syriacum voucher RNG:H50 clone 11 internal transcribed spacer 1, partial sequence; 5.8S ribosomal RNA gene, complete sequence; and internal transcribed spacer 2, partial sequence	900	900	86%	0.0	92%	JX163019.1	962,79	69,9%
Select seq gb|JX163018.1|	Origanum syriacum voucher RNG:H50 clone 10 internal transcribed spacer 1, partial sequence; 5.8S ribosomal RNA gene, complete sequence; and internal transcribed spacer 2, partial sequence	900	900	86%	0.0	92%	JX163018.1	962,79	69,9%
Select seq gb|JX163017.1|	Origanum syriacum voucher RNG:H50 clone 9 internal transcribed spacer 1, partial sequence; 5.8S ribosomal RNA gene, complete sequence; and internal transcribed spacer 2, partial sequence	900	900	86%	0.0	92%	JX163017.1	962,79	69,9%
Select seq gb|JX163054.1|	Origanum onites voucher SR810 internal transcribed spacer 1, partial sequence; 5.8S ribosomal RNA gene, complete sequence; and internal transcribed spacer 2, partial sequence	891	891	86%	0.0	91%	JX163054.1	942,80	68,5%
Select seq gb|JQ669139.1|	Zataria multiflora voucher Rechinger 51885 18S ribosomal RNA gene, internal transcribed spacer 1, 5.8S ribosomal RNA gene, internal transcribed spacer 2, and 26S ribosomal RNA gene, region	928	928	93%	0.0	91%	JQ669139.1	908,04	65,9%
Select seq gb|DQ667333.1|	Acanthomintha lanceolata isolate x545 18S ribosomal RNA gene, partial sequence; internal transcribed spacer 1, 5.8S ribosomal RNA gene, and internal transcribed spacer 2, complete sequence; and 28S ribosomal RNA gene, partial sequence	950	950	96%	0.0	91%	DQ667333.1	900,52	65,4%
Select seq gb|DQ667311.1|	Poliomintha palmeri isolate x259 18S ribosomal RNA gene, partial sequence; internal transcribed spacer 1, 5.8S ribosomal RNA gene, and internal transcribed spacer 2, complete sequence; and 28S ribosomal RNA gene, partial sequence	907	907	97%	0.0	89%	DQ667311.1	832,20	60,4%
Select seq gb|DQ667310.1|	Glechon thymoides isolate x256 18S ribosomal RNA gene, partial sequence; internal transcribed spacer 1, 5.8S ribosomal RNA gene, and internal transcribed spacer 2, complete sequence; and 28S ribosomal RNA gene, partial sequence	907	907	97%	0.0	89%	DQ667310.1	832,20	60,4%
Select seq gb|DQ667305.1|	Cunila microcephala isolate x226 18S ribosomal RNA gene, partial sequence; internal transcribed spacer 1, 5.8S ribosomal RNA gene, and internal transcribed spacer 2, complete sequence; and 28S ribosomal RNA gene, partial sequence	907	907	97%	0.0	89%	DQ667305.1	832,20	60,4%
Select seq gb|DQ667303.1|	Glechon marifolia isolate x223 18S ribosomal RNA gene, partial sequence; internal transcribed spacer 1, 5.8S ribosomal RNA gene, and internal transcribed spacer 2, complete sequence; and 28S ribosomal RNA gene, partial sequence	907	907	97%	0.0	89%	DQ667303.1	832,20	60,4%
Select seq gb|DQ667244.1|	Mentha spicata isolate x077 18S ribosomal RNA gene, partial sequence; internal transcribed spacer 1, 5.8S ribosomal RNA gene, and internal transcribed spacer 2, complete sequence; and 28S ribosomal RNA gene, partial sequence	905	905	97%	0.0	89%	DQ667244.1	830,36	60,3%
Select seq gb|DQ667325.1|	Mentha arvensis isolate x424 18S ribosomal RNA gene, partial sequence; internal transcribed spacer 1, 5.8S ribosomal RNA gene, and internal transcribed spacer 2, complete sequence; and 28S ribosomal RNA gene, partial sequence	902	902	97%	0.0	89%	DQ667325.1	827,61	60,1%
								0,00	0,0%
								0,00	
								0,00	
								0,00	

Select for downloading or viewing reports	Kh057_trnL Description	Max score	Total score	Query cover	E value	Ident	Accession	(Ident/Cover)* Max score	Deviation from top hit
Select seq gb|JX880022.1|	Origanum vulgare subsp. vulgare chloroplast, complete genome	294	294	80%	1,00E-75	76%	JX880022.1	279,30	100,0%
Select seq gb|JQ690290.1|	Origanum elongatum isolate H5_O_elo trnL-trnF intergenic spacer, partial sequence; chloroplast	294	294	80%	1,00E-75	76%	JQ690290.1	279,30	100,0%
Select seq gb|JQ690289.1|	Origanum rotundifolium isolate DNA3_O_rot trnL-trnF intergenic spacer, partial sequence; chloroplast	294	294	80%	1,00E-75	76%	JQ690289.1	279,30	100,0%
Select seq gb|GU381633.1|	Thymus caespititius voucher M:Heubl s.n. tRNA-Leu (trnL) gene and trnL-trnF intergenic spacer, partial sequence; chloroplast	294	294	80%	1,00E-75	76%	GU381633.1	279,30	100,0%
Select seq gb|AY570463.1|	Origanum vulgare voucher JBW 2567 tRNA-Leu and trnL-trnF intergenic spacer, partial sequence; chloroplast	294	294	80%	1,00E-75	76%	AY570463.1	279,30	100,0%
Select seq emb|AJ505543.1|	Origanum vulgare plastid trnL-trnF intergenic spacer, specimen voucher cult., K-000-69-19317, chase 13334 (K)	294	294	80%	1,00E-75	76%	AJ505543.1	279,30	100,0%
Select seq gb|AY840202.1|	Origanum vulgare tRNA-Leu (trnL) gene and trnL-trnF intergenic spacer, partial sequence; chloroplast	294	294	80%	1,00E-75	76%	AY840202.1	279,30	100,0%
Select seq gb|JQ690293.1|	Origanum dayi isolate H43_O_day trnL-trnF intergenic spacer, partial sequence; chloroplast	289	289	80%	7,00E-74	76%	JQ690293.1	274,55	98,3%
Select seq gb|GU381640.1|	Argantoniella salzmannii voucher M:Barra et al. 2673GL tRNA-Leu (trnL) gene and trnL-trnF intergenic spacer, partial sequence; chloroplast	289	289	80%	7,00E-74	76%	GU381640.1	274,55	98,3%
Select seq gb|JF301392.1|	Origanum vulgare voucher B. Drew 77 tRNA-Leu (trnL) gene and trnL-trnF intergenic spacer, partial sequence; chloroplast	287	287	80%	2,00E-73	76%	JF301392.1	272,65	97,6%
Select seq gb|AY506614.1|	Origanum vulgare tRNA-Leu (trnL) gene and trnL-trnF intergenic spacer, partial sequence; chloroplast	287	287	80%	2,00E-73	76%	AY506614.1	272,65	97,6%
Select seq gb|GU381567.1|	Hesperozygis nitida voucher M:Coudeiro & Ribes 1368 tRNA-Leu (trnL) gene and trnL-trnF intergenic spacer, partial sequence; chloroplast	222	222	80%	7,00E-54	74%	GU381567.1	205,35	73,5%
Select seq gb|AF350668.1|	Hofmeisterella eumicroscopica tRNA-Leu (trnL) gene and trnL-trnF intergenic spacer, partial sequence; chloroplast	52,8	52.8	3%	0.010	100%	AF350668.1	0,00	0,0%
Select seq gb|HM639301.1|	Saniella occidentalis tRNA-Leu (trnL) gene, partial sequence; trnL-trnF intergenic spacer, complete sequence; and tRNA-Phe (trnF) gene, partial sequence; chloroplast	67,6	67.6	4%	3,00E-07	100%	HM639301.1	0,00	0,0%
Select seq gb|AY424780.1|	Camptandra parvula tRNA-Leu (trnL) gene, partial sequence; trnL-trnF intergenic spacer, complete sequence; and tRNA-Phe (trnF) gene, partial sequence; chloroplast	63,9	63.9	4%	5,00E-06	100%	AY424780.1	0,00	0,0%
Select seq gb|EU647818.1|	Hypselodelphys velutina voucher Jongkind 4839 (WAG) tRNA-Leu (trnL) gene, partial sequence; trnL-trnF intergenic spacer, complete sequence; and tRNA-Phe (trnF) gene, partial sequence	73,1	73.1	5%	7,00E-09	98%	EU647818.1	0,00	0,0%
Select seq gb|GQ475230.1|	Paepalanthus viridulus tRNA-Leu (trnL) gene, partial sequence; trnL-trnF intergenic spacer, complete sequence; and tRNA-Phe (trnF) gene, partial sequence; chloroplast	71,3	71.3	5%	3,00E-08	96%	GQ475230.1	0,00	0,0%
Select seq gb|GQ475215.1|	Paepalanthus fulgidus tRNA-Leu (trnL) gene, partial sequence; trnL-trnF intergenic spacer, complete sequence; and tRNA-Phe (trnF) gene, partial sequence; chloroplast	71,3	71.3	5%	3,00E-08	96%	GQ475215.1	0,00	0,0%
Select seq gb|GQ475206.1|	Paepalanthus bonsai tRNA-Leu (trnL) gene, partial sequence; trnL-trnF intergenic spacer, complete sequence; and tRNA-Phe (trnF) gene, partial sequence; chloroplast	71,3	71.3	5%	3,00E-08	96%	GQ475206.1	0,00	0,0%
Select seq gb|HQ843087.1|	Paepalanthus sp. MJGA-2011 voucher MJG Andrade 625 (HUEFS) tRNA-Leu (trnL) gene, partial sequence; trnL-trnF intergenic spacer, complete sequence; and tRNA-Phe (trnF) gene, partial sequence; chloroplast	71,3	71.3	5%	3,00E-08	96%	HQ843087.1	0,00	0,0%
Select seq gb|AY237886.1|	Dupontia fisheri voucher 00-5-2 tRNA-Leu (trnL) gene, partial sequence; trnL-trnF intergenic spacer, complete sequence; and tRNA-Phe (trnF) gene, partial sequence; chloroplast	69,4	69.4	5%	1,00E-07	98%	AY237886.1	0,00	0,0%
Select seq gb|AF239556.1|AF239556	Kegeliella kupperi tRNA-Leu (trnL) gene, partial sequence; and trnL-F intergenic spacer region, complete sequence; chloroplast gene for chloroplast product	67,6	67.6	5%	3,00E-07	98%	AF239556.1	0,00	0,0%
Select seq gb|EU581025.1|	Margaranthus solanaceus voucher Olmstead S-37 (WTU) tRNA-Leu (trnL) gene and trnL-trnF intergenic spacer, partial sequence; chloroplast	67,6	67.6	5%	3,00E-07	95%	EU581025.1	0,00	0,0%
Select seq gb|GQ475222.1|	Paepalanthus mollis tRNA-Leu (trnL) gene, partial sequence; trnL-trnF intergenic spacer, complete sequence; and tRNA-Phe (trnF) gene, partial sequence; chloroplast	75	75.0	6%	2,00E-09	96%	GQ475222.1	0,00	0,0%
Select seq gb|EU395883.1|	Briza calotheca voucher ICN<BRA>:98.925 tRNA-Leu (trnL) gene, partial sequence; trnL-trnF intergenic spacer, complete sequence; and tRNA-Phe (trnF) gene, partial sequence; chloroplast	67,6	67.6	6%	3,00E-07	91%	EU395883.1	0,00	0,0%
Select seq gb|GQ475213.1|	Paepalanthus distichophyllus tRNA-Leu (trnL) gene, partial sequence; trnL-trnF intergenic spacer, complete sequence; and tRNA-Phe (trnF) gene, partial sequence; chloroplast	71,3	71.3	7%	3,00E-08	91%	GQ475213.1	0,00	0,0%

Select seq gb|GU381639.1|	Thymus saturejoides subsp. commutatus voucher M:Podlech 47906 tRNA-Leu (trnL) gene and trnL-trnF intergenic spacer, partial sequence; chloroplast	187	187	36%	3,00E-43	80%	GU381639.1	0,00	0,0%
								0,00	0,0%
								0,00	0,0%
								0,00	0,0%
								0,00	0,0%
								0,00	0,0%
								0,00	0,0%
								0,00	0,0%
								0,00	0,0%
								0,00	0,0%
								0,00	0,0%
								0,00	0,0%
								0,00	0,0%
								0,00	0,0%
								0,00	0,0%
								0,00	0,0%
								0,00	0,0%
								0,00	0,0%
								0,00	0,0%
								0,00	0,0%
								0,00	0,0%
								0,00	0,0%
								0,00	0,0%
								0,00	0,0%
								0,00	0,0%
								0,00	0,0%
								0,00	0,0%
								0,00	0,0%
								0,00	0,0%
								0,00	0,0%
								0,00	0,0%
								0,00	0,0%
								0,00	0,0%
								0,00	0,0%
								0,00	0,0%
								0,00	0,0%
								0,00	0,0%
								0,00	0,0%
								0,00	0,0%
								0,00	0,0%
								0,00	0,0%
								0,00	0,0%
								0,00	0,0%
								0,00	0,0%
								0,00	0,0%
								0,00	0,0%
								0,00	0,0%
								0,00	0,0%
								0,00	0,0%
								0,00	0,0%
								0,00	0,0%
								0,00	0,0%
								0,00	0,0%
								0,00	0,0%
								0,00	0,0%

0,00	0,0%
0,00	0,0%
0,00	0,0%
0,00	0,0%
0,00	0,0%
0,00	0,0%
0,00	0,0%
0,00	0,0%
0,00	0,0%
0,00	0,0%
0,00	0,0%
0,00	0,0%
0,00	0,0%
0,00	0,0%
0,00	0,0%
0,00	0,0%
0,00	0,0%
0,00	0,0%

Select for downloading or viewing reports	Kh058_ITS Description	Max score	Total score	Query cover	E value	Ident	Accession	(Ident/Cover)* Max score	Deviation from top hit
Select seq gb|AY603242.1|	Achillea pseudopectinata internal transcribed spacer 1, 5.8S ribosomal RNA gene, and internal transcribed spacer 2, complete sequence	1131	1131	96%	0.0	99%	AY603242.1	1166,34	100,0%
Select seq gb|AY603218.1|	Achillea biebersteinii internal transcribed spacer 1, 5.8S ribosomal RNA gene, and internal transcribed spacer 2, complete sequence	1131	1131	96%	0.0	99%	AY603218.1	1166,34	100,0%
Select seq gb|AY603220.1|	Achillea leptophylla internal transcribed spacer 1, 5.8S ribosomal RNA gene, and internal transcribed spacer 2, complete sequence	1118	1118	96%	0.0	99%	AY603220.1	1152,94	98,9%
Select seq gb|AY603245.1|	Achillea chamaemelifolia internal transcribed spacer 1, 5.8S ribosomal RNA gene, and internal transcribed spacer 2, complete sequence	1107	1107	96%	0.0	99%	AY603245.1	1141,59	97,9%
Select seq gb|AY603216.1|	Achillea clypeolata internal transcribed spacer 1, 5.8S ribosomal RNA gene, and internal transcribed spacer 2, complete sequence	1107	1107	96%	0.0	99%	AY603216.1	1141,59	97,9%
Select seq gb|AY603229.1|	Achillea clavennae internal transcribed spacer 1, 5.8S ribosomal RNA gene, and internal transcribed spacer 2, complete sequence	1101	1101	96%	0.0	99%	AY603229.1	1135,41	97,3%
Select seq gb|AY603221.1|	Achillea grandifolia internal transcribed spacer 1, 5.8S ribosomal RNA gene, and internal transcribed spacer 2, complete sequence	1101	1101	96%	0.0	99%	AY603221.1	1135,41	97,3%
Select seq gb|AY603215.1|	Achillea crithmifolia internal transcribed spacer 1, 5.8S ribosomal RNA gene, and internal transcribed spacer 2, complete sequence	1101	1101	96%	0.0	99%	AY603215.1	1135,41	97,3%
Select seq gb|AY603222.1|	Achillea holosericea internal transcribed spacer 1, 5.8S ribosomal RNA gene, and internal transcribed spacer 2, complete sequence	1096	1096	96%	0.0	98%	AY603222.1	1118,83	95,9%
Select seq gb|AY603211.1|	Achillea virescens internal transcribed spacer 1, 5.8S ribosomal RNA gene, and internal transcribed spacer 2, complete sequence	1096	1096	96%	0.0	98%	AY603211.1	1118,83	95,9%
Select seq gb|AY603213.1|	Achillea absinthoides internal transcribed spacer 1, 5.8S ribosomal RNA gene, and internal transcribed spacer 2, complete sequence	1096	1096	96%	0.0	98%	AY603213.1	1118,83	95,9%
Select seq gb|AY603187.1|	Achillea millefolium subsp. sudetica internal transcribed spacer 1, 5.8S ribosomal RNA gene, and internal transcribed spacer 2, complete sequence	1096	1096	96%	0.0	98%	AY603187.1	1118,83	95,9%
Select seq gb|AY603190.1|	Achillea styriaca internal transcribed spacer 1, 5.8S ribosomal RNA gene, and internal transcribed spacer 2, complete sequence	1092	1092	96%	0.0	98%	AY603190.1	1114,75	95,6%
Select seq gb|AY603186.1|	Achillea millefolium internal transcribed spacer 1, 5.8S ribosomal RNA gene, and internal transcribed spacer 2, complete sequence	1092	1092	96%	0.0	98%	AY603186.1	1114,75	95,6%
Select seq gb|KR150185.1|	Achillea sp. Kh17 internal transcribed spacer 1, partial sequence; 5.8S ribosomal RNA gene gene, complete sequence; and internal transcribed spacer 2 region, partial sequence	1114	1114	98%	0.0	98%	KR150185.1	1114,00	95,5%
Select seq gb|AY603212.1|	Achillea nobilis internal transcribed spacer 1, 5.8S ribosomal RNA gene, and internal transcribed spacer 2, complete sequence	1090	1090	96%	0.0	98%	AY603212.1	1112,71	95,4%
Select seq gb|AY603202.1|	Achillea euxina internal transcribed spacer 1, 5.8S ribosomal RNA gene, and internal transcribed spacer 2, complete sequence	1090	1090	96%	0.0	98%	AY603202.1	1112,71	95,4%
Select seq gb|AY603197.1|	Achillea setacea internal transcribed spacer 1, 5.8S ribosomal RNA gene, and internal transcribed spacer 2, complete sequence	1090	1090	96%	0.0	98%	AY603197.1	1112,71	95,4%
Select seq gb|AY603185.1|	Achillea millefolium internal transcribed spacer 1, 5.8S ribosomal RNA gene, and internal transcribed spacer 2, complete sequence	1090	1090	96%	0.0	98%	AY603185.1	1112,71	95,4%
Select seq gb|AY603184.1|	Achillea pannonica internal transcribed spacer 1, 5.8S ribosomal RNA gene, and internal transcribed spacer 2, complete sequence	1090	1090	96%	0.0	98%	AY603184.1	1112,71	95,4%
Select seq gb|EU796891.1|	Achillea millefolium 18S ribosomal RNA gene, partial sequence; internal transcribed spacer 1, 5.8S ribosomal RNA gene, and internal transcribed spacer 2, complete sequence; and 26S ribosomal RNA gene, partial sequence	1123	1123	99%	0.0	98%	EU796891.1	1111,66	95,3%
Select seq gb|AY603228.1|	Achillea clusiana internal transcribed spacer 1, 5.8S ribosomal RNA gene, and internal transcribed spacer 2, complete sequence	1088	1088	96%	0.0	98%	AY603228.1	1110,67	95,2%
Select seq gb|AY603205.1|	Achillea lanulosa subsp. alpicola internal transcribed spacer 1, 5.8S ribosomal RNA gene, and internal transcribed spacer 2, complete sequence	1088	1088	96%	0.0	98%	AY603205.1	1110,67	95,2%
Select seq gb|FJ553518.1|	Angiosperm environmental sample clone LTSP_EUKA_P3K07 18S ribosomal RNA, 18S-25/28S ribosomal RNA intergenic spacer, partial sequence	1120	1120	99%	0.0	98%	FJ553518.1	1108,69	95,1%
Select seq gb|AY603230.1|	Achillea clavennae internal transcribed spacer 1, 5.8S ribosomal RNA gene, and internal transcribed spacer 2, complete sequence	1086	1086	96%	0.0	98%	AY603230.1	1108,63	95,1%
Select seq gb|AY603204.1|	Achillea ceretanica internal transcribed spacer 1, 5.8S ribosomal RNA gene, and internal transcribed spacer 2, complete sequence	1086	1086	96%	0.0	98%	AY603204.1	1108,63	95,1%

Select seq gb|AY603200.1|	Achillea roseoalba internal transcribed spacer 1, 5.8S ribosomal RNA gene, and internal transcribed spacer 2, complete sequence	1086	1086	96%	0.0	98%	AY603200.1	1108,63	95,1%
Select seq gb|AY603195.1|	Achillea setacea internal transcribed spacer 1, 5.8S ribosomal RNA gene, and internal transcribed spacer 2, complete sequence	1086	1086	96%	0.0	98%	AY603195.1	1108,63	95,1%
Select seq gb|AY603207.1|	Achillea wilsoniana internal transcribed spacer 1, 5.8S ribosomal RNA gene, and internal transcribed spacer 2, complete sequence	1085	1085	96%	0.0	98%	AY603207.1	1107,60	95,0%
Select seq gb|AY603203.1|	Achillea ceretanica internal transcribed spacer 1, 5.8S ribosomal RNA gene, and internal transcribed spacer 2, complete sequence	1085	1085	96%	0.0	98%	AY603203.1	1107,60	95,0%
Select seq gb|AY603191.1|	Achillea setacea internal transcribed spacer 1, 5.8S ribosomal RNA gene, and internal transcribed spacer 2, complete sequence	1085	1085	96%	0.0	98%	AY603191.1	1107,60	95,0%
Select seq gb|AY603210.1|	Achillea monticola internal transcribed spacer 1, 5.8S ribosomal RNA gene, and internal transcribed spacer 2, complete sequence	1083	1083	96%	0.0	98%	AY603210.1	1105,56	94,8%
Select seq gb|AY603201.1|	Achillea asplenifolia internal transcribed spacer 1, 5.8S ribosomal RNA gene, and internal transcribed spacer 2, complete sequence	1083	1083	96%	0.0	98%	AY603201.1	1105,56	94,8%
Select seq gb|JQ230971.1|	Achillea millefolium voucher SBB-1156 18S ribosomal RNA gene, partial sequence; internal transcribed spacer 1, 5.8S ribosomal RNA gene, and internal transcribed spacer 2, complete sequence; and 28S ribosomal RNA gene, partial sequence	1114	1114	99%	0.0	98%	JQ230971.1	1102,75	94,5%
Select seq gb|FJ980358.1|	Achillea wilsoniana voucher PS0715MT02 18S ribosomal RNA gene, partial sequence; internal transcribed spacer 1, 5.8S ribosomal RNA gene, and internal transcribed spacer 2, complete sequence; and 28S ribosomal RNA gene, partial sequence	1114	1114	99%	0.0	98%	FJ980358.1	1102,75	94,5%
Select seq gb|AY603232.1|	Achillea fraasii internal transcribed spacer 1, 5.8S ribosomal RNA gene, and internal transcribed spacer 2, complete sequence	1079	1079	96%	0.0	98%	AY603232.1	1101,48	94,4%
Select seq gb|AY603214.1|	Achillea ageratum internal transcribed spacer 1, 5.8S ribosomal RNA gene, and internal transcribed spacer 2, complete sequence	1079	1079	96%	0.0	98%	AY603214.1	1101,48	94,4%
Select seq gb|AY603219.1|	Achillea filipendulina internal transcribed spacer 1, 5.8S ribosomal RNA gene, and internal transcribed spacer 2, complete sequence	1077	1077	96%	0.0	98%	AY603219.1	1099,44	94,3%
Select seq gb|FJ980357.1|	Achillea wilsoniana voucher PS0715MT01 18S ribosomal RNA gene, partial sequence; internal transcribed spacer 1, 5.8S ribosomal RNA gene, and internal transcribed spacer 2, complete sequence; and 28S ribosomal RNA gene, partial sequence	1110	1110	99%	0.0	98%	FJ980357.1	1098,79	94,2%
Select seq gb|AY603223.1|	Achillea micrantha internal transcribed spacer 1, 5.8S ribosomal RNA gene, and internal transcribed spacer 2, complete sequence	1074	1074	96%	0.0	98%	AY603223.1	1096,38	94,0%
Select seq gb|AY603227.1|	Achillea atrata internal transcribed spacer 1, 5.8S ribosomal RNA gene, and internal transcribed spacer 2, complete sequence	1072	1072	96%	0.0	98%	AY603227.1	1094,33	93,8%
Select seq gb|EF577285.1|	Achillea wilsoniana 18S ribosomal RNA gene, partial sequence; internal transcribed spacer 1, 5.8S ribosomal RNA gene, and internal transcribed spacer 2, complete sequence; and 28S ribosomal RNA gene, partial sequence	1103	1103	99%	0.0	98%	EF577285.1	1091,86	93,6%
Select seq gb|AY603240.1|	Achillea schurii internal transcribed spacer 1, 5.8S ribosomal RNA gene, and internal transcribed spacer 2, complete sequence	1062	1062	96%	0.0	97%	AY603240.1	1073,06	92,0%
Select seq gb|AY603238.1|	Achillea oxyloba internal transcribed spacer 1, 5.8S ribosomal RNA gene, and internal transcribed spacer 2, complete sequence	1051	1051	96%	0.0	97%	AY603238.1	1061,95	91,0%
Select seq gb|AY603241.1|	Achillea schurii internal transcribed spacer 1, 5.8S ribosomal RNA gene, and internal transcribed spacer 2, complete sequence	1046	1046	96%	0.0	97%	AY603241.1	1056,90	90,6%
Select seq gb|AY603239.1|	Achillea pindicola subsp. integrifolia internal transcribed spacer 1, 5.8S ribosomal RNA gene, and internal transcribed spacer 2, complete sequence	1046	1046	96%	0.0	97%	AY603239.1	1056,90	90,6%
Select seq gb|AY603249.1|	Achillea ptarmicifolia internal transcribed spacer 1, 5.8S ribosomal RNA gene, and internal transcribed spacer 2, complete sequence	1035	1035	96%	0.0	97%	AY603249.1	1045,78	89,7%
Select seq gb|AY603224.1|	Achillea ochroleuca internal transcribed spacer 1, 5.8S ribosomal RNA gene, and internal transcribed spacer 2, complete sequence	1031	1031	96%	0.0	96%	AY603224.1	1031,00	88,4%
Select seq gb|AY603256.1|	Achillea fragrantissima internal transcribed spacer 1, 5.8S ribosomal RNA gene, and internal transcribed spacer 2, complete sequence	1027	1027	96%	0.0	96%	AY603256.1	1027,00	88,1%
Select seq gb|AY603255.1|	Achillea teretifolia internal transcribed spacer 1, 5.8S ribosomal RNA gene, and internal transcribed spacer 2, complete sequence	1026	1026	96%	0.0	96%	AY603255.1	1026,00	88,0%
Select seq gb|AY603251.1|	Achillea salicifolia internal transcribed spacer 1, 5.8S ribosomal RNA gene, and internal transcribed spacer 2, complete sequence	1024	1024	96%	0.0	96%	AY603251.1	1024,00	87,8%
Select seq gb|AY603248.1|	Achillea ptarmica internal transcribed spacer 1, 5.8S ribosomal RNA gene, and internal transcribed spacer 2, complete sequence	1024	1024	96%	0.0	96%	AY603248.1	1024,00	87,8%
Select seq gb|AY603247.1|	Achillea pyrenaica internal transcribed spacer 1, 5.8S ribosomal RNA gene, and internal transcribed spacer 2, complete sequence	1024	1024	96%	0.0	96%	AY603247.1	1024,00	87,8%
Select seq gb|AY603246.1|	Achillea impatiens internal transcribed spacer 1, 5.8S ribosomal RNA gene, and internal transcribed spacer 2, complete sequence	1024	1024	96%	0.0	96%	AY603246.1	1024,00	87,8%

<table>
<tr><td>Select seq gb|AY603235.1|</td><td>Achillea moschata internal transcribed spacer 1, 5.8S ribosomal RNA gene, and internal transcribed spacer 2, complete sequence</td><td>1024</td><td>1024</td><td>96% 0.0</td><td>96% AY603235.1</td><td>1024,00</td><td>87,8%</td></tr>
<tr><td>Select seq gb|AY603237.1|</td><td>Achillea nana internal transcribed spacer 1, 5.8S ribosomal RNA gene, and internal transcribed spacer 2, complete sequence</td><td>1022</td><td>1022</td><td>96% 0.0</td><td>96% AY603237.1</td><td>1022,00</td><td>87,6%</td></tr>
<tr><td>Select seq gb|AY603233.1|</td><td>Achillea macrophylla internal transcribed spacer 1, 5.8S ribosomal RNA gene, and internal transcribed spacer 2, complete sequence</td><td>1020</td><td>1020</td><td>96% 0.0</td><td>96% AY603233.1</td><td>1020,00</td><td>87,5%</td></tr>
<tr><td>Select seq gb|AY603250.1|</td><td>Achillea biserrata internal transcribed spacer 1, 5.8S ribosomal RNA gene, and internal transcribed spacer 2, complete sequence</td><td>1018</td><td>1018</td><td>96% 0.0</td><td>96% AY603250.1</td><td>1018,00</td><td>87,3%</td></tr>
<tr><td>Select seq gb|AY603244.1|</td><td>Achillea salicifolia internal transcribed spacer 1, 5.8S ribosomal RNA gene, and internal transcribed spacer 2, complete sequence</td><td>1018</td><td>1018</td><td>96% 0.0</td><td>96% AY603244.1</td><td>1018,00</td><td>87,3%</td></tr>
<tr><td>Select seq gb|AY603226.1|</td><td>Achillea abrotanoides internal transcribed spacer 1, 5.8S ribosomal RNA gene, and internal transcribed spacer 2, complete sequence</td><td>1018</td><td>1018</td><td>96% 0.0</td><td>96% AY603226.1</td><td>1018,00</td><td>87,3%</td></tr>
<tr><td>Select seq gb|KC855670.1|</td><td>Achillea multifida isolate ACMt5 18S ribosomal RNA gene, partial sequence; internal transcribed spacer 1, 5.8S ribosomal RNA gene, and internal transcribed spacer 2, complete sequence; and 28S ribosomal RNA gene, partial sequence</td><td>1016</td><td>1016</td><td>96% 0.0</td><td>96% KC855670.1</td><td>1016,00</td><td>87,1%</td></tr>
<tr><td>Select seq gb|AY603234.1|</td><td>Achillea x morisiana internal transcribed spacer 1, 5.8S ribosomal RNA gene, and internal transcribed spacer 2, complete sequence</td><td>1016</td><td>1016</td><td>96% 0.0</td><td>96% AY603234.1</td><td>1016,00</td><td>87,1%</td></tr>
<tr><td>Select seq gb|AY603231.1|</td><td>Achillea erba-rotta internal transcribed spacer 1, 5.8S ribosomal RNA gene, and internal transcribed spacer 2, complete sequence</td><td>1016</td><td>1016</td><td>96% 0.0</td><td>96% AY603231.1</td><td>1016,00</td><td>87,1%</td></tr>
<tr><td>Select seq gb|KC855667.1|</td><td>Achillea multifida isolate ACMt2 18S ribosomal RNA gene, partial sequence; internal transcribed spacer 1, 5.8S ribosomal RNA gene, and internal transcribed spacer 2, complete sequence; and 28S ribosomal RNA gene, partial sequence</td><td>1011</td><td>1011</td><td>96% 0.0</td><td>96% KC855667.1</td><td>1011,00</td><td>86,7%</td></tr>
<tr><td>Select seq gb|AY603252.1|</td><td>Achillea cretica internal transcribed spacer 1, 5.8S ribosomal RNA gene, and internal transcribed spacer 2, complete sequence</td><td>1011</td><td>1011</td><td>96% 0.0</td><td>96% AY603252.1</td><td>1011,00</td><td>86,7%</td></tr>
<tr><td>Select seq gb|AY603254.1|</td><td>Achillea wilhelmsii internal transcribed spacer 1, 5.8S ribosomal RNA gene, and internal transcribed spacer 2, complete sequence</td><td>1009</td><td>1009</td><td>96% 0.0</td><td>96% AY603254.1</td><td>1009,00</td><td>86,5%</td></tr>
<tr><td>Select seq gb|KC855671.1|</td><td>Achillea multifida isolate ACMt6 18S ribosomal RNA gene, partial sequence; internal transcribed spacer 1, 5.8S ribosomal RNA gene, and internal transcribed spacer 2, complete sequence; and 28S ribosomal RNA gene, partial sequence</td><td>1018</td><td>1018</td><td>97% 0.0</td><td>96% KC855671.1</td><td>1007,51</td><td>86,4%</td></tr>
<tr><td>Select seq gb|KC816562.1|</td><td>Matricaria chamomilla var. recutita 18S ribosomal RNA gene, partial sequence; internal transcribed spacer 1, 5.8S ribosomal RNA gene, and internal transcribed spacer 2, complete sequence; and 28S ribosomal RNA gene, partial sequence</td><td>1038</td><td>1038</td><td>99% 0.0</td><td>96% KC816562.1</td><td>1006,55</td><td>86,3%</td></tr>
<tr><td>Select seq gb|KC855673.1|</td><td>Achillea multifida isolate ACMt8 18S ribosomal RNA gene, partial sequence; internal transcribed spacer 1, 5.8S ribosomal RNA gene, and internal transcribed spacer 2, complete sequence; and 28S ribosomal RNA gene, partial sequence</td><td>1005</td><td>1005</td><td>96% 0.0</td><td>96% KC855673.1</td><td>1005,00</td><td>86,2%</td></tr>
<tr><td>Select seq dbj|AB683260.1|</td><td>Tanacetum abrotanifolium gene for ITS1, 5.8S rRNA and ITS2, complete sequence, specimen_voucher: 68865 (TARI)</td><td>1005</td><td>1005</td><td>96% 0.0</td><td>96% AB683260.1</td><td>1005,00</td><td>86,2%</td></tr>
<tr><td>Select seq dbj|AB683267.1|</td><td>Tanacetum armenum gene for ITS1, 5.8S rRNA and ITS2, complete sequence, specimen_voucher: (W, 1972-03416)</td><td>1003</td><td>1003</td><td>96% 0.0</td><td>96% AB683267.1</td><td>1003,00</td><td>86,0%</td></tr>
<tr><td>Select seq gb|AY603253.1|</td><td>Achillea wilhelmsii internal transcribed spacer 1, 5.8S ribosomal RNA gene, and internal transcribed spacer 2, complete sequence</td><td>1003</td><td>1003</td><td>96% 0.0</td><td>96% AY603253.1</td><td>1003,00</td><td>86,0%</td></tr>
<tr><td>Select seq gb|AY603243.1|</td><td>Achillea acuminata internal transcribed spacer 1, 5.8S ribosomal RNA gene, and internal transcribed spacer 2, complete sequence</td><td>1002</td><td>1002</td><td>96% 0.0</td><td>96% AY603243.1</td><td>1002,00</td><td>85,9%</td></tr>
<tr><td>Select seq gb|KC855686.1|</td><td>Achillea multifida isolate ACMt21 18S ribosomal RNA gene, partial sequence; internal transcribed spacer 1, 5.8S ribosomal RNA gene, and internal transcribed spacer 2, complete sequence; and 28S ribosomal RNA gene, partial sequence</td><td>990</td><td>990</td><td>95% 0.0</td><td>96% KC855686.1</td><td>1000,42</td><td>85,8%</td></tr>
<tr><td>Select seq dbj|AB683285.1|</td><td>Tanacetum germanicopolitanum gene for ITS1, 5.8S rRNA and ITS2, complete sequence, specimen_voucher: 14254 (S)</td><td>994</td><td>994</td><td>96% 0.0</td><td>96% AB683285.1</td><td>994,00</td><td>85,2%</td></tr>
<tr><td>Select seq gb|DQ267612.1|</td><td>Achillea pseudoaleppica internal transcribed spacer 1, partial sequence; 5.8S ribosomal RNA gene, complete sequence; and internal transcribed spacer 2, partial sequence</td><td>994</td><td>994</td><td>96% 0.0</td><td>96% DQ267612.1</td><td>994,00</td><td>85,2%</td></tr>
<tr><td>Select seq dbj|AB683265.1|</td><td>Tanacetum argenteum subsp. canum gene for ITS1, 5.8S rRNA and ITS2, complete sequence, specimen_voucher: 46856 (B)</td><td>992</td><td>992</td><td>96% 0.0</td><td>96% AB683265.1</td><td>992,00</td><td>85,1%</td></tr>
<tr><td>Select seq dbj|AB683290.1|</td><td>Tanacetum khorassanicum gene for ITS1, 5.8S rRNA and ITS2, complete sequence, specimen_voucher: 4937 (S)</td><td>998</td><td>998</td><td>96% 0.0</td><td>95% AB683290.1</td><td>987,60</td><td>84,7%</td></tr>
<tr><td>Select seq gb|KC855666.1|</td><td>Achillea multifida isolate ACMt1 18S ribosomal RNA gene, partial sequence; internal transcribed spacer 1, 5.8S ribosomal RNA gene, and internal transcribed spacer 2, complete sequence; and 28S ribosomal RNA gene, partial sequence</td><td>994</td><td>994</td><td>96% 0.0</td><td>95% KC855666.1</td><td>983,65</td><td>84,3%</td></tr>
<tr><td>Select seq emb|FN823081.1|</td><td>Tanacetum coccineum genomic DNA containing ITS1, 5.8S rRNA gene, ITS2, specimen voucher MPH:1144</td><td>994</td><td>994</td><td>96% 0.0</td><td>95% FN823081.1</td><td>983,65</td><td>84,3%</td></tr>
<tr><td>Select seq gb|KC855679.1|</td><td>Achillea multifida isolate ACMt14 18S ribosomal RNA gene, partial sequence; internal transcribed spacer 1, 5.8S ribosomal RNA gene, and internal transcribed spacer 2, complete sequence; and 28S ribosomal RNA gene, partial sequence</td><td>983</td><td>983</td><td>95% 0.0</td><td>95% KC855679.1</td><td>983,00</td><td>84,3%</td></tr>
<tr><td>Select seq gb|EF577320.1|</td><td>Tanacetum parthenium 18S ribosomal RNA gene, partial sequence; internal transcribed spacer 1, 5.8S ribosomal RNA gene, and internal transcribed spacer 2, complete sequence; and 28S ribosomal RNA gene, partial sequence</td><td>1024</td><td>1024</td><td>99% 0.0</td><td>95% EF577320.1</td><td>982,63</td><td>84,2%</td></tr>
</table>

Select seq gb|AY603225.1|	Achillea ligustica internal transcribed spacer 1, 5.8S ribosomal RNA gene, and internal transcribed spacer 2, complete sequence	990	990	96% 0.0	95%	AY603225.1	979,69	84,0%
Select seq dbj|AB683304.1|	Tanacetum pinnatum gene for ITS1, 5.8S rRNA and ITS2, complete sequence, specimen_voucher: 1118 (MPH)	989	989	96% 0.0	95%	AB683304.1	978,70	83,9%
Select seq dbj|AB683271.1|	Tanacetum bamianicum gene for ITS1, 5.8S rRNA and ITS2, complete sequence, specimen_voucher: 18238 (B)	989	989	96% 0.0	95%	AB683271.1	978,70	83,9%
Select seq dbj|AB608333.1|	Tanacetum coccineum genes for ITS1, 5.8S rRNA, ITS2, complete sequence	989	989	96% 0.0	95%	AB608333.1	978,70	83,9%
Select seq gb|DQ267611.1|	Achillea membranacea internal transcribed spacer 1, partial sequence; 5.8S ribosomal RNA gene, complete sequence; and internal transcribed spacer 2, partial sequence	989	989	96% 0.0	95%	DQ267611.1	978,70	83,9%
Select seq gb|EF577323.1|	Tanacetum vulgare 18S ribosomal RNA gene, partial sequence; internal transcribed spacer 1, 5.8S ribosomal RNA gene, and internal transcribed spacer 2, complete sequence; and 28S ribosomal RNA gene, partial sequence	1018	1018	99% 0.0	95%	EF577323.1	976,87	83,8%
Select seq gb|EF577322.1|	Tanacetum ptarmiciflorum 18S ribosomal RNA gene, partial sequence; internal transcribed spacer 1, 5.8S ribosomal RNA gene, and internal transcribed spacer 2, complete sequence; and 28S ribosomal RNA gene, partial sequence	1018	1018	99% 0.0	95%	EF577322.1	976,87	83,8%
Select seq gb|DQ267613.1|	Achillea vermicularis internal transcribed spacer 1, partial sequence; 5.8S ribosomal RNA gene, complete sequence; and internal transcribed spacer 2, partial sequence	987	987	96% 0.0	95%	DQ267613.1	976,72	83,7%
Select seq dbj|AB683287.1|	Tanacetum haradjanii gene for ITS1, 5.8S rRNA and ITS2, complete sequence, specimen_voucher: 16366 (M)	983	983	96% 0.0	95%	AB683287.1	972,76	83,4%
Select seq dbj|AB683323.1|	Tanacetum sonbolii gene for ITS1, 5.8S rRNA and ITS2, complete sequence, specimen_voucher: 998 (MPH)	981	981	96% 0.0	95%	AB683323.1	970,78	83,2%
Select seq dbj|AB608331.1|	Tanacetum canescens genes for ITS1, 5.8S rRNA, ITS2, complete sequence	981	981	96% 0.0	95%	AB608331.1	970,78	83,2%
Select seq dbj|AB608336.1|	Tanacetum polycephalum genes for ITS1, 5.8S rRNA, ITS2, complete sequence	979	979	96% 0.0	95%	AB608336.1	968,80	83,1%
Select seq dbj|AB683303.1|	Tanacetum parthenifolium gene for ITS1, 5.8S rRNA and ITS2, complete sequence, specimen_voucher: 1127 (MPH)	972	972	96% 0.0	95%	AB683303.1	961,88	82,5%
Select seq gb|KM887397.1|	Anacyclus pyrethrum isolate SBB-1407 18S ribosomal RNA gene, partial sequence; internal transcribed spacer 1, 5.8S ribosomal RNA gene, and internal transcribed spacer 2, complete sequence; and 28S ribosomal RNA gene, partial sequence	1000	1000	99% 0.0	95%	KM887397.1	959,60	82,3%
Select seq gb|KM887396.1|	Anacyclus pyrethrum isolate SBB-1364 18S ribosomal RNA gene, partial sequence; internal transcribed spacer 1, 5.8S ribosomal RNA gene, and internal transcribed spacer 2, complete sequence; and 28S ribosomal RNA gene, partial sequence	1000	1000	99% 0.0	95%	KM887396.1	959,60	82,3%
Select seq gb|KR150159.1|	Anthemis sp. Kh95 internal transcribed spacer 1, partial sequence; 5.8S ribosomal RNA gene gene, complete sequence; and internal transcribed spacer 2 region, partial sequence	996	996	99% 0.0	95%	KR150159.1	955,76	81,9%
Select seq gb|EU179212.1|	Matricaria recutita 18S ribosomal RNA gene, partial sequence; internal transcribed spacer 1, 5.8S ribosomal RNA gene, and internal transcribed spacer 2, complete sequence; and 26S ribosomal RNA gene, partial sequence	985	985	99% 0.0	94%	EU179212.1	935,25	80,2%

Select for downloading or viewing reports	Kh058_trnL Description	Max score	Total score	Query cover	E value	Ident	Accession	(Ident/Cover)* Max score	Deviation from top hit
Select seq gb|AY603312.1|	Achillea macrophylla voucher Ehrendorfer, 2000.08.17 (WU) trnL gene, partial sequence; trnL-trnF intergenic spacer, complete sequence; and tRNA-Phe (trnF) gene, partial sequence; chloroplast	1157	1157	85%	0.0	99%	AY603312.1	1347,56	100,0%
Select seq gb|AY603303.1|	Achillea coarctata voucher Ehrendorfer & Danihelka, 2002.07.12-13 (WU) trnL gene, partial sequence; trnL-trnF intergenic spacer, complete sequence; and tRNA-Phe (trnF) gene, partial sequence; chloroplast	1157	1157	85%	0.0	99%	AY603303.1	1347,56	100,0%
Select seq gb|AY603290.1|	Achillea crithmifolia voucher Saukel B035 (WU) trnL gene, partial sequence; trnL-trnF intergenic spacer, complete sequence; and tRNA-Phe (trnF) gene, partial sequence; chloroplast	1157	1157	85%	0.0	99%	AY603290.1	1347,56	100,0%
Select seq gb|AY603307.1|	Achillea atrata voucher Schneeweiss 8943/2, 2002.08.23 (WU) trnL gene and trnL-trnF intergenic spacer, partial sequence; chloroplast	1138	1138	84%	0.0	99%	AY603307.1	1341,21	99,5%
Select seq gb|AY603279.1|	Achillea asplenifolia voucher Tod, 2001.10.03 (WU) trnL gene, partial sequence; and trnL-trnF intergenic spacer, complete sequence; chloroplast	1138	1138	84%	0.0	99%	AY603279.1	1341,21	99,5%
Select seq gb|AY603324.1|	Achillea impatiens voucher Tribsch 9491, 2003.07.26 (WU) trnL gene, partial sequence; trnL-trnF intergenic spacer, complete sequence; and tRNA-Phe (trnF) gene, partial sequence; chloroplast	1151	1151	85%	0.0	99%	AY603324.1	1340,58	99,5%
Select seq gb|AY603318.1|	Achillea erba-rotta voucher Schoenswetter & Tribsch, 1998 (WU) trnL gene, partial sequence; trnL-trnF intergenic spacer, complete sequence; and tRNA-Phe (trnF) gene, partial sequence; chloroplast	1151	1151	85%	0.0	99%	AY603318.1	1340,58	99,5%
Select seq gb|AY603313.1|	Achillea oxyloba voucher Ehrendorfer-Schratt, 2001.23.08 (WU) trnL gene, partial sequence; trnL-trnF intergenic spacer, complete sequence; and tRNA-Phe (trnF) gene, partial sequence; chloroplast	1151	1151	85%	0.0	99%	AY603313.1	1340,58	99,5%
Select seq gb|AY603305.1|	Achillea x morisiana voucher Schoenswetter & Tribsch, 1998.07.21 (WU) trnL gene, partial sequence; trnL-trnF intergenic spacer, complete sequence; and tRNA-Phe (trnF) gene, partial sequence; chloroplast	1151	1151	85%	0.0	99%	AY603305.1	1340,58	99,5%
Select seq gb|AY603292.1|	Achillea pseudopectinata voucher Ehrendorfer & Danihelka, 2002.07.12 (WU) trnL gene, partial sequence; trnL-trnF intergenic spacer, complete sequence; and tRNA-Phe (trnF) gene, partial sequence; chloroplast	1151	1151	85%	0.0	99%	AY603292.1	1340,58	99,5%
Select seq gb|EU128988.1|	Achillea millefolium subsp. ceretanica isolate Pyrenees no. 1 trnL-trnF intergenic spacer, partial sequence; chloroplast	1136	1136	84%	0.0	99%	EU128988.1	1338,86	99,4%
Select seq gb|EU128982.1|	Achillea asiatica isolate Huilang no. 1 trnL-trnF intergenic spacer, partial sequence; chloroplast	1136	1136	84%	0.0	99%	EU128982.1	1338,86	99,4%
Select seq gb|AY603322.1|	Achillea salicifolia voucher Ehrendorfer & Guo K14, 2003.07.28 (WU) trnL gene, partial sequence; trnL-trnF intergenic spacer, complete sequence; and tRNA-Phe (trnF) gene, partial sequence; chloroplast	1146	1146	85%	0.0	99%	AY603322.1	1334,75	99,0%
Select seq gb|AY603319.1|	Achillea pindicola subsp. integrifolia voucher Schneeweiss & Gutermann, 2000.07.01 (WU) trnL gene, partial sequence; trnL-trnF intergenic spacer, complete sequence; and tRNA-Phe (trnF) gene, partial sequence; chloroplast	1146	1146	85%	0.0	99%	AY603319.1	1334,75	99,0%
Select seq gb|AY603308.1|	Achillea clavennae voucher Saukel, 2003.06.21 (WU) trnL gene, partial sequence; trnL-trnF intergenic spacer, complete sequence; and tRNA-Phe (trnF) gene, partial sequence; chloroplast	1146	1146	85%	0.0	99%	AY603308.1	1334,75	99,0%
Select seq gb|AY603302.1|	Achillea ligustica voucher Ehrendorfer, 2001.09.21 (WU) trnL gene, partial sequence; trnL-trnF intergenic spacer, complete sequence; and tRNA-Phe (trnF) gene, partial sequence; chloroplast	1146	1146	85%	0.0	99%	AY603302.1	1334,75	99,0%
Select seq gb|AY603300.1|	Achillea filipendulina voucher Saukel GR105 (WU) trnL gene, partial sequence; trnL-trnF intergenic spacer, complete sequence; and tRNA-Phe (trnF) gene, partial sequence; chloroplast	1146	1146	85%	0.0	99%	AY603300.1	1334,75	99,0%
Select seq gb|AY603296.1|	Achillea ochroleuca voucher Ehrendorfer, 2003.06.07 (WU) trnL gene, partial sequence; trnL-trnF intergenic spacer, complete sequence; and tRNA-Phe (trnF) gene, partial sequence; chloroplast	1146	1146	85%	0.0	99%	AY603296.1	1334,75	99,0%
Select seq gb|AY603288.1|	Achillea virescens voucher Ehrendorfer, 2002.07.31 (WU) trnL gene, partial sequence; trnL-trnF intergenic spacer, complete sequence; and tRNA-Phe (trnF) gene, partial sequence; chloroplast	1146	1146	85%	0.0	99%	AY603288.1	1334,75	99,0%
Select seq gb|AY603269.1|	Achillea schmakovii voucher Staudinger 5304 (WU) trnL gene, partial sequence; trnL-trnF intergenic spacer, complete sequence; and tRNA-Phe (trnF) gene, partial sequence; chloroplast	1146	1146	85%	0.0	99%	AY603269.1	1334,75	99,0%
Select seq gb|AY603284.1|	Achillea asiatica voucher Tan, 2002.05.15 (WU) trnL gene, partial sequence; trnL-trnF intergenic spacer, complete sequence; and tRNA-Phe (trnF) gene, partial sequence; chloroplast	1146	1146	85%	0.0	99%	AY603284.1	1334,75	99,0%
Select seq gb|AY603283.1|	Achillea alpina voucher Guo & Rao BJ02-5 (WU) trnL gene, partial sequence; trnL-trnF intergenic spacer, complete sequence; and tRNA-Phe (trnF) gene, partial sequence; chloroplast	1146	1146	85%	0.0	99%	AY603283.1	1334,75	99,0%
Select seq gb|AY603282.1|	Achillea wilsoniana voucher Guo & Rao 0203 (WU) trnL gene, partial sequence; trnL-trnF intergenic spacer, complete sequence; and tRNA-Phe (trnF) gene, partial sequence; chloroplast	1146	1146	85%	0.0	99%	AY603282.1	1334,75	99,0%
Select seq gb|AY603280.1|	Achillea euxina voucher Schanzer Ach12 (WU) trnL gene, partial sequence; trnL-trnF intergenic spacer, complete sequence; and tRNA-Phe (trnF) gene, partial sequence; chloroplast	1146	1146	85%	0.0	99%	AY603280.1	1334,75	99,0%
Select seq gb|AY603278.1|	Achillea roseoalba voucher Ehrendorfer & Ehrendorfer-Schratt, 2001.07.21 (WU) trnL gene, partial sequence; trnL-trnF intergenic spacer, complete sequence; and tRNA-Phe (trnF) gene, partial sequence; chloroplast	1146	1146	85%	0.0	99%	AY603278.1	1334,75	99,0%
Select seq gb|AY603276.1|	Achillea collina voucher Ehrendorfer, 2001.07.22 (WU) trnL gene, partial sequence; trnL-trnF intergenic spacer, complete sequence; and tRNA-Phe (trnF) gene, partial sequence; chloroplast	1146	1146	85%	0.0	99%	AY603276.1	1334,75	99,0%

Select seq gb|AY603273.1|	Achillea setacea voucher Saukel GR246 (WU) trnL gene, partial sequence; trnL-trnF intergenic spacer, complete sequence; and tRNA-Phe (trnF) gene, partial sequence; chloroplast	1146	1146	85%	0.0	99% AY603273.1	1334,75	99,0%
Select seq gb|AY603271.1|	Achillea styriaca voucher Saukel, 2002.07 (WU) trnL gene, partial sequence; trnL-trnF intergenic spacer, complete sequence; and tRNA-Phe (trnF) gene, partial sequence; chloroplast	1146	1146	85%	0.0	99% AY603271.1	1334,75	99,0%
Select seq gb|AY603265.1|	Achillea pannonica voucher Saukel, 2002.06.04 (WU) trnL gene, partial sequence; trnL-trnF intergenic spacer, complete sequence; and tRNA-Phe (trnF) gene, partial sequence; chloroplast	1146	1146	85%	0.0	99% AY603265.1	1334,75	99,0%
Select seq gb|DQ267619.1|	Achillea latiloba tRNA-Leu (trnL) gene, partial sequence; trnL-trnF intergenic spacer, complete sequence; and tRNA-Phe (trnF) gene, partial sequence; chloroplast	1146	1146	85%	0.0	99% DQ267619.1	1334,75	99,0%
Select seq gb|EU385030.1|	Achillea millefolium tRNA-Leu (trnL) gene and trnL-trnF intergenic spacer, complete sequence; chloroplast	1158	1158	86%	0.0	99% EU385030.1	1333,05	98,9%
Select seq gb|EU129025.1|	Achillea millefolium var. gigantea isolate Selma no. 1 trnL-trnF intergenic spacer, partial sequence; chloroplast	1131	1131	84%	0.0	99% EU129025.1	1332,96	98,9%
Select seq gb|EU128991.1|	Achillea millefolium var. alpicola isolate Hurricane no. 1 trnL-trnF intergenic spacer, partial sequence; chloroplast	1131	1131	84%	0.0	99% EU128991.1	1332,96	98,9%
Select seq gb|AY603309.1|	Achillea clavennae voucher Schoenswetter & Tribsch 56, 2000.07.04 (WU) trnL gene, partial sequence; trnL-trnF intergenic spacer, complete sequence; and tRNA-Phe (trnF) gene, partial sequence; chloroplast	1144	1144	85%	0.0	99% AY603309.1	1332,42	98,9%
Select seq gb|AY603281.1|	Achillea millefolium subsp. ceretanica voucher Ehrendorfer, 2001 (WU) trnL gene, partial sequence; trnL-trnF intergenic spacer, complete sequence; and tRNA-Phe (trnF) gene, partial sequence; chloroplast	1142	1142	85%	0.0	99% AY603281.1	1330,09	98,7%
Select seq gb|AY603306.1|	Achillea multifida voucher Ehrendorfer, 1993.07.24 (WU) trnL gene, partial sequence; trnL-trnF intergenic spacer, complete sequence; and tRNA-Phe (trnF) gene, partial sequence; chloroplast	1140	1140	85%	0.0	99% AY603306.1	1327,76	98,5%
Select seq gb|AY603310.1|	Achillea clusiana voucher Saukel, 2003.06.21 (WU) trnL gene, partial sequence; trnL-trnF intergenic spacer, complete sequence; and tRNA-Phe (trnF) gene, partial sequence; chloroplast	1140	1140	85%	0.0	99% AY603310.1	1327,76	98,5%
Select seq gb|AY603301.1|	Achillea micrantha voucher Ehrendorfer & Danihelka, 2002.07.12-13 (WU) trnL gene, partial sequence; trnL-trnF intergenic spacer, complete sequence; and tRNA-Phe (trnF) gene, partial sequence; chloroplast	1140	1140	85%	0.0	99% AY603301.1	1327,76	98,5%
Select seq gb|AY603287.1|	Achillea monticola voucher Schneeweiss et al. 6396, 2001.07.14 (WU) trnL gene, partial sequence; trnL-trnF intergenic spacer, complete sequence; and tRNA-Phe (trnF) gene, partial sequence; chloroplast	1140	1140	85%	0.0	99% AY603287.1	1327,76	98,5%
Select seq gb|AY603285.1|	Achillea asiatica voucher Staudinger 5893 (WU) trnL gene, partial sequence; trnL-trnF intergenic spacer, complete sequence; and tRNA-Phe (trnF) gene, partial sequence; chloroplast	1140	1140	85%	0.0	99% AY603285.1	1327,76	98,5%
Select seq gb|EU128984.1|	Achillea asiatica isolate Altai 2x no. 1 trnL-trnF intergenic spacer, partial sequence; chloroplast	1125	1125	84%	0.0	99% EU128984.1	1325,89	98,4%
Select seq gb|AY603297.1|	Achillea holosericea voucher Schneeweiss & Gutermann, 2000.06.28 (WU) trnL gene, partial sequence; trnL-trnF intergenic spacer, complete sequence; and tRNA-Phe (trnF) gene, partial sequence; chloroplast	1138	1138	85%	0.0	99% AY603297.1	1325,44	98,4%
Select seq gb|AY603293.1|	Achillea absinthoides voucher Schneeweiss & Gutermann, 2000.07.01 (WU) trnL gene, partial sequence; trnL-trnF intergenic spacer, complete sequence; and tRNA-Phe (trnF) gene, partial sequence; chloroplast	1138	1138	85%	0.0	99% AY603293.1	1325,44	98,4%
Select seq gb|AY603326.1|	Achillea pyrenaica voucher Schoenswetter et al. 6395 (WU) trnL gene, partial sequence; trnL-trnF intergenic spacer, complete sequence; and tRNA-Phe (trnF) gene, partial sequence; chloroplast	1125	1125	85%	0.0	99% AY603326.1	1310,29	97,2%
Select seq gb|AY603323.1|	Achillea ptarmica voucher Ehrendorfer, 2003.09.26 (WU) trnL gene, partial sequence; trnL-trnF intergenic spacer, complete sequence; and tRNA-Phe (trnF) gene, partial sequence; chloroplast	1125	1125	85%	0.0	99% AY603323.1	1310,29	97,2%
Select seq gb|AY603316.1|	Achillea schurii voucher Ehrendorfer & Ehrendorfer-Schratt, 2002.07.12 (WU) trnL gene, partial sequence; trnL-trnF intergenic spacer, complete sequence; and tRNA-Phe (trnF) gene, partial sequence; chloroplast	1125	1125	85%	0.0	99% AY603316.1	1310,29	97,2%
Select seq gb|AY603270.1|	Achillea distans voucher Saukel 96010 (WU) trnL gene, partial sequence; trnL-trnF intergenic spacer, complete sequence; and tRNA-Phe (trnF) gene, partial sequence; chloroplast	1123	1123	85%	0.0	99% AY603270.1	1307,96	97,1%
Select seq gb|AY603274.1|	Achillea setacea voucher Ehrendorfer, 2002.03.24 (WU) trnL gene, partial sequence; trnL-trnF intergenic spacer, complete sequence; and tRNA-Phe (trnF) gene, partial sequence; chloroplast	1123	1123	85%	0.0	99% AY603274.1	1307,96	97,1%
Select seq gb|AY603320.1|	Achillea acuminata voucher Guo & Rao 0201 (WU) trnL gene, partial sequence; trnL-trnF intergenic spacer, complete sequence; and tRNA-Phe (trnF) gene, partial sequence; chloroplast	1120	1120	85%	0.0	99% AY603320.1	1304,47	96,8%
Select seq gb|AY603325.1|	Achillea chamaemelifolia voucher Ehrendorfer & Danihelka, 2002.07.13 (WU) trnL gene, partial sequence; trnL-trnF intergenic spacer, complete sequence; and tRNA-Phe (trnF) gene, partial sequence; chloroplast	1118	1118	85%	0.0	99% AY603325.1	1302,14	96,6%
Select seq gb|AY603315.1|	Achillea schurii voucher Ehrendorfer, 1998.07.08 (WU) trnL gene, partial sequence; trnL-trnF intergenic spacer, complete sequence; and tRNA-Phe (trnF) gene, partial sequence; chloroplast	1114	1114	85%	0.0	98% AY603315.1	1284,38	95,3%
Select seq gb|AY603275.1|	Achillea setacea voucher Tod, 1999.11.01 (WU) trnL gene, partial sequence; trnL-trnF intergenic spacer, complete sequence; and tRNA-Phe (trnF) gene, partial sequence; chloroplast	1096	1096	85%	0.0	98% AY603275.1	1263,62	93,8%
Select seq gb|AY603272.1|	Achillea setacea voucher Saukel, 2002.06.04 (WU) trnL gene, partial sequence; trnL-trnF intergenic spacer, complete sequence; and tRNA-Phe (trnF) gene, partial sequence; chloroplast	1096	1096	85%	0.0	98% AY603272.1	1263,62	93,8%

Select seq gb|JX073780.1|	Artemisia argentea tRNA-Leu (trnL) gene and trnL-trnF intergenic spacer, partial sequence; chloroplast	1099	1099	88% 0.0	97% JX073780.1	1211,40	89,9%
Select seq gb|JN867592.1|	Chrysanthemum indicum voucher NJ026 chloroplast, complete genome	1125	1125	91% 0.0	97% JN867592.1	1199,18	89,0%
Select seq gb|KF736805.1|	Artemisia spicigera tRNA-Leu (trnL) gene and trnL-trnF intergenic spacer, partial sequence; chloroplast	1057	1057	86% 0.0	96% KF736805.1	1179,91	87,6%
Select seq gb|JX073812.1|	Artemisia subdigitata tRNA-Leu (trnL) gene and trnL-trnF intergenic spacer, partial sequence; chloroplast	1079	1079	88% 0.0	96% JX073812.1	1177,09	87,3%
Select seq gb|JX073808.1|	Artemisia tilesii tRNA-Leu (trnL) gene and trnL-trnF intergenic spacer, partial sequence; chloroplast	1079	1079	88% 0.0	96% JX073808.1	1177,09	87,3%
Select seq gb|JX073748.1|	Artemisia santonicum tRNA-Leu (trnL) gene and trnL-trnF intergenic spacer, partial sequence; chloroplast	1079	1079	88% 0.0	96% JX073748.1	1177,09	87,3%
Select seq gb|HM000099.1|	Chrysanthemum indicum isolate HEBSJZ tRNA-Leu (trnL) gene, partial sequence; trnL-trnF intergenic spacer, complete sequence; and tRNA-Phe (trnF) gene, partial sequence; chloroplast	1114	1114	91% 0.0	96% HM000099.1	1175,21	87,2%
Select seq gb|HM000094.1|	Chrysanthemum indicum isolate HUBJZ tRNA-Leu (trnL) gene, partial sequence; trnL-trnF intergenic spacer, complete sequence; and tRNA-Phe (trnF) gene, partial sequence; chloroplast	1114	1114	91% 0.0	96% HM000094.1	1175,21	87,2%
Select seq gb|JX073816.1|	Artemisia elongata tRNA-Leu (trnL) gene and trnL-trnF intergenic spacer, partial sequence; chloroplast	1075	1075	88% 0.0	96% JX073816.1	1172,73	87,0%
Select seq dbj|AB551235.1|	Artemisia capillaris chloroplast genes for tRNA-Leu intron and 3' exon, trnL-trnF intergenic spacer and tRNA-Phe	1099	1099	90% 0.0	96% AB551235.1	1172,27	87,0%
Select seq gb|JX073828.1|	Kaschgaria komarovii tRNA-Leu (trnL) gene and trnL-trnF intergenic spacer, partial sequence; chloroplast	1074	1074	88% 0.0	96% JX073828.1	1171,64	86,9%
Select seq gb|JX073811.1|	Artemisia glomerata tRNA-Leu (trnL) gene and trnL-trnF intergenic spacer, partial sequence; chloroplast	1074	1074	88% 0.0	96% JX073811.1	1171,64	86,9%
Select seq gb|JX073805.1|	Artemisia gracilescens tRNA-Leu (trnL) gene and trnL-trnF intergenic spacer, partial sequence; chloroplast	1061	1061	87% 0.0	96% JX073805.1	1170,76	86,9%
Select seq gb|JX293720.1|	Artemisia frigida chloroplast, complete genome	1107	1107	91% 0.0	96% JX293720.1	1167,82	86,7%
Select seq gb|HM000093.1|	Chrysanthemum indicum isolate SCMY tRNA-Leu (trnL) gene, partial sequence; trnL-trnF intergenic spacer, complete sequence; and tRNA-Phe (trnF) gene, partial sequence; chloroplast	1107	1107	91% 0.0	96% HM000093.1	1167,82	86,7%
Select seq gb|HM000091.1|	Chrysanthemum indicum isolate HENXY tRNA-Leu (trnL) gene, partial sequence; trnL-trnF intergenic spacer, complete sequence; and tRNA-Phe (trnF) gene, partial sequence; chloroplast	1107	1107	91% 0.0	96% HM000091.1	1167,82	86,7%
Select seq gb|JX073804.1|	Artemisia chamaemelifolia tRNA-Leu (trnL) gene and trnL-trnF intergenic spacer, partial sequence; chloroplast	1070	1070	88% 0.0	96% JX073804.1	1167,27	86,6%
Select seq gb|JX073827.1|	Artemisia absinthium tRNA-Leu (trnL) gene and trnL-trnF intergenic spacer, partial sequence; chloroplast	1068	1068	88% 0.0	96% JX073827.1	1165,09	86,5%
Select seq gb|JX073814.1|	Mausolea eriocarpa tRNA-Leu (trnL) gene and trnL-trnF intergenic spacer, partial sequence; chloroplast	1068	1068	88% 0.0	96% JX073814.1	1165,09	86,5%
Select seq gb|JX073791.1|	Artemisia tomentella tRNA-Leu (trnL) gene and trnL-trnF intergenic spacer, partial sequence; chloroplast	1068	1068	88% 0.0	96% JX073791.1	1165,09	86,5%
Select seq gb|JX073803.1|	Artemisia tournefortiana tRNA-Leu (trnL) gene and trnL-trnF intergenic spacer, partial sequence; chloroplast	1068	1068	88% 0.0	96% JX073803.1	1165,09	86,5%
Select seq gb|JN867589.1|	Chrysanthemum indicum voucher HeN001 chloroplast, complete genome	1101	1101	91% 0.0	96% JN867589.1	1161,49	86,2%
Select seq gb|HM000097.1|	Chrysanthemum indicum isolate JXPX tRNA-Leu (trnL) gene, partial sequence; trnL-trnF intergenic spacer, complete sequence; and tRNA-Phe (trnF) gene, partial sequence; chloroplast	1101	1101	91% 0.0	96% HM000097.1	1161,49	86,2%
Select seq gb|HM000096.1|	Chrysanthemum indicum isolate YNKM tRNA-Leu (trnL) gene, partial sequence; trnL-trnF intergenic spacer, complete sequence; and tRNA-Phe (trnF) gene, partial sequence; chloroplast	1101	1101	91% 0.0	96% HM000096.1	1161,49	86,2%
Select seq gb|HM000095.1|	Chrysanthemum indicum isolate HUNZZ tRNA-Leu (trnL) gene, partial sequence; trnL-trnF intergenic spacer, complete sequence; and tRNA-Phe (trnF) gene, partial sequence; chloroplast	1101	1101	91% 0.0	96% HM000095.1	1161,49	86,2%
Select seq gb|HM000088.1|	Chrysanthemum indicum isolate GZGY tRNA-Leu (trnL) gene, partial sequence; trnL-trnF intergenic spacer, complete sequence; and tRNA-Phe (trnF) gene, partial sequence; chloroplast	1101	1101	91% 0.0	96% HM000088.1	1161,49	86,2%
Select seq gb|HM000087.1|	Chrysanthemum indicum isolate AHCZ tRNA-Leu (trnL) gene, partial sequence; trnL-trnF intergenic spacer, complete sequence; and tRNA-Phe (trnF) gene, partial sequence; chloroplast	1101	1101	91% 0.0	96% HM000087.1	1161,49	86,2%

Select seq gb|HM000085.1|	Chrysanthemum indicum isolate JSNJ tRNA-Leu (trnL) gene, partial sequence; trnL-trnF intergenic spacer, complete sequence; and tRNA-Phe (trnF) gene, partial sequence; chloroplast	1101	1101	91% 0.0	96%	HM000085.1	1161,49	86,2%
Select seq dbj|AB685322.1|	Chrysanthemum indicum chloroplast DNA, contains tRNA-Leu(trnL) gene (intron and 3' exon), trnL-trnF intergenic spacer and tRNA-Phe(trnF) gene	1088	1088	90% 0.0	96%	AB685322.1	1160,53	86,1%
Select seq gb|JX073820.1|	Artemisia kauaiensis tRNA-Leu (trnL) gene and trnL-trnF intergenic spacer, partial sequence; chloroplast	1062	1062	88% 0.0	96%	JX073820.1	1158,55	86,0%
Select seq gb|JQ362483.1|	Chrysanthemum x morifolium chloroplast, complete genome	1096	1096	91% 0.0	96%	JQ362483.1	1156,22	85,8%
Select seq gb|HM000092.1|	Chrysanthemum indicum isolate GXGL tRNA-Leu (trnL) gene, partial sequence; trnL-trnF intergenic spacer, complete sequence; and tRNA-Phe (trnF) gene, partial sequence; chloroplast	1096	1096	91% 0.0	96%	HM000092.1	1156,22	85,8%
Select seq gb|HM000086.1|	Chrysanthemum indicum isolate JSWX tRNA-Leu (trnL) gene, partial sequence; trnL-trnF intergenic spacer, complete sequence; and tRNA-Phe (trnF) gene, partial sequence; chloroplast	1096	1096	91% 0.0	96%	HM000086.1	1156,22	85,8%
Select seq gb|JX073823.1|	Artemisia globularia tRNA-Leu (trnL) gene and trnL-trnF intergenic spacer, partial sequence; chloroplast	1059	1059	88% 0.0	96%	JX073823.1	1155,27	85,7%
Select seq gb|KR150207.1|	Anthemis sp. 1 Kh95 trnL-trnF intergenic spacer region, partial sequence; chloroplast	1057	1211	88% 0.0	96%	KR150207.1	1153,09	85,6%
Select seq gb|JX073809.1|	Artemisia pattersonii tRNA-Leu (trnL) gene and trnL-trnF intergenic spacer, partial sequence; chloroplast	1057	1057	88% 0.0	96%	JX073809.1	1153,09	85,6%
Select seq gb|JX073807.1|	Artemisia martirensis tRNA-Leu (trnL) gene and trnL-trnF intergenic spacer, partial sequence; chloroplast	1057	1057	88% 0.0	96%	JX073807.1	1153,09	85,6%
Select seq gb|HM000098.1|	Chrysanthemum indicum isolate FJFZ tRNA-Leu (trnL) gene, partial sequence; trnL-trnF intergenic spacer, complete sequence; and tRNA-Phe (trnF) gene, partial sequence; chloroplast	1090	1090	91% 0.0	96%	HM000098.1	1149,89	85,3%
Select seq gb|HM000089.1|	Chrysanthemum indicum isolate AHHF tRNA-Leu (trnL) gene, partial sequence; trnL-trnF intergenic spacer, complete sequence; and tRNA-Phe (trnF) gene, partial sequence; chloroplast	1090	1090	91% 0.0	96%	HM000089.1	1149,89	85,3%
Select seq gb|HM000090.1|	Chrysanthemum indicum isolate ZJWZ tRNA-Leu (trnL) gene, partial sequence; trnL-trnF intergenic spacer, complete sequence; and tRNA-Phe (trnF) gene, partial sequence; chloroplast	1085	1085	91% 0.0	96%	HM000090.1	1144,62	84,9%
Select seq gb|KR150219.1|	Tripleurospermum sp. Kh63 trnL-trnF intergenic spacer region, partial sequence; chloroplast	1077	1077	90% 0.0	95%	KR150219.1	1136,83	84,4%
Select seq gb|JX073825.1|	Artemisia potentilloides tRNA-Leu (trnL) gene and trnL-trnF intergenic spacer, partial sequence; chloroplast	1053	1053	88% 0.0	95%	JX073825.1	1136,76	84,4%
Select seq gb|JX073752.1|	Artemisia rothrockii tRNA-Leu (trnL) gene and trnL-trnF intergenic spacer, partial sequence; chloroplast	1053	1053	88% 0.0	95%	JX073752.1	1136,76	84,4%
Select seq gb|GU817959.1|	Anacyclus valentinus isolate L1186 tRNA-Leu (trnL) gene, partial sequence; trnL-trnF intergenic spacer, complete sequence; and tRNA-Phe (trnF) gene, partial sequence; plastid	1064	1064	91% 0.0	95%	GU817959.1	1110,77	82,4%
Select seq gb|KF887960.1|	Artemisia montana chloroplast, complete genome	1062	1062	91% 0.0	95%	KF887960.1	1108,68	82,3%
							0,00	0,0%

Select for downloading or viewing reports	Kh059_ITS Description	Max score	Total score	Query cover	E value	Ident	Accession	(Ident/Cover)* Max score	Deviation from top hit
Select seq gb|GU166770.1|	Alcea acaulis isolate Iaca01 internal transcribed spacer 1, partial sequence; 5.8S ribosomal RNA gene, complete sequence; and internal transcribed spacer 2, partial sequence	789	789	78%	0.0	95%	GU166770.1	960,96	100,0%
Select seq gb|EF679716.1|	Alcea arbelensis voucher PE624 internal transcribed spacer 1, partial sequence; 5.8S ribosomal RNA gene, complete sequence; and internal transcribed spacer 2, partial sequence	985	985	99%	0.0	94%	EF679716.1	935,25	97,3%
Select seq gb|EF679725.1|	Alcea rechingeri voucher PE621 internal transcribed spacer 1, partial sequence; 5.8S ribosomal RNA gene, complete sequence; and internal transcribed spacer 2, partial sequence	981	981	99%	0.0	94%	EF679725.1	931,45	96,9%
Select seq gb|GU166768.1|	Alcea rufescens isolate Iruf01 internal transcribed spacer 1, partial sequence; 5.8S ribosomal RNA gene, complete sequence; and internal transcribed spacer 2, partial sequence	979	979	99%	0.0	94%	GU166768.1	929,56	96,7%
Select seq gb|EF679726.1|	Alcea sachsachanica voucher PE620 internal transcribed spacer 1, partial sequence; 5.8S ribosomal RNA gene, complete sequence; and internal transcribed spacer 2, partial sequence	979	979	99%	0.0	94%	EF679726.1	929,56	96,7%
Select seq gb|EF679722.1|	Alcea koelzii voucher PE609 internal transcribed spacer 1, partial sequence; 5.8S ribosomal RNA gene, complete sequence; and internal transcribed spacer 2, partial sequence	979	979	99%	0.0	94%	EF679722.1	929,56	96,7%
Select seq gb|EF679718.1|	Alcea excubita voucher PE417 internal transcribed spacer 1, partial sequence; 5.8S ribosomal RNA gene, complete sequence; and internal transcribed spacer 2, partial sequence	979	979	99%	0.0	94%	EF679718.1	929,56	96,7%
Select seq gb|EF679714.1|	Alcea rosea voucher PE422 internal transcribed spacer 1, partial sequence; 5.8S ribosomal RNA gene, complete sequence; and internal transcribed spacer 2, partial sequence	979	979	99%	0.0	94%	EF679714.1	929,56	96,7%
Select seq gb|JX017319.1|	Althaea rosea voucher 01 internal transcribed spacer 1, partial sequence; 5.8S ribosomal RNA and internal transcribed spacer 2, complete sequence; and 26S ribosomal RNA, partial sequence	976	976	99%	0.0	94%	JX017319.1	926,71	96,4%
Select seq gb|GU166758.1|	Alcea glabrata isolate Igla01 internal transcribed spacer 1, partial sequence; 5.8S ribosomal RNA gene, complete sequence; and internal transcribed spacer 2, partial sequence	976	976	99%	0.0	94%	GU166758.1	926,71	96,4%
Select seq gb|EF679728.1|	Alcea schirazana voucher PE618 internal transcribed spacer 1, partial sequence; 5.8S ribosomal RNA gene, complete sequence; and internal transcribed spacer 2, partial sequence	976	976	99%	0.0	94%	EF679728.1	926,71	96,4%
Select seq gb|EF679727.1|	Alcea setosa voucher PE424 internal transcribed spacer 1, partial sequence; 5.8S ribosomal RNA gene, complete sequence; and internal transcribed spacer 2, partial sequence	976	976	99%	0.0	94%	EF679727.1	926,71	96,4%
Select seq gb|EF419544.1|	Alcea rosea isolate Iros422 internal transcribed spacer 1, partial sequence; 5.8S ribosomal RNA gene, complete sequence; and internal transcribed spacer 2, partial sequence	976	976	99%	0.0	94%	EF419544.1	926,71	96,4%
Select seq gb|KR150170.1|	Alcea sp. Kh68 internal transcribed spacer 1, partial sequence; 5.8S ribosomal RNA gene gene, complete sequence; and internal transcribed spacer 2 region, partial sequence	974	974	99%	0.0	94%	KR150170.1	924,81	96,2%
Select seq gb|GU166764.1|	Alcea lavateriflora isolate Ilav01 internal transcribed spacer 1, partial sequence; 5.8S ribosomal RNA gene, complete sequence; and internal transcribed spacer 2, partial sequence	974	974	99%	0.0	94%	GU166764.1	924,81	96,2%
Select seq gb|GU166763.1|	Alcea hohenackeri isolate Ihoh02 internal transcribed spacer 1, partial sequence; 5.8S ribosomal RNA gene, complete sequence; and internal transcribed spacer 2, partial sequence	974	974	99%	0.0	94%	GU166763.1	924,81	96,2%
Select seq gb|EF679729.1|	Alcea sulphurea voucher PE610 internal transcribed spacer 1, partial sequence; 5.8S ribosomal RNA gene, complete sequence; and internal transcribed spacer 2, partial sequence	974	974	99%	0.0	94%	EF679729.1	924,81	96,2%
Select seq gb|EF679721.1|	Alcea glabrata voucher PE607 internal transcribed spacer 1, partial sequence; 5.8S ribosomal RNA gene, complete sequence; and internal transcribed spacer 2, partial sequence	974	974	99%	0.0	94%	EF679721.1	924,81	96,2%
Select seq gb|GU166765.1|	Alcea lavateriflora isolate Ilav02 internal transcribed spacer 1, partial sequence; 5.8S ribosomal RNA gene, complete sequence; and internal transcribed spacer 2, partial sequence	970	970	99%	0.0	94%	GU166765.1	921,01	95,8%
Select seq gb|GU166759.1|	Alcea glabrata isolate Igla03 internal transcribed spacer 1, partial sequence; 5.8S ribosomal RNA gene, complete sequence; and internal transcribed spacer 2, partial sequence	970	970	99%	0.0	94%	GU166759.1	921,01	95,8%
Select seq gb|GU166756.1|	Alcea apterocarpa isolate Iapt02 internal transcribed spacer 1, partial sequence; 5.8S ribosomal RNA gene, complete sequence; and internal transcribed spacer 2, partial sequence	970	970	99%	0.0	94%	GU166756.1	921,01	95,8%
Select seq gb|GU166754.1|	Alcea angulata isolate Iang01 internal transcribed spacer 1, partial sequence; 5.8S ribosomal RNA gene, complete sequence; and internal transcribed spacer 2, partial sequence	970	970	99%	0.0	94%	GU166754.1	921,01	95,8%
Select seq gb|GU166769.1|	Alcea striata isolate Istr01 internal transcribed spacer 1, partial sequence; 5.8S ribosomal RNA gene, complete sequence; and internal transcribed spacer 2, partial sequence	968	968	99%	0.0	94%	GU166769.1	919,11	95,6%
Select seq gb|EF679720.1|	Alcea gorganica voucher PE611 internal transcribed spacer 1, partial sequence; 5.8S ribosomal RNA gene, complete sequence; and internal transcribed spacer 2, partial sequence	968	968	99%	0.0	94%	EF679720.1	919,11	95,6%
Select seq gb|KR150156.1|	Alcea sp. Kh114 internal transcribed spacer 1, partial sequence; 5.8S ribosomal RNA gene gene, complete sequence; and internal transcribed spacer 2 region, partial sequence	798	798	82%	0.0	94%	KR150156.1	914,78	95,2%
Select seq gb|GU166761.1|	Alcea heldreichii isolate Ihel03 internal transcribed spacer 1, partial sequence; 5.8S ribosomal RNA gene, complete sequence; and internal transcribed spacer 2, partial sequence	963	963	99%	0.0	94%	GU166761.1	914,36	95,2%

Select seq gb|EF679717.1|	Alcea aucheri voucher PE617 internal transcribed spacer 1, partial sequence; 5.8S ribosomal RNA gene, complete sequence; and internal transcribed spacer 2, partial sequence	961	961	99% 0.0	94% EF679717.1	912,46	95,0%
Select seq gb|GU166762.1|	Alcea hohenackeri isolate Ihoh01 internal transcribed spacer 1, partial sequence; 5.8S ribosomal RNA gene, complete sequence; and internal transcribed spacer 2, partial sequence	965	965	99% 0.0	93% GU166762.1	906,52	94,3%
Select seq gb|GU166760.1|	Alcea heldreichii isolate Ihel01 internal transcribed spacer 1, partial sequence; 5.8S ribosomal RNA gene, complete sequence; and internal transcribed spacer 2, partial sequence	961	961	99% 0.0	93% GU166760.1	902,76	93,9%
Select seq gb|EF419543.1|	Alcea angulata isolate Iang615 internal transcribed spacer 1, partial sequence; 5.8S ribosomal RNA gene, complete sequence; and internal transcribed spacer 2, partial sequence	959	959	99% 0.0	93% EF419543.1	900,88	93,7%
Select seq gb|GU166755.1|	Alcea apterocarpa isolate Iapt01 internal transcribed spacer 1, partial sequence; 5.8S ribosomal RNA gene, complete sequence; and internal transcribed spacer 2, partial sequence	957	957	99% 0.0	93% GU166755.1	899,00	93,6%
Select seq gb|EF679715.1|	Alcea pallida voucher PE140 internal transcribed spacer 1, partial sequence; 5.8S ribosomal RNA gene, complete sequence; and internal transcribed spacer 2, partial sequence	952	952	99% 0.0	93% EF679715.1	894,30	93,1%
Select seq gb|EF419545.1|	Alcea pallida isolate IAsp0140 internal transcribed spacer 1, partial sequence; 5.8S ribosomal RNA gene, complete sequence; and internal transcribed spacer 2, partial sequence	952	952	99% 0.0	93% EF419545.1	894,30	93,1%
Select seq gb|GU166767.1|	Alcea peduncularis isolate Iped01 internal transcribed spacer 1, partial sequence; 5.8S ribosomal RNA gene, complete sequence; and internal transcribed spacer 2, partial sequence	946	946	99% 0.0	93% GU166767.1	888,67	92,5%
Select seq gb|EF419542.1|	Althaea armeniaca isolate IAar427 internal transcribed spacer 1, partial sequence; 5.8S ribosomal RNA gene, complete sequence; and internal transcribed spacer 2, partial sequence	795	795	100% 0.0	89% EF419542.1	707,55	73,6%
Select seq gb|EF679734.1|	Althaea armeniaca voucher PE427 internal transcribed spacer 1, partial sequence; 5.8S ribosomal RNA gene, complete sequence; and internal transcribed spacer 2, partial sequence	793	793	100% 0.0	89% EF679734.1	705,77	73,4%
Select seq gb|EF679733.1|	Althaea officinalis voucher PE512 internal transcribed spacer 1, partial sequence; 5.8S ribosomal RNA gene, complete sequence; and internal transcribed spacer 2, partial sequence	774	774	99% 0.0	88% EF679733.1	688,00	71,6%
Select seq gb|EF419539.1|	Althaea cannabina isolate IAca594 internal transcribed spacer 1, partial sequence; 5.8S ribosomal RNA gene, complete sequence; and internal transcribed spacer 2, partial sequence	774	774	99% 0.0	88% EF419539.1	688,00	71,6%
Select seq gb|EF419536.1|	Althaea officinalis isolate IAof604 internal transcribed spacer 1, partial sequence; 5.8S ribosomal RNA gene, complete sequence; and internal transcribed spacer 2, partial sequence	774	774	99% 0.0	88% EF419536.1	688,00	71,6%
Select seq gb|EF419537.1|	Althaea officinalis isolate IAof0330 internal transcribed spacer 1, partial sequence; 5.8S ribosomal RNA gene, complete sequence; and internal transcribed spacer 2, partial sequence	769	769	99% 0.0	88% EF419537.1	683,56	71,1%
Select seq gb|EF419540.1|	Althaea cannabina isolate IAca0345 internal transcribed spacer 1, partial sequence; 5.8S ribosomal RNA gene, complete sequence; and internal transcribed spacer 2, partial sequence	767	767	99% 0.0	88% EF419540.1	681,78	70,9%
Select seq gb|AF303026.1|	Althaea officinalis internal transcribed spacer 1, partial sequence; 5.8S ribosomal RNA gene, complete sequence; and internal transcribed spacer 2, partial sequence	767	767	99% 0.0	88% AF303026.1	681,78	70,9%
Select seq gb|AY591833.1|	Kearnemalvastrum lacteum internal transcribed spacer 1, 5.8S ribosomal RNA gene, and internal transcribed spacer 2, complete sequence	699	699	99% 0.0	86% AY591833.1	607,21	63,2%
Select seq gb|EF419547.1|	Anisodontea malvastroides internal transcribed spacer 1, partial sequence; 5.8S ribosomal RNA gene, complete sequence; and internal transcribed spacer 2, partial sequence	693	693	99% 0.0	86% EF419547.1	602,00	62,6%
Select seq gb|AY172190.1|	Modiola caroliniana internal transcribed spacer 1, 5.8S ribosomal RNA gene, and internal transcribed spacer 2, complete sequence	691	691	99% 0.0	86% AY172190.1	600,26	62,5%
Select seq gb|AY172229.1|	Andeimalva spiciformis internal transcribed spacer 1, 5.8S ribosomal RNA gene, and internal transcribed spacer 2, complete sequence	688	688	99% 0.0	86% AY172229.1	597,66	62,2%
Select seq gb|AY172219.1|	Andeimalva machupicchensis internal transcribed spacer 1, 5.8S ribosomal RNA gene, and internal transcribed spacer 2, complete sequence	684	684	99% 0.0	86% AY172219.1	594,18	61,8%
Select seq gb|AY172191.1|	Modiolastrum lateritium internal transcribed spacer 1, 5.8S ribosomal RNA gene, and internal transcribed spacer 2, complete sequence	680	680	99% 0.0	86% AY172191.1	590,71	61,5%
Select seq gb|AY172220.1|	Andeimalva mandonii internal transcribed spacer 1, 5.8S ribosomal RNA gene, and internal transcribed spacer 2, complete sequence	678	678	99% 0.0	86% AY172220.1	588,97	61,3%
Select seq gb|AY591834.1|	Kearnemalvastrum sp. Ventura A. 816 internal transcribed spacer 1, 5.8S ribosomal RNA gene, and internal transcribed spacer 2, complete sequence	675	675	99% 0.0	86% AY591834.1	586,36	61,0%
Select seq gb|AY591820.1|	Calyculogygas uruguayensis internal transcribed spacer 1, 5.8S ribosomal RNA gene, and internal transcribed spacer 2, complete sequence	665	665	99% 0.0	85% AY591820.1	570,96	59,4%
Select seq gb|AY591810.1|	Acaulimalva dryadifolia internal transcribed spacer 1, 5.8S ribosomal RNA gene, and internal transcribed spacer 2, complete sequence	660	660	99% 0.0	85% AY591810.1	566,67	59,0%
Select seq gb|AY591809.1|	Acaulimalva alismatifolia internal transcribed spacer 1, 5.8S ribosomal RNA gene, and internal transcribed spacer 2, complete sequence	660	660	99% 0.0	85% AY591809.1	566,67	59,0%

Select seq gb|AY591839.1|	Malacothamnus fasciculatus internal transcribed spacer 1, 5.8S ribosomal RNA gene, and internal transcribed spacer 2, complete sequence	640	640	98%	1,00E-179	85%	AY591839.1	555,10	57,8%
Select seq gb|AY172192.1|	Sphaeralcea angustifolia internal transcribed spacer 1, 5.8S ribosomal RNA gene, and internal transcribed spacer 2, complete sequence	643	643	99%	1,00E-180	85%	AY172192.1	552,07	57,4%
Select seq gb|AF271166.1|AF271166	Iliamna bakeri isolate 2McCloud internal transcribed spacer 1, 5.8S ribosomal RNA gene, and internal transcribed spacer 2, complete sequence	643	643	99%	1,00E-180	85%	AF271166.1	552,07	57,4%
Select seq gb|HQ632292.1|	Palaua dissecta voucher Schneider (et al.) 2978 (FR) clone 2978e internal transcribed spacer 1, partial sequence; 5.8S ribosomal RNA gene, complete sequence; and internal transcribed spacer 2, partial sequence	640	640	99%	1,00E-179	85%	HQ632292.1	549,49	57,2%
Select seq gb|AY172199.1|	Andeimalva chilensis internal transcribed spacer 1, 5.8S ribosomal RNA gene, and internal transcribed spacer 2, complete sequence	640	640	99%	1,00E-179	85%	AY172199.1	549,49	57,2%
Select seq emb|AJ512142.1|	Sphaeralcea ambigua 5.8S rRNA gene, internal transcribed spacer 1 (ITS1) and internal transcribed spacer 2 (ITS2), specimen voucher Andreasen 291 (UC)	640	640	99%	1,00E-179	85%	AJ512142.1	549,49	57,2%
Select seq gb|HQ632306.1|	Palaua dissecta voucher Schneider (et al.) 2771 (FR) clone 2771e internal transcribed spacer 1, partial sequence; 5.8S ribosomal RNA gene, complete sequence; and internal transcribed spacer 2, partial sequence	638	638	99%	5,00E-179	85%	HQ632306.1	547,78	57,0%
Select seq gb|DQ156307.1|	Palaua velutina voucher Schneider & Huertas 2749 internal transcribed spacer 1, 5.8S ribosomal RNA gene, and internal transcribed spacer 2, complete sequence	634	634	99%	7,00E-178	85%	DQ156307.1	544,34	56,6%
Select seq emb|AJ304940.1|	Napaea dioica 5.8S rRNA gene, internal transcribed spacer 1 (ITS1) and internal transcribed spacer 2 (ITS2)	641	641	99%	4,00E-180	84%	AJ304940.1	543,88	56,6%
Select seq gb|AY591842.1|	Malvastrum americanum internal transcribed spacer 1, 5.8S ribosomal RNA gene, and internal transcribed spacer 2, complete sequence	632	632	99%	2,00E-177	85%	AY591842.1	542,63	56,5%
Select seq gb|HQ632358.1|	Palaua dissecta voucher Schneider (et al.) 2888 (FR) clone 2888g internal transcribed spacer 1, partial sequence; 5.8S ribosomal RNA gene, complete sequence; and internal transcribed spacer 2, partial sequence	634	634	99%	7,00E-178	84%	HQ632358.1	537,94	56,0%
Select seq gb|HQ632324.1|	Palaua mollendoensis voucher Schneider (et al.) 2985 (FR) clone 2985a internal transcribed spacer 1, partial sequence; 5.8S ribosomal RNA gene, complete sequence; and internal transcribed spacer 2, partial sequence	634	634	99%	7,00E-178	84%	HQ632324.1	537,94	56,0%
Select seq gb|HQ632303.1|	Palaua sp. Schneider et al. 2777 voucher Schneider (et al.) 2777 (FR) clone 2777j internal transcribed spacer 1, partial sequence; 5.8S ribosomal RNA gene, complete sequence; and internal transcribed spacer 2, partial sequence	634	634	99%	7,00E-178	84%	HQ632303.1	537,94	56,0%
Select seq gb|HQ632285.1|	Palaua dissecta voucher Schneider (et al.) 2978 (FR) clone 2978d internal transcribed spacer 1, partial sequence; 5.8S ribosomal RNA gene, complete sequence; and internal transcribed spacer 2, partial sequence	634	634	99%	7,00E-178	84%	HQ632285.1	537,94	56,0%
Select seq gb|HQ632269.1|	Palaua cf. dissecta Schneider et al. 2802 voucher Schneider (et al.) 2802 (FR) clone 2802a internal transcribed spacer 1, partial sequence; 5.8S ribosomal RNA gene, complete sequence; and internal transcribed spacer 2, partial sequence	634	634	99%	7,00E-178	84%	HQ632269.1	537,94	56,0%
Select seq gb|DQ156306.1|	Palaua pusilla voucher Schneider & Huertas 2978 internal transcribed spacer 1, 5.8S ribosomal RNA gene, and internal transcribed spacer 2, complete sequence	634	634	99%	7,00E-178	84%	DQ156306.1	537,94	56,0%
Select seq gb|DQ156300.1|	Palaua malvifolia voucher Schneider & Huertas 2753 internal transcribed spacer 1, 5.8S ribosomal RNA gene, and internal transcribed spacer 2, complete sequence	634	634	99%	7,00E-178	84%	DQ156300.1	537,94	56,0%
Select seq gb|AY172200.1|	Tarasa albertii internal transcribed spacer 1, 5.8S ribosomal RNA gene, and internal transcribed spacer 2, complete sequence	634	634	99%	7,00E-178	84%	AY172200.1	537,94	56,0%
Select seq emb|AJ849674.1|	Eremalche parryi ITS1, 5.8S rRNA gene and ITS2, specimen voucher E. A. Cypher 2001-005 no. 25 (UPS)	634	634	99%	7,00E-178	84%	AJ849674.1	537,94	56,0%
Select seq emb|AJ304938.1|	Eremalche parryi 5.8S rRNA gene, internal transcribed spacer 1 (ITS1) and internal transcribed spacer 2 (ITS2)	634	634	99%	7,00E-178	84%	AJ304938.1	537,94	56,0%
Select seq gb|AY217204.1|	Tarasa albertii internal transcribed spacer 1, 5.8S ribosomal RNA gene, and internal transcribed spacer 2, complete sequence	632	632	99%	2,00E-177	84%	AY217204.1	536,24	55,8%
Select seq emb|AJ849672.1|	Eremalche parryi ITS1, 5.8S rRNA gene and ITS2, specimen voucher E. Cypher 2001-001 no. 23 (UPS)	632	632	99%	2,00E-177	84%	AJ849672.1	536,24	55,8%
Select seq gb|HQ632357.1|	Palaua dissecta voucher Schneider (et al.) 2888 (FR) clone 2888f internal transcribed spacer 1, partial sequence; 5.8S ribosomal RNA gene, complete sequence; and internal transcribed spacer 2, partial sequence	628	628	99%	3,00E-176	84%	HQ632357.1	532,85	55,4%
Select seq gb|HQ632356.1|	Palaua dissecta voucher Schneider (et al.) 2888 (FR) clone 2888d internal transcribed spacer 1, partial sequence; 5.8S ribosomal RNA gene, complete sequence; and internal transcribed spacer 2, partial sequence	628	628	99%	3,00E-176	84%	HQ632356.1	532,85	55,4%
Select seq gb|HQ632355.1|	Palaua mollendoensis voucher Schneider (et al.) 2813 (FR) clone 2813d internal transcribed spacer 1, partial sequence; 5.8S ribosomal RNA gene, complete sequence; and internal transcribed spacer 2, partial sequence	628	628	99%	3,00E-176	84%	HQ632355.1	532,85	55,4%
Select seq gb|HQ632351.1|	Palaua dissecta voucher Schneider (et al.) 2888 (FR) clone 2888a internal transcribed spacer 1, partial sequence; 5.8S ribosomal RNA gene, complete sequence; and internal transcribed spacer 2, partial sequence	628	628	99%	3,00E-176	84%	HQ632351.1	532,85	55,4%
Select seq gb|HQ632348.1|	Palaua mollendoensis voucher Schneider (et al.) 2985 (FR) clone 2985j internal transcribed spacer 1, partial sequence; 5.8S ribosomal RNA gene, complete sequence; and internal transcribed spacer 2, partial sequence	628	628	99%	3,00E-176	84%	HQ632348.1	532,85	55,4%

Select seq gb|HQ632343.1|	Palaua dissecta voucher Schneider (et al.) 2836 (FR) clone 2836d internal transcribed spacer 1, partial sequence; 5.8S ribosomal RNA gene, complete sequence; and internal transcribed spacer 2, partial sequence	628	628	99%	3,00E-176	84% HQ632343.1	532,85	55,4%
Select seq gb|HQ632340.1|	Palaua mollendoensis voucher Schneider (et al.) 2985 (FR) clone 2985k internal transcribed spacer 1, partial sequence; 5.8S ribosomal RNA gene, complete sequence; and internal transcribed spacer 2, partial sequence	628	628	99%	3,00E-176	84% HQ632340.1	532,85	55,4%
Select seq gb|HQ632332.1|	Palaua dissecta voucher Schneider (et al.) 2771 (FR) clone 2771k internal transcribed spacer 1, partial sequence; 5.8S ribosomal RNA gene, complete sequence; and internal transcribed spacer 2, partial sequence	628	628	99%	3,00E-176	84% HQ632332.1	532,85	55,4%
Select seq gb|HQ632329.1|	Palaua dissecta voucher Schneider (et al.) 2771 (FR) clone 2771j internal transcribed spacer 1, partial sequence; 5.8S ribosomal RNA gene, complete sequence; and internal transcribed spacer 2, partial sequence	628	628	99%	3,00E-176	84% HQ632329.1	532,85	55,4%
Select seq gb|HQ632308.1|	Palaua dissecta x Palaua tomentosa voucher Schneider (et al.) 2997 (FR) clone 2997e internal transcribed spacer 1, partial sequence; 5.8S ribosomal RNA gene, complete sequence; and internal transcribed spacer 2, partial sequence	628	628	99%	3,00E-176	84% HQ632308.1	532,85	55,4%
Select seq gb|HQ632318.1|	Palaua mollendoensis voucher Schneider (et al.) 2813 (FR) clone 2813b internal transcribed spacer 1, partial sequence; 5.8S ribosomal RNA gene, complete sequence; and internal transcribed spacer 2, partial sequence	628	628	99%	3,00E-176	84% HQ632318.1	532,85	55,4%
Select seq gb|HQ632296.1|	Palaua dissecta voucher Schneider (et al.) 2807 (FR) internal transcribed spacer 1, partial sequence; 5.8S ribosomal RNA gene, complete sequence; and internal transcribed spacer 2, partial sequence	628	628	99%	3,00E-176	84% HQ632296.1	532,85	55,4%
Select seq gb|HQ632286.1|	Palaua sp. Schneider et al. 2777 voucher Schneider (et al.) 2777 (FR) clone 2777b internal transcribed spacer 1, partial sequence; 5.8S ribosomal RNA gene, complete sequence; and internal transcribed spacer 2, partial sequence	628	628	99%	3,00E-176	84% HQ632286.1	532,85	55,4%
Select seq gb|HQ632284.1|	Palaua dissecta voucher Schneider (et al.) 2978 (FR) clone 2978b internal transcribed spacer 1, partial sequence; 5.8S ribosomal RNA gene, complete sequence; and internal transcribed spacer 2, partial sequence	628	628	99%	3,00E-176	84% HQ632284.1	532,85	55,4%
Select seq gb|HQ632200.1|	Palaua moschata voucher Schneider (et al.) 3112 (FR) internal transcribed spacer 1, partial sequence; 5.8S ribosomal RNA gene, complete sequence; and internal transcribed spacer 2, partial sequence	628	628	99%	3,00E-176	84% HQ632200.1	532,85	55,4%
Select seq gb|HQ632180.1|	Palaua cf. tomentosa Schneider et al. 2984 voucher Schneider (et al.) 2984 (FR) clone 2984k internal transcribed spacer 1, partial sequence; 5.8S ribosomal RNA gene, complete sequence; and internal transcribed spacer 2, partial sequence	628	628	99%	3,00E-176	84% HQ632180.1	532,85	55,4%
Select seq gb|HQ632176.1|	Palaua cf. tomentosa Schneider et al. 2984 voucher Schneider (et al.) 2984 (FR) clone 2984b internal transcribed spacer 1, partial sequence; 5.8S ribosomal RNA gene, complete sequence; and internal transcribed spacer 2, partial sequence	628	628	99%	3,00E-176	84% HQ632176.1	532,85	55,4%
Select seq emb|AJ849670.1|	Eremalche parryi ITS1, 5.8S rRNA gene and ITS2, specimen voucher E. Cypher 99-12 no. 15 (UPS)	628	628	99%	3,00E-176	84% AJ849670.1	532,85	55,4%
Select seq emb|AJ849667.1|	Eremalche parryi ITS1, 5.8S rRNA gene and ITS2, specimen voucher E. Cypher 99-10 no. 11 (UPS)	627	627	99%	1,00E-175	84% AJ849667.1	532,00	55,4%
Select seq gb|HQ632311.1|	Palaua dissecta voucher Schneider (et al.) 2778 (FR) internal transcribed spacer 1, partial sequence; 5.8S ribosomal RNA gene, complete sequence; and internal transcribed spacer 2, partial sequence	625	625	99%	4,00E-175	84% HQ632311.1	530,30	55,2%
Select seq emb|AJ849666.1|	Eremalche parryi ITS1, 5.8S rRNA gene and ITS2, specimen voucher E. Cypher 99-2 no. 10 (UPS)	625	625	99%	4,00E-175	84% AJ849666.1	530,30	55,2%
Select seq emb|AJ849668.1|	Eremalche parryi ITS1, 5.8S rRNA gene and ITS2, specimen voucher E. Cypher 99-10 no. 12 (UPS)	623	623	99%	1,00E-174	84% AJ849668.1	528,61	55,0%
Select seq emb|AJ416061.1|	Eremalche exilis 5.8S rRNA gene, internal transcribed spacer 1 (ITS1) and internal transcribed spacer 2 (ITS2), specimen voucher Heckard 4508	623	623	99%	1,00E-174	84% AJ416061.1	528,61	55,0%
Select seq gb|HQ632310.1|	Palaua dissecta voucher Schneider (et al.) 2824a (FR) internal transcribed spacer 1, partial sequence; 5.8S ribosomal RNA gene, complete sequence; and internal transcribed spacer 2, partial sequence	621	621	99%	5,00E-174	84% HQ632310.1	526,91	54,8%

Select for downloading or viewing reports	Kh062_trnL Description	Max score	Total score	Query cover	E value	Ident	Accession	(Ident/Cover)* Max score	Deviation from top hit
Select seq emb|AJ505541.1|	Mentha suaveolens plastid trnL-trnF intergenic spacer, specimen voucher cult., K-1970-3169 (K)	1212	1212	99%	0.0	99%	AJ505541.1	1212,00	100,0%
Select seq gb|GU381518.1|	Mentha spicata voucher M:Braeuchler 2377 tRNA-Leu (trnL) gene and trnL-trnF intergenic spacer, partial sequence; chloroplast	1210	1210	99%	0.0	99%	GU381518.1	1210,00	99,8%
Select seq gb|KC414276.1|	Mentha canadensis isolate 511190001 tRNA-Leu (trnL) gene and trnL-trnF intergenic spacer, partial sequence; chloroplast	1221	1221	100%	0.0	99%	KC414276.1	1208,79	99,7%
Select seq gb|JQ669054.1|	Mentha spicata voucher J. Walker 2566 tRNA-Leu (trnL) gene and trnL-trnF intergenic spacer, partial sequence; plastid	1192	1192	98%	0.0	99%	JQ669054.1	1204,16	99,4%
Select seq gb|AY618521.1|	Mentha longifolia tRNA-Leu (trnL) gene and trnL-trnF intergenic spacer, partial sequence; chloroplast	1192	1192	98%	0.0	99%	AY618521.1	1204,16	99,4%
Select seq gb|JQ669052.1|	Mentha spicata subsp. condensata voucher Riina 1575 tRNA-Leu (trnL) gene and trnL-trnF intergenic spacer, partial sequence; plastid	1188	1188	98%	0.0	99%	JQ669052.1	1200,12	99,0%
Select seq gb|GU381519.1|	Mentha suaveolens voucher M:Braeuchler 2380 tRNA-Leu (trnL) gene and trnL-trnF intergenic spacer, partial sequence; chloroplast	1194	1194	99%	0.0	99%	GU381519.1	1194,00	98,5%
Select seq gb|AY570461.1|	Mentha spicata voucher JBW 2566 tRNA-Leu and trnL-trnF intergenic spacer, partial sequence; chloroplast	1203	1203	100%	0.0	99%	AY570461.1	1190,97	98,3%
Select seq gb|AY618526.1|	Mentha suaveolens tRNA-Leu (trnL) gene and trnL-trnF intergenic spacer, partial sequence; chloroplast	1175	1175	98%	0.0	99%	AY618526.1	1186,99	97,9%
Select seq gb|JQ669053.1|	Mentha pulegium voucher Riina 1574 tRNA-Leu (trnL) gene and trnL-trnF intergenic spacer, partial sequence; plastid	1171	1171	98%	0.0	99%	JQ669053.1	1182,95	97,6%
Select seq gb|AY618524.1|	Mentha satureioides tRNA-Leu (trnL) gene and trnL-trnF intergenic spacer, partial sequence; chloroplast	1170	1170	98%	0.0	99%	AY618524.1	1181,94	97,5%
Select seq gb|AY618517.1|	Mentha cunninghamii tRNA-Leu (trnL) gene and trnL-trnF intergenic spacer, partial sequence; chloroplast	1170	1170	98%	0.0	99%	AY618517.1	1181,94	97,5%
Select seq gb|AY618514.1|	Mentha australis tRNA-Leu (trnL) gene and trnL-trnF intergenic spacer, partial sequence; chloroplast	1170	1170	98%	0.0	99%	AY618514.1	1181,94	97,5%
Select seq gb|GU381522.1|	Mentha cervina voucher M:Braeuchler 2394 tRNA-Leu (trnL) gene and trnL-trnF intergenic spacer, partial sequence; chloroplast	1177	1177	99%	0.0	99%	GU381522.1	1177,00	97,1%
Select seq gb|AY618522.1|	Mentha pulegium tRNA-Leu (trnL) gene and trnL-trnF intergenic spacer, partial sequence; chloroplast	1164	1164	98%	0.0	99%	AY618522.1	1175,88	97,0%
Select seq gb|AY618518.1|	Mentha diemenica tRNA-Leu (trnL) gene and trnL-trnF intergenic spacer, partial sequence; chloroplast	1164	1164	98%	0.0	99%	AY618518.1	1175,88	97,0%
Select seq gb|AY618516.1|	Mentha cervina tRNA-Leu (trnL) gene and trnL-trnF intergenic spacer, partial sequence; chloroplast	1160	1160	98%	0.0	99%	AY618516.1	1171,84	96,7%
Select seq gb|KR150193.1|	Mentha sp. 2 Kh53 trnL-trnF intergenic spacer region, partial sequence; chloroplast	1122	1122	95%	0.0	99%	KR150193.1	1169,24	96,5%
Select seq gb|AY618513.1|	Mentha arvensis tRNA-Leu (trnL) gene and trnL-trnF intergenic spacer, partial sequence; chloroplast	1157	1157	98%	0.0	99%	AY618513.1	1168,81	96,4%
Select seq gb|FJ593456.1|	Mentha sp. JSZ-2009a tRNA-Leu (trnL) gene and trnL-trnF intergenic spacer, partial sequence; chloroplast	1175	1175	100%	0.0	99%	FJ593456.1	1163,25	96,0%
Select seq gb|JF301387.1|	Mentha arvensis voucher B. Drew 82 tRNA-Leu (trnL) gene and trnL-trnF intergenic spacer, partial sequence; chloroplast	1138	1138	96%	0.0	98%	JF301387.1	1161,71	95,9%
Select seq gb|AY618515.1|	Mentha canadensis tRNA-Leu (trnL) gene and trnL-trnF intergenic spacer, partial sequence; chloroplast	1151	1151	98%	0.0	98%	AY618515.1	1151,00	95,0%
Select seq gb|AY618512.1|	Mentha aquatica tRNA-Leu (trnL) gene and trnL-trnF intergenic spacer, partial sequence; chloroplast	1151	1151	98%	0.0	98%	AY618512.1	1151,00	95,0%
Select seq gb|DQ667514.1|	Mentha arvensis isolate x424 tRNA-Leu (trnL) gene and trnL-trnF intergenic spacer, partial sequence; chloroplast	1170	1170	100%	0.0	98%	DQ667514.1	1146,60	94,6%
Select seq gb|GU381481.1|	Micromeria cf. madagascariensis Morawetz 205 tRNA-Leu (trnL) gene and trnL-trnF intergenic spacer, partial sequence; chloroplast	1155	1155	99%	0.0	98%	GU381481.1	1143,33	94,3%
Select seq gb|GU381485.1|	Micromeria sphaerophylla voucher E:Lewis et al 1064 tRNA-Leu (trnL) gene and trnL-trnF intergenic spacer, partial sequence; chloroplast	1153	1153	99%	0.0	98%	GU381485.1	1141,35	94,2%

Select seq gb|GU381521.1|	Mentha pulegium voucher M:Braeuchler 2300 tRNA-Leu (trnL) gene and trnL-trnF intergenic spacer, partial sequence; chloroplast	1146	1146	99%	0.0	98%	GU381521.1	1134,42	93,6%
Select seq gb|AY506610.1|	Mentha suaveolens tRNA-Leu (trnL) gene and trnL-trnF intergenic spacer, partial sequence; chloroplast	1094	1094	96%	0.0	98%	AY506610.1	1116,79	92,1%
Select seq gb|GU381484.1|	Micromeria flagellaris voucher E:van der Werff & McPherson 13570 tRNA-Leu (trnL) gene and trnL-trnF intergenic spacer, partial sequence; chloroplast	1127	1127	99%	0.0	98%	GU381484.1	1115,62	92,0%
Select seq gb|GU381483.1|	Micromeria flagellaris voucher E:Clement et al 2140 tRNA-Leu (trnL) gene and trnL-trnF intergenic spacer, partial sequence; chloroplast	1127	1127	99%	0.0	98%	GU381483.1	1115,62	92,0%
Select seq gb|AY618523.1|	Mentha requienii tRNA-Leu (trnL) gene and trnL-trnF intergenic spacer, partial sequence; chloroplast	1114	1114	97%	0.0	97%	AY618523.1	1114,00	91,9%
Select seq gb|AY618519.1|	Mentha gattefossei tRNA-Leu (trnL) gene and trnL-trnF intergenic spacer, partial sequence; chloroplast	1120	1120	98%	0.0	97%	AY618519.1	1108,57	91,5%
Select seq gb|JF301401.1|	Thymbra capitata voucher UCBG 96.0817 tRNA-Leu (trnL) gene and trnL-trnF intergenic spacer, partial sequence; chloroplast	1090	1090	96%	0.0	97%	JF301401.1	1101,35	90,9%
Select seq gb|GU381513.1|	Clinopodium nepalense voucher BM<GBR-LONDON>:Mikage et al. 9550294 tRNA-Leu (trnL) gene and trnL-trnF intergenic spacer, partial sequence; chloroplast	1083	1083	96%	0.0	97%	GU381513.1	1094,28	90,3%
Select seq gb|GU381629.1|	Thymbra capitata voucher M:Braeuchler 2518 tRNA-Leu (trnL) gene and trnL-trnF intergenic spacer, partial sequence; chloroplast	1116	1116	99%	0.0	97%	GU381629.1	1093,45	90,2%
Select seq gb|GU381632.1|	Thymbra spicata voucher M:Braeuchler 4548 tRNA-Leu (trnL) gene and trnL-trnF intergenic spacer, partial sequence; chloroplast	1112	1112	99%	0.0	97%	GU381632.1	1089,54	89,9%
Select seq gb|GU381517.1|	Clinopodium barosmum voucher BM<GBR-LONDON>:McLaren N193 tRNA-Leu (trnL) gene and trnL-trnF intergenic spacer, partial sequence; chloroplast	1110	1110	99%	0.0	97%	GU381517.1	1087,58	89,7%
Select seq gb|GU381516.1|	Clinopodium wardii voucher BM<GBR-LONDON>:Ludlow et al. 14234 tRNA-Leu (trnL) gene and trnL-trnF intergenic spacer, partial sequence; chloroplast	1110	1110	99%	0.0	97%	GU381516.1	1087,58	89,7%
Select seq gb|GU381515.1|	Clinopodium hydaspidis voucher BM<GBR-LONDON>:Mohd 133 tRNA-Leu (trnL) gene and trnL-trnF intergenic spacer, partial sequence; chloroplast	1110	1110	99%	0.0	97%	GU381515.1	1087,58	89,7%
Select seq gb|GU381514.1|	Clinopodium nepalense voucher FR:Stainton 6024 tRNA-Leu (trnL) gene and trnL-trnF intergenic spacer, partial sequence; chloroplast	1110	1110	99%	0.0	97%	GU381514.1	1087,58	89,7%
Select seq gb|GU381511.1|	Clinopodium piperitum voucher BM<GBR-LONDON>:Vickery 454 tRNA-Leu (trnL) gene and trnL-trnF intergenic spacer, partial sequence; chloroplast	1110	1110	99%	0.0	97%	GU381511.1	1087,58	89,7%
Select seq gb|AY840207.1|	Thymbra spicata tRNA-Leu (trnL) gene and trnL-trnF intergenic spacer, partial sequence; chloroplast	1110	1110	99%	0.0	97%	AY840207.1	1087,58	89,7%
Select seq gb|GU381495.1|	Killickia pilosa voucher M:Braeuchler 3810 tRNA-Leu (trnL) gene and trnL-trnF intergenic spacer, partial sequence; chloroplast	1086	1086	97%	0.0	97%	GU381495.1	1086,00	89,6%
Select seq gb|GU381627.1|	Thymbra sintenisii subsp. isaurica voucher E:Goener 12628 tRNA-Leu (trnL) gene and trnL-trnF intergenic spacer, partial sequence; chloroplast	1105	1105	99%	0.0	97%	GU381627.1	1082,68	89,3%
Select seq gb|GU381512.1|	Clinopodium piperitum voucher E:Stainton 7320 tRNA-Leu (trnL) gene and trnL-trnF intergenic spacer, partial sequence; chloroplast	1105	1105	99%	0.0	97%	GU381512.1	1082,68	89,3%
Select seq gb|GU381496.1|	Killickia pilosa voucher M:Braeuchler 3832 tRNA-Leu (trnL) gene and trnL-trnF intergenic spacer, partial sequence; chloroplast	1101	1101	99%	0.0	97%	GU381496.1	1078,76	89,0%
Select seq gb|GU381493.1|	Killickia grandiflora voucher M:Braeuchler 3811 tRNA-Leu (trnL) gene and trnL-trnF intergenic spacer, partial sequence; chloroplast	1101	1101	99%	0.0	97%	GU381493.1	1078,76	89,0%
Select seq gb|KR150198.1|	Ziziphora sp. 3 Kh112 trnL-trnF intergenic spacer region, partial sequence; chloroplast	1088	1088	98%	0.0	97%	KR150198.1	1076,90	88,9%
Select seq gb|AY840198.1|	Micromeria graeca tRNA-Leu (trnL) gene and trnL-trnF intergenic spacer, partial sequence; chloroplast	1098	1098	99%	0.0	97%	AY840198.1	1075,82	88,8%
Select seq gb|KR150233.1|	Thymus sp. 6 Kh133 trnL-trnF intergenic spacer region, partial sequence; chloroplast	1096	1096	99%	0.0	97%	KR150233.1	1073,86	88,6%
Select seq gb|GU381523.1|	Micromeria cymuligera voucher JE:Haussknecht 1265 tRNA-Leu (trnL) gene and trnL-trnF intergenic spacer, partial sequence; chloroplast	1096	1096	99%	0.0	97%	GU381523.1	1073,86	88,6%
Select seq gb|DQ667501.1|	Ziziphora taurica isolate x262 tRNA-Leu (trnL) gene and trnL-trnF intergenic spacer, partial sequence; chloroplast	1096	1096	99%	0.0	97%	DQ667501.1	1073,86	88,6%
Select seq gb|JQ669021.1|	Clinopodium acinos voucher Judziewicz 14160 tRNA-Leu (trnL) gene and trnL-trnF intergenic spacer, partial sequence; plastid	1083	1083	98%	0.0	97%	JQ669021.1	1071,95	88,4%
Select seq gb|GU381635.1|	Thymus broussonetii subsp. hannonis voucher MSB:Podlech 142 tRNA-Leu (trnL) gene and trnL-trnF intergenic spacer, partial sequence; chloroplast	1094	1094	99%	0.0	97%	GU381635.1	1071,90	88,4%

Select seq gb|GU381633.1|	Thymus caespititius voucher M:Heubl s.n. tRNA-Leu (trnL) gene and trnL-trnF intergenic spacer, partial sequence; chloroplast	1094	1094	99%	0.0	97%	GU381633.1	1071,90	88,4%
Select seq gb|GU381510.1|	Ziziphora pamiroalaica voucher C:Murray et al. 10090 tRNA-Leu (trnL) gene and trnL-trnF intergenic spacer, partial sequence; chloroplast	1094	1094	99%	0.0	97%	GU381510.1	1071,90	88,4%
Select seq gb|GU381505.1|	Clinopodium troodi voucher W:Davis 1856 tRNA-Leu (trnL) gene and trnL-trnF intergenic spacer, partial sequence; chloroplast	1094	1094	99%	0.0	97%	GU381505.1	1071,90	88,4%
Select seq gb|GU381501.1|	Clinopodium graveolens subsp. rotundifolium voucher M:Podlech 47181 tRNA-Leu (trnL) gene and trnL-trnF intergenic spacer, partial sequence; chloroplast	1094	1094	99%	0.0	97%	GU381501.1	1071,90	88,4%
Select seq gb|GU381499.1|	Clinopodium suaveolens voucher M:Erben s.n. tRNA-Leu (trnL) gene and trnL-trnF intergenic spacer, partial sequence; chloroplast	1094	1094	99%	0.0	97%	GU381499.1	1071,90	88,4%
Select seq gb|GU381500.1|	Clinopodium nanum voucher M:Braeuchler 2796 tRNA-Leu (trnL) gene and trnL-trnF intergenic spacer, partial sequence; chloroplast	1081	1081	98%	0.0	97%	GU381500.1	1069,97	88,3%
Select seq gb|GU381489.1|	Killickia lutea voucher NU<ZAF>:Hilliard & Burtt 9876 tRNA-Leu (trnL) gene and trnL-trnF intergenic spacer, partial sequence; chloroplast	1090	1090	99%	0.0	97%	GU381489.1	1067,98	88,1%
Select seq gb|GU381488.1|	Killickia compacta voucher M:Braeuchler 3816 tRNA-Leu (trnL) gene and trnL-trnF intergenic spacer, partial sequence; chloroplast	1090	1090	99%	0.0	97%	GU381488.1	1067,98	88,1%
Select seq gb|KR150238.1|	Ziziphora sp. 1 Kh75 trnL-trnF intergenic spacer region, partial sequence; chloroplast	1099	1099	100%	0.0	97%	KR150238.1	1066,03	88,0%
Select seq gb|KR150218.1|	Thymus sp. 2 Kh69 trnL-trnF intergenic spacer region, partial sequence; chloroplast	1099	1099	100%	0.0	97%	KR150218.1	1066,03	88,0%
Select seq gb|JX880022.1|	Origanum vulgare subsp. vulgare chloroplast, complete genome	1099	1099	100%	0.0	97%	JX880022.1	1066,03	88,0%
Select seq gb|JQ690290.1|	Origanum elongatum isolate H5_O_elo trnL-trnF intergenic spacer, partial sequence; chloroplast	1099	1099	100%	0.0	97%	JQ690290.1	1066,03	88,0%
Select seq gb|JQ690289.1|	Origanum rotundifolium isolate DNA3_O_rot trnL-trnF intergenic spacer, partial sequence; chloroplast	1099	1099	100%	0.0	97%	JQ690289.1	1066,03	88,0%
Select seq emb|AJ505543.1|	Origanum vulgare plastid trnL-trnF intergenic spacer, specimen voucher cult., K-000-69-19317, chase 13334 (K)	1099	1099	100%	0.0	97%	AJ505543.1	1066,03	88,0%
Select seq gb|GU381640.1|	Argantoniella salzmannii voucher M:Barra et al. 2673GL tRNA-Leu (trnL) gene and trnL-trnF intergenic spacer, partial sequence; chloroplast	1088	1088	99%	0.0	97%	GU381640.1	1066,02	88,0%
Select seq gb|GU381507.1|	Ziziphora tenuior voucher MSB:Fayvush et al. 03-1503 tRNA-Leu (trnL) gene and trnL-trnF intergenic spacer, partial sequence; chloroplast	1088	1088	99%	0.0	97%	GU381507.1	1066,02	88,0%
Select seq gb|GU381497.1|	Clinopodium acinos voucher M:Podlech 50287 tRNA-Leu (trnL) gene and trnL-trnF intergenic spacer, partial sequence; chloroplast	1088	1088	99%	0.0	97%	GU381497.1	1066,02	88,0%
Select seq gb|AY840202.1|	Origanum vulgare tRNA-Leu (trnL) gene and trnL-trnF intergenic spacer, partial sequence; chloroplast	1088	1088	99%	0.0	97%	AY840202.1	1066,02	88,0%
Select seq gb|JQ669056.1|	Micromeria juliana voucher Forbes s.n. tRNA-Leu (trnL) gene and trnL-trnF intergenic spacer, partial sequence; plastid	1088	1088	98%	0.0	96%	JQ669056.1	1065,80	87,9%
Select seq gb|GU381625.1|	Pentapleura subulifera voucher W:Rechinger 12085 tRNA-Leu (trnL) gene and trnL-trnF intergenic spacer, partial sequence; chloroplast	1086	1086	99%	0.0	97%	GU381625.1	1064,06	87,8%
Select seq gb|GU381628.1|	Thymbra calostachya voucher M:Ulrich s.n. tRNA-Leu (trnL) gene and trnL-trnF intergenic spacer, partial sequence; chloroplast	1085	1085	99%	0.0	97%	GU381628.1	1063,08	87,7%
Select seq gb|GU381631.1|	Satureja linearifolia voucher M:Brullo & Furnari s.n. tRNA-Leu (trnL) gene and trnL-trnF intergenic spacer, partial sequence; chloroplast	1083	1083	99%	0.0	97%	GU381631.1	1061,12	87,6%
Select seq gb|GU381528.1|	Cyclotrichium stamineum voucher M:Rechinger 11618 tRNA-Leu (trnL) gene and trnL-trnF intergenic spacer, partial sequence; chloroplast	1081	1081	99%	0.0	97%	GU381528.1	1059,16	87,4%
Select seq gb|AY840192.1|	Micromeria forbesii tRNA-Leu (trnL) gene and trnL-trnF intergenic spacer, partial sequence; chloroplast	1088	1088	99%	0.0	96%	AY840192.1	1055,03	87,0%
Select seq gb|AY840199.1|	Micromeria juliana tRNA-Leu (trnL) gene and trnL-trnF intergenic spacer, partial sequence; chloroplast	1088	1088	99%	0.0	96%	AY840199.1	1055,03	87,0%
Select seq gb|AY840196.1|	Micromeria fruticulosa tRNA-Leu (trnL) gene and trnL-trnF intergenic spacer, partial sequence; chloroplast	1088	1088	99%	0.0	96%	AY840196.1	1055,03	87,0%
Select seq gb|AY840194.1|	Micromeria cristata tRNA-Leu (trnL) gene and trnL-trnF intergenic spacer, partial sequence; chloroplast	1086	1086	99%	0.0	96%	AY840194.1	1053,09	86,9%
Select seq gb|AY840193.1|	Micromeria imbricata tRNA-Leu (trnL) gene and trnL-trnF intergenic spacer, partial sequence; chloroplast	1085	1085	99%	0.0	96%	AY840193.1	1052,12	86,8%

Select seq gb|KR063657.1|	Thymus sibthorpii tRNA-Leu (trnL) gene, partial sequence; trnL-trnF intergenic spacer, complete sequence; and tRNA-Phe (trnF) gene, partial sequence; chloroplast	1094	1094	100% 0.0	96%	KR063657.1	1050,24	86,7%
Select seq gb|KR150229.1|	Thymus sp. 5 Kh16 trnL-trnF intergenic spacer region, partial sequence; chloroplast	1094	1094	100% 0.0	96%	KR150229.1	1050,24	86,7%
Select seq gb|KR150225.1|	Thymus sp. 4 Kh24 trnL-trnF intergenic spacer region, partial sequence; chloroplast	1094	1094	100% 0.0	96%	KR150225.1	1050,24	86,7%
Select seq gb|KR150222.1|	Thymus sp. 3 Kh65 trnL-trnF intergenic spacer region, partial sequence; chloroplast	1094	1094	100% 0.0	96%	KR150222.1	1050,24	86,7%
Select seq gb|EU556532.1|	Thymus amurensis isolate HX tRNA-Leu (trnL) gene and trnL-trnF intergenic spacer, partial sequence; chloroplast	1094	1094	100% 0.0	96%	EU556532.1	1050,24	86,7%
Select seq gb|EU556525.1|	Thymus quinquecostatus isolate CL tRNA-Leu (trnL) gene and trnL-trnF intergenic spacer, partial sequence; chloroplast	1094	1094	100% 0.0	96%	EU556525.1	1050,24	86,7%
Select seq gb|KR150209.1|	Satureja sp. 3 Kh90 trnL-trnF intergenic spacer region, partial sequence; chloroplast	1083	1083	99% 0.0	96%	KR150209.1	1050,18	86,6%
Select seq gb|GU381636.1|	Thymus vulgaris voucher M:Braeuchler 3683 tRNA-Leu (trnL) gene and trnL-trnF intergenic spacer, partial sequence; chloroplast	1083	1083	99% 0.0	96%	GU381636.1	1050,18	86,6%
Select seq gb|KR063656.1|	Satureja pilosa subsp. origanita tRNA-Leu (trnL) gene, partial sequence; trnL-trnF intergenic spacer, complete sequence; and tRNA-Phe (trnF) gene, partial sequence; chloroplast	1090	1090	100% 0.0	96%	KR063656.1	1046,40	86,3%
Select seq gb|AY840195.1|	Micromeria fontanesii tRNA-Leu (trnL) gene and trnL-trnF intergenic spacer, partial sequence; chloroplast	1079	1079	99% 0.0	96%	AY840195.1	1046,30	86,3%
Select seq gb|JQ690293.1|	Origanum dayi isolate H43_O_day trnL-trnF intergenic spacer, partial sequence; chloroplast	1088	1088	100% 0.0	96%	JQ690293.1	1044,48	86,2%
Select seq gb|EU556539.1|	Thymus quinquecostatus isolate ZY tRNA-Leu (trnL) gene and trnL-trnF intergenic spacer, partial sequence; chloroplast	1086	1086	100% 0.0	96%	EU556539.1	1042,56	86,0%
Select seq gb|EU556538.1|	Thymus quinquecostatus isolate ZJ tRNA-Leu (trnL) gene and trnL-trnF intergenic spacer, partial sequence; chloroplast	1086	1086	100% 0.0	96%	EU556538.1	1042,56	86,0%
Select seq gb|EU556533.1|	Thymus quinquecostatus isolate HY tRNA-Leu (trnL) gene and trnL-trnF intergenic spacer, partial sequence; chloroplast	1086	1086	100% 0.0	96%	EU556533.1	1042,56	86,0%
Select seq gb|EU556527.1|	Thymus mongolicus isolate G2 tRNA-Leu (trnL) gene and trnL-trnF intergenic spacer, partial sequence; chloroplast	1086	1086	100% 0.0	96%	EU556527.1	1042,56	86,0%
Select seq gb|EU556526.1|	Thymus quinquecostatus isolate D2 tRNA-Leu (trnL) gene and trnL-trnF intergenic spacer, partial sequence; chloroplast	1086	1086	100% 0.0	96%	EU556526.1	1042,56	86,0%
Select seq gb|EU556531.1|	Thymus amurensis isolate HW tRNA-Leu (trnL) gene and trnL-trnF intergenic spacer, partial sequence; chloroplast	1081	1081	100% 0.0	96%	EU556531.1	1037,76	85,6%

Select for downloading or viewing reports	Kh063_ITS Description	Max score	Total score	Query cover	E value	Ident	Accession	(Ident/Cover)* Max score	Deviation from top hit
Select seq dbj|AB683327.1|	Tanacetum turcomanicum gene for ITS1, 5.8S rRNA and ITS2, complete sequence, specimen_voucher: 20867 (FUMH)	767	767	81%	0.0	97%	AB683327.1	918,51	100,0%
Select seq gb|JF907423.1|	Tripleurospermum maritimum subsp. inodorum voucher OSBU:2005 DE-0-OSN-2005-169 18S ribosomal RNA gene, partial sequence; internal transcribed spacer 1, 5.8S ribosomal RNA gene, and internal transcribed spacer 2, complete sequence; and 26S ribosomal RNA gene, partial sequence	915	915	100%	0.0	96%	JF907423.1	878,40	95,6%
Select seq dbj|AB683287.1|	Tanacetum haradjanii gene for ITS1, 5.8S rRNA and ITS2, complete sequence, specimen_voucher: 16366 (M)	826	826	100%	0.0	93%	AB683287.1	768,18	83,6%
Select seq dbj|AB683267.1|	Tanacetum armenum gene for ITS1, 5.8S rRNA and ITS2, complete sequence, specimen_voucher: (W, 1972-03416)	817	817	99%	0.0	93%	AB683267.1	767,48	83,6%
Select seq gb|AY603230.1|	Achillea clavennae internal transcribed spacer 1, 5.8S ribosomal RNA gene, and internal transcribed spacer 2, complete sequence	817	817	99%	0.0	93%	AY603230.1	767,48	83,6%
Select seq gb|AY603202.1|	Achillea euxina internal transcribed spacer 1, 5.8S ribosomal RNA gene, and internal transcribed spacer 2, complete sequence	817	817	99%	0.0	93%	AY603202.1	767,48	83,6%
Select seq gb|AY603191.1|	Achillea setacea internal transcribed spacer 1, 5.8S ribosomal RNA gene, and internal transcribed spacer 2, complete sequence	817	817	99%	0.0	93%	AY603191.1	767,48	83,6%
Select seq gb|AY603190.1|	Achillea styriaca internal transcribed spacer 1, 5.8S ribosomal RNA gene, and internal transcribed spacer 2, complete sequence	817	817	99%	0.0	93%	AY603190.1	767,48	83,6%
Select seq gb|AY603187.1|	Achillea millefolium subsp. sudetica internal transcribed spacer 1, 5.8S ribosomal RNA gene, and internal transcribed spacer 2, complete sequence	817	817	99%	0.0	93%	AY603187.1	767,48	83,6%
Select seq gb|AY603185.1|	Achillea millefolium internal transcribed spacer 1, 5.8S ribosomal RNA gene, and internal transcribed spacer 2, complete sequence	817	817	99%	0.0	93%	AY603185.1	767,48	83,6%
Select seq gb|AY603184.1|	Achillea pannonica internal transcribed spacer 1, 5.8S ribosomal RNA gene, and internal transcribed spacer 2, complete sequence	817	817	99%	0.0	93%	AY603184.1	767,48	83,6%
Select seq dbj|AB683268.1|	Tanacetum aucherianum gene for ITS1, 5.8S rRNA and ITS2, complete sequence, specimen_voucher: 44441 (HUB)	815	815	99%	0.0	93%	AB683268.1	765,61	83,4%
Select seq dbj|AB683260.1|	Tanacetum abrotanifolium gene for ITS1, 5.8S rRNA and ITS2, complete sequence, specimen_voucher: 68865 (TARI)	815	815	99%	0.0	93%	AB683260.1	765,61	83,4%
Select seq gb|EF577320.1|	Tanacetum parthenium 18S ribosomal RNA gene, partial sequence; internal transcribed spacer 1, 5.8S ribosomal RNA gene, and internal transcribed spacer 2, complete sequence; and 28S ribosomal RNA gene, partial sequence	815	815	99%	0.0	93%	EF577320.1	765,61	83,4%
Select seq gb|AY603205.1|	Achillea lanulosa subsp. alpicola internal transcribed spacer 1, 5.8S ribosomal RNA gene, and internal transcribed spacer 2, complete sequence	815	815	99%	0.0	93%	AY603205.1	765,61	83,4%
Select seq gb|EF577322.1|	Tanacetum ptarmiciflorum 18S ribosomal RNA gene, partial sequence; internal transcribed spacer 1, 5.8S ribosomal RNA gene, and internal transcribed spacer 2, complete sequence; and 28S ribosomal RNA gene, partial sequence	813	813	99%	0.0	93%	EF577322.1	763,73	83,1%
Select seq gb|AY603186.1|	Achillea millefolium internal transcribed spacer 1, 5.8S ribosomal RNA gene, and internal transcribed spacer 2, complete sequence	813	813	99%	0.0	93%	AY603186.1	763,73	83,1%
Select seq gb|FJ553518.1|	Angiosperm environmental sample clone LTSP_EUKA_P3K07 18S ribosomal RNA, 18S-25/28S ribosomal RNA intergenic spacer, partial sequence	811	811	99%	0.0	93%	FJ553518.1	761,85	82,9%
Select seq gb|AY603229.1|	Achillea clavennae internal transcribed spacer 1, 5.8S ribosomal RNA gene, and internal transcribed spacer 2, complete sequence	811	811	99%	0.0	93%	AY603229.1	761,85	82,9%
Select seq gb|AY603212.1|	Achillea nobilis internal transcribed spacer 1, 5.8S ribosomal RNA gene, and internal transcribed spacer 2, complete sequence	811	811	99%	0.0	93%	AY603212.1	761,85	82,9%
Select seq gb|AY603207.1|	Achillea wilsoniana internal transcribed spacer 1, 5.8S ribosomal RNA gene, and internal transcribed spacer 2, complete sequence	811	811	99%	0.0	93%	AY603207.1	761,85	82,9%
Select seq gb|AY603197.1|	Achillea setacea internal transcribed spacer 1, 5.8S ribosomal RNA gene, and internal transcribed spacer 2, complete sequence	811	811	99%	0.0	93%	AY603197.1	761,85	82,9%
Select seq dbj|AB683313.1|	Tanacetum punctatum gene for ITS1, 5.8S rRNA and ITS2, complete sequence, specimen_voucher: 49833 (IRAN)	809	809	99%	0.0	93%	AB683313.1	759,97	82,7%
Select seq dbj|AB683285.1|	Tanacetum germanicopolitanum gene for ITS1, 5.8S rRNA and ITS2, complete sequence, specimen_voucher: 14254 (S)	809	809	99%	0.0	93%	AB683285.1	759,97	82,7%
Select seq emb|FN823080.1|	Tanacetum parthenium genomic DNA containing ITS1, 5.8S rRNA gene, ITS2, specimen voucher MPH:1119	809	809	99%	0.0	93%	FN823080.1	759,97	82,7%
Select seq emb|FN823079.1|	Tanacetum chiliophyllum genomic DNA containing ITS1, 5.8S rRNA gene, ITS2, specimen voucher MPH:1124	809	809	99%	0.0	93%	FN823079.1	759,97	82,7%

Select seq gb|EU796891.1|	Achillea millefolium 18S ribosomal RNA gene, partial sequence; internal transcribed spacer 1, 5.8S ribosomal RNA gene, and internal transcribed spacer 2, complete sequence; and 26S ribosomal RNA gene, partial sequence	809	809	99% 0.0	93% EU796891.1	759,97	82,7%
Select seq dbj|AB683265.1|	Tanacetum argenteum subsp. canum gene for ITS1, 5.8S rRNA and ITS2, complete sequence, specimen_voucher: 46856 (B)	808	808	99% 0.0	93% AB683265.1	759,03	82,6%
Select seq dbj|AB608331.1|	Tanacetum canescens genes for ITS1, 5.8S rRNA, ITS2, complete sequence	808	808	99% 0.0	93% AB608331.1	759,03	82,6%
Select seq gb|AY603204.1|	Achillea ceretanica internal transcribed spacer 1, 5.8S ribosomal RNA gene, and internal transcribed spacer 2, complete sequence	808	808	99% 0.0	93% AY603204.1	759,03	82,6%
Select seq gb|AY603201.1|	Achillea asplenifolia internal transcribed spacer 1, 5.8S ribosomal RNA gene, and internal transcribed spacer 2, complete sequence	808	808	99% 0.0	93% AY603201.1	759,03	82,6%
Select seq gb|AY603200.1|	Achillea roseoalba internal transcribed spacer 1, 5.8S ribosomal RNA gene, and internal transcribed spacer 2, complete sequence	808	808	99% 0.0	93% AY603200.1	759,03	82,6%
Select seq gb|AY603195.1|	Achillea setacea internal transcribed spacer 1, 5.8S ribosomal RNA gene, and internal transcribed spacer 2, complete sequence	808	808	99% 0.0	93% AY603195.1	759,03	82,6%
Select seq dbj|AB683282.1|	Tanacetum elbursense gene for ITS1, 5.8S rRNA and ITS2, complete sequence, specimen_voucher: 1109 (MPH)	806	806	99% 0.0	93% AB683282.1	757,15	82,4%
Select seq dbj|AB683278.1|	Tanacetum demetrii gene for ITS1, 5.8S rRNA and ITS2, complete sequence, specimen_voucher: (W, 2001-10597)	806	806	99% 0.0	93% AB683278.1	757,15	82,4%
Select seq dbj|AB608336.1|	Tanacetum polycephalum genes for ITS1, 5.8S rRNA, ITS2, complete sequence	806	806	99% 0.0	93% AB608336.1	757,15	82,4%
Select seq gb|EF577285.1|	Achillea wilsoniana 18S ribosomal RNA gene, partial sequence; internal transcribed spacer 1, 5.8S ribosomal RNA gene, and internal transcribed spacer 2, complete sequence; and 28S ribosomal RNA gene, partial sequence	806	806	99% 0.0	93% EF577285.1	757,15	82,4%
Select seq gb|AY603232.1|	Achillea fraasii internal transcribed spacer 1, 5.8S ribosomal RNA gene, and internal transcribed spacer 2, complete sequence	806	806	99% 0.0	93% AY603232.1	757,15	82,4%
Select seq gb|AY603222.1|	Achillea holosericea internal transcribed spacer 1, 5.8S ribosomal RNA gene, and internal transcribed spacer 2, complete sequence	806	806	99% 0.0	93% AY603222.1	757,15	82,4%
Select seq gb|AY603211.1|	Achillea virescens internal transcribed spacer 1, 5.8S ribosomal RNA gene, and internal transcribed spacer 2, complete sequence	806	806	99% 0.0	93% AY603211.1	757,15	82,4%
Select seq gb|AY603216.1|	Achillea clypeolata internal transcribed spacer 1, 5.8S ribosomal RNA gene, and internal transcribed spacer 2, complete sequence	806	806	99% 0.0	93% AY603216.1	757,15	82,4%
Select seq gb|AY603203.1|	Achillea ceretanica internal transcribed spacer 1, 5.8S ribosomal RNA gene, and internal transcribed spacer 2, complete sequence	806	806	99% 0.0	93% AY603203.1	757,15	82,4%
Select seq dbj|AB683304.1|	Tanacetum pinnatum gene for ITS1, 5.8S rRNA and ITS2, complete sequence, specimen_voucher: 1118 (MPH)	804	804	99% 0.0	93% AB683304.1	755,27	82,2%
Select seq dbj|AB683303.1|	Tanacetum parthenifolium gene for ITS1, 5.8S rRNA and ITS2, complete sequence, specimen_voucher: 1127 (MPH)	804	804	99% 0.0	93% AB683303.1	755,27	82,2%
Select seq dbj|AB683289.1|	Tanacetum hololeucum gene for ITS1, 5.8S rRNA and ITS2, complete sequence, specimen_voucher: 6472 (S)	804	804	99% 0.0	93% AB683289.1	755,27	82,2%
Select seq dbj|AB683271.1|	Tanacetum bamianicum gene for ITS1, 5.8S rRNA and ITS2, complete sequence, specimen_voucher: 18238 (B)	804	804	99% 0.0	93% AB683271.1	755,27	82,2%
Select seq gb|EF577323.1|	Tanacetum vulgare 18S ribosomal RNA gene, partial sequence; internal transcribed spacer 1, 5.8S ribosomal RNA gene, and internal transcribed spacer 2, complete sequence; and 28S ribosomal RNA gene, partial sequence	804	804	99% 0.0	93% EF577323.1	755,27	82,2%
Select seq gb|AY603264.1|	Tanacetum vulgare internal transcribed spacer 1, 5.8S ribosomal RNA gene, and internal transcribed spacer 2, complete sequence	804	804	99% 0.0	93% AY603264.1	755,27	82,2%
Select seq emb|FN823081.1|	Tanacetum coccineum genomic DNA containing ITS1, 5.8S rRNA gene, ITS2, specimen voucher MPH:1144	804	804	99% 0.0	92% FN823081.1	747,15	81,3%
Select seq gb|AY603210.1|	Achillea monticola internal transcribed spacer 1, 5.8S ribosomal RNA gene, and internal transcribed spacer 2, complete sequence	804	804	99% 0.0	92% AY603210.1	747,15	81,3%
Select seq dbj|AB683323.1|	Tanacetum sonbolii gene for ITS1, 5.8S rRNA and ITS2, complete sequence, specimen_voucher: 998 (MPH)	802	802	99% 0.0	92% AB683323.1	745,29	81,1%
Select seq gb|JQ230971.1|	Achillea millefolium voucher SBB-1156 18S ribosomal RNA gene, partial sequence; internal transcribed spacer 1, 5.8S ribosomal RNA gene, and internal transcribed spacer 2, complete sequence; and 28S ribosomal RNA gene, partial sequence	800	800	99% 0.0	92% JQ230971.1	743,43	80,9%
Select seq dbj|AB683316.1|	Tanacetum salsugineum gene for ITS1, 5.8S rRNA and ITS2, complete sequence, specimen_voucher: 1407 (MPH)	800	800	99% 0.0	92% AB683316.1	743,43	80,9%
Select seq dbj|AB683290.1|	Tanacetum khorassanicum gene for ITS1, 5.8S rRNA and ITS2, complete sequence, specimen_voucher: 4937 (S)	800	800	99% 0.0	92% AB683290.1	743,43	80,9%

Select seq gb|AY603245.1|	Achillea chamaemelifolia internal transcribed spacer 1, 5.8S ribosomal RNA gene, and internal transcribed spacer 2, complete sequence	800	800	99% 0.0	92% AY603245.1	743,43	80,9%
Select seq gb|AY603214.1|	Achillea ageratum internal transcribed spacer 1, 5.8S ribosomal RNA gene, and internal transcribed spacer 2, complete sequence	800	800	99% 0.0	92% AY603214.1	743,43	80,9%
Select seq gb|AY603220.1|	Achillea leptophylla internal transcribed spacer 1, 5.8S ribosomal RNA gene, and internal transcribed spacer 2, complete sequence	800	800	99% 0.0	92% AY603220.1	743,43	80,9%
Select seq dbj|AB683308.1|	Tanacetum polycephalum subsp. farsicum gene for ITS1, 5.8S rRNA and ITS2, complete sequence, specimen_voucher: 1167 (MPH)	798	798	99% 0.0	92% AB683308.1	741,58	80,7%
Select seq dbj|AB683288.1|	Tanacetum haussknechtii gene for ITS1, 5.8S rRNA and ITS2, complete sequence, specimen_voucher: 1193 (HUB)	798	798	99% 0.0	92% AB683288.1	741,58	80,7%
Select seq dbj|AB608337.1|	Tanacetum tabrisianum genes for ITS1, 5.8S rRNA, ITS2, complete sequence	798	798	99% 0.0	92% AB608337.1	741,58	80,7%
Select seq dbj|AB608332.1|	Tanacetum chiliophyllum genes for ITS1, 5.8S rRNA, ITS2, complete sequence	798	798	99% 0.0	92% AB608332.1	741,58	80,7%
Select seq gb|AY603263.1|	Tanacetum millefolium internal transcribed spacer 1, 5.8S ribosomal RNA gene, and internal transcribed spacer 2, complete sequence	798	798	99% 0.0	92% AY603263.1	741,58	80,7%
Select seq gb|AY603228.1|	Achillea clusiana internal transcribed spacer 1, 5.8S ribosomal RNA gene, and internal transcribed spacer 2, complete sequence	798	798	99% 0.0	92% AY603228.1	741,58	80,7%
Select seq gb|AY603227.1|	Achillea atrata internal transcribed spacer 1, 5.8S ribosomal RNA gene, and internal transcribed spacer 2, complete sequence	798	798	99% 0.0	92% AY603227.1	741,58	80,7%
Select seq dbj|AB683270.1|	Tanacetum balsamita gene for ITS1, 5.8S rRNA and ITS2, complete sequence, specimen_voucher: 1126 (MPH)	797	797	99% 0.0	92% AB683270.1	740,65	80,6%
Select seq gb|AY603249.1|	Achillea ptarmicifolia internal transcribed spacer 1, 5.8S ribosomal RNA gene, and internal transcribed spacer 2, complete sequence	795	795	99% 0.0	92% AY603249.1	738,79	80,4%
Select seq gb|AY603238.1|	Achillea oxyloba internal transcribed spacer 1, 5.8S ribosomal RNA gene, and internal transcribed spacer 2, complete sequence	795	795	99% 0.0	92% AY603238.1	738,79	80,4%
Select seq gb|AY603223.1|	Achillea micrantha internal transcribed spacer 1, 5.8S ribosomal RNA gene, and internal transcribed spacer 2, complete sequence	795	795	99% 0.0	92% AY603223.1	738,79	80,4%
Select seq dbj|AB683305.1|	Tanacetum polycephalum subsp. argyrophyllum gene for ITS1, 5.8S rRNA and ITS2, complete sequence, specimen_voucher: 1125 (MPH)	793	793	99% 0.0	92% AB683305.1	736,93	80,2%
Select seq dbj|AB683301.1|	Tanacetum nitens gene for ITS1, 5.8S rRNA and ITS2, complete sequence, specimen_voucher: (W, 1977-16564)	793	793	99% 0.0	92% AB683301.1	736,93	80,2%
Select seq dbj|AB523749.1|	Tanacetum kotschyi genes for ITS1, 5.8S rRNA, ITS2, complete sequence, specimen_voucher: MPH:1129	793	793	99% 0.0	92% AB523749.1	736,93	80,2%
Select seq gb|KM887397.1|	Anacyclus pyrethrum isolate SBB-1407 18S ribosomal RNA gene, partial sequence; internal transcribed spacer 1, 5.8S ribosomal RNA gene, and internal transcribed spacer 2, complete sequence; and 28S ribosomal RNA gene, partial sequence	791	791	99% 0.0	92% KM887397.1	735,07	80,0%
Select seq gb|KM887396.1|	Anacyclus pyrethrum isolate SBB-1364 18S ribosomal RNA gene, partial sequence; internal transcribed spacer 1, 5.8S ribosomal RNA gene, and internal transcribed spacer 2, complete sequence; and 28S ribosomal RNA gene, partial sequence	791	791	99% 0.0	92% KM887396.1	735,07	80,0%
Select seq dbj|AB523751.1|	Tanacetum porphyrostephanum genes for ITS1, 5.8S rRNA, ITS2, complete sequence, specimen_voucher: W:1986-05841	791	791	99% 0.0	92% AB523751.1	735,07	80,0%
Select seq gb|AY603254.1|	Achillea wilhelmsii internal transcribed spacer 1, 5.8S ribosomal RNA gene, and internal transcribed spacer 2, complete sequence	797	797	100% 0.0	92% AY603254.1	733,24	79,8%
Select seq gb|EU179214.1|	Anthemis arvensis 18S ribosomal RNA gene, partial sequence; internal transcribed spacer 1, 5.8S ribosomal RNA gene, and internal transcribed spacer 2, complete sequence; and 26S ribosomal RNA gene, partial sequence	789	789	99% 0.0	92% EU179214.1	733,21	79,8%
Select seq dbj|AB683336.1|	Tanacetum zahlbruckneri gene for ITS1, 5.8S rRNA and ITS2, complete sequence, specimen_voucher: 602 (MPH)	787	787	99% 0.0	92% AB683336.1	731,35	79,6%
Select seq dbj|AB683302.1|	Tanacetum paleaceum gene for ITS1, 5.8S rRNA and ITS2, complete sequence, specimen_voucher: 5828 (B)	787	787	99% 0.0	92% AB683302.1	731,35	79,6%
Select seq dbj|AB683286.1|	Tanacetum griffithii gene for ITS1, 5.8S rRNA and ITS2, complete sequence, specimen_voucher: 37485 (B)	787	787	99% 0.0	92% AB683286.1	731,35	79,6%
Select seq dbj|AB683269.1|	Tanacetum bachtiaricum gene for ITS1, 5.8S rRNA and ITS2, complete sequence, specimen_voucher: 1174 (MPH)	787	787	99% 0.0	92% AB683269.1	731,35	79,6%
Select seq dbj|AB608333.1|	Tanacetum coccineum genes for ITS1, 5.8S rRNA, ITS2, complete sequence	787	787	99% 0.0	92% AB608333.1	731,35	79,6%
Select seq gb|EU179216.1|	Anthemis cotula 18S ribosomal RNA gene, partial sequence; internal transcribed spacer 1, 5.8S ribosomal RNA gene, and internal transcribed spacer 2, complete sequence; and 26S ribosomal RNA gene, partial sequence	787	787	99% 0.0	92% EU179216.1	731,35	79,6%

Select seq gb|AY603262.1|	Tanacetum macrophyllum internal transcribed spacer 1, 5.8S ribosomal RNA gene, and internal transcribed spacer 2, complete sequence	787	787	99%	0.0	92%	AY603262.1	731,35	79,6%
Select seq gb|AY603219.1|	Achillea filipendulina internal transcribed spacer 1, 5.8S ribosomal RNA gene, and internal transcribed spacer 2, complete sequence	787	787	99%	0.0	92%	AY603219.1	731,35	79,6%
Select seq dbj|AB683274.1|	Tanacetum budjnurdense gene for ITS1, 5.8S rRNA and ITS2, complete sequence, specimen_voucher: 38119 (FUMH)	785	785	99%	0.0	92%	AB683274.1	729,49	79,4%
Select seq dbj|AB683281.1|	Tanacetum dumosum gene for ITS1, 5.8S rRNA and ITS2, complete sequence, specimen_voucher: 1168 (MPH)	784	784	99%	0.0	92%	AB683281.1	728,57	79,3%
Select seq emb|HE602382.1|	Anacyclus pyrethrum genomic DNA containing ITS1, 5.8S rRNA gene, ITS2, specimen voucher Jury, SL. 17869	784	784	99%	0.0	92%	HE602382.1	728,57	79,3%
Select seq dbj|AB608330.1|	Tanacetum budjnurdense genes for ITS1, 5.8S rRNA, ITS2, complete sequence	784	784	99%	0.0	92%	AB608330.1	728,57	79,3%
Select seq gb|AY603253.1|	Achillea wilhelmsii internal transcribed spacer 1, 5.8S ribosomal RNA gene, and internal transcribed spacer 2, complete sequence	791	791	100%	0.0	92%	AY603253.1	727,72	79,2%
Select seq dbj|AB683339.1|	Xylanthemum paghmanense gene for ITS1, 5.8S rRNA and ITS2, complete sequence, specimen_voucher: (W-1986-05509)	782	782	99%	0.0	92%	AB683339.1	726,71	79,1%
Select seq gb|KM887400.1|	Anacyclus pyrethrum isolate SBB-1400 internal transcribed spacer 1, partial sequence; 5.8S ribosomal RNA gene, complete sequence; and internal transcribed spacer 2, partial sequence	780	780	99%	0.0	92%	KM887400.1	724,85	78,9%
Select seq dbj|AB683266.1|	Tanacetum argenteum subsp. flabellifolium gene for ITS1, 5.8S rRNA and ITS2, complete sequence, specimen_voucher: 1976 (HUB)	780	780	99%	0.0	92%	AB683266.1	724,85	78,9%
Select seq gb|KM887358.1|	Anacyclus pyrethrum isolate SBB-1390 internal transcribed spacer 1, partial sequence; 5.8S ribosomal RNA gene, complete sequence; and internal transcribed spacer 2, partial sequence	778	778	99%	0.0	92%	KM887358.1	722,99	78,7%
Select seq dbj|AB523748.1|	Tanacetum kotschyi genes for ITS1, 5.8S rRNA, ITS2, complete sequence, specimen_voucher: MPH:1423	778	778	99%	0.0	92%	AB523748.1	722,99	78,7%
Select seq gb|AY603235.1|	Achillea moschata internal transcribed spacer 1, 5.8S ribosomal RNA gene, and internal transcribed spacer 2, complete sequence	778	778	99%	0.0	92%	AY603235.1	722,99	78,7%
Select seq gb|AY603234.1|	Achillea x morisiana internal transcribed spacer 1, 5.8S ribosomal RNA gene, and internal transcribed spacer 2, complete sequence	778	778	99%	0.0	92%	AY603234.1	722,99	78,7%
Select seq gb|AY603231.1|	Achillea erba-rotta internal transcribed spacer 1, 5.8S ribosomal RNA gene, and internal transcribed spacer 2, complete sequence	778	778	99%	0.0	92%	AY603231.1	722,99	78,7%
Select seq gb|AY603224.1|	Achillea ochroleuca internal transcribed spacer 1, 5.8S ribosomal RNA gene, and internal transcribed spacer 2, complete sequence	778	778	99%	0.0	92%	AY603224.1	722,99	78,7%
								0,00	0,0%

Select for downloading or viewing reports	Kh063_trnL Description	Max score	Total score	Query cover	E value	Ident	Accession	(Ident/Cover)* Max score	Deviation from top hit
Select seq gb|JX073760.1|	Artemisia lagocephala tRNA-Leu (trnL) gene and trnL-trnF intergenic spacer, partial sequence; chloroplast	1105	1105	87%	0.0	98%	JX073760.1	1244,71	100,0%
Select seq gb|JX073787.1|	Artemisia ludoviciana tRNA-Leu (trnL) gene and trnL-trnF intergenic spacer, partial sequence; chloroplast	1094	1094	87%	0.0	97%	JX073787.1	1219,75	98,0%
Select seq gb|JX073755.1|	Artemisia cana tRNA-Leu (trnL) gene and trnL-trnF intergenic spacer, partial sequence; chloroplast	1088	1088	87%	0.0	97%	JX073755.1	1213,06	97,5%
Select seq gb|JX073752.1|	Artemisia rothrockii tRNA-Leu (trnL) gene and trnL-trnF intergenic spacer, partial sequence; chloroplast	1136	1136	91%	0.0	97%	JX073752.1	1210,90	97,3%
Select seq gb|JX073815.1|	Artemisia afra tRNA-Leu (trnL) gene and trnL-trnF intergenic spacer, partial sequence; chloroplast	1086	1086	87%	0.0	97%	JX073815.1	1210,83	97,3%
Select seq gb|JX073796.1|	Artemisia scoparia tRNA-Leu (trnL) gene and trnL-trnF intergenic spacer, partial sequence; chloroplast	1086	1086	87%	0.0	97%	JX073796.1	1210,83	97,3%
Select seq gb|JX073792.1|	Artemisia japonica tRNA-Leu (trnL) gene and trnL-trnF intergenic spacer, partial sequence; chloroplast	1086	1086	87%	0.0	97%	JX073792.1	1210,83	97,3%
Select seq gb|JX073790.1|	Artemisia pycnocephala tRNA-Leu (trnL) gene and trnL-trnF intergenic spacer, partial sequence; chloroplast	1086	1086	87%	0.0	97%	JX073790.1	1210,83	97,3%
Select seq gb|JX073783.1|	Artemisia schmidtiana tRNA-Leu (trnL) gene and trnL-trnF intergenic spacer, partial sequence; chloroplast	1086	1086	87%	0.0	97%	JX073783.1	1210,83	97,3%
Select seq gb|JX073777.1|	Artemisia armeniaca tRNA-Leu (trnL) gene and trnL-trnF intergenic spacer, partial sequence; chloroplast	1086	1086	87%	0.0	97%	JX073777.1	1210,83	97,3%
Select seq gb|JX073747.1|	Artemisia nitrosa tRNA-Leu (trnL) gene and trnL-trnF intergenic spacer, partial sequence; chloroplast	1086	1086	87%	0.0	97%	JX073747.1	1210,83	97,3%
Select seq gb|JX073749.1|	Artemisia gmelinii subsp. scheludjakoviae tRNA-Leu (trnL) gene and trnL-trnF intergenic spacer, partial sequence; chloroplast	1086	1086	87%	0.0	97%	JX073749.1	1210,83	97,3%
Select seq gb|FJ842617.1|	Artemisia fukudo voucher MPS001124 (KMH) tRNA-Leu (trnL) gene and trnL-trnF intergenic spacer, partial sequence; chloroplast	1086	1086	87%	0.0	97%	FJ842617.1	1210,83	97,3%
Select seq gb|FJ842615.1|	Artemisia capillaris voucher MPS001196 (KMH) tRNA-Leu (trnL) gene and trnL-trnF intergenic spacer, partial sequence; chloroplast	1086	1086	87%	0.0	97%	FJ842615.1	1210,83	97,3%
Select seq gb|JX073828.1|	Kaschgaria komarovii tRNA-Leu (trnL) gene and trnL-trnF intergenic spacer, partial sequence; chloroplast	1134	1134	91%	0.0	97%	JX073828.1	1208,77	97,1%
Select seq gb|JX073812.1|	Artemisia subdigitata tRNA-Leu (trnL) gene and trnL-trnF intergenic spacer, partial sequence; chloroplast	1134	1134	91%	0.0	97%	JX073812.1	1208,77	97,1%
Select seq gb|JX073748.1|	Artemisia santonicum tRNA-Leu (trnL) gene and trnL-trnF intergenic spacer, partial sequence; chloroplast	1134	1134	91%	0.0	97%	JX073748.1	1208,77	97,1%
Select seq gb|KF736807.1|	Artemisia turcomanica tRNA-Leu (trnL) gene and trnL-trnF intergenic spacer, partial sequence; chloroplast	1107	1107	89%	0.0	97%	KF736807.1	1206,51	96,9%
Select seq gb|KF736803.1|	Artemisia fragrans tRNA-Leu (trnL) gene and trnL-trnF intergenic spacer, partial sequence; chloroplast	1107	1107	89%	0.0	97%	KF736803.1	1206,51	96,9%
Select seq gb|KF736802.1|	Artemisia incana tRNA-Leu (trnL) gene and trnL-trnF intergenic spacer, partial sequence; chloroplast	1107	1107	89%	0.0	97%	KF736802.1	1206,51	96,9%
Select seq gb|JX073816.1|	Artemisia elongata tRNA-Leu (trnL) gene and trnL-trnF intergenic spacer, partial sequence; chloroplast	1131	1131	91%	0.0	97%	JX073816.1	1205,57	96,9%
Select seq gb|JX073826.1|	Artemisia atrata tRNA-Leu (trnL) gene and trnL-trnF intergenic spacer, partial sequence; chloroplast	1081	1081	87%	0.0	97%	JX073826.1	1205,25	96,8%
Select seq gb|JX073786.1|	Artemisia suksdorfii tRNA-Leu (trnL) gene and trnL-trnF intergenic spacer, partial sequence; chloroplast	1081	1081	87%	0.0	97%	JX073786.1	1205,25	96,8%
Select seq gb|JX073785.1|	Artemisia serrata tRNA-Leu (trnL) gene and trnL-trnF intergenic spacer, partial sequence; chloroplast	1081	1081	87%	0.0	97%	JX073785.1	1205,25	96,8%
Select seq gb|JX073779.1|	Artemisia alba tRNA-Leu (trnL) gene and trnL-trnF intergenic spacer, partial sequence; chloroplast	1081	1081	87%	0.0	97%	JX073779.1	1205,25	96,8%
Select seq gb|JX073775.1|	Artemisia laciniata tRNA-Leu (trnL) gene and trnL-trnF intergenic spacer, partial sequence; chloroplast	1081	1081	87%	0.0	97%	JX073775.1	1205,25	96,8%
Select seq gb|FJ842621.1|	Artemisia keiskeana voucher MPS001234 (KMH) tRNA-Leu (trnL) gene and trnL-trnF intergenic spacer, partial sequence; chloroplast	1081	1081	87%	0.0	97%	FJ842621.1	1205,25	96,8%
Select seq gb|FJ842619.1|	Artemisia japonica voucher MPS000177 (KMH) tRNA-Leu (trnL) gene and trnL-trnF intergenic spacer, partial sequence; chloroplast	1081	1081	87%	0.0	97%	FJ842619.1	1205,25	96,8%
Select seq gb|FJ842618.1|	Artemisia gmelinii voucher MPS001216 (KMH) tRNA-Leu (trnL) gene and trnL-trnF intergenic spacer, partial sequence; chloroplast	1081	1081	87%	0.0	97%	FJ842618.1	1205,25	96,8%
Select seq gb|FJ710526.1|	Artemisia absinthium tRNA-Leu (trnL) gene and trnL-trnF intergenic spacer, partial sequence; chloroplast	1081	1081	87%	0.0	97%	FJ710526.1	1205,25	96,8%
Select seq gb|FJ692341.1|	Artemisia annua voucher MPS 001275 (KMH) tRNA-Leu (trnL) gene and trnL-trnF intergenic spacer, partial sequence; chloroplast	1081	1081	87%	0.0	97%	FJ692341.1	1205,25	96,8%
Select seq dbj|AB551235.1|	Artemisia capillaris chloroplast genes for tRNA-Leu intron and 3' exon, trnL-trnF intergenic spacer and tRNA-Phe	1155	1155	93%	0.0	97%	AB551235.1	1204,68	96,8%

Select seq gb|JX073811.1|	Artemisia glomerata tRNA-Leu (trnL) gene and trnL-trnF intergenic spacer, partial sequence; chloroplast	1129	1129	91% 0.0	97% JX073811.1	1203,44	96,7%
Select seq gb|JX073808.1|	Artemisia tilesii tRNA-Leu (trnL) gene and trnL-trnF intergenic spacer, partial sequence; chloroplast	1129	1129	91% 0.0	97% JX073808.1	1203,44	96,7%
Select seq gb|JX073803.1|	Artemisia tournefortiana tRNA-Leu (trnL) gene and trnL-trnF intergenic spacer, partial sequence; chloroplast	1129	1129	91% 0.0	97% JX073803.1	1203,44	96,7%
Select seq gb|JX073805.1|	Artemisia gracilescens tRNA-Leu (trnL) gene and trnL-trnF intergenic spacer, partial sequence; chloroplast	1116	1116	90% 0.0	97% JX073805.1	1202,80	96,6%
Select seq gb|KF736826.1|	Artemisia aucheri tRNA-Leu (trnL) gene and trnL-trnF intergenic spacer, partial sequence; chloroplast	1101	1101	89% 0.0	97% KF736826.1	1199,97	96,4%
Select seq gb|KF736805.1|	Artemisia spicigera tRNA-Leu (trnL) gene and trnL-trnF intergenic spacer, partial sequence; chloroplast	1101	1101	89% 0.0	97% KF736805.1	1199,97	96,4%
Select seq gb|KF669899.1|	Artemisia absinthium tRNA-Leu (trnL) gene and trnL-trnF intergenic spacer, partial sequence; chloroplast	1101	1101	89% 0.0	97% KF669899.1	1199,97	96,4%
Select seq dbj|AB219593.1|	Artemisia sinanensis chloroplast trnL(UAA), trnF(GAA) genes for tRNA-Leu, tRNA-Phe, partial sequence, trnL-trnF spacer region, specimen_voucher: MAK:N. Fujii F01782	1101	1101	89% 0.0	97% AB219593.1	1199,97	96,4%
Select seq gb|JX073804.1|	Artemisia chamaemelifolia tRNA-Leu (trnL) gene and trnL-trnF intergenic spacer, partial sequence; chloroplast	1125	1125	91% 0.0	97% JX073804.1	1199,18	96,3%
Select seq gb|JX073821.1|	Artemisia mauiensis tRNA-Leu (trnL) gene and trnL-trnF intergenic spacer, partial sequence; chloroplast	1075	1075	87% 0.0	97% JX073821.1	1198,56	96,3%
Select seq gb|JX073819.1|	Artemisia australis tRNA-Leu (trnL) gene and trnL-trnF intergenic spacer, partial sequence; chloroplast	1075	1075	87% 0.0	97% JX073819.1	1198,56	96,3%
Select seq gb|JX073753.1|	Artemisia arbuscula tRNA-Leu (trnL) gene and trnL-trnF intergenic spacer, partial sequence; chloroplast	1075	1075	87% 0.0	97% JX073753.1	1198,56	96,3%
Select seq gb|JX073799.1|	Artemisia gorgonum tRNA-Leu (trnL) gene and trnL-trnF intergenic spacer, partial sequence; chloroplast	1075	1075	87% 0.0	97% JX073799.1	1198,56	96,3%
Select seq gb|JX073798.1|	Artemisia rigida tRNA-Leu (trnL) gene and trnL-trnF intergenic spacer, partial sequence; chloroplast	1075	1075	87% 0.0	97% JX073798.1	1198,56	96,3%
Select seq gb|JX073778.1|	Artemisia abrotanum tRNA-Leu (trnL) gene and trnL-trnF intergenic spacer, partial sequence; chloroplast	1075	1075	87% 0.0	97% JX073778.1	1198,56	96,3%
Select seq gb|FJ842620.1|	Artemisia manshurica voucher MPS00123 (KMH) tRNA-Leu (trnL) gene and trnL-trnF intergenic spacer, partial sequence; chloroplast	1075	1075	87% 0.0	97% FJ842620.1	1198,56	96,3%
Select seq gb|FJ710524.1|	Artemisia caruifolia var. apiacea tRNA-Leu (trnL) gene and trnL-trnF intergenic spacer, partial sequence; chloroplast	1075	1075	87% 0.0	97% FJ710524.1	1198,56	96,3%
Select seq gb|HM000099.1|	Chrysanthemum indicum isolate HEBSJZ tRNA-Leu (trnL) gene, partial sequence; trnL-trnF intergenic spacer, complete sequence; and tRNA-Phe (trnF) gene, partial sequence; chloroplast	1173	1173	95% 0.0	97% HM000099.1	1197,69	96,2%
Select seq gb|HM000094.1|	Chrysanthemum indicum isolate HUBJZ tRNA-Leu (trnL) gene, partial sequence; trnL-trnF intergenic spacer, complete sequence; and tRNA-Phe (trnF) gene, partial sequence; chloroplast	1173	1173	95% 0.0	97% HM000094.1	1197,69	96,2%
Select seq gb|JX073797.1|	Artemisia dracunculus tRNA-Leu (trnL) gene and trnL-trnF intergenic spacer, partial sequence; chloroplast	1074	1074	87% 0.0	97% JX073797.1	1197,45	96,2%
Select seq gb|JX073774.1|	Artemisia molinieri tRNA-Leu (trnL) gene and trnL-trnF intergenic spacer, partial sequence; chloroplast	1074	1074	87% 0.0	97% JX073774.1	1197,45	96,2%
Select seq gb|JX073761.1|	Artemisia anomala tRNA-Leu (trnL) gene and trnL-trnF intergenic spacer, partial sequence; chloroplast	1074	1074	87% 0.0	97% JX073761.1	1197,45	96,2%
Select seq gb|JX073758.1|	Artemisia pedatifida tRNA-Leu (trnL) gene and trnL-trnF intergenic spacer, partial sequence; chloroplast	1074	1074	87% 0.0	97% JX073758.1	1197,45	96,2%
Select seq gb|JX073827.1|	Artemisia absinthium tRNA-Leu (trnL) gene and trnL-trnF intergenic spacer, partial sequence; chloroplast	1123	1123	91% 0.0	97% JX073827.1	1197,04	96,2%
Select seq gb|JX073814.1|	Mausolea eriocarpa tRNA-Leu (trnL) gene and trnL-trnF intergenic spacer, partial sequence; chloroplast	1123	1123	91% 0.0	97% JX073814.1	1197,04	96,2%
Select seq gb|JX073791.1|	Artemisia tomentella tRNA-Leu (trnL) gene and trnL-trnF intergenic spacer, partial sequence; chloroplast	1123	1123	91% 0.0	97% JX073791.1	1197,04	96,2%
Select seq gb|KF736827.1|	Artemisia ciniformis tRNA-Leu (trnL) gene and trnL-trnF intergenic spacer, partial sequence; chloroplast	1096	1096	89% 0.0	97% KF736827.1	1194,52	96,0%
Select seq gb|KF736825.1|	Artemisia melanolepis tRNA-Leu (trnL) gene and trnL-trnF intergenic spacer, partial sequence; chloroplast	1096	1096	89% 0.0	97% KF736825.1	1194,52	96,0%
Select seq gb|KF736809.1|	Artemisia marschalliana tRNA-Leu (trnL) gene and trnL-trnF intergenic spacer, partial sequence; chloroplast	1096	1096	89% 0.0	97% KF736809.1	1194,52	96,0%
Select seq gb|KF736808.1|	Artemisia chamaemelifolia tRNA-Leu (trnL) gene and trnL-trnF intergenic spacer, partial sequence; chloroplast	1096	1096	89% 0.0	97% KF736808.1	1194,52	96,0%
Select seq gb|KF736806.1|	Artemisia haussknechtii tRNA-Leu (trnL) gene and trnL-trnF intergenic spacer, partial sequence; chloroplast	1096	1096	89% 0.0	97% KF736806.1	1194,52	96,0%
Select seq gb|JX073823.1|	Artemisia globularia tRNA-Leu (trnL) gene and trnL-trnF intergenic spacer, partial sequence; chloroplast	1120	1120	91% 0.0	97% JX073823.1	1193,85	95,9%
Select seq gb|JX073824.1|	Artemisia copa tRNA-Leu (trnL) gene and trnL-trnF intergenic spacer, partial sequence; chloroplast	1070	1070	87% 0.0	97% JX073824.1	1192,99	95,8%
Select seq gb|JX073793.1|	Artemisia campestris tRNA-Leu (trnL) gene and trnL-trnF intergenic spacer, partial sequence; chloroplast	1070	1070	87% 0.0	97% JX073793.1	1192,99	95,8%

Select seq gb|JX073782.1|	Artemisia nitida tRNA-Leu (trnL) gene and trnL-trnF intergenic spacer, partial sequence; chloroplast	1070	1070	87%	0.0	97%	JX073782.1	1192,99	95,8%
Select seq gb|JX073773.1|	Artemisia tridentata tRNA-Leu (trnL) gene and trnL-trnF intergenic spacer, partial sequence; chloroplast	1070	1070	87%	0.0	97%	JX073773.1	1192,99	95,8%
Select seq gb|JN315916.1|	Chrysanthemum indicum tRNA-Leu (trnL) gene and trnL-trnF intergenic spacer, partial sequence; chloroplast	1094	1094	89%	0.0	97%	JN315916.1	1192,34	95,8%
Select seq gb|JX073820.1|	Artemisia kauaiensis tRNA-Leu (trnL) gene and trnL-trnF intergenic spacer, partial sequence; chloroplast	1118	1118	91%	0.0	97%	JX073820.1	1191,71	95,7%
Select seq gb|JX073809.1|	Artemisia pattersonii tRNA-Leu (trnL) gene and trnL-trnF intergenic spacer, partial sequence; chloroplast	1118	1118	91%	0.0	97%	JX073809.1	1191,71	95,7%
Select seq gb|JX073807.1|	Artemisia martirensis tRNA-Leu (trnL) gene and trnL-trnF intergenic spacer, partial sequence; chloroplast	1118	1118	91%	0.0	97%	JX073807.1	1191,71	95,7%
Select seq dbj|AB685322.1|	Chrysanthemum indicum chloroplast DNA, contains tRNA-Leu(trnL) gene (intron and 3' exon), trnL-trnF intergenic spacer and tRNA-Phe(trnF) gene	1142	1142	93%	0.0	97%	AB685322.1	1191,12	95,7%
Select seq gb|KF736821.1|	Artemisia capillaris tRNA-Leu (trnL) gene and trnL-trnF intergenic spacer, partial sequence; chloroplast	1090	1090	89%	0.0	97%	KF736821.1	1187,98	95,4%
Select seq gb|JX073825.1|	Artemisia potentilloides tRNA-Leu (trnL) gene and trnL-trnF intergenic spacer, partial sequence; chloroplast	1114	1114	91%	0.0	96%	JX073825.1	1175,21	94,4%
Select seq gb|HM000091.1|	Chrysanthemum indicum isolate HENXY tRNA-Leu (trnL) gene, partial sequence; trnL-trnF intergenic spacer, complete sequence; and tRNA-Phe (trnF) gene, partial sequence; chloroplast	1160	1160	95%	0.0	96%	HM000091.1	1172,21	94,2%
Select seq gb|KF736817.1|	Artemisia tschernieviana tRNA-Leu (trnL) gene and trnL-trnF intergenic spacer, partial sequence; chloroplast	1083	1083	89%	0.0	96%	KF736817.1	1168,18	93,9%
Select seq gb|HM000097.1|	Chrysanthemum indicum isolate JXPX tRNA-Leu (trnL) gene, partial sequence; trnL-trnF intergenic spacer, complete sequence; and tRNA-Phe (trnF) gene, partial sequence; chloroplast	1155	1155	95%	0.0	96%	HM000097.1	1167,16	93,8%
Select seq gb|HM000096.1|	Chrysanthemum indicum isolate YNKM tRNA-Leu (trnL) gene, partial sequence; trnL-trnF intergenic spacer, complete sequence; and tRNA-Phe (trnF) gene, partial sequence; chloroplast	1155	1155	95%	0.0	96%	HM000096.1	1167,16	93,8%
Select seq gb|HM000095.1|	Chrysanthemum indicum isolate HUNZZ tRNA-Leu (trnL) gene, partial sequence; trnL-trnF intergenic spacer, complete sequence; and tRNA-Phe (trnF) gene, partial sequence; chloroplast	1155	1155	95%	0.0	96%	HM000095.1	1167,16	93,8%
Select seq gb|HM000088.1|	Chrysanthemum indicum isolate GZGY tRNA-Leu (trnL) gene, partial sequence; trnL-trnF intergenic spacer, complete sequence; and tRNA-Phe (trnF) gene, partial sequence; chloroplast	1155	1155	95%	0.0	96%	HM000088.1	1167,16	93,8%
Select seq gb|HM000087.1|	Chrysanthemum indicum isolate AHCZ tRNA-Leu (trnL) gene, partial sequence; trnL-trnF intergenic spacer, complete sequence; and tRNA-Phe (trnF) gene, partial sequence; chloroplast	1155	1155	95%	0.0	96%	HM000087.1	1167,16	93,8%
Select seq gb|HM000086.1|	Chrysanthemum indicum isolate JSWX tRNA-Leu (trnL) gene, partial sequence; trnL-trnF intergenic spacer, complete sequence; and tRNA-Phe (trnF) gene, partial sequence; chloroplast	1155	1155	95%	0.0	96%	HM000086.1	1167,16	93,8%
Select seq gb|HM000085.1|	Chrysanthemum indicum isolate JSNJ tRNA-Leu (trnL) gene, partial sequence; trnL-trnF intergenic spacer, complete sequence; and tRNA-Phe (trnF) gene, partial sequence; chloroplast	1155	1155	95%	0.0	96%	HM000085.1	1167,16	93,8%
Select seq gb|HM000093.1|	Chrysanthemum indicum isolate SCMY tRNA-Leu (trnL) gene, partial sequence; trnL-trnF intergenic spacer, complete sequence; and tRNA-Phe (trnF) gene, partial sequence; chloroplast	1149	1149	95%	0.0	96%	HM000093.1	1161,09	93,3%
Select seq gb|HM000092.1|	Chrysanthemum indicum isolate GXGL tRNA-Leu (trnL) gene, partial sequence; trnL-trnF intergenic spacer, complete sequence; and tRNA-Phe (trnF) gene, partial sequence; chloroplast	1149	1149	95%	0.0	96%	HM000092.1	1161,09	93,3%
Select seq gb|HM000089.1|	Chrysanthemum indicum isolate AHHF tRNA-Leu (trnL) gene, partial sequence; trnL-trnF intergenic spacer, complete sequence; and tRNA-Phe (trnF) gene, partial sequence; chloroplast	1149	1149	95%	0.0	96%	HM000089.1	1161,09	93,3%
Select seq gb|HM000098.1|	Chrysanthemum indicum isolate FJFZ tRNA-Leu (trnL) gene, partial sequence; trnL-trnF intergenic spacer, complete sequence; and tRNA-Phe (trnF) gene, partial sequence; chloroplast	1144	1144	95%	0.0	96%	HM000098.1	1156,04	92,9%
Select seq gb|HM000090.1|	Chrysanthemum indicum isolate ZJWZ tRNA-Leu (trnL) gene, partial sequence; trnL-trnF intergenic spacer, complete sequence; and tRNA-Phe (trnF) gene, partial sequence; chloroplast	1144	1144	95%	0.0	96%	HM000090.1	1156,04	92,9%
Select seq gb|GU817959.1|	Anacyclus valentinus isolate L1186 tRNA-Leu (trnL) gene, partial sequence; trnL-trnF intergenic spacer, complete sequence; and tRNA-Phe (trnF) gene, partial sequence; plastid	1110	1110	93%	0.0	96%	GU817959.1	1145,81	92,1%
Select seq gb|KR150196.1|	Achillea sp. 3 Kh58 trnL-trnF intergenic spacer region, partial sequence; chloroplast	1077	1077	93%	0.0	95%	KR150196.1	1100,16	88,4%
Select seq gb|KR150244.1|	Achillea sp. 1 Kh17 trnL-trnF intergenic spacer region, partial sequence; chloroplast	1072	1072	93%	0.0	95%	KR150244.1	1095,05	88,0%
								0,00	
								0,00	
								0,00	
								0,00	
								0,00	
								0,00	
								0,00	

Select for downloading or viewing reports	Kh065_trnL Description	Max score	Total score	Query cover	E value	Ident	Accession	(Ident/Cover)* Max score	Deviation from top hit
Select seq gb|AY506613.1|	Thymus vulgaris tRNA-Leu (trnL) gene and trnL-trnF intergenic spacer, partial sequence; chloroplast	1175	1175	95%	0.0	99%	AY506613.1	1224,47	100,0%
Select seq gb|GU381635.1|	Thymus broussonetii subsp. hannonis voucher MSB:Podlech 142 tRNA-Leu (trnL) gene and trnL-trnF intergenic spacer, partial sequence; chloroplast	1208	1208	98%	0.0	99%	GU381635.1	1220,33	99,7%
Select seq gb|EU556536.1|	Thymus marschallianus isolate XY tRNA-Leu (trnL) gene and trnL-trnF intergenic spacer, partial sequence; chloroplast	1194	1194	97%	0.0	99%	EU556536.1	1218,62	99,5%
Select seq gb|GU381638.1|	Thymus haussknechtii voucher M:Nydegger 43838 tRNA-Leu (trnL) gene and trnL-trnF intergenic spacer, partial sequence; chloroplast	1181	1181	96%	0.0	99%	GU381638.1	1217,91	99,5%
Select seq gb|JQ669069.1|	Thymus pulegioides voucher Riina 1577 tRNA-Leu (trnL) gene and trnL-trnF intergenic spacer, partial sequence; plastid	1203	1203	98%	0.0	99%	JQ669069.1	1215,28	99,2%
Select seq gb|GU381637.1|	Thymus serpyllum voucher M:Braeuchler 2514 tRNA-Leu (trnL) gene and trnL-trnF intergenic spacer, partial sequence; chloroplast	1203	1203	98%	0.0	99%	GU381637.1	1215,28	99,2%
Select seq gb|EU556530.1|	Thymus mandschuricus isolate HM tRNA-Leu (trnL) gene and trnL-trnF intergenic spacer, partial sequence; chloroplast	1203	1203	98%	0.0	99%	EU556530.1	1215,28	99,2%
Select seq gb|KR063657.1|	Thymus sibthorpii tRNA-Leu (trnL) gene, partial sequence; trnL-trnF intergenic spacer, complete sequence; and tRNA-Phe (trnF) gene, partial sequence; chloroplast	1227	1227	100%	0.0	99%	KR063657.1	1214,73	99,2%
Select seq gb|EU556532.1|	Thymus amurensis isolate HX tRNA-Leu (trnL) gene and trnL-trnF intergenic spacer, partial sequence; chloroplast	1227	1227	100%	0.0	99%	EU556532.1	1214,73	99,2%
Select seq gb|EU556525.1|	Thymus quinquecostatus isolate CL tRNA-Leu (trnL) gene and trnL-trnF intergenic spacer, partial sequence; chloroplast	1227	1227	100%	0.0	99%	EU556525.1	1214,73	99,2%
Select seq gb|EU556535.1|	Thymus proximus isolate XN tRNA-Leu (trnL) gene and trnL-trnF intergenic spacer, partial sequence; chloroplast	1164	1164	95%	0.0	99%	EU556535.1	1213,01	99,1%
Select seq gb|GU381634.1|	Thymus pulegioides voucher M:Braeuchler 3129 tRNA-Leu (trnL) gene and trnL-trnF intergenic spacer, partial sequence; chloroplast	1199	1199	98%	0.0	99%	GU381634.1	1211,23	98,9%
Select seq gb|GU381636.1|	Thymus vulgaris voucher M:Braeuchler 3683 tRNA-Leu (trnL) gene and trnL-trnF intergenic spacer, partial sequence; chloroplast	1197	1197	98%	0.0	99%	GU381636.1	1209,21	98,8%
Select seq gb|EU556539.1|	Thymus quinquecostatus isolate ZY tRNA-Leu (trnL) gene and trnL-trnF intergenic spacer, partial sequence; chloroplast	1219	1219	100%	0.0	99%	EU556539.1	1206,81	98,6%
Select seq gb|EU556538.1|	Thymus quinquecostatus isolate ZJ tRNA-Leu (trnL) gene and trnL-trnF intergenic spacer, partial sequence; chloroplast	1219	1219	100%	0.0	99%	EU556538.1	1206,81	98,6%
Select seq gb|EU556533.1|	Thymus quinquecostatus isolate HY tRNA-Leu (trnL) gene and trnL-trnF intergenic spacer, partial sequence; chloroplast	1219	1219	100%	0.0	99%	EU556533.1	1206,81	98,6%
Select seq gb|EU556527.1|	Thymus mongolicus isolate G2 tRNA-Leu (trnL) gene and trnL-trnF intergenic spacer, partial sequence; chloroplast	1219	1219	100%	0.0	99%	EU556527.1	1206,81	98,6%
Select seq gb|EU556526.1|	Thymus quinquecostatus isolate D2 tRNA-Leu (trnL) gene and trnL-trnF intergenic spacer, partial sequence; chloroplast	1219	1219	100%	0.0	99%	EU556526.1	1206,81	98,6%
Select seq gb|EU556534.1|	Thymus mongolicus isolate S1 tRNA-Leu (trnL) gene and trnL-trnF intergenic spacer, partial sequence; chloroplast	1158	1158	95%	0.0	99%	EU556534.1	1206,76	98,6%
Select seq gb|EU556531.1|	Thymus amurensis isolate HW tRNA-Leu (trnL) gene and trnL-trnF intergenic spacer, partial sequence; chloroplast	1214	1214	100%	0.0	99%	EU556531.1	1201,86	98,2%
Select seq gb|EU556529.1|	Thymus dahuricus isolate HD tRNA-Leu (trnL) gene and trnL-trnF intergenic spacer, partial sequence; chloroplast	1188	1188	98%	0.0	99%	EU556529.1	1200,12	98,0%
Select seq gb|GU381633.1|	Thymus caespititius voucher M:Heubl s.n. tRNA-Leu (trnL) gene and trnL-trnF intergenic spacer, partial sequence; chloroplast	1186	1186	98%	0.0	99%	GU381633.1	1198,10	97,8%
Select seq gb|EU556528.1|	Thymus mongolicus isolate G3 tRNA-Leu (trnL) gene and trnL-trnF intergenic spacer, partial sequence; chloroplast	1197	1197	99%	0.0	99%	EU556528.1	1197,00	97,8%
Select seq emb|AJ505544.1|	Thymus serpyllum var. citriodorum plastid trnL-trnF intergenic spacer, specimen voucher cult., K-1975-1177, Chase 13331 (K)	1208	1208	100%	0.0	99%	AJ505544.1	1195,92	97,7%
Select seq gb|GU381640.1|	Argantoniella salzmannii voucher M:Barra et al. 2673GL tRNA-Leu (trnL) gene and trnL-trnF intergenic spacer, partial sequence; chloroplast	1181	1181	98%	0.0	99%	GU381640.1	1193,05	97,4%
Select seq gb|AY840202.1|	Origanum vulgare tRNA-Leu (trnL) gene and trnL-trnF intergenic spacer, partial sequence; chloroplast	1181	1181	98%	0.0	99%	AY840202.1	1193,05	97,4%

Select seq gb|AY506614.1|	Origanum vulgare tRNA-Leu (trnL) gene and trnL-trnF intergenic spacer, partial sequence; chloroplast	1140	1140	95%	0.0	99%	AY506614.1	1188,00	97,0%
Select seq gb|JX880022.1|	Origanum vulgare subsp. vulgare chloroplast, complete genome	1199	1199	100%	0.0	99%	JX880022.1	1187,01	96,9%
Select seq gb|JQ690290.1|	Origanum elongatum isolate H5_O_elo trnL-trnF intergenic spacer, partial sequence; chloroplast	1199	1199	100%	0.0	99%	JQ690290.1	1187,01	96,9%
Select seq gb|JQ690289.1|	Origanum rotundifolium isolate DNA3_O_rot trnL-trnF intergenic spacer, partial sequence; chloroplast	1199	1199	100%	0.0	99%	JQ690289.1	1187,01	96,9%
Select seq emb|AJ505543.1|	Origanum vulgare plastid trnL-trnF intergenic spacer, specimen voucher cult., K-000-69-19317, chase 13334 (K)	1199	1199	100%	0.0	99%	AJ505543.1	1187,01	96,9%
Select seq gb|AY570463.1|	Origanum vulgare voucher JBW 2567 tRNA-Leu and trnL-trnF intergenic spacer, partial sequence; chloroplast	1173	1173	98%	0.0	99%	AY570463.1	1184,97	96,8%
Select seq gb|EU556537.1|	Thymus quinquecostatus isolate XZ tRNA-Leu (trnL) gene and trnL-trnF intergenic spacer, partial sequence; chloroplast	1195	1195	100%	0.0	99%	EU556537.1	1183,05	96,6%
Select seq gb|JF301392.1|	Origanum vulgare voucher B. Drew 77 tRNA-Leu (trnL) gene and trnL-trnF intergenic spacer, partial sequence; chloroplast	1147	1147	96%	0.0	99%	JF301392.1	1182,84	96,6%
Select seq gb|AY570502.1|	Thymus serpyllum voucher JBW 2564 tRNA-Leu and trnL-trnF intergenic spacer, partial sequence; chloroplast	1170	1170	98%	0.0	99%	AY570502.1	1181,94	96,5%
Select seq gb|JQ690293.1|	Origanum dayi isolate H43_O_day trnL-trnF intergenic spacer, partial sequence; chloroplast	1188	1188	100%	0.0	99%	JQ690293.1	1176,12	96,1%
Select seq gb|GU381629.1|	Thymbra capitata voucher M:Braeuchler 2518 tRNA-Leu (trnL) gene and trnL-trnF intergenic spacer, partial sequence; chloroplast	1164	1164	98%	0.0	98%	GU381629.1	1164,00	95,1%
Select seq gb|JQ669070.1|	Thymus serpyllum voucher J. Walker 2564 tRNA-Leu (trnL) gene and trnL-trnF intergenic spacer, partial sequence; plastid	1162	1162	98%	0.0	98%	JQ669070.1	1162,00	94,9%
Select seq gb|JF301401.1|	Thymbra capitata voucher UCBG 96.0817 tRNA-Leu (trnL) gene and trnL-trnF intergenic spacer, partial sequence; chloroplast	1138	1138	96%	0.0	98%	JF301401.1	1161,71	94,9%
Select seq gb|GU381484.1|	Micromeria flagellaris voucher E:van der Werff & McPherson 13570 tRNA-Leu (trnL) gene and trnL-trnF intergenic spacer, partial sequence; chloroplast	1158	1158	98%	0.0	98%	GU381484.1	1158,00	94,6%
Select seq gb|GU381483.1|	Micromeria flagellaris voucher E:Clement et al 2140 tRNA-Leu (trnL) gene and trnL-trnF intergenic spacer, partial sequence; chloroplast	1158	1158	98%	0.0	98%	GU381483.1	1158,00	94,6%
Select seq gb|AY840207.1|	Thymbra spicata tRNA-Leu (trnL) gene and trnL-trnF intergenic spacer, partial sequence; chloroplast	1158	1158	98%	0.0	98%	AY840207.1	1158,00	94,6%
Select seq gb|GU381632.1|	Thymbra spicata voucher M:Braeuchler 4548 tRNA-Leu (trnL) gene and trnL-trnF intergenic spacer, partial sequence; chloroplast	1155	1155	98%	0.0	98%	GU381632.1	1155,00	94,3%
Select seq gb|GU381627.1|	Thymbra sintenisii subsp. isaurica voucher E:Goener 12628 tRNA-Leu (trnL) gene and trnL-trnF intergenic spacer, partial sequence; chloroplast	1153	1153	98%	0.0	98%	GU381627.1	1153,00	94,2%
Select seq gb|GU381517.1|	Clinopodium barosmum voucher BM<GBR-LONDON>:McLaren N193 tRNA-Leu (trnL) gene and trnL-trnF intergenic spacer, partial sequence; chloroplast	1142	1142	98%	0.0	98%	GU381517.1	1142,00	93,3%
Select seq gb|GU381516.1|	Clinopodium wardii voucher BM<GBR-LONDON>:Ludlow et al. 14234 tRNA-Leu (trnL) gene and trnL-trnF intergenic spacer, partial sequence; chloroplast	1142	1142	98%	0.0	98%	GU381516.1	1142,00	93,3%
Select seq gb|GU381515.1|	Clinopodium hydaspidis voucher BM<GBR-LONDON>:Mohd 133 tRNA-Leu (trnL) gene and trnL-trnF intergenic spacer, partial sequence; chloroplast	1142	1142	98%	0.0	98%	GU381515.1	1142,00	93,3%
Select seq gb|GU381514.1|	Clinopodium nepalense voucher FR:Stainton 6024 tRNA-Leu (trnL) gene and trnL-trnF intergenic spacer, partial sequence; chloroplast	1142	1142	98%	0.0	98%	GU381514.1	1142,00	93,3%
Select seq gb|GU381511.1|	Clinopodium piperitum voucher BM<GBR-LONDON>:Vickery 454 tRNA-Leu (trnL) gene and trnL-trnF intergenic spacer, partial sequence; chloroplast	1142	1142	98%	0.0	98%	GU381511.1	1142,00	93,3%
Select seq gb|GU381513.1|	Clinopodium nepalense voucher BM<GBR-LONDON>:Mikage et al. 9550294 tRNA-Leu (trnL) gene and trnL-trnF intergenic spacer, partial sequence; chloroplast	1114	1114	96%	0.0	98%	GU381513.1	1137,21	92,9%
Select seq gb|GU381512.1|	Clinopodium piperitum voucher E:Stainton 7320 tRNA-Leu (trnL) gene and trnL-trnF intergenic spacer, partial sequence; chloroplast	1136	1136	98%	0.0	98%	GU381512.1	1136,00	92,8%
Select seq gb|GU381496.1|	Killickia pilosa voucher M:Braeuchler 3832 tRNA-Leu (trnL) gene and trnL-trnF intergenic spacer, partial sequence; chloroplast	1133	1133	98%	0.0	98%	GU381496.1	1133,00	92,5%
Select seq gb|GU381493.1|	Killickia grandiflora voucher M:Braeuchler 3811 tRNA-Leu (trnL) gene and trnL-trnF intergenic spacer, partial sequence; chloroplast	1133	1133	98%	0.0	98%	GU381493.1	1133,00	92,5%
Select seq gb|GU381631.1|	Satureja linearifolia voucher M:Brullo & Furnari s.n. tRNA-Leu (trnL) gene and trnL-trnF intergenic spacer, partial sequence; chloroplast	1131	1131	98%	0.0	98%	GU381631.1	1131,00	92,4%

Select seq gb|GU381495.1|	Killickia pilosa voucher M:Braeuchler 3810 tRNA-Leu (trnL) gene and trnL-trnF intergenic spacer, partial sequence; chloroplast	1118	1118	97%	0.0	98%	GU381495.1	1129,53	92,2%
Select seq gb|GU381628.1|	Thymbra calostachya voucher M:Ulrich s.n. tRNA-Leu (trnL) gene and trnL-trnF intergenic spacer, partial sequence; chloroplast	1127	1127	98%	0.0	98%	GU381628.1	1127,00	92,0%
Select seq gb|GU381481.1|	Micromeria cf. madagascariensis Morawetz 205 tRNA-Leu (trnL) gene and trnL-trnF intergenic spacer, partial sequence; chloroplast	1131	1131	98%	0.0	97%	GU381481.1	1119,46	91,4%
Select seq gb|GU381485.1|	Micromeria sphaerophylla voucher E:Lewis et al 1064 tRNA-Leu (trnL) gene and trnL-trnF intergenic spacer, partial sequence; chloroplast	1129	1129	98%	0.0	97%	GU381485.1	1117,48	91,3%
Select seq gb|GU381507.1|	Ziziphora tenuior voucher MSB:Fayvush et al. 03-1503 tRNA-Leu (trnL) gene and trnL-trnF intergenic spacer, partial sequence; chloroplast	1125	1125	98%	0.0	97%	GU381507.1	1113,52	90,9%
Select seq gb|GU381489.1|	Killickia lutea voucher NU<ZAF>:Hilliard & Burtt 9876 tRNA-Leu (trnL) gene and trnL-trnF intergenic spacer, partial sequence; chloroplast	1122	1122	98%	0.0	97%	GU381489.1	1110,55	90,7%
Select seq gb|GU381488.1|	Killickia compacta voucher M:Braeuchler 3816 tRNA-Leu (trnL) gene and trnL-trnF intergenic spacer, partial sequence; chloroplast	1122	1122	98%	0.0	97%	GU381488.1	1110,55	90,7%
Select seq gb|DQ667501.1|	Ziziphora taurica isolate x262 tRNA-Leu (trnL) gene and trnL-trnF intergenic spacer, partial sequence; chloroplast	1122	1122	98%	0.0	97%	DQ667501.1	1110,55	90,7%
Select seq gb|KR150214.1|	Satureja sp. 1 Kh04 trnL-trnF intergenic spacer region, partial sequence; chloroplast	1099	1099	96%	0.0	97%	KR150214.1	1110,45	90,7%
Select seq gb|KR150243.1|	Ziziphora sp. 2 Kh20 trnL-trnF intergenic spacer region, partial sequence; chloroplast	1110	1110	97%	0.0	97%	KR150243.1	1110,00	90,7%
Select seq gb|KR150198.1|	Ziziphora sp. 3 Kh112 trnL-trnF intergenic spacer region, partial sequence; chloroplast	1120	1120	98%	0.0	97%	KR150198.1	1108,57	90,5%
Select seq gb|GU381510.1|	Ziziphora pamiroalaica voucher C:Murray et al. 10090 tRNA-Leu (trnL) gene and trnL-trnF intergenic spacer, partial sequence; chloroplast	1120	1120	98%	0.0	97%	GU381510.1	1108,57	90,5%
Select seq gb|GU381505.1|	Clinopodium troodi voucher W:Davis 1856 tRNA-Leu (trnL) gene and trnL-trnF intergenic spacer, partial sequence; chloroplast	1120	1120	98%	0.0	97%	GU381505.1	1108,57	90,5%
Select seq gb|GU381501.1|	Clinopodium graveolens subsp. rotundifolium voucher M:Podlech 47181 tRNA-Leu (trnL) gene and trnL-trnF intergenic spacer, partial sequence; chloroplast	1120	1120	98%	0.0	97%	GU381501.1	1108,57	90,5%
Select seq gb|GU381499.1|	Clinopodium suaveolens voucher M:Erben s.n. tRNA-Leu (trnL) gene and trnL-trnF intergenic spacer, partial sequence; chloroplast	1120	1120	98%	0.0	97%	GU381499.1	1108,57	90,5%
Select seq gb|GU381500.1|	Clinopodium nanum voucher M:Braeuchler 2796 tRNA-Leu (trnL) gene and trnL-trnF intergenic spacer, partial sequence; chloroplast	1107	1107	97%	0.0	97%	GU381500.1	1107,00	90,4%
Select seq gb|GU381621.1|	Satureja cuneifolia voucher M:Rechinger 11142 tRNA-Leu (trnL) gene and trnL-trnF intergenic spacer, partial sequence; chloroplast	1116	1116	98%	0.0	97%	GU381621.1	1104,61	90,2%
Select seq gb|GU381612.1|	Gontscharovia popovii voucher BM<GBR-LONDON>:Schmid 2419 tRNA-Leu (trnL) gene and trnL-trnF intergenic spacer, partial sequence; chloroplast	1116	1116	98%	0.0	97%	GU381612.1	1104,61	90,2%
Select seq gb|AY840179.1|	Satureja montana tRNA-Leu (trnL) gene and trnL-trnF intergenic spacer, partial sequence; chloroplast	1116	1116	98%	0.0	97%	AY840179.1	1104,61	90,2%
Select seq gb|GU381497.1|	Clinopodium acinos voucher M:Podlech 50287 tRNA-Leu (trnL) gene and trnL-trnF intergenic spacer, partial sequence; chloroplast	1114	1114	98%	0.0	97%	GU381497.1	1102,63	90,0%
Select seq gb|KR063656.1|	Satureja pilosa subsp. origanita tRNA-Leu (trnL) gene, partial sequence; trnL-trnF intergenic spacer, complete sequence; and tRNA-Phe (trnF) gene, partial sequence; chloroplast	1134	1134	100%	0.0	97%	KR063656.1	1099,98	89,8%
Select seq gb|KR150238.1|	Ziziphora sp. 1 Kh75 trnL-trnF intergenic spacer region, partial sequence; chloroplast	1133	1133	100%	0.0	97%	KR150238.1	1099,01	89,8%
Select seq gb|JQ669067.1|	Satureja montana voucher UCBG 2002.0593, Forbes s.n tRNA-Leu (trnL) gene and trnL-trnF intergenic spacer, partial sequence; plastid	1110	1110	98%	0.0	97%	JQ669067.1	1098,67	89,7%
Select seq gb|GU381619.1|	Satureja mutica voucher M:Akhani 12362 tRNA-Leu (trnL) gene and trnL-trnF intergenic spacer, partial sequence; chloroplast	1110	1110	98%	0.0	97%	GU381619.1	1098,67	89,7%
Select seq gb|GU381614.1|	Satureja thymbra voucher M:Braeuchler 2896 tRNA-Leu (trnL) gene and trnL-trnF intergenic spacer, partial sequence; chloroplast	1110	1110	98%	0.0	97%	GU381614.1	1098,67	89,7%
Select seq gb|GU381611.1|	Gontscharovia popovii voucher M:Vvedensky s.n. tRNA-Leu (trnL) gene and trnL-trnF intergenic spacer, partial sequence; chloroplast	1110	1110	98%	0.0	97%	GU381611.1	1098,67	89,7%
Select seq gb|GU381521.1|	Mentha pulegium voucher M:Braeuchler 2300 tRNA-Leu (trnL) gene and trnL-trnF intergenic spacer, partial sequence; chloroplast	1110	1110	98%	0.0	97%	GU381521.1	1098,67	89,7%
Select seq gb|JQ669021.1|	Clinopodium acinos voucher Judziewicz 14160 tRNA-Leu (trnL) gene and trnL-trnF intergenic spacer, partial sequence; plastid	1109	1109	98%	0.0	97%	JQ669021.1	1097,68	89,6%

Select seq gb|KR150233.1|	Thymus sp. 6 Kh133 trnL-trnF intergenic spacer region, partial sequence; chloroplast	1120	1120	99% 0.0	97% KR150233.1	1097,37	89,6%
Select seq gb|KR150209.1|	Satureja sp. 3 Kh90 trnL-trnF intergenic spacer region, partial sequence; chloroplast	1120	1120	99% 0.0	97% KR150209.1	1097,37	89,6%
Select seq gb|GU381625.1|	Pentapleura subulifera voucher W:Rechinger 12085 tRNA-Leu (trnL) gene and trnL-trnF intergenic spacer, partial sequence; chloroplast	1107	1107	98% 0.0	97% GU381625.1	1095,70	89,5%
Select seq gb|GU381615.1|	Satureja innota voucher M:Barra et al. 2484GL tRNA-Leu (trnL) gene and trnL-trnF intergenic spacer, partial sequence; chloroplast	1105	1105	98% 0.0	97% GU381615.1	1093,72	89,3%
Select seq gb|JQ669068.1|	Satureja thymbra voucher UCBG 2002.0540, Forbes s.n. tRNA-Leu (trnL) gene and trnL-trnF intergenic spacer, partial sequence; plastid	1099	1099	98% 0.0	97% JQ669068.1	1087,79	88,8%
Select seq gb|GU381623.1|	Satureja spicigera voucher M:Kartli et al. 2726 tRNA-Leu (trnL) gene and trnL-trnF intergenic spacer, partial sequence; chloroplast	1099	1099	98% 0.0	97% GU381623.1	1087,79	88,8%
Select seq gb|GU381522.1|	Mentha cervina voucher M:Braeuchler 2394 tRNA-Leu (trnL) gene and trnL-trnF intergenic spacer, partial sequence; chloroplast	1098	1098	98% 0.0	97% GU381522.1	1086,80	88,8%
Select seq gb|KC414276.1|	Mentha canadensis isolate 511190001 tRNA-Leu (trnL) gene and trnL-trnF intergenic spacer, partial sequence; chloroplast	1110	1110	100% 0.0	97% KC414276.1	1076,70	87,9%
Select seq emb|AJ505541.1|	Mentha suaveolens plastid trnL-trnF intergenic spacer, specimen voucher cult., K-1970-3169 (K)	1109	1109	100% 0.0	97% AJ505541.1	1075,73	87,9%
Select seq gb|GU381630.1|	Satureja thymbrifolia voucher M:Danin s.n. tRNA-Leu (trnL) gene and trnL-trnF intergenic spacer, partial sequence; chloroplast	1098	1098	98% 0.0	96% GU381630.1	1075,59	87,8%
Select seq gb|DQ667492.1|	Lepechinia lancifolia isolate x232 tRNA-Leu (trnL) gene and trnL-trnF intergenic spacer, partial sequence; chloroplast	1107	1107	100% 0.0	97% DQ667492.1	1073,79	87,7%
Select seq gb|FJ593456.1|	Mentha sp. JSZ-2009a tRNA-Leu (trnL) gene and trnL-trnF intergenic spacer, partial sequence; chloroplast	1103	1103	100% 0.0	96% FJ593456.1	1058,88	86,5%
Select seq gb|KR150240.1|	Satureja sp. 2 Kh70 trnL-trnF intergenic spacer region, partial sequence; chloroplast	1101	1101	100% 0.0	96% KR150240.1	1056,96	86,3%
						0,00	0,0%
						0,00	0,0%
						0,00	0,0%
						0,00	0,0%

Select for downloading or viewing reports	Kh066_trnL Description	Max score	Total score	Query cover	E value	Ident	Accession	(Ident/Cover)* Max score	Deviation from top hit
Select seq gb|JN408625.1|	Teucrium capitatum tRNA-Leu (trnL) gene, partial sequence; and trnL-trnF intergenic spacer, complete sequence; chloroplast	1153	1153	93%	0.0	99%	JN408625.1	1227,39	100,0%
Select seq gb|JN408624.1|	Teucrium capitatum tRNA-Leu (trnL) gene, partial sequence; and trnL-trnF intergenic spacer, complete sequence; chloroplast	1153	1153	93%	0.0	99%	JN408624.1	1227,39	100,0%
Select seq gb|JN408623.1|	Teucrium aureum tRNA-Leu (trnL) gene, partial sequence; and trnL-trnF intergenic spacer, complete sequence; chloroplast	1153	1153	93%	0.0	99%	JN408623.1	1227,39	100,0%
Select seq gb|JN408621.1|	Teucrium pumilum tRNA-Leu (trnL) gene, partial sequence; and trnL-trnF intergenic spacer, complete sequence; chloroplast	1153	1153	93%	0.0	99%	JN408621.1	1227,39	100,0%
Select seq gb|JN408660.1|	Teucrium pyrenaicum tRNA-Leu (trnL) gene, partial sequence; and trnL-trnF intergenic spacer, complete sequence; chloroplast	1190	1190	96%	0.0	99%	JN408660.1	1227,19	100,0%
Select seq gb|JN408657.1|	Teucrium polium tRNA-Leu (trnL) gene, partial sequence; and trnL-trnF intergenic spacer, complete sequence; chloroplast	1151	1151	93%	0.0	99%	JN408657.1	1225,26	99,8%
Select seq gb|JN408646.1|	Teucrium montanum tRNA-Leu (trnL) gene, partial sequence; and trnL-trnF intergenic spacer, complete sequence; chloroplast	1151	1151	93%	0.0	99%	JN408646.1	1225,26	99,8%
Select seq gb|JQ044780.1|	Teucrium polium subsp. polium isolate TP12 tRNA-Leu (trnL) gene, partial sequence; trnL-trnF intergenic spacer, complete sequence; and tRNA-Phe (trnF) gene, partial sequence; chloroplast	1225	1225	99%	0.0	99%	JQ044780.1	1225,00	99,8%
Select seq gb|JQ044779.1|	Teucrium polium subsp. polium isolate TP11 tRNA-Leu (trnL) gene, partial sequence; trnL-trnF intergenic spacer, complete sequence; and tRNA-Phe (trnF) gene, partial sequence; chloroplast	1225	1225	99%	0.0	99%	JQ044779.1	1225,00	99,8%
Select seq gb|JQ044778.1|	Teucrium polium subsp. polium isolate TP10 tRNA-Leu (trnL) gene, partial sequence; trnL-trnF intergenic spacer, complete sequence; and tRNA-Phe (trnF) gene, partial sequence; chloroplast	1225	1225	99%	0.0	99%	JQ044778.1	1225,00	99,8%
Select seq gb|JN408620.1|	Teucrium aureum tRNA-Leu (trnL) gene, partial sequence; and trnL-trnF intergenic spacer, complete sequence; chloroplast	1199	1199	97%	0.0	99%	JN408620.1	1223,72	99,7%
Select seq gb|JN408619.1|	Teucrium capitatum tRNA-Leu (trnL) gene, partial sequence; and trnL-trnF intergenic spacer, complete sequence; chloroplast	1197	1197	97%	0.0	99%	JN408619.1	1221,68	99,5%
Select seq gb|JN408616.1|	Teucrium cyprium subsp. cyprium tRNA-Leu (trnL) gene, partial sequence; and trnL-trnF intergenic spacer, complete sequence; chloroplast	1195	1195	97%	0.0	99%	JN408616.1	1219,64	99,4%
Select seq gb|JQ044781.1|	Teucrium polium subsp. polium isolate TP19 tRNA-Leu (trnL) gene, partial sequence; trnL-trnF intergenic spacer, complete sequence; and tRNA-Phe (trnF) gene, partial sequence; chloroplast	1219	1219	99%	0.0	99%	JQ044781.1	1219,00	99,3%
Select seq gb|JQ044774.1|	Teucrium polium subsp. polium isolate TP20 tRNA-Leu (trnL) gene, partial sequence; trnL-trnF intergenic spacer, complete sequence; and tRNA-Phe (trnF) gene, partial sequence; chloroplast	1219	1219	99%	0.0	99%	JQ044774.1	1219,00	99,3%
Select seq gb|JN408597.1|	Teucrium alpestre tRNA-Leu (trnL) gene, partial sequence; trnL-trnF intergenic spacer, complete sequence; and tRNA-Phe (trnF) gene, partial sequence; chloroplast	1229	1229	100%	0.0	99%	JN408597.1	1216,71	99,1%
Select seq gb|FJ952080.1|	Teucrium pyrenaicum tRNA-Leu (trnL) gene, partial sequence; trnL-trnF intergenic spacer, complete sequence; and tRNA-Phe (trnF) gene, partial sequence; chloroplast	1229	1229	100%	0.0	99%	FJ952080.1	1216,71	99,1%
Select seq gb|JQ044782.1|	Teucrium polium subsp. polium isolate TP13 tRNA-Leu (trnL) gene, partial sequence; trnL-trnF intergenic spacer, complete sequence; and tRNA-Phe (trnF) gene, partial sequence; chloroplast	1214	1214	99%	0.0	98%	JQ044782.1	1201,74	97,9%
Select seq gb|JQ044777.1|	Teucrium polium subsp. polium isolate TP5 tRNA-Leu (trnL) gene, partial sequence; trnL-trnF intergenic spacer, complete sequence; and tRNA-Phe (trnF) gene, partial sequence; chloroplast	1214	1214	99%	0.0	98%	JQ044777.1	1201,74	97,9%
Select seq gb|JQ044776.1|	Teucrium polium subsp. polium isolate TP4 tRNA-Leu (trnL) gene, partial sequence; trnL-trnF intergenic spacer, complete sequence; and tRNA-Phe (trnF) gene, partial sequence; chloroplast	1214	1214	99%	0.0	98%	JQ044776.1	1201,74	97,9%
Select seq gb|JQ044775.1|	Teucrium polium subsp. polium isolate TP3 tRNA-Leu (trnL) gene, partial sequence; trnL-trnF intergenic spacer, complete sequence; and tRNA-Phe (trnF) gene, partial sequence; chloroplast	1214	1214	99%	0.0	98%	JQ044775.1	1201,74	97,9%
Select seq gb|JQ044771.1|	Teucrium polium subsp. capitatum isolate TPC2 tRNA-Leu (trnL) gene, partial sequence; trnL-trnF intergenic spacer, complete sequence; and tRNA-Phe (trnF) gene, partial sequence; chloroplast	1201	1201	99%	0.0	98%	JQ044771.1	1188,87	96,9%
Select seq gb|JQ044773.1|	Teucrium polium subsp. capitatum isolate TPC1 tRNA-Leu (trnL) gene, partial sequence; trnL-trnF intergenic spacer, complete sequence; and tRNA-Phe (trnF) gene, partial sequence; chloroplast	1195	1195	99%	0.0	98%	JQ044773.1	1182,93	96,4%
Select seq gb|JQ044772.1|	Teucrium polium subsp. capitatum isolate TPC4 tRNA-Leu (trnL) gene, partial sequence; trnL-trnF intergenic spacer, complete sequence; and tRNA-Phe (trnF) gene, partial sequence; chloroplast	1195	1195	99%	0.0	98%	JQ044772.1	1182,93	96,4%
Select seq gb|JN408632.1|	Teucrium heterophyllum tRNA-Leu (trnL) gene, partial sequence; and trnL-trnF intergenic spacer, complete sequence; chloroplast	1160	1160	97%	0.0	98%	JN408632.1	1171,96	95,5%
Select seq gb|JN408594.1|	Teucrium antitauricum tRNA-Leu (trnL) gene, partial sequence; and trnL-trnF intergenic spacer, complete sequence; chloroplast	1140	1140	97%	0.0	97%	JN408594.1	1140,00	92,9%

Select seq gb|JN408631.1|	Teucrium halacsyanum tRNA-Leu (trnL) gene, partial sequence; and trnL-trnF intergenic spacer, complete sequence; chloroplast	1099	1099	94% 0.0	97% JN408631.1	1134,07	92,4%
Select seq gb|JN408647.1|	Teucrium montbretii subsp. heliotropiifolium tRNA-Leu (trnL) gene, partial sequence; and trnL-trnF intergenic spacer, complete sequence; chloroplast	1068	1068	92% 0.0	97% JN408647.1	1126,04	91,7%
Select seq gb|JN408676.1|	Teucrium viscidum var. miquelianum tRNA-Leu (trnL) gene, partial sequence; and trnL-trnF intergenic spacer, complete sequence; chloroplast	1090	1090	94% 0.0	97% JN408676.1	1124,79	91,6%
Select seq gb|JN408672.1|	Teucrium lamiifolium subsp. stachyophyllum tRNA-Leu (trnL) gene, partial sequence; and trnL-trnF intergenic spacer, complete sequence; chloroplast	1122	1122	97% 0.0	97% JN408672.1	1122,00	91,4%
Select seq gb|JN408636.1|	Teucrium japonicum tRNA-Leu (trnL) gene, partial sequence; and trnL-trnF intergenic spacer, complete sequence; chloroplast	1120	1120	97% 0.0	97% JN408636.1	1120,00	91,3%
Select seq gb|JN408661.1|	Teucrium quadrifarium tRNA-Leu (trnL) gene, partial sequence; and trnL-trnF intergenic spacer, complete sequence; chloroplast	1072	1072	93% 0.0	97% JN408661.1	1118,11	91,1%
Select seq gb|JN408665.1|	Teucrium rupestre tRNA-Leu (trnL) gene, partial sequence; and trnL-trnF intergenic spacer, complete sequence; chloroplast	1057	1057	92% 0.0	97% JN408665.1	1114,45	90,8%
Select seq gb|JN408664.1|	Teucrium royleanum tRNA-Leu (trnL) gene, partial sequence; and trnL-trnF intergenic spacer, complete sequence; chloroplast	1114	1114	97% 0.0	97% JN408664.1	1114,00	90,8%
Select seq gb|JN408628.1|	Teucrium edelbergii tRNA-Leu (trnL) gene, partial sequence; and trnL-trnF intergenic spacer, complete sequence; chloroplast	1099	1099	96% 0.0	97% JN408628.1	1110,45	90,5%
Select seq gb|JN408618.1|	Teucrium demnatense tRNA-Leu (trnL) gene, partial sequence; and trnL-trnF intergenic spacer, complete sequence; chloroplast	1109	1109	97% 0.0	97% JN408618.1	1109,00	90,4%
Select seq gb|JN408649.1|	Teucrium odontites tRNA-Leu (trnL) gene, partial sequence; and trnL-trnF intergenic spacer, complete sequence; chloroplast	1118	1118	97% 0.0	96% JN408649.1	1106,47	90,1%
Select seq gb|JN408633.1|	Teucrium hircanicum tRNA-Leu (trnL) gene, partial sequence; and trnL-trnF intergenic spacer, complete sequence; chloroplast	1053	1053	92% 0.0	96% JN408633.1	1098,78	89,5%
Select seq gb|JN408598.1|	Teucrium arduinoi tRNA-Leu (trnL) gene, partial sequence; and trnL-trnF intergenic spacer, complete sequence; chloroplast	1110	1110	97% 0.0	96% JN408598.1	1098,56	89,5%
Select seq gb|JN408674.1|	Teucridium parvifolium tRNA-Leu (trnL) gene, partial sequence; trnL-trnF intergenic spacer, complete sequence; and tRNA-Phe (trnF) gene, partial sequence; chloroplast	1129	1129	99% 0.0	96% JN408674.1	1094,79	89,2%
Select seq gb|JN408640.1|	Teucrium lamiifolium subsp. lamiifolium tRNA-Leu (trnL) gene, partial sequence; and trnL-trnF intergenic spacer, complete sequence; chloroplast	1098	1098	97% 0.0	96% JN408640.1	1086,68	88,5%
Select seq gb|JN408669.1|	Teucrium scorodonia tRNA-Leu (trnL) gene, partial sequence; and trnL-trnF intergenic spacer, complete sequence; chloroplast	1085	1085	96% 0.0	96% JN408669.1	1085,00	88,4%
Select seq gb|JN408666.1|	Teucrium salviastrum tRNA-Leu (trnL) gene, partial sequence; and trnL-trnF intergenic spacer, complete sequence; chloroplast	1085	1085	96% 0.0	96% JN408666.1	1085,00	88,4%
Select seq gb|JN408603.1|	Teucrium asiaticum tRNA-Leu (trnL) gene, partial sequence; and trnL-trnF intergenic spacer, complete sequence; chloroplast	1085	1085	96% 0.0	96% JN408603.1	1085,00	88,4%
Select seq gb|JN408648.1|	Teucrium nudicaule tRNA-Leu (trnL) gene, partial sequence; and trnL-trnF intergenic spacer, complete sequence; chloroplast	1081	1081	96% 0.0	96% JN408648.1	1081,00	88,1%
Select seq gb|JN408627.1|	Teucrium scorodonia tRNA-Leu (trnL) gene, partial sequence; and trnL-trnF intergenic spacer, complete sequence; chloroplast	1046	1046	93% 0.0	96% JN408627.1	1079,74	88,0%
Select seq gb|JF694866.1|	Teucrium scorodonia isolate TSC8 tRNA-Leu (trnL) gene and trnL-trnF intergenic spacer, complete sequence; and tRNA-Phe (trnF) gene, partial sequence; plastid	1112	1112	99% 0.0	96% JF694866.1	1078,30	87,9%
Select seq gb|JF694861.1|	Teucrium scorodonia isolate TSC3 tRNA-Leu (trnL) gene and trnL-trnF intergenic spacer, complete sequence; and tRNA-Phe (trnF) gene, partial sequence; plastid	1112	1112	99% 0.0	96% JF694861.1	1078,30	87,9%
Select seq gb|JF694859.1|	Teucrium scorodonia isolate TSC1 tRNA-Leu (trnL) gene and trnL-trnF intergenic spacer, complete sequence; and tRNA-Phe (trnF) gene, partial sequence; plastid	1112	1112	99% 0.0	96% JF694859.1	1078,30	87,9%
Select seq gb|JN408607.1|	Teucrium bracteatum tRNA-Leu (trnL) gene, partial sequence; and trnL-trnF intergenic spacer, complete sequence; chloroplast	1088	1088	97% 0.0	96% JN408607.1	1076,78	87,7%
Select seq gb|JN408595.1|	Teucrium abutiloides tRNA-Leu (trnL) gene, partial sequence; trnL-trnF intergenic spacer, complete sequence; and tRNA-Phe (trnF) gene, partial sequence; chloroplast	1110	1110	99% 0.0	96% JN408595.1	1076,36	87,7%
Select seq gb|JN408600.1|	Teucrium cubense tRNA-Leu (trnL) gene, partial sequence; trnL-trnF intergenic spacer, complete sequence; and tRNA-Phe (trnF) gene, partial sequence; chloroplast	1120	1120	100% 0.0	96% JN408600.1	1075,20	87,6%
Select seq gb|JN408667.1|	Teucrium francoi tRNA-Leu (trnL) gene, partial sequence; and trnL-trnF intergenic spacer, complete sequence; chloroplast	1086	1086	97% 0.0	96% JN408667.1	1074,80	87,6%
Select seq gb|JN408643.1|	Teucrium massiliense tRNA-Leu (trnL) gene, partial sequence; and trnL-trnF intergenic spacer, complete sequence; chloroplast	1086	1086	97% 0.0	96% JN408643.1	1074,80	87,6%

Select seq gb|JN408605.1|	Teucrium bicolor tRNA-Leu (trnL) gene, partial sequence; and trnL-trnF intergenic spacer, complete sequence; chloroplast	1085	1085	97%	0.0	96%	JN408605.1	1073,81	87,5%
Select seq gb|JF694864.1|	Teucrium scorodonia isolate TSC6 tRNA-Leu (trnL) gene and trnL-trnF intergenic spacer, complete sequence; and tRNA-Phe (trnF) gene, partial sequence; plastid	1107	1107	99%	0.0	96%	JF694864.1	1073,45	87,5%
Select seq gb|JN408591.1|	Spartothamnella puberula tRNA-Leu (trnL) gene, partial sequence; trnL-trnF intergenic spacer, complete sequence; and tRNA-Phe (trnF) gene, partial sequence; chloroplast	1059	1059	95%	0.0	96%	JN408591.1	1070,15	87,2%
Select seq gb|JN408639.1|	Teucrium laciniatum tRNA-Leu (trnL) gene, partial sequence; and trnL-trnF intergenic spacer, complete sequence; chloroplast	1044	1044	94%	0.0	96%	JN408639.1	1066,21	86,9%
Select seq gb|JN408610.1|	Teucrium trifidum tRNA-Leu (trnL) gene, partial sequence; and trnL-trnF intergenic spacer, complete sequence; chloroplast	1044	1044	94%	0.0	96%	JN408610.1	1066,21	86,9%
Select seq gb|JN408675.1|	Teucrium trifidum tRNA-Leu (trnL) gene, partial sequence; and trnL-trnF intergenic spacer, complete sequence; chloroplast	1053	1053	95%	0.0	96%	JN408675.1	1064,08	86,7%
Select seq gb|JN408590.1|	Oncinocalyx betchei tRNA-Leu (trnL) gene, partial sequence; trnL-trnF intergenic spacer, complete sequence; and tRNA-Phe (trnF) gene, partial sequence; chloroplast	1053	1053	95%	0.0	96%	JN408590.1	1064,08	86,7%
Select seq gb|JN408658.1|	Teucrium pruinosum tRNA-Leu (trnL) gene, partial sequence; and trnL-trnF intergenic spacer, complete sequence; chloroplast	1068	1068	97%	0.0	95%	JN408658.1	1045,98	85,2%
Select seq gb|JN408677.1|	Teucrium webbianum tRNA-Leu (trnL) gene, partial sequence; and trnL-trnF intergenic spacer, complete sequence; chloroplast	1066	1066	97%	0.0	95%	JN408677.1	1044,02	85,1%
Select seq gb|JN408655.1|	Teucrium chamaedrys tRNA-Leu (trnL) gene, partial sequence; and trnL-trnF intergenic spacer, complete sequence; chloroplast	1011	1011	92%	0.0	95%	JN408655.1	1043,97	85,1%
Select seq gb|HQ646990.1|	Teucrium flavum subsp. glaucum isolate TFG64 tRNA-Leu (trnL) gene and trnL-trnF intergenic spacer, complete sequence; and tRNA-Phe (trnF) gene, partial sequence; chloroplast	1085	1085	99%	0.0	95%	HQ646990.1	1041,16	84,8%
Select seq gb|HQ646981.1|	Teucrium flavum subsp. glaucum isolate TFG54 tRNA-Leu (trnL) gene and trnL-trnF intergenic spacer, complete sequence; and tRNA-Phe (trnF) gene, partial sequence; chloroplast	1085	1085	99%	0.0	95%	HQ646981.1	1041,16	84,8%
Select seq gb|JN408650.1|	Teucrium oliverianum tRNA-Leu (trnL) gene, partial sequence; and trnL-trnF intergenic spacer, complete sequence; chloroplast	1062	1062	97%	0.0	95%	JN408650.1	1040,10	84,7%
Select seq gb|JN408626.1|	Teucrium chamaedrys tRNA-Leu (trnL) gene, partial sequence; and trnL-trnF intergenic spacer, complete sequence; chloroplast	1018	1018	93%	0.0	95%	JN408626.1	1039,89	84,7%
Select seq gb|JN408612.1|	Teucrium chamaedrys tRNA-Leu (trnL) gene, partial sequence; and trnL-trnF intergenic spacer, complete sequence; chloroplast	1059	1059	97%	0.0	95%	JN408612.1	1037,16	84,5%
Select seq gb|JN408670.1|	Teucrium sessiliflorum tRNA-Leu (trnL) gene, partial sequence; and trnL-trnF intergenic spacer, complete sequence; chloroplast	1014	1014	93%	0.0	95%	JN408670.1	1035,81	84,4%
Select seq gb|HQ646991.1|	Teucrium flavum subsp. flavum isolate TFF56 tRNA-Leu (trnL) gene and trnL-trnF intergenic spacer, complete sequence; and tRNA-Phe (trnF) gene, partial sequence; chloroplast	1079	1079	99%	0.0	95%	HQ646991.1	1035,40	84,4%
Select seq gb|JN408602.1|	Teucrium aroanium tRNA-Leu (trnL) gene, partial sequence; and trnL-trnF intergenic spacer, complete sequence; chloroplast	1057	1057	97%	0.0	95%	JN408602.1	1035,21	84,3%
Select seq gb|JN408635.1|	Teucrium intricatum tRNA-Leu (trnL) gene, partial sequence; and trnL-trnF intergenic spacer, complete sequence; chloroplast	1046	1046	96%	0.0	95%	JN408635.1	1035,10	84,3%
Select seq gb|JN408606.1|	Teucrium botrys tRNA-Leu (trnL) gene, partial sequence; and trnL-trnF intergenic spacer, complete sequence; chloroplast	1046	1046	96%	0.0	95%	JN408606.1	1035,10	84,3%
Select seq gb|JN408645.1|	Teucrium microphyllum tRNA-Leu (trnL) gene, partial sequence; and trnL-trnF intergenic spacer, complete sequence; chloroplast	1053	1053	97%	0.0	95%	JN408645.1	1031,29	84,0%
Select seq gb|JN408641.1|	Teucrium lucidum tRNA-Leu (trnL) gene, partial sequence; and trnL-trnF intergenic spacer, complete sequence; chloroplast	1053	1053	97%	0.0	95%	JN408641.1	1031,29	84,0%
Select seq gb|JN408593.1|	Teucrium flavum subsp. glaucum tRNA-Leu (trnL) gene, partial sequence; and trnL-trnF intergenic spacer, complete sequence; chloroplast	1053	1053	97%	0.0	95%	JN408593.1	1031,29	84,0%
Select seq gb|GU331787.1|	Teucrium marum isolate TM12N tRNA-Leu (trnL) gene, partial sequence; trnL-trnF intergenic spacer, complete sequence; and tRNA-Phe (trnF) gene, partial sequence; chloroplast	1074	1074	99%	0.0	95%	GU331787.1	1030,61	84,0%
Select seq gb|GU331782.1|	Teucrium marum isolate TM1C tRNA-Leu (trnL) gene, partial sequence; trnL-trnF intergenic spacer, complete sequence; and tRNA-Phe (trnF) gene, partial sequence; chloroplast	1074	1074	99%	0.0	95%	GU331782.1	1030,61	84,0%
Select seq gb|JN408668.1|	Teucrium scordium tRNA-Leu (trnL) gene, partial sequence; and trnL-trnF intergenic spacer, complete sequence; chloroplast	1051	1051	97%	0.0	95%	JN408668.1	1029,33	83,9%
Select seq gb|JN408673.1|	Teucrium subspinosum tRNA-Leu (trnL) gene, partial sequence; and trnL-trnF intergenic spacer, complete sequence; chloroplast	1038	1038	96%	0.0	95%	JN408673.1	1027,19	83,7%
Select seq gb|GU331791.1|	Teucrium marum isolate TM16W tRNA-Leu (trnL) gene, partial sequence; trnL-trnF intergenic spacer, complete sequence; and tRNA-Phe (trnF) gene, partial sequence; chloroplast	1068	1068	99%	0.0	95%	GU331791.1	1024,85	83,5%

Select seq gb|JN408644.1|	Teucrium melissoides tRNA-Leu (trnL) gene, partial sequence; and trnL-trnF intergenic spacer, complete sequence; chloroplast	1046	1046	97% 0.0	95% JN408644.1	1024,43	83,5%
Select seq gb|JN408604.1|	Teucrium betonicum tRNA-Leu (trnL) gene, partial sequence; trnL-trnF intergenic spacer, complete sequence; and tRNA-Phe (trnF) gene, partial sequence; chloroplast	1044	1044	97% 0.0	95% JN408604.1	1022,47	83,3%
Select seq gb|JN408652.1|	Teucrium oxylepis tRNA-Leu (trnL) gene, partial sequence; and trnL-trnF intergenic spacer, complete sequence; chloroplast	1042	1042	97% 0.0	95% JN408652.1	1020,52	83,1%
Select seq gb|GU331790.1|	Teucrium marum isolate TM23W tRNA-Leu (trnL) gene, partial sequence; trnL-trnF intergenic spacer, complete sequence; and tRNA-Phe (trnF) gene, partial sequence; chloroplast	1062	1062	99% 0.0	95% GU331790.1	1019,09	83,0%
Select seq gb|GU331789.1|	Teucrium marum isolate TM15S tRNA-Leu (trnL) gene, partial sequence; trnL-trnF intergenic spacer, complete sequence; and tRNA-Phe (trnF) gene, partial sequence; chloroplast	1062	1062	99% 0.0	95% GU331789.1	1019,09	83,0%
Select seq gb|GU331786.1|	Teucrium marum isolate TM5N tRNA-Leu (trnL) gene, partial sequence; trnL-trnF intergenic spacer, complete sequence; and tRNA-Phe (trnF) gene, partial sequence; chloroplast	1062	1062	99% 0.0	95% GU331786.1	1019,09	83,0%
Select seq gb|GU331785.1|	Teucrium marum isolate TM20C tRNA-Leu (trnL) gene, partial sequence; trnL-trnF intergenic spacer, complete sequence; and tRNA-Phe (trnF) gene, partial sequence; chloroplast	1062	1062	99% 0.0	95% GU331785.1	1019,09	83,0%
Select seq gb|GU331784.1|	Teucrium marum isolate TM8C tRNA-Leu (trnL) gene, partial sequence; trnL-trnF intergenic spacer, complete sequence; and tRNA-Phe (trnF) gene, partial sequence; chloroplast	1062	1062	99% 0.0	95% GU331784.1	1019,09	83,0%
Select seq gb|GU331783.1|	Teucrium marum isolate TM6C tRNA-Leu (trnL) gene, partial sequence; trnL-trnF intergenic spacer, complete sequence; and tRNA-Phe (trnF) gene, partial sequence; chloroplast	1062	1062	99% 0.0	95% GU331783.1	1019,09	83,0%
Select seq gb|JN408642.1|	Teucrium marum tRNA-Leu (trnL) gene, partial sequence; and trnL-trnF intergenic spacer, complete sequence; chloroplast	1040	1040	97% 0.0	95% JN408642.1	1018,56	83,0%
Select seq gb|JN408637.1|	Teucrium jolyi tRNA-Leu (trnL) gene, partial sequence; and trnL-trnF intergenic spacer, complete sequence; chloroplast	1038	1038	97% 0.0	95% JN408637.1	1016,60	82,8%
Select seq gb|GU331788.1|	Teucrium marum isolate TM25S tRNA-Leu (trnL) gene, partial sequence; trnL-trnF intergenic spacer, complete sequence; and tRNA-Phe (trnF) gene, partial sequence; chloroplast	1057	1057	99% 0.0	95% GU331788.1	1014,29	82,6%
Select seq gb|JN408611.1|	Teucrium chamaedrys tRNA-Leu (trnL) gene, partial sequence; and trnL-trnF intergenic spacer, complete sequence; chloroplast	1044	1044	97% 0.0	94% JN408611.1	1011,71	82,4%
Select seq gb|JN408653.1|	Teucrium pseudochamaepitys tRNA-Leu (trnL) gene, partial sequence; and trnL-trnF intergenic spacer, complete sequence; chloroplast	1037	1037	97% 0.0	94% JN408653.1	1004,93	81,9%
Select seq gb|JN408608.1|	Teucrium brevifolium tRNA-Leu (trnL) gene, partial sequence; and trnL-trnF intergenic spacer, complete sequence; chloroplast	1029	1029	97% 0.0	94% JN408608.1	997,18	81,2%
						0,00	0,0%
						0,00	0,0%

Select for downloading or viewing reports	Kh067_trnL Description	Max score	Total score	Query cover	E value	Ident	Accession	(Ident/Cover)* Max score	Deviation from top hit
Select seq gb|JQ669045.1|	Hymenocrater bituminosus voucher K. Tamanyan & George Fayvush tRNA-Leu (trnL) gene and trnL-trnF intergenic spacer, partial sequence; plastid	1181	1181	94%	0.0	99%	JQ669045.1	1243,82	100,0%
Select seq gb|JQ669049.1|	Marmoritis complanatum voucher D.E. Boufford et al., 32012 tRNA-Leu (trnL) gene and trnL-trnF intergenic spacer, partial sequence; plastid	1144	1144	92%	0.0	99%	JQ669049.1	1231,04	99,0%
Select seq gb|KM886627.1|	Marmoritis complanata voucher T. Deng 2359 (KUN) tRNA-Leu (trnL) gene, partial sequence; trnL-trnF intergenic spacer, complete sequence; and tRNA-Phe (trnF) gene, partial sequence; plastid	1133	1133	92%	0.0	99%	KM886627.1	1219,21	98,0%
Select seq emb|AJ505431.1|	Nepeta menthoides plastid trnL-trnF intergenic spacer, specimen voucher Jamzad s.n. (K)	1096	1096	97%	0.0	96%	AJ505431.1	1084,70	87,2%
Select seq gb|FJ593457.1|	Nepeta stewartiana tRNA-Leu (trnL) gene and trnL-trnF intergenic spacer, partial sequence; chloroplast	1118	1118	100%	0.0	96%	FJ593457.1	1073,28	86,3%
Select seq gb|DQ667492.1|	Lepechinia lancifolia isolate x232 tRNA-Leu (trnL) gene and trnL-trnF intergenic spacer, partial sequence; chloroplast	1103	1103	99%	0.0	96%	DQ667492.1	1069,58	86,0%
Select seq gb|KF307433.1|	Lepechinia urbanii voucher B. Drew 135 tRNA-Leu (trnL) gene and trnL-trnF intergenic spacer, partial sequence; chloroplast	1024	1024	92%	0.0	96%	KF307433.1	1068,52	85,9%
Select seq gb|JF301383.1|	Lepechinia salviifolia voucher R. Jabaily s.n. tRNA-Leu (trnL) gene and trnL-trnF intergenic spacer, partial sequence; chloroplast	1022	1022	92%	0.0	96%	JF301383.1	1066,43	85,7%
Select seq gb|KF307430.1|	Lepechinia schiedeana voucher B. Drew 157 tRNA-Leu (trnL) gene and trnL-trnF intergenic spacer, partial sequence; chloroplast	1013	1013	92%	0.0	96%	KF307430.1	1057,04	85,0%
Select seq gb|KF307411.1|	Lepechinia bella voucher Rachel Jabaily s.n. tRNA-Leu (trnL) gene and trnL-trnF intergenic spacer, partial sequence; chloroplast	1013	1013	92%	0.0	96%	KF307411.1	1057,04	85,0%
Select seq gb|JF301382.1|	Lepechinia radula voucher B. Drew 185 tRNA-Leu (trnL) gene and trnL-trnF intergenic spacer, partial sequence; chloroplast	1024	1024	93%	0.0	96%	JF301382.1	1057,03	85,0%
Select seq gb|DQ667477.1|	Melissa officinalis isolate x193 tRNA-Leu (trnL) gene and trnL-trnF intergenic spacer, partial sequence; chloroplast	1050	1050	96%	0.0	96%	DQ667477.1	1050,00	84,4%
Select seq emb|AJ505529.1|	Melissa officinalis plastid trnL-trnF intergenic spacer, specimen voucher Catino	1038	1038	95%	0.0	96%	AJ505529.1	1048,93	84,3%
Select seq gb|DQ667487.1|	Nepeta cataria isolate x220 tRNA-Leu (trnL) gene and trnL-trnF intergenic spacer, partial sequence; chloroplast	1092	1092	99%	0.0	95%	DQ667487.1	1047,88	84,2%
Select seq gb|DQ667517.1|	Drepanocaryum sewerzowii isolate x523 tRNA-Leu (trnL) gene and trnL-trnF intergenic spacer, partial sequence; chloroplast	1057	1057	96%	0.0	95%	DQ667517.1	1045,99	84,1%
Select seq gb|JF301386.1|	Melissa officinalis voucher B. Drew 70 tRNA-Leu (trnL) gene and trnL-trnF intergenic spacer, partial sequence; chloroplast	1011	1011	93%	0.0	96%	JF301386.1	1043,61	83,9%
Select seq gb|JF301391.1|	Nepeta cataria voucher B. Drew 72 tRNA-Leu (trnL) gene and trnL-trnF intergenic spacer, partial sequence; chloroplast	1018	1018	93%	0.0	95%	JF301391.1	1039,89	83,6%
Select seq gb|JF301361.1|	Chaunostoma mecistandrum voucher J.A. Monterrosa & R.A. Carballo 213 tRNA-Leu (trnL) gene and trnL-trnF intergenic spacer, partial sequence; chloroplast	1018	1018	93%	0.0	95%	JF301361.1	1039,89	83,6%
Select seq gb|KF307435.1|	Lepechinia yecorana voucher Henrickson 24691 tRNA-Leu (trnL) gene and trnL-trnF intergenic spacer, partial sequence; chloroplast	1007	1007	92%	0.0	95%	KF307435.1	1039,84	83,6%
Select seq gb|KF307432.1|	Lepechinia speciosa voucher Cordeno 3060 tRNA-Leu (trnL) gene and trnL-trnF intergenic spacer, partial sequence; chloroplast	1007	1007	92%	0.0	95%	KF307432.1	1039,84	83,6%
Select seq gb|KF307421.1|	Lepechinia heteromorpha voucher B. Drew 192 tRNA-Leu (trnL) gene and trnL-trnF intergenic spacer, partial sequence; chloroplast	1007	1007	92%	0.0	95%	KF307421.1	1039,84	83,6%
Select seq gb|KF307420.1|	Lepechinia graveolens voucher Fuentes 10351 tRNA-Leu (trnL) gene and trnL-trnF intergenic spacer, partial sequence; chloroplast	1007	1007	92%	0.0	95%	KF307420.1	1039,84	83,6%
Select seq gb|KF307418.1|	Lepechinia floribunda voucher B. Drew 172 tRNA-Leu (trnL) gene and trnL-trnF intergenic spacer, partial sequence; chloroplast	1007	1007	92%	0.0	95%	KF307418.1	1039,84	83,6%
Select seq gb|JF301381.1|	Lepechinia mexicana voucher B. Drew 164 tRNA-Leu (trnL) gene and trnL-trnF intergenic spacer, partial sequence; chloroplast	1005	1005	92%	0.0	95%	JF301381.1	1037,77	83,4%
Select seq gb|JF301380.1|	Lepechinia mexicana voucher B. Drew 127 tRNA-Leu (trnL) gene and trnL-trnF intergenic spacer, partial sequence; chloroplast	1013	1013	93%	0.0	95%	JF301380.1	1034,78	83,2%
Select seq gb|GU381495.1|	Killickia pilosa voucher M:Braeuchler 3810 tRNA-Leu (trnL) gene and trnL-trnF intergenic spacer, partial sequence; chloroplast	1013	1013	93%	0.0	95%	GU381495.1	1034,78	83,2%

Select seq gb|KF307422.1|	Lepechinia mexicana voucher B. Drew 130 tRNA-Leu (trnL) gene and trnL-trnF intergenic spacer, partial sequence; chloroplast	1002	1002	92% 0.0	95% KF307422.1	1034,67	83,2%
Select seq emb|AJ505432.1|	Nepeta racemosa plastid trnL-trnF intergenic spacer, specimen voucher Jamzad s.n. (TARI)	1055	1055	97% 0.0	95% AJ505432.1	1033,25	83,1%
Select seq gb|GU381479.1|	Nepeta supina voucher M:Groeger & Schewardnadse 1466 tRNA-Leu (trnL) gene and trnL-trnF intergenic spacer, partial sequence; chloroplast	1033	1033	95% 0.0	95% GU381479.1	1033,00	83,1%
Select seq gb|KF307416.1|	Lepechinia codon voucher B. Drew 177 tRNA-Leu (trnL) gene and trnL-trnF intergenic spacer, partial sequence; chloroplast	1000	1000	92% 0.0	95% KF307416.1	1032,61	83,0%
Select seq gb|AY570459.1|	Lepechinia chamaedryoides voucher JBW 2537 tRNA-Leu and trnL-trnF intergenic spacer, partial sequence; chloroplast	1083	1083	100% 0.0	95% AY570459.1	1028,85	82,7%
Select seq emb|AJ505433.1|	Nepeta straussii plastid trnL-trnF intergenic spacer, specimen voucher Jamzad etal 76846 (TARI)	1038	1038	96% 0.0	95% AJ505433.1	1027,19	82,6%
Select seq gb|GU381496.1|	Killickia pilosa voucher M:Braeuchler 3832 tRNA-Leu (trnL) gene and trnL-trnF intergenic spacer, partial sequence; chloroplast	1027	1027	95% 0.0	95% GU381496.1	1027,00	82,6%
Select seq gb|GU381493.1|	Killickia grandiflora voucher M:Braeuchler 3811 tRNA-Leu (trnL) gene and trnL-trnF intergenic spacer, partial sequence; chloroplast	1027	1027	95% 0.0	95% GU381493.1	1027,00	82,6%
Select seq gb|KF307419.1|	Lepechinia ganderi voucher B. Drew 24 tRNA-Leu (trnL) gene and trnL-trnF intergenic spacer, partial sequence; chloroplast	990	990	92% 0.0	95% KF307419.1	1022,28	82,2%
Select seq gb|DQ667494.1|	Lepechinia calycina isolate x246 tRNA-Leu (trnL) gene and trnL-trnF intergenic spacer, partial sequence; chloroplast	1031	1031	96% 0.0	95% DQ667494.1	1020,26	82,0%
Select seq gb|AY570458.1|	Lepechinia calycina voucher JBW 1344 tRNA-Leu and trnL-trnF intergenic spacer, partial sequence; chloroplast	1027	1027	96% 0.0	95% AY570458.1	1016,30	81,7%
Select seq gb|AY570460.1|	Lepechinia fragrans voucher JBW 1333 tRNA-Leu and trnL-trnF intergenic spacer, partial sequence; chloroplast	1016	1016	95% 0.0	95% AY570460.1	1016,00	81,7%
Select seq gb|KR150215.1|	Thymus sp. 1 Kh08 trnL-trnF intergenic spacer region, partial sequence; chloroplast	994	994	93% 0.0	95% KR150215.1	1015,38	81,6%
Select seq gb|GU381484.1|	Micromeria flagellaris voucher E:van der Werff & McPherson 13570 tRNA-Leu (trnL) gene and trnL-trnF intergenic spacer, partial sequence; chloroplast	1014	1014	95% 0.0	95% GU381484.1	1014,00	81,5%
Select seq gb|GU381483.1|	Micromeria flagellaris voucher E:Clement et al 2140 tRNA-Leu (trnL) gene and trnL-trnF intergenic spacer, partial sequence; chloroplast	1014	1014	95% 0.0	95% GU381483.1	1014,00	81,5%
Select seq gb|JF301376.1|	Lepechinia caulescens voucher B. Drew 106 tRNA-Leu (trnL) gene and trnL-trnF intergenic spacer, partial sequence; chloroplast	990	990	93% 0.0	95% JF301376.1	1011,29	81,3%
Select seq gb|JF301375.1|	Lepechinia calycina voucher B. Drew 197 tRNA-Leu (trnL) gene and trnL-trnF intergenic spacer, partial sequence; chloroplast	990	990	93% 0.0	95% JF301375.1	1011,29	81,3%
Select seq gb|JF301360.1|	Cedronella canariensis voucher UCBG 2004.0788 tRNA-Leu (trnL) gene and trnL-trnF intergenic spacer, partial sequence; chloroplast	989	989	93% 0.0	95% JF301360.1	1010,27	81,2%
Select seq gb|GU381629.1|	Thymbra capitata voucher M:Braeuchler 2518 tRNA-Leu (trnL) gene and trnL-trnF intergenic spacer, partial sequence; chloroplast	1009	1009	95% 0.0	95% GU381629.1	1009,00	81,1%
Select seq gb|AY840207.1|	Thymbra spicata tRNA-Leu (trnL) gene and trnL-trnF intergenic spacer, partial sequence; chloroplast	1009	1009	95% 0.0	95% AY840207.1	1009,00	81,1%
Select seq gb|KR150191.1|	Nepeta sp. Kh55 trnL-trnF intergenic spacer region, partial sequence; chloroplast	996	996	93% 0.0	94% KR150191.1	1006,71	80,9%
Select seq gb|GU381489.1|	Killickia lutea voucher NU<ZAF>:Hilliard & Burtt 9876 tRNA-Leu (trnL) gene and trnL-trnF intergenic spacer, partial sequence; chloroplast	1005	1005	95% 0.0	95% GU381489.1	1005,00	80,8%
Select seq gb|GU381488.1|	Killickia compacta voucher M:Braeuchler 3816 tRNA-Leu (trnL) gene and trnL-trnF intergenic spacer, partial sequence; chloroplast	1005	1005	95% 0.0	95% GU381488.1	1005,00	80,8%
Select seq gb|KR150243.1|	Ziziphora sp. 2 Kh20 trnL-trnF intergenic spacer region, partial sequence; chloroplast	1029	1029	97% 0.0	94% KR150243.1	997,18	80,2%
Select seq gb|GU381632.1|	Thymbra spicata voucher M:Braeuchler 4548 tRNA-Leu (trnL) gene and trnL-trnF intergenic spacer, partial sequence; chloroplast	1005	1005	95% 0.0	94% GU381632.1	994,42	79,9%
Select seq gb|EU244612.1|	Agastache rugosa tRNA-Leu (trnL) gene and trnL-trnF intergenic spacer, partial sequence	1046	1046	99% 0.0	94% EU244612.1	993,17	79,8%
Select seq gb|KR150238.1|	Ziziphora sp. 1 Kh75 trnL-trnF intergenic spacer region, partial sequence; chloroplast	1014	1014	96% 0.0	94% KR150238.1	992,88	79,8%
Select seq gb|GU381627.1|	Thymbra sintenisii subsp. isaurica voucher E:Goener 12628 tRNA-Leu (trnL) gene and trnL-trnF intergenic spacer, partial sequence; chloroplast	1003	1003	95% 0.0	94% GU381627.1	992,44	79,8%

Select seq gb|KR150198.1|	Ziziphora sp. 3 Kh112 trnL-trnF intergenic spacer region, partial sequence; chloroplast	1000	1000	95%	0.0	94%	KR150198.1	989,47	79,6%
Select seq gb|GU381517.1|	Clinopodium barosmum voucher BM<GBR-LONDON>:McLaren N193 tRNA-Leu (trnL) gene and trnL-trnF intergenic spacer, partial sequence; chloroplast	998	998	95%	0.0	94%	GU381517.1	987,49	79,4%
Select seq gb|GU381516.1|	Clinopodium wardii voucher BM<GBR-LONDON>:Ludlow et al. 14234 tRNA-Leu (trnL) gene and trnL-trnF intergenic spacer, partial sequence; chloroplast	998	998	95%	0.0	94%	GU381516.1	987,49	79,4%
Select seq gb|GU381515.1|	Clinopodium hydaspidis voucher BM<GBR-LONDON>:Mohd 133 tRNA-Leu (trnL) gene and trnL-trnF intergenic spacer, partial sequence; chloroplast	998	998	95%	0.0	94%	GU381515.1	987,49	79,4%
Select seq gb|GU381514.1|	Clinopodium nepalense voucher FR:Stainton 6024 tRNA-Leu (trnL) gene and trnL-trnF intergenic spacer, partial sequence; chloroplast	998	998	95%	0.0	94%	GU381514.1	987,49	79,4%
Select seq gb|GU381511.1|	Clinopodium piperitum voucher BM<GBR-LONDON>:Vickery 454 tRNA-Leu (trnL) gene and trnL-trnF intergenic spacer, partial sequence; chloroplast	998	998	95%	0.0	94%	GU381511.1	987,49	79,4%
Select seq gb|JX880022.1|	Origanum vulgare subsp. vulgare chloroplast, complete genome	1048	1048	100%	0.0	94%	JX880022.1	985,12	79,2%
Select seq gb|DQ667501.1|	Ziziphora taurica isolate x262 tRNA-Leu (trnL) gene and trnL-trnF intergenic spacer, partial sequence; chloroplast	994	994	95%	0.0	94%	DQ667501.1	983,54	79,1%
Select seq gb|KR063656.1|	Satureja pilosa subsp. origanita tRNA-Leu (trnL) gene, partial sequence; trnL-trnF intergenic spacer, complete sequence; and tRNA-Phe (trnF) gene, partial sequence; chloroplast	1035	1035	99%	0.0	94%	KR063656.1	982,73	79,0%
Select seq gb|DQ667493.1|	Lepechinia conferta isolate x234 tRNA-Leu (trnL) gene and trnL-trnF intergenic spacer, partial sequence; chloroplast	1014	1014	97%	0.0	94%	DQ667493.1	982,64	79,0%
Select seq emb|AJ505543.1|	Origanum vulgare plastid trnL-trnF intergenic spacer, specimen voucher cult., K-000-69-19317, chase 13334 (K)	1014	1014	97%	0.0	94%	AJ505543.1	982,64	79,0%
Select seq gb|KR150218.1|	Thymus sp. 2 Kh69 trnL-trnF intergenic spacer region, partial sequence; chloroplast	1024	1024	98%	0.0	94%	KR150218.1	982,20	79,0%
Select seq gb|JQ690290.1|	Origanum elongatum isolate H5_O_elo trnL-trnF intergenic spacer, partial sequence; chloroplast	1024	1024	98%	0.0	94%	JQ690290.1	982,20	79,0%
Select seq gb|JQ690289.1|	Origanum rotundifolium isolate DNA3_O_rot trnL-trnF intergenic spacer, partial sequence; chloroplast	1024	1024	98%	0.0	94%	JQ690289.1	982,20	79,0%
Select seq gb|GU381512.1|	Clinopodium piperitum voucher E:Stainton 7320 tRNA-Leu (trnL) gene and trnL-trnF intergenic spacer, partial sequence; chloroplast	992	992	95%	0.0	94%	GU381512.1	981,56	78,9%
Select seq gb|GU381510.1|	Ziziphora pamiroalaica voucher C:Murray et al. 10090 tRNA-Leu (trnL) gene and trnL-trnF intergenic spacer, partial sequence; chloroplast	992	992	95%	0.0	94%	GU381510.1	981,56	78,9%
Select seq gb|GU381505.1|	Clinopodium troodi voucher W:Davis 1856 tRNA-Leu (trnL) gene and trnL-trnF intergenic spacer, partial sequence; chloroplast	992	992	95%	0.0	94%	GU381505.1	981,56	78,9%
Select seq gb|GU381501.1|	Clinopodium graveolens subsp. rotundifolium voucher M:Podlech 47181 tRNA-Leu (trnL) gene and trnL-trnF intergenic spacer, partial sequence; chloroplast	992	992	95%	0.0	94%	GU381501.1	981,56	78,9%
Select seq gb|GU381499.1|	Clinopodium suaveolens voucher M:Erben s.n. tRNA-Leu (trnL) gene and trnL-trnF intergenic spacer, partial sequence; chloroplast	992	992	95%	0.0	94%	GU381499.1	981,56	78,9%
Select seq gb|AY570452.1|	Agastache urticifolia voucher JBW 815 tRNA-Leu and trnL-trnF intergenic spacer, partial sequence; chloroplast	1044	1044	100%	0.0	94%	AY570452.1	981,36	78,9%
Select seq gb|EU556532.1|	Thymus amurensis isolate HX tRNA-Leu (trnL) gene and trnL-trnF intergenic spacer, partial sequence; chloroplast	1033	1033	99%	0.0	94%	EU556532.1	980,83	78,9%
Select seq gb|GU381485.1|	Micromeria sphaerophylla voucher E:Lewis et al 1064 tRNA-Leu (trnL) gene and trnL-trnF intergenic spacer, partial sequence; chloroplast	990	990	95%	0.0	94%	GU381485.1	979,58	78,8%
Select seq gb|KR063657.1|	Thymus sibthorpii tRNA-Leu (trnL) gene, partial sequence; trnL-trnF intergenic spacer, complete sequence; and tRNA-Phe (trnF) gene, partial sequence; chloroplast	1037	1037	100%	0.0	94%	KR063657.1	974,78	78,4%
Select seq gb|KR150229.1|	Thymus sp. 5 Kh16 trnL-trnF intergenic spacer region, partial sequence; chloroplast	1026	1026	99%	0.0	94%	KR150229.1	974,18	78,3%
Select seq gb|KR150222.1|	Thymus sp. 3 Kh65 trnL-trnF intergenic spacer region, partial sequence; chloroplast	994	994	96%	0.0	94%	KR150222.1	973,29	78,3%
Select seq gb|DQ667495.1|	Cleonia lusitanica isolate x255 tRNA-Leu (trnL) gene and trnL-trnF intergenic spacer, partial sequence; chloroplast	1024	1024	99%	0.0	94%	DQ667495.1	972,28	78,2%
Select seq gb|DQ667488.1|	Lycopus uniflorus isolate x221 tRNA-Leu (trnL) gene and trnL-trnF intergenic spacer, partial sequence; chloroplast	1024	1024	99%	0.0	94%	DQ667488.1	972,28	78,2%
Select seq gb|JQ690293.1|	Origanum dayi isolate H43_O_day trnL-trnF intergenic spacer, partial sequence; chloroplast	1013	1013	98%	0.0	94%	JQ690293.1	971,65	78,1%

Select seq gb|KR150225.1|	Thymus sp. 4 Kh24 trnL-trnF intergenic spacer region, partial sequence; chloroplast	992	992	96%	0.0	94% KR150225.1	971,33	78,1%
Select seq gb|EU556525.1|	Thymus quinquecostatus isolate CL tRNA-Leu (trnL) gene and trnL-trnF intergenic spacer, partial sequence; chloroplast	1031	1031	100%	0.0	94% EU556525.1	969,14	77,9%
Select seq gb|JF301384.1|	Lophanthus lipskyanus voucher Vassiljeva s.n. tRNA-Leu (trnL) gene and trnL-trnF intergenic spacer, partial sequence; chloroplast	979	979	93%	0.0	92% JF301384.1	968,47	77,9%
Select seq gb|EU556539.1|	Thymus quinquecostatus isolate ZY tRNA-Leu (trnL) gene and trnL-trnF intergenic spacer, partial sequence; chloroplast	1029	1029	100%	0.0	94% EU556539.1	967,26	77,8%
Select seq gb|EU556538.1|	Thymus quinquecostatus isolate ZJ tRNA-Leu (trnL) gene and trnL-trnF intergenic spacer, partial sequence; chloroplast	1029	1029	100%	0.0	94% EU556538.1	967,26	77,8%
Select seq gb|EU556533.1|	Thymus quinquecostatus isolate HY tRNA-Leu (trnL) gene and trnL-trnF intergenic spacer, partial sequence; chloroplast	1029	1029	100%	0.0	94% EU556533.1	967,26	77,8%
Select seq gb|EU556531.1|	Thymus amurensis isolate HW tRNA-Leu (trnL) gene and trnL-trnF intergenic spacer, partial sequence; chloroplast	1029	1029	100%	0.0	94% EU556531.1	967,26	77,8%
Select seq gb|EU556527.1|	Thymus mongolicus isolate G2 tRNA-Leu (trnL) gene and trnL-trnF intergenic spacer, partial sequence; chloroplast	1029	1029	100%	0.0	94% EU556527.1	967,26	77,8%
Select seq gb|EU556526.1|	Thymus quinquecostatus isolate D2 tRNA-Leu (trnL) gene and trnL-trnF intergenic spacer, partial sequence; chloroplast	1029	1029	100%	0.0	94% EU556526.1	967,26	77,8%
Select seq gb|DQ667508.1|	Prunella vulgaris isolate x314 tRNA-Leu (trnL) gene and trnL-trnF intergenic spacer, partial sequence; chloroplast	1016	1016	99%	0.0	93% DQ667508.1	954,42	76,7%
Select seq gb|KC414276.1|	Mentha canadensis isolate 511190001 tRNA-Leu (trnL) gene and trnL-trnF intergenic spacer, partial sequence; chloroplast	1014	1014	100%	0.0	93% KC414276.1	943,02	75,8%
Select seq gb|EU556537.1|	Thymus quinquecostatus isolate XZ tRNA-Leu (trnL) gene and trnL-trnF intergenic spacer, partial sequence; chloroplast	996	996	99%	0.0	93% EU556537.1	935,64	75,2%
Select seq gb|AY570485.1|	Salvia lyrata voucher JBW 2554 tRNA-Leu and trnL-trnF intergenic spacer, partial sequence; chloroplast	1005	1005	100%	0.0	93% AY570485.1	934,65	75,1%
Select seq gb|FJ593456.1|	Mentha sp. JSZ-2009a tRNA-Leu (trnL) gene and trnL-trnF intergenic spacer, partial sequence; chloroplast	1002	1002	100%	0.0	93% FJ593456.1	931,86	74,9%
Select seq gb|KR232566.1|	Rosmarinus officinalis chloroplast, complete genome	992	992	100%	0.0	93% KR232566.1	922,56	74,2%
							0,00	0,0%
							0,00	0,0%

Select for downloading or viewing reports	Kh068_ITS Description	Max score	Total score	Query cover	E value	Ident	Accession	(Ident/Cover)* Max score	Deviation from top hit
Select seq gb|EF679716.1|	Alcea arbelensis voucher PE624 internal transcribed spacer 1, partial sequence; 5.8S ribosomal RNA gene, complete sequence; and internal transcribed spacer 2, partial sequence	1245	1245	100%	0.0	99%	EF679716.1	1232,55	100,0%
Select seq gb|EF679725.1|	Alcea rechingeri voucher PE621 internal transcribed spacer 1, partial sequence; 5.8S ribosomal RNA gene, complete sequence; and internal transcribed spacer 2, partial sequence	1242	1242	100%	0.0	99%	EF679725.1	1229,58	99,8%
Select seq gb|GU166768.1|	Alcea rufescens isolate Iruf01 internal transcribed spacer 1, partial sequence; 5.8S ribosomal RNA gene, complete sequence; and internal transcribed spacer 2, partial sequence	1240	1240	100%	0.0	99%	GU166768.1	1227,60	99,6%
Select seq gb|EF679726.1|	Alcea sachsachanica voucher PE620 internal transcribed spacer 1, partial sequence; 5.8S ribosomal RNA gene, complete sequence; and internal transcribed spacer 2, partial sequence	1240	1240	100%	0.0	99%	EF679726.1	1227,60	99,6%
Select seq gb|EF679722.1|	Alcea koelzii voucher PE609 internal transcribed spacer 1, partial sequence; 5.8S ribosomal RNA gene, complete sequence; and internal transcribed spacer 2, partial sequence	1240	1240	100%	0.0	99%	EF679722.1	1227,60	99,6%
Select seq gb|EF679718.1|	Alcea excubita voucher PE417 internal transcribed spacer 1, partial sequence; 5.8S ribosomal RNA gene, complete sequence; and internal transcribed spacer 2, partial sequence	1240	1240	100%	0.0	99%	EF679718.1	1227,60	99,6%
Select seq gb|EF679714.1|	Alcea rosea voucher PE422 internal transcribed spacer 1, partial sequence; 5.8S ribosomal RNA gene, complete sequence; and internal transcribed spacer 2, partial sequence	1240	1240	100%	0.0	99%	EF679714.1	1227,60	99,6%
Select seq gb|GU166758.1|	Alcea glabrata isolate Igla01 internal transcribed spacer 1, partial sequence; 5.8S ribosomal RNA gene, complete sequence; and internal transcribed spacer 2, partial sequence	1236	1236	100%	0.0	99%	GU166758.1	1223,64	99,3%
Select seq gb|EF679728.1|	Alcea schirazana voucher PE618 internal transcribed spacer 1, partial sequence; 5.8S ribosomal RNA gene, complete sequence; and internal transcribed spacer 2, partial sequence	1236	1236	100%	0.0	99%	EF679728.1	1223,64	99,3%
Select seq gb|EF679727.1|	Alcea setosa voucher PE424 internal transcribed spacer 1, partial sequence; 5.8S ribosomal RNA gene, complete sequence; and internal transcribed spacer 2, partial sequence	1236	1236	100%	0.0	99%	EF679727.1	1223,64	99,3%
Select seq gb|EF419544.1|	Alcea rosea isolate Iros422 internal transcribed spacer 1, partial sequence; 5.8S ribosomal RNA gene, complete sequence; and internal transcribed spacer 2, partial sequence	1236	1236	100%	0.0	99%	EF419544.1	1223,64	99,3%
Select seq gb|GU166763.1|	Alcea hohenackeri isolate Ihoh02 internal transcribed spacer 1, partial sequence; 5.8S ribosomal RNA gene, complete sequence; and internal transcribed spacer 2, partial sequence	1234	1234	100%	0.0	99%	GU166763.1	1221,66	99,1%
Select seq gb|EF679721.1|	Alcea glabrata voucher PE607 internal transcribed spacer 1, partial sequence; 5.8S ribosomal RNA gene, complete sequence; and internal transcribed spacer 2, partial sequence	1234	1234	100%	0.0	99%	EF679721.1	1221,66	99,1%
Select seq gb|GU166765.1|	Alcea lavateriflora isolate Ilav02 internal transcribed spacer 1, partial sequence; 5.8S ribosomal RNA gene, complete sequence; and internal transcribed spacer 2, partial sequence	1230	1230	100%	0.0	99%	GU166765.1	1217,70	98,8%
Select seq gb|GU166759.1|	Alcea glabrata isolate Igla03 internal transcribed spacer 1, partial sequence; 5.8S ribosomal RNA gene, complete sequence; and internal transcribed spacer 2, partial sequence	1230	1230	100%	0.0	99%	GU166759.1	1217,70	98,8%
Select seq gb|GU166756.1|	Alcea apterocarpa isolate Iapt02 internal transcribed spacer 1, partial sequence; 5.8S ribosomal RNA gene, complete sequence; and internal transcribed spacer 2, partial sequence	1230	1230	100%	0.0	99%	GU166756.1	1217,70	98,8%
Select seq gb|GU166754.1|	Alcea angulata isolate Iang01 internal transcribed spacer 1, partial sequence; 5.8S ribosomal RNA gene, complete sequence; and internal transcribed spacer 2, partial sequence	1230	1230	100%	0.0	99%	GU166754.1	1217,70	98,8%
Select seq gb|JX017319.1|	Althaea rosea voucher 01 internal transcribed spacer 1, partial sequence; 5.8S ribosomal RNA and internal transcribed spacer 2, complete sequence; and 26S ribosomal RNA, partial sequence	1229	1229	100%	0.0	99%	JX017319.1	1216,71	98,7%
Select seq gb|GU166769.1|	Alcea striata isolate Istr01 internal transcribed spacer 1, partial sequence; 5.8S ribosomal RNA gene, complete sequence; and internal transcribed spacer 2, partial sequence	1229	1229	100%	0.0	99%	GU166769.1	1216,71	98,7%
Select seq gb|GU166764.1|	Alcea lavateriflora isolate Ilav01 internal transcribed spacer 1, partial sequence; 5.8S ribosomal RNA gene, complete sequence; and internal transcribed spacer 2, partial sequence	1229	1229	100%	0.0	99%	GU166764.1	1216,71	98,7%
Select seq gb|GU166755.1|	Alcea apterocarpa isolate Iapt01 internal transcribed spacer 1, partial sequence; 5.8S ribosomal RNA gene, complete sequence; and internal transcribed spacer 2, partial sequence	1229	1229	100%	0.0	99%	GU166755.1	1216,71	98,7%
Select seq gb|EF679720.1|	Alcea gorganica voucher PE611 internal transcribed spacer 1, partial sequence; 5.8S ribosomal RNA gene, complete sequence; and internal transcribed spacer 2, partial sequence	1229	1229	100%	0.0	99%	EF679720.1	1216,71	98,7%
Select seq gb|EF679729.1|	Alcea sulphurea voucher PE610 internal transcribed spacer 1, partial sequence; 5.8S ribosomal RNA gene, complete sequence; and internal transcribed spacer 2, partial sequence	1227	1227	100%	0.0	99%	EF679729.1	1214,73	98,6%
Select seq gb|GU166762.1|	Alcea hohenackeri isolate Ihoh01 internal transcribed spacer 1, partial sequence; 5.8S ribosomal RNA gene, complete sequence; and internal transcribed spacer 2, partial sequence	1225	1225	100%	0.0	99%	GU166762.1	1212,75	98,4%
Select seq gb|GU166761.1|	Alcea heldreichii isolate Ihel03 internal transcribed spacer 1, partial sequence; 5.8S ribosomal RNA gene, complete sequence; and internal transcribed spacer 2, partial sequence	1223	1223	100%	0.0	99%	GU166761.1	1210,77	98,2%
Select seq gb|EF679717.1|	Alcea aucheri voucher PE617 internal transcribed spacer 1, partial sequence; 5.8S ribosomal RNA gene, complete sequence; and internal transcribed spacer 2, partial sequence	1221	1221	100%	0.0	99%	EF679717.1	1208,79	98,1%

Select seq gb|EF419543.1|	Alcea angulata isolate Iang615 internal transcribed spacer 1, partial sequence; 5.8S ribosomal RNA gene, complete sequence; and internal transcribed spacer 2, partial sequence	1219	1219	100% 0.0	99% EF419543.1	1206,81	97,9%
Select seq gb|GU166760.1|	Alcea heldreichii isolate Ihel01 internal transcribed spacer 1, partial sequence; 5.8S ribosomal RNA gene, complete sequence; and internal transcribed spacer 2, partial sequence	1218	1218	100% 0.0	99% GU166760.1	1205,82	97,8%
Select seq gb|GU166767.1|	Alcea peduncularis isolate Iped01 internal transcribed spacer 1, partial sequence; 5.8S ribosomal RNA gene, complete sequence; and internal transcribed spacer 2, partial sequence	1212	1212	100% 0.0	99% GU166767.1	1199,88	97,3%
Select seq gb|EF679715.1|	Alcea pallida voucher PE140 internal transcribed spacer 1, partial sequence; 5.8S ribosomal RNA gene, complete sequence; and internal transcribed spacer 2, partial sequence	1210	1210	100% 0.0	99% EF679715.1	1197,90	97,2%
Select seq gb|EF419545.1|	Alcea pallida isolate IAsp0140 internal transcribed spacer 1, partial sequence; 5.8S ribosomal RNA gene, complete sequence; and internal transcribed spacer 2, partial sequence	1210	1210	100% 0.0	99% EF419545.1	1197,90	97,2%
Select seq gb|KR150156.1|	Alcea sp. Kh114 internal transcribed spacer 1, partial sequence; 5.8S ribosomal RNA gene gene, complete sequence; and internal transcribed spacer 2 region, partial sequence	985	985	83% 0.0	98% KR150156.1	1163,01	94,4%
Select seq gb|GU166770.1|	Alcea acaulis isolate Iaca01 internal transcribed spacer 1, partial sequence; 5.8S ribosomal RNA gene, complete sequence; and internal transcribed spacer 2, partial sequence	966	1165	95% 0.0	99% GU166770.1	1006,67	81,7%
Select seq gb|KR150188.1|	Alcea sp. kh59 internal transcribed spacer 1, partial sequence; 5.8S ribosomal RNA gene gene, complete sequence; and internal transcribed spacer 2 region, partial sequence	974	974	95% 0.0	94% KR150188.1	963,75	78,2%
Select seq gb|EF419542.1|	Althaea armeniaca isolate IAar427 internal transcribed spacer 1, partial sequence; 5.8S ribosomal RNA gene, complete sequence; and internal transcribed spacer 2, partial sequence	1009	1009	100% 0.0	93% EF419542.1	938,37	76,1%
Select seq gb|EF679734.1|	Althaea armeniaca voucher PE427 internal transcribed spacer 1, partial sequence; 5.8S ribosomal RNA gene, complete sequence; and internal transcribed spacer 2, partial sequence	1005	1005	100% 0.0	93% EF679734.1	934,65	75,8%
Select seq gb|EF419539.1|	Althaea cannabina isolate IAca594 internal transcribed spacer 1, partial sequence; 5.8S ribosomal RNA gene, complete sequence; and internal transcribed spacer 2, partial sequence	994	994	100% 0.0	93% EF419539.1	924,42	75,0%
Select seq gb|EF679733.1|	Althaea officinalis voucher PE512 internal transcribed spacer 1, partial sequence; 5.8S ribosomal RNA gene, complete sequence; and internal transcribed spacer 2, partial sequence	990	990	100% 0.0	93% EF679733.1	920,70	74,7%
Select seq gb|EF419536.1|	Althaea officinalis isolate IAof604 internal transcribed spacer 1, partial sequence; 5.8S ribosomal RNA gene, complete sequence; and internal transcribed spacer 2, partial sequence	990	990	100% 0.0	93% EF419536.1	920,70	74,7%
Select seq gb|EF419540.1|	Althaea cannabina isolate IAca0345 internal transcribed spacer 1, partial sequence; 5.8S ribosomal RNA gene, complete sequence; and internal transcribed spacer 2, partial sequence	989	989	100% 0.0	93% EF419540.1	919,77	74,6%
Select seq gb|EF419537.1|	Althaea officinalis isolate IAof0330 internal transcribed spacer 1, partial sequence; 5.8S ribosomal RNA gene, complete sequence; and internal transcribed spacer 2, partial sequence	983	983	100% 0.0	93% EF419537.1	914,19	74,2%
Select seq gb|AF303026.1|	Althaea officinalis internal transcribed spacer 1, partial sequence; 5.8S ribosomal RNA gene, complete sequence; and internal transcribed spacer 2, partial sequence	983	983	100% 0.0	93% AF303026.1	914,19	74,2%
Select seq gb|EF419547.1|	Anisodontea malvastroides internal transcribed spacer 1, partial sequence; 5.8S ribosomal RNA gene, complete sequence; and internal transcribed spacer 2, partial sequence	913	913	99% 0.0	91% EF419547.1	839,22	68,1%
Select seq gb|AY172229.1|	Andeimalva spiciformis internal transcribed spacer 1, 5.8S ribosomal RNA gene, and internal transcribed spacer 2, complete sequence	907	907	99% 0.0	91% AY172229.1	833,71	67,6%
Select seq gb|AY172220.1|	Andeimalva mandonii internal transcribed spacer 1, 5.8S ribosomal RNA gene, and internal transcribed spacer 2, complete sequence	898	898	99% 0.0	91% AY172220.1	825,43	67,0%
Select seq gb|AY172219.1|	Andeimalva machupicchensis internal transcribed spacer 1, 5.8S ribosomal RNA gene, and internal transcribed spacer 2, complete sequence	898	898	99% 0.0	91% AY172219.1	825,43	67,0%
Select seq gb|AY591834.1|	Kearnemalvastrum sp. Ventura A. 816 internal transcribed spacer 1, 5.8S ribosomal RNA gene, and internal transcribed spacer 2, complete sequence	867	867	96% 0.0	90% AY591834.1	812,81	65,9%
Select seq gb|AY172190.1|	Modiola caroliniana internal transcribed spacer 1, 5.8S ribosomal RNA gene, and internal transcribed spacer 2, complete sequence	894	894	99% 0.0	90% AY172190.1	812,73	65,9%
Select seq gb|AY591833.1|	Kearnemalvastrum lacteum internal transcribed spacer 1, 5.8S ribosomal RNA gene, and internal transcribed spacer 2, complete sequence	891	891	99% 0.0	90% AY591833.1	810,00	65,7%
Select seq gb|AY172191.1|	Modiolastrum lateritium internal transcribed spacer 1, 5.8S ribosomal RNA gene, and internal transcribed spacer 2, complete sequence	883	883	99% 0.0	90% AY172191.1	802,73	65,1%
Select seq gb|AY591820.1|	Calyculogygas uruguayensis internal transcribed spacer 1, 5.8S ribosomal RNA gene, and internal transcribed spacer 2, complete sequence	869	869	99% 0.0	90% AY591820.1	790,00	64,1%
Select seq gb|HQ632292.1|	Palaua dissecta voucher Schneider (et al.) 2978 (FR) clone 2978e internal transcribed spacer 1, partial sequence; 5.8S ribosomal RNA gene, complete sequence; and internal transcribed spacer 2, partial sequence	863	863	99% 0.0	90% HQ632292.1	784,55	63,7%
Select seq gb|AY591842.1|	Malvastrum americanum internal transcribed spacer 1, 5.8S ribosomal RNA gene, and internal transcribed spacer 2, complete sequence	852	852	99% 0.0	90% AY591842.1	774,55	62,8%
Select seq gb|HQ632303.1|	Palaua sp. Schneider et al. 2777 voucher Schneider (et al.) 2777 (FR) clone 2777j internal transcribed spacer 1, partial sequence; 5.8S ribosomal RNA gene, complete sequence; and internal transcribed spacer 2, partial sequence	857	857	99% 0.0	89% HQ632303.1	770,43	62,5%

Select seq gb|DQ156306.1|	Palaua pusilla voucher Schneider & Huertas 2978 internal transcribed spacer 1, 5.8S ribosomal RNA gene, and internal transcribed spacer 2, complete sequence	857	857	99% 0.0	89% DQ156306.1	770,43	62,5%
Select seq gb|AY172192.1|	Sphaeralcea angustifolia internal transcribed spacer 1, 5.8S ribosomal RNA gene, and internal transcribed spacer 2, complete sequence	857	857	99% 0.0	89% AY172192.1	770,43	62,5%
Select seq emb|AJ512142.1|	Sphaeralcea ambigua 5.8S rRNA gene, internal transcribed spacer 1 (ITS1) and internal transcribed spacer 2 (ITS2), specimen voucher Andreasen 291 (UC)	856	856	99% 0.0	89% AJ512142.1	769,54	62,4%
Select seq gb|AY172199.1|	Andeimalva chilensis internal transcribed spacer 1, 5.8S ribosomal RNA gene, and internal transcribed spacer 2, complete sequence	854	854	99% 0.0	89% AY172199.1	767,74	62,3%
Select seq gb|HQ632358.1|	Palaua dissecta voucher Schneider (et al.) 2888 (FR) clone 2888g internal transcribed spacer 1, partial sequence; 5.8S ribosomal RNA gene, complete sequence; and internal transcribed spacer 2, partial sequence	852	852	99% 0.0	89% HQ632358.1	765,94	62,1%
Select seq gb|HQ632306.1|	Palaua dissecta voucher Schneider (et al.) 2771 (FR) clone 2771e internal transcribed spacer 1, partial sequence; 5.8S ribosomal RNA gene, complete sequence; and internal transcribed spacer 2, partial sequence	852	852	99% 0.0	89% HQ632306.1	765,94	62,1%
Select seq gb|HQ632296.1|	Palaua dissecta voucher Schneider (et al.) 2807 (FR) internal transcribed spacer 1, partial sequence; 5.8S ribosomal RNA gene, complete sequence; and internal transcribed spacer 2, partial sequence	852	852	99% 0.0	89% HQ632296.1	765,94	62,1%
Select seq gb|HQ632286.1|	Palaua sp. Schneider et al. 2777 voucher Schneider (et al.) 2777 (FR) clone 2777b internal transcribed spacer 1, partial sequence; 5.8S ribosomal RNA gene, complete sequence; and internal transcribed spacer 2, partial sequence	852	852	99% 0.0	89% HQ632286.1	765,94	62,1%
Select seq gb|HQ632285.1|	Palaua dissecta voucher Schneider (et al.) 2978 (FR) clone 2978d internal transcribed spacer 1, partial sequence; 5.8S ribosomal RNA gene, complete sequence; and internal transcribed spacer 2, partial sequence	852	852	99% 0.0	89% HQ632285.1	765,94	62,1%
Select seq gb|HQ632284.1|	Palaua dissecta voucher Schneider (et al.) 2978 (FR) clone 2978b internal transcribed spacer 1, partial sequence; 5.8S ribosomal RNA gene, complete sequence; and internal transcribed spacer 2, partial sequence	852	852	99% 0.0	89% HQ632284.1	765,94	62,1%
Select seq gb|HQ632107.1|	Palaua dissecta voucher Schneider (et al.) 2836 (FR) clone 2836b internal transcribed spacer 1, partial sequence; 5.8S ribosomal RNA gene, complete sequence; and internal transcribed spacer 2, partial sequence	852	852	99% 0.0	89% HQ632107.1	765,94	62,1%
Select seq gb|DQ156313.1|	Palaua modesta voucher Schneider & Huertas 2969 internal transcribed spacer 1, 5.8S ribosomal RNA gene, and internal transcribed spacer 2, complete sequence	852	852	99% 0.0	89% DQ156313.1	765,94	62,1%
Select seq gb|DQ156311.1|	Palaua inconspicua voucher Schneider & Huertas 3016 internal transcribed spacer 1, 5.8S ribosomal RNA gene, and internal transcribed spacer 2, complete sequence	852	852	99% 0.0	89% DQ156311.1	765,94	62,1%
Select seq gb|DQ156300.1|	Palaua malvifolia voucher Schneider & Huertas 2753 internal transcribed spacer 1, 5.8S ribosomal RNA gene, and internal transcribed spacer 2, complete sequence	852	852	99% 0.0	89% DQ156300.1	765,94	62,1%
Select seq gb|AY591810.1|	Acaulimalva dryadifolia internal transcribed spacer 1, 5.8S ribosomal RNA gene, and internal transcribed spacer 2, complete sequence	852	852	99% 0.0	89% AY591810.1	765,94	62,1%
Select seq gb|AY591809.1|	Acaulimalva alismatifolia internal transcribed spacer 1, 5.8S ribosomal RNA gene, and internal transcribed spacer 2, complete sequence	852	852	99% 0.0	89% AY591809.1	765,94	62,1%
Select seq emb|AJ304940.1|	Napaea dioica 5.8S rRNA gene, internal transcribed spacer 1 (ITS1) and internal transcribed spacer 2 (ITS2)	852	852	99% 0.0	89% AJ304940.1	765,94	62,1%
Select seq gb|HQ632355.1|	Palaua mollendoensis voucher Schneider (et al.) 2813 (FR) clone 2813d internal transcribed spacer 1, partial sequence; 5.8S ribosomal RNA gene, complete sequence; and internal transcribed spacer 2, partial sequence	848	848	99% 0.0	89% HQ632355.1	762,34	61,9%
Select seq gb|AY172200.1|	Tarasa albertii internal transcribed spacer 1, 5.8S ribosomal RNA gene, and internal transcribed spacer 2, complete sequence	848	848	99% 0.0	89% AY172200.1	762,34	61,9%
Select seq gb|HQ632351.1|	Palaua dissecta voucher Schneider (et al.) 2888 (FR) clone 2888a internal transcribed spacer 1, partial sequence; 5.8S ribosomal RNA gene, complete sequence; and internal transcribed spacer 2, partial sequence	846	846	99% 0.0	89% HQ632351.1	760,55	61,7%
Select seq gb|HQ632343.1|	Palaua dissecta voucher Schneider (et al.) 2836 (FR) clone 2836d internal transcribed spacer 1, partial sequence; 5.8S ribosomal RNA gene, complete sequence; and internal transcribed spacer 2, partial sequence	846	846	99% 0.0	89% HQ632343.1	760,55	61,7%
Select seq gb|HQ632332.1|	Palaua dissecta voucher Schneider (et al.) 2771 (FR) clone 2771k internal transcribed spacer 1, partial sequence; 5.8S ribosomal RNA gene, complete sequence; and internal transcribed spacer 2, partial sequence	846	846	99% 0.0	89% HQ632332.1	760,55	61,7%
Select seq gb|HQ632324.1|	Palaua mollendoensis voucher Schneider (et al.) 2985 (FR) clone 2985a internal transcribed spacer 1, partial sequence; 5.8S ribosomal RNA gene, complete sequence; and internal transcribed spacer 2, partial sequence	846	846	99% 0.0	89% HQ632324.1	760,55	61,7%
Select seq gb|HQ632308.1|	Palaua dissecta x Palaua tomentosa voucher Schneider (et al.) 2997 (FR) clone 2997e internal transcribed spacer 1, partial sequence; 5.8S ribosomal RNA gene, complete sequence; and internal transcribed spacer 2, partial sequence	846	846	99% 0.0	89% HQ632308.1	760,55	61,7%
Select seq gb|HQ632318.1|	Palaua mollendoensis voucher Schneider (et al.) 2813 (FR) clone 2813b internal transcribed spacer 1, partial sequence; 5.8S ribosomal RNA gene, complete sequence; and internal transcribed spacer 2, partial sequence	846	846	99% 0.0	89% HQ632318.1	760,55	61,7%
Select seq gb|HQ632302.1|	Palaua sp. Schneider et al. 2777 voucher Schneider (et al.) 2777 (FR) clone 2777e internal transcribed spacer 1, partial sequence; 5.8S ribosomal RNA gene, complete sequence; and internal transcribed spacer 2, partial sequence	846	846	99% 0.0	89% HQ632302.1	760,55	61,7%
Select seq gb|HQ632269.1|	Palaua cf. dissecta Schneider et al. 2802 voucher Schneider (et al.) 2802 (FR) clone 2802a internal transcribed spacer 1, partial sequence; 5.8S ribosomal RNA gene, complete sequence; and internal transcribed spacer 2, partial sequence	846	846	99% 0.0	89% HQ632269.1	760,55	61,7%
Select seq gb|HQ632263.1|	Palaua inconspicua voucher Schneider (et al.) 3016 (FR) clone 3016a internal transcribed spacer 1, partial sequence; 5.8S ribosomal RNA gene, complete sequence; and internal transcribed spacer 2, partial sequence	846	846	99% 0.0	89% HQ632263.1	760,55	61,7%

Select seq gb|HQ632180.1|	Palaua cf. tomentosa Schneider et al. 2984 voucher Schneider (et al.) 2984 (FR) clone 2984k internal transcribed spacer 1, partial sequence; 5.8S ribosomal RNA gene, complete sequence; and internal transcribed spacer 2, partial sequence	846	846	99% 0.0	89%	HQ632180.1	760,55	61,7%
Select seq gb|HQ632175.1|	Palaua tomentosa voucher Schneider (et al.) 3091 (FR) clone 3091d internal transcribed spacer 1, partial sequence; 5.8S ribosomal RNA gene, complete sequence; and internal transcribed spacer 2, partial sequence	846	846	99% 0.0	89%	HQ632175.1	760,55	61,7%
Select seq gb|HQ632145.1|	Palaua cf. dissecta Schneider et al. 2802 voucher Schneider (et al.) 2802 (FR) clone 2802e internal transcribed spacer 1, partial sequence; 5.8S ribosomal RNA gene, complete sequence; and internal transcribed spacer 2, partial sequence	846	846	99% 0.0	89%	HQ632145.1	760,55	61,7%
Select seq gb|HQ632122.1|	Palaua dissecta voucher Schneider (et al.) 3093 (FR) clone 3093e internal transcribed spacer 1, partial sequence; 5.8S ribosomal RNA gene, complete sequence; and internal transcribed spacer 2, partial sequence	846	846	99% 0.0	89%	HQ632122.1	760,55	61,7%
Select seq gb|HQ632113.1|	Palaua dissecta x Palaua tomentosa voucher Schneider (et al.) 3002 (FR) clone 3002a internal transcribed spacer 1, partial sequence; 5.8S ribosomal RNA gene, complete sequence; and internal transcribed spacer 2, partial sequence	846	846	99% 0.0	89%	HQ632113.1	760,55	61,7%
Select seq gb|HQ632105.1|	Palaua dissecta voucher Schneider (et al.) 2715 (FR) clone 2715k internal transcribed spacer 1, partial sequence; 5.8S ribosomal RNA gene, complete sequence; and internal transcribed spacer 2, partial sequence	846	846	99% 0.0	89%	HQ632105.1	760,55	61,7%
Select seq gb|EU784126.1|	Palaua dissecta voucher Schneider 3093 internal transcribed spacer 1, 5.8S ribosomal RNA gene, and internal transcribed spacer 2, complete sequence	846	846	99% 0.0	89%	EU784126.1	760,55	61,7%
Select seq gb|EU784123.1|	Palaua dissecta voucher Schneider & Huertas 2888 internal transcribed spacer 1, 5.8S ribosomal RNA gene, and internal transcribed spacer 2, complete sequence	846	846	99% 0.0	89%	EU784123.1	760,55	61,7%
Select seq gb|DQ156316.1|	Palaua sandemanii voucher Schneider & Huertas 2980 internal transcribed spacer 1, 5.8S ribosomal RNA gene, and internal transcribed spacer 2, complete sequence	846	846	99% 0.0	89%	DQ156316.1	760,55	61,7%
Select seq gb|DQ156308.1|	Palaua dissecta voucher Schneider & Huertas 2777 internal transcribed spacer 1, 5.8S ribosomal RNA gene, and internal transcribed spacer 2, complete sequence	846	846	99% 0.0	89%	DQ156308.1	760,55	61,7%
Select seq gb|AY591839.1|	Malacothamnus fasciculatus internal transcribed spacer 1, 5.8S ribosomal RNA gene, and internal transcribed spacer 2, complete sequence	846	846	99% 0.0	89%	AY591839.1	760,55	61,7%
Select seq gb|DQ156307.1|	Palaua velutina voucher Schneider & Huertas 2749 internal transcribed spacer 1, 5.8S ribosomal RNA gene, and internal transcribed spacer 2, complete sequence	845	845	99% 0.0	89%	DQ156307.1	759,65	61,6%
Select seq gb|AY591843.1|	Monteiroa glomerata internal transcribed spacer 1, 5.8S ribosomal RNA gene, and internal transcribed spacer 2, complete sequence	845	845	99% 0.0	89%	AY591843.1	759,65	61,6%
Select seq gb|HQ632313.1|	Palaua mollendoensis voucher Schneider (et al.) 2813 (FR) clone 2813e internal transcribed spacer 1, partial sequence; 5.8S ribosomal RNA gene, complete sequence; and internal transcribed spacer 2, partial sequence	843	843	99% 0.0	89%	HQ632313.1	757,85	61,5%
Select seq gb|HQ632369.1|	Palaua weberbaueri voucher Schneider (et al.) 3017 (FR) clone 3017j internal transcribed spacer 1, partial sequence; 5.8S ribosomal RNA gene, complete sequence; and internal transcribed spacer 2, partial sequence	841	841	99% 0.0	89%	HQ632369.1	756,05	61,3%
Select seq gb|HQ632367.1|	Palaua weberbaueri voucher Schneider (et al.) 3017 (FR) clone 3017c internal transcribed spacer 1, partial sequence; 5.8S ribosomal RNA gene, complete sequence; and internal transcribed spacer 2, partial sequence	841	841	99% 0.0	89%	HQ632367.1	756,05	61,3%
Select seq gb|DQ826561.1|	Palaua mollendoensis voucher Schneider & Huertas 2985 internal transcribed spacer 1, 5.8S ribosomal RNA gene, and internal transcribed spacer 2, complete sequence	839	839	99% 0.0	89%	DQ826561.1	754,25	61,2%

Select for downloading or viewing reports	Kh069_ITS Description	Max score	Total score	Query cover	E value	Ident	Accession	(Ident/Cover) *Max score	Deviation from top hit
Select seq gb|EU374715.1|	Thymus pubescens 18S ribosomal RNA gene, partial sequence; internal transcribed spacer 1, 5.8S ribosomal RNA gene, and internal transcribed spacer 2, complete sequence; and 25/28S ribosomal RNA gene, partial sequence	1142	1142	97%	0.0	100%	EU374715.1	1177,32	100,0%
Select seq gb|EU735059.1|	Thymus trautvetteri 18S ribosomal RNA gene, partial sequence; internal transcribed spacer 1, 5.8S ribosomal RNA gene, and internal transcribed spacer 2, complete sequence; and 28S ribosomal RNA gene, partial sequence	1168	1168	100%	0.0	100%	EU735059.1	1168,00	99,2%
Select seq gb|EU735058.1|	Thymus persicus isolate Ardabil 18S ribosomal RNA gene, partial sequence; internal transcribed spacer 1, 5.8S ribosomal RNA gene, and internal transcribed spacer 2, complete sequence; and 28S ribosomal RNA gene, partial sequence	1168	1168	100%	0.0	100%	EU735058.1	1168,00	99,2%
Select seq gb|GU381459.1|	Thymus serpyllum voucher M:Braeuchler 2514 internal transcribed spacer 1, partial sequence; 5.8S ribosomal RNA gene, complete sequence; and internal transcribed spacer 2, partial sequence	1110	1110	96%	0.0	99%	GU381459.1	1144,69	97,2%
Select seq gb|EU556508.1|	Thymus mongolicus isolate G1 internal transcribed spacer 1, partial sequence; 5.8S ribosomal RNA gene, complete sequence; and internal transcribed spacer 2, partial sequence	1120	1120	97%	0.0	99%	EU556508.1	1143,09	97,1%
Select seq gb|AY443448.1|	Thymus magnus JUNG-TM04 internal transcribed spacer 1, 5.8S ribosomal RNA and internal transcribed spcer 2 genes, complete sequence	1131	1131	98%	0.0	99%	AY443448.1	1142,54	97,0%
Select seq gb|AY443434.1|	Thymus quinquecostatus JUNG-TQ01 internal transcribed spacer 1, 5.8S ribosomal RNA and internal transcribed spcer 2 genes, complete sequence	1131	1131	98%	0.0	99%	AY443434.1	1142,54	97,0%
Select seq gb|EU556518.1|	Thymus mongolicus isolate S1 internal transcribed spacer 1, partial sequence; 5.8S ribosomal RNA gene, complete sequence; and internal transcribed spacer 2, partial sequence	1142	1142	99%	0.0	99%	EU556518.1	1142,00	97,0%
Select seq gb|EU556521.1|	Thymus mongolicus isolate XM internal transcribed spacer 1, partial sequence; 5.8S ribosomal RNA gene, complete sequence; and internal transcribed spacer 2, partial sequence	1083	1083	94%	0.0	99%	EU556521.1	1140,61	96,9%
Select seq gb|EU556519.1|	Thymus mongolicus isolate S2 internal transcribed spacer 1, partial sequence; 5.8S ribosomal RNA gene, complete sequence; and internal transcribed spacer 2, partial sequence	1138	1138	99%	0.0	99%	EU556519.1	1138,00	96,7%
Select seq gb|AY443445.1|	Thymus magnus JUNG-TM01 internal transcribed spacer 1, 5.8S ribosomal RNA and internal transcribed spcer 2 genes, complete sequence	1125	1125	98%	0.0	99%	AY443445.1	1136,48	96,5%
Select seq gb|EU556509.1|	Thymus mongolicus isolate G2 internal transcribed spacer 1, partial sequence; 5.8S ribosomal RNA gene, complete sequence; and internal transcribed spacer 2, partial sequence	1136	1136	99%	0.0	99%	EU556509.1	1136,00	96,5%
Select seq gb|EU556524.1|	Thymus quinquecostatus isolate ZY internal transcribed spacer 1, partial sequence; 5.8S ribosomal RNA gene, complete sequence; and internal transcribed spacer 2, partial sequence	1146	1146	100%	0.0	99%	EU556524.1	1134,54	96,4%
Select seq gb|EU556522.1|	Thymus quinquecostatus isolate ZJ internal transcribed spacer 1, partial sequence; 5.8S ribosomal RNA gene, complete sequence; and internal transcribed spacer 2, partial sequence	1146	1146	100%	0.0	99%	EU556522.1	1134,54	96,4%
Select seq gb|EU556520.1|	Thymus quinquecostatus isolate XZ internal transcribed spacer 1, partial sequence; 5.8S ribosomal RNA gene, complete sequence; and internal transcribed spacer 2, partial sequence	1146	1146	100%	0.0	99%	EU556520.1	1134,54	96,4%
Select seq gb|EU556517.1|	Thymus quinquecostatus isolate N2 internal transcribed spacer 1, partial sequence; 5.8S ribosomal RNA gene, complete sequence; and internal transcribed spacer 2, partial sequence	1146	1146	100%	0.0	99%	EU556517.1	1134,54	96,4%
Select seq gb|EU556516.1|	Thymus quinquecostatus isolate N1 internal transcribed spacer 1, partial sequence; 5.8S ribosomal RNA gene, complete sequence; and internal transcribed spacer 2, partial sequence	1146	1146	100%	0.0	99%	EU556516.1	1134,54	96,4%
Select seq gb|EU556511.1|	Thymus dahuricus isolate HD internal transcribed spacer 1, partial sequence; 5.8S ribosomal RNA gene, complete sequence; and internal transcribed spacer 2, partial sequence	1146	1146	100%	0.0	99%	EU556511.1	1134,54	96,4%
Select seq gb|EU556506.1|	Thymus quinquecostatus isolate D1 internal transcribed spacer 1, partial sequence; 5.8S ribosomal RNA gene, complete sequence; and internal transcribed spacer 2, partial sequence	1146	1146	100%	0.0	99%	EU556506.1	1134,54	96,4%
Select seq gb|FJ236468.1|	Thymus persicus isolate Tabriz 18S ribosomal RNA gene, partial sequence; internal transcribed spacer 1, 5.8S ribosomal RNA gene, and internal transcribed spacer 2, complete sequence; and 28S ribosomal RNA gene, partial sequence	1146	1146	100%	0.0	99%	FJ236468.1	1134,54	96,4%
Select seq gb|DQ667242.1|	Thymus serpyllum isolate x075 18S ribosomal RNA gene, partial sequence; internal transcribed spacer 1, 5.8S ribosomal RNA gene, and internal transcribed spacer 2, complete sequence; and 28S ribosomal RNA gene, partial sequence	1146	1146	100%	0.0	99%	DQ667242.1	1134,54	96,4%
Select seq gb|AY029168.1|	Thymus mastichina internal transcribed spacer 1, 5.8S ribosomal RNA gene, and internal transcribed spacer 2, complete sequence	1123	1123	98%	0.0	99%	AY029168.1	1134,46	96,4%
Select seq gb|EU556510.1|	Thymus mongolicus isolate G3 internal transcribed spacer 1, partial sequence; 5.8S ribosomal RNA gene, complete sequence; and internal transcribed spacer 2, partial sequence	1088	1088	95%	0.0	99%	EU556510.1	1133,81	96,3%
Select seq gb|AY443447.1|	Thymus magnus JUNG-TM03 internal transcribed spacer 1, 5.8S ribosomal RNA and internal transcribed spcer 2 genes, complete sequence	1120	1120	98%	0.0	99%	AY443447.1	1131,43	96,1%
Select seq gb|AY443438.1|	Thymus quinquecostatus JUNG-TQ05 internal transcribed spacer 1, 5.8S ribosomal RNA and internal transcribed spcer 2 genes, complete sequence	1120	1120	98%	0.0	99%	AY443438.1	1131,43	96,1%
Select seq gb|EU556507.1|	Thymus quinquecostatus isolate CL internal transcribed spacer 1, partial sequence; 5.8S ribosomal RNA gene, complete sequence; and internal transcribed spacer 2, partial sequence	1140	1140	100%	0.0	99%	EU556507.1	1128,60	95,9%

Select seq gb|AY443444.1|	Thymus quinquecostatus JUNG-TQ11 internal transcribed spacer 1, 5.8S ribosomal RNA and internal transcribed spcer 2 genes, complete sequence	1114	1114	98%	0.0	99%	AY443444.1	1125,37	95,6%
Select seq gb|AY443443.1|	Thymus quinquecostatus JUNG-TQ10 internal transcribed spacer 1, 5.8S ribosomal RNA and internal transcribed spcer 2 genes, complete sequence	1114	1114	98%	0.0	99%	AY443443.1	1125,37	95,6%
Select seq gb|AY443442.1|	Thymus quinquecostatus JUNG-TQ09 internal transcribed spacer 1, 5.8S ribosomal RNA and internal transcribed spcer 2 genes, complete sequence	1114	1114	98%	0.0	99%	AY443442.1	1125,37	95,6%
Select seq gb|AY443441.1|	Thymus quinquecostatus JUNG-TQ08 internal transcribed spacer 1, 5.8S ribosomal RNA and internal transcribed spcer 2 genes, complete sequence	1114	1114	98%	0.0	99%	AY443441.1	1125,37	95,6%
Select seq gb|AY443437.1|	Thymus quinquecostatus JUNG-TQ04 internal transcribed spacer 1, 5.8S ribosomal RNA and internal transcribed spcer 2 genes, complete sequence	1114	1114	98%	0.0	99%	AY443437.1	1125,37	95,6%
Select seq gb|AY443436.1|	Thymus quinquecostatus JUNG-TQ03 internal transcribed spacer 1, 5.8S ribosomal RNA and internal transcribed spcer 2 genes, complete sequence	1114	1114	98%	0.0	99%	AY443436.1	1125,37	95,6%
Select seq gb|JQ669138.1|	Thymus pulegioides voucher Riina 1577 18S ribosomal RNA gene, internal transcribed spacer 1, 5.8S ribosomal RNA gene, internal transcribed spacer 2, and 26S ribosomal RNA gene, region	1136	1136	100%	0.0	99%	JQ669138.1	1124,64	95,5%
Select seq gb|EU796890.1|	Thymus serpyllum 18S ribosomal RNA gene, partial sequence; internal transcribed spacer 1, 5.8S ribosomal RNA gene, and internal transcribed spacer 2, complete sequence; and 26S ribosomal RNA gene, partial sequence	1134	1134	100%	0.0	99%	EU796890.1	1122,66	95,4%
Select seq gb|GU381458.1|	Thymus broussonetii subsp. hannonis voucher MSB:Podlech 142 internal transcribed spacer 1, partial sequence; 5.8S ribosomal RNA gene, complete sequence; and internal transcribed spacer 2, partial sequence	1086	1086	96%	0.0	99%	GU381458.1	1119,94	95,1%
Select seq gb|EU785939.1|	Thymus vulgaris 18S ribosomal RNA gene, partial sequence; internal transcribed spacer 1, 5.8S ribosomal RNA gene, and internal transcribed spacer 2, complete sequence; and 26S ribosomal RNA gene, partial sequence	1131	1131	100%	0.0	99%	EU785939.1	1119,69	95,1%
Select seq gb|AY443446.1|	Thymus magnus JUNG-TM02 internal transcribed spacer 1, 5.8S ribosomal RNA and internal transcribed spcer 2 genes, complete sequence	1103	1103	98%	0.0	99%	AY443446.1	1114,26	94,6%
Select seq gb|AY443440.1|	Thymus quinquecostatus JUNG-TQ07 internal transcribed spacer 1, 5.8S ribosomal RNA and internal transcribed spcer 2 genes, complete sequence	1103	1103	98%	0.0	99%	AY443440.1	1114,26	94,6%
Select seq gb|EU556523.1|	Thymus marschallianus isolate XY internal transcribed spacer 1, partial sequence; 5.8S ribosomal RNA gene, complete sequence; and internal transcribed spacer 2, partial sequence	1123	1123	100%	0.0	99%	EU556523.1	1111,77	94,4%
Select seq gb|AY443435.1|	Thymus quinquecostatus JUNG-TQ02 internal transcribed spacer 1, 5.8S ribosomal RNA and internal transcribed spcer 2 genes, complete sequence	1092	1092	98%	0.0	98%	AY443435.1	1092,00	92,8%
Select seq gb|EU556512.1|	Thymus mandschuricus isolate HM internal transcribed spacer 1, partial sequence; 5.8S ribosomal RNA gene, complete sequence; and internal transcribed spacer 2, partial sequence	1081	1081	100%	0.0	98%	EU556512.1	1059,38	90,0%
Select seq gb|AY329369.1|	Thymus vulgaris internal transcribed spacer 1, partial sequence; 5.8S ribosomal RNA gene, complete sequence; and internal transcribed spacer 2, partial sequence	1072	1072	100%	0.0	97%	AY329369.1	1039,84	88,3%
Select seq gb|AY506646.1|	Thymus vulgaris internal transcribed spacer 1, partial sequence; 5.8S ribosomal RNA gene, complete sequence; and internal transcribed spacer 2, partial sequence	1068	1068	100%	0.0	97%	AY506646.1	1035,96	88,0%
Select seq gb|EU556515.1|	Thymus amurensis isolate HW internal transcribed spacer 1, partial sequence; 5.8S ribosomal RNA gene, complete sequence; and internal transcribed spacer 2, partial sequence	1064	1064	100%	0.0	97%	EU556515.1	1032,08	87,7%
Select seq gb|EU556514.1|	Thymus quinquecostatus isolate HY internal transcribed spacer 1, partial sequence; 5.8S ribosomal RNA gene, complete sequence; and internal transcribed spacer 2, partial sequence	946	946	91%	0.0	97%	EU556514.1	1008,37	85,6%
Select seq gb|EU556513.1|	Thymus przewalskii isolate HX internal transcribed spacer 1, partial sequence; 5.8S ribosomal RNA gene, complete sequence; and internal transcribed spacer 2, partial sequence	952	952	92%	0.0	96%	EU556513.1	993,39	84,4%
Select seq gb|GU381460.1|	Thymus saturejoides subsp. commutatus voucher M:Podlech 47906 internal transcribed spacer 1, partial sequence; 5.8S ribosomal RNA gene, complete sequence; and internal transcribed spacer 2, partial sequence	965	965	96%	0.0	95%	GU381460.1	954,95	81,1%
Select seq gb|GU381462.1|	Saccocalyx saturejoides voucher MSB:Faurel 5650 internal transcribed spacer 1, partial sequence; 5.8S ribosomal RNA gene, complete sequence; and internal transcribed spacer 2, partial sequence	953	953	96%	0.0	95%	GU381462.1	943,07	80,1%
Select seq gb|GU381457.1|	Thymus caespititius voucher M:Heubl s.n. internal transcribed spacer 1, partial sequence; 5.8S ribosomal RNA gene, complete sequence; and internal transcribed spacer 2, partial sequence	953	953	96%	0.0	95%	GU381457.1	943,07	80,1%
Select seq gb|GU381461.1|	Argantoniella salzmannii voucher M:Barra et al. 2673GL internal transcribed spacer 1, partial sequence; 5.8S ribosomal RNA gene, complete sequence; and internal transcribed spacer 2, partial sequence	948	948	96%	0.0	95%	GU381461.1	938,13	79,7%
Select seq gb|GU381451.1|	Thymbra sintenisii subsp. isaurica voucher E:Goener 12628 internal transcribed spacer 1, partial sequence; 5.8S ribosomal RNA gene, complete sequence; and internal transcribed spacer 2, partial sequence	915	915	96%	0.0	94%	GU381451.1	895,94	76,1%
Select seq gb|GU381452.1|	Thymbra calostachya voucher M:Ulrich s.n. internal transcribed spacer 1, partial sequence; 5.8S ribosomal RNA gene, complete sequence; and internal transcribed spacer 2, partial sequence	909	909	96%	0.0	94%	GU381452.1	890,06	75,6%
Select seq gb|GU381455.1|	Satureja linearifolia voucher M:Brullo & Furnari s.n. internal transcribed spacer 1, partial sequence; 5.8S ribosomal RNA gene, complete sequence; and internal transcribed spacer 2, partial sequence	907	907	96%	0.0	94%	GU381455.1	888,10	75,4%
Select seq gb|JQ669137.1|	Thymbra capitata voucher UCBG 96.0817 s.n. 18S ribosomal RNA gene, internal transcribed spacer 1, 5.8S ribosomal RNA gene, internal transcribed spacer 2, and 26S ribosomal RNA gene, region	935	935	100%	0.0	93%	JQ669137.1	869,55	73,9%

Select seq gb|GU381453.1|	Thymbra capitata voucher M:Braeuchler 2518 internal transcribed spacer 1, partial sequence; 5.8S ribosomal RNA gene, complete sequence; and internal transcribed spacer 2, partial sequence	896	896	96%	0.0	93%	GU381453.1	868,00	73,7%
Select seq gb|GU381454.1|	Satureja thymbrifolia voucher M:Danin s.n. internal transcribed spacer 1, partial sequence; 5.8S ribosomal RNA gene, complete sequence; and internal transcribed spacer 2, partial sequence	891	891	96%	0.0	93%	GU381454.1	863,16	73,3%
Select seq gb|JQ669127.1|	Origanum vulgare voucher B. Drew 77 18S ribosomal RNA gene, internal transcribed spacer 1, 5.8S ribosomal RNA gene, internal transcribed spacer 2, and 26S ribosomal RNA gene, region	905	905	100%	0.0	93%	JQ669127.1	841,65	71,5%
Select seq gb|JX162834.1|	Origanum majorana voucher OBI clone 3 internal transcribed spacer 1, partial sequence; 5.8S ribosomal RNA gene, complete sequence; and internal transcribed spacer 2, partial sequence	894	894	100%	0.0	92%	JX162834.1	822,48	69,9%
Select seq gb|JX162831.1|	Origanum majorana voucher Mira2-3 clone 10 internal transcribed spacer 1, partial sequence; 5.8S ribosomal RNA gene, complete sequence; and internal transcribed spacer 2, partial sequence	894	894	100%	0.0	92%	JX162831.1	822,48	69,9%
Select seq gb|AY506647.1|	Origanum vulgare internal transcribed spacer 1, partial sequence; 5.8S ribosomal RNA gene, complete sequence; and internal transcribed spacer 2, partial sequence	893	893	100%	0.0	92%	AY506647.1	821,56	69,8%
Select seq gb|JX163036.1|	Origanum syriacum voucher LC7 clone 8 internal transcribed spacer 1, partial sequence; 5.8S ribosomal RNA gene, complete sequence; and internal transcribed spacer 2, partial sequence	891	891	100%	0.0	92%	JX163036.1	819,72	69,6%
Select seq gb|JX162836.1|	Origanum majorana voucher OBI clone 5 internal transcribed spacer 1, partial sequence; 5.8S ribosomal RNA gene, complete sequence; and internal transcribed spacer 2, partial sequence	891	891	100%	0.0	92%	JX162836.1	819,72	69,6%
Select seq gb|DQ667243.1|	Origanum vulgare isolate x076 18S ribosomal RNA gene, partial sequence; internal transcribed spacer 1, 5.8S ribosomal RNA gene, and internal transcribed spacer 2, complete sequence; and 28S ribosomal RNA gene, partial sequence	891	891	100%	0.0	92%	DQ667243.1	819,72	69,6%
Select seq gb|JX162980.1|	Origanum onites voucher SR777 clone 14 internal transcribed spacer 1, partial sequence; 5.8S ribosomal RNA gene, complete sequence; and internal transcribed spacer 2, partial sequence	889	889	100%	0.0	92%	JX162980.1	817,88	69,5%
Select seq gb|JX162837.1|	Origanum majorana voucher OBI clone 6 internal transcribed spacer 1, partial sequence; 5.8S ribosomal RNA gene, complete sequence; and internal transcribed spacer 2, partial sequence	889	889	100%	0.0	92%	JX162837.1	817,88	69,5%
Select seq gb|JX162826.1|	Origanum majorana voucher Mira2-3 clone 4 internal transcribed spacer 1, partial sequence; 5.8S ribosomal RNA gene, complete sequence; and internal transcribed spacer 2, partial sequence	889	889	100%	0.0	92%	JX162826.1	817,88	69,5%
Select seq gb|JX162814.1|	Origanum majorana voucher SR562 clone 1 internal transcribed spacer 1, partial sequence; 5.8S ribosomal RNA gene, complete sequence; and internal transcribed spacer 2, partial sequence	889	889	100%	0.0	92%	JX162814.1	817,88	69,5%
Select seq gb|JX162807.1|	Origanum majorana voucher LC10 clone 4 internal transcribed spacer 1, partial sequence; 5.8S ribosomal RNA gene, complete sequence; and internal transcribed spacer 2, partial sequence	889	889	100%	0.0	92%	JX162807.1	817,88	69,5%
Select seq gb|JX162805.1|	Origanum majorana voucher LC10 clone 2 internal transcribed spacer 1, partial sequence; 5.8S ribosomal RNA gene, complete sequence; and internal transcribed spacer 2, partial sequence	889	889	100%	0.0	92%	JX162805.1	817,88	69,5%
Select seq gb|JX162830.1|	Origanum majorana voucher Mira2-3 clone 9 internal transcribed spacer 1, partial sequence; 5.8S ribosomal RNA gene, complete sequence; and internal transcribed spacer 2, partial sequence	885	885	100%	0.0	92%	JX162830.1	814,20	69,2%
Select seq gb|JX163016.1|	Origanum syriacum voucher RNG:H50 clone 8 internal transcribed spacer 1, partial sequence; 5.8S ribosomal RNA gene, complete sequence; and internal transcribed spacer 2, partial sequence	883	883	100%	0.0	92%	JX163016.1	812,36	69,0%
Select seq gb|JX163004.1|	Origanum onites voucher SR1372 clone 8 internal transcribed spacer 1, partial sequence; 5.8S ribosomal RNA gene, complete sequence; and internal transcribed spacer 2, partial sequence	883	883	100%	0.0	92%	JX163004.1	812,36	69,0%
Select seq gb|JX162978.1|	Origanum onites voucher SR777 clone 11 internal transcribed spacer 1, partial sequence; 5.8S ribosomal RNA gene, complete sequence; and internal transcribed spacer 2, partial sequence	883	883	100%	0.0	92%	JX162978.1	812,36	69,0%
Select seq gb|JX162966.1|	Origanum onites voucher SR530 clone 8 internal transcribed spacer 1, partial sequence; 5.8S ribosomal RNA gene, complete sequence; and internal transcribed spacer 2, partial sequence	883	883	100%	0.0	92%	JX162966.1	812,36	69,0%
Select seq gb|JX162832.1|	Origanum majorana voucher OBI clone 1 internal transcribed spacer 1, partial sequence; 5.8S ribosomal RNA gene, complete sequence; and internal transcribed spacer 2, partial sequence	883	883	100%	0.0	92%	JX162832.1	812,36	69,0%
Select seq gb|JX162829.1|	Origanum majorana voucher Mira2-3 clone 8 internal transcribed spacer 1, partial sequence; 5.8S ribosomal RNA gene, complete sequence; and internal transcribed spacer 2, partial sequence	883	883	100%	0.0	92%	JX162829.1	812,36	69,0%
Select seq gb|JX162823.1|	Origanum majorana voucher Mira2-3 clone 1 internal transcribed spacer 1, partial sequence; 5.8S ribosomal RNA gene, complete sequence; and internal transcribed spacer 2, partial sequence	883	883	100%	0.0	92%	JX162823.1	812,36	69,0%
Select seq gb|JX162818.1|	Origanum majorana voucher SR562 clone 5 internal transcribed spacer 1, partial sequence; 5.8S ribosomal RNA gene, complete sequence; and internal transcribed spacer 2, partial sequence	883	883	100%	0.0	92%	JX162818.1	812,36	69,0%
Select seq gb|JX162816.1|	Origanum majorana voucher SR562 clone 3 internal transcribed spacer 1, partial sequence; 5.8S ribosomal RNA gene, complete sequence; and internal transcribed spacer 2, partial sequence	883	883	100%	0.0	92%	JX162816.1	812,36	69,0%
Select seq gb|JX162815.1|	Origanum majorana voucher SR562 clone 2 internal transcribed spacer 1, partial sequence; 5.8S ribosomal RNA gene, complete sequence; and internal transcribed spacer 2, partial sequence	883	883	100%	0.0	92%	JX162815.1	812,36	69,0%
Select seq gb|JX163024.1|	Origanum syriacum voucher RNG:H50 clone 16 internal transcribed spacer 1, partial sequence; 5.8S ribosomal RNA gene, complete sequence; and internal transcribed spacer 2, partial sequence	880	880	100%	0.0	92%	JX163024.1	809,60	68,8%
Select seq gb|JX163022.1|	Origanum syriacum voucher RNG:H50 clone 14 internal transcribed spacer 1, partial sequence; 5.8S ribosomal RNA gene, complete sequence; and internal transcribed spacer 2, partial sequence	880	880	100%	0.0	92%	JX163022.1	809,60	68,8%

Select seq gb|JX163021.1|	Origanum syriacum voucher RNG:H50 clone 13 internal transcribed spacer 1, partial sequence; 5.8S ribosomal RNA gene, complete sequence; and internal transcribed spacer 2, partial sequence	880	880	100% 0.0	92% JX163021.1	809,60	68,8%
Select seq gb|JX163014.1|	Origanum syriacum voucher RNG:H50 clone 6 internal transcribed spacer 1, partial sequence; 5.8S ribosomal RNA gene, complete sequence; and internal transcribed spacer 2, partial sequence	880	880	100% 0.0	92% JX163014.1	809,60	68,8%
Select seq gb|JX163010.1|	Origanum syriacum voucher RNG:H50 clone 2 internal transcribed spacer 1, partial sequence; 5.8S ribosomal RNA gene, complete sequence; and internal transcribed spacer 2, partial sequence	880	880	100% 0.0	92% JX163010.1	809,60	68,8%
Select seq gb|JX163019.1|	Origanum syriacum voucher RNG:H50 clone 11 internal transcribed spacer 1, partial sequence; 5.8S ribosomal RNA gene, complete sequence; and internal transcribed spacer 2, partial sequence	878	878	100% 0.0	92% JX163019.1	807,76	68,6%
Select seq gb|JX163018.1|	Origanum syriacum voucher RNG:H50 clone 10 internal transcribed spacer 1, partial sequence; 5.8S ribosomal RNA gene, complete sequence; and internal transcribed spacer 2, partial sequence	878	878	100% 0.0	92% JX163018.1	807,76	68,6%
Select seq gb|JX163017.1|	Origanum syriacum voucher RNG:H50 clone 9 internal transcribed spacer 1, partial sequence; 5.8S ribosomal RNA gene, complete sequence; and internal transcribed spacer 2, partial sequence	878	878	100% 0.0	92% JX163017.1	807,76	68,6%
Select seq gb|JX163003.1|	Origanum onites voucher SR1372 clone 7 internal transcribed spacer 1, partial sequence; 5.8S ribosomal RNA gene, complete sequence; and internal transcribed spacer 2, partial sequence	878	878	100% 0.0	92% JX163003.1	807,76	68,6%
Select seq gb|JX162994.1|	Origanum onites voucher SR786 clone 13 internal transcribed spacer 1, partial sequence; 5.8S ribosomal RNA gene, complete sequence; and internal transcribed spacer 2, partial sequence	878	878	100% 0.0	92% JX162994.1	807,76	68,6%
Select seq gb|JX162974.1|	Origanum onites voucher SR777 clone 6 internal transcribed spacer 1, partial sequence; 5.8S ribosomal RNA gene, complete sequence; and internal transcribed spacer 2, partial sequence	878	878	100% 0.0	92% JX162974.1	807,76	68,6%
Select seq gb|JX162812.1|	Origanum majorana voucher LC10 clone 9 internal transcribed spacer 1, partial sequence; 5.8S ribosomal RNA gene, complete sequence; and internal transcribed spacer 2, partial sequence	878	878	100% 0.0	92% JX162812.1	807,76	68,6%
Select seq gb|JX162808.1|	Origanum majorana voucher LC10 clone 5 internal transcribed spacer 1, partial sequence; 5.8S ribosomal RNA gene, complete sequence; and internal transcribed spacer 2, partial sequence	878	878	100% 0.0	92% JX162808.1	807,76	68,6%
Select seq gb|JX163025.1|	Origanum syriacum var. sinaicum voucher ANAD 13338 clone 1 internal transcribed spacer 1, partial sequence; 5.8S ribosomal RNA gene, complete sequence; and internal transcribed spacer 2, partial sequence	874	874	100% 0.0	92% JX163025.1	804,08	68,3%
Select seq gb|JX163054.1|	Origanum onites voucher SR810 internal transcribed spacer 1, partial sequence; 5.8S ribosomal RNA gene, complete sequence; and internal transcribed spacer 2, partial sequence	869	869	100% 0.0	91% JX163054.1	790,79	67,2%
						0,00	0,0%
						0,00	0,0%
						0,00	0,0%
						0,00	0,0%

Select for downloading or viewing reports	Kh069_trnL Description	Max score	Total score	Query cover	E value	Ident	Accession	(Ident/Cover)* Max score	Deviation from top hit
Select seq gb|AY506613.1|	Thymus vulgaris tRNA-Leu (trnL) gene and trnL-trnF intergenic spacer, partial sequence; chloroplast	1177	1177	93%	0.0	99%	AY506613.1	1252,94	100,0%
Select seq gb|GU381635.1|	Thymus broussonetii subsp. hannonis voucher MSB:Podlech 142 tRNA-Leu (trnL) gene and trnL-trnF intergenic spacer, partial sequence; chloroplast	1210	1210	96%	0.0	99%	GU381635.1	1247,81	99,6%
Select seq gb|EU556525.1|	Thymus quinquecostatus isolate CL tRNA-Leu (trnL) gene and trnL-trnF intergenic spacer, partial sequence; chloroplast	1247	1247	99%	0.0	99%	EU556525.1	1247,00	99,5%
Select seq gb|EU556536.1|	Thymus marschallianus isolate XY tRNA-Leu (trnL) gene and trnL-trnF intergenic spacer, partial sequence; chloroplast	1195	1195	95%	0.0	99%	EU556536.1	1245,32	99,4%
Select seq gb|GU381638.1|	Thymus haussknechtii voucher M:Nydegger 43838 tRNA-Leu (trnL) gene and trnL-trnF intergenic spacer, partial sequence; chloroplast	1182	1182	94%	0.0	99%	GU381638.1	1244,87	99,4%
Select seq gb|JQ669069.1|	Thymus pulegioides voucher Riina 1577 tRNA-Leu (trnL) gene and trnL-trnF intergenic spacer, partial sequence; plastid	1205	1205	96%	0.0	99%	JQ669069.1	1242,66	99,2%
Select seq gb|GU381637.1|	Thymus serpyllum voucher M:Braeuchler 2514 tRNA-Leu (trnL) gene and trnL-trnF intergenic spacer, partial sequence; chloroplast	1205	1205	96%	0.0	99%	GU381637.1	1242,66	99,2%
Select seq gb|EU556530.1|	Thymus mandschuricus isolate HM tRNA-Leu (trnL) gene and trnL-trnF intergenic spacer, partial sequence; chloroplast	1205	1205	96%	0.0	99%	EU556530.1	1242,66	99,2%
Select seq gb|EU556535.1|	Thymus proximus isolate XN tRNA-Leu (trnL) gene and trnL-trnF intergenic spacer, partial sequence; chloroplast	1166	1166	93%	0.0	99%	EU556535.1	1241,23	99,1%
Select seq gb|KR063657.1|	Thymus sibthorpii tRNA-Leu (trnL) gene, partial sequence; trnL-trnF intergenic spacer, complete sequence; and tRNA-Phe (trnF) gene, partial sequence; chloroplast	1253	1253	100%	0.0	99%	KR063657.1	1240,47	99,0%
Select seq gb|EU556532.1|	Thymus amurensis isolate HX tRNA-Leu (trnL) gene and trnL-trnF intergenic spacer, partial sequence; chloroplast	1253	1253	100%	0.0	99%	EU556532.1	1240,47	99,0%
Select seq gb|GU381634.1|	Thymus pulegioides voucher M:Braeuchler 3129 tRNA-Leu (trnL) gene and trnL-trnF intergenic spacer, partial sequence; chloroplast	1201	1201	96%	0.0	99%	GU381634.1	1238,53	98,9%
Select seq gb|GU381636.1|	Thymus vulgaris voucher M:Braeuchler 3683 tRNA-Leu (trnL) gene and trnL-trnF intergenic spacer, partial sequence; chloroplast	1199	1199	96%	0.0	99%	GU381636.1	1236,47	98,7%
Select seq gb|EU556534.1|	Thymus mongolicus isolate S1 tRNA-Leu (trnL) gene and trnL-trnF intergenic spacer, partial sequence; chloroplast	1160	1160	93%	0.0	99%	EU556534.1	1234,84	98,6%
Select seq gb|EU556539.1|	Thymus quinquecostatus isolate ZY tRNA-Leu (trnL) gene and trnL-trnF intergenic spacer, partial sequence; chloroplast	1245	1245	100%	0.0	99%	EU556539.1	1232,55	98,4%
Select seq gb|EU556538.1|	Thymus quinquecostatus isolate ZJ tRNA-Leu (trnL) gene and trnL-trnF intergenic spacer, partial sequence; chloroplast	1245	1245	100%	0.0	99%	EU556538.1	1232,55	98,4%
Select seq gb|EU556533.1|	Thymus quinquecostatus isolate HY tRNA-Leu (trnL) gene and trnL-trnF intergenic spacer, partial sequence; chloroplast	1245	1245	100%	0.0	99%	EU556533.1	1232,55	98,4%
Select seq gb|EU556527.1|	Thymus mongolicus isolate G2 tRNA-Leu (trnL) gene and trnL-trnF intergenic spacer, partial sequence; chloroplast	1245	1245	100%	0.0	99%	EU556527.1	1232,55	98,4%
Select seq gb|EU556526.1|	Thymus quinquecostatus isolate D2 tRNA-Leu (trnL) gene and trnL-trnF intergenic spacer, partial sequence; chloroplast	1245	1245	100%	0.0	99%	EU556526.1	1232,55	98,4%
Select seq gb|EU556531.1|	Thymus amurensis isolate HW tRNA-Leu (trnL) gene and trnL-trnF intergenic spacer, partial sequence; chloroplast	1240	1240	100%	0.0	99%	EU556531.1	1227,60	98,0%
Select seq gb|EU556529.1|	Thymus dahuricus isolate HD tRNA-Leu (trnL) gene and trnL-trnF intergenic spacer, partial sequence; chloroplast	1190	1190	96%	0.0	99%	EU556529.1	1227,19	97,9%
Select seq gb|GU381633.1|	Thymus caespititius voucher M:Heubl s.n. tRNA-Leu (trnL) gene and trnL-trnF intergenic spacer, partial sequence; chloroplast	1188	1188	96%	0.0	99%	GU381633.1	1225,13	97,8%
Select seq gb|EU556528.1|	Thymus mongolicus isolate G3 tRNA-Leu (trnL) gene and trnL-trnF intergenic spacer, partial sequence; chloroplast	1199	1199	97%	0.0	99%	EU556528.1	1223,72	97,7%
Select seq gb|JQ690290.1|	Origanum elongatum isolate H5_O_elo trnL-trnF intergenic spacer, partial sequence; chloroplast	1219	1219	99%	0.0	99%	JQ690290.1	1219,00	97,3%
Select seq gb|JQ690289.1|	Origanum rotundifolium isolate DNA3_O_rot trnL-trnF intergenic spacer, partial sequence; chloroplast	1219	1219	99%	0.0	99%	JQ690289.1	1219,00	97,3%
Select seq gb|EU556537.1|	Thymus quinquecostatus isolate XZ tRNA-Leu (trnL) gene and trnL-trnF intergenic spacer, partial sequence; chloroplast	1219	1219	99%	0.0	99%	EU556537.1	1219,00	97,3%

Select seq gb|GU381640.1|	Argantoniella salzmannii voucher M:Barra et al. 2673GL tRNA-Leu (trnL) gene and trnL-trnF intergenic spacer, partial sequence; chloroplast	1182	1182	96%	0.0	99%	GU381640.1	1218,94	97,3%
Select seq gb|AY840202.1|	Origanum vulgare tRNA-Leu (trnL) gene and trnL-trnF intergenic spacer, partial sequence; chloroplast	1182	1182	96%	0.0	99%	AY840202.1	1218,94	97,3%
Select seq emb|AJ505544.1|	Thymus serpyllum var. citriodorum plastid trnL-trnF intergenic spacer, specimen voucher cult., K-1975-1177, Chase 13331 (K)	1218	1218	99%	0.0	99%	AJ505544.1	1218,00	97,2%
Select seq gb|AY506614.1|	Origanum vulgare tRNA-Leu (trnL) gene and trnL-trnF intergenic spacer, partial sequence; chloroplast	1142	1142	93%	0.0	99%	AY506614.1	1215,68	97,0%
Select seq gb|JX880022.1|	Origanum vulgare subsp. vulgare chloroplast, complete genome	1225	1225	100%	0.0	99%	JX880022.1	1212,75	96,8%
Select seq gb|AY570463.1|	Origanum vulgare voucher JBW 2567 tRNA-Leu and trnL-trnF intergenic spacer, partial sequence; chloroplast	1175	1175	96%	0.0	99%	AY570463.1	1211,72	96,7%
Select seq gb|JF301392.1|	Origanum vulgare voucher B. Drew 77 tRNA-Leu (trnL) gene and trnL-trnF intergenic spacer, partial sequence; chloroplast	1149	1149	94%	0.0	99%	JF301392.1	1210,12	96,6%
Select seq emb|AJ505543.1|	Origanum vulgare plastid trnL-trnF intergenic spacer, specimen voucher cult., K-000-69-19317, chase 13334 (K)	1210	1210	99%	0.0	99%	AJ505543.1	1210,00	96,6%
Select seq gb|JQ690293.1|	Origanum dayi isolate H43_O_day trnL-trnF intergenic spacer, partial sequence; chloroplast	1208	1208	99%	0.0	99%	JQ690293.1	1208,00	96,4%
Select seq gb|AY570502.1|	Thymus serpyllum voucher JBW 2564 tRNA-Leu and trnL-trnF intergenic spacer, partial sequence; chloroplast	1171	1171	96%	0.0	99%	AY570502.1	1207,59	96,4%
Select seq gb|GU381629.1|	Thymbra capitata voucher M:Braeuchler 2518 tRNA-Leu (trnL) gene and trnL-trnF intergenic spacer, partial sequence; chloroplast	1166	1166	96%	0.0	99%	GU381629.1	1202,44	96,0%
Select seq gb|JF301401.1|	Thymbra capitata voucher UCBG 96.0817 tRNA-Leu (trnL) gene and trnL-trnF intergenic spacer, partial sequence; chloroplast	1140	1140	94%	0.0	99%	JF301401.1	1200,64	95,8%
Select seq gb|JQ669070.1|	Thymus serpyllum voucher J. Walker 2564 tRNA-Leu (trnL) gene and trnL-trnF intergenic spacer, partial sequence; plastid	1164	1164	96%	0.0	99%	JQ669070.1	1200,38	95,8%
Select seq gb|GU381484.1|	Micromeria flagellaris voucher E:van der Werff & McPherson 13570 tRNA-Leu (trnL) gene and trnL-trnF intergenic spacer, partial sequence; chloroplast	1160	1160	96%	0.0	98%	GU381484.1	1184,17	94,5%
Select seq gb|GU381483.1|	Micromeria flagellaris voucher E:Clement et al 2140 tRNA-Leu (trnL) gene and trnL-trnF intergenic spacer, partial sequence; chloroplast	1160	1160	96%	0.0	98%	GU381483.1	1184,17	94,5%
Select seq gb|AY840207.1|	Thymbra spicata tRNA-Leu (trnL) gene and trnL-trnF intergenic spacer, partial sequence; chloroplast	1160	1160	96%	0.0	98%	AY840207.1	1184,17	94,5%
Select seq gb|GU381632.1|	Thymbra spicata voucher M:Braeuchler 4548 tRNA-Leu (trnL) gene and trnL-trnF intergenic spacer, partial sequence; chloroplast	1157	1157	96%	0.0	98%	GU381632.1	1181,10	94,3%
Select seq gb|GU381627.1|	Thymbra sintenisii subsp. isaurica voucher E:Goener 12628 tRNA-Leu (trnL) gene and trnL-trnF intergenic spacer, partial sequence; chloroplast	1155	1155	96%	0.0	98%	GU381627.1	1179,06	94,1%
Select seq gb|GU381517.1|	Clinopodium barosmum voucher BM<GBR-LONDON>:McLaren N193 tRNA-Leu (trnL) gene and trnL-trnF intergenic spacer, partial sequence; chloroplast	1144	1144	96%	0.0	98%	GU381517.1	1167,83	93,2%
Select seq gb|GU381516.1|	Clinopodium wardii voucher BM<GBR-LONDON>:Ludlow et al. 14234 tRNA-Leu (trnL) gene and trnL-trnF intergenic spacer, partial sequence; chloroplast	1144	1144	96%	0.0	98%	GU381516.1	1167,83	93,2%
Select seq gb|GU381515.1|	Clinopodium hydaspidis voucher BM<GBR-LONDON>:Mohd 133 tRNA-Leu (trnL) gene and trnL-trnF intergenic spacer, partial sequence; chloroplast	1144	1144	96%	0.0	98%	GU381515.1	1167,83	93,2%
Select seq gb|GU381514.1|	Clinopodium nepalense voucher FR:Stainton 6024 tRNA-Leu (trnL) gene and trnL-trnF intergenic spacer, partial sequence; chloroplast	1144	1144	96%	0.0	98%	GU381514.1	1167,83	93,2%
Select seq gb|GU381511.1|	Clinopodium piperitum voucher BM<GBR-LONDON>:Vickery 454 tRNA-Leu (trnL) gene and trnL-trnF intergenic spacer, partial sequence; chloroplast	1144	1144	96%	0.0	98%	GU381511.1	1167,83	93,2%
Select seq gb|GU381513.1|	Clinopodium nepalense voucher BM<GBR-LONDON>:Mikage et al. 9550294 tRNA-Leu (trnL) gene and trnL-trnF intergenic spacer, partial sequence; chloroplast	1116	1116	94%	0.0	98%	GU381513.1	1163,49	92,9%
Select seq gb|GU381512.1|	Clinopodium piperitum voucher E:Stainton 7320 tRNA-Leu (trnL) gene and trnL-trnF intergenic spacer, partial sequence; chloroplast	1138	1138	96%	0.0	98%	GU381512.1	1161,71	92,7%
Select seq gb|GU381496.1|	Killickia pilosa voucher M:Braeuchler 3832 tRNA-Leu (trnL) gene and trnL-trnF intergenic spacer, partial sequence; chloroplast	1134	1134	96%	0.0	98%	GU381496.1	1157,63	92,4%
Select seq gb|GU381493.1|	Killickia grandiflora voucher M:Braeuchler 3811 tRNA-Leu (trnL) gene and trnL-trnF intergenic spacer, partial sequence; chloroplast	1134	1134	96%	0.0	98%	GU381493.1	1157,63	92,4%
Select seq gb|GU381631.1|	Satureja linearifolia voucher M:Brullo & Furnari s.n. tRNA-Leu (trnL) gene and trnL-trnF intergenic spacer, partial sequence; chloroplast	1133	1133	96%	0.0	98%	GU381631.1	1156,60	92,3%

Select seq gb|GU381481.1|	Micromeria cf. madagascariensis Morawetz 205 tRNA-Leu (trnL) gene and trnL-trnF intergenic spacer, partial sequence; chloroplast	1133	1133	96%	0.0	98%	GU381481.1	1156,60	92,3%
Select seq gb|GU381495.1|	Killickia pilosa voucher M:Braeuchler 3810 tRNA-Leu (trnL) gene and trnL-trnF intergenic spacer, partial sequence; chloroplast	1120	1120	95%	0.0	98%	GU381495.1	1155,37	92,2%
Select seq gb|GU381485.1|	Micromeria sphaerophylla voucher E:Lewis et al 1064 tRNA-Leu (trnL) gene and trnL-trnF intergenic spacer, partial sequence; chloroplast	1131	1131	96%	0.0	98%	GU381485.1	1154,56	92,1%
Select seq gb|GU381628.1|	Thymbra calostachya voucher M:Ulrich s.n. tRNA-Leu (trnL) gene and trnL-trnF intergenic spacer, partial sequence; chloroplast	1129	1129	96%	0.0	98%	GU381628.1	1152,52	92,0%
Select seq gb|KR150243.1|	Ziziphora sp. 2 Kh20 trnL-trnF intergenic spacer region, partial sequence; chloroplast	1140	1140	97%	0.0	98%	KR150243.1	1151,75	91,9%
Select seq gb|KR150198.1|	Ziziphora sp. 3 Kh112 trnL-trnF intergenic spacer region, partial sequence; chloroplast	1127	1127	96%	0.0	98%	KR150198.1	1150,48	91,8%
Select seq gb|GU381507.1|	Ziziphora tenuior voucher MSB:Fayvush et al. 03-1503 tRNA-Leu (trnL) gene and trnL-trnF intergenic spacer, partial sequence; chloroplast	1127	1127	96%	0.0	98%	GU381507.1	1150,48	91,8%
Select seq gb|GU381489.1|	Killickia lutea voucher NU<ZAF>:Hilliard & Burtt 9876 tRNA-Leu (trnL) gene and trnL-trnF intergenic spacer, partial sequence; chloroplast	1123	1123	96%	0.0	98%	GU381489.1	1146,40	91,5%
Select seq gb|GU381488.1|	Killickia compacta voucher M:Braeuchler 3816 tRNA-Leu (trnL) gene and trnL-trnF intergenic spacer, partial sequence; chloroplast	1123	1123	96%	0.0	98%	GU381488.1	1146,40	91,5%
Select seq gb|DQ667501.1|	Ziziphora taurica isolate x262 tRNA-Leu (trnL) gene and trnL-trnF intergenic spacer, partial sequence; chloroplast	1123	1123	96%	0.0	97%	DQ667501.1	1134,70	90,6%
Select seq gb|GU381510.1|	Ziziphora pamiroalaica voucher C:Murray et al. 10090 tRNA-Leu (trnL) gene and trnL-trnF intergenic spacer, partial sequence; chloroplast	1122	1122	96%	0.0	97%	GU381510.1	1133,69	90,5%
Select seq gb|GU381505.1|	Clinopodium troodi voucher W:Davis 1856 tRNA-Leu (trnL) gene and trnL-trnF intergenic spacer, partial sequence; chloroplast	1122	1122	96%	0.0	97%	GU381505.1	1133,69	90,5%
Select seq gb|GU381501.1|	Clinopodium graveolens subsp. rotundifolium voucher M:Podlech 47181 tRNA-Leu (trnL) gene and trnL-trnF intergenic spacer, partial sequence; chloroplast	1122	1122	96%	0.0	97%	GU381501.1	1133,69	90,5%
Select seq gb|GU381499.1|	Clinopodium suaveolens voucher M:Erben s.n. tRNA-Leu (trnL) gene and trnL-trnF intergenic spacer, partial sequence; chloroplast	1122	1122	96%	0.0	97%	GU381499.1	1133,69	90,5%
Select seq gb|KR150214.1|	Satureja sp. 1 Kh04 trnL-trnF intergenic spacer region, partial sequence; chloroplast	1109	1109	95%	0.0	97%	KR150214.1	1132,35	90,4%
Select seq gb|GU381621.1|	Satureja cuneifolia voucher M:Rechinger 11142 tRNA-Leu (trnL) gene and trnL-trnF intergenic spacer, partial sequence; chloroplast	1118	1118	96%	0.0	97%	GU381621.1	1129,65	90,2%
Select seq gb|GU381612.1|	Gontscharovia popovii voucher BM<GBR-LONDON>:Schmid 2419 tRNA-Leu (trnL) gene and trnL-trnF intergenic spacer, partial sequence; chloroplast	1118	1118	96%	0.0	97%	GU381612.1	1129,65	90,2%
Select seq gb|AY840179.1|	Satureja montana tRNA-Leu (trnL) gene and trnL-trnF intergenic spacer, partial sequence; chloroplast	1118	1118	96%	0.0	97%	AY840179.1	1129,65	90,2%
Select seq gb|GU381497.1|	Clinopodium acinos voucher M:Podlech 50287 tRNA-Leu (trnL) gene and trnL-trnF intergenic spacer, partial sequence; chloroplast	1116	1116	96%	0.0	97%	GU381497.1	1127,63	90,0%
Select seq gb|KR150238.1|	Ziziphora sp. 1 Kh75 trnL-trnF intergenic spacer region, partial sequence; chloroplast	1138	1138	98%	0.0	97%	KR150238.1	1126,39	89,9%
Select seq gb|KR063656.1|	Satureja pilosa subsp. origanita tRNA-Leu (trnL) gene, partial sequence; trnL-trnF intergenic spacer, complete sequence; and tRNA-Phe (trnF) gene, partial sequence; chloroplast	1160	1160	100%	0.0	97%	KR063656.1	1125,20	89,8%
Select seq gb|KR150233.1|	Thymus sp. 6 Kh133 trnL-trnF intergenic spacer region, partial sequence; chloroplast	1125	1125	97%	0.0	97%	KR150233.1	1125,00	89,8%
Select seq gb|JQ669067.1|	Satureja montana voucher UCBG 2002.0593, Forbes s.n tRNA-Leu (trnL) gene and trnL-trnF intergenic spacer, partial sequence; plastid	1112	1112	96%	0.0	97%	JQ669067.1	1123,58	89,7%
Select seq gb|GU381619.1|	Satureja mutica voucher M:Akhani 12362 tRNA-Leu (trnL) gene and trnL-trnF intergenic spacer, partial sequence; chloroplast	1112	1112	96%	0.0	97%	GU381619.1	1123,58	89,7%
Select seq gb|GU381614.1|	Satureja thymbra voucher M:Braeuchler 2896 tRNA-Leu (trnL) gene and trnL-trnF intergenic spacer, partial sequence; chloroplast	1112	1112	96%	0.0	97%	GU381614.1	1123,58	89,7%
Select seq gb|GU381611.1|	Gontscharovia popovii voucher M:Vvedensky s.n. tRNA-Leu (trnL) gene and trnL-trnF intergenic spacer, partial sequence; chloroplast	1112	1112	96%	0.0	97%	GU381611.1	1123,58	89,7%
Select seq gb|GU381521.1|	Mentha pulegium voucher M:Braeuchler 2300 tRNA-Leu (trnL) gene and trnL-trnF intergenic spacer, partial sequence; chloroplast	1112	1112	96%	0.0	97%	GU381521.1	1123,58	89,7%
Select seq gb|KR150209.1|	Satureja sp. 3 Kh90 trnL-trnF intergenic spacer region, partial sequence; chloroplast	1122	1122	97%	0.0	97%	KR150209.1	1122,00	89,5%

Select seq gb|JQ669021.1|	Clinopodium acinos voucher Judziewicz 14160 tRNA-Leu (trnL) gene and trnL-trnF intergenic spacer, partial sequence; plastid	1110	1110	96%	0.0	97%	JQ669021.1	1121,56	89,5%
Select seq gb|GU381625.1|	Pentapleura subulifera voucher W:Rechinger 12085 tRNA-Leu (trnL) gene and trnL-trnF intergenic spacer, partial sequence; chloroplast	1109	1109	96%	0.0	97%	GU381625.1	1120,55	89,4%
Select seq gb|GU381500.1|	Clinopodium nanum voucher M:Braeuchler 2796 tRNA-Leu (trnL) gene and trnL-trnF intergenic spacer, partial sequence; chloroplast	1109	1109	96%	0.0	97%	GU381500.1	1120,55	89,4%
Select seq gb|GU381615.1|	Satureja innota voucher M:Barra et al. 2484GL tRNA-Leu (trnL) gene and trnL-trnF intergenic spacer, partial sequence; chloroplast	1107	1107	96%	0.0	97%	GU381615.1	1118,53	89,3%
Select seq gb|KC414276.1|	Mentha canadensis isolate 511190001 tRNA-Leu (trnL) gene and trnL-trnF intergenic spacer, partial sequence; chloroplast	1136	1136	100%	0.0	97%	KC414276.1	1101,92	87,9%
Select seq emb|AJ505541.1|	Mentha suaveolens plastid trnL-trnF intergenic spacer, specimen voucher cult., K-1970-3169 (K)	1122	1122	99%	0.0	97%	AJ505541.1	1099,33	87,7%
Select seq gb|DQ667492.1|	Lepechinia lancifolia isolate x232 tRNA-Leu (trnL) gene and trnL-trnF intergenic spacer, partial sequence; chloroplast	1133	1133	100%	0.0	97%	DQ667492.1	1099,01	87,7%
Select seq gb|GU381630.1|	Satureja thymbrifolia voucher M:Danin s.n. tRNA-Leu (trnL) gene and trnL-trnF intergenic spacer, partial sequence; chloroplast	1099	1099	96%	0.0	96%	GU381630.1	1099,00	87,7%
Select seq gb|FJ593456.1|	Mentha sp. JSZ-2009a tRNA-Leu (trnL) gene and trnL-trnF intergenic spacer, partial sequence; chloroplast	1129	1129	100%	0.0	97%	FJ593456.1	1095,13	87,4%
Select seq gb|DQ667514.1|	Mentha arvensis isolate x424 tRNA-Leu (trnL) gene and trnL-trnF intergenic spacer, partial sequence; chloroplast	1123	1123	100%	0.0	96%	DQ667514.1	1078,08	86,0%
Select seq gb|DQ667513.1|	Clinopodium vulgare isolate x423 tRNA-Leu (trnL) gene and trnL-trnF intergenic spacer, partial sequence; chloroplast	1116	1116	100%	0.0	96%	DQ667513.1	1071,36	85,5%
Select seq gb|DQ667504.1|	Cunila incana isolate x296 tRNA-Leu (trnL) gene and trnL-trnF intergenic spacer, partial sequence; chloroplast	1110	1110	100%	0.0	96%	DQ667504.1	1065,60	85,0%
Select seq gb|DQ667507.1|	Pycnanthemum virginianum isolate x313 tRNA-Leu (trnL) gene and trnL-trnF intergenic spacer, partial sequence; chloroplast	1105	1105	100%	0.0	96%	DQ667507.1	1060,80	84,7%
								0,00	0,0%
								0,00	0,0%
								0,00	
								0,00	

Select for downloading or viewing reports	Kh070_ITS Description	Max score	Total score	Query cover	E value	Ident	Accession	(Ident/Cover)* Max score	Deviation from top hit
Select seq gb|AY227143.1|	Satureja hortensis internal transcribed spacer 1, partial sequence; 5.8S ribosomal RNA, complete sequence; and internal transcribed spacer 2, partial sequence	1129	1129	98%	0.0	99%	AY227143.1	1140,52	100,0%
Select seq gb|GU381444.1|	Satureja pilosa voucher M:Braeuchler 4448 internal transcribed spacer 1, partial sequence; 5.8S ribosomal RNA gene, complete sequence; and internal transcribed spacer 2, partial sequence	998	998	94%	0.0	96%	GU381444.1	1019,23	89,4%
Select seq gb|GU381442.1|	Satureja montana voucher M:Braeuchler 2509 internal transcribed spacer 1, partial sequence; 5.8S ribosomal RNA gene, complete sequence; and internal transcribed spacer 2, partial sequence	992	992	94%	0.0	96%	GU381442.1	1013,11	88,8%
Select seq gb|JQ669135.1|	Satureja montana voucher UCBG 2002.0593, Forbes s.n 18S ribosomal RNA gene, internal transcribed spacer 1, 5.8S ribosomal RNA gene, internal transcribed spacer 2, and 26S ribosomal RNA gene, region	1033	1033	98%	0.0	96%	JQ669135.1	1011,92	88,7%
Select seq gb|GU381445.1|	Satureja spinosa voucher M:Braeuchler 4449 internal transcribed spacer 1, partial sequence; 5.8S ribosomal RNA gene, complete sequence; and internal transcribed spacer 2, partial sequence	990	990	94%	0.0	96%	GU381445.1	1011,06	88,6%
Select seq gb|EU823288.1|	Satureja subspicata internal transcribed spacer 1, partial sequence; 5.8S ribosomal RNA gene, complete sequence; and internal transcribed spacer 2, partial sequence	1031	1031	98%	0.0	96%	EU823288.1	1009,96	88,6%
Select seq gb|GU381443.1|	Satureja parnassica voucher M:Braeuchler 4453 internal transcribed spacer 1, partial sequence; 5.8S ribosomal RNA gene, complete sequence; and internal transcribed spacer 2, partial sequence	987	987	94%	0.0	96%	GU381443.1	1008,00	88,4%
Select seq gb|JQ669136.1|	Satureja thymbra voucher UCBG 2002.0540, Forbes s.n. 18S ribosomal RNA gene, internal transcribed spacer 1, 5.8S ribosomal RNA gene, internal transcribed spacer 2, and 26S ribosomal RNA gene, region	1009	1009	98%	0.0	95%	JQ669136.1	978,11	85,8%
Select seq gb|GU381440.1|	Satureja pallaryi voucher TUB:Frey & Kuerschner 83-747 internal transcribed spacer 1, partial sequence; 5.8S ribosomal RNA gene, complete sequence; and internal transcribed spacer 2, partial sequence	965	965	94%	0.0	95%	GU381440.1	975,27	85,5%
Select seq gb|EU823289.1|	Satureja visianii internal transcribed spacer 1, partial sequence; 5.8S ribosomal RNA gene, complete sequence; and internal transcribed spacer 2, partial sequence	998	998	98%	0.0	95%	EU823289.1	967,45	84,8%
Select seq gb|GU381441.1|	Satureja thymbra voucher M:Braeuchler 2896 internal transcribed spacer 1, partial sequence; 5.8S ribosomal RNA gene, complete sequence; and internal transcribed spacer 2, partial sequence	953	953	94%	0.0	95%	GU381441.1	963,14	84,4%
Select seq gb|GU381438.1|	Gontscharovia popovii voucher M:Vvedensky s.n. internal transcribed spacer 1, partial sequence; 5.8S ribosomal RNA gene, complete sequence; and internal transcribed spacer 2, partial sequence	933	933	94%	0.0	94%	GU381438.1	933,00	81,8%
Select seq gb|GU381439.1|	Gontscharovia popovii voucher BM<GBR-LONDON>:Schmid 2419 internal transcribed spacer 1, partial sequence; 5.8S ribosomal RNA gene, complete sequence; and internal transcribed spacer 2, partial sequence	904	904	94%	0.0	93%	GU381439.1	894,38	78,4%
Select seq gb|EU823290.1|	Satureja cuneifolia internal transcribed spacer 1, partial sequence; 5.8S ribosomal RNA gene, complete sequence; and internal transcribed spacer 2, partial sequence	898	898	97%	0.0	93%	EU823290.1	860,97	75,5%
Select seq gb|EU823287.1|	Satureja montana internal transcribed spacer 1, partial sequence; 5.8S ribosomal RNA gene, complete sequence; and internal transcribed spacer 2, partial sequence	893	893	98%	0.0	93%	EU823287.1	847,44	74,3%
Select seq gb|DQ017570.1|	Minthostachys elongata voucher Beck 26146 (LPB) internal transcribed spacer 1, partial sequence; 5.8S ribosomal RNA gene, complete sequence; and internal transcribed spacer 2, partial sequence	767	767	89%	0.0	91%	DQ017570.1	784,24	68,8%
Select seq gb|KR150164.1|	Satureja sp. Kh4 internal transcribed spacer 1, partial sequence; 5.8S ribosomal RNA gene gene, complete sequence; and internal transcribed spacer 2 region, partial sequence	791	791	92%	0.0	91%	KR150164.1	782,40	68,6%
Select seq gb|AF369167.1|	Blephilia ciliata isolate BleCili1786 internal transcribed spacer 1, 5.8S ribosomal RNA gene, and internal transcribed spacer 2, complete sequence	763	763	90%	0.0	90%	AF369167.1	763,00	66,9%
Select seq gb|GU381437.1|	Clinopodium sericeum voucher MSB:Weigend et al. 97-378 internal transcribed spacer 1, partial sequence; 5.8S ribosomal RNA gene, complete sequence; and internal transcribed spacer 2, partial sequence	771	771	91%	0.0	90%	GU381437.1	762,53	66,9%
Select seq gb|GU391426.1|	Hedeoma hyssopifolia voucher C:Boyd et al. 2315 internal transcribed spacer 1, partial sequence; 5.8S ribosomal RNA gene, complete sequence; and internal transcribed spacer 2, partial sequence	769	769	91%	0.0	90%	GU391426.1	760,55	66,7%
Select seq gb|GU381435.1|	Clinopodium flabellifolium voucher M:Weigend & Dostert 98-193 internal transcribed spacer 1, partial sequence; 5.8S ribosomal RNA gene, complete sequence; and internal transcribed spacer 2, partial sequence	765	765	91%	0.0	90%	GU381435.1	756,59	66,3%
Select seq gb|GU381375.1|	Micromeria flagellaris voucher MO:Phillipson et al 5776 internal transcribed spacer 1, partial sequence; 5.8S ribosomal RNA gene, complete sequence; and internal transcribed spacer 2, partial sequence	765	765	91%	0.0	90%	GU381375.1	756,59	66,3%
Select seq gb|GU381436.1|	Clinopodium revolutum voucher M:Weigend & Dostert 98-195 internal transcribed spacer 1, partial sequence; 5.8S ribosomal RNA gene, complete sequence; and internal transcribed spacer 2, partial sequence	763	763	91%	0.0	90%	GU381436.1	754,62	66,2%
Select seq gb|GU381430.1|	Obtegomeria caerulescens voucher K:Wood 4974 internal transcribed spacer 1, partial sequence; 5.8S ribosomal RNA gene, complete sequence; and internal transcribed spacer 2, partial sequence	763	763	91%	0.0	90%	GU381430.1	754,62	66,2%
Select seq gb|GU381374.1|	Micromeria cf. madagascariensis Morawetz 205 internal transcribed spacer 1, partial sequence; 5.8S ribosomal RNA gene, complete sequence; and internal transcribed spacer 2, partial sequence	767	767	92%	0.0	90%	GU381374.1	750,33	65,8%
Select seq gb|DQ017576.1|	Minthostachys setosa voucher Schmidt-Lebuhn 551 (GOET) internal transcribed spacer 1, partial sequence; 5.8S ribosomal RNA gene and internal transcribed spacer 2, complete sequence; and 28S ribosomal RNA gene, partial sequence	806	806	97%	0.0	90%	DQ017576.1	747,84	65,6%

Select seq gb|DQ017575.1|	Minthostachys acutifolia voucher Schmidt-Lebuhn 268 (GOET) internal transcribed spacer 1, partial sequence; 5.8S ribosomal RNA gene and internal transcribed spacer 2, complete sequence; and 28S ribosomal RNA gene, partial sequence	806	806	97%	0.0	90%	DQ017575.1	747,84	65,6%
Select seq gb|DQ017574.1|	Minthostachys mollis voucher Schmidt-Lebuhn 410 (GOET) internal transcribed spacer 1, partial sequence; 5.8S ribosomal RNA gene and internal transcribed spacer 2, complete sequence; and 28S ribosomal RNA gene, partial sequence	797	797	96%	0.0	90%	DQ017574.1	747,19	65,5%
Select seq gb|GU381383.1|	Killickia pilosa voucher M:Braeuchler 3832 internal transcribed spacer 1, partial sequence; 5.8S ribosomal RNA gene, complete sequence; and internal transcribed spacer 2, partial sequence	763	763	92%	0.0	90%	GU381383.1	746,41	65,4%
Select seq gb|GU381376.1|	Micromeria sphaerophylla voucher MO:Phillipson et al 5667 internal transcribed spacer 1, partial sequence; 5.8S ribosomal RNA gene, complete sequence; and internal transcribed spacer 2, partial sequence	763	763	92%	0.0	90%	GU381376.1	746,41	65,4%
Select seq gb|DQ017572.1|	Minthostachys verticillata voucher Schmidt-Lebuhn 294 (GOET) internal transcribed spacer 1, partial sequence; 5.8S ribosomal RNA gene and internal transcribed spacer 2, complete sequence; and 28S ribosomal RNA gene, partial sequence	804	804	97%	0.0	90%	DQ017572.1	745,98	65,4%
Select seq gb|DQ017563.1|	Clinopodium nubigenum voucher Schmidt-Lebuhn 376 (GOET) internal transcribed spacer 1, partial sequence; 5.8S ribosomal RNA gene and internal transcribed spacer 2, complete sequence; and 28S ribosomal RNA gene, partial sequence	795	795	96%	0.0	90%	DQ017563.1	745,31	65,3%
Select seq gb|GU381381.1|	Killickia pilosa voucher NU<ZAF>:Potgieter & Thompson 739 internal transcribed spacer 1, partial sequence; 5.8S ribosomal RNA gene, complete sequence; and internal transcribed spacer 2, partial sequence	761	761	92%	0.0	90%	GU381381.1	744,46	65,3%
Select seq gb|DQ017573.1|	Minthostachys andina voucher Schmidt-Lebuhn 277 (GOET) internal transcribed spacer 1, partial sequence; 5.8S ribosomal RNA gene and internal transcribed spacer 2, complete sequence; and 28S ribosomal RNA gene, partial sequence	802	802	97%	0.0	90%	DQ017573.1	744,12	65,2%
Select seq gb|DQ017560.1|	Clinopodium speciosum voucher Schmidt-Lebuhn 503 (GOET) internal transcribed spacer 1, partial sequence; 5.8S ribosomal RNA gene and internal transcribed spacer 2, complete sequence; and 28S ribosomal RNA gene, partial sequence	802	802	97%	0.0	90%	DQ017560.1	744,12	65,2%
Select seq gb|DQ017571.1|	Minthostachys sp. Hart 1274 internal transcribed spacer 1, partial sequence; 5.8S ribosomal RNA gene and internal transcribed spacer 2, complete sequence; and 28S ribosomal RNA gene, partial sequence	793	793	96%	0.0	90%	DQ017571.1	743,44	65,2%
Select seq gb|DQ017579.1|	Minthostachys acris voucher Schmidt-Lebuhn 564 (GOET) internal transcribed spacer 1, partial sequence; 5.8S ribosomal RNA gene and internal transcribed spacer 2, complete sequence; and 28S ribosomal RNA gene, partial sequence	800	800	97%	0.0	90%	DQ017579.1	742,27	65,1%
Select seq gb|DQ017578.1|	Minthostachys spicata voucher Schmidt-Lebuhn 496 (GOET) internal transcribed spacer 1, partial sequence; 5.8S ribosomal RNA gene and internal transcribed spacer 2, complete sequence; and 28S ribosomal RNA gene, partial sequence	797	797	97%	0.0	90%	DQ017578.1	739,48	64,8%
Select seq gb|AY506638.1|	Minthostachys mollis internal transcribed spacer 1, partial sequence; 5.8S ribosomal RNA gene, complete sequence; and internal transcribed spacer 2, partial sequence	804	804	98%	0.0	90%	AY506638.1	738,37	64,7%
Select seq gb|KC591663.1|	Acanthomintha lanceolata voucher SDSU17310 internal transcribed spacer 1, partial sequence; 5.8S ribosomal RNA gene and internal transcribed spacer 2, complete sequence; and 28S ribosomal RNA gene, partial sequence	819	819	100%	0.0	90%	KC591663.1	737,10	64,6%
Select seq gb|DQ667333.1|	Acanthomintha lanceolata isolate x545 18S ribosomal RNA gene, partial sequence; internal transcribed spacer 1, 5.8S ribosomal RNA gene, and internal transcribed spacer 2, complete sequence; and 28S ribosomal RNA gene, partial sequence	819	819	100%	0.0	90%	DQ667333.1	737,10	64,6%
Select seq gb|JQ669073.1|	Acanthomintha lanceolata voucher Crosby & Morin 14383 18S ribosomal RNA gene, internal transcribed spacer 1, 5.8S ribosomal RNA gene, internal transcribed spacer 2, and 26S ribosomal RNA gene, region	798	798	98%	0.0	90%	JQ669073.1	732,86	64,3%
Select seq gb|DQ017561.1|	Clinopodium sericeum voucher Schmidt-Lebuhn 465 (GOET) internal transcribed spacer 1, partial sequence; 5.8S ribosomal RNA gene and internal transcribed spacer 2, complete sequence; and 28S ribosomal RNA gene, partial sequence	793	793	97%	0.0	89%	DQ017561.1	727,60	63,8%
Select seq gb|DQ017557.1|	Clinopodium jacquelinae voucher Schmidt-Lebuhn 445 (GOET) internal transcribed spacer 1, partial sequence; 5.8S ribosomal RNA gene and internal transcribed spacer 2, complete sequence; and 28S ribosomal RNA gene, partial sequence	791	791	97%	0.0	89%	DQ017557.1	725,76	63,6%
Select seq gb|JQ669089.1|	Cuminia fernandezia voucher Stuessy et al., 11580 18S ribosomal RNA gene, internal transcribed spacer 1, 5.8S ribosomal RNA gene, internal transcribed spacer 2, and 26S ribosomal RNA gene, region	791	791	98%	0.0	89%	JQ669089.1	718,36	63,0%
Select seq gb|JQ669084.1|	Clinopodium taxifolium voucher B. Drew 228 18S ribosomal RNA gene, internal transcribed spacer 1, 5.8S ribosomal RNA gene, internal transcribed spacer 2, and 26S ribosomal RNA gene, region	780	780	97%	0.0	89%	JQ669084.1	715,67	62,7%
Select seq gb|DQ017558.1|	Clinopodium fasciculatum voucher Schmidt-Lebuhn 325 (GOET) internal transcribed spacer 1, partial sequence; 5.8S ribosomal RNA gene and internal transcribed spacer 2, complete sequence; and 28S ribosomal RNA gene, partial sequence	780	780	97%	0.0	89%	DQ017558.1	715,67	62,7%
Select seq gb|JQ669123.1|	Minthostachys mollis voucher B. Drew 349 18S ribosomal RNA gene, internal transcribed spacer 1, 5.8S ribosomal RNA gene, internal transcribed spacer 2, and 26S ribosomal RNA gene, region	787	787	98%	0.0	89%	JQ669123.1	714,72	62,7%
Select seq gb|JQ669122.1|	Minthostachys mollis voucher B. Drew 345 18S ribosomal RNA gene, internal transcribed spacer 1, 5.8S ribosomal RNA gene, internal transcribed spacer 2, and 26S ribosomal RNA gene, region	785	785	98%	0.0	89%	JQ669122.1	712,91	62,5%
Select seq gb|JF301343.1|	Hedeoma piperita voucher B. Drew 92 internal transcribed spacer 1, partial sequence; 5.8S ribosomal RNA gene and internal transcribed spacer 2, complete sequence; and 28S ribosomal RNA gene, partial sequence	782	782	98%	0.0	89%	JF301343.1	710,18	62,3%
Select seq gb|DQ017562.1|	Clinopodium cylindristachys voucher Schmidt-Lebuhn 486 (GOET) internal transcribed spacer 1, partial sequence; 5.8S ribosomal RNA gene and internal transcribed spacer 2, complete sequence; and 28S ribosomal RNA gene, partial sequence	773	773	97%	0.0	89%	DQ017562.1	709,25	62,2%
Select seq gb|JQ669137.1|	Thymbra capitata voucher UCBG 96.0817 s.n. 18S ribosomal RNA gene, internal transcribed spacer 1, 5.8S ribosomal RNA gene, internal transcribed spacer 2, and 26S ribosomal RNA gene, region	778	778	98%	0.0	89%	JQ669137.1	706,55	61,9%
Select seq gb|KC591661.1|	Mentha arvensis voucher UC1862058 internal transcribed spacer 1, partial sequence; 5.8S ribosomal RNA gene and internal transcribed spacer 2, complete sequence; and 28S ribosomal RNA gene, partial sequence	793	793	100%	0.0	89%	KC591661.1	705,77	61,9%
Select seq gb|KF735672.1|	Mentha aquatica strain NH 18S ribosomal RNA gene, partial sequence; internal transcribed spacer 1 and 5.8S ribosomal RNA gene, complete sequence; and internal transcribed spacer 2, partial sequence	761	761	96%	0.0	89%	KF735672.1	705,51	61,9%

Select seq gb|JQ669118.1|	Clinopodium dalmaticum voucher M. Kintgen s.n. 18S ribosomal RNA gene, internal transcribed spacer 1, 5.8S ribosomal RNA gene, internal transcribed spacer 2, and 26S ribosomal RNA gene, region	776	776	98%	0.0	89%	JQ669118.1	704,73	61,8%
Select seq gb|KC591688.1|	Pogogyne zizyphoroides voucher JEPS104333 internal transcribed spacer 1, partial sequence; 5.8S ribosomal RNA gene and internal transcribed spacer 2, complete sequence; and 28S ribosomal RNA gene, partial sequence	767	767	97%	0.0	89%	KC591688.1	703,74	61,7%
Select seq gb|DQ017566.1|	Clinopodium gilliesii voucher Hensen 2313 (LPB) internal transcribed spacer 1, partial sequence; 5.8S ribosomal RNA gene and internal transcribed spacer 2, complete sequence; and 28S ribosomal RNA gene, partial sequence	767	767	97%	0.0	89%	DQ017566.1	703,74	61,7%
Select seq gb|DQ017564.1|	Clinopodium bolivianum voucher Schmidt-Lebuhn 729 (GOET) internal transcribed spacer 1, partial sequence; 5.8S ribosomal RNA gene and internal transcribed spacer 2, complete sequence; and 28S ribosomal RNA gene, partial sequence	767	767	97%	0.0	89%	DQ017564.1	703,74	61,7%
Select seq gb|DQ017559.1|	Clinopodium tomentosum voucher Schmidt-Lebuhn 327 (GOET) internal transcribed spacer 1, partial sequence; 5.8S ribosomal RNA gene and internal transcribed spacer 2, complete sequence; and 28S ribosomal RNA gene, partial sequence	767	767	97%	0.0	89%	DQ017559.1	703,74	61,7%
Select seq gb|JQ669083.1|	Clinopodium macrostemum voucher B. Drew 147 18S ribosomal RNA gene, internal transcribed spacer 1, 5.8S ribosomal RNA gene, internal transcribed spacer 2, and 26S ribosomal RNA gene, region	774	774	98%	0.0	89%	JQ669083.1	702,92	61,6%
Select seq gb|JQ669121.1|	Clinopodium thymifolium voucher M. Kintgen s.n. 18S ribosomal RNA gene, internal transcribed spacer 1, 5.8S ribosomal RNA gene, internal transcribed spacer 2, and 26S ribosomal RNA gene, region	765	765	97%	0.0	89%	JQ669121.1	701,91	61,5%
Select seq gb|DQ017565.1|	Clinopodium axillare voucher Wester 9 (GOET) internal transcribed spacer 1, partial sequence; 5.8S ribosomal RNA gene and internal transcribed spacer 2, complete sequence; and 28S ribosomal RNA gene, partial sequence	763	763	97%	0.0	89%	DQ017565.1	700,07	61,4%
Select seq gb|JQ669090.1|	Cunila lythrifolia voucher Rzedowski 251 18S ribosomal RNA gene, internal transcribed spacer 1, 5.8S ribosomal RNA gene, internal transcribed spacer 2, and 26S ribosomal RNA gene, region	769	769	98%	0.0	89%	JQ669090.1	698,38	61,2%
Select seq gb|JQ669125.1|	Monardella villosa voucher B. Drew 66 18S ribosomal RNA gene, internal transcribed spacer 1, 5.8S ribosomal RNA gene, internal transcribed spacer 2, and 26S ribosomal RNA gene, region	767	767	98%	0.0	89%	JQ669125.1	696,56	61,1%
Select seq gb|JQ230966.1|	Mentha x piperita voucher SBB-1151 internal transcribed spacer 1, partial sequence; 5.8S ribosomal RNA gene and internal transcribed spacer 2, complete sequence; and 28S ribosomal RNA gene, partial sequence	782	782	100%	0.0	89%	JQ230966.1	695,98	61,0%
Select seq gb|JN407476.1|	Mentha canadensis isolate shawpc0994l 18S ribosomal RNA gene, partial sequence; internal transcribed spacer 1, 5.8S ribosomal RNA gene, and internal transcribed spacer 2, complete sequence; and 28S ribosomal RNA gene, partial sequence	782	782	100%	0.0	89%	JN407476.1	695,98	61,0%
Select seq gb|DQ667325.1|	Mentha arvensis isolate x424 18S ribosomal RNA gene, partial sequence; internal transcribed spacer 1, 5.8S ribosomal RNA gene, and internal transcribed spacer 2, complete sequence; and 28S ribosomal RNA gene, partial sequence	782	782	100%	0.0	89%	DQ667325.1	695,98	61,0%
Select seq gb|JQ669093.1|	Cunila pycnantha voucher Ruiz 3150 18S ribosomal RNA gene, internal transcribed spacer 1, 5.8S ribosomal RNA gene, internal transcribed spacer 2, and 26S ribosomal RNA gene, region	765	765	98%	0.0	89%	JQ669093.1	694,74	60,9%
Select seq gb|JQ669092.1|	Cunila origanoides voucher Stoots s.n. 18S ribosomal RNA gene, internal transcribed spacer 1, 5.8S ribosomal RNA gene, internal transcribed spacer 2, and 26S ribosomal RNA gene, region	765	765	98%	0.0	89%	JQ669092.1	694,74	60,9%
Select seq gb|DQ667305.1|	Cunila microcephala isolate x226 18S ribosomal RNA gene, partial sequence; internal transcribed spacer 1, 5.8S ribosomal RNA gene, and internal transcribed spacer 2, complete sequence; and 28S ribosomal RNA gene, partial sequence	780	780	100%	0.0	89%	DQ667305.1	694,20	60,9%
Select seq gb|JQ669091.1|	Cunila microcephala voucher Sytsma 7247 18S ribosomal RNA gene, internal transcribed spacer 1, 5.8S ribosomal RNA gene, internal transcribed spacer 2, and 26S ribosomal RNA gene, region	763	763	98%	0.0	89%	JQ669091.1	692,93	60,8%
Select seq gb|AY506636.1|	Cuminia fernandezia internal transcribed spacer 1, partial sequence; 5.8S ribosomal RNA gene, complete sequence; and internal transcribed spacer 2, partial sequence	763	763	98%	0.0	89%	AY506636.1	692,93	60,8%
Select seq gb|DQ667311.1|	Poliomintha palmeri isolate x259 18S ribosomal RNA gene, partial sequence; internal transcribed spacer 1, 5.8S ribosomal RNA gene, and internal transcribed spacer 2, complete sequence; and 28S ribosomal RNA gene, partial sequence	776	776	100%	0.0	89%	DQ667311.1	690,64	60,6%
Select seq gb|JQ669115.1|	Mentha arvensis voucher B. Drew 82 18S ribosomal RNA gene, internal transcribed spacer 1, 5.8S ribosomal RNA gene, internal transcribed spacer 2, and 26S ribosomal RNA gene, region	765	765	98%	0.0	88%	JQ669115.1	686,94	60,2%
Select seq gb|KC591689.1|	Pogogyne zizyphoroides voucher SDSU19277 internal transcribed spacer 1, partial sequence; 5.8S ribosomal RNA gene and internal transcribed spacer 2, complete sequence; and 28S ribosomal RNA gene, partial sequence	773	773	100%	0.0	88%	KC591689.1	680,24	59,6%
Select seq gb|KC591685.1|	Pogogyne serpylloides voucher SBBG119413 internal transcribed spacer 1, partial sequence; 5.8S ribosomal RNA gene and internal transcribed spacer 2, complete sequence; and 28S ribosomal RNA gene, partial sequence	773	773	100%	0.0	88%	KC591685.1	680,24	59,6%
Select seq gb|KC591683.1|	Pogogyne serpylloides voucher SDSU18437 internal transcribed spacer 1, partial sequence; 5.8S ribosomal RNA gene and internal transcribed spacer 2, complete sequence; and 28S ribosomal RNA gene, partial sequence	773	773	100%	0.0	88%	KC591683.1	680,24	59,6%
Select seq gb|KC591682.1|	Pogogyne zizyphoroides voucher SDSU18438 internal transcribed spacer 1, partial sequence; 5.8S ribosomal RNA gene and internal transcribed spacer 2, complete sequence; and 28S ribosomal RNA gene, partial sequence	773	773	100%	0.0	88%	KC591682.1	680,24	59,6%
Select seq gb|KC591681.1|	Pogogyne serpylloides voucher SDSU18436 internal transcribed spacer 1, partial sequence; 5.8S ribosomal RNA gene and internal transcribed spacer 2, complete sequence; and 28S ribosomal RNA gene, partial sequence	773	773	100%	0.0	88%	KC591681.1	680,24	59,6%
Select seq gb|KC591677.1|	Pogogyne floribunda voucher UC1609361 internal transcribed spacer 1, partial sequence; 5.8S ribosomal RNA gene and internal transcribed spacer 2, complete sequence; and 28S ribosomal RNA gene, partial sequence	773	773	100%	0.0	88%	KC591677.1	680,24	59,6%
Select seq gb|JQ669133.1|	Rhabdocaulon strictum voucher Singhurst s.n 18S ribosomal RNA gene, internal transcribed spacer 1, 5.8S ribosomal RNA gene, internal transcribed spacer 2, and 26S ribosomal RNA gene, region	756	756	98%	0.0	88%	JQ669133.1	678,86	59,5%
Select seq gb|KC591662.1|	Acanthomintha ilicifolia voucher SDSU12198 internal transcribed spacer 1, partial sequence; 5.8S ribosomal RNA gene and internal transcribed spacer 2, complete sequence; and 28S ribosomal RNA gene, partial sequence	769	769	100%	0.0	88%	KC591662.1	676,72	59,3%

Select seq gb|DQ667242.1|	Thymus serpyllum isolate x075 18S ribosomal RNA gene, partial sequence; internal transcribed spacer 1, 5.8S ribosomal RNA gene, and internal transcribed spacer 2, complete sequence; and 28S ribosomal RNA gene, partial sequence	769	769	100% 0.0	88% DQ667242.1	676,72	59,3%
Select seq gb|DQ667236.1|	Hedeoma costata isolate x067 18S ribosomal RNA gene, partial sequence; internal transcribed spacer 1, 5.8S ribosomal RNA gene, and internal transcribed spacer 2, complete sequence; and 28S ribosomal RNA gene, partial sequence	769	769	100% 0.0	88% DQ667236.1	676,72	59,3%
Select seq gb|KC591686.1|	Pogogyne serpylloides voucher SDSU19288 internal transcribed spacer 1, partial sequence; 5.8S ribosomal RNA gene and internal transcribed spacer 2, complete sequence; and 28S ribosomal RNA gene, partial sequence	767	767	100% 0.0	88% KC591686.1	674,96	59,2%
Select seq gb|KC591671.1|	Pogogyne douglasii voucher SDSU19273 internal transcribed spacer 1, partial sequence; 5.8S ribosomal RNA gene and internal transcribed spacer 2, complete sequence; and 28S ribosomal RNA gene, partial sequence	767	767	100% 0.0	88% KC591671.1	674,96	59,2%
Select seq gb|KC591670.1|	Pogogyne douglasii voucher JEPS100839 internal transcribed spacer 1, partial sequence; 5.8S ribosomal RNA gene and internal transcribed spacer 2, complete sequence; and 28S ribosomal RNA gene, partial sequence	767	767	100% 0.0	88% KC591670.1	674,96	59,2%
Select seq gb|KC591668.1|	Pogogyne clareana voucher SBBG111196 internal transcribed spacer 1, partial sequence; 5.8S ribosomal RNA gene and internal transcribed spacer 2, complete sequence; and 28S ribosomal RNA gene, partial sequence	765	765	100% 0.0	88% KC591668.1	673,20	59,0%
Select seq gb|DQ667319.1|	Pycnanthemum virginianum isolate x313 18S ribosomal RNA gene, partial sequence; internal transcribed spacer 1, 5.8S ribosomal RNA gene, and internal transcribed spacer 2, complete sequence; and 28S ribosomal RNA gene, partial sequence	765	765	100% 0.0	88% DQ667319.1	673,20	59,0%
Select seq gb|KC591675.1|	Pogogyne douglasii voucher SDSU19309 internal transcribed spacer 1, partial sequence; 5.8S ribosomal RNA gene and internal transcribed spacer 2, complete sequence; and 28S ribosomal RNA gene, partial sequence	763	763	100% 0.0	88% KC591675.1	671,44	58,9%
Select seq gb|DQ667303.1|	Glechon marifolia isolate x223 18S ribosomal RNA gene, partial sequence; internal transcribed spacer 1, 5.8S ribosomal RNA gene, and internal transcribed spacer 2, complete sequence; and 28S ribosomal RNA gene, partial sequence	763	763	100% 0.0	88% DQ667303.1	671,44	58,9%
Select seq gb|JQ669080.1|	Clinopodium arkansanum voucher B. Drew 80 18S ribosomal RNA gene, internal transcribed spacer 1, 5.8S ribosomal RNA gene, internal transcribed spacer 2, and 26S ribosomal RNA gene, region	747	747	98% 0.0	88% JQ669080.1	670,78	58,8%
Select seq gb|KR150165.1|	Thymus sp. Kh8 internal transcribed spacer 1, partial sequence; 5.8S ribosomal RNA gene gene, complete sequence; and internal transcribed spacer 2 region, partial sequence	761	761	100% 0.0	88% KR150165.1	669,68	58,7%
Select seq gb|KR150146.1|	Thymus sp. Kh57 internal transcribed spacer 1, partial sequence; 5.8S ribosomal RNA gene gene, complete sequence; and internal transcribed spacer 2 region, partial sequence	761	761	100% 0.0	88% KR150146.1	669,68	58,7%
Select seq gb|EU556517.1|	Thymus quinquecostatus isolate N2 internal transcribed spacer 1, partial sequence; 5.8S ribosomal RNA gene, complete sequence; and internal transcribed spacer 2, partial sequence	761	761	100% 0.0	88% EU556517.1	669,68	58,7%
Select seq gb|EU556506.1|	Thymus quinquecostatus isolate D1 internal transcribed spacer 1, partial sequence; 5.8S ribosomal RNA gene, complete sequence; and internal transcribed spacer 2, partial sequence	761	761	100% 0.0	88% EU556506.1	669,68	58,7%
Select seq gb|FJ236468.1|	Thymus persicus isolate Tabriz 18S ribosomal RNA gene, partial sequence; internal transcribed spacer 1, 5.8S ribosomal RNA gene, and internal transcribed spacer 2, complete sequence; and 28S ribosomal RNA gene, partial sequence	761	761	100% 0.0	88% FJ236468.1	669,68	58,7%
Select seq gb|EU735059.1|	Thymus trautvetteri 18S ribosomal RNA gene, partial sequence; internal transcribed spacer 1, 5.8S ribosomal RNA gene, and internal transcribed spacer 2, complete sequence; and 28S ribosomal RNA gene, partial sequence	761	761	100% 0.0	88% EU735059.1	669,68	58,7%
Select seq gb|KC591667.1|	Pogogyne abramsii voucher SDSU19270 internal transcribed spacer 1, partial sequence; 5.8S ribosomal RNA gene and internal transcribed spacer 2, complete sequence; and 28S ribosomal RNA gene, partial sequence	760	760	100% 0.0	88% KC591667.1	668,80	58,6%

Select for downloading or viewing reports	Kh070_trnL Description	Max score	Total score	Query cover	E value	Ident	Accession	(Ident/Cover)* Max score	Deviation from top hit
Select seq gb|GU381620.1|	Satureja hortensis voucher M:Braeuchler 2422 tRNA-Leu (trnL) gene and trnL-trnF intergenic spacer, partial sequence; chloroplast	1234	1234	98%	0.0	99%	GU381620.1	1246,59	100,0%
Select seq gb|GU381621.1|	Satureja cuneifolia voucher M:Rechinger 11142 tRNA-Leu (trnL) gene and trnL-trnF intergenic spacer, partial sequence; chloroplast	1203	1203	98%	0.0	99%	GU381621.1	1215,28	97,5%
Select seq gb|GU381612.1|	Gontscharovia popovii voucher BM<GBR-LONDON>:Schmid 2419 tRNA-Leu (trnL) gene and trnL-trnF intergenic spacer, partial sequence; chloroplast	1203	1203	98%	0.0	99%	GU381612.1	1215,28	97,5%
Select seq gb|AY840179.1|	Satureja montana tRNA-Leu (trnL) gene and trnL-trnF intergenic spacer, partial sequence; chloroplast	1203	1203	98%	0.0	99%	AY840179.1	1215,28	97,5%
Select seq gb|KR150214.1|	Satureja sp. 1 Kh04 trnL-trnF intergenic spacer region, partial sequence; chloroplast	1175	1175	96%	0.0	99%	KR150214.1	1211,72	97,2%
Select seq gb|JQ669067.1|	Satureja montana voucher UCBG 2002.0593, Forbes s.n tRNA-Leu (trnL) gene and trnL-trnF intergenic spacer, partial sequence; plastid	1197	1197	98%	0.0	99%	JQ669067.1	1209,21	97,0%
Select seq gb|GU381619.1|	Satureja mutica voucher M:Akhani 12362 tRNA-Leu (trnL) gene and trnL-trnF intergenic spacer, partial sequence; chloroplast	1197	1197	98%	0.0	99%	GU381619.1	1209,21	97,0%
Select seq gb|GU381614.1|	Satureja thymbra voucher M:Braeuchler 2896 tRNA-Leu (trnL) gene and trnL-trnF intergenic spacer, partial sequence; chloroplast	1197	1197	98%	0.0	99%	GU381614.1	1209,21	97,0%
Select seq gb|GU381611.1|	Gontscharovia popovii voucher M:Vvedensky s.n. tRNA-Leu (trnL) gene and trnL-trnF intergenic spacer, partial sequence; chloroplast	1197	1197	98%	0.0	99%	GU381611.1	1209,21	97,0%
Select seq gb|KR063656.1|	Satureja pilosa subsp. origanita tRNA-Leu (trnL) gene, partial sequence; trnL-trnF intergenic spacer, complete sequence; and tRNA-Phe (trnF) gene, partial sequence; chloroplast	1221	1221	100%	0.0	99%	KR063656.1	1208,79	97,0%
Select seq gb|KR150209.1|	Satureja sp. 3 Kh90 trnL-trnF intergenic spacer region, partial sequence; chloroplast	1206	1206	99%	0.0	99%	KR150209.1	1206,00	96,7%
Select seq gb|GU381622.1|	Satureja intermedia voucher M:Rechinger 43441 tRNA-Leu (trnL) gene and trnL-trnF intergenic spacer, partial sequence; chloroplast	1192	1192	98%	0.0	99%	GU381622.1	1204,16	96,6%
Select seq gb|GU381615.1|	Satureja innota voucher M:Barra et al. 2484GL tRNA-Leu (trnL) gene and trnL-trnF intergenic spacer, partial sequence; chloroplast	1192	1192	98%	0.0	99%	GU381615.1	1204,16	96,6%
Select seq gb|GU381623.1|	Satureja spicigera voucher M:Kartli et al. 2726 tRNA-Leu (trnL) gene and trnL-trnF intergenic spacer, partial sequence; chloroplast	1186	1186	98%	0.0	99%	GU381623.1	1198,10	96,1%
Select seq gb|JQ669068.1|	Satureja thymbra voucher UCBG 2002.0540, Forbes s.n. tRNA-Leu (trnL) gene and trnL-trnF intergenic spacer, partial sequence; plastid	1181	1181	98%	0.0	99%	JQ669068.1	1193,05	95,7%
Select seq gb|AY506611.1|	Satureja hortensis tRNA-Leu (trnL) gene and trnL-trnF intergenic spacer, partial sequence; chloroplast	1155	1155	95%	0.0	98%	AY506611.1	1191,47	95,6%
Select seq gb|GU381629.1|	Thymbra capitata voucher M:Braeuchler 2518 tRNA-Leu (trnL) gene and trnL-trnF intergenic spacer, partial sequence; chloroplast	1122	1122	98%	0.0	97%	GU381629.1	1110,55	89,1%
Select seq gb|GU381484.1|	Micromeria flagellaris voucher E:van der Werff & McPherson 13570 tRNA-Leu (trnL) gene and trnL-trnF intergenic spacer, partial sequence; chloroplast	1122	1122	98%	0.0	97%	GU381484.1	1110,55	89,1%
Select seq gb|GU381483.1|	Micromeria flagellaris voucher E:Clement et al 2140 tRNA-Leu (trnL) gene and trnL-trnF intergenic spacer, partial sequence; chloroplast	1122	1122	98%	0.0	97%	GU381483.1	1110,55	89,1%
Select seq gb|JF301401.1|	Thymbra capitata voucher UCBG 96.0817 tRNA-Leu (trnL) gene and trnL-trnF intergenic spacer, partial sequence; chloroplast	1096	1096	96%	0.0	97%	JF301401.1	1107,42	88,8%
Select seq gb|AY840207.1|	Thymbra spicata tRNA-Leu (trnL) gene and trnL-trnF intergenic spacer, partial sequence; chloroplast	1118	1118	98%	0.0	97%	AY840207.1	1106,59	88,8%
Select seq gb|GU381517.1|	Clinopodium barosmum voucher BM<GBR-LONDON>:McLaren N193 tRNA-Leu (trnL) gene and trnL-trnF intergenic spacer, partial sequence; chloroplast	1116	1116	98%	0.0	97%	GU381517.1	1104,61	88,6%
Select seq gb|GU381516.1|	Clinopodium wardii voucher BM<GBR-LONDON>:Ludlow et al. 14234 tRNA-Leu (trnL) gene and trnL-trnF intergenic spacer, partial sequence; chloroplast	1116	1116	98%	0.0	97%	GU381516.1	1104,61	88,6%
Select seq gb|GU381515.1|	Clinopodium hydaspidis voucher BM<GBR-LONDON>:Mohd 133 tRNA-Leu (trnL) gene and trnL-trnF intergenic spacer, partial sequence; chloroplast	1116	1116	98%	0.0	97%	GU381515.1	1104,61	88,6%
Select seq gb|GU381514.1|	Clinopodium nepalense voucher FR:Stainton 6024 tRNA-Leu (trnL) gene and trnL-trnF intergenic spacer, partial sequence; chloroplast	1116	1116	98%	0.0	97%	GU381514.1	1104,61	88,6%
Select seq gb|GU381511.1|	Clinopodium piperitum voucher BM<GBR-LONDON>:Vickery 454 tRNA-Leu (trnL) gene and trnL-trnF intergenic spacer, partial sequence; chloroplast	1116	1116	98%	0.0	97%	GU381511.1	1104,61	88,6%

Select seq gb|GU381632.1|	Thymbra spicata voucher M:Braeuchler 4548 tRNA-Leu (trnL) gene and trnL-trnF intergenic spacer, partial sequence; chloroplast	1112	1112	98% 0.0	97% GU381632.1	1100,65	88,3%
Select seq gb|GU381513.1|	Clinopodium nepalense voucher BM<GBR-LONDON>:Mikage et al. 9550294 tRNA-Leu (trnL) gene and trnL-trnF intergenic spacer, partial sequence; chloroplast	1088	1088	96% 0.0	97% GU381513.1	1099,33	88,2%
Select seq gb|GU381627.1|	Thymbra sintenisii subsp. isaurica voucher E:Goener 12628 tRNA-Leu (trnL) gene and trnL-trnF intergenic spacer, partial sequence; chloroplast	1110	1110	98% 0.0	97% GU381627.1	1098,67	88,1%
Select seq gb|GU381512.1|	Clinopodium piperitum voucher E:Stainton 7320 tRNA-Leu (trnL) gene and trnL-trnF intergenic spacer, partial sequence; chloroplast	1110	1110	98% 0.0	97% GU381512.1	1098,67	88,1%
Select seq gb|KR150198.1|	Ziziphora sp. 3 Kh112 trnL-trnF intergenic spacer region, partial sequence; chloroplast	1098	1098	98% 0.0	96% KR150198.1	1075,59	86,3%
Select seq gb|KR150243.1|	Ziziphora sp. 2 Kh20 trnL-trnF intergenic spacer region, partial sequence; chloroplast	1085	1085	97% 0.0	96% KR150243.1	1073,81	86,1%
Select seq gb|GU381496.1|	Killickia pilosa voucher M:Braeuchler 3832 tRNA-Leu (trnL) gene and trnL-trnF intergenic spacer, partial sequence; chloroplast	1096	1096	98% 0.0	96% GU381496.1	1073,63	86,1%
Select seq gb|GU381493.1|	Killickia grandiflora voucher M:Braeuchler 3811 tRNA-Leu (trnL) gene and trnL-trnF intergenic spacer, partial sequence; chloroplast	1096	1096	98% 0.0	96% GU381493.1	1073,63	86,1%
Select seq gb|DQ667501.1|	Ziziphora taurica isolate x262 tRNA-Leu (trnL) gene and trnL-trnF intergenic spacer, partial sequence; chloroplast	1096	1096	98% 0.0	96% DQ667501.1	1073,63	86,1%
Select seq gb|GU381633.1|	Thymus caespititius voucher M:Heubl s.n. tRNA-Leu (trnL) gene and trnL-trnF intergenic spacer, partial sequence; chloroplast	1094	1094	98% 0.0	96% GU381633.1	1071,67	86,0%
Select seq gb|GU381510.1|	Ziziphora pamiroalaica voucher C:Murray et al. 10090 tRNA-Leu (trnL) gene and trnL-trnF intergenic spacer, partial sequence; chloroplast	1094	1094	98% 0.0	96% GU381510.1	1071,67	86,0%
Select seq gb|GU381505.1|	Clinopodium troodi voucher W:Davis 1856 tRNA-Leu (trnL) gene and trnL-trnF intergenic spacer, partial sequence; chloroplast	1094	1094	98% 0.0	96% GU381505.1	1071,67	86,0%
Select seq gb|GU381501.1|	Clinopodium graveolens subsp. rotundifolium voucher M:Podlech 47181 tRNA-Leu (trnL) gene and trnL-trnF intergenic spacer, partial sequence; chloroplast	1094	1094	98% 0.0	96% GU381501.1	1071,67	86,0%
Select seq gb|GU381499.1|	Clinopodium suaveolens voucher M:Erben s.n. tRNA-Leu (trnL) gene and trnL-trnF intergenic spacer, partial sequence; chloroplast	1094	1094	98% 0.0	96% GU381499.1	1071,67	86,0%
Select seq gb|GU381485.1|	Micromeria sphaerophylla voucher E:Lewis et al 1064 tRNA-Leu (trnL) gene and trnL-trnF intergenic spacer, partial sequence; chloroplast	1094	1094	98% 0.0	96% GU381485.1	1071,67	86,0%
Select seq gb|GU381481.1|	Micromeria cf. madagascariensis Morawetz 205 tRNA-Leu (trnL) gene and trnL-trnF intergenic spacer, partial sequence; chloroplast	1094	1094	98% 0.0	96% GU381481.1	1071,67	86,0%
Select seq gb|GU381500.1|	Clinopodium nanum voucher M:Braeuchler 2796 tRNA-Leu (trnL) gene and trnL-trnF intergenic spacer, partial sequence; chloroplast	1081	1081	97% 0.0	96% GU381500.1	1069,86	85,8%
Select seq gb|GU381495.1|	Killickia pilosa voucher M:Braeuchler 3810 tRNA-Leu (trnL) gene and trnL-trnF intergenic spacer, partial sequence; chloroplast	1081	1081	97% 0.0	96% GU381495.1	1069,86	85,8%
Select seq gb|KR150218.1|	Thymus sp. 2 Kh69 trnL-trnF intergenic spacer region, partial sequence; chloroplast	1103	1103	99% 0.0	96% KR150218.1	1069,58	85,8%
Select seq gb|GU381631.1|	Satureja linearifolia voucher M:Brullo & Furnari s.n. tRNA-Leu (trnL) gene and trnL-trnF intergenic spacer, partial sequence; chloroplast	1090	1090	98% 0.0	96% GU381631.1	1067,76	85,7%
Select seq gb|KR150215.1|	Thymus sp. 1 Kh08 trnL-trnF intergenic spacer region, partial sequence; chloroplast	1066	1066	96% 0.0	96% KR150215.1	1066,00	85,5%
Select seq gb|GU381502.1|	Ziziphora hispanica subsp. aragonensis voucher M:Podlech & Lippert 2492 tRNA-Leu (trnL) gene and trnL-trnF intergenic spacer, partial sequence; chloroplast	1066	1066	96% 0.0	96% GU381502.1	1066,00	85,5%
Select seq gb|GU381640.1|	Argantoniella salzmannii voucher M:Barra et al. 2673GL tRNA-Leu (trnL) gene and trnL-trnF intergenic spacer, partial sequence; chloroplast	1088	1088	98% 0.0	96% GU381640.1	1065,80	85,5%
Select seq gb|GU381507.1|	Ziziphora tenuior voucher MSB:Fayvush et al. 03-1503 tRNA-Leu (trnL) gene and trnL-trnF intergenic spacer, partial sequence; chloroplast	1088	1088	98% 0.0	96% GU381507.1	1065,80	85,5%
Select seq gb|GU381497.1|	Clinopodium acinos voucher M:Podlech 50287 tRNA-Leu (trnL) gene and trnL-trnF intergenic spacer, partial sequence; chloroplast	1088	1088	98% 0.0	96% GU381497.1	1065,80	85,5%
Select seq gb|AY840202.1|	Origanum vulgare tRNA-Leu (trnL) gene and trnL-trnF intergenic spacer, partial sequence; chloroplast	1088	1088	98% 0.0	96% AY840202.1	1065,80	85,5%
Select seq gb|KR150225.1|	Thymus sp. 4 Kh24 trnL-trnF intergenic spacer region, partial sequence; chloroplast	1099	1099	99% 0.0	96% KR150225.1	1065,70	85,5%
Select seq gb|GU381628.1|	Thymbra calostachya voucher M:Ulrich s.n. tRNA-Leu (trnL) gene and trnL-trnF intergenic spacer, partial sequence; chloroplast	1086	1086	98% 0.0	96% GU381628.1	1063,84	85,3%

Select seq gb|GU381489.1|	Killickia lutea voucher NU<ZAF>:Hilliard & Burtt 9876 tRNA-Leu (trnL) gene and trnL-trnF intergenic spacer, partial sequence; chloroplast	1085	1085	98%	0.0	96%	GU381489.1	1062,86	85,3%
Select seq gb|GU381488.1|	Killickia compacta voucher M:Braeuchler 3816 tRNA-Leu (trnL) gene and trnL-trnF intergenic spacer, partial sequence; chloroplast	1085	1085	98%	0.0	96%	GU381488.1	1062,86	85,3%
Select seq gb|KR150238.1|	Ziziphora sp. 1 Kh75 trnL-trnF intergenic spacer region, partial sequence; chloroplast	1107	1107	100%	0.0	96%	KR150238.1	1062,72	85,3%
Select seq gb|JX880022.1|	Origanum vulgare subsp. vulgare chloroplast, complete genome	1107	1107	100%	0.0	96%	JX880022.1	1062,72	85,3%
Select seq gb|JQ690290.1|	Origanum elongatum isolate H5_O_elo trnL-trnF intergenic spacer, partial sequence; chloroplast	1107	1107	100%	0.0	96%	JQ690290.1	1062,72	85,3%
Select seq gb|JQ690289.1|	Origanum rotundifolium isolate DNA3_O_rot trnL-trnF intergenic spacer, partial sequence; chloroplast	1107	1107	100%	0.0	96%	JQ690289.1	1062,72	85,3%
Select seq emb|AJ505543.1|	Origanum vulgare plastid trnL-trnF intergenic spacer, specimen voucher cult., K-000-69-19317, chase 13334 (K)	1107	1107	100%	0.0	96%	AJ505543.1	1062,72	85,3%
Select seq gb|JQ669021.1|	Clinopodium acinos voucher Judziewicz 14160 tRNA-Leu (trnL) gene and trnL-trnF intergenic spacer, partial sequence; plastid	1083	1083	98%	0.0	96%	JQ669021.1	1060,90	85,1%
Select seq gb|GU381635.1|	Thymus broussonetii subsp. hannonis voucher MSB:Podlech 142 tRNA-Leu (trnL) gene and trnL-trnF intergenic spacer, partial sequence; chloroplast	1083	1083	98%	0.0	96%	GU381635.1	1060,90	85,1%
Select seq gb|AY570463.1|	Origanum vulgare voucher JBW 2567 tRNA-Leu and trnL-trnF intergenic spacer, partial sequence; chloroplast	1083	1083	98%	0.0	96%	AY570463.1	1060,90	85,1%
Select seq gb|AY618513.1|	Mentha arvensis tRNA-Leu (trnL) gene and trnL-trnF intergenic spacer, partial sequence; chloroplast	1070	1070	97%	0.0	96%	AY618513.1	1058,97	84,9%
Select seq gb|EU556536.1|	Thymus marschallianus isolate XY tRNA-Leu (trnL) gene and trnL-trnF intergenic spacer, partial sequence; chloroplast	1068	1068	97%	0.0	96%	EU556536.1	1056,99	84,8%
Select seq gb|EU556530.1|	Thymus mandschuricus isolate HM tRNA-Leu (trnL) gene and trnL-trnF intergenic spacer, partial sequence; chloroplast	1079	1079	98%	0.0	96%	EU556530.1	1056,98	84,8%
Select seq gb|KR063657.1|	Thymus sibthorpii tRNA-Leu (trnL) gene, partial sequence; trnL-trnF intergenic spacer, complete sequence; and tRNA-Phe (trnF) gene, partial sequence; chloroplast	1101	1101	100%	0.0	96%	KR063657.1	1056,96	84,8%
Select seq gb|KR150229.1|	Thymus sp. 5 Kh16 trnL-trnF intergenic spacer region, partial sequence; chloroplast	1101	1101	100%	0.0	96%	KR150229.1	1056,96	84,8%
Select seq gb|KR150222.1|	Thymus sp. 3 Kh65 trnL-trnF intergenic spacer region, partial sequence; chloroplast	1101	1101	100%	0.0	96%	KR150222.1	1056,96	84,8%
Select seq gb|EU556532.1|	Thymus amurensis isolate HX tRNA-Leu (trnL) gene and trnL-trnF intergenic spacer, partial sequence; chloroplast	1101	1101	100%	0.0	96%	EU556532.1	1056,96	84,8%
Select seq gb|EU556525.1|	Thymus quinquecostatus isolate CL tRNA-Leu (trnL) gene and trnL-trnF intergenic spacer, partial sequence; chloroplast	1101	1101	100%	0.0	96%	EU556525.1	1056,96	84,8%
Select seq gb|JQ669069.1|	Thymus pulegioides voucher Riina 1577 tRNA-Leu (trnL) gene and trnL-trnF intergenic spacer, partial sequence; plastid	1077	1077	98%	0.0	96%	JQ669069.1	1055,02	84,6%
Select seq gb|GU381637.1|	Thymus serpyllum voucher M:Braeuchler 2514 tRNA-Leu (trnL) gene and trnL-trnF intergenic spacer, partial sequence; chloroplast	1077	1077	98%	0.0	96%	GU381637.1	1055,02	84,6%
Select seq gb|AY618515.1|	Mentha canadensis tRNA-Leu (trnL) gene and trnL-trnF intergenic spacer, partial sequence; chloroplast	1064	1064	97%	0.0	96%	AY618515.1	1053,03	84,5%
Select seq gb|AY618512.1|	Mentha aquatica tRNA-Leu (trnL) gene and trnL-trnF intergenic spacer, partial sequence; chloroplast	1064	1064	97%	0.0	96%	AY618512.1	1053,03	84,5%
Select seq gb|JQ690293.1|	Origanum dayi isolate H43_O_day trnL-trnF intergenic spacer, partial sequence; chloroplast	1096	1096	100%	0.0	96%	JQ690293.1	1052,16	84,4%
Select seq gb|FJ593456.1|	Mentha sp. JSZ-2009a tRNA-Leu (trnL) gene and trnL-trnF intergenic spacer, partial sequence; chloroplast	1096	1096	100%	0.0	96%	FJ593456.1	1052,16	84,4%
Select seq gb|EU556539.1|	Thymus quinquecostatus isolate ZY tRNA-Leu (trnL) gene and trnL-trnF intergenic spacer, partial sequence; chloroplast	1096	1096	100%	0.0	96%	EU556539.1	1052,16	84,4%
Select seq gb|EU556538.1|	Thymus quinquecostatus isolate ZJ tRNA-Leu (trnL) gene and trnL-trnF intergenic spacer, partial sequence; chloroplast	1096	1096	100%	0.0	96%	EU556538.1	1052,16	84,4%
Select seq gb|EU556533.1|	Thymus quinquecostatus isolate HY tRNA-Leu (trnL) gene and trnL-trnF intergenic spacer, partial sequence; chloroplast	1096	1096	100%	0.0	96%	EU556533.1	1052,16	84,4%
Select seq gb|EU556527.1|	Thymus mongolicus isolate G2 tRNA-Leu (trnL) gene and trnL-trnF intergenic spacer, partial sequence; chloroplast	1096	1096	100%	0.0	96%	EU556527.1	1052,16	84,4%

Select seq gb|EU556526.1|	Thymus quinquecostatus isolate D2 tRNA-Leu (trnL) gene and trnL-trnF intergenic spacer, partial sequence; chloroplast	1096	1096	100% 0.0	96%	EU556526.1	1052,16	84,4%
Select seq gb|GU381634.1|	Thymus pulegioides voucher M:Braeuchler 3129 tRNA-Leu (trnL) gene and trnL-trnF intergenic spacer, partial sequence; chloroplast	1074	1074	98% 0.0	96%	GU381634.1	1052,08	84,4%
Select seq gb|GU381636.1|	Thymus vulgaris voucher M:Braeuchler 3683 tRNA-Leu (trnL) gene and trnL-trnF intergenic spacer, partial sequence; chloroplast	1072	1072	98% 0.0	96%	GU381636.1	1050,12	84,2%
Select seq gb|EU556531.1|	Thymus amurensis isolate HW tRNA-Leu (trnL) gene and trnL-trnF intergenic spacer, partial sequence; chloroplast	1090	1090	100% 0.0	96%	EU556531.1	1046,40	83,9%
Select seq gb|GU381613.1|	Satureja pallaryi voucher TUB:Frey & Kuerschner 83-747 tRNA-Leu (trnL) gene and trnL-trnF intergenic spacer, partial sequence; chloroplast	1079	1079	98% 0.0	95%	GU381613.1	1045,97	83,9%
Select seq gb|DQ667514.1|	Mentha arvensis isolate x424 tRNA-Leu (trnL) gene and trnL-trnF intergenic spacer, partial sequence; chloroplast	1088	1088	100% 0.0	96%	DQ667514.1	1044,48	83,8%
Select seq gb|GU381521.1|	Mentha pulegium voucher M:Braeuchler 2300 tRNA-Leu (trnL) gene and trnL-trnF intergenic spacer, partial sequence; chloroplast	1064	1064	98% 0.0	96%	GU381521.1	1042,29	83,6%
Select seq emb|AJ505544.1|	Thymus serpyllum var. citriodorum plastid trnL-trnF intergenic spacer, specimen voucher cult., K-1975-1177, Chase 13331 (K)	1085	1085	100% 0.0	96%	AJ505544.1	1041,60	83,6%
Select seq gb|EU556528.1|	Thymus mongolicus isolate G3 tRNA-Leu (trnL) gene and trnL-trnF intergenic spacer, partial sequence; chloroplast	1072	1072	99% 0.0	96%	EU556528.1	1039,52	83,4%
Select seq gb|GU381522.1|	Mentha cervina voucher M:Braeuchler 2394 tRNA-Leu (trnL) gene and trnL-trnF intergenic spacer, partial sequence; chloroplast	1062	1062	98% 0.0	95%	GU381522.1	1029,49	82,6%
Select seq gb|EU556529.1|	Thymus dahuricus isolate HD tRNA-Leu (trnL) gene and trnL-trnF intergenic spacer, partial sequence; chloroplast	1062	1062	98% 0.0	95%	EU556529.1	1029,49	82,6%
Select seq gb|GU381630.1|	Satureja thymbrifolia voucher M:Danin s.n. tRNA-Leu (trnL) gene and trnL-trnF intergenic spacer, partial sequence; chloroplast	1057	1057	98% 0.0	95%	GU381630.1	1024,64	82,2%
Select seq gb|DQ667492.1|	Lepechinia lancifolia isolate x232 tRNA-Leu (trnL) gene and trnL-trnF intergenic spacer, partial sequence; chloroplast	1077	1077	100% 0.0	95%	DQ667492.1	1023,15	82,1%
Select seq gb|KC414276.1|	Mentha canadensis isolate 511190001 tRNA-Leu (trnL) gene and trnL-trnF intergenic spacer, partial sequence; chloroplast	1074	1074	100% 0.0	95%	KC414276.1	1020,30	81,8%
Select seq emb|AJ505541.1|	Mentha suaveolens plastid trnL-trnF intergenic spacer, specimen voucher cult., K-1970-3169 (K)	1074	1074	100% 0.0	95%	AJ505541.1	1020,30	81,8%
Select seq gb|KR150233.1|	Thymus sp. 6 Kh133 trnL-trnF intergenic spacer region, partial sequence; chloroplast	1062	1062	99% 0.0	95%	KR150233.1	1019,09	81,8%
Select seq gb|EU556537.1|	Thymus quinquecostatus isolate XZ tRNA-Leu (trnL) gene and trnL-trnF intergenic spacer, partial sequence; chloroplast	1070	1070	100% 0.0	95%	EU556537.1	1016,50	81,5%

Select for downloading or viewing reports	Kh071_ITS Description	Max score	Total score	Query cover	E value	Ident	Accession	(Ident/Cover)* Max score	Deviation from top hit
Select seq gb|GQ379304.1|	Bunium capillifolium voucher Kamelin 1564 6-VII-1974 (LE) internal transcribed spacer 1, 5.8S ribosomal RNA gene, and internal transcribed spacer 2, complete sequence	835	835	79%	0.0	95%	GQ379304.1	1004,11	100,0%
Select seq gb|HM229365.1|	Elaeosticta knorringiana voucher M.G. Pimenov et al. 378 (MW) internal transcribed spacer 1, 5.8S ribosomal RNA gene, and internal transcribed spacer 2, complete sequence	782	782	79%	0.0	93%	HM229365.1	920,58	91,7%
Select seq gb|HM229357.1|	Elaeosticta conica voucher M.G. Pimenov et al. 426 (MW) internal transcribed spacer 1, 5.8S ribosomal RNA gene, and internal transcribed spacer 2, complete sequence	789	789	80%	0.0	93%	HM229357.1	917,21	91,3%
Select seq gb|HM229388.1|	Elaeosticta vvedenskyi voucher M.G. Pimenov et al. 749 (MW) internal transcribed spacer 1, 5.8S ribosomal RNA gene, and internal transcribed spacer 2, complete sequence	784	784	80%	0.0	93%	HM229388.1	911,40	90,8%
Select seq gb|HM229386.1|	Elaeosticta tschimganica voucher E.V. Kljuykov 29 (MW) internal transcribed spacer 1, 5.8S ribosomal RNA gene, and internal transcribed spacer 2, complete sequence	784	784	80%	0.0	93%	HM229386.1	911,40	90,8%
Select seq gb|HM229375.1|	Elaeosticta polycarpa voucher E.V. Kljuykov & M.G. Pimenov 561 (MW) internal transcribed spacer 1, 5.8S ribosomal RNA gene, and internal transcribed spacer 2, complete sequence	769	769	79%	0.0	93%	HM229375.1	905,28	90,2%
Select seq gb|HM229392.1|	Galagania platypoda voucher M.G. Pimenov et al. 440 (MW) internal transcribed spacer 1, 5.8S ribosomal RNA gene, and internal transcribed spacer 2, complete sequence	774	774	80%	0.0	93%	HM229392.1	899,78	89,6%
Select seq gb|KC784688.1|	Schrenkia vaginata voucher MW 74 internal transcribed spacer 1, 5.8S ribosomal RNA gene, and internal transcribed spacer 2, complete sequence	767	767	80%	0.0	93%	KC784688.1	891,64	88,8%
Select seq gb|KC784684.1|	Schrenkia papillaris voucher LE sn internal transcribed spacer 1, 5.8S ribosomal RNA gene, and internal transcribed spacer 2, complete sequence	767	767	80%	0.0	93%	KC784684.1	891,64	88,8%
Select seq gb|KC784673.1|	Schrenkia fasciculata voucher MW 708 internal transcribed spacer 1, 5.8S ribosomal RNA gene, and internal transcribed spacer 2, complete sequence	767	767	80%	0.0	93%	KC784673.1	891,64	88,8%
Select seq gb|HM229398.1|	Hyalolaena melanorrhiza voucher M.G. Pimenov et al. 1004 (MW) internal transcribed spacer 1, 5.8S ribosomal RNA gene, and internal transcribed spacer 2, complete sequence	800	800	84%	0.0	92%	HM229398.1	876,19	87,3%
Select seq gb|HM229373.1|	Elaeosticta platyphylla voucher M.G. Pimenov et al. 285 (MW) internal transcribed spacer 1, 5.8S ribosomal RNA gene, and internal transcribed spacer 2, complete sequence	800	800	84%	0.0	92%	HM229373.1	876,19	87,3%
Select seq gb|KC784696.1|	Schrenkia vaginata voucher MW sn internal transcribed spacer 1, 5.8S ribosomal RNA gene, and internal transcribed spacer 2, complete sequence	761	761	80%	0.0	92%	KC784696.1	875,15	87,2%
Select seq gb|HM229397.1|	Hyalolaena lipskyi voucher M.G. Pimenov et al. 316 (MW) internal transcribed spacer 1, 5.8S ribosomal RNA gene, and internal transcribed spacer 2, complete sequence	756	756	80%	0.0	92%	HM229397.1	869,40	86,6%
Select seq gb|GQ379313.1|	Ormopterum turcomanicum voucher Kamelin et al. 597 28-V-1982 (LE) internal transcribed spacer 1, 5.8S ribosomal RNA gene, and internal transcribed spacer 2, complete sequence	750	750	80%	0.0	92%	GQ379313.1	862,50	85,9%
Select seq gb|DQ516358.1|	Gongylotaxis rechingeri isolate K18 internal transcribed spacer 1, 5.8S ribosomal RNA gene, and internal transcribed spacer 2, complete sequence	750	750	80%	0.0	92%	DQ516358.1	862,50	85,9%
Select seq gb|DQ516363.1|	Pimpinella siifolia isolate K171 internal transcribed spacer 1, 5.8S ribosomal RNA gene, and internal transcribed spacer 2, complete sequence	745	745	80%	0.0	92%	DQ516363.1	856,75	85,3%
Select seq emb|HE602464.1|	Bunium bulbocastanum genomic DNA containing ITS1, 5.8S rRNA gene, ITS2, specimen voucher Montserrat, JM. s.n	754	754	81%	0.0	92%	HE602464.1	856,40	85,3%
Select seq emb|HE602463.1|	Bunium incrassatum genomic DNA containing ITS1, 5.8S rRNA gene, ITS2, specimen voucher Davis. 52337	754	754	81%	0.0	92%	HE602463.1	856,40	85,3%
Select seq emb|HE602447.1|	Bunium alpinum genomic DNA containing ITS1, 5.8S rRNA gene, ITS2, specimen voucher OPTIMA ITER V. 1064	754	754	81%	0.0	92%	HE602447.1	856,40	85,3%
Select seq gb|HM229390.1|	Galagania margiana voucher M.G. Pimenov & E.V. Kljuykov 267 (MW) internal transcribed spacer 1, 5.8S ribosomal RNA gene, and internal transcribed spacer 2, complete sequence	778	778	84%	0.0	92%	HM229390.1	852,10	84,9%
Select seq gb|AF008631.2|	Pyramidoptera cabulica internal transcribed spacer 1, 5.8S ribosomal RNA gene, and internal transcribed spacer 2, complete sequence	808	808	87%	0.0	91%	AF008631.2	845,15	84,2%
Select seq emb|HE602462.1|	Bunium pachypodum genomic DNA containing ITS1, 5.8S rRNA gene, ITS2, specimen voucher Montserrat, JM. 5835/3	721	721	78%	0.0	91%	HE602462.1	841,17	83,8%
Select seq gb|HM229354.1|	Elaeosticta allioides voucher M.G. Pimenov & E.V. Kljuykov 622 (MW) internal transcribed spacer 1, 5.8S ribosomal RNA gene, and internal transcribed spacer 2, complete sequence	802	802	87%	0.0	91%	HM229354.1	838,87	83,5%
Select seq emb|HE687357.1|	Bunium sp. EM410 genomic DNA containing ITS1, 5.8S rRNA gene, ITS2, specimen voucher EM410	719	719	78%	0.0	91%	HE687357.1	838,83	83,5%
Select seq gb|HM229360.1|	Elaeosticta ferganensis voucher E.V. Kljuykov & Ju.V. Baranova 1179 (MW) internal transcribed spacer 1, 5.8S ribosomal RNA gene, and internal transcribed spacer 2, complete sequence	765	765	83%	0.0	91%	HM229360.1	838,73	83,5%

Select seq gb|HM229403.1|	Oedibasis tamerlanii voucher M.G. Pimenov & E.V. Kljuykov 48 (MW) internal transcribed spacer 1, 5.8S ribosomal RNA gene, and internal transcribed spacer 2, complete sequence	800	800	87%	0.0	91%	HM229403.1	836,78	83,3%
Select seq gb|DQ516359.1|	Muretia lutea isolate K69 internal transcribed spacer 1, 5.8S ribosomal RNA gene, and internal transcribed spacer 2, complete sequence	800	800	87%	0.0	91%	DQ516359.1	836,78	83,3%
Select seq gb|HM229361.1|	Elaeosticta ferganensis voucher E.V. Kljuykov et al. 404 (MW) internal transcribed spacer 1, 5.8S ribosomal RNA gene, and internal transcribed spacer 2, complete sequence	763	763	83%	0.0	91%	HM229361.1	836,54	83,3%
Select seq gb|GQ379305.1|	Hyalolaena bupleuroides voucher Ikonnikov & Ladygina 9856 15-VIII-1972 (LE) internal transcribed spacer 1, 5.8S ribosomal RNA gene, and internal transcribed spacer 2, complete sequence	763	763	84%	0.0	91%	GQ379305.1	826,58	82,3%
Select seq gb|KF974537.1|	Hellenocarum multiflorum voucher MW<RUS>:Zakharova & Ukrainskaya 2 internal transcribed spacer 1, 5.8S ribosomal RNA gene, and internal transcribed spacer 2, complete sequence	717	717	79%	0.0	91%	KF974537.1	825,91	82,3%
Select seq gb|KF974534.1|	Hellenocarum strictum voucher MHA:Greuter 16058 internal transcribed spacer 1, 5.8S ribosomal RNA gene, and internal transcribed spacer 2, complete sequence	717	717	79%	0.0	91%	KF974534.1	825,91	82,3%
Select seq gb|HM229362.1|	Elaeosticta glaucescens voucher M.G. Pimenov et al. 348 (MW) internal transcribed spacer 1, 5.8S ribosomal RNA gene, and internal transcribed spacer 2, complete sequence	789	789	87%	0.0	91%	HM229362.1	825,28	82,2%
Select seq gb|KF974533.1|	Carum depressum voucher C:Kit Tan & Strid 20416 internal transcribed spacer 1, 5.8S ribosomal RNA gene, and internal transcribed spacer 2, complete sequence	725	725	80%	0.0	91%	KF974533.1	824,69	82,1%
Select seq gb|GQ379316.1|	Schulzia albiflora voucher Sapozhnikov s.n. 24-VII-1913 (LE) internal transcribed spacer 1, 5.8S ribosomal RNA gene, and internal transcribed spacer 2, complete sequence	787	787	87%	0.0	91%	GQ379316.1	823,18	82,0%
Select seq gb|JQ792227.1|	Tamamschjanella cruciata voucher Sintenis 7255 (LD) internal transcribed spacer 1, 5.8S ribosomal RNA gene, and internal transcribed spacer 2, complete sequence	723	723	80%	0.0	91%	JQ792227.1	822,41	81,9%
Select seq gb|KC784687.1|	Schrenkia ugamica voucher MW 1649 internal transcribed spacer 1, 5.8S ribosomal RNA gene, and internal transcribed spacer 2, complete sequence	785	785	87%	0.0	91%	KC784687.1	821,09	81,8%
Select seq gb|KC784675.1|	Schrenkia golickeana voucher MW 112a internal transcribed spacer 1, 5.8S ribosomal RNA gene, and internal transcribed spacer 2, complete sequence	785	785	87%	0.0	91%	KC784675.1	821,09	81,8%
Select seq gb|HM229356.1|	Elaeosticta bucharica voucher M.G. Pimenov & E.V. Kljuykov 409 (MW) internal transcribed spacer 1, 5.8S ribosomal RNA gene, and internal transcribed spacer 2, complete sequence	785	785	87%	0.0	91%	HM229356.1	821,09	81,8%
Select seq gb|HM229395.1|	Hyalolaena intermedia voucher M.G. Pimenov 828 (MW) internal transcribed spacer 1, 5.8S ribosomal RNA gene, and internal transcribed spacer 2, complete sequence	784	784	87%	0.0	91%	HM229395.1	820,05	81,7%
Select seq gb|HM229359.1|	Elaeosticta meifolia voucher 54987 (TARI) internal transcribed spacer 1, 5.8S ribosomal RNA gene, and internal transcribed spacer 2, complete sequence	784	784	87%	0.0	91%	HM229359.1	820,05	81,7%
Select seq gb|HM229367.1|	Elaeosticta korovinii voucher M.G. Pimenov & E.V. Kljuykov 129 (MW) internal transcribed spacer 1, 5.8S ribosomal RNA gene, and internal transcribed spacer 2, complete sequence	784	784	87%	0.0	91%	HM229367.1	820,05	81,7%
Select seq gb|HM229394.1|	Hyalolaena intermedia voucher M.G. Pimenov et al. K96-123 (MW) internal transcribed spacer 1, 5.8S ribosomal RNA gene, and internal transcribed spacer 2, complete sequence	782	782	87%	0.0	91%	HM229394.1	817,95	81,5%
Select seq gb|HM229389.1|	Galagania gracilis voucher M.G. Pimenov & E.V. Kljuykov 190 (MW) internal transcribed spacer 1, 5.8S ribosomal RNA gene, and internal transcribed spacer 2, complete sequence	782	782	87%	0.0	91%	HM229389.1	817,95	81,5%
Select seq gb|GQ379310.1|	Indoschulzia hameliana voucher Farille 78-79 26-X-1978 (G) internal transcribed spacer 1, 5.8S ribosomal RNA gene, and internal transcribed spacer 2, complete sequence	782	782	87%	0.0	91%	GQ379310.1	817,95	81,5%
Select seq gb|KC784685.1|	Schrenkia pulverulenta voucher MW K96-229 internal transcribed spacer 1, 5.8S ribosomal RNA gene, and internal transcribed spacer 2, complete sequence	780	780	87%	0.0	91%	KC784685.1	815,86	81,3%
Select seq gb|KC784681.1|	Schrenkia kultiassovii voucher MW 330 internal transcribed spacer 1, 5.8S ribosomal RNA gene, and internal transcribed spacer 2, complete sequence	780	780	87%	0.0	91%	KC784681.1	815,86	81,3%
Select seq gb|KC784677.1|	Schrenkia golickeana voucher MW 135 internal transcribed spacer 1, 5.8S ribosomal RNA gene, and internal transcribed spacer 2, complete sequence	780	780	87%	0.0	91%	KC784677.1	815,86	81,3%
Select seq gb|HM229368.1|	Elaeosticta lutea voucher Ju.V. Baranova & T.V. Lavrova 1037 (MW) internal transcribed spacer 1, 5.8S ribosomal RNA gene, and internal transcribed spacer 2, complete sequence	778	778	87%	0.0	91%	HM229368.1	813,77	81,0%
Select seq gb|GQ379315.1|	Schtschurowskia margaritae voucher Kamelin 1325 17-VI-1974 (LE) internal transcribed spacer 1, 5.8S ribosomal RNA gene, and internal transcribed spacer 2, complete sequence	776	776	87%	0.0	90%	GQ379315.1	802,76	79,9%
Select seq gb|KC784692.1|	Schrenkia vaginata voucher LE sn internal transcribed spacer 1, 5.8S ribosomal RNA gene, and internal transcribed spacer 2, complete sequence	774	774	87%	0.0	90%	KC784692.1	800,69	79,7%
Select seq gb|KC784676.1|	Schrenkia golickeana voucher MW 1214 internal transcribed spacer 1, 5.8S ribosomal RNA gene, and internal transcribed spacer 2, complete sequence	774	774	87%	0.0	90%	KC784676.1	800,69	79,7%
Select seq gb|GQ379311.1|	Kosopoljanskia turkestanica voucher Kamelin s.n. 5-VII-1973 (LE) internal transcribed spacer 1, 5.8S ribosomal RNA gene, and internal transcribed spacer 2, complete sequence	774	774	87%	0.0	90%	GQ379311.1	800,69	79,7%
Select seq gb|HM229401.1|	Mogoltavia narynensis voucher Aidarova et al. s.n. (MW) internal transcribed spacer 1, 5.8S ribosomal RNA gene, and internal transcribed spacer 2, complete sequence	773	773	87%	0.0	90%	HM229401.1	799,66	79,6%

Select seq gb|HM229391.1|	Galagania neglecta voucher M.G. Pimenov et al. 320 (MW) internal transcribed spacer 1, 5.8S ribosomal RNA gene, and internal transcribed spacer 2, complete sequence	771	771	87% 0.0	90% HM229391.1	797,59	79,4%
Select seq gb|KC784690.1|	Schrenkia vaginata voucher MW K96-154 internal transcribed spacer 1, 5.8S ribosomal RNA gene, and internal transcribed spacer 2, complete sequence	769	769	87% 0.0	90% KC784690.1	795,52	79,2%
Select seq gb|KC784682.1|	Schrenkia sp. 'minkwitzae' voucher LE sn internal transcribed spacer 1, 5.8S ribosomal RNA gene, and internal transcribed spacer 2, complete sequence	769	769	87% 0.0	90% KC784682.1	795,52	79,2%
Select seq gb|HM229402.1|	Oedibasis apiculata voucher M.G. Pimenov et al. 166 (MW) internal transcribed spacer 1, 5.8S ribosomal RNA gene, and internal transcribed spacer 2, complete sequence	769	769	87% 0.0	90% HM229402.1	795,52	79,2%
Select seq gb|FJ385059.1|	Schulzia albiflora voucher J106 (XAU) internal transcribed spacer 1, partial sequence; 5.8S ribosomal RNA gene, complete sequence; and internal transcribed spacer 2, partial sequence	769	769	87% 0.0	90% FJ385059.1	795,52	79,2%
Select seq emb|HE985079.1|	Scaligeria alziarii genomic DNA containing ITS1, 5.8S rRNA gene and ITS2, specimen voucher B:GT 0003944	697	697	79% 0.0	90% HE985079.1	794,05	79,1%
Select seq gb|JQ792222.1|	Carum heldreichii voucher Franzen 229 (LD) internal transcribed spacer 1, 5.8S ribosomal RNA gene, and internal transcribed spacer 2, complete sequence	695	695	79% 0.0	90% JQ792222.1	791,77	78,9%
Select seq gb|HM229396.1|	Hyalolaena issykkulensis voucher M.G. Pimenov & E.V. Kljuykov 907 (MW) internal transcribed spacer 1, 5.8S ribosomal RNA gene, and internal transcribed spacer 2, complete sequence	765	765	87% 0.0	90% HM229396.1	791,38	78,8%
Select seq gb|KF974536.1|	Hellenocarum pisidicum voucher E:Ayasligil 1378B internal transcribed spacer 1, 5.8S ribosomal RNA gene, and internal transcribed spacer 2, complete sequence	702	702	80% 0.0	90% KF974536.1	789,75	78,7%
Select seq gb|KF974535.1|	Hellenocarum amplifolium voucher TARI<IRN>:62101 internal transcribed spacer 1, 5.8S ribosomal RNA gene, and internal transcribed spacer 2, complete sequence	702	702	80% 0.0	90% KF974535.1	789,75	78,7%
Select seq gb|KC784695.1|	Schrenkia vaginata voucher MW 276 internal transcribed spacer 1, 5.8S ribosomal RNA gene, and internal transcribed spacer 2, complete sequence	763	763	87% 0.0	90% KC784695.1	789,31	78,6%
Select seq gb|KC784691.1|	Schrenkia vaginata voucher MW 270 internal transcribed spacer 1, 5.8S ribosomal RNA gene, and internal transcribed spacer 2, complete sequence	763	763	87% 0.0	90% KC784691.1	789,31	78,6%
Select seq gb|HM229406.1|	Schulzia prostrata voucher M.G. Pimenov & E.V. Kljuykov 790 (MW) internal transcribed spacer 1, 5.8S ribosomal RNA gene, and internal transcribed spacer 2, complete sequence	763	763	87% 0.0	90% HM229406.1	789,31	78,6%
Select seq gb|GQ379312.1|	Kosopoljanskia turkestanica voucher Kamelin 762 21-VII-1970 (LE) internal transcribed spacer 1, 5.8S ribosomal RNA gene, and internal transcribed spacer 2, complete sequence	763	763	87% 0.0	90% GQ379312.1	789,31	78,6%
Select seq gb|KC784668.1|	Kosopoljanskia turkestanica voucher MW 249 internal transcribed spacer 1, 5.8S ribosomal RNA gene, and internal transcribed spacer 2, complete sequence	761	761	87% 0.0	90% KC784668.1	787,24	78,4%
Select seq gb|HM229350.1|	Elaeosticta aitchisonii voucher Ju. Gubanov et al. 444a (MW) internal transcribed spacer 1, 5.8S ribosomal RNA gene, and internal transcribed spacer 2, complete sequence	761	761	87% 0.0	90% HM229350.1	787,24	78,4%
Select seq gb|HM229381.1|	Elaeosticta transcaspica voucher E.V. Kljuykov & Ju.V. Baranova 595 (MW) internal transcribed spacer 1, 5.8S ribosomal RNA gene, and internal transcribed spacer 2, complete sequence	758	758	87% 0.0	90% HM229381.1	784,14	78,1%
Select seq gb|GQ379307.1|	Hyalolaena jaxartica voucher Botchantsev & Takhtajan 23 20-VI-1964 (LE) internal transcribed spacer 1, 5.8S ribosomal RNA gene, and internal transcribed spacer 2, complete sequence	758	758	87% 0.0	90% GQ379307.1	784,14	78,1%
Select seq gb|KF974538.1|	Bunium elegans voucher MW<RUS>:Pimenov et al. 353 internal transcribed spacer 1, 5.8S ribosomal RNA gene, and internal transcribed spacer 2, complete sequence	756	756	87% 0.0	90% KF974538.1	782,07	77,9%
Select seq gb|HM229363.1|	Elaeosticta hirtula voucher I. Hedge & P. Wendelbo W9358 (MW) internal transcribed spacer 1, 5.8S ribosomal RNA gene, and internal transcribed spacer 2, complete sequence	756	756	87% 0.0	90% HM229363.1	782,07	77,9%
Select seq gb|JQ792218.1|	Carum heldreichii voucher Hartvig et al. 10471 (C) internal transcribed spacer 1, 5.8S ribosomal RNA gene, and internal transcribed spacer 2, complete sequence	684	684	79% 0.0	90% JQ792218.1	779,24	77,6%
Select seq gb|JQ792213.1|	Carum furcatum voucher Smith 5838 (UPS) internal transcribed spacer 1, 5.8S ribosomal RNA gene, and internal transcribed spacer 2, complete sequence	752	752	87% 0.0	90% JQ792213.1	777,93	77,5%
Select seq gb|HM229351.1|	Elaeosticta alaica voucher M.G. Pimenov et al. 71 (MW) internal transcribed spacer 1, 5.8S ribosomal RNA gene, and internal transcribed spacer 2, complete sequence	752	752	87% 0.0	90% HM229351.1	777,93	77,5%
Select seq gb|GQ379309.1|	Hyalolaena jaxartica voucher Novikova s.n. 17-V-1951 (LE) internal transcribed spacer 1, 5.8S ribosomal RNA gene, and internal transcribed spacer 2, complete sequence	752	752	87% 0.0	90% GQ379309.1	777,93	77,5%
Select seq gb|JQ792206.1|	Carum buriaticum voucher Pimenov & Vassilieva 97 (MW) internal transcribed spacer 1, 5.8S ribosomal RNA gene, and internal transcribed spacer 2, complete sequence	750	750	87% 0.0	90% JQ792206.1	775,86	77,3%
Select seq gb|KC784669.1|	Schrenkia sp. ET-2014 voucher MW 503 internal transcribed spacer 1, 5.8S ribosomal RNA gene, and internal transcribed spacer 2, complete sequence	747	747	87% 0.0	90% KC784669.1	772,76	77,0%
Select seq gb|KC784693.1|	Schrenkia vaginata voucher MW 177 internal transcribed spacer 1, 5.8S ribosomal RNA gene, and internal transcribed spacer 2, complete sequence	745	745	87% 0.0	90% KC784693.1	770,69	76,8%
Select seq gb|DQ516361.1|	Oreoschimperella verrucosa isolate K129 internal transcribed spacer 1, 5.8S ribosomal RNA gene, and internal transcribed spacer 2, complete sequence	719	719	84% 0.0	90% DQ516361.1	770,36	76,7%

Select seq gb|KJ473899.1|	Sison segetum internal transcribed spacer 1, partial sequence; 5.8S ribosomal RNA gene, complete sequence; and internal transcribed spacer 2, partial sequence	693	693	81%	0.0	90%	KJ473899.1	770,00	76,7%
Select seq gb|HM229405.1|	Scaligeria moreana voucher H. Runemark & S. Snogerup 20727 (LD) internal transcribed spacer 1, 5.8S ribosomal RNA gene, and internal transcribed spacer 2, complete sequence	704	704	83%	0.0	90%	HM229405.1	763,37	76,0%
Select seq gb|GQ379314.1|	Schtschurowskia margaritae voucher Mikeshin 453 19-VI-1937 (LE) internal transcribed spacer 1, 5.8S ribosomal RNA gene, and internal transcribed spacer 2, complete sequence	741	741	87%	0.0	89%	GQ379314.1	758,03	75,5%
Select seq gb|JX312805.1|	Bunium allioides voucher Bani 6580 internal transcribed spacer 1, 5.8S ribosomal RNA gene, and internal transcribed spacer 2, complete sequence	739	739	87%	0.0	89%	JX312805.1	755,99	75,3%
Select seq gb|HM229404.1|	Scaligeria halophila voucher B. Bentzer & H. Runemark 28236 (LD) internal transcribed spacer 1, 5.8S ribosomal RNA gene, and internal transcribed spacer 2, complete sequence	708	708	84%	0.0	89%	HM229404.1	750,14	74,7%
Select seq gb|EU169246.1|	Astomaea seselifolia voucher Danin & al. 8-80 (RNG) internal transcribed spacer 1, partial sequence; 5.8S ribosomal RNA gene, complete sequence; and internal transcribed spacer 2, partial sequence	730	730	87%	0.0	89%	EU169246.1	746,78	74,4%
Select seq gb|JQ792214.1|	Carum graecum subsp. serpentinicum voucher Hegenenn et al. s.n. (LD) internal transcribed spacer 1, 5.8S ribosomal RNA gene, and internal transcribed spacer 2, complete sequence	684	684	83%	0.0	89%	JQ792214.1	733,45	73,0%
Select seq gb|HM229364.1|	Elaeosticta hirtula voucher M.G. Pimenov et al. 587 (MW) internal transcribed spacer 1, 5.8S ribosomal RNA gene, and internal transcribed spacer 2, complete sequence	721	721	87%	0.0	88%	HM229364.1	729,29	72,6%
Select seq gb|KJ999478.1|	Conioselinum vaginatum isolate XJ1 18S ribosomal RNA gene, partial sequence; internal transcribed spacer 1 and 5.8S ribosomal RNA gene, complete sequence; and internal transcribed spacer 2, partial sequence	682	682	85%	0.0	88%	KJ999478.1	706,07	70,3%
Select seq gb|HQ202139.1|	Ammi seubertianum voucher M. Carine et al. 121 (AZB, BM) clone 4 internal transcribed spacer 1, partial sequence; 5.8S ribosomal RNA gene, complete sequence; and internal transcribed spacer 2, partial sequence	689	689	86%	0.0	88%	HQ202139.1	705,02	70,2%
Select seq gb|HQ202137.1|	Ammi seubertianum voucher M. Carine et al. 5 (AZB, BM) clone 1 internal transcribed spacer 1, partial sequence; 5.8S ribosomal RNA gene, complete sequence; and internal transcribed spacer 2, partial sequence	689	689	86%	0.0	88%	HQ202137.1	705,02	70,2%
Select seq gb|JQ230973.1|	Ammi majus voucher SBB-1158 internal transcribed spacer 1, partial sequence; 5.8S ribosomal RNA gene and internal transcribed spacer 2, complete sequence; and 28S ribosomal RNA gene, partial sequence	684	684	86%	0.0	88%	JQ230973.1	699,91	69,7%
Select seq gb|HQ202136.1|	Ammi seubertianum voucher H. Schaefer 2008-668 (BM) internal transcribed spacer 1, partial sequence; 5.8S ribosomal RNA gene, complete sequence; and internal transcribed spacer 2, partial sequence	684	684	86%	0.0	88%	HQ202136.1	699,91	69,7%
Select seq gb|HM229377.1|	Elaeosticta samarkandica voucher M.G. Pimenov & E.V. Kljuykov 252 (MW) internal transcribed spacer 1, 5.8S ribosomal RNA gene, and internal transcribed spacer 2, complete sequence	383	383	38%	3,00E-102	94%	HM229377.1	0,00	0,0%
								0,00	0,0%
								0,00	0,0%
								0,00	0,0%

Select for downloading or viewing reports	Kh071_trnL Description	Max score	Total score	Query cover	E value	Ident	Accession	(Ident/Cover)* Max score	Deviation from top hit
Select seq gb|HM596072.1|	Crithmum maritimum chloroplast, complete genome	1256	1256	100%	0.0	97%	HM596072.1	1218,32	100,0%
Select seq gb|EU001351.1|	Heracleum millefolium clone trn-yy16 tRNA-Leu (trnL) gene, partial sequence; and trnL-trnF intergenic spacer, complete sequence; chloroplast	1212	1212	97%	0.0	97%	EU001351.1	1212,00	99,5%
Select seq dbj|LC072694.1|	Peucedanum praeruptorum chloroplast DNA, trnL-trnF intergenic spacer, partial sequence, isolate: LJJ15	1210	1210	97%	0.0	97%	LC072694.1	1210,00	99,3%
Select seq gb|FJ475189.1|	Hymenidium brunonis tRNA-Leu (trnL) gene and trnL-trnF intergenic spacer, partial sequence; chloroplast	1206	1206	97%	0.0	97%	FJ475189.1	1206,00	99,0%
Select seq gb|KP665036.1|	Apiaceae sp. 13R31re1 tRNA-Leu (trnL) gene and trnL-trnF intergenic spacer, partial sequence; chloroplast	1192	1192	96%	0.0	97%	KP665036.1	1204,42	98,9%
Select seq gb|KP665035.1|	Apiaceae sp. 13R29re3 tRNA-Leu (trnL) gene and trnL-trnF intergenic spacer, partial sequence; chloroplast	1192	1192	96%	0.0	97%	KP665035.1	1204,42	98,9%
Select seq gb|AF444009.1|	Cymopterus globosus tRNA-Phe gene, partial sequence; tRNA-Leu gene, complete sequence; and tRNA-Thr gene, partial sequence; chloroplast genes for chloroplast products	1227	1227	100%	0.0	96%	AF444009.1	1177,92	96,7%
Select seq emb|AM109908.1|	Cnidium officinale plastid partial tRNA-Leu gene and partial IGS	1181	1181	97%	0.0	96%	AM109908.1	1168,82	95,9%
Select seq gb|KT963039.1|	Ligusticum tenuissimum voucher 13I-08 chloroplast, complete genome	1212	1212	100%	0.0	96%	KT963039.1	1163,52	95,5%
Select seq gb|AF444029.1|	Pteryxia terebinthina tRNA-Phe gene, partial sequence; and tRNA-Leu gene, complete sequence; chloroplast genes for chloroplast products	1212	1212	100%	0.0	96%	AF444029.1	1163,52	95,5%
Select seq gb|HQ246203.1|	Trachydium roylei tRNA-Leu (trnL) gene, partial sequence; trnL-trnF intergenic spacer, complete sequence; and tRNA-Phe (trnF) gene, partial sequence; chloroplast	1175	1175	97%	0.0	96%	HQ246203.1	1162,89	95,5%
Select seq gb|FJ475216.1|	Pterocyclus rivulorum tRNA-Leu (trnL) gene and trnL-trnF intergenic spacer, partial sequence; chloroplast	1175	1175	97%	0.0	96%	FJ475216.1	1162,89	95,5%
Select seq gb|HQ246204.1|	Trachydium subnudum tRNA-Leu (trnL) gene, partial sequence; trnL-trnF intergenic spacer, complete sequence; and tRNA-Phe (trnF) gene, partial sequence; chloroplast	1170	1170	97%	0.0	96%	HQ246204.1	1157,94	95,0%
Select seq gb|HQ246199.1|	Pseudotrachydium vesiculosoalatum tRNA-Leu (trnL) gene, partial sequence; trnL-trnF intergenic spacer, complete sequence; and tRNA-Phe (trnF) gene, partial sequence; chloroplast	1170	1170	97%	0.0	96%	HQ246199.1	1157,94	95,0%
Select seq gb|FJ475215.1|	Pterocyclus angelicoides tRNA-Leu (trnL) gene and trnL-trnF intergenic spacer, partial sequence; chloroplast	1170	1170	97%	0.0	96%	FJ475215.1	1157,94	95,0%
Select seq gb|FJ475186.1|	Hymenidium apiolens tRNA-Leu (trnL) gene and trnL-trnF intergenic spacer, partial sequence; chloroplast	1170	1170	97%	0.0	96%	FJ475186.1	1157,94	95,0%
Select seq gb|AF444020.1|	Lomatium junceum tRNA-Phe gene, partial sequence; tRNA-Leu gene, complete sequence; and tRNA-Thr gene, partial sequence; chloroplast genes for chloroplast products	1170	1170	97%	0.0	96%	AF444020.1	1157,94	95,0%
Select seq gb|KP665033.1|	Angelica sinensis isolate 13R17 tRNA-Leu (trnL) gene and trnL-trnF intergenic spacer, partial sequence; chloroplast	1155	1155	96%	0.0	96%	KP665033.1	1155,00	94,8%
Select seq gb|KP664978.1|	Angelica decursiva isolate 13Q18 tRNA-Leu (trnL) gene and trnL-trnF intergenic spacer, partial sequence; chloroplast	1155	1155	96%	0.0	96%	KP664978.1	1155,00	94,8%
Select seq gb|KP664977.1|	Angelica decursiva isolate 13Q16 tRNA-Leu (trnL) gene and trnL-trnF intergenic spacer, partial sequence; chloroplast	1155	1155	96%	0.0	96%	KP664977.1	1155,00	94,8%
Select seq gb|KP664976.1|	Angelica decursiva isolate 13Q15 tRNA-Leu (trnL) gene and trnL-trnF intergenic spacer, partial sequence; chloroplast	1155	1155	96%	0.0	96%	KP664976.1	1155,00	94,8%
Select seq gb|KP664975.1|	Angelica decursiva isolate 13Q13 tRNA-Leu (trnL) gene and trnL-trnF intergenic spacer, partial sequence; chloroplast	1155	1155	96%	0.0	96%	KP664975.1	1155,00	94,8%
Select seq gb|KP664974.1|	Angelica decursiva isolate 13Q12 tRNA-Leu (trnL) gene and trnL-trnF intergenic spacer, partial sequence; chloroplast	1155	1155	96%	0.0	96%	KP664974.1	1155,00	94,8%
Select seq gb|KP664973.1|	Angelica decursiva isolate 13Q11 tRNA-Leu (trnL) gene and trnL-trnF intergenic spacer, partial sequence; chloroplast	1155	1155	96%	0.0	96%	KP664973.1	1155,00	94,8%
Select seq gb|KP664972.1|	Angelica decursiva isolate 13Q10 tRNA-Leu (trnL) gene and trnL-trnF intergenic spacer, partial sequence; chloroplast	1155	1155	96%	0.0	96%	KP664972.1	1155,00	94,8%
Select seq gb|KP664971.1|	Angelica decursiva isolate 13Q09 tRNA-Leu (trnL) gene and trnL-trnF intergenic spacer, partial sequence; chloroplast	1155	1155	96%	0.0	96%	KP664971.1	1155,00	94,8%

Select seq gb|KP664970.1|	Angelica decursiva isolate 13Q08 tRNA-Leu (trnL) gene and trnL-trnF intergenic spacer, partial sequence; chloroplast	1155	1155	96% 0.0	96% KP664970.1	1155,00	94,8%
Select seq gb|KP664969.1|	Angelica decursiva isolate 13Q06 tRNA-Leu (trnL) gene and trnL-trnF intergenic spacer, partial sequence; chloroplast	1155	1155	96% 0.0	96% KP664969.1	1155,00	94,8%
Select seq gb|KP664968.1|	Angelica decursiva isolate 13Q05 tRNA-Leu (trnL) gene and trnL-trnF intergenic spacer, partial sequence; chloroplast	1155	1155	96% 0.0	96% KP664968.1	1155,00	94,8%
Select seq gb|KP664967.1|	Angelica decursiva isolate 13Q04 tRNA-Leu (trnL) gene and trnL-trnF intergenic spacer, partial sequence; chloroplast	1155	1155	96% 0.0	96% KP664967.1	1155,00	94,8%
Select seq gb|KP664966.1|	Angelica decursiva isolate 13Q03 tRNA-Leu (trnL) gene and trnL-trnF intergenic spacer, partial sequence; chloroplast	1155	1155	96% 0.0	96% KP664966.1	1155,00	94,8%
Select seq gb|KP664965.1|	Angelica decursiva isolate 13Q01 tRNA-Leu (trnL) gene and trnL-trnF intergenic spacer, partial sequence; chloroplast	1155	1155	96% 0.0	96% KP664965.1	1155,00	94,8%
Select seq gb|AF444012.1|	Cymopterus nivalis tRNA-Phe gene, partial sequence; tRNA-Leu gene, complete sequence; and tRNA-Thr gene, partial sequence; chloroplast genes for chloroplast products	1203	1203	100% 0.0	96% AF444012.1	1154,88	94,8%
Select seq gb|FJ475185.1|	Hymenidium amabile tRNA-Leu (trnL) gene and trnL-trnF intergenic spacer, partial sequence; chloroplast	1166	1166	97% 0.0	96% FJ475185.1	1153,98	94,7%
Select seq gb|AF444013.1|	Cymopterus purpureus tRNA-Phe gene, partial sequence; tRNA-Leu gene, complete sequence; and tRNA-Thr gene, partial sequence; chloroplast genes for chloroplast products	1201	1201	100% 0.0	96% AF444013.1	1152,96	94,6%
Select seq gb|FJ475206.1|	Hymenidium wilsonii tRNA-Leu (trnL) gene and trnL-trnF intergenic spacer, partial sequence; chloroplast	1188	1188	99% 0.0	96% FJ475206.1	1152,00	94,6%
Select seq gb|FJ475198.1|	Hymenidium huzhihaoi tRNA-Leu (trnL) gene and trnL-trnF intergenic spacer, partial sequence; chloroplast	1188	1188	99% 0.0	96% FJ475198.1	1152,00	94,6%
Select seq dbj|LC072690.1|	Libanotis buchtormensis chloroplast DNA, trnL-trnF intergenic spacer, partial sequence, isolate: LJJ11	1164	1164	97% 0.0	96% LC072690.1	1152,00	94,6%
Select seq gb|AF444011.1|	Cymopterus multinervatus tRNA-Phe gene, partial sequence; tRNA-Leu gene, complete sequence; and tRNA-Thr gene, partial sequence; chloroplast genes for chloroplast products	1199	1199	100% 0.0	96% AF444011.1	1151,04	94,5%
Select seq gb|KR011054.1|	Foeniculum vulgare chloroplast, complete genome	1197	1197	100% 0.0	96% KR011054.1	1149,12	94,3%
Select seq gb|GQ984062.1|	Coriandrum sativum tRNA-Leu (trnL) gene, partial sequence; trnL-trnF intergenic spacer, complete sequence; and tRNA-Phe (trnF) gene, partial sequence; chloroplast	1197	1197	100% 0.0	96% GQ984062.1	1149,12	94,3%
Select seq gb|AF444015.1|	Harbouria trachypleura tRNA-Phe gene, partial sequence; tRNA-Leu gene, complete sequence; and tRNA-Thr gene, partial sequence; chloroplast genes for chloroplast products	1194	1194	100% 0.0	96% AF444015.1	1146,24	94,1%
Select seq dbj|LC072693.1|	Libanotis spodotrichoma chloroplast DNA, trnL-trnF intergenic spacer, partial sequence, isolate: LJJ14	1158	1158	97% 0.0	96% LC072693.1	1146,06	94,1%
Select seq dbj|LC072692.1|	Libanotis buchtormensis chloroplast DNA, trnL-trnF intergenic spacer, partial sequence, isolate: LJJ13	1158	1158	97% 0.0	96% LC072692.1	1146,06	94,1%
Select seq gb|AF444019.1|	Lomatium graveolens tRNA-Phe gene, partial sequence; tRNA-Leu gene, complete sequence; and tRNA-Thr gene, partial sequence; chloroplast genes for chloroplast products	1192	1192	100% 0.0	96% AF444019.1	1144,32	93,9%
Select seq gb|KP665009.1|	Peucedanum praeruptorum isolate 13R37 tRNA-Leu (trnL) gene and trnL-trnF intergenic spacer, partial sequence; chloroplast	1149	1149	96% 0.0	95% KP665009.1	1137,03	93,3%
Select seq gb|KP665008.1|	Peucedanum praeruptorum isolate 13R36 tRNA-Leu (trnL) gene and trnL-trnF intergenic spacer, partial sequence; chloroplast	1149	1149	96% 0.0	95% KP665008.1	1137,03	93,3%
Select seq gb|KP665007.1|	Peucedanum praeruptorum isolate 13R35 tRNA-Leu (trnL) gene and trnL-trnF intergenic spacer, partial sequence; chloroplast	1149	1149	96% 0.0	95% KP665007.1	1137,03	93,3%
Select seq gb|KP665006.1|	Peucedanum praeruptorum isolate 13R34 tRNA-Leu (trnL) gene and trnL-trnF intergenic spacer, partial sequence; chloroplast	1149	1149	96% 0.0	95% KP665006.1	1137,03	93,3%
Select seq gb|KP665005.1|	Peucedanum praeruptorum isolate 13R31re2 tRNA-Leu (trnL) gene and trnL-trnF intergenic spacer, partial sequence; chloroplast	1149	1149	96% 0.0	95% KP665005.1	1137,03	93,3%
Select seq gb|KP665003.1|	Peucedanum praeruptorum isolate 13R28 tRNA-Leu (trnL) gene and trnL-trnF intergenic spacer, partial sequence; chloroplast	1149	1149	96% 0.0	95% KP665003.1	1137,03	93,3%
Select seq gb|KP665002.1|	Peucedanum praeruptorum isolate 13R25 tRNA-Leu (trnL) gene and trnL-trnF intergenic spacer, partial sequence; chloroplast	1149	1149	96% 0.0	95% KP665002.1	1137,03	93,3%
Select seq gb|KP665000.1|	Peucedanum praeruptorum isolate 13R23 tRNA-Leu (trnL) gene and trnL-trnF intergenic spacer, partial sequence; chloroplast	1149	1149	96% 0.0	95% KP665000.1	1137,03	93,3%
Select seq gb|KP664999.1|	Peucedanum praeruptorum isolate 13R22 tRNA-Leu (trnL) gene and trnL-trnF intergenic spacer, partial sequence; chloroplast	1149	1149	96% 0.0	95% KP664999.1	1137,03	93,3%

Select seq gb|KP664998.1|	Peucedanum praeruptorum isolate 13R21 tRNA-Leu (trnL) gene and trnL-trnF intergenic spacer, partial sequence; chloroplast	1149	1149	96%	0.0	95%	KP664998.1	1137,03	93,3%
Select seq gb|KP664997.1|	Peucedanum praeruptorum isolate 13R20 tRNA-Leu (trnL) gene and trnL-trnF intergenic spacer, partial sequence; chloroplast	1149	1149	96%	0.0	95%	KP664997.1	1137,03	93,3%
Select seq gb|KP664996.1|	Peucedanum praeruptorum isolate 13R19 tRNA-Leu (trnL) gene and trnL-trnF intergenic spacer, partial sequence; chloroplast	1149	1149	96%	0.0	95%	KP664996.1	1137,03	93,3%
Select seq gb|KP664995.1|	Peucedanum praeruptorum isolate 13R18 tRNA-Leu (trnL) gene and trnL-trnF intergenic spacer, partial sequence; chloroplast	1149	1149	96%	0.0	95%	KP664995.1	1137,03	93,3%
Select seq gb|KP664994.1|	Peucedanum praeruptorum isolate 13R15 tRNA-Leu (trnL) gene and trnL-trnF intergenic spacer, partial sequence; chloroplast	1149	1149	96%	0.0	95%	KP664994.1	1137,03	93,3%
Select seq gb|KP664993.1|	Peucedanum praeruptorum isolate 13R14 tRNA-Leu (trnL) gene and trnL-trnF intergenic spacer, partial sequence; chloroplast	1149	1149	96%	0.0	95%	KP664993.1	1137,03	93,3%
Select seq gb|KP664992.1|	Peucedanum praeruptorum isolate 13R13 tRNA-Leu (trnL) gene and trnL-trnF intergenic spacer, partial sequence; chloroplast	1149	1149	96%	0.0	95%	KP664992.1	1137,03	93,3%
Select seq gb|KP664991.1|	Peucedanum praeruptorum isolate 13R12 tRNA-Leu (trnL) gene and trnL-trnF intergenic spacer, partial sequence; chloroplast	1149	1149	96%	0.0	95%	KP664991.1	1137,03	93,3%
Select seq gb|KP664990.1|	Peucedanum praeruptorum isolate 13R11 tRNA-Leu (trnL) gene and trnL-trnF intergenic spacer, partial sequence; chloroplast	1149	1149	96%	0.0	95%	KP664990.1	1137,03	93,3%
Select seq gb|HQ246201.1|	Sinodielsia thibetica tRNA-Leu (trnL) gene and trnL-trnF intergenic spacer, complete sequence; and tRNA-Phe (trnF) gene, partial sequence; chloroplast	1158	1158	97%	0.0	95%	HQ246201.1	1134,12	93,1%
Select seq gb|EU076599.1|	Seseli mairei tRNA-Leu (trnL) gene and trnF-trnL intergenic spacer, partial sequence; chromoplast	1158	1158	97%	0.0	95%	EU076599.1	1134,12	93,1%
Select seq gb|EU076598.1|	Seseli squarrulosum tRNA-Leu (trnL) gene and trnF-trnL intergenic spacer, partial sequence; chromoplast	1157	1157	97%	0.0	95%	EU076598.1	1133,14	93,0%
Select seq gb|GQ351360.1|	Peucedanum japonicum tRNA-Leu (trnL) gene, partial sequence; trnL-trnF intergenic spacer, complete sequence; and tRNA-Phe (trnF) gene, partial sequence; chloroplast	1155	1155	97%	0.0	95%	GQ351360.1	1131,19	92,8%
Select seq gb|KT963036.1|	Angelica acutiloba voucher 13F-28 chloroplast, complete genome	1190	1190	100%	0.0	95%	KT963036.1	1130,50	92,8%
Select seq gb|FJ475191.1|	Hymenidium davidii tRNA-Leu (trnL) gene and trnL-trnF intergenic spacer, partial sequence; chloroplast	1153	1153	97%	0.0	95%	FJ475191.1	1129,23	92,7%
Select seq gb|FJ475182.1|	Aulacospermum simplex tRNA-Leu (trnL) gene and trnL-trnF intergenic spacer, partial sequence; chloroplast	1153	1153	97%	0.0	95%	FJ475182.1	1129,23	92,7%
Select seq gb|FJ475181.1|	Aulacospermum popovii tRNA-Leu (trnL) gene and trnL-trnF intergenic spacer, partial sequence; chloroplast	1153	1153	97%	0.0	95%	FJ475181.1	1129,23	92,7%
Select seq gb|FJ475180.1|	Aulacospermum anomalum tRNA-Leu (trnL) gene and trnL-trnF intergenic spacer, partial sequence; chloroplast	1153	1153	97%	0.0	95%	FJ475180.1	1129,23	92,7%
Select seq gb|EU001357.1|	Heracleum candicans clone trn-yy06 tRNA-Leu (trnL) gene, partial sequence; and trnL-trnF intergenic spacer, complete sequence; chloroplast	1151	1151	97%	0.0	95%	EU001357.1	1127,27	92,5%
Select seq gb|KR011055.1|	Anethum graveolens chloroplast, complete genome	1186	1186	100%	0.0	95%	KR011055.1	1126,70	92,5%
Select seq gb|AF444030.1|	Shoshonea pulvinata tRNA-Phe gene, partial sequence; tRNA-Leu gene, complete sequence; and tRNA-Thr gene, partial sequence; chloroplast genes for chloroplast products	1184	1184	100%	0.0	95%	AF444030.1	1124,80	92,3%
Select seq gb|AF444026.1|	Neoparrya lithophila tRNA-Phe gene, partial sequence; tRNA-Leu gene, complete sequence; and tRNA-Thr gene, partial sequence; chloroplast genes for chloroplast products	1184	1184	100%	0.0	95%	AF444026.1	1124,80	92,3%
Select seq gb|AF444025.1|	Musineon tenuifolium tRNA-Phe gene, partial sequence; tRNA-Leu gene, complete sequence; and tRNA-Thr gene, partial sequence; chloroplast genes for chloroplast products	1184	1184	100%	0.0	95%	AF444025.1	1124,80	92,3%
Select seq gb|AF444008.1|	Aletes anisatus tRNA-Phe gene, partial sequence; tRNA-Leu gene, complete sequence; and tRNA-Thr gene, partial sequence; chloroplast genes for chloroplast products	1184	1184	100%	0.0	95%	AF444008.1	1124,80	92,3%
Select seq gb|AF444018.1|	Lomatium dasycarpum tRNA-Phe gene, partial sequence; tRNA-Leu gene, complete sequence; and tRNA-Thr gene, partial sequence; chloroplast genes for chloroplast products	1181	1181	100%	0.0	95%	AF444018.1	1121,95	92,1%
Select seq gb|KT963038.1|	Angelica gigas voucher 13E-39-3 chloroplast, complete genome	1173	1173	100%	0.0	95%	KT963038.1	1114,35	91,5%
Select seq gb|KT963037.1|	Angelica dahurica voucher 13T-05 chloroplast, complete genome	1173	1173	100%	0.0	95%	KT963037.1	1114,35	91,5%
Select seq gb|KT852844.1|	Ostericum grosseserratum voucher 13A-14-2 chloroplast, complete genome	1173	1173	100%	0.0	95%	KT852844.1	1114,35	91,5%

Select seq gb|KT781591.1|	Angelica decursiva voucher 13Q-02-1 chloroplast, complete genome	1173	1173	100%	0.0	95%	KT781591.1	1114,35	91,5%
Select seq gb|AF444028.1|	Pteryxia hendersonii tRNA-Phe gene, partial sequence; tRNA-Leu gene, complete sequence; and tRNA-Thr gene, partial sequence; chloroplast genes for chloroplast products	1173	1173	100%	0.0	95%	AF444028.1	1114,35	91,5%
Select seq gb|AF444024.1|	Musineon divaricatum tRNA-Phe gene, partial sequence; tRNA-Leu gene, complete sequence; and tRNA-Thr gene, partial sequence; chloroplast genes for chloroplast products	1173	1173	100%	0.0	95%	AF444024.1	1114,35	91,5%
Select seq gb|AF444017.1|	Lomatium californicum tRNA-Phe gene, partial sequence; tRNA-Leu gene, complete sequence; and tRNA-Thr gene, partial sequence; chloroplast genes for chloroplast products	1173	1173	100%	0.0	95%	AF444017.1	1114,35	91,5%
Select seq gb|FJ475210.1|	Physospermopsis muktinathensis tRNA-Leu (trnL) gene and trnL-trnF intergenic spacer, partial sequence; chloroplast	1160	1160	99%	0.0	95%	FJ475210.1	1113,13	91,4%
Select seq emb|AJ430956.1|	Apium graveolens chloroplast tRNA-Leu gene for transfer RNA-Leu and partial tRNA-Phe gene for transfer RNA-Phe	1170	1170	100%	0.0	95%	AJ430956.1	1111,50	91,2%
Select seq gb|AF444023.1|	Lomatium nudicaule tRNA-Phe gene, partial sequence; tRNA-Leu gene, complete sequence; and tRNA-Thr gene, partial sequence; chloroplast genes for chloroplast products	1168	1168	100%	0.0	95%	AF444023.1	1109,60	91,1%
Select seq gb|AF444014.1|	Cymopterus williamsii tRNA-Phe gene, partial sequence; tRNA-Leu gene, complete sequence; and tRNA-Thr gene, partial sequence; chloroplast genes for chloroplast products	1168	1168	100%	0.0	95%	AF444014.1	1109,60	91,1%
Select seq gb|AF444007.1|	Angelica archangelica tRNA-Phe gene, partial sequence; tRNA-Leu gene, complete sequence; and tRNA-Thr gene, partial sequence; chloroplast genes for chloroplast products	1168	1168	100%	0.0	95%	AF444007.1	1109,60	91,1%
Select seq gb|KM035851.1|	Seseli montanum plastid, complete genome	1164	1164	100%	0.0	95%	KM035851.1	1105,80	90,8%
Select seq gb|AF444033.1|	Zizia aurea tRNA-Phe gene, partial sequence; tRNA-Leu gene, complete sequence; and tRNA-Thr gene, partial sequence; chloroplast genes for chloroplast products	1164	1164	100%	0.0	95%	AF444033.1	1105,80	90,8%
Select seq emb|AM109910.1|	Dystaenia ibukiensis plastid partial tRNA-Leu gene and partial IGS, isolate 7	1162	1162	100%	0.0	95%	AM109910.1	1103,90	90,6%
Select seq gb|AF444032.1|	Zizia aptera tRNA-Phe gene, partial sequence; tRNA-Leu gene, complete sequence; and tRNA-Thr gene, partial sequence; chloroplast genes for chloroplast products	1158	1158	100%	0.0	95%	AF444032.1	1100,10	90,3%
Select seq gb|AF444031.1|	Thaspium trifoliatum tRNA-Phe gene, partial sequence; and tRNA-Leu gene, complete sequence; chloroplast genes for chloroplast products	1158	1158	100%	0.0	95%	AF444031.1	1100,10	90,3%
Select seq gb|KM035850.1|	Pastinaca pimpinellifolia voucher M.G. Pimenov et al., 117 (MW) plastid, complete genome	1157	1157	100%	0.0	95%	KM035850.1	1099,15	90,2%
Select seq gb|AF444010.1|	Cymopterus montanus tRNA-Phe gene, partial sequence; tRNA-Leu gene, complete sequence; and tRNA-Thr gene, partial sequence; chloroplast genes for chloroplast products	1151	1151	100%	0.0	94%	AF444010.1	1081,94	88,8%
								0,00	0,0%

Select for downloading or viewing reports	Kh073_trnL Description	Max score	Total score	Query cover	E value	Ident	Accession	(Ident/Cover)* Max score	Deviation from top hit
Select seq gb|JQ044771.1|	Teucrium polium subsp. capitatum isolate TPC2 tRNA-Leu (trnL) gene, partial sequence; trnL-trnF intergenic spacer, complete sequence; and tRNA-Phe (trnF) gene, partial sequence; chloroplast	998	998	95%	0.0	97%	JQ044771.1	1019,01	100,0%
Select seq gb|JQ044773.1|	Teucrium polium subsp. capitatum isolate TPC1 tRNA-Leu (trnL) gene, partial sequence; trnL-trnF intergenic spacer, complete sequence; and tRNA-Phe (trnF) gene, partial sequence; chloroplast	992	992	95%	0.0	97%	JQ044773.1	1012,88	99,4%
Select seq gb|JQ044772.1|	Teucrium polium subsp. capitatum isolate TPC4 tRNA-Leu (trnL) gene, partial sequence; trnL-trnF intergenic spacer, complete sequence; and tRNA-Phe (trnF) gene, partial sequence; chloroplast	992	992	95%	0.0	97%	JQ044772.1	1012,88	99,4%
Select seq gb|JN408660.1|	Teucrium pyrenaicum tRNA-Leu (trnL) gene, partial sequence; and trnL-trnF intergenic spacer, complete sequence; chloroplast	1011	1011	98%	0.0	97%	JN408660.1	1000,68	98,2%
Select seq gb|JN408657.1|	Teucrium polium tRNA-Leu (trnL) gene, partial sequence; and trnL-trnF intergenic spacer, complete sequence; chloroplast	1011	1011	98%	0.0	97%	JN408657.1	1000,68	98,2%
Select seq gb|JN408646.1|	Teucrium montanum tRNA-Leu (trnL) gene, partial sequence; and trnL-trnF intergenic spacer, complete sequence; chloroplast	1011	1011	98%	0.0	97%	JN408646.1	1000,68	98,2%
Select seq gb|JN408625.1|	Teucrium capitatum tRNA-Leu (trnL) gene, partial sequence; and trnL-trnF intergenic spacer, complete sequence; chloroplast	1011	1011	98%	0.0	97%	JN408625.1	1000,68	98,2%
Select seq gb|JN408624.1|	Teucrium capitatum tRNA-Leu (trnL) gene, partial sequence; and trnL-trnF intergenic spacer, complete sequence; chloroplast	1011	1011	98%	0.0	97%	JN408624.1	1000,68	98,2%
Select seq gb|JN408623.1|	Teucrium aureum tRNA-Leu (trnL) gene, partial sequence; and trnL-trnF intergenic spacer, complete sequence; chloroplast	1011	1011	98%	0.0	97%	JN408623.1	1000,68	98,2%
Select seq gb|JN408621.1|	Teucrium pumilum tRNA-Leu (trnL) gene, partial sequence; and trnL-trnF intergenic spacer, complete sequence; chloroplast	1011	1011	98%	0.0	97%	JN408621.1	1000,68	98,2%
Select seq gb|JN408619.1|	Teucrium capitatum tRNA-Leu (trnL) gene, partial sequence; and trnL-trnF intergenic spacer, complete sequence; chloroplast	1011	1011	98%	0.0	97%	JN408619.1	1000,68	98,2%
Select seq gb|JN408616.1|	Teucrium cyprium subsp. cyprium tRNA-Leu (trnL) gene, partial sequence; and trnL-trnF intergenic spacer, complete sequence; chloroplast	1011	1011	98%	0.0	97%	JN408616.1	1000,68	98,2%
Select seq gb|JN408597.1|	Teucrium alpestre tRNA-Leu (trnL) gene, partial sequence; trnL-trnF intergenic spacer, complete sequence; and tRNA-Phe (trnF) gene, partial sequence; chloroplast	1011	1011	98%	0.0	97%	JN408597.1	1000,68	98,2%
Select seq gb|JQ044780.1|	Teucrium polium subsp. polium isolate TP12 tRNA-Leu (trnL) gene, partial sequence; trnL-trnF intergenic spacer, complete sequence; and tRNA-Phe (trnF) gene, partial sequence; chloroplast	1011	1011	98%	0.0	97%	JQ044780.1	1000,68	98,2%
Select seq gb|JQ044779.1|	Teucrium polium subsp. polium isolate TP11 tRNA-Leu (trnL) gene, partial sequence; trnL-trnF intergenic spacer, complete sequence; and tRNA-Phe (trnF) gene, partial sequence; chloroplast	1011	1011	98%	0.0	97%	JQ044779.1	1000,68	98,2%
Select seq gb|JQ044778.1|	Teucrium polium subsp. polium isolate TP10 tRNA-Leu (trnL) gene, partial sequence; trnL-trnF intergenic spacer, complete sequence; and tRNA-Phe (trnF) gene, partial sequence; chloroplast	1011	1011	98%	0.0	97%	JQ044778.1	1000,68	98,2%
Select seq gb|FJ952080.1|	Teucrium pyrenaicum tRNA-Leu (trnL) gene, partial sequence; trnL-trnF intergenic spacer, complete sequence; and tRNA-Phe (trnF) gene, partial sequence; chloroplast	1011	1011	98%	0.0	97%	FJ952080.1	1000,68	98,2%
Select seq gb|JQ044782.1|	Teucrium polium subsp. polium isolate TP13 tRNA-Leu (trnL) gene, partial sequence; trnL-trnF intergenic spacer, complete sequence; and tRNA-Phe (trnF) gene, partial sequence; chloroplast	1009	1009	98%	0.0	97%	JQ044782.1	998,70	98,0%
Select seq gb|JQ044781.1|	Teucrium polium subsp. polium isolate TP19 tRNA-Leu (trnL) gene, partial sequence; trnL-trnF intergenic spacer, complete sequence; and tRNA-Phe (trnF) gene, partial sequence; chloroplast	1009	1009	98%	0.0	97%	JQ044781.1	998,70	98,0%
Select seq gb|JN408620.1|	Teucrium aureum tRNA-Leu (trnL) gene, partial sequence; and trnL-trnF intergenic spacer, complete sequence; chloroplast	1005	1005	98%	0.0	97%	JN408620.1	994,74	97,6%
Select seq gb|JQ044776.1|	Teucrium polium subsp. polium isolate TP4 tRNA-Leu (trnL) gene, partial sequence; trnL-trnF intergenic spacer, complete sequence; and tRNA-Phe (trnF) gene, partial sequence; chloroplast	1005	1005	98%	0.0	97%	JQ044776.1	994,74	97,6%
Select seq gb|JQ044774.1|	Teucrium polium subsp. polium isolate TP20 tRNA-Leu (trnL) gene, partial sequence; trnL-trnF intergenic spacer, complete sequence; and tRNA-Phe (trnF) gene, partial sequence; chloroplast	1005	1005	98%	0.0	97%	JQ044774.1	994,74	97,6%
Select seq gb|JN408632.1|	Teucrium heterophyllum tRNA-Leu (trnL) gene, partial sequence; and trnL-trnF intergenic spacer, complete sequence; chloroplast	1000	1000	98%	0.0	97%	JN408632.1	989,80	97,1%
Select seq gb|JQ044777.1|	Teucrium polium subsp. polium isolate TP5 tRNA-Leu (trnL) gene, partial sequence; trnL-trnF intergenic spacer, complete sequence; and tRNA-Phe (trnF) gene, partial sequence; chloroplast	1000	1000	98%	0.0	97%	JQ044777.1	989,80	97,1%
Select seq gb|JQ044775.1|	Teucrium polium subsp. polium isolate TP3 tRNA-Leu (trnL) gene, partial sequence; trnL-trnF intergenic spacer, complete sequence; and tRNA-Phe (trnF) gene, partial sequence; chloroplast	1000	1000	98%	0.0	97%	JQ044775.1	989,80	97,1%
Select seq gb|KR150202.1|	Teucrium sp. 5 Kh108 trnL-trnF intergenic spacer region, partial sequence; chloroplast	961	961	95%	0.0	96%	KR150202.1	971,12	95,3%

Select seq gb|KR150230.1|	Teucrium sp. 3 Kh19 trnL-trnF intergenic spacer region, partial sequence; chloroplast	974	974	98% 0.0	96% KR150230.1	954,12	93,6%
Select seq gb|KR150221.1|	Teucrium sp. 1 Kh66 trnL-trnF intergenic spacer region, partial sequence; chloroplast	974	974	98% 0.0	96% KR150221.1	954,12	93,6%
Select seq gb|JN408594.1|	Teucrium antitauricum tRNA-Leu (trnL) gene, partial sequence; and trnL-trnF intergenic spacer, complete sequence; chloroplast	959	959	98% 0.0	95% JN408594.1	929,64	91,2%
Select seq gb|JN408631.1|	Teucrium halacsyanum tRNA-Leu (trnL) gene, partial sequence; and trnL-trnF intergenic spacer, complete sequence; chloroplast	948	948	98% 0.0	95% JN408631.1	918,98	90,2%
Select seq gb|JN408647.1|	Teucrium montbretii subsp. heliotropiifolium tRNA-Leu (trnL) gene, partial sequence; and trnL-trnF intergenic spacer, complete sequence; chloroplast	944	944	98% 0.0	95% JN408647.1	915,10	89,8%
Select seq gb|JN408676.1|	Teucrium viscidum var. miquelianum tRNA-Leu (trnL) gene, partial sequence; and trnL-trnF intergenic spacer, complete sequence; chloroplast	939	939	98% 0.0	95% JN408676.1	910,26	89,3%
Select seq gb|JN408672.1|	Teucrium lamiifolium subsp. stachyophyllum tRNA-Leu (trnL) gene, partial sequence; and trnL-trnF intergenic spacer, complete sequence; chloroplast	939	939	98% 0.0	95% JN408672.1	910,26	89,3%
Select seq gb|JN408636.1|	Teucrium japonicum tRNA-Leu (trnL) gene, partial sequence; and trnL-trnF intergenic spacer, complete sequence; chloroplast	939	939	98% 0.0	95% JN408636.1	910,26	89,3%
Select seq gb|JN408664.1|	Teucrium royleanum tRNA-Leu (trnL) gene, partial sequence; and trnL-trnF intergenic spacer, complete sequence; chloroplast	937	937	98% 0.0	95% JN408664.1	908,32	89,1%
Select seq gb|JN408661.1|	Teucrium quadrifarium tRNA-Leu (trnL) gene, partial sequence; and trnL-trnF intergenic spacer, complete sequence; chloroplast	937	937	98% 0.0	95% JN408661.1	908,32	89,1%
Select seq gb|JN408665.1|	Teucrium rupestre tRNA-Leu (trnL) gene, partial sequence; and trnL-trnF intergenic spacer, complete sequence; chloroplast	933	933	98% 0.0	95% JN408665.1	904,44	88,8%
Select seq gb|JN408618.1|	Teucrium demnatense tRNA-Leu (trnL) gene, partial sequence; and trnL-trnF intergenic spacer, complete sequence; chloroplast	933	933	98% 0.0	95% JN408618.1	904,44	88,8%
Select seq gb|JN408628.1|	Teucrium edelbergii tRNA-Leu (trnL) gene, partial sequence; and trnL-trnF intergenic spacer, complete sequence; chloroplast	931	931	98% 0.0	95% JN408628.1	902,50	88,6%
Select seq gb|JN408633.1|	Teucrium hircanicum tRNA-Leu (trnL) gene, partial sequence; and trnL-trnF intergenic spacer, complete sequence; chloroplast	929	929	98% 0.0	95% JN408633.1	900,56	88,4%
Select seq gb|JN408598.1|	Teucrium arduinoi tRNA-Leu (trnL) gene, partial sequence; and trnL-trnF intergenic spacer, complete sequence; chloroplast	928	928	98% 0.0	95% JN408598.1	899,59	88,3%
Select seq gb|JN408649.1|	Teucrium odontites tRNA-Leu (trnL) gene, partial sequence; and trnL-trnF intergenic spacer, complete sequence; chloroplast	935	935	98% 0.0	94% JN408649.1	896,84	88,0%
Select seq gb|JN408674.1|	Teucridium parvifolium tRNA-Leu (trnL) gene, partial sequence; trnL-trnF intergenic spacer, complete sequence; and tRNA-Phe (trnF) gene, partial sequence; chloroplast	924	924	98% 0.0	95% JN408674.1	895,71	87,9%
Select seq gb|JN408656.1|	Teucrium capitatum tRNA-Leu (trnL) gene, partial sequence; and trnL-trnF intergenic spacer, complete sequence; chloroplast	918	918	98% 0.0	94% JN408656.1	880,53	86,4%
Select seq gb|JN408600.1|	Teucrium cubense tRNA-Leu (trnL) gene, partial sequence; trnL-trnF intergenic spacer, complete sequence; and tRNA-Phe (trnF) gene, partial sequence; chloroplast	918	918	98% 0.0	94% JN408600.1	880,53	86,4%
Select seq gb|JN408648.1|	Teucrium nudicaule tRNA-Leu (trnL) gene, partial sequence; and trnL-trnF intergenic spacer, complete sequence; chloroplast	917	917	98% 0.0	94% JN408648.1	879,57	86,3%
Select seq gb|JN408669.1|	Teucrium scorodonia tRNA-Leu (trnL) gene, partial sequence; and trnL-trnF intergenic spacer, complete sequence; chloroplast	915	915	98% 0.0	94% JN408669.1	877,65	86,1%
Select seq gb|JN408667.1|	Teucrium francoi tRNA-Leu (trnL) gene, partial sequence; and trnL-trnF intergenic spacer, complete sequence; chloroplast	915	915	98% 0.0	94% JN408667.1	877,65	86,1%
Select seq gb|JN408666.1|	Teucrium salviastrum tRNA-Leu (trnL) gene, partial sequence; and trnL-trnF intergenic spacer, complete sequence; chloroplast	915	915	98% 0.0	94% JN408666.1	877,65	86,1%
Select seq gb|JN408643.1|	Teucrium massiliense tRNA-Leu (trnL) gene, partial sequence; and trnL-trnF intergenic spacer, complete sequence; chloroplast	915	915	98% 0.0	94% JN408643.1	877,65	86,1%
Select seq gb|JN408640.1|	Teucrium lamiifolium subsp. lamiifolium tRNA-Leu (trnL) gene, partial sequence; and trnL-trnF intergenic spacer, complete sequence; chloroplast	915	915	98% 0.0	94% JN408640.1	877,65	86,1%
Select seq gb|JN408627.1|	Teucrium scorodonia tRNA-Leu (trnL) gene, partial sequence; and trnL-trnF intergenic spacer, complete sequence; chloroplast	915	915	98% 0.0	94% JN408627.1	877,65	86,1%
Select seq gb|JN408603.1|	Teucrium asiaticum tRNA-Leu (trnL) gene, partial sequence; and trnL-trnF intergenic spacer, complete sequence; chloroplast	915	915	98% 0.0	94% JN408603.1	877,65	86,1%
Select seq gb|JF694866.1|	Teucrium scorodonia isolate TSC8 tRNA-Leu (trnL) gene and trnL-trnF intergenic spacer, complete sequence; and tRNA-Phe (trnF) gene, partial sequence; plastid	915	915	98% 0.0	94% JF694866.1	877,65	86,1%

Select seq gb|JF694861.1|	Teucrium scorodonia isolate TSC3 tRNA-Leu (trnL) gene and trnL-trnF intergenic spacer, complete sequence; and tRNA-Phe (trnF) gene, partial sequence; plastid	915	915	98% 0.0	94% JF694861.1	877,65	86,1%
Select seq gb|JF694859.1|	Teucrium scorodonia isolate TSC1 tRNA-Leu (trnL) gene and trnL-trnF intergenic spacer, complete sequence; and tRNA-Phe (trnF) gene, partial sequence; plastid	915	915	98% 0.0	94% JF694859.1	877,65	86,1%
Select seq gb|JN408607.1|	Teucrium bracteatum tRNA-Leu (trnL) gene, partial sequence; and trnL-trnF intergenic spacer, complete sequence; chloroplast	913	913	98% 0.0	94% JN408607.1	875,73	85,9%
Select seq gb|JN408605.1|	Teucrium bicolor tRNA-Leu (trnL) gene, partial sequence; and trnL-trnF intergenic spacer, complete sequence; chloroplast	911	911	98% 0.0	94% JN408605.1	873,82	85,8%
Select seq gb|JF694864.1|	Teucrium scorodonia isolate TSC6 tRNA-Leu (trnL) gene and trnL-trnF intergenic spacer, complete sequence; and tRNA-Phe (trnF) gene, partial sequence; plastid	909	909	98% 0.0	94% JF694864.1	871,90	85,6%
Select seq gb|JN408595.1|	Teucrium abutiloides tRNA-Leu (trnL) gene, partial sequence; trnL-trnF intergenic spacer, complete sequence; and tRNA-Phe (trnF) gene, partial sequence; chloroplast	907	907	98% 0.0	94% JN408595.1	869,98	85,4%
Select seq gb|JN408675.1|	Teucrium trifidum tRNA-Leu (trnL) gene, partial sequence; and trnL-trnF intergenic spacer, complete sequence; chloroplast	902	902	98% 0.0	94% JN408675.1	865,18	84,9%
Select seq gb|JN408610.1|	Teucrium trifidum tRNA-Leu (trnL) gene, partial sequence; and trnL-trnF intergenic spacer, complete sequence; chloroplast	902	902	98% 0.0	94% JN408610.1	865,18	84,9%
Select seq gb|JN408591.1|	Spartothamnella puberula tRNA-Leu (trnL) gene, partial sequence; trnL-trnF intergenic spacer, complete sequence; and tRNA-Phe (trnF) gene, partial sequence; chloroplast	902	902	98% 0.0	94% JN408591.1	865,18	84,9%
Select seq gb|JN408658.1|	Teucrium pruinosum tRNA-Leu (trnL) gene, partial sequence; and trnL-trnF intergenic spacer, complete sequence; chloroplast	896	896	98% 0.0	94% JN408658.1	859,43	84,3%
Select seq gb|JN408651.1|	Teucrium orientale subsp. gloeotrichum tRNA-Leu (trnL) gene, partial sequence; and trnL-trnF intergenic spacer, complete sequence; chloroplast	896	896	98% 0.0	94% JN408651.1	859,43	84,3%
Select seq gb|JN408650.1|	Teucrium oliverianum tRNA-Leu (trnL) gene, partial sequence; and trnL-trnF intergenic spacer, complete sequence; chloroplast	896	896	98% 0.0	94% JN408650.1	859,43	84,3%
Select seq gb|JN408639.1|	Teucrium laciniatum tRNA-Leu (trnL) gene, partial sequence; and trnL-trnF intergenic spacer, complete sequence; chloroplast	896	896	98% 0.0	94% JN408639.1	859,43	84,3%
Select seq gb|JN408590.1|	Oncinocalyx betchei tRNA-Leu (trnL) gene, partial sequence; trnL-trnF intergenic spacer, complete sequence; and tRNA-Phe (trnF) gene, partial sequence; chloroplast	896	896	98% 0.0	94% JN408590.1	859,43	84,3%
Select seq gb|JN408663.1|	Teucrium resupinatum tRNA-Leu (trnL) gene, partial sequence; and trnL-trnF intergenic spacer, complete sequence; chloroplast	869	969	98% 0.0	95% JN408663.1	842,40	82,7%
Select seq gb|JN408602.1|	Teucrium aroanium tRNA-Leu (trnL) gene, partial sequence; and trnL-trnF intergenic spacer, complete sequence; chloroplast	878	878	97% 0.0	93% JN408602.1	841,79	82,6%
Select seq gb|JN408608.1|	Teucrium brevifolium tRNA-Leu (trnL) gene, partial sequence; and trnL-trnF intergenic spacer, complete sequence; chloroplast	876	876	97% 0.0	93% JN408608.1	839,88	82,4%
Select seq gb|JN408617.1|	Teucrium decipiens tRNA-Leu (trnL) gene, partial sequence; and trnL-trnF intergenic spacer, complete sequence; chloroplast	865	966	98% 0.0	95% JN408617.1	838,52	82,3%
Select seq gb|JN408677.1|	Teucrium webbianum tRNA-Leu (trnL) gene, partial sequence; and trnL-trnF intergenic spacer, complete sequence; chloroplast	883	883	98% 0.0	93% JN408677.1	837,95	82,2%
Select seq gb|JN408670.1|	Teucrium sessiliflorum tRNA-Leu (trnL) gene, partial sequence; and trnL-trnF intergenic spacer, complete sequence; chloroplast	883	883	98% 0.0	93% JN408670.1	837,95	82,2%
Select seq gb|JN408655.1|	Teucrium chamaedrys tRNA-Leu (trnL) gene, partial sequence; and trnL-trnF intergenic spacer, complete sequence; chloroplast	883	883	98% 0.0	93% JN408655.1	837,95	82,2%
Select seq gb|JN408626.1|	Teucrium chamaedrys tRNA-Leu (trnL) gene, partial sequence; and trnL-trnF intergenic spacer, complete sequence; chloroplast	883	883	98% 0.0	93% JN408626.1	837,95	82,2%
Select seq gb|JN408611.1|	Teucrium chamaedrys tRNA-Leu (trnL) gene, partial sequence; and trnL-trnF intergenic spacer, complete sequence; chloroplast	883	883	98% 0.0	93% JN408611.1	837,95	82,2%
Select seq gb|JN408637.1|	Teucrium jolyi tRNA-Leu (trnL) gene, partial sequence; and trnL-trnF intergenic spacer, complete sequence; chloroplast	870	870	97% 0.0	93% JN408637.1	834,12	81,9%
Select seq gb|JN408645.1|	Teucrium microphyllum tRNA-Leu (trnL) gene, partial sequence; and trnL-trnF intergenic spacer, complete sequence; chloroplast	878	878	98% 0.0	93% JN408645.1	833,20	81,8%
Select seq gb|JN408652.1|	Teucrium oxylepis tRNA-Leu (trnL) gene, partial sequence; and trnL-trnF intergenic spacer, complete sequence; chloroplast	876	876	98% 0.0	93% JN408652.1	831,31	81,6%
Select seq gb|JN408641.1|	Teucrium lucidum tRNA-Leu (trnL) gene, partial sequence; and trnL-trnF intergenic spacer, complete sequence; chloroplast	876	876	98% 0.0	93% JN408641.1	831,31	81,6%
Select seq gb|JN408612.1|	Teucrium chamaedrys tRNA-Leu (trnL) gene, partial sequence; and trnL-trnF intergenic spacer, complete sequence; chloroplast	876	876	98% 0.0	93% JN408612.1	831,31	81,6%

Select seq gb|JN408604.1|	Teucrium betonicum tRNA-Leu (trnL) gene, partial sequence; trnL-trnF intergenic spacer, complete sequence; and tRNA-Phe (trnF) gene, partial sequence; chloroplast	876	876	98% 0.0	93% JN408604.1	831,31	81,6%
Select seq gb|HQ646991.1|	Teucrium flavum subsp. flavum isolate TFF56 tRNA-Leu (trnL) gene and trnL-trnF intergenic spacer, complete sequence; and tRNA-Phe (trnF) gene, partial sequence; chloroplast	876	876	98% 0.0	93% HQ646991.1	831,31	81,6%
Select seq gb|HQ646990.1|	Teucrium flavum subsp. glaucum isolate TFG64 tRNA-Leu (trnL) gene and trnL-trnF intergenic spacer, complete sequence; and tRNA-Phe (trnF) gene, partial sequence; chloroplast	876	876	98% 0.0	93% HQ646990.1	831,31	81,6%
Select seq gb|HQ646981.1|	Teucrium flavum subsp. glaucum isolate TFG54 tRNA-Leu (trnL) gene and trnL-trnF intergenic spacer, complete sequence; and tRNA-Phe (trnF) gene, partial sequence; chloroplast	876	876	98% 0.0	93% HQ646981.1	831,31	81,6%
Select seq gb|JN408668.1|	Teucrium scordium tRNA-Leu (trnL) gene, partial sequence; and trnL-trnF intergenic spacer, complete sequence; chloroplast	874	874	98% 0.0	93% JN408668.1	829,41	81,4%
Select seq gb|JN408644.1|	Teucrium melissoides tRNA-Leu (trnL) gene, partial sequence; and trnL-trnF intergenic spacer, complete sequence; chloroplast	874	874	98% 0.0	93% JN408644.1	829,41	81,4%
Select seq gb|JN408653.1|	Teucrium pseudochamaepitys tRNA-Leu (trnL) gene, partial sequence; and trnL-trnF intergenic spacer, complete sequence; chloroplast	865	865	97% 0.0	93% JN408653.1	829,33	81,4%
Select seq gb|JN408622.1|	Teucrium pseudochamaepitys tRNA-Leu (trnL) gene, partial sequence; and trnL-trnF intergenic spacer, complete sequence; chloroplast	865	865	97% 0.0	93% JN408622.1	829,33	81,4%
Select seq gb|JN408635.1|	Teucrium intricatum tRNA-Leu (trnL) gene, partial sequence; and trnL-trnF intergenic spacer, complete sequence; chloroplast	872	872	98% 0.0	93% JN408635.1	827,51	81,2%
Select seq gb|JN408606.1|	Teucrium botrys tRNA-Leu (trnL) gene, partial sequence; and trnL-trnF intergenic spacer, complete sequence; chloroplast	872	872	98% 0.0	93% JN408606.1	827,51	81,2%
Select seq gb|JN408654.1|	Teucrium divaricatum tRNA-Leu (trnL) gene, partial sequence; and trnL-trnF intergenic spacer, complete sequence; chloroplast	870	870	98% 0.0	93% JN408654.1	825,61	81,0%
Select seq gb|JN408638.1|	Teucrium kotschyanum tRNA-Leu (trnL) gene, partial sequence; and trnL-trnF intergenic spacer, complete sequence; chloroplast	870	870	98% 0.0	93% JN408638.1	825,61	81,0%
Select seq gb|JN408593.1|	Teucrium flavum subsp. glaucum tRNA-Leu (trnL) gene, partial sequence; and trnL-trnF intergenic spacer, complete sequence; chloroplast	870	870	98% 0.0	93% JN408593.1	825,61	81,0%
Select seq gb|JN408673.1|	Teucrium subspinosum tRNA-Leu (trnL) gene, partial sequence; and trnL-trnF intergenic spacer, complete sequence; chloroplast	865	865	98% 0.0	93% JN408673.1	820,87	80,6%
Select seq gb|JN408642.1|	Teucrium marum tRNA-Leu (trnL) gene, partial sequence; and trnL-trnF intergenic spacer, complete sequence; chloroplast	865	865	98% 0.0	93% JN408642.1	820,87	80,6%
Select seq gb|GU331787.1|	Teucrium marum isolate TM12N tRNA-Leu (trnL) gene, partial sequence; trnL-trnF intergenic spacer, complete sequence; and tRNA-Phe (trnF) gene, partial sequence; chloroplast	865	865	98% 0.0	93% GU331787.1	820,87	80,6%
Select seq gb|GU331782.1|	Teucrium marum isolate TM1C tRNA-Leu (trnL) gene, partial sequence; trnL-trnF intergenic spacer, complete sequence; and tRNA-Phe (trnF) gene, partial sequence; chloroplast	865	865	98% 0.0	93% GU331782.1	820,87	80,6%

Select for downloading or viewing reports	Kh074_ITS Description	Max score	Total score	Query cover	E value	Ident	Accession	(Ident/Cover)* Max score	Deviation from top hit
Select seq gb|KJ584242.1|	Perovskia atriplicifolia isolate 472 internal transcribed spacer 1, partial sequence; 5.8S ribosomal RNA gene, complete sequence; and internal transcribed spacer 2, partial sequence	263	263	33%	3,00E-66	90%	KJ584242.1	0,00	#DIV/0!
Select seq gb|GU997682.1|	Botryosphaeria dothidea strain SDAU07-114 18S ribosomal RNA gene, partial sequence; internal transcribed spacer 1, 5.8S ribosomal RNA gene, and internal transcribed spacer 2, complete sequence; and 28S ribosomal RNA gene, partial sequence	202	202	29%	8,00E-48	87%	GU997682.1	0,00	#DIV/0!
Select seq gb|FJ025236.1|	Fungal endophyte sp. O9-3171 18S ribosomal RNA gene, partial sequence; internal transcribed spacer 1, 5.8S ribosomal RNA gene, and internal transcribed spacer 2, complete sequence; and 28S ribosomal RNA gene, partial sequence	202	202	29%	8,00E-48	87%	FJ025236.1	0,00	#DIV/0!
Select seq gb|FJ171718.1|	Botryosphaeria dothidea strain SDAU07160 18S ribosomal RNA gene, partial sequence; internal transcribed spacer 1, 5.8S ribosomal RNA gene, and internal transcribed spacer 2, complete sequence; and 28S ribosomal RNA gene, partial sequence	202	202	29%	8,00E-48	87%	FJ171718.1	0,00	#DIV/0!
Select seq gb|KJ921848.1|	Botryosphaeria dothidea isolate MBAi126AG 18S ribosomal RNA gene, partial sequence; internal transcribed spacer 1 and 5.8S ribosomal RNA gene, complete sequence; and internal transcribed spacer 2, partial sequence	200	200	29%	3,00E-47	87%	KJ921848.1	0,00	#DIV/0!
Select seq gb|KT375698.1|	Fungal sp. strain J7C1 internal transcribed spacer 1, partial sequence; 5.8S ribosomal RNA gene and internal transcribed spacer 2, complete sequence; and 28S ribosomal RNA gene, partial sequence	198	198	29%	1,00E-46	86%	KT375698.1	0,00	#DIV/0!
Select seq gb|KP335213.1|	Fungal endophyte isolate SNP007 internal transcribed spacer 1, partial sequence; 5.8S ribosomal RNA gene and internal transcribed spacer 2, complete sequence; and 28S ribosomal RNA gene, partial sequence	198	198	29%	1,00E-46	86%	KP335213.1	0,00	#DIV/0!
Select seq gb|KF294012.1|	Botryosphaeria dothidea strain G02_4688165_B internal transcribed spacer 1, partial sequence; 5.8S ribosomal RNA gene and internal transcribed spacer 2, complete sequence; and 28S ribosomal RNA gene, partial sequence	198	198	29%	1,00E-46	86%	KF294012.1	0,00	#DIV/0!
Select seq gb|KF766153.1|	Dichomera saubinetii strain CBS 990.70 18S ribosomal RNA gene, partial sequence; internal transcribed spacer 1, 5.8S ribosomal RNA gene, and internal transcribed spacer 2, complete sequence; and 28S ribosomal RNA gene, partial sequence	198	198	29%	1,00E-46	86%	KF766153.1	0,00	#DIV/0!
Select seq gb|HQ529752.1|	Botryosphaeria dothidea isolate UCR750 18S ribosomal RNA gene, partial sequence; internal transcribed spacer 1, 5.8S ribosomal RNA gene, and internal transcribed spacer 2, complete sequence; and 28S ribosomal RNA gene, partial sequence	198	198	29%	1,00E-46	86%	HQ529752.1	0,00	#DIV/0!
Select seq gb|HQ529750.1|	Botryosphaeria dothidea isolate UCR453 18S ribosomal RNA gene, partial sequence; internal transcribed spacer 1, 5.8S ribosomal RNA gene, and internal transcribed spacer 2, complete sequence; and 28S ribosomal RNA gene, partial sequence	198	198	29%	1,00E-46	86%	HQ529750.1	0,00	#DIV/0!
Select seq gb|EU520052.1|	Botryosphaeria dothidea isolate NW231 internal transcribed spacer 1, partial sequence; 5.8S ribosomal RNA gene and internal transcribed spacer 2, complete sequence; and 28S ribosomal RNA gene, partial sequence	198	198	29%	1,00E-46	86%	EU520052.1	0,00	#DIV/0!
Select seq gb|AY744379.1|	Dichomera saubinettii strain CBS990_70 18S ribosomal RNA gene, partial sequence; internal transcribed spacer 1, 5.8S ribosomal RNA gene, and internal transcribed spacer 2, complete sequence; and 28S ribosomal RNA gene, partial sequence	198	198	29%	1,00E-46	86%	AY744379.1	0,00	#DIV/0!
Select seq gb|KT192428.1|	Botryosphaeria dothidea strain PZ-ZG-3-1-1 internal transcribed spacer 1, partial sequence; 5.8S ribosomal RNA gene and internal transcribed spacer 2, complete sequence; and 28S ribosomal RNA gene, partial sequence	202	202	30%	8,00E-48	87%	KT192428.1	0,00	#DIV/0!
Select seq gb|HQ660462.1|	Botryosphaeria dothidea isolate ZY-713 18S ribosomal RNA gene, partial sequence; internal transcribed spacer 1 and 5.8S ribosomal RNA gene, complete sequence; and internal transcribed spacer 2, partial sequence	202	202	30%	8,00E-48	87%	HQ660462.1	0,00	#DIV/0!
Select seq gb|HQ731645.1|	Botryosphaeria sp. Ponipodef 09 internal transcribed spacer 1, partial sequence; 5.8S ribosomal RNA gene and internal transcribed spacer 2, complete sequence; and 28S ribosomal RNA gene, partial sequence	202	202	30%	8,00E-48	87%	HQ731645.1	0,00	#DIV/0!
Select seq gb|GU944819.1|	Botryosphaeria dothidea strain DAR80010 18S ribosomal RNA gene, partial sequence; internal transcribed spacer 1, 5.8S ribosomal RNA gene, and internal transcribed spacer 2, complete sequence; and 28S ribosomal RNA gene, partial sequence	202	202	30%	8,00E-48	87%	GU944819.1	0,00	#DIV/0!
Select seq gb|FJ462744.1|	Botryosphaeria dothidea isolate T121 18S ribosomal RNA gene, partial sequence; internal transcribed spacer 1, 5.8S ribosomal RNA gene, and internal transcribed spacer 2, complete sequence; and 28S ribosomal RNA gene, partial sequence	202	202	30%	8,00E-48	87%	FJ462744.1	0,00	#DIV/0!
Select seq gb|EU520232.1|	Dothiorella gregaria isolate NW572 18S ribosomal RNA gene, partial sequence; internal transcribed spacer 1, 5.8S ribosomal RNA gene, and internal transcribed spacer 2, complete sequence; and 28S ribosomal RNA gene, partial sequence	202	202	30%	8,00E-48	87%	EU520232.1	0,00	#DIV/0!
Select seq gb|EU520152.1|	Botryosphaeria dothidea isolate NW276 internal transcribed spacer 1, partial sequence; 5.8S ribosomal RNA gene and internal transcribed spacer 2, complete sequence; and 28S ribosomal RNA gene, partial sequence	202	202	30%	8,00E-48	87%	EU520152.1	0,00	#DIV/0!
Select seq gb|EU169140.1|	Botryosphaeria dothidea strain P1 18S ribosomal RNA gene, partial sequence; internal transcribed spacer 1, 5.8S ribosomal RNA gene, and internal transcribed spacer 2, complete sequence; and 28S ribosomal RNA gene, partial sequence	202	202	30%	8,00E-48	87%	EU169140.1	0,00	#DIV/0!
Select seq gb|DQ667330.1|	Perovskia scrophulariifolia isolate x532 18S ribosomal RNA gene, partial sequence; internal transcribed spacer 1, 5.8S ribosomal RNA gene, and internal transcribed spacer 2, complete sequence; and 28S ribosomal RNA gene, partial sequence	272	272	40%	6,00E-69	86%	DQ667330.1	0,00	#DIV/0!
Select seq gb|KT735105.1|	Botryosphaeria dothidea strain bd-1818 internal transcribed spacer 1, partial sequence; 5.8S ribosomal RNA gene and internal transcribed spacer 2, complete sequence; and 28S ribosomal RNA gene, partial sequence	196	196	29%	4,00E-46	86%	KT735105.1	0,00	#DIV/0!
Select seq dbj|LC120711.1|	Botryosphaeria dothidea genes for 18S rRNA, ITS1, 5.8S rRNA, ITS2, 28S rRNA, partial and complete sequence	196	196	29%	4,00E-46	86%	LC120711.1	0,00	#DIV/0!
Select seq gb|KP183180.1|	Botryosphaeria dothidea isolate ZJXC1-2 internal transcribed spacer 1, partial sequence; 5.8S ribosomal RNA gene and internal transcribed spacer 2, complete sequence; and 28S ribosomal RNA gene, partial sequence	196	196	29%	4,00E-46	86%	KP183180.1	0,00	#DIV/0!
Select seq gb|KP183179.1|	Botryosphaeria dothidea isolate ZJXC1-1 internal transcribed spacer 1, partial sequence; 5.8S ribosomal RNA gene and internal transcribed spacer 2, complete sequence; and 28S ribosomal RNA gene, partial sequence	196	196	29%	4,00E-46	86%	KP183179.1	0,00	#DIV/0!

Select seq gb|KP183177.1|	Botryosphaeria dothidea isolate SHSJ5-2 internal transcribed spacer 1, partial sequence; 5.8S ribosomal RNA gene and internal transcribed spacer 2, complete sequence; and 28S ribosomal RNA gene, partial sequence	196	196	29%	4,00E-46	86%	KP183177.1	0,00	#DIV/0!
Select seq gb|KP183176.1|	Botryosphaeria dothidea isolate SHSJ2-1 internal transcribed spacer 1, partial sequence; 5.8S ribosomal RNA gene and internal transcribed spacer 2, complete sequence; and 28S ribosomal RNA gene, partial sequence	196	196	29%	4,00E-46	86%	KP183176.1	0,00	#DIV/0!
Select seq gb|KP183175.1|	Botryosphaeria dothidea isolate SHSJ1-1 internal transcribed spacer 1, partial sequence; 5.8S ribosomal RNA gene and internal transcribed spacer 2, complete sequence; and 28S ribosomal RNA gene, partial sequence	196	196	29%	4,00E-46	86%	KP183175.1	0,00	#DIV/0!
Select seq gb|KP183174.1|	Botryosphaeria dothidea isolate SD-B8 internal transcribed spacer 1, partial sequence; 5.8S ribosomal RNA gene and internal transcribed spacer 2, complete sequence; and 28S ribosomal RNA gene, partial sequence	196	196	29%	4,00E-46	86%	KP183174.1	0,00	#DIV/0!
Select seq gb|KP183173.1|	Botryosphaeria dothidea isolate SCDY10-1 internal transcribed spacer 1, partial sequence; 5.8S ribosomal RNA gene and internal transcribed spacer 2, complete sequence; and 28S ribosomal RNA gene, partial sequence	196	196	29%	4,00E-46	86%	KP183173.1	0,00	#DIV/0!
Select seq gb|KP183172.1|	Botryosphaeria dothidea isolate SCDY7-1 internal transcribed spacer 1, partial sequence; 5.8S ribosomal RNA gene and internal transcribed spacer 2, complete sequence; and 28S ribosomal RNA gene, partial sequence	196	196	29%	4,00E-46	86%	KP183172.1	0,00	#DIV/0!
Select seq gb|KP183171.1|	Botryosphaeria dothidea isolate SCDY2-3 internal transcribed spacer 1, partial sequence; 5.8S ribosomal RNA gene and internal transcribed spacer 2, complete sequence; and 28S ribosomal RNA gene, partial sequence	196	196	29%	4,00E-46	86%	KP183171.1	0,00	#DIV/0!
Select seq gb|KP183170.1|	Botryosphaeria dothidea isolate LNZHA7 internal transcribed spacer 1, partial sequence; 5.8S ribosomal RNA gene and internal transcribed spacer 2, complete sequence; and 28S ribosomal RNA gene, partial sequence	196	196	29%	4,00E-46	86%	KP183170.1	0,00	#DIV/0!
Select seq gb|KP183169.1|	Botryosphaeria dothidea isolate LNZHA3 internal transcribed spacer 1, partial sequence; 5.8S ribosomal RNA gene and internal transcribed spacer 2, complete sequence; and 28S ribosomal RNA gene, partial sequence	196	196	29%	4,00E-46	86%	KP183169.1	0,00	#DIV/0!
Select seq gb|KP183168.1|	Botryosphaeria dothidea isolate LNZHA2-1 internal transcribed spacer 1, partial sequence; 5.8S ribosomal RNA gene and internal transcribed spacer 2, complete sequence; and 28S ribosomal RNA gene, partial sequence	196	196	29%	4,00E-46	86%	KP183168.1	0,00	#DIV/0!
Select seq gb|KP183167.1|	Botryosphaeria dothidea isolate LNXC13-2 internal transcribed spacer 1, partial sequence; 5.8S ribosomal RNA gene and internal transcribed spacer 2, complete sequence; and 28S ribosomal RNA gene, partial sequence	196	196	29%	4,00E-46	86%	KP183167.1	0,00	#DIV/0!
Select seq gb|KP183166.1|	Botryosphaeria dothidea isolate LNXC8-1 internal transcribed spacer 1, partial sequence; 5.8S ribosomal RNA gene and internal transcribed spacer 2, complete sequence; and 28S ribosomal RNA gene, partial sequence	196	196	29%	4,00E-46	86%	KP183166.1	0,00	#DIV/0!
Select seq gb|KP183165.1|	Botryosphaeria dothidea isolate LNXC4-1 internal transcribed spacer 1, partial sequence; 5.8S ribosomal RNA gene and internal transcribed spacer 2, complete sequence; and 28S ribosomal RNA gene, partial sequence	196	196	29%	4,00E-46	86%	KP183165.1	0,00	#DIV/0!
Select seq gb|KP183164.1|	Botryosphaeria dothidea isolate LNPLD4 internal transcribed spacer 1, partial sequence; 5.8S ribosomal RNA gene and internal transcribed spacer 2, complete sequence; and 28S ribosomal RNA gene, partial sequence	196	196	29%	4,00E-46	86%	KP183164.1	0,00	#DIV/0!
Select seq gb|KP183163.1|	Botryosphaeria dothidea isolate JSGY7 internal transcribed spacer 1, partial sequence; 5.8S ribosomal RNA gene and internal transcribed spacer 2, complete sequence; and 28S ribosomal RNA gene, partial sequence	196	196	29%	4,00E-46	86%	KP183163.1	0,00	#DIV/0!
Select seq gb|KP183160.1|	Botryosphaeria dothidea isolate FJCT4-1 internal transcribed spacer 1, partial sequence; 5.8S ribosomal RNA gene and internal transcribed spacer 2, complete sequence; and 28S ribosomal RNA gene, partial sequence	196	196	29%	4,00E-46	86%	KP183160.1	0,00	#DIV/0!
Select seq gb|KT390194.1|	Botryosphaeria dothidea strain W-7 internal transcribed spacer 1, partial sequence; 5.8S ribosomal RNA gene and internal transcribed spacer 2, complete sequence; and 28S ribosomal RNA gene, partial sequence	196	196	29%	4,00E-46	86%	KT390194.1	0,00	#DIV/0!
Select seq gb|KT189494.1|	Botryosphaeria dothidea strain LPSU20120217 internal transcribed spacer 1, partial sequence; 5.8S ribosomal RNA gene and internal transcribed spacer 2, complete sequence; and 28S ribosomal RNA gene, partial sequence	196	196	29%	4,00E-46	86%	KT189494.1	0,00	#DIV/0!
Select seq gb|KM103222.1|	Botryosphaeria dothidea strain CMW35472 internal transcribed spacer 1, partial sequence; 5.8S ribosomal RNA gene, complete sequence; and internal transcribed spacer 2, partial sequence	196	196	29%	4,00E-46	86%	KM103222.1	0,00	#DIV/0!
Select seq gb|KM103220.1|	Botryosphaeria dothidea strain CMW35470 internal transcribed spacer 1, partial sequence; 5.8S ribosomal RNA gene, complete sequence; and internal transcribed spacer 2, partial sequence	196	196	29%	4,00E-46	86%	KM103220.1	0,00	#DIV/0!
Select seq gb|KM103217.1|	Botryosphaeria dothidea strain CMW35462 internal transcribed spacer 1, partial sequence; 5.8S ribosomal RNA gene, complete sequence; and internal transcribed spacer 2, partial sequence	196	196	29%	4,00E-46	86%	KM103217.1	0,00	#DIV/0!
Select seq gb|KM435317.1|	Botryosphaeria dothidea isolate Hen 4 18S ribosomal RNA gene, partial sequence; internal transcribed spacer 1, 5.8S ribosomal RNA gene, and internal transcribed spacer 2, complete sequence; and 28S ribosomal RNA gene, partial sequence	196	196	29%	4,00E-46	86%	KM435317.1	0,00	#DIV/0!
Select seq gb|KM435314.1|	Botryosphaeria dothidea isolate BD1 18S ribosomal RNA gene, partial sequence; internal transcribed spacer 1, 5.8S ribosomal RNA gene, and internal transcribed spacer 2, complete sequence; and 28S ribosomal RNA gene, partial sequence	196	196	29%	4,00E-46	86%	KM435314.1	0,00	#DIV/0!
Select seq gb|KM435312.1|	Botryosphaeria dothidea isolate Hen 1 18S ribosomal RNA gene, partial sequence; internal transcribed spacer 1, 5.8S ribosomal RNA gene, and internal transcribed spacer 2, complete sequence; and 28S ribosomal RNA gene, partial sequence	196	196	29%	4,00E-46	86%	KM435312.1	0,00	#DIV/0!
Select seq gb|KM435311.1|	Botryosphaeria dothidea isolate Kiru 1 18S ribosomal RNA gene, partial sequence; internal transcribed spacer 1, 5.8S ribosomal RNA gene, and internal transcribed spacer 2, complete sequence; and 28S ribosomal RNA gene, partial sequence	196	196	29%	4,00E-46	86%	KM435311.1	0,00	#DIV/0!
Select seq gb|KT192429.1|	Botryosphaeria dothidea strain PZ-ZG-3-2-2 internal transcribed spacer 1, partial sequence; 5.8S ribosomal RNA gene and internal transcribed spacer 2, complete sequence; and 28S ribosomal RNA gene, partial sequence	196	196	29%	4,00E-46	86%	KT192429.1	0,00	#DIV/0!
Select seq gb|KT192423.1|	Botryosphaeria dothidea strain PZ-ZG-1-2-2 internal transcribed spacer 1, partial sequence; 5.8S ribosomal RNA gene and internal transcribed spacer 2, complete sequence; and 28S ribosomal RNA gene, partial sequence	196	196	29%	4,00E-46	86%	KT192423.1	0,00	#DIV/0!
Select seq gb|KT192418.1|	Botryosphaeria dothidea strain P-ZG-3-2-3 18S ribosomal RNA gene, partial sequence; internal transcribed spacer 1, 5.8S ribosomal RNA gene, and internal transcribed spacer 2, complete sequence; and 28S ribosomal RNA gene, partial sequence	196	196	29%	4,00E-46	86%	KT192418.1	0,00	#DIV/0!

Select seq gb|KT192390.1|	Botryosphaeria dothidea strain ZG-2-2-4 internal transcribed spacer 1, partial sequence; 5.8S ribosomal RNA gene and internal transcribed spacer 2, complete sequence; and 28S ribosomal RNA gene, partial sequence	196	196	29%	4,00E-46	86%	KT192390.1	0,00	#DIV/0!
Select seq gb|KT192388.1|	Botryosphaeria dothidea strain ZG-2-2-2 internal transcribed spacer 1, partial sequence; 5.8S ribosomal RNA gene and internal transcribed spacer 2, complete sequence; and 28S ribosomal RNA gene, partial sequence	196	196	29%	4,00E-46	86%	KT192388.1	0,00	#DIV/0!
Select seq gb|KT192257.1|	Botryosphaeria dothidea strain DZ-3-3-4 18S ribosomal RNA gene, partial sequence; internal transcribed spacer 1, 5.8S ribosomal RNA gene, and internal transcribed spacer 2, complete sequence; and 28S ribosomal RNA gene, partial sequence	196	196	29%	4,00E-46	86%	KT192257.1	0,00	#DIV/0!
Select seq gb|KT192252.1|	Botryosphaeria dothidea strain DZ-3-2-2 internal transcribed spacer 1, partial sequence; 5.8S ribosomal RNA gene and internal transcribed spacer 2, complete sequence; and 28S ribosomal RNA gene, partial sequence	196	196	29%	4,00E-46	86%	KT192252.1	0,00	#DIV/0!
Select seq gb|KT192234.1|	Botryosphaeria dothidea strain DZ-1-4-4 internal transcribed spacer 1, partial sequence; 5.8S ribosomal RNA gene and internal transcribed spacer 2, complete sequence; and 28S ribosomal RNA gene, partial sequence	196	196	29%	4,00E-46	86%	KT192234.1	0,00	#DIV/0!
Select seq gb|KT192226.1|	Botryosphaeria dothidea strain DZ-1-1-2 internal transcribed spacer 1, partial sequence; 5.8S ribosomal RNA gene and internal transcribed spacer 2, complete sequence; and 28S ribosomal RNA gene, partial sequence	196	196	29%	4,00E-46	86%	KT192226.1	0,00	#DIV/0!
Select seq gb|KR232672.1|	Botryosphaeria dothidea strain NCPZ 18S ribosomal RNA internal transcribed spacer, partial sequence; 5.8S ribosomal RNA gene and internal transcribed spacer 2, complete sequence; and 26S ribosomal RNA gene, partial sequence	196	196	29%	4,00E-46	86%	KR232672.1	0,00	#DIV/0!
Select seq gb|KR232668.1|	Botryosphaeria dothidea strain HJPZ 18S ribosomal RNA internal transcribed spacer, partial sequence; 5.8S ribosomal RNA gene and internal transcribed spacer 2, complete sequence; and 26S ribosomal RNA gene, partial sequence	196	196	29%	4,00E-46	86%	KR232668.1	0,00	#DIV/0!
Select seq gb|KR822122.1|	Botryosphaeria sp. XY-1-1-2 18S ribosomal RNA gene, partial sequence; internal transcribed spacer 1, 5.8S ribosomal RNA gene, and internal transcribed spacer 2, complete sequence; and 28S ribosomal RNA gene, partial sequence	196	196	29%	4,00E-46	86%	KR822122.1	0,00	#DIV/0!
Select seq gb|KR261710.1|	Botryosphaeria dothidea strain CERC2021 internal transcribed spacer 1, partial sequence; 5.8S ribosomal RNA gene and internal transcribed spacer 2, complete sequence; and 28S ribosomal RNA gene, partial sequence	196	196	29%	4,00E-46	86%	KR261710.1	0,00	#DIV/0!
Select seq gb|KR261709.1|	Botryosphaeria dothidea strain CERC2020 internal transcribed spacer 1, partial sequence; 5.8S ribosomal RNA gene and internal transcribed spacer 2, complete sequence; and 28S ribosomal RNA gene, partial sequence	196	196	29%	4,00E-46	86%	KR261709.1	0,00	#DIV/0!
Select seq gb|KR261708.1|	Botryosphaeria dothidea strain CERC1979 internal transcribed spacer 1, partial sequence; 5.8S ribosomal RNA gene and internal transcribed spacer 2, complete sequence; and 28S ribosomal RNA gene, partial sequence	196	196	29%	4,00E-46	86%	KR261708.1	0,00	#DIV/0!
Select seq gb|KR261707.1|	Botryosphaeria dothidea strain CERC1977 internal transcribed spacer 1, partial sequence; 5.8S ribosomal RNA gene and internal transcribed spacer 2, complete sequence; and 28S ribosomal RNA gene, partial sequence	196	196	29%	4,00E-46	86%	KR261707.1	0,00	#DIV/0!
Select seq gb|KR261706.1|	Botryosphaeria dothidea strain CERC1976 internal transcribed spacer 1, partial sequence; 5.8S ribosomal RNA gene and internal transcribed spacer 2, complete sequence; and 28S ribosomal RNA gene, partial sequence	196	196	29%	4,00E-46	86%	KR261706.1	0,00	#DIV/0!
Select seq gb|KR261705.1|	Botryosphaeria dothidea strain CERC1972 internal transcribed spacer 1, partial sequence; 5.8S ribosomal RNA gene and internal transcribed spacer 2, complete sequence; and 28S ribosomal RNA gene, partial sequence	196	196	29%	4,00E-46	86%	KR261705.1	0,00	#DIV/0!
Select seq gb|KR261704.1|	Botryosphaeria dothidea strain CERC1969 internal transcribed spacer 1, partial sequence; 5.8S ribosomal RNA gene and internal transcribed spacer 2, complete sequence; and 28S ribosomal RNA gene, partial sequence	196	196	29%	4,00E-46	86%	KR261704.1	0,00	#DIV/0!
Select seq gb|KR261703.1|	Botryosphaeria dothidea strain CERC1963 internal transcribed spacer 1, partial sequence; 5.8S ribosomal RNA gene and internal transcribed spacer 2, complete sequence; and 28S ribosomal RNA gene, partial sequence	196	196	29%	4,00E-46	86%	KR261703.1	0,00	#DIV/0!
Select seq gb|KM268875.1|	Botryosphaeria dothidea strain PG1 internal transcribed spacer 1, partial sequence; 5.8S ribosomal RNA gene and internal transcribed spacer 2, complete sequence; and 28S ribosomal RNA gene, partial sequence	196	196	29%	4,00E-46	86%	KM268875.1	0,00	#DIV/0!
Select seq gb|KM205454.1|	Botryosphaeria dothidea strain RCEF6351 internal transcribed spacer 1, partial sequence; 5.8S ribosomal RNA gene, complete sequence; and internal transcribed spacer 2, partial sequence	196	196	29%	4,00E-46	86%	KM205454.1	0,00	#DIV/0!
Select seq gb|KR703274.1|	Botryosphaeria dothidea strain KFRD-1 internal transcribed spacer 1, partial sequence; 5.8S ribosomal RNA gene and internal transcribed spacer 2, complete sequence; and 28S ribosomal RNA gene, partial sequence	196	196	29%	4,00E-46	86%	KR703274.1	0,00	#DIV/0!
Select seq gb|KR080852.1|	Fungal endophyte strain I12F-01935 internal transcribed spacer 1, partial sequence; 5.8S ribosomal RNA gene and internal transcribed spacer 2, complete sequence; and 26S ribosomal RNA gene, partial sequence	196	196	29%	4,00E-46	86%	KR080852.1	0,00	#DIV/0!
Select seq gb|KP749828.1|	Botryosphaeria dothidea strain CXY1813 internal transcribed spacer 1, partial sequence; 5.8S ribosomal RNA gene and internal transcribed spacer 2, complete sequence; and 28S ribosomal RNA gene, partial sequence	196	196	29%	4,00E-46	86%	KP749828.1	0,00	#DIV/0!
Select seq gb|KJ433398.1|	Botryosphaeria dothidea strain PG20 internal transcribed spacer 1, partial sequence; 5.8S ribosomal RNA gene, complete sequence; and internal transcribed spacer 2, partial sequence	196	196	29%	4,00E-46	86%	KJ433398.1	0,00	#DIV/0!
Select seq gb|KM819462.1|	Botryosphaeria dothidea strain DLPZ-1 internal transcribed spacer 1, partial sequence; 5.8S ribosomal RNA gene and internal transcribed spacer 2, complete sequence; and 28S ribosomal RNA gene, partial sequence	196	196	29%	4,00E-46	86%	KM819462.1	0,00	#DIV/0!
Select seq gb|KM386080.1|	Dothiorella sp. HL7528S internal transcribed spacer 1, partial sequence; 5.8S ribosomal RNA gene and internal transcribed spacer 2, complete sequence; and 28S ribosomal RNA gene, partial sequence	196	196	29%	4,00E-46	86%	KM386080.1	0,00	#DIV/0!
Select seq gb|KJ921875.1|	Botryosphaeria sp. 1029_8 18S ribosomal RNA gene, partial sequence; internal transcribed spacer 1, 5.8S ribosomal RNA gene, and internal transcribed spacer 2, complete sequence; and 28S ribosomal RNA gene, partial sequence	196	196	29%	4,00E-46	86%	KJ921875.1	0,00	#DIV/0!
Select seq gb|KJ921874.1|	Botryosphaeria sp. 92_201 18S ribosomal RNA gene, partial sequence; internal transcribed spacer 1, 5.8S ribosomal RNA gene, and internal transcribed spacer 2, complete sequence; and 28S ribosomal RNA gene, partial sequence	196	196	29%	4,00E-46	86%	KJ921874.1	0,00	#DIV/0!
Select seq gb|KJ921873.1|	Botryosphaeria sp. D136_100 18S ribosomal RNA gene, partial sequence; internal transcribed spacer 1, 5.8S ribosomal RNA gene, and internal transcribed spacer 2, complete sequence; and 28S ribosomal RNA gene, partial sequence	196	196	29%	4,00E-46	86%	KJ921873.1	0,00	#DIV/0!

Select seq gb|KJ921872.1|	Botryosphaeria sp. D137_105 18S ribosomal RNA gene, partial sequence; internal transcribed spacer 1, 5.8S ribosomal RNA gene, and internal transcribed spacer 2, complete sequence; and 28S ribosomal RNA gene, partial sequence	196	196	29%	4,00E-46	86%	KJ921872.1	0,00	#DIV/0!
Select seq gb|KJ572244.1|	Botryosphaeria sp. XSH25 internal transcribed spacer 1, partial sequence; 5.8S ribosomal RNA gene and internal transcribed spacer 2, complete sequence; and 28S ribosomal RNA gene, partial sequence	196	196	29%	4,00E-46	86%	KJ572244.1	0,00	#DIV/0!
Select seq gb|FJ024085.1|	Botryosphaeria dothidea internal transcribed spacer 1, partial sequence; 5.8S ribosomal RNA gene, complete sequence; and internal transcribed spacer 2, partial sequence	196	196	29%	4,00E-46	86%	FJ024085.1	0,00	#DIV/0!
Select seq gb|EU520100.1|	Neofusicoccum ribis isolate NW282 18S ribosomal RNA gene, partial sequence; internal transcribed spacer 1, 5.8S ribosomal RNA gene, and internal transcribed spacer 2, complete sequence; and 28S ribosomal RNA gene, partial sequence	196	196	29%	4,00E-46	86%	EU520100.1	0,00	#DIV/0!
Select seq gb|KR232492.1|	Botryosphaeria dothidea isolate PAK4 internal transcribed spacer 1, partial sequence; 5.8S ribosomal RNA gene and internal transcribed spacer 2, complete sequence; and 28S ribosomal RNA gene, partial sequence	200	200	30%	3,00E-47	87%	KR232492.1	0,00	#DIV/0!
Select seq gb|KC507274.1|	Botryosphaeria sp. DT1 18S ribosomal RNA gene, partial sequence; internal transcribed spacer 1, 5.8S ribosomal RNA gene, and internal transcribed spacer 2, complete sequence; and 28S ribosomal RNA gene, partial sequence	200	200	30%	3,00E-47	87%	KC507274.1	0,00	#DIV/0!
Select seq gb|KC492473.1|	Botryosphaeria dothidea strain MGEF30 18S ribosomal RNA gene, partial sequence; internal transcribed spacer 1 and 5.8S ribosomal RNA gene, complete sequence; and internal transcribed spacer 2, partial sequence	200	200	30%	3,00E-47	87%	KC492473.1	0,00	#DIV/0!
Select seq gb|KC492466.1|	Botryosphaeria dothidea strain MGEF23 18S ribosomal RNA gene, partial sequence; internal transcribed spacer 1, 5.8S ribosomal RNA gene, and internal transcribed spacer 2, complete sequence; and 28S ribosomal RNA gene, partial sequence	200	200	30%	3,00E-47	87%	KC492466.1	0,00	#DIV/0!
Select seq gb|JN545840.1|	Botryosphaeria dothidea isolate BD140 18S ribosomal RNA gene, partial sequence; internal transcribed spacer 1, 5.8S ribosomal RNA gene, and internal transcribed spacer 2, complete sequence; and 28S ribosomal RNA gene, partial sequence	200	200	30%	3,00E-47	87%	JN545840.1	0,00	#DIV/0!
Select seq gb|GU944810.1|	Botryosphaeria dothidea strain B23 18S ribosomal RNA gene, partial sequence; internal transcribed spacer 1, 5.8S ribosomal RNA gene, and internal transcribed spacer 2, complete sequence; and 28S ribosomal RNA gene, partial sequence	200	200	30%	3,00E-47	87%	GU944810.1	0,00	#DIV/0!
Select seq gb|FJ790846.1|	Botryosphaeria dothidea strain UCD2461TXb internal transcribed spacer 1, partial sequence; 5.8S ribosomal RNA gene, complete sequence; and internal transcribed spacer 2, partial sequence	200	200	30%	3,00E-47	87%	FJ790846.1	0,00	#DIV/0!
Select seq gb|HQ418845.1|	Dorystaechas hastata internal transcribed spacer 1, 5.8S ribosomal RNA gene, and internal transcribed spacer 2, complete sequence	206	206	31%	6,00E-49	86%	HQ418845.1	0,00	#DIV/0!
Select seq gb|AY506648.1|	Perovskia abrotanoides internal transcribed spacer 1, partial sequence; 5.8S ribosomal RNA gene, complete sequence; and internal transcribed spacer 2, partial sequence	219	219	33%	8,00E-53	86%	AY506648.1	0,00	#DIV/0!
Select seq gb|DQ667223.1|	Perovskia atriplicifolia isolate x024 18S ribosomal RNA gene, partial sequence; internal transcribed spacer 1, 5.8S ribosomal RNA gene, and internal transcribed spacer 2, complete sequence; and 28S ribosomal RNA gene, partial sequence	257	257	39%	2,00E-64	86%	DQ667223.1	0,00	#DIV/0!
Select seq gb|KP127980.1|	Botryosphaeria dothidea isolate EPU26CB internal transcribed spacer 1, partial sequence; 5.8S ribosomal RNA gene, complete sequence; and internal transcribed spacer 2, partial sequence	196	196	30%	4,00E-46	86%	KP127980.1	0,00	#DIV/0!
Select seq gb|KJ584248.1|	Dorystaechas hastata isolate 213 internal transcribed spacer 1, partial sequence; 5.8S ribosomal RNA gene, complete sequence; and internal transcribed spacer 2, partial sequence	202	202	31%	8,00E-48	86%	KJ584248.1	0,00	#DIV/0!
Select seq gb|DQ667252.1|	Dorystaechas hastata isolate x108 18S ribosomal RNA gene, partial sequence; internal transcribed spacer 1, 5.8S ribosomal RNA gene, and internal transcribed spacer 2, complete sequence; and 28S ribosomal RNA gene, partial sequence	211	211	39%	1,00E-50	83%	DQ667252.1	0,00	#DIV/0!

Select for downloading or viewing reports	Kh074_trnL Description	Max score	Total score	Query cover	E value	Ident	Accession	(Ident/Cover)* Max score	Deviation from top hit
Select seq gb|AY506615.1|	Perovskia abrotanoides tRNA-Leu (trnL) gene and trnL-trnF intergenic spacer, partial sequence; chloroplast	869	869	93%	0.0	99%	AY506615.1	925,06	100,0%
Select seq gb|AY570464.1|	Perovskia atriplicifolia voucher JBW 2524 tRNA-Leu and trnL-trnF intergenic spacer, partial sequence; chloroplast	900	900	98%	0.0	99%	AY570464.1	909,18	98,3%
Select seq gb|DQ667492.1|	Lepechinia lancifolia isolate x232 tRNA-Leu (trnL) gene and trnL-trnF intergenic spacer, partial sequence; chloroplast	878	878	99%	0.0	98%	DQ667492.1	869,13	94,0%
Select seq gb|AY570459.1|	Lepechinia chamaedryoides voucher JBW 2537 tRNA-Leu and trnL-trnF intergenic spacer, partial sequence; chloroplast	878	878	99%	0.0	98%	AY570459.1	869,13	94,0%
Select seq gb|JF301383.1|	Lepechinia salviifolia voucher R. Jabaily s.n. tRNA-Leu (trnL) gene and trnL-trnF intergenic spacer, partial sequence; chloroplast	822	822	93%	0.0	98%	JF301383.1	866,19	93,6%
Select seq gb|KF307433.1|	Lepechinia urbanii voucher B. Drew 135 tRNA-Leu (trnL) gene and trnL-trnF intergenic spacer, partial sequence; chloroplast	813	813	92%	0.0	98%	KF307433.1	866,02	93,6%
Select seq gb|KF307432.1|	Lepechinia speciosa voucher Cordeno 3060 tRNA-Leu (trnL) gene and trnL-trnF intergenic spacer, partial sequence; chloroplast	813	813	92%	0.0	98%	KF307432.1	866,02	93,6%
Select seq gb|KF307429.1|	Lepechinia salviae voucher R. Jabaily s.n. tRNA-Leu (trnL) gene and trnL-trnF intergenic spacer, partial sequence; chloroplast	813	813	92%	0.0	98%	KF307429.1	866,02	93,6%
Select seq gb|KF307428.1|	Lepechinia rufocampii voucher B. Drew 244 tRNA-Leu (trnL) gene and trnL-trnF intergenic spacer, partial sequence; chloroplast	813	813	92%	0.0	98%	KF307428.1	866,02	93,6%
Select seq gb|KF307426.1|	Lepechinia paniculata voucher B. Drew 241 tRNA-Leu (trnL) gene and trnL-trnF intergenic spacer, partial sequence; chloroplast	813	813	92%	0.0	98%	KF307426.1	866,02	93,6%
Select seq gb|KF307421.1|	Lepechinia heteromorpha voucher B. Drew 192 tRNA-Leu (trnL) gene and trnL-trnF intergenic spacer, partial sequence; chloroplast	813	813	92%	0.0	98%	KF307421.1	866,02	93,6%
Select seq gb|KF307420.1|	Lepechinia graveolens voucher Fuentes 10351 tRNA-Leu (trnL) gene and trnL-trnF intergenic spacer, partial sequence; chloroplast	813	813	92%	0.0	98%	KF307420.1	866,02	93,6%
Select seq gb|KF307415.1|	Lepechinia caulescens voucher B. Drew 149 tRNA-Leu (trnL) gene and trnL-trnF intergenic spacer, partial sequence; chloroplast	813	813	92%	0.0	98%	KF307415.1	866,02	93,6%
Select seq gb|KF307411.1|	Lepechinia bella voucher Rachel Jabaily s.n. tRNA-Leu (trnL) gene and trnL-trnF intergenic spacer, partial sequence; chloroplast	813	813	92%	0.0	98%	KF307411.1	866,02	93,6%
Select seq gb|JF301382.1|	Lepechinia radula voucher B. Drew 185 tRNA-Leu (trnL) gene and trnL-trnF intergenic spacer, partial sequence; chloroplast	824	824	94%	0.0	98%	JF301382.1	859,06	92,9%
Select seq gb|JF301379.1|	Lepechinia lamiifolia voucher B. Drew 178 tRNA-Leu (trnL) gene and trnL-trnF intergenic spacer, partial sequence; chloroplast	824	824	94%	0.0	98%	JF301379.1	859,06	92,9%
Select seq gb|JF301378.1|	Lepechinia hastata voucher B. Drew 44 tRNA-Leu (trnL) gene and trnL-trnF intergenic spacer, partial sequence; chloroplast	824	824	94%	0.0	98%	JF301378.1	859,06	92,9%
Select seq gb|JF301376.1|	Lepechinia caulescens voucher B. Drew 106 tRNA-Leu (trnL) gene and trnL-trnF intergenic spacer, partial sequence; chloroplast	824	824	94%	0.0	98%	JF301376.1	859,06	92,9%
Select seq gb|JF301377.1|	Lepechinia flammea tRNA-Leu (trnL) gene and trnL-trnF intergenic spacer, partial sequence; chloroplast	819	819	94%	0.0	98%	JF301377.1	853,85	92,3%
Select seq gb|KF307418.1|	Lepechinia floribunda voucher B. Drew 172 tRNA-Leu (trnL) gene and trnL-trnF intergenic spacer, partial sequence; chloroplast	808	808	92%	0.0	97%	KF307418.1	851,91	92,1%
Select seq gb|KF307417.1|	Lepechinia dioica voucher B. Drew 232 tRNA-Leu (trnL) gene and trnL-trnF intergenic spacer, partial sequence; chloroplast	808	808	92%	0.0	97%	KF307417.1	851,91	92,1%
Select seq gb|KF307416.1|	Lepechinia codon voucher B. Drew 177 tRNA-Leu (trnL) gene and trnL-trnF intergenic spacer, partial sequence; chloroplast	806	806	92%	0.0	97%	KF307416.1	849,80	91,9%
Select seq gb|JF301381.1|	Lepechinia mexicana voucher B. Drew 164 tRNA-Leu (trnL) gene and trnL-trnF intergenic spacer, partial sequence; chloroplast	811	811	93%	0.0	97%	JF301381.1	845,88	91,4%
Select seq gb|KF307435.1|	Lepechinia yecorana voucher Henrickson 24691 tRNA-Leu (trnL) gene and trnL-trnF intergenic spacer, partial sequence; chloroplast	802	802	92%	0.0	97%	KF307435.1	845,59	91,4%
Select seq gb|KF307430.1|	Lepechinia schiedeana voucher B. Drew 157 tRNA-Leu (trnL) gene and trnL-trnF intergenic spacer, partial sequence; chloroplast	802	802	92%	0.0	97%	KF307430.1	845,59	91,4%
Select seq gb|KF307422.1|	Lepechinia mexicana voucher B. Drew 130 tRNA-Leu (trnL) gene and trnL-trnF intergenic spacer, partial sequence; chloroplast	802	802	92%	0.0	97%	KF307422.1	845,59	91,4%

Select seq gb|KF307412.1|	Lepechinia betonicifolia voucher B. Drew 224 tRNA-Leu (trnL) gene and trnL-trnF intergenic spacer, partial sequence; chloroplast	802	802	92%	0.0	97%	KF307412.1	845,59	91,4%
Select seq gb|DQ667493.1|	Lepechinia conferta isolate x234 tRNA-Leu (trnL) gene and trnL-trnF intergenic spacer, partial sequence; chloroplast	861	861	99%	0.0	97%	DQ667493.1	843,61	91,2%
Select seq gb|JF301380.1|	Lepechinia mexicana voucher B. Drew 127 tRNA-Leu (trnL) gene and trnL-trnF intergenic spacer, partial sequence; chloroplast	813	813	94%	0.0	97%	JF301380.1	838,95	90,7%
Select seq gb|JF301361.1|	Chaunostoma mecistandrum voucher J.A. Monterrosa & R.A. Carballo 213 tRNA-Leu (trnL) gene and trnL-trnF intergenic spacer, partial sequence; chloroplast	813	813	94%	0.0	97%	JF301361.1	838,95	90,7%
Select seq gb|DQ667519.1|	Perovskia scrophulariifolia isolate x532 tRNA-Leu (trnL) gene and trnL-trnF intergenic spacer, partial sequence; chloroplast	865	865	99%	0.0	96%	DQ667519.1	838,79	90,7%
Select seq gb|AY570460.1|	Lepechinia fragrans voucher JBW 1333 tRNA-Leu and trnL-trnF intergenic spacer, partial sequence; chloroplast	822	822	96%	0.0	97%	AY570460.1	830,56	89,8%
Select seq gb|KR232566.1|	Rosmarinus officinalis chloroplast, complete genome	846	846	99%	0.0	97%	KR232566.1	828,91	89,6%
Select seq gb|DQ667494.1|	Lepechinia calycina isolate x246 tRNA-Leu (trnL) gene and trnL-trnF intergenic spacer, partial sequence; chloroplast	837	837	98%	0.0	97%	DQ667494.1	828,46	89,6%
Select seq gb|EU244612.1|	Agastache rugosa tRNA-Leu (trnL) gene and trnL-trnF intergenic spacer, partial sequence	845	845	99%	0.0	97%	EU244612.1	827,93	89,5%
Select seq gb|GU381480.1|	Agastache foeniculum voucher M:Braeuchler 2513 tRNA-Leu (trnL) gene and trnL-trnF intergenic spacer, partial sequence; chloroplast	817	817	96%	0.0	97%	GU381480.1	825,51	89,2%
Select seq gb|JF301375.1|	Lepechinia calycina voucher B. Drew 197 tRNA-Leu (trnL) gene and trnL-trnF intergenic spacer, partial sequence; chloroplast	797	797	94%	0.0	97%	JF301375.1	822,44	88,9%
Select seq gb|AY570465.1|	Rosmarinus officinalis voucher JBW 2558 tRNA-Leu and trnL-trnF intergenic spacer, partial sequence; chloroplast	815	815	97%	0.0	97%	AY570465.1	815,00	88,1%
Select seq gb|AY570452.1|	Agastache urticifolia voucher JBW 815 tRNA-Leu and trnL-trnF intergenic spacer, partial sequence; chloroplast	839	839	99%	0.0	96%	AY570452.1	813,58	87,9%
Select seq gb|GU381484.1|	Micromeria flagellaris voucher E:van der Werff & McPherson 13570 tRNA-Leu (trnL) gene and trnL-trnF intergenic spacer, partial sequence; chloroplast	808	808	96%	0.0	96%	GU381484.1	808,00	87,3%
Select seq gb|GU381483.1|	Micromeria flagellaris voucher E:Clement et al 2140 tRNA-Leu (trnL) gene and trnL-trnF intergenic spacer, partial sequence; chloroplast	808	808	96%	0.0	96%	GU381483.1	808,00	87,3%
Select seq gb|GU381481.1|	Micromeria cf. madagascariensis Morawetz 205 tRNA-Leu (trnL) gene and trnL-trnF intergenic spacer, partial sequence; chloroplast	808	808	96%	0.0	96%	GU381481.1	808,00	87,3%
Select seq gb|DQ667488.1|	Lycopus uniflorus isolate x221 tRNA-Leu (trnL) gene and trnL-trnF intergenic spacer, partial sequence; chloroplast	833	833	99%	0.0	96%	DQ667488.1	807,76	87,3%
Select seq gb|AY570458.1|	Lepechinia calycina voucher JBW 1344 tRNA-Leu and trnL-trnF intergenic spacer, partial sequence; chloroplast	832	832	99%	0.0	96%	AY570458.1	806,79	87,2%
Select seq gb|JQ669022.1|	Agastache rugosa voucher H. Kanai, K Hasagawa, K. Ohkubo 8916 tRNA-Leu (trnL) gene and trnL-trnF intergenic spacer, partial sequence; plastid	806	806	96%	0.0	96%	JQ669022.1	806,00	87,1%
Select seq gb|GU381478.1|	Lycopus europaeus voucher M:Braeuchler 2505 tRNA-Leu (trnL) gene and trnL-trnF intergenic spacer, partial sequence; chloroplast	806	806	96%	0.0	96%	GU381478.1	806,00	87,1%
Select seq gb|KC414319.1|	Salvia sclarea isolate 511190054 tRNA-Leu (trnL) gene and trnL-trnF intergenic spacer, partial sequence; chloroplast	828	828	99%	0.0	96%	KC414319.1	802,91	86,8%
Select seq gb|DQ667450.1|	Salvia verbascifolia isolate x129 tRNA-Leu (trnL) gene and trnL-trnF intergenic spacer, partial sequence; chloroplast	828	828	99%	0.0	96%	DQ667450.1	802,91	86,8%
Select seq gb|AY570466.1|	Salvia aethiopis voucher Classen-Bockhoff 26/6/02 tRNA-Leu and trnL-trnF intergenic spacer, partial sequence; chloroplast	819	819	98%	0.0	96%	AY570466.1	802,29	86,7%
Select seq gb|GU381485.1|	Micromeria sphaerophylla voucher E:Lewis et al 1064 tRNA-Leu (trnL) gene and trnL-trnF intergenic spacer, partial sequence; chloroplast	802	802	96%	0.0	96%	GU381485.1	802,00	86,7%
Select seq gb|DQ667447.1|	Salvia candidissima isolate x121 tRNA-Leu (trnL) gene and trnL-trnF intergenic spacer, partial sequence; chloroplast	817	817	98%	0.0	96%	DQ667447.1	800,33	86,5%
Select seq gb|KR150233.1|	Thymus sp. 6 Kh133 trnL-trnF intergenic spacer region, partial sequence; chloroplast	815	815	98%	0.0	96%	KR150233.1	798,37	86,3%
Select seq gb|KC414321.1|	Salvia tesquicola isolate 511190056 tRNA-Leu (trnL) gene and trnL-trnF intergenic spacer, partial sequence; chloroplast	822	822	99%	0.0	96%	KC414321.1	797,09	86,2%
Select seq gb|DQ667509.1|	Salvia whitehousei isolate x333 tRNA-Leu (trnL) gene and trnL-trnF intergenic spacer, partial sequence; chloroplast	822	822	99%	0.0	96%	DQ667509.1	797,09	86,2%

Select seq gb|AY570485.1|	Salvia lyrata voucher JBW 2554 tRNA-Leu and trnL-trnF intergenic spacer, partial sequence; chloroplast	822	822	99% 0.0	96% AY570485.1	797,09	86,2%
Select seq gb|GU381625.1|	Pentapleura subulifera voucher W:Rechinger 12085 tRNA-Leu (trnL) gene and trnL-trnF intergenic spacer, partial sequence; chloroplast	797	797	96% 0.0	96% GU381625.1	797,00	86,2%
Select seq gb|GU381496.1|	Killickia pilosa voucher M:Braeuchler 3832 tRNA-Leu (trnL) gene and trnL-trnF intergenic spacer, partial sequence; chloroplast	797	797	96% 0.0	96% GU381496.1	797,00	86,2%
Select seq gb|GU381493.1|	Killickia grandiflora voucher M:Braeuchler 3811 tRNA-Leu (trnL) gene and trnL-trnF intergenic spacer, partial sequence; chloroplast	797	797	96% 0.0	96% GU381493.1	797,00	86,2%
Select seq gb|GU381489.1|	Killickia lutea voucher NU<ZAF>:Hilliard & Burtt 9876 tRNA-Leu (trnL) gene and trnL-trnF intergenic spacer, partial sequence; chloroplast	797	797	96% 0.0	96% GU381489.1	797,00	86,2%
Select seq gb|GU381488.1|	Killickia compacta voucher M:Braeuchler 3816 tRNA-Leu (trnL) gene and trnL-trnF intergenic spacer, partial sequence; chloroplast	797	797	96% 0.0	96% GU381488.1	797,00	86,2%
Select seq gb|AY570478.1|	Salvia disermas voucher Linder 4117 tRNA-Leu and trnL-trnF intergenic spacer, partial sequence; chloroplast	813	813	98% 0.0	96% AY570478.1	796,41	86,1%
Select seq emb|AJ505546.1|	Rosmarinus officinalis plastid trnL-trnF intergenic spacer, specimen voucher cult., K-1975-1177, Chase 13331	813	813	98% 0.0	96% AJ505546.1	796,41	86,1%
Select seq gb|JQ669066.1|	Salvia sclarea voucher J. Walker 2527 tRNA-Leu (trnL) gene and trnL-trnF intergenic spacer, partial sequence; plastid	795	795	96% 0.0	96% JQ669066.1	795,00	85,9%
Select seq gb|DQ667477.1|	Melissa officinalis isolate x193 tRNA-Leu (trnL) gene and trnL-trnF intergenic spacer, partial sequence; chloroplast	811	811	98% 0.0	96% DQ667477.1	794,45	85,9%
Select seq gb|AY570469.1|	Salvia canariensis voucher Royal Botanical Gardens, Edinburgh. #19860478 tRNA-Leu and trnL-trnF intergenic spacer, partial sequence; chloroplast	802	802	97% 0.0	96% AY570469.1	793,73	85,8%
Select seq gb|KM519773.1|	Salvia kronenburgii tRNA-Leu (trnL) gene, partial sequence; trnL-trnF intergenic spacer, complete sequence; and tRNA-Phe (trnF) gene, partial sequence; chloroplast	817	817	99% 0.0	96% KM519773.1	792,24	85,6%
Select seq gb|KM519771.1|	Salvia euphratica var. leiocalycina tRNA-Leu (trnL) gene, partial sequence; trnL-trnF intergenic spacer, complete sequence; and tRNA-Phe (trnF) gene, partial sequence; chloroplast	817	817	99% 0.0	96% KM519771.1	792,24	85,6%
Select seq gb|KM519770.1|	Salvia euphratica var. euphratica tRNA-Leu (trnL) gene, partial sequence; trnL-trnF intergenic spacer, complete sequence; and tRNA-Phe (trnF) gene, partial sequence; chloroplast	817	817	99% 0.0	96% KM519770.1	792,24	85,6%
Select seq gb|KC414280.1|	Salvia cavaleriei isolate 511190006 tRNA-Leu (trnL) gene and trnL-trnF intergenic spacer, partial sequence; chloroplast	817	817	99% 0.0	96% KC414280.1	792,24	85,6%
Select seq gb|DQ667512.1|	Salvia austriaca isolate x416 tRNA-Leu (trnL) gene and trnL-trnF intergenic spacer, partial sequence; chloroplast	817	817	99% 0.0	96% DQ667512.1	792,24	85,6%
Select seq emb|AJ505529.1|	Melissa officinalis plastid trnL-trnF intergenic spacer, specimen voucher Catino	800	800	97% 0.0	96% AJ505529.1	791,75	85,6%
Select seq gb|EU200169.1|	Salvia cabulica tRNA-Leu (trnL) gene and trnL-trnF intergenic spacer, partial sequence; chloroplast	808	808	98% 0.0	96% EU200169.1	791,51	85,6%
Select seq gb|DQ667472.1|	Salvia cabulica isolate x182 tRNA-Leu (trnL) gene and trnL-trnF intergenic spacer, partial sequence; chloroplast	808	808	98% 0.0	96% DQ667472.1	791,51	85,6%
Select seq gb|AY570499.1|	Salvia texana voucher JBW 2562 tRNA-Leu and trnL-trnF intergenic spacer, partial sequence; chloroplast	815	815	99% 0.0	96% AY570499.1	790,30	85,4%
Select seq gb|AY570492.1|	Salvia sclarea voucher JBW 2527 tRNA-Leu and trnL-trnF intergenic spacer, partial sequence; chloroplast	815	815	99% 0.0	96% AY570492.1	790,30	85,4%
Select seq gb|EU200167.1|	Salvia aucheri var. canescens tRNA-Leu (trnL) gene and trnL-trnF intergenic spacer, partial sequence; chloroplast	802	802	98% 0.0	96% EU200167.1	785,63	84,9%
Select seq gb|DQ667471.1|	Salvia aucheri var. canescens isolate x181 tRNA-Leu (trnL) gene and trnL-trnF intergenic spacer, partial sequence; chloroplast	802	802	98% 0.0	96% DQ667471.1	785,63	84,9%
Select seq gb|EU200175.1|	Salvia hydrangea tRNA-Leu (trnL) gene and trnL-trnF intergenic spacer, partial sequence; chloroplast	800	800	98% 0.0	96% EU200175.1	783,67	84,7%
Select seq gb|DQ667473.1|	Salvia hydrangea isolate x185 tRNA-Leu (trnL) gene and trnL-trnF intergenic spacer, partial sequence; chloroplast	800	800	98% 0.0	96% DQ667473.1	783,67	84,7%
Select seq gb|AY570496.1|	Salvia summa voucher JBW 1972 tRNA-Leu and trnL-trnF intergenic spacer, partial sequence; chloroplast	804	804	99% 0.0	96% AY570496.1	779,64	84,3%
Select seq gb|AY570490.1|	Salvia pratensis voucher JBW 2523 tRNA-Leu and trnL-trnF intergenic spacer, partial sequence; chloroplast	802	802	99% 0.0	96% AY570490.1	777,70	84,1%
Select seq gb|DQ667525.1|	Zhumeria majdae isolate x575 tRNA-Leu (trnL) gene and trnL-trnF intergenic spacer, partial sequence; chloroplast	808	808	99% 0.0	95% DQ667525.1	775,35	83,8%

Select seq gb|KC414276.1|	Mentha canadensis isolate 511190001 tRNA-Leu (trnL) gene and trnL-trnF intergenic spacer, partial sequence; chloroplast	804	804	99% 0.0	95% KC414276.1	771,52	83,4%
Select seq gb|DQ667444.1|	Salvia daghestanica isolate x118 tRNA-Leu (trnL) gene and trnL-trnF intergenic spacer, partial sequence; chloroplast	795	795	98% 0.0	95% DQ667444.1	770,66	83,3%
Select seq gb|DQ667524.1|	Zhumeria majdae isolate x573 tRNA-Leu (trnL) gene and trnL-trnF intergenic spacer, partial sequence; chloroplast	802	802	99% 0.0	95% DQ667524.1	769,60	83,2%
Select seq gb|EU200181.1|	Salvia santolinifolia tRNA-Leu (trnL) gene and trnL-trnF intergenic spacer, partial sequence; chloroplast	798	798	99% 0.0	95% EU200181.1	765,76	82,8%
Select seq gb|DQ667476.1|	Salvia santolinifolia isolate x191 tRNA-Leu (trnL) gene and trnL-trnF intergenic spacer, partial sequence; chloroplast	798	798	99% 0.0	95% DQ667476.1	765,76	82,8%
Select seq gb|KR063657.1|	Thymus sibthorpii tRNA-Leu (trnL) gene, partial sequence; trnL-trnF intergenic spacer, complete sequence; and tRNA-Phe (trnF) gene, partial sequence; chloroplast	797	797	99% 0.0	95% KR063657.1	764,80	82,7%
Select seq gb|KR150243.1|	Ziziphora sp. 2 Kh20 trnL-trnF intergenic spacer region, partial sequence; chloroplast	797	797	99% 0.0	95% KR150243.1	764,80	82,7%
Select seq gb|KR150218.1|	Thymus sp. 2 Kh69 trnL-trnF intergenic spacer region, partial sequence; chloroplast	797	797	99% 0.0	95% KR150218.1	764,80	82,7%
Select seq gb|JX880022.1|	Origanum vulgare subsp. vulgare chloroplast, complete genome	797	797	99% 0.0	95% JX880022.1	764,80	82,7%
Select seq gb|JQ690290.1|	Origanum elongatum isolate H5_O_elo trnL-trnF intergenic spacer, partial sequence; chloroplast	797	797	99% 0.0	95% JQ690290.1	764,80	82,7%
Select seq gb|JQ690289.1|	Origanum rotundifolium isolate DNA3_O_rot trnL-trnF intergenic spacer, partial sequence; chloroplast	797	797	99% 0.0	95% JQ690289.1	764,80	82,7%
Select seq gb|AY570482.1|	Salvia henryi voucher JBW 2516 tRNA-Leu and trnL-trnF intergenic spacer, partial sequence; chloroplast	797	797	99% 0.0	95% AY570482.1	764,80	82,7%
Select seq gb|KR063656.1|	Satureja pilosa subsp. origanita tRNA-Leu (trnL) gene, partial sequence; trnL-trnF intergenic spacer, complete sequence; and tRNA-Phe (trnF) gene, partial sequence; chloroplast	795	795	99% 0.0	95% KR063656.1	762,88	82,5%
Select seq gb|KR150220.1|	Hymenocrater sp. 1 Kh67 trnL-trnF intergenic spacer region, partial sequence; chloroplast	795	795	99% 0.0	95% KR150220.1	762,88	82,5%
Select seq emb|AJ505543.1|	Origanum vulgare plastid trnL-trnF intergenic spacer, specimen voucher cult., K-000-69-19317, chase 13334 (K)	793	793	99% 0.0	95% AJ505543.1	760,96	82,3%
Select seq emb|AJ505541.1|	Mentha suaveolens plastid trnL-trnF intergenic spacer, specimen voucher cult., K-1970-3169 (K)	793	793	99% 0.0	95% AJ505541.1	760,96	82,3%
Select seq gb|KR150238.1|	Ziziphora sp. 1 Kh75 trnL-trnF intergenic spacer region, partial sequence; chloroplast	791	791	99% 0.0	95% KR150238.1	759,04	82,1%

Select for downloading or viewing reports	Kh075_trnL Description	Max score	Total score	Query cover	E value	Ident	Accession	(Ident/Cover)* Max score	Deviation from top hit
Select seq gb|KP265673.1|	Ziziphora taurica subsp. cleonioides tRNA-Leu (trnL) gene and trnL-trnF intergenic spacer, partial sequence; chloroplast	1147	1147	92%	0.0	99%	KP265673.1	1234,27	100,0%
Select seq gb|KP265671.1|	Ziziphora serpyllacea tRNA-Leu (trnL) gene and trnL-trnF intergenic spacer, partial sequence; chloroplast	1147	1147	92%	0.0	99%	KP265671.1	1234,27	100,0%
Select seq gb|KP265670.1|	Ziziphora rigida tRNA-Leu (trnL) gene and trnL-trnF intergenic spacer, partial sequence; chloroplast	1147	1147	92%	0.0	99%	KP265670.1	1234,27	100,0%
Select seq gb|KP265669.1|	Ziziphora raddei tRNA-Leu (trnL) gene and trnL-trnF intergenic spacer, partial sequence; chloroplast	1147	1147	92%	0.0	99%	KP265669.1	1234,27	100,0%
Select seq gb|KP265666.1|	Ziziphora clinopodioides subsp. ronningeri tRNA-Leu (trnL) gene, partial sequence; trnL-trnF intergenic spacer, complete sequence; and tRNA-Phe (trnF) gene, partial sequence; chloroplast	1147	1147	92%	0.0	99%	KP265666.1	1234,27	100,0%
Select seq gb|KP265662.1|	Ziziphora clinopodioides subsp. filicaulis tRNA-Leu (trnL) gene, partial sequence; trnL-trnF intergenic spacer, complete sequence; and tRNA-Phe (trnF) gene, partial sequence; chloroplast	1147	1147	92%	0.0	99%	KP265662.1	1234,27	100,0%
Select seq gb|KP265661.1|	Ziziphora clinopodioides subsp. elbursensis tRNA-Leu (trnL) gene and trnL-trnF intergenic spacer, partial sequence; chloroplast	1147	1147	92%	0.0	99%	KP265661.1	1234,27	100,0%
Select seq gb|KP265659.1|	Ziziphora clinopodioides subsp. afghanica tRNA-Leu (trnL) gene, partial sequence; trnL-trnF intergenic spacer, complete sequence; and tRNA-Phe (trnF) gene, partial sequence; chloroplast	1147	1147	92%	0.0	99%	KP265659.1	1234,27	100,0%
Select seq gb|KP265658.1|	Ziziphora biebersteiniana trnL-trnF intergenic spacer, partial sequence; chloroplast	1147	1147	92%	0.0	99%	KP265658.1	1234,27	100,0%
Select seq gb|DQ667501.1|	Ziziphora taurica isolate x262 tRNA-Leu (trnL) gene and trnL-trnF intergenic spacer, partial sequence; chloroplast	1221	1221	98%	0.0	99%	DQ667501.1	1233,46	99,9%
Select seq gb|JQ669021.1|	Clinopodium acinos voucher Judziewicz 14160 tRNA-Leu (trnL) gene and trnL-trnF intergenic spacer, partial sequence; plastid	1208	1208	97%	0.0	99%	JQ669021.1	1232,91	99,9%
Select seq gb|GU381510.1|	Ziziphora pamiroalaica voucher C:Murray et al. 10090 tRNA-Leu (trnL) gene and trnL-trnF intergenic spacer, partial sequence; chloroplast	1219	1219	98%	0.0	99%	GU381510.1	1231,44	99,8%
Select seq gb|GU381505.1|	Clinopodium troodi voucher W:Davis 1856 tRNA-Leu (trnL) gene and trnL-trnF intergenic spacer, partial sequence; chloroplast	1219	1219	98%	0.0	99%	GU381505.1	1231,44	99,8%
Select seq gb|GU381501.1|	Clinopodium graveolens subsp. rotundifolium voucher M:Podlech 47181 tRNA-Leu (trnL) gene and trnL-trnF intergenic spacer, partial sequence; chloroplast	1219	1219	98%	0.0	99%	GU381501.1	1231,44	99,8%
Select seq gb|GU381499.1|	Clinopodium suaveolens voucher M:Erben s.n. tRNA-Leu (trnL) gene and trnL-trnF intergenic spacer, partial sequence; chloroplast	1219	1219	98%	0.0	99%	GU381499.1	1231,44	99,8%
Select seq gb|GU381500.1|	Clinopodium nanum voucher M:Braeuchler 2796 tRNA-Leu (trnL) gene and trnL-trnF intergenic spacer, partial sequence; chloroplast	1206	1206	97%	0.0	99%	GU381500.1	1230,87	99,7%
Select seq gb|AY506595.1|	Ziziphora hispanica tRNA-Leu (trnL) gene and trnL-trnF intergenic spacer, partial sequence; chloroplast	1181	1181	95%	0.0	99%	AY506595.1	1230,73	99,7%
Select seq gb|GU381502.1|	Ziziphora hispanica subsp. aragonensis voucher M:Podlech & Lippert 2492 tRNA-Leu (trnL) gene and trnL-trnF intergenic spacer, partial sequence; chloroplast	1192	1192	96%	0.0	99%	GU381502.1	1229,25	99,6%
Select seq gb|KP265668.1|	Ziziphora puschkinii tRNA-Leu (trnL) gene and trnL-trnF intergenic spacer, partial sequence; chloroplast	1142	1142	92%	0.0	99%	KP265668.1	1228,89	99,6%
Select seq gb|KP265667.1|	Ziziphora clinopodioides tRNA-Leu (trnL) gene and trnL-trnF intergenic spacer, partial sequence; chloroplast	1142	1142	92%	0.0	99%	KP265667.1	1228,89	99,6%
Select seq gb|KP265665.1|	Ziziphora clinopodioides subsp. rigida tRNA-Leu (trnL) gene, partial sequence; trnL-trnF intergenic spacer, complete sequence; and tRNA-Phe (trnF) gene, partial sequence; chloroplast	1142	1142	92%	0.0	99%	KP265665.1	1228,89	99,6%
Select seq gb|KP265664.1|	Ziziphora clinopodioides subsp. pseudodasyantha tRNA-Leu (trnL) gene and trnL-trnF intergenic spacer, partial sequence; chloroplast	1142	1142	92%	0.0	99%	KP265664.1	1228,89	99,6%
Select seq gb|KP265663.1|	Ziziphora clinopodioides subsp. kurdica tRNA-Leu (trnL) gene, partial sequence; trnL-trnF intergenic spacer, complete sequence; and tRNA-Phe (trnF) gene, partial sequence; chloroplast	1142	1142	92%	0.0	99%	KP265663.1	1228,89	99,6%
Select seq gb|KP265660.1|	Ziziphora clinopodioides subsp. bungeana tRNA-Leu (trnL) gene, partial sequence; trnL-trnF intergenic spacer, complete sequence; and tRNA-Phe (trnF) gene, partial sequence; chloroplast	1142	1142	92%	0.0	99%	KP265660.1	1228,89	99,6%
Select seq gb|GU381507.1|	Ziziphora tenuior voucher MSB:Fayvush et al. 03-1503 tRNA-Leu (trnL) gene and trnL-trnF intergenic spacer, partial sequence; chloroplast	1214	1214	98%	0.0	99%	GU381507.1	1226,39	99,4%
Select seq gb|GU381497.1|	Clinopodium acinos voucher M:Podlech 50287 tRNA-Leu (trnL) gene and trnL-trnF intergenic spacer, partial sequence; chloroplast	1214	1214	98%	0.0	99%	GU381497.1	1226,39	99,4%

Select seq gb|JF301402.1|	Ziziphora clinopodioides voucher DBG 980177 tRNA-Leu (trnL) gene and trnL-trnF intergenic spacer, partial sequence; chloroplast	1188	1188	96% 0.0	99% JF301402.1	1225,13	99,3%
Select seq gb|GU381506.1|	Ziziphora tenuior voucher MSB:Nydegger 43557 tRNA-Leu (trnL) gene and trnL-trnF intergenic spacer, partial sequence; chloroplast	1186	1186	96% 0.0	99% GU381506.1	1223,06	99,1%
Select seq gb|GU381503.1|	Ziziphora hispanica voucher M:Lippert 24827 tRNA-Leu (trnL) gene and trnL-trnF intergenic spacer, partial sequence; chloroplast	1186	1186	96% 0.0	99% GU381503.1	1223,06	99,1%
Select seq gb|KP265672.1|	Ziziphora taurica subsp. taurica tRNA-Leu (trnL) gene and trnL-trnF intergenic spacer, partial sequence; chloroplast	1136	1136	92% 0.0	99% KP265672.1	1222,43	99,0%
Select seq gb|AY840180.1|	Clinopodium alpinum tRNA-Leu (trnL) gene and trnL-trnF intergenic spacer, partial sequence; chloroplast	1182	1182	96% 0.0	99% AY840180.1	1218,94	98,8%
Select seq gb|GU381517.1|	Clinopodium barosmum voucher BM<GBR-LONDON>:McLaren N193 tRNA-Leu (trnL) gene and trnL-trnF intergenic spacer, partial sequence; chloroplast	1197	1197	98% 0.0	99% GU381517.1	1209,21	98,0%
Select seq gb|GU381516.1|	Clinopodium wardii voucher BM<GBR-LONDON>:Ludlow et al. 14234 tRNA-Leu (trnL) gene and trnL-trnF intergenic spacer, partial sequence; chloroplast	1197	1197	98% 0.0	99% GU381516.1	1209,21	98,0%
Select seq gb|GU381515.1|	Clinopodium hydaspidis voucher BM<GBR-LONDON>:Mohd 133 tRNA-Leu (trnL) gene and trnL-trnF intergenic spacer, partial sequence; chloroplast	1197	1197	98% 0.0	99% GU381515.1	1209,21	98,0%
Select seq gb|GU381514.1|	Clinopodium nepalense voucher FR:Stainton 6024 tRNA-Leu (trnL) gene and trnL-trnF intergenic spacer, partial sequence; chloroplast	1197	1197	98% 0.0	99% GU381514.1	1209,21	98,0%
Select seq gb|GU381511.1|	Clinopodium piperitum voucher BM<GBR-LONDON>:Vickery 454 tRNA-Leu (trnL) gene and trnL-trnF intergenic spacer, partial sequence; chloroplast	1197	1197	98% 0.0	99% GU381511.1	1209,21	98,0%
Select seq gb|GU381513.1|	Clinopodium nepalense voucher BM<GBR-LONDON>:Mikage et al. 9550294 tRNA-Leu (trnL) gene and trnL-trnF intergenic spacer, partial sequence; chloroplast	1170	1170	96% 0.0	99% GU381513.1	1206,56	97,8%
Select seq gb|GU381512.1|	Clinopodium piperitum voucher E:Stainton 7320 tRNA-Leu (trnL) gene and trnL-trnF intergenic spacer, partial sequence; chloroplast	1192	1192	98% 0.0	99% GU381512.1	1204,16	97,6%
Select seq gb|GU381509.1|	Ziziphora capitata voucher M:Braeuchler 3142 tRNA-Leu (trnL) gene and trnL-trnF intergenic spacer, partial sequence; chloroplast	1168	1168	98% 0.0	99% GU381509.1	1179,92	95,6%
Select seq gb|GU381484.1|	Micromeria flagellaris voucher E:van der Werff & McPherson 13570 tRNA-Leu (trnL) gene and trnL-trnF intergenic spacer, partial sequence; chloroplast	1170	1170	98% 0.0	98% GU381484.1	1170,00	94,8%
Select seq gb|GU381483.1|	Micromeria flagellaris voucher E:Clement et al 2140 tRNA-Leu (trnL) gene and trnL-trnF intergenic spacer, partial sequence; chloroplast	1170	1170	98% 0.0	98% GU381483.1	1170,00	94,8%
Select seq gb|AY506594.1|	Acinos alpinus tRNA-Leu (trnL) gene and trnL-trnF intergenic spacer, partial sequence; chloroplast	1127	1127	95% 0.0	98% AY506594.1	1162,59	94,2%
Select seq gb|GU381629.1|	Thymbra capitata voucher M:Braeuchler 2518 tRNA-Leu (trnL) gene and trnL-trnF intergenic spacer, partial sequence; chloroplast	1153	1153	98% 0.0	98% GU381629.1	1153,00	93,4%
Select seq gb|JF301401.1|	Thymbra capitata voucher UCBG 96.0817 tRNA-Leu (trnL) gene and trnL-trnF intergenic spacer, partial sequence; chloroplast	1127	1127	96% 0.0	98% JF301401.1	1150,48	93,2%
Select seq gb|GU381496.1|	Killickia pilosa voucher M:Braeuchler 3832 tRNA-Leu (trnL) gene and trnL-trnF intergenic spacer, partial sequence; chloroplast	1149	1149	98% 0.0	98% GU381496.1	1149,00	93,1%
Select seq gb|GU381493.1|	Killickia grandiflora voucher M:Braeuchler 3811 tRNA-Leu (trnL) gene and trnL-trnF intergenic spacer, partial sequence; chloroplast	1149	1149	98% 0.0	98% GU381493.1	1149,00	93,1%
Select seq gb|GU381481.1|	Micromeria cf. madagascariensis Morawetz 205 tRNA-Leu (trnL) gene and trnL-trnF intergenic spacer, partial sequence; chloroplast	1147	1147	98% 0.0	98% GU381481.1	1147,00	92,9%
Select seq gb|AY840207.1|	Thymbra spicata tRNA-Leu (trnL) gene and trnL-trnF intergenic spacer, partial sequence; chloroplast	1147	1147	98% 0.0	98% AY840207.1	1147,00	92,9%
Select seq gb|GU381485.1|	Micromeria sphaerophylla voucher E:Lewis et al 1064 tRNA-Leu (trnL) gene and trnL-trnF intergenic spacer, partial sequence; chloroplast	1146	1146	98% 0.0	98% GU381485.1	1146,00	92,8%
Select seq gb|GU381495.1|	Killickia pilosa voucher M:Braeuchler 3810 tRNA-Leu (trnL) gene and trnL-trnF intergenic spacer, partial sequence; chloroplast	1134	1134	97% 0.0	98% GU381495.1	1145,69	92,8%
Select seq gb|GU381632.1|	Thymbra spicata voucher M:Braeuchler 4548 tRNA-Leu (trnL) gene and trnL-trnF intergenic spacer, partial sequence; chloroplast	1144	1144	98% 0.0	98% GU381632.1	1144,00	92,7%
Select seq gb|GU381627.1|	Thymbra sintenisii subsp. isaurica voucher E:Goener 12628 tRNA-Leu (trnL) gene and trnL-trnF intergenic spacer, partial sequence; chloroplast	1142	1142	98% 0.0	98% GU381627.1	1142,00	92,5%
Select seq gb|GU381489.1|	Killickia lutea voucher NU<ZAF>:Hilliard & Burtt 9876 tRNA-Leu (trnL) gene and trnL-trnF intergenic spacer, partial sequence; chloroplast	1138	1138	98% 0.0	98% GU381489.1	1138,00	92,2%
Select seq gb|GU381488.1|	Killickia compacta voucher M:Braeuchler 3816 tRNA-Leu (trnL) gene and trnL-trnF intergenic spacer, partial sequence; chloroplast	1138	1138	98% 0.0	98% GU381488.1	1138,00	92,2%

Select seq gb|GU381633.1|	Thymus caespititius voucher M:Heubl s.n. tRNA-Leu (trnL) gene and trnL-trnF intergenic spacer, partial sequence; chloroplast	1131	1131	98%	0.0	97%	GU381633.1	1119,46	90,7%
Select seq gb|AY840202.1|	Origanum vulgare tRNA-Leu (trnL) gene and trnL-trnF intergenic spacer, partial sequence; chloroplast	1131	1131	98%	0.0	97%	AY840202.1	1119,46	90,7%
Select seq gb|JX880022.1|	Origanum vulgare subsp. vulgare chloroplast, complete genome	1153	1153	100%	0.0	97%	JX880022.1	1118,41	90,6%
Select seq gb|JQ690290.1|	Origanum elongatum isolate H5_O_elo trnL-trnF intergenic spacer, partial sequence; chloroplast	1153	1153	100%	0.0	97%	JQ690290.1	1118,41	90,6%
Select seq gb|JQ690289.1|	Origanum rotundifolium isolate DNA3_O_rot trnL-trnF intergenic spacer, partial sequence; chloroplast	1153	1153	100%	0.0	97%	JQ690289.1	1118,41	90,6%
Select seq emb|AJ505543.1|	Origanum vulgare plastid trnL-trnF intergenic spacer, specimen voucher cult., K-000-69-19317, chase 13334 (K)	1153	1153	100%	0.0	97%	AJ505543.1	1118,41	90,6%
Select seq gb|KR150229.1|	Thymus sp. 5 Kh16 trnL-trnF intergenic spacer region, partial sequence; chloroplast	1138	1138	99%	0.0	97%	KR150229.1	1115,01	90,3%
Select seq gb|KR150218.1|	Thymus sp. 2 Kh69 trnL-trnF intergenic spacer region, partial sequence; chloroplast	1138	1138	99%	0.0	97%	KR150218.1	1115,01	90,3%
Select seq gb|JQ669069.1|	Thymus pulegioides voucher Riina 1577 tRNA-Leu (trnL) gene and trnL-trnF intergenic spacer, partial sequence; plastid	1114	1114	97%	0.0	97%	JQ669069.1	1114,00	90,3%
Select seq gb|GU381640.1|	Argantoniella salzmannii voucher M:Barra et al. 2673GL tRNA-Leu (trnL) gene and trnL-trnF intergenic spacer, partial sequence; chloroplast	1125	1125	98%	0.0	97%	GU381640.1	1113,52	90,2%
Select seq gb|AY570463.1|	Origanum vulgare voucher JBW 2567 tRNA-Leu and trnL-trnF intergenic spacer, partial sequence; chloroplast	1123	1123	98%	0.0	97%	AY570463.1	1111,54	90,1%
Select seq gb|KR150225.1|	Thymus sp. 4 Kh24 trnL-trnF intergenic spacer region, partial sequence; chloroplast	1133	1133	99%	0.0	97%	KR150225.1	1110,11	89,9%
Select seq gb|KR150222.1|	Thymus sp. 3 Kh65 trnL-trnF intergenic spacer region, partial sequence; chloroplast	1133	1133	99%	0.0	97%	KR150222.1	1110,11	89,9%
Select seq gb|GU381635.1|	Thymus broussonetii subsp. hannonis voucher MSB:Podlech 142 tRNA-Leu (trnL) gene and trnL-trnF intergenic spacer, partial sequence; chloroplast	1120	1120	98%	0.0	97%	GU381635.1	1108,57	89,8%
Select seq gb|GU381631.1|	Satureja linearifolia voucher M:Brullo & Furnari s.n. tRNA-Leu (trnL) gene and trnL-trnF intergenic spacer, partial sequence; chloroplast	1120	1120	98%	0.0	97%	GU381631.1	1108,57	89,8%
Select seq gb|KR063657.1|	Thymus sibthorpii tRNA-Leu (trnL) gene, partial sequence; trnL-trnF intergenic spacer, complete sequence; and tRNA-Phe (trnF) gene, partial sequence; chloroplast	1142	1142	100%	0.0	97%	KR063657.1	1107,74	89,7%
Select seq gb|JQ690293.1|	Origanum dayi isolate H43_O_day trnL-trnF intergenic spacer, partial sequence; chloroplast	1142	1142	100%	0.0	97%	JQ690293.1	1107,74	89,7%
Select seq gb|EU556532.1|	Thymus amurensis isolate HX tRNA-Leu (trnL) gene and trnL-trnF intergenic spacer, partial sequence; chloroplast	1142	1142	100%	0.0	97%	EU556532.1	1107,74	89,7%
Select seq gb|EU556525.1|	Thymus quinquecostatus isolate CL tRNA-Leu (trnL) gene and trnL-trnF intergenic spacer, partial sequence; chloroplast	1142	1142	100%	0.0	97%	EU556525.1	1107,74	89,7%
Select seq gb|KR150209.1|	Satureja sp. 3 Kh90 trnL-trnF intergenic spacer region, partial sequence; chloroplast	1116	1116	98%	0.0	97%	KR150209.1	1104,61	89,5%
Select seq gb|GU381628.1|	Thymbra calostachya voucher M:Ulrich s.n. tRNA-Leu (trnL) gene and trnL-trnF intergenic spacer, partial sequence; chloroplast	1116	1116	98%	0.0	97%	GU381628.1	1104,61	89,5%
Select seq gb|GU381621.1|	Satureja cuneifolia voucher M:Rechinger 11142 tRNA-Leu (trnL) gene and trnL-trnF intergenic spacer, partial sequence; chloroplast	1116	1116	98%	0.0	97%	GU381621.1	1104,61	89,5%
Select seq gb|GU381612.1|	Gontscharovia popovii voucher BM<GBR-LONDON>:Schmid 2419 tRNA-Leu (trnL) gene and trnL-trnF intergenic spacer, partial sequence; chloroplast	1116	1116	98%	0.0	97%	GU381612.1	1104,61	89,5%
Select seq gb|GU381611.1|	Gontscharovia popovii voucher M:Vvedensky s.n. tRNA-Leu (trnL) gene and trnL-trnF intergenic spacer, partial sequence; chloroplast	1116	1116	98%	0.0	97%	GU381611.1	1104,61	89,5%
Select seq gb|AY840179.1|	Satureja montana tRNA-Leu (trnL) gene and trnL-trnF intergenic spacer, partial sequence; chloroplast	1116	1116	98%	0.0	97%	AY840179.1	1104,61	89,5%
Select seq gb|KR063656.1|	Satureja pilosa subsp. origanita tRNA-Leu (trnL) gene, partial sequence; trnL-trnF intergenic spacer, complete sequence; and tRNA-Phe (trnF) gene, partial sequence; chloroplast	1138	1138	100%	0.0	97%	KR063656.1	1103,86	89,4%
Select seq gb|EU556539.1|	Thymus quinquecostatus isolate ZY tRNA-Leu (trnL) gene and trnL-trnF intergenic spacer, partial sequence; chloroplast	1134	1134	100%	0.0	97%	EU556539.1	1099,98	89,1%
Select seq gb|EU556538.1|	Thymus quinquecostatus isolate ZJ tRNA-Leu (trnL) gene and trnL-trnF intergenic spacer, partial sequence; chloroplast	1134	1134	100%	0.0	97%	EU556538.1	1099,98	89,1%

Select seq gb|EU556533.1|	Thymus quinquecostatus isolate HY tRNA-Leu (trnL) gene and trnL-trnF intergenic spacer, partial sequence; chloroplast	1134	1134	100% 0.0	97% EU556533.1	1099,98	89,1%
Select seq gb|EU556531.1|	Thymus amurensis isolate HW tRNA-Leu (trnL) gene and trnL-trnF intergenic spacer, partial sequence; chloroplast	1134	1134	100% 0.0	97% EU556531.1	1099,98	89,1%
Select seq gb|EU556527.1|	Thymus mongolicus isolate G2 tRNA-Leu (trnL) gene and trnL-trnF intergenic spacer, partial sequence; chloroplast	1134	1134	100% 0.0	97% EU556527.1	1099,98	89,1%
Select seq gb|EU556526.1|	Thymus quinquecostatus isolate D2 tRNA-Leu (trnL) gene and trnL-trnF intergenic spacer, partial sequence; chloroplast	1134	1134	100% 0.0	97% EU556526.1	1099,98	89,1%
Select seq gb|DQ667513.1|	Clinopodium vulgare isolate x423 tRNA-Leu (trnL) gene and trnL-trnF intergenic spacer, partial sequence; chloroplast	1133	1133	100% 0.0	97% DQ667513.1	1099,01	89,0%
Select seq gb|DQ667504.1|	Cunila incana isolate x296 tRNA-Leu (trnL) gene and trnL-trnF intergenic spacer, partial sequence; chloroplast	1127	1127	100% 0.0	97% DQ667504.1	1093,19	88,6%
Select seq gb|KC414276.1|	Mentha canadensis isolate 511190001 tRNA-Leu (trnL) gene and trnL-trnF intergenic spacer, partial sequence; chloroplast	1125	1125	100% 0.0	97% KC414276.1	1091,25	88,4%
Select seq emb|AJ505547.1|	Clinopodium vulgare subsp. arundanum plastid trnL-trnF intergenic spacer, specimen voucher cult., K-453-79-04649	1125	1125	100% 0.0	97% AJ505547.1	1091,25	88,4%
Select seq emb|AJ505544.1|	Thymus serpyllum var. citriodorum plastid trnL-trnF intergenic spacer, specimen voucher cult., K-1975-1177, Chase 13331 (K)	1123	1123	100% 0.0	97% AJ505544.1	1089,31	88,3%
Select seq emb|AJ505541.1|	Mentha suaveolens plastid trnL-trnF intergenic spacer, specimen voucher cult., K-1970-3169 (K)	1123	1123	100% 0.0	97% AJ505541.1	1089,31	88,3%
Select seq gb|DQ667507.1|	Pycnanthemum virginianum isolate x313 tRNA-Leu (trnL) gene and trnL-trnF intergenic spacer, partial sequence; chloroplast	1122	1122	100% 0.0	97% DQ667507.1	1088,34	88,2%
Select seq gb|DQ667497.1|	Hoehnea epilobioides isolate x258 tRNA-Leu (trnL) gene and trnL-trnF intergenic spacer, partial sequence; chloroplast	1122	1122	100% 0.0	97% DQ667497.1	1088,34	88,2%
Select seq gb|DQ667492.1|	Lepechinia lancifolia isolate x232 tRNA-Leu (trnL) gene and trnL-trnF intergenic spacer, partial sequence; chloroplast	1116	1116	100% 0.0	97% DQ667492.1	1082,52	87,7%
Select seq gb|DQ667436.1|	Hedeoma costata isolate x067 tRNA-Leu (trnL) gene and trnL-trnF intergenic spacer, partial sequence; chloroplast	1116	1116	100% 0.0	97% DQ667436.1	1082,52	87,7%
Select seq gb|FJ593456.1|	Mentha sp. JSZ-2009a tRNA-Leu (trnL) gene and trnL-trnF intergenic spacer, partial sequence; chloroplast	1118	1118	100% 0.0	96% FJ593456.1	1073,28	87,0%
						0,00	0,0%
						0,00	0,0%

Select for downloading or viewing reports	Kh076_ITS Description	Max score	Total score	Query cover	E value	Ident	Accession	(Ident/Cover)* Max score	Deviation from top hit
Select seq gb|JF907423.1|	Tripleurospermum maritimum subsp. inodorum voucher OSBU:2005 DE-0-OSN-2005-169 18S ribosomal RNA gene, partial sequence; internal transcribed spacer 1, 5.8S ribosomal RNA gene, and internal transcribed spacer 2, complete sequence; and 26S ribosomal RNA gene, partial sequence	1046	1046	100%	0.0	96%	JF907423.1	1004,16	100,0%
Select seq dbj|AB683287.1|	Tanacetum haradjanii gene for ITS1, 5.8S rRNA and ITS2, complete sequence, specimen_voucher: 16366 (M)	907	907	96%	0.0	93%	AB683287.1	878,66	87,5%
Select seq dbj|AB683260.1|	Tanacetum abrotanifolium gene for ITS1, 5.8S rRNA and ITS2, complete sequence, specimen_voucher: 68865 (TARI)	902	902	96%	0.0	93%	AB683260.1	873,81	87,0%
Select seq dbj|AB683267.1|	Tanacetum armenum gene for ITS1, 5.8S rRNA and ITS2, complete sequence, specimen_voucher: (W, 1972-03416)	900	900	96%	0.0	93%	AB683267.1	871,88	86,8%
Select seq gb|AY603184.1|	Achillea pannonica internal transcribed spacer 1, 5.8S ribosomal RNA gene, and internal transcribed spacer 2, complete sequence	898	898	96%	0.0	93%	AY603184.1	869,94	86,6%
Select seq gb|EF577320.1|	Tanacetum parthenium 18S ribosomal RNA gene, partial sequence; internal transcribed spacer 1, 5.8S ribosomal RNA gene, and internal transcribed spacer 2, complete sequence; and 28S ribosomal RNA gene, partial sequence	935	935	100%	0.0	93%	EF577320.1	869,55	86,6%
Select seq dbj|AB683285.1|	Tanacetum germanicopolitanum gene for ITS1, 5.8S rRNA and ITS2, complete sequence, specimen_voucher: 14254 (S)	896	896	96%	0.0	93%	AB683285.1	868,00	86,4%
Select seq dbj|AB683265.1|	Tanacetum argenteum subsp. canum gene for ITS1, 5.8S rRNA and ITS2, complete sequence, specimen_voucher: 46856 (B)	894	894	96%	0.0	93%	AB683265.1	866,06	86,2%
Select seq gb|AY603190.1|	Achillea styriaca internal transcribed spacer 1, 5.8S ribosomal RNA gene, and internal transcribed spacer 2, complete sequence	894	894	96%	0.0	93%	AY603190.1	866,06	86,2%
Select seq gb|AY603230.1|	Achillea clavennae internal transcribed spacer 1, 5.8S ribosomal RNA gene, and internal transcribed spacer 2, complete sequence	893	893	96%	0.0	93%	AY603230.1	865,09	86,2%
Select seq gb|AY603202.1|	Achillea euxina internal transcribed spacer 1, 5.8S ribosomal RNA gene, and internal transcribed spacer 2, complete sequence	893	893	96%	0.0	93%	AY603202.1	865,09	86,2%
Select seq gb|AY603187.1|	Achillea millefolium subsp. sudetica internal transcribed spacer 1, 5.8S ribosomal RNA gene, and internal transcribed spacer 2, complete sequence	893	893	96%	0.0	93%	AY603187.1	865,09	86,2%
Select seq gb|EF577322.1|	Tanacetum ptarmiciflorum 18S ribosomal RNA gene, partial sequence; internal transcribed spacer 1, 5.8S ribosomal RNA gene, and internal transcribed spacer 2, complete sequence; and 28S ribosomal RNA gene, partial sequence	929	929	100%	0.0	93%	EF577322.1	863,97	86,0%
Select seq dbj|AB683313.1|	Tanacetum punctatum gene for ITS1, 5.8S rRNA and ITS2, complete sequence, specimen_voucher: 49833 (IRAN)	891	891	96%	0.0	93%	AB683313.1	863,16	86,0%
Select seq dbj|AB683304.1|	Tanacetum pinnatum gene for ITS1, 5.8S rRNA and ITS2, complete sequence, specimen_voucher: 1118 (MPH)	891	891	96%	0.0	93%	AB683304.1	863,16	86,0%
Select seq emb|FN823080.1|	Tanacetum parthenium genomic DNA containing ITS1, 5.8S rRNA gene, ITS2, specimen voucher MPH:1119	891	891	96%	0.0	93%	FN823080.1	863,16	86,0%
Select seq emb|FN823079.1|	Tanacetum chiliophyllum genomic DNA containing ITS1, 5.8S rRNA gene, ITS2, specimen voucher MPH:1124	891	891	96%	0.0	93%	FN823079.1	863,16	86,0%
Select seq gb|AY603205.1|	Achillea lanulosa subsp. alpicola internal transcribed spacer 1, 5.8S ribosomal RNA gene, and internal transcribed spacer 2, complete sequence	891	891	96%	0.0	93%	AY603205.1	863,16	86,0%
Select seq dbj|AB608331.1|	Tanacetum canescens genes for ITS1, 5.8S rRNA, ITS2, complete sequence	889	889	96%	0.0	93%	AB608331.1	861,22	85,8%
Select seq gb|AY603186.1|	Achillea millefolium internal transcribed spacer 1, 5.8S ribosomal RNA gene, and internal transcribed spacer 2, complete sequence	889	889	96%	0.0	93%	AY603186.1	861,22	85,8%
Select seq gb|EF577323.1|	Tanacetum vulgare 18S ribosomal RNA gene, partial sequence; internal transcribed spacer 1, 5.8S ribosomal RNA gene, and internal transcribed spacer 2, complete sequence; and 28S ribosomal RNA gene, partial sequence	924	924	100%	0.0	93%	EF577323.1	859,32	85,6%
Select seq gb|EU796891.1|	Achillea millefolium 18S ribosomal RNA gene, partial sequence; internal transcribed spacer 1, 5.8S ribosomal RNA gene, and internal transcribed spacer 2, complete sequence; and 26S ribosomal RNA gene, partial sequence	924	924	100%	0.0	93%	EU796891.1	859,32	85,6%
Select seq dbj|AB683290.1|	Tanacetum khorassanicum gene for ITS1, 5.8S rRNA and ITS2, complete sequence, specimen_voucher: 4937 (S)	887	887	96%	0.0	93%	AB683290.1	859,28	85,6%
Select seq dbj|AB683282.1|	Tanacetum elbursense gene for ITS1, 5.8S rRNA and ITS2, complete sequence, specimen_voucher: 1109 (MPH)	887	887	96%	0.0	93%	AB683282.1	859,28	85,6%
Select seq dbj|AB683278.1|	Tanacetum demetrii gene for ITS1, 5.8S rRNA and ITS2, complete sequence, specimen_voucher: (W, 2001-10597)	887	887	96%	0.0	93%	AB683278.1	859,28	85,6%
Select seq dbj|AB608336.1|	Tanacetum polycephalum genes for ITS1, 5.8S rRNA, ITS2, complete sequence	887	887	96%	0.0	93%	AB608336.1	859,28	85,6%

Select seq gb|AY603229.1|	Achillea clavennae internal transcribed spacer 1, 5.8S ribosomal RNA gene, and internal transcribed spacer 2, complete sequence	887	887	96% 0.0	93% AY603229.1	859,28	85,6%
Select seq gb|AY603212.1|	Achillea nobilis internal transcribed spacer 1, 5.8S ribosomal RNA gene, and internal transcribed spacer 2, complete sequence	887	887	96% 0.0	93% AY603212.1	859,28	85,6%
Select seq gb|AY603197.1|	Achillea setacea internal transcribed spacer 1, 5.8S ribosomal RNA gene, and internal transcribed spacer 2, complete sequence	887	887	96% 0.0	93% AY603197.1	859,28	85,6%
Select seq dbj|AB683303.1|	Tanacetum parthenifolium gene for ITS1, 5.8S rRNA and ITS2, complete sequence, specimen_voucher: 1127 (MPH)	885	885	96% 0.0	93% AB683303.1	857,34	85,4%
Select seq dbj|AB683271.1|	Tanacetum bamianicum gene for ITS1, 5.8S rRNA and ITS2, complete sequence, specimen_voucher: 18238 (B)	885	885	96% 0.0	93% AB683271.1	857,34	85,4%
Select seq gb|AY603264.1|	Tanacetum vulgare internal transcribed spacer 1, 5.8S ribosomal RNA gene, and internal transcribed spacer 2, complete sequence	885	885	96% 0.0	93% AY603264.1	857,34	85,4%
Select seq gb|AY603201.1|	Achillea asplenifolia internal transcribed spacer 1, 5.8S ribosomal RNA gene, and internal transcribed spacer 2, complete sequence	885	885	96% 0.0	93% AY603201.1	857,34	85,4%
Select seq gb|FJ553518.1|	Angiosperm environmental sample clone LTSP_EUKA_P3K07 18S ribosomal RNA, 18S-25/28S ribosomal RNA intergenic spacer, partial sequence	920	920	100% 0.0	93% FJ553518.1	855,60	85,2%
Select seq dbj|AB683323.1|	Tanacetum sonbolii gene for ITS1, 5.8S rRNA and ITS2, complete sequence, specimen_voucher: 998 (MPH)	883	883	96% 0.0	93% AB683323.1	855,41	85,2%
Select seq dbj|AB683270.1|	Tanacetum balsamita gene for ITS1, 5.8S rRNA and ITS2, complete sequence, specimen_voucher: 1126 (MPH)	883	883	96% 0.0	93% AB683270.1	855,41	85,2%
Select seq dbj|AB683268.1|	Tanacetum aucherianum gene for ITS1, 5.8S rRNA and ITS2, complete sequence, specimen_voucher: 44441 (HUB)	883	883	96% 0.0	93% AB683268.1	855,41	85,2%
Select seq gb|AY603204.1|	Achillea ceretanica internal transcribed spacer 1, 5.8S ribosomal RNA gene, and internal transcribed spacer 2, complete sequence	883	883	96% 0.0	93% AY603204.1	855,41	85,2%
Select seq gb|AY603200.1|	Achillea roseoalba internal transcribed spacer 1, 5.8S ribosomal RNA gene, and internal transcribed spacer 2, complete sequence	883	883	96% 0.0	93% AY603200.1	855,41	85,2%
Select seq gb|AY603195.1|	Achillea setacea internal transcribed spacer 1, 5.8S ribosomal RNA gene, and internal transcribed spacer 2, complete sequence	883	883	96% 0.0	93% AY603195.1	855,41	85,2%
Select seq dbj|AB683316.1|	Tanacetum salsugineum gene for ITS1, 5.8S rRNA and ITS2, complete sequence, specimen_voucher: 1407 (MPH)	881	881	96% 0.0	93% AB683316.1	853,47	85,0%
Select seq gb|AY603249.1|	Achillea ptarmicifolia internal transcribed spacer 1, 5.8S ribosomal RNA gene, and internal transcribed spacer 2, complete sequence	881	881	96% 0.0	93% AY603249.1	853,47	85,0%
Select seq gb|AY603232.1|	Achillea fraasii internal transcribed spacer 1, 5.8S ribosomal RNA gene, and internal transcribed spacer 2, complete sequence	881	881	96% 0.0	93% AY603232.1	853,47	85,0%
Select seq gb|AY603222.1|	Achillea holosericea internal transcribed spacer 1, 5.8S ribosomal RNA gene, and internal transcribed spacer 2, complete sequence	881	881	96% 0.0	93% AY603222.1	853,47	85,0%
Select seq gb|AY603211.1|	Achillea virescens internal transcribed spacer 1, 5.8S ribosomal RNA gene, and internal transcribed spacer 2, complete sequence	881	881	96% 0.0	93% AY603211.1	853,47	85,0%
Select seq gb|AY603207.1|	Achillea wilsoniana internal transcribed spacer 1, 5.8S ribosomal RNA gene, and internal transcribed spacer 2, complete sequence	881	881	96% 0.0	93% AY603207.1	853,47	85,0%
Select seq gb|AY603203.1|	Achillea ceretanica internal transcribed spacer 1, 5.8S ribosomal RNA gene, and internal transcribed spacer 2, complete sequence	881	881	96% 0.0	93% AY603203.1	853,47	85,0%
Select seq dbj|AB683289.1|	Tanacetum hololeucum gene for ITS1, 5.8S rRNA and ITS2, complete sequence, specimen_voucher: 6472 (S)	880	880	96% 0.0	93% AB683289.1	852,50	84,9%
Select seq dbj|AB683288.1|	Tanacetum haussknechtii gene for ITS1, 5.8S rRNA and ITS2, complete sequence, specimen_voucher: 1193 (HUB)	880	880	96% 0.0	93% AB683288.1	852,50	84,9%
Select seq gb|AY603263.1|	Tanacetum millefolium internal transcribed spacer 1, 5.8S ribosomal RNA gene, and internal transcribed spacer 2, complete sequence	880	880	96% 0.0	93% AY603263.1	852,50	84,9%
Select seq gb|JQ230971.1|	Achillea millefolium voucher SBB-1156 18S ribosomal RNA gene, partial sequence; internal transcribed spacer 1, 5.8S ribosomal RNA gene, and internal transcribed spacer 2, complete sequence; and 28S ribosomal RNA gene, partial sequence	915	915	100% 0.0	93% JQ230971.1	850,95	84,7%
Select seq gb|AY603254.1|	Achillea wilhelmsii internal transcribed spacer 1, 5.8S ribosomal RNA gene, and internal transcribed spacer 2, complete sequence	878	878	96% 0.0	93% AY603254.1	850,56	84,7%
Select seq gb|EU179214.1|	Anthemis arvensis 18S ribosomal RNA gene, partial sequence; internal transcribed spacer 1, 5.8S ribosomal RNA gene, and internal transcribed spacer 2, complete sequence; and 26S ribosomal RNA gene, partial sequence	904	904	99% 0.0	93% EU179214.1	849,21	84,6%
Select seq gb|EU179216.1|	Anthemis cotula 18S ribosomal RNA gene, partial sequence; internal transcribed spacer 1, 5.8S ribosomal RNA gene, and internal transcribed spacer 2, complete sequence; and 26S ribosomal RNA gene, partial sequence	902	902	99% 0.0	93% EU179216.1	847,33	84,4%

Select seq gb|FJ980358.1|	Achillea wilsoniana voucher PS0715MT02 18S ribosomal RNA gene, partial sequence; internal transcribed spacer 1, 5.8S ribosomal RNA gene, and internal transcribed spacer 2, complete sequence; and 28S ribosomal RNA gene, partial sequence	909	909	100%	0.0	93%	FJ980358.1	845,37	84,2%
Select seq gb|EF577285.1|	Achillea wilsoniana 18S ribosomal RNA gene, partial sequence; internal transcribed spacer 1, 5.8S ribosomal RNA gene, and internal transcribed spacer 2, complete sequence; and 28S ribosomal RNA gene, partial sequence	909	909	100%	0.0	93%	EF577285.1	845,37	84,2%
Select seq dbj|AB608332.1|	Tanacetum chiliophyllum genes for ITS1, 5.8S rRNA, ITS2, complete sequence	880	880	96%	0.0	92%	AB608332.1	843,33	84,0%
Select seq emb|FN823081.1|	Tanacetum coccineum genomic DNA containing ITS1, 5.8S rRNA gene, ITS2, specimen voucher MPH:1144	880	880	96%	0.0	92%	FN823081.1	843,33	84,0%
Select seq gb|AY603228.1|	Achillea clusiana internal transcribed spacer 1, 5.8S ribosomal RNA gene, and internal transcribed spacer 2, complete sequence	880	880	96%	0.0	92%	AY603228.1	843,33	84,0%
Select seq gb|AY603227.1|	Achillea atrata internal transcribed spacer 1, 5.8S ribosomal RNA gene, and internal transcribed spacer 2, complete sequence	880	880	96%	0.0	92%	AY603227.1	843,33	84,0%
Select seq gb|AY603210.1|	Achillea monticola internal transcribed spacer 1, 5.8S ribosomal RNA gene, and internal transcribed spacer 2, complete sequence	880	880	96%	0.0	92%	AY603210.1	843,33	84,0%
Select seq gb|KM887397.1|	Anacyclus pyrethrum isolate SBB-1407 18S ribosomal RNA gene, partial sequence; internal transcribed spacer 1, 5.8S ribosomal RNA gene, and internal transcribed spacer 2, complete sequence; and 28S ribosomal RNA gene, partial sequence	905	905	100%	0.0	93%	KM887397.1	841,65	83,8%
Select seq gb|KM887396.1|	Anacyclus pyrethrum isolate SBB-1364 18S ribosomal RNA gene, partial sequence; internal transcribed spacer 1, 5.8S ribosomal RNA gene, and internal transcribed spacer 2, complete sequence; and 28S ribosomal RNA gene, partial sequence	905	905	100%	0.0	93%	KM887396.1	841,65	83,8%
Select seq gb|AY603214.1|	Achillea ageratum internal transcribed spacer 1, 5.8S ribosomal RNA gene, and internal transcribed spacer 2, complete sequence	876	876	96%	0.0	92%	AY603214.1	839,50	83,6%
Select seq dbj|AB683305.1|	Tanacetum polycephalum subsp. argyrophyllum gene for ITS1, 5.8S rRNA and ITS2, complete sequence, specimen_voucher: 1125 (MPH)	874	874	96%	0.0	92%	AB683305.1	837,58	83,4%
Select seq dbj|AB683269.1|	Tanacetum bachtiaricum gene for ITS1, 5.8S rRNA and ITS2, complete sequence, specimen_voucher: 1174 (MPH)	874	874	96%	0.0	92%	AB683269.1	837,58	83,4%
Select seq dbj|AB608333.1|	Tanacetum coccineum genes for ITS1, 5.8S rRNA, ITS2, complete sequence	874	874	96%	0.0	92%	AB608333.1	837,58	83,4%
Select seq gb|AY603262.1|	Tanacetum macrophyllum internal transcribed spacer 1, 5.8S ribosomal RNA gene, and internal transcribed spacer 2, complete sequence	874	874	96%	0.0	92%	AY603262.1	837,58	83,4%
Select seq dbj|AB683301.1|	Tanacetum nitens gene for ITS1, 5.8S rRNA and ITS2, complete sequence, specimen_voucher: (W, 1977-16564)	872	872	96%	0.0	92%	AB683301.1	835,67	83,2%
Select seq dbj|AB683274.1|	Tanacetum budjnurdense gene for ITS1, 5.8S rRNA and ITS2, complete sequence, specimen_voucher: 38119 (FUMH)	872	872	96%	0.0	92%	AB683274.1	835,67	83,2%
Select seq dbj|AB683281.1|	Tanacetum dumosum gene for ITS1, 5.8S rRNA and ITS2, complete sequence, specimen_voucher: 1168 (MPH)	870	870	96%	0.0	92%	AB683281.1	833,75	83,0%
Select seq dbj|AB608330.1|	Tanacetum budjnurdense genes for ITS1, 5.8S rRNA, ITS2, complete sequence	870	870	96%	0.0	92%	AB608330.1	833,75	83,0%
Select seq gb|AY603238.1|	Achillea oxyloba internal transcribed spacer 1, 5.8S ribosomal RNA gene, and internal transcribed spacer 2, complete sequence	870	870	96%	0.0	92%	AY603238.1	833,75	83,0%
Select seq dbj|AB683336.1|	Tanacetum zahlbruckneri gene for ITS1, 5.8S rRNA and ITS2, complete sequence, specimen_voucher: 602 (MPH)	869	869	96%	0.0	92%	AB683336.1	832,79	82,9%
Select seq dbj|AB683302.1|	Tanacetum paleaceum gene for ITS1, 5.8S rRNA and ITS2, complete sequence, specimen_voucher: 5828 (B)	869	869	96%	0.0	92%	AB683302.1	832,79	82,9%
Select seq dbj|AB523751.1|	Tanacetum porphyrostephanum genes for ITS1, 5.8S rRNA, ITS2, complete sequence, specimen_voucher: W:1986-05841	869	869	96%	0.0	92%	AB523751.1	832,79	82,9%
Select seq gb|KC816562.1|	Matricaria chamomilla var. recutita 18S ribosomal RNA gene, partial sequence; internal transcribed spacer 1, 5.8S ribosomal RNA gene, and internal transcribed spacer 2, complete sequence; and 28S ribosomal RNA gene, partial sequence	905	905	100%	0.0	92%	KC816562.1	832,60	82,9%
Select seq gb|FJ980357.1|	Achillea wilsoniana voucher PS0715MT01 18S ribosomal RNA gene, partial sequence; internal transcribed spacer 1, 5.8S ribosomal RNA gene, and internal transcribed spacer 2, complete sequence; and 28S ribosomal RNA gene, partial sequence	905	905	100%	0.0	92%	FJ980357.1	832,60	82,9%
Select seq dbj|AB683266.1|	Tanacetum argenteum subsp. flabellifolium gene for ITS1, 5.8S rRNA and ITS2, complete sequence, specimen_voucher: 1976 (HUB)	867	867	96%	0.0	92%	AB683266.1	830,88	82,7%
Select seq gb|KR150147.1|	Achillea sp. Kh58 internal transcribed spacer 1, partial sequence; 5.8S ribosomal RNA gene gene, complete sequence; and internal transcribed spacer 2 region, partial sequence	891	891	99%	0.0	92%	KR150147.1	828,00	82,5%

Select seq dbj|AB683339.1|	Xylanthemum paghmanense gene for ITS1, 5.8S rRNA and ITS2, complete sequence, specimen_voucher: (W-1986-05509)	863	863	96%	0.0	92%	AB683339.1	827,04	82,4%
Select seq dbj|AB523750.1|	Tanacetum kotschyi genes for ITS1, 5.8S rRNA, ITS2, complete sequence, specimen_voucher: HUB:5534	863	863	96%	0.0	92%	AB523750.1	827,04	82,4%
Select seq gb|KM887400.1|	Anacyclus pyrethrum isolate SBB-1400 internal transcribed spacer 1, partial sequence; 5.8S ribosomal RNA gene, complete sequence; and internal transcribed spacer 2, partial sequence	854	854	95%	0.0	92%	KM887400.1	827,03	82,4%
Select seq dbj|AB683327.1|	Tanacetum turcomanicum gene for ITS1, 5.8S rRNA and ITS2, complete sequence, specimen_voucher: 20867 (FUMH)	861	861	96%	0.0	92%	AB683327.1	825,13	82,2%
Select seq dbj|AB683286.1|	Tanacetum griffithii gene for ITS1, 5.8S rRNA and ITS2, complete sequence, specimen_voucher: 37485 (B)	861	861	96%	0.0	92%	AB683286.1	825,13	82,2%
Select seq dbj|AB523749.1|	Tanacetum kotschyi genes for ITS1, 5.8S rRNA, ITS2, complete sequence, specimen_voucher: MPH:1129	861	861	96%	0.0	92%	AB523749.1	825,13	82,2%
Select seq emb|HE602382.1|	Anacyclus pyrethrum genomic DNA containing ITS1, 5.8S rRNA gene, ITS2, specimen voucher Jury, SL. 17869	859	859	96%	0.0	92%	HE602382.1	823,21	82,0%
Select seq gb|AY603235.1|	Achillea moschata internal transcribed spacer 1, 5.8S ribosomal RNA gene, and internal transcribed spacer 2, complete sequence	859	859	96%	0.0	92%	AY603235.1	823,21	82,0%
Select seq dbj|AB523748.1|	Tanacetum kotschyi genes for ITS1, 5.8S rRNA, ITS2, complete sequence, specimen_voucher: MPH:1423	854	854	96%	0.0	92%	AB523748.1	818,42	81,5%
Select seq emb|HE602385.1|	Anacyclus homogamos genomic DNA containing ITS1, 5.8S rRNA gene, ITS2, specimen voucher Jury, SL. 20904	861	861	97%	0.0	92%	HE602385.1	816,62	81,3%
Select seq gb|AY603234.1|	Achillea x morisiana internal transcribed spacer 1, 5.8S ribosomal RNA gene, and internal transcribed spacer 2, complete sequence	852	852	96%	0.0	92%	AY603234.1	816,50	81,3%
Select seq gb|AY603231.1|	Achillea erba-rotta internal transcribed spacer 1, 5.8S ribosomal RNA gene, and internal transcribed spacer 2, complete sequence	852	852	96%	0.0	92%	AY603231.1	816,50	81,3%
Select seq gb|KR150159.1|	Anthemis sp. Kh95 internal transcribed spacer 1, partial sequence; 5.8S ribosomal RNA gene gene, complete sequence; and internal transcribed spacer 2 region, partial sequence	885	885	100%	0.0	92%	KR150159.1	814,20	81,1%
Select seq gb|EU179212.1|	Matricaria recutita 18S ribosomal RNA gene, partial sequence; internal transcribed spacer 1, 5.8S ribosomal RNA gene, and internal transcribed spacer 2, complete sequence; and 26S ribosomal RNA gene, partial sequence	872	872	99%	0.0	92%	EU179212.1	810,34	80,7%
Select seq gb|KM887358.1|	Anacyclus pyrethrum isolate SBB-1390 internal transcribed spacer 1, partial sequence; 5.8S ribosomal RNA gene, complete sequence; and internal transcribed spacer 2, partial sequence	854	854	97%	0.0	92%	KM887358.1	809,98	80,7%
Select seq gb|EU179213.1|	Matricaria matricarioides 18S ribosomal RNA gene, partial sequence; internal transcribed spacer 1, 5.8S ribosomal RNA gene, and internal transcribed spacer 2, complete sequence; and 26S ribosomal RNA gene, partial sequence	859	859	99%	0.0	91%	EU179213.1	789,59	78,6%
Select seq emb|HE602383.1|	Anacyclus radiatus subsp. radiatus genomic DNA containing ITS1, 5.8S rRNA gene, ITS2, specimen voucher Jury, SL. 18550	839	839	97%	0.0	91%	HE602383.1	787,10	78,4%
Select seq gb|KC816561.1|	Achillea santolina 18S ribosomal RNA gene, partial sequence; internal transcribed spacer 1, 5.8S ribosomal RNA gene, and internal transcribed spacer 2, complete sequence; and 28S ribosomal RNA gene, partial sequence	859	859	100%	0.0	91%	KC816561.1	781,69	77,8%
								0,00	

Select for downloading or viewing reports	Kh076_trnL Description	Max score	Total score	Query cover	E value	Ident	Accession	(Ident/Cover)* Max score	Deviation from top hit
Select seq gb|JX073760.1|	Artemisia lagocephala tRNA-Leu (trnL) gene and trnL-trnF intergenic spacer, partial sequence; chloroplast	1072	1072	85%	0.0	97%	JX073760.1	1223,34	100,0%
Select seq gb|JX073787.1|	Artemisia ludoviciana tRNA-Leu (trnL) gene and trnL-trnF intergenic spacer, partial sequence; chloroplast	1061	1061	85%	0.0	97%	JX073787.1	1210,79	99,0%
Select seq gb|JX073755.1|	Artemisia cana tRNA-Leu (trnL) gene and trnL-trnF intergenic spacer, partial sequence; chloroplast	1055	1055	85%	0.0	97%	JX073755.1	1203,94	98,4%
Select seq gb|JX073815.1|	Artemisia afra tRNA-Leu (trnL) gene and trnL-trnF intergenic spacer, partial sequence; chloroplast	1053	1053	85%	0.0	97%	JX073815.1	1201,66	98,2%
Select seq gb|JX073796.1|	Artemisia scoparia tRNA-Leu (trnL) gene and trnL-trnF intergenic spacer, partial sequence; chloroplast	1053	1053	85%	0.0	97%	JX073796.1	1201,66	98,2%
Select seq gb|JX073792.1|	Artemisia japonica tRNA-Leu (trnL) gene and trnL-trnF intergenic spacer, partial sequence; chloroplast	1053	1053	85%	0.0	97%	JX073792.1	1201,66	98,2%
Select seq gb|JX073790.1|	Artemisia pycnocephala tRNA-Leu (trnL) gene and trnL-trnF intergenic spacer, partial sequence; chloroplast	1053	1053	85%	0.0	97%	JX073790.1	1201,66	98,2%
Select seq gb|JX073783.1|	Artemisia schmidtiana tRNA-Leu (trnL) gene and trnL-trnF intergenic spacer, partial sequence; chloroplast	1053	1053	85%	0.0	97%	JX073783.1	1201,66	98,2%
Select seq gb|JX073777.1|	Artemisia armeniaca tRNA-Leu (trnL) gene and trnL-trnF intergenic spacer, partial sequence; chloroplast	1053	1053	85%	0.0	97%	JX073777.1	1201,66	98,2%
Select seq gb|JX073747.1|	Artemisia nitrosa tRNA-Leu (trnL) gene and trnL-trnF intergenic spacer, partial sequence; chloroplast	1053	1053	85%	0.0	97%	JX073747.1	1201,66	98,2%
Select seq gb|JX073749.1|	Artemisia gmelinii subsp. scheludjakoviae tRNA-Leu (trnL) gene and trnL-trnF intergenic spacer, partial sequence; chloroplast	1053	1053	85%	0.0	97%	JX073749.1	1201,66	98,2%
Select seq gb|FJ842617.1|	Artemisia fukudo voucher MPS001124 (KMH) tRNA-Leu (trnL) gene and trnL-trnF intergenic spacer, partial sequence; chloroplast	1053	1053	85%	0.0	97%	FJ842617.1	1201,66	98,2%
Select seq gb|FJ842615.1|	Artemisia capillaris voucher MPS001196 (KMH) tRNA-Leu (trnL) gene and trnL-trnF intergenic spacer, partial sequence; chloroplast	1053	1053	85%	0.0	97%	FJ842615.1	1201,66	98,2%
Select seq gb|JX073758.1|	Artemisia pedatifida tRNA-Leu (trnL) gene and trnL-trnF intergenic spacer, partial sequence; chloroplast	1040	1040	84%	0.0	97%	JX073758.1	1200,95	98,2%
Select seq gb|KF736807.1|	Artemisia turcomanica tRNA-Leu (trnL) gene and trnL-trnF intergenic spacer, partial sequence; chloroplast	1074	1074	87%	0.0	97%	KF736807.1	1197,45	97,9%
Select seq gb|KF736803.1|	Artemisia fragrans tRNA-Leu (trnL) gene and trnL-trnF intergenic spacer, partial sequence; chloroplast	1074	1074	87%	0.0	97%	KF736803.1	1197,45	97,9%
Select seq gb|KF736802.1|	Artemisia incana tRNA-Leu (trnL) gene and trnL-trnF intergenic spacer, partial sequence; chloroplast	1074	1074	87%	0.0	97%	KF736802.1	1197,45	97,9%
Select seq gb|JX073826.1|	Artemisia atrata tRNA-Leu (trnL) gene and trnL-trnF intergenic spacer, partial sequence; chloroplast	1048	1048	85%	0.0	97%	JX073826.1	1195,95	97,8%
Select seq gb|JX073786.1|	Artemisia suksdorfii tRNA-Leu (trnL) gene and trnL-trnF intergenic spacer, partial sequence; chloroplast	1048	1048	85%	0.0	97%	JX073786.1	1195,95	97,8%
Select seq gb|JX073785.1|	Artemisia serrata tRNA-Leu (trnL) gene and trnL-trnF intergenic spacer, partial sequence; chloroplast	1048	1048	85%	0.0	97%	JX073785.1	1195,95	97,8%
Select seq gb|JX073779.1|	Artemisia alba tRNA-Leu (trnL) gene and trnL-trnF intergenic spacer, partial sequence; chloroplast	1048	1048	85%	0.0	97%	JX073779.1	1195,95	97,8%
Select seq gb|JX073775.1|	Artemisia laciniata tRNA-Leu (trnL) gene and trnL-trnF intergenic spacer, partial sequence; chloroplast	1048	1048	85%	0.0	97%	JX073775.1	1195,95	97,8%
Select seq gb|FJ842621.1|	Artemisia keiskeana voucher MPS001234 (KMH) tRNA-Leu (trnL) gene and trnL-trnF intergenic spacer, partial sequence; chloroplast	1048	1048	85%	0.0	97%	FJ842621.1	1195,95	97,8%
Select seq gb|FJ842619.1|	Artemisia japonica voucher MPS000177 (KMH) tRNA-Leu (trnL) gene and trnL-trnF intergenic spacer, partial sequence; chloroplast	1048	1048	85%	0.0	97%	FJ842619.1	1195,95	97,8%
Select seq gb|FJ842618.1|	Artemisia gmelinii voucher MPS001216 (KMH) tRNA-Leu (trnL) gene and trnL-trnF intergenic spacer, partial sequence; chloroplast	1048	1048	85%	0.0	97%	FJ842618.1	1195,95	97,8%
Select seq gb|FJ710526.1|	Artemisia absinthium tRNA-Leu (trnL) gene and trnL-trnF intergenic spacer, partial sequence; chloroplast	1048	1048	85%	0.0	97%	FJ710526.1	1195,95	97,8%
Select seq gb|FJ692341.1|	Artemisia annua voucher MPS 001275 (KMH) tRNA-Leu (trnL) gene and trnL-trnF intergenic spacer, partial sequence; chloroplast	1048	1048	85%	0.0	97%	FJ692341.1	1195,95	97,8%
Select seq gb|JX073805.1|	Artemisia gracilescens tRNA-Leu (trnL) gene and trnL-trnF intergenic spacer, partial sequence; chloroplast	1083	1083	88%	0.0	97%	JX073805.1	1193,76	97,6%
Select seq gb|JX073814.1|	Mausolea eriocarpa tRNA-Leu (trnL) gene and trnL-trnF intergenic spacer, partial sequence; chloroplast	1090	1090	89%	0.0	97%	JX073814.1	1187,98	97,1%
Select seq gb|KF736826.1|	Artemisia aucheri tRNA-Leu (trnL) gene and trnL-trnF intergenic spacer, partial sequence; chloroplast	1068	1068	87%	0.0	96%	KF736826.1	1178,48	96,3%

Select seq gb|KF736805.1|	Artemisia spicigera tRNA-Leu (trnL) gene and trnL-trnF intergenic spacer, partial sequence; chloroplast	1068	1068	87% 0.0	96% KF736805.1	1178,48	96,3%
Select seq gb|KF669899.1|	Artemisia absinthium tRNA-Leu (trnL) gene and trnL-trnF intergenic spacer, partial sequence; chloroplast	1068	1068	87% 0.0	96% KF669899.1	1178,48	96,3%
Select seq dbj|AB219593.1|	Artemisia sinanensis chloroplast trnL(UAA), trnF(GAA) genes for tRNA-Leu, tRNA-Phe, partial sequence, trnL-trnF spacer region, specimen voucher: MAK:N. Fujii F01782	1068	1068	87% 0.0	96% AB219593.1	1178,48	96,3%
Select seq gb|JX073821.1|	Artemisia mauiensis tRNA-Leu (trnL) gene and trnL-trnF intergenic spacer, partial sequence; chloroplast	1042	1042	85% 0.0	96% JX073821.1	1176,85	96,2%
Select seq gb|JX073819.1|	Artemisia australis tRNA-Leu (trnL) gene and trnL-trnF intergenic spacer, partial sequence; chloroplast	1042	1042	85% 0.0	96% JX073819.1	1176,85	96,2%
Select seq gb|JX073753.1|	Artemisia arbuscula tRNA-Leu (trnL) gene and trnL-trnF intergenic spacer, partial sequence; chloroplast	1042	1042	85% 0.0	96% JX073753.1	1176,85	96,2%
Select seq gb|JX073799.1|	Artemisia gorgonum tRNA-Leu (trnL) gene and trnL-trnF intergenic spacer, partial sequence; chloroplast	1042	1042	85% 0.0	96% JX073799.1	1176,85	96,2%
Select seq gb|JX073798.1|	Artemisia rigida tRNA-Leu (trnL) gene and trnL-trnF intergenic spacer, partial sequence; chloroplast	1042	1042	85% 0.0	96% JX073798.1	1176,85	96,2%
Select seq gb|JX073778.1|	Artemisia abrotanum tRNA-Leu (trnL) gene and trnL-trnF intergenic spacer, partial sequence; chloroplast	1042	1042	85% 0.0	96% JX073778.1	1176,85	96,2%
Select seq gb|FJ842620.1|	Artemisia manshurica voucher MPS00123 (KMH) tRNA-Leu (trnL) gene and trnL-trnF intergenic spacer, partial sequence; chloroplast	1042	1042	85% 0.0	96% FJ842620.1	1176,85	96,2%
Select seq gb|FJ710524.1|	Artemisia caruifolia var. apiacea tRNA-Leu (trnL) gene and trnL-trnF intergenic spacer, partial sequence; chloroplast	1042	1042	85% 0.0	96% FJ710524.1	1176,85	96,2%
Select seq gb|JX073797.1|	Artemisia dracunculus tRNA-Leu (trnL) gene and trnL-trnF intergenic spacer, partial sequence; chloroplast	1040	1040	85% 0.0	96% JX073797.1	1174,59	96,0%
Select seq gb|JX073774.1|	Artemisia molinieri tRNA-Leu (trnL) gene and trnL-trnF intergenic spacer, partial sequence; chloroplast	1040	1040	85% 0.0	96% JX073774.1	1174,59	96,0%
Select seq gb|JX073761.1|	Artemisia anomala tRNA-Leu (trnL) gene and trnL-trnF intergenic spacer, partial sequence; chloroplast	1040	1040	85% 0.0	96% JX073761.1	1174,59	96,0%
Select seq gb|KF736827.1|	Artemisia ciniformis tRNA-Leu (trnL) gene and trnL-trnF intergenic spacer, partial sequence; chloroplast	1062	1062	87% 0.0	96% KF736827.1	1171,86	95,8%
Select seq gb|KF736825.1|	Artemisia melanolepis tRNA-Leu (trnL) gene and trnL-trnF intergenic spacer, partial sequence; chloroplast	1062	1062	87% 0.0	96% KF736825.1	1171,86	95,8%
Select seq gb|KF736809.1|	Artemisia marschalliana tRNA-Leu (trnL) gene and trnL-trnF intergenic spacer, partial sequence; chloroplast	1062	1062	87% 0.0	96% KF736809.1	1171,86	95,8%
Select seq gb|KF736808.1|	Artemisia chamaemelifolia tRNA-Leu (trnL) gene and trnL-trnF intergenic spacer, partial sequence; chloroplast	1062	1062	87% 0.0	96% KF736808.1	1171,86	95,8%
Select seq gb|KF736806.1|	Artemisia haussknechtii tRNA-Leu (trnL) gene and trnL-trnF intergenic spacer, partial sequence; chloroplast	1062	1062	87% 0.0	96% KF736806.1	1171,86	95,8%
Select seq gb|JX073824.1|	Artemisia copa tRNA-Leu (trnL) gene and trnL-trnF intergenic spacer, partial sequence; chloroplast	1037	1037	85% 0.0	96% JX073824.1	1171,20	95,7%
Select seq gb|JX073822.1|	Artemisia chinensis tRNA-Leu (trnL) gene and trnL-trnF intergenic spacer, partial sequence; chloroplast	1037	1037	85% 0.0	96% JX073822.1	1171,20	95,7%
Select seq gb|JX073793.1|	Artemisia campestris tRNA-Leu (trnL) gene and trnL-trnF intergenic spacer, partial sequence; chloroplast	1037	1037	85% 0.0	96% JX073793.1	1171,20	95,7%
Select seq gb|JX073782.1|	Artemisia nitida tRNA-Leu (trnL) gene and trnL-trnF intergenic spacer, partial sequence; chloroplast	1037	1037	85% 0.0	96% JX073782.1	1171,20	95,7%
Select seq gb|JN315916.1|	Chrysanthemum indicum tRNA-Leu (trnL) gene and trnL-trnF intergenic spacer, partial sequence; chloroplast	1061	1061	87% 0.0	96% JN315916.1	1170,76	95,7%
Select seq gb|KF736821.1|	Artemisia capillaris tRNA-Leu (trnL) gene and trnL-trnF intergenic spacer, partial sequence; chloroplast	1057	1057	87% 0.0	96% KF736821.1	1166,34	95,3%
Select seq gb|JX073825.1|	Artemisia potentilloides tRNA-Leu (trnL) gene and trnL-trnF intergenic spacer, partial sequence; chloroplast	1081	1081	89% 0.0	96% JX073825.1	1166,02	95,3%
Select seq gb|KF736817.1|	Artemisia tschernieviana tRNA-Leu (trnL) gene and trnL-trnF intergenic spacer, partial sequence; chloroplast	1050	1050	87% 0.0	96% KF736817.1	1158,62	94,7%
Select seq gb|JX073752.1|	Artemisia rothrockii tRNA-Leu (trnL) gene and trnL-trnF intergenic spacer, partial sequence; chloroplast	1103	1165	93% 0.0	97% JX073752.1	1150,44	94,0%
Select seq gb|JX073828.1|	Kaschgaria komarovii tRNA-Leu (trnL) gene and trnL-trnF intergenic spacer, partial sequence; chloroplast	1101	1163	93% 0.0	97% JX073828.1	1148,35	93,9%
Select seq gb|JX073812.1|	Artemisia subdigitata tRNA-Leu (trnL) gene and trnL-trnF intergenic spacer, partial sequence; chloroplast	1101	1163	93% 0.0	97% JX073812.1	1148,35	93,9%
Select seq gb|JX073748.1|	Artemisia santonicum tRNA-Leu (trnL) gene and trnL-trnF intergenic spacer, partial sequence; chloroplast	1101	1163	93% 0.0	97% JX073748.1	1148,35	93,9%
Select seq gb|JX073816.1|	Artemisia elongata tRNA-Leu (trnL) gene and trnL-trnF intergenic spacer, partial sequence; chloroplast	1098	1160	93% 0.0	97% JX073816.1	1145,23	93,6%

Select seq gb|JX073811.1|	Artemisia glomerata tRNA-Leu (trnL) gene and trnL-trnF intergenic spacer, partial sequence; chloroplast	1096	1158	93% 0.0	97% JX073811.1	1143,14	93,4%
Select seq gb|JX073808.1|	Artemisia tilesii tRNA-Leu (trnL) gene and trnL-trnF intergenic spacer, partial sequence; chloroplast	1096	1158	93% 0.0	97% JX073808.1	1143,14	93,4%
Select seq gb|JX073803.1|	Artemisia tournefortiana tRNA-Leu (trnL) gene and trnL-trnF intergenic spacer, partial sequence; chloroplast	1096	1158	93% 0.0	97% JX073803.1	1143,14	93,4%
Select seq gb|JN867592.1|	Chrysanthemum indicum voucher NJ026 chloroplast, complete genome	1160	1233	99% 0.0	97% JN867592.1	1136,57	92,9%
Select seq gb|GU817959.1|	Anacyclus valentinus isolate L1186 tRNA-Leu (trnL) gene, partial sequence; trnL-trnF intergenic spacer, complete sequence; and tRNA-Phe (trnF) gene, partial sequence; plastid	1077	1077	91% 0.0	96% GU817959.1	1136,18	92,9%
Select seq dbj|AB551235.1|	Artemisia capillaris chloroplast genes for tRNA-Leu intron and 3' exon, trnL-trnF intergenic spacer and tRNA-Phe	1122	1195	96% 0.0	97% AB551235.1	1133,69	92,7%
Select seq gb|HM000099.1|	Chrysanthemum indicum isolate HEBSJZ tRNA-Leu (trnL) gene, partial sequence; trnL-trnF intergenic spacer, complete sequence; and tRNA-Phe (trnF) gene, partial sequence; chloroplast	1140	1213	98% 0.0	97% HM000099.1	1128,37	92,2%
Select seq gb|HM000094.1|	Chrysanthemum indicum isolate HUBJZ tRNA-Leu (trnL) gene, partial sequence; trnL-trnF intergenic spacer, complete sequence; and tRNA-Phe (trnF) gene, partial sequence; chloroplast	1140	1213	98% 0.0	97% HM000094.1	1128,37	92,2%
Select seq gb|JX073804.1|	Artemisia chamaemelifolia tRNA-Leu (trnL) gene and trnL-trnF intergenic spacer, partial sequence; chloroplast	1092	1154	93% 0.0	96% JX073804.1	1127,23	92,1%
Select seq gb|JX073827.1|	Artemisia absinthium tRNA-Leu (trnL) gene and trnL-trnF intergenic spacer, partial sequence; chloroplast	1090	1152	93% 0.0	96% JX073827.1	1125,16	92,0%
Select seq gb|JX073791.1|	Artemisia tomentella tRNA-Leu (trnL) gene and trnL-trnF intergenic spacer, partial sequence; chloroplast	1090	1152	93% 0.0	96% JX073791.1	1125,16	92,0%
Select seq gb|JX073823.1|	Artemisia globularia tRNA-Leu (trnL) gene and trnL-trnF intergenic spacer, partial sequence; chloroplast	1086	1149	93% 0.0	96% JX073823.1	1121,03	91,6%
Select seq gb|JX293720.1|	Artemisia frigida chloroplast, complete genome	1155	1228	99% 0.0	96% JX293720.1	1120,00	91,6%
Select seq gb|JX073820.1|	Artemisia kauaiensis tRNA-Leu (trnL) gene and trnL-trnF intergenic spacer, partial sequence; chloroplast	1085	1147	93% 0.0	96% JX073820.1	1120,00	91,6%
Select seq gb|JX073809.1|	Artemisia pattersonii tRNA-Leu (trnL) gene and trnL-trnF intergenic spacer, partial sequence; chloroplast	1085	1147	93% 0.0	96% JX073809.1	1120,00	91,6%
Select seq gb|JX073807.1|	Artemisia martirensis tRNA-Leu (trnL) gene and trnL-trnF intergenic spacer, partial sequence; chloroplast	1085	1147	93% 0.0	96% JX073807.1	1120,00	91,6%
Select seq gb|JQ362483.1|	Chrysanthemum x morifolium chloroplast, complete genome	1149	1222	99% 0.0	96% JQ362483.1	1114,18	91,1%
Select seq gb|JN867589.1|	Chrysanthemum indicum voucher HeN001 chloroplast, complete genome	1147	1221	99% 0.0	96% JN867589.1	1112,24	90,9%
Select seq dbj|AB685322.1|	Chrysanthemum indicum chloroplast DNA, contains tRNA-Leu(trnL) gene (intron and 3' exon), trnL-trnF intergenic spacer and tRNA-Phe(trnF) gene	1109	1182	96% 0.0	96% AB685322.1	1109,00	90,7%
Select seq gb|HM000091.1|	Chrysanthemum indicum isolate HENXY tRNA-Leu (trnL) gene, partial sequence; trnL-trnF intergenic spacer, complete sequence; and tRNA-Phe (trnF) gene, partial sequence; chloroplast	1127	1200	98% 0.0	96% HM000091.1	1104,00	90,2%
Select seq gb|HM000097.1|	Chrysanthemum indicum isolate JXPX tRNA-Leu (trnL) gene, partial sequence; trnL-trnF intergenic spacer, complete sequence; and tRNA-Phe (trnF) gene, partial sequence; chloroplast	1122	1195	98% 0.0	96% HM000097.1	1099,10	89,8%
Select seq gb|HM000096.1|	Chrysanthemum indicum isolate YNKM tRNA-Leu (trnL) gene, partial sequence; trnL-trnF intergenic spacer, complete sequence; and tRNA-Phe (trnF) gene, partial sequence; chloroplast	1122	1195	98% 0.0	96% HM000096.1	1099,10	89,8%
Select seq gb|HM000095.1|	Chrysanthemum indicum isolate HUNZZ tRNA-Leu (trnL) gene, partial sequence; trnL-trnF intergenic spacer, complete sequence; and tRNA-Phe (trnF) gene, partial sequence; chloroplast	1122	1195	98% 0.0	96% HM000095.1	1099,10	89,8%
Select seq gb|HM000088.1|	Chrysanthemum indicum isolate GZGY tRNA-Leu (trnL) gene, partial sequence; trnL-trnF intergenic spacer, complete sequence; and tRNA-Phe (trnF) gene, partial sequence; chloroplast	1122	1195	98% 0.0	96% HM000088.1	1099,10	89,8%
Select seq gb|HM000087.1|	Chrysanthemum indicum isolate AHCZ tRNA-Leu (trnL) gene, partial sequence; trnL-trnF intergenic spacer, complete sequence; and tRNA-Phe (trnF) gene, partial sequence; chloroplast	1122	1195	98% 0.0	96% HM000087.1	1099,10	89,8%
Select seq gb|HM000086.1|	Chrysanthemum indicum isolate JSWX tRNA-Leu (trnL) gene, partial sequence; trnL-trnF intergenic spacer, complete sequence; and tRNA-Phe (trnF) gene, partial sequence; chloroplast	1122	1195	98% 0.0	96% HM000086.1	1099,10	89,8%
Select seq gb|HM000085.1|	Chrysanthemum indicum isolate JSNJ tRNA-Leu (trnL) gene, partial sequence; trnL-trnF intergenic spacer, complete sequence; and tRNA-Phe (trnF) gene, partial sequence; chloroplast	1122	1195	98% 0.0	96% HM000085.1	1099,10	89,8%
Select seq gb|HM000093.1|	Chrysanthemum indicum isolate SCMY tRNA-Leu (trnL) gene, partial sequence; trnL-trnF intergenic spacer, complete sequence; and tRNA-Phe (trnF) gene, partial sequence; chloroplast	1116	1189	98% 0.0	96% HM000093.1	1093,22	89,4%
Select seq gb|HM000092.1|	Chrysanthemum indicum isolate GXGL tRNA-Leu (trnL) gene, partial sequence; trnL-trnF intergenic spacer, complete sequence; and tRNA-Phe (trnF) gene, partial sequence; chloroplast	1116	1189	98% 0.0	96% HM000092.1	1093,22	89,4%
Select seq gb|HM000089.1|	Chrysanthemum indicum isolate AHHF tRNA-Leu (trnL) gene, partial sequence; trnL-trnF intergenic spacer, complete sequence; and tRNA-Phe (trnF) gene, partial sequence; chloroplast	1116	1189	98% 0.0	96% HM000089.1	1093,22	89,4%
Select seq gb|HM000098.1|	Chrysanthemum indicum isolate FJFZ tRNA-Leu (trnL) gene, partial sequence; trnL-trnF intergenic spacer, complete sequence; and tRNA-Phe (trnF) gene, partial sequence; chloroplast	1110	1184	98% 0.0	96% HM000098.1	1087,35	88,9%
Select seq gb|HM000090.1|	Chrysanthemum indicum isolate ZJWZ tRNA-Leu (trnL) gene, partial sequence; trnL-trnF intergenic spacer, complete sequence; and tRNA-Phe (trnF) gene, partial sequence; chloroplast	1110	1184	98% 0.0	96% HM000090.1	1087,35	88,9%

Select seq gb|KF887960.1|	Artemisia montana chloroplast, complete genome	1110	1184	99% 0.0	95%	KF887960.1	1065,15	87,1%
Select seq gb|KR150244.1|	Achillea sp. 1 Kh17 trnL-trnF intergenic spacer region, partial sequence; chloroplast	1050	1123	96% 0.0	95%	KR150244.1	1039,06	84,9%
Select seq gb|KR150196.1|	Achillea sp. 3 Kh58 trnL-trnF intergenic spacer region, partial sequence; chloroplast	1048	1121	96% 0.0	95%	KR150196.1	1037,08	84,8%
							0,00	0,0%
							0,00	0,0%

Select for downloading or viewing reports	Kh082_trnL Description	Max score	Total score	Query cover	E value	Ident	Accession	(Ident/Cover)* Max score	Deviation from top hit
Select seq gb|JQ669045.1|	Hymenocrater bituminosus voucher K. Tamanyan & George Fayvush tRNA-Leu (trnL) gene and trnL-trnF intergenic spacer, partial sequence; plastid	1177	1177	98%	0.0	99%	JQ669045.1	1189,01	100,0%
Select seq gb|JQ669049.1|	Marmoritis complanatum voucher D.E. Boufford et al., 32012 tRNA-Leu (trnL) gene and trnL-trnF intergenic spacer, partial sequence; plastid	1129	1129	96%	0.0	99%	JQ669049.1	1164,28	97,9%
Select seq gb|KM886627.1|	Marmoritis complanata voucher T. Deng 2359 (KUN) tRNA-Leu (trnL) gene, partial sequence; trnL-trnF intergenic spacer, complete sequence; and tRNA-Phe (trnF) gene, partial sequence; plastid	1118	1118	96%	0.0	99%	KM886627.1	1152,94	97,0%
Select seq gb|KR150192.1|	Hymenocrater sp. 2 Kh51 trnL-trnF intergenic spacer region, partial sequence; chloroplast	994	994	86%	0.0	98%	KR150192.1	1132,70	95,3%
Select seq emb|AJ505431.1|	Nepeta menthoides plastid trnL-trnF intergenic spacer, specimen voucher Jamzad s.n. (K)	1083	1083	100%	0.0	97%	AJ505431.1	1050,51	88,4%
Select seq gb|KF307433.1|	Lepechinia urbanii voucher B. Drew 135 tRNA-Leu (trnL) gene and trnL-trnF intergenic spacer, partial sequence; chloroplast	1009	1009	95%	0.0	96%	KF307433.1	1019,62	85,8%
Select seq gb|FJ593457.1|	Nepeta stewartiana tRNA-Leu (trnL) gene and trnL-trnF intergenic spacer, partial sequence; chloroplast	1055	1055	100%	0.0	96%	FJ593457.1	1012,80	85,2%
Select seq gb|DQ667492.1|	Lepechinia lancifolia isolate x232 tRNA-Leu (trnL) gene and trnL-trnF intergenic spacer, partial sequence; chloroplast	1048	1048	100%	0.0	96%	DQ667492.1	1006,08	84,6%
Select seq gb|JF301382.1|	Lepechinia radula voucher B. Drew 185 tRNA-Leu (trnL) gene and trnL-trnF intergenic spacer, partial sequence; chloroplast	1009	1009	96%	0.0	95%	JF301382.1	998,49	84,0%
Select seq gb|KF307430.1|	Lepechinia schiedeana voucher B. Drew 157 tRNA-Leu (trnL) gene and trnL-trnF intergenic spacer, partial sequence; chloroplast	998	998	95%	0.0	95%	KF307430.1	998,00	83,9%
Select seq gb|KF307411.1|	Lepechinia bella voucher Rachel Jabaily s.n. tRNA-Leu (trnL) gene and trnL-trnF intergenic spacer, partial sequence; chloroplast	998	998	95%	0.0	95%	KF307411.1	998,00	83,9%
Select seq gb|JF301383.1|	Lepechinia salviifolia voucher R. Jabaily s.n. tRNA-Leu (trnL) gene and trnL-trnF intergenic spacer, partial sequence; chloroplast	1007	1007	96%	0.0	95%	JF301383.1	996,51	83,8%
Select seq emb|AJ505432.1|	Nepeta racemosa plastid trnL-trnF intergenic spacer, specimen voucher Jamzad s.n. (TARI)	1037	1037	99%	0.0	95%	AJ505432.1	995,10	83,7%
Select seq gb|GU381481.1|	Micromeria cf. madagascariensis Morawetz 205 tRNA-Leu (trnL) gene and trnL-trnF intergenic spacer, partial sequence; chloroplast	974	974	93%	0.0	95%	GU381481.1	994,95	83,7%
Select seq gb|JF301391.1|	Nepeta cataria voucher B. Drew 72 tRNA-Leu (trnL) gene and trnL-trnF intergenic spacer, partial sequence; chloroplast	1003	1003	96%	0.0	95%	JF301391.1	992,55	83,5%
Select seq gb|JF301361.1|	Chaunostoma mecistandrum voucher J.A. Monterrosa & R.A. Carballo 213 tRNA-Leu (trnL) gene and trnL-trnF intergenic spacer, partial sequence; chloroplast	1003	1003	96%	0.0	95%	JF301361.1	992,55	83,5%
Select seq gb|KF307435.1|	Lepechinia yecorana voucher Henrickson 24691 tRNA-Leu (trnL) gene and trnL-trnF intergenic spacer, partial sequence; chloroplast	992	992	95%	0.0	95%	KF307435.1	992,00	83,4%
Select seq gb|KF307432.1|	Lepechinia speciosa voucher Cordeno 3060 tRNA-Leu (trnL) gene and trnL-trnF intergenic spacer, partial sequence; chloroplast	992	992	95%	0.0	95%	KF307432.1	992,00	83,4%
Select seq gb|KF307421.1|	Lepechinia heteromorpha voucher B. Drew 192 tRNA-Leu (trnL) gene and trnL-trnF intergenic spacer, partial sequence; chloroplast	992	992	95%	0.0	95%	KF307421.1	992,00	83,4%
Select seq gb|KF307420.1|	Lepechinia graveolens voucher Fuentes 10351 tRNA-Leu (trnL) gene and trnL-trnF intergenic spacer, partial sequence; chloroplast	992	992	95%	0.0	95%	KF307420.1	992,00	83,4%
Select seq gb|KF307418.1|	Lepechinia floribunda voucher B. Drew 172 tRNA-Leu (trnL) gene and trnL-trnF intergenic spacer, partial sequence; chloroplast	992	992	95%	0.0	95%	KF307418.1	992,00	83,4%
Select seq gb|DQ667487.1|	Nepeta cataria isolate x220 tRNA-Leu (trnL) gene and trnL-trnF intergenic spacer, partial sequence; chloroplast	1042	1042	100%	0.0	95%	DQ667487.1	989,90	83,3%
Select seq gb|JF301380.1|	Lepechinia mexicana voucher B. Drew 127 tRNA-Leu (trnL) gene and trnL-trnF intergenic spacer, partial sequence; chloroplast	998	998	96%	0.0	95%	JF301380.1	987,60	83,1%
Select seq gb|KF307422.1|	Lepechinia mexicana voucher B. Drew 130 tRNA-Leu (trnL) gene and trnL-trnF intergenic spacer, partial sequence; chloroplast	987	987	95%	0.0	95%	KF307422.1	987,00	83,0%
Select seq gb|GU381479.1|	Nepeta supina voucher M:Groeger & Schewardnadse 1466 tRNA-Leu (trnL) gene and trnL-trnF intergenic spacer, partial sequence; chloroplast	1018	1018	98%	0.0	95%	GU381479.1	986,84	83,0%
Select seq gb|JF301386.1|	Melissa officinalis voucher B. Drew 70 tRNA-Leu (trnL) gene and trnL-trnF intergenic spacer, partial sequence; chloroplast	996	996	96%	0.0	95%	JF301386.1	985,63	82,9%

Select seq gb|KF307416.1|	Lepechinia codon voucher B. Drew 177 tRNA-Leu (trnL) gene and trnL-trnF intergenic spacer, partial sequence; chloroplast	985	985	95%	0.0	95%	KF307416.1	985,00	82,8%
Select seq gb|KR150191.1|	Nepeta sp. Kh55 trnL-trnF intergenic spacer region, partial sequence; chloroplast	1005	1005	97%	0.0	95%	KR150191.1	984,28	82,8%
Select seq gb|DQ667517.1|	Drepanocaryum sewerzowii isolate x523 tRNA-Leu (trnL) gene and trnL-trnF intergenic spacer, partial sequence; chloroplast	1005	1005	97%	0.0	95%	DQ667517.1	984,28	82,8%
Select seq emb|AJ505529.1|	Melissa officinalis plastid trnL-trnF intergenic spacer, specimen voucher Catino	1024	1024	99%	0.0	95%	AJ505529.1	982,63	82,6%
Select seq emb|AJ505433.1|	Nepeta straussii plastid trnL-trnF intergenic spacer, specimen voucher Jamzad etal 76846 (TARI)	1024	1024	99%	0.0	95%	AJ505433.1	982,63	82,6%
Select seq gb|GU381496.1|	Killickia pilosa voucher M:Braeuchler 3832 tRNA-Leu (trnL) gene and trnL-trnF intergenic spacer, partial sequence; chloroplast	1013	1013	98%	0.0	95%	GU381496.1	981,99	82,6%
Select seq gb|GU381493.1|	Killickia grandiflora voucher M:Braeuchler 3811 tRNA-Leu (trnL) gene and trnL-trnF intergenic spacer, partial sequence; chloroplast	1013	1013	98%	0.0	95%	GU381493.1	981,99	82,6%
Select seq gb|JF301381.1|	Lepechinia mexicana voucher B. Drew 164 tRNA-Leu (trnL) gene and trnL-trnF intergenic spacer, partial sequence; chloroplast	990	990	96%	0.0	95%	JF301381.1	979,69	82,4%
Select seq gb|DQ667477.1|	Melissa officinalis isolate x193 tRNA-Leu (trnL) gene and trnL-trnF intergenic spacer, partial sequence; chloroplast	1029	1029	100%	0.0	95%	DQ667477.1	977,55	82,2%
Select seq gb|GU381495.1|	Killickia pilosa voucher M:Braeuchler 3810 tRNA-Leu (trnL) gene and trnL-trnF intergenic spacer, partial sequence; chloroplast	998	998	97%	0.0	95%	GU381495.1	977,42	82,2%
Select seq gb|JF301377.1|	Lepechinia flammea tRNA-Leu (trnL) gene and trnL-trnF intergenic spacer, partial sequence; chloroplast	977	1076	96%	0.0	96%	JF301377.1	977,00	82,2%
Select seq gb|KF307419.1|	Lepechinia ganderi voucher B. Drew 24 tRNA-Leu (trnL) gene and trnL-trnF intergenic spacer, partial sequence; chloroplast	976	976	95%	0.0	95%	KF307419.1	976,00	82,1%
Select seq gb|AY570460.1|	Lepechinia fragrans voucher JBW 1333 tRNA-Leu and trnL-trnF intergenic spacer, partial sequence; chloroplast	1002	1002	98%	0.0	95%	AY570460.1	971,33	81,7%
Select seq gb|KF307429.1|	Lepechinia salviae voucher R. Jabaily s.n. tRNA-Leu (trnL) gene and trnL-trnF intergenic spacer, partial sequence; chloroplast	970	970	95%	0.0	95%	KF307429.1	970,00	81,6%
Select seq gb|GU381484.1|	Micromeria flagellaris voucher E:van der Werff & McPherson 13570 tRNA-Leu (trnL) gene and trnL-trnF intergenic spacer, partial sequence; chloroplast	1000	1000	98%	0.0	95%	GU381484.1	969,39	81,5%
Select seq gb|GU381483.1|	Micromeria flagellaris voucher E:Clement et al 2140 tRNA-Leu (trnL) gene and trnL-trnF intergenic spacer, partial sequence; chloroplast	1000	1000	98%	0.0	95%	GU381483.1	969,39	81,5%
Select seq gb|AY840207.1|	Thymbra spicata tRNA-Leu (trnL) gene and trnL-trnF intergenic spacer, partial sequence; chloroplast	1000	1000	98%	0.0	95%	AY840207.1	969,39	81,5%
Select seq gb|AY570459.1|	Lepechinia chamaedryoides voucher JBW 2537 tRNA-Leu and trnL-trnF intergenic spacer, partial sequence; chloroplast	1020	1020	100%	0.0	95%	AY570459.1	969,00	81,5%
Select seq gb|JF301376.1|	Lepechinia caulescens voucher B. Drew 106 tRNA-Leu (trnL) gene and trnL-trnF intergenic spacer, partial sequence; chloroplast	976	976	96%	0.0	95%	JF301376.1	965,83	81,2%
Select seq gb|JF301360.1|	Cedronella canariensis voucher UCBG 2004.0788 tRNA-Leu (trnL) gene and trnL-trnF intergenic spacer, partial sequence; chloroplast	974	974	96%	0.0	95%	JF301360.1	963,85	81,1%
Select seq gb|DQ667494.1|	Lepechinia calycina isolate x246 tRNA-Leu (trnL) gene and trnL-trnF intergenic spacer, partial sequence; chloroplast	1014	1014	100%	0.0	95%	DQ667494.1	963,30	81,0%
Select seq gb|JF301375.1|	Lepechinia calycina voucher B. Drew 197 tRNA-Leu (trnL) gene and trnL-trnF intergenic spacer, partial sequence; chloroplast	976	976	96%	0.0	94%	JF301375.1	955,67	80,4%
Select seq gb|GU381632.1|	Thymbra spicata voucher M:Braeuchler 4548 tRNA-Leu (trnL) gene and trnL-trnF intergenic spacer, partial sequence; chloroplast	996	996	98%	0.0	94%	GU381632.1	955,35	80,3%
Select seq gb|GU381629.1|	Thymbra capitata voucher M:Braeuchler 2518 tRNA-Leu (trnL) gene and trnL-trnF intergenic spacer, partial sequence; chloroplast	994	994	98%	0.0	94%	GU381629.1	953,43	80,2%
Select seq gb|GU381489.1|	Killickia lutea voucher NU<ZAF>:Hilliard & Burtt 9876 tRNA-Leu (trnL) gene and trnL-trnF intergenic spacer, partial sequence; chloroplast	990	990	98%	0.0	94%	GU381489.1	949,59	79,9%
Select seq gb|GU381488.1|	Killickia compacta voucher M:Braeuchler 3816 tRNA-Leu (trnL) gene and trnL-trnF intergenic spacer, partial sequence; chloroplast	990	990	98%	0.0	94%	GU381488.1	949,59	79,9%
Select seq gb|GU381627.1|	Thymbra sintenisii subsp. isaurica voucher E:Goener 12628 tRNA-Leu (trnL) gene and trnL-trnF intergenic spacer, partial sequence; chloroplast	989	989	98%	0.0	94%	GU381627.1	948,63	79,8%
Select seq gb|AY570458.1|	Lepechinia calycina voucher JBW 1344 tRNA-Leu and trnL-trnF intergenic spacer, partial sequence; chloroplast	1009	1009	100%	0.0	94%	AY570458.1	948,46	79,8%

Select seq gb|JF301401.1|	Thymbra capitata voucher UCBG 96.0817 tRNA-Leu (trnL) gene and trnL-trnF intergenic spacer, partial sequence; chloroplast	968	968	96% 0.0	94% JF301401.1	947,83	79,7%
Select seq gb|GU381517.1|	Clinopodium barosmum voucher BM<GBR-LONDON>:McLaren N193 tRNA-Leu (trnL) gene and trnL-trnF intergenic spacer, partial sequence; chloroplast	983	983	98% 0.0	94% GU381517.1	942,88	79,3%
Select seq gb|GU381516.1|	Clinopodium wardii voucher BM<GBR-LONDON>:Ludlow et al. 14234 tRNA-Leu (trnL) gene and trnL-trnF intergenic spacer, partial sequence; chloroplast	983	983	98% 0.0	94% GU381516.1	942,88	79,3%
Select seq gb|GU381515.1|	Clinopodium hydaspidis voucher BM<GBR-LONDON>:Mohd 133 tRNA-Leu (trnL) gene and trnL-trnF intergenic spacer, partial sequence; chloroplast	983	983	98% 0.0	94% GU381515.1	942,88	79,3%
Select seq gb|GU381514.1|	Clinopodium nepalense voucher FR:Stainton 6024 tRNA-Leu (trnL) gene and trnL-trnF intergenic spacer, partial sequence; chloroplast	983	983	98% 0.0	94% GU381514.1	942,88	79,3%
Select seq gb|GU381511.1|	Clinopodium piperitum voucher BM<GBR-LONDON>:Vickery 454 tRNA-Leu (trnL) gene and trnL-trnF intergenic spacer, partial sequence; chloroplast	983	983	98% 0.0	94% GU381511.1	942,88	79,3%
Select seq gb|KR150198.1|	Ziziphora sp. 3 Kh112 trnL-trnF intergenic spacer region, partial sequence; chloroplast	989	989	99% 0.0	94% KR150198.1	939,05	79,0%
Select seq gb|KR150243.1|	Ziziphora sp. 2 Kh20 trnL-trnF intergenic spacer region, partial sequence; chloroplast	977	977	98% 0.0	94% KR150243.1	937,12	78,8%
Select seq gb|GU381512.1|	Clinopodium piperitum voucher E:Stainton 7320 tRNA-Leu (trnL) gene and trnL-trnF intergenic spacer, partial sequence; chloroplast	977	977	98% 0.0	94% GU381512.1	937,12	78,8%
Select seq gb|GU381510.1|	Ziziphora pamiroalaica voucher C:Murray et al. 10090 tRNA-Leu (trnL) gene and trnL-trnF intergenic spacer, partial sequence; chloroplast	977	977	98% 0.0	94% GU381510.1	937,12	78,8%
Select seq gb|GU381505.1|	Clinopodium troodi voucher W:Davis 1856 tRNA-Leu (trnL) gene and trnL-trnF intergenic spacer, partial sequence; chloroplast	977	977	98% 0.0	94% GU381505.1	937,12	78,8%
Select seq gb|GU381501.1|	Clinopodium graveolens subsp. rotundifolium voucher M:Podlech 47181 tRNA-Leu (trnL) gene and trnL-trnF intergenic spacer, partial sequence; chloroplast	977	977	98% 0.0	94% GU381501.1	937,12	78,8%
Select seq gb|GU381499.1|	Clinopodium suaveolens voucher M:Erben s.n. tRNA-Leu (trnL) gene and trnL-trnF intergenic spacer, partial sequence; chloroplast	977	977	98% 0.0	94% GU381499.1	937,12	78,8%
Select seq gb|GU381485.1|	Micromeria sphaerophylla voucher E:Lewis et al 1064 tRNA-Leu (trnL) gene and trnL-trnF intergenic spacer, partial sequence; chloroplast	976	976	98% 0.0	94% GU381485.1	936,16	78,7%
Select seq gb|JF301384.1|	Lophanthus lipskyanus voucher Vassiljeva s.n. tRNA-Leu (trnL) gene and trnL-trnF intergenic spacer, partial sequence; chloroplast	976	976	96% 0.0	92% JF301384.1	935,33	78,7%
Select seq gb|GU381507.1|	Ziziphora tenuior voucher MSB:Fayvush et al. 03-1503 tRNA-Leu (trnL) gene and trnL-trnF intergenic spacer, partial sequence; chloroplast	974	974	98% 0.0	94% GU381507.1	934,24	78,6%
Select seq gb|GU381480.1|	Agastache foeniculum voucher M:Braeuchler 2513 tRNA-Leu (trnL) gene and trnL-trnF intergenic spacer, partial sequence; chloroplast	974	974	98% 0.0	94% GU381480.1	934,24	78,6%
Select seq gb|GU381633.1|	Thymus caespititius voucher M:Heubl s.n. tRNA-Leu (trnL) gene and trnL-trnF intergenic spacer, partial sequence; chloroplast	972	972	98% 0.0	94% GU381633.1	932,33	78,4%
Select seq gb|GU381497.1|	Clinopodium acinos voucher M:Podlech 50287 tRNA-Leu (trnL) gene and trnL-trnF intergenic spacer, partial sequence; chloroplast	972	972	98% 0.0	94% GU381497.1	932,33	78,4%
Select seq gb|AY840202.1|	Origanum vulgare tRNA-Leu (trnL) gene and trnL-trnF intergenic spacer, partial sequence; chloroplast	972	972	98% 0.0	94% AY840202.1	932,33	78,4%
Select seq gb|KR150218.1|	Thymus sp. 2 Kh69 trnL-trnF intergenic spacer region, partial sequence; chloroplast	981	981	99% 0.0	94% KR150218.1	931,45	78,3%
Select seq gb|KR150238.1|	Ziziphora sp. 1 Kh75 trnL-trnF intergenic spacer region, partial sequence; chloroplast	990	990	100% 0.0	94% KR150238.1	930,60	78,3%
Select seq gb|DQ667493.1|	Lepechinia conferta isolate x234 tRNA-Leu (trnL) gene and trnL-trnF intergenic spacer, partial sequence; chloroplast	990	990	100% 0.0	94% DQ667493.1	930,60	78,3%
Select seq gb|DQ667501.1|	Ziziphora taurica isolate x262 tRNA-Leu (trnL) gene and trnL-trnF intergenic spacer, partial sequence; chloroplast	979	979	99% 0.0	94% DQ667501.1	929,56	78,2%
Select seq gb|GU381621.1|	Satureja cuneifolia voucher M:Rechinger 11142 tRNA-Leu (trnL) gene and trnL-trnF intergenic spacer, partial sequence; chloroplast	968	968	98% 0.0	94% GU381621.1	928,49	78,1%
Select seq gb|GU381612.1|	Gontscharovia popovii voucher BM<GBR-LONDON>:Schmid 2419 tRNA-Leu (trnL) gene and trnL-trnF intergenic spacer, partial sequence; chloroplast	968	968	98% 0.0	94% GU381612.1	928,49	78,1%
Select seq gb|EU244612.1|	Agastache rugosa tRNA-Leu (trnL) gene and trnL-trnF intergenic spacer, partial sequence	987	987	100% 0.0	94% EU244612.1	927,78	78,0%
Select seq gb|KR063657.1|	Thymus sibthorpii tRNA-Leu (trnL) gene, partial sequence; trnL-trnF intergenic spacer, complete sequence; and tRNA-Phe (trnF) gene, partial sequence; chloroplast	976	976	99% 0.0	94% KR063657.1	926,71	77,9%

Select seq gb|KR150229.1|	Thymus sp. 5 Kh16 trnL-trnF intergenic spacer region, partial sequence; chloroplast	976	976	99%	0.0	94% KR150229.1	926,71	77,9%
Select seq gb|KR150225.1|	Thymus sp. 4 Kh24 trnL-trnF intergenic spacer region, partial sequence; chloroplast	976	976	99%	0.0	94% KR150225.1	926,71	77,9%
Select seq gb|KR150222.1|	Thymus sp. 3 Kh65 trnL-trnF intergenic spacer region, partial sequence; chloroplast	976	976	99%	0.0	94% KR150222.1	926,71	77,9%
Select seq gb|EU556532.1|	Thymus amurensis isolate HX tRNA-Leu (trnL) gene and trnL-trnF intergenic spacer, partial sequence; chloroplast	976	976	99%	0.0	94% EU556532.1	926,71	77,9%
Select seq gb|EU556525.1|	Thymus quinquecostatus isolate CL tRNA-Leu (trnL) gene and trnL-trnF intergenic spacer, partial sequence; chloroplast	976	976	99%	0.0	94% EU556525.1	926,71	77,9%
Select seq gb|JX880022.1|	Origanum vulgare subsp. vulgare chloroplast, complete genome	985	985	100%	0.0	94% JX880022.1	925,90	77,9%
Select seq gb|JQ690290.1|	Origanum elongatum isolate H5_O_elo trnL-trnF intergenic spacer, partial sequence; chloroplast	985	985	100%	0.0	94% JQ690290.1	925,90	77,9%
Select seq gb|JQ690289.1|	Origanum rotundifolium isolate DNA3_O_rot trnL-trnF intergenic spacer, partial sequence; chloroplast	985	985	100%	0.0	94% JQ690289.1	925,90	77,9%
Select seq emb|AJ505543.1|	Origanum vulgare plastid trnL-trnF intergenic spacer, specimen voucher cult., K-000-69-19317, chase 13334 (K)	985	985	100%	0.0	94% AJ505543.1	925,90	77,9%
Select seq gb|KR063656.1|	Satureja pilosa subsp. origanita tRNA-Leu (trnL) gene, partial sequence; trnL-trnF intergenic spacer, complete sequence; and tRNA-Phe (trnF) gene, partial sequence; chloroplast	981	981	100%	0.0	94% KR063656.1	922,14	77,6%
Select seq gb|AY570452.1|	Agastache urticifolia voucher JBW 815 tRNA-Leu and trnL-trnF intergenic spacer, partial sequence; chloroplast	981	981	100%	0.0	94% AY570452.1	922,14	77,6%
Select seq gb|JQ690293.1|	Origanum dayi isolate H43_O_day trnL-trnF intergenic spacer, partial sequence; chloroplast	974	974	100%	0.0	94% JQ690293.1	915,56	77,0%
Select seq gb|KR150209.1|	Satureja sp. 3 Kh90 trnL-trnF intergenic spacer region, partial sequence; chloroplast	972	972	99%	0.0	93% KR150209.1	913,09	76,8%
Select seq gb|EF153679.1|	Prunella grandiflora tRNA-Leu (trnL) and trnL-trnF intergenic spacer, partial sequence; chloroplast	977	977	100%	0.0	93% EF153679.1	908,61	76,4%
Select seq gb|DQ667508.1|	Prunella vulgaris isolate x314 tRNA-Leu (trnL) gene and trnL-trnF intergenic spacer, partial sequence; chloroplast	977	977	100%	0.0	93% DQ667508.1	908,61	76,4%
							0,00	0,0%
							0,00	0,0%

Select for downloading or viewing reports	Kh090_ITS Description	Max score	Total score	Query cover	E value	Ident	Accession	(Ident/Cover) *Max score	Deviation from top hit
Select seq gb|GU381440.1|	Satureja pallaryi voucher TUB:Frey & Kuerschner 83-747 internal transcribed spacer 1, partial sequence; 5.8S ribosomal RNA gene, complete sequence; and internal transcribed spacer 2, partial sequence	562	562	81%	2,00E-156	96%	GU381440.1	666,07	100,0%
Select seq gb|EU823289.1|	Satureja visianii internal transcribed spacer 1, partial sequence; 5.8S ribosomal RNA gene, complete sequence; and internal transcribed spacer 2, partial sequence	586	586	85%	1,00E-163	96%	EU823289.1	661,84	99,4%
Select seq gb|EU823288.1|	Satureja subspicata internal transcribed spacer 1, partial sequence; 5.8S ribosomal RNA gene, complete sequence; and internal transcribed spacer 2, partial sequence	586	586	85%	1,00E-163	96%	EU823288.1	661,84	99,4%
Select seq gb|JQ669136.1|	Satureja thymbra voucher UCBG 2002.0540, Forbes s.n. 18S ribosomal RNA gene, internal transcribed spacer 1, 5.8S ribosomal RNA gene, internal transcribed spacer 2, and 26S ribosomal RNA gene, region	612	612	89%	2,00E-171	96%	JQ669136.1	660,13	99,1%
Select seq gb|JQ669135.1|	Satureja montana voucher UCBG 2002.0593, Forbes s.n 18S ribosomal RNA gene, internal transcribed spacer 1, 5.8S ribosomal RNA gene, internal transcribed spacer 2, and 26S ribosomal RNA gene, region	660	660	96%	0.0	95%	JQ669135.1	653,13	98,1%
Select seq gb|GU381442.1|	Satureja montana voucher M:Braeuchler 2509 internal transcribed spacer 1, partial sequence; 5.8S ribosomal RNA gene, complete sequence; and internal transcribed spacer 2, partial sequence	555	555	81%	3,00E-154	95%	GU381442.1	650,93	97,7%
Select seq gb|GU381441.1|	Satureja thymbra voucher M:Braeuchler 2896 internal transcribed spacer 1, partial sequence; 5.8S ribosomal RNA gene, complete sequence; and internal transcribed spacer 2, partial sequence	549	549	81%	2,00E-152	95%	GU381441.1	643,89	96,7%
Select seq gb|EU823290.1|	Satureja cuneifolia internal transcribed spacer 1, partial sequence; 5.8S ribosomal RNA gene, complete sequence; and internal transcribed spacer 2, partial sequence	575	575	85%	3,00E-160	95%	EU823290.1	642,65	96,5%
Select seq gb|KR150181.1|	Satureja sp. Kh70 internal transcribed spacer 1, partial sequence; 5.8S ribosomal RNA gene gene, complete sequence; and internal transcribed spacer 2 region, partial sequence	553	553	82%	1,00E-153	95%	KR150181.1	640,67	96,2%
Select seq gb|GU381443.1|	Satureja parnassica voucher M:Braeuchler 4453 internal transcribed spacer 1, partial sequence; 5.8S ribosomal RNA gene, complete sequence; and internal transcribed spacer 2, partial sequence	544	544	81%	8,00E-151	95%	GU381443.1	638,02	95,8%
Select seq gb|GU381444.1|	Satureja pilosa voucher M:Braeuchler 4448 internal transcribed spacer 1, partial sequence; 5.8S ribosomal RNA gene, complete sequence; and internal transcribed spacer 2, partial sequence	538	538	81%	4,00E-149	95%	GU381444.1	630,99	94,7%
Select seq gb|GU381445.1|	Satureja spinosa voucher M:Braeuchler 4449 internal transcribed spacer 1, partial sequence; 5.8S ribosomal RNA gene, complete sequence; and internal transcribed spacer 2, partial sequence	534	534	81%	5,00E-148	94%	GU381445.1	619,70	93,0%
Select seq gb|AY227143.1|	Satureja hortensis internal transcribed spacer 1, partial sequence; 5.8S ribosomal RNA, complete sequence; and internal transcribed spacer 2, partial sequence	553	553	85%	1,00E-153	94%	AY227143.1	611,55	91,8%
Select seq gb|GU381438.1|	Gontscharovia popovii voucher M:Vvedensky s.n. internal transcribed spacer 1, partial sequence; 5.8S ribosomal RNA gene, complete sequence; and internal transcribed spacer 2, partial sequence	516	516	81%	2,00E-142	93%	GU381438.1	592,44	88,9%
Select seq gb|GU381439.1|	Gontscharovia popovii voucher BM<GBR-LONDON>:Schmid 2419 internal transcribed spacer 1, partial sequence; 5.8S ribosomal RNA gene, complete sequence; and internal transcribed spacer 2, partial sequence	510	510	81%	8,00E-141	93%	GU381439.1	585,56	87,9%
Select seq gb|JQ669090.1|	Cunila lythrifolia voucher Rzedowski 251 18S ribosomal RNA gene, internal transcribed spacer 1, 5.8S ribosomal RNA gene, internal transcribed spacer 2, and 26S ribosomal RNA gene, region	577	577	96%	7,00E-161	92%	JQ669090.1	552,96	83,0%
Select seq gb|JQ669073.1|	Acanthomintha lanceolata voucher Crosby & Morin 14383 18S ribosomal RNA gene, internal transcribed spacer 1, 5.8S ribosomal RNA gene, internal transcribed spacer 2, and 26S ribosomal RNA gene, region	534	534	89%	5,00E-148	92%	JQ669073.1	552,00	82,9%
Select seq gb|JQ669134.1|	Rhododon ciliatus voucher Singhurst s.n. 18S ribosomal RNA gene, internal transcribed spacer 1, 5.8S ribosomal RNA gene, internal transcribed spacer 2, and 26S ribosomal RNA gene, region	573	573	96%	1,00E-159	92%	JQ669134.1	549,13	82,4%
Select seq gb|JQ669122.1|	Minthostachys mollis voucher B. Drew 345 18S ribosomal RNA gene, internal transcribed spacer 1, 5.8S ribosomal RNA gene, internal transcribed spacer 2, and 26S ribosomal RNA gene, region	573	573	96%	1,00E-159	92%	JQ669122.1	549,13	82,4%
Select seq gb|DQ667333.1|	Acanthomintha lanceolata isolate x545 18S ribosomal RNA gene, partial sequence; internal transcribed spacer 1, 5.8S ribosomal RNA gene, and internal transcribed spacer 2, complete sequence; and 28S ribosomal RNA gene, partial sequence	566	566	95%	2,00E-157	92%	DQ667333.1	548,13	82,3%
Select seq gb|DQ667238.1|	Conradina canescens isolate x069 18S ribosomal RNA gene, partial sequence; internal transcribed spacer 1, 5.8S ribosomal RNA gene, and internal transcribed spacer 2, complete sequence; and 28S ribosomal RNA gene, partial sequence	566	566	95%	2,00E-157	92%	DQ667238.1	548,13	82,3%
Select seq gb|JQ669092.1|	Cunila origanoides voucher Stoots s.n. 18S ribosomal RNA gene, internal transcribed spacer 1, 5.8S ribosomal RNA gene, internal transcribed spacer 2, and 26S ribosomal RNA gene, region	571	571	96%	3,00E-159	92%	JQ669092.1	547,21	82,2%
Select seq gb|DQ667312.1|	Rhododon ciliatus isolate x260 18S ribosomal RNA gene, partial sequence; internal transcribed spacer 1, 5.8S ribosomal RNA gene, and internal transcribed spacer 2, complete sequence; and 28S ribosomal RNA gene, partial sequence	564	564	95%	6,00E-157	92%	DQ667312.1	546,19	82,0%
Select seq gb|JQ669088.1|	Conradina grandiflora voucher Bok Tower Gardens 38717 B. Drew s.n. 18S ribosomal RNA gene, internal transcribed spacer 1, 5.8S ribosomal RNA gene, internal transcribed spacer 2, and 26S ribosomal RNA gene, region	568	568	96%	4,00E-158	92%	JQ669088.1	544,33	81,7%
Select seq gb|JQ669121.1|	Clinopodium thymifolium voucher M. Kintgen s.n. 18S ribosomal RNA gene, internal transcribed spacer 1, 5.8S ribosomal RNA gene, internal transcribed spacer 2, and 26S ribosomal RNA gene, region	568	568	96%	4,00E-158	91%	JQ669121.1	538,42	80,8%
Select seq gb|JQ669093.1|	Cunila pycnantha voucher Ruiz 3150 18S ribosomal RNA gene, internal transcribed spacer 1, 5.8S ribosomal RNA gene, internal transcribed spacer 2, and 26S ribosomal RNA gene, region	568	568	96%	4,00E-158	91%	JQ669093.1	538,42	80,8%

Select seq gb|JQ669081.1|	Micromeria douglasii voucher B. Drew 206 18S ribosomal RNA gene, internal transcribed spacer 1, 5.8S ribosomal RNA gene, internal transcribed spacer 2, and 26S ribosomal RNA gene, region	566	566	96%	2,00E-157	91% JQ669081.1	536,52	80,5%
Select seq gb|JQ669139.1|	Zataria multiflora voucher Rechinger 51885 18S ribosomal RNA gene, internal transcribed spacer 1, 5.8S ribosomal RNA gene, internal transcribed spacer 2, and 26S ribosomal RNA gene, region	562	562	96%	2,00E-156	91% JQ669139.1	532,73	80,0%
Select seq gb|JQ669125.1|	Monardella villosa voucher B. Drew 66 18S ribosomal RNA gene, internal transcribed spacer 1, 5.8S ribosomal RNA gene, internal transcribed spacer 2, and 26S ribosomal RNA gene, region	562	562	96%	2,00E-156	91% JQ669125.1	532,73	80,0%
Select seq gb|JQ669089.1|	Cuminia fernandezia voucher Stuessy et al., 11580 18S ribosomal RNA gene, internal transcribed spacer 1, 5.8S ribosomal RNA gene, internal transcribed spacer 2, and 26S ribosomal RNA gene, region	521	521	89%	4,00E-144	91% JQ669089.1	532,71	80,0%
Select seq gb|JQ669137.1|	Thymbra capitata voucher UCBG 96.0817 s.n. 18S ribosomal RNA gene, internal transcribed spacer 1, 5.8S ribosomal RNA gene, internal transcribed spacer 2, and 26S ribosomal RNA gene, region	560	560	96%	8,00E-156	91% JQ669137.1	530,83	79,7%
Select seq gb|JQ669100.1|	Glechon thymoides voucher C. Mondin 1421 18S ribosomal RNA gene, internal transcribed spacer 1, 5.8S ribosomal RNA gene, internal transcribed spacer 2, and 26S ribosomal RNA gene, region	560	560	96%	8,00E-156	91% JQ669100.1	530,83	79,7%
Select seq gb|JQ669091.1|	Cunila microcephala voucher Sytsma 7247 18S ribosomal RNA gene, internal transcribed spacer 1, 5.8S ribosomal RNA gene, internal transcribed spacer 2, and 26S ribosomal RNA gene, region	560	560	96%	8,00E-156	91% JQ669091.1	530,83	79,7%
Select seq gb|JQ669123.1|	Minthostachys mollis voucher B. Drew 349 18S ribosomal RNA gene, internal transcribed spacer 1, 5.8S ribosomal RNA gene, internal transcribed spacer 2, and 26S ribosomal RNA gene, region	518	518	89%	5,00E-143	91% JQ669123.1	529,64	79,5%
Select seq gb|JQ669119.1|	Micromeria juliana voucher Forbes s.n. 18S ribosomal RNA gene, internal transcribed spacer 1, 5.8S ribosomal RNA gene, internal transcribed spacer 2, and 26S ribosomal RNA gene, region	523	523	90%	1,00E-144	91% JQ669119.1	528,81	79,4%
Select seq gb|DQ667310.1|	Glechon thymoides isolate x256 18S ribosomal RNA gene, partial sequence; internal transcribed spacer 1, 5.8S ribosomal RNA gene, and internal transcribed spacer 2, complete sequence; and 28S ribosomal RNA gene, partial sequence	562	562	97%	2,00E-156	91% DQ667310.1	527,24	79,2%
Select seq gb|DQ667305.1|	Cunila microcephala isolate x226 18S ribosomal RNA gene, partial sequence; internal transcribed spacer 1, 5.8S ribosomal RNA gene, and internal transcribed spacer 2, complete sequence; and 28S ribosomal RNA gene, partial sequence	562	562	97%	2,00E-156	91% DQ667305.1	527,24	79,2%
Select seq gb|JQ669101.1|	Hedeoma multiflora voucher K. Sytsma 7243 18S ribosomal RNA gene, internal transcribed spacer 1, 5.8S ribosomal RNA gene, internal transcribed spacer 2, and 26S ribosomal RNA gene, region	556	556	96%	1,00E-154	91% JQ669101.1	527,04	79,1%
Select seq gb|JQ669118.1|	Clinopodium dalmaticum voucher M. Kintgen s.n. 18S ribosomal RNA gene, internal transcribed spacer 1, 5.8S ribosomal RNA gene, internal transcribed spacer 2, and 26S ribosomal RNA gene, region	514	514	89%	6,00E-142	91% JQ669118.1	525,55	78,9%
Select seq gb|DQ667233.1|	Clinopodium coccineum isolate x058 18S ribosomal RNA gene, partial sequence; internal transcribed spacer 1, 5.8S ribosomal RNA gene, and internal transcribed spacer 2, complete sequence; and 28S ribosomal RNA gene, partial sequence	560	560	97%	8,00E-156	91% DQ667233.1	525,36	78,9%
Select seq gb|JQ669133.1|	Rhabdocaulon strictum voucher Singhurst s.n 18S ribosomal RNA gene, internal transcribed spacer 1, 5.8S ribosomal RNA gene, internal transcribed spacer 2, and 26S ribosomal RNA gene, region	553	553	96%	1,00E-153	91% JQ669133.1	524,20	78,7%
Select seq gb|JQ669115.1|	Mentha arvensis voucher B. Drew 82 18S ribosomal RNA gene, internal transcribed spacer 1, 5.8S ribosomal RNA gene, internal transcribed spacer 2, and 26S ribosomal RNA gene, region	553	553	96%	1,00E-153	91% JQ669115.1	524,20	78,7%
Select seq gb|JQ669083.1|	Clinopodium macrostemum voucher B. Drew 147 18S ribosomal RNA gene, internal transcribed spacer 1, 5.8S ribosomal RNA gene, internal transcribed spacer 2, and 26S ribosomal RNA gene, region	553	553	96%	1,00E-153	91% JQ669083.1	524,20	78,7%
Select seq gb|JQ669077.1|	Blephilia hirsuta voucher T. Cochrane 13609 18S ribosomal RNA gene, internal transcribed spacer 1, 5.8S ribosomal RNA gene, internal transcribed spacer 2, and 26S ribosomal RNA gene, region	553	553	96%	1,00E-153	91% JQ669077.1	524,20	78,7%
Select seq gb|DQ667316.1|	Cunila incana isolate x296 18S ribosomal RNA gene, partial sequence; internal transcribed spacer 1, 5.8S ribosomal RNA gene, and internal transcribed spacer 2, complete sequence; and 28S ribosomal RNA gene, partial sequence	558	558	97%	3,00E-155	91% DQ667316.1	523,48	78,6%
Select seq gb|DQ667325.1|	Mentha arvensis isolate x424 18S ribosomal RNA gene, partial sequence; internal transcribed spacer 1, 5.8S ribosomal RNA gene, and internal transcribed spacer 2, complete sequence; and 28S ribosomal RNA gene, partial sequence	551	551	96%	5,00E-153	91% DQ667325.1	522,30	78,4%
Select seq gb|JQ669102.1|	Hesperozygis rhododon voucher G. Hatschbach 44939 18S ribosomal RNA gene, internal transcribed spacer 1, 5.8S ribosomal RNA gene, internal transcribed spacer 2, and 26S ribosomal RNA gene, region	545	545	95%	2,00E-151	91% JQ669102.1	522,05	78,4%
Select seq gb|DQ667311.1|	Poliomintha palmeri isolate x259 18S ribosomal RNA gene, partial sequence; internal transcribed spacer 1, 5.8S ribosomal RNA gene, and internal transcribed spacer 2, complete sequence; and 28S ribosomal RNA gene, partial sequence	556	556	97%	1,00E-154	91% DQ667311.1	521,61	78,3%
Select seq gb|DQ667303.1|	Glechon marifolia isolate x223 18S ribosomal RNA gene, partial sequence; internal transcribed spacer 1, 5.8S ribosomal RNA gene, and internal transcribed spacer 2, complete sequence; and 28S ribosomal RNA gene, partial sequence	556	556	97%	1,00E-154	91% DQ667303.1	521,61	78,3%
Select seq gb|DQ667237.1|	Clinopodium ashei isolate x068 18S ribosomal RNA gene, partial sequence; internal transcribed spacer 1, 5.8S ribosomal RNA gene, and internal transcribed spacer 2, complete sequence; and 28S ribosomal RNA gene, partial sequence	556	556	97%	1,00E-154	91% DQ667237.1	521,61	78,3%
Select seq gb|KC473228.1|	Mentha canadensis isolate 511190201 18S ribosomal RNA gene, partial sequence; internal transcribed spacer 1, 5.8S ribosomal RNA gene, and internal transcribed spacer 2, complete sequence; and 28S ribosomal RNA gene, partial sequence	518	518	91%	5,00E-143	91% KC473228.1	518,00	77,8%
Select seq gb|DQ667332.1|	Salvia cynica isolate x537 18S ribosomal RNA gene, partial sequence; internal transcribed spacer 1, 5.8S ribosomal RNA gene, and internal transcribed spacer 2, complete sequence; and 28S ribosomal RNA gene, partial sequence	551	551	97%	5,00E-153	91% DQ667332.1	516,92	77,6%
Select seq gb|DQ667244.1|	Mentha spicata isolate x077 18S ribosomal RNA gene, partial sequence; internal transcribed spacer 1, 5.8S ribosomal RNA gene, and internal transcribed spacer 2, complete sequence; and 28S ribosomal RNA gene, partial sequence	542	542	96%	3,00E-150	91% DQ667244.1	513,77	77,1%
Select seq gb|JQ669104.1|	Hoehnea epilobioides voucher G. Hatschbach 8/3/1984 18S ribosomal RNA gene, internal transcribed spacer 1, 5.8S ribosomal RNA gene, internal transcribed spacer 2, and 26S ribosomal RNA gene, region	505	505	89%	4,00E-139	90% JQ669104.1	510,67	76,7%

Select seq gb|JQ669128.1|	Pogogyne douglasii voucher UCBG 91.1071 s.n. 18S ribosomal RNA gene, internal transcribed spacer 1, 5.8S ribosomal RNA gene, internal transcribed spacer 2, and 26S ribosomal RNA gene, region	544	544	96%	8,00E-151	90%	JQ669128.1	510,00	76,6%
Select seq gb|KF735667.1|	Mentha aquatica strain HY1 18S ribosomal RNA gene, partial sequence; internal transcribed spacer 1 and 5.8S ribosomal RNA gene, complete sequence; and internal transcribed spacer 2, partial sequence	532	532	94%	2,00E-147	90%	KF735667.1	509,36	76,5%
Select seq gb|EF373641.1|	Salvia cynica clone 3 18S ribosomal RNA gene, partial sequence; internal transcribed spacer 1, 5.8S ribosomal RNA gene, and internal transcribed spacer 2, complete sequence; and 28S ribosomal RNA gene, partial sequence	512	512	91%	2,00E-141	90%	EF373641.1	506,37	76,0%
Select seq gb|DQ667331.1|	Pogogyne floribunda isolate x534 18S ribosomal RNA gene, partial sequence; internal transcribed spacer 1, 5.8S ribosomal RNA gene, and internal transcribed spacer 2, complete sequence; and 28S ribosomal RNA gene, partial sequence	545	545	97%	2,00E-151	90%	DQ667331.1	505,67	75,9%
Select seq gb|DQ667234.1|	Dicerandra odoratissima isolate x059 18S ribosomal RNA gene, partial sequence; internal transcribed spacer 1, 5.8S ribosomal RNA gene, and internal transcribed spacer 2, complete sequence; and 28S ribosomal RNA gene, partial sequence	545	545	97%	2,00E-151	90%	DQ667234.1	505,67	75,9%
Select seq gb|KC473234.1|	Salvia cyclostegia isolate 511190209 18S ribosomal RNA gene, partial sequence; internal transcribed spacer 1, 5.8S ribosomal RNA gene, and internal transcribed spacer 2, complete sequence; and 28S ribosomal RNA gene, partial sequence	510	510	91%	8,00E-141	90%	KC473234.1	504,40	75,7%
Select seq gb|EF373642.1|	Salvia omeiana clone 1 18S ribosomal RNA gene, partial sequence; internal transcribed spacer 1, 5.8S ribosomal RNA gene, and internal transcribed spacer 2, complete sequence; and 28S ribosomal RNA gene, partial sequence	510	510	91%	8,00E-141	90%	EF373642.1	504,40	75,7%
Select seq gb|EF373640.1|	Salvia cynica clone 2 18S ribosomal RNA gene, partial sequence; internal transcribed spacer 1, 5.8S ribosomal RNA gene, and internal transcribed spacer 2, complete sequence; and 28S ribosomal RNA gene, partial sequence	510	510	91%	8,00E-141	90%	EF373640.1	504,40	75,7%
Select seq gb|JQ669117.1|	Mentha pulegium voucher Riina 1574 18S ribosomal RNA gene, internal transcribed spacer 1, 5.8S ribosomal RNA gene, internal transcribed spacer 2, and 26S ribosomal RNA gene, region	538	538	96%	4,00E-149	90%	JQ669117.1	504,38	75,7%
Select seq gb|JQ669082.1|	Clinopodium gracile voucher Yau 8715 18S ribosomal RNA gene, internal transcribed spacer 1, 5.8S ribosomal RNA gene, internal transcribed spacer 2, and 26S ribosomal RNA gene, region	538	538	96%	4,00E-149	90%	JQ669082.1	504,38	75,7%
Select seq gb|JQ669120.1|	Micromeria lanata voucher B. Drew s.n. 18S ribosomal RNA gene, internal transcribed spacer 1, 5.8S ribosomal RNA gene, internal transcribed spacer 2, and 26S ribosomal RNA gene, region	532	532	95%	2,00E-147	90%	JQ669120.1	504,00	75,7%
Select seq gb|JQ669072.1|	Acanthomintha duttonii voucher UCBG H. Forbes s.n. 18S ribosomal RNA gene, internal transcribed spacer 1, 5.8S ribosomal RNA gene, internal transcribed spacer 2, and 26S ribosomal RNA gene, region	532	532	95%	2,00E-147	90%	JQ669072.1	504,00	75,7%
Select seq gb|JN407476.1|	Mentha canadensis isolate shawpc0994I 18S ribosomal RNA gene, partial sequence; internal transcribed spacer 1, 5.8S ribosomal RNA gene, and internal transcribed spacer 2, complete sequence; and 28S ribosomal RNA gene, partial sequence	508	508	91%	3,00E-140	90%	JN407476.1	502,42	75,4%
Select seq gb|KJ397260.1|	Salvia przewalskii voucher SP201301 18S ribosomal RNA gene, partial sequence; internal transcribed spacer 1, 5.8S ribosomal RNA gene, and internal transcribed spacer 2, complete sequence; and 26S ribosomal RNA gene, partial sequence	523	523	94%	1,00E-144	90%	KJ397260.1	500,74	75,2%
Select seq gb|KJ397259.1|	Salvia digitaloides voucher SD201301 18S ribosomal RNA gene, partial sequence; internal transcribed spacer 1, 5.8S ribosomal RNA gene, and internal transcribed spacer 2, complete sequence; and 26S ribosomal RNA gene, partial sequence	523	523	94%	1,00E-144	90%	KJ397259.1	500,74	75,2%
Select seq gb|KF735668.1|	Mentha aquatica strain BS 18S ribosomal RNA gene, partial sequence; internal transcribed spacer 1 and 5.8S ribosomal RNA gene, complete sequence; and internal transcribed spacer 2, partial sequence	521	521	94%	4,00E-144	90%	KF735668.1	498,83	74,9%
Select seq gb|KF735666.1|	Mentha aquatica strain HY 18S ribosomal RNA gene, partial sequence; internal transcribed spacer 1 and 5.8S ribosomal RNA gene, complete sequence; and internal transcribed spacer 2, partial sequence	521	521	94%	4,00E-144	90%	KF735666.1	498,83	74,9%
Select seq gb|DQ667254.1|	Salvia przewalskii isolate x111 18S ribosomal RNA gene, partial sequence; internal transcribed spacer 1, 5.8S ribosomal RNA gene, and internal transcribed spacer 2, complete sequence; and 28S ribosomal RNA gene, partial sequence	525	525	95%	3,00E-145	90%	DQ667254.1	497,37	74,7%
Select seq gb|JQ669138.1|	Thymus pulegioides voucher Riina 1577 18S ribosomal RNA gene, internal transcribed spacer 1, 5.8S ribosomal RNA gene, internal transcribed spacer 2, and 26S ribosomal RNA gene, region	529	529	96%	2,00E-146	90%	JQ669138.1	495,94	74,5%
Select seq gb|DQ667255.1|	Salvia digitaloides isolate x112 18S ribosomal RNA gene, partial sequence; internal transcribed spacer 1, 5.8S ribosomal RNA gene, and internal transcribed spacer 2, complete sequence; and 28S ribosomal RNA gene, partial sequence	532	532	97%	2,00E-147	90%	DQ667255.1	493,61	74,1%
Select seq gb|DQ667289.1|	Salvia roborowskii isolate x189 18S ribosomal RNA gene, partial sequence; internal transcribed spacer 1, 5.8S ribosomal RNA gene, and internal transcribed spacer 2, complete sequence; and 28S ribosomal RNA gene, partial sequence	520	520	95%	1,00E-143	90%	DQ667289.1	492,63	74,0%
Select seq gb|KR150165.1|	Thymus sp. Kh8 internal transcribed spacer 1, partial sequence; 5.8S ribosomal RNA gene gene, complete sequence; and internal transcribed spacer 2 region, partial sequence	547	547	100%	6,00E-152	90%	KR150165.1	492,30	73,9%
Select seq gb|KR150146.1|	Thymus sp. Kh57 internal transcribed spacer 1, partial sequence; 5.8S ribosomal RNA gene gene, complete sequence; and internal transcribed spacer 2 region, partial sequence	547	547	100%	6,00E-152	90%	KR150146.1	492,30	73,9%
Select seq gb|DQ667252.1|	Dorystaechas hastata isolate x108 18S ribosomal RNA gene, partial sequence; internal transcribed spacer 1, 5.8S ribosomal RNA gene, and internal transcribed spacer 2, complete sequence; and 28S ribosomal RNA gene, partial sequence	518	518	95%	5,00E-143	90%	DQ667252.1	490,74	73,7%
Select seq gb|DQ667318.1|	Monarda fistulosa isolate x312 18S ribosomal RNA gene, partial sequence; internal transcribed spacer 1, 5.8S ribosomal RNA gene, and internal transcribed spacer 2, complete sequence; and 28S ribosomal RNA gene, partial sequence	523	523	96%	1,00E-144	90%	DQ667318.1	490,31	73,6%
Select seq gb|JQ669132.1|	Rhabdocaulon erythrostachys voucher Sergio A. L. Bordignon 851 18S ribosomal RNA gene, internal transcribed spacer 1, 5.8S ribosomal RNA gene, internal transcribed spacer 2, and 26S ribosomal RNA gene, region	521	521	96%	4,00E-144	90%	JQ669132.1	488,44	73,3%
Select seq gb|DQ667250.1|	Salvia glutinosa isolate x094 18S ribosomal RNA gene, partial sequence; internal transcribed spacer 1, 5.8S ribosomal RNA gene, and internal transcribed spacer 2, complete sequence; and 28S ribosomal RNA gene, partial sequence	510	510	94%	8,00E-141	90%	DQ667250.1	488,30	73,3%
Select seq gb|JQ669094.1|	Cyclotrichium stamineum voucher Gillett 9444 18S ribosomal RNA gene, internal transcribed spacer 1, 5.8S ribosomal RNA gene, internal transcribed spacer 2, and 26S ribosomal RNA gene, region	520	520	95%	1,00E-143	89%	JQ669094.1	487,16	73,1%

Select seq gb|DQ667247.1|	Agastache urticifolia isolate x090 18S ribosomal RNA gene, partial sequence; internal transcribed spacer 1, 5.8S ribosomal RNA gene, and internal transcribed spacer 2, complete sequence; and 28S ribosomal RNA gene, partial sequence	518	518	95%	5,00E-143	89%	DQ667247.1	485,28	72,9%
Select seq gb|JQ669078.1|	Bystropogon origanifolius voucher B. Drew s.n. 18S ribosomal RNA gene, internal transcribed spacer 1, 5.8S ribosomal RNA gene, internal transcribed spacer 2, and 26S ribosomal RNA gene, region	514	514	95%	6,00E-142	89%	JQ669078.1	481,54	72,3%
Select seq gb|JQ669099.1|	Glechoma hederacea voucher B. Drew 69 18S ribosomal RNA gene, internal transcribed spacer 1, 5.8S ribosomal RNA gene, internal transcribed spacer 2, and 26S ribosomal RNA gene, region	518	518	96%	5,00E-143	89%	JQ669099.1	480,23	72,1%
Select seq emb|AJ421001.1|	Agastache barberi 5.8S rRNA gene, internal transcribed spacer 1 (ITS1) and internal transcribed spacer 2 (ITS2)	536	536	100%	1,00E-148	89%	AJ421001.1	477,04	71,6%
Select seq emb|AJ421000.1|	Agastache cana 5.8S rRNA gene, internal transcribed spacer 1 (ITS1) and internal transcribed spacer 2 (ITS2)	534	534	100%	5,00E-148	89%	AJ421000.1	475,26	71,4%
Select seq gb|JQ669140.1|	Ziziphora clinopodioides voucher Denver Bot. Gardens 980177 18S ribosomal RNA gene, internal transcribed spacer 1, 5.8S ribosomal RNA gene, internal transcribed spacer 2, and 26S ribosomal RNA gene, region	512	512	96%	2,00E-141	89%	JQ669140.1	474,67	71,3%
Select seq gb|JQ669096.1|	Dracocephalum bullatum voucher Boufford et al. 31785 18S ribosomal RNA gene, internal transcribed spacer 1, 5.8S ribosomal RNA gene, internal transcribed spacer 2, and 26S ribosomal RNA gene, region	512	512	96%	2,00E-141	89%	JQ669096.1	474,67	71,3%
Select seq gb|JQ669074.1|	Clinopodium acinos voucher Judziewicz 14160 18S ribosomal RNA gene, internal transcribed spacer 1, 5.8S ribosomal RNA gene, internal transcribed spacer 2, and 26S ribosomal RNA gene, region	512	512	96%	2,00E-141	89%	JQ669074.1	474,67	71,3%
Select seq gb|KF800643.1|	Uncultured eukaryote clone CMH554 18S ribosomal RNA gene, partial sequence; internal transcribed spacer 1, 5.8S ribosomal RNA gene, and internal transcribed spacer 2, complete sequence; and 28S ribosomal RNA gene, partial sequence	531	531	100%	6,00E-147	89%	KF800643.1	472,59	71,0%
Select seq emb|AJ420997.1|	Lallemantia peltata 5.8S rRNA gene, internal transcribed spacer 1 (ITS1) and internal transcribed spacer 2 (ITS2)	529	529	100%	2,00E-146	89%	AJ420997.1	470,81	70,7%
Select seq gb|JQ669110.1|	Lycopus lucidus var. maackianus voucher Julita 661 18S ribosomal RNA gene, internal transcribed spacer 1, 5.8S ribosomal RNA gene, internal transcribed spacer 2, and 26S ribosomal RNA gene, region	507	507	96%	1,00E-139	89%	JQ669110.1	470,03	70,6%
Select seq gb|DQ667306.1|	Lepechinia lancifolia isolate x232 18S ribosomal RNA gene, partial sequence; internal transcribed spacer 1, 5.8S ribosomal RNA gene, and internal transcribed spacer 2, complete sequence; and 28S ribosomal RNA gene, partial sequence	508	508	97%	3,00E-140	89%	DQ667306.1	466,10	70,0%
Select seq gb|JN802670.1|	Schizonepeta tenuifolia clone HB11 18S ribosomal RNA gene, partial sequence; internal transcribed spacer 1, 5.8S ribosomal RNA gene, and internal transcribed spacer 2, complete sequence; and 28S ribosomal RNA gene, partial sequence	507	507	97%	1,00E-139	89%	JN802670.1	465,19	69,8%
Select seq gb|KM051459.1|	Schizonepeta tenuifolia isolate TKM201404 18S ribosomal RNA gene, partial sequence; internal transcribed spacer 1, 5.8S ribosomal RNA gene, and internal transcribed spacer 2, complete sequence; and 26S ribosomal RNA gene, partial sequence	518	518	100%	5,00E-143	89%	KM051459.1	461,02	69,2%
Select seq gb|EU383034.1|	Schizonepeta tenuifolia internal transcribed spacer 1, partial sequence; 5.8S ribosomal RNA gene, complete sequence; and internal transcribed spacer 2, partial sequence	518	518	100%	5,00E-143	89%	EU383034.1	461,02	69,2%
Select seq emb|LN590706.1|	Cyprinus carpio genome assembly common carp genome, scaffold: LG36, chromosome: 36	516	516	100%	2,00E-142	89%	LN590706.1	459,24	68,9%
Select seq emb|AJ420998.1|	Dracocephalum kotschyi 5.8S rRNA gene, internal transcribed spacer 1 (ITS1) and internal transcribed spacer 2 (ITS2)	512	512	100%	2,00E-141	88%	AJ420998.1	450,56	67,6%

Select for downloading or viewing reports	Kh090_trnL Description	Max score	Total score	Query cover	E value	Ident	Accession	(Ident/Cover)* Max score	Deviation from top hit
Select seq gb|GU381619.1|	Satureja mutica voucher M:Akhani 12362 tRNA-Leu (trnL) gene and trnL-trnF intergenic spacer, partial sequence; chloroplast	1238	1238	99%	0.0	100%	GU381619.1	1250,51	100,0%
Select seq gb|KR063656.1|	Satureja pilosa subsp. origanita tRNA-Leu (trnL) gene, partial sequence; trnL-trnF intergenic spacer, complete sequence; and tRNA-Phe (trnF) gene, partial sequence; chloroplast	1242	1242	99%	0.0	99%	KR063656.1	1242,00	99,3%
Select seq gb|JQ669067.1|	Satureja montana voucher UCBG 2002.0593, Forbes s.n tRNA-Leu (trnL) gene and trnL-trnF intergenic spacer, partial sequence; plastid	1227	1227	98%	0.0	99%	JQ669067.1	1239,52	99,1%
Select seq gb|GU381621.1|	Satureja cuneifolia voucher M:Rechinger 11142 tRNA-Leu (trnL) gene and trnL-trnF intergenic spacer, partial sequence; chloroplast	1232	1232	99%	0.0	99%	GU381621.1	1232,00	98,5%
Select seq gb|GU381615.1|	Satureja innota voucher M:Barra et al. 2484GL tRNA-Leu (trnL) gene and trnL-trnF intergenic spacer, partial sequence; chloroplast	1232	1232	99%	0.0	99%	GU381615.1	1232,00	98,5%
Select seq gb|GU381612.1|	Gontscharovia popovii voucher BM<GBR-LONDON>:Schmid 2419 tRNA-Leu (trnL) gene and trnL-trnF intergenic spacer, partial sequence; chloroplast	1232	1232	99%	0.0	99%	GU381612.1	1232,00	98,5%
Select seq gb|AY840179.1|	Satureja montana tRNA-Leu (trnL) gene and trnL-trnF intergenic spacer, partial sequence; chloroplast	1232	1232	99%	0.0	99%	AY840179.1	1232,00	98,5%
Select seq gb|GU381614.1|	Satureja thymbra voucher M:Braeuchler 2896 tRNA-Leu (trnL) gene and trnL-trnF intergenic spacer, partial sequence; chloroplast	1227	1227	99%	0.0	99%	GU381614.1	1227,00	98,1%
Select seq gb|GU381611.1|	Gontscharovia popovii voucher M:Vvedensky s.n. tRNA-Leu (trnL) gene and trnL-trnF intergenic spacer, partial sequence; chloroplast	1227	1227	99%	0.0	99%	GU381611.1	1227,00	98,1%
Select seq gb|JQ669068.1|	Satureja thymbra voucher UCBG 2002.0540, Forbes s.n. tRNA-Leu (trnL) gene and trnL-trnF intergenic spacer, partial sequence; plastid	1210	1210	98%	0.0	99%	JQ669068.1	1222,35	97,7%
Select seq gb|AY506611.1|	Satureja hortensis tRNA-Leu (trnL) gene and trnL-trnF intergenic spacer, partial sequence; chloroplast	1184	1184	96%	0.0	99%	AY506611.1	1221,00	97,6%
Select seq gb|GU381623.1|	Satureja spicigera voucher M:Kartli et al. 2726 tRNA-Leu (trnL) gene and trnL-trnF intergenic spacer, partial sequence; chloroplast	1216	1216	99%	0.0	99%	GU381623.1	1216,00	97,2%
Select seq gb|GU381620.1|	Satureja hortensis voucher M:Braeuchler 2422 tRNA-Leu (trnL) gene and trnL-trnF intergenic spacer, partial sequence; chloroplast	1208	1208	99%	0.0	99%	GU381620.1	1208,00	96,6%
Select seq gb|KR150240.1|	Satureja sp. 2 Kh70 trnL-trnF intergenic spacer region, partial sequence; chloroplast	1206	1206	99%	0.0	99%	KR150240.1	1206,00	96,4%
Select seq gb|GU381622.1|	Satureja intermedia voucher M:Rechinger 43441 tRNA-Leu (trnL) gene and trnL-trnF intergenic spacer, partial sequence; chloroplast	1205	1205	99%	0.0	99%	GU381622.1	1205,00	96,4%
Select seq gb|GU381629.1|	Thymbra capitata voucher M:Braeuchler 2518 tRNA-Leu (trnL) gene and trnL-trnF intergenic spacer, partial sequence; chloroplast	1151	1151	99%	0.0	98%	GU381629.1	1139,37	91,1%
Select seq gb|GU381484.1|	Micromeria flagellaris voucher E:van der Werff & McPherson 13570 tRNA-Leu (trnL) gene and trnL-trnF intergenic spacer, partial sequence; chloroplast	1151	1151	99%	0.0	98%	GU381484.1	1139,37	91,1%
Select seq gb|GU381483.1|	Micromeria flagellaris voucher E:Clement et al 2140 tRNA-Leu (trnL) gene and trnL-trnF intergenic spacer, partial sequence; chloroplast	1151	1151	99%	0.0	98%	GU381483.1	1139,37	91,1%
Select seq gb|JF301401.1|	Thymbra capitata voucher UCBG 96.0817 tRNA-Leu (trnL) gene and trnL-trnF intergenic spacer, partial sequence; chloroplast	1125	1125	97%	0.0	98%	JF301401.1	1136,60	90,9%
Select seq gb|AY840207.1|	Thymbra spicata tRNA-Leu (trnL) gene and trnL-trnF intergenic spacer, partial sequence; chloroplast	1147	1147	99%	0.0	98%	AY840207.1	1135,41	90,8%
Select seq gb|GU381632.1|	Thymbra spicata voucher M:Braeuchler 4548 tRNA-Leu (trnL) gene and trnL-trnF intergenic spacer, partial sequence; chloroplast	1142	1142	99%	0.0	98%	GU381632.1	1130,46	90,4%
Select seq gb|GU381627.1|	Thymbra sintenisii subsp. isaurica voucher E:Goener 12628 tRNA-Leu (trnL) gene and trnL-trnF intergenic spacer, partial sequence; chloroplast	1140	1140	99%	0.0	98%	GU381627.1	1128,48	90,2%
Select seq gb|GU381513.1|	Clinopodium nepalense voucher BM<GBR-LONDON>:Mikage et al. 9550294 tRNA-Leu (trnL) gene and trnL-trnF intergenic spacer, partial sequence; chloroplast	1107	1107	96%	0.0	97%	GU381513.1	1118,53	89,4%
Select seq gb|GU381517.1|	Clinopodium barosmum voucher BM<GBR-LONDON>:McLaren N193 tRNA-Leu (trnL) gene and trnL-trnF intergenic spacer, partial sequence; chloroplast	1134	1134	99%	0.0	97%	GU381517.1	1111,09	88,9%
Select seq gb|GU381516.1|	Clinopodium wardii voucher BM<GBR-LONDON>:Ludlow et al. 14234 tRNA-Leu (trnL) gene and trnL-trnF intergenic spacer, partial sequence; chloroplast	1134	1134	99%	0.0	97%	GU381516.1	1111,09	88,9%
Select seq gb|GU381515.1|	Clinopodium hydaspidis voucher BM<GBR-LONDON>:Mohd 133 tRNA-Leu (trnL) gene and trnL-trnF intergenic spacer, partial sequence; chloroplast	1134	1134	99%	0.0	97%	GU381515.1	1111,09	88,9%

<table>
<tr><td>Select seq gb|GU381514.1|</td><td>Clinopodium nepalense voucher FR:Stainton 6024 tRNA-Leu (trnL) gene and trnL-trnF intergenic spacer, partial sequence; chloroplast</td><td>1134</td><td>1134</td><td>99%</td><td>0.0</td><td>97%</td><td>GU381514.1</td><td>1111,09</td><td>88,9%</td></tr>
<tr><td>Select seq gb|GU381511.1|</td><td>Clinopodium piperitum voucher BM<GBR-LONDON>:Vickery 454 tRNA-Leu (trnL) gene and trnL-trnF intergenic spacer, partial sequence; chloroplast</td><td>1134</td><td>1134</td><td>99%</td><td>0.0</td><td>97%</td><td>GU381511.1</td><td>1111,09</td><td>88,9%</td></tr>
<tr><td>Select seq gb|GU381495.1|</td><td>Killickia pilosa voucher M:Braeuchler 3810 tRNA-Leu (trnL) gene and trnL-trnF intergenic spacer, partial sequence; chloroplast</td><td>1110</td><td>1110</td><td>97%</td><td>0.0</td><td>97%</td><td>GU381495.1</td><td>1110,00</td><td>88,8%</td></tr>
<tr><td>Select seq gb|GU381512.1|</td><td>Clinopodium piperitum voucher E:Stainton 7320 tRNA-Leu (trnL) gene and trnL-trnF intergenic spacer, partial sequence; chloroplast</td><td>1129</td><td>1129</td><td>99%</td><td>0.0</td><td>97%</td><td>GU381512.1</td><td>1106,19</td><td>88,5%</td></tr>
<tr><td>Select seq gb|JX880022.1|</td><td>Origanum vulgare subsp. vulgare chloroplast, complete genome</td><td>1127</td><td>1127</td><td>99%</td><td>0.0</td><td>97%</td><td>JX880022.1</td><td>1104,23</td><td>88,3%</td></tr>
<tr><td>Select seq gb|JQ690290.1|</td><td>Origanum elongatum isolate H5_O_elo trnL-trnF intergenic spacer, partial sequence; chloroplast</td><td>1127</td><td>1127</td><td>99%</td><td>0.0</td><td>97%</td><td>JQ690290.1</td><td>1104,23</td><td>88,3%</td></tr>
<tr><td>Select seq gb|JQ690289.1|</td><td>Origanum rotundifolium isolate DNA3_O_rot trnL-trnF intergenic spacer, partial sequence; chloroplast</td><td>1127</td><td>1127</td><td>99%</td><td>0.0</td><td>97%</td><td>JQ690289.1</td><td>1104,23</td><td>88,3%</td></tr>
<tr><td>Select seq emb|AJ505543.1|</td><td>Origanum vulgare plastid trnL-trnF intergenic spacer, specimen voucher cult., K-000-69-19317, chase 13334 (K)</td><td>1127</td><td>1127</td><td>99%</td><td>0.0</td><td>97%</td><td>AJ505543.1</td><td>1104,23</td><td>88,3%</td></tr>
<tr><td>Select seq gb|GU381496.1|</td><td>Killickia pilosa voucher M:Braeuchler 3832 tRNA-Leu (trnL) gene and trnL-trnF intergenic spacer, partial sequence; chloroplast</td><td>1125</td><td>1125</td><td>99%</td><td>0.0</td><td>97%</td><td>GU381496.1</td><td>1102,27</td><td>88,1%</td></tr>
<tr><td>Select seq gb|GU381493.1|</td><td>Killickia grandiflora voucher M:Braeuchler 3811 tRNA-Leu (trnL) gene and trnL-trnF intergenic spacer, partial sequence; chloroplast</td><td>1125</td><td>1125</td><td>99%</td><td>0.0</td><td>97%</td><td>GU381493.1</td><td>1102,27</td><td>88,1%</td></tr>
<tr><td>Select seq gb|GU381633.1|</td><td>Thymus caespititius voucher M:Heubl s.n. tRNA-Leu (trnL) gene and trnL-trnF intergenic spacer, partial sequence; chloroplast</td><td>1123</td><td>1123</td><td>99%</td><td>0.0</td><td>97%</td><td>GU381633.1</td><td>1100,31</td><td>88,0%</td></tr>
<tr><td>Select seq gb|GU381485.1|</td><td>Micromeria sphaerophylla voucher E:Lewis et al 1064 tRNA-Leu (trnL) gene and trnL-trnF intergenic spacer, partial sequence; chloroplast</td><td>1123</td><td>1123</td><td>99%</td><td>0.0</td><td>97%</td><td>GU381485.1</td><td>1100,31</td><td>88,0%</td></tr>
<tr><td>Select seq gb|GU381481.1|</td><td>Micromeria cf. madagascariensis Morawetz 205 tRNA-Leu (trnL) gene and trnL-trnF intergenic spacer, partial sequence; chloroplast</td><td>1123</td><td>1123</td><td>99%</td><td>0.0</td><td>97%</td><td>GU381481.1</td><td>1100,31</td><td>88,0%</td></tr>
<tr><td>Select seq gb|KR063657.1|</td><td>Thymus sibthorpii tRNA-Leu (trnL) gene, partial sequence; trnL-trnF intergenic spacer, complete sequence; and tRNA-Phe (trnF) gene, partial sequence; chloroplast</td><td>1122</td><td>1122</td><td>99%</td><td>0.0</td><td>97%</td><td>KR063657.1</td><td>1099,33</td><td>87,9%</td></tr>
<tr><td>Select seq gb|KR150229.1|</td><td>Thymus sp. 5 Kh16 trnL-trnF intergenic spacer region, partial sequence; chloroplast</td><td>1122</td><td>1122</td><td>99%</td><td>0.0</td><td>97%</td><td>KR150229.1</td><td>1099,33</td><td>87,9%</td></tr>
<tr><td>Select seq gb|KR150225.1|</td><td>Thymus sp. 4 Kh24 trnL-trnF intergenic spacer region, partial sequence; chloroplast</td><td>1122</td><td>1122</td><td>99%</td><td>0.0</td><td>97%</td><td>KR150225.1</td><td>1099,33</td><td>87,9%</td></tr>
<tr><td>Select seq gb|KR150218.1|</td><td>Thymus sp. 2 Kh69 trnL-trnF intergenic spacer region, partial sequence; chloroplast</td><td>1122</td><td>1122</td><td>99%</td><td>0.0</td><td>97%</td><td>KR150218.1</td><td>1099,33</td><td>87,9%</td></tr>
<tr><td>Select seq gb|EU556532.1|</td><td>Thymus amurensis isolate HX tRNA-Leu (trnL) gene and trnL-trnF intergenic spacer, partial sequence; chloroplast</td><td>1122</td><td>1122</td><td>99%</td><td>0.0</td><td>97%</td><td>EU556532.1</td><td>1099,33</td><td>87,9%</td></tr>
<tr><td>Select seq gb|EU556525.1|</td><td>Thymus quinquecostatus isolate CL tRNA-Leu (trnL) gene and trnL-trnF intergenic spacer, partial sequence; chloroplast</td><td>1122</td><td>1122</td><td>99%</td><td>0.0</td><td>97%</td><td>EU556525.1</td><td>1099,33</td><td>87,9%</td></tr>
<tr><td>Select seq gb|KR150222.1|</td><td>Thymus sp. 3 Kh65 trnL-trnF intergenic spacer region, partial sequence; chloroplast</td><td>1120</td><td>1120</td><td>99%</td><td>0.0</td><td>97%</td><td>KR150222.1</td><td>1097,37</td><td>87,8%</td></tr>
<tr><td>Select seq gb|GU381631.1|</td><td>Satureja linearifolia voucher M:Brullo & Furnari s.n. tRNA-Leu (trnL) gene and trnL-trnF intergenic spacer, partial sequence; chloroplast</td><td>1120</td><td>1120</td><td>99%</td><td>0.0</td><td>97%</td><td>GU381631.1</td><td>1097,37</td><td>87,8%</td></tr>
<tr><td>Select seq gb|KR150215.1|</td><td>Thymus sp. 1 Kh08 trnL-trnF intergenic spacer region, partial sequence; chloroplast</td><td>1085</td><td>1085</td><td>96%</td><td>0.0</td><td>97%</td><td>KR150215.1</td><td>1096,30</td><td>87,7%</td></tr>
<tr><td>Select seq gb|JQ669069.1|</td><td>Thymus pulegioides voucher Riina 1577 tRNA-Leu (trnL) gene and trnL-trnF intergenic spacer, partial sequence; plastid</td><td>1107</td><td>1107</td><td>98%</td><td>0.0</td><td>97%</td><td>JQ669069.1</td><td>1095,70</td><td>87,6%</td></tr>
<tr><td>Select seq gb|GU381640.1|</td><td>Argantoniella salzmannii voucher M:Barra et al. 2673GL tRNA-Leu (trnL) gene and trnL-trnF intergenic spacer, partial sequence; chloroplast</td><td>1118</td><td>1118</td><td>99%</td><td>0.0</td><td>97%</td><td>GU381640.1</td><td>1095,41</td><td>87,6%</td></tr>
<tr><td>Select seq gb|AY840202.1|</td><td>Origanum vulgare tRNA-Leu (trnL) gene and trnL-trnF intergenic spacer, partial sequence; chloroplast</td><td>1118</td><td>1118</td><td>99%</td><td>0.0</td><td>97%</td><td>AY840202.1</td><td>1095,41</td><td>87,6%</td></tr>
<tr><td>Select seq gb|KR150198.1|</td><td>Ziziphora sp. 3 Kh112 trnL-trnF intergenic spacer region, partial sequence; chloroplast</td><td>1105</td><td>1105</td><td>98%</td><td>0.0</td><td>97%</td><td>KR150198.1</td><td>1093,72</td><td>87,5%</td></tr>
<tr><td>Select seq gb|KR150238.1|</td><td>Ziziphora sp. 1 Kh75 trnL-trnF intergenic spacer region, partial sequence; chloroplast</td><td>1116</td><td>1116</td><td>99%</td><td>0.0</td><td>97%</td><td>KR150238.1</td><td>1093,45</td><td>87,4%</td></tr>
<tr><td>Select seq gb|JQ690293.1|</td><td>Origanum dayi isolate H43_O_day trnL-trnF intergenic spacer, partial sequence; chloroplast</td><td>1116</td><td>1116</td><td>99%</td><td>0.0</td><td>97%</td><td>JQ690293.1</td><td>1093,45</td><td>87,4%</td></tr>
</table>

Select seq gb|GU381628.1|	Thymbra calostachya voucher M:Ulrich s.n. tRNA-Leu (trnL) gene and trnL-trnF intergenic spacer, partial sequence; chloroplast	1116	1116	99%	0.0	97%	GU381628.1	1093,45	87,4%
Select seq gb|EU556539.1|	Thymus quinquecostatus isolate ZY tRNA-Leu (trnL) gene and trnL-trnF intergenic spacer, partial sequence; chloroplast	1116	1116	99%	0.0	97%	EU556539.1	1093,45	87,4%
Select seq gb|EU556538.1|	Thymus quinquecostatus isolate ZJ tRNA-Leu (trnL) gene and trnL-trnF intergenic spacer, partial sequence; chloroplast	1116	1116	99%	0.0	97%	EU556538.1	1093,45	87,4%
Select seq gb|EU556533.1|	Thymus quinquecostatus isolate HY tRNA-Leu (trnL) gene and trnL-trnF intergenic spacer, partial sequence; chloroplast	1116	1116	99%	0.0	97%	EU556533.1	1093,45	87,4%
Select seq gb|EU556527.1|	Thymus mongolicus isolate G2 tRNA-Leu (trnL) gene and trnL-trnF intergenic spacer, partial sequence; chloroplast	1116	1116	99%	0.0	97%	EU556527.1	1093,45	87,4%
Select seq gb|EU556526.1|	Thymus quinquecostatus isolate D2 tRNA-Leu (trnL) gene and trnL-trnF intergenic spacer, partial sequence; chloroplast	1116	1116	99%	0.0	97%	EU556526.1	1093,45	87,4%
Select seq gb|KR150243.1|	Ziziphora sp. 2 Kh20 trnL-trnF intergenic spacer region, partial sequence; chloroplast	1092	1092	97%	0.0	97%	KR150243.1	1092,00	87,3%
Select seq gb|GU381489.1|	Killickia lutea voucher NU<ZAF>:Hilliard & Burtt 9876 tRNA-Leu (trnL) gene and trnL-trnF intergenic spacer, partial sequence; chloroplast	1114	1114	99%	0.0	97%	GU381489.1	1091,49	87,3%
Select seq gb|GU381488.1|	Killickia compacta voucher M:Braeuchler 3816 tRNA-Leu (trnL) gene and trnL-trnF intergenic spacer, partial sequence; chloroplast	1114	1114	99%	0.0	97%	GU381488.1	1091,49	87,3%
Select seq gb|DQ667501.1|	Ziziphora taurica isolate x262 tRNA-Leu (trnL) gene and trnL-trnF intergenic spacer, partial sequence; chloroplast	1114	1114	99%	0.0	97%	DQ667501.1	1091,49	87,3%
Select seq gb|JQ669021.1|	Clinopodium acinos voucher Judziewicz 14160 tRNA-Leu (trnL) gene and trnL-trnF intergenic spacer, partial sequence; plastid	1101	1101	98%	0.0	97%	JQ669021.1	1089,77	87,1%
Select seq gb|GU381635.1|	Thymus broussonetii subsp. hannonis voucher MSB:Podlech 142 tRNA-Leu (trnL) gene and trnL-trnF intergenic spacer, partial sequence; chloroplast	1112	1112	99%	0.0	97%	GU381635.1	1089,54	87,1%
Select seq gb|GU381510.1|	Ziziphora pamiroalaica voucher C:Murray et al. 10090 tRNA-Leu (trnL) gene and trnL-trnF intergenic spacer, partial sequence; chloroplast	1112	1112	99%	0.0	97%	GU381510.1	1089,54	87,1%
Select seq gb|GU381505.1|	Clinopodium troodi voucher W:Davis 1856 tRNA-Leu (trnL) gene and trnL-trnF intergenic spacer, partial sequence; chloroplast	1112	1112	99%	0.0	97%	GU381505.1	1089,54	87,1%
Select seq gb|GU381501.1|	Clinopodium graveolens subsp. rotundifolium voucher M:Podlech 47181 tRNA-Leu (trnL) gene and trnL-trnF intergenic spacer, partial sequence; chloroplast	1112	1112	99%	0.0	97%	GU381501.1	1089,54	87,1%
Select seq gb|GU381499.1|	Clinopodium suaveolens voucher M:Erben s.n. tRNA-Leu (trnL) gene and trnL-trnF intergenic spacer, partial sequence; chloroplast	1112	1112	99%	0.0	97%	GU381499.1	1089,54	87,1%
Select seq gb|AY570463.1|	Origanum vulgare voucher JBW 2567 tRNA-Leu and trnL-trnF intergenic spacer, partial sequence; chloroplast	1112	1112	99%	0.0	97%	AY570463.1	1089,54	87,1%
Select seq gb|GU381500.1|	Clinopodium nanum voucher M:Braeuchler 2796 tRNA-Leu (trnL) gene and trnL-trnF intergenic spacer, partial sequence; chloroplast	1099	1099	98%	0.0	97%	GU381500.1	1087,79	87,0%
Select seq gb|EU556531.1|	Thymus amurensis isolate HW tRNA-Leu (trnL) gene and trnL-trnF intergenic spacer, partial sequence; chloroplast	1110	1110	99%	0.0	97%	EU556531.1	1087,58	87,0%
Select seq gb|EU556536.1|	Thymus marschallianus isolate XY tRNA-Leu (trnL) gene and trnL-trnF intergenic spacer, partial sequence; chloroplast	1098	1098	98%	0.0	97%	EU556536.1	1086,80	86,9%
Select seq gb|EU556530.1|	Thymus mandschuricus isolate HM tRNA-Leu (trnL) gene and trnL-trnF intergenic spacer, partial sequence; chloroplast	1109	1109	99%	0.0	97%	EU556530.1	1086,60	86,9%
Select seq emb|AJ505544.1|	Thymus serpyllum var. citriodorum plastid trnL-trnF intergenic spacer, specimen voucher cult., K-1975-1177, Chase 13331 (K)	1109	1109	99%	0.0	97%	AJ505544.1	1086,60	86,9%
Select seq gb|JF301392.1|	Origanum vulgare voucher B. Drew 77 tRNA-Leu (trnL) gene and trnL-trnF intergenic spacer, partial sequence; chloroplast	1085	1085	97%	0.0	97%	JF301392.1	1085,00	86,8%
Select seq gb|GU381637.1|	Thymus serpyllum voucher M:Braeuchler 2514 tRNA-Leu (trnL) gene and trnL-trnF intergenic spacer, partial sequence; chloroplast	1107	1107	99%	0.0	97%	GU381637.1	1084,64	86,7%
Select seq gb|GU381507.1|	Ziziphora tenuior voucher MSB:Fayvush et al. 03-1503 tRNA-Leu (trnL) gene and trnL-trnF intergenic spacer, partial sequence; chloroplast	1107	1107	99%	0.0	97%	GU381507.1	1084,64	86,7%
Select seq gb|GU381497.1|	Clinopodium acinos voucher M:Podlech 50287 tRNA-Leu (trnL) gene and trnL-trnF intergenic spacer, partial sequence; chloroplast	1107	1107	99%	0.0	97%	GU381497.1	1084,64	86,7%
Select seq gb|AY618513.1|	Mentha arvensis tRNA-Leu (trnL) gene and trnL-trnF intergenic spacer, partial sequence; chloroplast	1094	1094	98%	0.0	97%	AY618513.1	1082,84	86,6%
Select seq gb|GU381634.1|	Thymus pulegioides voucher M:Braeuchler 3129 tRNA-Leu (trnL) gene and trnL-trnF intergenic spacer, partial sequence; chloroplast	1103	1103	99%	0.0	97%	GU381634.1	1080,72	86,4%

Select seq gb|GU381636.1|	Thymus vulgaris voucher M:Braeuchler 3683 tRNA-Leu (trnL) gene and trnL-trnF intergenic spacer, partial sequence; chloroplast	1101	1101	99% 0.0	97%	GU381636.1	1078,76	86,3%
Select seq gb|FJ593456.1|	Mentha sp. JSZ-2009a tRNA-Leu (trnL) gene and trnL-trnF intergenic spacer, partial sequence; chloroplast	1110	1110	99% 0.0	96%	FJ593456.1	1076,36	86,1%
Select seq gb|DQ667514.1|	Mentha arvensis isolate x424 tRNA-Leu (trnL) gene and trnL-trnF intergenic spacer, partial sequence; chloroplast	1103	1103	99% 0.0	96%	DQ667514.1	1069,58	85,5%
Select seq gb|EU556528.1|	Thymus mongolicus isolate G3 tRNA-Leu (trnL) gene and trnL-trnF intergenic spacer, partial sequence; chloroplast	1101	1101	99% 0.0	96%	EU556528.1	1067,64	85,4%
Select seq gb|AY618515.1|	Mentha canadensis tRNA-Leu (trnL) gene and trnL-trnF intergenic spacer, partial sequence; chloroplast	1088	1088	98% 0.0	96%	AY618515.1	1065,80	85,2%
Select seq gb|AY618512.1|	Mentha aquatica tRNA-Leu (trnL) gene and trnL-trnF intergenic spacer, partial sequence; chloroplast	1088	1088	98% 0.0	96%	AY618512.1	1065,80	85,2%
Select seq gb|DQ667492.1|	Lepechinia lancifolia isolate x232 tRNA-Leu (trnL) gene and trnL-trnF intergenic spacer, partial sequence; chloroplast	1098	1098	99% 0.0	96%	DQ667492.1	1064,73	85,1%
Select seq emb|AJ505541.1|	Mentha suaveolens plastid trnL-trnF intergenic spacer, specimen voucher cult., K-1970-3169 (K)	1098	1098	99% 0.0	96%	AJ505541.1	1064,73	85,1%
Select seq gb|GU381613.1|	Satureja pallaryi voucher TUB:Frey & Kuerschner 83-747 tRNA-Leu (trnL) gene and trnL-trnF intergenic spacer, partial sequence; chloroplast	1109	1109	99% 0.0	95%	GU381613.1	1064,19	85,1%
Select seq gb|EU556537.1|	Thymus quinquecostatus isolate XZ tRNA-Leu (trnL) gene and trnL-trnF intergenic spacer, partial sequence; chloroplast	1096	1096	99% 0.0	96%	EU556537.1	1062,79	85,0%
Select seq gb|KC414276.1|	Mentha canadensis isolate 511190001 tRNA-Leu (trnL) gene and trnL-trnF intergenic spacer, partial sequence; chloroplast	1094	1094	99% 0.0	96%	KC414276.1	1060,85	84,8%
Select seq gb|GU381521.1|	Mentha pulegium voucher M:Braeuchler 2300 tRNA-Leu (trnL) gene and trnL-trnF intergenic spacer, partial sequence; chloroplast	1094	1094	99% 0.0	96%	GU381521.1	1060,85	84,8%
Select seq gb|GU381630.1|	Satureja thymbrifolia voucher M:Danin s.n. tRNA-Leu (trnL) gene and trnL-trnF intergenic spacer, partial sequence; chloroplast	1092	1092	99% 0.0	96%	GU381630.1	1058,91	84,7%
Select seq gb|GU381522.1|	Mentha cervina voucher M:Braeuchler 2394 tRNA-Leu (trnL) gene and trnL-trnF intergenic spacer, partial sequence; chloroplast	1092	1092	99% 0.0	96%	GU381522.1	1058,91	84,7%
Select seq gb|EU556529.1|	Thymus dahuricus isolate HD tRNA-Leu (trnL) gene and trnL-trnF intergenic spacer, partial sequence; chloroplast	1092	1092	99% 0.0	96%	EU556529.1	1058,91	84,7%
Select seq gb|AY570461.1|	Mentha spicata voucher JBW 2566 tRNA-Leu and trnL-trnF intergenic spacer, partial sequence; chloroplast	1086	1086	99% 0.0	96%	AY570461.1	1053,09	84,2%
							0,00	0,0%

Select for downloading or viewing reports	Kh095_ITS Description	Max score	Total score	Query cover	E value	Ident	Accession	(Ident/Cover)* Max score	Deviation from top hit
Select seq gb|EF577323.1|	Tanacetum vulgare 18S ribosomal RNA gene, partial sequence; internal transcribed spacer 1, 5.8S ribosomal RNA gene, and internal transcribed spacer 2, complete sequence; and 28S ribosomal RNA gene, partial sequence	1155	1155	93%	0.0	97%	EF577323.1	1204,68	100,0%
Select seq gb|EF577320.1|	Tanacetum parthenium 18S ribosomal RNA gene, partial sequence; internal transcribed spacer 1, 5.8S ribosomal RNA gene, and internal transcribed spacer 2, complete sequence; and 28S ribosomal RNA gene, partial sequence	1149	1149	93%	0.0	97%	EF577320.1	1198,42	99,5%
Select seq gb|EF577322.1|	Tanacetum ptarmiciflorum 18S ribosomal RNA gene, partial sequence; internal transcribed spacer 1, 5.8S ribosomal RNA gene, and internal transcribed spacer 2, complete sequence; and 28S ribosomal RNA gene, partial sequence	1144	1144	93%	0.0	97%	EF577322.1	1193,20	99,0%
Select seq gb|AY603264.1|	Tanacetum vulgare internal transcribed spacer 1, 5.8S ribosomal RNA gene, and internal transcribed spacer 2, complete sequence	1031	1031	84%	0.0	97%	AY603264.1	1190,56	98,8%
Select seq dbj|AB683268.1|	Tanacetum aucherianum gene for ITS1, 5.8S rRNA and ITS2, complete sequence, specimen_voucher: 44441 (HUB)	1029	1029	84%	0.0	97%	AB683268.1	1188,25	98,6%
Select seq dbj|AB683265.1|	Tanacetum argenteum subsp. canum gene for ITS1, 5.8S rRNA and ITS2, complete sequence, specimen_voucher: 46856 (B)	1029	1029	84%	0.0	97%	AB683265.1	1188,25	98,6%
Select seq dbj|AB683270.1|	Tanacetum balsamita gene for ITS1, 5.8S rRNA and ITS2, complete sequence, specimen_voucher: 1126 (MPH)	1029	1029	84%	0.0	96%	AB683270.1	1176,00	97,6%
Select seq dbj|AB683287.1|	Tanacetum haradjanii gene for ITS1, 5.8S rRNA and ITS2, complete sequence, specimen_voucher: 16366 (M)	1026	1026	84%	0.0	96%	AB683287.1	1172,57	97,3%
Select seq dbj|AB683260.1|	Tanacetum abrotanifolium gene for ITS1, 5.8S rRNA and ITS2, complete sequence, specimen_voucher: 68865 (TARI)	1026	1026	84%	0.0	96%	AB683260.1	1172,57	97,3%
Select seq emb|FN823079.1|	Tanacetum chiliophyllum genomic DNA containing ITS1, 5.8S rRNA gene, ITS2, specimen voucher MPH:1124	1026	1026	84%	0.0	96%	FN823079.1	1172,57	97,3%
Select seq dbj|AB683278.1|	Tanacetum demetrii gene for ITS1, 5.8S rRNA and ITS2, complete sequence, specimen_voucher: (W, 2001-10597)	1022	1022	84%	0.0	96%	AB683278.1	1168,00	97,0%
Select seq dbj|AB608336.1|	Tanacetum polycephalum genes for ITS1, 5.8S rRNA, ITS2, complete sequence	1022	1022	84%	0.0	96%	AB608336.1	1168,00	97,0%
Select seq dbj|AB683271.1|	Tanacetum bamianicum gene for ITS1, 5.8S rRNA and ITS2, complete sequence, specimen_voucher: 18238 (B)	1020	1020	84%	0.0	96%	AB683271.1	1165,71	96,8%
Select seq emb|FN823080.1|	Tanacetum parthenium genomic DNA containing ITS1, 5.8S rRNA gene, ITS2, specimen voucher MPH:1119	1020	1020	84%	0.0	96%	FN823080.1	1165,71	96,8%
Select seq dbj|AB683282.1|	Tanacetum elbursense gene for ITS1, 5.8S rRNA and ITS2, complete sequence, specimen_voucher: 1109 (MPH)	1018	1018	84%	0.0	96%	AB683282.1	1163,43	96,6%
Select seq dbj|AB608331.1|	Tanacetum canescens genes for ITS1, 5.8S rRNA, ITS2, complete sequence	1018	1018	84%	0.0	96%	AB608331.1	1163,43	96,6%
Select seq dbj|AB683308.1|	Tanacetum polycephalum subsp. farsicum gene for ITS1, 5.8S rRNA and ITS2, complete sequence, specimen_voucher: 1167 (MPH)	1014	1014	84%	0.0	96%	AB683308.1	1158,86	96,2%
Select seq dbj|AB683303.1|	Tanacetum parthenifolium gene for ITS1, 5.8S rRNA and ITS2, complete sequence, specimen_voucher: 1127 (MPH)	1014	1014	84%	0.0	96%	AB683303.1	1158,86	96,2%
Select seq dbj|AB683285.1|	Tanacetum germanicopolitanum gene for ITS1, 5.8S rRNA and ITS2, complete sequence, specimen_voucher: 14254 (S)	1014	1014	84%	0.0	96%	AB683285.1	1158,86	96,2%
Select seq dbj|AB608337.1|	Tanacetum tabrisianum genes for ITS1, 5.8S rRNA, ITS2, complete sequence	1014	1014	84%	0.0	96%	AB608337.1	1158,86	96,2%
Select seq dbj|AB683323.1|	Tanacetum sonbolii gene for ITS1, 5.8S rRNA and ITS2, complete sequence, specimen_voucher: 998 (MPH)	1013	1013	84%	0.0	96%	AB683323.1	1157,71	96,1%
Select seq dbj|AB683267.1|	Tanacetum armenum gene for ITS1, 5.8S rRNA and ITS2, complete sequence, specimen_voucher: (W, 1972-03416)	1013	1013	84%	0.0	96%	AB683267.1	1157,71	96,1%
Select seq dbj|AB608332.1|	Tanacetum chiliophyllum genes for ITS1, 5.8S rRNA, ITS2, complete sequence	1011	1011	84%	0.0	96%	AB608332.1	1155,43	95,9%
Select seq dbj|AB683304.1|	Tanacetum pinnatum gene for ITS1, 5.8S rRNA and ITS2, complete sequence, specimen_voucher: 1118 (MPH)	1009	1009	84%	0.0	96%	AB683304.1	1153,14	95,7%
Select seq dbj|AB683289.1|	Tanacetum hololeucum gene for ITS1, 5.8S rRNA and ITS2, complete sequence, specimen_voucher: 6472 (S)	1009	1009	84%	0.0	96%	AB683289.1	1153,14	95,7%
Select seq dbj|AB683269.1|	Tanacetum bachtiaricum gene for ITS1, 5.8S rRNA and ITS2, complete sequence, specimen_voucher: 1174 (MPH)	1009	1009	84%	0.0	96%	AB683269.1	1153,14	95,7%
Select seq gb|AY603263.1|	Tanacetum millefolium internal transcribed spacer 1, 5.8S ribosomal RNA gene, and internal transcribed spacer 2, complete sequence	1009	1009	84%	0.0	96%	AY603263.1	1153,14	95,7%

Select seq dbj|AB683290.1|	Tanacetum khorassanicum gene for ITS1, 5.8S rRNA and ITS2, complete sequence, specimen_voucher: 4937 (S)	1007	1007	84%	0.0	96%	AB683290.1	1150,86	95,5%
Select seq emb|FN823081.1|	Tanacetum coccineum genomic DNA containing ITS1, 5.8S rRNA gene, ITS2, specimen voucher MPH:1144	1007	1007	84%	0.0	96%	FN823081.1	1150,86	95,5%
Select seq dbj|AB683305.1|	Tanacetum polycephalum subsp. argyrophyllum gene for ITS1, 5.8S rRNA and ITS2, complete sequence, specimen_voucher: 1125 (MPH)	1005	1005	84%	0.0	96%	AB683305.1	1148,57	95,3%
Select seq dbj|AB683281.1|	Tanacetum dumosum gene for ITS1, 5.8S rRNA and ITS2, complete sequence, specimen_voucher: 1168 (MPH)	1005	1005	84%	0.0	96%	AB683281.1	1148,57	95,3%
Select seq dbj|AB683313.1|	Tanacetum punctatum gene for ITS1, 5.8S rRNA and ITS2, complete sequence, specimen_voucher: 49833 (IRAN)	1003	1003	84%	0.0	96%	AB683313.1	1146,29	95,2%
Select seq dbj|AB683288.1|	Tanacetum haussknechtii gene for ITS1, 5.8S rRNA and ITS2, complete sequence, specimen_voucher: 1193 (HUB)	1003	1003	84%	0.0	96%	AB683288.1	1146,29	95,2%
Select seq dbj|AB683301.1|	Tanacetum nitens gene for ITS1, 5.8S rRNA and ITS2, complete sequence, specimen_voucher: (W, 1977-16564)	1002	1002	84%	0.0	96%	AB683301.1	1145,14	95,1%
Select seq dbj|AB683286.1|	Tanacetum griffithii gene for ITS1, 5.8S rRNA and ITS2, complete sequence, specimen_voucher: 37485 (B)	1002	1002	84%	0.0	96%	AB683286.1	1145,14	95,1%
Select seq dbj|AB683266.1|	Tanacetum argenteum subsp. flabellifolium gene for ITS1, 5.8S rRNA and ITS2, complete sequence, specimen_voucher: 1976 (HUB)	1002	1002	84%	0.0	96%	AB683266.1	1145,14	95,1%
Select seq dbj|AB683302.1|	Tanacetum paleaceum gene for ITS1, 5.8S rRNA and ITS2, complete sequence, specimen_voucher: 5828 (B)	998	998	84%	0.0	96%	AB683302.1	1140,57	94,7%
Select seq dbj|AB608333.1|	Tanacetum coccineum genes for ITS1, 5.8S rRNA, ITS2, complete sequence	998	998	84%	0.0	96%	AB608333.1	1140,57	94,7%
Select seq gb|FJ553518.1|	Angiosperm environmental sample clone LTSP_EUKA_P3K07 18S ribosomal RNA, 18S-25/28S ribosomal RNA intergenic spacer, partial sequence	1184	1184	100%	0.0	96%	FJ553518.1	1136,64	94,4%
Select seq gb|EF577285.1|	Achillea wilsoniana 18S ribosomal RNA gene, partial sequence; internal transcribed spacer 1, 5.8S ribosomal RNA gene, and internal transcribed spacer 2, complete sequence; and 28S ribosomal RNA gene, partial sequence	1101	1101	93%	0.0	96%	EF577285.1	1136,52	94,3%
Select seq gb|AY603229.1|	Achillea clavennae internal transcribed spacer 1, 5.8S ribosomal RNA gene, and internal transcribed spacer 2, complete sequence	994	994	84%	0.0	96%	AY603229.1	1136,00	94,3%
Select seq gb|AY603212.1|	Achillea nobilis internal transcribed spacer 1, 5.8S ribosomal RNA gene, and internal transcribed spacer 2, complete sequence	994	994	84%	0.0	96%	AY603212.1	1136,00	94,3%
Select seq dbj|AB683327.1|	Tanacetum turcomanicum gene for ITS1, 5.8S rRNA and ITS2, complete sequence, specimen_voucher: 20867 (FUMH)	998	998	84%	0.0	95%	AB683327.1	1128,69	93,7%
Select seq dbj|AB683316.1|	Tanacetum salsugineum gene for ITS1, 5.8S rRNA and ITS2, complete sequence, specimen_voucher: 1407 (MPH)	996	996	84%	0.0	95%	AB683316.1	1126,43	93,5%
Select seq dbj|AB683274.1|	Tanacetum budjnurdense gene for ITS1, 5.8S rRNA and ITS2, complete sequence, specimen_voucher: 38119 (FUMH)	996	996	84%	0.0	95%	AB683274.1	1126,43	93,5%
Select seq dbj|AB608330.1|	Tanacetum budjnurdense genes for ITS1, 5.8S rRNA, ITS2, complete sequence	994	994	84%	0.0	95%	AB608330.1	1124,17	93,3%
Select seq dbj|AB683339.1|	Xylanthemum paghmanense gene for ITS1, 5.8S rRNA and ITS2, complete sequence, specimen_voucher: (W-1986-05509)	992	992	84%	0.0	95%	AB683339.1	1121,90	93,1%
Select seq dbj|AB683336.1|	Tanacetum zahlbruckneri gene for ITS1, 5.8S rRNA and ITS2, complete sequence, specimen_voucher: 602 (MPH)	992	992	84%	0.0	95%	AB683336.1	1121,90	93,1%
Select seq gb|EU796891.1|	Achillea millefolium 18S ribosomal RNA gene, partial sequence; internal transcribed spacer 1, 5.8S ribosomal RNA gene, and internal transcribed spacer 2, complete sequence; and 26S ribosomal RNA gene, partial sequence	1062	1062	90%	0.0	95%	EU796891.1	1121,00	93,1%
Select seq gb|AY603230.1|	Achillea clavennae internal transcribed spacer 1, 5.8S ribosomal RNA gene, and internal transcribed spacer 2, complete sequence	990	990	84%	0.0	95%	AY603230.1	1119,64	92,9%
Select seq dbj|AB523747.1|	Tanacetum tenuisectum genes for ITS1, 5.8S rRNA, ITS2, complete sequence, specimen_voucher: MPH:1087	989	989	84%	0.0	95%	AB523747.1	1118,51	92,8%
Select seq gb|AY603211.1|	Achillea virescens internal transcribed spacer 1, 5.8S ribosomal RNA gene, and internal transcribed spacer 2, complete sequence	989	989	84%	0.0	95%	AY603211.1	1118,51	92,8%
Select seq gb|AY603202.1|	Achillea euxina internal transcribed spacer 1, 5.8S ribosomal RNA gene, and internal transcribed spacer 2, complete sequence	989	989	84%	0.0	95%	AY603202.1	1118,51	92,8%
Select seq gb|AY603191.1|	Achillea setacea internal transcribed spacer 1, 5.8S ribosomal RNA gene, and internal transcribed spacer 2, complete sequence	989	989	84%	0.0	95%	AY603191.1	1118,51	92,8%
Select seq gb|AY603187.1|	Achillea millefolium subsp. sudetica internal transcribed spacer 1, 5.8S ribosomal RNA gene, and internal transcribed spacer 2, complete sequence	989	989	84%	0.0	95%	AY603187.1	1118,51	92,8%

Select seq gb|AY603205.1|	Achillea lanulosa subsp. alpicola internal transcribed spacer 1, 5.8S ribosomal RNA gene, and internal transcribed spacer 2, complete sequence	987	987	84% 0.0	95% AY603205.1	1116,25	92,7%
Select seq gb|AY603190.1|	Achillea styriaca internal transcribed spacer 1, 5.8S ribosomal RNA gene, and internal transcribed spacer 2, complete sequence	985	985	84% 0.0	95% AY603190.1	1113,99	92,5%
Select seq gb|AY603186.1|	Achillea millefolium internal transcribed spacer 1, 5.8S ribosomal RNA gene, and internal transcribed spacer 2, complete sequence	985	985	84% 0.0	95% AY603186.1	1113,99	92,5%
Select seq gb|AY603240.1|	Achillea schurii internal transcribed spacer 1, 5.8S ribosomal RNA gene, and internal transcribed spacer 2, complete sequence	983	983	84% 0.0	95% AY603240.1	1111,73	92,3%
Select seq gb|AY603197.1|	Achillea setacea internal transcribed spacer 1, 5.8S ribosomal RNA gene, and internal transcribed spacer 2, complete sequence	983	983	84% 0.0	95% AY603197.1	1111,73	92,3%
Select seq gb|AY603185.1|	Achillea millefolium internal transcribed spacer 1, 5.8S ribosomal RNA gene, and internal transcribed spacer 2, complete sequence	983	983	84% 0.0	95% AY603185.1	1111,73	92,3%
Select seq gb|AY603184.1|	Achillea pannonica internal transcribed spacer 1, 5.8S ribosomal RNA gene, and internal transcribed spacer 2, complete sequence	983	983	84% 0.0	95% AY603184.1	1111,73	92,3%
Select seq dbj|AB523751.1|	Tanacetum porphyrostephanum genes for ITS1, 5.8S rRNA, ITS2, complete sequence, specimen_voucher: W:1986-05841	981	981	84% 0.0	95% AB523751.1	1109,46	92,1%
Select seq dbj|AB523746.1|	Tanacetum joharchii genes for ITS1, 5.8S rRNA, ITS2, complete sequence, specimen_voucher: FUMH:29141	981	981	84% 0.0	95% AB523746.1	1109,46	92,1%
Select seq gb|AY603262.1|	Tanacetum macrophyllum internal transcribed spacer 1, 5.8S ribosomal RNA gene, and internal transcribed spacer 2, complete sequence	981	981	84% 0.0	95% AY603262.1	1109,46	92,1%
Select seq gb|JQ230971.1|	Achillea millefolium voucher SBB-1156 18S ribosomal RNA gene, partial sequence; internal transcribed spacer 1, 5.8S ribosomal RNA gene, and internal transcribed spacer 2, complete sequence; and 28S ribosomal RNA gene, partial sequence	1086	1086	93% 0.0	95% JQ230971.1	1109,35	92,1%
Select seq gb|EU179216.1|	Anthemis cotula 18S ribosomal RNA gene, partial sequence; internal transcribed spacer 1, 5.8S ribosomal RNA gene, and internal transcribed spacer 2, complete sequence; and 26S ribosomal RNA gene, partial sequence	1038	1038	89% 0.0	95% EU179216.1	1107,98	92,0%
Select seq gb|AY603204.1|	Achillea ceretanica internal transcribed spacer 1, 5.8S ribosomal RNA gene, and internal transcribed spacer 2, complete sequence	979	979	84% 0.0	95% AY603204.1	1107,20	91,9%
Select seq gb|AY603200.1|	Achillea roseoalba internal transcribed spacer 1, 5.8S ribosomal RNA gene, and internal transcribed spacer 2, complete sequence	979	979	84% 0.0	95% AY603200.1	1107,20	91,9%
Select seq gb|FJ980357.1|	Achillea wilsoniana voucher PS0715MT01 18S ribosomal RNA gene, partial sequence; internal transcribed spacer 1, 5.8S ribosomal RNA gene, and internal transcribed spacer 2, complete sequence; and 28S ribosomal RNA gene, partial sequence	1105	1105	95% 0.0	95% FJ980357.1	1105,00	91,7%
Select seq dbj|AB683340.1|	Xylanthemum pamiricum gene for ITS1, 5.8S rRNA and ITS2, complete sequence, specimen_voucher: 34987-E (IRAN)	977	977	84% 0.0	95% AB683340.1	1104,94	91,7%
Select seq gb|AY603241.1|	Achillea schurii internal transcribed spacer 1, 5.8S ribosomal RNA gene, and internal transcribed spacer 2, complete sequence	977	977	84% 0.0	95% AY603241.1	1104,94	91,7%
Select seq gb|AY603223.1|	Achillea micrantha internal transcribed spacer 1, 5.8S ribosomal RNA gene, and internal transcribed spacer 2, complete sequence	977	977	84% 0.0	95% AY603223.1	1104,94	91,7%
Select seq gb|AY603222.1|	Achillea holosericea internal transcribed spacer 1, 5.8S ribosomal RNA gene, and internal transcribed spacer 2, complete sequence	977	977	84% 0.0	95% AY603222.1	1104,94	91,7%
Select seq gb|AY603216.1|	Achillea clypeolata internal transcribed spacer 1, 5.8S ribosomal RNA gene, and internal transcribed spacer 2, complete sequence	977	977	84% 0.0	95% AY603216.1	1104,94	91,7%
Select seq gb|AY603213.1|	Achillea absinthoides internal transcribed spacer 1, 5.8S ribosomal RNA gene, and internal transcribed spacer 2, complete sequence	977	977	84% 0.0	95% AY603213.1	1104,94	91,7%
Select seq gb|AY603207.1|	Achillea wilsoniana internal transcribed spacer 1, 5.8S ribosomal RNA gene, and internal transcribed spacer 2, complete sequence	977	977	84% 0.0	95% AY603207.1	1104,94	91,7%
Select seq gb|AY603228.1|	Achillea clusiana internal transcribed spacer 1, 5.8S ribosomal RNA gene, and internal transcribed spacer 2, complete sequence	976	976	84% 0.0	95% AY603228.1	1103,81	91,6%
Select seq gb|AY603227.1|	Achillea atrata internal transcribed spacer 1, 5.8S ribosomal RNA gene, and internal transcribed spacer 2, complete sequence	976	976	84% 0.0	95% AY603227.1	1103,81	91,6%
Select seq gb|AY603210.1|	Achillea monticola internal transcribed spacer 1, 5.8S ribosomal RNA gene, and internal transcribed spacer 2, complete sequence	976	976	84% 0.0	95% AY603210.1	1103,81	91,6%
Select seq gb|FJ980358.1|	Achillea wilsoniana voucher PS0715MT02 18S ribosomal RNA gene, partial sequence; internal transcribed spacer 1, 5.8S ribosomal RNA gene, and internal transcribed spacer 2, complete sequence; and 28S ribosomal RNA gene, partial sequence	1101	1101	95% 0.0	95% FJ980358.1	1101,00	91,4%
Select seq gb|EU179214.1|	Anthemis arvensis 18S ribosomal RNA gene, partial sequence; internal transcribed spacer 1, 5.8S ribosomal RNA gene, and internal transcribed spacer 2, complete sequence; and 26S ribosomal RNA gene, partial sequence	1029	1029	89% 0.0	95% EU179214.1	1098,37	91,2%
Select seq gb|EU179212.1|	Matricaria recutita 18S ribosomal RNA gene, partial sequence; internal transcribed spacer 1, 5.8S ribosomal RNA gene, and internal transcribed spacer 2, complete sequence; and 26S ribosomal RNA gene, partial sequence	1026	1026	89% 0.0	95% EU179212.1	1095,17	90,9%

Select seq gb|KR150147.1|	Achillea sp. Kh58 internal transcribed spacer 1, partial sequence; 5.8S ribosomal RNA gene gene, complete sequence; and internal transcribed spacer 2 region, partial sequence	996	996	87%	0.0	95% KR150147.1	1087,59	90,3%
Select seq gb|KC855670.1|	Achillea multifida isolate ACMt5 18S ribosomal RNA gene, partial sequence; internal transcribed spacer 1, 5.8S ribosomal RNA gene, and internal transcribed spacer 2, complete sequence; and 28S ribosomal RNA gene, partial sequence	979	979	86%	0.0	94% KC855670.1	1070,07	88,8%
Select seq gb|JF907423.1|	Tripleurospermum maritimum subsp. inodorum voucher OSBU:2005 DE-0-OSN-2005-169 18S ribosomal RNA gene, partial sequence; internal transcribed spacer 1, 5.8S ribosomal RNA gene, and internal transcribed spacer 2, complete sequence; and 26S ribosomal RNA gene, partial sequence	1033	1033	91%	0.0	94% JF907423.1	1067,05	88,6%
Select seq gb|KC855671.1|	Achillea multifida isolate ACMt6 18S ribosomal RNA gene, partial sequence; internal transcribed spacer 1, 5.8S ribosomal RNA gene, and internal transcribed spacer 2, complete sequence; and 28S ribosomal RNA gene, partial sequence	981	981	87%	0.0	94% KC855671.1	1059,93	88,0%
Select seq gb|KC816562.1|	Matricaria chamomilla var. recutita 18S ribosomal RNA gene, partial sequence; internal transcribed spacer 1, 5.8S ribosomal RNA gene, and internal transcribed spacer 2, complete sequence; and 28S ribosomal RNA gene, partial sequence	1048	1048	93%	0.0	94% KC816562.1	1059,27	87,9%
Select seq gb|KC816561.1|	Achillea santolina 18S ribosomal RNA gene, partial sequence; internal transcribed spacer 1, 5.8S ribosomal RNA gene, and internal transcribed spacer 2, complete sequence; and 28S ribosomal RNA gene, partial sequence	1048	1048	93%	0.0	94% KC816561.1	1059,27	87,9%
Select seq gb|KM887397.1|	Anacyclus pyrethrum isolate SBB-1407 18S ribosomal RNA gene, partial sequence; internal transcribed spacer 1, 5.8S ribosomal RNA gene, and internal transcribed spacer 2, complete sequence; and 28S ribosomal RNA gene, partial sequence	1055	1055	94%	0.0	94% KM887397.1	1055,00	87,6%
Select seq gb|EU179213.1|	Matricaria matricarioides 18S ribosomal RNA gene, partial sequence; internal transcribed spacer 1, 5.8S ribosomal RNA gene, and internal transcribed spacer 2, complete sequence; and 26S ribosomal RNA gene, partial sequence	996	996	89%	0.0	94% EU179213.1	1051,96	87,3%
Select seq gb|KM887396.1|	Anacyclus pyrethrum isolate SBB-1364 18S ribosomal RNA gene, partial sequence; internal transcribed spacer 1, 5.8S ribosomal RNA gene, and internal transcribed spacer 2, complete sequence; and 28S ribosomal RNA gene, partial sequence	1035	1035	93%	0.0	94% KM887396.1	1046,13	86,8%
Select seq gb|EF577319.1|	Tanacetum cinerariifolium 18S ribosomal RNA gene, partial sequence; internal transcribed spacer 1, 5.8S ribosomal RNA gene, and internal transcribed spacer 2, complete sequence; and 28S ribosomal RNA gene, partial sequence	1031	1031	93%	0.0	94% EF577319.1	1042,09	86,5%
Select seq gb|KR150185.1|	Achillea sp. Kh17 internal transcribed spacer 1, partial sequence; 5.8S ribosomal RNA gene gene, complete sequence; and internal transcribed spacer 2 region, partial sequence	996	996	90%	0.0	94% KR150185.1	1040,27	86,4%
Select seq gb|GU818490.1|	Anacyclus valentinus isolate L1186 18S ribosomal RNA gene, partial sequence; internal transcribed spacer 1, 5.8S ribosomal RNA gene, and internal transcribed spacer 2, complete sequence; and 25S ribosomal RNA gene, partial sequence	1013	1013	94%	0.0	93% GU818490.1	1002,22	83,2%
Select seq gb|EF577292.1|	Chrysanthemum coronarium 18S ribosomal RNA gene, partial sequence; internal transcribed spacer 1, 5.8S ribosomal RNA gene, and internal transcribed spacer 2, complete sequence; and 28S ribosomal RNA gene, partial sequence	992	992	93%	0.0	93% EF577292.1	992,00	82,3%
Select seq gb|EF577287.1|	Argyranthemum frutescens 18S ribosomal RNA gene, partial sequence; internal transcribed spacer 1, 5.8S ribosomal RNA gene, and internal transcribed spacer 2, complete sequence; and 28S ribosomal RNA gene, partial sequence	992	992	93%	0.0	93% EF577287.1	992,00	82,3%
Select seq gb|GQ396673.1|	Artemisia argyi voucher SCMR9412304 18S ribosomal RNA gene, partial sequence; internal transcribed spacer 1, 5.8S ribosomal RNA gene, and internal transcribed spacer 2, complete sequence; and 28S ribosomal RNA gene, partial sequence	979	979	100%	0.0	91% GQ396673.1	890,89	74,0%
Select seq gb|KP856179.1|	Artemisia sp. Rst2 18S ribosomal RNA gene, partial sequence; internal transcribed spacer 1, 5.8S ribosomal RNA gene, and internal transcribed spacer 2, complete sequence; and 28S ribosomal RNA gene, partial sequence	979	979	100%	0.0	90% KP856179.1	881,10	73,1%

Select for downloading or viewing reports	Kh095_trnL Description	Max score	Total score	Query cover	E value	Ident	Accession	(Ident/Cover)* Max score	Deviation from top hit
Select seq gb|JX073760.1|	Artemisia lagocephala tRNA-Leu (trnL) gene and trnL-trnF intergenic spacer, partial sequence; chloroplast	1098	1098	73%	0.0	98%	JX073760.1	1474,03	100,0%
Select seq gb|JX073787.1|	Artemisia ludoviciana tRNA-Leu (trnL) gene and trnL-trnF intergenic spacer, partial sequence; chloroplast	1086	1086	73%	0.0	98%	JX073787.1	1457,92	98,9%
Select seq gb|JX073755.1|	Artemisia cana tRNA-Leu (trnL) gene and trnL-trnF intergenic spacer, partial sequence; chloroplast	1086	1086	73%	0.0	98%	JX073755.1	1457,92	98,9%
Select seq gb|JX073789.1|	Artemisia bigelovii tRNA-Leu (trnL) gene and trnL-trnF intergenic spacer, partial sequence; chloroplast	1068	1068	72%	0.0	98%	JX073789.1	1453,67	98,6%
Select seq gb|JX073815.1|	Artemisia afra tRNA-Leu (trnL) gene and trnL-trnF intergenic spacer, partial sequence; chloroplast	1079	1079	73%	0.0	98%	JX073815.1	1448,52	98,3%
Select seq gb|JX073796.1|	Artemisia scoparia tRNA-Leu (trnL) gene and trnL-trnF intergenic spacer, partial sequence; chloroplast	1079	1079	73%	0.0	98%	JX073796.1	1448,52	98,3%
Select seq gb|JX073792.1|	Artemisia japonica tRNA-Leu (trnL) gene and trnL-trnF intergenic spacer, partial sequence; chloroplast	1079	1079	73%	0.0	98%	JX073792.1	1448,52	98,3%
Select seq gb|JX073790.1|	Artemisia pycnocephala tRNA-Leu (trnL) gene and trnL-trnF intergenic spacer, partial sequence; chloroplast	1079	1079	73%	0.0	98%	JX073790.1	1448,52	98,3%
Select seq gb|JX073783.1|	Artemisia schmidtiana tRNA-Leu (trnL) gene and trnL-trnF intergenic spacer, partial sequence; chloroplast	1079	1079	73%	0.0	98%	JX073783.1	1448,52	98,3%
Select seq gb|JX073777.1|	Artemisia armeniaca tRNA-Leu (trnL) gene and trnL-trnF intergenic spacer, partial sequence; chloroplast	1079	1079	73%	0.0	98%	JX073777.1	1448,52	98,3%
Select seq gb|JX073747.1|	Artemisia nitrosa tRNA-Leu (trnL) gene and trnL-trnF intergenic spacer, partial sequence; chloroplast	1079	1079	73%	0.0	98%	JX073747.1	1448,52	98,3%
Select seq gb|JX073749.1|	Artemisia gmelinii subsp. scheludjakoviae tRNA-Leu (trnL) gene and trnL-trnF intergenic spacer, partial sequence; chloroplast	1079	1079	73%	0.0	98%	JX073749.1	1448,52	98,3%
Select seq gb|FJ842617.1|	Artemisia fukudo voucher MPS001124 (KMH) tRNA-Leu (trnL) gene and trnL-trnF intergenic spacer, partial sequence; chloroplast	1079	1079	73%	0.0	98%	FJ842617.1	1448,52	98,3%
Select seq gb|FJ842615.1|	Artemisia capillaris voucher MPS001196 (KMH) tRNA-Leu (trnL) gene and trnL-trnF intergenic spacer, partial sequence; chloroplast	1079	1079	73%	0.0	98%	FJ842615.1	1448,52	98,3%
Select seq gb|JX073753.1|	Artemisia arbuscula tRNA-Leu (trnL) gene and trnL-trnF intergenic spacer, partial sequence; chloroplast	1074	1074	73%	0.0	98%	JX073753.1	1441,81	97,8%
Select seq gb|JX073798.1|	Artemisia rigida tRNA-Leu (trnL) gene and trnL-trnF intergenic spacer, partial sequence; chloroplast	1074	1074	73%	0.0	98%	JX073798.1	1441,81	97,8%
Select seq gb|JX073786.1|	Artemisia suksdorfii tRNA-Leu (trnL) gene and trnL-trnF intergenic spacer, partial sequence; chloroplast	1074	1074	73%	0.0	98%	JX073786.1	1441,81	97,8%
Select seq gb|JX073785.1|	Artemisia serrata tRNA-Leu (trnL) gene and trnL-trnF intergenic spacer, partial sequence; chloroplast	1074	1074	73%	0.0	98%	JX073785.1	1441,81	97,8%
Select seq gb|JX073779.1|	Artemisia alba tRNA-Leu (trnL) gene and trnL-trnF intergenic spacer, partial sequence; chloroplast	1074	1074	73%	0.0	98%	JX073779.1	1441,81	97,8%
Select seq gb|FJ842621.1|	Artemisia keiskeana voucher MPS001234 (KMH) tRNA-Leu (trnL) gene and trnL-trnF intergenic spacer, partial sequence; chloroplast	1074	1074	73%	0.0	98%	FJ842621.1	1441,81	97,8%
Select seq gb|FJ842619.1|	Artemisia japonica voucher MPS000177 (KMH) tRNA-Leu (trnL) gene and trnL-trnF intergenic spacer, partial sequence; chloroplast	1074	1074	73%	0.0	98%	FJ842619.1	1441,81	97,8%
Select seq gb|FJ842618.1|	Artemisia gmelinii voucher MPS001216 (KMH) tRNA-Leu (trnL) gene and trnL-trnF intergenic spacer, partial sequence; chloroplast	1074	1074	73%	0.0	98%	FJ842618.1	1441,81	97,8%
Select seq gb|FJ710526.1|	Artemisia absinthium tRNA-Leu (trnL) gene and trnL-trnF intergenic spacer, partial sequence; chloroplast	1074	1074	73%	0.0	98%	FJ710526.1	1441,81	97,8%
Select seq gb|FJ692341.1|	Artemisia annua voucher MPS 001275 (KMH) tRNA-Leu (trnL) gene and trnL-trnF intergenic spacer, partial sequence; chloroplast	1074	1074	73%	0.0	98%	FJ692341.1	1441,81	97,8%
Select seq gb|JX073758.1|	Artemisia pedatifida tRNA-Leu (trnL) gene and trnL-trnF intergenic spacer, partial sequence; chloroplast	1072	1072	73%	0.0	98%	JX073758.1	1439,12	97,6%
Select seq gb|KF736807.1|	Artemisia turcomanica tRNA-Leu (trnL) gene and trnL-trnF intergenic spacer, partial sequence; chloroplast	1098	1098	75%	0.0	98%	KF736807.1	1434,72	97,3%

Select seq gb|KF736803.1|	Artemisia fragrans tRNA-Leu (trnL) gene and trnL-trnF intergenic spacer, partial sequence; chloroplast	1098	1098	75% 0.0	98% KF736803.1	1434,72	97,3%
Select seq gb|KF736802.1|	Artemisia incana tRNA-Leu (trnL) gene and trnL-trnF intergenic spacer, partial sequence; chloroplast	1098	1098	75% 0.0	98% KF736802.1	1434,72	97,3%
Select seq gb|KF736826.1|	Artemisia aucheri tRNA-Leu (trnL) gene and trnL-trnF intergenic spacer, partial sequence; chloroplast	1094	1094	75% 0.0	98% KF736826.1	1429,49	97,0%
Select seq gb|KF736805.1|	Artemisia spicigera tRNA-Leu (trnL) gene and trnL-trnF intergenic spacer, partial sequence; chloroplast	1092	1092	75% 0.0	98% KF736805.1	1426,88	96,8%
Select seq gb|KF669899.1|	Artemisia absinthium tRNA-Leu (trnL) gene and trnL-trnF intergenic spacer, partial sequence; chloroplast	1092	1092	75% 0.0	98% KF669899.1	1426,88	96,8%
Select seq dbj|AB219593.1|	Artemisia sinanensis chloroplast trnL(UAA), trnF(GAA) genes for tRNA-Leu, tRNA-Phe, partial sequence, trnL-trnF spacer region, specimen_voucher: MAK:N. Fujii F01782	1092	1092	75% 0.0	98% AB219593.1	1426,88	96,8%
Select seq gb|JX073805.1|	Artemisia gracilescens tRNA-Leu (trnL) gene and trnL-trnF intergenic spacer, partial sequence; chloroplast	1103	1103	76% 0.0	98% JX073805.1	1422,29	96,5%
Select seq gb|JX073824.1|	Artemisia copa tRNA-Leu (trnL) gene and trnL-trnF intergenic spacer, partial sequence; chloroplast	1068	1068	73% 0.0	97% JX073824.1	1419,12	96,3%
Select seq gb|JX073821.1|	Artemisia mauiensis tRNA-Leu (trnL) gene and trnL-trnF intergenic spacer, partial sequence; chloroplast	1068	1068	73% 0.0	97% JX073821.1	1419,12	96,3%
Select seq gb|JX073819.1|	Artemisia australis tRNA-Leu (trnL) gene and trnL-trnF intergenic spacer, partial sequence; chloroplast	1068	1068	73% 0.0	97% JX073819.1	1419,12	96,3%
Select seq gb|JX073799.1|	Artemisia gorgonum tRNA-Leu (trnL) gene and trnL-trnF intergenic spacer, partial sequence; chloroplast	1068	1068	73% 0.0	97% JX073799.1	1419,12	96,3%
Select seq gb|JX073797.1|	Artemisia dracunculus tRNA-Leu (trnL) gene and trnL-trnF intergenic spacer, partial sequence; chloroplast	1068	1068	73% 0.0	97% JX073797.1	1419,12	96,3%
Select seq gb|JX073793.1|	Artemisia campestris tRNA-Leu (trnL) gene and trnL-trnF intergenic spacer, partial sequence; chloroplast	1068	1068	73% 0.0	97% JX073793.1	1419,12	96,3%
Select seq gb|JX073778.1|	Artemisia abrotanum tRNA-Leu (trnL) gene and trnL-trnF intergenic spacer, partial sequence; chloroplast	1068	1068	73% 0.0	97% JX073778.1	1419,12	96,3%
Select seq gb|JX073773.1|	Artemisia tridentata tRNA-Leu (trnL) gene and trnL-trnF intergenic spacer, partial sequence; chloroplast	1068	1068	73% 0.0	97% JX073773.1	1419,12	96,3%
Select seq gb|JX073771.1|	Artemisia nesiotica tRNA-Leu (trnL) gene and trnL-trnF intergenic spacer, partial sequence; chloroplast	1068	1068	73% 0.0	97% JX073771.1	1419,12	96,3%
Select seq gb|JX073757.1|	Artemisia filifolia tRNA-Leu (trnL) gene and trnL-trnF intergenic spacer, partial sequence; chloroplast	1068	1068	73% 0.0	97% JX073757.1	1419,12	96,3%
Select seq gb|JX073756.1|	Artemisia franserioides tRNA-Leu (trnL) gene and trnL-trnF intergenic spacer, partial sequence; chloroplast	1068	1068	73% 0.0	97% JX073756.1	1419,12	96,3%
Select seq gb|JX073751.1|	Artemisia tripartita tRNA-Leu (trnL) gene and trnL-trnF intergenic spacer, partial sequence; chloroplast	1068	1068	73% 0.0	97% JX073751.1	1419,12	96,3%
Select seq gb|FJ842620.1|	Artemisia manshurica voucher MPS00123 (KMH) tRNA-Leu (trnL) gene and trnL-trnF intergenic spacer, partial sequence; chloroplast	1068	1068	73% 0.0	97% FJ842620.1	1419,12	96,3%
Select seq gb|FJ710524.1|	Artemisia caruifolia var. apiacea tRNA-Leu (trnL) gene and trnL-trnF intergenic spacer, partial sequence; chloroplast	1068	1068	73% 0.0	97% FJ710524.1	1419,12	96,3%
Select seq gb|JX073774.1|	Artemisia molinieri tRNA-Leu (trnL) gene and trnL-trnF intergenic spacer, partial sequence; chloroplast	1066	1066	73% 0.0	97% JX073774.1	1416,47	96,1%
Select seq gb|JX073761.1|	Artemisia anomala tRNA-Leu (trnL) gene and trnL-trnF intergenic spacer, partial sequence; chloroplast	1066	1066	73% 0.0	97% JX073761.1	1416,47	96,1%
Select seq gb|KF736827.1|	Artemisia ciniformis tRNA-Leu (trnL) gene and trnL-trnF intergenic spacer, partial sequence; chloroplast	1088	1088	75% 0.0	97% KF736827.1	1407,15	95,5%
Select seq gb|KF736825.1|	Artemisia melanolepis tRNA-Leu (trnL) gene and trnL-trnF intergenic spacer, partial sequence; chloroplast	1086	1086	75% 0.0	97% KF736825.1	1404,56	95,3%
Select seq gb|KF736809.1|	Artemisia marschalliana tRNA-Leu (trnL) gene and trnL-trnF intergenic spacer, partial sequence; chloroplast	1086	1086	75% 0.0	97% KF736809.1	1404,56	95,3%
Select seq gb|KF736808.1|	Artemisia chamaemelifolia tRNA-Leu (trnL) gene and trnL-trnF intergenic spacer, partial sequence; chloroplast	1086	1086	75% 0.0	97% KF736808.1	1404,56	95,3%
Select seq gb|KF736806.1|	Artemisia haussknechtii tRNA-Leu (trnL) gene and trnL-trnF intergenic spacer, partial sequence; chloroplast	1086	1086	75% 0.0	97% KF736806.1	1404,56	95,3%

Select seq gb|JN315916.1|	Chrysanthemum indicum tRNA-Leu (trnL) gene and trnL-trnF intergenic spacer, partial sequence; chloroplast	1086	1086	75% 0.0	97% JN315916.1	1404,56	95,3%
Select seq gb|KF736821.1|	Artemisia capillaris tRNA-Leu (trnL) gene and trnL-trnF intergenic spacer, partial sequence; chloroplast	1083	1083	75% 0.0	97% KF736821.1	1400,68	95,0%
Select seq gb|JX073814.1|	Mausolea eriocarpa tRNA-Leu (trnL) gene and trnL-trnF intergenic spacer, partial sequence; chloroplast	1110	1110	77% 0.0	97% JX073814.1	1398,31	94,9%
Select seq gb|JX073825.1|	Artemisia potentilloides tRNA-Leu (trnL) gene and trnL-trnF intergenic spacer, partial sequence; chloroplast	1107	1107	77% 0.0	97% JX073825.1	1394,53	94,6%
Select seq gb|KF736817.1|	Artemisia tschernieviana tRNA-Leu (trnL) gene and trnL-trnF intergenic spacer, partial sequence; chloroplast	1074	1074	75% 0.0	97% KF736817.1	1389,04	94,2%
Select seq gb|JX073752.1|	Artemisia rothrockii tRNA-Leu (trnL) gene and trnL-trnF intergenic spacer, partial sequence; chloroplast	1129	1235	83% 0.0	98% JX073752.1	1333,04	90,4%
Select seq gb|JX073812.1|	Artemisia subdigitata tRNA-Leu (trnL) gene and trnL-trnF intergenic spacer, partial sequence; chloroplast	1122	1227	83% 0.0	98% JX073812.1	1324,77	89,9%
Select seq gb|JX073748.1|	Artemisia santonicum tRNA-Leu (trnL) gene and trnL-trnF intergenic spacer, partial sequence; chloroplast	1122	1227	83% 0.0	98% JX073748.1	1324,77	89,9%
Select seq gb|GU817959.1|	Anacyclus valentinus isolate L1186 tRNA-Leu (trnL) gene, partial sequence; trnL-trnF intergenic spacer, complete sequence; and tRNA-Phe (trnF) gene, partial sequence; plastid	1090	1090	79% 0.0	96% GU817959.1	1324,56	89,9%
Select seq gb|JX073816.1|	Artemisia elongata tRNA-Leu (trnL) gene and trnL-trnF intergenic spacer, partial sequence; chloroplast	1118	1223	83% 0.0	97% JX073816.1	1306,58	88,6%
Select seq gb|JX073811.1|	Artemisia glomerata tRNA-Leu (trnL) gene and trnL-trnF intergenic spacer, partial sequence; chloroplast	1116	1222	83% 0.0	97% JX073811.1	1304,24	88,5%
Select seq gb|JX073808.1|	Artemisia tilesii tRNA-Leu (trnL) gene and trnL-trnF intergenic spacer, partial sequence; chloroplast	1116	1222	83% 0.0	97% JX073808.1	1304,24	88,5%
Select seq gb|JX073823.1|	Artemisia globularia tRNA-Leu (trnL) gene and trnL-trnF intergenic spacer, partial sequence; chloroplast	1112	1218	83% 0.0	97% JX073823.1	1299,57	88,2%
Select seq gb|JX073804.1|	Artemisia chamaemelifolia tRNA-Leu (trnL) gene and trnL-trnF intergenic spacer, partial sequence; chloroplast	1112	1218	83% 0.0	97% JX073804.1	1299,57	88,2%
Select seq gb|JX073828.1|	Kaschgaria komarovii tRNA-Leu (trnL) gene and trnL-trnF intergenic spacer, partial sequence; chloroplast	1110	1216	83% 0.0	97% JX073828.1	1297,23	88,0%
Select seq gb|JX073827.1|	Artemisia absinthium tRNA-Leu (trnL) gene and trnL-trnF intergenic spacer, partial sequence; chloroplast	1110	1216	83% 0.0	97% JX073827.1	1297,23	88,0%
Select seq gb|JX073791.1|	Artemisia tomentella tRNA-Leu (trnL) gene and trnL-trnF intergenic spacer, partial sequence; chloroplast	1110	1216	83% 0.0	97% JX073791.1	1297,23	88,0%
Select seq gb|JX073809.1|	Artemisia pattersonii tRNA-Leu (trnL) gene and trnL-trnF intergenic spacer, partial sequence; chloroplast	1110	1216	83% 0.0	97% JX073809.1	1297,23	88,0%
Select seq gb|JX073807.1|	Artemisia martirensis tRNA-Leu (trnL) gene and trnL-trnF intergenic spacer, partial sequence; chloroplast	1110	1216	83% 0.0	97% JX073807.1	1297,23	88,0%
Select seq gb|JX073820.1|	Artemisia kauaiensis tRNA-Leu (trnL) gene and trnL-trnF intergenic spacer, partial sequence; chloroplast	1105	1211	83% 0.0	97% JX073820.1	1291,39	87,6%
Select seq gb|JX073803.1|	Artemisia tournefortiana tRNA-Leu (trnL) gene and trnL-trnF intergenic spacer, partial sequence; chloroplast	1105	1211	83% 0.0	97% JX073803.1	1291,39	87,6%
Select seq dbj|AB551235.1|	Artemisia capillaris chloroplast genes for tRNA-Leu intron and 3' exon, trnL-trnF intergenic spacer and tRNA-Phe	1142	1288	87% 0.0	98% AB551235.1	1286,39	87,3%
Select seq dbj|AB685322.1|	Chrysanthemum indicum chloroplast DNA, contains tRNA-Leu(trnL) gene (intron and 3' exon), trnL-trnF intergenic spacer and tRNA-Phe(trnF) gene	1131	1277	87% 0.0	97% AB685322.1	1261,00	85,5%
Select seq gb|HM000099.1|	Chrysanthemum indicum isolate HEBSJZ tRNA-Leu (trnL) gene, partial sequence; trnL-trnF intergenic spacer, complete sequence; and tRNA-Phe (trnF) gene, partial sequence; chloroplast	1162	1320	94% 0.0	97% HM000099.1	1199,09	81,3%
Select seq gb|HM000094.1|	Chrysanthemum indicum isolate HUBJZ tRNA-Leu (trnL) gene, partial sequence; trnL-trnF intergenic spacer, complete sequence; and tRNA-Phe (trnF) gene, partial sequence; chloroplast	1162	1320	94% 0.0	97% HM000094.1	1199,09	81,3%
Select seq gb|JN867592.1|	Chrysanthemum indicum voucher NJ026 chloroplast, complete genome	1182	1395	97% 0.0	98% JN867592.1	1194,19	81,0%
Select seq gb|HM000091.1|	Chrysanthemum indicum isolate HENXY tRNA-Leu (trnL) gene, partial sequence; trnL-trnF intergenic spacer, complete sequence; and tRNA-Phe (trnF) gene, partial sequence; chloroplast	1149	1307	94% 0.0	97% HM000091.1	1185,67	80,4%
Select seq gb|HM000086.1|	Chrysanthemum indicum isolate JSWX tRNA-Leu (trnL) gene, partial sequence; trnL-trnF intergenic spacer, complete sequence; and tRNA-Phe (trnF) gene, partial sequence; chloroplast	1144	1301	94% 0.0	97% HM000086.1	1180,51	80,1%

Select seq gb|HM000085.1|	Chrysanthemum indicum isolate JSNJ tRNA-Leu (trnL) gene, partial sequence; trnL-trnF intergenic spacer, complete sequence; and tRNA-Phe (trnF) gene, partial sequence; chloroplast	1144	1301	94% 0.0	97%	HM000085.1	1180,51	80,1%
Select seq gb|HM000097.1|	Chrysanthemum indicum isolate JXPX tRNA-Leu (trnL) gene, partial sequence; trnL-trnF intergenic spacer, complete sequence; and tRNA-Phe (trnF) gene, partial sequence; chloroplast	1142	1299	94% 0.0	97%	HM000097.1	1178,45	79,9%
Select seq gb|HM000096.1|	Chrysanthemum indicum isolate YNKM tRNA-Leu (trnL) gene, partial sequence; trnL-trnF intergenic spacer, complete sequence; and tRNA-Phe (trnF) gene, partial sequence; chloroplast	1142	1299	94% 0.0	97%	HM000096.1	1178,45	79,9%
Select seq gb|HM000095.1|	Chrysanthemum indicum isolate HUNZZ tRNA-Leu (trnL) gene, partial sequence; trnL-trnF intergenic spacer, complete sequence; and tRNA-Phe (trnF) gene, partial sequence; chloroplast	1142	1299	94% 0.0	97%	HM000095.1	1178,45	79,9%
Select seq gb|HM000088.1|	Chrysanthemum indicum isolate GZGY tRNA-Leu (trnL) gene, partial sequence; trnL-trnF intergenic spacer, complete sequence; and tRNA-Phe (trnF) gene, partial sequence; chloroplast	1142	1299	94% 0.0	97%	HM000088.1	1178,45	79,9%
Select seq gb|HM000087.1|	Chrysanthemum indicum isolate AHCZ tRNA-Leu (trnL) gene, partial sequence; trnL-trnF intergenic spacer, complete sequence; and tRNA-Phe (trnF) gene, partial sequence; chloroplast	1142	1299	94% 0.0	97%	HM000087.1	1178,45	79,9%
Select seq gb|JX293720.1|	Artemisia frigida chloroplast, complete genome	1175	1388	97% 0.0	97%	JX293720.1	1175,00	79,7%
Select seq gb|HM000089.1|	Chrysanthemum indicum isolate AHHF tRNA-Leu (trnL) gene, partial sequence; trnL-trnF intergenic spacer, complete sequence; and tRNA-Phe (trnF) gene, partial sequence; chloroplast	1138	1296	94% 0.0	97%	HM000089.1	1174,32	79,7%
Select seq gb|HM000093.1|	Chrysanthemum indicum isolate SCMY tRNA-Leu (trnL) gene, partial sequence; trnL-trnF intergenic spacer, complete sequence; and tRNA-Phe (trnF) gene, partial sequence; chloroplast	1136	1294	94% 0.0	97%	HM000093.1	1172,26	79,5%
Select seq gb|HM000092.1|	Chrysanthemum indicum isolate GXGL tRNA-Leu (trnL) gene, partial sequence; trnL-trnF intergenic spacer, complete sequence; and tRNA-Phe (trnF) gene, partial sequence; chloroplast	1136	1294	94% 0.0	97%	HM000092.1	1172,26	79,5%
Select seq gb|JN867589.1|	Chrysanthemum indicum voucher HeN001 chloroplast, complete genome	1170	1382	97% 0.0	97%	JN867589.1	1170,00	79,4%
Select seq gb|JQ362483.1|	Chrysanthemum x morifolium chloroplast, complete genome	1170	1382	97% 0.0	97%	JQ362483.1	1170,00	79,4%
Select seq gb|HM000098.1|	Chrysanthemum indicum isolate FJFZ tRNA-Leu (trnL) gene, partial sequence; trnL-trnF intergenic spacer, complete sequence; and tRNA-Phe (trnF) gene, partial sequence; chloroplast	1133	1290	94% 0.0	97%	HM000098.1	1169,16	79,3%
Select seq gb|HM000090.1|	Chrysanthemum indicum isolate ZJWZ tRNA-Leu (trnL) gene, partial sequence; trnL-trnF intergenic spacer, complete sequence; and tRNA-Phe (trnF) gene, partial sequence; chloroplast	1131	1288	94% 0.0	97%	HM000090.1	1167,10	79,2%
Select seq gb|KF887960.1|	Artemisia montana chloroplast, complete genome	1131	1344	97% 0.0	96%	KF887960.1	1119,34	75,9%
							0,00	0,0%
							0,00	0,0%

Select for downloading or viewing reports	Kh102_ITS Description	Max score	Total score	Query cover	E value	Ident	Accession	(Ident/Cover)* Max score	Deviation from top hit
Select seq gb|DQ250287.1|	Papaver bracteatum voucher 2000.0031 internal transcribed spacer 1, partial sequence; 5.8S ribosomal RNA gene, complete sequence; and internal transcribed spacer 2, partial sequence	1190	1190	83%	0.0	99%	DQ250287.1	1419,40	100,0%
Select seq gb|DQ250286.1|	Papaver bracteatum voucher 2000.0028 internal transcribed spacer 1, partial sequence; 5.8S ribosomal RNA gene, complete sequence; and internal transcribed spacer 2, partial sequence	1171	1171	83%	0.0	99%	DQ250286.1	1396,73	98,4%
Select seq gb|DQ912881.1|	Papaver bracteatum 18S ribosomal RNA gene, partial sequence; internal transcribed spacer 1, 5.8S ribosomal RNA gene, and internal transcribed spacer 2, complete sequence; and 28S ribosomal RNA gene, partial sequence	1349	1349	96%	0.0	99%	DQ912881.1	1391,16	98,0%
Select seq gb|DQ250321.1|	Papaver pilosum voucher 2000.0768 internal transcribed spacer 1, partial sequence; 5.8S ribosomal RNA gene, complete sequence; and internal transcribed spacer 2, partial sequence	1072	1072	83%	0.0	97%	DQ250321.1	1252,82	88,3%
Select seq gb|DQ250320.1|	Papaver pilosum voucher 2000.0182 internal transcribed spacer 1, partial sequence; 5.8S ribosomal RNA gene, complete sequence; and internal transcribed spacer 2, partial sequence	1066	1066	83%	0.0	96%	DQ250320.1	1232,96	86,9%
Select seq gb|DQ250294.1|	Papaver armeniacum voucher 2000.0107 internal transcribed spacer 1, partial sequence; 5.8S ribosomal RNA gene, complete sequence; and internal transcribed spacer 2, partial sequence	1066	1066	83%	0.0	96%	DQ250294.1	1232,96	86,9%
Select seq gb|DQ250297.1|	Papaver armeniacum voucher 2000.0604 internal transcribed spacer 1, partial sequence; 5.8S ribosomal RNA gene, complete sequence; and internal transcribed spacer 2, partial sequence	1061	1061	83%	0.0	96%	DQ250297.1	1227,18	86,5%
Select seq gb|AY328298.1|	Papaver sp. Yuan 2000611 internal transcribed spacer 1, partial sequence; 5.8S ribosomal RNA gene, complete sequence; and internal transcribed spacer 2, partial sequence	1085	1085	85%	0.0	96%	AY328298.1	1225,41	86,3%
Select seq gb|DQ250302.1|	Papaver armeniacum voucher 2000.1717 internal transcribed spacer 1, partial sequence; 5.8S ribosomal RNA gene, complete sequence; and internal transcribed spacer 2, partial sequence	1055	1055	83%	0.0	96%	DQ250302.1	1220,24	86,0%
Select seq gb|DQ250311.1|	Papaver armeniacum voucher 2000.0651 internal transcribed spacer 1, partial sequence; 5.8S ribosomal RNA gene, complete sequence; and internal transcribed spacer 2, partial sequence	1050	1050	83%	0.0	96%	DQ250311.1	1214,46	85,6%
Select seq gb|DQ250259.1|	Papaver armeniacum voucher 2000.0793 internal transcribed spacer 1, partial sequence; 5.8S ribosomal RNA gene, complete sequence; and internal transcribed spacer 2, partial sequence	1050	1050	83%	0.0	96%	DQ250259.1	1214,46	85,6%
Select seq gb|JX078983.1|	Papaver lateritium voucher TEX:W.Xiao 090527-3 internal transcribed spacer 1, partial sequence; 5.8S ribosomal RNA gene, complete sequence; and internal transcribed spacer 2, partial sequence	1075	1075	85%	0.0	96%	JX078983.1	1214,12	85,5%
Select seq gb|DQ912882.1|	Papaver orientale 18S ribosomal RNA gene, partial sequence; internal transcribed spacer 1, 5.8S ribosomal RNA gene, and internal transcribed spacer 2, complete sequence; and 28S ribosomal RNA gene, partial sequence	1214	1214	96%	0.0	96%	DQ912882.1	1214,00	85,5%
Select seq gb|DQ250269.1|	Papaver pseudoorientale voucher 2000.0014 internal transcribed spacer 1, partial sequence; 5.8S ribosomal RNA gene, complete sequence; and internal transcribed spacer 2, partial sequence	1046	1046	83%	0.0	96%	DQ250269.1	1209,83	85,2%
Select seq gb|DQ364699.1|	Papaver somniferum cultivar Nigrum 18S ribosomal RNA gene, partial sequence; internal transcribed spacer 1, 5.8S ribosomal RNA gene, and internal transcribed spacer 2, complete sequence; and 28S ribosomal RNA gene, partial sequence	1155	1155	92%	0.0	96%	DQ364699.1	1205,22	84,9%
Select seq gb|DQ250308.1|	Papaver glaucum voucher 2000.0189 internal transcribed spacer 1, partial sequence; 5.8S ribosomal RNA gene, complete sequence; and internal transcribed spacer 2, partial sequence	1038	1038	83%	0.0	96%	DQ250308.1	1200,58	84,6%
Select seq gb|DQ250275.1|	Papaver macrostomum voucher RBGE 34139 internal transcribed spacer 1, partial sequence; 5.8S ribosomal RNA gene, complete sequence; and internal transcribed spacer 2, partial sequence	1038	1038	83%	0.0	96%	DQ250275.1	1200,58	84,6%
Select seq gb|DQ250306.1|	Papaver somniferum voucher 2000.0142 internal transcribed spacer 1, partial sequence; 5.8S ribosomal RNA gene, complete sequence; and internal transcribed spacer 2, partial sequence	1037	1037	83%	0.0	96%	DQ250306.1	1199,42	84,5%
Select seq gb|DQ912886.1|	Papaver rhoeas 18S ribosomal RNA gene, partial sequence; internal transcribed spacer 1, 5.8S ribosomal RNA gene, and internal transcribed spacer 2, complete sequence; and 28S ribosomal RNA gene, partial sequence	1199	1199	96%	0.0	96%	DQ912886.1	1199,00	84,5%
Select seq gb|DQ250292.1|	Papaver orientale voucher 2000.0011 internal transcribed spacer 1, partial sequence; 5.8S ribosomal RNA gene, complete sequence; and internal transcribed spacer 2, partial sequence	1035	1035	83%	0.0	96%	DQ250292.1	1197,11	84,3%
Select seq gb|JN584656.1|	Papaver somniferum subsp. setigerum isolate 1 clone 1 18S ribosomal RNA gene, partial sequence; internal transcribed spacer 1, 5.8S ribosomal RNA gene, and internal transcribed spacer 2, complete sequence; and 28S ribosomal RNA gene, partial sequence	1197	1197	96%	0.0	96%	JN584656.1	1197,00	84,3%
Select seq gb|DQ250279.1|	Papaver somniferum voucher 2000.0016 internal transcribed spacer 1, partial sequence; 5.8S ribosomal RNA gene, complete sequence; and internal transcribed spacer 2, partial sequence	1031	1031	83%	0.0	96%	DQ250279.1	1192,48	84,0%
Select seq gb|AY689330.1|	Papaver somniferum 18S ribosomal RNA gene, partial sequence; internal transcribed spacer 1, 5.8S ribosomal RNA gene, and internal transcribed spacer 2, complete sequence; and 28S ribosomal RNA gene, partial sequence	1136	1136	91%	0.0	95%	AY689330.1	1185,93	83,6%
Select seq gb|DQ912880.1|	Papaver somniferum 18S ribosomal RNA gene, partial sequence; internal transcribed spacer 1, 5.8S ribosomal RNA gene, and internal transcribed spacer 2, complete sequence; and 28S ribosomal RNA gene, partial sequence	1192	1192	96%	0.0	95%	DQ912880.1	1179,58	83,1%
Select seq gb|DQ250309.1|	Papaver glaucum voucher 2000.0177 internal transcribed spacer 1, partial sequence; 5.8S ribosomal RNA gene, complete sequence; and internal transcribed spacer 2, partial sequence	1027	1027	83%	0.0	95%	DQ250309.1	1175,48	82,8%
Select seq gb|DQ250280.1|	Papaver somniferum voucher 2000.0130 internal transcribed spacer 1, partial sequence; 5.8S ribosomal RNA gene, complete sequence; and internal transcribed spacer 2, partial sequence	1026	1026	83%	0.0	95%	DQ250280.1	1174,34	82,7%

Select seq gb|JN584657.1|	Papaver somniferum subsp. setigerum isolate 1 clone 2 18S ribosomal RNA gene, partial sequence; internal transcribed spacer 1, 5.8S ribosomal RNA gene, and internal transcribed spacer 2, complete sequence; and 28S ribosomal RNA gene, partial sequence	1186	1186	96%	0.0	95%	JN584657.1	1173,65	82,7%
Select seq gb|JN115039.1|	Papaver somniferum voucher SBB-0468 internal transcribed spacer 1, partial sequence; 5.8S ribosomal RNA gene, complete sequence; and internal transcribed spacer 2, partial sequence	1074	1074	87%	0.0	95%	JN115039.1	1172,76	82,6%
Select seq gb|DQ250312.1|	Papaver armeniacum voucher 2000.0795 internal transcribed spacer 1, partial sequence; 5.8S ribosomal RNA gene, complete sequence; and internal transcribed spacer 2, partial sequence	1022	1022	83%	0.0	95%	DQ250312.1	1169,76	82,4%
Select seq gb|DQ250273.1|	Papaver rhoeas voucher KJ93/7 Kadereit et al., 1996 internal transcribed spacer 1, partial sequence; 5.8S ribosomal RNA gene, complete sequence; and internal transcribed spacer 2, partial sequence	1022	1022	83%	0.0	95%	DQ250273.1	1169,76	82,4%
Select seq gb|DQ250272.1|	Papaver rhoeas voucher 2000.0090 internal transcribed spacer 1, partial sequence; 5.8S ribosomal RNA gene, complete sequence; and internal transcribed spacer 2, partial sequence	1022	1022	83%	0.0	95%	DQ250272.1	1169,76	82,4%
Select seq gb|DQ250282.1|	Papaver somniferum voucher 2000.0068 internal transcribed spacer 1, partial sequence; 5.8S ribosomal RNA gene, complete sequence; and internal transcribed spacer 2, partial sequence	1020	1020	83%	0.0	95%	DQ250282.1	1167,47	82,3%
Select seq gb|AY328297.1|	Papaver somniferum specimen-voucher Yuan 200271102 internal transcribed spacer 1, partial sequence; 5.8S ribosomal RNA gene, complete sequence; and internal transcribed spacer 2, partial sequence	1042	1042	85%	0.0	95%	AY328297.1	1164,59	82,0%
Select seq gb|DQ250313.1|	Papaver commutatum voucher 2000.0605 internal transcribed spacer 1, partial sequence; 5.8S ribosomal RNA gene, complete sequence; and internal transcribed spacer 2, partial sequence	1016	1016	83%	0.0	95%	DQ250313.1	1162,89	81,9%
Select seq gb|DQ250310.1|	Papaver glaucum voucher 2000.0609 internal transcribed spacer 1, partial sequence; 5.8S ribosomal RNA gene, complete sequence; and internal transcribed spacer 2, partial sequence	1016	1016	83%	0.0	95%	DQ250310.1	1162,89	81,9%
Select seq gb|DQ250285.1|	Papaver pseudoorientale voucher 2000.0794 internal transcribed spacer 1, partial sequence; 5.8S ribosomal RNA gene, complete sequence; and internal transcribed spacer 2, partial sequence	1016	1016	83%	0.0	95%	DQ250285.1	1162,89	81,9%
Select seq gb|FJ469600.1|	Papaver rhoeas isolate 2 18S ribosomal RNA gene, partial sequence; internal transcribed spacer 1, 5.8S ribosomal RNA gene, and internal transcribed spacer 2, complete sequence; and 28S ribosomal RNA gene, partial sequence	1171	1171	96%	0.0	95%	FJ469600.1	1158,80	81,6%
Select seq gb|JN584660.1|	Papaver somniferum subsp. setigerum isolate 2 clone 7 18S ribosomal RNA gene, partial sequence; internal transcribed spacer 1, 5.8S ribosomal RNA gene, and internal transcribed spacer 2, complete sequence; and 28S ribosomal RNA gene, partial sequence	1170	1170	96%	0.0	95%	JN584660.1	1157,81	81,6%
Select seq gb|JN584658.1|	Papaver somniferum subsp. setigerum isolate 2 clone 2 18S ribosomal RNA gene, partial sequence; internal transcribed spacer 1, 5.8S ribosomal RNA gene, and internal transcribed spacer 2, complete sequence; and 28S ribosomal RNA gene, partial sequence	1170	1170	96%	0.0	95%	JN584658.1	1157,81	81,6%
Select seq gb|DQ250322.1|	Papaver dubium voucher 2000.0606 internal transcribed spacer 1, partial sequence; 5.8S ribosomal RNA gene, complete sequence; and internal transcribed spacer 2, partial sequence	1005	1005	83%	0.0	95%	DQ250322.1	1150,30	81,0%
Select seq gb|DQ250305.1|	Papaver somniferum voucher 2000.0063 internal transcribed spacer 1, partial sequence; 5.8S ribosomal RNA gene, complete sequence; and internal transcribed spacer 2, partial sequence	1003	1003	83%	0.0	95%	DQ250305.1	1148,01	80,9%
Select seq gb|DQ250288.1|	Papaver pseudoorientale voucher 2000.0039 internal transcribed spacer 1, partial sequence; 5.8S ribosomal RNA gene, complete sequence; and internal transcribed spacer 2, partial sequence	1002	1002	83%	0.0	95%	DQ250288.1	1146,87	80,8%
Select seq gb|DQ250291.1|	Papaver orientale voucher RBGE 19880542A internal transcribed spacer 1, partial sequence; 5.8S ribosomal RNA gene, complete sequence; and internal transcribed spacer 2, partial sequence	1000	1000	83%	0.0	95%	DQ250291.1	1144,58	80,6%
Select seq gb|AF098920.1|AF098920	Papaver rhoeas internal transcribed spacer 1, partial sequence; 5.8S ribosomal RNA gene, complete sequence; and internal transcribed spacer 2, partial sequence	1027	1027	85%	0.0	94%	AF098920.1	1135,74	80,0%
Select seq gb|DQ250319.1|	Papaver dubium voucher 2000.01706 internal transcribed spacer 1, partial sequence; 5.8S ribosomal RNA gene, complete sequence; and internal transcribed spacer 2, partial sequence	996	996	83%	0.0	94%	DQ250319.1	1128,00	79,5%
Select seq gb|DQ250289.1|	Papaver orientale voucher 2000.0035 internal transcribed spacer 1, partial sequence; 5.8S ribosomal RNA gene, complete sequence; and internal transcribed spacer 2, partial sequence	994	994	83%	0.0	94%	DQ250289.1	1125,73	79,3%
Select seq gb|JN584659.1|	Papaver somniferum subsp. setigerum isolate 1 clone 8 18S ribosomal RNA gene, partial sequence; internal transcribed spacer 1, 5.8S ribosomal RNA gene, and internal transcribed spacer 2, complete sequence; and 28S ribosomal RNA gene, partial sequence	1147	1147	96%	0.0	94%	JN584659.1	1123,10	79,1%
Select seq gb|DQ250281.1|	Papaver somniferum voucher 2000.0052 internal transcribed spacer 1, partial sequence; 5.8S ribosomal RNA gene, complete sequence; and internal transcribed spacer 2, partial sequence	990	990	83%	0.0	94%	DQ250281.1	1121,20	79,0%
Select seq gb|DQ250267.1|	Papaver dubium voucher 2000.0600 internal transcribed spacer 1, partial sequence; 5.8S ribosomal RNA gene, complete sequence; and internal transcribed spacer 2, partial sequence	990	990	83%	0.0	94%	DQ250267.1	1121,20	79,0%
Select seq gb|DQ250271.1|	Papaver dubium voucher 2000.0610 internal transcribed spacer 1, partial sequence; 5.8S ribosomal RNA gene, complete sequence; and internal transcribed spacer 2, partial sequence	989	989	83%	0.0	94%	DQ250271.1	1120,07	78,9%
Select seq gb|DQ250270.1|	Papaver dubium voucher 2000.0024 internal transcribed spacer 1, partial sequence; 5.8S ribosomal RNA gene, complete sequence; and internal transcribed spacer 2, partial sequence	985	985	83%	0.0	94%	DQ250270.1	1115,54	78,6%
Select seq gb|DQ250304.1|	Papaver somniferum voucher 2000.0166 internal transcribed spacer 1, partial sequence; 5.8S ribosomal RNA gene, complete sequence; and internal transcribed spacer 2, partial sequence	976	976	83%	0.0	94%	DQ250304.1	1105,35	77,9%
Select seq gb|DQ250296.1|	Papaver pseudoorientale voucher 2000.0632 internal transcribed spacer 1, partial sequence; 5.8S ribosomal RNA gene, complete sequence; and internal transcribed spacer 2, partial sequence	941	941	83%	0.0	93%	DQ250296.1	1054,37	74,3%

Select seq gb|DQ250314.1|	Papaver rupifragum voucher 1999.0342 internal transcribed spacer 1, partial sequence; 5.8S ribosomal RNA gene, complete sequence; and internal transcribed spacer 2, partial sequence	926	926	83% 0.0	92%	DQ250314.1	1026,41	72,3%
Select seq gb|DQ250315.1|	Papaver atlanticum voucher 2000.0615 internal transcribed spacer 1, partial sequence; 5.8S ribosomal RNA gene, complete sequence; and internal transcribed spacer 2, partial sequence	920	920	83% 0.0	92%	DQ250315.1	1019,76	71,8%
Select seq gb|DQ250307.1|	Papaver atlanticum voucher 2000.0603 internal transcribed spacer 1, partial sequence; 5.8S ribosomal RNA gene, complete sequence; and internal transcribed spacer 2, partial sequence	920	920	83% 0.0	92%	DQ250307.1	1019,76	71,8%
Select seq gb|DQ250303.1|	Papaver atlanticum voucher 2000.0156 internal transcribed spacer 1, partial sequence; 5.8S ribosomal RNA gene, complete sequence; and internal transcribed spacer 2, partial sequence	920	920	83% 0.0	92%	DQ250303.1	1019,76	71,8%
Select seq gb|DQ250293.1|	Papaver atlanticum voucher 2000.0472 internal transcribed spacer 1, partial sequence; 5.8S ribosomal RNA gene, complete sequence; and internal transcribed spacer 2, partial sequence	920	920	83% 0.0	92%	DQ250293.1	1019,76	71,8%
Select seq gb|AY328299.1|	Meconopsis cambrica specimen-voucher Yuan 200271106 internal transcribed spacer 1, partial sequence; 5.8S ribosomal RNA gene, complete sequence; and internal transcribed spacer 2, partial sequence	933	933	85% 0.0	92%	AY328299.1	1009,84	71,1%
Select seq gb|JF774151.1|	Meconopsis cambrica isolate IB2 internal transcribed spacer 1, partial sequence; 5.8S ribosomal RNA gene, complete sequence; and internal transcribed spacer 2, partial sequence	907	907	83% 0.0	92%	JF774151.1	1005,35	70,8%
Select seq gb|JF774141.1|	Meconopsis cambrica isolate GB7 internal transcribed spacer 1, partial sequence; 5.8S ribosomal RNA gene, complete sequence; and internal transcribed spacer 2, partial sequence	907	907	83% 0.0	92%	JF774141.1	1005,35	70,8%
Select seq gb|JF774160.1|	Meconopsis cambrica isolate PY2 internal transcribed spacer 1, partial sequence; 5.8S ribosomal RNA gene, complete sequence; and internal transcribed spacer 2, partial sequence	902	902	83% 0.0	92%	JF774160.1	999,81	70,4%
Select seq gb|JF774158.1|	Meconopsis cambrica isolate OR3-2 internal transcribed spacer 1, partial sequence; 5.8S ribosomal RNA gene, complete sequence; and internal transcribed spacer 2, partial sequence	902	902	83% 0.0	92%	JF774158.1	999,81	70,4%
Select seq gb|JF774155.1|	Meconopsis cambrica isolate OR1 internal transcribed spacer 1, partial sequence; 5.8S ribosomal RNA gene, complete sequence; and internal transcribed spacer 2, partial sequence	902	902	83% 0.0	92%	JF774155.1	999,81	70,4%
Select seq gb|JF774152.1|	Meconopsis cambrica isolate IB3 internal transcribed spacer 1, partial sequence; 5.8S ribosomal RNA gene, complete sequence; and internal transcribed spacer 2, partial sequence	902	902	83% 0.0	92%	JF774152.1	999,81	70,4%
Select seq gb|JF774140.1|	Meconopsis cambrica isolate GB6 internal transcribed spacer 1, partial sequence; 5.8S ribosomal RNA gene, complete sequence; and internal transcribed spacer 2, partial sequence	902	902	83% 0.0	92%	JF774140.1	999,81	70,4%
Select seq gb|JF774135.1|	Meconopsis cambrica isolate GB1 internal transcribed spacer 1, partial sequence; 5.8S ribosomal RNA gene, complete sequence; and internal transcribed spacer 2, partial sequence	902	902	83% 0.0	92%	JF774135.1	999,81	70,4%
Select seq gb|DQ250278.1|	Meconopsis cambrica voucher 1992.0611 internal transcribed spacer 1, partial sequence; 5.8S ribosomal RNA gene, complete sequence; and internal transcribed spacer 2, partial sequence	898	898	83% 0.0	92%	DQ250278.1	995,37	70,1%
Select seq gb|JX078996.1|	Meconopsis cambrica voucher E:W.Xiao RICB1 internal transcribed spacer 1, partial sequence; 5.8S ribosomal RNA gene, complete sequence; and internal transcribed spacer 2, partial sequence	918	918	85% 0.0	91%	JX078996.1	982,80	69,2%
Select seq gb|JF774146.1|	Meconopsis cambrica isolate GB12 internal transcribed spacer 1, partial sequence; 5.8S ribosomal RNA gene, complete sequence; and internal transcribed spacer 2, partial sequence	885	885	83% 0.0	91%	JF774146.1	970,30	68,4%
Select seq gb|JF774137.1|	Meconopsis cambrica isolate GB3 internal transcribed spacer 1, partial sequence; 5.8S ribosomal RNA gene, complete sequence; and internal transcribed spacer 2, partial sequence	885	885	83% 0.0	91%	JF774137.1	970,30	68,4%
Select seq gb|DQ250290.1|	Papaver orientale voucher 2000.0135 internal transcribed spacer 1, partial sequence; 5.8S ribosomal RNA gene, complete sequence; and internal transcribed spacer 2, partial sequence	883	883	83% 0.0	91%	DQ250290.1	968,11	68,2%
Select seq gb|DQ250277.1|	Meconopsis cambrica voucher 2000.0001 internal transcribed spacer 1, partial sequence; 5.8S ribosomal RNA gene, complete sequence; and internal transcribed spacer 2, partial sequence	856	856	82% 0.0	91%	DQ250277.1	949,95	66,9%
Select seq gb|JX079012.1|	Papaver sp. Xiao 090527-1 internal transcribed spacer 1, partial sequence; 5.8S ribosomal RNA gene, complete sequence; and internal transcribed spacer 2, partial sequence	808	808	85% 0.0	89%	JX079012.1	846,02	59,6%
Select seq gb|DQ250318.1|	Papaver californicum voucher 2000.0170 internal transcribed spacer 1, partial sequence; 5.8S ribosomal RNA gene, complete sequence; and internal transcribed spacer 2, partial sequence	787	787	83% 0.0	89%	DQ250318.1	843,89	59,5%
Select seq gb|DQ250317.1|	Papaver aculeatum voucher 2000.0131 internal transcribed spacer 1, partial sequence; 5.8S ribosomal RNA gene, complete sequence; and internal transcribed spacer 2, partial sequence	787	787	83% 0.0	89%	DQ250317.1	843,89	59,5%
Select seq gb|DQ250316.1|	Papaver aculeatum voucher 2000.0659 internal transcribed spacer 1, partial sequence; 5.8S ribosomal RNA gene, complete sequence; and internal transcribed spacer 2, partial sequence	782	782	83% 0.0	89%	DQ250316.1	838,53	59,1%
Select seq gb|AY328296.1|	Papaver spicatum specimen-voucher Yuan 200271101 internal transcribed spacer 1, partial sequence; 5.8S ribosomal RNA gene, complete sequence; and internal transcribed spacer 2, partial sequence	780	780	85% 0.0	88%	AY328296.1	807,53	56,9%
Select seq gb|DQ912885.1|	Papaver nudicaule 18S ribosomal RNA gene, partial sequence; internal transcribed spacer 1, 5.8S ribosomal RNA gene, and internal transcribed spacer 2, complete sequence; and 28S ribosomal RNA gene, partial sequence	874	874	96% 0.0	88%	DQ912885.1	801,17	56,4%
Select seq gb|JF411034.1|	Meconopsis racemosa voucher Li & Dao 8A internal transcribed spacer 1, partial sequence; 5.8S ribosomal RNA gene, complete sequence; and internal transcribed spacer 2, partial sequence	800	800	88% 0.0	88%	JF411034.1	800,00	56,4%
Select seq gb|JF411036.1|	Meconopsis racemosa voucher Li & Dao 8C internal transcribed spacer 1, partial sequence; 5.8S ribosomal RNA gene, complete sequence; and internal transcribed spacer 2, partial sequence	795	795	88% 0.0	88%	JF411036.1	795,00	56,0%

Select seq gb|AY328260.1|	Meconopsis racemosa specimen-voucher Boufford 29724 internal transcribed spacer 1, partial sequence; 5.8S ribosomal RNA gene, complete sequence; and internal transcribed spacer 2, partial sequence	767	767	85% 0.0	88% AY328260.1	794,07	55,9%
Select seq gb|AY328257.1|	Meconopsis racemosa specimen-voucher Boufford 29486 internal transcribed spacer 1, partial sequence; 5.8S ribosomal RNA gene, complete sequence; and internal transcribed spacer 2, partial sequence	767	767	85% 0.0	88% AY328257.1	794,07	55,9%
Select seq gb|DQ912879.1|	Papaver radicatum 18S ribosomal RNA gene, partial sequence; internal transcribed spacer 1, 5.8S ribosomal RNA gene, and internal transcribed spacer 2, complete sequence; and 28S ribosomal RNA gene, partial sequence	865	865	96% 0.0	88% DQ912879.1	792,92	55,9%
Select seq gb|KF022360.1|	Papaver nudicaule isolate 01 18S ribosomal RNA gene, partial sequence; internal transcribed spacer 1, 5.8S ribosomal RNA gene, and internal transcribed spacer 2, complete sequence; and 28S ribosomal RNA gene, partial sequence	841	841	94% 0.0	88% KF022360.1	787,32	55,5%
Select seq gb|AY328295.1|	Meconopsis quintuplinervia specimen-voucher Yuan 200271107 internal transcribed spacer 1, partial sequence; 5.8S ribosomal RNA gene, complete sequence; and internal transcribed spacer 2, partial sequence	761	761	85% 0.0	87% AY328295.1	778,91	54,9%
Select seq gb|KF688598.1|	Meconopsis racemosa isolate ZZ internal transcribed spacer 1, partial sequence; 5.8S ribosomal RNA gene, complete sequence; and internal transcribed spacer 2, partial sequence	758	758	85% 0.0	87% KF688598.1	775,84	54,7%
Select seq gb|JX079000.1|	Meconopsis horridula voucher HUH:Boufford 38099 internal transcribed spacer 1, partial sequence; 5.8S ribosomal RNA gene, complete sequence; and internal transcribed spacer 2, partial sequence	758	758	85% 0.0	87% JX079000.1	775,84	54,7%
Select seq gb|JX078978.1|	Meconopsis horridula voucher HUH:Boufford 33724 internal transcribed spacer 1, partial sequence; 5.8S ribosomal RNA gene, complete sequence; and internal transcribed spacer 2, partial sequence	758	758	85% 0.0	87% JX078978.1	775,84	54,7%
Select seq gb|AY328261.1|	Meconopsis horridula specimen-voucher Boufford 30011 internal transcribed spacer 1, partial sequence; 5.8S ribosomal RNA gene, complete sequence; and internal transcribed spacer 2, partial sequence	758	758	85% 0.0	87% AY328261.1	775,84	54,7%
Select seq gb|AY328258.1|	Meconopsis horridula specimen-voucher Boufford 30022 internal transcribed spacer 1, partial sequence; 5.8S ribosomal RNA gene, complete sequence; and internal transcribed spacer 2, partial sequence	758	758	85% 0.0	87% AY328258.1	775,84	54,7%
Select seq gb|JX079044.1|	Meconopsis horridula voucher E:ACE 1773 internal transcribed spacer 1, partial sequence; 5.8S ribosomal RNA gene, complete sequence; and internal transcribed spacer 2, partial sequence	756	756	85% 0.0	87% JX079044.1	773,79	54,5%
Select seq gb|AY328262.1|	Meconopsis racemosa specimen-voucher Yuan 2000635 internal transcribed spacer 1, partial sequence; 5.8S ribosomal RNA gene, complete sequence; and internal transcribed spacer 2, partial sequence	756	756	85% 0.0	87% AY328262.1	773,79	54,5%
Select seq gb|JX078981.1|	Meconopsis horridula voucher HUH:Boufford 33530 internal transcribed spacer 1, partial sequence; 5.8S ribosomal RNA gene, complete sequence; and internal transcribed spacer 2, partial sequence	752	752	85% 0.0	87% JX078981.1	769,69	54,2%
Select seq gb|JX078988.1|	Meconopsis horridula voucher TEX:W.Xiao 080616 internal transcribed spacer 1, partial sequence; 5.8S ribosomal RNA gene, complete sequence; and internal transcribed spacer 2, partial sequence	747	747	85% 0.0	87% JX078988.1	764,58	53,9%
Select seq gb|JF411037.1|	Meconopsis racemosa voucher Li & Dao 40 internal transcribed spacer 1, partial sequence; 5.8S ribosomal RNA gene, complete sequence; and internal transcribed spacer 2, partial sequence	773	773	88% 0.0	87% JF411037.1	764,22	53,8%
Select seq gb|JF411033.1|	Meconopsis lancifolia voucher Li & Dao 44 internal transcribed spacer 1, partial sequence; 5.8S ribosomal RNA gene, complete sequence; and internal transcribed spacer 2, partial sequence	767	767	88% 0.0	87% JF411033.1	758,28	53,4%
Select seq gb|JF411032.1|	Meconopsis integrifolia voucher Li & Dao 43 internal transcribed spacer 1, partial sequence; 5.8S ribosomal RNA gene, complete sequence; and internal transcribed spacer 2, partial sequence	756	756	88% 0.0	87% JF411032.1	747,41	52,7%
Select seq gb|JF411031.1|	Meconopsis integrifolia voucher Li & Dao 31 internal transcribed spacer 1, partial sequence; 5.8S ribosomal RNA gene, complete sequence; and internal transcribed spacer 2, partial sequence	750	750	88% 0.0	87% JF411031.1	741,48	

Select for downloading or viewing reports	Kh102_trnL Description	Max score	Total score	Query cover	E value	Ident	Accession	(Ident/Cover)* Max score	Deviation from top hit
Select seq gb|DQ251143.1|	Papaver orientale voucher 2000.0011 tRNA-Leu (trnL) gene, partial sequence; trnL-trnF intergenic spacer, complete sequence; and tRNA-Phe (trnF) gene, partial sequence; chloroplast	909	909	95%	0.0	95%	DQ251143.1	909,00	100,0%
Select seq gb|DQ251137.1|	Papaver bracteatum voucher 2000.0028 tRNA-Leu (trnL) gene, partial sequence; trnL-trnF intergenic spacer, complete sequence; and tRNA-Phe (trnF) gene, partial sequence; chloroplast	902	902	95%	0.0	95%	DQ251137.1	902,00	99,2%
Select seq gb|DQ251138.1|	Papaver bracteatum voucher 2000.0031 tRNA-Leu (trnL) gene, partial sequence; trnL-trnF intergenic spacer, complete sequence; and tRNA-Phe (trnF) gene, partial sequence; chloroplast	891	891	95%	0.0	94%	DQ251138.1	881,62	97,0%
Select seq gb|JX087776.1|	Papaver lateritium voucher TEX:W.Xiao 090527-3 tRNA-Leu (trnL) gene and trnL-trnF intergenic spacer, partial sequence; chloroplast	887	887	95%	0.0	94%	JX087776.1	877,66	96,6%
Select seq gb|DQ251120.1|	Papaver pseudo-orientale voucher 2000.0014 tRNA-Leu (trnL) gene, partial sequence; trnL-trnF intergenic spacer, complete sequence; and tRNA-Phe (trnF) gene, partial sequence; chloroplast	887	887	95%	0.0	94%	DQ251120.1	877,66	96,6%
Select seq gb|AY328241.1|	Papaver sp. Yuan 2000611 tRNA-Leu (trnL) gene and trnL-trnF intergenic spacer, complete sequence; and tRNA-Phe (trnF) gene, partial sequence; chloroplast	887	887	95%	0.0	94%	AY328241.1	877,66	96,6%
Select seq gb|DQ251142.1|	Papaver orientale voucher RBGE 19880542A tRNA-Leu (trnL) gene, partial sequence; trnL-trnF intergenic spacer, complete sequence; and tRNA-Phe (trnF) gene, partial sequence; chloroplast	881	881	95%	0.0	94%	DQ251142.1	871,73	95,9%
Select seq gb|DQ251136.1|	Papaver pseudo-orientale voucher 2000.0794 tRNA-Leu (trnL) gene, partial sequence; trnL-trnF intergenic spacer, complete sequence; and tRNA-Phe (trnF) gene, partial sequence; chloroplast	881	881	95%	0.0	94%	DQ251136.1	871,73	95,9%
Select seq gb|DQ251110.1|	Papaver armeniacum voucher 2000.0793 tRNA-Leu (trnL) gene, partial sequence; trnL-trnF intergenic spacer, complete sequence; and tRNA-Phe (trnF) gene, partial sequence; chloroplast	876	876	95%	0.0	94%	DQ251110.1	866,78	95,4%
Select seq gb|DQ251147.1|	Papaver pseudo-orientale voucher 2000.0632 tRNA-Leu (trnL) gene, partial sequence; trnL-trnF intergenic spacer, complete sequence; and tRNA-Phe (trnF) gene, partial sequence; chloroplast	876	876	95%	0.0	94%	DQ251147.1	866,78	95,4%
Select seq emb|AM397153.1|	Papaver triniaefolium tRNA-Leu gene (partial), trnL-trnF intergenic spacer and tRNA-Phe gene (partial), specimen voucher A. Worberg 018 (BONN)	870	870	95%	0.0	94%	AM397153.1	860,84	94,7%
Select seq gb|DQ251162.1|	Papaver armeniacum voucher 2000.0651 tRNA-Leu (trnL) gene, partial sequence; trnL-trnF intergenic spacer, complete sequence; and tRNA-Phe (trnF) gene, partial sequence; chloroplast	870	870	95%	0.0	94%	DQ251162.1	860,84	94,7%
Select seq gb|DQ251148.1|	Papaver armeniacum voucher 2000.0604 tRNA-Leu (trnL) gene, partial sequence; trnL-trnF intergenic spacer, complete sequence; and tRNA-Phe (trnF) gene, partial sequence; chloroplast	870	870	95%	0.0	94%	DQ251148.1	860,84	94,7%
Select seq gb|DQ251170.1|	Papaver dubium voucher 2000.01706 tRNA-Leu (trnL) gene, partial sequence; trnL-trnF intergenic spacer, complete sequence; and tRNA-Phe (trnF) gene, partial sequence; chloroplast	859	859	95%	0.0	93%	DQ251170.1	840,92	92,5%
Select seq gb|DQ251118.1|	Papaver dubium voucher 2000.0600 tRNA-Leu (trnL) gene, partial sequence; trnL-trnF intergenic spacer, complete sequence; and tRNA-Phe (trnF) gene, partial sequence; chloroplast	859	859	95%	0.0	93%	DQ251118.1	840,92	92,5%
Select seq gb|KF513555.1|	Meconopsis wilsonii voucher 20100701056 tRNA-Leu (trnL) gene, partial sequence; and trnL-trnF intergenic spacer, complete sequence; chloroplast	686	686	76%	0.0	93%	KF513555.1	839,45	92,3%
Select seq gb|JX087749.1|	Meconopsis lancifolia voucher GH:Boufford 33308 tRNA-Leu (trnL) gene and trnL-trnF intergenic spacer, partial sequence; chloroplast	686	686	76%	0.0	93%	JX087749.1	839,45	92,3%
Select seq gb|JX087740.1|	Meconopsis wilsonii voucher GH:Boufford 32733 tRNA-Leu (trnL) gene and trnL-trnF intergenic spacer, partial sequence; chloroplast	686	686	76%	0.0	93%	JX087740.1	839,45	92,3%
Select seq gb|AY328228.1|	Meconopsis napaulensis specimen-voucher Gong 20020611 tRNA-Leu (trnL) gene and trnL-trnF intergenic spacer, complete sequence; and tRNA-Phe (trnF) gene, partial sequence; chloroplast	686	686	76%	0.0	93%	AY328228.1	839,45	92,3%
Select seq gb|DQ251121.1|	Papaver dubium voucher 2000.0024 tRNA-Leu (trnL) gene, partial sequence; trnL-trnF intergenic spacer, complete sequence; and tRNA-Phe (trnF) gene, partial sequence; chloroplast	854	854	95%	0.0	93%	DQ251121.1	836,02	92,0%
Select seq gb|DQ251141.1|	Papaver orientale voucher 2000.0135 tRNA-Leu (trnL) gene, partial sequence; trnL-trnF intergenic spacer, complete sequence; and tRNA-Phe (trnF) gene, partial sequence; chloroplast	852	852	95%	0.0	93%	DQ251141.1	834,06	91,8%
Select seq gb|DQ251166.1|	Papaver atlanticum voucher 2000.0615 tRNA-Leu (trnL) gene, partial sequence; trnL-trnF intergenic spacer, complete sequence; and tRNA-Phe (trnF) gene, partial sequence; chloroplast	843	843	94%	0.0	93%	DQ251166.1	834,03	91,8%
Select seq gb|DQ251154.1|	Papaver atlanticum voucher 2000.0156 tRNA-Leu (trnL) gene, partial sequence; trnL-trnF intergenic spacer, complete sequence; and tRNA-Phe (trnF) gene, partial sequence; chloroplast	843	843	94%	0.0	93%	DQ251154.1	834,03	91,8%
Select seq gb|KU204905.1|	Papaver somniferum chloroplast, complete genome	848	848	95%	0.0	93%	KU204905.1	830,15	91,3%
Select seq gb|DQ251133.1|	Papaver somniferum voucher 2000.0068 tRNA-Leu (trnL) gene, partial sequence; trnL-trnF intergenic spacer, complete sequence; and tRNA-Phe (trnF) gene, partial sequence; chloroplast	848	848	95%	0.0	93%	DQ251133.1	830,15	91,3%
Select seq gb|DQ251132.1|	Papaver somniferum voucher 2000.0052 tRNA-Leu (trnL) gene, partial sequence; trnL-trnF intergenic spacer, complete sequence; and tRNA-Phe (trnF) gene, partial sequence; chloroplast	848	848	95%	0.0	93%	DQ251132.1	830,15	91,3%

Select seq gb|DQ251144.1|	Papaver atlanticum voucher 2000.0472 tRNA-Leu (trnL) gene, partial sequence; trnL-trnF intergenic spacer, complete sequence; and tRNA-Phe (trnF) gene, partial sequence; chloroplast	837	837	94% 0.0	93% DQ251144.1	828,10	91,1%
Select seq gb|JX087750.1|	Meconopsis lancifolia voucher TEX:W.Xiao 080621-1 tRNA-Leu (trnL) gene and trnL-trnF intergenic spacer, partial sequence; chloroplast	676	676	76% 0.0	93% JX087750.1	827,21	91,0%
Select seq gb|JX087782.1|	Meconopsis sp. Xiao 090705-1 tRNA-Leu (trnL) gene and trnL-trnF intergenic spacer, partial sequence; chloroplast	675	675	76% 0.0	93% JX087782.1	825,99	90,9%
Select seq gb|JX087746.1|	Meconopsis lancifolia voucher E:ACE 568 tRNA-Leu (trnL) gene and trnL-trnF intergenic spacer, partial sequence; chloroplast	675	675	76% 0.0	93% JX087746.1	825,99	90,9%
Select seq gb|AY328213.1|	Meconopsis impedita specimen-voucher Yuan 2000667 tRNA-Leu (trnL) gene and trnL-trnF intergenic spacer, complete sequence; and tRNA-Phe (trnF) gene, partial sequence; chloroplast	675	675	76% 0.0	93% AY328213.1	825,99	90,9%
Select seq gb|AY328212.1|	Meconopsis lancifolia specimen-voucher Yuan 2000657 tRNA-Leu (trnL) gene and trnL-trnF intergenic spacer, complete sequence; and tRNA-Phe (trnF) gene, partial sequence; chloroplast	675	675	76% 0.0	93% AY328212.1	825,99	90,9%
Select seq gb|DQ251157.1|	Papaver somniferum voucher 2000.0142 tRNA-Leu (trnL) gene, partial sequence; trnL-trnF intergenic spacer, complete sequence; and tRNA-Phe (trnF) gene, partial sequence; chloroplast	843	843	95% 0.0	93% DQ251157.1	825,25	90,8%
Select seq gb|DQ251155.1|	Papaver somniferum voucher 2000.0166 tRNA-Leu (trnL) gene, partial sequence; trnL-trnF intergenic spacer, complete sequence; and tRNA-Phe (trnF) gene, partial sequence; chloroplast	843	843	95% 0.0	93% DQ251155.1	825,25	90,8%
Select seq gb|DQ251122.1|	Papaver dubium voucher 2000.0610 tRNA-Leu (trnL) gene, partial sequence; trnL-trnF intergenic spacer, complete sequence; and tRNA-Phe (trnF) gene, partial sequence; chloroplast	824	824	93% 0.0	93% DQ251122.1	824,00	90,6%
Select seq gb|DQ251158.1|	Papaver atlanticum voucher 2000.0603 tRNA-Leu (trnL) gene, partial sequence; trnL-trnF intergenic spacer, complete sequence; and tRNA-Phe (trnF) gene, partial sequence; chloroplast	832	832	94% 0.0	93% DQ251158.1	823,15	90,6%
Select seq gb|DQ251139.1|	Papaver pseudo-orientale voucher 2000.0039 tRNA-Leu (trnL) gene, partial sequence; trnL-trnF intergenic spacer, complete sequence; and tRNA-Phe (trnF) gene, partial sequence; chloroplast	835	835	95% 0.0	93% DQ251139.1	817,42	89,9%
Select seq gb|DQ251159.1|	Papaver glaucum voucher 2000.0189 tRNA-Leu (trnL) gene, partial sequence; trnL-trnF intergenic spacer, complete sequence; and tRNA-Phe (trnF) gene, partial sequence; chloroplast	817	817	93% 0.0	93% DQ251159.1	817,00	89,9%
Select seq gb|DQ251140.1|	Papaver orientale voucher 2000.0035 tRNA-Leu (trnL) gene, partial sequence; trnL-trnF intergenic spacer, complete sequence; and tRNA-Phe (trnF) gene, partial sequence; chloroplast	830	830	95% 0.0	93% DQ251140.1	812,53	89,4%
Select seq gb|KF513556.1|	Meconopsis zangnanensis voucher 20090704008 tRNA-Leu (trnL) gene, partial sequence; and trnL-trnF intergenic spacer, complete sequence; chloroplast	665	665	76% 0.0	92% KF513556.1	805,00	88,6%
Select seq gb|JX087778.1|	Meconopsis argemonantha voucher E:Bowes Lyon 11101 tRNA-Leu (trnL) gene and trnL-trnF intergenic spacer, partial sequence; chloroplast	664	664	76% 0.0	92% JX087778.1	803,79	88,4%
Select seq gb|KM044487.1|	Meconopsis pseudohorridula voucher Tibet-64 tRNA-Leu (trnL) gene and trnL-trnF intergenic spacer, partial sequence; chloroplast	662	662	76% 0.0	92% KM044487.1	801,37	88,2%
Select seq gb|JX087781.1|	Meconopsis speciosa voucher TEX:W.Xiao 090703-2 tRNA-Leu (trnL) gene and trnL-trnF intergenic spacer, partial sequence; chloroplast	662	662	76% 0.0	92% JX087781.1	801,37	88,2%
Select seq gb|AY328220.1|	Meconopsis speciosa specimen-voucher Xie 199810 tRNA-Leu (trnL) gene and trnL-trnF intergenic spacer, complete sequence; and tRNA-Phe (trnF) gene, partial sequence; chloroplast	662	662	76% 0.0	92% AY328220.1	801,37	88,2%
Select seq gb|AY328218.1|	Meconopsis bella specimen-voucher E00045403 tRNA-Leu (trnL) gene and trnL-trnF intergenic spacer, complete sequence; and tRNA-Phe (trnF) gene, partial sequence; chloroplast	660	660	76% 0.0	92% AY328218.1	798,95	87,9%
Select seq gb|DQ251172.1|	Papaver pilosum voucher 2000.0768 tRNA-Leu (trnL) gene, partial sequence; trnL-trnF intergenic spacer, complete sequence; and tRNA-Phe (trnF) gene, partial sequence; chloroplast	824	824	95% 0.0	92% DQ251172.1	797,98	87,8%
Select seq gb|KM044489.1|	Meconopsis pseudohorridula voucher Tibet-97 tRNA-Leu (trnL) gene and trnL-trnF intergenic spacer, partial sequence; chloroplast	656	656	76% 0.0	92% KM044489.1	794,11	87,4%
Select seq gb|FJ626566.1|	Papaver rhoeas tRNA-Leu (trnL) gene and trnL-trnF intergenic spacer, complete sequence; and tRNA-Phe (trnF) gene, partial sequence; chloroplast	802	802	93% 0.0	92% FJ626566.1	793,38	87,3%
Select seq gb|DQ251123.1|	Papaver rhoeas voucher 2000.0090 tRNA-Leu (trnL) gene, partial sequence; trnL-trnF intergenic spacer, complete sequence; and tRNA-Phe (trnF) gene, partial sequence; chloroplast	802	802	93% 0.0	92% DQ251123.1	793,38	87,3%
Select seq gb|DQ251171.1|	Papaver pilosum voucher 2000.0182 tRNA-Leu (trnL) gene, partial sequence; trnL-trnF intergenic spacer, complete sequence; and tRNA-Phe (trnF) gene, partial sequence; chloroplast	819	819	95% 0.0	92% DQ251171.1	793,14	87,3%
Select seq gb|JQ798376.1|	Meconopsis simplicifolia voucher Sejilashan2 tRNA-Leu (trnL) gene and trnL-trnF intergenic spacer, partial sequence; chloroplast	654	654	76% 0.0	92% JQ798376.1	791,68	87,1%
Select seq gb|JQ798375.1|	Meconopsis simplicifolia voucher Sejilashan1 tRNA-Leu (trnL) gene and trnL-trnF intergenic spacer, partial sequence; chloroplast	654	654	76% 0.0	92% JQ798375.1	791,68	87,1%
Select seq gb|AY328235.1|	Meconopsis grandis specimen-voucher Yuan 200271110 tRNA-Leu (trnL) gene and trnL-trnF intergenic spacer, complete sequence; and tRNA-Phe (trnF) gene, partial sequence; chloroplast	654	654	76% 0.0	92% AY328235.1	791,68	87,1%
Select seq gb|DQ251164.1|	Papaver commutatum voucher 2000.0605 tRNA-Leu (trnL) gene, partial sequence; trnL-trnF intergenic spacer, complete sequence; and tRNA-Phe (trnF) gene, partial sequence; chloroplast	797	797	93% 0.0	92% DQ251164.1	788,43	86,7%

Select seq gb|DQ251173.1|	Papaver dubium voucher 2000.0606 tRNA-Leu (trnL) gene, partial sequence; trnL-trnF intergenic spacer, complete sequence; and tRNA-Phe (trnF) gene, partial sequence; chloroplast	813	813	95%	0.0	92%	DQ251173.1	787,33	86,6%
Select seq gb|DQ251128.1|	Meconopsis cambrica voucher 2000.0001 tRNA-Leu (trnL) gene, partial sequence; trnL-trnF intergenic spacer, complete sequence; and tRNA-Phe (trnF) gene, partial sequence; chloroplast	813	813	95%	0.0	92%	DQ251128.1	787,33	86,6%
Select seq gb|DQ251145.1|	Papaver armeniacum voucher 2000.0107 tRNA-Leu (trnL) gene, partial sequence; trnL-trnF intergenic spacer, complete sequence; and tRNA-Phe (trnF) gene, partial sequence; chloroplast	808	808	95%	0.0	92%	DQ251145.1	782,48	86,1%
Select seq gb|AY328243.1|	Meconopsis cambrica specimen-voucher Yuan 200271106 tRNA-Leu (trnL) gene and trnL-trnF intergenic spacer, complete sequence; and tRNA-Phe (trnF) gene, partial sequence; chloroplast	808	808	95%	0.0	92%	AY328243.1	782,48	86,1%
Select seq gb|DQ251156.1|	Papaver somniferum voucher 2000.0063 tRNA-Leu (trnL) gene, partial sequence; trnL-trnF intergenic spacer, complete sequence; and tRNA-Phe (trnF) gene, partial sequence; chloroplast	802	802	95%	0.0	92%	DQ251156.1	776,67	85,4%
Select seq gb|AY328240.1|	Papaver rhoeas specimen-voucher Gong 2003030701 tRNA-Leu (trnL) gene and trnL-trnF intergenic spacer, complete sequence; and tRNA-Phe (trnF) gene, partial sequence; chloroplast	787	787	93%	0.0	91%	AY328240.1	770,08	84,7%
Select seq gb|DQ251130.1|	Papaver somniferum voucher 2000.0016 tRNA-Leu (trnL) gene, partial sequence; trnL-trnF intergenic spacer, complete sequence; and tRNA-Phe (trnF) gene, partial sequence; chloroplast	797	797	95%	0.0	91%	DQ251130.1	763,44	84,0%
Select seq gb|DQ251129.1|	Meconopsis cambrica voucher 1992.0611 tRNA-Leu (trnL) gene, partial sequence; trnL-trnF intergenic spacer, complete sequence; and tRNA-Phe (trnF) gene, partial sequence; chloroplast	797	797	95%	0.0	91%	DQ251129.1	763,44	84,0%
Select seq gb|DQ251161.1|	Papaver glaucum voucher 2000.0609 tRNA-Leu (trnL) gene, partial sequence; trnL-trnF intergenic spacer, complete sequence; and tRNA-Phe (trnF) gene, partial sequence; chloroplast	778	778	93%	0.0	91%	DQ251161.1	761,27	83,7%
Select seq gb|DQ251131.1|	Papaver somniferum voucher 2000.0130 tRNA-Leu (trnL) gene, partial sequence; trnL-trnF intergenic spacer, complete sequence; and tRNA-Phe (trnF) gene, partial sequence; chloroplast	791	791	95%	0.0	91%	DQ251131.1	757,69	83,4%
Select seq gb|DQ251160.1|	Papaver glaucum voucher 2000.0177 tRNA-Leu (trnL) gene, partial sequence; trnL-trnF intergenic spacer, complete sequence; and tRNA-Phe (trnF) gene, partial sequence; chloroplast	773	773	93%	0.0	91%	DQ251160.1	756,38	83,2%
Select seq gb|JX087752.1|	Papaver sp. WX-2012 tRNA-Leu (trnL) gene and trnL-trnF intergenic spacer, partial sequence; chloroplast	780	780	95%	0.0	91%	JX087752.1	747,16	82,2%
Select seq gb|DQ251126.1|	Papaver macrostomum voucher RBGE 34139 tRNA-Leu (trnL) gene, partial sequence; trnL-trnF intergenic spacer, complete sequence; and tRNA-Phe (trnF) gene, partial sequence; chloroplast	756	756	93%	0.0	91%	DQ251126.1	739,74	81,4%
Select seq gb|DQ251124.1|	Papaver rhoeas voucher KJ93/7 Kadereit et al., 1996 tRNA-Leu (trnL) gene, partial sequence; trnL-trnF intergenic spacer, complete sequence; and tRNA-Phe (trnF) gene, partial sequence; chloroplast	756	756	93%	0.0	91%	DQ251124.1	739,74	81,4%
Select seq gb|DQ251146.1|	Stylomecon heterophylla voucher 2000.0183 tRNA-Leu (trnL) gene, partial sequence; trnL-trnF intergenic spacer, complete sequence; and tRNA-Phe (trnF) gene, partial sequence; chloroplast	763	763	95%	0.0	90%	DQ251146.1	722,84	79,5%
Select seq gb|AY328244.1|	Papaver spicatum specimen-voucher Yuan 200271101 tRNA-Leu (trnL) gene and trnL-trnF intergenic spacer, complete sequence; and tRNA-Phe (trnF) gene, partial sequence; chloroplast	726	726	95%	0.0	89%	AY328244.1	680,15	74,8%
Select seq gb|DQ251119.1|	Papaver alpinum voucher 2000.0568 tRNA-Leu (trnL) gene, partial sequence; trnL-trnF intergenic spacer, complete sequence; and tRNA-Phe (trnF) gene, partial sequence; chloroplast	725	725	95%	0.0	89%	DQ251119.1	679,21	74,7%
Select seq gb|DQ251114.1|	Papaver nudicaule var. aquilegioides voucher 2000.1321 tRNA-Leu (trnL) gene, partial sequence; trnL-trnF intergenic spacer, complete sequence; and tRNA-Phe (trnF) gene, partial sequence; chloroplast	719	719	95%	0.0	89%	DQ251114.1	673,59	74,1%
Select seq gb|DQ251109.1|	Papaver nudicaule voucher 1999.00340 tRNA-Leu (trnL) gene, partial sequence; trnL-trnF intergenic spacer, complete sequence; and tRNA-Phe (trnF) gene, partial sequence; chloroplast	719	719	95%	0.0	89%	DQ251109.1	673,59	74,1%
Select seq gb|KF513558.1|	Papaver radicatum voucher cb-rad-001 tRNA-Leu (trnL) gene, partial sequence; and trnL-trnF intergenic spacer, complete sequence; chloroplast	713	713	95%	0.0	89%	KF513558.1	667,97	73,5%
Select seq gb|KF513557.1|	Papaver nudicaule voucher bj-nud-001 tRNA-Leu (trnL) gene, partial sequence; and trnL-trnF intergenic spacer, complete sequence; chloroplast	713	713	95%	0.0	89%	KF513557.1	667,97	73,5%
Select seq gb|JX087766.1|	Papaver alpinum voucher TEX:W.Xiao 090527-2 tRNA-Leu (trnL) gene and trnL-trnF intergenic spacer, partial sequence; chloroplast	713	713	95%	0.0	89%	JX087766.1	667,97	73,5%
Select seq gb|DQ251135.1|	Papaver nudicaule voucher AS 95/23 Kadereit et al., 1996 tRNA-Leu (trnL) gene, partial sequence; trnL-trnF intergenic spacer, complete sequence; and tRNA-Phe (trnF) gene, partial sequence; chloroplast	713	713	95%	0.0	89%	DQ251135.1	667,97	73,5%
Select seq gb|DQ251127.1|	Papaver nudicaule voucher 1999.0339 tRNA-Leu (trnL) gene, partial sequence; trnL-trnF intergenic spacer, complete sequence; and tRNA-Phe (trnF) gene, partial sequence; chloroplast	713	713	95%	0.0	89%	DQ251127.1	667,97	73,5%
Select seq gb|DQ251115.1|	Papaver nudicaule var. aquilegioides voucher 2000.1806 tRNA-Leu (trnL) gene, partial sequence; trnL-trnF intergenic spacer, complete sequence; and tRNA-Phe (trnF) gene, partial sequence; chloroplast	713	713	95%	0.0	89%	DQ251115.1	667,97	73,5%
Select seq gb|DQ251113.1|	Papaver radicatum voucher 2000.0769 tRNA-Leu (trnL) gene, partial sequence; trnL-trnF intergenic spacer, complete sequence; and tRNA-Phe (trnF) gene, partial sequence; chloroplast	713	713	95%	0.0	89%	DQ251113.1	667,97	73,5%
Select seq gb|DQ251111.1|	Papaver nudicaule voucher 2000.0598 tRNA-Leu (trnL) gene, partial sequence; trnL-trnF intergenic spacer, complete sequence; and tRNA-Phe (trnF) gene, partial sequence; chloroplast	713	713	95%	0.0	89%	DQ251111.1	667,97	73,5%
Select seq gb|DQ251108.1|	Papaver nudicaule voucher 2000.0104 tRNA-Leu (trnL) gene, partial sequence; trnL-trnF intergenic spacer, complete sequence; and tRNA-Phe (trnF) gene, partial sequence; chloroplast	713	713	95%	0.0	89%	DQ251108.1	667,97	73,5%

Select seq gb|DQ251167.1|	Papaver aculeatum voucher 2000.0659 tRNA-Leu (trnL) gene, partial sequence; trnL-trnF intergenic spacer, complete sequence; and tRNA-Phe (trnF) gene, partial sequence; chloroplast	712	712	95%	0.0	89%	DQ251167.1	667,03	73,4%
Select seq gb|KM044498.1|	Papaver pavoninum voucher Xingjiang-56 tRNA-Leu (trnL) gene and trnL-trnF intergenic spacer, partial sequence; chloroplast	652	652	87%	0.0	89%	KM044498.1	666,99	73,4%
Select seq gb|DQ251117.1|	Papaver nudicaule voucher 2000.0653 tRNA-Leu (trnL) gene, partial sequence; trnL-trnF intergenic spacer, complete sequence; and tRNA-Phe (trnF) gene, partial sequence; chloroplast	701	701	95%	0.0	88%	DQ251117.1	649,35	71,4%
Select seq gb|JX087747.1|	Meconopsis wallichii voucher E:Miyamoto 9584100 tRNA-Leu (trnL) gene and trnL-trnF intergenic spacer, partial sequence; chloroplast	688	688	95%	0.0	88%	JX087747.1	637,31	70,1%
Select seq gb|DQ251150.1|	Roemeria refracta voucher 2000.0171 tRNA-Leu (trnL) gene, partial sequence; trnL-trnF intergenic spacer, complete sequence; and tRNA-Phe (trnF) gene, partial sequence; chloroplast	682	682	95%	0.0	88%	DQ251150.1	631,75	69,5%
Select seq gb|AY328226.1|	Meconopsis latifolia specimen-voucher Stewart 22563a tRNA-Leu (trnL) gene and trnL-trnF intergenic spacer, complete sequence; and tRNA-Phe (trnF) gene, partial sequence; chloroplast	682	682	95%	0.0	88%	AY328226.1	631,75	69,5%
Select seq gb|AY328227.1|	Meconopsis aculeata specimen-voucher NA 2091890 tRNA-Leu (trnL) gene and trnL-trnF intergenic spacer, complete sequence; and tRNA-Phe (trnF) gene, partial sequence; chloroplast	669	669	94%	0.0	88%	AY328227.1	626,30	68,9%
Select seq gb|JX087780.1|	Meconopsis wallichii voucher TEX:W.Xiao 090522 tRNA-Leu (trnL) gene and trnL-trnF intergenic spacer, partial sequence; chloroplast	664	664	95%	0.0	87%	JX087780.1	608,08	66,9%
Select seq gb|DQ251116.1|	Papaver nudicaule voucher 2000.0186 tRNA-Leu (trnL) gene, partial sequence; trnL-trnF intergenic spacer, complete sequence; and tRNA-Phe (trnF) gene, partial sequence; chloroplast	662	662	95%	0.0	87%	DQ251116.1	606,25	66,7%
Select seq gb|AY328234.1|	Meconopsis x sheldonii specimen-voucher Yuan 200271112 tRNA-Leu (trnL) gene and trnL-trnF intergenic spacer, complete sequence; and tRNA-Phe (trnF) gene, partial sequence; chloroplast	662	662	95%	0.0	87%	AY328234.1	606,25	66,7%
Select seq gb|KF513554.1|	Meconopsis simplicifolia voucher 20090704005 tRNA-Leu (trnL) gene, partial sequence; and trnL-trnF intergenic spacer, complete sequence; chloroplast	660	660	95%	0.0	87%	KF513554.1	604,42	66,5%
Select seq gb|JX087751.1|	Meconopsis simplicifolia voucher Egan 4 tRNA-Leu (trnL) gene and trnL-trnF intergenic spacer, partial sequence; chloroplast	656	656	95%	0.0	87%	JX087751.1	600,76	66,1%
Select seq gb|JQ798379.1|	Meconopsis betonicifolia voucher Milin1 tRNA-Leu (trnL) gene and trnL-trnF intergenic spacer, partial sequence; chloroplast	656	656	95%	0.0	87%	JQ798379.1	600,76	66,1%
Select seq gb|JQ798374.1|	Meconopsis simplicifolia voucher Cuona tRNA-Leu (trnL) gene and trnL-trnF intergenic spacer, partial sequence; chloroplast	656	656	95%	0.0	87%	JQ798374.1	600,76	66,1%
Select seq gb|DQ251174.1|	Meconopsis betonicifolia voucher 1998.0451 tRNA-Leu (trnL) gene, partial sequence; trnL-trnF intergenic spacer, complete sequence; and tRNA-Phe (trnF) gene, partial sequence; chloroplast	656	656	95%	0.0	87%	DQ251174.1	600,76	66,1%
Select seq gb|AY328230.1|	Meconopsis simplicifolia specimen-voucher Miyamota 9420218 tRNA-Leu (trnL) gene and trnL-trnF intergenic spacer, complete sequence; and tRNA-Phe (trnF) gene, partial sequence; chloroplast	656	656	95%	0.0	87%	AY328230.1	600,76	66,1%
Select seq gb|JQ798333.1|	Meconopsis integrifolia isolate H12 tRNA-Leu (trnL) gene and trnL-trnF intergenic spacer, partial sequence; chloroplast	654	654	95%	0.0	87%	JQ798333.1	598,93	

Select for downloading or viewing reports	Kh108_trnL Description	Max score	Total score	Query cover	E value	Ident	Accession	(Ident/Cover)* Max score	Deviation from top hit
Select seq gb|JQ044780.1|	Teucrium polium subsp. polium isolate TP12 tRNA-Leu (trnL) gene, partial sequence; trnL-trnF intergenic spacer, complete sequence; and tRNA-Phe (trnF) gene, partial sequence; chloroplast	1149	1149	99%	0.0	99%	JQ044780.1	1149,00	100,0%
Select seq gb|JQ044779.1|	Teucrium polium subsp. polium isolate TP11 tRNA-Leu (trnL) gene, partial sequence; trnL-trnF intergenic spacer, complete sequence; and tRNA-Phe (trnF) gene, partial sequence; chloroplast	1149	1149	99%	0.0	99%	JQ044779.1	1149,00	100,0%
Select seq gb|JQ044778.1|	Teucrium polium subsp. polium isolate TP10 tRNA-Leu (trnL) gene, partial sequence; trnL-trnF intergenic spacer, complete sequence; and tRNA-Phe (trnF) gene, partial sequence; chloroplast	1149	1149	99%	0.0	99%	JQ044778.1	1149,00	100,0%
Select seq gb|JN408660.1|	Teucrium pyrenaicum tRNA-Leu (trnL) gene, partial sequence; and trnL-trnF intergenic spacer, complete sequence; chloroplast	1114	1114	96%	0.0	99%	JN408660.1	1148,81	100,0%
Select seq gb|JN408625.1|	Teucrium capitatum tRNA-Leu (trnL) gene, partial sequence; and trnL-trnF intergenic spacer, complete sequence; chloroplast	1077	1077	93%	0.0	99%	JN408625.1	1146,48	99,8%
Select seq gb|JN408624.1|	Teucrium capitatum tRNA-Leu (trnL) gene, partial sequence; and trnL-trnF intergenic spacer, complete sequence; chloroplast	1077	1077	93%	0.0	99%	JN408624.1	1146,48	99,8%
[truncated: 621,737 more chars]
