# Supplementary material for: Unidentifiable by morphology: DNA barcoding of plant material in local markets in Iran
Source: PLoS One. 2017 Apr 18;12(4):e0175722. doi: 10.1371/journal.pone.0175722 (PMC5395179; doi:10.1371/journal.pone.0175722)
Supplement: S5 Table — Identifications are assigned based on a combination of the identity score (High identity: i ≥ 95%; Medium identity: 90% ≤ i < 95%; Low identity: i < 90%) and the number of species within 1% deviation of the calculated similarity score. (PDF) [file pone.0175722.s005.pdf]

S5 Table. Optimized BLAST similarity identifications per sample. Identifications are assigned based on a combination of the identity score (High identity: $i \geq 95\%$; Medium identity: $90\% \leq i < 95\%$; Low identity: $i < 90\%$) and the number of species within 1% deviation of the calculated similarity score.

Sample	Marker	Identity	1% top hits	Confidence	Identification
Kh001	ITS	96	5	High identity (i>95%). 1 species within 1% deviation of top hit. Species-level confidence.	Cuminum cyminum
Kh001	trnL-F	100	1	High identity (i>95%). 1 species within 1% deviation of top hit. Species-level confidence.	Osmorhiza bipatriata
Kh003	ITS	99	3	High identity (i>95%). 3 species within 1% deviation of top hit. Genus-level confidence.	Nepeta sp.
Kh003	trnL-F	99	1	High identity (i>95%). 1 species within 1% deviation of top hit. Species-level confidence.	Nepeta racemosa
Kh004	ITS	91	2	Medium identity (90%<i<95%). 2 species within 1% deviation of top hit. Genus-level confidence.	Satureja sp.
Kh004	trnL-F	99	2	High identity (i>95%). 2 species within 1% deviation of top hit. Genus-level confidence.	Satureja sp.
Kh007	ITS	92	1	Medium identity (90%<i<95%). 1 species within 1% deviation of top hit. Genus-level confidence.	Echium sp.
Kh007	trnL-F	92	1	Medium identity (90%<i<95%). 1 species within 1% deviation of top hit. Genus-level confidence.	Echium sp.
Kh008	ITS	92	3	Medium identity (90%<i<95%). 3 species within 1% deviation of top hit. Genus-level confidence.	Thymus sp.
Kh008	trnL-F	99	7	High identity (i>95%). 6 species within 1% deviation of top hit. Genus-level confidence.	Thymus sp.
Kh009	ITS	98	0	High identity (i>95%). Query coverage < 70% No identification.	Unidentified
Kh009	trnL-F	100	0	High identity (i>95%). Query coverage < 70% No identification.	Unidentified
Kh010	trnL-F	99	1	High identity (i>95%). 1 species within 1% deviation of top hit. Species-level confidence.	Hymenocrater bituminosus
Kh011	ITS	94	1	Medium identity (90%<i<95%). 1 species within 1% deviation of top hit. Genus-level confidence.	Anthemis sp.
Kh011	trnL-F	99	2	High identity (i>95%). 2 species within 1% deviation of top hit. Genus-level confidence.	Artemisia sp.
Kh012	ITS	95	1	High identity (i>95%). 1 species within 1% deviation of top hit. Species-level confidence.	Althaea cannabina
Kh016	ITS	100	3	High identity (i>95%). 3 species within 1% deviation of top hit. Genus-level confidence.	Thymus sp.
Kh016	trnL-F	99	7	High identity (i>95%). 7 species within 1% deviation of top hit. Genus-level confidence.	Thymus sp.
Kh017	ITS	98	2	High identity (i>95%). 2 species within 1% deviation of top hit. Genus-level confidence.	Achillea sp.
Kh017	trnL-F	99	16	High identity (i>95%). 14 species within 1% deviation of top hit. Genus-level confidence.	Achillea sp.
Kh018	ITS	89	5	Low identity (i<90%). 5 species within 1% deviation of top hit. Family-level confidence	Lamiaceae
Kh018	trnL-F	98	5	High identity (i>95%). 5 species (2 genera) within 1% deviation of top hit. Family-level confidence	Lamiaceae
Kh019	trnL-F	99	17	High identity (i>95%). 9 species within 1% deviation of top hit. Genus-level confidence.	Teucrium sp.
Kh020	trnL-F	100	17	High identity (i>95%). 15 species (2 genera) within 1% deviation of top hit. Family-level confidence	Lamiaceae
Kh021	ITS	100	1	High identity (i>95%). 1 species within 1% deviation of top hit. Species-level confidence.	Althaea armeniaca
Kh023	trnL-F	94	3	Medium identity (90%<i<95%). 3 species within 1% deviation of top hit. Genus-level confidence.	Teucrium sp.
Kh024	ITS	100	3	High identity (i>95%). 3 species within 1% deviation of top hit. Genus-level confidence.	Thymus sp.
kh024	trnL-F	99	11	High identity (i>95%). 11 species within 1% deviation of top hit. Genus-level confidence.	Thymus sp.
Kh025	ITS	100	2	High identity (i>95%). 2 species within 1% deviation of top hit. Genus-level confidence.	Carthamus sp.
Kh028	ITS	100	1	High identity (i>95%). 1 species within 1% deviation of top hit. Species-level confidence.	Hypericum scabrum
Kh028	trnL-F	97	0	High identity (i>95%). Query coverage < 70% No identification.	Unidentified
Kh031	ITS	90	0	Medium identity (90%<i<95%). Query coverage < 70% No identification.	Unidentified
Kh031	trnL-F	100	2	High identity (i>95%). 1 species within 1% deviation of top hit. Species-level confidence.	Physalis alkekengi
Kh032	trnL-F	99	6	High identity (i>95%). 4 species within 1% deviation of top hit. Genus-level confidence.	Ephedra sp.
kh033	trnL-F	99	0	High identity (i>95%). Query coverage < 70% No identification.	Unidentified
Kh034	trnL-F	100	2	High identity (i>95%). 2 species within 1% deviation of top hit. Genus-level confidence.	Fraxinus sp.
Kh036	ITS	99	4	High identity (i>95%). 1 species within 1% deviation of top hit. Species-level confidence.	Viola alba
Kh036	trnL-F	99	8	High identity (i>95%). 5 species within 1% deviation of top hit. Genus-level confidence.	Viola sp.
Kh038	ITS	100	1	High identity (i>95%). 1 species within 1% deviation of top hit. Species-level confidence.	Cuminum cyminum
Kh039	ITS	89	2	Low identity (i<90%). 2 species within 1% deviation of top hit. Family-level confidence	Plantaginaceae
Kh039	trnL-F	99	2	High identity (i>95%). 2 species within 1% deviation of top hit. Genus-level confidence.	Plantago sp.
Kh044	ITS	99	4	High identity (i>95%). 3 species within 1% deviation of top hit. Genus-level confidence.	Berberis sp.
Kh045	ITS	93	1	Medium identity (90%<i<95%). 1 species within 1% deviation of top hit. Genus-level confidence.	Bunium sp.
Kh045	trnL-F	80	1	Low identity (i<90%). 1 species within 1% deviation of top hit. Family-level confidence	Apiaceae
Kh048	ITS	98	1	High identity (i>95%). 1 species within 1% deviation of top hit. Species-level confidence.	Zosima absinthifolia
kh048	trnL-F	99	1	High identity (i>95%). 1 species within 1% deviation of top hit. Species-level confidence.	Zosima absinthifolia
Kh051	trnL-F	98	1	High identity (i>95%). 1 species within 1% deviation of top hit. Species-level confidence.	Hymenocrater bituminosus
Kh053	ITS	84	1	Low identity (i<90%). 1 species within 1% deviation of top hit. Family-level confidence	Lamiaceae
Kh053	trnL-F	100	1	High identity (i>95%). 1 species within 1% deviation of top hit. Species-level confidence.	Mentha arvensis
Kh055	trnL-F	97	1	High identity (i>95%). 1 species within 1% deviation of top hit. Species-level confidence.	Nepeta menthoides
Kh056	ITS	98	1	High identity (i>95%). 1 species within 1% deviation of top hit. Species-level confidence.	Allium atroviolaceum
Kh056	trnL-F	98	5	High identity (i>95%). 5 species within 1% deviation of top hit. Genus-level confidence.	Allium sp.
Kh057	ITS	100	3	High identity (i>95%). 3 species within 1% deviation of top hit. Genus-level confidence.	Thymus sp.
Kh057	trnL-F	76	7	Low identity (i<90%). 7 species within 1% deviation of top hit. Family-level confidence	Lamiaceae
Kh058	ITS	99	2	High identity (i>95%). 2 species within 1% deviation of top hit. Genus-level confidence.	Achillea sp.
Kh058	trnL-F	99	33	High identity (i>95%). 32 species within 1% deviation of top hit. Genus-level confidence.	Achillea sp.
Kh059	ITS	95	1	High identity (i>95%). 1 species within 1% deviation of top hit. Species-level confidence.	Alcea acaulis
Kh062	trnL-F	99	6	High identity (i>95%). 4 species within 1% deviation of top hit. Genus-level confidence.	Mentha sp.
Kh063	ITS	97	1	High identity (i>95%). 1 species within 1% deviation of top hit. Species-level confidence.	Tanacetum turcomanicum
Kh063	trnL-F	98	1	High identity (i>95%). 1 species within 1% deviation of top hit. Species-level confidence.	Artemisia lagocephala
Kh065	trnL-F	99	11	High identity (i>95%). 11 species within 1% deviation of top hit. Genus-level confidence.	Thymus sp.
Kh066	trnL-F	99	17	High identity (i>95%). 10 species within 1% deviation of top hit. Genus-level confidence.	Teucrium sp.
Kh067	trnL-F	99	2	High identity (i>95%). 2 species within 1% deviation of top hit. Family-level confidence	Lamiaceae
Kh068	ITS	99	13	High identity (i>95%). 13 species within 1% deviation of top hit. Genus-level confidence.	Alcea sp.
Kh069	ITS	100	3	High identity (i>95%). 3 species within 1% deviation of top hit. Genus-level confidence.	Thymus sp.
Kh069	trnL-F	99	11	High identity (i>95%). 11 species within 1% deviation of top hit. Genus-level confidence.	Thymus sp.
Kh070	ITS	99	1	High identity (i>95%). 1 species within 1% deviation of top hit. Species-level confidence.	Satureja hortensis
Kh070	trnL-F	99	1	High identity (i>95%). 1 species within 1% deviation of top hit. Species-level confidence.	Satureja hortensis
Kh071	ITS	95	1	High identity (i>95%). 1 species within 1% deviation of top hit. Species-level confidence.	Bunium capillifolium
Kh071	trnL-F	97	4	High identity (i>95%). 4 species within 1% deviation of top hit. Family-level confidence	Apiaceae
Kh073	trnL-F	97	3	High identity (i>95%). 1 species within 1% deviation of top hit. Species-level confidence.	Teucrium polium
Kh074	ITS	90	0	Medium identity (90%<i<95%). Query coverage < 70% No identification.	Unidentified
Kh074	trnL-F	99	1	High identity (i>95%). 1 species within 1% deviation of top hit. Species-level confidence.	Perovskia abrotanoides
Kh075	trnL-F	99	30	High identity (i>95%). 25 species (2 genera) within 1% deviation of top hit. Family-level confidence	Lamiaceae
Kh076	ITS	96	1	High identity (i>95%). 1 species within 1% deviation of top hit. Species-level confidence.	Tripleurospermum maritimum
Kh076	trnL-F	97	2	High identity (i>95%). 2 species within 1% deviation of top hit. Genus-level confidence.	Artemisia sp.
Kh082	trnL-F	99	1	High identity (i>95%). 1 species within 1% deviation of top hit. Species-level confidence.	Hymenocrater bituminosus
Kh090	ITS	96	4	High identity (i>95%). 4 species within 1% deviation of top hit. Genus-level confidence.	Satureja sp.
Kh090	trnL-F	99	3	High identity (i>95%). 3 species within 1% deviation of top hit. Genus-level confidence.	Satureja sp.
Kh095	ITS	97	3	High identity (i>95%). 3 species within 1% deviation of top hit. Genus-level confidence.	Tanacetum sp.
Kh095	trnL-F	98	1	High identity (i>95%). 1 species within 1% deviation of top hit. Species-level confidence.	Artemisia lagocephala
Kh102	ITS	99	1	High identity (i>95%). 1 species within 1% deviation of top hit. Species-level confidence.	Papaver bracteatum
Kh102	trnL-F	95	2	High identity (i>95%). 2 species within 1% deviation of top hit. Genus-level confidence.	Papaver sp.
Kh108	trnL-F	99	16	High identity (i>95%). 10 species within 1% deviation of top hit. Genus-level confidence.	Teucrium sp.
Kh110	trnL-F	99	1	High identity (i>95%). 1 species within 1% deviation of top hit. Species-level confidence.	Achillea wilhelmsii
Kh111	ITS	99	12	High identity (i>95%). 6 species within 1% deviation of top hit. Genus-level confidence.	Amaranthus sp.
Kh111	trnL-F	94	2	Medium identity (90%<i<95%). 2 species within 1% deviation of top hit. Genus-level confidence.	Amaranthus sp.
Kh112	trnL-F	100	17	High identity (i>95%). 17 species (2 genera) within 1% deviation of top hit. Family-level confidence	Lamiaceae
Kh113	ITS	94	6	Medium identity (90%<i<95%). 4 species within 1% deviation of top hit. Genus-level confidence.	Echium sp.
Kh113	trnL-F	99	1	High identity (i>95%). 1 species within 1% deviation of top hit. Species-level confidence.	Lobostemon fruticosus
Kh114	ITS	99	1	High identity (i>95%). 1 species within 1% deviation of top hit. Species-level confidence.	Alcea koelzii
Kh115	ITS	93	1	Medium identity (90%<i<95%). 1 species within 1% deviation of top hit. Genus-level confidence.	Bunium sp.
Kh115	trnL-F	97	2	High identity (i>95%). 2 species within 1% deviation of top hit. Family-level confidence	Apiaceae
Kh116	ITS	100	2	High identity (i>95%). 2 species within 1% deviation of top hit. Genus-level confidence.	Berberis sp.
Kh116	trnL-F	98	3	High identity (i>95%). 3 species within 1% deviation of top hit. Genus-level confidence.	Berberis sp.
Kh117	ITS	100	3	High identity (i>95%). 1 species within 1% deviation of top hit. Species-level confidence.	Urtica dioica
Kh117	trnL-F	100	1	High identity (i>95%). 1 species within 1% deviation of top hit. Species-level confidence.	Urtica dioica
Kh118	ITS	99	1	High identity (i>95%). 1 species within 1% deviation of top hit. Species-level confidence.	Astragalus commixtus
Kh118	trnL-F	97	4	High identity (i>95%). 4 species within 1% deviation of top hit. Genus-level confidence.	Astragalus sp.
Kh132	ITS	100	2	High identity (i>95%). 2 species within 1% deviation of top hit. Genus-level confidence.	Astragalus sp.
Kh132	trnL-F	100	2	High identity (i>95%). 1 species within 1% deviation of top hit. Species-level confidence.	Astragalus edulis
Kh133	ITS	99	2	High identity (i>95%). 1 species within 1% deviation of top hit. Species-level confidence.	Zataria multiflora
Kh133	trnL-F	100	1	High identity (i>95%). 1 species within 1% deviation of top hit. Species-level confidence.	Pentapleura subulifera
Kh135	trnL-F	95	1	High identity (i>95%). 1 species within 1% deviation of top hit. Species-level confidence.	Pentaplaris doroteae
Kh138	ITS	93	1	Medium identity (90%<i<95%). 1 species within 1% deviation of top hit. Genus-level confidence.	Bunium sp.
Kh138	trnL-F	87	1	Low identity (i<90%). 1 species within 1% deviation of top hit. Family-level confidence	Apiaceae
Kh141	ITS	90	1	Medium identity (90%<i<95%). 1 species within 1% deviation of top hit. Genus-level confidence.	Pimpinella sp.
Kh141	trnL-F	90	1	Medium identity (90%<i<95%). 1 species within 1% deviation of top hit. Genus-level confidence.	Heteromorpha sp.
