# Supplementary material for: Unidentifiable by morphology: DNA barcoding of plant material in local markets in Iran
Source: PLoS One. 2017 Apr 18;12(4):e0175722. doi: 10.1371/journal.pone.0175722 (PMC5395179; doi:10.1371/journal.pone.0175722)
Supplement: S6 Table — (PDF) [file pone.0175722.s006.pdf]

S6 Table. Samples for which family and genus identifications based on DNA barcoding results did not match putative species identifications from the official herbal pharmacopeia.

Vernacular name	Family	Scientific name based on morphology	Identification based on matching vernacular names with literature	Identification by DNA barcoding integrative approach
Gole roomi	Apiaceae	*Prangos* sp.	*Levisticum officinale* W.D. J Koch	*Zosima absinthifolia* Link
Shirin badyan	Apiaceae	Apiaceae	*Foeniculum vulgare* Mill.	*Pimpinella anisum* L.
Zireh siyah	Apiaceae	*Bunium* sp.	*Bunium persicum* (Boiss.) B.Fedtsch.	*Bunium* sp.
Babooneh	Asteraceae	*Anthemis* sp.	*Matricaria recutita* L. *Matricaria* sp.	*Anthemis cotula* L.
Babooneh	Asteraceae	*Tripleurospermum* sp.	*Matricaria recutita* L. *Matricaria* sp.	*Tripleurospermum* sp.
Babooneh	Asteraceae	*Tanacetum* sp.	*Matricaria recutita* L. *Matricaria* sp.	*Tanacetum parthenium* (L.) Sch.Bip.
Gole zoofa	Lamiaceae	*Nepeta* sp.	*Hyssopus officinalis* L.	*Nepeta menthoides* Boiss. & Buhse
Badranjbooyeh	Lamiaceae	*Hymenocrater* sp.	*Dracocephalum moldavica* L., *Asperugo procumbens* L., *Clinopodium nepeta* subsp. *glandulosum* (Req.) Govaerts, *Melissa officinalis* L.	*Hymenocrater bituminosus* Fisch. & C.A.Mey.
Badranjbooyeh	Lamiaceae	*Hymenocrater* sp.	*Dracocephalum moldavica* L., *Asperugo procumbens* L., *Clinopodium nepeta* subsp. *glandulosum* (Req.) Govaerts, *Melissa officinalis* L.	*Hymenocrater bituminosus* Fisch. & C.A.Mey.
Badranjbooyeh	Lamiaceae	*Hymenocrater* sp.	*Dracocephalum moldavica* L., *Asperugo procumbens* L., *Clinopodium nepeta* subsp. *glandulosum* (Req.) Govaerts, *Melissa officinalis* L.	*Hymenocrater bituminosus* Fisch. & C.A.Mey.
Marzanjoosh	Lamiaceae	*Thymus* sp.	*Origanum vulgare* L.	*Thymus serpyllum* L.
Badranjbooyeh	Lamiaceae	*Hymenocrater* sp.	*Dracocephalum moldavica* L., *Asperugo procumbens* L., *Clinopodium nepeta* subsp. *glandulosum* (Req.) Govaerts, *Melissa officinalis* L.	*Hymenocrater bituminosus* Fisch. & C.A.Mey.

Avishan	Lamiaceae	*Thymus* sp.	*Thymus vulgaris* L., *Thymus serpyllum* L.	*Zataria multiflora* Boiss.
Ostokhodoos	Lamiaceae	*Stachys* sp.	*Lavandula* sp.	*Stachys* sp.
Gol kooyid	Lamiaceae	*Perovskia* sp.	Unidentifiable	*Perovskia atriplicifolia* Benth.
Maryam goli	Malvaceae	Genus unknown	*Salvia sclarea* L., *Salvia officinalis* L.	*Althaea cannabina* L.
Charme giyah	Malvaceae	Genus unknown	*Alcea* ssp. (*Alcea lavateriflora* (DC.) Boiss., *Alcea digitata* Alef.)	*Althaea cannabina* L.
Gol khatmi	Malvaceae	*Malva* sp.	*Alcea* ssp. (*Alcea lavateriflora* (DC.) Boiss., *Alcea digitata* Alef.)	*Malva nicaeensis* All.
Kaganj	Solanaceae	*Physalis* sp.	Unidentifiable	*Physalis alkekengi* L.
Marzeh	Urticaceae	Genus unknown	*Satureja laxiflora* C.Koch, *Satureja hortensis* L.	*Urtica dioica* L.
