# Supplementary material for: Unidentifiable by morphology: DNA barcoding of plant material in local markets in Iran
Source: PLoS One. 2017 Apr 18;12(4):e0175722. doi: 10.1371/journal.pone.0175722 (PMC5395179; doi:10.1371/journal.pone.0175722)
Supplement: S1 Text — (PDF) [file pone.0175722.s007.pdf]

S1 Text. An example of the integrative approach advocated in this paper.

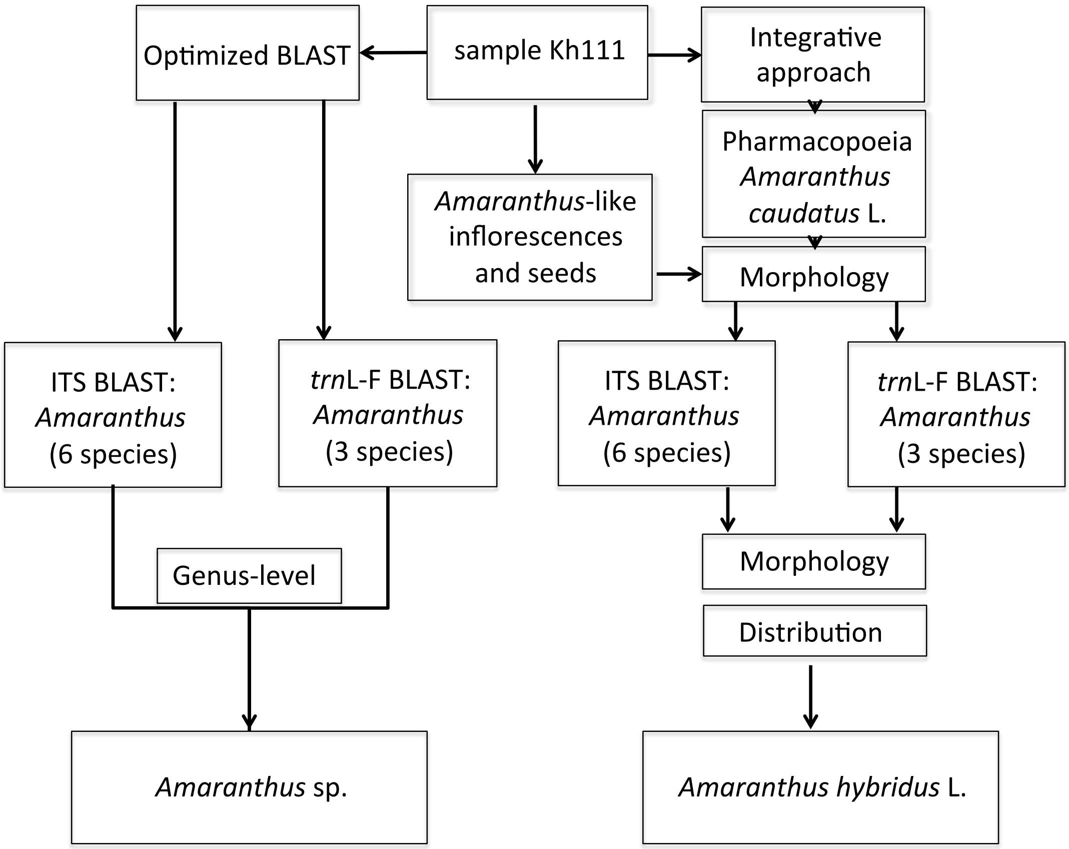

Figure. Diagram comparing the information and choice stream using the optimized BLAST and integrative approach.

A priori data. Sample Kh111 has the vernacular name "Taj khoroos" in Persian. According to the Iranian Herbal Pharmacopoeia (2002) this is the vernacular name for *Amaranthus caudatus* L. On the basis of inflorescence and seed morphology sample Kh111 was identified as a species of *Amaranthus* lacking spiny hairs. Ethnobotanical market data supports that the sample could be an *Amaranthus* species based on described morphology of the living material, growth habit and growing location.

Simple BLAST sequence similarity matching. In simple BLAST-based sequence matching, if there are two or more equally high-scoring hits within the same genus then only a genus level is assigned. All first hits within 10 max score points of each other were considered equally likely. Using the BLAST-based sequence matching approach the top scoring hits for this sample for the ITS amplicon was *Amaranthus hybridus* L. Whereas for trnL-F spacer, the amplicon was identified as *Amaranthus spinosus* L. As there was no consensus for

identification by both markers, it is considered as *Amaranthus* sp. for the combined ITS and trnL identification.

Optimized BLAST sequence similarity matching. For the optimized method a similarity score was calculated for each of the 100 BLAST hits if the query cover was 70% or higher: max score*(query cover/identity). Subsequently all hits were ordered by this score, and the deviation for each similarity score value from the highest similarity score was calculated (Appendix S4). Identifications were assigned based on a combination of the identity score (High identity: $i \geq 95\%$; Medium identity: $90\% \leq i < 95\%$; Low identity: $i < 90\%$) and the number of species within 1% deviation of the calculated similarity score: High identity and one species within 1% deviation -> species-level confidence; High and more than one species -> genus-level confidence; Medium identity and one or more species within the same genus -> genus-level confidence; Medium identity and species from more than one genus -> family-level confidence; Low identity -> family-level confidence. For Kh111 ITS there were 12 hits representing 6 species falling within the 1% deviation from top hit, all belonging to the genus *Amaranthus* and a genus level identification was assigned. For Kh111 *trn*L-F spacer there were 2 hits representing 2 species falling within the 1% deviation from top hit, both belonging to the genus *Amaranthus* and here too a genus level identification was assigned. The combined identification for both markers was *Amaranthus* sp.

Integrative approach. In the integrative approach one takes into account *a priori* data from morphological characteristics of the material, identifications based on available literature, and interview data on vernacular names along with *a posteriori* data from multiple molecular markers and data on traditional use, occurrence, and distribution of putative species in the study area. In this example, *a priori* data (see above) suggested *Amaranthus caudatus* L. based on literature, *Amaranthus* sp. based on ethnobotanical interview data and *Amaranthus* sp. without spiny hairs based on morphology. The BLAST hits suggested either *Amaranthus hybridus* L. (based on ITS) or *Amaranthus spinosus* L. (based on *trn*L-F spacer). *A posteriori* data gives two additional clues that aid the identification process: 1) Consulting the Flora of Iran and other literature shows us that *Amaranthus spinosus* L. does not occur in Iran; and 2) *Amaranthus spinosus* L. has spiny hairs on the inflorescence which are absent in this sample. As a result, using an integrative approach the identification of this sample is *Amaranthus hybridus* L.
